# Supplementary material for: Mapping risks of health conditions in people with atopic eczema in English primary care and hospital data
Source: Nat Commun. 2025 Dec 20;17:555. doi: 10.1038/s41467-025-67247-w (PMC12808256; doi:10.1038/s41467-025-67247-w)
Supplement: Supplementary file 1 — Supplementary Information [file 41467_2025_67247_MOESM1_ESM.pdf]

Supplementary Table 1: Baseline characteristics			
Characteristic	without eczema[1]	with eczema[1]	SMD[2]
any age -			
N	14,103,462 (100%)	3,077,362 (100%)	NA
Male	6,222,354 (44%)	1,376,488 (45%)	NA
Age at index	27 (7, 50)	24 (5, 49)	0.05
Follow-up time	4.7 (1.8, 10.0)	5.5 (2.1, 11.0)	-0.10
Cons. in year pre index	5,578,487 (40%)	1,645,237 (53%)	-0.28
any age - Atopic and allergic			
Asthma	1,222,609 (8.7%)	521,073 (17%)	-0.25
Food allergy	92,327 (0.7%)	66,564 (2.2%)	-0.13
Allergic Rhinitis	788,710 (5.6%)	359,113 (12%)	-0.22
Allergic Conjunctivitis	103,347 (0.7%)	58,391 (1.9%)	-0.10
Eosinophilic Eosophagitis	480 (<0.1%)	227 (<0.1%)	-0.01
any age - Immune mediated			
Alopecia Areata	23,480 (0.2%)	10,607 (0.3%)	-0.04
Urticaria	298,195 (2.1%)	139,207 (4.5%)	-0.13
COPD	191,149 (1.4%)	57,785 (1.9%)	-0.04
any age - Mental health and substance use			
Anxiety	1,060,930 (7.5%)	306,920 (10.0%)	-0.09
Depression	1,552,123 (11%)	430,690 (14%)	-0.09
Alcohol abuse	104,431 (0.7%)	29,033 (0.9%)	-0.02
Cigarette smoking	3,609,461 (26%)	865,234 (28%)	-0.06
any age - ADHD and autism			
ADHD	45,124 (0.3%)	12,043 (0.4%)	-0.01
Autism	44,226 (0.3%)	15,221 (0.5%)	-0.03
any age - Cardiovascular			
Hypertension	1,314,047 (9.3%)	316,381 (10%)	-0.03
Coronary artery disease	640,034 (4.5%)	166,593 (5.4%)	-0.04
Peripheral artery disease	77,786 (0.6%)	22,983 (0.7%)	-0.02
Myocardial infarction	125,198 (0.9%)	31,140 (1.0%)	-0.01
Stroke	105,289 (0.7%)	27,466 (0.9%)	-0.02
Heart failure	108,327 (0.8%)	29,405 (1.0%)	-0.02
Thromboembolic diseases	108,636 (0.8%)	32,983 (1.1%)	-0.03
any age - Metabolic			
Obesity	395,015 (2.8%)	116,931 (3.8%)	-0.06
Dyslipidemia	523,266 (3.7%)	134,710 (4.4%)	-0.03
Diabetes mellitus	469,967 (3.3%)	117,708 (3.8%)	-0.03
Metabolic syndrome	2,712 (<0.1%)	838 (<0.1%)	-0.01
any age - Bone health			
Hip fracture	43,575 (0.3%)	10,940 (0.4%)	-0.01

[1] Median and interquartile range is given for Age at index and Follow-up time; all others are counts and percentage.

[2] Standardised mean difference

Supplementary Table 1: Baseline characteristics			
Characteristic	without eczema[1]	with eczema[1]	SMD[2]
Pelvis fracture	18,629 (0.1%)	4,834 (0.2%)	-0.01
Spine fracture	27,829 (0.2%)	7,510 (0.2%)	-0.01
Wrist fracture	295,000 (2.1%)	76,181 (2.5%)	-0.03
Osteoporosis	157,486 (1.1%)	42,055 (1.4%)	-0.02
any age - Skin infection			
Molluscum contagiosum	146,684 (1.0%)	89,752 (2.9%)	-0.14
Impetigo	394,360 (2.8%)	201,644 (6.6%)	-0.18
Herpes simplex	190,422 (1.4%)	77,891 (2.5%)	-0.09
Dermatophyte infection	629,392 (4.5%)	288,839 (9.4%)	-0.19
Cutaneous warts	792,026 (5.6%)	297,022 (9.7%)	-0.15
any age - Cancer			
Lung cancer	8,836 (<0.1%)	2,446 (<0.1%)	-0.01
Breast cancer	74,899 (0.5%)	18,168 (0.6%)	-0.01
Prostate cancer	50,641 (0.4%)	12,245 (0.4%)	-0.01
Pancreatic cancer	1,307 (<0.1%)	352 (<0.1%)	0.00
Non-hodgkin lymphoma	12,300 (<0.1%)	3,328 (0.1%)	-0.01
Hodgkin lymphoma	3,325 (<0.1%)	954 (<0.1%)	0.00
Myeloma	3,272 (<0.1%)	748 (<0.1%)	0.00
CNS cancers	10,959 (<0.1%)	2,884 (<0.1%)	-0.01
Melanoma	41,371 (0.3%)	10,770 (0.3%)	-0.01
Nonmelanoma skin cancer	156,091 (1.1%)	41,308 (1.3%)	-0.02
any age - Neurological			
Alzheimer's dementia	40,335 (0.3%)	9,307 (0.3%)	0.00
Vascular dementia	20,791 (0.1%)	4,929 (0.2%)	0.00
Epilepsy	109,550 (0.8%)	31,479 (1.0%)	-0.03
Migraine	448,754 (3.2%)	134,735 (4.4%)	-0.06
Multiple sclerosis	15,974 (0.1%)	4,236 (0.1%)	-0.01
Parkinson's disease	24,898 (0.2%)	5,370 (0.2%)	0.00
Peripheral neuropathies	662,781 (4.7%)	191,667 (6.2%)	-0.07
any age - Digestive system			
Abdominal hernia	353,442 (2.5%)	94,890 (3.1%)	-0.04
Appendicitis	131,921 (0.9%)	32,882 (1.1%)	-0.01
Barett's oesophagus	25,032 (0.2%)	6,916 (0.2%)	-0.01
Coeliac disease	24,758 (0.2%)	8,161 (0.3%)	-0.02
Crohn's disease	20,829 (0.1%)	7,969 (0.3%)	-0.02
Diverticular disease	188,648 (1.3%)	52,246 (1.7%)	-0.03
Gastritis and duodenitis	248,985 (1.8%)	78,099 (2.5%)	-0.05
Gastro oesophageal reflux	561,049 (4.0%)	177,375 (5.8%)	-0.08
Irritable bowel syndrome	345,139 (2.4%)	111,849 (3.6%)	-0.07
Oesophageal ulcer	256,539 (1.8%)	80,121 (2.6%)	-0.05
Pancreatitis	24,860 (0.2%)	6,576 (0.2%)	-0.01
Peptic ulcer disease	69,376 (0.5%)	18,469 (0.6%)	-0.01

[1] Median and interquartile range is given for Age at index and Follow-up time; all others are counts and percentage.

[2] Standardised mean difference

Supplementary Table 1: Baseline characteristics

Characteristic	without eczema[1]	with eczema[1]	SMD[2]
Peritonitis	9,448 (<0.1%)	2,365 (<0.1%)	0.00
Ulcerative colitis	30,640 (0.2%)	10,366 (0.3%)	-0.02
any age - Liver			
Autoimmune liver disease	4,146 (<0.1%)	1,197 (<0.1%)	-0.01
Cholecystitis	38,343 (0.3%)	10,288 (0.3%)	-0.01
Fatty liver	50,688 (0.4%)	16,009 (0.5%)	-0.02
Fibrosis/sclerosis/cirrhosis	13,958 (<0.1%)	4,331 (0.1%)	-0.01
Oesophageal varices	3,296 (<0.1%)	986 (<0.1%)	-0.01
18+ -			
N	10,605,853 (100%)	2,167,108 (100%)	NA
Male	4,431,643 (42%)	903,026 (42%)	NA
Age at index	37 (23, 58)	37 (23, 59)	-0.02
Follow-up time	4.4 (1.8, 9.3)	4.8 (1.9, 9.9)	-0.06
Cons. in year pre index	4,893,589 (46%)	1,398,020 (65%)	-0.38
18+ - Atopic and allergic			
Asthma	1,234,248 (12%)	495,151 (23%)	-0.30
Food allergy	85,028 (0.8%)	46,079 (2.1%)	-0.11
Allergic Rhinitis	875,117 (8.3%)	368,215 (17%)	-0.27
Allergic Conjunctivitis	120,141 (1.1%)	59,572 (2.7%)	-0.12
Eosinophilic Eosophagitis	555 (<0.1%)	256 (<0.1%)	-0.01
18+ - Immune mediated			
Alopecia Areata	25,818 (0.2%)	11,205 (0.5%)	-0.04
Urticaria	315,375 (3.0%)	128,031 (5.9%)	-0.14
COPD	192,222 (1.8%)	57,946 (2.7%)	-0.06
18+ - Mental health and substance use			
Anxiety	1,122,564 (11%)	322,655 (15%)	-0.13
Depression	1,612,428 (15%)	449,111 (21%)	-0.14
Alcohol abuse	104,670 (1.0%)	29,123 (1.3%)	-0.03
Cigarette smoking	3,709,250 (35%)	893,121 (41%)	-0.13
18+ - ADHD and autism			
ADHD	51,678 (0.5%)	12,103 (0.6%)	-0.01
Autism	49,653 (0.5%)	13,462 (0.6%)	-0.02
18+ - Cardiovascular			
Hypertension	1,314,149 (12%)	316,170 (15%)	-0.06
Coronary artery disease	639,324 (6.0%)	166,602 (7.7%)	-0.07
Peripheral artery disease	79,447 (0.7%)	23,539 (1.1%)	-0.04
Myocardial infarction	125,009 (1.2%)	31,139 (1.4%)	-0.02
Stroke	104,897 (1.0%)	27,346 (1.3%)	-0.03
Heart failure	107,091 (1.0%)	29,277 (1.4%)	-0.03
Thromboembolic diseases	108,806 (1.0%)	33,180 (1.5%)	-0.04

[1] Median and interquartile range is given for Age at index and Follow-up time; all others are counts and percentage.

[2] Standardised mean difference

Supplementary Table 1: Baseline characteristics			
Characteristic	without eczema[1]	with eczema[1]	SMD[2]
18+ - Metabolic			
Obesity	402,981 (3.8%)	118,834 (5.5%)	-0.08
Dyslipidemia	523,680 (4.9%)	134,784 (6.2%)	-0.06
Diabetes mellitus	472,368 (4.5%)	117,877 (5.4%)	-0.05
Metabolic syndrome	2,726 (<0.1%)	846 (<0.1%)	-0.01
18+ - Bone health			
Hip fracture	43,828 (0.4%)	10,970 (0.5%)	-0.01
Pelvis fracture	19,317 (0.2%)	5,005 (0.2%)	-0.01
Spine fracture	28,833 (0.3%)	7,729 (0.4%)	-0.02
Wrist fracture	338,711 (3.2%)	83,507 (3.9%)	-0.04
Osteoporosis	158,047 (1.5%)	42,185 (1.9%)	-0.04
18+ - Skin infection			
Molluscum contagiosum	194,304 (1.8%)	83,829 (3.9%)	-0.12
Impetigo	432,957 (4.1%)	178,705 (8.2%)	-0.17
Herpes simplex	202,523 (1.9%)	77,197 (3.6%)	-0.10
Dermatophyte infection	662,551 (6.2%)	277,474 (13%)	-0.22
Cutaneous warts	981,375 (9.3%)	322,054 (15%)	-0.17
18+ - Cancer			
Lung cancer	8,678 (<0.1%)	2,436 (0.1%)	-0.01
Breast cancer	74,747 (0.7%)	18,178 (0.8%)	-0.02
Prostate cancer	50,873 (0.5%)	12,243 (0.6%)	-0.01
Pancreatic cancer	1,285 (<0.1%)	354 (<0.1%)	0.00
Non-hodgkin lymphoma	12,455 (0.1%)	3,333 (0.2%)	-0.01
Hodgkin lymphoma	3,456 (<0.1%)	1,010 (<0.1%)	-0.01
Myeloma	3,352 (<0.1%)	748 (<0.1%)	0.00
CNS cancers	10,896 (0.1%)	2,877 (0.1%)	-0.01
Melanoma	41,166 (0.4%)	10,719 (0.5%)	-0.02
Nonmelanoma skin cancer	156,122 (1.5%)	41,315 (1.9%)	-0.03
18+ - Neurological			
Alzheimer's dementia	40,145 (0.4%)	9,326 (0.4%)	-0.01
Vascular dementia	20,756 (0.2%)	4,922 (0.2%)	-0.01
Epilepsy	108,905 (1.0%)	30,926 (1.4%)	-0.04
Migraine	493,450 (4.7%)	146,192 (6.7%)	-0.09
Multiple sclerosis	15,922 (0.2%)	4,245 (0.2%)	-0.01
Parkinson's disease	24,920 (0.2%)	5,372 (0.2%)	0.00
Peripheral neuropathies	664,777 (6.3%)	191,806 (8.9%)	-0.10
18+ - Digestive system			
Abdominal hernia	286,265 (2.7%)	72,461 (3.3%)	-0.04
Appendicitis	145,789 (1.4%)	35,705 (1.6%)	-0.02
Barett's oesophagus	24,999 (0.2%)	6,939 (0.3%)	-0.02
Coeliac disease	25,285 (0.2%)	8,101 (0.4%)	-0.02

[1] Median and interquartile range is given for Age at index and Follow-up time; all others are counts and percentage.

[2] Standardised mean difference

Supplementary Table 1: Baseline characteristics			
Characteristic	without eczema[1]	with eczema[1]	SMD[2]
Crohn's disease	22,397 (0.2%)	8,416 (0.4%)	-0.03
Diverticular disease	188,506 (1.8%)	52,228 (2.4%)	-0.04
Gastritis and duodenitis	254,816 (2.4%)	78,445 (3.6%)	-0.07
Gastro oesophageal reflux	415,368 (3.9%)	126,694 (5.8%)	-0.09
Irritable bowel syndrome	356,169 (3.4%)	115,391 (5.3%)	-0.10
Oesophageal ulcer	242,547 (2.3%)	74,675 (3.4%)	-0.07
Pancreatitis	25,028 (0.2%)	6,648 (0.3%)	-0.01
Peptic ulcer disease	69,170 (0.7%)	18,504 (0.9%)	-0.02
Peritonitis	9,671 (<0.1%)	2,405 (0.1%)	-0.01
Ulcerative colitis	31,321 (0.3%)	10,573 (0.5%)	-0.03
18+ - Liver			
Autoimmune liver disease	4,267 (<0.1%)	1,219 (<0.1%)	-0.01
Cholecystitis	38,448 (0.4%)	10,335 (0.5%)	-0.02
Fatty liver	51,022 (0.5%)	16,154 (0.7%)	-0.03
Fibrosis/sclerosis/cirrhosis	13,955 (0.1%)	4,318 (0.2%)	-0.02
Oesophageal varices	3,317 (<0.1%)	984 (<0.1%)	-0.01
40+ -			
N	5,873,150 (100%)	1,202,474 (100%)	NA
Male	2,487,358 (42%)	509,194 (42%)	NA
Age at index	55 (43, 69)	56 (43, 70)	-0.03
Follow-up time	5.8 (2.5, 10.8)	6.2 (2.7, 11.3)	-0.05
Cons. in year pre index	3,207,342 (55%)	843,989 (70%)	-0.33
40+ - Atopic and allergic			
Asthma	615,931 (10%)	225,131 (19%)	-0.23
Food allergy	28,900 (0.5%)	11,175 (0.9%)	-0.05
Allergic Rhinitis	399,891 (6.8%)	153,553 (13%)	-0.20
Allergic Conjunctivitis	60,001 (1.0%)	29,110 (2.4%)	-0.11
Eosinophilic Eosophagitis	249 (<0.1%)	102 (<0.1%)	-0.01
40+ - Immune mediated			
Alopecia Areata	13,648 (0.2%)	5,695 (0.5%)	-0.04
Urticaria	153,364 (2.6%)	64,373 (5.4%)	-0.14
COPD	190,152 (3.2%)	56,912 (4.7%)	-0.08
40+ - Mental health and substance use			
Anxiety	735,641 (13%)	204,416 (17%)	-0.13
Depression	1,126,879 (19%)	304,057 (25%)	-0.15
Alcohol abuse	90,659 (1.5%)	25,208 (2.1%)	-0.04
Cigarette smoking	2,407,939 (41%)	564,196 (47%)	-0.12
40+ - ADHD and autism			
ADHD	2,331 (<0.1%)	648 (<0.1%)	-0.01
Autism	3,354 (<0.1%)	1,312 (0.1%)	-0.02
40+ - Cardiovascular			

[1] Median and interquartile range is given for Age at index and Follow-up time; all others are counts and percentage.

[2] Standardised mean difference

Supplementary Table 1: Baseline characteristics			
Characteristic	without eczema[1]	with eczema[1]	SMD[2]
Hypertension	1,311,220 (22%)	315,565 (26%)	-0.09
Coronary artery disease	645,785 (11%)	168,509 (14%)	-0.09
Peripheral artery disease	69,165 (1.2%)	20,173 (1.7%)	-0.04
Myocardial infarction	123,373 (2.1%)	30,779 (2.6%)	-0.03
Stroke	102,507 (1.7%)	26,766 (2.2%)	-0.03
Heart failure	106,574 (1.8%)	29,024 (2.4%)	-0.04
Thromboembolic diseases	102,151 (1.7%)	31,091 (2.6%)	-0.06
40+ - Metabolic			
Obesity	344,910 (5.9%)	98,421 (8.2%)	-0.09
Dyslipidemia	517,580 (8.8%)	133,075 (11%)	-0.08
Diabetes mellitus	453,376 (7.7%)	113,130 (9.4%)	-0.06
Metabolic syndrome	2,609 (<0.1%)	806 (<0.1%)	-0.01
40+ - Bone health			
Hip fracture	42,271 (0.7%)	10,581 (0.9%)	-0.02
Pelvis fracture	15,797 (0.3%)	4,054 (0.3%)	-0.01
Spine fracture	23,834 (0.4%)	6,443 (0.5%)	-0.02
Wrist fracture	147,902 (2.5%)	35,427 (2.9%)	-0.03
Osteoporosis	154,605 (2.6%)	40,971 (3.4%)	-0.05
40+ - Skin infection			
Molluscum contagiosum	7,980 (0.1%)	2,920 (0.2%)	-0.02
Impetigo	84,427 (1.4%)	36,361 (3.0%)	-0.11
Herpes simplex	114,192 (1.9%)	41,360 (3.4%)	-0.09
Dermatophyte infection	423,613 (7.2%)	169,264 (14%)	-0.22
Cutaneous warts	390,155 (6.6%)	122,114 (10%)	-0.13
40+ - Cancer			
Lung cancer	8,722 (0.1%)	2,421 (0.2%)	-0.01
Breast cancer	75,400 (1.3%)	18,189 (1.5%)	-0.02
Prostate cancer	50,537 (0.9%)	12,128 (1.0%)	-0.02
Pancreatic cancer	1,249 (<0.1%)	348 (<0.1%)	0.00
Non-hodgkin lymphoma	11,594 (0.2%)	3,134 (0.3%)	-0.01
Hodgkin lymphoma	2,226 (<0.1%)	687 (<0.1%)	-0.01
Myeloma	3,365 (<0.1%)	744 (<0.1%)	0.00
CNS cancers	9,322 (0.2%)	2,408 (0.2%)	-0.01
Melanoma	39,683 (0.7%)	10,136 (0.8%)	-0.02
Nonmelanoma skin cancer	156,596 (2.7%)	41,296 (3.4%)	-0.04
40+ - Neurological			
Alzheimer's dementia	40,453 (0.7%)	9,330 (0.8%)	-0.01
Vascular dementia	20,670 (0.4%)	4,940 (0.4%)	-0.01
Epilepsy	67,580 (1.2%)	19,627 (1.6%)	-0.04
Migraine	279,686 (4.8%)	79,192 (6.6%)	-0.08
Multiple sclerosis	14,963 (0.3%)	3,907 (0.3%)	-0.01

[1] Median and interquartile range is given for Age at index and Follow-up time; all others are counts and percentage.

[2] Standardised mean difference

Supplementary Table 1: Baseline characteristics			
Characteristic	without eczema[1]	with eczema[1]	SMD[2]
Parkinson's disease	24,805 (0.4%)	5,369 (0.4%)	0.00
Peripheral neuropathies	633,963 (11%)	180,408 (15%)	-0.13
40+ - Digestive system			
Abdominal hernia	238,468 (4.1%)	59,439 (4.9%)	-0.04
Appendicitis	61,707 (1.1%)	14,256 (1.2%)	-0.01
Barett's oesophagus	24,748 (0.4%)	6,863 (0.6%)	-0.02
Coeliac disease	15,929 (0.3%)	4,961 (0.4%)	-0.02
Crohn's disease	15,546 (0.3%)	5,242 (0.4%)	-0.03
Diverticular disease	189,288 (3.2%)	52,332 (4.4%)	-0.06
Gastritis and duodenitis	202,446 (3.4%)	60,761 (5.1%)	-0.08
Gastro oesophageal reflux	350,610 (6.0%)	105,448 (8.8%)	-0.11
Irritable bowel syndrome	256,647 (4.4%)	80,667 (6.7%)	-0.10
Oesophageal ulcer	225,636 (3.8%)	68,437 (5.7%)	-0.09
Pancreatitis	22,724 (0.4%)	5,927 (0.5%)	-0.02
Peptic ulcer disease	66,563 (1.1%)	17,664 (1.5%)	-0.03
Peritonitis	7,813 (0.1%)	1,901 (0.2%)	-0.01
Ulcerative colitis	25,794 (0.4%)	8,212 (0.7%)	-0.03
40+ - Liver			
Autoimmune liver disease	3,778 (<0.1%)	1,058 (<0.1%)	-0.01
Cholecystitis	36,644 (0.6%)	9,648 (0.8%)	-0.02
Fatty liver	49,350 (0.8%)	15,243 (1.3%)	-0.04
Fibrosis/sclerosis/cirrhosis	13,794 (0.2%)	4,244 (0.4%)	-0.02
Oesophageal varices	3,252 (<0.1%)	953 (<0.1%)	-0.01
<18 -			
N	6,054,252 (100%)	1,424,335 (100%)	NA
Male	2,974,532 (49%)	711,754 (50%)	NA
Age at index	4 (1, 13)	4 (2, 12)	0.05
Follow-up time	3.9 (1.5, 9.1)	5.2 (2.0, 10.8)	-0.16
Cons. in year pre index	1,464,135 (24%)	487,852 (34%)	-0.22
<18 - Atopic and allergic			
Asthma	386,222 (6.4%)	192,274 (13%)	-0.24
Food allergy	46,431 (0.8%)	44,241 (3.1%)	-0.17
Allergic Rhinitis	232,553 (3.8%)	132,302 (9.3%)	-0.22
Allergic Conjunctivitis	33,224 (0.5%)	22,837 (1.6%)	-0.10
<18 - Immune mediated			
Alopecia Areata	5,888 (<0.1%)	2,898 (0.2%)	-0.03
Urticaria	113,247 (1.9%)	59,243 (4.2%)	-0.13
COPD	1,942 (<0.1%)	876 (<0.1%)	-0.01
<18 - Mental health and substance use			
Anxiety	161,974 (2.7%)	47,560 (3.3%)	-0.04
Depression	177,780 (2.9%)	47,255 (3.3%)	-0.02

[1] Median and interquartile range is given for Age at index and Follow-up time; all others are counts and percentage.

[2] Standardised mean difference

Supplementary Table 1: Baseline characteristics			
Characteristic	without eczema[1]	with eczema[1]	SMD[2]
Alcohol abuse	4,429 (<0.1%)	979 (<0.1%)	0.00
Cigarette smoking	468,067 (7.7%)	121,591 (8.5%)	-0.03
<18 - ADHD and autism			
ADHD	34,156 (0.6%)	8,734 (0.6%)	-0.01
Autism	35,302 (0.6%)	11,363 (0.8%)	-0.03
<18 - Cardiovascular			
Hypertension	5,594 (<0.1%)	1,423 (<0.1%)	0.00
Coronary artery disease	667 (<0.1%)	255 (<0.1%)	-0.01
Peripheral artery disease	4,651 (<0.1%)	1,670 (0.1%)	-0.01
Myocardial infarction	691 (<0.1%)	183 (<0.1%)	0.00
Stroke	1,957 (<0.1%)	532 (<0.1%)	0.00
Heart failure	1,267 (<0.1%)	340 (<0.1%)	0.00
Thromboembolic diseases	3,173 (<0.1%)	910 (<0.1%)	0.00
<18 - Metabolic			
Obesity	28,013 (0.5%)	9,493 (0.7%)	-0.03
Dyslipidemia	4,389 (<0.1%)	1,142 (<0.1%)	0.00
Diabetes mellitus	11,467 (0.2%)	2,676 (0.2%)	0.00
<18 - Bone health			
Hip fracture	900 (<0.1%)	223 (<0.1%)	0.00
Pelvis fracture	1,200 (<0.1%)	359 (<0.1%)	0.00
Spine fracture	1,639 (<0.1%)	420 (<0.1%)	0.00
Wrist fracture	82,959 (1.4%)	23,700 (1.7%)	-0.02
Osteoporosis	1,933 (<0.1%)	514 (<0.1%)	0.00
<18 - Skin infection			
Molluscum contagiosum	119,971 (2.0%)	78,159 (5.5%)	-0.19
Impetigo	249,959 (4.1%)	137,124 (9.6%)	-0.22
Herpes simplex	47,178 (0.8%)	23,848 (1.7%)	-0.08
Dermatophyte infection	148,086 (2.4%)	86,252 (6.1%)	-0.18
Cutaneous warts	285,312 (4.7%)	130,243 (9.1%)	-0.18
<18 - Cancer			
Non-hodgkin lymphoma	411 (<0.1%)	137 (<0.1%)	0.00
Hodgkin lymphoma	388 (<0.1%)	107 (<0.1%)	0.00
CNS cancers	1,093 (<0.1%)	270 (<0.1%)	0.00
Melanoma	938 (<0.1%)	446 (<0.1%)	-0.01
<18 - Digestive system			
Abdominal hernia	107,056 (1.8%)	32,820 (2.3%)	-0.04
Appendicitis	33,004 (0.5%)	8,863 (0.6%)	-0.01
Coeliac disease	5,922 (<0.1%)	2,061 (0.1%)	-0.01
Crohn's disease	2,249 (<0.1%)	977 (<0.1%)	-0.01
Gastritis and duodenitis	27,950 (0.5%)	10,173 (0.7%)	-0.03
Gastro oesophageal reflux	193,570 (3.2%)	64,001 (4.5%)	-0.07

[1] Median and interquartile range is given for Age at index and Follow-up time; all others are counts and percentage.

[2] Standardised mean difference

Supplementary Table 1: Baseline characteristics			
Characteristic	without eczema[1]	with eczema[1]	SMD[2]
Irritable bowel syndrome	33,226 (0.5%)	11,078 (0.8%)	-0.03
Oesophageal ulcer	26,166 (0.4%)	9,473 (0.7%)	-0.03
Pancreatitis	912 (<0.1%)	238 (<0.1%)	0.00
Peptic ulcer disease	961 (<0.1%)	290 (<0.1%)	0.00
Peritonitis	908 (<0.1%)	247 (<0.1%)	0.00
Ulcerative colitis	2,109 (<0.1%)	764 (<0.1%)	-0.01
<18 - Liver			
Cholecystitis	1,003 (<0.1%)	284 (<0.1%)	0.00
Fatty liver	1,776 (<0.1%)	482 (<0.1%)	0.00
<18 - Neurological			
Epilepsy	25,838 (0.4%)	6,723 (0.5%)	-0.01
Migraine	81,867 (1.4%)	26,094 (1.8%)	-0.04
Peripheral neuropathies	22,993 (0.4%)	6,640 (0.5%)	-0.01
hospitalised -			
N	10,571,189 (100%)	2,435,001 (100%)	NA
Male	4,534,880 (43%)	1,067,196 (44%)	NA
Age at index	30 (6, 55)	26 (5, 53)	0.07
Follow-up time	4.7 (1.9, 9.4)	5.2 (2.1, 10.1)	-0.08
Cons. in year pre index	4,910,747 (46%)	1,360,996 (56%)	-0.19
hospitalised - Atopic and allergic			
Asthma	1,084,680 (10%)	437,131 (18%)	-0.22
Food allergy	77,587 (0.7%)	56,828 (2.3%)	-0.13
Allergic Rhinitis	694,223 (6.6%)	299,414 (12%)	-0.20
Allergic Conjunctivitis	97,505 (0.9%)	52,351 (2.1%)	-0.10
Eosinophilic Eosophagitis	700 (<0.1%)	264 (<0.1%)	0.00
hospitalised - Immune mediated			
Alopecia Areata	21,715 (0.2%)	9,105 (0.4%)	-0.03
Urticaria	283,121 (2.7%)	125,424 (5.2%)	-0.13
COPD	207,102 (2.0%)	60,286 (2.5%)	-0.04
hospitalised - Mental health and substance use			
Anxiety	1,032,394 (9.8%)	281,310 (12%)	-0.06
Depression	1,549,101 (15%)	401,815 (17%)	-0.05
Alcohol abuse	113,440 (1.1%)	28,362 (1.2%)	-0.01
Cigarette smoking	3,193,677 (30%)	756,990 (31%)	-0.02
hospitalised - ADHD and autism			
ADHD	39,177 (0.4%)	10,278 (0.4%)	-0.01
Autism	39,629 (0.4%)	13,118 (0.5%)	-0.02
hospitalised - Cardiovascular			
Hypertension	1,313,426 (12%)	311,061 (13%)	-0.01
Coronary artery disease	690,211 (6.5%)	172,480 (7.1%)	-0.02

[1] Median and interquartile range is given for Age at index and Follow-up time; all others are counts and percentage.

[2] Standardised mean difference

Supplementary Table 1: Baseline characteristics

Characteristic	without eczema[1]	with eczema[1]	SMD[2]
Peripheral artery disease	83,654 (0.8%)	23,354 (1.0%)	-0.02
Myocardial infarction	147,185 (1.4%)	33,959 (1.4%)	0.00
Stroke	120,087 (1.1%)	30,027 (1.2%)	-0.01
Heart failure	123,385 (1.2%)	31,916 (1.3%)	-0.01
Thromboembolic diseases	123,365 (1.2%)	35,211 (1.4%)	-0.02
hospitalised - Metabolic			
Obesity	401,323 (3.8%)	114,185 (4.7%)	-0.04
Dyslipidemia	536,131 (5.1%)	134,222 (5.5%)	-0.02
Diabetes mellitus	483,228 (4.6%)	116,987 (4.8%)	-0.01
Metabolic syndrome	2,952 (<0.1%)	867 (<0.1%)	0.00
hospitalised - Bone health			
Hip fracture	52,147 (0.5%)	12,377 (0.5%)	0.00
Pelvis fracture	21,182 (0.2%)	5,127 (0.2%)	0.00
Spine fracture	31,905 (0.3%)	7,970 (0.3%)	0.00
Wrist fracture	293,825 (2.8%)	69,994 (2.9%)	-0.01
Osteoporosis	169,971 (1.6%)	44,606 (1.8%)	-0.02
hospitalised - Skin infection			
Molluscum contagiosum	120,247 (1.1%)	70,284 (2.9%)	-0.12
Impetigo	349,957 (3.3%)	168,333 (6.9%)	-0.16
Herpes simplex	185,290 (1.8%)	71,661 (2.9%)	-0.08
Dermatophyte infection	600,933 (5.7%)	256,233 (11%)	-0.18
Cutaneous warts	731,181 (6.9%)	255,819 (11%)	-0.13
hospitalised - Cancer			
Lung cancer	11,011 (0.1%)	2,931 (0.1%)	0.00
Breast cancer	91,299 (0.9%)	20,295 (0.8%)	0.00
Prostate cancer	60,101 (0.6%)	13,798 (0.6%)	0.00
Pancreatic cancer	1,624 (<0.1%)	443 (<0.1%)	0.00
Non-hodgkin lymphoma	15,368 (0.1%)	3,914 (0.2%)	0.00
Hodgkin lymphoma	4,018 (<0.1%)	1,142 (<0.1%)	0.00
Myeloma	4,449 (<0.1%)	946 (<0.1%)	0.00
CNS cancers	13,089 (0.1%)	3,135 (0.1%)	0.00
Melanoma	47,025 (0.4%)	11,468 (0.5%)	0.00
Nonmelanoma skin cancer	176,412 (1.7%)	45,059 (1.9%)	-0.01
hospitalised - Neurological			
Alzheimer's dementia	40,947 (0.4%)	9,603 (0.4%)	0.00
Vascular dementia	21,458 (0.2%)	5,198 (0.2%)	0.00
Epilepsy	113,365 (1.1%)	30,053 (1.2%)	-0.02
Migraine	434,659 (4.1%)	122,685 (5.0%)	-0.04
Multiple sclerosis	17,794 (0.2%)	4,269 (0.2%)	0.00
Parkinson's disease	26,332 (0.2%)	5,628 (0.2%)	0.00
Peripheral neuropathies	703,573 (6.7%)	193,371 (7.9%)	-0.05

[1] Median and interquartile range is given for Age at index and Follow-up time; all others are counts and percentage.

[2] Standardised mean difference

Supplementary Table 1: Baseline characteristics			
Characteristic	without eczema[1]	with eczema[1]	SMD[2]
hospitalised - Digestive system			
Abdominal hernia	389,682 (3.7%)	96,133 (3.9%)	-0.01
Appendicitis	156,542 (1.5%)	34,630 (1.4%)	0.00
Barett's oesophagus	30,187 (0.3%)	7,750 (0.3%)	-0.01
Coeliac disease	27,021 (0.3%)	8,413 (0.3%)	-0.02
Crohn's disease	25,284 (0.2%)	8,472 (0.3%)	-0.02
Diverticular disease	217,706 (2.1%)	57,586 (2.4%)	-0.02
Gastritis and duodenitis	276,680 (2.6%)	79,448 (3.3%)	-0.04
Gastro oesophageal reflux	590,227 (5.6%)	176,166 (7.2%)	-0.07
Irritable bowel syndrome	351,337 (3.3%)	105,751 (4.3%)	-0.05
Oesophageal ulcer	286,158 (2.7%)	82,937 (3.4%)	-0.04
Pancreatitis	30,459 (0.3%)	7,257 (0.3%)	0.00
Peptic ulcer disease	79,157 (0.7%)	19,471 (0.8%)	-0.01
Peritonitis	11,593 (0.1%)	2,576 (0.1%)	0.00
Ulcerative colitis	36,561 (0.3%)	10,972 (0.5%)	-0.02
hospitalised - Liver			
Autoimmune liver disease	4,843 (<0.1%)	1,282 (<0.1%)	0.00
Cholecystitis	46,403 (0.4%)	11,282 (0.5%)	0.00
Fatty liver	56,315 (0.5%)	16,509 (0.7%)	-0.02
Fibrosis/sclerosis/cirrhosis	16,818 (0.2%)	4,685 (0.2%)	-0.01
Oesophageal varices	4,173 (<0.1%)	1,101 (<0.1%)	0.00

[1] Median and interquartile range is given for Age at index and Follow-up time; all others are counts and percentage.

[2] Standardised mean difference

Supplementary Table 2: Rates, events, and rate differences from all cohorts

Outcome	events (overall)	person-years	Rate (per 1,000 person-years) exposed	unexposed (observed)	unexposed (estimated)[2]	Rate difference (per 1,000 person-years) observed[3]	crude estimated[4]	adjusted estimated[5]	Hazard ratio (99% confidence interval)[1] crude	comorbidity-adjusted
any age - A00-B99										
A02	1,720	114,221,040	0.02	0.01	0.01	0.00	0.00	0.00	1.23 [1.05-1.44]	1.15 [0.98-1.36]
A04	51,460	113,666,050	0.51	0.44	0.41	0.08	0.11	0.09	1.27 [1.23-1.31]*	1.21 [1.17-1.24]*
A05	520	114,263,960	0.01	0.00	0.00	0.00	0.00	0.00	1.26 [0.93-1.69]	1.20 [0.89-1.63]
A07	970	114,252,240	0.01	0.01	0.01	0.00	0.00	0.00	1.21 [0.98-1.50]	1.14 [0.92-1.42]
A08	62,940	112,513,630	0.68	0.53	0.57	0.15	0.11	0.09	1.20 [1.17-1.23]*	1.15 [1.12-1.18]*
A09	223,490	112,582,510	2.38	1.89	1.84	0.49	0.54	0.40	1.29 [1.28-1.31]*	1.20 [1.18-1.22]*
A15	1,320	114,244,380	0.01	0.01	0.01	0.00	0.00	0.00	1.09 [0.90-1.32]	1.06 [0.87-1.29]
A16	2,620	114,216,350	0.03	0.02	0.02	0.01	0.01	0.01	1.33 [1.17-1.51]*	1.28 [1.12-1.46]*
A18	1,360	114,233,310	0.01	0.01	0.01	0.00	0.00	0.00	1.19 [0.99-1.42]	1.14 [0.95-1.38]
A31	1,100	114,260,560	0.01	0.01	0.01	0.00	0.00	0.00	1.41 [1.16-1.71]*	1.26 [1.03-1.54]
A38	2,000	114,245,630	0.02	0.02	0.02	0.00	0.00	0.00	1.08 [0.93-1.25]	1.06 [0.91-1.22]
A39	2,380	114,167,390	0.02	0.02	0.02	0.00	0.00	0.00	1.06 [0.92-1.21]	1.03 [0.90-1.18]
A40	10,380	114,180,090	0.11	0.09	0.08	0.02	0.03	0.02	1.33 [1.25-1.43]*	1.28 [1.20-1.37]*
A41	195,330	113,361,790	1.96	1.67	1.56	0.29	0.39	0.34	1.25 [1.23-1.27]*	1.21 [1.19-1.23]*
A46	680	114,251,130	0.01	0.01	0.00	0.00	0.01	0.01	2.16 [1.72-2.72]*	2.11 [1.67-2.66]*
A48	1,020	114,255,470	0.01	0.01	0.01	0.00	0.00	0.00	1.21 [0.98-1.49]	1.22 [0.99-1.51]
A49	28,210	113,966,200	0.30	0.24	0.22	0.06	0.08	0.07	1.34 [1.29-1.40]*	1.28 [1.23-1.34]*
A63	940	114,178,580	0.01	0.01	0.01	0.00	0.00	0.00	1.49 [1.21-1.83]*	1.35 [1.09-1.67]*
A69	750	114,260,870	0.01	0.01	0.01	0.00	0.00	0.00	1.25 [0.99-1.59]	1.15 [0.90-1.47]
A86	1,500	114,250,770	0.01	0.01	0.01	0.00	0.00	0.00	1.01 [0.84-1.21]	0.98 [0.81-1.17]
A87	3,700	114,154,320	0.04	0.03	0.03	0.01	0.01	0.01	1.31 [1.17-1.45]*	1.20 [1.08-1.34]*
B00	10,350	114,046,030	0.18	0.07	0.07	0.11	0.11	0.10	2.52 [2.38-2.67]*	2.34 [2.21-2.49]*
B01	9,210	114,001,710	0.12	0.07	0.08	0.05	0.05	0.04	1.61 [1.52-1.72]*	1.56 [1.46-1.66]*
B02	14,520	114,138,900	0.16	0.12	0.11	0.04	0.05	0.04	1.42 [1.34-1.50]*	1.34 [1.26-1.42]*
B07	6,380	114,112,930	0.07	0.05	0.05	0.02	0.02	0.02	1.41 [1.30-1.53]*	1.30 [1.20-1.42]*
B08	3,300	114,191,180	0.04	0.03	0.03	0.02	0.02	0.01	1.60 [1.44-1.77]*	1.52 [1.37-1.69]*
B09	1,460	114,221,410	0.02	0.01	0.01	0.00	0.00	0.00	1.19 [1.01-1.41]	1.18 [0.99-1.40]
B15	800	114,254,390	0.01	0.01	0.01	0.00	0.00	0.00	1.10 [0.86-1.39]	1.06 [0.83-1.36]
B16	1,380	114,232,470	0.01	0.01	0.01	0.00	0.00	0.00	1.34 [1.12-1.59]*	1.31 [1.09-1.56]*
B17	2,140	114,224,270	0.02	0.02	0.02	0.00	0.01	0.01	1.39 [1.21-1.60]*	1.33 [1.15-1.53]*
B18	8,820	114,110,750	0.08	0.08	0.07	0.01	0.01	0.01	1.19 [1.11-1.28]*	1.16 [1.08-1.25]*
B19	560	114,260,820	0.01	0.00	0.00	0.00	0.00	0.00	1.16 [0.87-1.54]	1.07 [0.80-1.43]
B25	1,720	114,247,760	0.02	0.01	0.01	0.00	0.01	0.00	1.41 [1.21-1.64]*	1.35 [1.15-1.58]*
B26	640	114,257,510	0.01	0.01	0.01	0.00	0.00	0.00	1.20 [0.92-1.56]	1.21 [0.92-1.58]
B27	6,690	114,156,630	0.07	0.06	0.06	0.02	0.01	0.01	1.15 [1.06-1.24]*	1.10 [1.01-1.19]
B30	650	114,257,500	0.01	0.01	0.01	0.00	0.00	0.00	1.26 [0.98-1.62]	1.16 [0.89-1.50]
B33	470	114,259,950	0.00	0.00	0.00	0.00	0.00	0.00	1.24 [0.93-1.66]	1.22 [0.91-1.64]
B34	138,970	110,561,040	1.89	1.11	1.23	0.78	0.66	0.61	1.54 [1.52-1.57]*	1.48 [1.45-1.50]*
B35	6,180	114,201,260	0.08	0.05	0.05	0.03	0.03	0.03	1.72 [1.58-1.86]*	1.58 [1.45-1.71]*
B36	2,280	114,252,190	0.03	0.02	0.02	0.01	0.01	0.01	1.87 [1.63-2.14]*	1.72 [1.50-1.97]*
B37	81,580	113,457,400	0.87	0.68	0.64	0.18	0.23	0.17	1.36 [1.33-1.39]*	1.25 [1.22-1.28]*
B44	3,180	114,242,930	0.04	0.03	0.02	0.01	0.02	0.01	1.69 [1.51-1.88]*	1.40 [1.24-1.57]*
B49	1,890	114,256,280	0.02	0.02	0.01	0.01	0.01	0.01	1.63 [1.41-1.88]*	1.54 [1.33-1.79]*
B50	1,050	114,239,870	0.01	0.01	0.01	0.00	0.00	0.00	0.95 [0.76-1.19]	0.97 [0.77-1.22]
B59	940	114,264,600	0.01	0.01	0.01	0.00	0.00	0.00	1.53 [1.24-1.88]*	1.44 [1.16-1.78]*
B80	2,610	114,229,070	0.03	0.02	0.02	0.01	0.00	0.00	1.21 [1.06-1.37]*	1.14 [1.00-1.30]
B85	1,080	114,251,810	0.01	0.01	0.01	0.00	0.00	0.00	1.12 [0.91-1.37]	1.11 [0.90-1.36]
B86	1,750	114,232,630	0.03	0.01	0.01	0.02	0.02	0.02	2.53 [2.19-2.93]*	2.49 [2.15-2.89]*
B90	740	114,262,250	0.01	0.01	0.01	0.00	0.00	0.00	1.60 [1.25-2.03]*	1.46 [1.14-1.87]*
B91	1,180	114,252,480	0.01	0.01	0.01	0.00	0.00	0.00	1.30 [1.07-1.58]*	1.30 [1.06-1.58]
B94	2,030	114,246,300	0.02	0.02	0.02	0.00	0.01	0.00	1.32 [1.14-1.52]*	1.19 [1.02-1.39]
B95	118,310	112,611,290	1.37	0.97	0.91	0.40	0.46	0.42	1.51 [1.48-1.54]*	1.44 [1.42-1.47]*
B96	235,480	111,557,930	2.38	2.05	1.94	0.34	0.44	0.35	1.23 [1.21-1.24]*	1.17 [1.16-1.19]*
B97	64,700	113,345,550	0.72	0.54	0.54	0.18	0.17	0.14	1.32 [1.29-1.35]*	1.23 [1.20-1.27]*
B98	23,060	114,109,250	0.22	0.20	0.19	0.03	0.04	0.03	1.20 [1.14-1.25]*	1.14 [1.09-1.19]*
B99	6,520	114,237,370	0.07	0.06	0.05	0.01	0.01	0.01	1.24 [1.14-1.36]*	1.20 [1.10-1.31]*
any age - C00-D49										
C00	780	114,260,080	0.01	0.01	0.01	0.00	0.00	0.00	1.04 [0.81-1.35]	1.03 [0.80-1.33]
C01	1,640	114,258,730	0.01	0.01	0.01	0.00	0.00	0.00	1.08 [0.91-1.28]	1.08 [0.90-1.28]
C02	2,530	114,243,900	0.02	0.02	0.02	0.00	0.01	0.01	1.28 [1.13-1.46]*	1.27 [1.11-1.45]*
C03	580	114,266,480	0.01	0.00	0.00	0.00	0.00	0.00	1.47 [1.12-1.92]*	1.46 [1.11-1.91]*
C04	750	114,261,650	0.01	0.01	0.01	0.00	0.00	0.00	1.13 [0.88-1.45]	1.14 [0.89-1.47]
C05	820	114,262,400	0.01	0.01	0.01	0.00	0.00	0.00	1.28 [1.01-1.62]	1.29 [1.02-1.64]
C06	1,170	114,260,580	0.01	0.01	0.01	0.00	0.00	0.00	1.25 [1.03-1.52]	1.24 [1.01-1.51]
C07	1,180	114,257,890	0.01	0.01	0.01	0.00	0.00	0.00	1.03 [0.84-1.27]	1.02 [0.83-1.25]
C09	2,110	114,249,450	0.02	0.02	0.02	0.00	0.00	0.00	1.07 [0.92-1.25]	1.04 [0.89-1.22]
C10	1,070	114,264,510	0.01	0.01	0.01	0.00	0.00	0.00	1.11 [0.90-1.37]	1.09 [0.88-1.35]
C11	560	114,265,080	0.01	0.00	0.00	0.00	0.00	0.00	1.19 [0.90-1.58]	1.19 [0.89-1.58]
C12	580	114,267,020	0.01	0.00	0.00	0.00	0.00	0.00	1.24 [0.94-1.63]	1.25 [0.94-1.65]
C13	650	114,268,040	0.01	0.01	0.01	0.00	0.00	0.00	1.16 [0.89-1.52]	1.20 [0.91-1.57]
C14	1,210	114,264,160	0.01	0.01	0.01	0.00	0.00	0.00	1.10 [0.90-1.34]	1.08 [0.88-1.33]
C15	16,200	114,213,050	0.15	0.14	0.13	0.01	0.02	0.02	1.18 [1.12-1.25]*	1.17 [1.11-1.24]*
C16	13,250	114,215,520	0.11	0.12	0.11	0.00	0.01	0.01	1.08 [1.01-1.15]	1.07 [1.01-1.14]
C17	2,680	114,255,610	0.02	0.02	0.02	0.00	0.00	0.00	1.11 [0.98-1.27]	1.10 [0.96-1.26]
C18	45,750	113,878,370	0.38	0.41	0.37	−0.03	0.01	0.00	1.01 [0.98-1.05]	1.00 [0.97-1.04]
C19	9,480	114,197,600	0.07	0.09	0.08	−0.01	0.00	0.00	0.95 [0.88-1.02]	0.95 [0.88-1.02]
C20	19,640	114,069,270	0.15	0.18	0.16	−0.02	−0.01	0.00	0.97 [0.92-1.02]	0.98 [0.93-1.03]
C21	2,440	114,245,640	0.02	0.02	0.02	0.00	0.00	0.00	1.08 [0.94-1.24]	1.07 [0.93-1.23]
C22	9,010	114,248,510	0.09	0.08	0.07	0.01	0.02	0.02	1.25 [1.16-1.34]*	1.21 [1.13-1.31]*
C23	1,320	114,268,220	0.01	0.01	0.01	0.00	0.00	0.00	1.07 [0.88-1.31]	1.07 [0.88-1.30]
C24	2,450	114,261,970	0.02	0.02	0.02	0.00	0.00	0.00	1.09 [0.94-1.25]	1.08 [0.94-1.25]
C25	15,190	114,243,700	0.14	0.13	0.12	0.01	0.02	0.02	1.16 [1.10-1.23]*	1.15 [1.09-1.22]*
C26	3,390	114,256,370	0.03	0.03	0.03	0.00	0.00	0.00	1.13 [1.00-1.28]	1.10 [0.98-1.25]
C30	570	114,264,750	0.00	0.01	0.00	0.00	0.00	0.00	1.07 [0.80-1.43]	1.06 [0.79-1.42]
C32	3,540	114,230,960	0.03	0.03	0.03	0.00	0.00	0.00	1.14 [1.02-1.28]	1.16 [1.03-1.30]
C34	65,460	114,089,990	0.60	0.57	0.52	0.03	0.08	0.07	1.15 [1.12-1.18]*	1.14 [1.11-1.17]*
C38	880	114,266,870	0.01	0.01	0.01	0.00	0.00	0.00	1.07 [0.85-1.35]	1.08 [0.85-1.37]
C40	690	114,261,970	0.01	0.01	0.01	0.00	0.00	0.00	1.05 [0.80-1.37]	1.02 [0.78-1.34]
C41	1,890	114,254,810	0.02	0.02	0.01	0.00	0.00	0.00	1.25 [1.07-1.46]*	1.24 [1.06-1.46]*
C43	18,240	114,055,240	0.14	0.16	0.15	−0.02	−0.01	−0.01	0.95 [0.90-1.01]	0.94 [0.89-1.00]
C44	129,100	112,713,740	1.16	1.14	1.05	0.01	0.10	0.07	1.10 [1.08-1.12]*	1.07 [1.05-1.09]*
C45	4,100	114,262,400	0.03	0.04	0.03	0.00	0.00	0.00	0.98 [0.88-1.10]	0.99 [0.89-1.11]
C48	2,300	114,260,420	0.02	0.02	0.02	0.00	0.00	0.00	1.05 [0.90-1.21]	1.03 [0.89-1.20]
C49	4,240	114,229,950	0.04	0.04	0.03	0.00	0.00	0.00	1.08 [0.97-1.20]	1.06 [0.95-1.18]
C50	77,280	113,050,660	0.66	0.69	0.63	−0.03	0.03	0.02	1.04 [1.02-1.07]*	1.04 [1.01-1.07]*
C51	2,060	114,247,150	0.02	0.02	0.02	0.00	0.00	0.00	1.20 [1.04-1.39]	1.18 [1.02-1.38]
C52	650	114,266,240	0.01	0.01	0.01	0.00	0.00	0.00	0.98 [0.74-1.29]	1.00 [0.76-1.33]
C53	4,290	114,206,450	0.03	0.04	0.04	0.00	0.00	0.00	0.96 [0.86-1.07]	0.97 [0.87-1.09]

C54	12,680	114,108,260	0.11	0.11	0.10	0.00	0.00	0.01	1.04 [0.98-1.11]	1.06 [0.99-1.12]
C55	1,740	114,256,580	0.01	0.02	0.01	0.00	0.00	0.00	1.06 [0.90-1.25]	1.05 [0.88-1.24]
C56	11,460	114,171,690	0.10	0.10	0.09	−0.01	0.00	0.00	1.03 [0.97-1.10]	1.04 [0.97-1.11]
C57	1,270	114,264,240	0.01	0.01	0.01	0.00	0.00	0.00	1.18 [0.97-1.42]	1.20 [0.99-1.46]
C60	980	114,258,200	0.01	0.01	0.01	0.00	0.00	0.00	1.19 [0.95-1.48]	1.20 [0.96-1.50]
C61	72,980	113,592,100	0.63	0.65	0.59	−0.02	0.03	0.02	1.06 [1.03-1.09]*	1.04 [1.01-1.06]*
C62	2,020	114,222,460	0.02	0.02	0.02	0.00	0.00	0.00	1.04 [0.89-1.22]	1.02 [0.87-1.20]
C64	15,190	114,147,330	0.13	0.13	0.12	0.00	0.01	0.01	1.11 [1.05-1.18]*	1.09 [1.03-1.15]*
C65	1,340	114,260,100	0.01	0.01	0.01	0.00	0.00	0.00	1.22 [1.01-1.47]	1.24 [1.02-1.50]
C66	1,870	114,257,520	0.02	0.02	0.01	0.00	0.00	0.00	1.10 [0.93-1.29]	1.09 [0.93-1.29]
C67	33,740	113,869,260	0.29	0.30	0.27	0.00	0.02	0.02	1.08 [1.04-1.12]*	1.07 [1.03-1.12]*
C68	720	114,264,870	0.01	0.01	0.01	0.00	0.00	0.00	1.03 [0.79-1.35]	1.02 [0.78-1.33]
C69	1,400	114,245,660	0.01	0.01	0.01	0.00	0.00	0.00	0.98 [0.81-1.18]	0.96 [0.79-1.16]
C71	8,490	114,216,660	0.08	0.07	0.07	0.00	0.01	0.01	1.12 [1.04-1.21]*	1.11 [1.03-1.19]*
C72	540	114,263,370	0.01	0.00	0.00	0.00	0.00	0.00	1.20 [0.90-1.59]	1.11 [0.82-1.49]
C73	4,310	114,206,790	0.04	0.04	0.04	0.00	0.00	0.00	1.06 [0.96-1.18]	1.04 [0.93-1.16]
C74	690	114,262,330	0.01	0.01	0.01	0.00	0.00	0.00	1.20 [0.93-1.55]	1.19 [0.92-1.54]
C75	720	114,263,440	0.01	0.01	0.01	0.00	0.00	0.00	1.07 [0.83-1.39]	1.04 [0.80-1.36]
C76	2,400	114,252,700	0.02	0.02	0.02	0.00	0.00	0.00	1.10 [0.96-1.27]	1.11 [0.96-1.28]
C77	75,990	113,728,310	0.65	0.67	0.61	−0.02	0.04	0.04	1.06 [1.03-1.09]*	1.06 [1.04-1.09]*
C78	111,980	114,049,850	0.96	0.99	0.90	−0.03	0.06	0.06	1.06 [1.04-1.09]*	1.07 [1.04-1.09]*
C79	94,520	114,075,420	0.82	0.83	0.76	−0.02	0.06	0.06	1.08 [1.05-1.10]*	1.08 [1.06-1.11]*
C80	33,220	114,186,460	0.29	0.29	0.26	0.00	0.03	0.03	1.10 [1.06-1.15]*	1.10 [1.06-1.14]*
C81	3,570	114,211,100	0.04	0.03	0.03	0.02	0.02	0.02	1.71 [1.54-1.90]*	1.64 [1.48-1.83]*
C82	4,030	114,221,070	0.04	0.04	0.03	0.00	0.00	0.00	1.16 [1.04-1.29]*	1.10 [0.99-1.23]
C83	11,500	114,181,920	0.11	0.10	0.09	0.01	0.02	0.02	1.24 [1.17-1.32]*	1.21 [1.13-1.29]*
C84	1,600	114,255,930	0.02	0.01	0.01	0.01	0.01	0.01	2.44 [2.11-2.83]*	2.41 [2.08-2.80]*
C85	14,480	114,111,140	0.14	0.12	0.11	0.02	0.04	0.03	1.33 [1.25-1.40]*	1.28 [1.21-1.35]*
C88	1,900	114,257,780	0.02	0.02	0.01	0.00	0.00	0.00	1.28 [1.10-1.50]*	1.26 [1.08-1.48]*
C90	10,050	114,204,890	0.09	0.09	0.08	0.00	0.01	0.01	1.13 [1.06-1.22]*	1.11 [1.03-1.19]*
C91	10,940	114,159,750	0.10	0.10	0.09	0.00	0.01	0.01	1.07 [1.00-1.15]	1.05 [0.99-1.13]
C92	8,090	114,221,140	0.08	0.07	0.06	0.01	0.02	0.02	1.28 [1.18-1.38]*	1.25 [1.16-1.35]*
C93	1,040	114,268,110	0.01	0.01	0.01	0.00	0.00	0.00	1.17 [0.94-1.46]	1.19 [0.95-1.48]
C94	490	114,268,490	0.00	0.00	0.00	0.00	0.00	0.00	1.25 [0.92-1.70]	1.24 [0.91-1.70]
C95	1,800	114,258,090	0.02	0.02	0.01	0.00	0.00	0.00	1.22 [1.04-1.43]	1.19 [1.02-1.40]
C97	8,550	114,245,010	0.07	0.08	0.07	0.00	0.00	0.00	1.06 [0.98-1.15]	1.04 [0.96-1.12]
D00	940	114,261,430	0.01	0.01	0.01	0.00	0.00	0.00	1.09 [0.87-1.36]	1.05 [0.84-1.32]
D01	950	114,259,340	0.01	0.01	0.01	0.00	0.00	0.00	1.10 [0.88-1.38]	1.04 [0.82-1.30]
D02	940	114,261,100	0.01	0.01	0.01	0.00	0.00	0.00	1.27 [1.02-1.57]	1.23 [0.99-1.54]
D03	6,960	114,184,000	0.06	0.06	0.06	0.00	0.00	0.00	1.06 [0.97-1.15]	1.02 [0.94-1.11]
D04	14,400	114,128,590	0.14	0.12	0.11	0.02	0.03	0.02	1.25 [1.18-1.33]*	1.19 [1.12-1.27]*
D05	12,660	114,042,010	0.10	0.11	0.10	−0.01	0.00	0.00	1.01 [0.95-1.07]	0.99 [0.93-1.06]
D06	10,930	113,899,090	0.09	0.10	0.09	0.00	0.00	0.00	1.05 [0.98-1.12]	1.03 [0.96-1.11]
D07	3,930	114,205,110	0.04	0.03	0.03	0.00	0.01	0.00	1.20 [1.08-1.33]*	1.14 [1.02-1.28]
D09	3,550	114,234,110	0.03	0.03	0.03	0.00	0.00	0.00	1.12 [1.00-1.26]	1.11 [0.98-1.25]
D10	4,090	114,181,840	0.04	0.03	0.03	0.01	0.01	0.01	1.27 [1.15-1.41]*	1.21 [1.09-1.35]*
D11	4,380	114,187,160	0.04	0.04	0.04	0.00	0.00	0.00	1.04 [0.94-1.16]	1.04 [0.93-1.16]
D12	123,720	112,693,960	1.18	1.08	0.98	0.11	0.21	0.15	1.21 [1.19-1.24]*	1.14 [1.12-1.16]*
D13	8,410	114,058,360	0.08	0.07	0.07	0.01	0.01	0.01	1.22 [1.13-1.31]*	1.12 [1.04-1.21]*
D14	2,760	114,213,450	0.03	0.02	0.02	0.00	0.01	0.01	1.31 [1.15-1.48]*	1.23 [1.08-1.40]*
D15	1,060	114,258,150	0.01	0.01	0.01	0.00	0.00	0.00	1.15 [0.93-1.42]	1.14 [0.92-1.41]
D16	4,960	114,176,090	0.05	0.04	0.04	0.01	0.01	0.01	1.18 [1.07-1.29]*	1.14 [1.03-1.25]*
D17	39,130	113,238,760	0.36	0.34	0.31	0.02	0.05	0.03	1.16 [1.12-1.20]*	1.10 [1.07-1.14]*
D18	17,970	113,893,460	0.17	0.15	0.15	0.02	0.02	0.02	1.17 [1.11-1.23]*	1.11 [1.05-1.17]*
D21	4,320	114,179,870	0.04	0.04	0.03	0.01	0.01	0.01	1.28 [1.16-1.42]*	1.23 [1.11-1.36]*
D22	40,170	112,843,030	0.38	0.35	0.33	0.03	0.05	0.03	1.14 [1.10-1.18]*	1.09 [1.05-1.13]*
D23	26,120	113,493,750	0.26	0.22	0.21	0.04	0.05	0.04	1.23 [1.18-1.28]*	1.17 [1.13-1.22]*
D24	13,600	113,927,600	0.12	0.12	0.11	0.00	0.01	0.00	1.05 [0.99-1.11]	1.01 [0.95-1.07]
D25	64,900	112,701,380	0.58	0.58	0.54	0.00	0.04	0.02	1.07 [1.04-1.10]*	1.04 [1.01-1.07]*
D26	2,140	114,210,440	0.02	0.02	0.02	0.00	0.00	0.00	1.12 [0.96-1.29]	1.09 [0.94-1.27]
D27	15,320	113,980,960	0.13	0.13	0.13	0.00	0.00	0.00	1.04 [0.98-1.10]	1.02 [0.96-1.08]
D28	1,610	114,229,980	0.02	0.01	0.01	0.00	0.00	0.00	1.25 [1.06-1.47]*	1.19 [1.00-1.41]
D29	1,270	114,248,200	0.01	0.01	0.01	0.00	0.00	0.00	1.23 [1.02-1.49]	1.19 [0.98-1.45]
D30	2,350	114,239,560	0.02	0.02	0.02	0.00	0.00	0.00	1.11 [0.96-1.28]	1.08 [0.93-1.25]
D31	2,530	114,234,340	0.03	0.02	0.02	0.00	0.01	0.01	1.28 [1.12-1.46]*	1.25 [1.09-1.43]*
D32	8,570	114,191,750	0.08	0.07	0.07	0.00	0.01	0.01	1.13 [1.05-1.22]*	1.09 [1.01-1.18]
D33	3,400	114,221,500	0.03	0.03	0.03	0.00	0.00	0.00	1.13 [1.00-1.27]	1.05 [0.93-1.18]
D34	2,270	114,226,280	0.02	0.02	0.02	0.00	0.00	0.00	1.07 [0.93-1.24]	1.03 [0.88-1.19]
D35	12,650	114,117,610	0.11	0.11	0.10	0.00	0.01	0.01	1.13 [1.06-1.20]*	1.08 [1.02-1.15]
D36	6,150	114,135,610	0.06	0.05	0.05	0.01	0.01	0.01	1.22 [1.12-1.33]*	1.14 [1.04-1.24]*
D37	16,960	114,098,180	0.15	0.15	0.14	0.00	0.01	0.01	1.10 [1.04-1.16]*	1.07 [1.01-1.13]
D38	4,000	114,241,040	0.04	0.03	0.03	0.00	0.01	0.01	1.22 [1.10-1.36]*	1.20 [1.08-1.34]*
D39	2,470	114,228,430	0.02	0.02	0.02	0.00	0.00	0.00	1.06 [0.92-1.22]	1.06 [0.92-1.22]
D40	830	114,255,000	0.01	0.01	0.01	0.00	0.00	0.00	1.12 [0.88-1.43]	1.11 [0.87-1.43]
D41	11,860	114,134,340	0.10	0.10	0.10	0.00	0.01	0.01	1.09 [1.02-1.16]*	1.07 [1.00-1.15]
D43	4,130	114,235,150	0.04	0.04	0.03	0.00	0.00	0.00	1.13 [1.02-1.26]	1.11 [0.99-1.23]
D44	2,470	114,232,440	0.02	0.02	0.02	0.00	0.00	0.00	1.17 [1.02-1.34]	1.10 [0.96-1.27]
D45	4,420	114,199,650	0.04	0.04	0.03	0.00	0.00	0.00	1.10 [0.99-1.23]	1.09 [0.98-1.21]
D46	10,260	114,218,650	0.10	0.09	0.08	0.02	0.03	0.02	1.32 [1.24-1.42]*	1.29 [1.20-1.38]*
D47	16,650	114,171,230	0.17	0.14	0.13	0.03	0.03	0.03	1.26 [1.20-1.33]*	1.20 [1.13-1.26]*
D48	8,690	114,113,920	0.08	0.08	0.07	0.00	0.01	0.00	1.09 [1.01-1.17]	1.05 [0.97-1.13]
any age - D50-D89										
D50	231,860	112,083,610	2.46	1.97	1.86	0.49	0.60	0.49	1.32 [1.31-1.34]*	1.25 [1.23-1.27]*
D51	20,450	114,061,640	0.21	0.17	0.16	0.04	0.06	0.05	1.38 [1.32-1.44]*	1.29 [1.23-1.35]*
D52	15,540	114,194,900	0.16	0.13	0.12	0.03	0.03	0.03	1.28 [1.21-1.35]*	1.23 [1.17-1.31]*
D53	4,190	114,241,410	0.05	0.03	0.03	0.01	0.01	0.01	1.42 [1.28-1.58]*	1.34 [1.20-1.49]*
D55	700	114,258,530	0.01	0.01	0.01	0.00	0.00	0.00	1.50 [1.17-1.91]*	1.47 [1.14-1.89]*
D56	8,860	114,122,140	0.10	0.07	0.07	0.03	0.03	0.03	1.40 [1.31-1.50]*	1.37 [1.27-1.47]*
D57	10,180	114,052,440	0.12	0.08	0.09	0.03	0.03	0.03	1.31 [1.23-1.40]*	1.28 [1.20-1.37]*
D58	2,470	114,231,070	0.02	0.02	0.02	0.00	0.00	0.00	1.21 [1.05-1.38]*	1.19 [1.04-1.37]
D59	3,080	114,232,240	0.03	0.03	0.02	0.01	0.01	0.01	1.44 [1.28-1.62]*	1.42 [1.26-1.59]*
D61	14,570	114,194,810	0.15	0.12	0.11	0.03	0.04	0.03	1.31 [1.24-1.39]*	1.27 [1.20-1.35]*
D62	7,010	114,145,740	0.07	0.06	0.06	0.00	0.01	0.01	1.13 [1.04-1.23]*	1.12 [1.03-1.22]*
D63	22,310	114,142,120	0.22	0.19	0.17	0.03	0.05	0.04	1.28 [1.23-1.34]*	1.24 [1.19-1.30]*
D64	308,600	111,309,680	3.23	2.66	2.50	0.57	0.74	0.63	1.30 [1.28-1.31]*	1.24 [1.23-1.26]*
D65	1,060	114,264,970	0.01	0.01	0.01	0.00	0.00	0.00	1.19 [0.96-1.46]	1.17 [0.94-1.44]
D66	1,140	114,244,450	0.01	0.01	0.01	0.00	0.00	0.00	1.15 [0.94-1.40]	1.11 [0.90-1.36]
D68	17,780	114,016,940	0.18	0.15	0.14	0.03	0.04	0.03	1.29 [1.22-1.35]*	1.19 [1.13-1.25]*
D69	53,400	113,746,430	0.54	0.45	0.44	0.09	0.10	0.09	1.23 [1.20-1.27]*	1.20 [1.16-1.23]*
D70	45,520	113,893,820	0.45	0.39	0.36	0.06	0.08	0.08	1.23 [1.19-1.27]*	1.20 [1.16-1.24]*
D72	9,910	114,193,710	0.11	0.08	0.08	0.03	0.03	0.03	1.43 [1.34-1.53]*	1.31 [1.22-1.40]*
D73	10,330	114,177,740	0.10	0.09	0.08	0.01	0.02	0.02	1.25 [1.17-1.34]*	1.20 [1.12-1.28]*
D75	12,720	114,139,920	0.12	0.11	0.10	0.01	0.02	0.02	1.21 [1.14-1.29]*	1.17 [1.10-1.24]*
D76	940	114,250,730	0.01	0.01	0.01	0.00	0.00	0.00	1.27 [1.02-1.57]	1.20 [0.97-1.50]
D80	2,970	114,229,710	0.04	0.02	0.02	0.01	0.01	0.01	1.54 [1.37-1.73]*	1.36 [1.20-1.53]*
D82	790	114,256,460	0.01	0.01	0.01	0.00	0.00	0.00	1.31 [1.04-1.65]	1.19 [0.94-1.51]
D83	360	114,264,620	0.01	0.00	0.00	0.00	0.00	0.00	2.01 [1.46-2.75]*	1.76 [1.27-2.43]*
D84	1,610	114,247,990	0.02	0.01	0.01	0.01	0.01	0.01	1.71 [1.47-1.99]*	1.43 [1.22-1.69]*

D86	6,350	114,175,010	0.07	0.05	0.05	0.02	0.03	0.02	1.58 [1.46-1.71]*	1.47 [1.35-1.59]*
D89	5,210	114,223,090	0.06	0.04	0.04	0.02	0.02	0.01	1.43 [1.31-1.57]*	1.33 [1.21-1.46]*
any age - E00-E89										
E02	1,930	114,261,430	0.02	0.02	0.02	0.00	0.00	0.00	1.14 [0.97-1.35]	1.12 [0.95-1.33]
E03	222,190	111,509,480	2.35	1.91	1.76	0.45	0.60	0.51	1.34 [1.32-1.36]*	1.28 [1.26-1.30]*
E04	23,040	113,951,780	0.21	0.20	0.19	0.01	0.03	0.02	1.15 [1.10-1.20]*	1.09 [1.04-1.14]*
E05	37,470	113,814,430	0.38	0.32	0.29	0.06	0.09	0.07	1.29 [1.25-1.34]*	1.23 [1.18-1.27]*
E06	4,130	114,212,330	0.04	0.04	0.03	0.01	0.01	0.01	1.22 [1.10-1.35]*	1.17 [1.05-1.30]*
E07	6,400	114,196,100	0.06	0.05	0.05	0.01	0.01	0.01	1.26 [1.16-1.37]*	1.17 [1.07-1.27]*
E10	48,670	113,231,490	0.49	0.42	0.40	0.07	0.09	0.06	1.23 [1.19-1.26]*	1.15 [1.12-1.19]*
E11	344,180	109,746,160	3.53	3.04	2.81	0.49	0.72	0.56	1.26 [1.24-1.27]*	1.19 [1.18-1.20]*
E13	4,480	114,235,960	0.05	0.04	0.03	0.01	0.01	0.01	1.41 [1.28-1.55]*	1.25 [1.13-1.39]*
E14	48,270	113,678,920	0.51	0.40	0.37	0.10	0.14	0.10	1.37 [1.33-1.41]*	1.25 [1.22-1.29]*
E16	57,430	113,875,050	0.58	0.49	0.46	0.09	0.12	0.09	1.25 [1.22-1.29]*	1.18 [1.14-1.21]*
E20	1,540	114,255,280	0.02	0.01	0.01	0.00	0.01	0.00	1.44 [1.21-1.70]*	1.26 [1.06-1.50]*
E21	17,410	114,133,900	0.16	0.15	0.14	0.01	0.02	0.02	1.17 [1.11-1.23]*	1.12 [1.06-1.18]*
E22	11,740	114,178,290	0.13	0.10	0.09	0.04	0.04	0.04	1.48 [1.39-1.57]*	1.41 [1.32-1.50]*
E23	6,800	114,166,710	0.08	0.06	0.05	0.02	0.02	0.02	1.39 [1.28-1.50]*	1.29 [1.19-1.40]*
E24	1,380	114,247,680	0.02	0.01	0.01	0.01	0.01	0.00	1.60 [1.35-1.90]*	1.32 [1.10-1.58]*
E26	810	114,257,610	0.01	0.01	0.01	0.00	0.00	0.00	1.21 [0.95-1.54]	1.13 [0.88-1.44]
E27	12,740	114,155,760	0.14	0.10	0.10	0.04	0.05	0.04	1.49 [1.40-1.57]*	1.34 [1.26-1.42]*
E28	31,970	113,839,200	0.33	0.27	0.27	0.06	0.06	0.03	1.21 [1.17-1.26]*	1.11 [1.07-1.15]*
E29	1,780	114,247,310	0.02	0.01	0.01	0.01	0.01	0.01	1.71 [1.47-1.98]*	1.50 [1.28-1.75]*
E30	3,170	114,225,040	0.04	0.03	0.03	0.01	0.01	0.01	1.24 [1.11-1.39]*	1.19 [1.06-1.34]*
E34	6,000	114,180,060	0.07	0.05	0.06	0.02	0.01	0.01	1.23 [1.13-1.34]*	1.14 [1.05-1.24]*
E41	650	114,268,540	0.01	0.01	0.01	0.00	0.00	0.00	1.07 [0.80-1.42]	1.10 [0.82-1.47]
E43	750	114,267,340	0.01	0.01	0.01	0.00	0.00	0.00	1.20 [0.94-1.54]	1.18 [0.92-1.53]
E46	9,660	114,225,920	0.09	0.08	0.08	0.01	0.02	0.01	1.22 [1.13-1.31]*	1.19 [1.11-1.28]*
E51	930	114,261,790	0.01	0.01	0.01	0.00	0.00	0.00	1.47 [1.19-1.82]*	1.39 [1.12-1.73]*
E53	54,380	113,982,430	0.56	0.46	0.43	0.10	0.13	0.11	1.30 [1.27-1.34]*	1.23 [1.20-1.27]*
E55	74,830	113,919,860	0.83	0.62	0.60	0.21	0.22	0.17	1.37 [1.34-1.40]*	1.26 [1.23-1.29]*
E56	780	114,264,910	0.01	0.01	0.01	0.00	0.00	0.00	1.27 [1.00-1.62]	1.19 [0.93-1.53]
E58	550	114,266,570	0.01	0.00	0.00	0.00	0.00	0.00	1.55 [1.18-2.04]*	1.48 [1.11-1.95]*
E61	11,560	114,197,480	0.13	0.09	0.09	0.03	0.04	0.03	1.38 [1.30-1.47]*	1.26 [1.18-1.34]*
E63	710	114,265,240	0.01	0.01	0.01	0.00	0.00	0.00	1.30 [1.01-1.67]	1.24 [0.96-1.60]
E65	480	114,251,420	0.01	0.00	0.00	0.00	0.00	0.00	1.45 [1.08-1.95]	1.33 [0.98-1.80]
E66	389,760	110,758,960	4.14	3.37	3.17	0.77	0.97	0.69	1.30 [1.29-1.32]*	1.20 [1.19-1.21]*
E70	510	114,255,830	0.01	0.00	0.00	0.00	0.00	0.00	1.35 [1.02-1.79]	1.30 [0.98-1.74]
E72	880	114,255,440	0.01	0.01	0.01	0.00	0.00	0.00	1.17 [0.93-1.46]	1.09 [0.87-1.37]
E73	4,380	114,193,970	0.05	0.04	0.04	0.02	0.02	0.01	1.41 [1.28-1.55]*	1.22 [1.11-1.35]*
E74	1,520	114,247,230	0.02	0.01	0.01	0.00	0.00	0.00	1.35 [1.14-1.60]*	1.24 [1.05-1.48]
E75	650	114,260,440	0.01	0.01	0.01	0.00	0.00	0.00	1.20 [0.93-1.55]	1.17 [0.90-1.52]
E78	448,700	108,519,310	4.62	4.02	3.76	0.61	0.87	0.68	1.23 [1.22-1.24]*	1.17 [1.16-1.18]*
E79	610	114,264,990	0.01	0.01	0.00	0.00	0.00	0.00	1.32 [1.01-1.72]	1.25 [0.95-1.65]
E80	6,170	114,191,470	0.06	0.05	0.05	0.01	0.01	0.01	1.17 [1.07-1.28]*	1.09 [1.00-1.20]
E83	83,760	113,857,720	0.82	0.72	0.67	0.10	0.14	0.11	1.21 [1.18-1.24]*	1.16 [1.13-1.19]*
E84	720	114,234,860	0.01	0.01	0.01	0.00	0.00	0.00	1.23 [0.96-1.58]	1.11 [0.86-1.43]
E85	3,060	114,252,710	0.03	0.03	0.02	0.00	0.01	0.00	1.25 [1.10-1.41]*	1.20 [1.06-1.36]*
E86	222,050	113,007,960	2.19	1.91	1.84	0.28	0.36	0.31	1.20 [1.18-1.21]*	1.16 [1.14-1.18]*
E87	330,700	112,670,290	3.40	2.82	2.65	0.58	0.75	0.64	1.28 [1.27-1.30]*	1.23 [1.22-1.25]*
E88	16,000	114,181,140	0.16	0.13	0.13	0.03	0.04	0.03	1.29 [1.23-1.36]*	1.23 [1.17-1.30]*
E89	21,020	114,047,640	0.21	0.18	0.17	0.03	0.04	0.03	1.25 [1.19-1.31]*	1.17 [1.12-1.23]*
any age - F01-F99										
F00	51,390	114,115,300	0.46	0.45	0.44	0.02	0.03	0.03	1.06 [1.03-1.10]*	1.06 [1.03-1.10]*
F01	58,730	114,084,850	0.54	0.51	0.49	0.03	0.05	0.04	1.10 [1.07-1.13]*	1.08 [1.05-1.12]*
F02	10,210	114,238,310	0.09	0.09	0.09	0.00	0.00	0.00	1.01 [0.94-1.09]	1.02 [0.95-1.10]
F03	141,060	113,801,810	1.29	1.23	1.20	0.06	0.09	0.08	1.07 [1.05-1.09]*	1.07 [1.05-1.09]*
F05	129,730	113,952,600	1.25	1.11	1.07	0.14	0.18	0.16	1.17 [1.15-1.20]*	1.14 [1.12-1.17]*
F06	20,420	114,173,640	0.19	0.18	0.17	0.02	0.03	0.02	1.16 [1.10-1.22]*	1.13 [1.07-1.19]*
F07	1,390	114,248,780	0.01	0.01	0.01	0.00	0.00	0.00	1.28 [1.07-1.53]*	1.18 [0.99-1.42]
F10	111,920	112,396,930	1.08	0.98	0.90	0.10	0.18	0.16	1.20 [1.18-1.23]*	1.17 [1.15-1.19]*
F11	8,350	114,034,480	0.08	0.07	0.07	0.00	0.01	0.01	1.17 [1.08-1.26]*	1.08 [1.00-1.17]
F12	18,400	114,061,480	0.17	0.16	0.16	0.01	0.01	0.00	1.08 [1.03-1.14]*	1.01 [0.96-1.07]
F13	1,870	114,240,750	0.02	0.02	0.02	0.00	0.00	0.00	1.26 [1.08-1.47]*	1.14 [0.97-1.33]
F14	7,220	114,174,230	0.07	0.06	0.06	0.00	0.01	0.00	1.12 [1.03-1.21]*	1.04 [0.95-1.13]
F15	2,620	114,221,740	0.02	0.02	0.02	0.00	0.00	0.00	1.03 [0.90-1.19]	0.97 [0.85-1.12]
F16	800	114,251,510	0.01	0.01	0.01	0.00	0.00	0.00	0.95 [0.73-1.22]	0.91 [0.70-1.19]
F17	533,320	109,366,710	5.14	4.81	4.55	0.32	0.58	0.51	1.13 [1.12-1.14]*	1.11 [1.10-1.12]*
F19	7,920	114,137,290	0.07	0.07	0.07	0.00	0.00	0.00	1.07 [0.99-1.16]	1.00 [0.92-1.09]
F20	15,750	113,872,320	0.16	0.13	0.12	0.02	0.03	0.03	1.28 [1.21-1.34]*	1.26 [1.19-1.33]*
F22	7,080	114,184,140	0.07	0.06	0.06	0.01	0.02	0.01	1.29 [1.19-1.40]*	1.23 [1.14-1.34]*
F23	4,440	114,180,250	0.04	0.04	0.04	0.00	0.01	0.00	1.16 [1.05-1.29]*	1.13 [1.02-1.26]
F25	3,270	114,184,600	0.03	0.03	0.03	0.01	0.01	0.01	1.36 [1.21-1.52]*	1.32 [1.17-1.49]*
F29	8,190	114,172,850	0.08	0.07	0.07	0.01	0.01	0.01	1.22 [1.13-1.32]*	1.16 [1.07-1.25]*
F30	2,140	114,212,070	0.02	0.02	0.02	0.00	0.01	0.00	1.34 [1.16-1.54]*	1.28 [1.11-1.48]*
F31	19,010	113,948,710	0.20	0.16	0.15	0.04	0.05	0.03	1.31 [1.24-1.37]*	1.19 [1.13-1.25]*
F32	352,560	110,591,970	3.90	3.02	2.84	0.88	1.05	0.74	1.37 [1.35-1.38]*	1.23 [1.22-1.25]*
F33	10,900	114,015,750	0.11	0.09	0.08	0.02	0.03	0.02	1.31 [1.23-1.39]*	1.18 [1.10-1.26]*
F34	1,830	114,242,170	0.02	0.02	0.01	0.01	0.01	0.00	1.44 [1.24-1.68]*	1.25 [1.06-1.46]*
F38	880	114,261,580	0.01	0.01	0.01	0.00	0.00	0.00	1.30 [1.04-1.64]	1.19 [0.94-1.51]
F39	1,550	114,252,080	0.02	0.01	0.01	0.00	0.00	0.00	1.33 [1.12-1.57]*	1.20 [1.00-1.42]
F40	34,110	113,960,660	0.36	0.28	0.29	0.08	0.07	0.05	1.26 [1.21-1.30]*	1.16 [1.12-1.21]*
F41	301,930	112,056,780	3.29	2.55	2.45	0.73	0.83	0.56	1.34 [1.32-1.36]*	1.20 [1.19-1.22]*
F42	7,540	114,189,110	0.10	0.06	0.06	0.04	0.04	0.03	1.64 [1.52-1.76]*	1.43 [1.32-1.54]*
F43	17,280	114,012,180	0.18	0.15	0.14	0.03	0.04	0.02	1.26 [1.20-1.33]*	1.11 [1.05-1.17]*
F44	4,140	114,219,250	0.05	0.03	0.03	0.01	0.01	0.01	1.40 [1.26-1.55]*	1.21 [1.09-1.34]*
F45	4,960	114,184,440	0.06	0.04	0.04	0.01	0.02	0.01	1.45 [1.32-1.59]*	1.28 [1.16-1.41]*
F48	1,360	114,251,430	0.02	0.01	0.01	0.00	0.01	0.00	1.55 [1.30-1.84]*	1.35 [1.12-1.62]*
F50	8,450	114,155,800	0.09	0.07	0.07	0.02	0.02	0.01	1.28 [1.19-1.37]*	1.17 [1.08-1.26]*
F51	1,780	114,250,060	0.02	0.01	0.01	0.01	0.01	0.00	1.43 [1.23-1.67]*	1.30 [1.11-1.52]*
F52	2,710	114,223,540	0.03	0.02	0.02	0.01	0.01	0.01	1.49 [1.32-1.69]*	1.35 [1.19-1.53]*
F53	2,660	114,222,820	0.03	0.02	0.02	0.01	0.01	0.01	1.35 [1.19-1.53]*	1.24 [1.08-1.41]*
F60	14,190	114,029,770	0.15	0.12	0.12	0.04	0.04	0.02	1.34 [1.27-1.42]*	1.15 [1.09-1.22]*
F61	540	114,260,920	0.01	0.00	0.00	0.00	0.00	0.00	1.13 [0.85-1.51]	0.99 [0.73-1.35]
F69	1,170	114,247,550	0.01	0.01	0.01	0.00	0.00	0.00	1.47 [1.22-1.77]*	1.27 [1.05-1.55]
F70	2,200	114,226,460	0.03	0.02	0.02	0.01	0.01	0.01	1.72 [1.51-1.97]*	1.54 [1.34-1.76]*
F71	850	114,252,140	0.01	0.01	0.01	0.01	0.01	0.01	2.16 [1.76-2.65]*	1.93 [1.56-2.39]*
F72	1,670	114,238,650	0.02	0.01	0.01	0.01	0.01	0.01	1.67 [1.43-1.94]*	1.51 [1.29-1.76]*
F79	10,330	114,131,310	0.13	0.08	0.08	0.05	0.05	0.04	1.56 [1.47-1.66]*	1.44 [1.35-1.54]*
F80	11,880	114,137,020	0.13	0.10	0.11	0.03	0.02	0.02	1.22 [1.15-1.29]*	1.16 [1.10-1.24]*
F81	30,440	113,772,470	0.38	0.24	0.25	0.14	0.14	0.12	1.56 [1.50-1.61]*	1.44 [1.39-1.50]*
F82	1,680	114,245,650	0.02	0.01	0.02	0.00	0.00	0.00	1.19 [1.02-1.39]	1.11 [0.95-1.31]
F83	1,530	114,253,820	0.01	0.01	0.02	0.00	0.00	0.00	0.95 [0.80-1.13]	0.89 [0.74-1.06]
F84	28,810	113,981,400	0.36	0.23	0.27	0.13	0.09	0.06	1.32 [1.28-1.38]*	1.18 [1.14-1.23]*
F89	7,340	114,209,390	0.08	0.06	0.07	0.01	0.00	0.00	1.02 [0.95-1.10]	0.94 [0.87-1.02]
F90	14,870	114,132,570	0.18	0.12	0.15	0.06	0.03	0.02	1.22 [1.15-1.28]*	1.12 [1.06-1.19]*
F91	3,210	114,211,000	0.04	0.03	0.03	0.01	0.01	0.01	1.27 [1.13-1.42]*	1.19 [1.06-1.34]*

F94	610	114,265,240	0.01	0.00	0.01	0.00	0.00	0.00	1.22 [0.94-1.58]	1.15 [0.88-1.51]
F95	1,710	114,253,610	0.02	0.01	0.02	0.01	0.01	0.00	1.38 [1.18-1.61]*	1.23 [1.05-1.45]
F98	2,190	114,238,850	0.03	0.02	0.02	0.01	0.01	0.00	1.32 [1.15-1.52]*	1.22 [1.06-1.41]*
F99	5,910	114,147,030	0.06	0.05	0.05	0.01	0.01	0.01	1.24 [1.14-1.36]*	1.16 [1.06-1.27]*
any age - G00-G99										
G00	1,720	114,207,870	0.02	0.01	0.01	0.00	0.00	0.00	1.12 [0.95-1.31]	1.07 [0.90-1.26]
G01	910	114,232,140	0.01	0.01	0.01	0.00	0.00	0.00	1.11 [0.89-1.39]	1.06 [0.84-1.33]
G02	780	114,245,840	0.01	0.01	0.01	0.00	0.00	0.00	1.08 [0.85-1.39]	0.98 [0.76-1.27]
G03	2,130	114,211,870	0.02	0.02	0.02	0.00	0.00	0.00	1.24 [1.07-1.43]*	1.15 [1.00-1.34]
G04	4,690	114,217,390	0.05	0.04	0.04	0.01	0.01	0.01	1.25 [1.13-1.38]*	1.19 [1.08-1.32]*
G05	1,210	114,254,830	0.01	0.01	0.01	0.00	0.00	0.00	1.42 [1.17-1.71]*	1.37 [1.14-1.66]*
G06	2,080	114,243,350	0.02	0.02	0.02	0.00	0.00	0.00	1.25 [1.08-1.45]*	1.20 [1.03-1.40]
G08	1,160	114,256,880	0.01	0.01	0.01	0.00	0.00	0.00	1.16 [0.95-1.42]	1.12 [0.92-1.38]
G09	1,310	114,246,960	0.01	0.01	0.01	0.00	0.00	0.00	1.36 [1.14-1.63]*	1.28 [1.06-1.54]*
G10	600	114,262,480	0.01	0.01	0.00	0.00	0.00	0.00	1.40 [1.07-1.84]	1.42 [1.08-1.87]
G11	2,160	114,242,600	0.02	0.02	0.02	0.00	0.00	0.00	1.17 [1.01-1.36]	1.13 [0.97-1.31]
G12	4,500	114,245,650	0.04	0.04	0.04	0.00	0.00	0.00	1.08 [0.97-1.20]	1.04 [0.93-1.15]
G20	39,140	114,013,930	0.34	0.34	0.32	0.00	0.02	0.02	1.06 [1.02-1.09]*	1.05 [1.01-1.09]
G21	3,400	114,252,110	0.04	0.03	0.03	0.01	0.01	0.01	1.34 [1.19-1.50]*	1.31 [1.17-1.48]*
G23	2,110	114,261,620	0.02	0.02	0.02	0.00	0.00	0.00	1.17 [1.00-1.36]	1.14 [0.97-1.33]
G24	4,860	114,205,480	0.05	0.04	0.04	0.01	0.01	0.00	1.14 [1.03-1.25]*	1.09 [0.98-1.20]
G25	22,100	114,103,630	0.22	0.19	0.18	0.04	0.05	0.03	1.28 [1.23-1.34]*	1.16 [1.11-1.22]*
G30	68,860	114,028,030	0.62	0.60	0.58	0.02	0.04	0.04	1.07 [1.04-1.10]*	1.06 [1.03-1.09]*
G31	49,740	114,035,670	0.47	0.43	0.40	0.04	0.07	0.06	1.17 [1.13-1.21]*	1.14 [1.10-1.18]*
G32	790	114,263,120	0.01	0.01	0.01	0.00	0.00	0.00	1.39 [1.10-1.76]*	1.30 [1.02-1.66]
G35	10,520	114,020,700	0.10	0.09	0.08	0.01	0.02	0.01	1.23 [1.15-1.31]*	1.16 [1.08-1.24]*
G37	2,930	114,209,940	0.03	0.02	0.02	0.00	0.01	0.01	1.28 [1.14-1.45]*	1.22 [1.07-1.38]*
G40	75,530	112,792,190	0.80	0.64	0.61	0.16	0.19	0.14	1.31 [1.28-1.34]*	1.21 [1.18-1.24]*
G41	5,770	114,183,590	0.06	0.05	0.05	0.01	0.01	0.01	1.20 [1.10-1.31]*	1.13 [1.04-1.24]*
G43	70,130	113,469,880	0.75	0.59	0.57	0.17	0.18	0.11	1.32 [1.28-1.35]*	1.16 [1.13-1.19]*
G44	10,050	114,123,630	0.11	0.08	0.08	0.02	0.03	0.02	1.33 [1.24-1.42]*	1.20 [1.12-1.28]*
G45	47,400	113,709,290	0.45	0.41	0.38	0.04	0.07	0.05	1.18 [1.15-1.22]*	1.14 [1.10-1.17]*
G46	3,110	114,250,730	0.03	0.03	0.02	0.00	0.01	0.00	1.22 [1.07-1.38]*	1.19 [1.04-1.35]*
G47	90,940	113,138,040	1.09	0.74	0.74	0.35	0.35	0.27	1.48 [1.45-1.51]*	1.32 [1.30-1.35]*
G50	7,720	114,178,330	0.08	0.06	0.06	0.02	0.02	0.02	1.42 [1.32-1.53]*	1.29 [1.20-1.39]*
G51	17,200	114,044,900	0.17	0.15	0.14	0.02	0.03	0.02	1.21 [1.15-1.28]*	1.15 [1.09-1.21]*
G52	1,270	114,255,190	0.01	0.01	0.01	0.00	0.00	0.00	1.23 [1.02-1.49]	1.17 [0.97-1.42]
G53	2,700	114,248,480	0.03	0.02	0.02	0.01	0.01	0.01	1.33 [1.17-1.52]*	1.26 [1.10-1.44]*
G54	4,900	114,205,020	0.05	0.04	0.04	0.01	0.01	0.01	1.33 [1.21-1.46]*	1.21 [1.10-1.33]*
G55	55,280	113,576,570	0.55	0.47	0.43	0.07	0.12	0.08	1.27 [1.24-1.31]*	1.17 [1.14-1.21]*
G56	82,090	112,484,540	0.85	0.70	0.63	0.15	0.22	0.16	1.35 [1.32-1.38]*	1.24 [1.21-1.27]*
G57	9,610	114,099,740	0.10	0.08	0.07	0.02	0.03	0.02	1.39 [1.30-1.48]*	1.28 [1.19-1.37]*
G58	4,940	114,196,590	0.05	0.04	0.04	0.01	0.01	0.01	1.32 [1.20-1.45]*	1.20 [1.09-1.32]*
G59	3,720	114,223,160	0.04	0.03	0.03	0.01	0.01	0.01	1.49 [1.34-1.65]*	1.31 [1.18-1.47]*
G60	2,360	114,238,510	0.02	0.02	0.02	0.00	0.01	0.00	1.28 [1.11-1.46]*	1.17 [1.02-1.34]
G61	2,940	114,221,830	0.03	0.03	0.02	0.00	0.01	0.00	1.25 [1.11-1.41]*	1.21 [1.07-1.37]*
G62	30,530	114,039,910	0.32	0.26	0.24	0.06	0.08	0.06	1.35 [1.30-1.40]*	1.23 [1.18-1.28]*
G63	15,950	114,162,100	0.18	0.13	0.12	0.04	0.05	0.04	1.45 [1.38-1.53]*	1.30 [1.23-1.37]*
G70	2,910	114,230,380	0.03	0.03	0.02	0.00	0.00	0.00	1.12 [0.99-1.28]	1.07 [0.94-1.22]
G71	2,590	114,224,610	0.03	0.02	0.02	0.00	0.00	0.00	1.18 [1.04-1.35]	1.15 [1.00-1.31]
G72	3,460	114,233,280	0.04	0.03	0.03	0.01	0.01	0.01	1.41 [1.26-1.58]*	1.32 [1.18-1.48]*
G80	8,700	114,046,970	0.08	0.08	0.08	0.00	0.00	0.00	1.01 [0.94-1.09]	0.96 [0.89-1.04]
G81	56,170	113,743,260	0.53	0.48	0.45	0.05	0.08	0.07	1.17 [1.14-1.20]*	1.14 [1.11-1.18]*
G82	6,180	114,160,580	0.06	0.05	0.05	0.00	0.01	0.01	1.15 [1.05-1.25]*	1.10 [1.01-1.20]
G83	8,700	114,178,200	0.08	0.07	0.07	0.01	0.01	0.01	1.21 [1.12-1.30]*	1.15 [1.06-1.23]*
G90	4,610	114,223,450	0.05	0.04	0.04	0.01	0.01	0.01	1.26 [1.15-1.40]*	1.17 [1.05-1.29]*
G91	11,190	114,128,260	0.11	0.10	0.09	0.01	0.02	0.01	1.17 [1.10-1.25]*	1.15 [1.08-1.23]*
G93	45,820	113,861,400	0.46	0.39	0.37	0.08	0.10	0.07	1.26 [1.22-1.30]*	1.17 [1.13-1.21]*
G94	1,690	114,257,180	0.02	0.01	0.01	0.00	0.00	0.00	1.18 [1.00-1.39]	1.17 [0.99-1.38]
G95	12,040	114,156,690	0.11	0.10	0.10	0.01	0.02	0.01	1.19 [1.12-1.27]*	1.14 [1.07-1.21]*
G96	3,850	114,217,310	0.04	0.03	0.03	0.01	0.01	0.01	1.30 [1.17-1.44]*	1.20 [1.07-1.34]*
G97	3,740	114,211,560	0.04	0.03	0.03	0.01	0.01	0.00	1.22 [1.09-1.36]*	1.11 [0.99-1.24]
G98	1,280	114,261,690	0.01	0.01	0.01	0.00	0.00	0.00	1.49 [1.25-1.79]*	1.33 [1.10-1.60]*
G99	18,300	114,091,180	0.18	0.16	0.14	0.02	0.04	0.03	1.26 [1.20-1.33]*	1.18 [1.12-1.24]*
any age - H00-H59										
H00	17,570	113,819,440	0.18	0.15	0.15	0.04	0.04	0.03	1.25 [1.19-1.31]*	1.20 [1.14-1.27]*
H01	11,540	114,128,780	0.13	0.10	0.09	0.03	0.04	0.03	1.42 [1.33-1.51]*	1.33 [1.25-1.42]*
H02	60,580	113,228,740	0.62	0.51	0.48	0.11	0.15	0.12	1.31 [1.27-1.34]*	1.24 [1.21-1.28]*
H03	890	114,253,930	0.01	0.01	0.01	0.00	0.00	0.00	1.61 [1.30-1.98]*	1.50 [1.20-1.86]*
H04	30,700	113,743,620	0.33	0.25	0.24	0.08	0.10	0.08	1.40 [1.35-1.45]*	1.30 [1.25-1.35]*
H05	11,070	114,051,510	0.13	0.09	0.09	0.04	0.04	0.03	1.40 [1.32-1.49]*	1.36 [1.28-1.44]*
H06	1,190	114,251,230	0.01	0.01	0.01	0.00	0.00	0.00	1.49 [1.23-1.79]*	1.43 [1.18-1.73]*
H10	17,430	113,936,020	0.20	0.14	0.14	0.06	0.06	0.05	1.43 [1.36-1.50]*	1.34 [1.27-1.41]*
H11	12,680	114,076,650	0.13	0.11	0.10	0.02	0.02	0.02	1.24 [1.16-1.31]*	1.19 [1.12-1.26]*
H13	980	114,251,940	0.01	0.01	0.01	0.00	0.00	0.00	1.47 [1.20-1.80]*	1.37 [1.11-1.69]*
H15	1,150	114,253,710	0.01	0.01	0.01	0.00	0.00	0.00	1.41 [1.17-1.71]*	1.31 [1.08-1.60]*
H16	5,520	114,185,990	0.07	0.04	0.04	0.03	0.03	0.03	1.71 [1.57-1.86]*	1.60 [1.47-1.75]*
H17	3,180	114,231,140	0.04	0.03	0.03	0.01	0.01	0.01	1.41 [1.26-1.59]*	1.34 [1.19-1.51]*
H18	21,950	114,067,940	0.24	0.18	0.17	0.06	0.07	0.06	1.38 [1.32-1.44]*	1.31 [1.25-1.37]*
H19	1,390	114,252,480	0.02	0.01	0.01	0.01	0.01	0.01	1.81 [1.53-2.14]*	1.60 [1.34-1.90]*
H20	5,960	114,181,370	0.07	0.05	0.05	0.02	0.02	0.02	1.43 [1.32-1.56]*	1.38 [1.27-1.50]*
H21	9,290	114,178,110	0.09	0.08	0.07	0.01	0.01	0.01	1.18 [1.10-1.27]*	1.14 [1.06-1.23]*
H22	440	114,265,760	0.01	0.00	0.00	0.00	0.00	0.00	1.46 [1.07-1.99]	1.40 [1.02-1.91]
H25	220,680	111,840,070	2.14	1.93	1.83	0.21	0.31	0.25	1.17 [1.15-1.19]*	1.13 [1.12-1.15]*
H26	311,990	110,390,570	3.15	2.75	2.61	0.40	0.55	0.45	1.21 [1.19-1.23]*	1.17 [1.15-1.18]*
H27	4,460	114,201,180	0.04	0.04	0.04	0.00	0.01	0.01	1.19 [1.08-1.32]*	1.16 [1.05-1.29]*
H28	1,630	114,251,990	0.02	0.01	0.01	0.00	0.00	0.00	1.32 [1.12-1.55]*	1.24 [1.05-1.47]
H30	530	114,261,530	0.01	0.00	0.00	0.00	0.00	0.00	1.33 [1.00-1.76]	1.26 [0.94-1.68]
H31	5,310	114,220,520	0.05	0.05	0.04	0.00	0.00	0.00	1.11 [1.01-1.22]	1.09 [0.99-1.20]
H33	21,100	113,879,380	0.18	0.19	0.17	−0.01	0.01	0.01	1.05 [1.00-1.10]	1.03 [0.98-1.08]
H34	12,590	114,145,760	0.12	0.11	0.10	0.01	0.02	0.01	1.17 [1.10-1.24]*	1.14 [1.07-1.21]*
H35	115,730	113,214,060	1.10	1.00	0.94	0.10	0.16	0.13	1.17 [1.14-1.19]*	1.13 [1.11-1.16]*
H36	45,990	113,813,430	0.45	0.39	0.36	0.06	0.09	0.07	1.25 [1.21-1.29]*	1.18 [1.14-1.22]*
H40	106,080	113,192,320	1.01	0.92	0.86	0.09	0.15	0.13	1.18 [1.15-1.20]*	1.14 [1.12-1.17]*
H43	21,680	114,020,430	0.20	0.19	0.17	0.01	0.02	0.02	1.13 [1.07-1.18]*	1.08 [1.03-1.14]*
H44	7,060	114,161,440	0.07	0.06	0.06	0.01	0.02	0.01	1.30 [1.20-1.40]*	1.24 [1.14-1.34]*
H46	2,150	114,239,310	0.02	0.02	0.02	0.01	0.01	0.01	1.37 [1.19-1.58]*	1.33 [1.15-1.54]*
H47	9,550	114,165,530	0.10	0.08	0.08	0.01	0.01	0.01	1.18 [1.10-1.26]*	1.13 [1.06-1.22]*
H49	6,050	114,196,800	0.05	0.05	0.05	0.00	0.00	0.00	1.06 [0.97-1.16]	1.01 [0.92-1.11]
H50	28,450	113,669,630	0.28	0.24	0.26	0.04	0.02	0.01	1.07 [1.03-1.12]*	1.04 [1.00-1.08]
H51	1,120	114,254,270	0.01	0.01	0.01	0.00	0.00	0.00	1.20 [0.98-1.46]	1.12 [0.91-1.38]
H52	37,710	113,946,940	0.35	0.33	0.31	0.02	0.04	0.03	1.13 [1.09-1.17]*	1.10 [1.06-1.14]*
H53	75,260	113,343,430	0.76	0.64	0.62	0.12	0.15	0.10	1.24 [1.21-1.27]*	1.16 [1.13-1.18]*
H54	53,750	113,902,700	0.52	0.46	0.44	0.06	0.09	0.07	1.20 [1.16-1.24]*	1.16 [1.12-1.20]*
H55	5,860	114,198,220	0.06	0.05	0.05	0.01	0.01	0.00	1.12 [1.03-1.23]	1.07 [0.98-1.17]
H57	12,420	114,120,420	0.13	0.10	0.10	0.03	0.03	0.02	1.27 [1.20-1.35]*	1.19 [1.12-1.27]*
H58	2,390	114,248,580	0.02	0.02	0.02	0.00	0.00	0.00	1.23 [1.07-1.42]*	1.18 [1.02-1.36]

H59	4,180	114,207,230	0.04	0.04	0.03	0.00	0.01	0.00	1.17 [1.05-1.30]*	1.14 [1.03-1.27]
any age - H60-H95										
H60	12,310	114,030,500	0.15	0.10	0.10	0.06	0.06	0.05	1.60 [1.51-1.69]*	1.50 [1.42-1.59]*
H61	24,120	113,844,430	0.27	0.20	0.20	0.08	0.07	0.06	1.35 [1.29-1.40]*	1.30 [1.24-1.35]*
H65	68,730	112,511,020	0.77	0.57	0.64	0.20	0.13	0.11	1.21 [1.18-1.24]*	1.17 [1.14-1.20]*
H66	33,480	113,341,490	0.38	0.28	0.30	0.10	0.08	0.07	1.26 [1.22-1.31]*	1.21 [1.17-1.26]*
H69	2,430	114,223,780	0.03	0.02	0.02	0.01	0.01	0.01	1.46 [1.28-1.66]*	1.36 [1.19-1.56]*
H70	3,140	114,215,150	0.03	0.03	0.03	0.01	0.00	0.00	1.18 [1.05-1.33]*	1.13 [1.00-1.28]
H71	7,220	114,112,790	0.07	0.06	0.06	0.01	0.01	0.01	1.10 [1.02-1.19]	1.08 [0.99-1.17]
H72	14,500	113,917,460	0.15	0.12	0.12	0.03	0.03	0.03	1.24 [1.18-1.31]*	1.19 [1.13-1.26]*
H73	10,030	114,058,810	0.11	0.08	0.09	0.03	0.02	0.02	1.22 [1.14-1.30]*	1.17 [1.10-1.25]*
H74	7,430	114,104,090	0.08	0.06	0.06	0.01	0.01	0.01	1.19 [1.10-1.29]*	1.15 [1.06-1.24]*
H80	1,500	114,234,030	0.01	0.01	0.01	0.00	0.00	0.00	1.09 [0.91-1.30]	1.07 [0.90-1.29]
H81	22,090	114,069,080	0.22	0.19	0.18	0.03	0.04	0.03	1.26 [1.20-1.32]*	1.17 [1.12-1.23]*
H83	9,460	114,127,220	0.09	0.08	0.07	0.01	0.02	0.01	1.26 [1.18-1.35]*	1.17 [1.09-1.25]*
H90	29,430	113,934,360	0.32	0.24	0.25	0.07	0.07	0.06	1.29 [1.24-1.34]*	1.22 [1.17-1.27]*
H91	140,390	113,276,850	1.41	1.20	1.15	0.21	0.26	0.21	1.23 [1.20-1.25]*	1.17 [1.15-1.20]*
H92	11,510	114,042,320	0.13	0.09	0.10	0.04	0.03	0.03	1.34 [1.26-1.42]*	1.25 [1.18-1.33]*
H93	13,030	114,128,310	0.14	0.11	0.10	0.03	0.04	0.03	1.35 [1.27-1.43]*	1.26 [1.19-1.34]*
H95	1,510	114,235,370	0.02	0.01	0.01	0.00	0.00	0.00	1.27 [1.07-1.50]*	1.24 [1.05-1.48]
any age - I00-I99										
I05	7,130	114,185,600	0.07	0.06	0.06	0.01	0.02	0.01	1.30 [1.20-1.40]*	1.22 [1.12-1.32]*
I06	690	114,257,890	0.01	0.01	0.01	0.00	0.00	0.00	1.34 [1.05-1.73]	1.26 [0.98-1.63]
I07	18,210	114,186,150	0.19	0.15	0.14	0.03	0.04	0.04	1.31 [1.24-1.38]*	1.25 [1.19-1.32]*
I08	93,060	113,808,890	0.91	0.80	0.75	0.11	0.16	0.13	1.21 [1.19-1.24]*	1.17 [1.14-1.20]*
I09	1,060	114,258,150	0.01	0.01	0.01	0.00	0.00	0.00	1.42 [1.17-1.74]*	1.36 [1.11-1.67]*
I10	923,670	100,710,140	10.51	8.85	8.46	1.66	2.05	1.67	1.24 [1.23-1.25]*	1.19 [1.18-1.20]*
I11	5,330	114,209,680	0.05	0.05	0.04	0.00	0.01	0.01	1.21 [1.10-1.33]*	1.15 [1.04-1.27]*
I12	52,680	113,792,280	0.52	0.45	0.40	0.07	0.12	0.10	1.29 [1.26-1.33]*	1.25 [1.21-1.29]*
I13	2,620	114,262,390	0.03	0.02	0.02	0.00	0.01	0.00	1.30 [1.13-1.49]*	1.22 [1.06-1.40]*
I15	3,980	114,220,120	0.04	0.03	0.03	0.01	0.01	0.01	1.28 [1.15-1.42]*	1.22 [1.09-1.36]*
I20	203,010	110,384,760	2.10	1.78	1.64	0.32	0.45	0.34	1.28 [1.26-1.29]*	1.20 [1.18-1.21]*
I21	138,620	112,614,840	1.29	1.22	1.12	0.08	0.17	0.15	1.16 [1.13-1.18]*	1.13 [1.11-1.15]*
I22	12,550	114,094,910	0.12	0.11	0.10	0.02	0.03	0.02	1.28 [1.20-1.36]*	1.22 [1.15-1.30]*
I23	790	114,259,200	0.01	0.01	0.01	0.00	0.00	0.00	1.24 [0.98-1.57]	1.22 [0.95-1.55]
I24	39,460	113,982,260	0.39	0.34	0.31	0.05	0.07	0.06	1.24 [1.20-1.28]*	1.18 [1.13-1.22]*
I25	348,810	109,192,490	3.58	3.10	2.93	0.47	0.64	0.50	1.22 [1.21-1.23]*	1.16 [1.15-1.18]*
I26	71,350	113,645,530	0.65	0.62	0.57	0.02	0.07	0.05	1.13 [1.10-1.16]*	1.08 [1.05-1.11]*
I27	43,020	114,049,030	0.44	0.36	0.33	0.08	0.11	0.09	1.32 [1.28-1.37]*	1.25 [1.21-1.29]*
I28	1,310	114,257,620	0.01	0.01	0.01	0.00	0.00	0.00	1.16 [0.96-1.41]	1.07 [0.88-1.30]
I30	2,240	114,228,230	0.02	0.02	0.02	0.00	0.00	0.00	1.24 [1.07-1.42]*	1.19 [1.03-1.37]
I31	27,080	114,041,680	0.26	0.23	0.21	0.03	0.05	0.04	1.22 [1.17-1.28]*	1.17 [1.12-1.22]*
I33	4,440	114,230,360	0.05	0.04	0.03	0.01	0.01	0.01	1.35 [1.23-1.50]*	1.30 [1.17-1.44]*
I34	60,900	113,768,400	0.58	0.52	0.49	0.05	0.09	0.07	1.18 [1.14-1.21]*	1.13 [1.10-1.16]*
I35	88,920	113,628,130	0.87	0.76	0.71	0.11	0.16	0.13	1.23 [1.20-1.26]*	1.17 [1.15-1.20]*
I36	6,560	114,238,880	0.06	0.06	0.05	0.01	0.01	0.01	1.23 [1.12-1.34]*	1.16 [1.06-1.26]*
I37	7,200	114,227,670	0.07	0.06	0.06	0.01	0.01	0.01	1.22 [1.12-1.32]*	1.15 [1.06-1.25]*
I38	9,950	114,156,420	0.10	0.08	0.08	0.02	0.02	0.02	1.29 [1.21-1.38]*	1.23 [1.15-1.32]*
I40	750	114,261,620	0.01	0.01	0.01	0.00	0.00	0.00	1.29 [1.01-1.64]	1.23 [0.96-1.59]
I42	22,830	114,017,610	0.22	0.20	0.18	0.02	0.04	0.03	1.21 [1.16-1.27]*	1.15 [1.10-1.20]*
I43	960	114,266,280	0.01	0.01	0.01	0.00	0.00	0.00	0.98 [0.78-1.25]	0.94 [0.74-1.20]
I44	139,340	113,350,830	1.36	1.20	1.13	0.16	0.23	0.18	1.20 [1.18-1.22]*	1.16 [1.13-1.18]*
I45	87,760	113,679,790	0.85	0.75	0.71	0.10	0.14	0.11	1.20 [1.18-1.23]*	1.15 [1.12-1.18]*
I46	45,670	114,131,180	0.44	0.39	0.36	0.05	0.08	0.07	1.23 [1.19-1.27]*	1.19 [1.15-1.23]*
I47	51,050	113,582,230	0.50	0.44	0.41	0.06	0.09	0.07	1.22 [1.19-1.26]*	1.15 [1.12-1.19]*
I48	387,240	111,079,210	3.79	3.41	3.26	0.37	0.53	0.42	1.16 [1.15-1.18]*	1.13 [1.11-1.14]*
I49	60,360	113,711,580	0.58	0.52	0.49	0.06	0.09	0.06	1.18 [1.15-1.22]*	1.12 [1.09-1.15]*
I50	294,080	112,545,450	2.99	2.52	2.37	0.47	0.62	0.51	1.26 [1.25-1.28]*	1.20 [1.19-1.22]*
I51	164,050	113,262,330	1.63	1.41	1.31	0.22	0.32	0.25	1.25 [1.23-1.27]*	1.18 [1.16-1.21]*
I60	9,660	114,144,600	0.09	0.08	0.08	0.00	0.01	0.01	1.16 [1.08-1.24]*	1.15 [1.07-1.24]*
I61	24,080	114,113,730	0.22	0.21	0.19	0.02	0.03	0.03	1.17 [1.12-1.23]*	1.16 [1.11-1.22]*
I62	13,830	114,182,210	0.13	0.12	0.11	0.01	0.02	0.02	1.16 [1.09-1.23]*	1.14 [1.07-1.21]*
I63	113,000	113,453,940	1.01	0.99	0.93	0.02	0.08	0.07	1.09 [1.07-1.11]*	1.08 [1.06-1.10]*
I64	29,380	113,926,560	0.27	0.26	0.23	0.01	0.03	0.03	1.15 [1.10-1.19]*	1.13 [1.08-1.18]*
I65	27,300	114,004,160	0.26	0.24	0.22	0.02	0.04	0.03	1.17 [1.13-1.22]*	1.14 [1.10-1.19]*
I66	1,780	114,255,750	0.02	0.02	0.01	0.00	0.00	0.00	1.15 [0.97-1.36]	1.11 [0.94-1.32]
I67	170,160	113,461,500	1.63	1.47	1.38	0.17	0.25	0.21	1.18 [1.16-1.20]*	1.15 [1.13-1.17]*
I68	1,000	114,267,090	0.01	0.01	0.01	0.00	0.00	0.00	1.22 [0.99-1.52]	1.20 [0.96-1.49]
I69	50,450	113,820,340	0.48	0.44	0.40	0.04	0.07	0.06	1.18 [1.15-1.22]*	1.15 [1.12-1.19]*
I70	42,380	113,957,290	0.42	0.36	0.33	0.06	0.09	0.07	1.26 [1.21-1.30]*	1.21 [1.17-1.26]*
I71	51,970	113,903,530	0.48	0.45	0.42	0.04	0.07	0.06	1.17 [1.13-1.20]*	1.15 [1.12-1.19]*
I72	12,450	114,157,250	0.12	0.11	0.10	0.01	0.02	0.01	1.17 [1.10-1.25]*	1.14 [1.07-1.21]*
I73	93,220	113,349,500	1.00	0.78	0.72	0.22	0.28	0.24	1.40 [1.37-1.43]*	1.32 [1.29-1.35]*
I74	24,020	114,036,070	0.22	0.21	0.19	0.02	0.03	0.03	1.18 [1.13-1.23]*	1.14 [1.09-1.19]*
I77	31,750	113,922,830	0.32	0.27	0.25	0.06	0.08	0.07	1.32 [1.27-1.37]*	1.26 [1.21-1.31]*
I78	8,230	114,163,730	0.08	0.07	0.06	0.01	0.02	0.02	1.30 [1.21-1.40]*	1.24 [1.15-1.34]*
I79	3,130	114,240,520	0.03	0.03	0.02	0.01	0.01	0.01	1.34 [1.19-1.51]*	1.24 [1.10-1.40]*
I80	64,000	113,400,140	0.63	0.55	0.50	0.08	0.13	0.10	1.25 [1.22-1.29]*	1.20 [1.17-1.23]*
I81	3,600	114,253,110	0.03	0.03	0.03	0.00	0.00	0.00	1.14 [1.01-1.28]	1.08 [0.96-1.22]
I82	8,300	114,204,010	0.08	0.07	0.07	0.01	0.01	0.01	1.21 [1.12-1.30]*	1.15 [1.07-1.25]*
I83	73,770	112,508,400	0.89	0.60	0.55	0.29	0.34	0.32	1.63 [1.59-1.67]*	1.57 [1.54-1.61]*
I84	115,820	111,349,500	1.22	1.00	0.89	0.22	0.33	0.26	1.37 [1.34-1.39]*	1.28 [1.25-1.30]*
I85	9,960	114,176,370	0.11	0.08	0.07	0.03	0.03	0.03	1.46 [1.37-1.55]*	1.37 [1.29-1.46]*
I86	8,510	114,148,180	0.09	0.07	0.07	0.02	0.02	0.02	1.31 [1.22-1.41]*	1.22 [1.14-1.32]*
I87	16,270	114,150,460	0.19	0.13	0.12	0.06	0.07	0.07	1.60 [1.52-1.69]*	1.52 [1.44-1.60]*
I88	14,270	114,038,230	0.17	0.11	0.13	0.06	0.04	0.03	1.28 [1.22-1.36]*	1.25 [1.18-1.32]*
I89	21,910	114,120,760	0.25	0.18	0.16	0.07	0.09	0.08	1.53 [1.46-1.59]*	1.43 [1.37-1.50]*
I95	207,390	113,135,460	2.03	1.79	1.69	0.24	0.34	0.27	1.20 [1.18-1.22]*	1.16 [1.14-1.17]*
I97	2,640	114,215,960	0.03	0.02	0.02	0.00	0.01	0.01	1.33 [1.17-1.51]*	1.26 [1.10-1.43]*
I98	6,720	114,222,560	0.07	0.06	0.05	0.02	0.02	0.02	1.45 [1.34-1.56]*	1.34 [1.24-1.45]*
I99	3,840	114,232,630	0.04	0.03	0.03	0.00	0.01	0.01	1.25 [1.11-1.39]*	1.20 [1.07-1.34]*
any age - J00-J99										
J00	13,540	113,939,120	0.16	0.11	0.11	0.05	0.05	0.04	1.41 [1.33-1.48]*	1.33 [1.26-1.40]*
J01	3,390	114,210,530	0.04	0.03	0.03	0.01	0.01	0.01	1.46 [1.31-1.63]*	1.31 [1.17-1.47]*
J02	25,070	113,831,290	0.28	0.21	0.21	0.08	0.07	0.06	1.32 [1.27-1.38]*	1.24 [1.19-1.29]*
J03	130,520	111,894,800	1.49	1.09	1.22	0.40	0.27	0.22	1.22 [1.20-1.24]*	1.17 [1.15-1.19]*
J04	3,040	114,214,990	0.03	0.03	0.02	0.01	0.01	0.01	1.34 [1.19-1.51]*	1.23 [1.09-1.39]*
J05	22,750	113,550,870	0.22	0.20	0.22	0.03	0.00	0.00	1.02 [0.97-1.06]	0.99 [0.95-1.04]
J06	86,740	110,834,210	1.03	0.72	0.80	0.31	0.24	0.20	1.30 [1.27-1.33]*	1.24 [1.21-1.27]*
J10	20,020	114,149,530	0.22	0.16	0.16	0.05	0.06	0.04	1.35 [1.28-1.41]*	1.22 [1.16-1.28]*
J11	4,260	114,202,770	0.05	0.04	0.03	0.01	0.01	0.01	1.35 [1.22-1.49]*	1.24 [1.12-1.38]*
J12	31,080	114,194,250	0.32	0.26	0.25	0.06	0.07	0.05	1.28 [1.23-1.33]*	1.19 [1.14-1.24]*
J13	6,920	114,195,510	0.07	0.06	0.05	0.01	0.02	0.01	1.32 [1.22-1.43]*	1.21 [1.12-1.32]*
J14	2,900	114,244,570	0.03	0.02	0.02	0.01	0.01	0.00	1.33 [1.17-1.50]*	1.17 [1.03-1.33]
J15	15,170	114,125,420	0.16	0.13	0.12	0.04	0.04	0.03	1.38 [1.31-1.45]*	1.26 [1.19-1.33]*
J17	2,990	114,245,460	0.03	0.02	0.02	0.01	0.01	0.01	1.49 [1.32-1.67]*	1.38 [1.23-1.56]*

J18	438,130	111,330,990	4.54	3.79	3.57	0.75	0.97	0.78	1.27 [1.26-1.28]*	1.21 [1.20-1.22]*
J20	5,960	114,163,670	0.06	0.05	0.05	0.01	0.01	0.01	1.29 [1.18-1.40]*	1.18 [1.08-1.29]*
J21	5,040	112,463,840	0.05	0.04	0.05	0.01	0.01	0.00	1.14 [1.05-1.25]*	1.07 [0.97-1.17]
J22	285,020	110,816,300	3.19	2.43	2.34	0.76	0.85	0.68	1.36 [1.35-1.38]*	1.27 [1.26-1.29]*
J30	51,600	113,739,920	0.81	0.37	0.38	0.45	0.43	0.34	2.14 [2.08-2.20]*	1.73 [1.68-1.78]*
J31	11,650	114,053,390	0.15	0.09	0.09	0.06	0.07	0.05	1.75 [1.66-1.86]*	1.44 [1.35-1.53]*
J32	27,210	113,745,000	0.30	0.22	0.21	0.08	0.09	0.06	1.42 [1.36-1.47]*	1.22 [1.18-1.28]*
J33	17,910	113,746,650	0.20	0.15	0.13	0.06	0.07	0.05	1.56 [1.49-1.64]*	1.29 [1.23-1.36]*
J34	63,770	112,468,740	0.76	0.52	0.50	0.24	0.25	0.20	1.51 [1.47-1.54]*	1.35 [1.31-1.38]*
J35	101,640	111,518,930	1.21	0.84	0.94	0.36	0.27	0.23	1.28 [1.26-1.31]*	1.23 [1.21-1.26]*
J36	11,690	114,030,650	0.11	0.10	0.10	0.01	0.01	0.01	1.14 [1.07-1.22]*	1.11 [1.04-1.18]*
J37	610	114,251,570	0.01	0.01	0.00	0.00	0.00	0.00	1.39 [1.07-1.81]	1.24 [0.95-1.63]
J38	20,980	113,877,360	0.21	0.18	0.16	0.04	0.05	0.04	1.30 [1.24-1.36]*	1.21 [1.15-1.27]*
J39	11,180	114,102,740	0.12	0.09	0.09	0.03	0.03	0.02	1.33 [1.25-1.42]*	1.23 [1.15-1.31]*
J40	11,910	114,110,590	0.13	0.10	0.09	0.03	0.04	0.03	1.44 [1.36-1.53]*	1.29 [1.21-1.37]*
J41	580	114,263,520	0.01	0.00	0.00	0.00	0.00	0.00	1.44 [1.09-1.89]*	1.32 [0.99-1.74]
J42	4,820	114,204,800	0.05	0.04	0.04	0.01	0.02	0.01	1.45 [1.32-1.60]*	1.28 [1.16-1.41]*
J43	65,960	113,834,960	0.69	0.55	0.51	0.13	0.18	0.14	1.35 [1.32-1.39]*	1.25 [1.22-1.29]*
J44	262,890	111,926,660	2.94	2.21	2.02	0.73	0.92	0.66	1.46 [1.44-1.48]*	1.29 [1.27-1.31]*
J45	503,210	103,273,790	8.34	4.05	3.93	4.29	4.41	3.16	2.12 [2.11-2.14]*	1.61 [1.59-1.62]*
J46	9,490	113,926,720	0.18	0.06	0.06	0.12	0.12	0.10	2.95 [2.79-3.13]*	2.29 [2.15-2.44]*
J47	48,030	113,922,430	0.54	0.39	0.36	0.15	0.18	0.12	1.49 [1.45-1.54]*	1.29 [1.25-1.33]*
J61	6,510	114,232,750	0.07	0.05	0.05	0.01	0.02	0.02	1.39 [1.28-1.52]*	1.32 [1.21-1.43]*
J67	1,930	114,256,010	0.02	0.02	0.01	0.01	0.01	0.01	1.61 [1.39-1.86]*	1.44 [1.24-1.67]*
J69	64,700	114,106,290	0.62	0.55	0.53	0.06	0.09	0.08	1.17 [1.14-1.20]*	1.15 [1.12-1.18]*
J70	1,310	114,263,830	0.01	0.01	0.01	0.00	0.01	0.00	1.52 [1.27-1.82]*	1.42 [1.18-1.70]*
J80	5,040	114,235,320	0.05	0.04	0.04	0.01	0.01	0.01	1.37 [1.25-1.51]*	1.31 [1.19-1.44]*
J81	22,390	114,120,810	0.22	0.19	0.17	0.03	0.05	0.05	1.30 [1.24-1.36]*	1.25 [1.20-1.31]*
J82	1,750	114,259,390	0.03	0.01	0.01	0.01	0.02	0.01	2.40 [2.08-2.78]*	1.72 [1.47-2.02]*
J84	41,200	114,083,230	0.45	0.34	0.31	0.11	0.14	0.12	1.44 [1.39-1.49]*	1.36 [1.31-1.41]*
J85	2,310	114,251,300	0.02	0.02	0.02	0.00	0.01	0.00	1.31 [1.14-1.51]*	1.21 [1.05-1.39]*
J86	8,030	114,183,450	0.08	0.07	0.06	0.01	0.01	0.01	1.20 [1.12-1.30]*	1.17 [1.08-1.26]*
J90	210,860	113,227,020	2.03	1.82	1.71	0.21	0.33	0.28	1.19 [1.17-1.21]*	1.16 [1.14-1.18]*
J91	5,070	114,250,760	0.05	0.04	0.04	0.01	0.01	0.01	1.24 [1.12-1.37]*	1.21 [1.09-1.33]*
J92	23,580	114,154,380	0.23	0.20	0.18	0.03	0.05	0.04	1.26 [1.20-1.31]*	1.22 [1.16-1.27]*
J93	20,490	114,050,810	0.19	0.18	0.17	0.01	0.02	0.02	1.15 [1.09-1.20]*	1.12 [1.06-1.17]*
J94	7,920	114,221,710	0.07	0.07	0.06	0.01	0.01	0.01	1.18 [1.09-1.28]*	1.15 [1.07-1.25]*
J95	6,590	114,177,850	0.06	0.06	0.05	0.01	0.01	0.01	1.21 [1.11-1.31]*	1.17 [1.08-1.27]*
J96	134,510	113,785,820	1.38	1.14	1.05	0.24	0.33	0.26	1.32 [1.30-1.34]*	1.23 [1.21-1.25]*
J98	129,100	113,382,360	1.29	1.10	1.03	0.19	0.26	0.19	1.25 [1.23-1.28]*	1.17 [1.15-1.20]*
J99	3,890	114,238,600	0.05	0.03	0.03	0.02	0.02	0.01	1.60 [1.45-1.77]*	1.39 [1.25-1.54]*
any age - K00-K95										
K00	26,680	113,812,440	0.30	0.22	0.26	0.08	0.04	0.03	1.16 [1.12-1.21]*	1.12 [1.08-1.17]*
K01	64,810	112,598,380	0.69	0.55	0.57	0.14	0.12	0.09	1.21 [1.18-1.25]*	1.16 [1.13-1.19]*
K02	191,290	110,815,100	2.00	1.66	1.80	0.34	0.19	0.13	1.11 [1.09-1.13]*	1.07 [1.05-1.09]*
K03	4,670	114,208,490	0.05	0.04	0.04	0.01	0.01	0.01	1.17 [1.07-1.29]*	1.12 [1.01-1.23]
K04	41,400	113,490,440	0.41	0.35	0.36	0.06	0.05	0.03	1.14 [1.10-1.18]*	1.09 [1.05-1.13]*
K05	36,920	113,595,030	0.39	0.31	0.30	0.08	0.09	0.07	1.29 [1.25-1.34]*	1.22 [1.17-1.26]*
K06	4,860	114,196,150	0.05	0.04	0.04	0.01	0.01	0.01	1.38 [1.26-1.52]*	1.31 [1.19-1.44]*
K07	38,970	113,505,900	0.42	0.32	0.36	0.10	0.07	0.05	1.19 [1.15-1.23]*	1.14 [1.10-1.18]*
K08	57,980	113,263,340	0.59	0.49	0.49	0.09	0.09	0.07	1.19 [1.15-1.22]*	1.13 [1.10-1.16]*
K09	4,320	114,162,120	0.04	0.04	0.04	0.01	0.01	0.01	1.26 [1.14-1.40]*	1.24 [1.12-1.38]*
K10	5,530	114,199,020	0.06	0.05	0.05	0.01	0.01	0.01	1.25 [1.14-1.36]*	1.15 [1.05-1.26]*
K11	13,230	114,035,250	0.14	0.11	0.11	0.03	0.03	0.03	1.31 [1.24-1.39]*	1.24 [1.17-1.32]*
K12	20,820	114,033,530	0.22	0.17	0.17	0.05	0.05	0.04	1.29 [1.24-1.35]*	1.24 [1.19-1.30]*
K13	27,380	113,790,920	0.30	0.23	0.22	0.07	0.08	0.07	1.37 [1.31-1.42]*	1.30 [1.25-1.35]*
K14	13,280	114,066,920	0.14	0.11	0.10	0.03	0.04	0.03	1.39 [1.31-1.47]*	1.28 [1.21-1.36]*
K20	81,790	112,805,980	0.85	0.70	0.64	0.15	0.21	0.15	1.33 [1.30-1.36]*	1.22 [1.19-1.25]*
K21	315,740	109,902,060	3.44	2.74	2.54	0.70	0.90	0.60	1.36 [1.34-1.37]*	1.21 [1.20-1.23]*
K22	120,180	112,636,160	1.22	1.03	0.94	0.19	0.28	0.21	1.29 [1.27-1.32]*	1.21 [1.18-1.23]*
K23	1,390	114,260,330	0.02	0.01	0.01	0.00	0.01	0.00	1.54 [1.30-1.84]*	1.38 [1.16-1.65]*
K25	59,100	113,430,030	0.60	0.50	0.46	0.09	0.14	0.11	1.29 [1.26-1.33]*	1.22 [1.18-1.25]*
K26	42,600	113,641,690	0.40	0.37	0.34	0.04	0.07	0.06	1.19 [1.15-1.23]*	1.16 [1.12-1.20]*
K27	7,230	114,168,840	0.07	0.06	0.06	0.01	0.02	0.01	1.29 [1.20-1.40]*	1.20 [1.10-1.30]*
K28	1,040	114,258,320	0.01	0.01	0.01	0.00	0.00	0.00	1.47 [1.20-1.79]*	1.24 [1.00-1.52]
K29	306,110	109,363,150	3.30	2.68	2.50	0.63	0.80	0.61	1.32 [1.31-1.34]*	1.22 [1.21-1.24]*
K30	73,680	112,429,780	0.78	0.63	0.57	0.15	0.21	0.15	1.36 [1.33-1.39]*	1.24 [1.21-1.27]*
K31	102,340	113,237,500	1.04	0.87	0.80	0.17	0.24	0.16	1.30 [1.27-1.33]*	1.18 [1.16-1.21]*
K35	64,400	112,976,120	0.62	0.56	0.57	0.06	0.04	0.03	1.08 [1.05-1.11]*	1.04 [1.01-1.07]*
K36	1,370	114,244,690	0.01	0.01	0.01	0.00	0.00	0.00	1.17 [0.98-1.40]	1.15 [0.96-1.38]
K37	12,120	114,036,730	0.12	0.10	0.11	0.02	0.01	0.01	1.14 [1.07-1.21]*	1.07 [1.01-1.14]
K38	8,010	114,148,810	0.08	0.07	0.07	0.01	0.01	0.01	1.15 [1.07-1.24]*	1.10 [1.01-1.18]
K40	121,060	111,486,670	1.11	1.08	1.01	0.03	0.10	0.06	1.10 [1.07-1.12]*	1.06 [1.04-1.08]*
K41	8,350	114,134,000	0.08	0.07	0.07	0.01	0.01	0.01	1.19 [1.10-1.28]*	1.13 [1.05-1.22]*
K42	54,130	113,303,900	0.55	0.46	0.43	0.09	0.12	0.08	1.29 [1.25-1.32]*	1.17 [1.14-1.21]*
K43	41,540	113,607,970	0.41	0.35	0.33	0.06	0.08	0.06	1.26 [1.22-1.30]*	1.16 [1.12-1.20]*
K44	295,680	109,862,970	3.12	2.59	2.39	0.53	0.73	0.51	1.31 [1.29-1.32]*	1.20 [1.18-1.21]*
K45	1,930	114,247,050	0.02	0.02	0.02	0.00	0.00	0.00	1.22 [1.04-1.42]	1.12 [0.95-1.31]
K46	7,960	114,208,020	0.08	0.07	0.06	0.01	0.02	0.01	1.31 [1.21-1.41]*	1.18 [1.09-1.28]*
K50	22,990	113,770,810	0.29	0.18	0.17	0.11	0.12	0.09	1.70 [1.63-1.77]*	1.47 [1.41-1.54]*
K51	32,390	113,601,250	0.37	0.27	0.24	0.10	0.12	0.10	1.51 [1.46-1.56]*	1.35 [1.30-1.40]*
K52	179,030	109,897,870	1.95	1.55	1.44	0.39	0.51	0.41	1.35 [1.33-1.38]*	1.27 [1.25-1.29]*
K55	25,930	114,088,570	0.25	0.22	0.20	0.03	0.05	0.04	1.25 [1.20-1.30]*	1.19 [1.14-1.24]*
K56	80,780	113,496,030	0.78	0.69	0.65	0.09	0.13	0.10	1.21 [1.18-1.24]*	1.15 [1.12-1.17]*
K57	337,850	110,472,750	3.42	2.97	2.73	0.45	0.69	0.51	1.25 [1.24-1.27]*	1.17 [1.16-1.19]*
K58	96,360	113,076,990	1.12	0.79	0.74	0.33	0.37	0.24	1.50 [1.47-1.53]*	1.27 [1.24-1.30]*
K59	332,310	111,007,730	3.53	2.87	2.77	0.66	0.76	0.58	1.27 [1.26-1.29]*	1.20 [1.18-1.21]*
K60	29,190	113,534,470	0.34	0.24	0.22	0.10	0.11	0.10	1.52 [1.47-1.58]*	1.41 [1.36-1.46]*
K61	18,450	113,826,890	0.20	0.15	0.14	0.04	0.06	0.05	1.39 [1.33-1.46]*	1.32 [1.26-1.39]*
K62	177,520	110,848,840	1.87	1.54	1.40	0.33	0.47	0.37	1.33 [1.31-1.35]*	1.25 [1.23-1.27]*
K63	178,680	112,477,140	1.79	1.54	1.40	0.25	0.39	0.28	1.27 [1.25-1.30]*	1.19 [1.17-1.21]*
K64	126,200	113,775,740	1.30	1.06	0.99	0.24	0.31	0.23	1.31 [1.29-1.34]*	1.21 [1.19-1.23]*
K65	19,400	114,105,660	0.18	0.17	0.15	0.02	0.03	0.02	1.19 [1.13-1.25]*	1.16 [1.10-1.21]*
K66	68,500	113,521,050	0.66	0.59	0.55	0.07	0.11	0.07	1.19 [1.16-1.22]*	1.12 [1.09-1.15]*
K70	22,450	114,021,850	0.24	0.19	0.17	0.05	0.07	0.06	1.44 [1.38-1.51]*	1.37 [1.31-1.43]*
K71	1,450	114,253,650	0.02	0.01	0.01	0.00	0.00	0.00	1.37 [1.15-1.62]*	1.29 [1.08-1.54]*
K72	14,090	114,212,050	0.15	0.12	0.11	0.03	0.04	0.03	1.36 [1.28-1.43]*	1.28 [1.21-1.36]*
K73	1,380	114,241,240	0.02	0.01	0.01	0.00	0.00	0.00	1.48 [1.25-1.75]*	1.39 [1.16-1.65]*
K74	20,900	114,113,730	0.23	0.17	0.16	0.06	0.07	0.06	1.46 [1.39-1.52]*	1.35 [1.29-1.42]*
K75	13,910	114,143,540	0.15	0.11	0.11	0.04	0.04	0.04	1.40 [1.33-1.48]*	1.30 [1.23-1.38]*
K76	100,850	113,575,820	1.05	0.85	0.79	0.20	0.26	0.19	1.33 [1.31-1.36]*	1.23 [1.20-1.25]*
K80	191,490	111,433,250	1.83	1.69	1.57	0.14	0.26	0.17	1.16 [1.14-1.18]*	1.10 [1.09-1.12]*
K81	35,060	113,724,020	0.33	0.30	0.28	0.03	0.05	0.04	1.19 [1.15-1.23]*	1.12 [1.08-1.17]*
K82	26,820	113,922,940	0.25	0.23	0.21	0.02	0.04	0.03	1.18 [1.13-1.23]*	1.11 [1.07-1.16]*
K83	50,290	113,808,320	0.47	0.44	0.41	0.03	0.06	0.05	1.16 [1.12-1.19]*	1.11 [1.08-1.15]*
K85	33,650	113,797,680	0.32	0.29	0.27	0.03	0.05	0.04	1.20 [1.16-1.25]*	1.14 [1.10-1.18]*
K86	28,040	114,026,110	0.27	0.24	0.22	0.03	0.05	0.03	1.21 [1.17-1.27]*	1.15 [1.10-1.19]*
K90	28,570	113,768,930	0.39	0.22	0.22	0.17	0.17	0.14	1.78 [1.72-1.85]*	1.56 [1.50-1.62]*

K91	25,800	113,951,270	0.26	0.22	0.20	0.04	0.06	0.04	1.29 [1.24-1.35]*	1.20 [1.15-1.25]*
K92	224,610	112,058,810	2.31	1.93	1.80	0.38	0.51	0.40	1.28 [1.27-1.30]*	1.21 [1.19-1.23]*
any age - L00-L99										
L00	720	114,229,250	0.01	0.01	0.01	0.00	0.00	0.00	1.54 [1.22-1.93]*	1.54 [1.22-1.93]*
L01	3,670	114,151,010	0.06	0.03	0.03	0.04	0.03	0.03	2.28 [2.08-2.51]*	2.10 [1.91-2.32]*
L02	50,310	113,294,260	0.55	0.42	0.40	0.13	0.15	0.13	1.38 [1.34-1.42]*	1.30 [1.26-1.34]*
L03	175,050	112,175,720	2.12	1.43	1.32	0.70	0.80	0.73	1.61 [1.58-1.63]*	1.53 [1.50-1.55]*
L04	5,080	114,164,240	0.05	0.04	0.05	0.01	0.01	0.01	1.21 [1.11-1.33]*	1.19 [1.08-1.30]*
L05	17,880	113,734,920	0.17	0.15	0.15	0.02	0.02	0.02	1.15 [1.09-1.21]*	1.11 [1.05-1.17]*
L08	47,270	113,790,560	0.55	0.38	0.36	0.17	0.19	0.18	1.54 [1.50-1.59]*	1.47 [1.43-1.52]*
L10	560	114,264,510	0.01	0.00	0.00	0.00	0.00	0.00	2.28 [1.75-2.95]*	2.11 [1.62-2.76]*
L11	460	114,264,280	0.01	0.00	0.00	0.00	0.00	0.00	1.67 [1.24-2.25]*	1.57 [1.16-2.13]*
L12	3,810	114,246,770	0.06	0.03	0.02	0.04	0.04	0.04	2.86 [2.59-3.16]*	2.75 [2.48-3.04]*
L13	680	114,260,200	0.01	0.00	0.00	0.01	0.01	0.01	3.14 [2.51-3.92]*	2.84 [2.26-3.58]*
L20	7,190	113,992,090	0.28	0.01	0.01	0.27	0.27	0.27	24.72 [22.44-27.23]*	21.74 [19.72-23.97]*
L21	1,860	114,212,560	0.04	0.01	0.01	0.02	0.02	0.02	3.29 [2.87-3.77]*	3.09 [2.69-3.56]*
L22	2,060	114,160,750	0.02	0.02	0.02	0.00	0.00	0.00	1.05 [0.90-1.22]	1.01 [0.87-1.18]
L23	2,260	114,222,200	0.05	0.01	0.01	0.04	0.04	0.03	3.75 [3.33-4.22]*	3.33 [2.94-3.76]*
L24	10,730	114,250,180	0.12	0.09	0.08	0.03	0.04	0.03	1.45 [1.35-1.55]*	1.39 [1.30-1.49]*
L25	2,100	114,239,310	0.04	0.01	0.01	0.03	0.03	0.03	2.99 [2.63-3.39]*	2.84 [2.49-3.23]*
L26	220	114,267,870	0.01	0.00	0.00	0.00	0.00	0.00	6.48 [4.30-9.76]*	6.06 [3.99-9.21]*
L27	10,870	114,129,140	0.14	0.08	0.08	0.06	0.06	0.05	1.75 [1.65-1.86]*	1.61 [1.51-1.71]*
L28	3,570	114,212,920	0.08	0.02	0.02	0.06	0.06	0.06	4.46 [4.06-4.91]*	4.14 [3.75-4.56]*
L29	17,360	113,985,790	0.22	0.14	0.13	0.09	0.09	0.08	1.72 [1.64-1.80]*	1.60 [1.52-1.68]*
L30	109,410	112,014,620	3.64	0.35	0.35	3.29	3.29	3.26	10.30 [10.10-10.50]*	9.48 [9.30-9.67]*
L40	42,900	113,784,710	0.64	0.31	0.29	0.33	0.35	0.34	2.23 [2.17-2.30]*	2.13 [2.07-2.20]*
L42	730	114,262,580	0.01	0.01	0.01	0.00	0.00	0.00	1.54 [1.22-1.95]*	1.44 [1.13-1.83]*
L43	4,550	114,196,140	0.07	0.03	0.03	0.03	0.04	0.03	2.18 [1.99-2.38]*	2.03 [1.86-2.23]*
L44	260	114,265,110	0.00	0.00	0.00	0.00	0.00	0.00	3.12 [2.21-4.41]*	2.92 [2.05-4.16]*
L50	12,880	114,038,850	0.19	0.09	0.10	0.10	0.09	0.07	1.93 [1.83-2.03]*	1.64 [1.55-1.73]*
L51	3,180	114,198,300	0.03	0.03	0.03	0.01	0.01	0.01	1.28 [1.14-1.43]*	1.21 [1.07-1.35]*
L52	1,260	114,245,010	0.02	0.01	0.01	0.00	0.00	0.00	1.49 [1.25-1.79]*	1.38 [1.15-1.66]*
L53	15,850	114,084,140	0.20	0.12	0.12	0.08	0.09	0.08	1.75 [1.66-1.84]*	1.66 [1.58-1.75]*
L55	540	114,260,680	0.01	0.00	0.00	0.00	0.00	0.00	1.15 [0.86-1.53]	1.10 [0.82-1.48]
L56	620	114,260,860	0.01	0.00	0.00	0.01	0.01	0.01	2.80 [2.22-3.53]*	2.65 [2.09-3.36]*
L57	34,630	113,880,160	0.36	0.29	0.27	0.06	0.09	0.07	1.32 [1.27-1.37]*	1.25 [1.21-1.30]*
L59	520	114,264,400	0.01	0.00	0.00	0.00	0.00	0.00	1.42 [1.07-1.89]	1.38 [1.02-1.85]
L60	14,740	113,848,160	0.17	0.12	0.12	0.05	0.05	0.04	1.41 [1.34-1.49]*	1.30 [1.23-1.38]*
L63	720	114,260,800	0.01	0.00	0.00	0.01	0.01	0.01	2.87 [2.32-3.56]*	2.27 [1.80-2.85]*
L65	2,500	114,241,560	0.04	0.02	0.02	0.02	0.02	0.02	1.99 [1.76-2.25]*	1.75 [1.54-1.99]*
L66	500	114,262,820	0.01	0.00	0.00	0.00	0.00	0.00	1.73 [1.31-2.27]*	1.62 [1.22-2.14]*
L68	910	114,251,310	0.01	0.01	0.01	0.00	0.00	0.00	1.25 [1.00-1.56]	1.19 [0.95-1.49]
L70	3,340	114,232,290	0.04	0.03	0.03	0.01	0.01	0.01	1.45 [1.30-1.62]*	1.32 [1.18-1.49]*
L71	3,530	114,234,320	0.04	0.03	0.03	0.01	0.01	0.01	1.55 [1.39-1.72]*	1.44 [1.29-1.61]*
L72	53,830	112,748,340	0.50	0.47	0.43	0.03	0.07	0.05	1.16 [1.13-1.19]*	1.11 [1.08-1.14]*
L73	6,820	114,132,620	0.08	0.05	0.05	0.03	0.03	0.02	1.57 [1.46-1.70]*	1.44 [1.33-1.56]*
L80	1,580	114,253,190	0.02	0.01	0.01	0.01	0.01	0.01	1.97 [1.69-2.29]*	1.80 [1.54-2.11]*
L81	8,340	114,122,460	0.09	0.07	0.07	0.02	0.02	0.02	1.29 [1.20-1.38]*	1.23 [1.14-1.32]*
L82	23,390	113,887,380	0.22	0.20	0.18	0.02	0.04	0.03	1.22 [1.17-1.28]*	1.16 [1.11-1.21]*
L83	1,600	114,246,470	0.02	0.01	0.01	0.01	0.01	0.01	1.67 [1.43-1.96]*	1.56 [1.33-1.84]*
L84	2,570	114,227,230	0.03	0.02	0.02	0.01	0.01	0.01	1.49 [1.31-1.69]*	1.39 [1.22-1.58]*
L85	9,870	114,147,190	0.12	0.08	0.07	0.04	0.04	0.04	1.62 [1.52-1.73]*	1.54 [1.44-1.65]*
L88	570	114,263,170	0.01	0.00	0.00	0.00	0.00	0.00	1.56 [1.20-2.04]*	1.37 [1.04-1.80]
L89	121,870	113,961,150	1.20	1.04	0.98	0.16	0.22	0.20	1.22 [1.20-1.25]*	1.20 [1.17-1.22]*
L90	25,210	113,726,160	0.26	0.21	0.20	0.04	0.06	0.05	1.29 [1.24-1.34]*	1.22 [1.17-1.27]*
L91	9,360	114,010,680	0.10	0.08	0.07	0.02	0.03	0.02	1.35 [1.26-1.44]*	1.27 [1.19-1.36]*
L92	7,520	114,106,240	0.08	0.06	0.06	0.02	0.02	0.01	1.30 [1.20-1.40]*	1.21 [1.12-1.31]*
L93	2,690	114,232,710	0.04	0.02	0.02	0.02	0.02	0.01	1.86 [1.66-2.10]*	1.67 [1.48-1.88]*
L94	2,020	114,241,050	0.02	0.02	0.02	0.01	0.01	0.01	1.55 [1.35-1.78]*	1.45 [1.26-1.68]*
L95	2,440	114,239,660	0.03	0.02	0.02	0.01	0.01	0.01	1.61 [1.42-1.83]*	1.50 [1.31-1.71]*
L97	86,530	113,764,860	0.99	0.71	0.64	0.28	0.35	0.33	1.54 [1.51-1.58]*	1.49 [1.46-1.53]*
L98	73,890	113,100,030	0.80	0.62	0.57	0.18	0.22	0.20	1.39 [1.36-1.42]*	1.32 [1.29-1.36]*
any age - M00-M99										
M00	9,110	114,140,710	0.10	0.07	0.07	0.03	0.03	0.03	1.48 [1.39-1.59]*	1.41 [1.32-1.51]*
M02	2,370	114,233,040	0.03	0.02	0.02	0.01	0.00	0.00	1.22 [1.07-1.40]*	1.18 [1.03-1.35]
M05	10,180	114,141,640	0.11	0.08	0.08	0.02	0.03	0.03	1.40 [1.32-1.50]*	1.35 [1.27-1.44]*
M06	74,500	113,334,180	0.80	0.62	0.57	0.18	0.23	0.19	1.40 [1.37-1.44]*	1.31 [1.28-1.34]*
M07	8,830	114,157,050	0.11	0.07	0.06	0.04	0.05	0.04	1.75 [1.64-1.87]*	1.62 [1.52-1.74]*
M08	3,230	114,204,690	0.04	0.03	0.03	0.01	0.01	0.01	1.33 [1.19-1.48]*	1.28 [1.14-1.43]*
M10	84,410	113,647,810	0.88	0.71	0.65	0.17	0.23	0.20	1.36 [1.33-1.39]*	1.29 [1.26-1.32]*
M11	9,460	114,187,290	0.10	0.08	0.08	0.02	0.02	0.02	1.29 [1.20-1.39]*	1.21 [1.12-1.30]*
M12	2,050	114,239,420	0.02	0.02	0.02	0.00	0.01	0.00	1.40 [1.21-1.61]*	1.27 [1.10-1.48]*
M13	213,590	112,162,460	2.26	1.82	1.68	0.44	0.57	0.44	1.34 [1.32-1.36]*	1.24 [1.22-1.26]*
M14	2,240	114,243,800	0.03	0.02	0.02	0.01	0.01	0.01	1.61 [1.41-1.85]*	1.46 [1.28-1.68]*
M15	78,160	113,535,800	0.83	0.66	0.60	0.17	0.23	0.17	1.38 [1.34-1.41]*	1.26 [1.23-1.29]*
M16	131,070	112,378,840	1.20	1.16	1.06	0.04	0.13	0.10	1.13 [1.10-1.15]*	1.09 [1.07-1.11]*
M17	211,420	110,949,980	2.13	1.85	1.70	0.28	0.43	0.32	1.25 [1.23-1.27]*	1.18 [1.16-1.20]*
M18	11,530	114,116,800	0.12	0.10	0.09	0.03	0.04	0.03	1.43 [1.35-1.52]*	1.27 [1.19-1.35]*
M19	330,800	111,418,910	3.55	2.83	2.62	0.72	0.93	0.71	1.36 [1.34-1.37]*	1.25 [1.24-1.27]*
M20	50,220	113,125,210	0.51	0.43	0.39	0.08	0.12	0.09	1.30 [1.26-1.34]*	1.22 [1.18-1.26]*
M21	30,160	113,891,470	0.30	0.26	0.25	0.04	0.05	0.03	1.19 [1.15-1.24]*	1.12 [1.07-1.16]*
M22	9,680	114,002,890	0.10	0.08	0.08	0.02	0.02	0.02	1.32 [1.24-1.41]*	1.20 [1.12-1.29]*
M23	98,050	111,891,390	0.95	0.86	0.78	0.09	0.17	0.12	1.22 [1.19-1.25]*	1.14 [1.12-1.17]*
M24	39,650	113,571,400	0.40	0.34	0.32	0.06	0.08	0.06	1.26 [1.22-1.30]*	1.17 [1.13-1.21]*
M25	225,360	111,218,950	2.38	1.94	1.84	0.44	0.54	0.40	1.29 [1.27-1.31]*	1.20 [1.18-1.22]*
M30	2,040	114,229,470	0.02	0.02	0.02	0.01	0.01	0.01	1.39 [1.21-1.59]*	1.28 [1.11-1.48]*
M31	11,970	114,148,760	0.12	0.10	0.09	0.02	0.03	0.03	1.34 [1.26-1.42]*	1.26 [1.18-1.34]*
M32	5,620	114,173,440	0.07	0.04	0.04	0.03	0.03	0.03	1.79 [1.65-1.95]*	1.63 [1.49-1.77]*
M33	1,280	114,251,860	0.02	0.01	0.01	0.01	0.01	0.01	2.07 [1.75-2.45]*	2.00 [1.69-2.38]*
M34	2,210	114,239,620	0.03	0.02	0.02	0.01	0.01	0.01	1.75 [1.53-2.00]*	1.62 [1.41-1.85]*
M35	57,660	113,762,580	0.64	0.47	0.45	0.17	0.20	0.16	1.44 [1.40-1.48]*	1.33 [1.29-1.36]*
M36	540	114,261,490	0.01	0.00	0.00	0.00	0.00	0.00	1.43 [1.07-1.89]	1.30 [0.97-1.74]
M40	13,790	114,197,230	0.14	0.12	0.11	0.02	0.03	0.02	1.25 [1.18-1.33]*	1.18 [1.11-1.25]*
M41	39,770	113,936,200	0.41	0.34	0.33	0.07	0.08	0.06	1.24 [1.20-1.28]*	1.16 [1.12-1.20]*
M42	810	114,261,550	0.01	0.01	0.01	0.00	0.00	0.00	1.13 [0.89-1.44]	0.98 [0.76-1.26]
M43	31,610	113,918,390	0.32	0.27	0.25	0.06	0.07	0.05	1.29 [1.24-1.34]*	1.18 [1.14-1.23]*
M45	7,300	114,164,290	0.08	0.06	0.05	0.02	0.03	0.02	1.50 [1.40-1.62]*	1.39 [1.29-1.50]*
M46	24,800	114,021,180	0.27	0.21	0.19	0.06	0.08	0.06	1.42 [1.36-1.48]*	1.28 [1.22-1.33]*
M47	140,600	112,837,020	1.48	1.19	1.09	0.29	0.39	0.28	1.35 [1.33-1.38]*	1.23 [1.21-1.26]*
M48	82,960	113,519,610	0.84	0.71	0.65	0.13	0.19	0.13	1.29 [1.26-1.32]*	1.19 [1.16-1.22]*
M49	3,000	114,256,140	0.03	0.03	0.02	0.00	0.00	0.00	1.08 [0.95-1.23]	1.06 [0.93-1.21]
M50	21,770	114,024,370	0.22	0.18	0.17	0.04	0.06	0.04	1.33 [1.27-1.40]*	1.22 [1.17-1.28]*
M51	105,880	112,836,700	1.08	0.91	0.83	0.17	0.25	0.17	1.30 [1.28-1.33]*	1.19 [1.16-1.21]*
M53	6,730	114,163,050	0.07	0.06	0.05	0.01	0.01	0.01	1.26 [1.16-1.36]*	1.12 [1.03-1.22]*
M54	267,020	110,400,930	2.82	2.32	2.17	0.50	0.65	0.45	1.30 [1.28-1.32]*	1.19 [1.17-1.21]*

M60	5,520	114,203,100	0.06	0.04	0.04	0.02	0.02	0.02	1.45 [1.33-1.58]*	1.38 [1.27-1.51]*
M62	23,300	114,043,900	0.23	0.20	0.19	0.03	0.04	0.03	1.22 [1.17-1.28]*	1.16 [1.11-1.22]*
M65	46,990	113,425,640	0.50	0.39	0.37	0.10	0.13	0.10	1.36 [1.32-1.40]*	1.25 [1.21-1.29]*
M66	4,650	114,190,050	0.05	0.04	0.04	0.01	0.01	0.01	1.29 [1.17-1.42]*	1.21 [1.10-1.34]*
M67	34,180	113,501,310	0.35	0.29	0.28	0.06	0.07	0.05	1.27 [1.22-1.31]*	1.19 [1.14-1.23]*
M70	18,030	113,983,870	0.20	0.15	0.14	0.05	0.06	0.05	1.44 [1.37-1.51]*	1.31 [1.25-1.38]*
M71	11,430	114,126,080	0.12	0.10	0.09	0.02	0.03	0.02	1.34 [1.26-1.42]*	1.24 [1.16-1.32]*
M72	25,940	113,780,260	0.23	0.23	0.21	0.00	0.02	0.02	1.12 [1.07-1.17]*	1.08 [1.03-1.13]*
M75	72,830	112,974,880	0.74	0.62	0.56	0.12	0.18	0.13	1.32 [1.28-1.35]*	1.20 [1.17-1.23]*
M76	6,040	114,192,140	0.07	0.05	0.05	0.02	0.02	0.01	1.42 [1.31-1.54]*	1.28 [1.18-1.40]*
M77	14,150	114,001,050	0.16	0.12	0.11	0.04	0.05	0.04	1.47 [1.40-1.56]*	1.33 [1.26-1.40]*
M79	223,590	111,199,670	2.48	1.90	1.78	0.58	0.70	0.53	1.39 [1.37-1.41]*	1.27 [1.25-1.29]*
M80	26,260	114,072,310	0.27	0.22	0.21	0.04	0.06	0.05	1.28 [1.23-1.34]*	1.22 [1.16-1.27]*
M81	160,120	113,090,090	1.66	1.36	1.27	0.30	0.39	0.30	1.31 [1.28-1.33]*	1.22 [1.20-1.24]*
M83	1,020	114,256,300	0.01	0.01	0.01	0.00	0.00	0.00	1.34 [1.09-1.65]*	1.22 [0.98-1.51]
M84	24,110	113,895,630	0.24	0.21	0.19	0.03	0.04	0.03	1.23 [1.17-1.28]*	1.16 [1.11-1.21]*
M85	35,720	114,049,690	0.37	0.30	0.28	0.07	0.09	0.06	1.31 [1.27-1.36]*	1.20 [1.16-1.25]*
M86	16,520	114,088,300	0.18	0.14	0.13	0.04	0.05	0.04	1.37 [1.30-1.44]*	1.31 [1.25-1.38]*
M87	7,040	114,168,920	0.07	0.06	0.05	0.01	0.02	0.01	1.28 [1.18-1.39]*	1.21 [1.11-1.31]*
M88	4,420	114,196,890	0.04	0.04	0.03	0.00	0.01	0.00	1.17 [1.05-1.30]*	1.13 [1.01-1.26]
M89	18,810	113,941,420	0.19	0.16	0.15	0.03	0.04	0.03	1.29 [1.23-1.35]*	1.20 [1.14-1.26]*
M90	7,280	114,244,340	0.06	0.06	0.06	0.00	0.00	0.00	1.06 [0.97-1.15]	1.06 [0.97-1.15]
M91	1,780	114,230,500	0.02	0.01	0.02	0.00	0.00	0.00	1.19 [1.02-1.39]	1.16 [0.99-1.36]
M92	2,640	114,227,410	0.03	0.02	0.02	0.01	0.01	0.01	1.45 [1.28-1.64]*	1.31 [1.16-1.50]*
M93	5,140	114,166,960	0.05	0.04	0.04	0.01	0.01	0.01	1.31 [1.20-1.44]*	1.22 [1.11-1.34]*
M94	13,420	114,082,290	0.15	0.11	0.11	0.04	0.04	0.03	1.41 [1.33-1.49]*	1.26 [1.19-1.34]*
M95	3,480	114,166,320	0.04	0.03	0.03	0.01	0.01	0.01	1.36 [1.21-1.51]*	1.27 [1.13-1.42]*
M96	8,910	114,184,610	0.09	0.07	0.07	0.02	0.02	0.02	1.28 [1.19-1.38]*	1.20 [1.12-1.30]*
M99	2,560	114,248,490	0.03	0.02	0.02	0.00	0.01	0.00	1.34 [1.17-1.53]*	1.20 [1.05-1.38]*

any age - N00-N99

N00	590	114,260,870	0.01	0.00	0.00	0.00	0.00	0.00	1.32 [1.01-1.73]	1.30 [0.98-1.71]
N02	7,120	114,131,150	0.07	0.06	0.06	0.01	0.02	0.02	1.32 [1.22-1.43]*	1.27 [1.17-1.37]*
N03	27,140	114,059,330	0.28	0.23	0.21	0.05	0.07	0.06	1.34 [1.28-1.39]*	1.27 [1.22-1.32]*
N04	4,800	114,193,950	0.05	0.04	0.04	0.01	0.01	0.01	1.34 [1.22-1.48]*	1.28 [1.16-1.41]*
N05	4,660	114,194,980	0.05	0.04	0.04	0.02	0.02	0.02	1.48 [1.35-1.63]*	1.42 [1.29-1.56]*
N08	12,410	114,162,810	0.13	0.10	0.09	0.03	0.04	0.03	1.43 [1.35-1.52]*	1.35 [1.27-1.43]*
N10	5,850	114,176,950	0.06	0.05	0.05	0.01	0.01	0.01	1.17 [1.07-1.28]*	1.10 [1.01-1.21]
N11	2,670	114,219,050	0.03	0.02	0.02	0.00	0.01	0.00	1.25 [1.10-1.42]*	1.20 [1.05-1.37]*
N12	32,490	113,816,420	0.32	0.28	0.27	0.04	0.05	0.03	1.18 [1.13-1.22]*	1.10 [1.06-1.15]*
N13	68,690	113,613,290	0.60	0.61	0.57	−0.01	0.03	0.03	1.06 [1.03-1.09]*	1.05 [1.02-1.08]*
N14	960	114,264,240	0.01	0.01	0.01	0.00	0.00	0.00	1.34 [1.08-1.66]*	1.29 [1.03-1.61]
N15	1,770	114,250,340	0.02	0.02	0.01	0.00	0.00	0.00	1.20 [1.02-1.41]	1.13 [0.96-1.33]
N16	860	114,260,020	0.01	0.01	0.01	0.00	0.00	0.00	1.61 [1.29-2.00]*	1.53 [1.22-1.92]*
N17	358,760	113,040,440	3.54	3.09	2.89	0.46	0.65	0.56	1.23 [1.21-1.24]*	1.19 [1.17-1.20]*
N18	300,050	112,886,960	3.01	2.57	2.39	0.43	0.62	0.51	1.26 [1.24-1.28]*	1.21 [1.19-1.22]*
N19	44,610	113,877,480	0.45	0.38	0.34	0.07	0.10	0.09	1.30 [1.26-1.34]*	1.24 [1.20-1.28]*
N20	54,980	113,371,610	0.50	0.48	0.44	0.02	0.06	0.04	1.15 [1.11-1.18]*	1.10 [1.06-1.13]*
N21	13,520	114,098,760	0.11	0.12	0.11	−0.01	0.00	0.00	1.02 [0.96-1.08]	1.00 [0.94-1.07]
N23	12,720	113,886,210	0.12	0.11	0.10	0.01	0.02	0.01	1.17 [1.11-1.25]*	1.08 [1.02-1.15]
N25	2,660	114,238,490	0.03	0.02	0.02	0.00	0.01	0.01	1.31 [1.15-1.48]*	1.25 [1.10-1.43]*
N26	5,200	114,238,570	0.05	0.04	0.04	0.01	0.01	0.01	1.20 [1.09-1.32]*	1.14 [1.03-1.26]*
N27	2,920	114,237,040	0.03	0.02	0.02	0.01	0.01	0.01	1.30 [1.15-1.47]*	1.27 [1.12-1.44]*
N28	73,690	113,670,770	0.70	0.64	0.59	0.07	0.11	0.08	1.18 [1.15-1.22]*	1.13 [1.11-1.16]*
N29	680	114,259,590	0.01	0.01	0.01	0.00	0.00	0.00	1.32 [1.02-1.71]	1.30 [1.00-1.68]
N30	33,960	113,705,020	0.34	0.29	0.27	0.05	0.07	0.06	1.27 [1.22-1.32]*	1.20 [1.16-1.25]*
N31	10,280	114,042,850	0.11	0.09	0.08	0.02	0.03	0.02	1.35 [1.26-1.43]*	1.24 [1.16-1.32]*
N32	99,370	112,750,230	0.98	0.86	0.79	0.12	0.18	0.14	1.23 [1.20-1.26]*	1.17 [1.14-1.19]*
N34	1,740	114,230,940	0.02	0.01	0.01	0.00	0.00	0.00	1.35 [1.16-1.58]*	1.30 [1.11-1.53]*
N35	34,270	113,608,330	0.35	0.29	0.27	0.06	0.08	0.07	1.31 [1.27-1.36]*	1.25 [1.20-1.30]*
N36	9,740	114,098,100	0.10	0.08	0.08	0.01	0.02	0.01	1.23 [1.15-1.32]*	1.17 [1.10-1.26]*
N39	448,510	109,115,830	4.58	4.00	3.82	0.59	0.76	0.61	1.20 [1.19-1.21]*	1.15 [1.14-1.17]*
N40	144,020	112,509,190	1.39	1.25	1.19	0.13	0.20	0.15	1.16 [1.14-1.19]*	1.12 [1.10-1.14]*
N41	10,590	114,084,470	0.09	0.09	0.08	0.00	0.01	0.01	1.15 [1.08-1.23]*	1.10 [1.02-1.17]*
N42	13,750	114,059,490	0.12	0.12	0.11	0.00	0.01	0.01	1.11 [1.05-1.18]*	1.07 [1.00-1.13]
N43	21,610	113,825,610	0.21	0.19	0.19	0.02	0.02	0.02	1.13 [1.08-1.18]*	1.10 [1.05-1.15]*
N44	7,350	114,153,960	0.08	0.06	0.07	0.03	0.01	0.01	1.18 [1.09-1.27]*	1.16 [1.07-1.25]*
N45	15,500	114,008,990	0.15	0.13	0.13	0.02	0.03	0.02	1.21 [1.15-1.28]*	1.15 [1.09-1.22]*
N47	58,760	113,030,600	0.71	0.47	0.49	0.24	0.22	0.20	1.46 [1.42-1.50]*	1.40 [1.37-1.44]*
N48	30,710	113,710,890	0.35	0.25	0.25	0.10	0.10	0.09	1.40 [1.35-1.46]*	1.34 [1.29-1.40]*
N49	3,590	114,215,000	0.04	0.03	0.03	0.01	0.01	0.01	1.35 [1.21-1.50]*	1.29 [1.16-1.45]*
N50	33,020	113,634,280	0.34	0.28	0.28	0.06	0.06	0.05	1.22 [1.17-1.26]*	1.16 [1.12-1.21]*
N51	450	114,264,070	0.00	0.00	0.00	0.00	0.00	0.00	1.48 [1.08-2.03]	1.37 [0.99-1.90]
N60	10,790	113,963,480	0.10	0.09	0.09	0.01	0.01	0.01	1.17 [1.10-1.25]*	1.13 [1.06-1.21]*
N61	7,260	114,106,170	0.08	0.06	0.06	0.02	0.02	0.02	1.34 [1.24-1.44]*	1.28 [1.18-1.38]*
N62	6,270	114,065,990	0.06	0.05	0.05	0.01	0.01	0.01	1.28 [1.18-1.39]*	1.21 [1.11-1.32]*
N63	11,490	113,955,840	0.11	0.10	0.09	0.01	0.01	0.01	1.16 [1.09-1.23]*	1.11 [1.04-1.18]*
N64	11,000	114,038,650	0.11	0.09	0.09	0.01	0.02	0.02	1.25 [1.18-1.34]*	1.19 [1.12-1.27]*
N70	7,890	114,082,010	0.07	0.07	0.07	0.00	0.00	0.00	1.07 [0.99-1.15]	1.03 [0.95-1.11]
N71	3,790	114,181,890	0.04	0.03	0.03	0.00	0.01	0.00	1.17 [1.05-1.31]*	1.08 [0.97-1.21]
N72	7,510	113,992,030	0.07	0.07	0.06	0.00	0.01	0.00	1.09 [1.01-1.18]	1.04 [0.96-1.13]
N73	42,730	113,241,550	0.40	0.37	0.35	0.03	0.05	0.03	1.14 [1.10-1.17]*	1.07 [1.04-1.11]*
N75	7,440	114,060,100	0.07	0.07	0.06	0.00	0.00	0.00	1.06 [0.97-1.15]	1.04 [0.95-1.12]
N76	11,480	113,999,530	0.13	0.09	0.09	0.03	0.04	0.03	1.39 [1.31-1.47]*	1.31 [1.23-1.39]*
N77	14,070	114,011,200	0.15	0.12	0.11	0.03	0.03	0.02	1.28 [1.21-1.36]*	1.19 [1.12-1.26]*
N80	42,360	113,185,470	0.40	0.37	0.35	0.03	0.05	0.02	1.13 [1.09-1.17]*	1.06 [1.02-1.10]*
N81	68,030	112,718,500	0.66	0.59	0.54	0.07	0.11	0.08	1.21 [1.18-1.24]*	1.14 [1.12-1.17]*
N82	3,230	114,224,420	0.03	0.03	0.03	0.01	0.01	0.01	1.32 [1.18-1.49]*	1.24 [1.11-1.40]*
N83	70,540	112,757,740	0.67	0.61	0.59	0.06	0.08	0.04	1.13 [1.10-1.16]*	1.07 [1.04-1.10]*
N84	56,920	112,868,180	0.52	0.50	0.46	0.02	0.06	0.05	1.12 [1.09-1.16]*	1.10 [1.07-1.13]*
N85	45,220	113,342,110	0.43	0.39	0.37	0.04	0.06	0.05	1.17 [1.14-1.21]*	1.13 [1.09-1.16]*
N86	13,580	113,875,230	0.13	0.12	0.11	0.01	0.02	0.02	1.19 [1.12-1.26]*	1.13 [1.07-1.20]*
N87	16,590	113,540,450	0.15	0.15	0.13	0.00	0.01	0.01	1.10 [1.05-1.17]*	1.06 [1.01-1.12]
N88	13,420	113,961,110	0.13	0.12	0.11	0.01	0.02	0.02	1.21 [1.14-1.28]*	1.16 [1.10-1.23]*
N89	23,000	113,670,890	0.24	0.19	0.18	0.04	0.05	0.04	1.29 [1.23-1.34]*	1.21 [1.16-1.27]*
N90	21,160	113,798,310	0.23	0.18	0.16	0.05	0.06	0.05	1.38 [1.32-1.45]*	1.31 [1.25-1.37]*
N91	2,610	114,203,250	0.03	0.02	0.02	0.01	0.01	0.00	1.29 [1.13-1.47]*	1.22 [1.06-1.39]*
N92	92,940	111,576,480	0.93	0.81	0.77	0.12	0.16	0.11	1.21 [1.18-1.23]*	1.13 [1.11-1.16]*
N93	43,170	113,028,540	0.42	0.37	0.35	0.05	0.07	0.05	1.20 [1.16-1.24]*	1.14 [1.10-1.17]*
N94	35,030	113,126,440	0.36	0.30	0.28	0.06	0.07	0.05	1.26 [1.21-1.30]*	1.16 [1.12-1.20]*
N95	48,570	113,029,600	0.48	0.42	0.38	0.06	0.10	0.08	1.25 [1.21-1.29]*	1.20 [1.16-1.24]*
N96	680	114,255,380	0.01	0.01	0.01	0.00	0.00	0.00	1.08 [0.83-1.41]	1.07 [0.81-1.41]
N97	7,800	113,712,940	0.06	0.07	0.06	−0.01	0.00	0.00	0.93 [0.86-1.01]	0.92 [0.85-1.00]
N98	950	114,241,510	0.01	0.01	0.01	0.00	0.00	0.00	0.80 [0.63-1.03]	0.76 [0.59-0.98]
N99	12,220	114,038,560	0.12	0.10	0.10	0.01	0.02	0.02	1.22 [1.15-1.30]*	1.17 [1.10-1.25]*

any age - O00-O9A

O00	13,390	113,900,020	0.12	0.12	0.11	0.00	0.00	0.00	1.04 [0.98-1.11]	1.02 [0.95-1.08]
O01	800	114,247,910	0.01	0.01	0.01	0.00	0.00	0.00	1.17 [0.92-1.49]	1.19 [0.92-1.53]

O02	30,480	113,193,690	0.28	0.27	0.25	0.01	0.02	0.02	1.10 [1.05-1.14]*	1.07 [1.02-1.11]*
O03	46,050	112,760,510	0.41	0.41	0.39	0.01	0.02	0.01	1.06 [1.02-1.09]*	1.04 [1.00-1.07]
O04	59,590	112,296,900	0.54	0.53	0.53	0.01	0.01	0.00	1.02 [1.00-1.05]	1.01 [0.98-1.04]
O08	4,820	114,155,580	0.04	0.04	0.04	0.00	0.00	0.00	1.05 [0.95-1.16]	1.01 [0.91-1.12]
O10	4,880	114,160,380	0.05	0.04	0.04	0.00	0.01	0.00	1.13 [1.02-1.25]	1.10 [0.99-1.21]
O11	700	114,254,240	0.01	0.01	0.01	0.00	0.00	0.00	1.17 [0.90-1.51]	1.12 [0.86-1.45]
O12	10,150	113,910,560	0.10	0.09	0.08	0.01	0.01	0.01	1.13 [1.06-1.21]*	1.08 [1.01-1.16]
O13	28,060	113,330,620	0.26	0.25	0.24	0.01	0.02	0.01	1.08 [1.04-1.13]*	1.06 [1.01-1.11]*
O14	20,420	113,620,810	0.19	0.18	0.17	0.01	0.02	0.01	1.10 [1.05-1.16]*	1.07 [1.02-1.13]*
O15	810	114,244,590	0.01	0.01	0.01	0.00	0.00	0.00	1.10 [0.86-1.41]	1.08 [0.84-1.39]
O16	22,710	113,298,600	0.21	0.20	0.19	0.01	0.02	0.02	1.12 [1.07-1.17]*	1.09 [1.04-1.14]*
O20	40,380	112,914,650	0.37	0.35	0.35	0.02	0.02	0.01	1.06 [1.03-1.10]*	1.03 [0.99-1.07]
O21	28,640	113,456,490	0.27	0.25	0.25	0.03	0.03	0.01	1.11 [1.06-1.15]*	1.04 [1.00-1.09]
O22	4,560	114,161,090	0.04	0.04	0.04	0.00	0.00	0.00	1.11 [1.00-1.23]	1.09 [0.98-1.21]
O23	31,230	113,319,110	0.31	0.27	0.26	0.04	0.04	0.03	1.16 [1.12-1.20]*	1.11 [1.07-1.15]*
O24	37,970	113,620,160	0.35	0.33	0.33	0.02	0.02	0.02	1.07 [1.03-1.11]*	1.05 [1.01-1.09]*
O26	116,280	110,304,620	1.19	1.02	1.03	0.17	0.16	0.11	1.16 [1.13-1.18]*	1.10 [1.08-1.13]*
O28	11,630	114,067,930	0.11	0.10	0.10	0.01	0.01	0.00	1.06 [0.99-1.13]	1.01 [0.95-1.08]
O30	14,430	113,890,610	0.12	0.13	0.12	−0.01	0.00	0.00	1.00 [0.94-1.06]	0.98 [0.92-1.04]
O31	810	114,249,620	0.01	0.01	0.01	0.00	0.00	0.00	1.05 [0.82-1.34]	1.01 [0.78-1.30]
O32	57,040	112,728,690	0.51	0.51	0.49	0.00	0.02	0.01	1.03 [1.00-1.06]	1.02 [0.99-1.05]
O33	1,350	114,201,270	0.01	0.01	0.01	0.00	0.00	0.00	1.15 [0.95-1.39]	1.13 [0.93-1.37]
O34	93,950	112,009,270	0.87	0.83	0.80	0.04	0.07	0.06	1.09 [1.06-1.11]*	1.07 [1.05-1.10]*
O35	14,160	113,953,850	0.12	0.12	0.12	0.00	0.00	0.00	1.02 [0.96-1.08]	1.00 [0.94-1.06]
O36	154,890	110,226,410	1.57	1.37	1.41	0.20	0.16	0.12	1.12 [1.10-1.14]*	1.08 [1.06-1.10]*
O40	12,970	114,033,840	0.12	0.11	0.11	0.01	0.01	0.01	1.09 [1.03-1.16]*	1.05 [0.99-1.12]
O41	17,530	113,721,110	0.16	0.15	0.15	0.01	0.01	0.01	1.10 [1.04-1.16]*	1.08 [1.02-1.14]*
O42	93,460	111,714,650	0.85	0.83	0.83	0.02	0.03	0.02	1.03 [1.01-1.06]*	1.03 [1.01-1.05]
O43	12,900	113,868,720	0.11	0.11	0.11	0.00	0.00	0.00	1.03 [0.97-1.10]	1.02 [0.96-1.09]
O44	8,740	114,036,220	0.08	0.08	0.07	0.00	0.00	0.00	1.03 [0.95-1.11]	0.99 [0.91-1.07]
O45	3,840	114,160,950	0.03	0.03	0.03	0.00	0.00	0.00	1.02 [0.91-1.14]	1.00 [0.89-1.12]
O46	43,420	112,887,170	0.41	0.38	0.37	0.03	0.04	0.03	1.12 [1.09-1.16]*	1.09 [1.05-1.13]*
O47	30,950	112,334,650	0.29	0.27	0.25	0.02	0.04	0.03	1.16 [1.12-1.21]*	1.12 [1.07-1.16]*
O48	57,620	112,150,800	0.50	0.52	0.50	−0.01	0.01	0.01	1.02 [0.99-1.05]	1.02 [0.99-1.05]
O60	48,180	112,889,580	0.44	0.42	0.41	0.02	0.03	0.02	1.08 [1.04-1.11]*	1.05 [1.01-1.08]*
O61	9,070	113,980,620	0.08	0.08	0.08	0.00	0.01	0.00	1.08 [1.01-1.17]	1.05 [0.97-1.13]
O62	36,590	113,332,360	0.33	0.32	0.31	0.01	0.02	0.02	1.05 [1.01-1.09]*	1.05 [1.01-1.09]
O63	83,060	111,318,330	0.74	0.75	0.73	0.00	0.01	0.01	1.02 [0.99-1.04]	1.01 [0.98-1.03]
O64	16,270	113,596,780	0.14	0.14	0.14	−0.01	0.00	0.00	1.02 [0.97-1.08]	1.02 [0.96-1.08]
O65	1,190	114,198,580	0.01	0.01	0.01	0.00	0.00	0.00	0.98 [0.80-1.21]	0.99 [0.80-1.22]
O66	15,620	113,743,600	0.14	0.14	0.13	0.00	0.00	0.00	1.01 [0.96-1.07]	1.00 [0.94-1.06]
O67	4,160	114,121,330	0.04	0.04	0.03	0.00	0.00	0.00	1.02 [0.91-1.13]	1.01 [0.90-1.13]
O68	173,530	108,628,640	1.69	1.57	1.62	0.12	0.07	0.06	1.04 [1.03-1.06]*	1.03 [1.02-1.05]*
O69	16,010	113,458,940	0.14	0.14	0.13	0.00	0.01	0.01	1.09 [1.03-1.15]*	1.08 [1.02-1.14]*
O70	232,730	106,299,640	2.34	2.15	2.26	0.19	0.08	0.08	1.03 [1.02-1.05]*	1.04 [1.02-1.05]*
O71	14,160	113,835,660	0.12	0.12	0.12	0.00	0.00	0.00	1.01 [0.96-1.08]	1.01 [0.95-1.07]
O72	117,020	111,597,950	1.09	1.04	1.04	0.06	0.06	0.05	1.05 [1.03-1.08]*	1.05 [1.02-1.07]*
O73	8,220	113,964,730	0.07	0.07	0.07	0.00	0.01	0.01	1.10 [1.02-1.19]	1.09 [1.01-1.18]
O74	2,230	114,210,570	0.02	0.02	0.02	0.00	0.00	0.00	1.16 [1.00-1.34]	1.12 [0.96-1.30]
O75	58,240	112,705,400	0.53	0.51	0.51	0.01	0.02	0.02	1.04 [1.01-1.07]*	1.04 [1.01-1.07]
O80	62,900	111,215,660	0.58	0.56	0.54	0.02	0.04	0.05	1.08 [1.05-1.11]*	1.09 [1.06-1.12]*
O81	990	114,158,510	0.01	0.01	0.01	0.00	0.00	0.00	1.07 [0.86-1.34]	1.06 [0.84-1.33]
O82	9,060	113,774,780	0.08	0.08	0.07	0.00	0.01	0.01	1.10 [1.03-1.19]*	1.08 [1.00-1.16]
O85	4,840	114,198,180	0.05	0.04	0.04	0.00	0.00	0.00	1.07 [0.97-1.19]	1.03 [0.93-1.15]
O86	17,780	113,820,400	0.17	0.15	0.15	0.02	0.02	0.02	1.15 [1.09-1.21]*	1.12 [1.06-1.18]*
O87	2,590	114,172,450	0.02	0.02	0.02	0.00	0.00	0.00	1.19 [1.04-1.36]	1.17 [1.02-1.34]
O88	950	114,251,140	0.01	0.01	0.01	0.00	0.00	0.00	1.15 [0.92-1.44]	1.07 [0.85-1.35]
O89	760	114,246,810	0.01	0.01	0.01	0.00	0.00	0.00	1.32 [1.03-1.68]	1.25 [0.97-1.62]
O90	20,880	113,847,250	0.20	0.18	0.17	0.03	0.03	0.02	1.18 [1.12-1.24]*	1.14 [1.08-1.19]*
O91	2,130	114,226,930	0.02	0.02	0.02	0.00	0.00	0.00	1.02 [0.87-1.18]	0.99 [0.84-1.16]
O92	3,220	114,176,540	0.03	0.03	0.03	0.00	0.00	0.00	1.11 [0.98-1.25]	1.07 [0.94-1.21]
O94	670	114,264,770	0.01	0.01	0.01	0.00	0.00	0.00	1.26 [0.97-1.63]	1.14 [0.86-1.50]
O98	7,740	114,083,070	0.08	0.07	0.07	0.01	0.01	0.01	1.15 [1.07-1.25]*	1.11 [1.02-1.20]
O99	127,350	110,852,290	1.30	1.11	1.12	0.19	0.18	0.13	1.16 [1.14-1.19]*	1.11 [1.09-1.13]*
any age - P00-P96										
P83	1,660	113,235,600	0.02	0.01	0.02	0.00	0.00	0.00	1.02 [0.87-1.20]	1.02 [0.86-1.20]
any age - Q00-Q99										
Q02	2,060	114,217,950	0.02	0.02	0.02	0.00	0.00	0.00	1.10 [0.96-1.27]	1.05 [0.91-1.21]
Q03	850	114,229,970	0.01	0.01	0.01	0.00	0.00	0.00	1.20 [0.96-1.50]	1.15 [0.91-1.45]
Q04	2,220	114,196,740	0.02	0.02	0.02	0.00	0.00	0.00	1.08 [0.94-1.25]	1.01 [0.87-1.17]
Q05	2,140	114,210,590	0.02	0.02	0.02	0.00	0.00	0.00	1.29 [1.12-1.49]*	1.18 [1.02-1.37]
Q06	650	114,258,070	0.01	0.01	0.01	0.00	0.00	0.00	0.91 [0.70-1.20]	0.88 [0.66-1.16]
Q07	2,530	114,236,140	0.03	0.02	0.02	0.01	0.00	0.00	1.20 [1.05-1.36]*	1.09 [0.95-1.24]
Q10	2,600	114,203,990	0.03	0.02	0.02	0.01	0.00	0.00	1.19 [1.05-1.35]*	1.18 [1.04-1.34]
Q12	1,410	114,232,500	0.01	0.01	0.01	0.00	0.00	0.00	1.22 [1.02-1.46]	1.17 [0.98-1.41]
Q13	510	114,254,330	0.01	0.00	0.00	0.00	0.00	0.00	1.33 [1.00-1.77]	1.21 [0.90-1.62]
Q14	680	114,258,230	0.01	0.01	0.01	0.00	0.00	0.00	1.11 [0.86-1.44]	1.07 [0.82-1.39]
Q16	430	114,260,970	0.00	0.00	0.00	0.00	0.00	0.00	1.28 [0.93-1.75]	1.24 [0.90-1.70]
Q17	6,290	114,016,810	0.06	0.05	0.06	0.01	0.00	0.00	1.08 [0.99-1.17]	1.06 [0.97-1.15]
Q18	3,470	114,172,350	0.04	0.03	0.03	0.01	0.01	0.00	1.16 [1.04-1.30]*	1.13 [1.01-1.27]
Q20	660	114,225,600	0.01	0.01	0.01	0.00	0.00	0.00	1.01 [0.77-1.32]	0.98 [0.75-1.30]
Q21	10,710	113,839,690	0.11	0.09	0.09	0.01	0.01	0.01	1.16 [1.09-1.24]*	1.10 [1.03-1.18]*
Q22	1,360	114,205,230	0.01	0.01	0.01	0.00	0.00	0.00	0.96 [0.79-1.15]	0.96 [0.79-1.17]
Q23	4,780	114,181,960	0.05	0.04	0.04	0.00	0.01	0.00	1.15 [1.04-1.26]*	1.11 [1.01-1.23]
Q24	3,670	114,156,270	0.04	0.03	0.03	0.01	0.01	0.00	1.17 [1.05-1.31]*	1.10 [0.99-1.23]
Q25	3,950	113,944,950	0.04	0.03	0.04	0.00	0.00	0.00	1.06 [0.95-1.18]	1.03 [0.92-1.14]
Q26	640	114,251,980	0.01	0.01	0.01	0.00	0.00	0.00	1.11 [0.85-1.44]	1.04 [0.80-1.37]
Q27	3,180	114,209,260	0.03	0.03	0.03	0.00	0.00	0.00	1.08 [0.96-1.22]	1.04 [0.92-1.18]
Q28	1,700	114,237,990	0.02	0.01	0.01	0.00	0.00	0.00	1.32 [1.12-1.54]*	1.27 [1.08-1.49]*
Q31	1,360	114,183,290	0.01	0.01	0.01	0.00	0.00	0.00	1.03 [0.86-1.24]	0.94 [0.78-1.14]
Q32	590	114,251,740	0.01	0.01	0.01	0.00	0.00	0.00	0.96 [0.73-1.27]	0.85 [0.64-1.13]
Q33	540	114,235,040	0.01	0.00	0.00	0.00	0.00	0.00	1.05 [0.79-1.41]	0.93 [0.69-1.26]
Q35	1,500	114,200,270	0.02	0.01	0.01	0.00	0.00	0.00	1.11 [0.94-1.32]	1.06 [0.89-1.26]
Q38	10,400	113,612,180	0.11	0.09	0.10	0.02	0.01	0.01	1.14 [1.07-1.21]*	1.11 [1.04-1.18]*
Q39	1,690	114,236,620	0.02	0.01	0.01	0.00	0.00	0.00	1.27 [1.08-1.49]*	1.17 [0.99-1.39]
Q40	1,070	114,173,750	0.01	0.01	0.01	0.00	0.00	0.00	1.24 [1.01-1.52]	1.11 [0.90-1.38]
Q43	4,350	114,155,360	0.04	0.04	0.04	0.01	0.01	0.00	1.17 [1.06-1.29]*	1.10 [0.99-1.22]
Q44	1,380	114,244,540	0.01	0.01	0.01	0.00	0.00	0.00	1.15 [0.95-1.38]	1.09 [0.90-1.31]
Q45	570	114,261,670	0.01	0.00	0.00	0.00	0.00	0.00	1.34 [1.02-1.76]	1.24 [0.93-1.64]
Q50	3,050	114,197,920	0.03	0.03	0.02	0.01	0.01	0.01	1.25 [1.11-1.41]*	1.19 [1.06-1.35]*
Q51	3,040	114,210,700	0.03	0.03	0.03	0.00	0.00	0.00	1.06 [0.94-1.21]	1.01 [0.89-1.15]
Q52	1,600	114,220,550	0.02	0.01	0.01	0.00	0.00	0.00	1.21 [1.03-1.42]	1.12 [0.94-1.32]
Q53	13,720	113,693,310	0.14	0.12	0.14	0.02	0.00	0.00	1.00 [0.94-1.06]	0.98 [0.92-1.04]
Q54	4,020	114,021,240	0.04	0.03	0.04	0.01	0.00	0.00	1.11 [1.00-1.23]	1.11 [0.99-1.23]
Q55	8,670	114,071,870	0.10	0.07	0.09	0.03	0.01	0.01	1.13 [1.06-1.22]*	1.11 [1.03-1.19]*
Q60	2,940	114,218,010	0.03	0.03	0.02	0.00	0.00	0.00	1.17 [1.03-1.33]	1.12 [0.99-1.28]
Q61	7,280	114,141,410	0.07	0.06	0.06	0.01	0.01	0.01	1.16 [1.07-1.26]*	1.11 [1.03-1.21]

Q62	1,720	114,068,190	0.02	0.01	0.02	0.00	0.00	0.00	1.00 [0.85-1.18]	0.98 [0.83-1.16]
Q63	5,700	114,135,950	0.06	0.05	0.05	0.01	0.01	0.00	1.16 [1.06-1.27]*	1.09 [0.99-1.19]
Q64	1,260	114,213,790	0.01	0.01	0.01	0.00	0.00	0.00	1.15 [0.95-1.38]	1.09 [0.90-1.32]
Q65	4,710	114,080,300	0.04	0.04	0.04	0.00	0.00	0.00	1.04 [0.94-1.15]	0.99 [0.89-1.09]
Q66	5,520	113,410,300	0.06	0.05	0.05	0.02	0.01	0.01	1.26 [1.15-1.37]*	1.19 [1.09-1.30]*
Q67	3,570	114,163,700	0.04	0.03	0.03	0.01	0.01	0.00	1.20 [1.07-1.33]*	1.14 [1.02-1.28]
Q68	1,240	114,224,690	0.01	0.01	0.01	0.00	0.00	0.00	1.21 [1.00-1.46]	1.16 [0.96-1.40]
Q69	630	114,162,420	0.01	0.01	0.01	0.00	0.00	0.00	0.90 [0.69-1.18]	0.88 [0.67-1.15]
Q70	600	114,213,280	0.01	0.01	0.01	0.00	0.00	0.00	0.87 [0.65-1.16]	0.87 [0.65-1.16]
Q72	540	114,253,520	0.01	0.00	0.00	0.00	0.00	0.00	1.18 [0.88-1.57]	1.15 [0.86-1.54]
Q74	3,740	114,182,900	0.04	0.03	0.03	0.01	0.01	0.01	1.21 [1.09-1.35]*	1.17 [1.05-1.31]*
Q75	1,990	114,181,440	0.02	0.02	0.02	0.00	0.00	0.00	1.03 [0.89-1.19]	0.99 [0.85-1.15]
Q76	2,910	114,204,820	0.03	0.02	0.03	0.00	0.00	0.00	1.15 [1.02-1.31]	1.06 [0.94-1.21]
Q78	1,860	114,236,790	0.02	0.02	0.02	0.00	0.00	0.00	1.22 [1.05-1.42]	1.15 [0.99-1.35]
Q79	3,700	114,186,580	0.04	0.03	0.03	0.01	0.01	0.00	1.35 [1.22-1.51]*	1.13 [1.01-1.26]
Q80	280	114,263,110	0.01	0.00	0.00	0.00	0.00	0.00	2.79 [1.97-3.95]*	2.58 [1.81-3.68]*
Q82	3,160	113,550,950	0.04	0.03	0.03	0.01	0.01	0.01	1.36 [1.21-1.52]*	1.28 [1.14-1.44]*
Q83	1,700	114,203,600	0.02	0.01	0.01	0.00	0.00	0.00	1.13 [0.96-1.33]	1.06 [0.90-1.26]
Q85	2,610	114,216,440	0.02	0.02	0.02	0.00	0.00	0.00	1.02 [0.89-1.16]	0.97 [0.85-1.12]
Q87	3,680	114,172,710	0.04	0.03	0.03	0.01	0.01	0.01	1.23 [1.10-1.37]*	1.15 [1.03-1.29]
Q89	3,360	114,191,290	0.03	0.03	0.03	0.00	0.00	0.00	1.09 [0.97-1.22]	1.06 [0.94-1.19]
Q90	2,300	114,135,210	0.04	0.02	0.01	0.02	0.02	0.02	2.77 [2.46-3.12]*	2.68 [2.37-3.02]*
Q92	650	114,261,790	0.01	0.01	0.01	0.00	0.00	0.00	1.12 [0.87-1.45]	1.07 [0.83-1.40]
Q93	1,770	114,242,640	0.02	0.01	0.02	0.01	0.00	0.00	1.27 [1.09-1.47]*	1.17 [1.00-1.36]
Q96	500	114,254,210	0.01	0.00	0.00	0.00	0.00	0.00	1.62 [1.23-2.13]*	1.65 [1.24-2.19]*
Q98	500	114,258,130	0.01	0.00	0.00	0.00	0.00	0.00	1.77 [1.35-2.32]*	1.60 [1.21-2.11]*
Q99	2,120	114,235,370	0.02	0.02	0.02	0.01	0.00	0.00	1.16 [1.00-1.33]	1.08 [0.94-1.25]
any age - R00-R99										
R00	206,340	112,193,240	2.07	1.79	1.71	0.28	0.36	0.26	1.21 [1.19-1.23]*	1.14 [1.13-1.16]*
R01	33,350	113,688,640	0.34	0.28	0.28	0.06	0.06	0.04	1.21 [1.17-1.26]*	1.15 [1.11-1.19]*
R02	30,180	114,087,600	0.29	0.26	0.24	0.04	0.05	0.05	1.22 [1.17-1.27]*	1.19 [1.14-1.24]*
R03	71,720	113,546,050	0.69	0.62	0.58	0.08	0.11	0.09	1.18 [1.15-1.22]*	1.14 [1.11-1.17]*
R04	80,700	113,133,360	0.85	0.68	0.64	0.17	0.21	0.17	1.33 [1.30-1.36]*	1.25 [1.22-1.28]*
R05	85,010	113,011,960	0.95	0.70	0.69	0.25	0.26	0.20	1.38 [1.35-1.41]*	1.27 [1.24-1.30]*
R06	282,150	109,316,170	3.56	2.35	2.35	1.22	1.22	1.07	1.52 [1.50-1.54]*	1.43 [1.41-1.44]*
R07	341,950	107,976,700	3.67	3.05	2.84	0.62	0.83	0.60	1.29 [1.28-1.31]*	1.20 [1.18-1.21]*
R09	30,610	113,956,370	0.32	0.26	0.24	0.06	0.07	0.06	1.31 [1.26-1.36]*	1.22 [1.17-1.26]*
R10	498,030	103,240,900	5.74	4.60	4.52	1.13	1.21	0.88	1.27 [1.26-1.28]*	1.18 [1.17-1.19]*
R11	292,230	109,627,550	3.11	2.56	2.53	0.56	0.58	0.42	1.23 [1.22-1.25]*	1.16 [1.14-1.17]*
R12	18,070	113,951,110	0.19	0.15	0.14	0.04	0.05	0.03	1.35 [1.29-1.42]*	1.22 [1.16-1.28]*
R13	133,640	113,119,120	1.39	1.13	1.06	0.25	0.33	0.25	1.31 [1.29-1.34]*	1.22 [1.19-1.24]*
R14	31,190	113,859,920	0.32	0.26	0.25	0.06	0.08	0.05	1.31 [1.27-1.36]*	1.20 [1.16-1.25]*
R15	56,780	113,978,110	0.55	0.48	0.46	0.07	0.09	0.08	1.20 [1.16-1.24]*	1.16 [1.13-1.20]*
R16	21,970	114,065,630	0.24	0.18	0.17	0.05	0.07	0.05	1.38 [1.32-1.45]*	1.30 [1.24-1.36]*
R17	18,030	114,036,950	0.17	0.15	0.14	0.02	0.03	0.03	1.22 [1.16-1.28]*	1.19 [1.13-1.25]*
R18	45,510	114,016,960	0.43	0.39	0.36	0.04	0.07	0.06	1.20 [1.17-1.24]*	1.17 [1.14-1.21]*
R19	172,930	112,074,130	1.83	1.47	1.37	0.36	0.46	0.35	1.34 [1.31-1.36]*	1.23 [1.21-1.25]*
R20	47,880	113,608,950	0.49	0.41	0.38	0.08	0.10	0.07	1.27 [1.23-1.30]*	1.16 [1.12-1.20]*
R21	76,360	112,572,500	0.92	0.62	0.63	0.30	0.29	0.26	1.46 [1.42-1.49]*	1.39 [1.35-1.42]*
R22	37,270	113,599,990	0.41	0.31	0.30	0.10	0.11	0.09	1.38 [1.34-1.43]*	1.29 [1.25-1.34]*
R23	30,470	113,695,690	0.33	0.25	0.26	0.07	0.06	0.05	1.25 [1.20-1.29]*	1.19 [1.14-1.24]*
R25	28,180	113,750,170	0.29	0.24	0.23	0.05	0.05	0.04	1.24 [1.19-1.29]*	1.15 [1.10-1.20]*
R26	208,060	113,406,160	2.07	1.78	1.71	0.29	0.36	0.29	1.21 [1.19-1.23]*	1.16 [1.15-1.18]*
R27	8,710	114,174,010	0.09	0.07	0.07	0.01	0.01	0.01	1.18 [1.10-1.27]*	1.12 [1.04-1.21]*
R29	273,190	112,756,550	2.69	2.36	2.26	0.33	0.43	0.34	1.19 [1.18-1.21]*	1.15 [1.13-1.16]*
R30	22,530	113,905,650	0.24	0.19	0.18	0.06	0.06	0.05	1.35 [1.29-1.41]*	1.24 [1.19-1.30]*
R31	149,230	111,674,990	1.45	1.31	1.21	0.14	0.24	0.19	1.19 [1.17-1.22]*	1.15 [1.13-1.17]*
R32	109,890	113,571,950	1.07	0.94	0.90	0.13	0.17	0.14	1.19 [1.17-1.22]*	1.15 [1.13-1.18]*
R33	206,830	112,713,930	1.99	1.80	1.70	0.19	0.28	0.23	1.17 [1.15-1.19]*	1.13 [1.11-1.15]*
R34	5,610	114,236,720	0.06	0.05	0.05	0.01	0.01	0.01	1.21 [1.11-1.33]*	1.15 [1.05-1.27]*
R35	34,910	113,516,350	0.37	0.29	0.27	0.07	0.09	0.08	1.35 [1.30-1.40]*	1.26 [1.21-1.30]*
R36	760	114,259,380	0.01	0.01	0.01	0.00	0.00	0.00	1.28 [1.00-1.63]	1.25 [0.97-1.60]
R39	40,110	113,494,870	0.42	0.34	0.31	0.08	0.11	0.08	1.35 [1.30-1.39]*	1.25 [1.21-1.29]*
R40	33,190	114,004,880	0.33	0.28	0.28	0.05	0.06	0.04	1.21 [1.17-1.26]*	1.15 [1.11-1.20]*
R41	199,520	113,248,680	1.93	1.72	1.64	0.21	0.29	0.24	1.17 [1.16-1.19]*	1.14 [1.12-1.16]*
R42	111,950	113,041,220	1.12	0.96	0.91	0.17	0.21	0.15	1.24 [1.21-1.26]*	1.15 [1.13-1.18]*
R43	1,500	114,257,410	0.02	0.01	0.01	0.00	0.00	0.00	1.35 [1.14-1.61]*	1.23 [1.03-1.47]
R44	29,420	114,078,790	0.29	0.25	0.24	0.04	0.05	0.04	1.21 [1.16-1.26]*	1.15 [1.11-1.20]*
R45	67,540	113,855,600	0.69	0.57	0.58	0.12	0.11	0.08	1.19 [1.16-1.22]*	1.13 [1.10-1.16]*
R46	19,220	114,159,970	0.18	0.17	0.16	0.02	0.02	0.02	1.13 [1.08-1.19]*	1.11 [1.06-1.17]*
R47	55,830	113,859,260	0.53	0.48	0.46	0.05	0.08	0.06	1.17 [1.13-1.20]*	1.13 [1.09-1.16]*
R48	2,280	114,252,380	0.03	0.02	0.02	0.01	0.01	0.00	1.29 [1.12-1.48]*	1.15 [1.00-1.33]
R49	11,260	114,060,520	0.12	0.09	0.09	0.02	0.03	0.02	1.35 [1.27-1.44]*	1.23 [1.16-1.32]*
R50	125,160	112,045,030	1.33	1.07	1.09	0.27	0.24	0.19	1.22 [1.20-1.25]*	1.17 [1.15-1.19]*
R51	164,460	111,561,010	1.77	1.40	1.38	0.37	0.39	0.27	1.28 [1.26-1.30]*	1.18 [1.16-1.20]*
R52	25,490	113,999,400	0.28	0.21	0.20	0.06	0.08	0.06	1.40 [1.34-1.46]*	1.26 [1.21-1.31]*
R53	106,190	113,406,850	1.08	0.90	0.87	0.17	0.21	0.16	1.24 [1.21-1.27]*	1.18 [1.15-1.20]*
R54	128,060	113,818,500	1.22	1.10	1.05	0.12	0.18	0.15	1.17 [1.14-1.19]*	1.14 [1.12-1.17]*
R55	204,250	111,935,580	2.02	1.78	1.69	0.24	0.33	0.26	1.20 [1.18-1.21]*	1.15 [1.13-1.17]*
R56	96,350	112,250,170	0.96	0.83	0.84	0.13	0.12	0.09	1.14 [1.11-1.16]*	1.10 [1.08-1.13]*
R57	17,300	114,219,960	0.16	0.15	0.14	0.02	0.03	0.02	1.18 [1.12-1.25]*	1.15 [1.09-1.22]*
R58	2,750	114,246,900	0.03	0.02	0.02	0.01	0.01	0.01	1.35 [1.19-1.53]*	1.30 [1.14-1.48]*
R59	59,160	113,578,790	0.64	0.49	0.47	0.15	0.17	0.15	1.35 [1.32-1.39]*	1.31 [1.28-1.35]*
R60	92,340	113,763,250	0.96	0.78	0.72	0.18	0.24	0.20	1.33 [1.30-1.36]*	1.26 [1.23-1.29]*
R61	10,700	114,111,200	0.11	0.09	0.08	0.03	0.03	0.02	1.36 [1.27-1.45]*	1.24 [1.16-1.32]*
R62	16,720	113,655,560	0.17	0.14	0.16	0.03	0.01	0.00	1.07 [1.01-1.12]	1.02 [0.97-1.07]
R63	200,090	112,055,920	2.05	1.72	1.65	0.32	0.40	0.31	1.24 [1.22-1.26]*	1.18 [1.16-1.20]*
R64	5,220	114,260,690	0.05	0.05	0.04	0.00	0.01	0.01	1.16 [1.05-1.28]*	1.17 [1.06-1.30]*
R65	11,950	114,240,850	0.12	0.10	0.09	0.02	0.02	0.02	1.23 [1.16-1.31]*	1.18 [1.10-1.26]*
R68	19,650	113,923,150	0.19	0.17	0.16	0.02	0.03	0.03	1.21 [1.15-1.27]*	1.19 [1.13-1.25]*
R69	169,740	106,597,460	1.78	1.55	1.43	0.24	0.35	0.29	1.25 [1.23-1.27]*	1.19 [1.18-1.22]*
R70	1,440	114,239,870	0.02	0.01	0.01	0.00	0.01	0.00	1.50 [1.27-1.78]*	1.43 [1.20-1.70]*
R71	1,190	114,257,880	0.01	0.01	0.01	0.00	0.00	0.00	1.22 [1.00-1.49]	1.18 [0.97-1.45]
R72	6,570	114,210,350	0.06	0.06	0.05	0.01	0.01	0.01	1.23 [1.13-1.34]*	1.18 [1.09-1.29]*
R73	24,160	114,027,320	0.25	0.20	0.19	0.05	0.06	0.04	1.31 [1.26-1.37]*	1.21 [1.16-1.27]*
R74	10,960	114,178,830	0.11	0.09	0.09	0.01	0.02	0.01	1.21 [1.14-1.30]*	1.15 [1.07-1.23]*
R76	3,080	114,226,880	0.03	0.03	0.02	0.01	0.01	0.01	1.38 [1.23-1.56]*	1.31 [1.16-1.47]*
R77	6,710	114,229,350	0.07	0.06	0.05	0.01	0.01	0.01	1.26 [1.16-1.37]*	1.18 [1.09-1.29]*
R78	900	114,255,170	0.01	0.01	0.01	0.00	0.00	0.00	1.19 [0.95-1.49]	1.15 [0.91-1.45]
R79	129,520	113,225,980	1.29	1.11	1.04	0.18	0.25	0.19	1.24 [1.22-1.26]*	1.18 [1.15-1.20]*
R80	5,510	114,174,500	0.06	0.05	0.04	0.01	0.01	0.01	1.33 [1.22-1.45]*	1.28 [1.17-1.40]*
R81	1,600	114,177,010	0.02	0.01	0.01	0.00	0.00	0.00	1.20 [1.02-1.42]	1.15 [0.97-1.36]
R82	11,310	114,135,950	0.11	0.10	0.09	0.01	0.02	0.01	1.20 [1.13-1.28]*	1.15 [1.07-1.22]*
R83	490	114,263,100	0.00	0.00	0.00	0.00	0.00	0.00	1.20 [0.89-1.62]	1.14 [0.84-1.56]
R84	3,660	114,244,720	0.04	0.03	0.03	0.01	0.01	0.01	1.29 [1.15-1.44]*	1.16 [1.03-1.30]
R85	1,960	114,222,400	0.02	0.02	0.02	0.00	0.01	0.00	1.32 [1.14-1.53]*	1.21 [1.04-1.40]
R87	9,950	113,723,210	0.09	0.09	0.08	0.00	0.01	0.01	1.16 [1.08-1.24]*	1.12 [1.05-1.20]*
R89	1,830	114,250,300	0.02	0.01	0.01	0.01	0.01	0.01	1.48 [1.27-1.73]*	1.41 [1.21-1.65]*

R90	6,170	114,209,920	0.06	0.05	0.05	0.01	0.01	0.01	1.21 [1.11-1.32]*	1.16 [1.06-1.27]*
R91	63,380	113,817,410	0.63	0.54	0.50	0.09	0.13	0.11	1.26 [1.22-1.29]*	1.20 [1.17-1.24]*
R92	590	114,263,450	0.00	0.01	0.00	0.00	0.00	0.00	1.09 [0.81-1.46]	1.06 [0.78-1.43]
R93	64,880	113,674,380	0.63	0.56	0.52	0.07	0.11	0.09	1.21 [1.18-1.25]*	1.16 [1.13-1.19]*
R94	114,510	113,305,880	1.16	0.98	0.92	0.18	0.24	0.20	1.26 [1.24-1.29]*	1.21 [1.18-1.23]*
any age - S00-T88										
S00	126,580	112,940,920	1.23	1.10	1.08	0.13	0.14	0.11	1.13 [1.11-1.15]*	1.10 [1.08-1.12]*
S01	135,300	112,669,540	1.32	1.17	1.16	0.14	0.15	0.12	1.13 [1.11-1.15]*	1.10 [1.08-1.12]*
S02	52,400	113,241,270	0.50	0.45	0.45	0.04	0.05	0.04	1.11 [1.07-1.14]*	1.08 [1.04-1.11]*
S03	2,210	114,240,290	0.02	0.02	0.02	0.00	0.00	0.00	1.08 [0.94-1.25]	1.04 [0.90-1.21]
S05	11,420	114,107,500	0.11	0.10	0.09	0.01	0.01	0.01	1.14 [1.06-1.21]*	1.11 [1.03-1.18]*
S06	36,390	113,869,270	0.34	0.31	0.31	0.03	0.03	0.03	1.11 [1.07-1.15]*	1.09 [1.05-1.13]*
S09	72,790	112,786,440	0.72	0.63	0.63	0.10	0.10	0.07	1.16 [1.13-1.19]*	1.11 [1.08-1.14]*
S10	3,900	114,213,540	0.04	0.03	0.03	0.00	0.00	0.00	1.05 [0.94-1.17]	1.00 [0.89-1.12]
S11	2,080	114,239,200	0.02	0.02	0.02	0.00	0.00	0.00	1.19 [1.03-1.38]	1.13 [0.97-1.31]
S12	8,700	114,194,430	0.08	0.07	0.07	0.01	0.01	0.01	1.18 [1.09-1.27]*	1.15 [1.06-1.24]*
S13	2,320	114,217,790	0.02	0.02	0.02	0.00	0.00	0.00	1.20 [1.04-1.38]	1.12 [0.97-1.30]
S14	1,700	114,246,740	0.02	0.01	0.01	0.00	0.00	0.00	1.07 [0.90-1.27]	1.07 [0.90-1.27]
S19	3,000	114,209,090	0.03	0.03	0.02	0.00	0.01	0.00	1.24 [1.10-1.41]*	1.15 [1.02-1.30]
S20	13,120	114,136,550	0.13	0.11	0.11	0.02	0.02	0.01	1.18 [1.11-1.25]*	1.12 [1.05-1.19]*
S21	2,880	114,227,160	0.03	0.02	0.02	0.00	0.00	0.00	1.11 [0.97-1.26]	1.06 [0.93-1.21]
S22	42,790	113,855,770	0.41	0.37	0.34	0.05	0.07	0.06	1.21 [1.17-1.25]*	1.15 [1.12-1.19]*
S27	13,690	114,108,850	0.12	0.12	0.11	0.00	0.01	0.01	1.11 [1.05-1.18]*	1.08 [1.02-1.15]
S29	3,400	114,230,700	0.04	0.03	0.03	0.01	0.01	0.01	1.38 [1.23-1.55]*	1.27 [1.13-1.43]*
S30	25,390	113,987,990	0.25	0.22	0.21	0.03	0.04	0.03	1.18 [1.13-1.23]*	1.12 [1.07-1.17]*
S31	8,010	114,130,960	0.08	0.07	0.07	0.01	0.01	0.01	1.11 [1.03-1.19]*	1.07 [0.99-1.16]
S32	47,250	113,876,780	0.44	0.41	0.39	0.03	0.05	0.04	1.13 [1.09-1.16]*	1.09 [1.05-1.13]*
S33	600	114,260,130	0.01	0.01	0.01	0.00	0.00	0.00	1.08 [0.81-1.45]	1.02 [0.76-1.37]
S35	590	114,264,450	0.01	0.01	0.01	0.00	0.00	0.00	1.08 [0.82-1.43]	1.09 [0.82-1.45]
S36	5,840	114,189,370	0.05	0.05	0.05	0.00	0.01	0.00	1.11 [1.01-1.22]	1.09 [1.00-1.20]
S37	4,310	114,219,690	0.04	0.04	0.04	0.00	0.00	0.00	1.00 [0.90-1.12]	0.98 [0.87-1.09]
S39	10,090	114,106,080	0.10	0.09	0.08	0.01	0.01	0.01	1.17 [1.09-1.25]*	1.09 [1.02-1.17]
S40	19,220	114,125,830	0.18	0.16	0.16	0.02	0.02	0.01	1.14 [1.08-1.20]*	1.09 [1.03-1.15]*
S41	7,810	114,195,570	0.08	0.07	0.06	0.01	0.01	0.01	1.22 [1.13-1.32]*	1.16 [1.07-1.26]*
S42	68,860	113,381,550	0.66	0.60	0.59	0.06	0.06	0.05	1.11 [1.08-1.14]*	1.08 [1.06-1.11]*
S43	9,780	114,140,440	0.09	0.09	0.08	0.00	0.01	0.01	1.10 [1.02-1.18]*	1.07 [0.99-1.15]
S44	550	114,261,770	0.01	0.00	0.00	0.00	0.00	0.00	1.24 [0.93-1.66]	1.21 [0.90-1.63]
S46	4,000	114,229,020	0.04	0.03	0.03	0.00	0.00	0.00	1.10 [0.99-1.23]	1.05 [0.94-1.18]
S49	5,700	114,215,910	0.06	0.05	0.05	0.01	0.01	0.01	1.20 [1.09-1.31]*	1.12 [1.02-1.23]
S50	21,230	114,116,770	0.21	0.18	0.18	0.03	0.03	0.02	1.16 [1.10-1.22]*	1.11 [1.05-1.16]*
S51	30,580	114,007,980	0.30	0.26	0.26	0.04	0.04	0.02	1.15 [1.11-1.20]*	1.09 [1.04-1.14]*
S52	114,140	112,223,550	1.09	1.00	1.03	0.09	0.06	0.04	1.06 [1.04-1.08]*	1.04 [1.02-1.06]*
S53	3,250	114,209,060	0.03	0.03	0.03	0.00	0.00	0.00	0.98 [0.87-1.11]	0.95 [0.84-1.08]
S54	1,370	114,239,230	0.01	0.01	0.01	0.00	0.00	0.00	1.13 [0.94-1.35]	1.09 [0.90-1.32]
S55	600	114,258,500	0.01	0.01	0.00	0.00	0.00	0.00	1.34 [1.02-1.75]	1.33 [1.01-1.76]
S56	4,720	114,180,870	0.04	0.04	0.04	0.00	0.00	0.00	1.00 [0.90-1.11]	0.98 [0.88-1.09]
S59	2,430	114,243,250	0.02	0.02	0.02	0.00	0.00	0.00	1.24 [1.08-1.43]*	1.17 [1.01-1.35]
S60	17,040	114,106,760	0.16	0.15	0.14	0.02	0.02	0.01	1.14 [1.08-1.20]*	1.10 [1.04-1.16]*
S61	72,940	113,057,610	0.67	0.64	0.64	0.03	0.03	0.01	1.05 [1.02-1.08]*	1.02 [0.99-1.04]
S62	51,530	113,409,820	0.47	0.45	0.45	0.02	0.02	0.01	1.05 [1.02-1.08]*	1.02 [0.98-1.05]
S63	7,660	114,139,110	0.07	0.07	0.06	0.01	0.01	0.01	1.19 [1.11-1.29]*	1.14 [1.06-1.24]*
S64	10,010	114,049,320	0.08	0.09	0.08	0.00	0.00	0.00	1.00 [0.93-1.07]	0.98 [0.91-1.06]
S65	2,720	114,215,310	0.02	0.02	0.02	0.00	0.00	0.00	1.02 [0.89-1.17]	1.03 [0.90-1.18]
S66	18,380	113,853,050	0.16	0.16	0.15	0.00	0.00	0.00	1.03 [0.98-1.09]	1.01 [0.95-1.06]
S67	2,790	114,200,260	0.02	0.02	0.03	0.00	0.00	0.00	0.87 [0.76-1.00]	0.86 [0.75-0.98]
S68	6,380	114,130,470	0.05	0.06	0.06	0.00	0.00	0.00	0.98 [0.90-1.07]	0.98 [0.90-1.08]
S69	6,770	114,177,830	0.07	0.06	0.06	0.01	0.01	0.01	1.21 [1.11-1.32]*	1.15 [1.05-1.25]*
S70	18,860	114,128,460	0.18	0.16	0.16	0.02	0.02	0.02	1.16 [1.10-1.22]*	1.11 [1.05-1.17]*
S71	4,800	114,201,600	0.05	0.04	0.04	0.01	0.01	0.00	1.07 [0.97-1.18]	1.02 [0.92-1.13]
S72	128,350	113,226,460	1.19	1.12	1.08	0.07	0.11	0.10	1.10 [1.08-1.12]*	1.09 [1.07-1.11]*
S73	1,830	114,242,960	0.02	0.02	0.02	0.00	0.00	0.00	1.09 [0.93-1.28]	1.04 [0.88-1.22]
S76	3,980	114,221,130	0.04	0.03	0.03	0.00	0.00	0.00	1.14 [1.02-1.27]	1.07 [0.96-1.20]
S79	9,520	114,195,470	0.09	0.08	0.08	0.01	0.02	0.02	1.25 [1.16-1.34]*	1.20 [1.11-1.29]*
S80	36,840	113,968,360	0.35	0.32	0.31	0.04	0.04	0.02	1.14 [1.10-1.18]*	1.08 [1.04-1.12]*
S81	28,110	113,996,560	0.28	0.24	0.23	0.04	0.05	0.03	1.20 [1.15-1.26]*	1.12 [1.07-1.17]*
S82	81,330	112,712,560	0.75	0.72	0.69	0.03	0.06	0.04	1.09 [1.06-1.11]*	1.05 [1.03-1.08]*
S83	10,720	114,056,350	0.10	0.09	0.09	0.01	0.01	0.01	1.15 [1.08-1.23]*	1.08 [1.01-1.16]
S86	5,340	114,144,630	0.05	0.05	0.04	0.00	0.01	0.00	1.14 [1.04-1.25]*	1.08 [0.98-1.19]
S89	7,640	114,181,540	0.08	0.06	0.06	0.01	0.01	0.01	1.23 [1.14-1.33]*	1.15 [1.07-1.25]*
S90	9,660	114,170,210	0.09	0.08	0.08	0.01	0.01	0.00	1.11 [1.04-1.20]*	1.06 [0.98-1.14]
S91	10,680	114,107,390	0.11	0.09	0.09	0.02	0.01	0.01	1.12 [1.05-1.19]*	1.07 [1.00-1.15]
S92	16,520	113,997,170	0.15	0.14	0.14	0.01	0.02	0.01	1.13 [1.07-1.19]*	1.08 [1.02-1.14]*
S93	5,220	114,193,160	0.05	0.04	0.04	0.01	0.01	0.01	1.19 [1.09-1.31]*	1.12 [1.01-1.23]
S96	1,280	114,247,900	0.01	0.01	0.01	0.00	0.00	0.00	1.14 [0.95-1.38]	1.06 [0.87-1.29]
S99	3,990	114,220,780	0.04	0.03	0.03	0.00	0.00	0.00	1.15 [1.03-1.28]	1.06 [0.94-1.18]
T00	8,680	114,200,910	0.08	0.07	0.07	0.01	0.01	0.01	1.12 [1.04-1.21]*	1.07 [0.99-1.16]
T01	4,150	114,237,960	0.04	0.04	0.04	0.01	0.00	0.00	1.12 [1.00-1.25]	1.05 [0.94-1.18]
T02	2,800	114,236,060	0.03	0.02	0.02	0.00	0.00	0.00	1.17 [1.03-1.33]	1.15 [1.01-1.32]
T09	1,400	114,248,940	0.01	0.01	0.01	0.00	0.00	0.00	1.22 [1.02-1.47]	1.14 [0.95-1.38]
T11	5,760	114,220,790	0.06	0.05	0.05	0.01	0.01	0.01	1.19 [1.08-1.31]*	1.12 [1.01-1.23]
T13	3,680	114,228,430	0.03	0.03	0.03	0.00	0.00	0.00	1.17 [1.04-1.31]	1.10 [0.97-1.24]
T14	3,670	114,165,040	0.03	0.03	0.03	0.00	0.00	0.00	1.16 [1.03-1.29]	1.09 [0.97-1.22]
T15	880	114,249,860	0.01	0.01	0.01	0.00	0.00	0.00	1.05 [0.83-1.32]	1.03 [0.82-1.30]
T16	8,910	114,111,310	0.09	0.07	0.09	0.02	0.00	0.00	1.05 [0.98-1.12]	1.03 [0.96-1.11]
T17	13,540	114,041,250	0.13	0.11	0.12	0.02	0.02	0.01	1.14 [1.07-1.20]*	1.08 [1.02-1.15]*
T18	10,690	114,086,140	0.11	0.09	0.09	0.02	0.01	0.01	1.16 [1.09-1.24]*	1.09 [1.02-1.16]
T19	1,020	114,252,480	0.01	0.01	0.01	0.00	0.00	0.00	1.19 [0.96-1.46]	1.11 [0.89-1.37]
T20	4,680	114,165,740	0.04	0.04	0.04	0.00	0.00	0.00	0.99 [0.89-1.09]	0.98 [0.88-1.08]
T21	6,670	114,135,910	0.06	0.06	0.06	0.00	0.00	0.00	1.00 [0.92-1.08]	0.99 [0.91-1.08]
T22	5,570	114,153,870	0.05	0.05	0.05	0.00	0.00	0.00	1.01 [0.92-1.11]	1.00 [0.91-1.09]
T23	5,650	114,150,400	0.05	0.05	0.05	0.00	0.00	0.00	1.01 [0.93-1.11]	1.00 [0.91-1.09]
T24	4,870	114,182,010	0.04	0.04	0.05	0.00	0.00	0.00	0.92 [0.83-1.02]	0.92 [0.83-1.02]
T25	2,490	114,222,310	0.02	0.02	0.02	0.00	0.00	0.00	0.88 [0.77-1.02]	0.85 [0.74-0.98]
T29	1,080	114,248,500	0.01	0.01	0.01	0.00	0.00	0.00	0.93 [0.75-1.15]	0.90 [0.73-1.12]
T31	15,310	113,970,280	0.14	0.13	0.14	0.01	0.00	−0.01	0.97 [0.92-1.02]	0.95 [0.90-1.01]
T36	3,430	114,188,880	0.04	0.03	0.03	0.01	0.01	0.01	1.26 [1.13-1.41]*	1.17 [1.04-1.32]*
T37	1,290	114,241,450	0.01	0.01	0.01	0.00	0.00	0.00	1.30 [1.08-1.56]*	1.21 [1.00-1.46]
T38	5,380	114,184,200	0.06	0.04	0.04	0.01	0.01	0.01	1.26 [1.15-1.37]*	1.14 [1.04-1.25]*
T39	64,110	112,832,120	0.63	0.55	0.57	0.08	0.06	0.03	1.10 [1.07-1.13]*	1.05 [1.02-1.08]*
T40	27,960	113,812,350	0.27	0.24	0.23	0.03	0.04	0.02	1.18 [1.13-1.22]*	1.08 [1.03-1.12]*
T41	730	114,260,920	0.01	0.01	0.01	0.00	0.00	0.00	1.18 [0.92-1.50]	1.06 [0.82-1.37]
T42	20,040	113,769,620	0.19	0.17	0.17	0.02	0.03	0.01	1.17 [1.12-1.23]*	1.08 [1.02-1.13]*
T43	32,120	113,520,310	0.32	0.27	0.27	0.04	0.04	0.03	1.16 [1.12-1.21]*	1.09 [1.05-1.13]*
T44	6,900	114,140,510	0.07	0.06	0.06	0.01	0.01	0.01	1.16 [1.07-1.26]*	1.10 [1.01-1.19]
T45	9,170	114,112,260	0.10	0.08	0.08	0.02	0.02	0.01	1.26 [1.18-1.35]*	1.15 [1.08-1.24]*
T46	6,220	114,185,630	0.06	0.05	0.05	0.01	0.01	0.01	1.19 [1.10-1.30]*	1.11 [1.02-1.22]
T47	3,580	114,212,760	0.04	0.03	0.03	0.01	0.00	0.00	1.15 [1.03-1.29]	1.04 [0.92-1.17]
T48	1,910	114,224,170	0.02	0.02	0.02	0.00	0.00	0.00	1.20 [1.04-1.40]	1.08 [0.93-1.26]

T49	1,610	114,226,920	0.02	0.01	0.01	0.00	0.00	0.00	1.02 [0.87-1.21]	0.98 [0.82-1.16]
T50	8,420	114,035,030	0.08	0.07	0.07	0.01	0.01	0.01	1.18 [1.09-1.27]*	1.08 [1.00-1.17]
T51	26,670	113,639,370	0.25	0.23	0.22	0.02	0.03	0.02	1.14 [1.10-1.19]*	1.07 [1.03-1.12]*
T52	1,610	114,229,250	0.01	0.01	0.02	0.00	0.00	0.00	0.87 [0.74-1.04]	0.85 [0.72-1.02]
T54	1,630	114,242,270	0.02	0.01	0.02	0.00	0.00	0.00	0.98 [0.82-1.16]	0.95 [0.80-1.14]
T56	720	114,255,770	0.01	0.01	0.01	0.00	0.00	0.00	1.17 [0.91-1.50]	1.10 [0.85-1.42]
T58	600	114,254,090	0.01	0.01	0.00	0.00	0.00	0.00	1.08 [0.81-1.43]	1.07 [0.80-1.43]
T59	2,130	114,223,220	0.02	0.02	0.02	0.00	0.00	0.00	1.05 [0.90-1.23]	1.02 [0.87-1.19]
T62	780	114,249,880	0.01	0.01	0.01	0.00	0.00	0.00	1.24 [0.98-1.56]	1.17 [0.92-1.48]
T63	1,010	114,246,240	0.01	0.01	0.01	0.00	0.00	0.00	1.10 [0.89-1.37]	1.02 [0.82-1.27]
T65	1,640	114,233,320	0.02	0.01	0.01	0.00	0.00	0.00	1.05 [0.89-1.24]	1.00 [0.84-1.18]
T68	9,510	114,232,630	0.09	0.08	0.08	0.00	0.01	0.01	1.10 [1.02-1.19]	1.12 [1.04-1.21]*
T71	870	114,257,950	0.01	0.01	0.01	0.00	0.00	0.00	0.96 [0.76-1.22]	0.94 [0.74-1.21]
T74	1,580	114,238,740	0.01	0.01	0.02	0.00	0.00	0.00	0.91 [0.76-1.08]	0.86 [0.72-1.03]
T75	1,080	114,241,720	0.01	0.01	0.01	0.00	0.00	0.00	1.09 [0.89-1.34]	1.02 [0.83-1.26]
T78	24,690	113,700,390	0.48	0.15	0.16	0.33	0.33	0.29	3.07 [2.96-3.18]*	2.54 [2.45-2.64]*
T79	12,940	114,111,220	0.12	0.11	0.11	0.00	0.01	0.01	1.09 [1.03-1.17]*	1.07 [1.00-1.14]
T80	5,910	114,184,060	0.06	0.05	0.05	0.01	0.02	0.01	1.33 [1.23-1.45]*	1.27 [1.16-1.39]*
T81	180,710	111,293,390	1.89	1.56	1.46	0.33	0.43	0.34	1.29 [1.27-1.31]*	1.22 [1.20-1.24]*
T82	42,800	113,763,580	0.44	0.36	0.34	0.08	0.10	0.09	1.31 [1.27-1.35]*	1.25 [1.21-1.29]*
T83	42,140	113,964,420	0.39	0.37	0.34	0.02	0.05	0.04	1.14 [1.10-1.18]*	1.11 [1.07-1.15]*
T84	56,750	113,346,570	0.58	0.48	0.44	0.10	0.14	0.11	1.31 [1.28-1.35]*	1.24 [1.20-1.27]*
T85	38,000	113,776,720	0.39	0.32	0.30	0.07	0.09	0.07	1.30 [1.26-1.35]*	1.23 [1.19-1.28]*
T86	5,280	114,190,880	0.05	0.04	0.04	0.01	0.01	0.01	1.33 [1.22-1.46]*	1.29 [1.18-1.41]*
T87	3,190	114,227,160	0.03	0.03	0.02	0.01	0.01	0.01	1.35 [1.20-1.52]*	1.25 [1.11-1.41]*
T88	11,840	114,017,800	0.13	0.10	0.09	0.04	0.04	0.03	1.47 [1.39-1.56]*	1.33 [1.25-1.41]*
T90	9,870	114,056,180	0.10	0.08	0.08	0.02	0.02	0.02	1.29 [1.21-1.38]*	1.23 [1.15-1.31]*
T91	4,200	114,187,190	0.04	0.04	0.03	0.01	0.01	0.01	1.30 [1.17-1.44]*	1.19 [1.07-1.32]*
T92	7,940	114,081,110	0.07	0.07	0.06	0.01	0.01	0.01	1.17 [1.09-1.26]*	1.12 [1.04-1.21]*
T93	10,340	114,041,320	0.10	0.09	0.08	0.01	0.02	0.01	1.22 [1.15-1.31]*	1.15 [1.07-1.23]*
T94	900	114,253,490	0.01	0.01	0.01	0.00	0.00	0.00	1.26 [1.01-1.57]	1.21 [0.96-1.52]
T95	1,110	114,243,120	0.01	0.01	0.01	0.00	0.00	0.00	0.97 [0.79-1.19]	0.96 [0.78-1.18]
T98	1,600	114,242,620	0.02	0.01	0.01	0.00	0.00	0.00	1.19 [1.01-1.41]	1.10 [0.92-1.30]
any age - U00-U89										
U07	52,700	114,218,800	0.54	0.44	0.43	0.10	0.11	0.08	1.27 [1.23-1.31]*	1.18 [1.15-1.22]*
U80	18,650	114,146,070	0.21	0.15	0.14	0.06	0.07	0.06	1.49 [1.42-1.56]*	1.40 [1.33-1.47]*
U81	1,080	114,265,170	0.01	0.01	0.01	0.00	0.00	0.00	1.19 [0.96-1.46]	1.14 [0.92-1.41]
U82	37,090	114,163,650	0.41	0.30	0.29	0.10	0.12	0.10	1.40 [1.35-1.45]*	1.32 [1.27-1.37]*
U83	22,120	114,211,120	0.24	0.18	0.18	0.06	0.06	0.05	1.35 [1.29-1.41]*	1.28 [1.22-1.34]*
U84	450	114,269,520	0.00	0.00	0.00	0.00	0.00	0.00	1.40 [1.02-1.93]	1.35 [0.97-1.87]
U88	4,540	114,243,610	0.05	0.04	0.04	0.01	0.01	0.01	1.29 [1.16-1.42]*	1.22 [1.10-1.35]*
U89	5,180	114,237,580	0.06	0.04	0.04	0.01	0.01	0.01	1.34 [1.22-1.47]*	1.26 [1.14-1.38]*
any age - V00-Y99										
V01	690	114,258,600	0.01	0.01	0.01	0.00	0.00	0.00	0.99 [0.76-1.29]	0.96 [0.73-1.26]
V02	600	114,257,400	0.01	0.01	0.01	0.00	0.00	0.00	0.99 [0.74-1.32]	0.99 [0.74-1.33]
V03	11,280	114,025,790	0.10	0.10	0.10	0.00	0.00	0.00	1.02 [0.96-1.09]	1.00 [0.94-1.07]
V04	700	114,254,370	0.01	0.01	0.01	0.00	0.00	0.00	1.32 [1.03-1.70]	1.27 [0.98-1.64]
V09	2,480	114,235,660	0.03	0.02	0.02	0.00	0.00	0.00	1.21 [1.05-1.38]*	1.13 [0.98-1.30]
V11	620	114,261,740	0.01	0.01	0.01	0.00	0.00	0.00	1.00 [0.76-1.31]	0.95 [0.71-1.26]
V13	3,230	114,208,290	0.03	0.03	0.03	0.00	0.00	0.00	0.91 [0.80-1.03]	0.91 [0.80-1.03]
V17	1,250	114,250,880	0.01	0.01	0.01	0.00	0.00	0.00	0.98 [0.80-1.19]	0.97 [0.79-1.18]
V18	19,430	113,897,890	0.18	0.17	0.18	0.01	0.00	0.00	1.02 [0.97-1.07]	1.00 [0.95-1.05]
V19	2,920	114,218,500	0.03	0.02	0.03	0.00	0.00	0.00	1.07 [0.95-1.22]	1.05 [0.92-1.19]
V23	4,020	114,183,990	0.03	0.04	0.03	0.00	0.00	0.00	0.96 [0.86-1.08]	0.96 [0.86-1.08]
V27	980	114,249,490	0.01	0.01	0.01	0.00	0.00	0.00	0.87 [0.69-1.10]	0.82 [0.64-1.04]
V28	5,530	114,158,440	0.05	0.05	0.05	0.00	0.00	0.00	1.02 [0.92-1.12]	0.99 [0.90-1.09]
V29	1,380	114,234,020	0.01	0.01	0.01	0.00	0.00	0.00	1.05 [0.87-1.26]	1.01 [0.83-1.22]
V43	9,210	114,052,090	0.09	0.08	0.07	0.01	0.01	0.01	1.14 [1.06-1.22]*	1.09 [1.01-1.17]
V44	850	114,249,730	0.01	0.01	0.01	0.00	0.00	0.00	1.19 [0.95-1.50]	1.11 [0.87-1.40]
V47	4,250	114,183,920	0.04	0.04	0.04	0.00	0.01	0.00	1.16 [1.04-1.28]*	1.11 [1.00-1.24]
V48	5,190	114,189,050	0.05	0.04	0.04	0.00	0.01	0.00	1.14 [1.03-1.25]*	1.09 [0.99-1.21]
V49	3,980	114,164,690	0.04	0.03	0.03	0.00	0.00	0.00	1.11 [0.99-1.23]	1.08 [0.96-1.20]
V78	1,940	114,248,810	0.02	0.02	0.02	0.00	0.00	0.00	1.14 [0.98-1.34]	1.09 [0.93-1.29]
V79	660	114,260,040	0.01	0.01	0.01	0.00	0.00	0.00	1.08 [0.83-1.42]	1.03 [0.78-1.35]
V80	5,990	114,148,660	0.05	0.05	0.05	0.00	0.00	0.00	0.95 [0.86-1.04]	0.93 [0.85-1.02]
V82	480	114,264,050	0.01	0.00	0.00	0.00	0.00	0.00	1.42 [1.05-1.92]	1.42 [1.05-1.93]
V86	1,430	114,252,040	0.01	0.01	0.01	0.00	0.00	0.00	1.08 [0.90-1.29]	1.02 [0.85-1.23]
V89	1,790	114,193,790	0.02	0.02	0.01	0.00	0.00	0.00	1.20 [1.02-1.40]	1.12 [0.95-1.32]
W00	7,880	114,139,160	0.07	0.07	0.06	0.00	0.01	0.00	1.09 [1.01-1.18]	1.07 [0.99-1.16]
W01	163,090	112,375,980	1.57	1.42	1.38	0.15	0.19	0.15	1.14 [1.12-1.16]*	1.10 [1.08-1.12]*
W02	8,990	114,090,990	0.09	0.08	0.09	0.01	0.00	0.00	1.01 [0.94-1.09]	0.99 [0.92-1.07]
W03	12,540	114,034,150	0.12	0.11	0.12	0.01	0.01	0.00	1.05 [0.99-1.11]	1.01 [0.95-1.08]
W04	1,980	114,152,760	0.02	0.02	0.02	0.00	0.00	0.00	1.01 [0.87-1.18]	1.00 [0.86-1.17]
W05	3,280	114,243,210	0.03	0.03	0.03	0.01	0.01	0.01	1.24 [1.10-1.39]*	1.19 [1.05-1.34]*
W06	38,680	113,959,670	0.38	0.33	0.32	0.05	0.05	0.04	1.17 [1.12-1.21]*	1.13 [1.09-1.17]*
W07	23,040	114,022,370	0.22	0.20	0.20	0.03	0.03	0.02	1.14 [1.09-1.19]*	1.11 [1.06-1.16]*
W08	7,900	114,138,180	0.08	0.07	0.07	0.01	0.01	0.00	1.09 [1.00-1.17]	1.06 [0.98-1.15]
W09	23,160	113,882,380	0.24	0.19	0.23	0.05	0.01	0.01	1.05 [1.01-1.10]	1.04 [0.99-1.08]
W10	66,850	113,331,210	0.63	0.58	0.55	0.05	0.08	0.06	1.15 [1.12-1.18]*	1.11 [1.08-1.14]*
W11	9,730	114,073,200	0.08	0.09	0.08	0.00	0.00	0.00	1.06 [0.98-1.13]	1.02 [0.94-1.09]
W12	590	114,256,110	0.01	0.01	0.00	0.00	0.00	0.00	1.19 [0.90-1.57]	1.16 [0.88-1.55]
W13	8,370	114,075,130	0.08	0.07	0.08	0.00	0.00	0.00	0.99 [0.92-1.07]	0.97 [0.90-1.05]
W14	2,960	114,202,700	0.03	0.03	0.03	0.00	0.00	0.00	0.99 [0.87-1.12]	0.99 [0.87-1.12]
W17	15,790	113,943,730	0.15	0.14	0.14	0.01	0.01	0.00	1.05 [0.99-1.11]	1.02 [0.97-1.08]
W18	108,010	113,415,770	1.07	0.93	0.90	0.14	0.17	0.14	1.19 [1.16-1.22]*	1.15 [1.12-1.17]*
W19	243,800	112,102,860	2.40	2.12	2.04	0.28	0.36	0.29	1.17 [1.16-1.19]*	1.14 [1.12-1.16]*
W20	7,300	114,125,080	0.07	0.06	0.06	0.00	0.00	0.00	1.03 [0.95-1.11]	0.99 [0.91-1.07]
W21	6,690	114,143,180	0.07	0.06	0.06	0.01	0.01	0.01	1.11 [1.03-1.21]	1.08 [0.99-1.18]
W22	35,960	113,708,720	0.35	0.31	0.31	0.04	0.04	0.02	1.12 [1.08-1.16]*	1.08 [1.04-1.12]*
W23	23,440	113,856,700	0.22	0.20	0.21	0.02	0.00	0.00	1.02 [0.98-1.07]	1.00 [0.96-1.05]
W25	11,920	113,960,230	0.11	0.10	0.10	0.00	0.00	0.00	1.02 [0.96-1.09]	1.00 [0.94-1.07]
W26	10,520	114,105,850	0.10	0.09	0.09	0.01	0.01	0.00	1.06 [0.99-1.13]	1.02 [0.95-1.09]
W27	4,890	114,161,880	0.04	0.04	0.04	0.00	0.00	0.00	0.99 [0.89-1.10]	0.95 [0.86-1.06]
W28	670	114,256,060	0.00	0.01	0.01	0.00	0.00	0.00	0.85 [0.63-1.13]	0.83 [0.62-1.11]
W29	5,040	114,182,900	0.04	0.04	0.04	0.00	0.00	0.00	0.99 [0.89-1.09]	0.98 [0.88-1.08]
W31	4,680	114,163,100	0.04	0.04	0.04	0.00	0.00	0.00	1.02 [0.92-1.13]	1.02 [0.92-1.13]
W34	790	114,249,910	0.01	0.01	0.01	0.00	0.00	0.00	1.06 [0.83-1.35]	1.03 [0.80-1.33]
W44	25,020	113,834,870	0.25	0.21	0.23	0.04	0.02	0.01	1.10 [1.06-1.15]*	1.06 [1.02-1.11]*
W45	8,300	114,089,460	0.08	0.07	0.07	0.01	0.01	0.00	1.07 [0.99-1.15]	1.03 [0.96-1.12]
W46	990	114,264,200	0.01	0.01	0.01	0.00	0.00	0.00	1.08 [0.86-1.34]	0.99 [0.78-1.24]
W49	2,110	114,217,390	0.02	0.02	0.02	0.00	0.00	0.00	1.19 [1.03-1.38]	1.18 [1.02-1.37]
W50	11,980	114,046,680	0.12	0.10	0.11	0.02	0.01	0.01	1.11 [1.05-1.18]*	1.08 [1.01-1.15]
W51	9,270	114,083,900	0.09	0.08	0.09	0.01	0.01	0.00	1.06 [0.99-1.14]	1.03 [0.96-1.10]
W54	12,010	114,086,980	0.11	0.11	0.11	0.00	0.00	−0.01	0.98 [0.92-1.05]	0.95 [0.89-1.01]
W55	5,310	114,191,360	0.04	0.05	0.05	0.00	0.00	0.00	0.98 [0.89-1.08]	0.92 [0.83-1.02]
W57	6,480	114,173,710	0.07	0.05	0.05	0.02	0.02	0.02	1.40 [1.29-1.52]*	1.31 [1.21-1.43]*
W60	590	114,261,330	0.01	0.00	0.00	0.00	0.00	0.00	1.35 [1.04-1.76]	1.27 [0.97-1.67]

W64	1,330	114,257,110	0.01	0.01	0.01	0.00	0.00	0.00	1.04 [0.86-1.26]	1.03 [0.85-1.25]
W78	3,720	114,257,790	0.03	0.03	0.03	0.00	0.00	0.00	1.10 [0.97-1.23]	1.07 [0.95-1.21]
W79	5,690	114,228,490	0.06	0.05	0.05	0.01	0.01	0.01	1.27 [1.16-1.39]*	1.21 [1.10-1.32]*
W80	2,320	114,256,690	0.02	0.02	0.02	0.00	0.00	0.00	1.18 [1.02-1.37]	1.12 [0.96-1.30]
W84	2,160	114,264,860	0.02	0.02	0.02	0.00	0.00	0.00	1.06 [0.90-1.24]	1.03 [0.88-1.21]
X04	700	114,255,340	0.01	0.01	0.01	0.00	0.00	0.00	1.02 [0.78-1.33]	0.99 [0.75-1.29]
X08	660	114,257,940	0.01	0.01	0.01	0.00	0.00	0.00	1.04 [0.79-1.37]	1.04 [0.79-1.37]
X09	930	114,250,620	0.01	0.01	0.01	0.00	0.00	0.00	1.01 [0.80-1.27]	0.98 [0.78-1.25]
X10	4,420	114,173,720	0.04	0.04	0.04	0.00	0.00	0.00	1.00 [0.91-1.11]	0.99 [0.89-1.10]
X11	1,400	114,237,020	0.01	0.01	0.01	0.00	0.00	0.00	0.97 [0.81-1.17]	0.94 [0.78-1.14]
X12	2,670	114,206,380	0.02	0.02	0.03	0.00	0.00	0.00	0.94 [0.82-1.08]	0.92 [0.81-1.06]
X15	1,860	114,234,120	0.02	0.02	0.02	0.00	0.00	0.00	1.04 [0.89-1.22]	1.03 [0.88-1.21]
X16	1,320	114,249,860	0.01	0.01	0.01	0.00	0.00	0.00	0.99 [0.82-1.21]	0.96 [0.79-1.17]
X19	860	114,255,330	0.01	0.01	0.01	0.00	0.00	0.00	1.12 [0.89-1.41]	1.07 [0.85-1.36]
X23	1,330	114,246,880	0.01	0.01	0.01	0.00	0.00	0.00	1.17 [0.97-1.41]	1.09 [0.90-1.32]
X31	1,320	114,263,320	0.01	0.01	0.01	0.00	0.00	0.00	1.05 [0.85-1.29]	1.06 [0.86-1.31]
X40	15,820	113,938,010	0.15	0.14	0.14	0.02	0.01	0.00	1.07 [1.02-1.13]*	1.03 [0.97-1.09]
X41	9,200	114,055,650	0.09	0.08	0.08	0.01	0.01	0.01	1.15 [1.08-1.24]*	1.08 [1.01-1.16]
X42	8,270	114,166,080	0.08	0.07	0.07	0.01	0.01	0.01	1.22 [1.13-1.31]*	1.11 [1.03-1.20]*
X43	1,940	114,235,500	0.02	0.02	0.02	0.00	0.00	0.00	1.10 [0.94-1.29]	1.05 [0.90-1.24]
X44	12,970	114,020,420	0.13	0.11	0.11	0.02	0.02	0.01	1.17 [1.10-1.24]*	1.09 [1.03-1.16]*
X45	3,580	114,181,050	0.03	0.03	0.03	0.00	0.00	0.00	1.12 [1.00-1.26]	1.07 [0.95-1.21]
X46	1,100	114,239,960	0.01	0.01	0.01	0.00	0.00	0.00	0.91 [0.73-1.12]	0.88 [0.71-1.09]
X47	1,300	114,244,730	0.01	0.01	0.01	0.00	0.00	0.00	1.11 [0.92-1.34]	1.08 [0.89-1.31]
X49	5,210	114,144,650	0.06	0.04	0.04	0.02	0.02	0.01	1.35 [1.24-1.47]*	1.27 [1.16-1.38]*
X50	18,770	113,982,060	0.17	0.16	0.16	0.01	0.02	0.01	1.11 [1.06-1.17]*	1.05 [1.00-1.10]
X51	1,240	114,250,550	0.01	0.01	0.01	0.00	0.00	0.00	1.19 [0.98-1.44]	1.15 [0.95-1.40]
X58	5,410	114,195,550	0.07	0.04	0.04	0.02	0.03	0.02	1.60 [1.47-1.75]*	1.44 [1.32-1.58]*
X59	77,870	112,918,590	0.78	0.67	0.65	0.11	0.13	0.09	1.19 [1.16-1.22]*	1.14 [1.11-1.17]*
X60	50,280	113,147,240	0.50	0.43	0.45	0.07	0.05	0.03	1.11 [1.08-1.15]*	1.06 [1.02-1.09]*
X61	34,660	113,448,130	0.34	0.30	0.29	0.04	0.05	0.03	1.16 [1.12-1.21]*	1.08 [1.04-1.13]*
X62	19,650	113,924,460	0.19	0.17	0.17	0.02	0.03	0.01	1.16 [1.10-1.22]*	1.05 [1.00-1.11]
X63	4,860	114,180,910	0.05	0.04	0.04	0.01	0.01	0.00	1.17 [1.06-1.28]*	1.10 [1.00-1.21]
X64	17,510	113,879,610	0.18	0.15	0.15	0.03	0.03	0.02	1.20 [1.14-1.26]*	1.10 [1.05-1.16]*
X65	23,830	113,735,250	0.23	0.21	0.20	0.02	0.03	0.02	1.15 [1.10-1.21]*	1.08 [1.03-1.13]*
X69	1,820	114,237,990	0.02	0.02	0.02	0.00	0.00	0.00	1.07 [0.91-1.26]	1.02 [0.86-1.20]
X70	1,380	114,253,350	0.01	0.01	0.01	0.00	0.00	0.00	0.97 [0.81-1.17]	0.96 [0.79-1.17]
X78	12,040	114,090,340	0.12	0.10	0.11	0.02	0.01	0.00	1.10 [1.03-1.17]*	1.02 [0.96-1.09]
X79	470	114,265,450	0.00	0.00	0.00	0.00	0.00	0.00	1.23 [0.90-1.67]	1.08 [0.78-1.50]
X83	1,030	114,259,270	0.01	0.01	0.01	0.00	0.00	0.00	1.03 [0.83-1.28]	0.95 [0.76-1.18]
X84	1,540	114,247,360	0.01	0.01	0.01	0.00	0.00	0.00	1.04 [0.87-1.24]	0.94 [0.78-1.13]
X99	5,250	114,156,100	0.05	0.05	0.05	0.00	0.00	0.00	1.06 [0.96-1.16]	1.04 [0.94-1.14]
Y00	2,670	114,197,940	0.02	0.02	0.02	0.00	0.00	0.00	1.08 [0.94-1.23]	1.03 [0.89-1.18]
Y04	23,200	113,652,980	0.21	0.20	0.20	0.01	0.01	0.01	1.07 [1.02-1.12]*	1.03 [0.98-1.08]
Y07	1,630	114,234,220	0.01	0.01	0.02	0.00	0.00	0.00	0.92 [0.77-1.09]	0.88 [0.74-1.05]
Y08	1,070	114,245,730	0.01	0.01	0.01	0.00	0.00	0.00	0.92 [0.74-1.15]	0.90 [0.71-1.12]
Y09	3,180	114,165,910	0.03	0.03	0.03	0.00	0.00	0.00	1.09 [0.96-1.23]	1.05 [0.92-1.19]
Y28	800	114,250,470	0.01	0.01	0.01	0.00	0.00	0.00	1.04 [0.81-1.33]	0.96 [0.75-1.24]
Y34	700	114,247,750	0.01	0.01	0.01	0.00	0.00	0.00	1.12 [0.86-1.44]	1.03 [0.79-1.34]
Y40	19,800	114,043,720	0.23	0.16	0.15	0.07	0.08	0.06	1.51 [1.44-1.58]*	1.39 [1.32-1.45]*
Y41	4,020	114,224,040	0.04	0.03	0.03	0.01	0.01	0.01	1.39 [1.26-1.55]*	1.26 [1.14-1.41]*
Y42	15,560	114,115,160	0.17	0.13	0.12	0.05	0.05	0.04	1.45 [1.38-1.53]*	1.29 [1.22-1.36]*
Y43	33,250	114,029,700	0.32	0.28	0.27	0.04	0.06	0.05	1.22 [1.17-1.26]*	1.18 [1.14-1.23]*
Y44	10,940	114,181,470	0.11	0.09	0.09	0.02	0.02	0.02	1.27 [1.18-1.35]*	1.20 [1.13-1.29]*
Y45	25,310	114,023,280	0.27	0.21	0.19	0.05	0.07	0.06	1.36 [1.31-1.42]*	1.26 [1.21-1.32]*
Y46	3,660	114,227,620	0.04	0.03	0.03	0.01	0.01	0.01	1.40 [1.25-1.56]*	1.28 [1.14-1.43]*
Y47	1,900	114,250,440	0.02	0.02	0.02	0.00	0.00	0.00	1.27 [1.09-1.49]*	1.17 [1.00-1.38]
Y48	2,200	114,242,900	0.02	0.02	0.02	0.00	0.01	0.00	1.32 [1.14-1.52]*	1.20 [1.04-1.39]
Y49	6,150	114,208,920	0.06	0.05	0.05	0.01	0.02	0.01	1.35 [1.24-1.47]*	1.29 [1.18-1.41]*
Y51	9,440	114,185,110	0.09	0.08	0.07	0.01	0.02	0.01	1.22 [1.13-1.31]*	1.18 [1.10-1.27]*
Y52	22,410	114,101,370	0.23	0.19	0.17	0.04	0.06	0.05	1.32 [1.26-1.39]*	1.26 [1.20-1.32]*
Y53	4,290	114,239,850	0.05	0.04	0.03	0.01	0.01	0.01	1.38 [1.25-1.53]*	1.25 [1.13-1.39]*
Y54	17,710	114,167,870	0.19	0.15	0.14	0.04	0.05	0.04	1.35 [1.28-1.42]*	1.27 [1.21-1.34]*
Y55	2,550	114,245,460	0.03	0.02	0.02	0.01	0.01	0.01	1.46 [1.28-1.66]*	1.28 [1.12-1.46]*
Y56	2,020	114,234,130	0.03	0.02	0.02	0.01	0.01	0.01	1.80 [1.57-2.06]*	1.62 [1.41-1.87]*
Y57	10,280	114,181,770	0.11	0.08	0.08	0.03	0.03	0.03	1.43 [1.34-1.53]*	1.31 [1.22-1.40]*
Y59	1,330	114,244,010	0.02	0.01	0.01	0.01	0.01	0.00	1.52 [1.28-1.81]*	1.40 [1.17-1.68]*
Y60	12,830	114,156,750	0.13	0.11	0.10	0.02	0.02	0.02	1.24 [1.17-1.32]*	1.18 [1.11-1.25]*
Y65	1,810	114,249,730	0.02	0.02	0.01	0.00	0.00	0.00	1.20 [1.02-1.41]	1.14 [0.97-1.34]
Y71	790	114,260,210	0.01	0.01	0.01	0.00	0.00	0.00	1.59 [1.27-1.99]*	1.51 [1.20-1.90]*
Y73	780	114,261,840	0.01	0.01	0.01	0.00	0.00	0.00	0.97 [0.75-1.25]	0.93 [0.71-1.21]
Y76	450	114,259,720	0.00	0.00	0.00	0.00	0.00	0.00	1.35 [1.00-1.82]	1.32 [0.97-1.79]
Y77	840	114,262,560	0.01	0.01	0.01	0.00	0.00	0.00	1.01 [0.78-1.30]	0.98 [0.76-1.27]
Y79	1,820	114,241,430	0.02	0.02	0.01	0.00	0.00	0.00	1.20 [1.02-1.41]	1.15 [0.97-1.35]
Y82	680	114,259,610	0.01	0.01	0.01	0.00	0.00	0.00	1.31 [1.01-1.68]	1.24 [0.96-1.61]
Y83	237,780	110,687,950	2.48	2.07	1.94	0.42	0.54	0.43	1.28 [1.26-1.30]*	1.21 [1.19-1.22]*
Y84	51,560	113,838,910	0.51	0.44	0.41	0.07	0.10	0.08	1.24 [1.21-1.28]*	1.19 [1.16-1.23]*
Y85	5,600	114,156,010	0.06	0.05	0.04	0.01	0.01	0.01	1.30 [1.19-1.42]*	1.23 [1.12-1.34]*
Y86	10,780	114,034,430	0.10	0.09	0.09	0.01	0.01	0.01	1.16 [1.09-1.24]*	1.11 [1.03-1.18]*
Y87	1,770	114,230,000	0.02	0.01	0.01	0.00	0.00	0.00	1.23 [1.05-1.44]*	1.15 [0.97-1.35]
Y88	1,940	114,239,820	0.02	0.02	0.02	0.00	0.00	0.00	1.17 [1.00-1.37]	1.07 [0.91-1.26]
Y89	640	114,257,270	0.01	0.01	0.01	0.00	0.00	0.00	1.26 [0.96-1.64]	1.21 [0.92-1.59]
Y90	1,140	114,256,100	0.01	0.01	0.01	0.00	0.00	0.00	1.13 [0.93-1.39]	1.10 [0.90-1.36]
Y91	2,290	114,222,180	0.02	0.02	0.02	0.00	0.00	0.00	1.15 [1.00-1.33]	1.12 [0.96-1.29]
Y95	117,620	113,985,260	1.17	1.00	0.94	0.18	0.23	0.19	1.25 [1.22-1.27]*	1.19 [1.16-1.21]*
any age - Z00-Z99										
Z00	5,350	114,100,500	0.05	0.05	0.04	0.00	0.01	0.01	1.19 [1.08-1.30]*	1.12 [1.02-1.23]
Z01	31,760	112,840,420	0.54	0.22	0.23	0.32	0.31	0.26	2.34 [2.27-2.42]*	1.96 [1.90-2.03]*
Z02	19,320	114,072,870	0.18	0.17	0.15	0.01	0.02	0.02	1.16 [1.10-1.22]*	1.11 [1.05-1.17]*
Z03	156,740	110,281,390	1.73	1.35	1.29	0.38	0.44	0.34	1.34 [1.32-1.36]*	1.25 [1.23-1.27]*
Z04	29,550	113,115,220	0.30	0.25	0.24	0.05	0.05	0.04	1.22 [1.17-1.27]*	1.16 [1.11-1.21]*
Z08	44,890	113,470,140	0.39	0.40	0.36	−0.01	0.03	0.02	1.08 [1.05-1.12]*	1.07 [1.03-1.10]*
Z09	87,880	112,560,570	0.90	0.75	0.70	0.14	0.20	0.14	1.28 [1.25-1.31]*	1.19 [1.17-1.22]*
Z11	272,790	113,709,140	2.77	2.31	2.27	0.46	0.50	0.36	1.22 [1.20-1.23]*	1.15 [1.13-1.16]*
Z12	63,210	113,641,280	0.57	0.55	0.50	0.02	0.08	0.06	1.16 [1.13-1.19]*	1.12 [1.09-1.15]*
Z13	175,920	111,670,540	1.85	1.51	1.44	0.34	0.42	0.33	1.29 [1.27-1.31]*	1.22 [1.19-1.24]*
Z20	9,680	114,195,190	0.10	0.08	0.08	0.02	0.02	0.02	1.25 [1.17-1.34]*	1.19 [1.10-1.28]*
Z22	81,500	113,387,010	0.90	0.68	0.64	0.22	0.25	0.22	1.40 [1.36-1.43]*	1.32 [1.29-1.35]*
Z24	1,760	114,153,520	0.02	0.02	0.02	0.00	0.00	0.00	1.07 [0.91-1.26]	1.07 [0.90-1.27]
Z25	840	114,248,140	0.02	0.01	0.01	0.01	0.01	0.01	2.82 [2.31-3.44]*	2.27 [1.84-2.80]*
Z27	4,890	114,095,560	0.08	0.03	0.03	0.04	0.04	0.04	2.36 [2.17-2.57]*	2.10 [1.92-2.29]*
Z29	45,890	113,064,430	0.47	0.39	0.37	0.08	0.09	0.07	1.25 [1.21-1.29]*	1.19 [1.15-1.23]*
Z30	53,370	111,703,510	0.50	0.47	0.43	0.02	0.07	0.05	1.16 [1.13-1.20]*	1.12 [1.09-1.15]*
Z31	910	114,157,150	0.01	0.01	0.01	0.00	0.00	0.00	1.02 [0.81-1.28]	0.97 [0.77-1.23]
Z32	3,850	114,094,760	0.04	0.03	0.03	0.00	0.01	0.01	1.23 [1.10-1.37]*	1.17 [1.05-1.31]*
Z33	35,520	113,139,030	0.36	0.30	0.30	0.06	0.07	0.05	1.23 [1.18-1.27]*	1.14 [1.10-1.19]*
Z34	97,020	111,476,280	0.93	0.85	0.86	0.08	0.08	0.06	1.09 [1.07-1.11]*	1.06 [1.04-1.09]*

Z35	86,100	112,684,920	0.80	0.75	0.75	0.05	0.05	0.03	1.07 [1.04-1.09]*	1.04 [1.02-1.07]*
Z36	18,140	113,067,420	0.17	0.16	0.15	0.01	0.02	0.02	1.12 [1.07-1.18]*	1.10 [1.04-1.16]*
Z37	337,140	99,896,670	3.96	3.23	3.69	0.73	0.27	0.23	1.07 [1.06-1.09]*	1.06 [1.05-1.08]*
Z38	1,010	88,256,730	0.01	0.01	0.01	0.00	0.00	0.00	1.09 [0.88-1.35]	1.09 [0.88-1.36]
Z39	29,460	113,210,160	0.27	0.26	0.25	0.01	0.02	0.02	1.08 [1.04-1.12]*	1.07 [1.03-1.12]*
Z40	5,720	114,190,700	0.05	0.05	0.05	0.00	0.01	0.00	1.12 [1.02-1.22]	1.10 [1.00-1.20]
Z41	6,560	114,032,850	0.07	0.05	0.05	0.02	0.02	0.02	1.34 [1.24-1.45]*	1.29 [1.19-1.40]*
Z42	15,180	113,966,600	0.14	0.13	0.12	0.01	0.02	0.02	1.18 [1.12-1.25]*	1.16 [1.09-1.22]*
Z43	22,450	113,877,910	0.21	0.19	0.18	0.01	0.03	0.02	1.15 [1.10-1.20]*	1.11 [1.06-1.16]*
Z44	490	114,254,290	0.00	0.00	0.00	0.00	0.00	0.00	1.32 [0.98-1.79]	1.28 [0.93-1.74]
Z45	46,900	113,671,940	0.45	0.40	0.38	0.05	0.07	0.06	1.20 [1.16-1.24]*	1.14 [1.11-1.18]*
Z46	57,850	113,377,840	0.53	0.51	0.46	0.02	0.07	0.05	1.14 [1.11-1.17]*	1.11 [1.08-1.14]*
Z47	46,750	113,063,620	0.46	0.40	0.41	0.06	0.05	0.03	1.12 [1.08-1.15]*	1.07 [1.04-1.11]*
Z48	17,260	113,816,190	0.17	0.15	0.14	0.02	0.03	0.02	1.20 [1.14-1.26]*	1.15 [1.10-1.22]*
Z49	8,820	114,166,210	0.09	0.07	0.07	0.02	0.03	0.03	1.44 [1.34-1.54]*	1.40 [1.31-1.50]*
Z50	331,390	112,224,960	3.26	2.88	2.75	0.38	0.51	0.41	1.19 [1.17-1.20]*	1.14 [1.13-1.16]*
Z51	394,130	111,027,540	3.91	3.46	3.22	0.45	0.69	0.56	1.21 [1.20-1.23]*	1.17 [1.16-1.18]*
Z52	2,200	114,227,270	0.02	0.02	0.02	0.00	0.00	0.00	1.00 [0.86-1.17]	1.01 [0.86-1.18]
Z53	342,090	108,273,730	3.73	3.02	2.84	0.71	0.89	0.70	1.31 [1.30-1.33]*	1.23 [1.22-1.25]*
Z54	2,890	114,195,540	0.03	0.02	0.02	0.01	0.01	0.01	1.40 [1.24-1.58]*	1.36 [1.20-1.54]*
Z55	1,590	114,250,160	0.02	0.01	0.01	0.01	0.00	0.00	1.33 [1.13-1.56]*	1.25 [1.06-1.48]*
Z56	4,120	114,215,600	0.04	0.04	0.03	0.00	0.01	0.00	1.17 [1.06-1.31]*	1.09 [0.98-1.22]
Z57	11,960	114,199,590	0.12	0.10	0.09	0.02	0.03	0.02	1.27 [1.20-1.35]*	1.21 [1.14-1.29]*
Z58	9,050	114,194,770	0.10	0.07	0.07	0.02	0.02	0.02	1.32 [1.23-1.41]*	1.22 [1.14-1.31]*
Z59	9,280	114,159,310	0.08	0.08	0.08	0.00	0.01	0.00	1.09 [1.01-1.17]	1.05 [0.97-1.13]
Z60	106,020	113,087,560	1.01	0.92	0.85	0.08	0.15	0.12	1.18 [1.15-1.21]*	1.14 [1.11-1.16]*
Z61	4,780	114,202,950	0.05	0.04	0.04	0.01	0.00	0.00	1.06 [0.96-1.17]	1.00 [0.90-1.11]
Z62	1,090	114,246,470	0.01	0.01	0.01	0.00	0.00	0.00	1.01 [0.82-1.23]	0.93 [0.76-1.15]
Z63	35,170	113,816,780	0.34	0.30	0.31	0.04	0.04	0.02	1.12 [1.08-1.16]*	1.05 [1.01-1.09]
Z64	900	114,229,890	0.01	0.01	0.01	0.00	0.00	0.00	0.99 [0.78-1.25]	0.94 [0.74-1.20]
Z65	2,850	114,228,600	0.03	0.02	0.03	0.00	0.00	0.00	1.09 [0.96-1.24]	1.04 [0.91-1.18]
Z71	35,110	113,762,180	0.45	0.28	0.27	0.17	0.18	0.15	1.65 [1.59-1.70]*	1.49 [1.44-1.55]*
Z72	229,660	108,372,280	2.26	2.09	1.88	0.17	0.37	0.32	1.20 [1.18-1.22]*	1.17 [1.15-1.18]*
Z73	54,930	114,010,380	0.53	0.47	0.45	0.06	0.08	0.06	1.18 [1.14-1.22]*	1.13 [1.10-1.17]*
Z74	42,210	114,016,830	0.40	0.36	0.34	0.03	0.06	0.05	1.18 [1.14-1.22]*	1.16 [1.12-1.20]*
Z75	83,140	113,864,080	0.78	0.72	0.69	0.06	0.09	0.08	1.13 [1.10-1.16]*	1.11 [1.08-1.14]*
Z76	6,650	113,221,920	0.06	0.06	0.05	0.01	0.01	0.01	1.20 [1.10-1.30]*	1.18 [1.08-1.28]*
Z80	90,850	113,023,020	0.85	0.79	0.73	0.06	0.12	0.09	1.17 [1.14-1.20]*	1.11 [1.09-1.14]*
Z81	14,740	114,014,230	0.16	0.12	0.13	0.03	0.02	0.01	1.18 [1.11-1.24]*	1.11 [1.05-1.17]*
Z82	194,870	110,845,770	2.08	1.68	1.60	0.40	0.48	0.38	1.30 [1.28-1.32]*	1.23 [1.21-1.24]*
Z83	75,220	112,474,520	0.79	0.64	0.64	0.16	0.15	0.12	1.23 [1.21-1.27]*	1.17 [1.14-1.20]*
Z84	10,920	113,982,680	0.15	0.08	0.09	0.07	0.07	0.06	1.76 [1.66-1.87]*	1.69 [1.59-1.80]*
Z85	321,340	110,790,740	3.10	2.85	2.66	0.25	0.44	0.36	1.17 [1.15-1.18]*	1.13 [1.12-1.15]*
Z86	1,016,710	103,940,520	11.18	9.44	8.88	1.74	2.30	1.81	1.26 [1.25-1.27]*	1.19 [1.19-1.20]*
Z87	537,640	107,644,600	5.91	4.77	4.59	1.14	1.33	0.98	1.29 [1.28-1.30]*	1.20 [1.19-1.21]*
Z88	527,060	107,275,920	6.33	4.57	4.32	1.75	2.01	1.56	1.47 [1.45-1.48]*	1.33 [1.31-1.34]*
Z89	15,080	114,138,110	0.15	0.13	0.12	0.02	0.03	0.03	1.28 [1.21-1.35]*	1.23 [1.16-1.30]*
Z90	278,340	111,791,120	2.77	2.42	2.26	0.34	0.50	0.36	1.22 [1.21-1.24]*	1.15 [1.13-1.16]*
Z91	228,630	111,758,600	3.12	1.79	1.78	1.33	1.34	1.09	1.76 [1.73-1.78]*	1.54 [1.52-1.56]*
Z92	656,400	108,686,690	6.78	5.86	5.48	0.92	1.30	1.01	1.24 [1.23-1.25]*	1.17 [1.16-1.18]*
Z93	65,200	113,637,850	0.62	0.56	0.52	0.06	0.10	0.08	1.19 [1.16-1.23]*	1.15 [1.12-1.18]*
Z94	13,060	114,062,060	0.14	0.11	0.10	0.03	0.04	0.03	1.36 [1.28-1.44]*	1.31 [1.23-1.38]*
Z95	213,710	111,781,730	2.12	1.86	1.73	0.26	0.39	0.31	1.22 [1.21-1.24]*	1.17 [1.15-1.19]*
Z96	415,170	110,481,860	4.18	3.66	3.43	0.52	0.74	0.59	1.22 [1.20-1.23]*	1.17 [1.15-1.18]*
Z97	41,380	113,915,180	0.42	0.35	0.33	0.07	0.09	0.07	1.26 [1.22-1.31]*	1.18 [1.14-1.23]*
Z98	55,760	113,621,780	0.57	0.47	0.44	0.09	0.13	0.09	1.29 [1.26-1.33]*	1.18 [1.15-1.22]*
Z99	68,690	113,775,710	0.74	0.57	0.53	0.17	0.21	0.16	1.39 [1.36-1.43]*	1.28 [1.25-1.31]*
18+ - A00-B99										
A02	1,240	80,137,040	0.02	0.01	0.02	0.00	0.00	0.00	1.17 [0.97-1.42]	1.04 [0.86-1.27]
A04	49,160	79,601,460	0.76	0.59	0.59	0.17	0.17	0.11	1.28 [1.24-1.32]*	1.18 [1.14-1.22]*
A05	460	80,166,580	0.01	0.01	0.01	0.00	0.00	0.00	1.31 [0.96-1.80]	1.23 [0.89-1.70]
A07	660	80,159,900	0.01	0.01	0.01	0.00	0.00	0.00	1.19 [0.91-1.56]	1.04 [0.79-1.38]
A08	25,100	79,670,080	0.40	0.30	0.30	0.10	0.10	0.06	1.31 [1.26-1.37]*	1.17 [1.12-1.22]*
A09	196,020	78,925,500	3.18	2.33	2.41	0.85	0.78	0.46	1.32 [1.30-1.34]*	1.17 [1.15-1.19]*
A15	1,240	80,147,010	0.02	0.02	0.02	0.00	0.00	0.00	1.13 [0.93-1.37]	1.08 [0.88-1.32]
A16	2,320	80,121,820	0.04	0.03	0.03	0.01	0.01	0.01	1.41 [1.23-1.61]*	1.30 [1.14-1.50]*
A18	1,200	80,137,380	0.02	0.01	0.01	0.00	0.00	0.00	1.35 [1.11-1.63]*	1.31 [1.08-1.60]*
A31	910	80,164,630	0.02	0.01	0.01	0.01	0.01	0.00	1.63 [1.32-2.01]*	1.39 [1.12-1.73]*
A39	580	80,128,400	0.01	0.01	0.01	0.00	0.00	0.00	1.10 [0.82-1.46]	1.06 [0.79-1.42]
A40	9,550	80,104,470	0.15	0.11	0.11	0.04	0.04	0.03	1.35 [1.26-1.45]*	1.27 [1.18-1.36]*
A41	189,650	79,399,180	2.93	2.27	2.33	0.65	0.60	0.43	1.26 [1.24-1.28]*	1.17 [1.15-1.19]*
A46	660	80,154,330	0.01	0.01	0.01	0.01	0.01	0.01	2.10 [1.67-2.65]*	1.95 [1.54-2.48]*
A48	840	80,160,380	0.01	0.01	0.01	0.00	0.00	0.00	1.26 [1.00-1.59]	1.20 [0.95-1.52]
A49	25,660	79,924,900	0.41	0.30	0.30	0.11	0.11	0.08	1.36 [1.31-1.42]*	1.25 [1.20-1.31]*
A63	880	80,081,650	0.01	0.01	0.01	0.00	0.00	0.00	1.47 [1.19-1.81]*	1.31 [1.06-1.63]
A69	620	80,164,210	0.01	0.01	0.01	0.00	0.00	0.00	1.23 [0.94-1.61]	1.06 [0.80-1.41]
A86	1,230	80,157,210	0.02	0.01	0.02	0.00	0.00	0.00	1.09 [0.89-1.33]	1.01 [0.82-1.23]
A87	3,120	80,087,040	0.05	0.04	0.04	0.02	0.01	0.01	1.40 [1.25-1.58]*	1.22 [1.08-1.38]*
B00	5,800	80,050,970	0.13	0.06	0.06	0.07	0.06	0.06	2.06 [1.91-2.24]*	1.80 [1.66-1.96]*
B01	1,110	80,096,880	0.02	0.01	0.01	0.01	0.01	0.00	1.39 [1.14-1.68]*	1.27 [1.04-1.55]
B02	13,430	80,050,650	0.22	0.16	0.16	0.07	0.06	0.05	1.41 [1.33-1.49]*	1.29 [1.21-1.37]*
B07	5,300	80,021,600	0.09	0.06	0.06	0.03	0.02	0.02	1.40 [1.28-1.53]*	1.25 [1.14-1.37]*
B08	490	80,145,560	0.01	0.01	0.01	0.00	0.00	0.00	1.95 [1.48-2.56]*	1.70 [1.27-2.27]*
B15	650	80,157,960	0.01	0.01	0.01	0.00	0.00	0.00	1.24 [0.95-1.60]	1.17 [0.89-1.53]
B16	1,350	80,135,350	0.02	0.02	0.02	0.01	0.01	0.01	1.39 [1.17-1.66]*	1.37 [1.14-1.64]*
B17	2,030	80,126,740	0.03	0.02	0.02	0.01	0.01	0.01	1.41 [1.22-1.62]*	1.26 [1.08-1.46]*
B18	8,690	80,015,250	0.13	0.10	0.10	0.02	0.02	0.01	1.23 [1.14-1.32]*	1.13 [1.05-1.22]*
B25	1,540	80,154,650	0.03	0.02	0.02	0.01	0.01	0.01	1.40 [1.19-1.65]*	1.27 [1.07-1.50]*
B27	3,910	80,064,570	0.06	0.05	0.05	0.01	0.01	0.00	1.11 [1.00-1.24]	1.02 [0.91-1.14]
B34	29,660	79,096,870	0.48	0.35	0.36	0.12	0.12	0.06	1.33 [1.28-1.38]*	1.15 [1.11-1.20]*
B35	5,530	80,113,490	0.11	0.06	0.06	0.05	0.05	0.04	1.77 [1.62-1.93]*	1.56 [1.43-1.71]*
B36	2,070	80,158,400	0.04	0.02	0.02	0.02	0.02	0.02	1.86 [1.62-2.14]*	1.66 [1.44-1.92]*
B37	77,500	79,516,330	1.27	0.91	0.93	0.36	0.34	0.21	1.37 [1.34-1.40]*	1.20 [1.17-1.23]*
B44	2,860	80,146,770	0.06	0.03	0.03	0.03	0.03	0.02	1.84 [1.64-2.06]*	1.47 [1.30-1.66]*
B49	1,700	80,160,430	0.03	0.02	0.02	0.01	0.01	0.01	1.53 [1.30-1.78]*	1.40 [1.19-1.65]*
B50	800	80,144,800	0.01	0.01	0.01	0.00	0.00	0.00	0.98 [0.76-1.26]	1.05 [0.81-1.37]
B59	910	80,167,300	0.02	0.01	0.01	0.01	0.01	0.00	1.55 [1.26-1.91]*	1.44 [1.16-1.78]*
B80	1,230	80,140,030	0.02	0.01	0.01	0.00	0.00	0.00	1.24 [1.03-1.50]	1.13 [0.93-1.37]
B85	560	80,161,670	0.01	0.01	0.01	0.00	0.00	0.00	1.19 [0.89-1.59]	1.09 [0.81-1.47]
B86	1,480	80,146,040	0.04	0.01	0.01	0.02	0.02	0.02	2.59 [2.22-3.04]*	2.43 [2.07-2.86]*
B90	730	80,164,240	0.01	0.01	0.01	0.00	0.00	0.00	1.43 [1.12-1.82]*	1.29 [1.00-1.65]
B91	1,190	80,154,460	0.02	0.01	0.01	0.00	0.00	0.00	1.29 [1.06-1.57]	1.27 [1.04-1.56]
B94	1,580	80,153,800	0.02	0.02	0.02	0.01	0.01	0.00	1.31 [1.11-1.55]*	1.14 [0.96-1.36]
B95	104,630	78,835,720	1.83	1.22	1.22	0.62	0.62	0.51	1.50 [1.47-1.54]*	1.39 [1.36-1.42]*
B96	220,280	77,863,530	3.44	2.70	2.78	0.75	0.66	0.43	1.24 [1.22-1.26]*	1.14 [1.13-1.16]*
B97	42,520	79,817,470	0.68	0.50	0.53	0.18	0.15	0.09	1.29 [1.25-1.34]*	1.15 [1.11-1.19]*
B98	22,620	80,013,200	0.34	0.27	0.28	0.07	0.06	0.04	1.22 [1.16-1.27]*	1.13 [1.08-1.18]*
B99	6,210	80,145,070	0.09	0.07	0.07	0.02	0.02	0.01	1.26 [1.16-1.38]*	1.17 [1.07-1.28]*

18+ - C00-D49

C00	790	80,162,340	0.01	0.01	0.01	0.00	0.00	0.00	1.08 [0.84-1.40]	1.05 [0.81-1.36]
C01	1,610	80,161,150	0.02	0.02	0.02	0.00	0.00	0.00	1.09 [0.92-1.30]	1.05 [0.88-1.26]
C02	2,540	80,146,300	0.04	0.03	0.03	0.01	0.01	0.01	1.31 [1.15-1.50]*	1.24 [1.08-1.42]*
C03	590	80,168,690	0.01	0.01	0.01	0.00	0.00	0.00	1.38 [1.06-1.81]	1.38 [1.05-1.82]
C04	780	80,163,560	0.01	0.01	0.01	0.00	0.00	0.00	1.15 [0.90-1.48]	1.08 [0.84-1.39]
C05	810	80,164,400	0.01	0.01	0.01	0.00	0.00	0.00	1.32 [1.05-1.67]	1.29 [1.01-1.63]
C06	1,160	80,162,860	0.02	0.01	0.01	0.00	0.00	0.00	1.24 [1.02-1.52]	1.18 [0.96-1.44]
C07	1,160	80,159,990	0.01	0.01	0.01	0.00	0.00	0.00	1.05 [0.85-1.29]	1.00 [0.80-1.24]
C09	2,070	80,152,020	0.03	0.03	0.02	0.00	0.00	0.00	1.12 [0.97-1.31]	1.06 [0.91-1.24]
C10	1,060	80,166,870	0.01	0.01	0.01	0.00	0.00	0.00	1.18 [0.95-1.45]	1.10 [0.88-1.36]
C11	530	80,167,510	0.01	0.01	0.01	0.00	0.00	0.00	1.20 [0.90-1.60]	1.17 [0.87-1.56]
C12	570	80,169,190	0.01	0.01	0.01	0.00	0.00	0.00	1.29 [0.98-1.70]	1.24 [0.93-1.65]
C13	630	80,170,290	0.01	0.01	0.01	0.00	0.00	0.00	1.25 [0.95-1.63]	1.24 [0.94-1.63]
C14	1,180	80,166,460	0.02	0.01	0.01	0.00	0.00	0.00	1.12 [0.91-1.36]	1.06 [0.87-1.31]
C15	16,370	80,115,170	0.23	0.20	0.20	0.03	0.03	0.02	1.15 [1.09-1.22]*	1.12 [1.06-1.18]*
C16	13,260	80,117,230	0.18	0.16	0.17	0.01	0.01	0.01	1.07 [1.00-1.13]	1.04 [0.98-1.11]
C17	2,700	80,157,710	0.04	0.03	0.03	0.00	0.00	0.00	1.15 [1.01-1.32]	1.09 [0.95-1.25]
C18	45,800	79,778,300	0.58	0.57	0.58	0.01	0.01	0.00	1.01 [0.98-1.05]	1.00 [0.97-1.03]
C19	9,430	80,099,230	0.11	0.12	0.12	0.00	0.00	0.00	0.96 [0.89-1.04]	0.96 [0.89-1.04]
C20	19,540	79,971,740	0.24	0.25	0.25	−0.01	−0.01	−0.01	0.97 [0.92-1.02]	0.98 [0.93-1.03]
C21	2,390	80,148,280	0.03	0.03	0.03	0.00	0.00	0.00	1.08 [0.94-1.25]	1.05 [0.91-1.21]
C22	9,020	80,151,390	0.14	0.11	0.11	0.03	0.03	0.02	1.25 [1.16-1.34]*	1.18 [1.10-1.27]*
C23	1,320	80,170,360	0.02	0.02	0.02	0.00	0.00	0.00	1.06 [0.87-1.29]	1.04 [0.85-1.26]
C24	2,450	80,164,240	0.03	0.03	0.03	0.00	0.00	0.00	1.07 [0.93-1.24]	1.04 [0.90-1.20]
C25	15,100	80,146,000	0.21	0.18	0.19	0.03	0.03	0.02	1.14 [1.08-1.21]*	1.10 [1.04-1.17]*
C26	3,400	80,158,470	0.05	0.04	0.04	0.01	0.00	0.00	1.10 [0.97-1.24]	1.05 [0.93-1.19]
C30	540	80,167,150	0.01	0.01	0.01	0.00	0.00	0.00	1.12 [0.84-1.51]	1.09 [0.81-1.46]
C32	3,480	80,133,450	0.05	0.04	0.04	0.01	0.01	0.01	1.17 [1.04-1.32]*	1.14 [1.01-1.28]
C34	65,420	79,991,990	0.92	0.80	0.80	0.13	0.12	0.07	1.15 [1.12-1.18]*	1.09 [1.06-1.12]*
C38	910	80,169,620	0.01	0.01	0.01	0.00	0.00	0.00	0.97 [0.76-1.22]	0.95 [0.74-1.20]
C41	1,710	80,158,210	0.02	0.02	0.02	0.00	0.00	0.00	1.23 [1.04-1.45]	1.19 [1.00-1.40]
C43	18,260	79,957,940	0.22	0.23	0.23	−0.01	−0.01	−0.01	0.97 [0.92-1.02]	0.95 [0.90-1.01]
C44	128,910	78,616,050	1.80	1.61	1.62	0.19	0.17	0.11	1.11 [1.08-1.13]*	1.07 [1.04-1.09]*
C45	4,100	80,164,600	0.05	0.05	0.05	0.00	0.00	0.00	1.00 [0.89-1.12]	1.01 [0.90-1.13]
C48	2,250	80,163,070	0.03	0.03	0.03	0.00	0.00	0.00	1.07 [0.93-1.24]	1.07 [0.92-1.24]
C49	3,900	80,134,780	0.05	0.05	0.05	0.01	0.01	0.01	1.13 [1.01-1.26]	1.10 [0.99-1.24]
C50	77,330	78,953,740	1.02	0.97	0.99	0.05	0.04	0.03	1.04 [1.01-1.06]*	1.03 [1.00-1.05]
C51	2,070	80,149,550	0.03	0.02	0.02	0.01	0.01	0.01	1.24 [1.07-1.44]*	1.22 [1.05-1.42]*
C52	660	80,168,560	0.01	0.01	0.01	0.00	0.00	0.00	0.90 [0.68-1.19]	0.90 [0.68-1.19]
C53	4,300	80,108,340	0.05	0.05	0.06	0.00	0.00	0.00	0.96 [0.86-1.07]	0.97 [0.87-1.08]
C54	12,830	80,010,230	0.17	0.16	0.16	0.01	0.01	0.01	1.04 [0.98-1.11]	1.06 [1.00-1.13]
C55	1,790	80,158,940	0.02	0.02	0.02	0.00	0.00	0.00	1.02 [0.86-1.20]	1.01 [0.85-1.19]
C56	11,460	80,073,480	0.15	0.14	0.14	0.00	0.00	0.00	1.01 [0.95-1.08]	1.02 [0.95-1.09]
C57	1,270	80,166,510	0.02	0.02	0.02	0.00	0.00	0.00	1.19 [0.99-1.44]	1.22 [1.00-1.47]
C60	930	80,160,630	0.01	0.01	0.01	0.00	0.00	0.00	1.30 [1.04-1.62]	1.29 [1.03-1.62]
C61	72,820	79,495,220	0.97	0.90	0.92	0.07	0.05	0.03	1.06 [1.03-1.09]*	1.03 [1.00-1.06]
C62	1,940	80,125,010	0.02	0.02	0.02	0.00	0.00	0.00	1.02 [0.87-1.20]	0.99 [0.84-1.17]
C64	14,940	80,054,640	0.20	0.18	0.18	0.02	0.02	0.01	1.10 [1.04-1.17]*	1.05 [0.99-1.11]
C65	1,350	80,162,050	0.02	0.02	0.02	0.00	0.00	0.00	1.22 [1.01-1.47]	1.18 [0.98-1.43]
C66	1,880	80,159,820	0.03	0.02	0.02	0.00	0.00	0.00	1.09 [0.92-1.28]	1.05 [0.89-1.24]
C67	33,640	79,771,300	0.45	0.41	0.42	0.04	0.04	0.03	1.09 [1.05-1.13]*	1.06 [1.02-1.10]*
C68	750	80,167,240	0.01	0.01	0.01	0.00	0.00	0.00	1.05 [0.81-1.37]	1.04 [0.80-1.36]
C69	1,350	80,152,360	0.02	0.02	0.02	0.00	0.00	0.00	0.97 [0.80-1.17]	0.95 [0.78-1.16]
C71	7,480	80,126,100	0.10	0.09	0.09	0.01	0.01	0.01	1.15 [1.06-1.24]*	1.12 [1.03-1.21]*
C73	4,260	80,108,450	0.06	0.05	0.05	0.00	0.00	0.00	1.06 [0.95-1.18]	1.02 [0.92-1.14]
C74	510	80,167,620	0.01	0.01	0.01	0.00	0.00	0.00	1.13 [0.83-1.52]	1.06 [0.77-1.44]
C75	680	80,165,830	0.01	0.01	0.01	0.00	0.00	0.00	1.00 [0.76-1.32]	0.96 [0.73-1.27]
C76	2,220	80,156,940	0.03	0.03	0.03	0.00	0.00	0.00	1.11 [0.95-1.28]	1.09 [0.94-1.27]
C77	75,680	79,632,510	1.00	0.94	0.95	0.06	0.05	0.04	1.05 [1.03-1.08]*	1.04 [1.01-1.07]*
C78	111,800	79,951,960	1.47	1.38	1.40	0.09	0.08	0.05	1.05 [1.03-1.08]*	1.04 [1.01-1.06]*
C79	93,930	79,978,990	1.25	1.16	1.17	0.10	0.09	0.06	1.07 [1.05-1.10]*	1.05 [1.03-1.08]*
C80	33,240	80,089,150	0.45	0.41	0.41	0.04	0.04	0.02	1.09 [1.05-1.13]*	1.06 [1.02-1.10]*
C81	3,220	80,113,110	0.06	0.04	0.04	0.03	0.03	0.02	1.72 [1.54-1.91]*	1.65 [1.48-1.85]*
C82	4,000	80,123,180	0.06	0.05	0.05	0.01	0.01	0.00	1.10 [0.99-1.23]	1.04 [0.93-1.16]
C83	11,180	80,086,150	0.17	0.13	0.13	0.03	0.03	0.03	1.26 [1.18-1.34]*	1.22 [1.14-1.30]*
C84	1,490	80,159,180	0.04	0.01	0.01	0.02	0.02	0.02	2.71 [2.33-3.15]*	2.64 [2.26-3.08]*
C85	14,210	80,014,610	0.22	0.17	0.17	0.05	0.05	0.04	1.31 [1.24-1.39]*	1.26 [1.19-1.33]*
C88	1,900	80,159,950	0.03	0.02	0.02	0.01	0.01	0.00	1.23 [1.06-1.44]*	1.21 [1.03-1.42]
C90	9,990	80,107,150	0.14	0.12	0.12	0.02	0.02	0.02	1.15 [1.07-1.23]*	1.13 [1.05-1.21]*
C91	9,460	80,077,760	0.13	0.12	0.11	0.01	0.01	0.01	1.12 [1.04-1.21]*	1.08 [1.01-1.17]
C92	7,770	80,126,140	0.12	0.09	0.09	0.03	0.03	0.02	1.29 [1.20-1.39]*	1.26 [1.16-1.36]*
C93	1,050	80,170,360	0.02	0.01	0.01	0.00	0.00	0.00	1.18 [0.95-1.47]	1.14 [0.91-1.42]
C94	460	80,171,030	0.01	0.01	0.01	0.00	0.00	0.00	1.31 [0.96-1.80]	1.27 [0.92-1.74]
C95	1,540	80,162,350	0.02	0.02	0.02	0.01	0.01	0.01	1.34 [1.13-1.60]*	1.27 [1.06-1.52]*
C97	8,460	80,147,380	0.11	0.10	0.10	0.01	0.01	0.00	1.08 [1.00-1.17]	1.04 [0.96-1.13]
D00	960	80,163,610	0.01	0.01	0.01	0.00	0.00	0.00	1.05 [0.84-1.32]	0.99 [0.79-1.24]
D01	950	80,161,630	0.01	0.01	0.01	0.00	0.00	0.00	1.11 [0.89-1.40]	1.02 [0.81-1.29]
D02	970	80,163,240	0.01	0.01	0.01	0.00	0.00	0.00	1.20 [0.97-1.49]	1.13 [0.91-1.41]
D03	6,900	80,086,960	0.09	0.09	0.08	0.01	0.01	0.00	1.08 [0.99-1.18]	1.04 [0.95-1.13]
D04	14,350	80,031,760	0.22	0.17	0.17	0.05	0.05	0.03	1.26 [1.19-1.34]*	1.18 [1.11-1.25]*
D05	12,600	79,943,710	0.16	0.16	0.16	0.00	0.00	0.00	1.01 [0.95-1.07]	1.00 [0.93-1.06]
D06	10,880	79,802,200	0.15	0.13	0.14	0.01	0.01	0.00	1.06 [0.99-1.13]	1.00 [0.93-1.07]
D07	3,830	80,108,230	0.06	0.05	0.04	0.01	0.01	0.01	1.28 [1.15-1.42]*	1.20 [1.07-1.34]*
D09	3,570	80,136,310	0.05	0.04	0.04	0.01	0.01	0.00	1.13 [1.00-1.27]	1.08 [0.96-1.21]
D10	3,550	80,087,610	0.06	0.04	0.04	0.01	0.01	0.01	1.33 [1.20-1.49]*	1.19 [1.06-1.33]*
D11	4,320	80,089,110	0.06	0.05	0.05	0.00	0.00	0.00	1.04 [0.94-1.16]	1.00 [0.90-1.12]
D12	123,630	78,593,830	1.84	1.52	1.52	0.32	0.32	0.18	1.21 [1.19-1.24]*	1.11 [1.09-1.13]*
D13	8,450	79,961,050	0.12	0.10	0.10	0.02	0.02	0.01	1.21 [1.12-1.30]*	1.09 [1.01-1.17]
D14	2,610	80,117,590	0.04	0.03	0.03	0.01	0.01	0.01	1.30 [1.14-1.48]*	1.16 [1.02-1.33]
D15	1,000	80,161,660	0.01	0.01	0.01	0.00	0.00	0.00	1.15 [0.92-1.43]	1.11 [0.89-1.38]
D16	2,990	80,084,290	0.05	0.04	0.04	0.01	0.01	0.01	1.27 [1.13-1.43]*	1.18 [1.04-1.33]
D17	38,020	79,151,530	0.54	0.47	0.46	0.07	0.07	0.04	1.16 [1.12-1.20]*	1.08 [1.05-1.12]*
D18	15,290	79,904,850	0.22	0.19	0.19	0.03	0.03	0.01	1.16 [1.10-1.23]*	1.07 [1.01-1.13]
D21	3,990	80,086,100	0.06	0.05	0.05	0.01	0.02	0.01	1.34 [1.21-1.49]*	1.24 [1.11-1.38]*
D22	33,250	79,176,850	0.47	0.41	0.41	0.06	0.06	0.04	1.15 [1.11-1.20]*	1.09 [1.05-1.13]*
D23	21,150	79,455,400	0.32	0.26	0.25	0.06	0.06	0.05	1.26 [1.20-1.32]*	1.17 [1.12-1.23]*
D24	13,030	79,826,070	0.17	0.16	0.17	0.01	0.01	0.00	1.04 [0.98-1.11]	0.99 [0.93-1.06]
D25	64,730	78,603,730	0.90	0.81	0.83	0.09	0.07	0.04	1.08 [1.05-1.11]*	1.05 [1.02-1.08]*
D26	2,190	80,112,140	0.03	0.03	0.03	0.00	0.00	0.00	1.10 [0.95-1.27]	1.08 [0.93-1.25]
D27	15,010	79,883,220	0.20	0.19	0.19	0.01	0.01	0.00	1.06 [1.00-1.12]	1.01 [0.96-1.07]
D28	1,560	80,133,260	0.02	0.02	0.02	0.00	0.00	0.00	1.26 [1.06-1.49]*	1.19 [1.00-1.41]
D29	1,170	80,151,300	0.02	0.01	0.01	0.00	0.00	0.00	1.21 [0.99-1.49]	1.16 [0.95-1.43]
D30	2,350	80,142,180	0.03	0.03	0.03	0.00	0.00	0.00	1.08 [0.94-1.25]	1.02 [0.88-1.18]
D31	2,120	80,140,740	0.03	0.03	0.02	0.01	0.01	0.01	1.30 [1.12-1.50]*	1.23 [1.06-1.43]*
D32	8,640	80,093,100	0.12	0.11	0.11	0.01	0.01	0.00	1.09 [1.01-1.18]	1.03 [0.95-1.11]
D33	3,180	80,126,400	0.04	0.04	0.04	0.00	0.00	0.00	1.08 [0.96-1.22]	0.98 [0.87-1.11]

D34	2,270	80,128,250	0.03	0.03	0.03	0.00	0.00	0.00	1.04 [0.90-1.21]	0.98 [0.84-1.14]
D35	12,550	80,020,830	0.18	0.15	0.16	0.02	0.02	0.01	1.13 [1.06-1.20]*	1.05 [0.99-1.12]
D36	5,530	80,044,060	0.08	0.07	0.07	0.01	0.01	0.01	1.23 [1.12-1.34]*	1.13 [1.03-1.24]
D37	16,780	80,001,960	0.23	0.21	0.21	0.02	0.02	0.01	1.10 [1.05-1.16]*	1.06 [1.00-1.12]
D38	3,970	80,143,440	0.06	0.05	0.05	0.01	0.01	0.01	1.21 [1.09-1.35]*	1.15 [1.03-1.28]
D39	2,360	80,130,930	0.03	0.03	0.03	0.00	0.00	0.00	1.02 [0.88-1.18]	1.02 [0.88-1.18]
D40	790	80,157,400	0.01	0.01	0.01	0.00	0.00	0.00	1.13 [0.88-1.45]	1.11 [0.86-1.43]
D41	11,780	80,037,680	0.16	0.14	0.15	0.02	0.01	0.01	1.09 [1.02-1.17]*	1.06 [0.99-1.13]
D43	3,580	80,141,700	0.05	0.04	0.04	0.01	0.01	0.00	1.11 [0.99-1.25]	1.07 [0.95-1.21]
D44	2,300	80,135,570	0.03	0.03	0.03	0.00	0.00	0.00	1.16 [1.01-1.34]	1.07 [0.92-1.24]
D45	4,400	80,102,540	0.06	0.05	0.05	0.01	0.01	0.00	1.13 [1.01-1.25]	1.06 [0.95-1.18]
D46	10,110	80,122,030	0.16	0.12	0.12	0.04	0.04	0.04	1.34 [1.26-1.44]*	1.28 [1.19-1.37]*
D47	16,380	80,076,130	0.26	0.19	0.20	0.06	0.05	0.04	1.27 [1.20-1.34]*	1.18 [1.11-1.24]*
D48	8,020	80,024,330	0.11	0.10	0.10	0.01	0.01	0.01	1.12 [1.03-1.21]*	1.06 [0.98-1.15]
18+ - D50-D89										
D50	223,670	78,080,690	3.67	2.69	2.78	0.98	0.89	0.62	1.32 [1.30-1.34]*	1.20 [1.18-1.22]*
D51	20,560	79,963,700	0.33	0.24	0.24	0.09	0.09	0.06	1.35 [1.29-1.42]*	1.23 [1.17-1.29]*
D52	15,390	80,097,780	0.24	0.18	0.18	0.06	0.06	0.04	1.31 [1.24-1.38]*	1.22 [1.15-1.29]*
D53	4,040	80,145,140	0.07	0.05	0.05	0.02	0.02	0.02	1.43 [1.28-1.59]*	1.30 [1.16-1.45]*
D55	430	80,166,800	0.01	0.00	0.00	0.00	0.00	0.00	1.61 [1.18-2.18]*	1.50 [1.09-2.06]
D56	7,920	80,035,810	0.13	0.09	0.09	0.04	0.04	0.04	1.42 [1.32-1.53]*	1.37 [1.27-1.48]*
D57	7,490	79,996,600	0.12	0.09	0.09	0.04	0.03	0.03	1.37 [1.27-1.47]*	1.32 [1.22-1.43]*
D58	2,020	80,142,250	0.03	0.02	0.02	0.01	0.01	0.00	1.24 [1.07-1.44]*	1.19 [1.02-1.39]
D59	2,600	80,141,520	0.05	0.03	0.03	0.02	0.01	0.01	1.47 [1.30-1.67]*	1.43 [1.25-1.63]*
D61	13,840	80,103,750	0.22	0.16	0.16	0.06	0.05	0.04	1.33 [1.25-1.41]*	1.26 [1.19-1.34]*
D62	6,890	80,048,390	0.10	0.08	0.09	0.02	0.01	0.01	1.13 [1.04-1.23]*	1.10 [1.01-1.20]
D63	21,970	80,047,120	0.34	0.26	0.26	0.08	0.07	0.06	1.29 [1.23-1.35]*	1.21 [1.15-1.27]*
D64	302,270	77,299,520	4.93	3.69	3.79	1.24	1.14	0.85	1.30 [1.29-1.32]*	1.21 [1.19-1.22]*
D65	900	80,168,020	0.01	0.01	0.01	0.00	0.00	0.00	1.25 [1.00-1.57]	1.21 [0.96-1.51]
D66	880	80,152,510	0.01	0.01	0.01	0.00	0.00	0.00	1.13 [0.90-1.43]	1.03 [0.81-1.31]
D68	16,230	79,935,350	0.25	0.19	0.19	0.06	0.06	0.04	1.32 [1.25-1.39]*	1.18 [1.11-1.24]*
D69	43,460	79,771,080	0.67	0.52	0.52	0.15	0.14	0.11	1.27 [1.23-1.31]*	1.20 [1.16-1.24]*
D70	41,210	79,850,660	0.62	0.49	0.50	0.12	0.12	0.10	1.24 [1.20-1.28]*	1.19 [1.15-1.23]*
D72	9,080	80,104,200	0.15	0.10	0.11	0.05	0.05	0.03	1.44 [1.35-1.55]*	1.27 [1.18-1.37]*
D73	9,910	80,083,030	0.15	0.12	0.12	0.03	0.03	0.02	1.24 [1.16-1.32]*	1.15 [1.07-1.23]*
D75	12,550	80,046,470	0.19	0.15	0.15	0.04	0.03	0.02	1.22 [1.15-1.30]*	1.12 [1.05-1.19]*
D76	640	80,157,350	0.01	0.01	0.01	0.00	0.00	0.00	1.37 [1.06-1.78]	1.26 [0.96-1.65]
D80	2,340	80,139,550	0.04	0.03	0.03	0.02	0.02	0.01	1.56 [1.36-1.77]*	1.32 [1.15-1.52]*
D82	490	80,163,810	0.01	0.01	0.01	0.00	0.00	0.00	1.51 [1.12-2.02]*	1.33 [0.98-1.80]
D83	330	80,167,000	0.01	0.00	0.00	0.00	0.00	0.00	2.05 [1.48-2.85]*	1.75 [1.24-2.46]*
D84	1,100	80,156,070	0.02	0.01	0.01	0.01	0.01	0.01	1.70 [1.41-2.05]*	1.35 [1.10-1.65]*
D86	6,290	80,076,740	0.11	0.07	0.07	0.04	0.04	0.03	1.56 [1.44-1.69]*	1.41 [1.30-1.54]*
D89	4,940	80,127,480	0.08	0.06	0.06	0.03	0.02	0.02	1.42 [1.29-1.56]*	1.29 [1.17-1.42]*
18+ - E00-E89										
E02	1,850	80,164,040	0.03	0.02	0.02	0.01	0.00	0.00	1.20 [1.02-1.41]	1.16 [0.98-1.37]
E03	220,270	77,442,500	3.63	2.67	2.72	0.96	0.91	0.71	1.34 [1.32-1.36]*	1.24 [1.23-1.26]*
E04	22,870	79,854,010	0.32	0.28	0.29	0.04	0.04	0.02	1.13 [1.08-1.18]*	1.05 [1.01-1.10]
E05	36,880	79,718,180	0.58	0.44	0.45	0.14	0.13	0.09	1.29 [1.24-1.33]*	1.18 [1.14-1.22]*
E06	3,960	80,115,030	0.06	0.05	0.05	0.01	0.01	0.01	1.22 [1.10-1.36]*	1.13 [1.01-1.26]
E07	6,230	80,099,420	0.10	0.07	0.07	0.02	0.02	0.01	1.29 [1.19-1.41]*	1.17 [1.08-1.28]*
E10	39,890	79,193,410	0.61	0.48	0.47	0.13	0.14	0.07	1.30 [1.25-1.34]*	1.12 [1.09-1.16]*
E11	343,850	75,646,640	5.54	4.33	4.41	1.21	1.14	0.61	1.26 [1.24-1.27]*	1.12 [1.11-1.14]*
E13	4,340	80,138,520	0.07	0.05	0.05	0.02	0.02	0.01	1.39 [1.26-1.54]*	1.18 [1.06-1.31]*
E14	47,840	79,581,090	0.78	0.56	0.57	0.22	0.22	0.12	1.38 [1.34-1.42]*	1.19 [1.15-1.23]*
E16	51,520	79,856,800	0.80	0.61	0.63	0.19	0.17	0.08	1.27 [1.24-1.31]*	1.11 [1.08-1.14]*
E20	1,410	80,158,600	0.02	0.02	0.02	0.01	0.01	0.01	1.49 [1.25-1.77]*	1.30 [1.09-1.56]*
E21	17,310	80,037,460	0.25	0.21	0.21	0.04	0.04	0.02	1.17 [1.11-1.23]*	1.08 [1.02-1.14]*
E22	11,350	80,084,240	0.20	0.13	0.13	0.07	0.06	0.05	1.47 [1.38-1.56]*	1.37 [1.28-1.46]*
E23	5,200	80,080,540	0.09	0.06	0.06	0.03	0.03	0.02	1.43 [1.30-1.56]*	1.27 [1.15-1.39]*
E24	1,300	80,150,800	0.02	0.01	0.02	0.01	0.01	0.00	1.46 [1.22-1.74]*	1.12 [0.93-1.36]
E26	770	80,160,640	0.01	0.01	0.01	0.00	0.00	0.00	1.16 [0.91-1.49]	1.04 [0.80-1.34]
E27	11,690	80,067,770	0.20	0.13	0.13	0.06	0.06	0.04	1.48 [1.39-1.57]*	1.28 [1.20-1.36]*
E28	31,030	79,740,110	0.49	0.37	0.39	0.12	0.10	0.04	1.24 [1.20-1.29]*	1.08 [1.04-1.12]*
E29	1,700	80,150,470	0.03	0.02	0.02	0.01	0.01	0.01	1.76 [1.51-2.05]*	1.42 [1.21-1.67]*
E34	1,770	80,110,540	0.03	0.02	0.02	0.01	0.01	0.00	1.31 [1.12-1.53]*	1.13 [0.96-1.34]
E41	630	80,170,980	0.01	0.01	0.01	0.00	0.00	0.00	1.05 [0.78-1.41]	1.06 [0.78-1.42]
E43	760	80,169,640	0.01	0.01	0.01	0.00	0.00	0.00	1.09 [0.84-1.40]	1.00 [0.77-1.30]
E46	9,480	80,130,200	0.14	0.11	0.12	0.02	0.02	0.01	1.18 [1.10-1.27]*	1.10 [1.02-1.18]
E51	940	80,163,950	0.02	0.01	0.01	0.00	0.00	0.00	1.41 [1.14-1.74]*	1.23 [0.99-1.54]
E53	54,100	79,884,220	0.86	0.64	0.65	0.22	0.20	0.13	1.31 [1.27-1.35]*	1.18 [1.15-1.22]*
E55	70,300	79,861,030	1.17	0.82	0.85	0.36	0.32	0.21	1.38 [1.34-1.42]*	1.22 [1.19-1.25]*
E56	680	80,168,120	0.01	0.01	0.01	0.00	0.00	0.00	1.29 [1.00-1.67]	1.14 [0.87-1.48]
E58	520	80,169,190	0.01	0.01	0.01	0.00	0.00	0.00	1.41 [1.06-1.88]	1.25 [0.93-1.68]
E61	10,600	80,106,180	0.18	0.12	0.13	0.05	0.05	0.03	1.39 [1.30-1.48]*	1.21 [1.14-1.30]*
E63	650	80,168,850	0.01	0.01	0.01	0.00	0.00	0.00	1.17 [0.89-1.52]	1.08 [0.82-1.41]
E65	440	80,153,630	0.01	0.01	0.00	0.00	0.00	0.00	1.54 [1.14-2.08]*	1.39 [1.02-1.90]
E66	382,520	76,679,140	6.33	4.70	4.80	1.63	1.53	0.85	1.32 [1.30-1.33]*	1.15 [1.14-1.17]*
E72	610	80,162,480	0.01	0.01	0.01	0.00	0.00	0.00	1.39 [1.07-1.81]	1.24 [0.94-1.62]
E73	2,470	80,146,600	0.04	0.03	0.03	0.01	0.01	0.00	1.38 [1.21-1.57]*	1.11 [0.96-1.27]
E74	1,140	80,155,760	0.02	0.01	0.01	0.00	0.00	0.00	1.34 [1.10-1.63]*	1.22 [1.00-1.49]
E78	448,200	74,422,520	7.31	5.74	5.94	1.57	1.37	0.76	1.23 [1.22-1.24]*	1.12 [1.10-1.13]*
E79	590	80,167,340	0.01	0.01	0.01	0.00	0.00	0.00	1.35 [1.02-1.77]	1.21 [0.91-1.60]
E80	5,590	80,108,470	0.08	0.07	0.07	0.01	0.01	0.01	1.21 [1.11-1.33]*	1.11 [1.01-1.22]
E83	81,730	79,797,030	1.23	0.98	1.01	0.25	0.22	0.13	1.22 [1.19-1.25]*	1.12 [1.09-1.15]*
E85	2,950	80,156,370	0.04	0.04	0.04	0.01	0.01	0.01	1.22 [1.07-1.38]*	1.14 [1.00-1.29]
E86	207,200	79,213,790	3.12	2.51	2.62	0.62	0.50	0.33	1.19 [1.17-1.21]*	1.12 [1.10-1.14]*
E87	323,540	78,737,560	5.16	3.88	4.01	1.27	1.14	0.80	1.29 [1.27-1.30]*	1.18 [1.17-1.20]*
E88	14,800	80,099,900	0.23	0.17	0.18	0.06	0.06	0.04	1.32 [1.24-1.39]*	1.21 [1.14-1.28]*
E89	20,760	79,950,920	0.32	0.25	0.25	0.07	0.07	0.04	1.26 [1.20-1.32]*	1.14 [1.09-1.20]*
18+ - F01-F99										
F00	51,300	80,017,420	0.72	0.62	0.67	0.09	0.04	0.03	1.06 [1.03-1.10]*	1.04 [1.01-1.08]
F01	58,730	79,987,690	0.83	0.71	0.76	0.12	0.07	0.04	1.10 [1.06-1.13]*	1.05 [1.02-1.08]*
F02	10,280	80,140,290	0.13	0.13	0.13	0.01	0.00	0.00	1.01 [0.94-1.09]	0.99 [0.92-1.07]
F03	141,440	79,704,490	1.99	1.73	1.87	0.27	0.12	0.08	1.06 [1.04-1.09]*	1.04 [1.02-1.06]*
F05	129,540	79,856,300	1.93	1.56	1.65	0.38	0.28	0.18	1.17 [1.15-1.19]*	1.10 [1.08-1.13]*
F06	20,330	80,075,900	0.30	0.24	0.26	0.05	0.04	0.03	1.16 [1.10-1.22]*	1.10 [1.04-1.16]*
F07	1,240	80,151,110	0.02	0.01	0.02	0.01	0.00	0.00	1.29 [1.07-1.55]*	1.16 [0.96-1.41]
F10	105,170	78,294,640	1.58	1.29	1.29	0.29	0.29	0.16	1.23 [1.20-1.25]*	1.11 [1.09-1.14]*
F11	8,220	79,936,370	0.12	0.10	0.10	0.02	0.02	−0.01	1.18 [1.10-1.28]*	0.95 [0.88-1.03]
F12	16,910	79,960,920	0.24	0.21	0.21	0.03	0.02	−0.01	1.12 [1.06-1.18]*	0.95 [0.90-1.00]
F13	1,820	80,143,720	0.03	0.02	0.02	0.01	0.01	0.00	1.24 [1.06-1.45]*	0.98 [0.83-1.15]
F14	7,070	80,076,110	0.10	0.09	0.09	0.01	0.01	0.00	1.16 [1.07-1.26]*	0.97 [0.89-1.05]
F15	2,380	80,123,500	0.03	0.03	0.03	0.00	0.00	0.00	1.04 [0.90-1.20]	0.93 [0.80-1.08]
F16	710	80,153,180	0.01	0.01	0.01	0.00	0.00	0.00	0.87 [0.66-1.15]	0.78 [0.59-1.03]
F17	522,180	75,245,470	7.89	6.73	6.86	1.16	1.03	0.16	1.15 [1.14-1.16]*	1.02 [1.01-1.03]*
F19	7,530	80,039,390	0.10	0.09	0.09	0.01	0.01	−0.01	1.08 [1.00-1.17]	0.89 [0.82-0.97]*

F20	15,640	79,774,600	0.24	0.19	0.19	0.05	0.05	0.04	1.27 [1.20-1.34]*	1.17 [1.11-1.24]*
F22	6,980	80,086,630	0.11	0.08	0.08	0.02	0.02	0.02	1.28 [1.18-1.39]*	1.18 [1.08-1.28]*
F23	4,240	80,081,270	0.06	0.05	0.05	0.01	0.01	0.00	1.12 [1.01-1.24]	1.05 [0.94-1.17]
F25	3,290	80,086,970	0.05	0.04	0.04	0.01	0.01	0.01	1.34 [1.20-1.50]*	1.19 [1.06-1.34]*
F29	7,820	80,074,980	0.12	0.09	0.09	0.03	0.02	0.01	1.26 [1.17-1.36]*	1.11 [1.03-1.20]*
F30	2,050	80,114,130	0.03	0.02	0.02	0.01	0.01	0.01	1.33 [1.15-1.54]*	1.21 [1.05-1.41]
F31	18,770	79,851,170	0.30	0.22	0.23	0.08	0.07	0.02	1.32 [1.26-1.39]*	1.08 [1.03-1.14]*
F32	344,020	76,481,710	5.91	4.19	4.29	1.72	1.63	0.68	1.38 [1.36-1.40]*	1.13 [1.12-1.14]*
F33	10,780	79,917,480	0.17	0.13	0.13	0.04	0.04	0.01	1.30 [1.22-1.39]*	1.07 [1.00-1.14]
F34	1,720	80,144,770	0.03	0.02	0.02	0.01	0.01	0.00	1.47 [1.26-1.72]*	1.18 [1.00-1.39]
F38	810	80,163,640	0.01	0.01	0.01	0.00	0.00	0.00	1.27 [1.00-1.62]	1.09 [0.85-1.40]
F39	1,360	80,154,190	0.02	0.02	0.02	0.01	0.01	0.00	1.35 [1.13-1.61]*	1.10 [0.92-1.33]
F40	29,180	79,880,880	0.46	0.34	0.36	0.12	0.11	0.05	1.30 [1.25-1.35]*	1.13 [1.09-1.18]*
F41	289,410	77,972,800	4.84	3.47	3.58	1.37	1.25	0.50	1.35 [1.33-1.37]*	1.11 [1.10-1.13]*
F42	6,670	80,091,320	0.13	0.07	0.08	0.05	0.05	0.03	1.67 [1.54-1.80]*	1.33 [1.22-1.44]*
F43	16,300	79,913,940	0.25	0.19	0.20	0.06	0.06	0.00	1.28 [1.21-1.35]*	1.01 [0.95-1.06]
F44	3,700	80,122,590	0.06	0.04	0.04	0.02	0.02	0.01	1.43 [1.28-1.59]*	1.11 [1.00-1.25]
F45	4,560	80,088,620	0.08	0.05	0.05	0.02	0.02	0.01	1.40 [1.27-1.54]*	1.15 [1.04-1.27]*
F48	1,250	80,153,460	0.02	0.01	0.01	0.01	0.01	0.00	1.56 [1.30-1.87]*	1.27 [1.05-1.54]
F50	5,670	80,058,330	0.09	0.07	0.07	0.02	0.02	0.01	1.29 [1.18-1.41]*	1.08 [0.99-1.18]
F51	1,170	80,156,970	0.02	0.01	0.01	0.01	0.01	0.01	1.57 [1.30-1.89]*	1.32 [1.09-1.60]*
F52	2,680	80,126,510	0.04	0.03	0.03	0.01	0.01	0.01	1.48 [1.30-1.67]*	1.25 [1.10-1.42]*
F53	2,640	80,124,710	0.04	0.03	0.03	0.01	0.01	0.01	1.34 [1.18-1.52]*	1.14 [0.99-1.30]
F60	13,830	79,931,610	0.23	0.16	0.17	0.07	0.06	0.01	1.39 [1.31-1.47]*	1.05 [0.99-1.11]
F61	520	80,163,070	0.01	0.01	0.01	0.00	0.00	0.00	1.17 [0.88-1.56]	0.97 [0.71-1.32]
F69	910	80,151,420	0.02	0.01	0.01	0.01	0.01	0.00	1.62 [1.31-1.99]*	1.27 [1.01-1.58]
F70	1,780	80,130,620	0.04	0.02	0.02	0.02	0.02	0.01	1.87 [1.62-2.16]*	1.65 [1.42-1.92]*
F71	680	80,155,010	0.02	0.01	0.01	0.01	0.01	0.01	2.40 [1.92-3.00]*	2.23 [1.77-2.82]*
F72	1,100	80,144,110	0.03	0.01	0.01	0.01	0.01	0.01	2.15 [1.80-2.57]*	2.09 [1.74-2.52]*
F79	6,820	80,056,830	0.14	0.07	0.07	0.07	0.07	0.06	1.89 [1.76-2.04]*	1.74 [1.61-1.88]*
F80	2,960	80,124,610	0.05	0.03	0.03	0.01	0.01	0.01	1.38 [1.22-1.56]*	1.29 [1.13-1.46]*
F81	18,290	79,755,950	0.38	0.20	0.20	0.18	0.18	0.17	1.92 [1.83-2.00]*	1.79 [1.71-1.88]*
F84	8,200	79,985,810	0.15	0.09	0.10	0.06	0.05	0.04	1.54 [1.44-1.65]*	1.34 [1.24-1.44]*
F89	650	80,159,130	0.01	0.01	0.01	0.00	0.00	0.00	1.53 [1.20-1.96]*	1.33 [1.02-1.73]
F90	4,690	80,072,810	0.07	0.06	0.06	0.02	0.01	0.01	1.23 [1.12-1.35]*	1.09 [0.99-1.21]
F91	770	80,132,720	0.01	0.01	0.01	0.00	0.00	0.00	1.19 [0.93-1.52]	1.08 [0.84-1.39]
F95	550	80,160,130	0.01	0.01	0.01	0.00	0.00	0.00	1.52 [1.16-1.98]*	1.23 [0.93-1.64]
F98	580	80,152,410	0.01	0.01	0.01	0.00	0.00	0.00	1.26 [0.95-1.66]	1.08 [0.81-1.44]
F99	5,420	80,049,540	0.08	0.06	0.07	0.02	0.02	0.01	1.27 [1.16-1.39]*	1.08 [0.99-1.19]
18+ - G00-G99										
G00	1,300	80,145,200	0.02	0.02	0.02	0.00	0.00	0.00	1.20 [1.00-1.44]	1.12 [0.92-1.35]
G02	620	80,163,070	0.01	0.01	0.01	0.00	0.00	0.00	1.19 [0.90-1.56]	1.04 [0.78-1.39]
G03	1,540	80,144,640	0.02	0.02	0.02	0.00	0.00	0.00	1.17 [0.98-1.39]	1.03 [0.86-1.24]
G04	3,780	80,130,350	0.06	0.04	0.05	0.01	0.01	0.01	1.28 [1.15-1.43]*	1.17 [1.04-1.30]*
G05	960	80,161,550	0.02	0.01	0.01	0.01	0.01	0.01	1.55 [1.26-1.91]*	1.46 [1.18-1.81]*
G06	1,790	80,149,230	0.03	0.02	0.02	0.00	0.00	0.00	1.20 [1.02-1.41]	1.14 [0.97-1.34]
G08	910	80,161,160	0.01	0.01	0.01	0.00	0.00	0.00	1.32 [1.06-1.65]	1.26 [1.00-1.58]
G09	960	80,154,610	0.02	0.01	0.01	0.01	0.00	0.00	1.38 [1.12-1.70]*	1.27 [1.03-1.57]
G10	620	80,164,800	0.01	0.01	0.01	0.00	0.00	0.00	1.33 [1.02-1.74]	1.39 [1.05-1.83]
G11	1,820	80,147,570	0.03	0.02	0.02	0.00	0.01	0.00	1.25 [1.07-1.46]*	1.16 [0.99-1.36]
G12	4,240	80,151,580	0.06	0.05	0.05	0.01	0.01	0.00	1.12 [1.00-1.24]	1.07 [0.96-1.20]
G20	39,030	79,917,280	0.53	0.48	0.50	0.04	0.03	0.01	1.05 [1.01-1.09]*	1.02 [0.99-1.06]
G21	3,390	80,154,550	0.05	0.04	0.04	0.01	0.01	0.01	1.27 [1.13-1.43]*	1.20 [1.06-1.35]*
G23	2,110	80,164,420	0.03	0.03	0.03	0.00	0.00	0.00	1.12 [0.96-1.30]	1.07 [0.92-1.25]
G24	3,890	80,116,180	0.06	0.05	0.05	0.01	0.01	0.01	1.20 [1.08-1.34]*	1.10 [0.98-1.23]
G25	20,980	80,033,280	0.33	0.25	0.25	0.08	0.08	0.04	1.30 [1.24-1.36]*	1.12 [1.07-1.17]*
G30	68,840	79,929,490	0.96	0.84	0.90	0.12	0.07	0.05	1.07 [1.04-1.10]*	1.05 [1.02-1.08]*
G31	49,290	79,945,050	0.72	0.59	0.61	0.12	0.11	0.08	1.18 [1.14-1.22]*	1.12 [1.08-1.16]*
G32	800	80,165,300	0.01	0.01	0.01	0.00	0.00	0.00	1.29 [1.02-1.63]	1.12 [0.88-1.43]
G35	10,540	79,921,820	0.16	0.13	0.13	0.03	0.03	0.01	1.22 [1.15-1.30]*	1.08 [1.01-1.15]
G37	2,670	80,113,840	0.04	0.03	0.03	0.01	0.01	0.01	1.30 [1.14-1.47]*	1.16 [1.02-1.33]
G40	62,420	78,853,860	1.01	0.74	0.74	0.27	0.27	0.16	1.36 [1.32-1.40]*	1.19 [1.15-1.22]*
G41	3,840	80,113,080	0.06	0.05	0.05	0.02	0.02	0.01	1.33 [1.20-1.48]*	1.20 [1.07-1.34]*
G43	64,020	79,382,920	1.04	0.76	0.78	0.28	0.25	0.08	1.32 [1.29-1.36]*	1.08 [1.05-1.11]*
G44	9,430	80,026,910	0.15	0.11	0.11	0.04	0.04	0.02	1.31 [1.23-1.40]*	1.12 [1.04-1.20]*
G45	47,120	79,613,830	0.69	0.57	0.58	0.12	0.11	0.06	1.18 [1.15-1.22]*	1.10 [1.07-1.14]*
G46	3,070	80,152,960	0.05	0.04	0.04	0.01	0.01	0.00	1.18 [1.04-1.34]*	1.12 [0.98-1.27]
G47	60,510	79,340,920	1.09	0.69	0.69	0.40	0.40	0.24	1.58 [1.54-1.62]*	1.28 [1.24-1.31]*
G50	7,680	80,081,670	0.13	0.09	0.09	0.04	0.04	0.02	1.43 [1.33-1.55]*	1.22 [1.13-1.32]*
G51	15,090	79,965,510	0.23	0.18	0.18	0.05	0.04	0.03	1.24 [1.17-1.31]*	1.13 [1.06-1.19]*
G52	1,160	80,158,070	0.02	0.01	0.01	0.00	0.00	0.00	1.32 [1.08-1.60]*	1.18 [0.97-1.45]
G53	2,650	80,151,010	0.04	0.03	0.03	0.01	0.01	0.01	1.32 [1.15-1.51]*	1.21 [1.06-1.39]*
G54	4,970	80,108,040	0.08	0.06	0.06	0.02	0.02	0.01	1.29 [1.17-1.42]*	1.10 [1.00-1.22]
G55	54,890	79,479,170	0.84	0.66	0.66	0.18	0.18	0.08	1.27 [1.23-1.31]*	1.11 [1.07-1.14]*
G56	82,240	78,384,650	1.32	0.99	0.99	0.33	0.33	0.20	1.34 [1.31-1.37]*	1.18 [1.15-1.21]*
G57	9,510	80,000,380	0.15	0.11	0.11	0.04	0.04	0.03	1.40 [1.31-1.50]*	1.25 [1.17-1.34]*
G58	4,760	80,100,780	0.08	0.06	0.05	0.02	0.02	0.01	1.40 [1.27-1.54]*	1.21 [1.09-1.33]*
G59	3,740	80,125,150	0.06	0.04	0.04	0.02	0.02	0.01	1.47 [1.33-1.64]*	1.18 [1.05-1.32]*
G60	2,180	80,141,610	0.03	0.03	0.03	0.01	0.01	0.00	1.26 [1.10-1.46]*	1.12 [0.97-1.30]
G61	2,730	80,125,460	0.04	0.03	0.03	0.01	0.01	0.01	1.26 [1.11-1.43]*	1.19 [1.05-1.36]*
G62	30,200	79,943,570	0.48	0.36	0.36	0.13	0.13	0.07	1.36 [1.30-1.41]*	1.16 [1.12-1.21]*
G63	15,880	80,064,770	0.27	0.18	0.19	0.09	0.08	0.04	1.44 [1.37-1.52]*	1.16 [1.10-1.23]*
G70	2,660	80,136,020	0.04	0.03	0.03	0.01	0.01	0.00	1.17 [1.02-1.33]	1.09 [0.95-1.25]
G71	1,790	80,135,240	0.03	0.02	0.02	0.01	0.00	0.00	1.22 [1.04-1.43]	1.16 [0.98-1.36]
G72	3,180	80,138,570	0.05	0.04	0.04	0.02	0.02	0.01	1.42 [1.27-1.60]*	1.28 [1.13-1.44]*
G80	3,500	80,023,650	0.06	0.04	0.04	0.02	0.02	0.01	1.39 [1.25-1.55]*	1.33 [1.19-1.49]*
G81	53,970	79,669,480	0.79	0.65	0.67	0.14	0.13	0.08	1.19 [1.15-1.23]*	1.12 [1.08-1.15]*
G82	5,380	80,071,480	0.08	0.06	0.06	0.01	0.01	0.01	1.22 [1.11-1.34]*	1.14 [1.04-1.25]*
G83	8,340	80,086,550	0.12	0.10	0.10	0.02	0.02	0.01	1.20 [1.12-1.30]*	1.09 [1.01-1.17]
G90	4,420	80,127,690	0.07	0.05	0.05	0.01	0.01	0.00	1.23 [1.11-1.36]*	1.07 [0.97-1.19]
G91	9,700	80,064,700	0.14	0.12	0.12	0.03	0.03	0.02	1.21 [1.13-1.30]*	1.16 [1.08-1.25]*
G93	40,710	79,815,670	0.63	0.48	0.49	0.14	0.13	0.07	1.27 [1.23-1.31]*	1.12 [1.08-1.16]*
G94	1,330	80,162,300	0.02	0.02	0.02	0.00	0.00	0.00	1.25 [1.04-1.50]	1.22 [1.01-1.47]
G95	11,430	80,062,640	0.17	0.14	0.14	0.03	0.03	0.02	1.22 [1.14-1.29]*	1.13 [1.06-1.21]*
G96	3,430	80,124,140	0.05	0.04	0.04	0.01	0.01	0.01	1.35 [1.20-1.50]*	1.18 [1.05-1.32]*
G97	3,220	80,116,420	0.05	0.04	0.04	0.01	0.01	0.00	1.26 [1.12-1.42]*	1.09 [0.97-1.23]
G98	1,160	80,164,560	0.02	0.01	0.01	0.01	0.01	0.00	1.46 [1.20-1.76]*	1.18 [0.97-1.45]
G99	18,070	79,994,670	0.27	0.22	0.21	0.06	0.06	0.03	1.28 [1.21-1.34]*	1.13 [1.08-1.19]*
18+ - H00-H59										
H00	11,560	79,772,400	0.17	0.14	0.14	0.03	0.04	0.03	1.27 [1.19-1.35]*	1.22 [1.14-1.29]*
H01	10,800	80,042,890	0.18	0.13	0.13	0.05	0.05	0.04	1.39 [1.30-1.48]*	1.29 [1.21-1.38]*
H02	57,230	79,164,860	0.90	0.68	0.69	0.22	0.22	0.16	1.32 [1.28-1.35]*	1.22 [1.19-1.26]*
H03	590	80,158,970	0.01	0.01	0.01	0.00	0.00	0.00	1.41 [1.07-1.84]	1.25 [0.95-1.65]
H04	26,580	79,731,600	0.45	0.31	0.31	0.14	0.14	0.11	1.44 [1.38-1.50]*	1.32 [1.27-1.37]*
H05	4,880	80,053,780	0.08	0.06	0.06	0.03	0.02	0.02	1.43 [1.30-1.57]*	1.31 [1.19-1.45]*
H06	1,180	80,153,280	0.02	0.01	0.01	0.01	0.01	0.00	1.45 [1.21-1.75]*	1.28 [1.05-1.55]
H10	11,670	80,047,420	0.20	0.14	0.14	0.06	0.06	0.04	1.43 [1.34-1.52]*	1.29 [1.21-1.38]*

H11	11,910	79,990,360	0.18	0.14	0.14	0.04	0.03	0.03	1.24 [1.16-1.32]*	1.18 [1.11-1.26]*
H13	660	80,161,870	0.01	0.01	0.01	0.00	0.00	0.00	1.59 [1.24-2.03]*	1.50 [1.16-1.93]*
H15	1,070	80,156,610	0.02	0.01	0.01	0.01	0.01	0.00	1.45 [1.19-1.77]*	1.28 [1.05-1.58]
H16	5,130	80,092,560	0.09	0.06	0.06	0.04	0.04	0.03	1.64 [1.50-1.80]*	1.52 [1.39-1.67]*
H17	3,010	80,135,600	0.05	0.03	0.04	0.02	0.01	0.01	1.40 [1.24-1.57]*	1.30 [1.15-1.48]*
H18	21,360	79,971,630	0.35	0.25	0.26	0.10	0.09	0.08	1.36 [1.30-1.43]*	1.27 [1.22-1.34]*
H19	1,300	80,155,570	0.03	0.01	0.01	0.01	0.01	0.01	1.85 [1.55-2.19]*	1.59 [1.33-1.90]*
H20	5,410	80,087,020	0.09	0.06	0.06	0.03	0.02	0.02	1.38 [1.26-1.50]*	1.29 [1.18-1.41]*
H21	9,050	80,083,880	0.13	0.11	0.11	0.02	0.02	0.01	1.18 [1.09-1.26]*	1.12 [1.04-1.21]*
H22	420	80,168,300	0.01	0.00	0.01	0.00	0.00	0.00	1.45 [1.06-1.98]	1.28 [0.92-1.77]
H25	220,590	77,744,420	3.34	2.73	2.85	0.61	0.49	0.35	1.17 [1.16-1.19]*	1.12 [1.10-1.13]*
H26	310,740	76,301,420	4.93	3.89	4.08	1.04	0.85	0.62	1.21 [1.19-1.22]*	1.14 [1.13-1.16]*
H27	4,110	80,108,110	0.06	0.05	0.05	0.01	0.01	0.01	1.22 [1.09-1.35]*	1.16 [1.04-1.29]*
H28	1,610	80,154,350	0.03	0.02	0.02	0.01	0.01	0.00	1.35 [1.14-1.59]*	1.22 [1.03-1.44]
H30	490	80,164,030	0.01	0.01	0.01	0.00	0.00	0.00	1.29 [0.96-1.74]	1.17 [0.87-1.59]
H31	5,180	80,123,870	0.07	0.06	0.07	0.01	0.01	0.01	1.13 [1.02-1.24]	1.09 [0.98-1.20]
H33	20,280	79,786,420	0.27	0.25	0.25	0.02	0.02	0.01	1.08 [1.02-1.13]*	1.05 [1.00-1.11]
H34	12,600	80,048,470	0.18	0.15	0.16	0.03	0.03	0.02	1.17 [1.10-1.24]*	1.11 [1.04-1.18]*
H35	114,660	79,168,620	1.69	1.40	1.44	0.30	0.25	0.18	1.17 [1.15-1.20]*	1.12 [1.10-1.15]*
H36	45,930	79,714,600	0.70	0.55	0.56	0.15	0.14	0.06	1.25 [1.21-1.29]*	1.09 [1.06-1.13]*
H40	105,600	79,099,740	1.55	1.29	1.32	0.26	0.23	0.18	1.18 [1.15-1.20]*	1.13 [1.11-1.16]*
H43	21,350	79,925,070	0.30	0.26	0.27	0.04	0.03	0.02	1.13 [1.07-1.18]*	1.07 [1.01-1.12]
H44	6,590	80,085,460	0.10	0.08	0.08	0.02	0.02	0.02	1.32 [1.22-1.43]*	1.25 [1.15-1.36]*
H46	1,960	80,142,220	0.03	0.02	0.02	0.01	0.01	0.01	1.41 [1.22-1.63]*	1.27 [1.09-1.48]*
H47	7,460	80,079,790	0.11	0.09	0.09	0.02	0.02	0.01	1.23 [1.13-1.33]*	1.13 [1.04-1.23]*
H49	5,000	80,107,370	0.07	0.06	0.06	0.01	0.01	0.00	1.10 [1.00-1.21]	1.02 [0.92-1.13]
H50	8,680	79,816,600	0.13	0.10	0.10	0.03	0.03	0.02	1.27 [1.18-1.37]*	1.19 [1.11-1.28]*
H51	800	80,160,280	0.01	0.01	0.01	0.00	0.00	0.00	1.25 [0.98-1.59]	1.12 [0.88-1.44]
H52	34,000	79,881,490	0.48	0.41	0.42	0.07	0.06	0.04	1.13 [1.09-1.17]*	1.09 [1.05-1.13]*
H53	66,430	79,290,290	1.02	0.80	0.82	0.22	0.20	0.10	1.25 [1.22-1.28]*	1.11 [1.08-1.14]*
H54	50,990	79,833,400	0.76	0.61	0.63	0.15	0.14	0.10	1.22 [1.18-1.25]*	1.14 [1.11-1.18]*
H55	4,360	80,122,850	0.06	0.05	0.05	0.01	0.01	0.01	1.19 [1.07-1.32]*	1.09 [0.98-1.21]
H57	10,630	80,050,920	0.16	0.13	0.13	0.04	0.03	0.02	1.27 [1.19-1.36]*	1.15 [1.07-1.23]*
H58	2,220	80,152,120	0.03	0.03	0.03	0.01	0.01	0.00	1.26 [1.09-1.46]*	1.15 [1.00-1.34]
H59	4,090	80,110,430	0.06	0.05	0.05	0.01	0.01	0.01	1.16 [1.05-1.30]*	1.13 [1.01-1.26]
18+ - H60-H95										
H60	8,970	79,975,150	0.17	0.10	0.10	0.07	0.07	0.06	1.71 [1.60-1.83]*	1.54 [1.43-1.64]*
H61	12,720	79,877,880	0.22	0.15	0.15	0.07	0.07	0.06	1.45 [1.37-1.54]*	1.35 [1.27-1.43]*
H65	7,060	79,326,880	0.11	0.08	0.08	0.03	0.03	0.02	1.35 [1.25-1.46]*	1.22 [1.12-1.32]*
H66	8,700	79,756,590	0.15	0.10	0.10	0.05	0.05	0.04	1.53 [1.43-1.64]*	1.39 [1.29-1.49]*
H69	1,670	80,133,000	0.03	0.02	0.02	0.01	0.01	0.01	1.49 [1.27-1.74]*	1.28 [1.09-1.51]*
H70	1,660	80,138,850	0.03	0.02	0.02	0.01	0.01	0.00	1.38 [1.17-1.63]*	1.23 [1.04-1.46]
H71	4,440	80,029,630	0.06	0.05	0.05	0.01	0.01	0.00	1.12 [1.01-1.24]	1.08 [0.98-1.21]
H72	7,880	79,869,510	0.12	0.09	0.10	0.02	0.02	0.02	1.25 [1.16-1.35]*	1.18 [1.09-1.27]*
H73	3,390	80,021,070	0.05	0.04	0.04	0.01	0.01	0.01	1.32 [1.18-1.48]*	1.23 [1.09-1.38]*
H74	4,210	80,029,740	0.06	0.05	0.05	0.01	0.01	0.01	1.24 [1.12-1.37]*	1.16 [1.04-1.29]*
H80	1,440	80,136,440	0.02	0.02	0.02	0.00	0.00	0.00	1.12 [0.93-1.34]	1.09 [0.91-1.32]
H81	21,900	79,971,160	0.34	0.26	0.27	0.08	0.07	0.04	1.26 [1.20-1.32]*	1.14 [1.09-1.20]*
H83	8,840	80,033,850	0.14	0.11	0.11	0.03	0.03	0.02	1.28 [1.19-1.38]*	1.16 [1.08-1.25]*
H90	19,960	79,934,330	0.33	0.23	0.24	0.09	0.09	0.06	1.36 [1.30-1.43]*	1.23 [1.17-1.29]*
H91	128,680	79,322,380	1.98	1.55	1.60	0.43	0.38	0.28	1.24 [1.21-1.26]*	1.16 [1.14-1.19]*
H92	6,490	80,000,300	0.11	0.07	0.07	0.04	0.04	0.03	1.49 [1.37-1.61]*	1.31 [1.20-1.42]*
H93	12,120	80,039,050	0.20	0.14	0.14	0.05	0.05	0.04	1.37 [1.29-1.46]*	1.23 [1.16-1.31]*
H95	970	80,140,400	0.02	0.01	0.01	0.00	0.00	0.00	1.36 [1.10-1.67]*	1.27 [1.03-1.58]
18+ - I00-I99										
I05	7,070	80,086,800	0.11	0.08	0.09	0.03	0.02	0.02	1.27 [1.17-1.37]*	1.17 [1.07-1.27]*
I06	720	80,160,050	0.01	0.01	0.01	0.00	0.00	0.00	1.25 [0.97-1.60]	1.14 [0.89-1.48]
I07	17,970	80,091,840	0.28	0.21	0.22	0.07	0.07	0.05	1.30 [1.23-1.37]*	1.20 [1.14-1.26]*
I08	92,780	79,713,320	1.40	1.11	1.15	0.29	0.25	0.17	1.22 [1.19-1.25]*	1.13 [1.11-1.16]*
I09	1,040	80,160,100	0.02	0.01	0.01	0.01	0.01	0.00	1.43 [1.17-1.74]*	1.35 [1.10-1.65]*
I10	920,510	66,639,780	17.21	13.07	13.86	4.15	3.35	1.94	1.24 [1.23-1.25]*	1.13 [1.12-1.14]*
I11	5,240	80,112,440	0.08	0.06	0.06	0.01	0.01	0.01	1.21 [1.10-1.33]*	1.08 [0.98-1.19]
I12	52,310	79,698,950	0.81	0.62	0.62	0.18	0.19	0.13	1.30 [1.27-1.34]*	1.19 [1.15-1.23]*
I13	2,600	80,164,800	0.04	0.03	0.03	0.01	0.01	0.01	1.29 [1.12-1.48]*	1.15 [1.00-1.33]
I15	3,370	80,128,940	0.05	0.04	0.04	0.01	0.01	0.01	1.31 [1.17-1.47]*	1.17 [1.04-1.32]
I20	203,410	76,281,770	3.29	2.53	2.58	0.76	0.71	0.40	1.27 [1.26-1.29]*	1.14 [1.12-1.15]*
I21	138,650	78,518,590	2.01	1.71	1.74	0.30	0.27	0.16	1.15 [1.13-1.18]*	1.08 [1.06-1.11]*
I22	12,540	79,997,550	0.19	0.15	0.15	0.04	0.04	0.03	1.28 [1.21-1.36]*	1.16 [1.09-1.23]*
I23	790	80,161,780	0.01	0.01	0.01	0.00	0.00	0.00	1.23 [0.97-1.56]	1.15 [0.90-1.47]
I24	39,370	79,883,590	0.60	0.47	0.48	0.13	0.12	0.07	1.25 [1.21-1.29]*	1.13 [1.09-1.17]*
I25	348,390	75,094,330	5.63	4.42	4.61	1.21	1.02	0.57	1.22 [1.21-1.24]*	1.11 [1.10-1.13]*
I26	71,410	79,547,270	1.00	0.88	0.89	0.12	0.11	0.05	1.12 [1.09-1.15]*	1.05 [1.02-1.08]*
I27	42,540	79,972,120	0.68	0.50	0.51	0.18	0.16	0.11	1.32 [1.28-1.37]*	1.19 [1.15-1.23]*
I28	1,120	80,161,690	0.02	0.01	0.01	0.00	0.00	0.00	1.19 [0.97-1.46]	1.06 [0.86-1.31]
I30	2,140	80,131,130	0.03	0.03	0.03	0.01	0.01	0.00	1.20 [1.04-1.39]	1.13 [0.98-1.31]
I31	26,240	79,955,950	0.39	0.32	0.32	0.07	0.07	0.04	1.23 [1.18-1.28]*	1.13 [1.08-1.18]*
I33	4,320	80,134,200	0.07	0.05	0.05	0.02	0.02	0.01	1.36 [1.23-1.51]*	1.26 [1.13-1.40]*
I34	60,300	79,676,220	0.89	0.73	0.74	0.16	0.14	0.09	1.19 [1.16-1.22]*	1.11 [1.08-1.14]*
I35	88,160	79,535,630	1.34	1.06	1.09	0.28	0.25	0.16	1.23 [1.20-1.26]*	1.14 [1.11-1.17]*
I36	6,350	80,142,560	0.10	0.08	0.08	0.02	0.02	0.01	1.21 [1.11-1.32]*	1.12 [1.02-1.22]
I37	6,690	80,134,830	0.10	0.08	0.08	0.02	0.02	0.01	1.26 [1.15-1.37]*	1.17 [1.07-1.27]*
I38	9,390	80,079,940	0.15	0.11	0.11	0.04	0.03	0.02	1.30 [1.21-1.40]*	1.20 [1.11-1.29]*
I40	710	80,164,370	0.01	0.01	0.01	0.00	0.00	0.00	1.23 [0.96-1.58]	1.19 [0.92-1.54]
I42	22,020	79,937,940	0.33	0.26	0.27	0.06	0.06	0.03	1.23 [1.18-1.29]*	1.12 [1.07-1.17]*
I43	920	80,168,950	0.01	0.01	0.01	0.00	0.00	0.00	1.07 [0.85-1.36]	0.99 [0.78-1.27]
I44	139,120	79,258,740	2.09	1.68	1.74	0.41	0.36	0.23	1.20 [1.18-1.23]*	1.13 [1.10-1.15]*
I45	86,440	79,590,720	1.30	1.04	1.08	0.26	0.22	0.14	1.21 [1.18-1.24]*	1.12 [1.09-1.15]*
I46	44,890	80,041,190	0.67	0.54	0.54	0.13	0.13	0.09	1.24 [1.20-1.28]*	1.15 [1.11-1.19]*
I47	49,570	79,507,640	0.74	0.60	0.61	0.14	0.13	0.07	1.22 [1.18-1.26]*	1.11 [1.08-1.15]*
I48	387,130	76,982,860	5.92	4.84	5.09	1.09	0.83	0.53	1.16 [1.15-1.18]*	1.10 [1.09-1.11]*
I49	59,100	79,625,140	0.87	0.71	0.73	0.16	0.14	0.07	1.19 [1.15-1.22]*	1.09 [1.06-1.12]*
I50	293,480	78,465,420	4.65	3.54	3.69	1.10	0.95	0.62	1.26 [1.24-1.27]*	1.15 [1.14-1.17]*
I51	162,320	79,188,930	2.50	1.95	2.00	0.54	0.50	0.30	1.25 [1.23-1.27]*	1.14 [1.12-1.16]*
I60	9,480	80,048,470	0.13	0.12	0.11	0.02	0.02	0.02	1.16 [1.08-1.25]*	1.13 [1.05-1.21]*
I61	23,720	80,021,680	0.34	0.29	0.29	0.05	0.05	0.04	1.17 [1.12-1.23]*	1.14 [1.09-1.20]*
I62	13,630	80,089,880	0.20	0.16	0.17	0.03	0.03	0.02	1.18 [1.11-1.25]*	1.13 [1.07-1.21]*
I63	112,580	79,364,690	1.56	1.39	1.43	0.17	0.13	0.07	1.09 [1.07-1.12]*	1.05 [1.03-1.07]*
I64	29,280	79,830,210	0.41	0.36	0.36	0.06	0.05	0.03	1.14 [1.09-1.19]*	1.09 [1.04-1.13]*
I65	27,100	79,907,090	0.39	0.33	0.34	0.07	0.06	0.03	1.17 [1.12-1.22]*	1.09 [1.04-1.14]*
I66	1,670	80,158,680	0.02	0.02	0.02	0.00	0.00	0.00	1.14 [0.96-1.36]	1.08 [0.91-1.29]
I67	169,440	79,369,040	2.53	2.05	2.14	0.48	0.39	0.25	1.18 [1.16-1.20]*	1.11 [1.09-1.13]*
I68	970	80,169,470	0.02	0.01	0.01	0.00	0.00	0.00	1.24 [1.00-1.54]	1.19 [0.95-1.49]
I69	49,620	79,732,260	0.73	0.60	0.61	0.13	0.12	0.07	1.19 [1.15-1.23]*	1.11 [1.07-1.14]*
I70	42,110	79,860,360	0.65	0.50	0.52	0.15	0.13	0.08	1.25 [1.21-1.30]*	1.14 [1.10-1.18]*
I71	51,810	79,806,720	0.75	0.63	0.64	0.12	0.11	0.07	1.17 [1.13-1.21]*	1.11 [1.07-1.14]*
I72	12,400	80,060,340	0.18	0.15	0.15	0.03	0.03	0.01	1.18 [1.11-1.25]*	1.09 [1.02-1.16]*
I73	92,740	79,255,430	1.54	1.09	1.10	0.45	0.44	0.30	1.40 [1.37-1.43]*	1.24 [1.21-1.27]*

I74	24,030	79,940,130	0.35	0.29	0.30	0.05	0.05	0.02	1.17 [1.12-1.22]*	1.07 [1.02-1.12]*
I77	31,420	79,828,680	0.50	0.37	0.37	0.12	0.12	0.08	1.32 [1.27-1.37]*	1.20 [1.15-1.24]*
I78	7,740	80,069,570	0.12	0.09	0.09	0.03	0.03	0.02	1.35 [1.25-1.45]*	1.24 [1.14-1.34]*
I79	3,150	80,142,840	0.05	0.04	0.04	0.01	0.01	0.01	1.35 [1.20-1.52]*	1.13 [1.00-1.28]
I80	63,680	79,306,270	0.97	0.77	0.78	0.20	0.19	0.13	1.24 [1.21-1.28]*	1.16 [1.12-1.19]*
I81	3,590	80,156,140	0.05	0.04	0.04	0.01	0.01	0.00	1.17 [1.04-1.31]*	1.08 [0.96-1.22]
I82	8,000	80,109,230	0.12	0.10	0.10	0.02	0.02	0.01	1.21 [1.12-1.31]*	1.12 [1.04-1.21]*
I83	73,640	78,408,760	1.38	0.84	0.85	0.54	0.53	0.49	1.63 [1.59-1.67]*	1.55 [1.52-1.59]*
I84	115,340	77,259,280	1.89	1.41	1.38	0.48	0.51	0.37	1.37 [1.34-1.39]*	1.24 [1.22-1.27]*
I85	9,770	80,080,350	0.17	0.11	0.11	0.05	0.06	0.04	1.51 [1.41-1.61]*	1.35 [1.26-1.44]*
I86	7,630	80,052,140	0.12	0.09	0.09	0.03	0.03	0.02	1.36 [1.26-1.47]*	1.22 [1.13-1.32]*
I87	16,050	80,056,380	0.29	0.18	0.18	0.11	0.11	0.09	1.61 [1.53-1.69]*	1.48 [1.40-1.55]*
I88	2,590	80,031,900	0.04	0.03	0.03	0.01	0.01	0.01	1.34 [1.18-1.52]*	1.22 [1.07-1.39]*
I89	21,540	80,031,070	0.38	0.25	0.25	0.13	0.13	0.10	1.52 [1.46-1.59]*	1.37 [1.31-1.44]*
I95	204,290	79,078,050	3.11	2.47	2.57	0.63	0.53	0.34	1.21 [1.19-1.23]*	1.12 [1.11-1.14]*
I97	2,570	80,121,490	0.04	0.03	0.03	0.01	0.01	0.01	1.34 [1.18-1.53]*	1.25 [1.09-1.43]*
I98	6,690	80,125,740	0.11	0.08	0.08	0.03	0.04	0.03	1.49 [1.38-1.61]*	1.31 [1.21-1.42]*
I99	3,790	80,136,630	0.06	0.05	0.05	0.01	0.01	0.01	1.22 [1.09-1.37]*	1.12 [1.00-1.26]
18+ - J00-J99										
J00	4,950	80,036,670	0.08	0.06	0.06	0.02	0.02	0.01	1.35 [1.23-1.48]*	1.22 [1.11-1.34]*
J01	2,620	80,115,540	0.04	0.03	0.03	0.01	0.01	0.01	1.38 [1.21-1.56]*	1.18 [1.03-1.35]
J02	15,040	79,862,920	0.25	0.18	0.18	0.07	0.07	0.05	1.39 [1.31-1.46]*	1.24 [1.17-1.31]*
J03	35,700	79,006,920	0.58	0.42	0.44	0.15	0.14	0.08	1.31 [1.27-1.36]*	1.17 [1.13-1.21]*
J04	2,280	80,128,880	0.04	0.03	0.03	0.01	0.01	0.01	1.47 [1.28-1.68]*	1.29 [1.12-1.48]*
J05	1,040	80,017,400	0.01	0.01	0.01	0.00	0.00	0.00	1.04 [0.84-1.28]	0.97 [0.78-1.21]
J06	16,240	79,271,580	0.27	0.19	0.19	0.08	0.07	0.04	1.38 [1.31-1.45]*	1.18 [1.12-1.25]*
J10	16,600	80,089,790	0.28	0.19	0.20	0.08	0.08	0.04	1.40 [1.33-1.47]*	1.19 [1.13-1.26]*
J11	3,580	80,116,630	0.06	0.04	0.04	0.02	0.01	0.01	1.34 [1.20-1.50]*	1.17 [1.04-1.32]*
J12	29,160	80,131,670	0.47	0.34	0.36	0.12	0.10	0.06	1.29 [1.23-1.34]*	1.15 [1.10-1.20]*
J13	6,450	80,108,650	0.10	0.08	0.08	0.03	0.03	0.02	1.37 [1.26-1.48]*	1.20 [1.10-1.31]*
J14	2,740	80,150,580	0.04	0.03	0.03	0.01	0.01	0.01	1.38 [1.21-1.56]*	1.14 [0.99-1.30]
J15	13,530	80,056,610	0.23	0.16	0.16	0.07	0.07	0.04	1.42 [1.34-1.50]*	1.24 [1.17-1.32]*
J17	2,800	80,151,940	0.05	0.03	0.03	0.02	0.02	0.01	1.49 [1.32-1.68]*	1.34 [1.18-1.51]*
J18	409,970	77,814,630	6.52	5.00	5.14	1.52	1.38	0.91	1.27 [1.26-1.28]*	1.16 [1.15-1.18]*
J20	4,980	80,109,720	0.08	0.06	0.06	0.02	0.02	0.01	1.28 [1.16-1.41]*	1.12 [1.02-1.24]
J21	1,320	79,993,820	0.02	0.02	0.02	0.01	0.01	0.00	1.46 [1.22-1.75]*	1.21 [1.00-1.46]
J22	233,090	77,686,310	3.86	2.81	2.86	1.05	1.00	0.67	1.35 [1.33-1.37]*	1.21 [1.19-1.23]*
J30	37,490	79,709,390	0.75	0.41	0.42	0.34	0.32	0.18	1.76 [1.71-1.82]*	1.32 [1.27-1.36]*
J31	9,050	79,977,450	0.17	0.10	0.10	0.07	0.07	0.04	1.70 [1.59-1.81]*	1.33 [1.24-1.43]*
J32	24,430	79,663,000	0.41	0.28	0.28	0.13	0.13	0.07	1.46 [1.40-1.52]*	1.20 [1.15-1.26]*
J33	17,240	79,649,340	0.30	0.20	0.19	0.10	0.11	0.06	1.54 [1.47-1.62]*	1.25 [1.19-1.32]*
J34	49,820	78,477,910	0.87	0.58	0.58	0.29	0.29	0.19	1.51 [1.46-1.55]*	1.29 [1.25-1.33]*
J35	17,950	78,443,410	0.29	0.22	0.22	0.07	0.07	0.04	1.30 [1.24-1.36]*	1.17 [1.11-1.23]*
J36	9,820	79,932,360	0.14	0.12	0.12	0.02	0.02	0.01	1.16 [1.08-1.24]*	1.11 [1.03-1.19]*
J37	550	80,154,210	0.01	0.01	0.01	0.00	0.00	0.00	1.40 [1.07-1.84]	1.15 [0.86-1.53]
J38	18,780	79,836,750	0.29	0.22	0.22	0.07	0.07	0.04	1.31 [1.25-1.38]*	1.15 [1.10-1.21]*
J39	9,340	80,035,450	0.15	0.11	0.11	0.04	0.04	0.02	1.34 [1.25-1.44]*	1.18 [1.10-1.26]*
J40	11,790	80,016,140	0.20	0.14	0.14	0.06	0.06	0.04	1.44 [1.36-1.53]*	1.23 [1.16-1.31]*
J41	580	80,165,720	0.01	0.01	0.01	0.00	0.00	0.00	1.38 [1.05-1.81]	1.18 [0.88-1.57]
J42	4,760	80,108,380	0.08	0.06	0.06	0.02	0.02	0.01	1.42 [1.29-1.56]*	1.19 [1.08-1.32]*
J43	65,760	79,737,350	1.06	0.77	0.78	0.28	0.28	0.16	1.35 [1.32-1.39]*	1.18 [1.15-1.21]*
J44	262,180	77,840,340	4.57	3.11	3.14	1.46	1.43	0.77	1.45 [1.44-1.47]*	1.20 [1.19-1.22]*
J45	392,250	70,400,710	9.41	4.74	4.76	4.67	4.65	2.40	1.98 [1.96-2.00]*	1.34 [1.33-1.36]*
J46	4,510	79,914,140	0.11	0.04	0.04	0.07	0.07	0.04	2.46 [2.25-2.68]*	1.55 [1.40-1.72]*
J47	47,260	79,830,350	0.83	0.54	0.55	0.29	0.28	0.17	1.51 [1.47-1.56]*	1.27 [1.23-1.31]*
J61	6,540	80,134,890	0.11	0.08	0.08	0.03	0.03	0.02	1.38 [1.27-1.50]*	1.27 [1.16-1.38]*
J67	1,920	80,158,270	0.03	0.02	0.02	0.01	0.01	0.01	1.60 [1.38-1.85]*	1.38 [1.19-1.61]*
J69	63,850	80,020,380	0.94	0.77	0.80	0.18	0.14	0.10	1.18 [1.14-1.21]*	1.12 [1.09-1.16]*
J70	1,300	80,166,240	0.02	0.02	0.02	0.01	0.01	0.00	1.44 [1.21-1.73]*	1.27 [1.06-1.53]
J80	4,870	80,140,330	0.08	0.06	0.06	0.02	0.02	0.02	1.40 [1.27-1.54]*	1.27 [1.15-1.40]*
J81	22,160	80,027,380	0.34	0.26	0.27	0.08	0.08	0.05	1.29 [1.23-1.35]*	1.18 [1.13-1.24]*
J82	1,700	80,162,140	0.04	0.02	0.02	0.02	0.02	0.01	2.31 [2.00-2.68]*	1.55 [1.31-1.82]*
J84	40,930	79,988,140	0.70	0.47	0.48	0.23	0.22	0.18	1.45 [1.41-1.50]*	1.34 [1.29-1.38]*
J85	2,090	80,155,860	0.03	0.02	0.02	0.01	0.01	0.01	1.37 [1.19-1.59]*	1.23 [1.06-1.43]*
J86	6,930	80,102,480	0.10	0.08	0.08	0.02	0.02	0.01	1.24 [1.14-1.35]*	1.16 [1.07-1.27]*
J90	207,560	79,167,580	3.09	2.52	2.59	0.57	0.50	0.33	1.19 [1.17-1.21]*	1.12 [1.10-1.14]*
J91	4,960	80,153,760	0.07	0.06	0.06	0.02	0.01	0.01	1.25 [1.13-1.38]*	1.17 [1.06-1.30]*
J92	23,420	80,056,920	0.36	0.28	0.28	0.08	0.08	0.06	1.28 [1.22-1.34]*	1.21 [1.15-1.26]*
J93	19,220	79,965,420	0.27	0.23	0.24	0.04	0.03	0.02	1.14 [1.08-1.20]*	1.07 [1.01-1.12]
J94	7,760	80,126,590	0.11	0.09	0.10	0.02	0.02	0.01	1.18 [1.09-1.28]*	1.10 [1.02-1.19]
J95	6,050	80,092,970	0.09	0.07	0.07	0.02	0.01	0.01	1.20 [1.10-1.31]*	1.11 [1.02-1.22]
J96	132,870	79,708,820	2.11	1.57	1.61	0.54	0.50	0.30	1.31 [1.29-1.34]*	1.17 [1.14-1.19]*
J98	121,570	79,447,590	1.85	1.46	1.50	0.39	0.36	0.19	1.24 [1.22-1.26]*	1.12 [1.09-1.14]*
J99	3,720	80,142,050	0.07	0.04	0.04	0.03	0.03	0.02	1.66 [1.50-1.83]*	1.41 [1.27-1.57]*
18+ - K00-K95										
K00	6,740	79,806,690	0.10	0.08	0.08	0.01	0.01	0.00	1.15 [1.06-1.25]*	1.05 [0.96-1.14]
K01	43,140	78,500,620	0.66	0.53	0.54	0.14	0.12	0.08	1.23 [1.19-1.27]*	1.13 [1.10-1.17]*
K02	81,570	77,772,280	1.27	1.00	1.02	0.27	0.25	0.14	1.25 [1.22-1.28]*	1.12 [1.10-1.15]*
K03	2,060	80,122,560	0.03	0.02	0.02	0.01	0.01	0.01	1.46 [1.27-1.68]*	1.34 [1.16-1.56]*
K04	27,810	79,509,460	0.41	0.34	0.34	0.08	0.07	0.04	1.22 [1.17-1.27]*	1.10 [1.05-1.15]*
K05	34,970	79,511,180	0.57	0.41	0.43	0.15	0.14	0.09	1.33 [1.28-1.38]*	1.20 [1.15-1.24]*
K06	4,150	80,104,060	0.07	0.05	0.05	0.02	0.02	0.01	1.37 [1.23-1.51]*	1.27 [1.15-1.42]*
K07	17,780	79,453,790	0.27	0.21	0.22	0.05	0.05	0.03	1.22 [1.16-1.28]*	1.11 [1.05-1.17]*
K08	42,180	79,275,510	0.65	0.51	0.52	0.14	0.13	0.08	1.25 [1.21-1.29]*	1.14 [1.10-1.17]*
K09	3,540	80,078,270	0.05	0.04	0.04	0.01	0.01	0.01	1.26 [1.13-1.41]*	1.18 [1.05-1.33]*
K10	5,110	80,103,760	0.08	0.06	0.06	0.02	0.02	0.01	1.27 [1.15-1.39]*	1.11 [1.01-1.22]
K11	10,620	79,961,330	0.17	0.12	0.12	0.04	0.04	0.03	1.36 [1.28-1.45]*	1.25 [1.17-1.33]*
K12	15,950	79,997,940	0.25	0.19	0.19	0.07	0.06	0.05	1.33 [1.26-1.40]*	1.22 [1.16-1.29]*
K13	22,550	79,730,660	0.37	0.26	0.27	0.10	0.10	0.08	1.38 [1.32-1.44]*	1.26 [1.21-1.32]*
K14	11,970	79,984,680	0.19	0.14	0.14	0.05	0.06	0.04	1.40 [1.32-1.49]*	1.24 [1.17-1.32]*
K20	79,860	78,715,280	1.27	0.96	0.96	0.31	0.31	0.19	1.32 [1.29-1.36]*	1.18 [1.15-1.20]*
K21	307,650	76,400,580	5.22	3.77	3.82	1.45	1.39	0.79	1.36 [1.35-1.38]*	1.18 [1.17-1.19]*
K22	118,410	78,566,380	1.86	1.43	1.43	0.43	0.43	0.28	1.30 [1.27-1.32]*	1.18 [1.16-1.20]*
K23	1,410	80,162,610	0.02	0.02	0.02	0.01	0.01	0.00	1.50 [1.26-1.78]*	1.23 [1.02-1.47]
K25	58,620	79,335,080	0.91	0.70	0.70	0.21	0.21	0.13	1.30 [1.26-1.33]*	1.17 [1.14-1.20]*
K26	42,110	79,545,760	0.62	0.51	0.51	0.11	0.11	0.07	1.21 [1.17-1.25]*	1.14 [1.10-1.18]*
K27	7,160	80,071,040	0.11	0.08	0.09	0.03	0.02	0.01	1.28 [1.19-1.39]*	1.13 [1.04-1.22]*
K28	1,020	80,160,630	0.02	0.01	0.01	0.01	0.01	0.00	1.46 [1.19-1.79]*	1.19 [0.96-1.47]
K29	294,590	75,378,480	4.97	3.68	3.74	1.29	1.24	0.78	1.33 [1.31-1.35]*	1.19 [1.17-1.20]*
K30	73,260	78,333,000	1.19	0.88	0.88	0.31	0.31	0.20	1.36 [1.33-1.39]*	1.20 [1.17-1.23]*
K31	100,920	79,149,690	1.60	1.21	1.22	0.39	0.38	0.21	1.31 [1.28-1.33]*	1.15 [1.12-1.17]*
K35	40,380	78,970,850	0.55	0.50	0.50	0.05	0.05	0.02	1.09 [1.05-1.13]*	1.03 [1.00-1.07]
K36	1,000	80,147,490	0.01	0.01	0.01	0.00	0.00	0.00	1.20 [0.97-1.48]	1.11 [0.89-1.38]
K37	7,580	79,951,510	0.11	0.09	0.09	0.01	0.01	0.00	1.13 [1.04-1.22]*	1.01 [0.93-1.10]
K38	5,800	80,057,190	0.08	0.07	0.07	0.01	0.01	0.00	1.16 [1.06-1.27]*	1.06 [0.97-1.16]
K40	109,720	77,749,940	1.54	1.38	1.39	0.16	0.16	0.11	1.11 [1.09-1.14]*	1.08 [1.06-1.10]*
K41	8,290	80,037,240	0.12	0.10	0.10	0.02	0.02	0.01	1.18 [1.09-1.27]*	1.12 [1.04-1.21]*

K42	48,410	79,359,750	0.75	0.58	0.58	0.17	0.17	0.09	1.29 [1.26-1.33]*	1.14 [1.11-1.18]*
K43	39,560	79,538,540	0.60	0.47	0.47	0.13	0.13	0.07	1.27 [1.23-1.32]*	1.14 [1.10-1.18]*
K44	294,760	75,772,100	4.89	3.67	3.71	1.22	1.18	0.73	1.32 [1.30-1.33]*	1.17 [1.16-1.19]*
K45	1,920	80,149,920	0.03	0.02	0.02	0.01	0.00	0.00	1.18 [1.01-1.38]	1.04 [0.88-1.22]
K46	7,780	80,113,930	0.12	0.09	0.09	0.03	0.03	0.02	1.34 [1.24-1.45]*	1.16 [1.07-1.25]*
K50	20,710	79,672,650	0.38	0.23	0.23	0.15	0.15	0.10	1.65 [1.58-1.72]*	1.35 [1.29-1.42]*
K51	30,970	79,504,500	0.54	0.36	0.36	0.18	0.18	0.12	1.51 [1.46-1.57]*	1.29 [1.24-1.34]*
K52	155,430	76,793,940	2.60	1.90	1.88	0.71	0.73	0.51	1.39 [1.36-1.41]*	1.24 [1.22-1.26]*
K55	25,690	79,993,560	0.39	0.31	0.31	0.08	0.08	0.05	1.25 [1.20-1.30]*	1.14 [1.10-1.20]*
K56	76,910	79,455,890	1.15	0.93	0.95	0.22	0.20	0.12	1.21 [1.18-1.24]*	1.11 [1.09-1.14]*
K57	337,950	76,374,500	5.37	4.22	4.30	1.15	1.07	0.65	1.25 [1.23-1.26]*	1.14 [1.12-1.15]*
K58	94,390	78,979,430	1.69	1.09	1.11	0.60	0.58	0.27	1.52 [1.49-1.55]*	1.19 [1.17-1.22]*
K59	294,580	77,471,250	4.78	3.59	3.71	1.19	1.07	0.69	1.29 [1.27-1.30]*	1.17 [1.15-1.18]*
K60	27,110	79,475,140	0.48	0.31	0.31	0.16	0.17	0.13	1.54 [1.48-1.60]*	1.38 [1.32-1.43]*
K61	16,990	79,752,570	0.27	0.20	0.20	0.07	0.08	0.06	1.39 [1.33-1.46]*	1.26 [1.19-1.32]*
K62	173,220	76,810,080	2.83	2.13	2.12	0.70	0.71	0.49	1.34 [1.31-1.36]*	1.21 [1.19-1.23]*
K63	176,740	78,390,690	2.74	2.15	2.16	0.60	0.58	0.34	1.27 [1.25-1.29]*	1.14 [1.12-1.16]*
K64	125,380	79,678,900	2.00	1.48	1.53	0.52	0.48	0.30	1.31 [1.29-1.34]*	1.18 [1.15-1.20]*
K65	18,740	80,011,490	0.27	0.23	0.23	0.04	0.04	0.02	1.18 [1.12-1.24]*	1.10 [1.05-1.16]*
K66	66,890	79,435,870	0.99	0.81	0.82	0.18	0.16	0.08	1.20 [1.17-1.23]*	1.09 [1.06-1.12]*
K70	22,350	79,924,270	0.37	0.26	0.25	0.11	0.12	0.08	1.46 [1.40-1.52]*	1.29 [1.23-1.35]*
K71	1,400	80,156,260	0.02	0.02	0.02	0.01	0.01	0.01	1.40 [1.17-1.67]*	1.28 [1.07-1.54]*
K72	13,970	80,118,260	0.22	0.16	0.16	0.06	0.06	0.04	1.35 [1.28-1.43]*	1.22 [1.15-1.29]*
K73	1,310	80,143,630	0.02	0.02	0.01	0.01	0.01	0.01	1.55 [1.30-1.84]*	1.41 [1.18-1.69]*
K74	20,660	80,017,650	0.35	0.24	0.24	0.12	0.12	0.08	1.49 [1.42-1.55]*	1.31 [1.25-1.37]*
K75	13,480	80,048,610	0.22	0.16	0.16	0.07	0.07	0.05	1.42 [1.34-1.50]*	1.26 [1.19-1.33]*
K76	99,490	79,488,170	1.60	1.18	1.20	0.42	0.40	0.23	1.34 [1.31-1.36]*	1.17 [1.14-1.19]*
K80	190,040	77,339,500	2.83	2.38	2.43	0.45	0.41	0.20	1.17 [1.15-1.19]*	1.08 [1.06-1.09]*
K81	34,900	79,627,020	0.51	0.42	0.43	0.09	0.08	0.04	1.19 [1.15-1.24]*	1.09 [1.05-1.13]*
K82	26,510	79,826,840	0.39	0.32	0.33	0.07	0.06	0.03	1.19 [1.14-1.24]*	1.09 [1.04-1.14]*
K83	49,650	79,717,100	0.72	0.60	0.62	0.12	0.10	0.05	1.17 [1.13-1.20]*	1.08 [1.05-1.12]*
K85	32,930	79,701,870	0.49	0.40	0.40	0.09	0.09	0.05	1.21 [1.17-1.26]*	1.11 [1.07-1.15]*
K86	27,360	79,934,090	0.41	0.33	0.33	0.08	0.08	0.04	1.24 [1.19-1.29]*	1.12 [1.07-1.17]*
K90	20,980	79,831,340	0.39	0.24	0.24	0.15	0.14	0.10	1.60 [1.53-1.67]*	1.36 [1.30-1.42]*
K91	24,040	79,877,100	0.38	0.28	0.29	0.09	0.09	0.06	1.32 [1.26-1.37]*	1.18 [1.13-1.23]*
K92	216,660	78,072,810	3.45	2.63	2.66	0.82	0.79	0.51	1.30 [1.28-1.31]*	1.17 [1.16-1.19]*
18+ - L00-L99										
L01	1,070	80,125,110	0.03	0.01	0.01	0.02	0.02	0.01	2.65 [2.21-3.17]*	2.19 [1.81-2.65]*
L02	42,440	79,323,530	0.70	0.50	0.50	0.19	0.19	0.13	1.38 [1.34-1.43]*	1.23 [1.19-1.27]*
L03	160,820	78,260,380	3.02	1.85	1.83	1.17	1.18	1.01	1.64 [1.62-1.67]*	1.50 [1.48-1.53]*
L04	1,280	80,128,380	0.02	0.02	0.02	0.00	0.00	0.00	1.29 [1.07-1.55]*	1.21 [1.00-1.46]
L05	15,430	79,764,450	0.22	0.19	0.19	0.03	0.03	0.02	1.17 [1.11-1.24]*	1.08 [1.02-1.14]*
L08	42,770	79,792,010	0.75	0.49	0.50	0.26	0.25	0.21	1.51 [1.46-1.56]*	1.38 [1.34-1.43]*
L10	550	80,166,890	0.01	0.01	0.01	0.01	0.01	0.01	2.25 [1.74-2.92]*	2.06 [1.57-2.69]*
L11	450	80,166,650	0.01	0.01	0.01	0.00	0.00	0.00	1.64 [1.21-2.22]*	1.56 [1.15-2.13]*
L12	3,760	80,149,530	0.10	0.04	0.04	0.06	0.06	0.06	2.81 [2.54-3.11]*	2.65 [2.39-2.93]*
L13	620	80,162,910	0.02	0.01	0.01	0.01	0.01	0.01	3.31 [2.62-4.18]*	2.99 [2.35-3.79]*
L20	3,160	80,035,160	0.20	0.01	0.01	0.19	0.19	0.19	31.99 [27.28-37.50]*	27.25 [23.19-32.01]*
L21	1,700	80,150,390	0.05	0.02	0.02	0.03	0.03	0.03	3.13 [2.71-3.62]*	2.85 [2.45-3.30]*
L22	800	80,163,560	0.01	0.01	0.01	0.00	0.00	0.00	1.08 [0.83-1.40]	1.02 [0.79-1.33]
L23	1,510	80,135,300	0.05	0.01	0.01	0.03	0.03	0.03	3.56 [3.07-4.12]*	3.19 [2.74-3.71]*
L24	10,580	80,154,260	0.18	0.12	0.13	0.06	0.05	0.04	1.42 [1.32-1.52]*	1.31 [1.22-1.41]*
L25	1,780	80,147,250	0.05	0.02	0.02	0.04	0.04	0.03	3.18 [2.77-3.66]*	2.96 [2.57-3.41]*
L26	210	80,170,040	0.01	0.00	0.00	0.01	0.01	0.01	6.67 [4.43-10.04]*	5.73 [3.75-8.75]*
L27	9,290	80,059,650	0.17	0.10	0.11	0.07	0.06	0.05	1.60 [1.50-1.71]*	1.45 [1.36-1.56]*
L28	3,360	80,117,170	0.11	0.03	0.03	0.08	0.09	0.08	4.18 [3.79-4.61]*	3.82 [3.45-4.22]*
L29	15,850	79,897,580	0.31	0.18	0.18	0.13	0.12	0.10	1.68 [1.60-1.76]*	1.50 [1.42-1.58]*
L30	59,960	78,892,840	2.94	0.30	0.29	2.64	2.65	2.61	10.25 [9.99-10.52]*	9.13 [8.89-9.38]*
L40	41,930	79,691,250	0.96	0.43	0.44	0.52	0.52	0.48	2.19 [2.13-2.26]*	2.02 [1.96-2.08]*
L42	620	80,165,640	0.01	0.01	0.01	0.00	0.00	0.00	1.63 [1.27-2.11]*	1.40 [1.07-1.83]
L43	4,530	80,097,550	0.10	0.05	0.05	0.05	0.05	0.05	2.15 [1.97-2.35]*	1.98 [1.81-2.17]*
L50	6,820	80,038,330	0.13	0.07	0.08	0.06	0.06	0.04	1.78 [1.65-1.92]*	1.45 [1.34-1.56]*
L51	1,340	80,134,920	0.03	0.01	0.01	0.01	0.01	0.01	1.83 [1.54-2.16]*	1.60 [1.35-1.91]*
L52	930	80,149,370	0.02	0.01	0.01	0.01	0.01	0.00	1.50 [1.22-1.84]*	1.33 [1.07-1.64]
L53	13,720	80,031,940	0.27	0.15	0.15	0.12	0.12	0.11	1.81 [1.71-1.91]*	1.65 [1.56-1.75]*
L56	560	80,163,460	0.01	0.01	0.01	0.01	0.01	0.01	2.85 [2.23-3.63]*	2.58 [2.01-3.31]*
L57	34,870	79,782,240	0.55	0.41	0.42	0.14	0.13	0.10	1.31 [1.26-1.35]*	1.22 [1.18-1.27]*
L59	470	80,166,960	0.01	0.01	0.01	0.00	0.00	0.00	1.44 [1.07-1.93]	1.41 [1.04-1.91]
L60	9,780	79,755,050	0.17	0.11	0.11	0.05	0.05	0.04	1.48 [1.39-1.58]*	1.33 [1.24-1.42]*
L63	610	80,163,440	0.02	0.01	0.01	0.01	0.01	0.01	2.84 [2.24-3.59]*	2.08 [1.61-2.68]*
L65	2,040	80,146,200	0.04	0.02	0.02	0.02	0.02	0.02	2.04 [1.78-2.33]*	1.73 [1.50-1.99]*
L66	440	80,165,240	0.01	0.00	0.00	0.00	0.00	0.00	1.93 [1.44-2.57]*	1.79 [1.33-2.42]*
L68	720	80,153,740	0.01	0.01	0.01	0.00	0.00	0.00	1.28 [1.00-1.64]	1.12 [0.87-1.45]
L70	2,600	80,136,020	0.04	0.03	0.03	0.01	0.01	0.01	1.42 [1.25-1.61]*	1.22 [1.07-1.39]*
L71	3,450	80,137,240	0.06	0.04	0.04	0.02	0.02	0.02	1.59 [1.43-1.78]*	1.49 [1.33-1.66]*
L72	49,960	78,687,070	0.71	0.62	0.61	0.10	0.10	0.06	1.17 [1.14-1.21]*	1.09 [1.06-1.12]*
L73	6,480	80,036,470	0.12	0.07	0.07	0.04	0.04	0.03	1.56 [1.44-1.68]*	1.35 [1.24-1.46]*
L80	1,410	80,156,560	0.03	0.02	0.02	0.01	0.01	0.01	1.85 [1.57-2.18]*	1.65 [1.40-1.96]*
L81	7,250	80,045,070	0.11	0.09	0.09	0.03	0.02	0.02	1.29 [1.19-1.39]*	1.20 [1.11-1.30]*
L82	23,170	79,791,410	0.35	0.28	0.28	0.07	0.07	0.05	1.25 [1.19-1.31]*	1.17 [1.12-1.22]*
L83	1,180	80,149,670	0.02	0.01	0.01	0.01	0.01	0.01	1.82 [1.51-2.18]*	1.60 [1.32-1.93]*
L84	2,500	80,129,300	0.04	0.03	0.03	0.01	0.01	0.01	1.47 [1.29-1.67]*	1.33 [1.16-1.51]*
L85	9,540	80,051,700	0.17	0.11	0.11	0.06	0.06	0.05	1.57 [1.47-1.68]*	1.46 [1.37-1.57]*
L88	560	80,165,350	0.01	0.01	0.01	0.00	0.00	0.00	1.68 [1.29-2.19]*	1.40 [1.07-1.85]
L89	121,240	79,869,450	1.84	1.45	1.52	0.40	0.32	0.24	1.21 [1.19-1.24]*	1.15 [1.13-1.18]*
L90	22,670	79,644,660	0.36	0.27	0.27	0.09	0.09	0.07	1.33 [1.28-1.39]*	1.24 [1.18-1.29]*
L91	7,100	79,940,370	0.11	0.08	0.08	0.03	0.03	0.02	1.35 [1.25-1.46]*	1.26 [1.16-1.36]*
L92	5,760	80,039,050	0.09	0.07	0.07	0.03	0.03	0.02	1.40 [1.28-1.52]*	1.26 [1.15-1.37]*
L93	2,660	80,135,060	0.05	0.03	0.03	0.03	0.03	0.02	1.86 [1.66-2.10]*	1.61 [1.42-1.82]*
L94	1,700	80,144,470	0.03	0.02	0.02	0.01	0.01	0.01	1.55 [1.33-1.81]*	1.41 [1.20-1.65]*
L95	2,200	80,144,190	0.04	0.02	0.02	0.02	0.02	0.02	1.78 [1.56-2.04]*	1.63 [1.42-1.87]*
L97	86,550	79,669,000	1.53	0.99	1.00	0.54	0.53	0.46	1.53 [1.50-1.57]*	1.42 [1.39-1.46]*
L98	68,620	79,064,650	1.13	0.81	0.81	0.32	0.32	0.26	1.40 [1.36-1.44]*	1.30 [1.27-1.33]*
18+ - M00-M99										
M00	7,860	80,065,920	0.13	0.09	0.09	0.04	0.04	0.04	1.50 [1.39-1.61]*	1.37 [1.27-1.48]*
M02	1,200	80,149,530	0.02	0.01	0.01	0.01	0.01	0.00	1.42 [1.18-1.72]*	1.29 [1.06-1.57]
M05	10,240	80,043,880	0.17	0.12	0.12	0.05	0.05	0.04	1.39 [1.30-1.48]*	1.30 [1.22-1.39]*
M06	74,630	79,237,340	1.24	0.88	0.89	0.36	0.35	0.26	1.40 [1.36-1.43]*	1.27 [1.23-1.30]*
M07	8,760	80,059,300	0.17	0.10	0.10	0.07	0.07	0.06	1.70 [1.59-1.82]*	1.51 [1.41-1.61]*
M08	780	80,126,530	0.01	0.01	0.01	0.00	0.00	0.00	1.38 [1.09-1.73]*	1.26 [1.00-1.60]
M10	84,450	79,550,070	1.36	1.00	1.00	0.36	0.36	0.26	1.36 [1.33-1.39]*	1.24 [1.21-1.27]*
M11	9,430	80,089,630	0.15	0.11	0.12	0.04	0.03	0.02	1.30 [1.21-1.40]*	1.19 [1.11-1.28]*
M12	1,950	80,142,740	0.03	0.02	0.02	0.01	0.01	0.00	1.33 [1.15-1.54]*	1.17 [1.01-1.37]
M13	212,780	78,075,530	3.49	2.56	2.63	0.94	0.87	0.56	1.33 [1.31-1.35]*	1.19 [1.17-1.21]*

M14	2,200	80,146,590	0.04	0.02	0.02	0.01	0.02	0.01	1.62 [1.42-1.86]*	1.33 [1.16-1.54]*
M15	78,160	79,437,580	1.28	0.92	0.93	0.36	0.35	0.23	1.37 [1.34-1.41]*	1.22 [1.19-1.25]*
M16	131,110	78,280,840	1.86	1.63	1.66	0.22	0.20	0.12	1.12 [1.10-1.14]*	1.07 [1.05-1.09]*
M17	211,460	76,849,020	3.33	2.63	2.66	0.71	0.67	0.45	1.25 [1.23-1.27]*	1.16 [1.14-1.18]*
M18	11,580	80,019,170	0.19	0.13	0.14	0.06	0.06	0.03	1.42 [1.33-1.50]*	1.22 [1.14-1.29]*
M19	329,730	77,326,230	5.56	3.99	4.08	1.57	1.47	0.98	1.36 [1.34-1.38]*	1.21 [1.20-1.23]*
M20	47,520	79,034,860	0.74	0.57	0.57	0.17	0.17	0.12	1.30 [1.26-1.34]*	1.20 [1.16-1.24]*
M21	25,190	79,820,250	0.38	0.30	0.31	0.08	0.07	0.05	1.24 [1.19-1.29]*	1.14 [1.09-1.19]*
M22	8,590	79,905,460	0.14	0.10	0.10	0.03	0.03	0.02	1.35 [1.26-1.44]*	1.18 [1.10-1.27]*
M23	93,680	77,788,550	1.40	1.16	1.14	0.24	0.26	0.16	1.22 [1.20-1.25]*	1.13 [1.10-1.15]*
M24	34,170	79,538,560	0.53	0.41	0.41	0.12	0.12	0.07	1.29 [1.24-1.33]*	1.16 [1.11-1.20]*
M25	206,140	77,261,860	3.34	2.52	2.57	0.81	0.77	0.48	1.30 [1.28-1.32]*	1.17 [1.15-1.18]*
M30	910	80,152,640	0.02	0.01	0.01	0.01	0.01	0.00	1.47 [1.20-1.82]*	1.23 [0.99-1.53]
M31	11,820	80,051,160	0.19	0.14	0.14	0.05	0.05	0.04	1.34 [1.26-1.43]*	1.24 [1.16-1.32]*
M32	5,490	80,075,430	0.11	0.06	0.06	0.05	0.05	0.04	1.75 [1.61-1.90]*	1.52 [1.39-1.65]*
M33	1,140	80,154,720	0.02	0.01	0.01	0.01	0.01	0.01	2.10 [1.76-2.50]*	1.98 [1.65-2.37]*
M34	2,190	80,141,850	0.04	0.02	0.02	0.02	0.02	0.01	1.66 [1.45-1.89]*	1.49 [1.30-1.71]*
M35	54,420	79,679,330	0.93	0.63	0.64	0.30	0.28	0.22	1.44 [1.40-1.49]*	1.31 [1.27-1.34]*
M36	450	80,164,480	0.01	0.01	0.01	0.00	0.00	0.00	1.41 [1.03-1.93]	1.27 [0.92-1.76]
M40	13,340	80,101,810	0.20	0.16	0.16	0.05	0.04	0.02	1.24 [1.17-1.32]*	1.14 [1.07-1.21]*
M41	34,460	79,855,970	0.53	0.41	0.42	0.12	0.11	0.07	1.27 [1.23-1.32]*	1.16 [1.12-1.21]*
M42	690	80,163,660	0.01	0.01	0.01	0.00	0.00	0.00	1.22 [0.94-1.57]	1.01 [0.77-1.32]
M43	29,950	79,837,360	0.47	0.36	0.36	0.11	0.11	0.06	1.29 [1.24-1.34]*	1.14 [1.09-1.19]*
M45	7,210	80,067,950	0.13	0.08	0.08	0.04	0.05	0.04	1.56 [1.45-1.69]*	1.39 [1.29-1.50]*
M46	24,580	79,925,120	0.41	0.29	0.29	0.12	0.12	0.07	1.41 [1.36-1.48]*	1.22 [1.17-1.28]*
M47	140,280	78,738,700	2.30	1.67	1.69	0.63	0.61	0.37	1.36 [1.34-1.38]*	1.19 [1.17-1.21]*
M48	82,570	79,423,300	1.29	0.98	1.00	0.31	0.29	0.17	1.29 [1.26-1.32]*	1.15 [1.12-1.17]*
M49	2,980	80,158,680	0.04	0.04	0.04	0.00	0.00	0.00	1.07 [0.94-1.21]	1.03 [0.90-1.17]
M50	21,750	79,927,330	0.34	0.26	0.26	0.09	0.08	0.04	1.32 [1.26-1.38]*	1.14 [1.09-1.20]*
M51	105,430	78,739,650	1.66	1.27	1.28	0.40	0.39	0.19	1.30 [1.27-1.33]*	1.13 [1.11-1.15]*
M53	6,410	80,067,990	0.10	0.08	0.08	0.02	0.02	0.01	1.27 [1.17-1.38]*	1.08 [0.99-1.18]
M54	258,750	76,320,770	4.25	3.20	3.28	1.05	0.98	0.50	1.30 [1.28-1.32]*	1.13 [1.12-1.15]*
M60	4,300	80,113,880	0.07	0.05	0.05	0.02	0.02	0.02	1.48 [1.34-1.63]*	1.36 [1.23-1.51]*
M62	20,940	79,964,450	0.32	0.25	0.25	0.07	0.07	0.04	1.26 [1.20-1.33]*	1.16 [1.10-1.22]*
M65	42,540	79,378,090	0.68	0.50	0.50	0.18	0.18	0.12	1.35 [1.31-1.39]*	1.21 [1.17-1.25]*
M66	4,600	80,091,610	0.07	0.05	0.05	0.02	0.02	0.01	1.31 [1.18-1.44]*	1.21 [1.09-1.34]*
M67	28,010	79,452,230	0.43	0.34	0.33	0.10	0.10	0.07	1.30 [1.25-1.35]*	1.19 [1.14-1.24]*
M70	17,700	79,888,590	0.30	0.21	0.20	0.09	0.09	0.06	1.44 [1.37-1.52]*	1.28 [1.21-1.34]*
M71	11,350	80,028,530	0.18	0.13	0.14	0.05	0.04	0.03	1.33 [1.25-1.41]*	1.20 [1.13-1.28]*
M72	25,760	79,685,080	0.35	0.32	0.31	0.03	0.04	0.02	1.12 [1.07-1.17]*	1.07 [1.02-1.11]*
M75	72,230	78,880,850	1.15	0.87	0.87	0.28	0.28	0.16	1.32 [1.29-1.35]*	1.17 [1.14-1.20]*
M76	5,820	80,094,420	0.10	0.07	0.07	0.03	0.03	0.02	1.41 [1.29-1.53]*	1.23 [1.12-1.34]*
M77	14,060	79,903,840	0.24	0.16	0.16	0.07	0.08	0.05	1.48 [1.40-1.56]*	1.29 [1.22-1.36]*
M79	209,720	77,215,060	3.61	2.52	2.57	1.09	1.04	0.66	1.41 [1.39-1.43]*	1.22 [1.21-1.24]*
M80	26,210	79,976,240	0.41	0.31	0.32	0.10	0.09	0.06	1.29 [1.23-1.34]*	1.18 [1.13-1.23]*
M81	159,490	78,992,300	2.57	1.90	1.98	0.67	0.59	0.38	1.30 [1.28-1.32]*	1.17 [1.15-1.19]*
M83	1,040	80,158,720	0.02	0.01	0.01	0.00	0.00	0.00	1.23 [1.00-1.52]	1.13 [0.91-1.39]
M84	21,980	79,809,540	0.33	0.26	0.26	0.07	0.07	0.04	1.25 [1.19-1.31]*	1.14 [1.09-1.20]*
M85	34,610	79,961,330	0.56	0.41	0.42	0.15	0.13	0.08	1.32 [1.27-1.37]*	1.17 [1.13-1.22]*
M86	14,680	80,015,070	0.24	0.17	0.17	0.06	0.07	0.05	1.38 [1.31-1.46]*	1.25 [1.18-1.32]*
M87	6,690	80,072,680	0.10	0.08	0.08	0.02	0.02	0.01	1.27 [1.17-1.38]*	1.16 [1.07-1.26]*
M88	4,480	80,098,880	0.06	0.05	0.05	0.01	0.01	0.01	1.15 [1.03-1.28]	1.11 [0.99-1.24]
M89	16,290	79,858,610	0.26	0.19	0.20	0.06	0.06	0.04	1.30 [1.24-1.37]*	1.17 [1.11-1.24]*
M90	7,220	80,148,000	0.09	0.09	0.09	0.00	0.00	0.00	1.03 [0.95-1.13]	1.02 [0.94-1.11]
M91	700	80,143,080	0.01	0.01	0.01	0.00	0.00	0.00	1.27 [0.99-1.64]	1.21 [0.93-1.56]
M92	1,740	80,130,940	0.03	0.02	0.02	0.01	0.01	0.01	1.43 [1.22-1.67]*	1.27 [1.08-1.49]*
M93	3,450	80,070,960	0.05	0.04	0.04	0.01	0.01	0.01	1.30 [1.16-1.46]*	1.15 [1.02-1.29]
M94	12,150	79,985,750	0.20	0.14	0.14	0.06	0.06	0.04	1.41 [1.33-1.50]*	1.21 [1.14-1.28]*
M95	3,020	80,069,300	0.05	0.04	0.03	0.01	0.01	0.01	1.34 [1.19-1.51]*	1.20 [1.06-1.35]*
M96	8,700	80,087,970	0.14	0.10	0.11	0.04	0.03	0.02	1.31 [1.21-1.41]*	1.18 [1.10-1.28]*
M99	2,480	80,151,010	0.04	0.03	0.03	0.01	0.01	0.00	1.32 [1.15-1.51]*	1.13 [0.98-1.30]
18+ - N00-N99										
N02	6,690	80,036,670	0.11	0.08	0.08	0.03	0.03	0.02	1.36 [1.25-1.47]*	1.28 [1.17-1.38]*
N03	26,900	79,962,920	0.43	0.32	0.32	0.11	0.11	0.08	1.34 [1.29-1.40]*	1.22 [1.17-1.27]*
N04	3,680	80,110,780	0.06	0.04	0.04	0.02	0.02	0.02	1.50 [1.35-1.67]*	1.37 [1.23-1.52]*
N05	3,720	80,105,790	0.06	0.04	0.04	0.02	0.02	0.02	1.49 [1.34-1.66]*	1.36 [1.22-1.51]*
N08	12,160	80,066,520	0.21	0.14	0.14	0.06	0.06	0.04	1.45 [1.36-1.53]*	1.26 [1.18-1.33]*
N10	5,120	80,082,160	0.07	0.06	0.06	0.01	0.01	0.00	1.18 [1.07-1.29]*	1.04 [0.94-1.15]
N11	2,520	80,122,870	0.04	0.03	0.03	0.01	0.01	0.00	1.24 [1.08-1.41]*	1.14 [1.00-1.31]
N12	29,140	79,734,820	0.43	0.35	0.36	0.08	0.07	0.03	1.21 [1.16-1.25]*	1.07 [1.03-1.12]*
N13	64,780	79,598,450	0.87	0.80	0.81	0.07	0.06	0.03	1.07 [1.04-1.10]*	1.03 [1.01-1.06]
N14	930	80,166,550	0.01	0.01	0.01	0.00	0.00	0.00	1.31 [1.05-1.63]	1.20 [0.96-1.51]
N15	1,670	80,153,520	0.02	0.02	0.02	0.00	0.00	0.00	1.21 [1.03-1.43]	1.08 [0.91-1.29]
N16	820	80,162,670	0.01	0.01	0.01	0.01	0.01	0.01	1.65 [1.32-2.06]*	1.51 [1.20-1.89]*
N17	356,820	78,959,680	5.47	4.32	4.48	1.15	0.99	0.65	1.22 [1.21-1.23]*	1.13 [1.12-1.15]*
N18	299,100	78,798,540	4.66	3.61	3.70	1.06	0.96	0.62	1.26 [1.24-1.28]*	1.15 [1.14-1.17]*
N19	43,440	79,796,710	0.68	0.52	0.51	0.16	0.16	0.12	1.31 [1.27-1.36]*	1.21 [1.17-1.25]*
N20	54,130	79,281,100	0.77	0.66	0.67	0.10	0.10	0.05	1.15 [1.12-1.19]*	1.07 [1.04-1.10]*
N21	13,440	80,001,560	0.17	0.17	0.17	0.00	0.00	0.00	1.01 [0.95-1.08]	0.98 [0.92-1.05]
N23	12,520	79,789,240	0.18	0.15	0.15	0.03	0.03	0.01	1.19 [1.12-1.26]*	1.06 [1.00-1.13]
N25	2,500	80,144,100	0.04	0.03	0.03	0.01	0.01	0.01	1.39 [1.22-1.59]*	1.29 [1.12-1.48]*
N26	4,990	80,141,950	0.07	0.06	0.06	0.01	0.01	0.01	1.18 [1.07-1.30]*	1.07 [0.97-1.19]
N27	2,460	80,144,840	0.04	0.03	0.03	0.01	0.01	0.01	1.34 [1.17-1.53]*	1.25 [1.09-1.43]*
N28	71,180	79,625,890	1.05	0.86	0.88	0.19	0.17	0.09	1.19 [1.16-1.22]*	1.10 [1.07-1.13]*
N29	520	80,165,150	0.01	0.01	0.01	0.00	0.00	0.00	1.48 [1.11-1.97]*	1.35 [1.01-1.82]
N30	33,380	79,610,190	0.51	0.40	0.40	0.11	0.11	0.07	1.27 [1.22-1.31]*	1.17 [1.12-1.21]*
N31	9,300	79,955,110	0.15	0.11	0.11	0.04	0.04	0.03	1.39 [1.30-1.48]*	1.20 [1.12-1.28]*
N32	96,620	78,669,860	1.47	1.18	1.20	0.29	0.27	0.17	1.22 [1.20-1.25]*	1.13 [1.10-1.16]*
N34	1,460	80,134,840	0.02	0.02	0.02	0.01	0.01	0.00	1.34 [1.13-1.59]*	1.25 [1.05-1.49]
N35	32,380	79,524,340	0.51	0.39	0.39	0.12	0.12	0.09	1.31 [1.26-1.36]*	1.21 [1.16-1.26]*
N36	8,810	80,010,340	0.13	0.10	0.10	0.03	0.03	0.02	1.29 [1.20-1.38]*	1.21 [1.12-1.30]*
N39	423,010	75,628,220	6.71	5.35	5.59	1.37	1.13	0.71	1.20 [1.19-1.21]*	1.12 [1.11-1.13]*
N40	143,600	78,410,740	2.16	1.76	1.86	0.40	0.30	0.20	1.16 [1.14-1.18]*	1.10 [1.08-1.12]*
N41	10,600	79,986,600	0.14	0.13	0.13	0.01	0.02	0.01	1.14 [1.07-1.22]*	1.08 [1.00-1.15]
N42	13,660	79,962,430	0.19	0.17	0.17	0.02	0.02	0.01	1.11 [1.05-1.18]*	1.06 [1.00-1.13]
N43	13,510	79,848,640	0.19	0.16	0.16	0.03	0.03	0.03	1.21 [1.14-1.28]*	1.16 [1.09-1.23]*
N44	1,390	80,082,730	0.02	0.02	0.02	0.00	0.00	0.00	1.10 [0.92-1.32]	1.02 [0.85-1.24]
N45	12,340	79,930,080	0.18	0.15	0.15	0.04	0.04	0.02	1.26 [1.18-1.33]*	1.15 [1.08-1.22]*
N47	29,840	79,260,330	0.49	0.35	0.34	0.14	0.15	0.12	1.43 [1.38-1.48]*	1.32 [1.27-1.38]*
N48	20,130	79,733,710	0.33	0.24	0.23	0.09	0.10	0.08	1.44 [1.37-1.50]*	1.32 [1.26-1.39]*
N49	3,110	80,123,630	0.05	0.04	0.04	0.01	0.01	0.01	1.38 [1.23-1.56]*	1.27 [1.13-1.44]*
N50	24,000	79,606,140	0.35	0.29	0.28	0.06	0.07	0.05	1.25 [1.20-1.31]*	1.16 [1.11-1.21]*
N60	10,740	79,865,610	0.15	0.13	0.13	0.02	0.02	0.01	1.14 [1.07-1.22]*	1.09 [1.02-1.16]
N61	6,870	80,012,930	0.11	0.08	0.08	0.03	0.03	0.02	1.38 [1.27-1.49]*	1.24 [1.14-1.35]*
N62	5,790	79,967,830	0.09	0.07	0.07	0.02	0.02	0.01	1.28 [1.17-1.39]*	1.15 [1.05-1.26]*
N63	11,310	79,859,760	0.16	0.14	0.14	0.02	0.02	0.01	1.17 [1.09-1.24]*	1.09 [1.02-1.16]
N64	10,770	79,941,660	0.16	0.13	0.13	0.03	0.03	0.02	1.25 [1.17-1.33]*	1.15 [1.07-1.23]*
N70	7,730	79,983,400	0.11	0.09	0.10	0.01	0.01	0.00	1.08 [1.00-1.17]	1.01 [0.93-1.09]

N71	3,730	80,084,160	0.06	0.04	0.05	0.01	0.01	0.01	1.23 [1.10-1.37]*	1.12 [1.01-1.26]
N72	7,410	79,893,680	0.10	0.09	0.09	0.01	0.01	0.01	1.12 [1.04-1.21]*	1.06 [0.98-1.15]
N73	41,930	79,139,910	0.61	0.51	0.53	0.10	0.08	0.03	1.16 [1.12-1.20]*	1.06 [1.02-1.09]*
N75	7,320	79,963,230	0.10	0.09	0.09	0.01	0.00	0.00	1.04 [0.96-1.13]	0.99 [0.91-1.07]
N76	9,810	79,914,850	0.17	0.11	0.12	0.05	0.05	0.04	1.44 [1.35-1.53]*	1.29 [1.21-1.38]*
N77	13,250	79,919,600	0.21	0.16	0.16	0.05	0.05	0.02	1.30 [1.23-1.38]*	1.13 [1.06-1.20]*
N80	41,990	79,084,600	0.61	0.51	0.53	0.09	0.08	0.02	1.15 [1.11-1.18]*	1.04 [1.00-1.07]
N81	68,190	78,616,600	1.02	0.83	0.85	0.18	0.17	0.11	1.20 [1.17-1.23]*	1.13 [1.10-1.16]*
N82	3,220	80,127,410	0.05	0.04	0.04	0.01	0.01	0.01	1.32 [1.17-1.48]*	1.21 [1.07-1.36]*
N83	66,730	78,657,570	0.97	0.82	0.85	0.15	0.13	0.04	1.15 [1.12-1.18]*	1.04 [1.01-1.07]*
N84	56,920	78,770,070	0.80	0.71	0.71	0.10	0.09	0.07	1.13 [1.09-1.16]*	1.09 [1.06-1.12]*
N85	44,850	79,242,320	0.66	0.55	0.56	0.12	0.11	0.07	1.19 [1.16-1.23]*	1.11 [1.08-1.15]*
N86	13,370	79,777,460	0.20	0.16	0.17	0.04	0.03	0.02	1.20 [1.13-1.27]*	1.12 [1.05-1.19]*
N87	16,420	79,444,200	0.23	0.20	0.20	0.03	0.03	0.01	1.13 [1.07-1.19]*	1.06 [1.00-1.12]
N88	13,410	79,863,860	0.20	0.16	0.16	0.04	0.03	0.03	1.21 [1.14-1.28]*	1.15 [1.08-1.22]*
N89	21,720	79,582,710	0.34	0.26	0.26	0.08	0.08	0.05	1.30 [1.25-1.36]*	1.18 [1.13-1.24]*
N90	20,520	79,704,310	0.34	0.24	0.25	0.10	0.09	0.07	1.37 [1.31-1.43]*	1.26 [1.20-1.32]*
N91	2,220	80,103,500	0.03	0.03	0.03	0.01	0.01	0.00	1.30 [1.13-1.49]*	1.14 [0.99-1.32]
N92	90,160	77,478,560	1.39	1.11	1.15	0.27	0.24	0.13	1.21 [1.18-1.24]*	1.10 [1.08-1.13]*
N93	41,760	78,933,430	0.63	0.51	0.52	0.12	0.11	0.05	1.21 [1.17-1.25]*	1.10 [1.06-1.13]*
N94	33,220	79,027,970	0.52	0.40	0.41	0.12	0.12	0.06	1.28 [1.24-1.33]*	1.13 [1.09-1.17]*
N95	48,690	78,929,200	0.75	0.59	0.60	0.16	0.15	0.11	1.25 [1.21-1.29]*	1.18 [1.15-1.22]*
N96	710	80,157,340	0.01	0.01	0.01	0.00	0.00	0.00	1.11 [0.85-1.44]	1.07 [0.81-1.41]
N97	7,810	79,616,230	0.09	0.10	0.10	−0.01	−0.01	−0.01	0.95 [0.87-1.03]	0.93 [0.86-1.01]
N98	970	80,143,350	0.01	0.01	0.01	0.00	0.00	0.00	0.81 [0.64-1.04]	0.76 [0.59-0.98]
N99	11,530	79,948,240	0.17	0.14	0.14	0.04	0.04	0.03	1.25 [1.18-1.33]*	1.17 [1.10-1.25]*
18+ - O00-O9A										
O00	13,100	79,801,330	0.18	0.16	0.17	0.02	0.02	0.01	1.09 [1.03-1.16]*	1.03 [0.97-1.10]
O01	790	80,149,540	0.01	0.01	0.01	0.00	0.00	0.00	1.18 [0.93-1.51]	1.17 [0.91-1.50]
O02	30,130	79,092,520	0.42	0.37	0.38	0.05	0.04	0.03	1.11 [1.06-1.15]*	1.07 [1.03-1.11]*
O03	45,160	78,658,480	0.63	0.56	0.59	0.07	0.04	0.02	1.07 [1.04-1.11]*	1.03 [1.00-1.07]
O04	53,100	78,170,760	0.74	0.67	0.71	0.08	0.03	0.00	1.04 [1.01-1.07]*	0.99 [0.96-1.03]
O08	4,680	80,056,880	0.06	0.06	0.06	0.01	0.01	0.00	1.09 [0.98-1.20]	1.01 [0.91-1.12]
O10	4,860	80,062,620	0.07	0.06	0.06	0.01	0.01	0.00	1.16 [1.05-1.28]*	1.07 [0.97-1.19]
O11	680	80,155,860	0.01	0.01	0.01	0.00	0.00	0.00	1.26 [0.98-1.63]	1.16 [0.88-1.52]
O12	9,940	79,813,350	0.15	0.12	0.13	0.03	0.02	0.01	1.15 [1.08-1.23]*	1.06 [0.99-1.14]
O13	27,630	79,231,360	0.39	0.34	0.35	0.05	0.04	0.03	1.11 [1.07-1.16]*	1.08 [1.04-1.13]*
O14	20,080	79,521,210	0.29	0.24	0.26	0.04	0.03	0.02	1.12 [1.07-1.18]*	1.09 [1.03-1.14]*
O15	780	80,146,950	0.01	0.01	0.01	0.00	0.00	0.00	1.12 [0.88-1.44]	1.08 [0.83-1.39]
O16	22,430	79,198,340	0.32	0.28	0.29	0.04	0.04	0.03	1.12 [1.07-1.18]*	1.08 [1.03-1.14]*
O20	39,250	78,815,340	0.56	0.48	0.51	0.08	0.05	0.02	1.10 [1.06-1.14]*	1.03 [1.00-1.07]
O21	27,820	79,355,160	0.41	0.34	0.36	0.07	0.06	0.02	1.16 [1.11-1.21]*	1.05 [1.00-1.09]
O22	4,630	80,063,430	0.06	0.06	0.06	0.01	0.01	0.00	1.10 [1.00-1.22]	1.07 [0.96-1.19]
O23	30,190	79,217,370	0.46	0.36	0.38	0.10	0.08	0.04	1.20 [1.15-1.24]*	1.09 [1.05-1.14]*
O24	37,780	79,520,960	0.54	0.46	0.49	0.07	0.05	0.03	1.09 [1.06-1.13]*	1.07 [1.03-1.11]*
O26	112,960	76,196,660	1.81	1.41	1.52	0.40	0.29	0.16	1.19 [1.17-1.22]*	1.10 [1.07-1.12]*
O28	11,600	79,969,550	0.16	0.14	0.15	0.02	0.01	0.00	1.09 [1.02-1.16]*	1.01 [0.94-1.08]
O30	14,380	79,793,260	0.19	0.18	0.18	0.01	0.00	0.00	1.01 [0.96-1.08]	0.98 [0.92-1.04]
O31	780	80,151,690	0.01	0.01	0.01	0.00	0.00	0.00	1.10 [0.86-1.41]	1.01 [0.78-1.31]
O32	56,540	78,630,390	0.79	0.70	0.74	0.08	0.04	0.03	1.06 [1.03-1.09]*	1.04 [1.01-1.07]
O33	1,350	80,103,240	0.02	0.02	0.02	0.00	0.00	0.00	1.19 [0.99-1.44]	1.16 [0.96-1.40]
O34	93,700	77,911,470	1.36	1.17	1.23	0.19	0.13	0.10	1.10 [1.08-1.13]*	1.08 [1.06-1.11]*
O35	13,960	79,854,480	0.19	0.17	0.18	0.02	0.01	0.01	1.06 [1.00-1.13]	1.03 [0.97-1.10]
O36	151,870	76,116,270	2.42	1.90	2.09	0.51	0.32	0.19	1.15 [1.13-1.17]*	1.09 [1.07-1.11]*
O40	12,940	79,935,190	0.18	0.16	0.17	0.03	0.02	0.01	1.10 [1.04-1.17]*	1.04 [0.98-1.11]
O41	17,210	79,621,930	0.24	0.21	0.22	0.03	0.03	0.02	1.12 [1.06-1.18]*	1.08 [1.02-1.14]*
O42	92,130	77,616,700	1.32	1.16	1.24	0.16	0.07	0.06	1.06 [1.04-1.08]*	1.04 [1.02-1.07]*
O43	12,820	79,767,780	0.17	0.16	0.16	0.02	0.01	0.00	1.06 [0.99-1.12]	1.03 [0.97-1.10]
O44	8,680	79,938,290	0.12	0.11	0.11	0.01	0.00	0.00	1.04 [0.96-1.12]	0.99 [0.92-1.07]
O45	3,850	80,063,590	0.05	0.05	0.05	0.00	0.00	0.00	1.05 [0.94-1.17]	1.01 [0.90-1.14]
O46	42,510	78,789,400	0.63	0.52	0.54	0.11	0.08	0.05	1.16 [1.12-1.19]*	1.09 [1.06-1.13]*
O47	29,930	78,234,860	0.44	0.37	0.38	0.07	0.07	0.04	1.17 [1.13-1.22]*	1.10 [1.06-1.14]*
O48	56,850	78,046,270	0.77	0.72	0.75	0.06	0.02	0.02	1.03 [1.00-1.06]	1.03 [1.00-1.06]
O60	47,310	78,791,130	0.68	0.58	0.61	0.10	0.07	0.04	1.11 [1.07-1.14]*	1.05 [1.02-1.09]*
O61	9,080	79,882,130	0.13	0.11	0.11	0.02	0.01	0.01	1.11 [1.03-1.19]*	1.06 [0.98-1.14]
O62	36,040	79,234,370	0.51	0.44	0.47	0.06	0.04	0.03	1.08 [1.04-1.12]*	1.06 [1.02-1.10]*
O63	82,080	77,220,080	1.14	1.05	1.11	0.10	0.04	0.03	1.03 [1.01-1.06]	1.02 [1.00-1.05]
O64	16,050	79,496,560	0.21	0.20	0.20	0.01	0.01	0.01	1.03 [0.97-1.09]	1.03 [0.97-1.09]
O65	1,150	80,100,630	0.01	0.01	0.01	0.00	0.00	0.00	1.01 [0.82-1.24]	1.04 [0.84-1.29]
O66	15,500	79,643,040	0.21	0.19	0.20	0.01	0.01	0.00	1.03 [0.97-1.09]	1.01 [0.96-1.07]
O67	4,050	80,023,680	0.05	0.05	0.05	0.00	0.00	0.00	1.07 [0.96-1.19]	1.06 [0.95-1.19]
O68	170,860	74,525,240	2.63	2.22	2.44	0.41	0.18	0.14	1.08 [1.06-1.09]*	1.06 [1.04-1.07]*
O69	15,930	79,359,800	0.22	0.20	0.20	0.03	0.02	0.02	1.10 [1.04-1.16]*	1.10 [1.04-1.16]*
O70	227,580	72,189,890	3.65	3.04	3.44	0.60	0.21	0.19	1.06 [1.04-1.08]*	1.06 [1.04-1.07]*
O71	13,960	79,736,300	0.19	0.17	0.18	0.01	0.01	0.01	1.05 [0.99-1.11]	1.05 [0.98-1.11]
O72	115,960	77,496,740	1.69	1.45	1.57	0.24	0.12	0.11	1.08 [1.06-1.10]*	1.07 [1.05-1.09]*
O73	8,140	79,866,150	0.11	0.10	0.10	0.01	0.01	0.01	1.11 [1.03-1.20]*	1.08 [1.00-1.17]
O74	2,220	80,112,630	0.03	0.03	0.03	0.01	0.00	0.00	1.15 [1.00-1.34]	1.10 [0.95-1.28]
O75	57,770	78,605,140	0.81	0.72	0.76	0.09	0.05	0.04	1.06 [1.03-1.09]*	1.06 [1.03-1.09]*
O80	61,780	77,113,120	0.89	0.78	0.82	0.11	0.07	0.07	1.09 [1.06-1.12]*	1.09 [1.06-1.12]*
O81	950	80,059,650	0.01	0.01	0.01	0.00	0.00	0.00	1.05 [0.84-1.32]	1.04 [0.83-1.31]
O82	9,110	79,677,790	0.12	0.11	0.11	0.01	0.01	0.01	1.08 [1.00-1.16]	1.05 [0.98-1.13]
O85	4,840	80,099,980	0.07	0.06	0.06	0.01	0.01	0.00	1.08 [0.98-1.20]	1.02 [0.92-1.13]
O86	17,630	79,721,450	0.26	0.21	0.22	0.05	0.04	0.03	1.18 [1.12-1.24]*	1.13 [1.08-1.20]*
O87	2,560	80,074,810	0.04	0.03	0.03	0.01	0.01	0.01	1.24 [1.09-1.42]*	1.21 [1.06-1.39]*
O88	920	80,153,650	0.01	0.01	0.01	0.00	0.00	0.00	1.20 [0.96-1.50]	1.10 [0.87-1.39]
O89	760	80,148,980	0.01	0.01	0.01	0.00	0.00	0.00	1.34 [1.06-1.71]	1.30 [1.02-1.67]
O90	20,850	79,748,790	0.31	0.25	0.26	0.06	0.05	0.04	1.20 [1.14-1.25]*	1.13 [1.08-1.19]*
O91	2,110	80,129,070	0.03	0.03	0.03	0.00	0.00	0.00	1.06 [0.91-1.23]	1.02 [0.87-1.20]
O92	3,200	80,079,340	0.04	0.04	0.04	0.01	0.00	0.00	1.12 [0.99-1.26]	1.07 [0.95-1.22]
O94	680	80,167,110	0.01	0.01	0.01	0.00	0.00	0.00	1.18 [0.92-1.53]	1.04 [0.79-1.36]
O98	7,610	79,984,320	0.12	0.09	0.10	0.03	0.02	0.01	1.21 [1.12-1.31]*	1.14 [1.05-1.23]*
O99	125,400	76,748,410	2.01	1.55	1.69	0.46	0.33	0.19	1.19 [1.17-1.22]*	1.11 [1.08-1.13]*
18+ - Q00-Q99										
Q03	440	80,154,340	0.01	0.00	0.01	0.00	0.00	0.00	1.58 [1.17-2.12]*	1.47 [1.08-2.00]
Q04	850	80,150,240	0.01	0.01	0.01	0.00	0.00	0.00	1.21 [0.96-1.53]	1.07 [0.84-1.36]
Q05	1,800	80,123,680	0.03	0.02	0.02	0.01	0.01	0.00	1.33 [1.14-1.55]*	1.20 [1.02-1.40]
Q07	1,560	80,147,510	0.03	0.02	0.02	0.01	0.01	0.00	1.34 [1.14-1.58]*	1.12 [0.94-1.34]
Q10	460	80,149,410	0.01	0.01	0.01	0.00	0.00	0.00	1.46 [1.08-1.97]	1.40 [1.02-1.90]
Q12	880	80,150,000	0.01	0.01	0.01	0.00	0.00	0.00	1.26 [1.00-1.58]	1.19 [0.94-1.51]
Q18	1,570	80,111,760	0.02	0.02	0.02	0.00	0.00	0.00	1.10 [0.93-1.31]	1.01 [0.84-1.20]
Q21	7,160	80,008,080	0.11	0.09	0.09	0.02	0.02	0.01	1.24 [1.14-1.34]*	1.14 [1.06-1.24]*
Q23	3,520	80,117,360	0.05	0.04	0.04	0.01	0.01	0.00	1.17 [1.04-1.31]*	1.10 [0.98-1.24]
Q24	2,400	80,119,680	0.04	0.03	0.03	0.01	0.01	0.01	1.36 [1.19-1.55]*	1.23 [1.07-1.41]*
Q25	1,730	80,106,240	0.03	0.02	0.02	0.00	0.00	0.00	1.19 [1.01-1.40]	1.11 [0.94-1.31]
Q27	1,950	80,136,840	0.03	0.02	0.02	0.00	0.00	0.00	1.20 [1.03-1.40]	1.13 [0.96-1.32]

Q28	1,400	80,144,200	0.02	0.02	0.02	0.01	0.01	0.01	1.46 [1.23-1.74]*	1.36 [1.14-1.63]*
Q38	3,260	80,062,820	0.05	0.04	0.04	0.01	0.01	0.00	1.16 [1.02-1.31]	1.08 [0.95-1.22]
Q39	1,580	80,152,680	0.02	0.02	0.02	0.01	0.01	0.00	1.30 [1.09-1.53]*	1.16 [0.98-1.38]
Q40	1,000	80,151,610	0.01	0.01	0.01	0.00	0.00	0.00	1.20 [0.97-1.48]	1.03 [0.82-1.28]
Q43	3,370	80,112,740	0.05	0.04	0.04	0.01	0.01	0.00	1.23 [1.10-1.38]*	1.10 [0.98-1.24]
Q44	1,200	80,156,550	0.02	0.01	0.01	0.00	0.00	0.00	1.21 [1.00-1.47]	1.10 [0.91-1.34]
Q45	500	80,167,050	0.01	0.01	0.01	0.00	0.00	0.00	1.36 [1.01-1.83]	1.13 [0.82-1.54]
Q50	2,780	80,102,130	0.04	0.03	0.03	0.01	0.01	0.01	1.30 [1.14-1.47]*	1.19 [1.04-1.35]
Q51	2,900	80,112,880	0.04	0.04	0.04	0.00	0.00	0.00	1.10 [0.97-1.25]	1.02 [0.89-1.16]
Q52	880	80,142,290	0.01	0.01	0.01	0.00	0.00	0.00	1.22 [0.97-1.52]	1.14 [0.90-1.44]
Q53	640	80,025,360	0.01	0.01	0.01	0.00	0.00	0.00	1.12 [0.85-1.46]	1.05 [0.79-1.38]
Q54	1,410	80,114,160	0.02	0.02	0.02	0.00	0.00	0.00	1.24 [1.03-1.49]	1.17 [0.97-1.41]
Q55	770	80,091,590	0.01	0.01	0.01	0.00	0.00	0.00	1.22 [0.96-1.55]	1.10 [0.86-1.42]
Q60	2,350	80,139,320	0.04	0.03	0.03	0.01	0.01	0.00	1.20 [1.05-1.38]*	1.08 [0.94-1.25]
Q61	6,530	80,077,270	0.09	0.08	0.08	0.01	0.01	0.00	1.14 [1.05-1.24]*	1.04 [0.95-1.14]
Q62	740	80,134,140	0.01	0.01	0.01	0.00	0.00	0.00	1.01 [0.77-1.31]	0.98 [0.75-1.29]
Q63	4,170	80,101,230	0.06	0.05	0.05	0.01	0.01	0.00	1.17 [1.05-1.30]*	1.05 [0.94-1.17]
Q64	530	80,151,070	0.01	0.01	0.01	0.00	0.00	0.00	1.31 [0.99-1.74]	1.21 [0.91-1.62]
Q65	2,480	80,103,570	0.04	0.03	0.03	0.01	0.01	0.00	1.17 [1.02-1.34]	1.07 [0.93-1.23]
Q66	2,290	80,040,100	0.04	0.03	0.03	0.01	0.01	0.01	1.38 [1.20-1.57]*	1.25 [1.09-1.44]*
Q67	1,390	80,133,010	0.02	0.02	0.02	0.01	0.01	0.01	1.41 [1.19-1.68]*	1.30 [1.09-1.56]*
Q74	1,430	80,129,100	0.02	0.02	0.02	0.01	0.00	0.00	1.28 [1.08-1.53]*	1.16 [0.97-1.40]
Q76	2,040	80,128,960	0.03	0.02	0.03	0.01	0.01	0.00	1.23 [1.06-1.42]*	1.05 [0.90-1.22]
Q78	1,040	80,150,320	0.02	0.01	0.01	0.00	0.00	0.00	1.31 [1.07-1.61]	1.17 [0.95-1.45]
Q79	2,710	80,131,760	0.04	0.03	0.03	0.01	0.01	0.00	1.34 [1.18-1.52]*	1.01 [0.88-1.16]
Q82	1,350	80,094,200	0.03	0.02	0.01	0.01	0.01	0.01	1.75 [1.47-2.07]*	1.56 [1.31-1.86]*
Q83	1,400	80,111,110	0.02	0.02	0.02	0.00	0.00	0.00	1.15 [0.96-1.38]	1.05 [0.87-1.26]
Q85	1,680	80,132,350	0.02	0.02	0.02	0.00	0.00	0.00	1.05 [0.89-1.24]	0.95 [0.80-1.14]
Q87	1,700	80,116,430	0.03	0.02	0.02	0.01	0.01	0.01	1.44 [1.24-1.69]*	1.30 [1.10-1.52]*
Q89	1,500	80,127,150	0.02	0.02	0.02	0.00	0.00	0.00	1.20 [1.01-1.43]	1.09 [0.91-1.30]
Q90	1,640	80,087,070	0.05	0.01	0.01	0.04	0.04	0.04	3.76 [3.28-4.32]*	3.86 [3.35-4.45]*
Q99	680	80,155,540	0.01	0.01	0.01	0.00	0.00	0.00	1.28 [1.00-1.65]	1.14 [0.88-1.47]
18+ - R00-R99										
R00	192,580	78,229,360	2.95	2.36	2.42	0.59	0.53	0.29	1.22 [1.20-1.24]*	1.11 [1.09-1.13]*
R01	25,540	79,871,390	0.38	0.31	0.31	0.08	0.07	0.04	1.22 [1.17-1.27]*	1.12 [1.07-1.17]*
R02	29,260	79,998,790	0.44	0.35	0.36	0.09	0.08	0.05	1.23 [1.18-1.28]*	1.13 [1.08-1.18]*
R03	69,240	79,463,910	1.03	0.84	0.86	0.19	0.16	0.11	1.19 [1.16-1.22]*	1.12 [1.09-1.15]*
R04	70,990	79,136,520	1.14	0.84	0.85	0.29	0.29	0.20	1.35 [1.31-1.38]*	1.22 [1.18-1.25]*
R05	62,990	79,430,140	1.03	0.74	0.76	0.28	0.27	0.16	1.35 [1.32-1.39]*	1.19 [1.15-1.22]*
R06	180,450	77,612,430	2.97	2.19	2.22	0.78	0.75	0.45	1.34 [1.32-1.36]*	1.18 [1.16-1.20]*
R07	330,860	73,910,210	5.59	4.23	4.29	1.36	1.30	0.76	1.30 [1.29-1.32]*	1.16 [1.14-1.17]*
R09	28,530	79,896,050	0.45	0.34	0.34	0.11	0.11	0.07	1.32 [1.27-1.37]*	1.17 [1.12-1.22]*
R10	412,880	69,634,660	7.38	5.60	5.76	1.78	1.62	0.91	1.28 [1.27-1.29]*	1.14 [1.13-1.15]*
R11	227,170	76,777,900	3.60	2.82	2.87	0.78	0.73	0.39	1.25 [1.23-1.27]*	1.12 [1.10-1.14]*
R12	17,770	79,855,150	0.29	0.21	0.21	0.08	0.07	0.04	1.35 [1.28-1.42]*	1.18 [1.12-1.24]*
R13	130,210	79,048,840	2.09	1.55	1.59	0.54	0.50	0.32	1.32 [1.29-1.34]*	1.18 [1.16-1.21]*
R14	29,400	79,834,070	0.47	0.35	0.35	0.12	0.11	0.06	1.32 [1.27-1.37]*	1.15 [1.11-1.20]*
R15	55,150	79,891,400	0.83	0.66	0.69	0.17	0.14	0.09	1.20 [1.17-1.24]*	1.13 [1.09-1.16]*
R16	19,700	80,000,540	0.33	0.23	0.23	0.10	0.10	0.07	1.44 [1.38-1.51]*	1.28 [1.22-1.35]*
R17	17,050	79,988,040	0.25	0.20	0.20	0.05	0.05	0.04	1.24 [1.18-1.31]*	1.18 [1.12-1.24]*
R18	44,410	79,927,710	0.65	0.53	0.54	0.12	0.12	0.08	1.22 [1.18-1.26]*	1.15 [1.11-1.19]*
R19	166,250	78,069,200	2.74	2.00	2.02	0.74	0.71	0.47	1.35 [1.33-1.37]*	1.21 [1.19-1.23]*
R20	45,150	79,515,400	0.70	0.54	0.55	0.16	0.15	0.07	1.27 [1.23-1.31]*	1.11 [1.07-1.14]*
R21	43,940	79,405,940	0.84	0.49	0.50	0.35	0.35	0.30	1.70 [1.65-1.75]*	1.55 [1.50-1.60]*
R22	30,100	79,631,430	0.49	0.35	0.36	0.13	0.13	0.09	1.37 [1.31-1.42]*	1.22 [1.17-1.27]*
R23	16,820	79,956,570	0.27	0.20	0.20	0.07	0.07	0.05	1.33 [1.26-1.40]*	1.21 [1.15-1.28]*
R25	23,960	79,919,330	0.37	0.28	0.29	0.09	0.08	0.05	1.28 [1.23-1.34]*	1.14 [1.09-1.19]*
R26	199,480	79,388,970	3.06	2.40	2.54	0.66	0.52	0.33	1.20 [1.19-1.22]*	1.12 [1.10-1.14]*
R27	6,420	80,094,190	0.09	0.08	0.08	0.02	0.01	0.01	1.18 [1.08-1.29]*	1.07 [0.98-1.16]
R29	269,000	79,132,700	4.09	3.25	3.43	0.84	0.66	0.39	1.19 [1.18-1.21]*	1.10 [1.09-1.12]*
R30	19,180	79,829,070	0.31	0.23	0.23	0.08	0.08	0.04	1.34 [1.28-1.41]*	1.17 [1.11-1.23]*
R31	143,650	77,624,320	2.16	1.78	1.81	0.37	0.35	0.23	1.19 [1.17-1.21]*	1.12 [1.10-1.14]*
R32	104,820	79,501,710	1.57	1.27	1.32	0.30	0.24	0.15	1.18 [1.16-1.21]*	1.10 [1.08-1.13]*
R33	203,160	78,648,870	3.03	2.49	2.61	0.54	0.42	0.27	1.16 [1.14-1.18]*	1.10 [1.08-1.11]*
R34	5,050	80,146,660	0.08	0.06	0.06	0.02	0.02	0.01	1.30 [1.18-1.43]*	1.17 [1.06-1.30]*
R35	32,500	79,438,270	0.52	0.39	0.39	0.13	0.13	0.09	1.33 [1.29-1.38]*	1.20 [1.15-1.25]*
R36	680	80,162,720	0.01	0.01	0.01	0.00	0.00	0.00	1.35 [1.04-1.75]	1.23 [0.94-1.61]
R39	37,920	79,427,170	0.61	0.45	0.45	0.16	0.16	0.10	1.35 [1.31-1.40]*	1.21 [1.16-1.25]*
R40	29,980	79,948,600	0.46	0.36	0.37	0.10	0.08	0.05	1.23 [1.18-1.28]*	1.12 [1.07-1.16]*
R41	194,300	79,182,880	2.90	2.36	2.46	0.54	0.43	0.29	1.18 [1.16-1.20]*	1.11 [1.09-1.13]*
R42	106,100	78,959,630	1.63	1.28	1.31	0.35	0.32	0.17	1.25 [1.22-1.27]*	1.12 [1.09-1.14]*
R43	1,460	80,159,530	0.02	0.02	0.02	0.01	0.00	0.00	1.28 [1.07-1.52]*	1.12 [0.93-1.34]
R44	26,350	79,989,560	0.40	0.31	0.32	0.08	0.07	0.04	1.22 [1.17-1.28]*	1.11 [1.07-1.17]*
R45	55,460	79,792,570	0.82	0.67	0.69	0.15	0.13	0.05	1.19 [1.15-1.22]*	1.06 [1.03-1.09]*
R46	16,950	80,079,460	0.24	0.21	0.21	0.03	0.03	0.02	1.13 [1.07-1.19]*	1.07 [1.01-1.13]
R47	53,720	79,774,510	0.79	0.65	0.67	0.14	0.11	0.06	1.17 [1.13-1.20]*	1.08 [1.05-1.12]*
R48	1,560	80,156,470	0.02	0.02	0.02	0.01	0.01	0.00	1.32 [1.11-1.56]*	1.10 [0.92-1.32]
R49	10,120	79,975,150	0.16	0.12	0.12	0.04	0.04	0.02	1.35 [1.27-1.44]*	1.17 [1.10-1.26]*
R50	75,200	79,048,190	1.17	0.90	0.92	0.26	0.25	0.17	1.27 [1.24-1.30]*	1.17 [1.14-1.20]*
R51	137,650	77,590,440	2.22	1.68	1.72	0.55	0.50	0.26	1.29 [1.27-1.32]*	1.13 [1.11-1.15]*
R52	24,030	79,907,530	0.40	0.28	0.28	0.12	0.12	0.07	1.42 [1.36-1.48]*	1.20 [1.15-1.25]*
R53	96,380	79,444,960	1.48	1.16	1.20	0.32	0.28	0.16	1.23 [1.20-1.26]*	1.12 [1.10-1.15]*
R54	127,790	79,721,980	1.89	1.54	1.62	0.35	0.28	0.19	1.17 [1.15-1.19]*	1.11 [1.09-1.14]*
R55	191,080	77,903,180	2.90	2.36	2.42	0.55	0.49	0.31	1.20 [1.18-1.22]*	1.12 [1.10-1.14]*
R56	53,580	79,207,090	0.82	0.65	0.65	0.17	0.17	0.11	1.26 [1.22-1.29]*	1.15 [1.12-1.19]*
R57	16,660	80,130,760	0.24	0.20	0.20	0.04	0.04	0.03	1.20 [1.13-1.26]*	1.12 [1.06-1.18]*
R58	2,550	80,152,890	0.04	0.03	0.03	0.01	0.01	0.01	1.34 [1.17-1.52]*	1.22 [1.07-1.40]*
R59	48,530	79,608,580	0.78	0.57	0.57	0.21	0.21	0.17	1.36 [1.32-1.40]*	1.27 [1.23-1.31]*
R60	91,280	79,680,660	1.46	1.08	1.10	0.38	0.36	0.26	1.33 [1.30-1.36]*	1.22 [1.19-1.24]*
R61	9,730	80,020,340	0.16	0.11	0.11	0.04	0.04	0.03	1.38 [1.29-1.48]*	1.21 [1.13-1.30]*
R62	2,060	80,025,770	0.03	0.03	0.03	0.00	0.00	0.00	1.09 [0.93-1.27]	1.00 [0.85-1.17]
R63	181,590	78,602,310	2.82	2.20	2.26	0.62	0.56	0.35	1.25 [1.23-1.27]*	1.14 [1.12-1.16]*
R64	5,210	80,163,000	0.07	0.06	0.07	0.01	0.01	0.01	1.12 [1.01-1.24]	1.09 [0.99-1.21]
R65	11,770	80,144,340	0.18	0.14	0.15	0.04	0.03	0.02	1.22 [1.14-1.30]*	1.12 [1.05-1.20]*
R68	17,480	80,038,470	0.26	0.21	0.21	0.05	0.05	0.04	1.24 [1.18-1.31]*	1.18 [1.12-1.25]*
R69	157,270	75,394,290	2.49	2.00	2.00	0.49	0.49	0.33	1.24 [1.22-1.27]*	1.15 [1.13-1.17]*
R70	1,300	80,144,060	0.02	0.01	0.02	0.01	0.01	0.01	1.46 [1.22-1.75]*	1.35 [1.13-1.62]*
R71	1,120	80,162,110	0.02	0.01	0.01	0.00	0.00	0.00	1.11 [0.90-1.36]	1.05 [0.85-1.30]
R72	6,120	80,117,630	0.09	0.07	0.08	0.02	0.01	0.01	1.20 [1.10-1.31]*	1.10 [1.01-1.21]
R73	22,750	79,954,770	0.36	0.27	0.27	0.09	0.09	0.05	1.32 [1.26-1.38]*	1.17 [1.12-1.23]*
R74	10,150	80,091,930	0.15	0.12	0.13	0.03	0.02	0.01	1.17 [1.09-1.26]*	1.06 [0.98-1.14]
R76	2,640	80,133,350	0.04	0.03	0.03	0.01	0.01	0.01	1.37 [1.21-1.56]*	1.26 [1.11-1.44]*
R77	6,380	80,138,030	0.10	0.08	0.08	0.02	0.02	0.01	1.29 [1.18-1.40]*	1.16 [1.07-1.27]*
R78	850	80,158,980	0.01	0.01	0.01	0.00	0.00	0.00	1.16 [0.91-1.46]	1.04 [0.81-1.32]
R79	126,160	79,188,200	1.93	1.52	1.56	0.41	0.37	0.23	1.24 [1.21-1.26]*	1.14 [1.11-1.16]*
R80	4,430	80,085,640	0.07	0.05	0.05	0.02	0.02	0.01	1.38 [1.25-1.53]*	1.26 [1.14-1.40]*
R81	1,360	80,082,290	0.02	0.02	0.02	0.00	0.00	0.00	1.15 [0.96-1.38]	1.09 [0.90-1.31]
R82	9,840	80,060,100	0.15	0.12	0.12	0.03	0.03	0.01	1.21 [1.13-1.30]*	1.10 [1.03-1.18]*

R84	2,850	80,155,190	0.05	0.03	0.03	0.01	0.01	0.01	1.36 [1.20-1.54]*	1.15 [1.01-1.31]
R85	1,790	80,125,620	0.03	0.02	0.02	0.01	0.01	0.01	1.39 [1.19-1.62]*	1.25 [1.06-1.46]*
R87	9,800	79,625,700	0.14	0.12	0.12	0.02	0.02	0.01	1.17 [1.09-1.25]*	1.11 [1.03-1.19]*
R89	1,580	80,155,780	0.03	0.02	0.02	0.01	0.01	0.01	1.44 [1.22-1.70]*	1.31 [1.11-1.55]*
R90	5,560	80,123,330	0.08	0.07	0.07	0.01	0.01	0.01	1.19 [1.08-1.30]*	1.10 [1.00-1.21]
R91	62,470	79,740,830	0.95	0.75	0.76	0.21	0.19	0.12	1.25 [1.22-1.29]*	1.15 [1.12-1.18]*
R92	570	80,165,850	0.01	0.01	0.01	0.00	0.00	0.00	1.09 [0.81-1.46]	0.97 [0.72-1.32]
R93	63,010	79,633,310	0.93	0.76	0.77	0.17	0.16	0.10	1.21 [1.18-1.25]*	1.12 [1.09-1.15]*
R94	111,220	79,247,940	1.72	1.34	1.36	0.38	0.36	0.25	1.26 [1.24-1.29]*	1.17 [1.14-1.19]*
18+ - S00-T88										
S00	101,420	79,211,750	1.52	1.23	1.28	0.29	0.24	0.15	1.19 [1.16-1.21]*	1.11 [1.08-1.13]*
S01	102,230	78,962,470	1.52	1.25	1.29	0.28	0.24	0.16	1.18 [1.16-1.21]*	1.12 [1.09-1.14]*
S02	39,290	79,240,280	0.56	0.48	0.49	0.08	0.08	0.05	1.16 [1.12-1.20]*	1.09 [1.05-1.13]*
S03	970	80,152,920	0.01	0.01	0.01	0.00	0.00	0.00	1.14 [0.91-1.41]	1.05 [0.84-1.31]
S05	9,650	80,025,460	0.14	0.12	0.12	0.02	0.02	0.01	1.15 [1.07-1.23]*	1.08 [1.01-1.16]
S06	32,240	79,818,420	0.46	0.39	0.40	0.06	0.05	0.03	1.13 [1.08-1.17]*	1.07 [1.03-1.11]*
S09	48,480	79,139,860	0.73	0.59	0.60	0.14	0.13	0.07	1.21 [1.18-1.25]*	1.11 [1.07-1.15]*
S10	2,900	80,122,220	0.04	0.04	0.04	0.00	0.00	0.00	1.13 [0.99-1.29]	1.02 [0.89-1.17]
S11	1,720	80,143,230	0.03	0.02	0.02	0.00	0.00	0.00	1.19 [1.01-1.40]	1.09 [0.92-1.29]
S12	8,500	80,096,430	0.12	0.10	0.11	0.02	0.02	0.01	1.18 [1.09-1.27]*	1.11 [1.02-1.20]
S13	1,930	80,121,470	0.03	0.02	0.02	0.00	0.00	0.00	1.20 [1.03-1.40]	1.09 [0.92-1.27]
S14	1,600	80,149,460	0.02	0.02	0.02	0.00	0.00	0.00	1.07 [0.90-1.28]	1.04 [0.87-1.24]
S19	2,380	80,113,760	0.04	0.03	0.03	0.01	0.01	0.00	1.30 [1.13-1.49]*	1.14 [0.99-1.32]
S20	11,700	80,051,260	0.18	0.14	0.15	0.04	0.03	0.02	1.22 [1.15-1.31]*	1.11 [1.03-1.18]*
S21	2,280	80,131,810	0.03	0.03	0.03	0.01	0.00	0.00	1.15 [1.00-1.33]	1.05 [0.90-1.22]
S22	41,950	79,762,840	0.62	0.50	0.52	0.12	0.11	0.06	1.21 [1.17-1.25]*	1.11 [1.07-1.15]*
S27	12,860	80,013,670	0.18	0.16	0.16	0.02	0.02	0.01	1.10 [1.04-1.18]*	1.04 [0.98-1.11]
S29	3,190	80,133,270	0.05	0.04	0.04	0.02	0.02	0.01	1.44 [1.28-1.62]*	1.28 [1.13-1.44]*
S30	20,230	79,934,440	0.30	0.24	0.25	0.06	0.05	0.03	1.22 [1.16-1.28]*	1.10 [1.04-1.15]*
S31	5,020	80,057,000	0.07	0.06	0.06	0.01	0.01	0.00	1.13 [1.02-1.25]	1.02 [0.93-1.13]
S32	45,970	79,780,790	0.66	0.56	0.58	0.10	0.08	0.05	1.14 [1.10-1.17]*	1.07 [1.04-1.11]*
S33	590	80,162,150	0.01	0.01	0.01	0.00	0.00	0.00	1.00 [0.75-1.35]	0.92 [0.68-1.25]
S35	550	80,166,830	0.01	0.01	0.01	0.00	0.00	0.00	1.17 [0.87-1.56]	1.17 [0.87-1.59]
S36	4,760	80,095,200	0.07	0.06	0.06	0.01	0.01	0.00	1.12 [1.01-1.24]	1.06 [0.96-1.18]
S37	3,740	80,124,410	0.05	0.05	0.05	0.00	0.00	0.00	1.05 [0.93-1.18]	1.01 [0.89-1.14]
S39	8,050	80,021,160	0.12	0.10	0.10	0.02	0.02	0.01	1.18 [1.09-1.27]*	1.06 [0.98-1.15]
S40	17,010	80,045,050	0.25	0.20	0.21	0.05	0.04	0.02	1.17 [1.10-1.23]*	1.07 [1.01-1.13]
S41	6,640	80,102,290	0.10	0.08	0.08	0.02	0.02	0.01	1.26 [1.16-1.38]*	1.14 [1.04-1.25]*
S42	50,890	79,453,520	0.72	0.62	0.64	0.10	0.09	0.05	1.14 [1.10-1.17]*	1.08 [1.04-1.11]*
S43	9,460	80,042,740	0.13	0.12	0.12	0.02	0.01	0.01	1.11 [1.03-1.19]*	1.05 [0.98-1.13]
S46	3,860	80,131,520	0.05	0.05	0.05	0.01	0.01	0.00	1.12 [1.00-1.25]	1.04 [0.92-1.16]
S49	5,240	80,120,950	0.08	0.06	0.06	0.02	0.02	0.01	1.28 [1.16-1.40]*	1.15 [1.04-1.27]*
S50	17,920	80,036,180	0.27	0.21	0.22	0.05	0.04	0.02	1.19 [1.13-1.26]*	1.10 [1.04-1.16]*
S51	26,820	79,918,860	0.40	0.32	0.33	0.08	0.07	0.03	1.21 [1.16-1.26]*	1.09 [1.04-1.14]*
S52	64,300	78,494,210	0.89	0.80	0.81	0.09	0.08	0.05	1.10 [1.07-1.13]*	1.06 [1.03-1.09]*
S53	1,660	80,123,130	0.02	0.02	0.02	0.00	0.00	0.00	1.02 [0.86-1.21]	0.95 [0.79-1.13]
S54	1,060	80,142,700	0.02	0.01	0.01	0.00	0.00	0.00	1.23 [1.00-1.51]	1.09 [0.88-1.36]
S55	520	80,161,200	0.01	0.01	0.01	0.00	0.00	0.00	1.47 [1.11-1.95]*	1.37 [1.03-1.84]
S56	3,890	80,085,310	0.05	0.05	0.05	0.00	0.00	0.00	1.09 [0.97-1.22]	1.04 [0.92-1.16]
S59	1,990	80,149,950	0.03	0.02	0.02	0.01	0.01	0.01	1.31 [1.12-1.53]*	1.19 [1.02-1.40]
S60	13,840	80,030,760	0.20	0.17	0.17	0.04	0.03	0.02	1.17 [1.10-1.25]*	1.09 [1.02-1.15]*
S61	52,580	79,131,000	0.72	0.65	0.66	0.07	0.06	0.02	1.09 [1.06-1.13]*	1.03 [1.00-1.06]
S62	37,610	79,371,480	0.51	0.47	0.47	0.04	0.04	0.01	1.08 [1.04-1.12]*	1.02 [0.98-1.06]
S63	6,610	80,044,760	0.10	0.08	0.08	0.02	0.02	0.01	1.20 [1.10-1.31]*	1.11 [1.02-1.21]
S64	8,200	79,957,580	0.10	0.10	0.10	0.00	0.00	0.00	1.01 [0.94-1.09]	0.96 [0.89-1.04]
S65	2,260	80,119,240	0.03	0.03	0.03	0.00	0.00	0.00	1.03 [0.88-1.19]	0.99 [0.85-1.15]
S66	15,580	79,764,590	0.20	0.19	0.19	0.01	0.01	0.00	1.04 [0.99-1.10]	1.00 [0.94-1.05]
S67	1,250	80,122,960	0.01	0.02	0.02	0.00	0.00	0.00	0.78 [0.63-0.97]	0.79 [0.63-0.98]
S68	3,810	80,066,070	0.05	0.05	0.05	0.00	0.00	0.00	0.97 [0.86-1.09]	0.97 [0.86-1.09]
S69	5,200	80,093,320	0.08	0.06	0.06	0.02	0.02	0.01	1.26 [1.14-1.39]*	1.13 [1.02-1.25]
S70	16,830	80,047,640	0.25	0.20	0.21	0.04	0.03	0.02	1.16 [1.10-1.23]*	1.07 [1.01-1.13]
S71	3,120	80,109,680	0.04	0.04	0.04	0.01	0.01	0.00	1.14 [1.01-1.29]	1.02 [0.89-1.16]
S72	123,580	79,193,870	1.78	1.51	1.59	0.26	0.18	0.14	1.11 [1.09-1.14]*	1.08 [1.06-1.11]*
S73	1,510	80,148,320	0.02	0.02	0.02	0.00	0.00	0.00	1.09 [0.91-1.31]	1.02 [0.85-1.23]
S76	3,580	80,124,650	0.05	0.04	0.04	0.01	0.01	0.00	1.16 [1.03-1.30]	1.08 [0.96-1.22]
S79	9,020	80,101,460	0.14	0.11	0.11	0.03	0.03	0.02	1.23 [1.14-1.32]*	1.14 [1.06-1.23]*
S80	32,330	79,906,560	0.48	0.39	0.41	0.09	0.07	0.03	1.18 [1.13-1.22]*	1.07 [1.03-1.12]*
S81	24,430	79,915,160	0.37	0.29	0.30	0.08	0.07	0.04	1.24 [1.18-1.29]*	1.11 [1.06-1.16]*
S82	65,090	78,708,760	0.90	0.81	0.81	0.08	0.08	0.03	1.10 [1.07-1.13]*	1.04 [1.01-1.06]
S83	8,820	79,959,810	0.12	0.11	0.11	0.02	0.02	0.01	1.17 [1.08-1.25]*	1.07 [1.00-1.16]
S86	4,840	80,047,500	0.07	0.06	0.06	0.01	0.01	0.01	1.18 [1.07-1.30]*	1.10 [1.00-1.22]
S89	6,780	80,089,780	0.10	0.08	0.08	0.02	0.02	0.01	1.24 [1.14-1.34]*	1.12 [1.03-1.22]*
S90	7,900	80,090,010	0.11	0.10	0.10	0.01	0.01	0.00	1.13 [1.04-1.22]*	1.02 [0.94-1.10]
S91	6,780	80,044,570	0.10	0.08	0.08	0.02	0.02	0.01	1.23 [1.13-1.34]*	1.10 [1.01-1.20]
S92	14,330	79,906,560	0.21	0.17	0.18	0.03	0.03	0.01	1.15 [1.09-1.22]*	1.05 [0.99-1.12]
S93	4,780	80,096,520	0.07	0.06	0.06	0.01	0.01	0.00	1.13 [1.02-1.25]	1.01 [0.91-1.12]
S96	960	80,151,660	0.01	0.01	0.01	0.00	0.00	0.00	1.11 [0.89-1.38]	0.95 [0.75-1.20]
S99	3,340	80,128,550	0.05	0.04	0.04	0.01	0.01	0.00	1.19 [1.05-1.34]*	1.02 [0.90-1.16]
T00	7,150	80,116,560	0.10	0.09	0.09	0.02	0.01	0.00	1.15 [1.06-1.26]*	1.05 [0.96-1.15]
T01	3,530	80,140,620	0.05	0.04	0.04	0.01	0.01	0.00	1.14 [1.01-1.29]	0.99 [0.87-1.12]
T02	2,280	80,139,930	0.03	0.03	0.03	0.01	0.00	0.00	1.18 [1.02-1.36]	1.13 [0.98-1.31]
T09	1,230	80,153,370	0.02	0.01	0.01	0.00	0.00	0.00	1.20 [0.99-1.47]	1.11 [0.90-1.36]
T11	4,870	80,128,330	0.07	0.06	0.06	0.02	0.01	0.01	1.22 [1.10-1.35]*	1.09 [0.98-1.21]
T13	3,110	80,137,090	0.05	0.04	0.04	0.01	0.01	0.00	1.24 [1.09-1.40]*	1.12 [0.98-1.27]
T14	3,010	80,080,450	0.04	0.04	0.04	0.01	0.01	0.00	1.19 [1.05-1.35]*	1.07 [0.94-1.21]
T16	780	80,109,200	0.01	0.01	0.01	0.00	0.00	0.00	1.14 [0.89-1.47]	1.01 [0.78-1.31]
T17	6,670	80,059,520	0.11	0.08	0.08	0.03	0.03	0.02	1.36 [1.25-1.48]*	1.22 [1.12-1.33]*
T18	6,520	80,042,250	0.10	0.08	0.08	0.02	0.02	0.01	1.31 [1.21-1.42]*	1.18 [1.08-1.28]*
T19	780	80,155,890	0.01	0.01	0.01	0.00	0.00	0.00	1.29 [1.01-1.64]	1.18 [0.92-1.51]
T20	2,110	80,119,760	0.03	0.03	0.03	0.00	0.00	0.00	1.11 [0.96-1.29]	1.06 [0.91-1.23]
T21	3,060	80,112,730	0.04	0.04	0.04	0.01	0.01	0.00	1.14 [1.00-1.29]	1.07 [0.94-1.22]
T22	2,680	80,115,030	0.04	0.03	0.03	0.00	0.00	0.00	1.09 [0.95-1.24]	1.01 [0.88-1.16]
T23	2,620	80,108,220	0.03	0.03	0.03	0.00	0.00	0.00	1.03 [0.90-1.18]	0.98 [0.85-1.13]
T24	2,890	80,118,950	0.04	0.04	0.04	0.00	0.00	0.00	0.98 [0.85-1.12]	0.94 [0.82-1.08]
T25	1,170	80,149,350	0.01	0.01	0.01	0.00	0.00	0.00	0.98 [0.79-1.21]	0.91 [0.73-1.13]
T29	560	80,160,900	0.01	0.01	0.01	0.00	0.00	0.00	1.26 [0.94-1.68]	1.20 [0.89-1.62]
T31	6,570	80,029,540	0.09	0.08	0.08	0.01	0.00	0.00	1.05 [0.97-1.15]	1.00 [0.91-1.09]
T36	2,360	80,094,790	0.04	0.03	0.03	0.01	0.01	0.00	1.30 [1.13-1.49]*	1.09 [0.94-1.25]
T37	990	80,146,250	0.02	0.01	0.01	0.00	0.00	0.00	1.40 [1.14-1.73]*	1.20 [0.97-1.50]
T38	4,210	80,093,140	0.07	0.05	0.05	0.02	0.02	0.01	1.34 [1.21-1.48]*	1.09 [0.98-1.21]
T39	43,010	78,802,400	0.61	0.53	0.54	0.08	0.07	−0.01	1.13 [1.09-1.16]*	0.99 [0.95-1.02]
T40	24,340	79,720,540	0.36	0.29	0.30	0.07	0.06	0.00	1.21 [1.16-1.26]*	1.00 [0.95-1.04]
T41	620	80,163,860	0.01	0.01	0.01	0.00	0.00	0.00	1.17 [0.90-1.54]	1.06 [0.80-1.40]
T42	18,000	79,684,780	0.27	0.22	0.22	0.05	0.05	0.00	1.23 [1.17-1.29]*	1.00 [0.95-1.06]
T43	27,020	79,440,670	0.40	0.33	0.33	0.08	0.07	0.01	1.22 [1.17-1.27]*	1.02 [0.97-1.06]
T44	5,520	80,053,800	0.08	0.07	0.07	0.01	0.01	0.00	1.22 [1.11-1.33]*	1.04 [0.94-1.14]
T45	6,510	80,030,970	0.10	0.08	0.08	0.02	0.02	0.01	1.31 [1.21-1.42]*	1.10 [1.01-1.20]
T46	5,400	80,098,330	0.08	0.06	0.06	0.02	0.02	0.00	1.23 [1.12-1.36]*	1.06 [0.96-1.17]

T47	2,740	80,119,560	0.04	0.03	0.03	0.01	0.01	0.00	1.26 [1.11-1.43]*	1.00 [0.88-1.15]
T48	850	80,144,340	0.01	0.01	0.01	0.00	0.00	0.00	1.42 [1.14-1.77]*	1.11 [0.87-1.41]
T49	480	80,149,180	0.01	0.01	0.01	0.00	0.00	0.00	1.30 [0.96-1.77]	1.08 [0.78-1.48]
T50	6,650	79,952,190	0.10	0.08	0.08	0.02	0.02	0.00	1.23 [1.14-1.34]*	1.02 [0.94-1.12]
T51	24,580	79,542,270	0.35	0.30	0.30	0.05	0.05	−0.01	1.16 [1.11-1.21]*	0.98 [0.94-1.02]
T54	830	80,152,230	0.01	0.01	0.01	0.00	0.00	0.00	1.01 [0.79-1.29]	0.94 [0.73-1.20]
T56	620	80,159,030	0.01	0.01	0.01	0.00	0.00	0.00	1.17 [0.90-1.54]	1.05 [0.80-1.39]
T59	1,740	80,132,410	0.02	0.02	0.02	0.00	0.00	0.00	1.08 [0.91-1.27]	0.97 [0.82-1.16]
T63	840	80,150,410	0.01	0.01	0.01	0.00	0.00	0.00	0.99 [0.77-1.26]	0.88 [0.68-1.13]
T65	720	80,152,180	0.01	0.01	0.01	0.00	0.00	0.00	1.14 [0.88-1.47]	0.96 [0.73-1.25]
T68	9,240	80,137,470	0.13	0.11	0.12	0.02	0.02	0.01	1.13 [1.05-1.22]*	1.12 [1.04-1.21]*
T74	680	80,158,250	0.01	0.01	0.01	0.00	0.00	0.00	1.17 [0.90-1.52]	0.95 [0.72-1.25]
T75	620	80,151,990	0.01	0.01	0.01	0.00	0.00	0.00	1.24 [0.95-1.63]	1.13 [0.85-1.49]
T78	14,180	79,777,970	0.30	0.15	0.15	0.15	0.15	0.11	1.99 [1.89-2.09]*	1.58 [1.49-1.66]*
T79	11,930	80,022,060	0.16	0.15	0.15	0.02	0.02	0.01	1.11 [1.04-1.18]*	1.04 [0.97-1.11]
T80	4,980	80,107,410	0.08	0.06	0.06	0.02	0.02	0.02	1.39 [1.27-1.53]*	1.28 [1.17-1.41]*
T81	163,730	77,368,020	2.62	2.01	2.02	0.62	0.61	0.41	1.30 [1.28-1.32]*	1.19 [1.17-1.21]*
T82	39,240	79,719,890	0.63	0.46	0.47	0.16	0.16	0.11	1.34 [1.30-1.39]*	1.21 [1.17-1.25]*
T83	41,320	79,875,030	0.59	0.50	0.51	0.09	0.08	0.05	1.15 [1.12-1.20]*	1.10 [1.06-1.14]*
T84	54,600	79,256,690	0.86	0.65	0.65	0.21	0.21	0.15	1.32 [1.28-1.36]*	1.21 [1.18-1.25]*
T85	33,240	79,734,140	0.52	0.39	0.40	0.13	0.13	0.09	1.32 [1.28-1.37]*	1.21 [1.17-1.26]*
T86	4,710	80,098,320	0.08	0.06	0.06	0.02	0.02	0.02	1.36 [1.24-1.49]*	1.26 [1.15-1.39]*
T87	3,050	80,131,490	0.05	0.04	0.04	0.01	0.01	0.01	1.30 [1.16-1.47]*	1.14 [1.01-1.29]
T88	10,200	79,964,560	0.18	0.12	0.12	0.06	0.06	0.04	1.49 [1.40-1.59]*	1.29 [1.20-1.37]*
T90	8,480	79,964,840	0.13	0.10	0.10	0.03	0.03	0.02	1.31 [1.22-1.41]*	1.19 [1.11-1.29]*
T91	4,080	80,090,070	0.06	0.05	0.05	0.01	0.01	0.00	1.24 [1.11-1.37]*	1.08 [0.97-1.21]
T92	6,870	79,989,050	0.10	0.08	0.08	0.02	0.02	0.01	1.20 [1.11-1.30]*	1.11 [1.02-1.21]
T93	9,080	79,946,010	0.13	0.11	0.11	0.03	0.03	0.02	1.25 [1.16-1.34]*	1.14 [1.06-1.22]*
T94	850	80,156,110	0.01	0.01	0.01	0.00	0.00	0.00	1.24 [0.98-1.56]	1.12 [0.88-1.42]
T95	570	80,151,750	0.01	0.01	0.01	0.00	0.00	0.00	1.31 [0.99-1.72]	1.26 [0.95-1.68]
T98	1,420	80,146,700	0.02	0.02	0.02	0.01	0.01	0.00	1.34 [1.13-1.60]*	1.21 [1.01-1.44]
18+ - U00-U89										
U07	51,760	80,122,450	0.82	0.61	0.65	0.21	0.16	0.09	1.25 [1.21-1.29]*	1.13 [1.09-1.17]*
U80	17,660	80,066,780	0.31	0.20	0.20	0.11	0.10	0.08	1.50 [1.43-1.57]*	1.36 [1.29-1.43]*
U81	1,050	80,167,920	0.02	0.01	0.01	0.00	0.00	0.00	1.18 [0.95-1.46]	1.08 [0.87-1.34]
U82	34,980	80,078,740	0.59	0.40	0.42	0.19	0.17	0.12	1.39 [1.34-1.44]*	1.25 [1.20-1.30]*
U83	20,870	80,120,260	0.34	0.24	0.26	0.10	0.09	0.06	1.34 [1.28-1.40]*	1.20 [1.14-1.26]*
U88	4,320	80,150,500	0.07	0.05	0.05	0.02	0.01	0.01	1.28 [1.15-1.42]*	1.16 [1.04-1.29]*
U89	4,860	80,145,240	0.08	0.06	0.06	0.02	0.02	0.01	1.34 [1.21-1.47]*	1.20 [1.08-1.32]*
18+ - V00-Y99										
V03	6,030	79,967,890	0.08	0.07	0.07	0.01	0.01	0.00	1.10 [1.00-1.20]	1.05 [0.96-1.15]
V04	500	80,157,460	0.01	0.01	0.01	0.00	0.00	0.00	1.18 [0.87-1.61]	1.14 [0.83-1.55]
V09	1,680	80,145,620	0.02	0.02	0.02	0.00	0.01	0.00	1.29 [1.09-1.53]*	1.13 [0.95-1.35]
V13	2,210	80,113,960	0.03	0.03	0.03	0.00	0.00	0.00	0.90 [0.77-1.05]	0.90 [0.77-1.06]
V17	700	80,156,250	0.01	0.01	0.01	0.00	0.00	0.00	0.84 [0.63-1.11]	0.79 [0.59-1.05]
V18	8,350	79,864,680	0.10	0.10	0.10	0.00	0.00	0.00	1.02 [0.94-1.10]	1.00 [0.93-1.09]
V19	1,320	80,128,720	0.02	0.02	0.02	0.00	0.00	0.00	1.01 [0.84-1.23]	0.99 [0.81-1.21]
V23	3,570	80,084,500	0.04	0.05	0.04	0.00	0.00	0.00	0.97 [0.86-1.09]	0.94 [0.83-1.06]
V27	790	80,151,570	0.01	0.01	0.01	0.00	0.00	0.00	0.84 [0.65-1.09]	0.78 [0.59-1.03]
V28	4,120	80,059,270	0.05	0.05	0.05	0.00	0.00	0.00	0.98 [0.88-1.09]	0.93 [0.82-1.04]
V29	1,050	80,136,530	0.01	0.01	0.01	0.00	0.00	0.00	1.06 [0.86-1.32]	1.00 [0.80-1.25]
V43	8,300	79,959,530	0.12	0.10	0.10	0.01	0.01	0.01	1.14 [1.06-1.23]*	1.05 [0.98-1.14]
V44	790	80,152,690	0.01	0.01	0.01	0.00	0.00	0.00	1.10 [0.87-1.41]	1.04 [0.81-1.33]
V47	3,790	80,087,750	0.06	0.05	0.05	0.01	0.01	0.01	1.24 [1.11-1.38]*	1.16 [1.04-1.30]
V48	4,790	80,093,080	0.07	0.06	0.06	0.01	0.01	0.00	1.15 [1.05-1.28]*	1.07 [0.97-1.19]
V49	3,360	80,070,950	0.05	0.04	0.04	0.01	0.00	0.00	1.12 [0.99-1.26]	1.03 [0.92-1.17]
V78	1,880	80,152,350	0.03	0.02	0.02	0.00	0.00	0.00	1.19 [1.01-1.40]	1.12 [0.95-1.32]
V79	590	80,162,900	0.01	0.01	0.01	0.00	0.00	0.00	1.02 [0.76-1.36]	0.89 [0.66-1.21]
V80	4,020	80,057,370	0.05	0.05	0.05	−0.01	−0.01	−0.01	0.88 [0.79-0.99]	0.87 [0.77-0.98]
V82	470	80,166,550	0.01	0.01	0.01	0.00	0.00	0.00	1.52 [1.12-2.07]*	1.47 [1.08-2.00]
V86	720	80,155,990	0.01	0.01	0.01	0.00	0.00	0.00	1.04 [0.80-1.35]	0.96 [0.73-1.26]
V89	1,460	80,099,500	0.02	0.02	0.02	0.00	0.00	0.00	1.28 [1.08-1.53]*	1.19 [0.99-1.42]
W00	7,320	80,044,360	0.10	0.09	0.09	0.01	0.01	0.01	1.10 [1.02-1.19]	1.08 [0.99-1.17]
W01	142,600	78,473,160	2.11	1.75	1.82	0.35	0.28	0.18	1.16 [1.13-1.18]*	1.09 [1.07-1.11]*
W02	2,860	80,021,660	0.04	0.04	0.04	0.00	0.00	0.00	1.01 [0.89-1.15]	0.98 [0.85-1.12]
W03	5,590	79,962,300	0.07	0.07	0.07	0.00	0.00	0.00	1.06 [0.96-1.16]	0.99 [0.90-1.09]
W05	3,200	80,147,700	0.05	0.04	0.04	0.01	0.01	0.01	1.26 [1.11-1.42]*	1.15 [1.01-1.30]
W06	33,580	79,988,210	0.51	0.40	0.42	0.10	0.08	0.05	1.19 [1.15-1.24]*	1.11 [1.07-1.16]*
W07	18,170	80,024,190	0.27	0.22	0.23	0.05	0.04	0.03	1.19 [1.13-1.25]*	1.12 [1.06-1.18]*
W08	4,390	80,115,420	0.07	0.05	0.05	0.01	0.01	0.01	1.24 [1.11-1.37]*	1.14 [1.02-1.27]
W09	760	79,984,690	0.01	0.01	0.01	0.00	0.00	0.00	1.23 [0.97-1.57]	1.17 [0.91-1.50]
W10	58,160	79,379,430	0.85	0.71	0.71	0.14	0.14	0.09	1.19 [1.16-1.23]*	1.11 [1.08-1.15]*
W11	9,410	79,979,400	0.12	0.12	0.11	0.00	0.01	0.00	1.06 [0.99-1.14]	1.02 [0.94-1.10]
W12	540	80,158,480	0.01	0.01	0.01	0.00	0.00	0.00	1.15 [0.86-1.54]	1.14 [0.85-1.54]
W13	4,150	80,018,020	0.05	0.05	0.05	0.00	0.00	0.00	1.05 [0.94-1.17]	0.98 [0.88-1.10]
W17	7,440	79,948,390	0.10	0.09	0.09	0.01	0.01	0.01	1.16 [1.07-1.25]*	1.09 [1.00-1.18]
W18	96,200	79,431,120	1.46	1.16	1.20	0.30	0.25	0.16	1.21 [1.18-1.24]*	1.13 [1.10-1.15]*
W19	225,320	78,191,600	3.43	2.76	2.89	0.66	0.54	0.37	1.19 [1.17-1.21]*	1.12 [1.10-1.14]*
W20	4,700	80,059,100	0.06	0.06	0.06	0.00	0.00	0.00	1.08 [0.97-1.19]	1.01 [0.91-1.12]
W21	2,580	80,062,700	0.03	0.03	0.03	0.00	0.00	0.00	1.11 [0.97-1.27]	1.08 [0.94-1.24]
W22	22,440	79,761,900	0.33	0.27	0.28	0.05	0.05	0.02	1.17 [1.12-1.23]*	1.08 [1.03-1.13]*
W23	9,910	79,916,890	0.13	0.12	0.12	0.01	0.01	0.00	1.08 [1.01-1.16]	1.03 [0.96-1.11]
W25	8,020	79,891,260	0.11	0.10	0.10	0.01	0.01	0.00	1.08 [1.00-1.17]	1.02 [0.94-1.10]
W26	8,340	80,015,690	0.11	0.10	0.10	0.01	0.01	0.00	1.12 [1.04-1.20]*	1.03 [0.96-1.12]
W27	3,900	80,071,850	0.05	0.05	0.05	0.00	0.00	0.00	1.03 [0.92-1.16]	0.98 [0.87-1.10]
W29	4,750	80,086,990	0.06	0.06	0.06	0.00	0.00	0.00	0.98 [0.88-1.08]	0.95 [0.85-1.05]
W31	4,160	80,070,110	0.05	0.05	0.05	0.00	0.00	0.00	1.03 [0.92-1.14]	1.02 [0.91-1.14]
W44	9,060	79,939,830	0.14	0.11	0.11	0.03	0.03	0.02	1.30 [1.21-1.40]*	1.18 [1.09-1.27]*
W45	4,870	80,026,510	0.07	0.06	0.06	0.01	0.01	0.00	1.13 [1.03-1.25]	1.05 [0.95-1.16]
W46	910	80,167,010	0.01	0.01	0.01	0.00	0.00	0.00	1.17 [0.93-1.46]	0.95 [0.75-1.21]
W49	1,360	80,128,420	0.02	0.02	0.02	0.00	0.00	0.00	1.29 [1.08-1.54]*	1.22 [1.01-1.47]
W50	6,270	79,975,140	0.09	0.08	0.08	0.01	0.01	0.01	1.17 [1.07-1.27]*	1.08 [0.99-1.18]
W51	3,920	80,017,140	0.05	0.05	0.05	0.01	0.01	0.00	1.11 [1.00-1.24]	1.05 [0.94-1.17]
W54	7,810	80,028,180	0.10	0.10	0.10	0.01	0.00	0.00	1.04 [0.97-1.13]	0.97 [0.90-1.05]
W55	4,530	80,099,020	0.06	0.06	0.06	0.00	0.00	−0.01	1.00 [0.90-1.11]	0.91 [0.81-1.01]
W57	5,080	80,086,930	0.08	0.06	0.06	0.03	0.03	0.02	1.45 [1.32-1.59]*	1.31 [1.19-1.44]*
W60	500	80,164,570	0.01	0.01	0.01	0.00	0.00	0.00	1.47 [1.10-1.96]*	1.37 [1.02-1.84]
W64	1,130	80,160,880	0.02	0.01	0.01	0.00	0.00	0.00	1.10 [0.89-1.36]	1.02 [0.82-1.26]
W78	3,640	80,163,690	0.05	0.04	0.05	0.01	0.01	0.00	1.11 [0.98-1.25]	1.06 [0.94-1.20]
W79	5,140	80,141,540	0.08	0.06	0.06	0.02	0.02	0.01	1.26 [1.14-1.39]*	1.17 [1.06-1.29]*
W80	2,090	80,165,370	0.03	0.03	0.03	0.01	0.01	0.00	1.23 [1.05-1.44]*	1.12 [0.95-1.31]
W84	2,140	80,167,710	0.03	0.03	0.03	0.00	0.00	0.00	1.06 [0.91-1.24]	0.99 [0.85-1.17]
X09	700	80,156,530	0.01	0.01	0.01	0.00	0.00	0.00	1.02 [0.78-1.34]	0.98 [0.75-1.29]
X10	1,330	80,140,210	0.02	0.02	0.02	0.00	0.00	0.00	1.19 [0.99-1.43]	1.09 [0.90-1.32]
X12	1,180	80,147,230	0.02	0.01	0.02	0.00	0.00	0.00	1.01 [0.82-1.24]	0.93 [0.75-1.15]
X16	900	80,163,270	0.01	0.01	0.01	0.00	0.00	0.00	1.14 [0.89-1.45]	1.05 [0.82-1.35]
X23	1,070	80,151,580	0.02	0.01	0.01	0.00	0.00	0.00	1.19 [0.97-1.47]	1.11 [0.89-1.37]

X31	1,300	80,165,890	0.02	0.02	0.02	0.00	0.00	0.00	1.00 [0.81-1.24]	0.99 [0.80-1.23]
X40	9,270	79,921,370	0.13	0.11	0.12	0.02	0.02	0.00	1.16 [1.08-1.24]*	1.01 [0.94-1.08]
X41	6,870	79,990,790	0.10	0.08	0.08	0.02	0.02	0.01	1.26 [1.17-1.37]*	1.06 [0.97-1.15]
X42	7,390	80,076,470	0.11	0.09	0.09	0.03	0.02	0.00	1.28 [1.18-1.38]*	1.04 [0.96-1.13]
X43	1,290	80,149,660	0.02	0.02	0.02	0.00	0.00	0.00	1.20 [0.98-1.46]	1.08 [0.88-1.32]
X44	7,950	80,014,310	0.12	0.09	0.10	0.03	0.03	0.01	1.29 [1.20-1.39]*	1.10 [1.02-1.19]
X45	3,020	80,086,820	0.04	0.04	0.04	0.01	0.01	0.00	1.16 [1.03-1.31]	0.99 [0.87-1.13]
X47	1,020	80,151,400	0.01	0.01	0.01	0.00	0.00	0.00	1.10 [0.89-1.37]	1.00 [0.80-1.25]
X49	2,250	80,103,320	0.04	0.03	0.03	0.01	0.01	0.01	1.38 [1.20-1.58]*	1.20 [1.04-1.38]
X50	15,580	79,894,920	0.22	0.19	0.19	0.03	0.03	0.01	1.15 [1.09-1.21]*	1.05 [1.00-1.12]
X51	1,190	80,153,010	0.02	0.01	0.01	0.00	0.00	0.00	1.14 [0.94-1.39]	1.10 [0.90-1.34]
X58	4,120	80,114,110	0.07	0.05	0.05	0.02	0.02	0.02	1.45 [1.31-1.61]*	1.28 [1.15-1.43]*
X59	58,500	79,077,690	0.89	0.71	0.72	0.18	0.17	0.10	1.24 [1.20-1.27]*	1.13 [1.10-1.17]*
X60	35,590	79,033,630	0.50	0.44	0.45	0.06	0.06	−0.01	1.13 [1.09-1.17]*	0.98 [0.95-1.02]
X61	30,370	79,346,610	0.45	0.37	0.37	0.08	0.08	0.00	1.21 [1.16-1.25]*	1.00 [0.96-1.05]
X62	16,980	79,824,230	0.25	0.21	0.21	0.04	0.04	−0.01	1.19 [1.13-1.25]*	0.97 [0.92-1.02]
X63	4,120	80,081,820	0.06	0.05	0.05	0.01	0.01	0.00	1.21 [1.09-1.34]*	1.01 [0.90-1.12]
X64	13,370	79,780,540	0.20	0.16	0.16	0.04	0.04	0.00	1.23 [1.16-1.30]*	1.02 [0.96-1.09]
X65	22,350	79,634,790	0.32	0.27	0.27	0.05	0.04	−0.01	1.16 [1.11-1.22]*	0.98 [0.93-1.02]
X69	1,210	80,140,730	0.01	0.02	0.02	0.00	0.00	0.00	0.94 [0.77-1.16]	0.81 [0.65-1.00]
X70	980	80,156,280	0.01	0.01	0.01	0.00	0.00	0.00	0.99 [0.79-1.24]	0.87 [0.69-1.10]
X78	7,580	79,991,860	0.10	0.09	0.09	0.01	0.01	−0.01	1.09 [1.00-1.17]	0.90 [0.82-0.97]*
X83	700	80,162,020	0.01	0.01	0.01	0.00	0.00	0.00	1.13 [0.88-1.47]	0.97 [0.74-1.27]
X84	1,070	80,149,800	0.01	0.01	0.01	0.00	0.00	0.00	1.04 [0.84-1.28]	0.81 [0.65-1.02]
X99	4,390	80,057,570	0.06	0.05	0.05	0.00	0.00	0.00	1.09 [0.98-1.21]	1.02 [0.92-1.14]
Y00	2,320	80,099,550	0.03	0.03	0.03	0.00	0.00	0.00	1.12 [0.97-1.29]	0.99 [0.85-1.15]
Y04	18,850	79,553,480	0.26	0.23	0.23	0.03	0.03	0.01	1.12 [1.07-1.18]*	1.02 [0.97-1.08]
Y07	770	80,153,460	0.01	0.01	0.01	0.00	0.00	0.00	1.01 [0.78-1.30]	0.82 [0.63-1.08]
Y08	940	80,148,860	0.01	0.01	0.01	0.00	0.00	0.00	1.00 [0.79-1.26]	0.93 [0.73-1.19]
Y09	2,820	80,069,120	0.04	0.03	0.03	0.00	0.00	0.00	1.13 [1.00-1.29]	1.02 [0.89-1.16]
Y40	17,840	79,974,450	0.32	0.20	0.21	0.11	0.11	0.08	1.52 [1.45-1.60]*	1.34 [1.27-1.41]*
Y41	3,750	80,128,630	0.06	0.04	0.04	0.02	0.02	0.01	1.45 [1.30-1.62]*	1.27 [1.14-1.42]*
Y42	14,950	80,020,910	0.25	0.17	0.18	0.08	0.08	0.04	1.43 [1.36-1.51]*	1.20 [1.14-1.27]*
Y43	31,040	79,949,230	0.46	0.37	0.38	0.09	0.09	0.07	1.23 [1.18-1.28]*	1.18 [1.13-1.22]*
Y44	10,690	80,085,470	0.16	0.13	0.13	0.04	0.03	0.02	1.26 [1.18-1.34]*	1.16 [1.08-1.24]*
Y45	24,700	79,930,580	0.40	0.29	0.29	0.11	0.10	0.07	1.35 [1.30-1.41]*	1.20 [1.15-1.25]*
Y46	3,410	80,132,080	0.06	0.04	0.04	0.02	0.02	0.01	1.40 [1.25-1.57]*	1.25 [1.11-1.40]*
Y47	1,670	80,155,480	0.03	0.02	0.02	0.01	0.01	0.00	1.37 [1.16-1.62]*	1.22 [1.03-1.45]
Y48	1,920	80,147,210	0.03	0.02	0.02	0.01	0.01	0.00	1.32 [1.13-1.54]*	1.16 [0.99-1.35]
Y49	5,990	80,111,520	0.10	0.07	0.07	0.03	0.02	0.02	1.35 [1.24-1.47]*	1.23 [1.12-1.34]*
Y51	9,330	80,088,160	0.14	0.11	0.11	0.03	0.03	0.02	1.27 [1.18-1.36]*	1.18 [1.10-1.27]*
Y52	22,390	80,003,590	0.35	0.26	0.26	0.09	0.09	0.06	1.34 [1.28-1.40]*	1.21 [1.16-1.27]*
Y53	4,190	80,144,200	0.07	0.05	0.05	0.02	0.02	0.01	1.39 [1.25-1.54]*	1.19 [1.07-1.32]*
Y54	17,560	80,071,460	0.29	0.20	0.21	0.08	0.08	0.05	1.37 [1.30-1.44]*	1.23 [1.17-1.30]*
Y55	2,280	80,149,480	0.04	0.03	0.03	0.01	0.01	0.01	1.43 [1.25-1.63]*	1.20 [1.04-1.39]
Y56	1,780	80,138,740	0.04	0.02	0.02	0.02	0.02	0.01	1.87 [1.62-2.17]*	1.63 [1.40-1.89]*
Y57	9,910	80,089,770	0.16	0.12	0.12	0.05	0.04	0.03	1.38 [1.29-1.47]*	1.21 [1.13-1.30]*
Y59	900	80,161,790	0.02	0.01	0.01	0.01	0.01	0.00	1.55 [1.25-1.92]*	1.38 [1.11-1.72]*
Y60	12,400	80,062,560	0.19	0.15	0.15	0.04	0.04	0.03	1.27 [1.20-1.35]*	1.17 [1.10-1.25]*
Y65	1,620	80,154,170	0.02	0.02	0.02	0.00	0.00	0.00	1.24 [1.05-1.47]	1.13 [0.95-1.35]
Y71	730	80,163,590	0.01	0.01	0.01	0.00	0.00	0.00	1.59 [1.26-2.02]*	1.42 [1.11-1.82]*
Y73	740	80,164,620	0.01	0.01	0.01	0.00	0.00	0.00	0.94 [0.72-1.23]	0.89 [0.67-1.17]
Y76	460	80,162,470	0.01	0.01	0.01	0.00	0.00	0.00	1.37 [1.01-1.85]	1.26 [0.92-1.71]
Y77	820	80,164,790	0.01	0.01	0.01	0.00	0.00	0.00	0.98 [0.77-1.26]	0.91 [0.70-1.17]
Y79	1,750	80,143,830	0.03	0.02	0.02	0.00	0.01	0.00	1.24 [1.06-1.46]*	1.16 [0.98-1.37]
Y82	600	80,163,540	0.01	0.01	0.01	0.00	0.00	0.00	1.27 [0.97-1.68]	1.16 [0.88-1.54]
Y83	217,020	76,786,630	3.48	2.68	2.70	0.80	0.78	0.52	1.29 [1.27-1.31]*	1.17 [1.16-1.19]*
Y84	49,500	79,773,520	0.75	0.59	0.60	0.16	0.15	0.10	1.25 [1.22-1.29]*	1.16 [1.12-1.19]*
Y85	5,330	80,057,740	0.08	0.06	0.06	0.02	0.02	0.01	1.30 [1.19-1.42]*	1.15 [1.05-1.27]*
Y86	9,340	79,943,580	0.13	0.11	0.11	0.02	0.02	0.01	1.20 [1.12-1.29]*	1.10 [1.02-1.18]
Y87	1,590	80,133,190	0.02	0.02	0.02	0.01	0.01	0.00	1.34 [1.14-1.57]*	1.18 [0.99-1.40]
Y88	1,800	80,143,330	0.03	0.02	0.02	0.00	0.00	0.00	1.18 [1.00-1.38]	1.01 [0.85-1.20]
Y89	590	80,159,310	0.01	0.01	0.01	0.00	0.00	0.00	1.31 [1.00-1.72]	1.19 [0.90-1.57]
Y90	1,020	80,158,460	0.01	0.01	0.01	0.00	0.00	0.00	1.17 [0.94-1.44]	1.07 [0.86-1.33]
Y91	2,240	80,123,340	0.03	0.03	0.03	0.00	0.00	0.00	1.17 [1.01-1.35]	1.07 [0.92-1.24]
Y95	117,240	79,889,980	1.81	1.39	1.46	0.42	0.35	0.22	1.24 [1.21-1.27]*	1.14 [1.11-1.16]*
18+ - Z00-Z99										
Z00	4,760	80,053,020	0.07	0.06	0.06	0.01	0.01	0.01	1.22 [1.11-1.34]*	1.13 [1.03-1.25]
Z01	13,740	79,268,950	0.23	0.16	0.16	0.06	0.07	0.04	1.41 [1.34-1.49]*	1.22 [1.15-1.29]*
Z02	19,070	79,974,960	0.27	0.23	0.23	0.04	0.04	0.02	1.17 [1.11-1.23]*	1.07 [1.02-1.13]*
Z03	131,600	77,667,030	2.13	1.60	1.61	0.54	0.52	0.33	1.32 [1.30-1.35]*	1.19 [1.16-1.21]*
Z04	20,310	79,330,850	0.31	0.24	0.24	0.07	0.07	0.04	1.27 [1.21-1.33]*	1.16 [1.10-1.21]*
Z08	43,850	79,379,910	0.59	0.54	0.54	0.04	0.04	0.03	1.08 [1.05-1.12]*	1.05 [1.02-1.09]*
Z09	80,390	78,605,890	1.25	0.97	0.97	0.28	0.28	0.17	1.29 [1.26-1.32]*	1.16 [1.13-1.19]*
Z11	250,740	79,677,600	3.81	3.00	3.14	0.81	0.67	0.33	1.21 [1.20-1.23]*	1.10 [1.08-1.11]*
Z12	62,920	79,548,940	0.88	0.77	0.76	0.11	0.12	0.10	1.16 [1.13-1.19]*	1.13 [1.09-1.16]*
Z13	159,270	78,022,810	2.50	1.94	1.98	0.55	0.52	0.34	1.26 [1.24-1.28]*	1.16 [1.14-1.18]*
Z20	8,290	80,137,560	0.13	0.10	0.10	0.03	0.02	0.02	1.24 [1.15-1.34]*	1.13 [1.05-1.23]*
Z22	78,700	79,358,270	1.34	0.92	0.95	0.43	0.39	0.31	1.41 [1.38-1.45]*	1.29 [1.26-1.33]*
Z24	1,540	80,113,220	0.02	0.02	0.02	0.00	0.00	0.00	1.13 [0.95-1.35]	1.12 [0.93-1.33]
Z27	3,010	80,085,420	0.04	0.04	0.04	0.00	0.00	0.00	1.06 [0.93-1.20]	1.01 [0.89-1.15]
Z29	42,460	79,728,430	0.65	0.51	0.52	0.15	0.13	0.09	1.25 [1.21-1.29]*	1.15 [1.11-1.19]*
Z30	52,160	77,607,840	0.76	0.65	0.65	0.11	0.11	0.07	1.17 [1.14-1.21]*	1.10 [1.06-1.13]*
Z31	950	80,060,180	0.01	0.01	0.01	0.00	0.00	0.00	1.06 [0.85-1.33]	0.98 [0.77-1.23]
Z32	3,720	79,996,490	0.06	0.04	0.05	0.01	0.01	0.01	1.23 [1.10-1.37]*	1.13 [1.01-1.26]
Z33	34,560	79,037,830	0.55	0.41	0.43	0.13	0.11	0.06	1.26 [1.22-1.31]*	1.12 [1.07-1.16]*
Z34	94,890	77,369,210	1.43	1.18	1.28	0.25	0.15	0.09	1.12 [1.09-1.14]*	1.07 [1.04-1.09]*
Z35	84,850	78,583,120	1.22	1.05	1.13	0.17	0.09	0.05	1.08 [1.05-1.11]*	1.05 [1.02-1.07]*
Z36	17,740	78,969,760	0.25	0.22	0.23	0.03	0.03	0.02	1.12 [1.07-1.18]*	1.09 [1.03-1.14]*
Z37	324,930	65,865,400	6.34	4.62	5.67	1.72	0.67	0.49	1.12 [1.10-1.13]*	1.08 [1.07-1.10]*
Z38	960	77,989,370	0.01	0.01	0.01	0.00	0.00	0.00	1.18 [0.95-1.47]	1.18 [0.94-1.48]
Z39	28,840	79,114,330	0.41	0.35	0.38	0.06	0.04	0.03	1.09 [1.05-1.14]*	1.07 [1.03-1.12]*
Z40	5,140	80,095,860	0.07	0.06	0.06	0.01	0.01	0.00	1.11 [1.00-1.22]	1.05 [0.95-1.16]
Z41	4,360	80,004,280	0.07	0.05	0.05	0.01	0.01	0.01	1.24 [1.12-1.37]*	1.13 [1.02-1.26]
Z42	14,240	79,876,310	0.20	0.17	0.17	0.03	0.03	0.02	1.17 [1.11-1.24]*	1.10 [1.04-1.17]*
Z43	19,240	79,830,190	0.28	0.23	0.23	0.05	0.05	0.04	1.24 [1.18-1.30]*	1.17 [1.11-1.23]*
Z45	42,100	79,622,760	0.62	0.51	0.51	0.11	0.12	0.07	1.23 [1.19-1.27]*	1.13 [1.10-1.17]*
Z46	54,790	79,317,560	0.78	0.67	0.67	0.10	0.10	0.07	1.15 [1.12-1.19]*	1.10 [1.06-1.13]*
Z47	26,200	79,136,700	0.38	0.32	0.32	0.06	0.06	0.03	1.19 [1.14-1.24]*	1.10 [1.06-1.15]*
Z48	12,110	79,815,110	0.18	0.14	0.14	0.04	0.04	0.03	1.30 [1.22-1.38]*	1.21 [1.14-1.29]*
Z49	8,500	80,071,260	0.14	0.10	0.10	0.04	0.05	0.04	1.46 [1.37-1.57]*	1.35 [1.25-1.45]*
Z50	322,690	78,181,860	4.91	3.96	4.13	0.95	0.77	0.48	1.19 [1.17-1.20]*	1.11 [1.09-1.12]*
Z51	378,850	77,113,120	5.80	4.72	4.81	1.08	1.00	0.66	1.21 [1.19-1.22]*	1.13 [1.12-1.14]*
Z52	2,000	80,130,110	0.02	0.02	0.02	0.00	0.00	0.00	1.02 [0.87-1.19]	1.03 [0.88-1.21]
Z53	304,520	74,616,060	5.11	3.86	3.87	1.26	1.25	0.84	1.32 [1.31-1.34]*	1.20 [1.18-1.21]*
Z54	2,900	80,101,430	0.05	0.03	0.03	0.01	0.01	0.01	1.35 [1.20-1.53]*	1.29 [1.14-1.46]*
Z56	4,120	80,117,970	0.06	0.05	0.05	0.01	0.01	0.00	1.16 [1.05-1.29]*	1.00 [0.89-1.11]

Z57	12,070	80,101,130	0.18	0.14	0.14	0.04	0.04	0.03	1.26 [1.18-1.34]*	1.16 [1.09-1.24]*
Z58	6,910	80,132,670	0.11	0.08	0.09	0.02	0.02	0.01	1.23 [1.13-1.33]*	1.09 [1.00-1.19]
Z59	8,860	80,068,980	0.12	0.11	0.11	0.01	0.01	0.00	1.09 [1.01-1.17]	0.98 [0.91-1.06]
Z60	104,450	79,022,890	1.53	1.28	1.30	0.26	0.24	0.15	1.18 [1.16-1.21]*	1.11 [1.08-1.13]*
Z61	2,500	80,118,310	0.03	0.03	0.03	0.00	0.00	−0.01	1.03 [0.89-1.18]	0.86 [0.74-0.99]
Z63	27,110	79,826,810	0.40	0.33	0.34	0.07	0.06	0.01	1.17 [1.12-1.22]*	1.02 [0.98-1.07]
Z64	810	80,131,510	0.01	0.01	0.01	0.00	0.00	0.00	1.01 [0.79-1.29]	0.90 [0.69-1.17]
Z65	2,280	80,137,010	0.03	0.03	0.03	0.00	0.00	0.00	1.14 [0.99-1.32]	0.99 [0.86-1.15]
Z71	26,060	79,816,380	0.39	0.31	0.32	0.08	0.07	0.04	1.22 [1.16-1.27]*	1.11 [1.06-1.16]*
Z72	224,150	74,263,690	3.46	2.92	2.87	0.54	0.60	0.29	1.21 [1.19-1.22]*	1.09 [1.07-1.11]*
Z73	54,340	79,913,130	0.81	0.65	0.68	0.15	0.12	0.07	1.18 [1.14-1.22]*	1.09 [1.06-1.12]*
Z74	41,950	79,925,930	0.61	0.51	0.52	0.11	0.09	0.07	1.17 [1.13-1.21]*	1.12 [1.08-1.16]*
Z75	82,230	79,782,870	1.18	1.00	1.06	0.18	0.12	0.08	1.12 [1.09-1.15]*	1.07 [1.04-1.10]*
Z76	5,930	79,953,090	0.09	0.07	0.07	0.01	0.01	0.01	1.17 [1.07-1.28]*	1.15 [1.05-1.26]*
Z80	89,420	78,940,610	1.29	1.10	1.10	0.19	0.19	0.11	1.17 [1.15-1.20]*	1.09 [1.07-1.12]*
Z81	8,810	80,068,070	0.13	0.11	0.11	0.02	0.02	0.00	1.16 [1.08-1.24]*	1.00 [0.92-1.07]
Z82	166,430	77,553,150	2.54	2.06	2.07	0.48	0.47	0.26	1.23 [1.21-1.25]*	1.11 [1.09-1.13]*
Z83	61,650	79,377,470	0.94	0.74	0.77	0.20	0.17	0.11	1.22 [1.19-1.26]*	1.13 [1.10-1.16]*
Z84	5,110	80,078,420	0.08	0.06	0.06	0.02	0.02	0.01	1.25 [1.14-1.37]*	1.18 [1.07-1.30]*
Z85	318,270	76,715,550	4.81	4.01	4.12	0.80	0.69	0.45	1.17 [1.15-1.18]*	1.10 [1.09-1.12]*
Z86	992,770	70,039,250	17.52	13.44	13.85	4.07	3.67	2.15	1.26 [1.26-1.27]*	1.14 [1.13-1.15]*
Z87	475,440	74,393,440	8.00	6.04	6.18	1.96	1.82	1.08	1.30 [1.28-1.31]*	1.16 [1.14-1.17]*
Z88	493,180	73,415,660	9.21	6.18	6.30	3.04	2.91	1.97	1.46 [1.45-1.48]*	1.27 [1.26-1.29]*
Z89	14,800	80,043,520	0.23	0.18	0.18	0.05	0.05	0.03	1.29 [1.22-1.36]*	1.17 [1.10-1.24]*
Z90	273,740	77,718,280	4.22	3.37	3.45	0.85	0.77	0.42	1.22 [1.21-1.24]*	1.11 [1.09-1.12]*
Z91	184,150	77,895,770	3.39	2.14	2.20	1.25	1.19	0.76	1.54 [1.52-1.56]*	1.29 [1.27-1.31]*
Z92	646,880	74,685,820	10.53	8.26	8.50	2.27	2.02	1.21	1.24 [1.23-1.25]*	1.13 [1.12-1.14]*
Z93	60,630	79,615,330	0.90	0.73	0.74	0.17	0.16	0.12	1.22 [1.19-1.26]*	1.15 [1.12-1.18]*
Z94	11,670	79,976,850	0.19	0.14	0.13	0.06	0.06	0.05	1.42 [1.33-1.50]*	1.32 [1.24-1.40]*
Z95	210,610	77,719,030	3.26	2.59	2.64	0.67	0.62	0.37	1.23 [1.22-1.25]*	1.13 [1.11-1.15]*
Z96	409,870	76,418,260	6.46	5.13	5.31	1.33	1.15	0.79	1.22 [1.20-1.23]*	1.14 [1.13-1.15]*
Z97	39,340	79,832,180	0.61	0.47	0.48	0.15	0.14	0.09	1.29 [1.24-1.33]*	1.16 [1.12-1.21]*
Z98	53,090	79,569,170	0.84	0.63	0.64	0.21	0.20	0.11	1.32 [1.28-1.36]*	1.16 [1.12-1.19]*
Z99	65,050	79,711,970	1.10	0.76	0.77	0.34	0.32	0.21	1.42 [1.38-1.45]*	1.23 [1.20-1.27]*
40+ - A00-B99										
A02	940	51,040,700	0.02	0.02	0.02	0.00	0.00	0.00	1.27 [1.02-1.58]	1.12 [0.90-1.41]
A04	45,010	50,557,300	1.10	0.85	0.86	0.25	0.24	0.16	1.28 [1.24-1.32]*	1.17 [1.14-1.21]*
A08	20,340	50,890,030	0.51	0.38	0.39	0.13	0.12	0.08	1.31 [1.25-1.38]*	1.17 [1.12-1.23]*
A09	161,490	50,169,410	4.07	3.04	3.14	1.04	0.93	0.54	1.30 [1.27-1.32]*	1.15 [1.13-1.17]*
A15	880	51,045,060	0.02	0.02	0.02	0.00	0.00	0.00	1.29 [1.03-1.61]	1.17 [0.93-1.48]
A16	1,770	51,026,060	0.05	0.03	0.03	0.02	0.01	0.01	1.43 [1.23-1.67]*	1.30 [1.11-1.52]*
A18	850	51,036,800	0.02	0.02	0.02	0.01	0.01	0.00	1.35 [1.08-1.68]*	1.28 [1.02-1.61]
A31	780	51,056,100	0.02	0.01	0.01	0.01	0.01	0.01	1.69 [1.36-2.12]*	1.43 [1.13-1.80]*
A40	8,830	51,006,130	0.23	0.16	0.16	0.07	0.06	0.05	1.39 [1.29-1.49]*	1.29 [1.20-1.39]*
A41	179,460	50,379,250	4.38	3.39	3.50	0.99	0.88	0.63	1.25 [1.23-1.27]*	1.17 [1.15-1.19]*
A46	600	51,045,250	0.02	0.01	0.01	0.01	0.01	0.01	2.09 [1.64-2.66]*	1.97 [1.54-2.53]*
A48	790	51,051,600	0.02	0.01	0.02	0.00	0.00	0.00	1.21 [0.95-1.55]	1.16 [0.91-1.49]
A49	24,020	50,862,880	0.61	0.44	0.45	0.16	0.15	0.11	1.34 [1.28-1.40]*	1.23 [1.18-1.29]*
A86	990	51,051,740	0.02	0.02	0.02	0.00	0.00	0.00	1.18 [0.95-1.47]	1.08 [0.86-1.35]
A87	900	51,018,090	0.02	0.02	0.02	0.01	0.01	0.00	1.38 [1.12-1.71]*	1.19 [0.96-1.49]
B00	4,330	51,004,460	0.13	0.08	0.08	0.06	0.06	0.05	1.73 [1.58-1.91]*	1.54 [1.40-1.70]*
B01	590	51,042,270	0.02	0.01	0.01	0.00	0.00	0.00	1.43 [1.10-1.87]*	1.29 [0.99-1.70]
B02	12,700	50,956,510	0.33	0.23	0.23	0.10	0.09	0.07	1.40 [1.32-1.49]*	1.29 [1.21-1.37]*
B07	4,200	50,950,770	0.11	0.08	0.08	0.03	0.03	0.02	1.39 [1.26-1.54]*	1.28 [1.15-1.42]*
B15	480	51,052,240	0.01	0.01	0.01	0.00	0.00	0.00	1.35 [1.00-1.82]	1.26 [0.93-1.72]
B16	920	51,033,550	0.02	0.02	0.02	0.01	0.01	0.01	1.54 [1.25-1.90]*	1.45 [1.17-1.81]*
B17	1,480	51,024,580	0.04	0.03	0.03	0.01	0.01	0.01	1.52 [1.29-1.80]*	1.35 [1.13-1.60]*
B18	6,420	50,927,850	0.16	0.12	0.12	0.04	0.04	0.02	1.30 [1.20-1.41]*	1.18 [1.08-1.29]*
B25	1,140	51,049,320	0.03	0.02	0.02	0.01	0.01	0.01	1.45 [1.19-1.75]*	1.32 [1.09-1.61]*
B34	17,310	50,733,850	0.43	0.32	0.33	0.11	0.11	0.06	1.33 [1.27-1.40]*	1.16 [1.10-1.22]*
B35	5,050	51,011,710	0.15	0.09	0.09	0.07	0.06	0.05	1.71 [1.57-1.87]*	1.52 [1.39-1.67]*
B36	1,970	51,049,810	0.06	0.03	0.03	0.03	0.03	0.02	1.77 [1.53-2.05]*	1.58 [1.36-1.84]*
B37	65,140	50,617,320	1.69	1.20	1.23	0.49	0.46	0.29	1.38 [1.34-1.41]*	1.21 [1.18-1.24]*
B44	2,580	51,040,820	0.08	0.04	0.04	0.04	0.04	0.03	1.90 [1.68-2.14]*	1.50 [1.32-1.70]*
B49	1,560	51,051,710	0.04	0.03	0.03	0.01	0.01	0.01	1.51 [1.29-1.78]*	1.39 [1.18-1.64]*
B59	860	51,057,220	0.02	0.02	0.02	0.01	0.01	0.01	1.39 [1.12-1.73]*	1.30 [1.04-1.62]
B80	730	51,050,390	0.02	0.01	0.01	0.00	0.00	0.00	1.21 [0.95-1.54]	1.14 [0.88-1.47]
B86	1,410	51,045,110	0.06	0.02	0.02	0.03	0.03	0.03	2.65 [2.26-3.12]*	2.49 [2.11-2.94]*
B90	700	51,054,280	0.02	0.01	0.01	0.01	0.01	0.00	1.39 [1.08-1.79]*	1.25 [0.97-1.62]
B91	1,180	51,043,880	0.03	0.02	0.02	0.01	0.01	0.01	1.33 [1.09-1.62]*	1.29 [1.06-1.59]
B94	1,290	51,048,630	0.03	0.02	0.02	0.01	0.01	0.00	1.33 [1.11-1.60]*	1.17 [0.97-1.41]
B95	88,090	50,078,410	2.43	1.61	1.63	0.82	0.81	0.67	1.50 [1.46-1.53]*	1.38 [1.35-1.41]*
B96	200,020	49,133,560	4.99	3.87	4.02	1.12	0.98	0.64	1.24 [1.22-1.26]*	1.15 [1.13-1.17]*
B97	34,790	50,878,560	0.88	0.64	0.68	0.24	0.20	0.11	1.30 [1.25-1.35]*	1.14 [1.10-1.19]*
B98	20,340	50,916,460	0.48	0.38	0.39	0.09	0.08	0.05	1.21 [1.15-1.27]*	1.12 [1.07-1.18]*
B99	5,750	51,040,400	0.14	0.11	0.11	0.03	0.02	0.02	1.21 [1.10-1.33]*	1.12 [1.02-1.24]
40+ - C00-D49										
C00	740	51,051,840	0.02	0.01	0.01	0.00	0.00	0.00	1.10 [0.85-1.42]	1.05 [0.81-1.37]
C01	1,620	51,050,510	0.03	0.03	0.03	0.00	0.00	0.00	1.07 [0.90-1.27]	1.04 [0.87-1.24]
C02	2,450	51,036,110	0.06	0.05	0.04	0.01	0.01	0.01	1.32 [1.16-1.51]*	1.27 [1.11-1.46]*
C03	540	51,058,060	0.01	0.01	0.01	0.01	0.00	0.00	1.44 [1.09-1.91]	1.42 [1.07-1.88]
C04	760	51,053,070	0.02	0.01	0.01	0.00	0.00	0.00	1.12 [0.87-1.44]	1.06 [0.82-1.37]
C05	780	51,053,970	0.02	0.01	0.01	0.00	0.00	0.00	1.32 [1.04-1.68]	1.27 [0.99-1.62]
C06	1,110	51,052,070	0.03	0.02	0.02	0.01	0.00	0.00	1.21 [0.99-1.48]	1.13 [0.92-1.39]
C07	1,070	51,050,250	0.02	0.02	0.02	0.00	0.00	0.00	1.01 [0.81-1.25]	0.97 [0.78-1.21]
C09	2,050	51,041,720	0.04	0.04	0.04	0.00	0.01	0.00	1.15 [0.99-1.33]	1.09 [0.93-1.27]
C10	1,040	51,056,350	0.02	0.02	0.02	0.00	0.00	0.00	1.16 [0.94-1.44]	1.09 [0.87-1.35]
C11	510	51,057,520	0.01	0.01	0.01	0.00	0.00	0.00	1.36 [1.01-1.83]	1.31 [0.97-1.77]
C12	560	51,058,430	0.01	0.01	0.01	0.00	0.00	0.00	1.35 [1.02-1.78]	1.31 [0.99-1.74]
C13	640	51,059,530	0.01	0.01	0.01	0.00	0.00	0.00	1.22 [0.93-1.60]	1.20 [0.91-1.58]
C14	1,170	51,055,760	0.03	0.02	0.02	0.00	0.00	0.00	1.06 [0.87-1.30]	1.00 [0.81-1.23]
C15	16,270	51,004,350	0.36	0.31	0.31	0.05	0.05	0.04	1.17 [1.10-1.23]*	1.13 [1.07-1.19]*
C16	13,070	51,007,200	0.28	0.25	0.25	0.02	0.02	0.02	1.09 [1.02-1.16]*	1.06 [0.99-1.13]
C17	2,640	51,047,120	0.06	0.05	0.05	0.01	0.01	0.01	1.13 [0.99-1.29]	1.10 [0.96-1.26]
C18	45,170	50,672,280	0.91	0.89	0.90	0.02	0.01	0.00	1.01 [0.98-1.05]	1.00 [0.96-1.03]
C19	9,190	50,989,590	0.18	0.18	0.18	0.00	−0.01	−0.01	0.96 [0.89-1.04]	0.96 [0.89-1.04]
C20	19,410	50,861,270	0.37	0.38	0.38	−0.01	−0.01	−0.01	0.97 [0.92-1.02]	0.97 [0.92-1.03]
C21	2,400	51,037,490	0.05	0.05	0.05	0.00	0.00	0.00	1.09 [0.94-1.25]	1.05 [0.91-1.21]
C22	8,940	51,041,580	0.21	0.17	0.17	0.04	0.04	0.03	1.23 [1.14-1.32]*	1.17 [1.08-1.26]*
C23	1,310	51,059,660	0.03	0.03	0.03	0.00	0.00	0.00	1.06 [0.87-1.29]	1.05 [0.86-1.28]
C24	2,470	51,053,600	0.05	0.05	0.05	0.00	0.00	0.00	1.07 [0.92-1.23]	1.02 [0.88-1.18]
C25	15,060	51,035,790	0.33	0.29	0.29	0.05	0.04	0.03	1.15 [1.09-1.22]*	1.11 [1.05-1.18]*
C26	3,300	51,047,880	0.07	0.06	0.07	0.01	0.01	0.01	1.12 [0.99-1.26]	1.08 [0.95-1.22]
C30	530	51,056,660	0.01	0.01	0.01	0.00	0.00	0.00	1.16 [0.86-1.56]	1.12 [0.83-1.52]
C32	3,470	51,023,110	0.08	0.07	0.07	0.01	0.01	0.01	1.16 [1.03-1.30]	1.12 [0.99-1.26]
C34	65,170	50,883,280	1.45	1.24	1.26	0.21	0.19	0.12	1.15 [1.12-1.18]*	1.09 [1.06-1.12]*
C38	830	51,059,370	0.02	0.02	0.02	0.00	0.00	0.00	1.00 [0.79-1.27]	0.99 [0.77-1.26]

C41	1,520	51,050,330	0.04	0.03	0.03	0.01	0.01	0.01	1.21 [1.02-1.44]	1.17 [0.98-1.39]
C43	16,870	50,858,180	0.32	0.33	0.34	−0.01	−0.02	−0.02	0.96 [0.90-1.01]	0.94 [0.89-0.99]
C44	126,920	49,518,040	2.82	2.51	2.56	0.32	0.26	0.17	1.10 [1.08-1.13]*	1.06 [1.04-1.08]*
C45	4,090	51,054,030	0.08	0.08	0.08	0.00	0.00	0.00	1.01 [0.90-1.13]	1.02 [0.91-1.14]
C48	2,220	51,052,900	0.05	0.04	0.04	0.00	0.00	0.00	1.06 [0.91-1.23]	1.04 [0.90-1.21]
C49	3,550	51,028,900	0.08	0.07	0.07	0.01	0.01	0.01	1.13 [1.00-1.27]	1.09 [0.96-1.23]
C50	74,410	49,849,330	1.56	1.48	1.51	0.08	0.05	0.03	1.03 [1.01-1.06]	1.02 [0.99-1.05]
C51	2,010	51,039,090	0.05	0.04	0.04	0.01	0.01	0.01	1.27 [1.09-1.47]*	1.22 [1.05-1.42]*
C52	670	51,057,980	0.01	0.01	0.01	0.00	0.00	0.00	0.92 [0.70-1.22]	0.93 [0.70-1.23]
C53	3,050	51,006,030	0.06	0.06	0.06	0.00	0.00	0.00	0.96 [0.84-1.09]	0.98 [0.86-1.12]
C54	12,680	50,899,930	0.26	0.25	0.25	0.01	0.01	0.01	1.03 [0.97-1.09]	1.05 [0.98-1.11]
C55	1,740	51,048,320	0.04	0.03	0.03	0.00	0.00	0.00	1.01 [0.85-1.19]	1.01 [0.85-1.20]
C56	10,950	50,968,380	0.22	0.21	0.22	0.01	0.00	0.01	1.02 [0.96-1.09]	1.02 [0.96-1.10]
C57	1,260	51,055,660	0.03	0.02	0.02	0.01	0.00	0.01	1.19 [0.99-1.44]	1.21 [1.00-1.47]
C60	940	51,050,040	0.02	0.02	0.02	0.00	0.00	0.00	1.21 [0.97-1.51]	1.24 [0.99-1.56]
C61	73,070	50,383,270	1.54	1.43	1.47	0.11	0.08	0.04	1.05 [1.03-1.08]*	1.02 [1.00-1.05]
C62	860	51,027,880	0.02	0.02	0.02	0.00	0.00	0.00	0.99 [0.78-1.26]	0.95 [0.74-1.22]
C64	14,700	50,946,120	0.32	0.28	0.28	0.03	0.03	0.02	1.11 [1.05-1.18]*	1.06 [1.00-1.12]
C65	1,340	51,051,280	0.03	0.03	0.03	0.01	0.01	0.01	1.22 [1.01-1.48]	1.21 [1.00-1.47]
C66	1,910	51,049,020	0.04	0.04	0.04	0.00	0.00	0.00	1.08 [0.92-1.28]	1.04 [0.88-1.23]
C67	33,560	50,662,650	0.71	0.65	0.66	0.06	0.06	0.04	1.08 [1.04-1.13]*	1.06 [1.02-1.10]*
C68	730	51,056,610	0.01	0.01	0.01	0.00	0.00	0.00	1.11 [0.85-1.44]	1.10 [0.84-1.43]
C69	1,220	51,043,890	0.02	0.02	0.02	0.00	0.00	0.00	1.03 [0.84-1.26]	1.00 [0.81-1.23]
C71	6,850	51,029,800	0.15	0.13	0.13	0.02	0.02	0.01	1.13 [1.04-1.23]*	1.10 [1.01-1.20]
C73	3,350	51,007,370	0.07	0.07	0.06	0.00	0.00	0.00	1.06 [0.94-1.20]	1.03 [0.92-1.17]
C75	620	51,056,320	0.01	0.01	0.01	0.00	0.00	0.00	1.07 [0.81-1.41]	1.00 [0.75-1.33]
C76	2,190	51,047,490	0.05	0.04	0.04	0.00	0.00	0.00	1.07 [0.92-1.25]	1.06 [0.91-1.24]
C77	73,140	50,532,770	1.52	1.43	1.45	0.09	0.08	0.06	1.05 [1.03-1.08]*	1.04 [1.01-1.07]*
C78	109,940	50,850,120	2.29	2.13	2.16	0.16	0.13	0.10	1.06 [1.04-1.08]*	1.04 [1.02-1.07]*
C79	92,510	50,875,120	1.95	1.79	1.81	0.16	0.14	0.11	1.08 [1.05-1.10]*	1.06 [1.03-1.08]*
C80	32,730	50,980,460	0.70	0.63	0.63	0.07	0.06	0.05	1.10 [1.06-1.14]*	1.07 [1.03-1.12]*
C81	2,300	51,021,060	0.07	0.04	0.04	0.02	0.03	0.02	1.62 [1.42-1.85]*	1.52 [1.33-1.74]*
C82	3,860	51,014,590	0.08	0.07	0.08	0.01	0.01	0.00	1.10 [0.98-1.23]	1.05 [0.94-1.17]
C83	10,750	50,979,900	0.25	0.20	0.20	0.05	0.05	0.04	1.23 [1.15-1.31]*	1.19 [1.11-1.27]*
C84	1,410	51,049,430	0.05	0.02	0.02	0.03	0.03	0.03	2.56 [2.19-2.99]*	2.48 [2.11-2.90]*
C85	13,510	50,911,760	0.33	0.25	0.25	0.07	0.08	0.06	1.30 [1.23-1.38]*	1.23 [1.16-1.31]*
C88	1,850	51,049,540	0.04	0.03	0.03	0.01	0.01	0.01	1.28 [1.10-1.51]*	1.24 [1.06-1.46]*
C90	10,000	50,997,600	0.22	0.19	0.19	0.03	0.03	0.02	1.14 [1.06-1.22]*	1.10 [1.03-1.18]*
C91	9,230	50,981,940	0.20	0.18	0.18	0.02	0.02	0.01	1.09 [1.02-1.18]	1.05 [0.98-1.13]
C92	7,310	51,023,350	0.18	0.14	0.14	0.04	0.04	0.04	1.28 [1.19-1.39]*	1.25 [1.15-1.35]*
C93	1,020	51,059,740	0.02	0.02	0.02	0.00	0.00	0.00	1.10 [0.88-1.37]	1.07 [0.85-1.34]
C94	480	51,060,480	0.01	0.01	0.01	0.00	0.00	0.00	1.23 [0.90-1.68]	1.17 [0.85-1.61]
C95	1,490	51,054,140	0.04	0.03	0.03	0.01	0.01	0.01	1.32 [1.11-1.57]*	1.24 [1.03-1.48]
C97	8,500	51,036,540	0.18	0.16	0.17	0.01	0.01	0.00	1.05 [0.97-1.14]	1.02 [0.94-1.10]
D00	940	51,053,050	0.02	0.02	0.02	0.00	0.00	0.00	1.07 [0.85-1.34]	0.98 [0.77-1.23]
D01	900	51,051,330	0.02	0.02	0.02	0.00	0.00	0.00	1.13 [0.89-1.42]	1.04 [0.82-1.31]
D02	960	51,052,740	0.02	0.02	0.02	0.00	0.00	0.00	1.22 [0.98-1.52]	1.14 [0.92-1.43]
D03	6,530	50,979,550	0.14	0.13	0.13	0.01	0.01	0.01	1.08 [0.99-1.18]	1.04 [0.95-1.14]
D04	14,210	50,921,750	0.34	0.27	0.27	0.08	0.07	0.05	1.26 [1.19-1.34]*	1.17 [1.10-1.24]*
D05	12,310	50,833,500	0.24	0.24	0.24	0.00	0.00	0.00	1.00 [0.94-1.06]	0.98 [0.92-1.05]
D06	2,480	50,823,020	0.05	0.05	0.05	0.00	0.00	0.00	1.03 [0.90-1.18]	0.99 [0.86-1.14]
D07	3,620	50,998,440	0.08	0.07	0.07	0.01	0.01	0.01	1.22 [1.09-1.37]*	1.16 [1.03-1.30]
D09	3,540	51,025,720	0.08	0.07	0.07	0.01	0.01	0.01	1.13 [1.01-1.27]	1.10 [0.97-1.23]
D10	2,590	50,994,710	0.06	0.05	0.05	0.02	0.02	0.01	1.35 [1.19-1.54]*	1.21 [1.06-1.38]*
D11	3,770	50,985,820	0.08	0.07	0.07	0.00	0.00	0.00	1.04 [0.93-1.16]	0.99 [0.88-1.11]
D12	120,430	49,508,060	2.85	2.34	2.36	0.51	0.49	0.27	1.21 [1.18-1.23]*	1.11 [1.08-1.13]*
D13	7,840	50,856,160	0.18	0.15	0.15	0.03	0.03	0.01	1.21 [1.12-1.31]*	1.09 [1.01-1.18]
D14	1,990	51,017,110	0.05	0.04	0.04	0.01	0.01	0.01	1.32 [1.14-1.53]*	1.22 [1.05-1.42]*
D15	920	51,052,090	0.02	0.02	0.02	0.00	0.00	0.00	1.16 [0.93-1.45]	1.12 [0.89-1.40]
D16	1,880	51,017,370	0.04	0.04	0.04	0.01	0.01	0.00	1.20 [1.03-1.40]	1.09 [0.93-1.28]
D17	31,530	50,120,670	0.69	0.62	0.61	0.08	0.08	0.04	1.13 [1.09-1.18]*	1.06 [1.02-1.10]*
D18	12,590	50,857,730	0.29	0.24	0.24	0.05	0.05	0.02	1.19 [1.12-1.26]*	1.09 [1.02-1.16]*
D21	3,320	50,985,550	0.08	0.06	0.06	0.02	0.02	0.01	1.25 [1.12-1.40]*	1.17 [1.04-1.31]
D22	19,170	50,412,570	0.41	0.37	0.37	0.04	0.04	0.02	1.11 [1.06-1.17]*	1.05 [0.99-1.10]
D23	16,910	50,487,720	0.40	0.32	0.32	0.07	0.08	0.06	1.26 [1.20-1.32]*	1.17 [1.11-1.24]*
D24	7,130	50,817,110	0.15	0.14	0.14	0.01	0.00	0.00	1.02 [0.94-1.11]	0.98 [0.90-1.07]
D25	53,810	49,537,950	1.18	1.07	1.09	0.11	0.09	0.06	1.09 [1.06-1.12]*	1.05 [1.02-1.08]*
D26	1,780	51,006,230	0.04	0.03	0.03	0.00	0.00	0.00	1.08 [0.92-1.27]	1.04 [0.88-1.23]
D27	10,290	50,823,440	0.21	0.20	0.20	0.01	0.01	0.00	1.04 [0.97-1.11]	0.99 [0.93-1.07]
D28	1,090	51,027,240	0.02	0.02	0.02	0.00	0.00	0.00	1.14 [0.93-1.40]	1.11 [0.90-1.37]
D29	1,040	51,043,870	0.02	0.02	0.02	0.00	0.00	0.00	1.14 [0.92-1.42]	1.10 [0.89-1.38]
D30	2,220	51,032,530	0.05	0.04	0.04	0.00	0.00	0.00	1.08 [0.94-1.26]	1.03 [0.89-1.20]
D31	1,850	51,037,510	0.04	0.03	0.03	0.01	0.01	0.01	1.26 [1.07-1.47]*	1.20 [1.02-1.41]
D32	8,190	50,985,200	0.18	0.16	0.16	0.02	0.02	0.01	1.11 [1.03-1.21]*	1.05 [0.97-1.14]
D33	2,780	51,021,540	0.06	0.05	0.05	0.01	0.01	0.00	1.11 [0.97-1.27]	1.02 [0.89-1.17]
D34	1,810	51,023,250	0.04	0.04	0.04	0.00	0.00	0.00	1.04 [0.89-1.23]	1.00 [0.85-1.19]
D35	11,130	50,923,410	0.25	0.21	0.22	0.03	0.03	0.01	1.13 [1.05-1.20]*	1.04 [0.98-1.12]
D36	4,300	50,955,120	0.10	0.08	0.08	0.02	0.02	0.01	1.24 [1.12-1.37]*	1.13 [1.02-1.26]
D37	16,360	50,898,110	0.35	0.32	0.32	0.04	0.03	0.02	1.11 [1.05-1.17]*	1.06 [1.00-1.12]
D38	3,860	51,034,270	0.09	0.07	0.07	0.02	0.01	0.01	1.20 [1.08-1.34]*	1.14 [1.02-1.27]
D39	1,880	51,026,350	0.04	0.04	0.04	0.00	0.00	0.00	0.97 [0.83-1.14]	0.94 [0.79-1.11]
D40	620	51,049,490	0.01	0.01	0.01	0.00	0.00	0.00	1.21 [0.92-1.59]	1.17 [0.88-1.54]
D41	11,660	50,929,190	0.25	0.22	0.23	0.02	0.02	0.02	1.10 [1.03-1.18]*	1.06 [1.00-1.14]
D43	3,220	51,039,400	0.07	0.06	0.06	0.01	0.00	0.00	1.06 [0.94-1.20]	1.01 [0.89-1.14]
D44	1,940	51,031,000	0.04	0.04	0.03	0.01	0.01	0.01	1.26 [1.08-1.47]*	1.16 [0.99-1.35]
D45	4,180	50,994,040	0.09	0.08	0.08	0.01	0.01	0.00	1.06 [0.96-1.19]	1.00 [0.89-1.11]
D46	10,030	51,012,800	0.25	0.19	0.19	0.07	0.06	0.05	1.33 [1.24-1.42]*	1.26 [1.18-1.35]*
D47	15,930	50,969,740	0.39	0.30	0.31	0.09	0.08	0.05	1.25 [1.19-1.32]*	1.16 [1.10-1.23]*
D48	6,730	50,937,780	0.15	0.13	0.13	0.02	0.02	0.01	1.11 [1.02-1.20]	1.05 [0.96-1.15]
40+ - D50-D89										
D50	202,630	49,229,630	5.26	3.87	3.98	1.39	1.28	0.88	1.32 [1.30-1.34]*	1.20 [1.18-1.22]*
D51	18,880	50,867,700	0.48	0.35	0.35	0.13	0.13	0.09	1.36 [1.30-1.43]*	1.24 [1.18-1.31]*
D52	14,730	50,991,170	0.36	0.27	0.28	0.09	0.08	0.06	1.30 [1.23-1.38]*	1.20 [1.14-1.28]*
D53	3,800	51,037,310	0.10	0.07	0.07	0.04	0.04	0.03	1.53 [1.37-1.71]*	1.37 [1.23-1.54]*
D56	4,380	50,977,920	0.12	0.08	0.08	0.04	0.04	0.04	1.54 [1.40-1.70]*	1.42 [1.28-1.57]*
D57	3,780	50,962,930	0.10	0.07	0.07	0.03	0.02	0.02	1.30 [1.17-1.44]*	1.23 [1.10-1.37]*
D58	1,550	51,043,150	0.04	0.03	0.03	0.01	0.01	0.00	1.18 [0.99-1.40]	1.12 [0.94-1.34]
D59	2,340	51,038,000	0.06	0.04	0.04	0.02	0.02	0.02	1.43 [1.25-1.64]*	1.38 [1.20-1.58]*
D61	12,880	51,005,100	0.32	0.24	0.24	0.08	0.07	0.06	1.30 [1.22-1.38]*	1.22 [1.15-1.30]*
D62	3,370	50,981,740	0.08	0.06	0.06	0.01	0.01	0.01	1.18 [1.05-1.33]*	1.12 [0.99-1.26]
D63	21,270	50,943,080	0.51	0.40	0.40	0.11	0.11	0.08	1.27 [1.21-1.33]*	1.18 [1.13-1.24]*
D64	277,310	48,504,870	7.21	5.40	5.54	1.82	1.67	1.24	1.30 [1.29-1.32]*	1.21 [1.19-1.22]*
D65	840	51,058,690	0.02	0.02	0.02	0.00	0.00	0.00	1.21 [0.95-1.53]	1.17 [0.92-1.49]
D66	610	51,049,570	0.01	0.01	0.01	0.00	0.00	0.00	1.18 [0.90-1.57]	1.09 [0.82-1.45]
D68	11,730	50,884,800	0.29	0.22	0.22	0.07	0.07	0.04	1.34 [1.26-1.42]*	1.18 [1.11-1.26]*
D69	37,580	50,794,980	0.92	0.70	0.71	0.21	0.20	0.15	1.28 [1.24-1.33]*	1.20 [1.16-1.25]*
D70	37,520	50,799,270	0.88	0.71	0.71	0.17	0.17	0.14	1.23 [1.19-1.28]*	1.19 [1.15-1.23]*
D72	7,890	51,006,700	0.21	0.14	0.15	0.06	0.06	0.04	1.40 [1.30-1.51]*	1.25 [1.16-1.35]*

D73	8,890	50,984,730	0.21	0.17	0.17	0.05	0.04	0.03	1.26 [1.17-1.35]*	1.16 [1.08-1.25]*
D75	11,670	50,945,750	0.27	0.22	0.23	0.05	0.05	0.03	1.21 [1.14-1.29]*	1.12 [1.05-1.19]*
D76	490	51,051,290	0.01	0.01	0.01	0.00	0.00	0.00	1.38 [1.03-1.85]	1.27 [0.94-1.72]
D80	2,020	51,039,960	0.06	0.04	0.04	0.02	0.02	0.01	1.46 [1.27-1.69]*	1.21 [1.04-1.41]
D84	850	51,052,290	0.03	0.01	0.01	0.01	0.01	0.01	1.75 [1.42-2.16]*	1.38 [1.10-1.72]*
D86	5,630	50,971,560	0.15	0.10	0.10	0.05	0.05	0.04	1.51 [1.39-1.64]*	1.35 [1.23-1.47]*
D89	4,650	51,019,410	0.12	0.08	0.09	0.04	0.04	0.03	1.41 [1.28-1.55]*	1.28 [1.16-1.41]*
40+ - E00-E89										
E02	1,720	51,054,180	0.04	0.03	0.03	0.01	0.00	0.00	1.11 [0.93-1.32]	1.07 [0.89-1.28]
E03	198,660	48,514,780	5.22	3.85	3.92	1.37	1.31	1.01	1.33 [1.31-1.35]*	1.24 [1.22-1.26]*
E04	20,340	50,767,650	0.45	0.39	0.40	0.06	0.05	0.02	1.12 [1.07-1.17]*	1.04 [0.99-1.09]
E05	31,650	50,660,640	0.78	0.59	0.61	0.18	0.17	0.11	1.27 [1.22-1.32]*	1.17 [1.12-1.21]*
E06	3,000	51,014,260	0.07	0.06	0.06	0.01	0.01	0.01	1.24 [1.10-1.40]*	1.16 [1.02-1.31]
E07	5,570	50,994,750	0.13	0.10	0.11	0.03	0.03	0.02	1.27 [1.16-1.39]*	1.15 [1.05-1.26]*
E10	33,960	50,276,570	0.84	0.64	0.64	0.20	0.20	0.10	1.32 [1.27-1.36]*	1.13 [1.09-1.17]*
E11	333,460	46,606,620	8.69	6.82	6.97	1.87	1.72	0.87	1.25 [1.23-1.26]*	1.11 [1.10-1.12]*
E13	3,810	51,032,600	0.10	0.07	0.07	0.03	0.03	0.02	1.43 [1.29-1.59]*	1.21 [1.09-1.35]*
E14	45,140	50,502,840	1.17	0.84	0.85	0.33	0.31	0.17	1.37 [1.33-1.41]*	1.17 [1.13-1.21]*
E16	48,180	50,807,870	1.17	0.90	0.92	0.27	0.25	0.11	1.27 [1.23-1.31]*	1.10 [1.07-1.14]*
E20	1,260	51,050,580	0.03	0.02	0.02	0.01	0.01	0.01	1.53 [1.27-1.84]*	1.33 [1.10-1.62]*
E21	16,620	50,931,760	0.38	0.32	0.33	0.06	0.05	0.03	1.16 [1.10-1.22]*	1.07 [1.02-1.13]
E22	10,340	50,984,450	0.29	0.18	0.19	0.10	0.10	0.08	1.51 [1.41-1.61]*	1.41 [1.32-1.50]*
E23	3,970	51,001,610	0.11	0.07	0.07	0.04	0.03	0.03	1.49 [1.34-1.65]*	1.33 [1.20-1.48]*
E24	1,030	51,044,870	0.03	0.02	0.02	0.01	0.01	0.01	1.61 [1.33-1.96]*	1.24 [1.01-1.52]
E26	640	51,051,290	0.01	0.01	0.01	0.00	0.00	0.00	1.14 [0.87-1.50]	0.97 [0.73-1.29]
E27	10,280	50,977,920	0.27	0.19	0.19	0.09	0.08	0.05	1.45 [1.36-1.54]*	1.25 [1.17-1.34]*
E28	4,540	50,886,220	0.11	0.08	0.09	0.03	0.02	0.00	1.26 [1.15-1.39]*	1.05 [0.95-1.16]
E29	1,450	51,043,050	0.04	0.03	0.02	0.02	0.02	0.01	1.76 [1.50-2.08]*	1.40 [1.18-1.66]*
E34	1,080	51,044,630	0.03	0.02	0.02	0.01	0.01	0.00	1.39 [1.14-1.70]*	1.23 [1.00-1.50]
E41	610	51,060,640	0.01	0.01	0.01	0.00	0.00	0.00	1.10 [0.82-1.48]	1.09 [0.81-1.48]
E43	650	51,059,840	0.01	0.01	0.01	0.00	0.00	0.00	1.24 [0.94-1.63]	1.20 [0.91-1.60]
E46	8,690	51,026,740	0.20	0.16	0.17	0.03	0.03	0.02	1.16 [1.07-1.25]*	1.09 [1.01-1.18]
E51	860	51,053,310	0.02	0.02	0.02	0.01	0.01	0.00	1.48 [1.19-1.84]*	1.28 [1.02-1.61]
E53	48,410	50,810,220	1.20	0.90	0.93	0.30	0.26	0.17	1.28 [1.24-1.33]*	1.17 [1.13-1.21]*
E55	61,600	50,809,510	1.59	1.13	1.17	0.46	0.42	0.27	1.35 [1.32-1.39]*	1.20 [1.17-1.24]*
E56	620	51,058,320	0.01	0.01	0.01	0.00	0.00	0.00	1.21 [0.92-1.60]	1.10 [0.83-1.46]
E61	8,900	51,010,460	0.23	0.16	0.17	0.07	0.07	0.04	1.39 [1.29-1.49]*	1.22 [1.13-1.31]*
E63	570	51,059,030	0.01	0.01	0.01	0.00	0.00	0.00	1.46 [1.11-1.94]*	1.32 [0.99-1.77]
E66	271,010	48,354,460	7.09	5.29	5.35	1.80	1.74	0.91	1.32 [1.31-1.34]*	1.15 [1.13-1.16]*
E72	420	51,056,390	0.01	0.01	0.01	0.00	0.00	0.00	1.52 [1.12-2.08]*	1.39 [1.01-1.90]
E73	1,540	51,048,610	0.04	0.03	0.03	0.01	0.01	0.00	1.35 [1.14-1.59]*	1.10 [0.92-1.30]
E74	920	51,050,320	0.02	0.02	0.02	0.01	0.01	0.00	1.37 [1.10-1.70]*	1.25 [1.01-1.56]
E78	441,840	45,348,790	11.83	9.29	9.62	2.54	2.21	1.17	1.23 [1.22-1.24]*	1.11 [1.10-1.12]*
E79	500	51,057,690	0.01	0.01	0.01	0.00	0.00	0.00	1.43 [1.07-1.92]	1.25 [0.92-1.69]
E80	4,020	51,017,990	0.09	0.08	0.08	0.02	0.02	0.01	1.21 [1.09-1.35]*	1.13 [1.01-1.26]
E83	76,890	50,728,850	1.81	1.45	1.51	0.36	0.30	0.17	1.20 [1.17-1.23]*	1.10 [1.08-1.13]*
E85	2,890	51,047,110	0.07	0.05	0.06	0.01	0.01	0.01	1.18 [1.04-1.35]	1.10 [0.96-1.25]
E86	194,120	50,308,450	4.63	3.69	3.90	0.93	0.73	0.48	1.19 [1.17-1.21]*	1.12 [1.10-1.14]*
E87	311,620	49,733,080	7.89	5.92	6.15	1.98	1.74	1.21	1.28 [1.27-1.30]*	1.18 [1.17-1.20]*
E88	13,510	51,003,820	0.34	0.25	0.26	0.09	0.08	0.06	1.31 [1.23-1.39]*	1.20 [1.13-1.27]*
E89	18,800	50,855,880	0.45	0.35	0.36	0.10	0.09	0.05	1.25 [1.19-1.31]*	1.13 [1.08-1.19]*
40+ - F01-F99										
F00	51,220	50,906,960	1.13	0.98	1.06	0.16	0.07	0.05	1.07 [1.03-1.10]*	1.05 [1.01-1.08]*
F01	58,800	50,876,570	1.31	1.12	1.21	0.19	0.11	0.05	1.09 [1.06-1.12]*	1.04 [1.00-1.07]
F02	10,170	51,030,340	0.21	0.20	0.21	0.01	0.00	0.00	1.02 [0.95-1.10]	1.00 [0.93-1.08]
F03	141,060	50,594,100	3.16	2.71	2.95	0.45	0.20	0.13	1.07 [1.05-1.09]*	1.04 [1.02-1.06]*
F05	128,470	50,751,990	3.04	2.42	2.60	0.62	0.44	0.28	1.17 [1.15-1.19]*	1.10 [1.08-1.13]*
F06	19,870	50,969,820	0.46	0.37	0.39	0.08	0.07	0.04	1.17 [1.11-1.23]*	1.11 [1.05-1.16]*
F07	910	51,047,000	0.02	0.02	0.02	0.01	0.01	0.00	1.35 [1.09-1.68]*	1.23 [0.98-1.53]
F10	76,830	49,711,970	1.86	1.48	1.48	0.38	0.39	0.25	1.26 [1.23-1.29]*	1.15 [1.12-1.18]*
F11	3,890	50,914,360	0.09	0.07	0.07	0.02	0.02	0.00	1.30 [1.17-1.45]*	1.05 [0.94-1.17]
F12	4,870	50,981,300	0.12	0.09	0.09	0.02	0.03	0.01	1.28 [1.17-1.41]*	1.06 [0.96-1.17]
F13	1,040	51,041,610	0.03	0.02	0.02	0.01	0.01	0.00	1.42 [1.16-1.74]*	1.14 [0.92-1.41]
F14	1,740	51,018,850	0.05	0.03	0.03	0.01	0.01	0.01	1.42 [1.22-1.65]*	1.13 [0.97-1.33]
F15	740	51,040,460	0.02	0.01	0.01	0.00	0.00	0.00	1.16 [0.91-1.48]	0.98 [0.76-1.27]
F17	323,530	47,834,770	7.63	6.58	6.63	1.06	1.01	0.30	1.15 [1.14-1.17]*	1.04 [1.03-1.05]*
F19	2,580	50,991,480	0.06	0.05	0.05	0.01	0.01	0.00	1.22 [1.07-1.39]*	0.96 [0.84-1.10]
F20	11,300	50,741,280	0.28	0.21	0.21	0.07	0.07	0.06	1.32 [1.24-1.40]*	1.25 [1.18-1.34]*
F22	5,830	50,989,350	0.14	0.11	0.11	0.04	0.04	0.03	1.32 [1.21-1.45]*	1.22 [1.11-1.33]*
F23	2,300	51,003,230	0.06	0.04	0.04	0.01	0.01	0.01	1.28 [1.12-1.48]*	1.20 [1.04-1.38]
F25	2,230	50,991,550	0.06	0.04	0.04	0.02	0.02	0.01	1.38 [1.20-1.58]*	1.27 [1.10-1.46]*
F29	4,710	50,999,260	0.12	0.09	0.09	0.04	0.04	0.02	1.40 [1.28-1.54]*	1.24 [1.13-1.37]*
F30	1,490	51,014,580	0.04	0.03	0.03	0.01	0.01	0.01	1.42 [1.21-1.68]*	1.31 [1.11-1.55]*
F31	12,140	50,810,570	0.30	0.23	0.23	0.08	0.08	0.03	1.33 [1.25-1.41]*	1.12 [1.06-1.20]*
F32	249,590	48,243,350	6.81	4.82	4.90	1.99	1.91	0.90	1.39 [1.37-1.41]*	1.15 [1.14-1.17]*
F33	8,570	50,840,480	0.21	0.16	0.16	0.05	0.05	0.01	1.28 [1.19-1.37]*	1.05 [0.98-1.14]
F34	1,240	51,039,970	0.03	0.02	0.02	0.01	0.01	0.01	1.52 [1.27-1.83]*	1.24 [1.02-1.50]
F38	590	51,055,860	0.01	0.01	0.01	0.00	0.00	0.00	1.21 [0.92-1.61]	1.04 [0.78-1.39]
F39	1,030	51,048,340	0.03	0.02	0.02	0.01	0.01	0.00	1.33 [1.08-1.64]*	1.15 [0.93-1.43]
F40	19,620	50,875,490	0.49	0.36	0.38	0.12	0.11	0.06	1.30 [1.24-1.36]*	1.14 [1.08-1.19]*
F41	199,420	49,443,920	5.25	3.77	3.86	1.47	1.38	0.62	1.36 [1.34-1.38]*	1.13 [1.12-1.15]*
F42	3,130	51,016,800	0.10	0.05	0.05	0.05	0.05	0.03	1.84 [1.65-2.06]*	1.53 [1.37-1.72]*
F43	8,660	50,886,440	0.22	0.16	0.16	0.06	0.06	0.01	1.35 [1.26-1.45]*	1.07 [0.99-1.15]
F44	2,150	51,028,350	0.06	0.04	0.04	0.02	0.02	0.01	1.45 [1.27-1.67]*	1.15 [0.99-1.33]
F45	3,460	50,992,520	0.09	0.06	0.07	0.03	0.02	0.01	1.37 [1.23-1.53]*	1.16 [1.04-1.30]
F48	830	51,048,760	0.02	0.01	0.02	0.01	0.01	0.00	1.51 [1.21-1.87]*	1.20 [0.95-1.51]
F50	1,890	51,023,680	0.05	0.03	0.03	0.01	0.01	0.01	1.42 [1.22-1.64]*	1.19 [1.02-1.39]
F51	800	51,053,200	0.02	0.01	0.01	0.01	0.01	0.00	1.50 [1.20-1.88]*	1.27 [1.00-1.60]
F52	2,440	51,017,710	0.06	0.04	0.04	0.02	0.02	0.01	1.48 [1.30-1.68]*	1.26 [1.10-1.44]*
F60	5,580	50,915,090	0.16	0.10	0.10	0.06	0.06	0.03	1.59 [1.46-1.72]*	1.23 [1.13-1.35]*
F69	520	51,050,000	0.02	0.01	0.01	0.01	0.01	0.01	1.81 [1.39-2.36]*	1.49 [1.13-1.97]*
F70	1,020	51,034,890	0.04	0.02	0.02	0.02	0.02	0.02	2.17 [1.80-2.61]*	1.81 [1.49-2.20]*
F71	360	51,050,760	0.02	0.01	0.01	0.01	0.01	0.01	2.90 [2.15-3.91]*	2.62 [1.92-3.56]*
F72	600	51,044,950	0.02	0.01	0.01	0.02	0.02	0.01	2.79 [2.21-3.52]*	2.58 [2.02-3.29]*
F79	4,320	50,988,970	0.15	0.07	0.07	0.08	0.08	0.07	2.13 [1.94-2.33]*	1.93 [1.76-2.12]*
F80	2,620	51,040,090	0.07	0.05	0.05	0.02	0.02	0.02	1.41 [1.24-1.61]*	1.31 [1.14-1.49]*
F81	11,100	50,831,980	0.38	0.18	0.18	0.20	0.21	0.19	2.15 [2.03-2.27]*	1.99 [1.88-2.11]*
F84	2,250	51,026,060	0.08	0.04	0.04	0.04	0.04	0.03	2.15 [1.90-2.44]*	1.73 [1.51-1.97]*
F90	500	51,057,130	0.02	0.01	0.01	0.01	0.01	0.00	1.75 [1.34-2.29]*	1.26 [0.94-1.68]
F91	560	51,054,260	0.01	0.01	0.01	0.00	0.00	0.00	1.09 [0.81-1.47]	1.01 [0.74-1.37]
F99	3,250	50,975,700	0.08	0.06	0.06	0.02	0.02	0.01	1.30 [1.16-1.46]*	1.12 [0.99-1.26]
40+ - G00-G99										
G00	940	51,046,440	0.02	0.02	0.02	0.00	0.00	0.00	1.25 [1.01-1.55]	1.20 [0.96-1.49]
G03	1,040	51,047,570	0.02	0.02	0.02	0.00	0.00	0.00	1.13 [0.92-1.40]	1.00 [0.80-1.24]
G04	3,180	51,031,760	0.08	0.06	0.06	0.02	0.01	0.01	1.23 [1.09-1.39]*	1.12 [0.99-1.27]
G05	820	51,054,380	0.02	0.01	0.01	0.01	0.01	0.01	1.60 [1.28-2.00]*	1.48 [1.18-1.86]*

G06	1,580	51,043,730	0.04	0.03	0.03	0.01	0.01	0.00	1.18 [1.00-1.41]	1.11 [0.93-1.33]
G08	600	51,055,130	0.01	0.01	0.01	0.00	0.00	0.00	1.14 [0.86-1.51]	1.06 [0.79-1.42]
G09	800	51,050,030	0.02	0.01	0.02	0.00	0.00	0.00	1.24 [0.98-1.56]	1.11 [0.87-1.41]
G10	540	51,054,360	0.01	0.01	0.01	0.00	0.00	0.00	1.26 [0.94-1.67]	1.29 [0.97-1.73]
G11	1,590	51,041,700	0.04	0.03	0.03	0.01	0.01	0.00	1.20 [1.02-1.43]	1.10 [0.93-1.31]
G12	4,150	51,043,510	0.09	0.08	0.08	0.01	0.01	0.00	1.10 [0.99-1.22]	1.06 [0.95-1.18]
G20	39,000	50,807,970	0.83	0.75	0.78	0.07	0.05	0.02	1.06 [1.02-1.10]*	1.03 [0.99-1.07]
G21	3,270	51,045,340	0.08	0.06	0.06	0.02	0.02	0.02	1.34 [1.19-1.51]*	1.28 [1.13-1.44]*
G23	2,060	51,054,230	0.05	0.04	0.04	0.01	0.00	0.00	1.08 [0.93-1.27]	1.03 [0.88-1.21]
G24	3,260	51,018,790	0.08	0.06	0.06	0.02	0.01	0.01	1.21 [1.08-1.37]*	1.11 [0.99-1.26]
G25	19,670	50,937,650	0.49	0.36	0.38	0.12	0.11	0.05	1.28 [1.22-1.35]*	1.11 [1.06-1.17]*
G30	68,520	50,819,790	1.52	1.31	1.41	0.21	0.11	0.08	1.08 [1.05-1.11]*	1.06 [1.03-1.09]*
G31	48,660	50,840,080	1.12	0.92	0.96	0.20	0.16	0.10	1.17 [1.13-1.20]*	1.10 [1.07-1.14]*
G32	690	51,054,850	0.02	0.01	0.01	0.01	0.01	0.00	1.47 [1.15-1.89]*	1.30 [1.01-1.68]
G35	8,150	50,835,220	0.19	0.15	0.15	0.03	0.03	0.02	1.23 [1.14-1.32]*	1.09 [1.01-1.18]
G37	1,770	51,015,600	0.04	0.03	0.03	0.01	0.01	0.01	1.36 [1.17-1.59]*	1.24 [1.06-1.46]*
G40	48,710	50,113,760	1.26	0.91	0.91	0.35	0.35	0.21	1.38 [1.34-1.42]*	1.20 [1.16-1.23]*
G41	3,140	51,025,780	0.08	0.06	0.06	0.02	0.02	0.01	1.29 [1.15-1.46]*	1.15 [1.02-1.30]
G43	38,220	50,543,910	0.97	0.71	0.72	0.25	0.24	0.09	1.33 [1.29-1.38]*	1.10 [1.06-1.14]*
G44	5,910	50,957,330	0.15	0.11	0.11	0.04	0.04	0.02	1.37 [1.26-1.49]*	1.15 [1.06-1.26]*
G45	46,400	50,511,080	1.07	0.89	0.91	0.19	0.16	0.09	1.18 [1.14-1.22]*	1.10 [1.06-1.13]*
G46	3,030	51,042,520	0.07	0.06	0.06	0.01	0.01	0.01	1.19 [1.05-1.36]*	1.12 [0.98-1.28]
G47	52,080	50,370,280	1.48	0.94	0.94	0.55	0.55	0.32	1.58 [1.54-1.63]*	1.27 [1.24-1.31]*
G50	6,930	50,974,860	0.18	0.13	0.13	0.06	0.06	0.04	1.44 [1.33-1.56]*	1.24 [1.15-1.35]*
G51	13,270	50,891,810	0.31	0.25	0.26	0.06	0.05	0.03	1.21 [1.14-1.29]*	1.10 [1.04-1.17]*
G52	1,040	51,048,930	0.03	0.02	0.02	0.01	0.01	0.00	1.28 [1.04-1.57]	1.15 [0.93-1.43]
G53	2,510	51,041,530	0.06	0.05	0.05	0.02	0.02	0.01	1.35 [1.18-1.55]*	1.25 [1.09-1.44]*
G54	4,130	51,004,160	0.10	0.08	0.08	0.03	0.03	0.01	1.35 [1.22-1.50]*	1.16 [1.04-1.29]*
G55	45,780	50,421,160	1.11	0.86	0.87	0.25	0.24	0.11	1.28 [1.24-1.32]*	1.11 [1.08-1.15]*
G56	72,630	49,335,500	1.85	1.39	1.39	0.46	0.46	0.27	1.33 [1.30-1.36]*	1.17 [1.14-1.20]*
G57	8,260	50,900,430	0.21	0.15	0.15	0.05	0.05	0.03	1.35 [1.26-1.45]*	1.19 [1.11-1.28]*
G58	4,040	50,998,280	0.10	0.07	0.07	0.03	0.03	0.02	1.41 [1.27-1.56]*	1.22 [1.10-1.36]*
G59	3,570	51,015,790	0.10	0.06	0.06	0.03	0.03	0.01	1.49 [1.34-1.66]*	1.18 [1.05-1.32]*
G60	1,900	51,037,270	0.05	0.04	0.04	0.01	0.01	0.00	1.26 [1.09-1.47]*	1.11 [0.95-1.30]
G61	2,350	51,021,330	0.06	0.04	0.04	0.01	0.01	0.01	1.24 [1.08-1.43]*	1.17 [1.02-1.35]
G62	28,670	50,845,350	0.72	0.53	0.54	0.20	0.19	0.10	1.35 [1.30-1.40]*	1.15 [1.11-1.20]*
G63	15,330	50,958,270	0.41	0.28	0.28	0.14	0.13	0.06	1.46 [1.38-1.54]*	1.17 [1.11-1.24]*
G70	2,390	51,030,860	0.05	0.05	0.05	0.01	0.01	0.00	1.17 [1.02-1.35]	1.09 [0.94-1.26]
G71	1,360	51,036,470	0.03	0.03	0.03	0.00	0.00	0.00	1.14 [0.95-1.36]	1.08 [0.89-1.30]
G72	2,900	51,032,450	0.08	0.05	0.05	0.02	0.02	0.02	1.41 [1.25-1.59]*	1.29 [1.14-1.46]*
G80	2,150	50,996,680	0.06	0.04	0.04	0.02	0.02	0.02	1.66 [1.46-1.90]*	1.59 [1.38-1.82]*
G81	52,240	50,596,550	1.21	0.99	1.03	0.22	0.18	0.11	1.17 [1.14-1.21]*	1.10 [1.07-1.13]*
G82	4,620	50,981,500	0.11	0.09	0.09	0.02	0.02	0.01	1.21 [1.10-1.34]*	1.13 [1.03-1.26]
G83	7,060	50,990,170	0.17	0.13	0.14	0.03	0.03	0.02	1.23 [1.13-1.33]*	1.12 [1.03-1.21]*
G90	3,600	51,024,660	0.08	0.07	0.07	0.02	0.01	0.01	1.22 [1.08-1.36]*	1.08 [0.96-1.22]
G91	8,690	50,982,410	0.20	0.16	0.17	0.03	0.03	0.02	1.17 [1.09-1.26]*	1.13 [1.04-1.21]*
G93	32,090	50,813,540	0.76	0.60	0.61	0.16	0.15	0.08	1.25 [1.21-1.30]*	1.11 [1.07-1.16]*
G94	1,170	51,054,740	0.03	0.02	0.02	0.01	0.00	0.00	1.19 [0.98-1.45]	1.16 [0.95-1.42]
G95	10,450	50,964,070	0.24	0.20	0.20	0.05	0.04	0.03	1.22 [1.14-1.31]*	1.14 [1.06-1.22]*
G96	2,670	51,022,950	0.07	0.05	0.05	0.02	0.02	0.01	1.37 [1.21-1.55]*	1.21 [1.06-1.37]*
G97	1,820	51,023,060	0.04	0.03	0.03	0.01	0.01	0.00	1.24 [1.06-1.45]*	1.09 [0.93-1.28]
G98	950	51,055,440	0.03	0.02	0.02	0.01	0.01	0.00	1.53 [1.24-1.88]*	1.22 [0.98-1.52]
G99	16,820	50,889,580	0.40	0.32	0.32	0.08	0.08	0.04	1.26 [1.19-1.32]*	1.12 [1.06-1.18]*

40+ - H00-H59

H00	7,560	50,806,010	0.18	0.14	0.14	0.03	0.03	0.03	1.25 [1.15-1.34]*	1.18 [1.10-1.28]*
H01	10,160	50,945,090	0.26	0.19	0.19	0.08	0.08	0.06	1.40 [1.31-1.50]*	1.31 [1.22-1.40]*
H02	53,810	50,138,060	1.35	1.01	1.03	0.34	0.32	0.24	1.31 [1.28-1.35]*	1.22 [1.19-1.26]*
H03	510	51,053,730	0.01	0.01	0.01	0.00	0.00	0.00	1.30 [0.96-1.76]	1.18 [0.87-1.61]
H04	25,350	50,664,750	0.68	0.46	0.47	0.21	0.21	0.16	1.44 [1.39-1.51]*	1.32 [1.26-1.37]*
H05	3,780	51,003,050	0.10	0.07	0.07	0.04	0.03	0.03	1.47 [1.33-1.63]*	1.35 [1.21-1.50]*
H06	990	51,043,580	0.03	0.02	0.02	0.01	0.01	0.01	1.47 [1.20-1.80]*	1.36 [1.11-1.68]*
H10	10,670	50,988,090	0.27	0.20	0.20	0.08	0.07	0.05	1.35 [1.26-1.45]*	1.24 [1.16-1.33]*
H11	10,450	50,902,940	0.25	0.20	0.20	0.06	0.05	0.04	1.27 [1.19-1.36]*	1.21 [1.13-1.29]*
H13	550	51,054,510	0.02	0.01	0.01	0.01	0.01	0.01	1.78 [1.35-2.34]*	1.63 [1.23-2.16]*
H15	890	51,047,690	0.02	0.02	0.02	0.01	0.01	0.00	1.30 [1.04-1.62]	1.18 [0.94-1.48]
H16	4,370	50,997,020	0.12	0.08	0.08	0.05	0.05	0.04	1.58 [1.44-1.75]*	1.48 [1.34-1.64]*
H17	2,720	51,028,400	0.07	0.05	0.05	0.02	0.02	0.02	1.37 [1.21-1.56]*	1.28 [1.12-1.46]*
H18	18,490	50,891,460	0.44	0.35	0.36	0.10	0.09	0.07	1.24 [1.18-1.31]*	1.19 [1.13-1.25]*
H19	1,140	51,046,730	0.03	0.02	0.02	0.01	0.01	0.01	1.77 [1.47-2.14]*	1.54 [1.27-1.88]*
H20	4,700	50,988,650	0.12	0.09	0.09	0.03	0.03	0.03	1.38 [1.25-1.51]*	1.30 [1.18-1.43]*
H21	8,860	50,977,310	0.20	0.17	0.17	0.03	0.03	0.02	1.15 [1.07-1.24]*	1.09 [1.01-1.18]
H25	219,740	48,641,500	5.34	4.34	4.51	1.00	0.83	0.60	1.18 [1.17-1.20]*	1.13 [1.11-1.14]*
H26	308,810	47,219,390	7.92	6.24	6.55	1.67	1.36	0.98	1.21 [1.19-1.22]*	1.14 [1.13-1.16]*
H27	3,880	51,003,690	0.09	0.07	0.08	0.02	0.01	0.01	1.19 [1.07-1.33]*	1.14 [1.01-1.27]
H28	1,610	51,044,140	0.04	0.03	0.03	0.01	0.01	0.00	1.28 [1.08-1.51]*	1.13 [0.95-1.34]
H31	5,020	51,015,020	0.11	0.10	0.10	0.02	0.01	0.01	1.10 [1.00-1.22]	1.06 [0.96-1.17]
H33	18,780	50,703,150	0.38	0.37	0.37	0.01	0.02	0.01	1.04 [0.99-1.10]	1.02 [0.97-1.08]
H34	12,450	50,937,910	0.28	0.24	0.25	0.05	0.04	0.02	1.15 [1.08-1.22]*	1.09 [1.02-1.16]*
H35	113,160	50,085,220	2.65	2.18	2.28	0.47	0.37	0.26	1.16 [1.14-1.19]*	1.11 [1.09-1.13]*
H36	44,100	50,619,510	1.06	0.83	0.85	0.23	0.21	0.08	1.24 [1.20-1.28]*	1.08 [1.05-1.12]*
H40	104,530	50,002,190	2.45	2.01	2.08	0.43	0.37	0.28	1.18 [1.15-1.20]*	1.13 [1.11-1.16]*
H43	20,450	50,824,110	0.46	0.39	0.40	0.06	0.06	0.03	1.14 [1.09-1.20]*	1.08 [1.03-1.13]*
H44	6,190	50,985,090	0.15	0.12	0.12	0.03	0.03	0.03	1.29 [1.19-1.41]*	1.22 [1.12-1.33]*
H46	1,450	51,039,480	0.04	0.03	0.03	0.01	0.01	0.01	1.33 [1.12-1.58]*	1.20 [1.01-1.44]
H47	5,330	50,998,810	0.12	0.10	0.10	0.02	0.02	0.02	1.24 [1.13-1.36]*	1.18 [1.08-1.30]*
H49	4,380	51,011,310	0.09	0.08	0.08	0.01	0.01	0.00	1.13 [1.02-1.26]	1.05 [0.94-1.17]
H50	5,860	50,919,680	0.14	0.11	0.11	0.03	0.03	0.02	1.29 [1.18-1.40]*	1.20 [1.10-1.31]*
H51	610	51,054,620	0.01	0.01	0.01	0.00	0.00	0.00	1.11 [0.84-1.48]	1.01 [0.76-1.35]
H52	32,660	50,799,520	0.73	0.63	0.65	0.10	0.08	0.05	1.12 [1.08-1.17]*	1.08 [1.04-1.12]*
H53	49,140	50,447,830	1.18	0.93	0.95	0.25	0.24	0.13	1.25 [1.21-1.29]*	1.13 [1.09-1.16]*
H54	48,680	50,759,720	1.15	0.92	0.95	0.23	0.20	0.14	1.21 [1.17-1.25]*	1.14 [1.10-1.17]*
H55	3,690	51,025,380	0.09	0.07	0.07	0.02	0.01	0.00	1.15 [1.03-1.29]	1.04 [0.93-1.17]
H57	9,250	50,965,450	0.22	0.17	0.18	0.05	0.05	0.03	1.28 [1.19-1.37]*	1.16 [1.08-1.25]*
H58	2,130	51,043,570	0.05	0.04	0.04	0.01	0.01	0.00	1.17 [1.01-1.36]	1.06 [0.91-1.23]
H59	4,040	51,002,540	0.09	0.08	0.08	0.01	0.01	0.00	1.10 [0.98-1.22]	1.06 [0.95-1.18]

40+ - H60-H95

H60	6,300	50,931,690	0.20	0.11	0.11	0.09	0.09	0.08	1.85 [1.71-2.00]*	1.67 [1.54-1.81]*
H61	10,750	50,879,750	0.29	0.20	0.20	0.09	0.09	0.07	1.45 [1.36-1.55]*	1.34 [1.26-1.43]*
H65	5,350	50,898,270	0.13	0.10	0.10	0.03	0.03	0.03	1.35 [1.24-1.48]*	1.24 [1.13-1.35]*
H66	5,900	50,915,990	0.16	0.11	0.11	0.06	0.06	0.04	1.52 [1.40-1.65]*	1.37 [1.26-1.50]*
H69	1,080	51,038,920	0.03	0.02	0.02	0.01	0.01	0.01	1.47 [1.21-1.79]*	1.27 [1.04-1.55]
H70	1,280	51,042,520	0.03	0.02	0.03	0.01	0.01	0.00	1.24 [1.03-1.50]	1.13 [0.93-1.38]
H71	2,670	50,978,220	0.06	0.05	0.05	0.01	0.01	0.00	1.12 [0.98-1.28]	1.07 [0.93-1.22]
H72	5,080	50,894,630	0.12	0.09	0.10	0.03	0.03	0.02	1.31 [1.19-1.43]*	1.20 [1.10-1.32]*
H73	1,990	51,000,810	0.05	0.04	0.04	0.01	0.01	0.01	1.34 [1.16-1.55]*	1.23 [1.06-1.42]*
H74	2,830	50,984,940	0.07	0.05	0.05	0.02	0.02	0.01	1.33 [1.18-1.51]*	1.24 [1.09-1.41]*
H80	1,080	51,028,370	0.02	0.02	0.02	0.00	0.00	0.00	1.14 [0.93-1.40]	1.12 [0.90-1.38]

H81	20,920	50,866,910	0.51	0.39	0.40	0.12	0.10	0.06	1.26 [1.20-1.32]*	1.14 [1.09-1.20]*
H83	7,820	50,938,780	0.19	0.15	0.15	0.04	0.04	0.02	1.26 [1.17-1.36]*	1.13 [1.04-1.22]*
H90	17,240	50,907,620	0.44	0.32	0.32	0.13	0.12	0.09	1.37 [1.30-1.44]*	1.24 [1.18-1.31]*
H91	124,020	50,364,520	3.01	2.35	2.44	0.66	0.57	0.41	1.23 [1.21-1.26]*	1.16 [1.13-1.18]*
H92	4,600	50,967,670	0.12	0.08	0.09	0.04	0.04	0.03	1.43 [1.30-1.57]*	1.26 [1.14-1.39]*
H93	10,700	50,948,080	0.27	0.20	0.20	0.07	0.07	0.05	1.36 [1.27-1.44]*	1.22 [1.14-1.30]*
H95	560	51,042,870	0.01	0.01	0.01	0.00	0.00	0.00	1.36 [1.04-1.79]	1.30 [0.99-1.72]
40+ - I00-I99										
I05	6,840	50,979,600	0.17	0.13	0.13	0.04	0.04	0.03	1.32 [1.21-1.43]*	1.21 [1.11-1.32]*
I06	680	51,049,560	0.02	0.01	0.01	0.00	0.00	0.00	1.36 [1.06-1.75]	1.22 [0.95-1.58]
I07	17,330	50,988,030	0.43	0.32	0.33	0.11	0.10	0.07	1.31 [1.25-1.38]*	1.20 [1.14-1.27]*
I08	91,440	50,611,190	2.19	1.73	1.80	0.46	0.39	0.25	1.22 [1.19-1.25]*	1.13 [1.10-1.16]*
I09	1,000	51,049,990	0.03	0.02	0.02	0.01	0.01	0.01	1.52 [1.24-1.86]*	1.39 [1.13-1.71]*
I10	896,210	37,682,130	29.42	22.54	23.76	6.88	5.66	3.13	1.24 [1.23-1.25]*	1.12 [1.11-1.13]*
I11	5,190	51,002,590	0.12	0.10	0.10	0.02	0.02	0.01	1.22 [1.11-1.35]*	1.10 [0.99-1.21]
I12	51,700	50,598,080	1.26	0.97	0.96	0.29	0.30	0.20	1.31 [1.27-1.35]*	1.19 [1.16-1.23]*
I13	2,610	51,054,060	0.06	0.05	0.05	0.02	0.01	0.01	1.28 [1.11-1.47]*	1.14 [0.99-1.31]
I15	3,150	51,025,550	0.08	0.06	0.06	0.02	0.02	0.01	1.26 [1.12-1.42]*	1.11 [0.98-1.26]
I20	201,260	47,185,870	5.27	4.05	4.12	1.22	1.14	0.64	1.28 [1.26-1.30]*	1.14 [1.12-1.15]*
I21	137,370	49,410,410	3.18	2.70	2.75	0.48	0.42	0.24	1.15 [1.13-1.18]*	1.08 [1.06-1.10]*
I22	12,570	50,887,210	0.30	0.24	0.23	0.06	0.07	0.04	1.29 [1.22-1.37]*	1.16 [1.09-1.24]*
I23	750	51,051,210	0.02	0.01	0.01	0.00	0.00	0.00	1.30 [1.02-1.66]	1.29 [1.00-1.65]
I24	38,920	50,775,070	0.94	0.73	0.76	0.21	0.18	0.09	1.24 [1.20-1.28]*	1.11 [1.07-1.15]*
I25	345,430	46,002,200	9.11	7.16	7.45	1.95	1.66	0.90	1.22 [1.21-1.24]*	1.11 [1.10-1.12]*
I26	67,350	50,474,730	1.48	1.30	1.33	0.18	0.15	0.06	1.12 [1.09-1.15]*	1.04 [1.01-1.07]*
I27	42,020	50,869,870	1.05	0.78	0.80	0.27	0.25	0.16	1.31 [1.27-1.36]*	1.18 [1.14-1.22]*
I28	950	51,053,440	0.02	0.02	0.02	0.00	0.00	0.00	1.16 [0.92-1.45]	1.01 [0.80-1.28]
I30	1,600	51,029,510	0.04	0.03	0.03	0.01	0.01	0.01	1.26 [1.07-1.49]*	1.19 [1.00-1.41]
I31	23,390	50,876,190	0.55	0.44	0.45	0.11	0.10	0.06	1.22 [1.17-1.28]*	1.13 [1.08-1.18]*
I33	3,960	51,027,570	0.10	0.07	0.08	0.03	0.03	0.02	1.34 [1.20-1.49]*	1.25 [1.12-1.39]*
I34	58,560	50,585,480	1.36	1.11	1.14	0.25	0.22	0.13	1.19 [1.16-1.23]*	1.10 [1.07-1.14]*
I35	86,930	50,439,200	2.10	1.64	1.70	0.45	0.40	0.26	1.23 [1.20-1.26]*	1.14 [1.11-1.17]*
I36	6,100	51,033,870	0.15	0.11	0.12	0.03	0.03	0.02	1.24 [1.13-1.35]*	1.13 [1.04-1.24]*
I37	6,060	51,034,000	0.15	0.11	0.11	0.03	0.03	0.02	1.29 [1.18-1.41]*	1.17 [1.07-1.28]*
I38	8,930	50,980,570	0.22	0.16	0.17	0.06	0.06	0.04	1.33 [1.24-1.43]*	1.22 [1.14-1.32]*
I40	440	51,056,380	0.01	0.01	0.01	0.00	0.00	0.00	1.50 [1.10-2.04]	1.39 [1.01-1.91]
I42	20,590	50,844,760	0.48	0.39	0.39	0.09	0.09	0.05	1.22 [1.16-1.28]*	1.10 [1.05-1.16]*
I43	900	51,058,390	0.02	0.02	0.02	0.00	0.00	0.00	0.98 [0.77-1.25]	0.92 [0.72-1.18]
I44	137,620	50,162,310	3.29	2.63	2.73	0.66	0.56	0.36	1.20 [1.18-1.23]*	1.12 [1.10-1.14]*
I45	82,590	50,528,220	1.96	1.56	1.63	0.40	0.33	0.20	1.20 [1.17-1.23]*	1.11 [1.08-1.14]*
I46	43,180	50,937,680	1.01	0.81	0.83	0.20	0.19	0.12	1.23 [1.19-1.27]*	1.14 [1.10-1.18]*
I47	44,890	50,474,580	1.05	0.85	0.87	0.20	0.19	0.10	1.22 [1.18-1.26]*	1.11 [1.07-1.15]*
I48	383,660	47,896,520	9.46	7.70	8.13	1.76	1.33	0.81	1.16 [1.15-1.18]*	1.09 [1.08-1.11]*
I49	54,720	50,555,180	1.27	1.04	1.08	0.23	0.20	0.10	1.18 [1.15-1.22]*	1.09 [1.06-1.12]*
I50	291,040	49,373,470	7.36	5.58	5.82	1.78	1.53	0.98	1.26 [1.25-1.28]*	1.15 [1.14-1.17]*
I51	158,350	50,112,670	3.86	3.01	3.09	0.85	0.77	0.46	1.25 [1.23-1.27]*	1.13 [1.11-1.16]*
I60	8,810	50,943,160	0.19	0.17	0.17	0.03	0.02	0.02	1.14 [1.06-1.22]*	1.11 [1.03-1.19]*
I61	23,220	50,917,940	0.53	0.44	0.45	0.09	0.08	0.06	1.17 [1.12-1.22]*	1.13 [1.08-1.19]*
I62	13,430	50,983,440	0.31	0.25	0.26	0.05	0.04	0.03	1.17 [1.10-1.25]*	1.12 [1.05-1.19]*
I63	111,470	50,267,290	2.44	2.17	2.25	0.28	0.20	0.10	1.09 [1.07-1.11]*	1.04 [1.02-1.07]*
I64	29,140	50,722,790	0.65	0.56	0.57	0.09	0.08	0.05	1.15 [1.10-1.19]*	1.09 [1.05-1.14]*
I65	27,030	50,798,120	0.62	0.51	0.53	0.11	0.09	0.05	1.17 [1.12-1.22]*	1.08 [1.04-1.13]*
I66	1,630	51,048,900	0.03	0.03	0.03	0.00	0.00	0.00	1.08 [0.90-1.28]	1.00 [0.84-1.20]
I67	168,530	50,265,350	3.99	3.22	3.37	0.77	0.62	0.39	1.18 [1.16-1.20]*	1.11 [1.09-1.13]*
I68	980	51,058,710	0.02	0.02	0.02	0.00	0.00	0.00	1.22 [0.98-1.52]	1.16 [0.92-1.45]
I69	48,840	50,632,850	1.13	0.93	0.96	0.21	0.18	0.10	1.18 [1.15-1.22]*	1.10 [1.06-1.13]*
I70	41,950	50,751,360	1.02	0.78	0.81	0.24	0.21	0.13	1.27 [1.22-1.31]*	1.15 [1.11-1.19]*
I71	51,380	50,700,530	1.17	0.98	1.00	0.19	0.18	0.12	1.18 [1.14-1.21]*	1.11 [1.08-1.15]*
I72	11,930	50,953,610	0.27	0.23	0.23	0.05	0.04	0.02	1.18 [1.10-1.25]*	1.09 [1.02-1.16]*
I73	89,050	50,175,750	2.33	1.66	1.68	0.68	0.65	0.44	1.39 [1.36-1.42]*	1.23 [1.20-1.26]*
I74	23,300	50,833,930	0.53	0.44	0.45	0.09	0.09	0.04	1.19 [1.14-1.25]*	1.09 [1.04-1.14]*
I77	30,470	50,728,300	0.76	0.57	0.57	0.20	0.19	0.13	1.33 [1.28-1.39]*	1.20 [1.16-1.25]*
I78	7,260	50,972,070	0.18	0.13	0.14	0.05	0.04	0.03	1.31 [1.21-1.42]*	1.21 [1.12-1.31]*
I79	3,090	51,032,090	0.08	0.06	0.06	0.02	0.02	0.01	1.35 [1.20-1.52]*	1.13 [1.00-1.28]
I80	58,600	50,251,150	1.41	1.11	1.13	0.30	0.28	0.20	1.25 [1.21-1.28]*	1.16 [1.13-1.20]*
I81	3,330	51,046,990	0.07	0.06	0.07	0.01	0.01	0.00	1.14 [1.01-1.28]	1.06 [0.94-1.19]
I82	6,980	51,009,600	0.16	0.13	0.13	0.03	0.03	0.02	1.20 [1.11-1.30]*	1.11 [1.02-1.20]
I83	64,120	49,388,940	1.96	1.16	1.17	0.80	0.79	0.73	1.68 [1.64-1.72]*	1.59 [1.55-1.63]*
I84	98,060	48,352,680	2.55	1.92	1.90	0.63	0.65	0.47	1.34 [1.32-1.37]*	1.22 [1.20-1.25]*
I85	9,220	50,974,330	0.25	0.17	0.17	0.08	0.08	0.06	1.47 [1.38-1.57]*	1.31 [1.22-1.40]*
I86	5,300	50,981,350	0.13	0.10	0.10	0.03	0.03	0.02	1.36 [1.24-1.49]*	1.21 [1.10-1.33]*
I87	14,890	50,953,980	0.44	0.26	0.26	0.17	0.17	0.15	1.65 [1.56-1.74]*	1.51 [1.43-1.60]*
I88	1,330	51,039,960	0.04	0.02	0.02	0.01	0.01	0.01	1.53 [1.29-1.82]*	1.45 [1.22-1.74]*
I89	20,640	50,929,470	0.57	0.37	0.38	0.20	0.19	0.15	1.51 [1.44-1.58]*	1.36 [1.30-1.43]*
I95	195,670	50,039,650	4.71	3.74	3.91	0.97	0.80	0.50	1.20 [1.19-1.22]*	1.12 [1.10-1.14]*
I97	2,500	51,012,680	0.06	0.05	0.05	0.01	0.01	0.01	1.28 [1.12-1.46]*	1.19 [1.04-1.36]
I98	6,240	51,015,930	0.16	0.11	0.11	0.05	0.05	0.03	1.45 [1.34-1.58]*	1.27 [1.17-1.38]*
I99	3,630	51,028,970	0.09	0.07	0.07	0.02	0.02	0.01	1.24 [1.11-1.39]*	1.14 [1.02-1.28]
40+ - J00-J99										
J00	3,250	50,991,260	0.08	0.06	0.06	0.02	0.02	0.02	1.38 [1.23-1.55]*	1.25 [1.11-1.41]*
J01	1,650	51,024,000	0.04	0.03	0.03	0.01	0.01	0.01	1.38 [1.18-1.62]*	1.16 [0.99-1.37]
J02	8,890	50,911,160	0.23	0.16	0.16	0.07	0.07	0.05	1.44 [1.34-1.54]*	1.26 [1.18-1.36]*
J03	5,280	50,858,200	0.13	0.10	0.10	0.03	0.02	0.01	1.25 [1.14-1.36]*	1.08 [0.98-1.19]
J04	1,720	51,033,090	0.04	0.03	0.03	0.01	0.01	0.01	1.41 [1.21-1.65]*	1.19 [1.01-1.40]
J05	800	51,047,720	0.02	0.02	0.02	0.00	0.00	0.00	1.12 [0.88-1.43]	1.03 [0.80-1.32]
J06	11,120	50,862,820	0.28	0.21	0.20	0.07	0.07	0.04	1.36 [1.28-1.45]*	1.18 [1.11-1.26]*
J10	13,950	51,003,630	0.36	0.26	0.26	0.10	0.09	0.05	1.36 [1.28-1.44]*	1.17 [1.10-1.24]*
J11	2,600	51,024,320	0.07	0.05	0.05	0.02	0.02	0.01	1.35 [1.19-1.54]*	1.20 [1.05-1.37]*
J12	27,470	51,030,540	0.70	0.50	0.53	0.19	0.16	0.10	1.31 [1.25-1.36]*	1.16 [1.11-1.21]*
J13	5,960	51,008,600	0.15	0.11	0.11	0.04	0.04	0.02	1.36 [1.24-1.48]*	1.19 [1.09-1.30]*
J14	2,560	51,043,050	0.06	0.05	0.05	0.02	0.02	0.01	1.35 [1.19-1.54]*	1.11 [0.96-1.27]
J15	12,540	50,973,360	0.33	0.23	0.23	0.10	0.10	0.06	1.43 [1.35-1.52]*	1.23 [1.16-1.31]*
J17	2,590	51,044,320	0.07	0.05	0.05	0.02	0.02	0.02	1.46 [1.29-1.65]*	1.31 [1.15-1.50]*
J18	393,090	49,071,460	9.93	7.60	7.87	2.33	2.05	1.34	1.26 [1.25-1.28]*	1.16 [1.14-1.17]*
J20	4,360	51,017,010	0.11	0.08	0.08	0.03	0.02	0.01	1.26 [1.14-1.40]*	1.12 [1.00-1.24]
J21	1,190	51,051,270	0.03	0.02	0.02	0.01	0.01	0.01	1.47 [1.22-1.78]*	1.25 [1.03-1.53]
J22	212,150	49,077,990	5.50	4.07	4.17	1.43	1.32	0.89	1.32 [1.30-1.34]*	1.19 [1.17-1.21]*
J30	22,750	50,810,500	0.68	0.40	0.41	0.28	0.27	0.15	1.67 [1.60-1.74]*	1.29 [1.23-1.35]*
J31	6,340	50,935,290	0.18	0.11	0.11	0.07	0.07	0.03	1.59 [1.47-1.72]*	1.24 [1.14-1.34]*
J32	17,800	50,651,120	0.46	0.33	0.33	0.14	0.14	0.07	1.42 [1.35-1.49]*	1.18 [1.13-1.25]*
J33	13,520	50,590,400	0.37	0.25	0.24	0.12	0.13	0.07	1.52 [1.44-1.61]*	1.25 [1.18-1.32]*
J34	27,720	49,934,600	0.75	0.51	0.50	0.23	0.24	0.16	1.48 [1.43-1.54]*	1.28 [1.23-1.33]*
J35	5,320	50,754,380	0.13	0.10	0.10	0.02	0.02	0.01	1.23 [1.13-1.35]*	1.10 [1.01-1.21]
J36	2,750	50,951,020	0.06	0.05	0.05	0.01	0.01	0.00	1.14 [1.00-1.30]	1.09 [0.95-1.25]
J37	500	51,044,810	0.01	0.01	0.01	0.00	0.00	0.00	1.31 [0.98-1.75]	1.10 [0.81-1.49]
J38	16,580	50,765,550	0.40	0.31	0.31	0.09	0.09	0.05	1.30 [1.24-1.37]*	1.16 [1.10-1.22]*
J39	7,980	50,949,860	0.20	0.15	0.15	0.05	0.05	0.03	1.32 [1.23-1.43]*	1.18 [1.09-1.27]*

J40	10,940	50,916,280	0.29	0.20	0.20	0.09	0.09	0.06	1.45 [1.36-1.54]*	1.25 [1.17-1.33]*
J41	540	51,055,440	0.01	0.01	0.01	0.00	0.00	0.00	1.36 [1.03-1.81]	1.18 [0.88-1.58]
J42	4,500	50,999,150	0.12	0.08	0.08	0.04	0.04	0.02	1.42 [1.29-1.56]*	1.18 [1.07-1.31]*
J43	64,610	50,639,430	1.65	1.20	1.22	0.45	0.43	0.25	1.36 [1.32-1.39]*	1.18 [1.15-1.22]*
J44	260,140	48,748,310	7.26	4.93	5.01	2.33	2.24	1.24	1.45 [1.43-1.47]*	1.21 [1.19-1.22]*
J45	267,020	44,751,780	9.53	5.20	5.17	4.33	4.36	2.26	1.84 [1.82-1.87]*	1.31 [1.29-1.33]*
J46	2,640	50,939,850	0.09	0.04	0.04	0.05	0.05	0.02	2.08 [1.85-2.33]*	1.35 [1.18-1.54]*
J47	46,310	50,734,820	1.28	0.84	0.85	0.44	0.43	0.27	1.50 [1.45-1.55]*	1.26 [1.22-1.30]*
J61	6,480	51,024,720	0.17	0.12	0.12	0.05	0.05	0.04	1.40 [1.28-1.52]*	1.27 [1.17-1.38]*
J67	1,840	51,048,120	0.05	0.03	0.03	0.02	0.02	0.01	1.59 [1.37-1.84]*	1.40 [1.20-1.63]*
J69	61,920	50,925,540	1.45	1.17	1.23	0.28	0.22	0.16	1.18 [1.14-1.21]*	1.12 [1.09-1.15]*
J70	1,250	51,055,650	0.03	0.02	0.02	0.01	0.01	0.01	1.51 [1.25-1.81]*	1.36 [1.13-1.64]*
J80	4,280	51,035,810	0.11	0.08	0.08	0.03	0.03	0.03	1.45 [1.31-1.60]*	1.31 [1.18-1.45]*
J81	21,550	50,925,270	0.53	0.40	0.40	0.12	0.12	0.09	1.31 [1.25-1.37]*	1.21 [1.15-1.27]*
J82	1,430	51,052,560	0.05	0.02	0.02	0.03	0.03	0.02	2.11 [1.80-2.47]*	1.49 [1.25-1.77]*
J84	40,380	50,881,670	1.09	0.73	0.75	0.36	0.33	0.27	1.44 [1.40-1.49]*	1.33 [1.28-1.37]*
J85	1,870	51,048,100	0.05	0.03	0.03	0.01	0.01	0.01	1.37 [1.18-1.59]*	1.22 [1.05-1.43]
J86	6,360	51,005,380	0.15	0.12	0.12	0.03	0.03	0.02	1.25 [1.15-1.36]*	1.16 [1.07-1.27]*
J90	200,180	50,136,900	4.74	3.83	3.96	0.91	0.78	0.52	1.20 [1.18-1.22]*	1.12 [1.10-1.14]*
J91	4,760	51,045,380	0.11	0.09	0.09	0.02	0.02	0.01	1.21 [1.09-1.34]*	1.13 [1.02-1.25]
J92	23,440	50,946,430	0.56	0.44	0.45	0.12	0.11	0.09	1.26 [1.20-1.31]*	1.18 [1.13-1.24]*
J93	16,040	50,914,360	0.36	0.31	0.31	0.05	0.05	0.03	1.17 [1.10-1.23]*	1.10 [1.04-1.16]*
J94	7,380	51,021,310	0.17	0.14	0.14	0.03	0.03	0.02	1.20 [1.11-1.30]*	1.12 [1.03-1.22]*
J95	5,680	50,992,850	0.13	0.11	0.11	0.02	0.03	0.02	1.24 [1.13-1.35]*	1.16 [1.06-1.27]*
J96	128,820	50,629,500	3.22	2.40	2.48	0.83	0.75	0.43	1.30 [1.28-1.33]*	1.16 [1.13-1.18]*
J98	114,890	50,436,960	2.76	2.17	2.25	0.59	0.52	0.28	1.23 [1.21-1.26]*	1.11 [1.09-1.13]*
J99	3,400	51,035,020	0.10	0.06	0.06	0.04	0.04	0.03	1.71 [1.54-1.91]*	1.47 [1.32-1.64]*
40+ - K00-K95										
K00	1,340	51,009,730	0.03	0.03	0.03	0.01	0.01	0.00	1.22 [1.02-1.47]	1.12 [0.93-1.35]
K01	7,610	50,446,540	0.17	0.15	0.15	0.02	0.02	0.02	1.15 [1.06-1.24]*	1.10 [1.02-1.19]
K02	38,990	50,050,970	0.96	0.74	0.75	0.22	0.21	0.12	1.28 [1.24-1.32]*	1.15 [1.11-1.19]*
K03	1,160	51,035,740	0.03	0.02	0.02	0.01	0.01	0.01	1.42 [1.18-1.72]*	1.27 [1.05-1.54]
K04	16,390	50,623,350	0.39	0.31	0.31	0.08	0.08	0.04	1.25 [1.19-1.32]*	1.13 [1.07-1.19]*
K05	9,760	50,756,730	0.24	0.18	0.18	0.06	0.06	0.04	1.33 [1.24-1.42]*	1.21 [1.13-1.29]*
K06	3,220	51,011,860	0.08	0.06	0.06	0.02	0.02	0.02	1.37 [1.22-1.54]*	1.25 [1.11-1.40]*
K07	4,320	50,910,920	0.11	0.08	0.08	0.03	0.02	0.01	1.29 [1.17-1.42]*	1.14 [1.03-1.27]
K08	26,860	50,500,010	0.65	0.51	0.51	0.15	0.15	0.09	1.29 [1.24-1.34]*	1.15 [1.11-1.20]*
K09	2,490	50,993,720	0.05	0.05	0.05	0.01	0.01	0.00	1.13 [0.99-1.30]	1.08 [0.94-1.24]
K10	4,020	51,008,940	0.10	0.07	0.08	0.02	0.02	0.01	1.27 [1.15-1.41]*	1.12 [1.01-1.25]
K11	8,450	50,900,390	0.21	0.16	0.16	0.06	0.06	0.04	1.36 [1.27-1.46]*	1.23 [1.15-1.33]*
K12	12,360	50,947,860	0.31	0.23	0.23	0.09	0.08	0.06	1.35 [1.28-1.44]*	1.25 [1.18-1.33]*
K13	18,320	50,718,530	0.47	0.34	0.34	0.13	0.13	0.10	1.38 [1.32-1.45]*	1.26 [1.20-1.32]*
K14	10,390	50,902,160	0.26	0.19	0.19	0.07	0.07	0.05	1.37 [1.29-1.46]*	1.23 [1.15-1.31]*
K20	72,930	49,690,260	1.83	1.39	1.40	0.44	0.43	0.26	1.30 [1.27-1.34]*	1.16 [1.13-1.19]*
K21	270,840	47,612,590	7.35	5.33	5.42	2.02	1.93	1.09	1.36 [1.34-1.37]*	1.17 [1.16-1.19]*
K22	110,630	49,543,140	2.75	2.12	2.13	0.63	0.62	0.41	1.29 [1.26-1.32]*	1.17 [1.15-1.20]*
K23	1,340	51,052,370	0.04	0.02	0.02	0.01	0.01	0.01	1.49 [1.24-1.78]*	1.27 [1.05-1.52]
K25	54,880	50,258,530	1.35	1.04	1.05	0.32	0.30	0.19	1.29 [1.25-1.32]*	1.17 [1.13-1.20]*
K26	39,990	50,464,380	0.92	0.76	0.77	0.16	0.15	0.10	1.20 [1.16-1.24]*	1.13 [1.09-1.17]*
K27	6,650	50,967,060	0.16	0.12	0.12	0.04	0.04	0.02	1.29 [1.19-1.40]*	1.15 [1.05-1.25]*
K28	870	51,051,410	0.02	0.02	0.02	0.01	0.01	0.00	1.38 [1.11-1.72]*	1.07 [0.84-1.35]
K29	261,190	46,666,980	7.11	5.27	5.35	1.84	1.76	1.12	1.33 [1.31-1.35]*	1.19 [1.17-1.20]*
K30	62,230	49,366,650	1.61	1.19	1.18	0.42	0.42	0.27	1.36 [1.32-1.39]*	1.21 [1.17-1.24]*
K31	95,600	50,087,860	2.39	1.81	1.84	0.58	0.55	0.30	1.30 [1.27-1.33]*	1.14 [1.12-1.17]*
K35	18,130	50,545,970	0.37	0.36	0.36	0.02	0.02	0.00	1.05 [1.00-1.11]	1.01 [0.95-1.06]
K37	2,660	50,979,500	0.06	0.05	0.05	0.01	0.01	0.00	1.15 [1.01-1.32]	1.07 [0.93-1.23]
K38	3,040	51,005,450	0.06	0.06	0.06	0.01	0.01	0.00	1.10 [0.97-1.25]	1.05 [0.92-1.19]
K40	99,720	48,919,820	2.24	1.99	2.02	0.25	0.22	0.15	1.11 [1.08-1.13]*	1.07 [1.05-1.10]*
K41	7,810	50,932,750	0.17	0.15	0.15	0.02	0.02	0.02	1.16 [1.08-1.26]*	1.10 [1.02-1.19]
K42	41,090	50,347,830	1.00	0.78	0.78	0.23	0.23	0.12	1.29 [1.25-1.34]*	1.14 [1.10-1.17]*
K43	35,500	50,475,010	0.86	0.67	0.67	0.19	0.19	0.10	1.28 [1.24-1.33]*	1.14 [1.10-1.18]*
K44	272,460	46,830,490	7.27	5.50	5.59	1.77	1.69	1.02	1.30 [1.29-1.32]*	1.16 [1.15-1.18]*
K45	1,750	51,040,260	0.04	0.03	0.03	0.01	0.01	0.00	1.20 [1.02-1.41]	1.07 [0.90-1.26]
K46	7,280	51,007,690	0.18	0.13	0.13	0.05	0.05	0.03	1.35 [1.25-1.46]*	1.17 [1.08-1.27]*
K50	13,400	50,711,460	0.37	0.24	0.24	0.13	0.13	0.08	1.56 [1.47-1.64]*	1.29 [1.22-1.36]*
K51	22,750	50,516,360	0.60	0.42	0.42	0.19	0.19	0.12	1.45 [1.39-1.51]*	1.24 [1.19-1.30]*
K52	129,920	48,538,230	3.41	2.52	2.50	0.89	0.91	0.63	1.36 [1.34-1.39]*	1.23 [1.21-1.25]*
K55	24,920	50,889,930	0.60	0.47	0.48	0.13	0.12	0.07	1.25 [1.20-1.31]*	1.14 [1.09-1.19]*
K56	71,360	50,423,700	1.66	1.36	1.39	0.30	0.28	0.16	1.20 [1.17-1.23]*	1.11 [1.08-1.14]*
K57	332,810	47,286,730	8.57	6.71	6.86	1.86	1.71	1.03	1.25 [1.23-1.26]*	1.14 [1.12-1.15]*
K58	65,400	50,128,800	1.84	1.19	1.21	0.65	0.64	0.30	1.53 [1.49-1.56]*	1.20 [1.17-1.23]*
K59	263,370	48,994,650	6.73	5.08	5.29	1.65	1.45	0.93	1.27 [1.26-1.29]*	1.16 [1.14-1.18]*
K60	17,290	50,499,480	0.48	0.31	0.31	0.16	0.16	0.13	1.53 [1.46-1.60]*	1.37 [1.30-1.43]*
K61	9,900	50,748,290	0.25	0.18	0.18	0.07	0.07	0.05	1.39 [1.31-1.49]*	1.25 [1.17-1.34]*
K62	149,260	48,028,240	3.85	2.95	2.94	0.90	0.91	0.61	1.31 [1.29-1.33]*	1.19 [1.17-1.21]*
K63	164,640	49,385,930	4.01	3.19	3.21	0.82	0.80	0.45	1.25 [1.23-1.27]*	1.13 [1.11-1.15]*
K64	109,720	50,625,920	2.72	2.05	2.11	0.67	0.61	0.38	1.29 [1.27-1.32]*	1.16 [1.14-1.19]*
K65	16,840	50,926,190	0.38	0.32	0.32	0.06	0.05	0.03	1.17 [1.11-1.23]*	1.10 [1.04-1.16]*
K66	51,430	50,444,680	1.17	0.99	1.00	0.19	0.18	0.08	1.18 [1.14-1.21]*	1.07 [1.04-1.11]*
K70	20,430	50,823,510	0.54	0.37	0.37	0.16	0.17	0.12	1.46 [1.40-1.53]*	1.28 [1.23-1.34]*
K71	1,100	51,049,810	0.03	0.02	0.02	0.01	0.01	0.01	1.52 [1.25-1.85]*	1.39 [1.14-1.70]*
K72	12,850	51,016,820	0.33	0.24	0.24	0.09	0.09	0.07	1.40 [1.32-1.48]*	1.25 [1.18-1.33]*
K73	1,150	51,036,220	0.03	0.02	0.02	0.01	0.01	0.01	1.51 [1.25-1.82]*	1.36 [1.12-1.64]*
K74	19,570	50,915,160	0.53	0.35	0.36	0.18	0.17	0.12	1.49 [1.42-1.56]*	1.30 [1.24-1.37]*
K75	12,200	50,952,900	0.32	0.22	0.23	0.10	0.09	0.06	1.41 [1.33-1.49]*	1.25 [1.18-1.33]*
K76	90,370	50,433,750	2.28	1.69	1.71	0.60	0.57	0.32	1.33 [1.30-1.36]*	1.16 [1.14-1.19]*
K80	158,440	48,531,720	3.73	3.16	3.23	0.56	0.50	0.23	1.15 [1.13-1.17]*	1.07 [1.05-1.08]*
K81	29,360	50,567,190	0.68	0.56	0.57	0.12	0.11	0.05	1.19 [1.14-1.24]*	1.09 [1.04-1.13]*
K82	21,120	50,758,780	0.48	0.40	0.41	0.08	0.07	0.04	1.18 [1.13-1.24]*	1.08 [1.03-1.14]*
K83	44,220	50,685,040	1.01	0.84	0.87	0.17	0.14	0.07	1.16 [1.12-1.20]*	1.07 [1.04-1.11]*
K85	27,620	50,650,630	0.63	0.53	0.53	0.11	0.10	0.05	1.19 [1.14-1.24]*	1.08 [1.04-1.13]*
K86	25,230	50,848,970	0.60	0.47	0.49	0.12	0.11	0.06	1.23 [1.18-1.29]*	1.11 [1.06-1.16]*
K90	14,920	50,814,430	0.43	0.26	0.27	0.16	0.16	0.12	1.61 [1.53-1.70]*	1.37 [1.30-1.44]*
K91	21,360	50,800,860	0.51	0.40	0.40	0.11	0.11	0.07	1.28 [1.22-1.34]*	1.16 [1.11-1.22]*
K92	186,280	49,300,040	4.66	3.59	3.65	1.07	1.01	0.65	1.28 [1.26-1.30]*	1.16 [1.14-1.18]*
40+ - L00-L99										
L01	780	51,051,490	0.03	0.01	0.01	0.01	0.02	0.01	2.27 [1.83-2.82]*	1.96 [1.57-2.46]*
L02	26,710	50,491,250	0.68	0.50	0.50	0.19	0.19	0.13	1.37 [1.32-1.43]*	1.23 [1.18-1.28]*
L03	145,920	49,405,490	4.36	2.65	2.66	1.70	1.69	1.44	1.64 [1.61-1.66]*	1.49 [1.47-1.52]*
L04	850	51,043,620	0.02	0.02	0.02	0.01	0.01	0.01	1.37 [1.09-1.71]*	1.32 [1.05-1.66]
L05	2,570	50,911,590	0.06	0.05	0.05	0.01	0.01	0.01	1.30 [1.15-1.48]*	1.17 [1.03-1.34]
L08	38,510	50,758,350	1.06	0.69	0.71	0.37	0.36	0.29	1.51 [1.46-1.56]*	1.37 [1.32-1.42]*
L10	510	51,056,770	0.02	0.01	0.01	0.01	0.01	0.01	2.27 [1.74-2.97]*	2.01 [1.53-2.66]*
L11	440	51,056,040	0.01	0.01	0.01	0.01	0.01	0.01	1.81 [1.33-2.46]*	1.77 [1.29-2.42]*
L12	3,750	51,039,440	0.15	0.06	0.06	0.10	0.10	0.10	2.80 [2.54-3.10]*	2.63 [2.37-2.91]*
L13	540	51,053,390	0.02	0.01	0.01	0.02	0.02	0.02	3.00 [2.34-3.86]*	2.70 [2.08-3.49]*
									35.83 [29.13-	31.58 [25.62-

L20	2,030	51,021,680	0.20	0.01	0.01	0.19	0.19	0.19	44.08]*	38.94]*
L21	1,520	51,045,840	0.07	0.02	0.02	0.05	0.05	0.05	3.22 [2.77-3.75]*	2.91 [2.49-3.40]*
L22	800	51,060,420	0.02	0.02	0.02	0.00	0.00	0.00	1.00 [0.77-1.31]	0.96 [0.73-1.25]
L23	1,140	51,037,130	0.05	0.02	0.01	0.04	0.04	0.04	3.72 [3.14-4.40]*	3.39 [2.86-4.03]*
L24	10,430	51,045,380	0.28	0.19	0.20	0.09	0.08	0.06	1.41 [1.32-1.51]*	1.29 [1.20-1.39]*
L25	1,380	51,043,700	0.06	0.02	0.02	0.04	0.04	0.04	3.33 [2.85-3.90]*	3.10 [2.64-3.64]*
L27	7,700	50,977,330	0.22	0.14	0.14	0.09	0.08	0.07	1.60 [1.49-1.72]*	1.46 [1.36-1.58]*
L28	3,020	51,013,060	0.15	0.04	0.04	0.12	0.12	0.11	3.99 [3.59-4.42]*	3.61 [3.25-4.01]*
L29	9,110	50,891,530	0.31	0.15	0.15	0.16	0.15	0.14	1.99 [1.87-2.12]*	1.78 [1.67-1.90]*
L30	42,300	50,387,880	3.15	0.35	0.33	2.80	2.81	2.78	9.44 [9.16-9.73]*	8.61 [8.35-8.89]*
L40	34,620	50,650,630	1.25	0.56	0.57	0.69	0.68	0.63	2.21 [2.14-2.28]*	2.03 [1.97-2.10]*
L42	410	51,057,560	0.01	0.01	0.01	0.01	0.01	0.01	1.79 [1.32-2.43]*	1.61 [1.18-2.22]*
L43	4,180	50,990,470	0.15	0.07	0.07	0.08	0.08	0.07	2.15 [1.96-2.36]*	1.99 [1.81-2.18]*
L50	4,600	50,989,330	0.15	0.08	0.08	0.07	0.07	0.05	1.86 [1.70-2.03]*	1.50 [1.37-1.65]*
L51	980	51,043,170	0.03	0.02	0.02	0.02	0.02	0.01	2.03 [1.67-2.46]*	1.77 [1.45-2.16]*
L52	510	51,048,170	0.01	0.01	0.01	0.01	0.00	0.00	1.49 [1.12-1.96]*	1.28 [0.96-1.71]
L53	11,930	50,947,250	0.37	0.20	0.21	0.17	0.17	0.15	1.82 [1.72-1.93]*	1.66 [1.57-1.77]*
L56	470	51,054,690	0.02	0.01	0.01	0.01	0.01	0.01	2.99 [2.29-3.90]*	2.67 [2.03-3.51]*
L57	34,150	50,677,710	0.86	0.63	0.65	0.22	0.21	0.16	1.32 [1.28-1.37]*	1.24 [1.19-1.28]*
L59	440	51,056,810	0.01	0.01	0.01	0.00	0.00	0.00	1.40 [1.03-1.91]	1.40 [1.01-1.92]
L60	6,410	50,847,190	0.17	0.12	0.12	0.06	0.06	0.04	1.51 [1.39-1.63]*	1.32 [1.22-1.44]*
L63	400	51,056,070	0.02	0.01	0.01	0.01	0.01	0.01	2.62 [1.97-3.48]*	2.06 [1.53-2.79]*
L65	1,440	51,044,600	0.05	0.02	0.02	0.03	0.03	0.02	2.09 [1.78-2.45]*	1.77 [1.50-2.08]*
L66	360	51,056,530	0.01	0.01	0.01	0.01	0.01	0.00	1.82 [1.32-2.51]*	1.64 [1.18-2.28]*
L70	710	51,048,370	0.02	0.01	0.01	0.01	0.01	0.00	1.44 [1.13-1.83]*	1.24 [0.97-1.60]
L71	3,220	51,027,650	0.09	0.06	0.06	0.03	0.03	0.03	1.52 [1.36-1.70]*	1.40 [1.25-1.57]*
L72	35,780	49,839,570	0.81	0.70	0.69	0.11	0.12	0.07	1.17 [1.13-1.21]*	1.09 [1.06-1.14]*
L73	3,380	50,965,490	0.09	0.06	0.06	0.03	0.03	0.03	1.58 [1.42-1.75]*	1.38 [1.24-1.55]*
L80	1,170	51,048,810	0.04	0.02	0.02	0.02	0.02	0.02	1.90 [1.59-2.27]*	1.67 [1.39-2.01]*
L81	6,210	50,953,950	0.15	0.12	0.12	0.03	0.03	0.02	1.27 [1.17-1.38]*	1.19 [1.09-1.30]*
L82	22,160	50,689,060	0.53	0.42	0.42	0.11	0.11	0.08	1.26 [1.20-1.31]*	1.17 [1.12-1.23]*
L83	1,040	51,044,820	0.03	0.02	0.02	0.01	0.01	0.01	1.71 [1.41-2.08]*	1.56 [1.27-1.90]*
L84	2,260	51,022,610	0.06	0.04	0.04	0.02	0.02	0.02	1.50 [1.31-1.72]*	1.35 [1.18-1.55]*
L85	9,140	50,948,720	0.25	0.16	0.16	0.09	0.09	0.08	1.55 [1.45-1.66]*	1.45 [1.35-1.55]*
L88	450	51,056,190	0.01	0.01	0.01	0.01	0.01	0.00	1.68 [1.25-2.26]*	1.45 [1.07-1.97]
L89	119,510	50,772,560	2.88	2.24	2.37	0.64	0.51	0.38	1.22 [1.19-1.24]*	1.15 [1.13-1.17]*
L90	17,690	50,642,140	0.44	0.33	0.33	0.11	0.11	0.08	1.32 [1.25-1.38]*	1.21 [1.15-1.28]*
L91	4,370	50,903,250	0.11	0.08	0.08	0.03	0.03	0.02	1.36 [1.23-1.50]*	1.25 [1.13-1.38]*
L92	4,230	50,964,650	0.11	0.08	0.08	0.03	0.03	0.02	1.37 [1.24-1.52]*	1.24 [1.12-1.38]*
L93	2,200	51,028,700	0.07	0.04	0.04	0.03	0.03	0.03	1.84 [1.62-2.10]*	1.58 [1.38-1.81]*
L94	1,340	51,039,790	0.04	0.02	0.02	0.02	0.02	0.02	1.81 [1.53-2.14]*	1.60 [1.34-1.90]*
L95	1,910	51,038,980	0.06	0.03	0.03	0.03	0.02	0.02	1.70 [1.47-1.96]*	1.53 [1.32-1.77]*
L97	85,290	50,567,910	2.39	1.54	1.56	0.85	0.83	0.70	1.53 [1.50-1.57]*	1.42 [1.38-1.45]*
L98	61,110	50,117,810	1.59	1.14	1.14	0.45	0.45	0.36	1.39 [1.35-1.43]*	1.29 [1.25-1.33]*
40+ - M00-M99										
M00	7,070	50,975,720	0.19	0.13	0.13	0.07	0.07	0.06	1.54 [1.42-1.66]*	1.41 [1.30-1.52]*
M02	840	51,047,870	0.02	0.02	0.02	0.01	0.01	0.00	1.34 [1.07-1.67]	1.23 [0.97-1.54]
M05	9,650	50,935,100	0.25	0.18	0.18	0.07	0.07	0.06	1.37 [1.28-1.47]*	1.29 [1.21-1.38]*
M06	70,210	50,160,110	1.84	1.31	1.33	0.54	0.52	0.38	1.39 [1.36-1.42]*	1.26 [1.23-1.29]*
M07	7,400	50,958,680	0.22	0.13	0.13	0.09	0.09	0.07	1.70 [1.59-1.83]*	1.50 [1.40-1.62]*
M10	83,120	50,446,500	2.12	1.55	1.56	0.58	0.56	0.39	1.36 [1.33-1.39]*	1.23 [1.20-1.26]*
M11	9,460	50,978,620	0.23	0.18	0.18	0.06	0.05	0.03	1.29 [1.20-1.38]*	1.17 [1.09-1.26]*
M12	1,530	51,037,560	0.04	0.03	0.03	0.01	0.01	0.01	1.36 [1.16-1.61]*	1.16 [0.98-1.38]
M13	207,040	49,017,270	5.44	3.96	4.08	1.47	1.36	0.87	1.33 [1.31-1.35]*	1.19 [1.17-1.21]*
M14	2,040	51,038,340	0.06	0.04	0.04	0.02	0.02	0.01	1.62 [1.41-1.87]*	1.31 [1.14-1.52]*
M15	77,580	50,329,230	2.02	1.44	1.48	0.57	0.54	0.35	1.36 [1.33-1.40]*	1.21 [1.18-1.24]*
M16	128,650	49,182,620	2.92	2.55	2.62	0.36	0.30	0.16	1.11 [1.09-1.14]*	1.06 [1.04-1.08]*
M17	204,510	47,786,480	5.19	4.08	4.14	1.10	1.04	0.69	1.25 [1.23-1.27]*	1.15 [1.13-1.17]*
M18	11,340	50,909,240	0.30	0.21	0.21	0.09	0.09	0.06	1.44 [1.35-1.53]*	1.24 [1.17-1.32]*
M19	324,110	48,247,880	8.79	6.28	6.49	2.51	2.30	1.49	1.35 [1.34-1.37]*	1.20 [1.19-1.22]*
M20	41,130	50,058,940	1.02	0.78	0.78	0.24	0.24	0.17	1.31 [1.26-1.35]*	1.21 [1.17-1.25]*
M21	22,370	50,786,790	0.53	0.42	0.43	0.11	0.10	0.06	1.23 [1.18-1.29]*	1.13 [1.08-1.19]*
M22	4,760	50,877,800	0.12	0.09	0.09	0.03	0.03	0.02	1.33 [1.21-1.46]*	1.18 [1.07-1.29]*
M23	68,540	49,075,880	1.63	1.35	1.32	0.29	0.31	0.19	1.23 [1.20-1.27]*	1.13 [1.10-1.16]*
M24	22,110	50,648,670	0.54	0.41	0.42	0.13	0.12	0.07	1.29 [1.23-1.35]*	1.16 [1.11-1.21]*
M25	177,440	48,655,010	4.55	3.45	3.54	1.10	1.01	0.61	1.29 [1.26-1.31]*	1.15 [1.14-1.17]*
M30	810	51,050,740	0.02	0.01	0.02	0.01	0.01	0.00	1.42 [1.13-1.78]*	1.18 [0.93-1.50]
M31	11,460	50,945,160	0.29	0.21	0.22	0.08	0.07	0.05	1.33 [1.25-1.42]*	1.22 [1.15-1.30]*
M32	4,110	50,980,650	0.12	0.07	0.07	0.05	0.05	0.04	1.69 [1.54-1.86]*	1.48 [1.34-1.63]*
M33	1,020	51,047,000	0.03	0.02	0.02	0.02	0.02	0.02	2.02 [1.67-2.43]*	1.92 [1.58-2.32]*
M34	2,030	51,033,040	0.06	0.04	0.04	0.02	0.02	0.02	1.65 [1.44-1.90]*	1.49 [1.29-1.72]*
M35	49,890	50,609,480	1.33	0.91	0.93	0.42	0.40	0.31	1.43 [1.39-1.47]*	1.30 [1.26-1.34]*
M40	12,660	50,999,830	0.31	0.24	0.25	0.07	0.06	0.04	1.24 [1.17-1.32]*	1.15 [1.08-1.22]*
M41	29,740	50,846,100	0.73	0.55	0.58	0.18	0.15	0.10	1.27 [1.22-1.32]*	1.16 [1.11-1.21]*
M43	27,460	50,767,230	0.69	0.51	0.53	0.18	0.16	0.09	1.30 [1.25-1.35]*	1.15 [1.10-1.19]*
M45	6,330	50,964,930	0.17	0.11	0.12	0.06	0.06	0.04	1.48 [1.37-1.61]*	1.31 [1.20-1.42]*
M46	23,210	50,824,720	0.61	0.42	0.43	0.18	0.18	0.11	1.41 [1.35-1.47]*	1.21 [1.16-1.27]*
M47	135,350	49,647,300	3.53	2.55	2.62	0.98	0.91	0.53	1.35 [1.32-1.37]*	1.18 [1.16-1.20]*
M48	79,900	50,326,640	1.99	1.50	1.55	0.49	0.45	0.24	1.29 [1.26-1.32]*	1.14 [1.11-1.16]*
M49	2,780	51,049,910	0.06	0.05	0.05	0.01	0.01	0.01	1.16 [1.01-1.32]	1.12 [0.98-1.28]
M50	20,050	50,820,660	0.50	0.37	0.37	0.12	0.12	0.07	1.33 [1.27-1.39]*	1.15 [1.10-1.21]*
M51	88,370	49,743,600	2.23	1.68	1.69	0.55	0.53	0.28	1.32 [1.29-1.34]*	1.14 [1.12-1.17]*
M53	5,000	50,973,140	0.12	0.09	0.09	0.03	0.03	0.01	1.30 [1.18-1.42]*	1.09 [0.99-1.21]
M54	207,120	47,902,330	5.44	4.08	4.18	1.35	1.26	0.66	1.30 [1.28-1.32]*	1.14 [1.12-1.16]*
M60	3,580	51,016,780	0.10	0.06	0.07	0.03	0.03	0.03	1.48 [1.33-1.65]*	1.38 [1.24-1.54]*
M62	18,120	50,894,960	0.43	0.34	0.35	0.09	0.09	0.06	1.25 [1.19-1.32]*	1.16 [1.10-1.23]*
M65	36,350	50,352,890	0.92	0.68	0.68	0.24	0.24	0.15	1.35 [1.30-1.39]*	1.20 [1.16-1.24]*
M66	4,190	50,986,080	0.10	0.08	0.08	0.02	0.02	0.02	1.30 [1.17-1.44]*	1.18 [1.06-1.31]*
M67	19,770	50,512,190	0.49	0.37	0.37	0.12	0.12	0.08	1.33 [1.27-1.39]*	1.21 [1.15-1.26]*
M70	15,780	50,798,130	0.42	0.29	0.29	0.13	0.13	0.09	1.43 [1.36-1.51]*	1.26 [1.20-1.33]*
M71	10,540	50,926,780	0.26	0.20	0.20	0.07	0.06	0.04	1.32 [1.24-1.41]*	1.19 [1.11-1.27]*
M72	24,430	50,585,450	0.53	0.47	0.47	0.05	0.06	0.03	1.12 [1.07-1.17]*	1.07 [1.02-1.12]*
M75	65,480	49,832,850	1.65	1.24	1.25	0.41	0.40	0.24	1.32 [1.29-1.36]*	1.17 [1.14-1.20]*
M76	4,590	50,997,300	0.12	0.08	0.09	0.04	0.03	0.02	1.41 [1.28-1.55]*	1.21 [1.10-1.33]*
M77	12,120	50,806,020	0.32	0.22	0.22	0.10	0.10	0.07	1.47 [1.39-1.55]*	1.27 [1.19-1.35]*
M79	175,120	48,561,580	4.81	3.35	3.40	1.47	1.41	0.91	1.41 [1.39-1.44]*	1.23 [1.21-1.25]*
M80	25,810	50,870,160	0.64	0.48	0.50	0.16	0.15	0.10	1.30 [1.24-1.35]*	1.19 [1.14-1.24]*
M81	158,100	49,894,880	4.03	2.98	3.10	1.05	0.93	0.60	1.30 [1.28-1.32]*	1.18 [1.16-1.20]*
M83	920	51,049,120	0.02	0.02	0.02	0.01	0.01	0.00	1.28 [1.02-1.59]	1.16 [0.92-1.45]
M84	17,320	50,797,050	0.42	0.32	0.33	0.09	0.09	0.06	1.26 [1.20-1.33]*	1.16 [1.10-1.22]*
M85	32,980	50,873,120	0.83	0.61	0.64	0.22	0.19	0.11	1.29 [1.25-1.34]*	1.16 [1.11-1.20]*
M86	13,640	50,931,610	0.35	0.25	0.25	0.10	0.09	0.06	1.37 [1.30-1.45]*	1.23 [1.16-1.30]*
M87	5,920	50,975,430	0.14	0.11	0.11	0.03	0.03	0.02	1.28 [1.17-1.39]*	1.16 [1.06-1.27]*
M88	4,220	50,989,970	0.09	0.08	0.08	0.01	0.01	0.01	1.18 [1.05-1.31]*	1.13 [1.01-1.27]
M89	13,010	50,826,590	0.32	0.24	0.24	0.08	0.08	0.05	1.32 [1.24-1.40]*	1.18 [1.11-1.25]*
M90	6,970	51,041,940	0.14	0.14	0.14	0.01	0.01	0.00	1.04 [0.96-1.13]	1.02 [0.94-1.12]
M92	920	51,043,500	0.02	0.02	0.02	0.01	0.01	0.01	1.45 [1.17-1.78]*	1.27 [1.02-1.58]

M93	2,060	51,012,090	0.05	0.04	0.04	0.01	0.01	0.01	1.30 [1.13-1.50]*	1.15 [0.99-1.33]
M94	8,210	50,921,420	0.22	0.15	0.15	0.08	0.07	0.05	1.49 [1.39-1.60]*	1.27 [1.18-1.36]*
M95	1,400	51,000,180	0.03	0.03	0.03	0.01	0.01	0.00	1.31 [1.10-1.56]*	1.14 [0.95-1.37]
M96	8,220	50,982,080	0.21	0.15	0.15	0.06	0.05	0.04	1.35 [1.25-1.46]*	1.23 [1.14-1.33]*
M99	2,340	51,040,950	0.06	0.04	0.04	0.01	0.01	0.01	1.32 [1.15-1.52]*	1.11 [0.97-1.29]
40+ - N00-N99										
N02	5,750	50,941,160	0.15	0.11	0.11	0.04	0.04	0.03	1.36 [1.25-1.48]*	1.27 [1.17-1.39]*
N03	26,580	50,855,870	0.66	0.49	0.49	0.17	0.17	0.12	1.35 [1.29-1.41]*	1.22 [1.17-1.28]*
N04	3,150	51,019,170	0.08	0.06	0.06	0.03	0.03	0.02	1.47 [1.31-1.65]*	1.33 [1.19-1.50]*
N05	3,130	51,012,810	0.08	0.06	0.06	0.03	0.03	0.02	1.49 [1.33-1.67]*	1.36 [1.21-1.53]*
N08	11,340	50,966,170	0.30	0.21	0.21	0.10	0.09	0.06	1.43 [1.35-1.52]*	1.22 [1.15-1.31]*
N10	2,850	51,011,270	0.07	0.05	0.05	0.01	0.01	0.01	1.21 [1.07-1.37]*	1.09 [0.96-1.24]
N11	1,860	51,026,130	0.04	0.03	0.03	0.01	0.01	0.01	1.29 [1.11-1.51]*	1.18 [1.01-1.38]
N12	15,700	50,820,600	0.38	0.29	0.30	0.09	0.08	0.04	1.28 [1.21-1.35]*	1.13 [1.07-1.19]*
N13	57,260	50,595,600	1.21	1.12	1.13	0.09	0.08	0.05	1.07 [1.04-1.10]*	1.04 [1.01-1.07]
N14	860	51,056,380	0.02	0.02	0.02	0.01	0.01	0.00	1.41 [1.12-1.77]*	1.26 [1.00-1.59]
N15	1,330	51,047,440	0.03	0.03	0.03	0.01	0.01	0.00	1.21 [1.00-1.46]	1.10 [0.91-1.34]
N16	630	51,054,080	0.02	0.01	0.01	0.01	0.01	0.00	1.51 [1.17-1.95]*	1.34 [1.03-1.75]
N17	346,860	49,910,840	8.45	6.63	6.90	1.83	1.55	1.02	1.23 [1.21-1.24]*	1.14 [1.12-1.15]*
N18	296,040	49,719,170	7.36	5.66	5.87	1.70	1.49	0.93	1.25 [1.24-1.27]*	1.15 [1.13-1.16]*
N19	42,040	50,712,120	1.03	0.79	0.80	0.25	0.23	0.17	1.29 [1.25-1.34]*	1.19 [1.15-1.23]*
N20	43,790	50,267,470	0.98	0.85	0.85	0.13	0.13	0.06	1.15 [1.11-1.19]*	1.07 [1.03-1.10]*
N21	12,350	50,900,450	0.25	0.24	0.24	0.01	0.01	0.00	1.03 [0.96-1.10]	1.00 [0.94-1.07]
N23	8,060	50,742,700	0.18	0.16	0.15	0.02	0.02	0.00	1.14 [1.05-1.23]*	1.01 [0.94-1.09]
N25	2,230	51,037,330	0.06	0.04	0.04	0.02	0.02	0.01	1.39 [1.21-1.60]*	1.28 [1.11-1.48]*
N26	4,600	51,035,350	0.11	0.09	0.09	0.02	0.02	0.01	1.18 [1.06-1.31]*	1.07 [0.96-1.18]
N27	2,150	51,040,720	0.06	0.04	0.04	0.02	0.01	0.01	1.37 [1.19-1.58]*	1.29 [1.11-1.49]*
N28	67,240	50,564,440	1.56	1.28	1.31	0.28	0.25	0.14	1.19 [1.16-1.22]*	1.10 [1.07-1.13]*
N30	29,730	50,551,720	0.70	0.56	0.57	0.13	0.13	0.08	1.23 [1.18-1.28]*	1.14 [1.09-1.18]*
N31	8,000	50,868,690	0.21	0.15	0.15	0.06	0.06	0.04	1.40 [1.30-1.51]*	1.22 [1.13-1.31]*
N32	90,090	49,632,990	2.16	1.74	1.77	0.42	0.39	0.26	1.22 [1.19-1.25]*	1.14 [1.11-1.16]*
N34	1,160	51,032,240	0.03	0.02	0.02	0.01	0.01	0.00	1.25 [1.03-1.52]	1.19 [0.97-1.45]
N35	28,420	50,487,340	0.70	0.53	0.54	0.16	0.16	0.12	1.30 [1.25-1.35]*	1.21 [1.16-1.26]*
N36	7,810	50,920,200	0.18	0.15	0.15	0.04	0.03	0.02	1.23 [1.14-1.32]*	1.15 [1.06-1.24]*
N39	387,940	47,241,490	9.84	7.86	8.20	1.99	1.64	1.06	1.20 [1.19-1.21]*	1.12 [1.11-1.13]*
N40	143,300	49,304,800	3.44	2.79	2.96	0.65	0.48	0.31	1.16 [1.14-1.18]*	1.10 [1.08-1.12]*
N41	10,300	50,881,250	0.22	0.20	0.20	0.02	0.02	0.01	1.11 [1.03-1.18]*	1.05 [0.98-1.12]
N42	13,500	50,853,590	0.29	0.26	0.26	0.03	0.03	0.02	1.13 [1.06-1.20]*	1.08 [1.01-1.14]
N43	11,260	50,842,980	0.25	0.21	0.21	0.04	0.04	0.03	1.19 [1.12-1.27]*	1.14 [1.07-1.22]*
N45	8,340	50,899,600	0.20	0.16	0.16	0.04	0.04	0.03	1.25 [1.16-1.35]*	1.15 [1.07-1.24]*
N47	19,550	50,699,390	0.49	0.36	0.36	0.13	0.14	0.11	1.38 [1.32-1.45]*	1.28 [1.22-1.35]*
N48	16,280	50,760,480	0.42	0.30	0.30	0.12	0.12	0.09	1.40 [1.33-1.48]*	1.29 [1.23-1.36]*
N49	2,520	51,023,580	0.06	0.05	0.05	0.02	0.02	0.01	1.41 [1.23-1.60]*	1.28 [1.12-1.47]*
N50	17,650	50,664,170	0.42	0.33	0.33	0.08	0.09	0.06	1.26 [1.20-1.33]*	1.17 [1.11-1.23]*
N60	8,450	50,778,840	0.18	0.16	0.17	0.02	0.02	0.01	1.11 [1.03-1.19]*	1.05 [0.97-1.13]
N61	3,970	50,941,190	0.10	0.07	0.07	0.03	0.03	0.02	1.39 [1.25-1.54]*	1.25 [1.12-1.39]*
N62	3,450	50,922,020	0.08	0.06	0.06	0.02	0.02	0.01	1.27 [1.13-1.42]*	1.11 [0.99-1.25]
N63	9,150	50,784,980	0.21	0.17	0.18	0.03	0.03	0.01	1.16 [1.08-1.25]*	1.08 [1.00-1.16]
N64	8,290	50,864,260	0.19	0.16	0.16	0.04	0.03	0.02	1.21 [1.12-1.30]*	1.11 [1.03-1.20]*
N70	4,170	50,911,340	0.09	0.08	0.08	0.01	0.00	0.00	1.06 [0.95-1.18]	1.00 [0.89-1.11]
N71	2,420	50,991,040	0.06	0.05	0.05	0.01	0.01	0.01	1.25 [1.09-1.42]*	1.13 [0.99-1.30]
N72	4,020	50,832,040	0.09	0.08	0.08	0.01	0.01	0.01	1.12 [1.01-1.24]	1.07 [0.96-1.19]
N73	20,330	50,301,110	0.45	0.39	0.40	0.06	0.05	0.02	1.13 [1.08-1.18]*	1.04 [0.99-1.09]
N75	2,780	50,917,450	0.06	0.05	0.05	0.00	0.00	0.00	1.06 [0.93-1.20]	1.02 [0.89-1.16]
N76	5,800	50,888,620	0.16	0.10	0.11	0.06	0.05	0.04	1.51 [1.39-1.64]*	1.37 [1.26-1.49]*
N77	4,580	50,961,170	0.12	0.08	0.09	0.04	0.03	0.02	1.40 [1.27-1.54]*	1.19 [1.07-1.31]*
N80	19,480	50,218,740	0.42	0.38	0.39	0.04	0.03	0.00	1.09 [1.03-1.14]*	1.00 [0.95-1.05]
N81	62,640	49,529,080	1.48	1.22	1.24	0.26	0.24	0.15	1.20 [1.17-1.23]*	1.12 [1.09-1.15]*
N82	2,740	51,021,850	0.07	0.05	0.05	0.02	0.02	0.01	1.30 [1.15-1.48]*	1.19 [1.05-1.36]*
N83	35,460	49,962,450	0.79	0.69	0.71	0.10	0.08	0.03	1.12 [1.08-1.16]*	1.04 [1.00-1.07]
N84	49,890	49,695,760	1.11	0.98	0.99	0.13	0.12	0.09	1.12 [1.09-1.16]*	1.09 [1.06-1.13]*
N85	35,810	50,197,380	0.82	0.69	0.70	0.14	0.12	0.08	1.18 [1.14-1.22]*	1.11 [1.07-1.15]*
N86	3,500	50,806,740	0.08	0.07	0.07	0.01	0.01	0.01	1.23 [1.10-1.37]*	1.17 [1.04-1.31]*
N87	5,150	50,558,960	0.11	0.10	0.10	0.01	0.01	0.00	1.06 [0.97-1.17]	1.01 [0.91-1.11]
N88	10,980	50,776,480	0.25	0.21	0.21	0.04	0.04	0.03	1.19 [1.12-1.27]*	1.12 [1.05-1.20]*
N89	9,340	50,706,230	0.23	0.17	0.18	0.06	0.05	0.04	1.30 [1.22-1.39]*	1.21 [1.13-1.29]*
N90	13,870	50,691,310	0.37	0.25	0.26	0.12	0.11	0.09	1.43 [1.36-1.51]*	1.32 [1.25-1.39]*
N91	640	51,025,050	0.02	0.01	0.01	0.01	0.01	0.01	1.52 [1.19-1.93]*	1.41 [1.10-1.81]*
N92	58,270	48,611,360	1.39	1.16	1.18	0.24	0.21	0.11	1.18 [1.15-1.21]*	1.09 [1.06-1.12]*
N93	20,700	50,194,750	0.48	0.40	0.40	0.08	0.07	0.04	1.18 [1.13-1.24]*	1.09 [1.04-1.14]*
N94	13,520	50,229,810	0.33	0.26	0.26	0.07	0.07	0.04	1.27 [1.20-1.34]*	1.14 [1.08-1.21]*
N95	48,440	49,820,420	1.18	0.93	0.95	0.25	0.23	0.17	1.24 [1.21-1.28]*	1.17 [1.14-1.21]*
N97	740	50,640,650	0.01	0.01	0.01	0.00	0.00	0.00	0.97 [0.75-1.26]	0.98 [0.76-1.27]
N99	9,740	50,859,510	0.23	0.18	0.18	0.04	0.04	0.03	1.22 [1.14-1.31]*	1.14 [1.06-1.22]*
40+ - O00-O9A										
O00	800	50,856,230	0.02	0.02	0.02	0.00	0.00	0.00	1.03 [0.81-1.31]	1.02 [0.80-1.30]
O02	3,470	50,399,380	0.07	0.07	0.07	0.00	0.00	0.00	1.02 [0.91-1.15]	1.00 [0.89-1.13]
O03	5,060	50,210,800	0.11	0.10	0.10	0.01	0.00	0.00	1.04 [0.95-1.14]	1.03 [0.94-1.14]
O04	3,100	50,308,600	0.06	0.06	0.06	0.00	−0.01	−0.01	0.92 [0.81-1.04]	0.90 [0.79-1.03]
O10	790	50,987,040	0.02	0.01	0.01	0.00	0.00	0.00	1.27 [1.01-1.59]	1.06 [0.83-1.34]
O13	1,690	50,552,980	0.03	0.03	0.03	0.00	0.00	0.00	0.96 [0.81-1.14]	0.96 [0.81-1.14]
O14	1,180	50,732,120	0.02	0.02	0.02	0.00	0.00	0.00	0.98 [0.80-1.20]	0.96 [0.78-1.18]
O16	1,360	50,526,200	0.03	0.03	0.03	0.00	0.00	0.00	1.03 [0.86-1.24]	1.02 [0.84-1.24]
O20	2,010	50,437,370	0.04	0.04	0.04	0.00	0.00	0.00	0.97 [0.83-1.13]	0.98 [0.84-1.15]
O23	840	50,711,160	0.02	0.02	0.02	0.00	0.00	0.00	0.93 [0.73-1.19]	0.89 [0.69-1.14]
O24	3,710	50,710,150	0.08	0.07	0.07	0.00	0.00	0.00	1.04 [0.93-1.16]	1.03 [0.92-1.16]
O26	4,120	49,332,280	0.09	0.08	0.09	0.00	0.00	−0.01	0.95 [0.85-1.05]	0.92 [0.83-1.03]
O28	720	50,966,200	0.01	0.01	0.01	0.00	0.00	0.00	0.97 [0.75-1.25]	0.94 [0.72-1.23]
O30	1,100	50,841,560	0.02	0.02	0.02	0.00	0.00	0.00	0.86 [0.69-1.07]	0.88 [0.70-1.10]
O32	3,370	50,219,210	0.07	0.07	0.07	0.00	0.00	0.00	0.96 [0.85-1.08]	0.98 [0.87-1.11]
O34	7,340	49,685,010	0.15	0.15	0.15	0.01	0.00	0.00	1.00 [0.93-1.08]	1.03 [0.95-1.12]
O35	1,420	50,902,510	0.03	0.03	0.03	0.00	0.00	0.00	0.97 [0.80-1.16]	0.95 [0.78-1.15]
O36	5,790	49,331,580	0.13	0.11	0.13	0.01	0.00	0.00	1.02 [0.93-1.11]	1.02 [0.93-1.11]
O40	940	50,942,470	0.02	0.02	0.02	0.00	0.00	0.00	0.95 [0.76-1.19]	0.96 [0.76-1.22]
O41	770	50,802,530	0.01	0.02	0.01	0.00	0.00	0.00	0.99 [0.77-1.27]	1.05 [0.81-1.36]
O42	3,440	49,887,940	0.06	0.07	0.07	−0.01	−0.01	−0.01	0.84 [0.75-0.95]*	0.89 [0.78-1.00]
O43	620	50,856,460	0.01	0.01	0.01	0.00	0.00	0.00	0.94 [0.71-1.25]	0.92 [0.69-1.23]
O44	790	50,915,210	0.01	0.02	0.02	0.00	0.00	0.00	0.98 [0.76-1.25]	0.97 [0.75-1.26]
O46	1,800	50,423,420	0.04	0.04	0.04	0.00	0.00	0.00	1.04 [0.89-1.22]	1.04 [0.88-1.22]
O47	850	50,093,870	0.02	0.02	0.02	0.00	0.00	0.00	0.95 [0.75-1.20]	0.91 [0.71-1.16]
O48	1,800	50,013,340	0.03	0.04	0.04	−0.01	−0.01	0.00	0.84 [0.71-1.00]	0.89 [0.75-1.06]
O60	2,540	50,383,810	0.05	0.05	0.05	0.00	0.00	0.00	1.06 [0.93-1.21]	1.03 [0.90-1.18]
O61	700	50,899,040	0.01	0.01	0.01	0.00	0.00	0.00	0.98 [0.75-1.27]	0.92 [0.70-1.21]
O62	1,570	50,623,230	0.03	0.03	0.03	0.00	0.00	0.00	0.96 [0.81-1.15]	0.99 [0.83-1.19]
O63	2,810	49,613,830	0.06	0.06	0.06	0.00	−0.01	0.00	0.91 [0.80-1.03]	0.96 [0.84-1.10]
O66	690	50,787,050	0.01	0.01	0.01	0.00	0.00	0.00	0.95 [0.73-1.24]	0.97 [0.74-1.28]
O68	6,150	48,362,750	0.14	0.13	0.15	0.01	−0.01	−0.01	0.93 [0.85-1.01]	0.95 [0.87-1.04]

O69	760	50,588,540	0.02	0.01	0.01	0.00	0.00	0.00	1.18 [0.93-1.49]	1.22 [0.95-1.56]
O70	5,740	47,338,080	0.14	0.12	0.15	0.02	−0.01	0.00	0.93 [0.85-1.01]	1.00 [0.92-1.10]
O72	5,900	49,797,520	0.12	0.12	0.13	0.00	0.00	0.00	0.97 [0.89-1.06]	1.00 [0.92-1.10]
O75	2,460	50,317,650	0.05	0.05	0.05	0.00	0.00	0.00	0.91 [0.79-1.05]	0.97 [0.83-1.12]
O80	1,620	49,503,870	0.04	0.03	0.03	0.00	0.00	0.00	1.05 [0.89-1.24]	1.11 [0.94-1.32]
O82	780	50,708,280	0.01	0.02	0.02	0.00	0.00	0.00	0.86 [0.67-1.11]	0.91 [0.69-1.18]
O86	980	50,851,790	0.02	0.02	0.02	0.00	0.00	0.00	1.20 [0.97-1.47]	1.16 [0.93-1.43]
O90	1,250	50,865,190	0.03	0.02	0.02	0.00	0.00	0.00	1.10 [0.91-1.33]	1.08 [0.89-1.31]
O99	6,040	49,408,590	0.14	0.12	0.13	0.02	0.01	0.00	1.06 [0.97-1.15]	1.02 [0.93-1.11]
40+ - Q00-Q99										
Q05	1,210	51,029,190	0.03	0.02	0.02	0.01	0.01	0.00	1.32 [1.10-1.60]*	1.16 [0.96-1.41]
Q07	650	51,050,160	0.02	0.01	0.01	0.00	0.00	0.00	1.31 [1.02-1.69]	1.10 [0.84-1.43]
Q12	610	51,048,900	0.01	0.01	0.01	0.00	0.00	0.00	1.23 [0.93-1.62]	1.18 [0.88-1.56]
Q18	730	51,034,680	0.02	0.01	0.01	0.00	0.00	0.00	1.18 [0.92-1.50]	1.10 [0.86-1.42]
Q21	5,110	50,977,840	0.12	0.10	0.10	0.03	0.03	0.02	1.28 [1.16-1.40]*	1.16 [1.06-1.28]*
Q23	2,820	51,028,170	0.06	0.05	0.05	0.01	0.01	0.01	1.14 [1.00-1.30]	1.10 [0.96-1.25]
Q24	1,510	51,038,180	0.04	0.03	0.03	0.01	0.01	0.01	1.40 [1.19-1.66]*	1.24 [1.04-1.47]
Q25	1,070	51,044,910	0.02	0.02	0.02	0.00	0.00	0.00	1.19 [0.97-1.46]	1.08 [0.87-1.33]
Q27	1,340	51,041,150	0.03	0.03	0.03	0.00	0.00	0.00	1.19 [0.99-1.43]	1.11 [0.91-1.34]
Q28	1,020	51,040,250	0.03	0.02	0.02	0.01	0.01	0.01	1.36 [1.11-1.66]*	1.25 [1.02-1.54]
Q38	2,850	51,029,560	0.07	0.05	0.06	0.01	0.01	0.01	1.20 [1.05-1.37]*	1.10 [0.97-1.26]
Q39	1,500	51,045,930	0.04	0.03	0.03	0.01	0.01	0.01	1.29 [1.08-1.53]*	1.16 [0.97-1.39]
Q40	900	51,052,630	0.02	0.02	0.02	0.00	0.00	0.00	1.17 [0.94-1.47]	1.03 [0.82-1.30]
Q43	2,500	51,026,550	0.06	0.05	0.05	0.01	0.01	0.01	1.22 [1.07-1.39]*	1.10 [0.96-1.26]
Q44	1,010	51,050,000	0.02	0.02	0.02	0.00	0.00	0.00	1.11 [0.90-1.38]	1.01 [0.81-1.26]
Q50	1,050	51,015,110	0.02	0.02	0.02	0.00	0.00	0.00	1.12 [0.91-1.37]	1.04 [0.84-1.28]
Q51	1,300	51,021,230	0.03	0.02	0.02	0.00	0.00	0.00	1.20 [1.00-1.44]	1.08 [0.89-1.31]
Q52	450	51,050,330	0.01	0.01	0.01	0.00	0.00	0.00	1.45 [1.08-1.96]	1.32 [0.97-1.80]
Q54	1,270	51,043,560	0.03	0.02	0.02	0.01	0.00	0.00	1.17 [0.96-1.41]	1.12 [0.92-1.36]
Q60	1,830	51,039,870	0.04	0.03	0.04	0.01	0.01	0.00	1.23 [1.05-1.44]	1.13 [0.96-1.32]
Q61	5,560	50,985,020	0.13	0.11	0.11	0.02	0.02	0.01	1.19 [1.08-1.30]*	1.10 [1.01-1.21]
Q63	2,890	51,020,860	0.06	0.06	0.06	0.01	0.01	0.00	1.09 [0.96-1.24]	0.97 [0.85-1.10]
Q65	1,180	51,034,930	0.03	0.02	0.02	0.00	0.00	0.00	1.18 [0.97-1.43]	1.08 [0.88-1.32]
Q66	1,410	51,032,650	0.04	0.02	0.03	0.02	0.02	0.01	1.60 [1.36-1.89]*	1.44 [1.21-1.71]*
Q67	730	51,050,250	0.02	0.01	0.01	0.01	0.01	0.01	1.56 [1.23-1.99]*	1.47 [1.15-1.87]*
Q74	710	51,049,320	0.02	0.01	0.01	0.00	0.00	0.00	1.35 [1.06-1.72]	1.18 [0.91-1.52]
Q76	1,340	51,037,170	0.03	0.02	0.02	0.01	0.01	0.00	1.35 [1.13-1.61]*	1.11 [0.92-1.34]
Q78	780	51,051,370	0.02	0.01	0.02	0.01	0.00	0.00	1.30 [1.03-1.63]	1.12 [0.88-1.42]
Q79	1,190	51,045,600	0.03	0.02	0.02	0.00	0.00	0.00	1.19 [0.98-1.45]	0.91 [0.74-1.12]
Q82	990	51,040,910	0.03	0.02	0.02	0.01	0.01	0.01	1.81 [1.49-2.21]*	1.58 [1.29-1.94]*
Q85	1,140	51,038,160	0.02	0.02	0.02	0.00	0.00	0.00	1.08 [0.88-1.32]	1.00 [0.81-1.24]
Q87	870	51,039,250	0.02	0.02	0.02	0.01	0.01	0.01	1.59 [1.29-1.97]*	1.42 [1.14-1.77]*
Q89	1,000	51,041,070	0.02	0.02	0.02	0.00	0.00	0.00	1.15 [0.93-1.43]	1.08 [0.87-1.34]
Q90	1,120	51,012,000	0.06	0.01	0.04	0.04	0.04	0.04	4.16 [3.54-4.89]*	4.05 [3.44-4.78]*
40+ - R00-R99										
R00	154,590	49,519,820	3.73	2.99	3.07	0.74	0.66	0.37	1.22 [1.20-1.24]*	1.11 [1.09-1.13]*
R01	21,740	50,859,420	0.52	0.41	0.42	0.11	0.10	0.06	1.23 [1.18-1.29]*	1.14 [1.08-1.19]*
R02	27,160	50,904,940	0.64	0.51	0.53	0.14	0.12	0.07	1.22 [1.17-1.28]*	1.12 [1.07-1.17]*
R03	54,600	50,501,440	1.28	1.04	1.06	0.24	0.22	0.15	1.20 [1.17-1.24]*	1.13 [1.10-1.16]*
R04	63,950	50,218,000	1.63	1.20	1.21	0.43	0.41	0.28	1.34 [1.31-1.38]*	1.21 [1.18-1.24]*
R05	54,200	50,521,300	1.39	1.00	1.03	0.39	0.37	0.22	1.36 [1.32-1.39]*	1.19 [1.15-1.22]*
R06	153,380	49,259,560	3.96	2.93	2.98	1.03	0.99	0.59	1.33 [1.31-1.35]*	1.18 [1.16-1.20]*
R07	267,960	45,545,490	7.35	5.56	5.61	1.78	1.73	1.00	1.31 [1.29-1.33]*	1.16 [1.14-1.17]*
R09	24,530	50,834,620	0.60	0.46	0.46	0.15	0.14	0.09	1.31 [1.25-1.36]*	1.17 [1.11-1.22]*
R10	243,520	44,601,240	6.80	5.16	5.22	1.64	1.58	0.93	1.30 [1.29-1.32]*	1.16 [1.14-1.18]*
R11	171,590	48,900,060	4.27	3.34	3.41	0.93	0.87	0.50	1.25 [1.23-1.27]*	1.13 [1.11-1.15]*
R12	14,020	50,777,760	0.35	0.26	0.26	0.09	0.09	0.05	1.34 [1.27-1.42]*	1.17 [1.11-1.24]*
R13	121,980	50,012,610	3.09	2.30	2.35	0.80	0.74	0.48	1.31 [1.29-1.34]*	1.18 [1.16-1.21]*
R14	23,820	50,780,200	0.58	0.44	0.45	0.14	0.14	0.07	1.30 [1.25-1.36]*	1.15 [1.10-1.20]*
R15	52,810	50,808,340	1.25	1.00	1.05	0.25	0.20	0.14	1.19 [1.15-1.23]*	1.12 [1.09-1.16]*
R16	16,440	50,930,150	0.43	0.30	0.30	0.13	0.13	0.09	1.41 [1.34-1.49]*	1.27 [1.20-1.34]*
R17	15,360	50,905,080	0.36	0.29	0.29	0.07	0.07	0.05	1.23 [1.17-1.30]*	1.17 [1.11-1.24]*
R18	41,020	50,850,550	0.95	0.78	0.78	0.18	0.17	0.13	1.22 [1.18-1.26]*	1.15 [1.11-1.19]*
R19	141,980	49,191,500	3.66	2.72	2.74	0.94	0.91	0.59	1.33 [1.31-1.36]*	1.19 [1.17-1.21]*
R20	32,460	50,545,700	0.79	0.61	0.62	0.18	0.17	0.08	1.27 [1.23-1.32]*	1.11 [1.07-1.15]*
R21	34,340	50,635,460	1.07	0.60	0.60	0.47	0.47	0.41	1.78 [1.72-1.84]*	1.63 [1.58-1.69]*
R22	23,860	50,647,180	0.61	0.44	0.45	0.17	0.16	0.11	1.36 [1.31-1.42]*	1.23 [1.18-1.29]*
R23	14,860	50,940,630	0.37	0.28	0.28	0.09	0.09	0.06	1.33 [1.26-1.41]*	1.21 [1.14-1.28]*
R25	20,420	50,877,980	0.50	0.38	0.39	0.12	0.11	0.06	1.28 [1.22-1.35]*	1.14 [1.09-1.20]*
R26	195,630	50,335,360	4.74	3.70	3.95	1.04	0.80	0.50	1.20 [1.18-1.22]*	1.12 [1.10-1.14]*
R27	5,600	51,005,730	0.13	0.11	0.11	0.03	0.02	0.01	1.20 [1.09-1.31]*	1.09 [0.99-1.20]
R29	261,830	50,125,150	6.32	4.99	5.31	1.34	1.01	0.60	1.19 [1.17-1.21]*	1.11 [1.09-1.12]*
R30	13,690	50,821,060	0.35	0.25	0.26	0.10	0.09	0.05	1.36 [1.29-1.44]*	1.18 [1.11-1.25]*
R31	130,760	48,722,830	3.12	2.59	2.63	0.53	0.48	0.31	1.18 [1.16-1.21]*	1.11 [1.09-1.13]*
R32	99,600	50,469,900	2.35	1.89	2.00	0.45	0.35	0.22	1.17 [1.15-1.20]*	1.10 [1.08-1.13]*
R33	191,170	49,675,710	4.52	3.70	3.90	0.81	0.62	0.40	1.16 [1.14-1.18]*	1.10 [1.08-1.12]*
R34	4,660	51,041,130	0.11	0.09	0.09	0.03	0.02	0.01	1.26 [1.14-1.39]*	1.15 [1.03-1.27]*
R35	27,640	50,422,950	0.69	0.52	0.52	0.17	0.17	0.11	1.32 [1.27-1.38]*	1.19 [1.14-1.24]*
R36	570	51,054,680	0.01	0.01	0.01	0.00	0.00	0.00	1.39 [1.05-1.85]	1.24 [0.92-1.66]
R39	32,840	50,387,690	0.82	0.62	0.62	0.20	0.20	0.13	1.33 [1.28-1.38]*	1.19 [1.15-1.23]*
R40	26,600	50,894,060	0.65	0.50	0.52	0.15	0.12	0.07	1.24 [1.18-1.29]*	1.12 [1.08-1.17]*
R41	188,980	50,162,900	4.47	3.62	3.81	0.85	0.66	0.43	1.17 [1.15-1.19]*	1.11 [1.09-1.13]*
R42	89,870	50,066,350	2.19	1.71	1.76	0.48	0.43	0.23	1.24 [1.22-1.27]*	1.12 [1.09-1.15]*
R43	1,100	51,051,570	0.03	0.02	0.02	0.01	0.01	0.00	1.36 [1.11-1.66]*	1.14 [0.92-1.41]
R44	23,180	50,928,440	0.55	0.44	0.45	0.11	0.10	0.06	1.22 [1.17-1.28]*	1.12 [1.07-1.17]*
R45	41,290	50,843,770	0.99	0.77	0.80	0.21	0.19	0.10	1.24 [1.20-1.28]*	1.11 [1.07-1.15]*
R46	15,420	50,991,930	0.34	0.29	0.30	0.05	0.04	0.02	1.12 [1.06-1.19]*	1.07 [1.01-1.13]
R47	50,890	50,700,370	1.17	0.97	1.01	0.20	0.16	0.08	1.16 [1.13-1.20]*	1.08 [1.04-1.11]*
R48	780	51,055,800	0.02	0.01	0.01	0.01	0.00	0.00	1.31 [1.03-1.66]	1.13 [0.88-1.45]
R49	9,190	50,884,460	0.23	0.17	0.17	0.06	0.06	0.03	1.32 [1.24-1.42]*	1.14 [1.07-1.23]*
R50	58,530	50,402,530	1.43	1.10	1.12	0.32	0.30	0.21	1.27 [1.24-1.31]*	1.17 [1.14-1.21]*
R51	85,750	49,412,160	2.20	1.64	1.66	0.56	0.54	0.29	1.32 [1.29-1.35]*	1.15 [1.13-1.18]*
R52	20,000	50,843,250	0.52	0.37	0.37	0.16	0.15	0.09	1.42 [1.35-1.48]*	1.21 [1.15-1.27]*
R53	86,400	50,484,940	2.08	1.63	1.70	0.45	0.38	0.22	1.22 [1.19-1.25]*	1.12 [1.09-1.14]*
R54	127,830	50,611,500	3.00	2.43	2.57	0.57	0.43	0.29	1.17 [1.14-1.19]*	1.11 [1.08-1.13]*
R55	168,480	49,214,460	4.07	3.28	3.38	0.79	0.69	0.45	1.20 [1.18-1.22]*	1.12 [1.10-1.14]*
R56	42,880	50,599,490	1.03	0.81	0.82	0.23	0.21	0.15	1.26 [1.22-1.30]*	1.16 [1.13-1.20]*
R57	15,970	51,025,010	0.37	0.30	0.31	0.07	0.05	0.03	1.18 [1.11-1.24]*	1.10 [1.04-1.16]*
R58	2,310	51,046,040	0.06	0.04	0.04	0.02	0.02	0.01	1.34 [1.17-1.54]*	1.22 [1.06-1.41]*
R59	41,420	50,657,440	1.03	0.77	0.78	0.26	0.25	0.20	1.32 [1.28-1.37]*	1.24 [1.20-1.29]*
R60	88,320	50,597,450	2.23	1.64	1.67	0.59	0.57	0.41	1.34 [1.31-1.37]*	1.22 [1.19-1.25]*
R61	7,100	50,951,720	0.18	0.13	0.13	0.05	0.05	0.03	1.34 [1.24-1.45]*	1.17 [1.08-1.27]*
R62	1,020	51,054,970	0.03	0.02	0.02	0.01	0.01	0.00	1.26 [1.02-1.56]	1.18 [0.95-1.46]
R63	163,750	49,789,660	4.01	3.13	3.22	0.88	0.79	0.50	1.24 [1.22-1.27]*	1.14 [1.12-1.16]*
R64	5,150	51,053,120	0.11	0.10	0.10	0.01	0.01	0.01	1.10 [1.00-1.22]	1.08 [0.97-1.20]
R65	11,030	51,037,250	0.26	0.21	0.21	0.06	0.05	0.03	1.23 [1.15-1.32]*	1.13 [1.05-1.21]*
R68	16,230	50,972,260	0.39	0.30	0.31	0.08	0.07	0.06	1.24 [1.17-1.31]*	1.17 [1.11-1.24]*

R69	128,150	47,668,880	3.22	2.57	2.59	0.65	0.63	0.44	1.24 [1.22-1.27]*	1.16 [1.14-1.18]*
R70	1,220	51,035,870	0.03	0.02	0.02	0.01	0.01	0.01	1.48 [1.23-1.77]*	1.37 [1.13-1.65]*
R71	1,010	51,052,870	0.02	0.02	0.02	0.00	0.00	0.00	1.13 [0.91-1.40]	1.06 [0.85-1.33]
R72	5,180	51,020,190	0.12	0.10	0.10	0.02	0.02	0.01	1.20 [1.09-1.32]*	1.13 [1.03-1.25]
R73	20,780	50,879,400	0.52	0.39	0.39	0.13	0.12	0.07	1.31 [1.25-1.38]*	1.16 [1.11-1.22]*
R74	8,640	50,996,020	0.20	0.16	0.17	0.04	0.03	0.02	1.21 [1.12-1.30]*	1.10 [1.02-1.19]
R76	2,070	51,028,640	0.05	0.04	0.04	0.02	0.01	0.01	1.38 [1.20-1.60]*	1.26 [1.09-1.46]*
R77	5,610	51,034,410	0.14	0.10	0.11	0.03	0.03	0.02	1.27 [1.16-1.40]*	1.17 [1.06-1.28]*
R78	740	51,051,060	0.02	0.01	0.02	0.00	0.00	0.00	1.12 [0.88-1.45]	1.02 [0.79-1.32]
R79	119,070	50,142,330	2.89	2.26	2.33	0.62	0.56	0.35	1.24 [1.22-1.26]*	1.14 [1.11-1.16]*
R80	3,400	50,996,480	0.09	0.06	0.06	0.03	0.03	0.02	1.45 [1.29-1.62]*	1.31 [1.17-1.47]*
R82	7,610	50,984,340	0.17	0.14	0.15	0.03	0.02	0.01	1.16 [1.07-1.25]*	1.06 [0.98-1.15]
R84	2,580	51,047,160	0.07	0.05	0.05	0.02	0.02	0.01	1.45 [1.28-1.65]*	1.24 [1.09-1.42]*
R85	1,410	51,019,260	0.03	0.03	0.03	0.01	0.01	0.00	1.32 [1.11-1.58]*	1.16 [0.97-1.39]
R87	3,940	50,652,250	0.08	0.08	0.08	0.01	0.01	0.00	1.08 [0.97-1.21]	1.03 [0.92-1.15]
R89	1,360	51,047,170	0.04	0.02	0.02	0.01	0.01	0.01	1.47 [1.23-1.75]*	1.35 [1.13-1.62]*
R90	4,770	51,022,770	0.11	0.09	0.09	0.01	0.01	0.01	1.15 [1.04-1.27]*	1.08 [0.98-1.20]
R91	60,460	50,650,720	1.46	1.14	1.17	0.32	0.29	0.19	1.25 [1.22-1.29]*	1.15 [1.12-1.18]*
R92	560	51,055,190	0.01	0.01	0.01	0.00	0.00	0.00	1.07 [0.80-1.44]	1.01 [0.75-1.37]
R93	57,480	50,576,680	1.34	1.09	1.12	0.25	0.23	0.14	1.20 [1.17-1.24]*	1.11 [1.08-1.15]*
R94	97,580	50,269,430	2.39	1.84	1.90	0.55	0.49	0.34	1.26 [1.23-1.29]*	1.16 [1.14-1.19]*
40+ - S00-T88										
S00	89,310	50,460,570	2.13	1.69	1.77	0.44	0.36	0.23	1.20 [1.17-1.23]*	1.12 [1.09-1.15]*
S01	87,380	50,298,170	2.09	1.66	1.74	0.43	0.36	0.25	1.20 [1.18-1.23]*	1.14 [1.11-1.17]*
S02	20,990	50,641,960	0.49	0.40	0.40	0.09	0.09	0.06	1.21 [1.16-1.27]*	1.14 [1.09-1.20]*
S03	520	51,054,710	0.01	0.01	0.01	0.00	0.00	0.00	1.16 [0.86-1.57]	1.07 [0.79-1.45]
S05	7,860	50,966,860	0.18	0.15	0.15	0.03	0.03	0.02	1.21 [1.11-1.31]*	1.14 [1.05-1.23]*
S06	27,530	50,839,170	0.62	0.52	0.55	0.09	0.07	0.05	1.14 [1.09-1.19]*	1.08 [1.03-1.13]*
S09	39,960	50,552,650	0.97	0.75	0.77	0.22	0.20	0.13	1.25 [1.21-1.30]*	1.15 [1.11-1.19]*
S10	1,920	51,034,480	0.04	0.04	0.04	0.01	0.00	0.00	1.09 [0.93-1.29]	0.97 [0.82-1.15]
S11	910	51,046,210	0.02	0.02	0.02	0.01	0.01	0.01	1.46 [1.17-1.82]*	1.34 [1.06-1.68]
S12	7,500	51,002,670	0.17	0.14	0.15	0.03	0.03	0.02	1.18 [1.08-1.28]*	1.10 [1.01-1.20]
S13	1,250	51,030,320	0.03	0.02	0.02	0.01	0.01	0.00	1.21 [1.00-1.47]	1.12 [0.92-1.36]
S14	1,250	51,045,880	0.03	0.02	0.02	0.00	0.00	0.00	1.08 [0.89-1.32]	1.06 [0.87-1.30]
S19	1,570	51,029,330	0.04	0.03	0.03	0.01	0.01	0.00	1.28 [1.08-1.52]*	1.11 [0.93-1.32]
S20	10,170	50,970,910	0.25	0.19	0.20	0.06	0.05	0.03	1.25 [1.17-1.34]*	1.13 [1.05-1.21]*
S21	1,150	51,043,830	0.03	0.02	0.02	0.01	0.01	0.01	1.45 [1.19-1.77]*	1.32 [1.08-1.63]*
S22	37,920	50,710,200	0.90	0.71	0.73	0.19	0.17	0.10	1.24 [1.19-1.28]*	1.13 [1.09-1.17]*
S27	9,750	50,949,500	0.21	0.19	0.19	0.03	0.03	0.02	1.15 [1.07-1.23]*	1.08 [1.00-1.16]
S29	2,720	51,031,230	0.07	0.05	0.05	0.02	0.02	0.02	1.48 [1.30-1.68]*	1.30 [1.14-1.48]*
S30	16,650	50,919,870	0.40	0.31	0.31	0.09	0.09	0.06	1.29 [1.22-1.36]*	1.17 [1.11-1.24]*
S31	2,520	51,015,690	0.06	0.05	0.05	0.02	0.02	0.01	1.36 [1.19-1.56]*	1.21 [1.05-1.39]*
S32	42,330	50,740,110	0.97	0.81	0.84	0.16	0.13	0.09	1.16 [1.12-1.20]*	1.10 [1.06-1.14]*
S36	2,890	51,020,890	0.07	0.05	0.06	0.01	0.01	0.01	1.17 [1.03-1.33]	1.13 [0.99-1.28]
S37	2,740	51,033,330	0.06	0.05	0.06	0.00	0.00	0.00	1.02 [0.89-1.17]	0.98 [0.85-1.13]
S39	5,970	50,975,010	0.14	0.11	0.11	0.03	0.03	0.01	1.22 [1.12-1.34]*	1.10 [1.00-1.21]
S40	15,260	50,973,710	0.36	0.29	0.30	0.08	0.06	0.04	1.21 [1.14-1.28]*	1.11 [1.04-1.18]*
S41	5,020	51,026,960	0.13	0.09	0.10	0.04	0.03	0.02	1.34 [1.21-1.48]*	1.21 [1.09-1.34]*
S42	45,110	50,582,710	1.02	0.86	0.89	0.15	0.13	0.08	1.14 [1.10-1.18]*	1.08 [1.05-1.12]*
S43	7,940	50,958,290	0.17	0.15	0.16	0.02	0.02	0.01	1.09 [1.01-1.19]	1.05 [0.97-1.14]
S46	3,160	51,027,860	0.07	0.06	0.06	0.01	0.00	0.00	1.07 [0.95-1.22]	0.97 [0.85-1.10]
S49	4,770	51,022,330	0.12	0.09	0.09	0.03	0.02	0.02	1.27 [1.14-1.40]*	1.15 [1.04-1.28]*
S50	15,840	50,974,110	0.38	0.30	0.31	0.08	0.07	0.04	1.23 [1.16-1.30]*	1.13 [1.07-1.20]*
S51	21,730	50,917,850	0.53	0.40	0.43	0.13	0.11	0.06	1.25 [1.19-1.31]*	1.13 [1.07-1.19]*
S52	54,650	50,175,110	1.20	1.07	1.08	0.13	0.12	0.07	1.11 [1.07-1.14]*	1.07 [1.03-1.10]*
S53	1,160	51,037,270	0.02	0.02	0.02	0.00	0.00	0.00	1.07 [0.87-1.31]	0.98 [0.80-1.21]
S54	460	51,046,080	0.01	0.01	0.01	0.00	0.00	0.00	1.35 [1.00-1.83]	1.28 [0.94-1.75]
S56	1,580	51,016,180	0.03	0.03	0.03	0.00	0.00	0.00	1.09 [0.91-1.29]	1.05 [0.87-1.25]
S59	1,720	51,048,150	0.04	0.03	0.03	0.01	0.01	0.01	1.39 [1.18-1.64]*	1.28 [1.08-1.51]*
S60	11,290	50,972,580	0.26	0.21	0.22	0.05	0.04	0.03	1.20 [1.12-1.28]*	1.11 [1.04-1.19]*
S61	31,840	50,464,500	0.71	0.61	0.63	0.10	0.08	0.04	1.14 [1.09-1.18]*	1.06 [1.02-1.11]*
S62	21,090	50,650,250	0.46	0.41	0.42	0.05	0.04	0.01	1.09 [1.04-1.15]*	1.03 [0.98-1.08]
S63	4,110	50,979,690	0.09	0.08	0.08	0.02	0.02	0.01	1.21 [1.09-1.34]*	1.14 [1.02-1.27]
S64	3,710	50,939,860	0.07	0.07	0.07	0.00	0.00	0.00	1.01 [0.91-1.14]	0.98 [0.87-1.10]
S65	1,120	51,030,690	0.02	0.02	0.02	0.00	0.00	0.00	0.99 [0.80-1.22]	0.96 [0.78-1.19]
S66	6,880	50,830,300	0.14	0.14	0.13	0.00	0.00	0.00	1.00 [0.92-1.09]	0.97 [0.89-1.05]
S68	2,540	50,992,280	0.05	0.05	0.05	0.00	0.00	0.00	0.97 [0.85-1.12]	0.99 [0.86-1.14]
S69	3,950	51,013,120	0.09	0.07	0.07	0.02	0.02	0.01	1.29 [1.15-1.44]*	1.15 [1.02-1.28]
S70	15,470	50,965,780	0.36	0.29	0.31	0.07	0.06	0.04	1.19 [1.13-1.26]*	1.11 [1.05-1.18]*
S71	1,480	51,036,390	0.04	0.03	0.03	0.01	0.01	0.01	1.30 [1.09-1.55]*	1.20 [1.00-1.44]
S72	121,160	50,172,280	2.77	2.34	2.48	0.43	0.29	0.22	1.12 [1.10-1.14]*	1.08 [1.06-1.11]*
S73	1,260	51,044,420	0.03	0.02	0.02	0.00	0.00	0.00	1.17 [0.96-1.42]	1.07 [0.87-1.31]
S76	3,010	51,022,170	0.06	0.06	0.06	0.01	0.01	0.00	1.14 [1.01-1.30]	1.07 [0.94-1.22]
S79	8,580	51,000,920	0.21	0.16	0.17	0.05	0.04	0.03	1.25 [1.15-1.34]*	1.16 [1.07-1.25]*
S80	29,510	50,864,090	0.69	0.56	0.58	0.13	0.11	0.05	1.19 [1.14-1.24]*	1.07 [1.03-1.12]*
S81	21,220	50,896,270	0.52	0.40	0.40	0.12	0.12	0.07	1.29 [1.23-1.36]*	1.16 [1.10-1.22]*
S82	49,160	50,050,160	1.08	0.96	0.98	0.12	0.10	0.04	1.10 [1.07-1.14]*	1.04 [1.01-1.07]
S83	4,560	50,935,820	0.10	0.09	0.09	0.02	0.02	0.01	1.18 [1.06-1.30]*	1.07 [0.97-1.19]
S86	3,380	50,956,260	0.08	0.06	0.06	0.01	0.01	0.01	1.20 [1.07-1.34]*	1.11 [0.98-1.24]
S89	6,070	51,001,030	0.15	0.11	0.12	0.04	0.03	0.02	1.27 [1.16-1.38]*	1.15 [1.05-1.26]*
S90	6,700	51,007,120	0.15	0.13	0.13	0.02	0.02	0.00	1.11 [1.02-1.21]	1.01 [0.92-1.10]
S91	4,790	50,998,550	0.12	0.09	0.09	0.03	0.03	0.02	1.30 [1.17-1.43]*	1.16 [1.05-1.29]*
S92	10,420	50,873,910	0.24	0.20	0.20	0.05	0.04	0.02	1.20 [1.12-1.28]*	1.09 [1.02-1.17]
S93	3,350	51,007,730	0.08	0.06	0.06	0.01	0.01	0.01	1.21 [1.07-1.36]*	1.08 [0.96-1.22]
S96	530	51,049,950	0.01	0.01	0.01	0.00	0.00	0.00	1.19 [0.89-1.59]	1.00 [0.73-1.36]
S99	2,730	51,031,700	0.06	0.05	0.05	0.01	0.01	0.00	1.16 [1.01-1.32]	1.02 [0.89-1.17]
T00	6,030	51,030,220	0.14	0.11	0.12	0.03	0.02	0.01	1.19 [1.08-1.31]*	1.09 [0.99-1.20]
T01	2,540	51,045,610	0.06	0.05	0.05	0.01	0.01	0.00	1.18 [1.02-1.36]	1.05 [0.90-1.21]
T02	1,800	51,041,080	0.04	0.03	0.03	0.01	0.01	0.01	1.31 [1.12-1.54]*	1.26 [1.07-1.48]*
T09	950	51,049,120	0.02	0.02	0.02	0.00	0.00	0.00	1.22 [0.97-1.52]	1.10 [0.87-1.38]
T11	4,190	51,035,990	0.10	0.08	0.08	0.02	0.02	0.01	1.28 [1.14-1.43]*	1.15 [1.03-1.29]
T13	2,790	51,036,580	0.07	0.05	0.05	0.01	0.01	0.01	1.26 [1.10-1.44]*	1.13 [0.98-1.30]
T14	2,410	50,999,610	0.06	0.05	0.05	0.01	0.01	0.00	1.23 [1.07-1.41]*	1.10 [0.95-1.26]
T16	460	51,054,930	0.01	0.01	0.01	0.00	0.00	0.00	1.34 [0.98-1.83]	1.16 [0.83-1.60]
T17	5,800	50,998,940	0.14	0.11	0.11	0.04	0.03	0.02	1.30 [1.19-1.43]*	1.18 [1.08-1.30]*
T18	5,010	50,980,710	0.12	0.09	0.09	0.02	0.02	0.01	1.26 [1.15-1.39]*	1.13 [1.03-1.25]
T20	1,270	51,037,870	0.03	0.02	0.02	0.01	0.01	0.00	1.28 [1.06-1.54]	1.20 [0.99-1.46]
T21	2,270	51,033,170	0.05	0.04	0.04	0.01	0.01	0.01	1.20 [1.04-1.39]	1.11 [0.96-1.29]
T22	1,700	51,032,910	0.04	0.03	0.03	0.00	0.00	0.00	1.13 [0.96-1.34]	1.06 [0.89-1.26]
T23	1,530	51,029,930	0.03	0.03	0.03	0.00	0.00	0.00	1.09 [0.92-1.31]	1.02 [0.85-1.22]
T24	2,090	51,034,370	0.04	0.04	0.04	0.00	0.00	0.00	0.97 [0.82-1.13]	0.93 [0.79-1.10]
T25	800	51,050,430	0.02	0.02	0.02	0.00	0.00	0.00	0.92 [0.71-1.19]	0.81 [0.61-1.06]
T31	4,120	50,994,360	0.09	0.08	0.08	0.01	0.01	0.00	1.10 [0.99-1.23]	1.03 [0.93-1.16]
T36	920	51,033,520	0.02	0.02	0.02	0.01	0.01	0.01	1.50 [1.22-1.85]*	1.25 [1.01-1.56]
T37	460	51,050,610	0.01	0.01	0.01	0.00	0.00	0.00	1.55 [1.14-2.09]*	1.28 [0.93-1.76]
T38	2,880	51,014,870	0.07	0.05	0.05	0.02	0.02	0.01	1.37 [1.21-1.54]*	1.11 [0.98-1.26]
T39	16,440	50,481,170	0.40	0.31	0.31	0.08	0.08	0.03	1.26 [1.20-1.33]*	1.08 [1.02-1.14]*
T40	11,900	50,827,710	0.29	0.22	0.22	0.07	0.07	0.02	1.32 [1.24-1.40]*	1.06 [0.99-1.13]

T42	9,520	50,726,630	0.23	0.18	0.18	0.05	0.05	0.01	1.30 [1.21-1.39]*	1.06 [0.99-1.14]
T43	10,760	50,652,530	0.26	0.20	0.20	0.06	0.06	0.02	1.32 [1.24-1.40]*	1.08 [1.01-1.15]
T44	2,910	50,994,120	0.07	0.05	0.05	0.01	0.01	0.00	1.23 [1.09-1.40]*	1.02 [0.90-1.17]
T45	3,670	50,996,490	0.09	0.07	0.07	0.02	0.02	0.01	1.35 [1.21-1.51]*	1.14 [1.02-1.28]
T46	4,560	51,005,860	0.11	0.09	0.09	0.02	0.02	0.01	1.26 [1.13-1.39]*	1.06 [0.96-1.18]
T47	1,620	51,033,280	0.04	0.03	0.03	0.01	0.01	0.00	1.26 [1.07-1.49]*	0.99 [0.83-1.18]
T48	400	51,051,790	0.01	0.01	0.01	0.01	0.01	0.00	1.77 [1.30-2.40]*	1.44 [1.04-1.99]
T50	3,900	50,930,850	0.09	0.07	0.07	0.02	0.02	0.00	1.26 [1.13-1.40]*	1.03 [0.92-1.15]
T51	9,680	50,698,730	0.23	0.18	0.18	0.04	0.05	0.01	1.26 [1.18-1.35]*	1.03 [0.96-1.11]
T56	500	51,051,660	0.01	0.01	0.01	0.00	0.00	0.00	1.18 [0.88-1.59]	1.07 [0.79-1.45]
T59	1,360	51,034,740	0.03	0.03	0.03	0.00	0.00	0.00	1.13 [0.93-1.37]	1.03 [0.84-1.25]
T63	660	51,045,030	0.01	0.01	0.01	0.00	0.00	0.00	0.95 [0.72-1.25]	0.88 [0.66-1.16]
T65	460	51,053,480	0.01	0.01	0.01	0.00	0.00	0.00	1.29 [0.95-1.75]	1.10 [0.80-1.52]
T68	8,900	51,032,870	0.20	0.17	0.18	0.03	0.02	0.02	1.11 [1.03-1.20]*	1.11 [1.02-1.20]
T78	9,310	50,863,760	0.28	0.16	0.16	0.12	0.12	0.09	1.72 [1.61-1.83]*	1.45 [1.36-1.55]*
T79	9,850	50,957,870	0.22	0.19	0.19	0.03	0.02	0.01	1.12 [1.04-1.21]*	1.07 [0.99-1.15]
T80	4,300	51,011,140	0.11	0.08	0.08	0.03	0.03	0.02	1.38 [1.25-1.52]*	1.26 [1.14-1.40]*
T81	137,430	48,761,730	3.46	2.68	2.69	0.79	0.77	0.53	1.29 [1.26-1.31]*	1.18 [1.16-1.20]*
T82	36,330	50,654,740	0.92	0.67	0.68	0.24	0.23	0.16	1.34 [1.30-1.39]*	1.21 [1.17-1.25]*
T83	37,600	50,796,320	0.84	0.72	0.74	0.12	0.10	0.06	1.14 [1.09-1.18]*	1.08 [1.04-1.12]*
T84	47,800	50,248,290	1.20	0.90	0.90	0.30	0.30	0.22	1.34 [1.30-1.38]*	1.23 [1.19-1.27]*
T85	28,850	50,712,970	0.71	0.54	0.54	0.17	0.17	0.12	1.31 [1.26-1.36]*	1.20 [1.16-1.25]*
T86	3,760	51,002,780	0.09	0.07	0.07	0.02	0.03	0.02	1.38 [1.24-1.53]*	1.25 [1.12-1.40]*
T87	2,860	51,024,850	0.07	0.05	0.05	0.02	0.02	0.01	1.28 [1.13-1.45]*	1.08 [0.95-1.23]
T88	8,270	50,896,500	0.22	0.15	0.15	0.07	0.07	0.05	1.46 [1.36-1.56]*	1.28 [1.19-1.38]*
T90	4,750	50,934,430	0.12	0.09	0.09	0.03	0.03	0.02	1.38 [1.26-1.52]*	1.22 [1.11-1.35]*
T91	3,100	50,994,840	0.07	0.06	0.06	0.02	0.02	0.01	1.28 [1.13-1.44]*	1.13 [0.99-1.27]
T92	4,720	50,927,970	0.11	0.09	0.09	0.02	0.02	0.01	1.24 [1.12-1.36]*	1.15 [1.04-1.27]*
T93	6,020	50,898,210	0.14	0.11	0.11	0.03	0.03	0.02	1.28 [1.18-1.39]*	1.16 [1.06-1.26]*
T94	670	51,049,040	0.02	0.01	0.01	0.00	0.00	0.00	1.11 [0.85-1.44]	0.99 [0.76-1.29]
T98	1,170	51,040,320	0.03	0.02	0.02	0.01	0.01	0.01	1.35 [1.12-1.63]*	1.23 [1.01-1.50]
40+ - U00-U89										
U07	46,240	51,019,040	1.15	0.85	0.90	0.29	0.25	0.15	1.28 [1.24-1.32]*	1.15 [1.11-1.19]*
U80	16,160	50,973,850	0.44	0.29	0.30	0.15	0.15	0.11	1.49 [1.41-1.57]*	1.34 [1.27-1.41]*
U81	980	51,057,570	0.02	0.02	0.02	0.00	0.00	0.00	1.11 [0.89-1.39]	1.04 [0.83-1.31]
U82	31,620	50,982,600	0.83	0.57	0.61	0.26	0.23	0.16	1.38 [1.32-1.43]*	1.24 [1.19-1.29]*
U83	19,020	51,017,570	0.50	0.35	0.37	0.15	0.13	0.09	1.35 [1.29-1.42]*	1.21 [1.15-1.28]*
U88	4,010	51,042,810	0.10	0.07	0.08	0.03	0.02	0.02	1.30 [1.17-1.45]*	1.19 [1.06-1.33]*
U89	4,450	51,039,670	0.11	0.08	0.09	0.03	0.03	0.02	1.35 [1.22-1.49]*	1.21 [1.09-1.34]*
40+ - V00-Y99										
V03	4,260	50,972,610	0.10	0.08	0.08	0.02	0.01	0.01	1.18 [1.06-1.31]*	1.14 [1.02-1.26]
V09	1,440	51,046,090	0.03	0.03	0.03	0.01	0.01	0.00	1.30 [1.08-1.56]*	1.13 [0.94-1.37]
V13	1,300	51,033,280	0.02	0.03	0.03	0.00	0.00	0.00	0.90 [0.74-1.11]	0.90 [0.73-1.11]
V18	5,000	50,963,010	0.10	0.10	0.10	0.00	0.00	0.00	1.05 [0.95-1.16]	1.04 [0.94-1.15]
V19	770	51,046,250	0.02	0.01	0.01	0.00	0.00	0.00	1.10 [0.86-1.41]	1.10 [0.85-1.42]
V23	1,310	51,014,060	0.02	0.03	0.02	0.00	0.00	0.00	0.95 [0.78-1.16]	0.91 [0.74-1.12]
V28	1,700	51,006,060	0.03	0.03	0.03	0.00	0.00	0.00	1.03 [0.87-1.23]	0.96 [0.81-1.15]
V43	4,800	50,927,570	0.11	0.09	0.09	0.02	0.01	0.01	1.16 [1.05-1.28]*	1.06 [0.96-1.18]
V47	1,730	51,021,580	0.04	0.03	0.03	0.01	0.01	0.01	1.26 [1.07-1.48]*	1.15 [0.98-1.36]
V48	3,280	51,016,860	0.08	0.06	0.06	0.01	0.01	0.01	1.21 [1.07-1.36]*	1.12 [0.99-1.26]
V49	1,840	51,004,350	0.04	0.03	0.03	0.01	0.01	0.00	1.21 [1.03-1.41]	1.12 [0.95-1.32]
V78	1,770	51,043,650	0.04	0.03	0.03	0.01	0.01	0.00	1.17 [0.99-1.38]	1.10 [0.92-1.30]
V79	550	51,054,350	0.01	0.01	0.01	0.00	0.00	0.00	1.01 [0.74-1.38]	0.93 [0.68-1.28]
V80	2,090	51,006,750	0.03	0.04	0.04	−0.01	−0.01	−0.01	0.76 [0.64-0.90]*	0.76 [0.64-0.91]*
V82	430	51,056,820	0.01	0.01	0.01	0.00	0.00	0.00	1.37 [0.99-1.90]	1.33 [0.95-1.85]
V89	650	51,020,080	0.02	0.01	0.01	0.00	0.00	0.00	1.39 [1.08-1.79]	1.23 [0.94-1.59]
W00	6,330	50,952,220	0.13	0.12	0.12	0.01	0.01	0.01	1.08 [0.99-1.18]	1.06 [0.97-1.16]
W01	134,320	49,695,770	3.16	2.60	2.72	0.55	0.44	0.28	1.16 [1.14-1.18]*	1.10 [1.08-1.12]*
W02	1,300	51,024,040	0.03	0.03	0.02	0.00	0.00	0.00	1.02 [0.84-1.24]	1.00 [0.82-1.22]
W03	1,990	51,009,050	0.04	0.04	0.04	0.00	0.00	0.00	1.03 [0.88-1.21]	0.97 [0.82-1.14]
W05	3,040	51,039,900	0.07	0.06	0.06	0.02	0.02	0.01	1.26 [1.12-1.43]*	1.14 [1.01-1.30]
W06	33,080	50,919,760	0.79	0.62	0.66	0.17	0.13	0.08	1.19 [1.14-1.24]*	1.11 [1.07-1.16]*
W07	17,700	50,953,100	0.42	0.33	0.35	0.09	0.07	0.05	1.21 [1.15-1.28]*	1.13 [1.07-1.20]*
W08	4,120	51,030,180	0.10	0.08	0.08	0.02	0.02	0.01	1.22 [1.10-1.37]*	1.13 [1.01-1.26]
W10	51,500	50,425,230	1.19	0.98	1.00	0.21	0.19	0.12	1.19 [1.16-1.23]*	1.11 [1.08-1.15]*
W11	8,440	50,882,950	0.17	0.16	0.17	0.01	0.00	0.00	1.02 [0.95-1.10]	0.98 [0.90-1.05]
W13	2,200	51,000,760	0.05	0.04	0.04	0.01	0.01	0.00	1.14 [0.98-1.31]	1.07 [0.92-1.24]
W17	5,460	50,956,560	0.12	0.10	0.10	0.02	0.02	0.01	1.19 [1.09-1.31]*	1.12 [1.02-1.23]
W18	91,330	50,489,480	2.19	1.73	1.80	0.46	0.39	0.26	1.22 [1.19-1.25]*	1.13 [1.11-1.16]*
W19	213,280	49,475,690	5.17	4.13	4.36	1.04	0.81	0.55	1.19 [1.17-1.20]*	1.12 [1.10-1.14]*
W20	2,950	50,997,110	0.06	0.06	0.06	0.01	0.01	0.00	1.13 [1.00-1.28]	1.04 [0.92-1.19]
W21	760	51,034,420	0.01	0.01	0.02	0.00	0.00	0.00	0.94 [0.73-1.22]	0.88 [0.68-1.15]
W22	15,230	50,865,230	0.36	0.29	0.30	0.07	0.06	0.03	1.20 [1.13-1.27]*	1.09 [1.03-1.16]*
W23	6,210	50,934,810	0.13	0.12	0.12	0.01	0.02	0.01	1.14 [1.04-1.25]*	1.10 [1.01-1.20]
W25	2,690	50,939,990	0.06	0.05	0.05	0.01	0.01	0.00	1.16 [1.02-1.32]	1.06 [0.93-1.22]
W26	3,720	50,971,280	0.08	0.07	0.07	0.01	0.01	0.00	1.10 [0.98-1.23]	1.02 [0.91-1.14]
W27	2,140	50,996,190	0.04	0.04	0.04	0.00	0.00	0.00	1.08 [0.93-1.25]	1.01 [0.87-1.18]
W29	3,030	50,997,270	0.06	0.06	0.06	0.00	0.00	0.00	0.99 [0.87-1.13]	0.96 [0.84-1.10]
W31	2,670	50,982,250	0.05	0.05	0.05	0.00	0.00	0.00	0.95 [0.82-1.09]	0.94 [0.82-1.08]
W44	6,640	50,952,150	0.16	0.12	0.12	0.04	0.04	0.02	1.30 [1.19-1.41]*	1.18 [1.08-1.28]*
W45	2,840	50,978,850	0.06	0.06	0.05	0.00	0.00	0.00	1.08 [0.95-1.23]	0.99 [0.87-1.13]
W46	510	51,058,690	0.01	0.01	0.01	0.00	0.00	0.00	1.32 [0.99-1.75]	1.05 [0.78-1.41]
W49	830	51,036,320	0.02	0.02	0.02	0.00	0.00	0.00	1.32 [1.05-1.66]	1.25 [0.99-1.58]
W50	1,510	51,008,220	0.03	0.03	0.03	0.01	0.01	0.00	1.23 [1.03-1.45]	1.11 [0.93-1.33]
W51	1,100	51,024,990	0.02	0.02	0.02	0.00	0.00	0.00	1.10 [0.89-1.35]	1.06 [0.85-1.31]
W54	5,280	50,977,320	0.12	0.10	0.10	0.01	0.01	0.00	1.13 [1.03-1.24]*	1.04 [0.95-1.15]
W55	3,450	51,008,430	0.07	0.07	0.07	0.00	0.00	−0.01	0.97 [0.86-1.09]	0.88 [0.78-1.00]
W57	3,400	51,000,210	0.09	0.06	0.06	0.03	0.03	0.02	1.42 [1.27-1.58]*	1.28 [1.14-1.43]*
W60	410	51,055,620	0.01	0.01	0.01	0.00	0.00	0.00	1.46 [1.07-1.99]	1.38 [1.01-1.89]
W64	900	51,054,220	0.02	0.02	0.02	0.00	0.00	0.00	1.13 [0.90-1.43]	1.06 [0.84-1.35]
W78	3,490	51,054,280	0.08	0.07	0.07	0.01	0.01	0.00	1.10 [0.97-1.25]	1.05 [0.92-1.19]
W79	4,770	51,038,320	0.11	0.09	0.09	0.02	0.02	0.01	1.19 [1.08-1.32]*	1.13 [1.02-1.25]
W80	1,960	51,057,350	0.05	0.04	0.04	0.01	0.01	0.01	1.27 [1.08-1.50]*	1.15 [0.98-1.36]
W84	2,070	51,057,600	0.05	0.04	0.04	0.01	0.00	0.00	1.08 [0.92-1.27]	1.03 [0.87-1.21]
X09	520	51,052,100	0.01	0.01	0.01	0.00	0.00	0.00	1.11 [0.82-1.51]	1.05 [0.77-1.43]
X10	940	51,048,790	0.02	0.02	0.02	0.00	0.00	0.00	1.19 [0.95-1.50]	1.10 [0.88-1.39]
X12	810	51,051,350	0.02	0.02	0.02	0.00	0.00	0.00	1.09 [0.86-1.40]	0.99 [0.77-1.28]
X16	800	51,055,590	0.02	0.02	0.02	0.00	0.00	0.00	1.04 [0.80-1.34]	0.98 [0.75-1.27]
X23	860	51,045,630	0.02	0.02	0.02	0.00	0.00	0.00	1.13 [0.89-1.42]	1.04 [0.82-1.31]
X31	1,230	51,056,720	0.02	0.02	0.02	0.00	0.00	0.00	1.00 [0.80-1.23]	0.99 [0.80-1.24]
X40	4,560	50,955,290	0.11	0.09	0.09	0.02	0.02	0.01	1.26 [1.14-1.39]*	1.07 [0.96-1.18]
X41	3,890	50,955,540	0.09	0.07	0.07	0.02	0.02	0.01	1.29 [1.16-1.43]*	1.08 [0.96-1.20]
X42	4,580	51,009,200	0.12	0.08	0.08	0.03	0.03	0.01	1.38 [1.25-1.52]*	1.11 [1.00-1.23]
X43	1,060	51,049,480	0.02	0.02	0.02	0.00	0.00	0.00	1.16 [0.93-1.45]	1.03 [0.82-1.29]
X44	6,190	50,979,200	0.15	0.11	0.12	0.04	0.04	0.01	1.30 [1.19-1.42]*	1.11 [1.01-1.21]
X45	1,170	51,015,360	0.03	0.02	0.02	0.00	0.01	0.00	1.23 [1.02-1.50]	1.00 [0.81-1.22]
X47	760	51,048,690	0.02	0.01	0.01	0.00	0.00	0.00	1.17 [0.91-1.51]	1.12 [0.86-1.45]

X49	1,420	51,028,550	0.03	0.03	0.03	0.01	0.01	0.01	1.30 [1.09-1.55]*	1.17 [0.98-1.40]
X50	10,070	50,877,430	0.22	0.19	0.19	0.03	0.03	0.01	1.15 [1.08-1.24]*	1.06 [0.99-1.14]
X51	970	51,045,260	0.02	0.02	0.02	0.00	0.00	0.00	1.16 [0.93-1.45]	1.10 [0.88-1.38]
X58	3,130	51,028,680	0.08	0.06	0.06	0.02	0.02	0.02	1.39 [1.23-1.56]*	1.26 [1.11-1.42]*
X59	46,780	50,405,730	1.14	0.88	0.90	0.26	0.24	0.16	1.26 [1.22-1.30]*	1.16 [1.12-1.20]*
X60	12,520	50,581,340	0.30	0.24	0.24	0.07	0.07	0.03	1.29 [1.21-1.36]*	1.09 [1.03-1.16]*
X61	12,390	50,572,330	0.30	0.23	0.23	0.07	0.07	0.02	1.32 [1.25-1.40]*	1.08 [1.02-1.15]
X62	6,950	50,886,530	0.17	0.13	0.13	0.04	0.04	0.00	1.29 [1.20-1.40]*	1.02 [0.94-1.11]
X63	1,750	51,010,940	0.04	0.03	0.03	0.01	0.01	0.00	1.26 [1.08-1.48]*	1.01 [0.85-1.19]
X64	5,800	50,866,930	0.15	0.11	0.11	0.04	0.04	0.01	1.37 [1.26-1.48]*	1.10 [1.01-1.20]
X65	8,940	50,748,800	0.21	0.17	0.17	0.04	0.05	0.01	1.28 [1.20-1.37]*	1.04 [0.97-1.12]
X78	2,530	50,992,350	0.06	0.05	0.05	0.01	0.01	0.00	1.30 [1.14-1.48]*	1.05 [0.91-1.20]
X99	850	51,013,000	0.02	0.02	0.02	0.00	0.00	0.00	1.17 [0.93-1.47]	1.03 [0.81-1.31]
Y00	800	51,023,090	0.02	0.02	0.01	0.00	0.00	0.00	1.25 [0.99-1.57]	1.06 [0.83-1.35]
Y04	4,900	50,799,460	0.11	0.09	0.09	0.02	0.02	0.01	1.23 [1.12-1.35]*	1.06 [0.96-1.17]
Y09	900	51,008,440	0.02	0.02	0.02	0.00	0.00	0.00	1.27 [1.02-1.58]	1.09 [0.87-1.37]
Y40	15,490	50,903,870	0.43	0.28	0.28	0.16	0.15	0.11	1.53 [1.45-1.61]*	1.36 [1.29-1.43]*
Y41	3,230	51,025,320	0.09	0.06	0.06	0.03	0.03	0.02	1.45 [1.29-1.62]*	1.26 [1.12-1.43]*
Y42	13,520	50,929,340	0.35	0.25	0.25	0.11	0.10	0.05	1.40 [1.32-1.48]*	1.18 [1.11-1.25]*
Y43	28,560	50,875,230	0.66	0.54	0.54	0.12	0.11	0.09	1.21 [1.16-1.26]*	1.16 [1.11-1.21]*
Y44	10,240	50,980,380	0.25	0.19	0.20	0.05	0.05	0.03	1.24 [1.16-1.33]*	1.14 [1.06-1.22]*
Y45	22,060	50,850,030	0.56	0.41	0.42	0.15	0.14	0.09	1.34 [1.28-1.40]*	1.20 [1.14-1.25]*
Y46	3,100	51,027,090	0.08	0.06	0.06	0.02	0.02	0.01	1.35 [1.20-1.52]*	1.20 [1.07-1.36]*
Y47	1,450	51,047,640	0.04	0.03	0.03	0.01	0.01	0.01	1.40 [1.17-1.67]*	1.26 [1.05-1.51]
Y48	1,520	51,041,880	0.04	0.03	0.03	0.01	0.01	0.00	1.28 [1.08-1.52]*	1.13 [0.94-1.35]
Y49	5,320	51,010,810	0.13	0.10	0.10	0.04	0.03	0.03	1.35 [1.23-1.48]*	1.24 [1.13-1.36]*
Y51	9,110	50,980,270	0.21	0.17	0.17	0.04	0.04	0.03	1.24 [1.16-1.34]*	1.16 [1.08-1.25]*
Y52	22,210	50,894,680	0.55	0.41	0.42	0.14	0.13	0.09	1.30 [1.24-1.36]*	1.19 [1.13-1.24]*
Y53	3,970	51,036,550	0.10	0.07	0.08	0.03	0.03	0.02	1.37 [1.23-1.53]*	1.20 [1.07-1.34]*
Y54	17,330	50,961,890	0.45	0.32	0.33	0.13	0.12	0.08	1.36 [1.29-1.43]*	1.23 [1.17-1.30]*
Y55	960	51,049,740	0.03	0.02	0.02	0.01	0.01	0.01	1.55 [1.27-1.90]*	1.24 [1.00-1.53]
Y56	1,470	51,036,640	0.05	0.02	0.03	0.02	0.02	0.02	1.88 [1.60-2.21]*	1.61 [1.37-1.91]*
Y57	9,140	50,991,230	0.23	0.17	0.17	0.07	0.07	0.04	1.39 [1.29-1.49]*	1.22 [1.13-1.31]*
Y59	720	51,055,770	0.02	0.01	0.01	0.01	0.01	0.00	1.44 [1.13-1.83]*	1.29 [1.01-1.65]
Y60	11,130	50,963,400	0.27	0.21	0.21	0.06	0.05	0.04	1.25 [1.17-1.33]*	1.16 [1.09-1.24]*
Y65	1,420	51,046,800	0.03	0.03	0.03	0.01	0.01	0.00	1.21 [1.01-1.45]	1.10 [0.92-1.33]
Y71	690	51,053,850	0.02	0.01	0.01	0.01	0.01	0.01	1.56 [1.22-2.00]*	1.43 [1.11-1.84]*
Y73	650	51,054,920	0.01	0.01	0.01	0.00	0.00	0.00	0.99 [0.75-1.30]	0.90 [0.68-1.20]
Y77	850	51,054,290	0.02	0.02	0.02	0.00	0.00	0.00	0.99 [0.78-1.28]	0.95 [0.74-1.22]
Y79	1,620	51,035,910	0.04	0.03	0.03	0.01	0.01	0.00	1.19 [1.00-1.40]	1.11 [0.94-1.32]
Y82	510	51,054,200	0.01	0.01	0.01	0.00	0.00	0.00	1.31 [0.98-1.76]	1.19 [0.88-1.60]
Y83	184,430	48,241,050	4.68	3.64	3.67	1.04	1.00	0.66	1.27 [1.25-1.29]*	1.17 [1.15-1.18]*
Y84	46,100	50,704,500	1.10	0.87	0.89	0.23	0.21	0.14	1.24 [1.20-1.28]*	1.14 [1.11-1.18]*
Y85	3,540	50,977,960	0.09	0.07	0.06	0.02	0.02	0.02	1.38 [1.24-1.54]*	1.25 [1.12-1.40]*
Y86	6,050	50,903,380	0.14	0.11	0.11	0.03	0.03	0.02	1.24 [1.14-1.35]*	1.13 [1.03-1.23]*
Y87	610	51,039,800	0.02	0.01	0.01	0.00	0.00	0.00	1.39 [1.07-1.80]	1.23 [0.94-1.61]
Y88	1,490	51,037,240	0.03	0.03	0.03	0.01	0.01	0.00	1.24 [1.04-1.47]	1.06 [0.88-1.27]
Y90	640	51,053,610	0.01	0.01	0.01	0.00	0.00	0.00	1.19 [0.91-1.55]	1.05 [0.80-1.38]
Y91	1,270	51,030,540	0.03	0.02	0.02	0.01	0.01	0.00	1.27 [1.05-1.53]	1.10 [0.91-1.34]
Y95	115,490	50,790,640	2.82	2.16	2.26	0.66	0.55	0.33	1.24 [1.22-1.27]*	1.13 [1.11-1.16]*
40+ - Z00-Z99										
Z00	3,400	50,974,830	0.08	0.06	0.06	0.01	0.02	0.01	1.25 [1.11-1.40]*	1.15 [1.02-1.29]
Z01	9,340	50,494,740	0.24	0.17	0.17	0.07	0.07	0.05	1.43 [1.34-1.53]*	1.26 [1.18-1.35]*
Z02	14,350	50,920,290	0.32	0.27	0.27	0.05	0.05	0.02	1.16 [1.10-1.24]*	1.08 [1.01-1.14]
Z03	108,930	49,056,990	2.81	2.09	2.12	0.72	0.70	0.45	1.33 [1.30-1.36]*	1.19 [1.17-1.22]*
Z04	13,000	50,589,870	0.32	0.24	0.24	0.08	0.08	0.06	1.32 [1.24-1.40]*	1.22 [1.15-1.29]*
Z08	42,720	50,289,960	0.91	0.84	0.84	0.07	0.07	0.04	1.08 [1.05-1.12]*	1.05 [1.02-1.09]*
Z09	70,680	49,668,620	1.74	1.35	1.35	0.39	0.39	0.25	1.29 [1.26-1.32]*	1.17 [1.14-1.20]*
Z11	184,560	50,698,810	4.42	3.47	3.61	0.95	0.82	0.42	1.23 [1.21-1.25]*	1.10 [1.09-1.12]*
Z12	60,680	50,457,040	1.35	1.17	1.17	0.18	0.17	0.14	1.15 [1.12-1.18]*	1.11 [1.08-1.15]*
Z13	114,180	49,557,230	2.82	2.19	2.23	0.63	0.60	0.38	1.27 [1.24-1.29]*	1.15 [1.13-1.18]*
Z20	6,930	51,041,130	0.17	0.13	0.14	0.04	0.03	0.02	1.25 [1.15-1.36]*	1.13 [1.04-1.24]*
Z22	45,470	50,602,950	1.24	0.83	0.84	0.42	0.40	0.31	1.48 [1.43-1.53]*	1.34 [1.30-1.38]*
Z29	27,530	50,822,190	0.67	0.51	0.52	0.16	0.15	0.10	1.29 [1.23-1.34]*	1.17 [1.12-1.22]*
Z30	16,350	48,995,000	0.39	0.32	0.32	0.07	0.06	0.04	1.20 [1.14-1.26]*	1.11 [1.05-1.17]*
Z33	1,370	50,579,860	0.03	0.03	0.03	0.01	0.01	0.00	1.19 [1.00-1.42]	1.08 [0.90-1.29]
Z34	3,070	49,929,150	0.06	0.06	0.06	0.00	0.00	0.00	0.97 [0.85-1.09]	0.96 [0.84-1.09]
Z35	9,440	50,136,550	0.19	0.19	0.20	0.00	−0.01	−0.01	0.96 [0.89-1.03]	0.97 [0.90-1.05]
Z36	940	50,378,890	0.02	0.02	0.02	0.00	0.00	0.00	1.10 [0.89-1.36]	1.13 [0.91-1.42]
Z37	7,950	44,231,080	0.27	0.16	0.26	0.11	0.02	0.03	1.06 [0.99-1.13]	1.12 [1.04-1.20]*
Z39	1,310	50,519,870	0.03	0.03	0.03	0.00	0.00	0.00	1.03 [0.86-1.24]	1.06 [0.88-1.29]
Z40	4,140	50,990,660	0.09	0.08	0.08	0.01	0.01	0.01	1.11 [1.00-1.24]	1.06 [0.96-1.19]
Z41	2,520	50,959,890	0.06	0.05	0.05	0.02	0.02	0.01	1.34 [1.18-1.52]*	1.23 [1.08-1.40]*
Z42	11,660	50,812,640	0.26	0.22	0.22	0.03	0.03	0.02	1.15 [1.09-1.23]*	1.10 [1.03-1.17]*
Z43	17,470	50,767,660	0.39	0.33	0.33	0.06	0.06	0.04	1.18 [1.12-1.24]*	1.12 [1.07-1.19]*
Z45	38,570	50,608,490	0.89	0.73	0.74	0.16	0.15	0.09	1.21 [1.16-1.25]*	1.11 [1.07-1.15]*
Z46	50,490	50,291,920	1.12	0.98	0.98	0.14	0.14	0.09	1.14 [1.10-1.17]*	1.08 [1.05-1.12]*
Z47	16,710	50,452,450	0.38	0.32	0.32	0.06	0.07	0.04	1.21 [1.15-1.28]*	1.13 [1.07-1.19]*
Z48	10,030	50,797,130	0.23	0.19	0.19	0.04	0.04	0.03	1.22 [1.15-1.31]*	1.15 [1.08-1.23]*
Z49	7,880	50,970,560	0.21	0.14	0.14	0.07	0.06	0.05	1.44 [1.34-1.55]*	1.30 [1.21-1.40]*
Z50	305,560	49,223,110	7.39	5.95	6.22	1.44	1.17	0.73	1.19 [1.17-1.20]*	1.11 [1.09-1.12]*
Z51	347,970	48,474,320	8.46	6.90	7.05	1.56	1.41	0.93	1.20 [1.19-1.21]*	1.12 [1.11-1.14]*
Z52	1,370	51,028,040	0.03	0.03	0.03	0.00	0.00	0.00	0.97 [0.80-1.18]	0.99 [0.82-1.20]
Z53	255,900	46,463,760	6.87	5.21	5.24	1.66	1.63	1.09	1.31 [1.29-1.33]*	1.19 [1.17-1.20]*
Z54	2,850	50,995,890	0.07	0.05	0.05	0.02	0.02	0.02	1.40 [1.24-1.59]*	1.33 [1.17-1.51]*
Z56	2,280	51,023,250	0.05	0.04	0.04	0.01	0.01	0.00	1.14 [0.99-1.32]	0.99 [0.85-1.14]
Z57	11,760	50,992,300	0.28	0.22	0.22	0.06	0.06	0.04	1.27 [1.19-1.35]*	1.17 [1.10-1.25]*
Z58	5,770	51,033,050	0.14	0.11	0.11	0.03	0.03	0.01	1.25 [1.14-1.37]*	1.11 [1.01-1.22]
Z59	5,820	50,991,540	0.13	0.11	0.11	0.01	0.01	0.00	1.11 [1.01-1.21]	1.00 [0.91-1.10]
Z60	101,090	49,981,860	2.35	1.95	1.99	0.40	0.36	0.23	1.18 [1.16-1.21]*	1.11 [1.08-1.13]*
Z61	830	51,048,640	0.02	0.02	0.02	0.01	0.00	0.00	1.31 [1.05-1.64]	1.05 [0.83-1.32]
Z63	17,920	50,867,620	0.42	0.34	0.34	0.09	0.08	0.03	1.23 [1.17-1.30]*	1.08 [1.02-1.14]*
Z65	970	51,044,870	0.02	0.02	0.02	0.00	0.00	0.00	1.18 [0.95-1.46]	1.00 [0.80-1.25]
Z71	22,080	50,826,660	0.51	0.42	0.43	0.09	0.08	0.04	1.18 [1.12-1.23]*	1.09 [1.04-1.15]*
Z72	151,770	46,608,240	3.79	3.14	3.10	0.65	0.69	0.40	1.22 [1.20-1.24]*	1.12 [1.10-1.14]*
Z73	51,700	50,830,510	1.21	0.98	1.02	0.23	0.19	0.11	1.19 [1.15-1.22]*	1.10 [1.07-1.14]*
Z74	41,560	50,823,900	0.95	0.79	0.82	0.17	0.14	0.10	1.17 [1.13-1.21]*	1.11 [1.08-1.15]*
Z75	80,540	50,705,850	1.83	1.54	1.63	0.30	0.20	0.13	1.12 [1.09-1.15]*	1.07 [1.05-1.10]*
Z76	1,600	50,982,820	0.04	0.03	0.03	0.01	0.01	0.01	1.43 [1.21-1.69]*	1.36 [1.15-1.61]*
Z80	76,350	49,921,660	1.72	1.49	1.50	0.23	0.22	0.12	1.15 [1.12-1.18]*	1.08 [1.05-1.10]*
Z81	2,460	51,024,600	0.06	0.05	0.04	0.01	0.01	0.01	1.33 [1.16-1.51]*	1.14 [1.00-1.31]
Z82	129,130	48,875,960	3.13	2.54	2.53	0.59	0.59	0.32	1.23 [1.21-1.26]*	1.11 [1.09-1.14]*
Z83	30,150	50,623,530	0.70	0.57	0.58	0.13	0.12	0.07	1.20 [1.16-1.25]*	1.11 [1.07-1.15]*
Z84	1,860	51,021,690	0.05	0.03	0.03	0.01	0.01	0.01	1.41 [1.22-1.64]*	1.30 [1.12-1.52]*
Z85	308,340	47,696,830	7.49	6.24	6.41	1.25	1.08	0.70	1.17 [1.15-1.18]*	1.10 [1.09-1.12]*
Z86	821,250	42,469,790	23.96	18.33	18.79	5.63	5.17	3.25	1.28 [1.27-1.29]*	1.16 [1.15-1.17]*
Z87	371,500	46,470,050	9.99	7.56	7.74	2.43	2.25	1.36	1.29 [1.28-1.31]*	1.16 [1.14-1.17]*

Z88	392,750	45,656,180	11.78	7.91	8.09	3.87	3.70	2.56	1.46 [1.44-1.47]*	1.28 [1.26-1.29]*
Z89	14,220	50,938,610	0.35	0.26	0.27	0.08	0.08	0.05	1.29 [1.22-1.36]*	1.15 [1.09-1.22]*
Z90	246,980	48,797,600	6.03	4.85	4.98	1.18	1.05	0.57	1.21 [1.20-1.23]*	1.10 [1.09-1.12]*
Z91	120,030	49,676,090	3.47	2.19	2.23	1.28	1.24	0.86	1.56 [1.53-1.59]*	1.33 [1.30-1.36]*
Z92	617,510	45,851,230	16.36	12.85	13.28	3.51	3.07	1.78	1.23 [1.22-1.24]*	1.12 [1.11-1.13]*
Z93	57,190	50,568,990	1.31	1.09	1.11	0.22	0.21	0.15	1.19 [1.16-1.22]*	1.13 [1.09-1.16]*
Z94	9,560	50,901,700	0.24	0.18	0.17	0.07	0.07	0.06	1.40 [1.31-1.50]*	1.30 [1.21-1.39]*
Z95	206,250	48,662,020	5.12	4.05	4.15	1.07	0.96	0.55	1.23 [1.21-1.25]*	1.12 [1.10-1.14]*
Z96	400,100	47,405,490	10.20	8.06	8.38	2.14	1.82	1.25	1.22 [1.20-1.23]*	1.14 [1.13-1.15]*
Z97	31,530	50,786,970	0.77	0.59	0.61	0.18	0.16	0.11	1.26 [1.22-1.31]*	1.16 [1.12-1.21]*
Z98	46,690	50,543,240	1.14	0.88	0.89	0.26	0.25	0.14	1.28 [1.25-1.32]*	1.14 [1.10-1.17]*
Z99	61,140	50,651,900	1.62	1.12	1.15	0.51	0.48	0.30	1.41 [1.38-1.45]*	1.23 [1.19-1.26]*
<18 - A00-B99										
A02	640	46,396,080	0.02	0.01	0.01	0.00	0.00	0.00	1.25 [0.97-1.61]	1.26 [0.97-1.63]
A04	3,620	46,359,160	0.08	0.08	0.08	0.01	0.01	0.00	1.08 [0.97-1.21]	1.03 [0.91-1.15]
A07	380	46,408,660	0.01	0.01	0.01	0.00	0.00	0.00	1.52 [1.10-2.10]	1.44 [1.03-2.00]
A08	40,120	44,886,310	0.98	0.87	0.86	0.11	0.12	0.10	1.14 [1.10-1.17]*	1.11 [1.08-1.15]*
A09	43,710	45,776,210	1.09	0.92	0.92	0.17	0.17	0.13	1.19 [1.15-1.22]*	1.14 [1.10-1.17]*
A38	1,980	46,393,700	0.05	0.04	0.04	0.00	0.00	0.00	1.05 [0.91-1.22]	1.03 [0.89-1.20]
A39	1,910	46,334,260	0.04	0.04	0.04	0.00	0.00	0.00	1.05 [0.91-1.22]	1.04 [0.89-1.21]
A40	1,090	46,388,560	0.02	0.02	0.02	0.00	0.00	0.00	1.11 [0.90-1.35]	1.05 [0.86-1.30]
A41	10,020	46,237,800	0.23	0.21	0.21	0.02	0.02	0.02	1.10 [1.03-1.18]*	1.08 [1.01-1.16]
A49	3,100	46,336,740	0.07	0.06	0.06	0.01	0.01	0.01	1.19 [1.06-1.34]*	1.17 [1.04-1.32]
A87	1,420	46,372,210	0.03	0.03	0.03	0.00	0.01	0.01	1.27 [1.06-1.51]*	1.24 [1.03-1.49]
B00	5,190	46,273,200	0.24	0.08	0.08	0.16	0.16	0.16	3.13 [2.90-3.38]*	2.97 [2.74-3.22]*
B01	8,250	46,179,670	0.25	0.16	0.15	0.09	0.10	0.09	1.63 [1.53-1.74]*	1.58 [1.48-1.69]*
B02	1,410	46,399,680	0.04	0.03	0.03	0.01	0.01	0.01	1.41 [1.20-1.67]*	1.35 [1.13-1.60]*
B07	1,380	46,395,490	0.04	0.03	0.03	0.01	0.01	0.01	1.45 [1.22-1.71]*	1.32 [1.11-1.58]*
B08	2,920	46,352,060	0.08	0.06	0.05	0.03	0.03	0.03	1.53 [1.37-1.71]*	1.48 [1.32-1.66]*
B09	1,340	46,371,370	0.03	0.03	0.03	0.01	0.00	0.00	1.16 [0.97-1.38]	1.14 [0.95-1.36]
B27	5,600	46,358,420	0.13	0.12	0.12	0.02	0.02	0.01	1.16 [1.06-1.26]*	1.10 [1.01-1.21]
B34	114,870	43,223,860	3.72	2.37	2.36	1.35	1.36	1.28	1.57 [1.55-1.60]*	1.53 [1.50-1.55]*
B35	860	46,403,080	0.03	0.02	0.02	0.01	0.01	0.01	1.47 [1.19-1.82]*	1.38 [1.10-1.72]*
B37	9,010	46,192,150	0.20	0.19	0.18	0.01	0.02	0.01	1.11 [1.04-1.20]*	1.06 [0.99-1.14]
B80	1,640	46,396,700	0.04	0.03	0.03	0.01	0.01	0.00	1.18 [1.00-1.38]	1.12 [0.95-1.32]
B85	570	46,406,160	0.01	0.01	0.01	0.00	0.00	0.00	0.95 [0.71-1.26]	0.95 [0.71-1.26]
B94	570	46,409,480	0.01	0.01	0.01	0.00	0.00	0.00	1.23 [0.93-1.61]	1.25 [0.94-1.65]
B95	19,300	45,943,210	0.56	0.38	0.37	0.17	0.19	0.17	1.50 [1.43-1.57]*	1.45 [1.39-1.52]*
B96	22,970	45,843,080	0.52	0.50	0.49	0.02	0.03	0.02	1.07 [1.02-1.12]*	1.04 [1.00-1.09]
B97	24,830	45,750,600	0.67	0.51	0.50	0.16	0.17	0.15	1.34 [1.29-1.39]*	1.28 [1.23-1.34]*
B98	1,120	46,411,550	0.03	0.02	0.02	0.00	0.00	0.00	1.01 [0.82-1.25]	1.00 [0.80-1.24]
B99	570	46,409,480	0.01	0.01	0.01	0.00	0.00	0.00	1.25 [0.95-1.63]	1.18 [0.89-1.56]
<18 - C00-D49										
C71	1,170	46,403,980	0.02	0.03	0.02	0.00	0.00	0.00	0.99 [0.81-1.21]	1.02 [0.83-1.24]
C77	640	46,414,630	0.01	0.01	0.01	0.00	0.00	0.00	1.14 [0.86-1.51]	1.14 [0.86-1.53]
C78	710	46,414,060	0.01	0.02	0.01	0.00	0.00	0.00	1.01 [0.78-1.32]	1.03 [0.78-1.35]
C79	810	46,413,510	0.02	0.02	0.02	0.00	0.00	0.00	0.98 [0.77-1.25]	0.99 [0.77-1.27]
C81	720	46,410,670	0.02	0.01	0.01	0.00	0.01	0.00	1.41 [1.11-1.79]*	1.33 [1.04-1.71]
C91	1,520	46,392,600	0.03	0.03	0.03	0.00	0.00	0.00	0.97 [0.81-1.15]	0.97 [0.81-1.15]
C92	470	46,412,240	0.01	0.01	0.01	0.00	0.00	0.00	1.09 [0.81-1.49]	1.10 [0.80-1.51]
D06	1,880	46,404,400	0.03	0.04	0.03	−0.01	0.00	0.00	0.92 [0.77-1.10]	0.92 [0.77-1.11]
D10	800	46,407,280	0.02	0.02	0.02	0.00	0.00	0.00	1.14 [0.90-1.45]	1.13 [0.88-1.44]
D12	690	46,412,000	0.02	0.01	0.01	0.00	0.00	0.00	1.26 [0.96-1.64]	1.22 [0.93-1.61]
D16	2,610	46,386,870	0.06	0.06	0.06	0.01	0.00	0.00	1.07 [0.94-1.22]	1.06 [0.92-1.21]
D17	2,390	46,387,140	0.05	0.05	0.05	0.00	0.01	0.00	1.13 [0.98-1.30]	1.07 [0.93-1.23]
D18	3,470	46,275,080	0.08	0.07	0.07	0.01	0.01	0.01	1.14 [1.02-1.28]	1.13 [1.01-1.27]
D22	11,280	45,851,480	0.26	0.24	0.23	0.02	0.03	0.02	1.15 [1.08-1.22]*	1.11 [1.04-1.18]*
D23	6,100	46,295,700	0.14	0.13	0.12	0.01	0.02	0.01	1.14 [1.05-1.24]*	1.11 [1.02-1.21]
D24	3,050	46,390,290	0.07	0.07	0.06	0.00	0.01	0.01	1.13 [1.00-1.28]	1.12 [0.98-1.27]
D25	920	46,413,240	0.02	0.02	0.02	0.00	0.00	0.00	1.14 [0.89-1.44]	1.12 [0.87-1.44]
D27	1,690	46,405,030	0.04	0.04	0.03	0.00	0.00	0.00	1.07 [0.90-1.28]	1.04 [0.87-1.25]
D31	530	46,409,600	0.01	0.01	0.01	0.00	0.00	0.00	1.33 [1.01-1.74]	1.29 [0.98-1.70]
D36	1,010	46,403,900	0.02	0.02	0.02	0.00	0.00	0.00	1.04 [0.84-1.29]	0.99 [0.79-1.24]
D43	680	46,410,070	0.02	0.01	0.01	0.00	0.00	0.00	1.22 [0.95-1.57]	1.23 [0.95-1.59]
D47	390	46,414,010	0.01	0.01	0.01	0.00	0.00	0.00	1.36 [0.98-1.88]	1.30 [0.92-1.82]
D48	1,120	46,401,200	0.02	0.02	0.02	0.00	0.00	0.00	0.93 [0.75-1.15]	0.92 [0.74-1.15]
<18 - D50-D89										
D50	15,810	46,230,920	0.41	0.32	0.32	0.08	0.09	0.08	1.28 [1.21-1.35]*	1.24 [1.18-1.31]*
D51	570	46,414,090	0.01	0.01	0.01	0.00	0.00	0.00	1.43 [1.07-1.91]	1.33 [0.99-1.80]
D56	1,930	46,393,150	0.05	0.04	0.04	0.01	0.01	0.01	1.34 [1.16-1.56]*	1.31 [1.12-1.53]*
D57	3,800	46,347,970	0.11	0.08	0.08	0.03	0.03	0.03	1.36 [1.23-1.51]*	1.36 [1.22-1.51]*
D58	590	46,403,380	0.01	0.01	0.01	0.00	0.00	0.00	1.21 [0.92-1.59]	1.20 [0.90-1.58]
D59	530	46,406,910	0.01	0.01	0.01	0.00	0.00	0.00	1.27 [0.96-1.67]	1.25 [0.94-1.67]
D61	1,150	46,405,460	0.03	0.02	0.02	0.00	0.00	0.00	1.07 [0.87-1.30]	1.04 [0.84-1.27]
D62	1,070	46,409,860	0.02	0.02	0.02	0.00	0.00	0.00	0.99 [0.79-1.23]	0.98 [0.78-1.23]
D63	450	46,412,650	0.01	0.01	0.01	0.00	0.00	0.00	1.18 [0.86-1.62]	1.16 [0.83-1.60]
D64	14,860	46,229,200	0.35	0.31	0.30	0.04	0.05	0.04	1.17 [1.11-1.23]*	1.14 [1.08-1.21]*
D68	2,870	46,379,830	0.07	0.06	0.06	0.00	0.01	0.00	1.09 [0.96-1.23]	1.03 [0.90-1.17]
D69	12,100	46,220,060	0.28	0.26	0.26	0.02	0.02	0.02	1.06 [1.00-1.13]	1.06 [1.00-1.13]
D70	5,720	46,329,550	0.13	0.12	0.12	0.01	0.02	0.01	1.14 [1.04-1.24]*	1.11 [1.02-1.22]
D72	1,330	46,404,250	0.04	0.03	0.03	0.01	0.01	0.01	1.41 [1.18-1.68]*	1.31 [1.10-1.58]*
D73	810	46,410,060	0.02	0.02	0.02	0.00	0.00	0.00	0.92 [0.72-1.18]	0.95 [0.74-1.22]
D75	480	46,410,930	0.01	0.01	0.01	0.00	0.00	0.00	1.18 [0.86-1.62]	1.12 [0.81-1.55]
D80	780	46,403,570	0.02	0.02	0.02	0.01	0.01	0.01	1.47 [1.18-1.84]*	1.33 [1.06-1.68]
D84	580	46,409,000	0.02	0.01	0.01	0.01	0.01	0.01	1.76 [1.37-2.27]*	1.59 [1.22-2.06]*
D89	410	46,414,590	0.01	0.01	0.01	0.00	0.00	0.00	1.53 [1.13-2.08]*	1.37 [1.00-1.89]
<18 - E00-E89										
E03	7,130	46,337,800	0.16	0.15	0.14	0.01	0.03	0.03	1.20 [1.11-1.31]*	1.18 [1.09-1.29]*
E04	780	46,411,490	0.02	0.02	0.01	0.00	0.00	0.00	1.24 [0.98-1.58]	1.24 [0.97-1.59]
E05	1,810	46,404,840	0.04	0.04	0.03	0.00	0.01	0.01	1.28 [1.09-1.50]*	1.24 [1.05-1.46]
E10	10,960	46,265,590	0.24	0.24	0.24	0.00	−0.01	−0.01	0.97 [0.91-1.04]	0.96 [0.90-1.03]
E11	2,660	46,395,940	0.06	0.06	0.05	0.01	0.01	0.01	1.25 [1.09-1.43]*	1.23 [1.08-1.41]*
E14	1,180	46,404,520	0.02	0.03	0.03	0.00	0.00	0.00	0.92 [0.75-1.14]	0.92 [0.74-1.14]
E16	7,370	46,304,330	0.17	0.16	0.15	0.01	0.01	0.01	1.07 [0.99-1.15]	1.05 [0.97-1.14]
E22	750	46,410,010	0.02	0.02	0.01	0.00	0.01	0.00	1.35 [1.06-1.71]	1.28 [1.00-1.63]
E23	2,050	46,390,370	0.05	0.04	0.04	0.01	0.01	0.01	1.24 [1.07-1.43]*	1.18 [1.02-1.37]
E27	1,590	46,398,330	0.05	0.03	0.03	0.01	0.01	0.01	1.46 [1.24-1.71]*	1.32 [1.12-1.55]*
E28	10,770	46,351,270	0.24	0.23	0.21	0.00	0.03	0.02	1.13 [1.06-1.21]*	1.10 [1.03-1.18]*
E30	3,130	46,379,870	0.08	0.06	0.07	0.02	0.01	0.01	1.22 [1.09-1.37]*	1.17 [1.04-1.32]*
E34	4,560	46,359,100	0.12	0.09	0.10	0.02	0.02	0.01	1.19 [1.08-1.31]*	1.11 [1.01-1.22]
E46	530	46,412,930	0.01	0.01	0.01	0.00	0.00	0.00	1.29 [0.97-1.73]	1.25 [0.93-1.69]
E53	2,670	46,403,450	0.07	0.06	0.05	0.01	0.01	0.01	1.27 [1.11-1.45]*	1.19 [1.04-1.37]
E55	8,180	46,345,750	0.23	0.16	0.17	0.07	0.06	0.05	1.34 [1.25-1.45]*	1.26 [1.17-1.35]*
E61	1,810	46,403,550	0.05	0.04	0.04	0.02	0.01	0.01	1.35 [1.16-1.57]*	1.27 [1.09-1.48]*
E66	49,920	46,117,900	1.14	1.07	0.97	0.08	0.18	0.13	1.18 [1.14-1.22]*	1.13 [1.10-1.17]*
E73	2,380	46,359,090	0.07	0.05	0.05	0.02	0.02	0.01	1.36 [1.20-1.55]*	1.23 [1.08-1.41]*

E74	470	46,409,180	0.01	0.01	0.01	0.00	0.00	0.00	1.32 [0.98-1.78]	1.22 [0.89-1.66]
E78	1,280	46,407,770	0.03	0.03	0.02	0.00	0.00	0.00	1.18 [0.98-1.43]	1.16 [0.95-1.41]
E80	1,220	46,394,600	0.03	0.03	0.02	0.00	0.00	0.00	1.12 [0.92-1.36]	1.05 [0.86-1.29]
E83	3,900	46,360,830	0.09	0.08	0.08	0.01	0.01	0.01	1.08 [0.96-1.20]	1.06 [0.95-1.19]
E86	21,080	45,992,530	0.50	0.45	0.44	0.05	0.06	0.04	1.13 [1.08-1.18]*	1.10 [1.05-1.15]*
E87	12,040	46,201,390	0.28	0.26	0.26	0.02	0.02	0.02	1.09 [1.02-1.15]*	1.06 [1.00-1.13]
E88	1,770	46,393,580	0.04	0.04	0.04	0.00	0.01	0.00	1.16 [0.99-1.36]	1.12 [0.95-1.32]
E89	620	46,413,460	0.01	0.01	0.01	0.00	0.00	0.00	1.11 [0.84-1.47]	1.09 [0.81-1.47]
<18 - F01-F99										
F05	730	46,413,190	0.02	0.02	0.02	0.00	0.00	0.00	1.00 [0.77-1.30]	1.01 [0.78-1.32]
F10	18,800	46,218,030	0.37	0.42	0.37	−0.05	−0.01	−0.01	0.98 [0.93-1.04]	0.97 [0.92-1.02]
F11	1,080	46,407,880	0.02	0.02	0.02	0.00	0.00	0.00	1.00 [0.81-1.25]	1.07 [0.85-1.35]
F12	8,690	46,362,670	0.18	0.19	0.18	−0.01	−0.01	−0.01	0.96 [0.89-1.04]	0.97 [0.89-1.05]
F14	2,700	46,401,920	0.05	0.06	0.05	−0.01	0.00	0.00	1.02 [0.89-1.16]	1.03 [0.89-1.18]
F15	1,050	46,409,140	0.02	0.02	0.02	0.00	0.00	0.00	0.95 [0.76-1.18]	0.92 [0.73-1.16]
F17	107,180	45,702,090	2.17	2.39	2.19	−0.22	−0.02	0.00	0.99 [0.97-1.01]	1.00 [0.98-1.02]
F19	2,700	46,401,130	0.05	0.06	0.06	−0.01	0.00	0.00	0.93 [0.81-1.06]	0.96 [0.83-1.10]
F20	1,640	46,405,740	0.03	0.04	0.03	0.00	0.00	0.00	1.00 [0.84-1.20]	1.04 [0.86-1.25]
F22	540	46,414,470	0.01	0.01	0.01	0.00	0.00	0.00	1.00 [0.74-1.36]	0.99 [0.72-1.35]
F23	1,020	46,410,230	0.02	0.02	0.02	0.00	0.00	0.00	0.94 [0.75-1.17]	0.93 [0.74-1.17]
F29	1,900	46,405,370	0.04	0.04	0.04	0.00	0.00	0.00	0.91 [0.77-1.07]	0.92 [0.77-1.09]
F31	2,680	46,400,320	0.06	0.06	0.05	0.00	0.00	0.00	1.07 [0.93-1.23]	1.08 [0.93-1.24]
F32	49,230	46,109,190	1.12	1.05	0.99	0.07	0.13	0.13	1.14 [1.10-1.17]*	1.13 [1.09-1.16]*
F33	670	46,411,700	0.01	0.01	0.01	0.00	0.00	0.00	1.10 [0.84-1.44]	1.18 [0.89-1.56]
F40	9,680	46,347,870	0.23	0.20	0.20	0.03	0.03	0.02	1.13 [1.05-1.21]*	1.08 [1.01-1.16]
F41	54,020	46,147,660	1.29	1.14	1.11	0.15	0.18	0.15	1.16 [1.13-1.20]*	1.14 [1.10-1.17]*
F42	2,510	46,401,930	0.06	0.05	0.05	0.01	0.01	0.01	1.25 [1.09-1.43]*	1.15 [1.00-1.32]
F43	4,250	46,391,970	0.09	0.09	0.09	0.00	0.00	0.00	1.00 [0.90-1.12]	0.99 [0.89-1.11]
F44	1,130	46,410,550	0.03	0.02	0.02	0.00	0.01	0.00	1.27 [1.04-1.55]	1.19 [0.97-1.47]
F45	860	46,409,370	0.02	0.02	0.02	0.00	0.00	0.00	1.27 [1.02-1.60]	1.16 [0.91-1.46]
F50	4,950	46,380,030	0.12	0.10	0.11	0.02	0.01	0.01	1.13 [1.02-1.24]	1.09 [0.99-1.20]
F51	730	46,409,980	0.02	0.01	0.01	0.00	0.00	0.00	1.33 [1.05-1.68]	1.31 [1.03-1.67]
F53	710	46,413,260	0.01	0.02	0.01	0.00	0.00	0.00	0.96 [0.73-1.26]	0.95 [0.71-1.26]
F60	4,420	46,391,610	0.09	0.10	0.09	0.00	0.00	0.00	1.02 [0.92-1.14]	1.02 [0.91-1.14]
F70	790	46,411,970	0.02	0.02	0.02	0.00	0.00	0.00	1.19 [0.94-1.51]	1.14 [0.89-1.46]
F72	850	46,409,990	0.02	0.02	0.02	0.00	0.00	0.00	1.03 [0.82-1.29]	1.01 [0.80-1.29]
F79	4,870	46,371,880	0.12	0.10	0.11	0.01	0.01	0.00	1.06 [0.96-1.16]	1.03 [0.93-1.13]
F80	9,110	46,310,380	0.23	0.19	0.19	0.04	0.03	0.02	1.16 [1.09-1.24]*	1.11 [1.04-1.19]*
F81	15,820	46,230,150	0.39	0.33	0.34	0.06	0.05	0.03	1.13 [1.07-1.19]*	1.08 [1.03-1.14]*
F82	1,560	46,395,720	0.04	0.03	0.03	0.00	0.00	0.00	1.12 [0.95-1.33]	1.07 [0.90-1.26]
F83	1,490	46,402,370	0.03	0.03	0.03	0.00	0.00	0.00	0.91 [0.76-1.09]	0.88 [0.73-1.05]
F84	24,870	46,204,610	0.67	0.50	0.55	0.17	0.12	0.09	1.22 [1.17-1.27]*	1.15 [1.11-1.20]*
F89	7,040	46,361,330	0.16	0.15	0.16	0.01	0.00	−0.01	0.97 [0.90-1.05]	0.92 [0.85-1.00]
F90	13,340	46,306,380	0.35	0.27	0.30	0.08	0.05	0.04	1.16 [1.09-1.22]*	1.12 [1.06-1.19]*
F91	2,570	46,376,820	0.07	0.05	0.05	0.02	0.02	0.01	1.30 [1.15-1.48]*	1.24 [1.09-1.41]*
F94	550	46,414,050	0.01	0.01	0.01	0.00	0.00	0.00	1.19 [0.90-1.56]	1.14 [0.86-1.51]
F95	1,360	46,407,270	0.04	0.03	0.03	0.01	0.01	0.01	1.30 [1.10-1.55]*	1.22 [1.02-1.45]
F98	1,840	46,395,030	0.05	0.04	0.04	0.01	0.01	0.01	1.30 [1.12-1.51]*	1.25 [1.08-1.46]*
F99	1,360	46,407,120	0.03	0.03	0.03	0.00	0.00	0.00	1.10 [0.91-1.32]	1.09 [0.90-1.33]
<18 - G00-G99										
G00	550	46,375,780	0.01	0.01	0.01	0.00	0.00	0.00	0.92 [0.69-1.24]	0.90 [0.66-1.21]
G01	530	46,391,620	0.01	0.01	0.01	0.00	0.00	0.00	1.09 [0.82-1.45]	1.11 [0.83-1.49]
G03	820	46,379,080	0.02	0.02	0.02	0.00	0.01	0.00	1.32 [1.06-1.65]	1.27 [1.01-1.60]
G04	1,200	46,399,560	0.03	0.03	0.03	0.00	0.00	0.00	1.10 [0.91-1.33]	1.08 [0.89-1.31]
G24	1,340	46,402,880	0.03	0.03	0.03	0.00	−0.01	−0.01	0.82 [0.67-1.00]	0.79 [0.64-0.96]
G25	1,570	46,382,060	0.04	0.03	0.03	0.00	0.00	0.00	1.07 [0.91-1.27]	1.04 [0.87-1.24]
G31	580	46,408,680	0.01	0.01	0.01	0.00	0.00	0.00	1.04 [0.78-1.37]	1.01 [0.76-1.35]
G35	630	46,413,860	0.01	0.01	0.01	0.00	0.00	0.00	1.14 [0.85-1.53]	1.15 [0.85-1.55]
G37	540	46,412,650	0.01	0.01	0.01	0.00	0.00	0.00	1.04 [0.77-1.40]	1.02 [0.75-1.39]
G40	18,330	46,093,440	0.42	0.39	0.39	0.02	0.03	0.02	1.08 [1.02-1.13]*	1.06 [1.00-1.11]
G41	2,260	46,377,050	0.05	0.05	0.05	0.00	0.00	0.00	0.96 [0.84-1.11]	0.95 [0.83-1.10]
G43	16,520	46,290,750	0.40	0.35	0.33	0.05	0.07	0.05	1.20 [1.14-1.26]*	1.13 [1.07-1.20]*
G44	1,990	46,403,270	0.05	0.04	0.04	0.01	0.01	0.01	1.22 [1.05-1.42]	1.19 [1.02-1.39]
G47	33,010	46,023,030	0.91	0.66	0.69	0.24	0.22	0.18	1.31 [1.27-1.36]*	1.25 [1.21-1.30]*
G51	2,660	46,381,550	0.06	0.06	0.06	0.00	0.00	0.00	1.09 [0.95-1.24]	1.09 [0.95-1.24]
G55	2,210	46,405,110	0.05	0.05	0.04	0.00	0.01	0.00	1.12 [0.96-1.30]	1.06 [0.90-1.23]
G56	1,900	46,401,820	0.04	0.04	0.04	0.00	0.01	0.01	1.23 [1.06-1.44]*	1.16 [0.98-1.36]
G62	940	46,410,940	0.02	0.02	0.02	0.00	0.00	0.00	0.97 [0.78-1.22]	0.94 [0.74-1.19]
G71	950	46,403,570	0.02	0.02	0.02	0.00	0.00	0.00	1.00 [0.81-1.25]	1.01 [0.81-1.26]
G80	5,760	46,293,480	0.10	0.13	0.13	−0.03	−0.03	−0.03	0.79 [0.72-0.87]*	0.77 [0.70-0.85]*
G81	2,570	46,375,380	0.05	0.06	0.06	0.00	0.00	0.00	0.98 [0.86-1.13]	0.98 [0.85-1.12]
G82	1,170	46,399,780	0.02	0.03	0.03	−0.01	−0.01	−0.01	0.75 [0.61-0.93]*	0.73 [0.59-0.91]*
G83	820	46,406,260	0.02	0.02	0.02	0.00	0.00	0.00	0.97 [0.76-1.24]	0.95 [0.74-1.22]
G90	660	46,411,610	0.01	0.01	0.01	0.00	0.00	0.00	1.01 [0.77-1.33]	0.94 [0.71-1.25]
G91	1,820	46,368,200	0.04	0.04	0.04	0.00	0.00	0.00	0.97 [0.82-1.14]	0.97 [0.82-1.14]
G93	8,880	46,312,950	0.21	0.19	0.18	0.02	0.02	0.02	1.13 [1.05-1.22]*	1.08 [1.01-1.17]
G95	960	46,408,850	0.02	0.02	0.02	0.00	0.00	0.00	1.08 [0.87-1.35]	1.02 [0.82-1.28]
G96	690	46,409,180	0.01	0.02	0.01	0.00	0.00	0.00	1.03 [0.79-1.33]	0.97 [0.74-1.26]
G97	1,040	46,408,540	0.02	0.02	0.02	0.00	0.00	0.00	1.12 [0.91-1.39]	1.09 [0.88-1.35]
<18 - H00-H59										
H00	7,670	46,292,230	0.19	0.16	0.16	0.03	0.03	0.03	1.20 [1.11-1.29]*	1.17 [1.09-1.26]*
H01	1,020	46,399,880	0.03	0.02	0.02	0.01	0.01	0.01	1.49 [1.23-1.81]*	1.39 [1.14-1.70]*
H02	4,250	46,344,630	0.10	0.09	0.09	0.02	0.02	0.02	1.22 [1.10-1.35]*	1.18 [1.06-1.31]*
H03	360	46,411,020	0.01	0.01	0.01	0.01	0.01	0.00	1.74 [1.27-2.38]*	1.60 [1.16-2.21]*
H04	4,340	46,302,430	0.10	0.09	0.09	0.01	0.02	0.01	1.20 [1.09-1.32]*	1.15 [1.04-1.26]*
H05	6,760	46,273,120	0.18	0.14	0.14	0.05	0.05	0.04	1.33 [1.23-1.44]*	1.29 [1.19-1.39]*
H10	6,180	46,168,510	0.18	0.12	0.13	0.06	0.05	0.04	1.42 [1.32-1.54]*	1.32 [1.22-1.43]*
H11	1,260	46,396,840	0.03	0.03	0.03	0.00	0.00	0.00	1.10 [0.91-1.32]	1.07 [0.88-1.30]
H16	700	46,406,540	0.02	0.01	0.01	0.01	0.01	0.01	1.92 [1.54-2.41]*	1.75 [1.39-2.21]*
H18	2,230	46,401,710	0.07	0.04	0.04	0.03	0.04	0.03	1.91 [1.67-2.18]*	1.70 [1.48-1.95]*
H20	830	46,409,100	0.02	0.02	0.02	0.01	0.01	0.01	1.40 [1.12-1.74]*	1.35 [1.08-1.70]*
H26	1,510	46,395,670	0.04	0.03	0.03	0.02	0.02	0.02	1.60 [1.36-1.87]*	1.56 [1.32-1.83]*
H33	1,090	46,404,830	0.02	0.02	0.02	0.00	0.00	0.00	1.10 [0.89-1.35]	1.08 [0.87-1.34]
H35	1,320	46,353,910	0.03	0.03	0.03	0.00	0.00	0.00	1.16 [0.96-1.39]	1.14 [0.94-1.38]
H36	570	46,415,230	0.01	0.01	0.01	0.00	0.00	0.00	1.09 [0.81-1.48]	1.14 [0.83-1.57]
H40	650	46,408,090	0.02	0.01	0.01	0.00	0.00	0.00	1.29 [1.00-1.67]	1.26 [0.97-1.64]
H44	580	46,391,750	0.01	0.01	0.01	0.00	0.00	0.00	1.25 [0.95-1.64]	1.14 [0.87-1.51]
H46	410	46,414,300	0.01	0.01	0.01	0.00	0.00	0.00	1.43 [1.04-1.97]	1.45 [1.04-2.02]
H47	3,090	46,390,770	0.07	0.06	0.07	0.01	0.01	0.00	1.10 [0.97-1.24]	1.06 [0.93-1.20]
H49	1,250	46,402,370	0.03	0.03	0.03	0.00	0.00	0.00	0.97 [0.80-1.18]	0.92 [0.75-1.12]
H50	20,760	46,019,910	0.46	0.45	0.46	0.01	0.00	−0.01	1.01 [0.96-1.05]	0.99 [0.94-1.04]
H52	4,230	46,368,660	0.10	0.09	0.09	0.01	0.01	0.01	1.13 [1.02-1.25]	1.10 [0.99-1.21]
H53	15,770	46,262,430	0.38	0.33	0.32	0.05	0.06	0.04	1.18 [1.12-1.24]*	1.13 [1.07-1.19]*
H54	3,590	46,370,660	0.08	0.08	0.08	0.00	0.00	0.00	1.00 [0.89-1.12]	0.96 [0.86-1.08]
H55	1,800	46,387,640	0.04	0.04	0.04	0.00	0.00	0.00	0.95 [0.80-1.11]	0.92 [0.78-1.09]
H57	2,400	46,376,690	0.06	0.05	0.05	0.02	0.01	0.01	1.28 [1.13-1.46]*	1.22 [1.07-1.40]*

<18 - H60-H95										
H60	4,500	46,338,940	0.12	0.09	0.09	0.03	0.03	0.03	1.32 [1.20-1.46]*	1.28 [1.16-1.41]*
H61	12,220	46,206,730	0.32	0.25	0.25	0.07	0.06	0.06	1.26 [1.19-1.33]*	1.22 [1.15-1.29]*
H65	62,420	44,949,020	1.59	1.33	1.33	0.25	0.26	0.22	1.20 [1.17-1.23]*	1.16 [1.13-1.19]*
H66	25,910	45,698,900	0.64	0.55	0.53	0.10	0.11	0.09	1.21 [1.16-1.25]*	1.17 [1.13-1.22]*
H69	1,040	46,400,890	0.03	0.02	0.02	0.01	0.01	0.01	1.43 [1.18-1.73]*	1.44 [1.18-1.76]*
H70	1,620	46,387,430	0.04	0.03	0.03	0.00	0.00	0.00	1.04 [0.88-1.23]	1.01 [0.85-1.20]
H71	3,430	46,369,850	0.08	0.07	0.08	0.01	0.00	0.00	1.07 [0.95-1.19]	1.05 [0.94-1.18]
H72	7,870	46,291,160	0.19	0.16	0.16	0.03	0.03	0.02	1.17 [1.09-1.26]*	1.14 [1.06-1.23]*
H73	7,240	46,297,110	0.18	0.15	0.15	0.03	0.03	0.03	1.19 [1.10-1.28]*	1.16 [1.07-1.25]*
H74	3,780	46,355,750	0.09	0.08	0.08	0.01	0.01	0.01	1.10 [0.99-1.23]	1.06 [0.95-1.19]
H81	410	46,415,370	0.01	0.01	0.01	0.00	0.00	0.00	1.31 [0.94-1.81]	1.23 [0.87-1.73]
H83	900	46,405,990	0.02	0.02	0.02	0.00	0.00	0.00	1.18 [0.95-1.47]	1.12 [0.89-1.40]
H90	10,630	46,263,300	0.26	0.22	0.23	0.04	0.04	0.03	1.16 [1.09-1.23]*	1.12 [1.05-1.19]*
H91	13,600	46,174,140	0.33	0.29	0.29	0.04	0.04	0.03	1.15 [1.08-1.21]*	1.11 [1.05-1.17]*
H92	5,810	46,313,000	0.15	0.12	0.12	0.03	0.03	0.02	1.23 [1.13-1.33]*	1.18 [1.08-1.29]*
H93	1,520	46,400,020	0.04	0.03	0.03	0.01	0.01	0.01	1.20 [1.01-1.43]	1.16 [0.98-1.39]
H95	700	46,408,100	0.02	0.01	0.01	0.00	0.00	0.00	1.20 [0.93-1.54]	1.17 [0.90-1.51]
<18 - I00-I99										
I07	620	46,411,290	0.01	0.01	0.01	0.00	0.00	0.00	0.89 [0.67-1.18]	0.89 [0.67-1.20]
I08	800	46,411,680	0.02	0.02	0.02	0.00	0.00	0.00	1.08 [0.84-1.37]	1.06 [0.83-1.37]
I10	7,710	46,347,770	0.18	0.16	0.15	0.02	0.03	0.03	1.21 [1.12-1.31]*	1.17 [1.08-1.27]*
I15	710	46,406,890	0.02	0.01	0.02	0.00	0.00	0.00	1.07 [0.84-1.38]	1.08 [0.84-1.39]
I25	720	46,412,220	0.01	0.02	0.01	0.00	0.00	0.00	1.06 [0.81-1.37]	1.08 [0.83-1.41]
I26	1,370	46,409,580	0.03	0.03	0.03	0.00	0.00	0.00	1.07 [0.88-1.29]	1.03 [0.84-1.26]
I27	580	46,392,720	0.01	0.01	0.01	0.00	0.00	0.00	0.85 [0.63-1.15]	0.77 [0.57-1.05]
I31	2,180	46,391,700	0.04	0.05	0.04	0.00	0.00	0.00	1.02 [0.88-1.19]	0.99 [0.85-1.16]
I34	1,150	46,405,630	0.03	0.02	0.03	0.00	0.00	0.00	0.99 [0.81-1.21]	0.93 [0.76-1.15]
I35	1,130	46,404,640	0.02	0.02	0.03	0.00	0.00	0.00	0.95 [0.78-1.17]	0.96 [0.78-1.18]
I37	820	46,407,830	0.02	0.02	0.02	0.00	0.00	0.00	0.95 [0.75-1.20]	0.94 [0.74-1.20]
I38	680	46,390,600	0.02	0.01	0.01	0.00	0.00	0.00	1.09 [0.84-1.40]	1.06 [0.82-1.38]
I42	1,070	46,392,960	0.02	0.02	0.02	0.00	0.00	0.00	1.17 [0.96-1.43]	1.18 [0.95-1.45]
I44	1,050	46,406,130	0.03	0.02	0.02	0.00	0.01	0.01	1.29 [1.04-1.58]	1.26 [1.02-1.56]
I45	3,300	46,386,840	0.07	0.07	0.07	0.00	0.00	0.00	1.03 [0.91-1.16]	0.99 [0.87-1.12]
I46	1,270	46,407,590	0.03	0.03	0.03	0.00	0.00	0.00	1.08 [0.89-1.30]	1.04 [0.85-1.26]
I47	3,280	46,365,780	0.08	0.07	0.07	0.01	0.01	0.01	1.16 [1.03-1.31]	1.11 [0.98-1.25]
I48	1,020	46,407,780	0.02	0.02	0.02	0.00	0.00	0.00	1.06 [0.85-1.32]	1.01 [0.80-1.27]
I49	3,060	46,388,440	0.07	0.06	0.06	0.01	0.01	0.01	1.16 [1.03-1.31]	1.10 [0.96-1.24]
I50	1,220	46,392,750	0.03	0.03	0.02	0.00	0.00	0.00	1.09 [0.90-1.33]	1.10 [0.90-1.35]
I51	3,230	46,377,740	0.07	0.07	0.07	0.00	0.01	0.00	1.09 [0.97-1.23]	1.07 [0.95-1.22]
I61	560	46,409,390	0.01	0.01	0.01	0.00	0.00	0.00	0.88 [0.65-1.19]	0.88 [0.64-1.20]
I63	720	46,405,470	0.02	0.02	0.01	0.00	0.00	0.00	1.10 [0.86-1.42]	1.17 [0.90-1.51]
I67	640	46,412,070	0.01	0.01	0.01	0.00	0.00	0.00	1.08 [0.82-1.41]	1.08 [0.82-1.43]
I69	770	46,405,670	0.02	0.02	0.02	0.00	0.00	0.00	0.94 [0.73-1.21]	0.91 [0.70-1.17]
I73	1,830	46,402,510	0.05	0.04	0.03	0.01	0.01	0.01	1.44 [1.24-1.68]*	1.35 [1.15-1.58]*
I77	610	46,411,330	0.01	0.01	0.01	0.00	0.00	0.00	1.19 [0.91-1.56]	1.13 [0.85-1.49]
I78	580	46,407,870	0.01	0.01	0.01	0.00	0.00	0.00	1.03 [0.77-1.36]	1.01 [0.76-1.35]
I80	1,680	46,402,560	0.04	0.04	0.03	0.00	0.01	0.00	1.15 [0.98-1.36]	1.11 [0.93-1.32]
I82	710	46,410,470	0.01	0.02	0.01	0.00	0.00	0.00	1.00 [0.77-1.29]	0.98 [0.75-1.29]
I83	1,420	46,407,240	0.03	0.03	0.02	−0.01	0.00	0.00	1.05 [0.86-1.27]	1.01 [0.82-1.24]
I84	3,640	46,369,670	0.08	0.08	0.07	0.00	0.01	0.01	1.21 [1.08-1.36]*	1.17 [1.04-1.31]*
I86	1,950	46,398,510	0.04	0.04	0.04	0.00	0.00	0.00	1.03 [0.88-1.21]	0.99 [0.84-1.17]
I87	570	46,412,600	0.01	0.01	0.01	0.00	0.00	0.00	1.06 [0.79-1.42]	1.03 [0.76-1.39]
I88	12,370	46,238,610	0.33	0.25	0.26	0.08	0.07	0.06	1.26 [1.19-1.33]*	1.22 [1.15-1.29]*
I89	760	46,405,850	0.02	0.02	0.02	0.00	0.00	0.00	1.16 [0.91-1.47]	1.08 [0.84-1.39]
I95	6,070	46,346,760	0.14	0.13	0.13	0.01	0.01	0.01	1.06 [0.97-1.16]	1.04 [0.95-1.14]
<18 - J00-J99										
J00	9,280	46,179,660	0.26	0.19	0.18	0.07	0.07	0.07	1.41 [1.32-1.50]*	1.35 [1.27-1.44]*
J01	1,120	46,404,840	0.03	0.02	0.02	0.01	0.01	0.01	1.44 [1.19-1.74]*	1.36 [1.12-1.66]*
J02	13,140	46,194,760	0.33	0.27	0.28	0.06	0.05	0.04	1.19 [1.13-1.26]*	1.15 [1.09-1.22]*
J03	113,070	44,582,020	2.92	2.43	2.44	0.48	0.48	0.40	1.20 [1.17-1.22]*	1.16 [1.14-1.18]*
J04	950	46,397,920	0.02	0.02	0.02	0.01	0.01	0.01	1.32 [1.07-1.63]*	1.29 [1.05-1.60]
J05	21,810	45,724,110	0.47	0.48	0.47	0.00	0.00	−0.01	1.01 [0.96-1.05]	0.98 [0.94-1.03]
J06	72,590	43,287,340	2.00	1.59	1.55	0.41	0.45	0.39	1.29 [1.26-1.32]*	1.25 [1.22-1.27]*
J10	4,640	46,367,210	0.12	0.10	0.10	0.02	0.02	0.01	1.23 [1.12-1.35]*	1.15 [1.04-1.26]*
J11	1,070	46,399,400	0.03	0.02	0.02	0.01	0.00	0.00	1.18 [0.97-1.44]	1.13 [0.92-1.38]
J12	2,540	46,376,190	0.06	0.05	0.05	0.01	0.01	0.01	1.16 [1.02-1.32]	1.10 [0.96-1.25]
J13	610	46,401,710	0.01	0.01	0.01	0.00	0.00	0.00	1.18 [0.90-1.53]	1.09 [0.83-1.43]
J15	1,920	46,372,000	0.05	0.04	0.04	0.01	0.01	0.01	1.22 [1.05-1.41]*	1.16 [1.00-1.35]
J18	33,390	45,603,240	0.87	0.69	0.67	0.18	0.20	0.18	1.30 [1.26-1.35]*	1.25 [1.21-1.30]*
J20	1,230	46,361,620	0.03	0.03	0.02	0.01	0.01	0.01	1.30 [1.09-1.56]*	1.25 [1.04-1.50]
J21	3,820	44,624,160	0.09	0.09	0.08	0.00	0.00	0.00	1.04 [0.94-1.16]	1.00 [0.90-1.11]
J22	59,190	45,172,410	1.70	1.20	1.19	0.50	0.51	0.44	1.43 [1.39-1.46]*	1.35 [1.31-1.38]*
J30	20,680	46,235,050	0.93	0.32	0.33	0.61	0.60	0.54	2.83 [2.72-2.95]*	2.38 [2.28-2.48]*
J31	3,600	46,361,390	0.13	0.06	0.06	0.06	0.06	0.05	1.96 [1.78-2.17]*	1.68 [1.52-1.87]*
J32	4,940	46,361,870	0.13	0.10	0.10	0.03	0.03	0.02	1.31 [1.20-1.44]*	1.21 [1.10-1.33]*
J33	1,800	46,398,900	0.05	0.04	0.03	0.01	0.01	0.01	1.41 [1.21-1.64]*	1.21 [1.03-1.42]
J34	23,060	46,069,540	0.65	0.46	0.44	0.19	0.21	0.18	1.48 [1.42-1.54]*	1.37 [1.31-1.43]*
J35	90,040	44,505,370	2.43	1.91	1.90	0.51	0.53	0.45	1.28 [1.25-1.30]*	1.23 [1.20-1.26]*
J36	5,480	46,364,010	0.12	0.12	0.11	0.00	0.01	0.01	1.11 [1.01-1.22]	1.10 [1.00-1.21]
J38	2,730	46,339,560	0.07	0.06	0.05	0.01	0.01	0.01	1.23 [1.08-1.39]*	1.12 [0.99-1.28]
J39	2,310	46,373,680	0.06	0.05	0.05	0.01	0.01	0.01	1.27 [1.11-1.45]*	1.17 [1.02-1.34]
J40	440	46,410,240	0.01	0.01	0.01	0.00	0.00	0.00	1.32 [0.96-1.82]	1.24 [0.89-1.73]
J43	660	46,411,630	0.02	0.01	0.01	0.00	0.00	0.00	1.21 [0.93-1.57]	1.17 [0.89-1.53]
J44	990	46,401,090	0.03	0.02	0.02	0.01	0.01	0.01	1.81 [1.49-2.20]*	1.54 [1.26-1.89]*
J45	161,550	43,482,590	6.89	2.87	2.76	4.02	4.13	3.54	2.50 [2.46-2.53]*	2.06 [2.02-2.09]*
J46	5,670	46,251,990	0.27	0.08	0.08	0.19	0.19	0.17	3.34 [3.10-3.60]*	2.76 [2.55-2.99]*
J47	1,040	46,404,390	0.03	0.02	0.02	0.01	0.01	0.00	1.29 [1.06-1.58]	1.15 [0.93-1.42]
J69	1,690	46,398,510	0.03	0.04	0.04	0.00	0.00	0.00	0.93 [0.78-1.10]	0.90 [0.76-1.07]
J81	520	46,410,290	0.01	0.01	0.01	0.00	0.00	0.00	0.97 [0.72-1.31]	0.96 [0.70-1.30]
J86	1,270	46,393,690	0.03	0.03	0.03	0.00	0.00	0.00	1.07 [0.89-1.30]	1.10 [0.91-1.33]
J90	5,940	46,342,440	0.14	0.13	0.12	0.01	0.01	0.01	1.11 [1.01-1.21]	1.11 [1.02-1.22]
J93	2,910	46,382,590	0.06	0.06	0.06	0.00	0.00	0.00	1.05 [0.93-1.20]	1.06 [0.93-1.21]
J95	650	46,399,290	0.01	0.01	0.01	0.00	0.00	0.00	0.98 [0.75-1.27]	0.91 [0.70-1.20]
J96	3,060	46,383,370	0.07	0.06	0.06	0.01	0.01	0.01	1.21 [1.07-1.36]*	1.12 [0.99-1.26]
J98	10,240	46,193,100	0.26	0.21	0.21	0.05	0.05	0.04	1.25 [1.17-1.33]*	1.17 [1.10-1.25]*
<18 - K00-K95										
K00	23,020	46,121,370	0.58	0.48	0.51	0.10	0.07	0.06	1.14 [1.09-1.19]*	1.11 [1.06-1.16]*
K01	36,540	46,015,180	0.88	0.77	0.75	0.11	0.13	0.11	1.18 [1.14-1.22]*	1.14 [1.10-1.18]*
K02	124,750	44,514,260	2.94	2.77	2.87	0.17	0.07	0.03	1.02 [1.00-1.04]	1.01 [0.99-1.03]
K03	3,080	46,391,910	0.07	0.06	0.07	0.01	0.00	0.00	1.00 [0.89-1.13]	0.98 [0.86-1.11]
K04	18,040	46,189,010	0.40	0.39	0.40	0.01	0.00	0.00	1.00 [0.95-1.05]	0.99 [0.94-1.05]
K05	12,650	46,314,290	0.29	0.27	0.24	0.02	0.05	0.04	1.21 [1.14-1.29]*	1.16 [1.09-1.24]*
K06	1,160	46,402,690	0.03	0.02	0.02	0.00	0.00	0.00	1.19 [0.98-1.44]	1.12 [0.92-1.37]
K07	28,850	46,042,070	0.71	0.60	0.62	0.10	0.09	0.07	1.15 [1.10-1.19]*	1.11 [1.07-1.16]*
K08	21,350	46,141,220	0.48	0.46	0.46	0.02	0.02	0.01	1.04 [0.99-1.09]	1.02 [0.98-1.07]

K09	1,230	46,390,240	0.03	0.03	0.03	0.01	0.01	0.01	1.23 [1.02-1.48]	1.19 [0.98-1.44]
K10	920	46,408,610	0.02	0.02	0.02	0.00	0.00	0.00	1.26 [1.02-1.57]	1.21 [0.97-1.52]
K11	3,480	46,369,940	0.09	0.07	0.07	0.01	0.01	0.01	1.16 [1.04-1.30]*	1.12 [1.00-1.25]
K12	6,550	46,319,930	0.16	0.14	0.14	0.03	0.03	0.02	1.19 [1.10-1.29]*	1.17 [1.08-1.27]*
K13	6,360	46,324,230	0.17	0.13	0.13	0.04	0.04	0.03	1.30 [1.20-1.41]*	1.26 [1.16-1.37]*
K14	1,920	46,387,870	0.05	0.04	0.04	0.01	0.01	0.01	1.32 [1.14-1.53]*	1.26 [1.09-1.47]*
K20	4,150	46,378,550	0.12	0.08	0.08	0.03	0.04	0.03	1.49 [1.35-1.64]*	1.34 [1.21-1.49]*
K21	20,450	45,682,780	0.50	0.43	0.40	0.07	0.09	0.05	1.23 [1.18-1.29]*	1.11 [1.06-1.17]*
K22	4,700	46,359,560	0.11	0.10	0.09	0.01	0.02	0.01	1.21 [1.10-1.33]*	1.12 [1.01-1.24]
K25	1,540	46,405,300	0.04	0.03	0.03	0.01	0.01	0.01	1.30 [1.10-1.54]*	1.27 [1.07-1.52]*
K26	1,110	46,408,850	0.03	0.02	0.02	0.00	0.00	0.00	1.21 [0.99-1.48]	1.18 [0.95-1.46]
K29	22,420	46,150,230	0.55	0.47	0.45	0.08	0.10	0.08	1.23 [1.17-1.28]*	1.16 [1.11-1.22]*
K30	3,350	46,382,730	0.07	0.07	0.06	0.00	0.01	0.01	1.19 [1.06-1.34]*	1.12 [0.99-1.26]
K31	2,820	46,389,310	0.06	0.06	0.06	0.00	0.01	0.00	1.11 [0.97-1.26]	1.04 [0.91-1.19]
K35	35,200	45,972,980	0.79	0.76	0.75	0.03	0.03	0.02	1.04 [1.00-1.08]	1.02 [0.98-1.06]
K36	580	46,411,650	0.01	0.01	0.01	0.00	0.00	0.00	1.35 [1.03-1.76]	1.36 [1.03-1.79]
K37	7,090	46,334,220	0.16	0.15	0.15	0.01	0.01	0.01	1.08 [0.99-1.17]	1.04 [0.95-1.13]
K38	3,670	46,381,200	0.08	0.08	0.08	0.01	0.01	0.00	1.08 [0.97-1.21]	1.05 [0.94-1.18]
K40	14,550	45,882,350	0.31	0.32	0.31	0.00	0.01	−0.01	1.02 [0.96-1.08]	0.98 [0.93-1.04]
K42	6,960	46,207,300	0.17	0.15	0.14	0.02	0.03	0.01	1.19 [1.10-1.28]*	1.09 [1.00-1.18]
K43	2,800	46,368,610	0.06	0.06	0.05	0.00	0.01	0.00	1.13 [1.00-1.28]	1.05 [0.92-1.20]
K44	7,250	46,367,100	0.17	0.15	0.13	0.02	0.04	0.03	1.27 [1.17-1.38]*	1.17 [1.08-1.28]*
K50	5,460	46,361,370	0.17	0.10	0.10	0.07	0.08	0.07	1.80 [1.65-1.96]*	1.67 [1.53-1.82]*
K51	4,730	46,374,900	0.13	0.10	0.09	0.03	0.04	0.03	1.44 [1.31-1.58]*	1.36 [1.23-1.50]*
K52	31,400	45,010,920	0.77	0.68	0.63	0.09	0.14	0.12	1.22 [1.18-1.27]*	1.19 [1.14-1.23]*
K56	5,940	46,320,500	0.14	0.13	0.13	0.01	0.01	0.01	1.08 [0.99-1.18]	1.04 [0.95-1.14]
K57	1,080	46,411,950	0.02	0.02	0.02	0.00	0.00	0.00	1.15 [0.93-1.43]	1.06 [0.84-1.32]
K58	12,600	46,336,490	0.30	0.26	0.24	0.04	0.07	0.05	1.29 [1.21-1.37]*	1.21 [1.14-1.29]*
K59	50,040	45,515,250	1.26	1.06	1.05	0.20	0.20	0.16	1.19 [1.16-1.23]*	1.15 [1.11-1.18]*
K60	4,900	46,337,650	0.13	0.10	0.09	0.03	0.04	0.03	1.40 [1.28-1.53]*	1.34 [1.22-1.47]*
K61	4,140	46,356,790	0.10	0.09	0.08	0.02	0.03	0.03	1.32 [1.20-1.47]*	1.32 [1.19-1.47]*
K62	11,350	46,253,380	0.29	0.23	0.21	0.05	0.07	0.06	1.34 [1.26-1.42]*	1.26 [1.18-1.35]*
K63	5,740	46,365,800	0.15	0.12	0.11	0.03	0.04	0.03	1.40 [1.29-1.53]*	1.30 [1.19-1.42]*
K64	6,340	46,392,880	0.16	0.13	0.12	0.03	0.04	0.03	1.34 [1.23-1.46]*	1.27 [1.16-1.39]*
K65	1,420	46,403,100	0.03	0.03	0.03	0.00	0.00	0.00	1.05 [0.88-1.26]	1.06 [0.88-1.28]
K66	6,890	46,368,100	0.15	0.15	0.14	0.00	0.02	0.01	1.11 [1.02-1.21]	1.07 [0.99-1.17]
K72	650	46,410,730	0.01	0.01	0.01	0.00	0.00	0.00	0.89 [0.67-1.18]	0.88 [0.66-1.17]
K75	890	46,408,700	0.02	0.02	0.02	0.01	0.01	0.01	1.33 [1.07-1.66]	1.29 [1.03-1.63]
K76	4,220	46,386,910	0.10	0.09	0.09	0.01	0.01	0.01	1.12 [1.00-1.24]	1.07 [0.96-1.20]
K80	9,980	46,342,480	0.20	0.22	0.19	−0.02	0.01	0.00	1.05 [0.97-1.12]	1.02 [0.95-1.10]
K81	1,700	46,406,240	0.03	0.04	0.03	0.00	0.00	0.00	1.06 [0.89-1.26]	1.03 [0.86-1.24]
K82	1,790	46,406,070	0.03	0.04	0.04	0.00	0.00	0.00	0.99 [0.84-1.17]	0.96 [0.81-1.15]
K83	1,910	46,396,030	0.04	0.04	0.04	0.00	0.00	0.00	1.05 [0.89-1.23]	1.01 [0.85-1.19]
K85	2,460	46,399,400	0.05	0.05	0.05	0.00	0.00	0.00	1.03 [0.89-1.18]	1.02 [0.88-1.18]
K86	1,330	46,405,120	0.02	0.03	0.03	−0.01	0.00	0.00	0.86 [0.70-1.05]	0.84 [0.69-1.03]
K90	10,230	46,202,780	0.36	0.18	0.18	0.18	0.19	0.16	2.04 [1.93-2.17]*	1.76 [1.65-1.87]*
K91	2,600	46,377,470	0.06	0.06	0.06	0.00	0.00	0.00	1.06 [0.93-1.21]	0.99 [0.86-1.13]
K92	19,970	46,176,390	0.47	0.42	0.40	0.05	0.08	0.05	1.19 [1.14-1.25]*	1.13 [1.08-1.19]*
<18 - L00-L99										
L00	690	46,378,430	0.02	0.01	0.01	0.01	0.01	0.01	1.55 [1.23-1.94]*	1.54 [1.22-1.94]*
L01	2,750	46,316,580	0.10	0.05	0.05	0.06	0.06	0.05	2.22 [2.00-2.48]*	2.09 [1.87-2.33]*
L02	14,140	46,174,860	0.36	0.29	0.28	0.07	0.08	0.07	1.29 [1.22-1.36]*	1.26 [1.19-1.33]*
L03	19,260	46,107,130	0.53	0.39	0.38	0.14	0.14	0.13	1.38 [1.32-1.44]*	1.34 [1.28-1.40]*
L04	3,950	46,337,620	0.10	0.08	0.08	0.01	0.01	0.01	1.14 [1.03-1.26]	1.12 [1.01-1.25]
L05	10,020	46,196,660	0.22	0.22	0.20	0.00	0.02	0.01	1.09 [1.02-1.17]	1.06 [0.98-1.14]
L08	6,040	46,276,120	0.19	0.11	0.11	0.07	0.08	0.08	1.72 [1.59-1.86]*	1.68 [1.55-1.82]*
L20	4,620	46,203,740	0.39	0.02	0.02	0.37	0.37	0.37	18.85 [16.88-21.04]*	16.21 [14.49-18.13]*
L21	250	46,378,120	0.01	0.00	0.00	0.01	0.01	0.01	3.22 [2.24-4.64]*	3.12 [2.12-4.57]*
L22	1,270	46,310,890	0.03	0.03	0.03	0.00	0.00	0.00	1.01 [0.84-1.21]	0.95 [0.78-1.15]
L23	880	46,399,270	0.05	0.01	0.01	0.03	0.04	0.03	4.13 [3.41-5.01]*	3.36 [2.75-4.12]*
L25	480	46,408,790	0.02	0.01	0.01	0.01	0.01	0.01	1.95 [1.49-2.56]*	1.80 [1.36-2.37]*
L27	2,290	46,374,940	0.08	0.04	0.04	0.04	0.04	0.04	2.18 [1.93-2.47]*	1.91 [1.68-2.18]*
L28	330	46,412,720	0.02	0.00	0.00	0.02	0.02	0.02	6.73 [4.77-9.50]*	6.04 [4.24-8.60]*
L29	3,750	46,384,700	0.10	0.08	0.07	0.03	0.03	0.03	1.46 [1.31-1.62]*	1.36 [1.21-1.51]*
L30	57,680	45,032,470	4.41	0.45	0.44	3.96	3.96	3.92	9.92 [9.66-10.19]*	9.12 [8.88-9.37]*
L40	3,650	46,386,640	0.12	0.07	0.06	0.05	0.06	0.06	2.04 [1.84-2.26]*	2.01 [1.80-2.23]*
L50	7,050	46,279,420	0.25	0.12	0.13	0.13	0.13	0.11	2.03 [1.89-2.17]*	1.75 [1.62-1.88]*
L51	2,000	46,370,980	0.04	0.04	0.04	0.00	0.00	0.00	0.97 [0.83-1.13]	0.95 [0.82-1.11]
L52	510	46,411,610	0.01	0.01	0.01	0.00	0.00	0.00	1.47 [1.11-1.96]*	1.36 [1.01-1.82]
L53	2,830	46,358,490	0.08	0.06	0.06	0.02	0.02	0.02	1.45 [1.29-1.63]*	1.37 [1.21-1.54]*
L60	6,790	46,320,020	0.18	0.14	0.13	0.04	0.04	0.03	1.32 [1.22-1.43]*	1.24 [1.14-1.34]*
L65	730	46,410,610	0.02	0.01	0.01	0.01	0.01	0.01	1.78 [1.41-2.24]*	1.64 [1.29-2.08]*
L70	1,880	46,402,950	0.05	0.04	0.04	0.01	0.01	0.01	1.37 [1.17-1.59]*	1.27 [1.08-1.49]*
L72	8,300	46,302,020	0.18	0.18	0.16	0.00	0.02	0.01	1.10 [1.02-1.19]	1.07 [0.99-1.16]
L73	1,620	46,403,510	0.04	0.03	0.03	0.01	0.01	0.01	1.32 [1.12-1.55]*	1.24 [1.05-1.47]
L80	280	46,415,240	0.01	0.00	0.00	0.01	0.01	0.01	2.46 [1.72-3.53]*	2.34 [1.60-3.42]*
L81	1,330	46,389,820	0.04	0.03	0.03	0.01	0.01	0.01	1.50 [1.26-1.78]*	1.45 [1.21-1.72]*
L83	470	46,413,550	0.02	0.01	0.01	0.01	0.01	0.01	1.58 [1.18-2.12]*	1.51 [1.11-2.04]*
L85	490	46,411,350	0.02	0.01	0.01	0.01	0.01	0.01	2.18 [1.67-2.84]*	2.18 [1.66-2.87]*
L89	1,310	46,405,190	0.03	0.03	0.03	0.00	0.00	0.00	0.99 [0.82-1.20]	0.97 [0.80-1.18]
L90	4,070	46,358,720	0.09	0.09	0.09	0.00	0.00	0.00	1.04 [0.94-1.16]	1.02 [0.92-1.14]
L91	3,380	46,356,840	0.08	0.07	0.07	0.01	0.02	0.02	1.26 [1.12-1.40]*	1.25 [1.11-1.40]*
L92	2,300	46,369,450	0.05	0.05	0.05	0.01	0.01	0.00	1.11 [0.97-1.28]	1.04 [0.90-1.20]
L94	440	46,413,380	0.01	0.01	0.01	0.01	0.01	0.00	1.53 [1.14-2.06]*	1.54 [1.13-2.09]*
L97	450	46,413,760	0.01	0.01	0.01	0.00	0.00	0.00	1.28 [0.94-1.75]	1.34 [0.97-1.85]
L98	7,640	46,283,450	0.19	0.16	0.15	0.04	0.04	0.04	1.28 [1.19-1.38]*	1.25 [1.16-1.35]*
<18 - M00-M99										
M00	1,530	46,383,650	0.04	0.03	0.03	0.01	0.01	0.01	1.40 [1.20-1.64]*	1.37 [1.16-1.61]*
M02	1,340	46,397,250	0.03	0.03	0.03	0.00	0.00	0.00	1.13 [0.94-1.34]	1.11 [0.93-1.33]
M06	1,190	46,409,890	0.03	0.02	0.02	0.00	0.01	0.00	1.29 [1.06-1.57]	1.21 [0.99-1.49]
M07	520	46,414,600	0.01	0.01	0.01	0.00	0.00	0.00	1.52 [1.14-2.03]*	1.46 [1.08-1.97]
M08	2,670	46,373,880	0.07	0.05	0.05	0.02	0.02	0.02	1.34 [1.19-1.51]*	1.30 [1.15-1.47]*
M13	2,500	46,388,440	0.06	0.05	0.05	0.01	0.02	0.01	1.36 [1.19-1.55]*	1.27 [1.11-1.45]*
M16	570	46,414,570	0.01	0.01	0.01	0.00	0.00	0.00	1.12 [0.84-1.49]	1.08 [0.80-1.46]
M17	1,760	46,404,790	0.04	0.04	0.03	0.00	0.00	0.00	1.14 [0.96-1.35]	1.09 [0.91-1.30]
M19	1,450	46,409,170	0.03	0.03	0.03	0.00	0.01	0.00	1.26 [1.05-1.51]*	1.14 [0.94-1.39]
M20	4,620	46,360,320	0.11	0.10	0.09	0.01	0.02	0.01	1.19 [1.08-1.32]*	1.15 [1.04-1.27]*
M21	6,080	46,345,830	0.14	0.13	0.14	0.01	0.00	0.00	1.00 [0.92-1.09]	0.97 [0.88-1.06]
M22	2,600	46,391,030	0.06	0.05	0.05	0.01	0.01	0.01	1.26 [1.10-1.44]*	1.18 [1.03-1.35]
M23	14,610	46,282,650	0.32	0.31	0.28	0.00	0.04	0.03	1.14 [1.08-1.21]*	1.09 [1.03-1.16]*
M24	10,700	46,244,310	0.24	0.23	0.21	0.01	0.03	0.02	1.15 [1.08-1.23]*	1.10 [1.03-1.18]*
M25	29,840	46,051,740	0.74	0.62	0.62	0.12	0.13	0.10	1.21 [1.16-1.25]*	1.15 [1.10-1.19]*
M30	1,200	46,390,000	0.03	0.02	0.02	0.01	0.01	0.01	1.28 [1.07-1.53]*	1.29 [1.08-1.55]*
M32	510	46,413,280	0.02	0.01	0.01	0.01	0.01	0.01	1.73 [1.30-2.30]*	1.73 [1.29-2.32]*
M35	5,300	46,382,020	0.15	0.10	0.10	0.04	0.04	0.04	1.43 [1.31-1.56]*	1.32 [1.21-1.45]*

M40	810	46,410,480	0.02	0.02	0.02	0.00	0.00	0.00	1.04 [0.82-1.32]	0.98 [0.77-1.26]
M41	7,660	46,342,280	0.18	0.16	0.17	0.01	0.01	0.01	1.07 [0.99-1.15]	1.03 [0.96-1.12]
M43	2,600	46,382,240	0.06	0.06	0.06	0.00	0.00	0.00	1.02 [0.90-1.17]	0.98 [0.85-1.12]
M46	600	46,411,340	0.01	0.01	0.01	0.00	0.00	0.00	1.10 [0.83-1.45]	1.08 [0.81-1.44]
M47	1,000	46,412,300	0.02	0.02	0.02	0.00	0.00	0.00	1.07 [0.85-1.35]	1.00 [0.79-1.27]
M48	900	46,412,470	0.02	0.02	0.02	0.00	0.00	0.00	1.12 [0.88-1.41]	1.05 [0.82-1.33]
M51	4,230	46,391,040	0.09	0.09	0.08	0.00	0.02	0.01	1.24 [1.11-1.38]*	1.17 [1.04-1.30]*
M53	670	46,410,130	0.01	0.01	0.01	0.00	0.00	0.00	1.03 [0.78-1.36]	0.99 [0.75-1.31]
M54	26,110	46,193,160	0.59	0.56	0.52	0.04	0.08	0.05	1.15 [1.10-1.19]*	1.09 [1.04-1.14]*
M60	1,490	46,401,730	0.04	0.03	0.03	0.01	0.01	0.01	1.35 [1.15-1.59]*	1.31 [1.11-1.55]*
M62	3,390	46,380,020	0.07	0.07	0.08	0.00	0.00	0.00	0.99 [0.88-1.12]	0.94 [0.83-1.06]
M65	6,860	46,323,100	0.17	0.14	0.14	0.03	0.04	0.03	1.26 [1.17-1.37]*	1.23 [1.13-1.33]*
M67	8,910	46,302,020	0.21	0.19	0.18	0.02	0.02	0.02	1.13 [1.05-1.21]*	1.08 [1.01-1.16]
M70	890	46,408,790	0.02	0.02	0.02	0.00	0.00	0.00	1.16 [0.93-1.46]	1.13 [0.89-1.43]
M71	400	46,413,360	0.01	0.01	0.01	0.00	0.00	0.00	1.51 [1.09-2.08]	1.44 [1.04-2.02]
M72	500	46,412,970	0.01	0.01	0.01	0.00	0.00	0.00	1.23 [0.91-1.67]	1.18 [0.85-1.62]
M75	2,060	46,397,090	0.04	0.04	0.04	0.00	0.01	0.00	1.18 [1.01-1.37]	1.12 [0.96-1.31]
M76	610	46,412,830	0.02	0.01	0.01	0.00	0.00	0.00	1.24 [0.95-1.63]	1.13 [0.85-1.50]
M77	680	46,412,700	0.01	0.01	0.01	0.00	0.00	0.00	0.95 [0.73-1.26]	0.88 [0.66-1.17]
M79	24,580	46,138,930	0.60	0.52	0.50	0.08	0.10	0.08	1.20 [1.15-1.25]*	1.15 [1.10-1.20]*
M81	780	46,411,960	0.02	0.02	0.01	0.00	0.01	0.00	1.38 [1.09-1.75]*	1.30 [1.02-1.66]
M84	4,090	46,364,800	0.09	0.09	0.08	0.00	0.01	0.01	1.11 [1.00-1.23]	1.08 [0.96-1.20]
M85	1,820	46,396,390	0.04	0.04	0.04	0.01	0.01	0.01	1.18 [1.01-1.39]	1.13 [0.96-1.33]
M86	2,210	46,377,340	0.06	0.04	0.04	0.02	0.02	0.02	1.36 [1.19-1.56]*	1.33 [1.16-1.53]*
M87	620	46,410,470	0.01	0.01	0.01	0.00	0.00	0.00	1.22 [0.93-1.59]	1.16 [0.88-1.53]
M89	3,700	46,367,470	0.09	0.08	0.08	0.01	0.01	0.01	1.12 [1.01-1.25]	1.09 [0.97-1.22]
M91	1,170	46,394,180	0.03	0.02	0.02	0.00	0.00	0.00	1.16 [0.96-1.39]	1.12 [0.92-1.35]
M92	1,360	46,403,520	0.04	0.03	0.03	0.01	0.01	0.01	1.31 [1.10-1.56]*	1.19 [0.99-1.43]
M93	2,310	46,386,780	0.06	0.05	0.05	0.01	0.01	0.01	1.25 [1.09-1.43]*	1.18 [1.03-1.36]
M94	2,870	46,395,940	0.07	0.06	0.06	0.01	0.01	0.01	1.24 [1.09-1.40]*	1.18 [1.04-1.35]*
M95	1,220	46,402,980	0.03	0.03	0.02	0.00	0.00	0.00	1.21 [1.00-1.46]	1.20 [0.98-1.46]
<18 - N00-N99										
N02	740	46,409,020	0.01	0.02	0.02	0.00	0.00	0.00	0.86 [0.66-1.12]	0.84 [0.64-1.10]
N04	1,260	46,393,120	0.03	0.03	0.03	0.00	0.00	0.00	0.99 [0.82-1.20]	0.98 [0.81-1.19]
N05	1,200	46,399,460	0.03	0.02	0.02	0.01	0.01	0.01	1.36 [1.13-1.63]*	1.32 [1.10-1.60]*
N10	1,850	46,399,340	0.04	0.04	0.04	0.00	0.00	0.00	1.07 [0.92-1.26]	1.05 [0.89-1.24]
N12	10,270	46,324,510	0.22	0.22	0.21	0.00	0.01	0.00	1.05 [0.98-1.12]	1.02 [0.95-1.10]
N13	6,560	46,284,850	0.14	0.14	0.13	0.00	0.01	0.00	1.05 [0.97-1.15]	1.02 [0.94-1.12]
N17	6,260	46,371,570	0.14	0.13	0.13	0.00	0.01	0.01	1.07 [0.98-1.17]	1.07 [0.97-1.17]
N18	1,730	46,396,910	0.04	0.04	0.03	0.00	0.00	0.00	1.14 [0.97-1.34]	1.12 [0.95-1.33]
N19	1,440	46,390,540	0.03	0.03	0.03	0.00	0.00	0.00	1.08 [0.91-1.29]	1.05 [0.87-1.26]
N20	4,090	46,384,780	0.07	0.09	0.08	−0.02	−0.01	−0.01	0.94 [0.84-1.05]	0.92 [0.81-1.03]
N23	1,700	46,401,970	0.03	0.04	0.03	0.00	0.00	0.00	1.03 [0.87-1.22]	0.98 [0.83-1.18]
N27	580	46,408,770	0.01	0.01	0.01	0.00	0.00	0.00	0.96 [0.73-1.27]	0.94 [0.71-1.25]
N28	3,820	46,342,330	0.08	0.08	0.08	0.00	0.00	0.00	0.99 [0.89-1.11]	0.98 [0.88-1.10]
N30	2,060	46,397,820	0.05	0.04	0.04	0.01	0.01	0.01	1.40 [1.21-1.62]*	1.33 [1.15-1.55]*
N31	1,520	46,396,590	0.03	0.03	0.03	0.00	0.00	0.00	0.98 [0.82-1.16]	0.95 [0.79-1.13]
N32	4,700	46,371,770	0.11	0.10	0.10	0.02	0.02	0.01	1.16 [1.05-1.27]*	1.10 [0.99-1.21]
N35	3,260	46,375,500	0.08	0.07	0.06	0.02	0.02	0.02	1.33 [1.19-1.49]*	1.29 [1.15-1.45]*
N36	1,260	46,398,140	0.03	0.03	0.03	0.00	0.00	0.00	1.03 [0.85-1.24]	0.99 [0.82-1.21]
N39	39,040	45,467,300	0.91	0.84	0.81	0.07	0.10	0.08	1.12 [1.09-1.16]*	1.09 [1.05-1.13]*
N43	9,220	46,226,250	0.21	0.20	0.20	0.01	0.01	0.01	1.05 [0.98-1.12]	1.02 [0.96-1.10]
N44	6,960	46,332,880	0.18	0.14	0.16	0.03	0.02	0.02	1.14 [1.05-1.23]*	1.11 [1.03-1.21]*
N45	5,160	46,357,660	0.12	0.11	0.11	0.01	0.01	0.01	1.12 [1.02-1.23]	1.09 [0.99-1.20]
N47	35,010	45,707,570	1.01	0.70	0.69	0.31	0.31	0.29	1.45 [1.41-1.50]*	1.41 [1.36-1.46]*
N48	12,360	46,206,640	0.34	0.25	0.25	0.09	0.09	0.08	1.35 [1.28-1.43]*	1.32 [1.24-1.40]*
N49	730	46,405,580	0.02	0.02	0.02	0.00	0.00	0.00	1.13 [0.88-1.44]	1.11 [0.87-1.43]
N50	12,460	46,248,750	0.30	0.26	0.27	0.04	0.03	0.02	1.12 [1.05-1.19]*	1.08 [1.02-1.15]
N60	510	46,413,220	0.01	0.01	0.01	0.00	0.00	0.00	1.37 [1.01-1.85]	1.34 [0.98-1.84]
N61	1,100	46,402,960	0.03	0.02	0.02	0.00	0.01	0.00	1.26 [1.03-1.54]	1.22 [0.99-1.51]
N62	1,400	46,398,490	0.03	0.03	0.03	0.01	0.01	0.01	1.30 [1.09-1.55]*	1.26 [1.05-1.51]
N63	740	46,409,290	0.02	0.02	0.01	0.00	0.00	0.00	1.19 [0.93-1.53]	1.16 [0.90-1.50]
N64	900	46,409,530	0.02	0.02	0.02	0.00	0.01	0.01	1.37 [1.09-1.71]*	1.35 [1.07-1.70]
N70	900	46,411,510	0.02	0.02	0.02	0.00	0.00	0.00	1.08 [0.85-1.37]	1.06 [0.83-1.35]
N72	690	46,413,020	0.01	0.02	0.01	0.00	0.00	0.00	0.95 [0.72-1.26]	0.96 [0.72-1.28]
N73	6,510	46,366,710	0.14	0.14	0.12	0.00	0.01	0.01	1.11 [1.01-1.21]	1.08 [0.98-1.18]
N75	1,660	46,404,420	0.03	0.04	0.03	−0.01	0.00	0.00	0.93 [0.77-1.11]	0.93 [0.77-1.12]
N76	3,120	46,378,000	0.08	0.06	0.06	0.01	0.01	0.01	1.22 [1.09-1.38]*	1.17 [1.04-1.32]*
N77	4,470	46,375,890	0.09	0.10	0.08	0.00	0.01	0.01	1.10 [0.99-1.22]	1.06 [0.95-1.18]
N80	6,590	46,372,750	0.15	0.14	0.13	0.00	0.02	0.02	1.16 [1.06-1.27]*	1.12 [1.02-1.22]
N81	600	46,414,220	0.01	0.01	0.01	0.00	0.00	0.00	1.27 [0.95-1.69]	1.26 [0.94-1.70]
N83	14,260	46,300,080	0.32	0.31	0.28	0.01	0.03	0.02	1.11 [1.05-1.17]*	1.06 [1.00-1.13]
N84	930	46,411,810	0.02	0.02	0.02	0.00	0.00	0.00	1.17 [0.93-1.48]	1.09 [0.86-1.39]
N85	2,370	46,403,460	0.05	0.05	0.04	0.00	0.01	0.01	1.17 [1.01-1.35]	1.13 [0.97-1.32]
N86	3,150	46,394,140	0.06	0.07	0.06	−0.01	0.00	0.00	1.07 [0.94-1.22]	1.04 [0.91-1.19]
N87	2,350	46,399,970	0.05	0.05	0.04	−0.01	0.01	0.01	1.13 [0.98-1.31]	1.15 [0.98-1.34]
N88	470	46,414,920	0.01	0.01	0.01	0.00	0.00	0.00	1.08 [0.78-1.49]	1.10 [0.78-1.55]
N89	5,590	46,356,520	0.12	0.12	0.11	0.00	0.01	0.01	1.10 [1.00-1.21]	1.07 [0.97-1.17]
N90	2,440	46,389,420	0.06	0.05	0.05	0.00	0.01	0.01	1.23 [1.08-1.41]*	1.22 [1.06-1.41]*
N91	1,030	46,409,930	0.02	0.02	0.02	0.00	0.00	0.00	1.14 [0.92-1.41]	1.11 [0.89-1.38]
N92	9,390	46,352,420	0.23	0.20	0.19	0.03	0.04	0.03	1.21 [1.13-1.30]*	1.16 [1.08-1.25]*
N93	8,160	46,339,660	0.18	0.18	0.15	0.00	0.02	0.02	1.14 [1.06-1.23]*	1.11 [1.02-1.20]
N94	7,730	46,350,760	0.18	0.16	0.15	0.02	0.04	0.03	1.24 [1.15-1.34]*	1.18 [1.09-1.28]*
N99	950	46,403,080	0.02	0.02	0.02	0.00	0.00	0.00	1.01 [0.80-1.26]	0.99 [0.79-1.25]
<18 - O00-O9A										
O00	3,220	46,393,840	0.06	0.07	0.06	−0.01	0.00	0.00	1.02 [0.90-1.16]	0.99 [0.87-1.13]
O02	6,150	46,364,790	0.12	0.14	0.11	−0.02	0.01	0.00	1.05 [0.95-1.15]	1.04 [0.94-1.14]
O03	11,330	46,320,250	0.22	0.25	0.22	−0.04	0.00	0.00	1.00 [0.94-1.07]	0.99 [0.92-1.06]
O04	28,250	46,116,890	0.55	0.63	0.56	−0.08	−0.01	−0.01	0.98 [0.94-1.03]	0.98 [0.94-1.03]
O08	1,690	46,404,950	0.03	0.04	0.03	0.00	0.00	0.00	0.99 [0.83-1.18]	0.99 [0.83-1.18]
O10	710	46,413,940	0.01	0.02	0.01	0.00	0.00	0.00	0.95 [0.72-1.25]	0.95 [0.71-1.27]
O12	3,090	46,391,400	0.06	0.07	0.06	0.00	0.01	0.00	1.10 [0.97-1.25]	1.08 [0.95-1.23]
O13	7,440	46,356,100	0.14	0.17	0.14	−0.02	0.01	0.00	1.04 [0.96-1.13]	1.02 [0.93-1.11]
O14	6,260	46,367,010	0.13	0.14	0.12	−0.01	0.01	0.01	1.12 [1.02-1.22]	1.12 [1.02-1.22]
O16	5,430	46,362,320	0.11	0.12	0.10	−0.01	0.01	0.01	1.10 [1.00-1.21]	1.08 [0.98-1.19]
O20	12,990	46,303,170	0.25	0.29	0.25	−0.04	0.00	0.00	1.00 [0.94-1.07]	1.00 [0.93-1.06]
O21	12,120	46,321,330	0.24	0.27	0.24	−0.02	0.01	0.00	1.03 [0.97-1.10]	1.01 [0.94-1.08]
O22	880	46,412,180	0.02	0.02	0.02	0.00	0.00	0.00	0.95 [0.74-1.22]	0.90 [0.69-1.17]
O23	12,510	46,303,890	0.25	0.28	0.24	−0.02	0.01	0.01	1.03 [0.97-1.10]	1.02 [0.96-1.09]
O24	8,020	46,373,460	0.15	0.18	0.15	−0.03	0.00	0.00	1.00 [0.92-1.09]	1.02 [0.94-1.11]
O26	41,270	46,042,770	0.84	0.91	0.80	−0.07	0.04	0.02	1.05 [1.01-1.08]	1.02 [0.99-1.06]
O28	4,070	46,396,240	0.08	0.09	0.08	−0.01	0.00	0.00	0.98 [0.87-1.10]	0.97 [0.86-1.09]
O30	2,880	46,397,210	0.06	0.06	0.05	−0.01	0.00	0.00	1.08 [0.94-1.23]	1.08 [0.94-1.24]
O32	14,040	46,318,610	0.26	0.31	0.27	−0.05	−0.01	−0.02	0.96 [0.90-1.02]	0.95 [0.89-1.01]
O34	16,530	46,311,030	0.31	0.37	0.31	−0.07	−0.01	−0.01	0.98 [0.92-1.04]	0.97 [0.91-1.03]
O35	3,620	46,390,580	0.07	0.08	0.07	−0.01	0.00	0.00	1.00 [0.89-1.13]	0.99 [0.88-1.13]
O36	57,740	45,989,100	1.18	1.28	1.15	−0.09	0.04	0.02	1.03 [1.00-1.06]	1.02 [0.99-1.05]

O40	3,440	46,396,580	0.07	0.08	0.07	−0.01	0.00	0.00	1.04 [0.92-1.18]	1.06 [0.93-1.20]
O41	5,210	46,375,470	0.10	0.12	0.10	−0.02	0.00	0.00	0.99 [0.89-1.09]	1.00 [0.90-1.10]
O42	27,500	46,200,250	0.53	0.61	0.53	−0.09	0.00	0.00	1.00 [0.96-1.05]	1.00 [0.95-1.04]
O43	3,490	46,390,040	0.06	0.08	0.07	−0.02	0.00	0.00	0.94 [0.83-1.06]	0.96 [0.84-1.09]
O44	1,530	46,407,940	0.03	0.03	0.03	−0.01	0.00	0.00	0.90 [0.74-1.09]	0.87 [0.71-1.06]
O45	1,010	46,410,090	0.02	0.02	0.02	0.00	0.00	0.00	1.01 [0.80-1.26]	1.03 [0.82-1.31]
O46	13,090	46,299,330	0.26	0.29	0.25	−0.03	0.01	0.00	1.04 [0.97-1.10]	1.02 [0.96-1.09]
O47	7,840	46,321,600	0.15	0.17	0.14	−0.03	0.01	0.01	1.05 [0.97-1.14]	1.04 [0.95-1.13]
O48	15,840	46,271,490	0.29	0.36	0.30	−0.06	−0.01	−0.01	0.97 [0.92-1.03]	0.97 [0.91-1.03]
O60	13,890	46,305,570	0.27	0.31	0.27	−0.04	0.00	0.00	1.02 [0.96-1.08]	1.02 [0.96-1.09]
O61	2,280	46,401,390	0.05	0.05	0.04	0.00	0.00	0.00	1.10 [0.94-1.27]	1.05 [0.90-1.23]
O62	10,920	46,340,220	0.21	0.24	0.21	−0.04	−0.01	−0.01	0.97 [0.91-1.04]	0.97 [0.90-1.04]
O63	23,750	46,215,960	0.45	0.53	0.45	−0.08	0.00	−0.01	1.00 [0.95-1.05]	0.98 [0.93-1.03]
O64	3,630	46,379,830	0.07	0.08	0.07	−0.01	0.01	0.00	1.08 [0.96-1.22]	1.07 [0.94-1.20]
O66	4,050	46,385,170	0.07	0.09	0.08	−0.02	−0.01	−0.01	0.90 [0.80-1.01]	0.90 [0.80-1.01]
O67	900	46,410,690	0.02	0.02	0.02	0.00	0.00	0.00	1.07 [0.85-1.36]	1.10 [0.86-1.41]
O68	55,640	45,962,070	1.10	1.24	1.10	−0.14	0.00	0.00	1.00 [0.97-1.03]	1.00 [0.97-1.03]
O69	3,910	46,384,670	0.07	0.09	0.07	−0.02	0.00	0.00	0.99 [0.88-1.11]	0.99 [0.88-1.12]
O70	80,450	45,732,820	1.57	1.81	1.61	−0.25	−0.05	−0.04	0.97 [0.95-1.00]	0.98 [0.95-1.00]
O71	4,190	46,380,220	0.08	0.09	0.08	−0.01	0.00	0.00	1.00 [0.90-1.12]	1.02 [0.91-1.14]
O72	33,630	46,182,990	0.65	0.75	0.66	−0.11	−0.01	−0.02	0.98 [0.94-1.02]	0.97 [0.93-1.02]
O73	1,760	46,403,150	0.03	0.04	0.03	−0.01	0.00	0.00	1.10 [0.93-1.31]	1.11 [0.93-1.32]
O74	690	46,413,310	0.01	0.02	0.01	0.00	0.00	0.00	0.88 [0.66-1.16]	0.87 [0.65-1.16]
O75	16,610	46,292,450	0.31	0.37	0.33	−0.06	−0.02	−0.02	0.95 [0.90-1.01]	0.95 [0.89-1.01]
O80	17,100	46,236,870	0.30	0.39	0.32	−0.09	−0.02	−0.01	0.94 [0.89-1.00]	0.96 [0.91-1.02]
O82	1,060	46,408,780	0.02	0.02	0.02	0.00	0.00	0.00	1.07 [0.86-1.33]	1.07 [0.85-1.34]
O85	1,680	46,408,060	0.03	0.04	0.04	0.00	0.00	0.00	0.93 [0.78-1.11]	0.90 [0.74-1.08]
O86	5,280	46,378,130	0.11	0.12	0.10	−0.01	0.00	0.00	1.03 [0.93-1.13]	1.02 [0.92-1.13]
O90	5,960	46,378,100	0.12	0.13	0.12	−0.01	0.01	0.00	1.06 [0.97-1.17]	1.04 [0.95-1.15]
O91	510	46,414,720	0.01	0.01	0.01	0.00	0.00	0.00	1.07 [0.78-1.46]	1.10 [0.79-1.53]
O92	770	46,412,400	0.01	0.02	0.02	0.00	0.00	0.00	0.96 [0.74-1.24]	0.91 [0.69-1.20]
O98	2,600	46,400,190	0.05	0.06	0.05	0.00	0.00	0.00	1.06 [0.92-1.22]	1.04 [0.90-1.20]
O99	41,790	46,125,480	0.86	0.92	0.82	−0.06	0.04	0.02	1.05 [1.01-1.08]	1.03 [0.99-1.07]
<18 - P00-P96										
P83	1,640	45,386,040	0.04	0.04	0.04	0.00	0.00	0.00	1.03 [0.88-1.21]	1.01 [0.86-1.19]
<18 - Q00-Q99										
Q02	1,930	46,369,840	0.04	0.04	0.04	0.00	0.00	0.00	1.08 [0.93-1.25]	1.05 [0.90-1.22]
Q04	1,520	46,356,970	0.03	0.03	0.03	0.00	0.00	0.00	1.01 [0.85-1.20]	0.97 [0.81-1.16]
Q05	520	46,401,240	0.01	0.01	0.01	0.00	0.00	0.00	1.08 [0.81-1.45]	1.03 [0.76-1.39]
Q07	1,390	46,401,650	0.03	0.03	0.03	0.00	0.00	0.00	1.02 [0.85-1.23]	0.96 [0.80-1.16]
Q10	2,240	46,360,730	0.05	0.05	0.05	0.01	0.01	0.01	1.14 [1.00-1.31]	1.12 [0.98-1.29]
Q12	620	46,396,860	0.02	0.01	0.01	0.00	0.00	0.00	1.25 [0.97-1.63]	1.21 [0.93-1.57]
Q17	6,100	46,209,660	0.14	0.13	0.13	0.01	0.01	0.01	1.07 [0.98-1.16]	1.06 [0.97-1.15]
Q18	2,220	46,360,690	0.05	0.05	0.05	0.01	0.01	0.01	1.19 [1.04-1.36]	1.17 [1.02-1.34]
Q21	4,270	46,098,230	0.09	0.09	0.09	0.00	0.00	0.00	1.01 [0.91-1.12]	0.98 [0.88-1.09]
Q22	980	46,362,480	0.02	0.02	0.02	0.00	0.00	0.00	0.87 [0.69-1.09]	0.87 [0.70-1.10]
Q23	1,580	46,371,540	0.04	0.03	0.03	0.00	0.00	0.00	1.07 [0.91-1.27]	1.04 [0.87-1.23]
Q24	1,660	46,338,740	0.04	0.04	0.04	0.00	0.00	0.00	0.94 [0.79-1.11]	0.92 [0.77-1.09]
Q25	2,510	46,120,720	0.05	0.06	0.05	0.00	0.00	0.00	0.96 [0.84-1.10]	0.93 [0.81-1.07]
Q27	1,460	46,384,050	0.03	0.03	0.03	0.00	0.00	0.00	1.01 [0.84-1.20]	1.00 [0.83-1.19]
Q28	460	46,411,140	0.01	0.01	0.01	0.00	0.00	0.00	0.98 [0.71-1.35]	0.98 [0.71-1.36]
Q31	1,040	46,336,300	0.02	0.02	0.02	0.00	0.00	0.00	0.98 [0.80-1.21]	0.87 [0.70-1.08]
Q35	1,290	46,354,740	0.03	0.03	0.03	0.00	0.00	0.00	1.10 [0.92-1.32]	1.04 [0.87-1.26]
Q38	7,430	45,808,640	0.18	0.16	0.16	0.02	0.02	0.02	1.11 [1.03-1.20]*	1.09 [1.01-1.18]
Q43	1,430	46,346,450	0.03	0.03	0.03	0.00	0.00	0.00	0.98 [0.81-1.17]	0.95 [0.79-1.14]
Q50	840	46,408,370	0.02	0.02	0.02	0.00	0.01	0.00	1.41 [1.12-1.77]*	1.28 [1.00-1.63]
Q51	600	46,413,090	0.01	0.01	0.01	0.00	0.00	0.00	1.09 [0.83-1.44]	1.08 [0.81-1.43]
Q52	960	46,388,380	0.02	0.02	0.02	0.00	0.00	0.00	1.13 [0.91-1.40]	1.07 [0.86-1.33]
Q53	13,320	45,876,510	0.30	0.29	0.30	0.01	0.00	−0.01	0.99 [0.93-1.05]	0.97 [0.91-1.03]
Q54	2,650	46,192,910	0.06	0.06	0.06	0.00	0.00	0.00	1.07 [0.95-1.22]	1.07 [0.94-1.22]
Q55	8,260	46,244,830	0.20	0.17	0.18	0.03	0.02	0.02	1.11 [1.03-1.19]*	1.08 [1.01-1.16]
Q60	800	46,392,770	0.02	0.02	0.02	0.00	0.00	0.00	0.92 [0.72-1.18]	0.92 [0.72-1.17]
Q61	1,080	46,373,710	0.02	0.02	0.02	0.00	0.00	0.00	1.05 [0.86-1.29]	1.00 [0.82-1.23]
Q62	1,130	46,229,470	0.02	0.02	0.02	0.00	0.00	0.00	0.99 [0.81-1.21]	0.97 [0.79-1.19]
Q63	2,050	46,337,060	0.05	0.04	0.04	0.01	0.01	0.01	1.16 [1.00-1.34]	1.12 [0.96-1.30]
Q64	810	46,373,830	0.02	0.02	0.02	0.00	0.00	0.00	1.06 [0.84-1.33]	1.02 [0.80-1.29]
Q65	2,750	46,268,650	0.05	0.06	0.06	−0.01	−0.01	−0.01	0.89 [0.78-1.02]	0.86 [0.75-0.99]
Q66	3,680	45,607,920	0.09	0.08	0.08	0.01	0.01	0.01	1.12 [1.01-1.25]	1.08 [0.97-1.21]
Q67	2,600	46,332,630	0.06	0.05	0.06	0.01	0.00	0.00	1.08 [0.95-1.23]	1.01 [0.89-1.16]
Q68	900	46,381,530	0.02	0.02	0.02	0.00	0.00	0.00	1.11 [0.90-1.39]	1.06 [0.85-1.33]
Q69	600	46,312,180	0.01	0.01	0.01	0.00	0.00	0.00	0.91 [0.69-1.20]	0.89 [0.67-1.18]
Q74	2,690	46,351,650	0.07	0.06	0.06	0.01	0.01	0.01	1.17 [1.03-1.33]	1.15 [1.01-1.30]
Q75	1,850	46,333,260	0.04	0.04	0.04	0.00	0.00	0.00	1.00 [0.86-1.17]	0.97 [0.83-1.13]
Q76	1,150	46,386,030	0.02	0.02	0.03	0.00	0.00	0.00	0.91 [0.75-1.12]	0.87 [0.71-1.07]
Q78	910	46,399,590	0.02	0.02	0.02	0.00	0.00	0.00	1.06 [0.85-1.32]	1.03 [0.82-1.29]
Q79	1,780	46,360,580	0.05	0.04	0.04	0.01	0.01	0.00	1.24 [1.06-1.45]*	1.12 [0.95-1.32]
Q82	1,940	45,727,890	0.05	0.04	0.04	0.01	0.01	0.01	1.19 [1.03-1.38]	1.16 [1.00-1.34]
Q83	800	46,401,820	0.02	0.02	0.02	0.00	0.00	0.00	0.97 [0.76-1.25]	0.93 [0.73-1.20]
Q85	1,180	46,395,920	0.02	0.03	0.03	0.00	0.00	0.00	0.96 [0.79-1.17]	0.94 [0.76-1.15]
Q87	2,310	46,357,240	0.05	0.05	0.05	0.00	0.00	0.00	1.05 [0.91-1.21]	1.02 [0.88-1.17]
Q89	2,050	46,368,770	0.05	0.04	0.04	0.00	0.00	0.00	1.02 [0.88-1.18]	1.01 [0.87-1.17]
Q90	810	46,348,380	0.02	0.02	0.02	0.00	0.00	0.00	1.03 [0.81-1.31]	1.00 [0.79-1.29]
Q92	510	46,410,650	0.01	0.01	0.01	0.00	0.00	0.00	0.98 [0.73-1.31]	0.95 [0.71-1.29]
Q93	1,560	46,394,160	0.04	0.03	0.03	0.01	0.01	0.00	1.19 [1.01-1.41]	1.12 [0.95-1.32]
Q99	1,650	46,392,600	0.04	0.03	0.04	0.01	0.00	0.00	1.12 [0.95-1.31]	1.07 [0.91-1.26]
<18 - R00-R99										
R00	30,990	46,118,240	0.73	0.66	0.64	0.08	0.09	0.07	1.14 [1.10-1.18]*	1.10 [1.06-1.15]*
R01	9,310	46,076,890	0.24	0.19	0.19	0.04	0.04	0.03	1.21 [1.13-1.29]*	1.16 [1.09-1.24]*
R02	1,690	46,401,560	0.04	0.04	0.04	0.00	0.00	0.00	1.08 [0.92-1.28]	1.07 [0.91-1.27]
R03	7,750	46,354,640	0.17	0.17	0.15	0.01	0.02	0.02	1.13 [1.04-1.22]*	1.10 [1.02-1.20]
R04	12,720	46,211,430	0.32	0.26	0.26	0.06	0.06	0.05	1.24 [1.17-1.31]*	1.19 [1.12-1.26]*
R05	25,410	45,768,050	0.70	0.51	0.51	0.19	0.19	0.16	1.37 [1.32-1.43]*	1.30 [1.25-1.35]*
R06	111,440	43,508,850	3.86	2.21	2.20	1.65	1.66	1.58	1.76 [1.72-1.79]*	1.70 [1.67-1.73]*
R07	33,900	46,143,470	0.78	0.72	0.68	0.06	0.10	0.07	1.14 [1.10-1.18]*	1.10 [1.06-1.15]*
R09	3,660	46,360,440	0.09	0.08	0.07	0.01	0.02	0.01	1.21 [1.08-1.34]*	1.15 [1.03-1.29]
R10	155,120	44,478,720	3.89	3.38	3.28	0.52	0.61	0.48	1.19 [1.17-1.21]*	1.14 [1.12-1.16]*
R11	90,830	44,502,810	2.29	1.97	1.96	0.32	0.33	0.26	1.17 [1.14-1.19]*	1.13 [1.10-1.15]*
R12	1,560	46,406,290	0.03	0.03	0.03	0.00	0.00	0.00	1.08 [0.90-1.28]	1.00 [0.83-1.20]
R13	5,820	46,360,480	0.14	0.12	0.12	0.02	0.02	0.02	1.21 [1.11-1.32]*	1.12 [1.02-1.23]
R14	3,820	46,321,680	0.09	0.08	0.08	0.01	0.01	0.01	1.16 [1.04-1.30]*	1.11 [0.99-1.24]
R15	2,370	46,392,990	0.06	0.05	0.05	0.01	0.01	0.00	1.14 [0.99-1.31]	1.09 [0.95-1.25]
R16	3,680	46,364,740	0.08	0.08	0.08	0.01	0.01	0.01	1.11 [0.99-1.24]	1.08 [0.96-1.21]
R17	1,550	46,354,710	0.04	0.03	0.03	0.00	0.00	0.00	1.12 [0.94-1.33]	1.13 [0.95-1.34]
R18	2,190	46,395,170	0.04	0.05	0.05	0.00	0.00	0.00	0.94 [0.81-1.10]	0.93 [0.80-1.09]
R19	15,580	46,235,200	0.37	0.33	0.31	0.04	0.06	0.05	1.19 [1.13-1.26]*	1.14 [1.08-1.20]*
R20	7,360	46,360,970	0.17	0.16	0.15	0.01	0.02	0.01	1.15 [1.06-1.24]*	1.09 [1.00-1.18]

R21	36,580	45,262,870	0.91	0.78	0.78	0.13	0.14	0.11	1.17 [1.14-1.21]*	1.13 [1.10-1.17]*
R22	9,420	46,226,520	0.26	0.19	0.18	0.08	0.08	0.07	1.42 [1.33-1.52]*	1.36 [1.27-1.45]*
R23	14,500	45,984,010	0.36	0.30	0.31	0.05	0.05	0.04	1.16 [1.10-1.22]*	1.12 [1.06-1.18]*
R25	5,600	46,109,690	0.13	0.12	0.12	0.01	0.01	0.01	1.10 [1.01-1.20]	1.06 [0.97-1.16]
R26	10,160	46,302,530	0.24	0.21	0.22	0.03	0.02	0.02	1.10 [1.03-1.17]*	1.07 [1.00-1.15]
R27	2,550	46,384,800	0.06	0.05	0.05	0.01	0.01	0.01	1.17 [1.03-1.33]	1.12 [0.98-1.28]
R29	6,690	45,882,410	0.15	0.14	0.14	0.01	0.01	0.00	1.07 [0.99-1.17]	1.02 [0.94-1.11]
R30	5,820	46,354,070	0.14	0.12	0.12	0.02	0.02	0.02	1.18 [1.08-1.29]*	1.14 [1.05-1.25]*
R31	9,870	46,294,670	0.23	0.21	0.20	0.02	0.03	0.02	1.14 [1.06-1.22]*	1.09 [1.02-1.17]
R32	6,830	46,350,530	0.17	0.14	0.15	0.03	0.02	0.02	1.15 [1.07-1.25]*	1.10 [1.02-1.20]
R33	7,920	46,333,920	0.18	0.17	0.16	0.02	0.03	0.02	1.16 [1.08-1.25]*	1.10 [1.02-1.19]
R34	680	46,407,710	0.01	0.01	0.02	0.00	0.00	0.00	0.95 [0.73-1.23]	0.92 [0.71-1.21]
R35	4,290	46,366,990	0.11	0.09	0.09	0.02	0.02	0.02	1.22 [1.10-1.35]*	1.19 [1.07-1.32]*
R39	4,040	46,357,980	0.10	0.08	0.08	0.02	0.02	0.02	1.31 [1.19-1.45]*	1.25 [1.13-1.39]*
R40	4,860	46,346,960	0.11	0.10	0.11	0.01	0.01	0.00	1.05 [0.95-1.16]	1.02 [0.92-1.13]
R41	7,630	46,334,260	0.18	0.16	0.16	0.02	0.01	0.01	1.09 [1.01-1.18]	1.04 [0.96-1.13]
R42	12,210	46,318,570	0.29	0.26	0.26	0.03	0.03	0.02	1.11 [1.05-1.18]*	1.07 [1.01-1.14]
R44	4,580	46,383,030	0.11	0.10	0.10	0.02	0.01	0.01	1.14 [1.03-1.26]*	1.10 [1.00-1.22]
R45	20,300	46,278,510	0.49	0.42	0.46	0.07	0.03	0.02	1.06 [1.02-1.12]*	1.04 [0.99-1.10]
R46	3,120	46,386,690	0.07	0.07	0.07	0.01	0.00	0.00	1.06 [0.94-1.19]	1.00 [0.89-1.14]
R47	3,190	46,387,090	0.07	0.07	0.07	0.00	0.01	0.00	1.09 [0.97-1.22]	1.04 [0.92-1.18]
R48	1,200	46,409,960	0.03	0.02	0.03	0.01	0.00	0.00	1.16 [0.96-1.40]	1.09 [0.90-1.32]
R49	1,440	46,394,180	0.04	0.03	0.03	0.01	0.01	0.01	1.25 [1.05-1.48]	1.20 [1.01-1.42]
R50	57,030	45,017,920	1.42	1.22	1.24	0.20	0.19	0.15	1.15 [1.12-1.18]*	1.12 [1.09-1.15]*
R51	47,230	45,915,700	1.17	0.99	0.97	0.18	0.20	0.15	1.20 [1.17-1.24]*	1.15 [1.12-1.19]*
R52	2,700	46,394,350	0.06	0.06	0.06	0.00	0.01	0.00	1.12 [0.98-1.28]	1.06 [0.93-1.22]
R53	13,740	46,207,110	0.35	0.28	0.28	0.06	0.06	0.05	1.22 [1.16-1.29]*	1.18 [1.11-1.25]*
R55	23,120	46,162,510	0.54	0.49	0.49	0.04	0.05	0.03	1.10 [1.05-1.14]*	1.05 [1.01-1.10]
R56	47,630	44,996,960	1.05	1.06	1.03	−0.01	0.02	0.01	1.02 [0.99-1.05]	1.01 [0.98-1.04]
R57	820	46,406,860	0.02	0.02	0.02	0.00	0.00	0.00	1.01 [0.80-1.29]	1.03 [0.80-1.32]
R59	13,730	46,204,070	0.36	0.28	0.28	0.08	0.08	0.07	1.29 [1.22-1.36]*	1.25 [1.18-1.32]*
R60	2,030	46,391,110	0.05	0.04	0.04	0.01	0.01	0.01	1.21 [1.05-1.40]	1.16 [1.00-1.35]
R61	1,920	46,396,990	0.05	0.04	0.04	0.01	0.01	0.01	1.24 [1.07-1.45]*	1.18 [1.01-1.37]
R62	15,210	45,834,030	0.34	0.33	0.33	0.02	0.02	0.01	1.06 [1.00-1.12]	1.02 [0.96-1.07]
R63	26,750	45,601,210	0.66	0.57	0.57	0.09	0.08	0.06	1.14 [1.10-1.19]*	1.11 [1.06-1.15]*
R65	530	46,415,020	0.01	0.01	0.01	0.00	0.00	0.00	1.21 [0.90-1.63]	1.20 [0.88-1.63]
R68	2,700	46,173,350	0.06	0.06	0.06	0.00	0.00	0.00	1.03 [0.91-1.17]	1.01 [0.89-1.15]
R69	20,610	42,786,150	0.52	0.47	0.43	0.05	0.09	0.07	1.20 [1.15-1.26]*	1.17 [1.11-1.22]*
R72	870	46,407,240	0.02	0.02	0.02	0.00	0.00	0.00	1.22 [0.97-1.52]	1.16 [0.92-1.46]
R73	1,950	46,379,520	0.05	0.04	0.04	0.01	0.01	0.01	1.20 [1.04-1.40]	1.13 [0.97-1.32]
R74	1,580	46,399,680	0.04	0.03	0.03	0.01	0.01	0.00	1.24 [1.05-1.46]	1.14 [0.96-1.36]
R76	740	46,410,740	0.02	0.01	0.01	0.00	0.00	0.00	1.29 [1.02-1.64]	1.24 [0.97-1.59]
R77	690	46,408,180	0.02	0.01	0.02	0.00	0.00	0.00	1.12 [0.87-1.45]	1.12 [0.86-1.46]
R79	6,350	46,329,520	0.15	0.13	0.13	0.02	0.02	0.02	1.18 [1.09-1.28]*	1.14 [1.05-1.25]*
R80	1,460	46,398,560	0.04	0.03	0.03	0.01	0.01	0.01	1.18 [0.99-1.40]	1.17 [0.98-1.40]
R81	470	46,409,460	0.01	0.01	0.01	0.00	0.00	0.00	1.24 [0.92-1.67]	1.24 [0.91-1.69]
R82	2,430	46,382,320	0.06	0.05	0.05	0.01	0.01	0.01	1.18 [1.03-1.35]	1.15 [1.00-1.32]
R84	970	46,408,040	0.02	0.02	0.02	0.00	0.00	0.00	1.05 [0.84-1.30]	0.99 [0.80-1.24]
R87	1,030	46,409,240	0.02	0.02	0.02	0.00	0.00	0.00	1.09 [0.87-1.37]	1.08 [0.86-1.37]
R89	350	46,413,860	0.01	0.01	0.01	0.00	0.00	0.00	1.53 [1.10-2.14]	1.40 [0.99-1.97]
R90	990	46,402,320	0.02	0.02	0.02	0.00	0.00	0.00	1.17 [0.95-1.45]	1.14 [0.92-1.41]
R91	1,520	46,387,970	0.04	0.03	0.03	0.01	0.01	0.01	1.29 [1.09-1.53]*	1.26 [1.06-1.50]*
R93	4,080	46,335,540	0.09	0.09	0.09	0.01	0.01	0.00	1.07 [0.96-1.19]	1.04 [0.93-1.16]
R94	9,160	46,320,910	0.22	0.19	0.19	0.02	0.03	0.02	1.16 [1.08-1.24]*	1.12 [1.04-1.21]*
<18 - S00-T88										
S00	31,040	45,843,480	0.66	0.68	0.68	−0.02	−0.02	−0.03	0.97 [0.93-1.01]	0.95 [0.92-0.99]
S01	40,950	45,768,950	0.86	0.90	0.89	−0.04	−0.02	−0.03	0.97 [0.94-1.01]	0.96 [0.93-1.00]
S02	23,260	46,081,850	0.49	0.51	0.48	−0.02	0.01	0.00	1.02 [0.98-1.07]	1.01 [0.96-1.06]
S03	1,500	46,399,700	0.03	0.03	0.03	0.00	0.00	0.00	1.04 [0.88-1.23]	1.02 [0.85-1.21]
S05	2,620	46,376,700	0.05	0.06	0.06	0.00	0.00	0.00	0.95 [0.83-1.09]	0.93 [0.81-1.07]
S06	6,820	46,304,130	0.15	0.15	0.14	0.00	0.01	0.01	1.05 [0.97-1.14]	1.04 [0.95-1.13]
S09	28,170	45,654,460	0.63	0.61	0.59	0.01	0.03	0.02	1.05 [1.01-1.10]*	1.04 [0.99-1.08]
S10	1,500	46,400,010	0.03	0.03	0.03	0.00	0.00	0.00	0.96 [0.81-1.15]	0.96 [0.80-1.15]
S11	810	46,409,930	0.02	0.02	0.02	0.00	0.00	0.00	1.05 [0.82-1.33]	1.01 [0.79-1.30]
S12	740	46,411,490	0.01	0.02	0.01	0.00	0.00	0.00	1.00 [0.77-1.29]	1.01 [0.77-1.32]
S13	730	46,408,810	0.02	0.02	0.02	0.00	0.00	0.00	1.01 [0.79-1.30]	0.98 [0.76-1.27]
S19	990	46,404,100	0.02	0.02	0.02	0.00	0.00	0.00	1.10 [0.89-1.36]	1.06 [0.86-1.32]
S20	2,120	46,391,390	0.04	0.05	0.05	−0.01	0.00	−0.01	0.90 [0.77-1.05]	0.89 [0.76-1.04]
S21	1,300	46,405,640	0.02	0.03	0.03	0.00	0.00	−0.01	0.85 [0.70-1.04]	0.83 [0.67-1.02]
S22	2,630	46,390,750	0.05	0.06	0.05	0.00	0.00	0.00	1.05 [0.91-1.20]	1.05 [0.91-1.21]
S27	2,720	46,396,010	0.06	0.06	0.06	0.00	0.00	0.00	1.02 [0.89-1.16]	1.02 [0.89-1.18]
S30	6,800	46,322,450	0.14	0.15	0.15	0.00	0.00	−0.01	0.97 [0.89-1.05]	0.95 [0.87-1.03]
S31	4,460	46,357,270	0.10	0.10	0.09	0.00	0.00	0.00	1.03 [0.93-1.15]	1.03 [0.93-1.14]
S32	3,030	46,388,820	0.06	0.07	0.06	−0.01	0.00	0.00	0.95 [0.84-1.08]	0.95 [0.83-1.08]
S36	2,200	46,396,890	0.05	0.05	0.05	0.00	0.00	0.00	0.99 [0.85-1.15]	1.00 [0.86-1.16]
S37	1,090	46,406,280	0.02	0.02	0.02	0.00	0.00	0.00	0.99 [0.80-1.22]	1.00 [0.80-1.24]
S39	2,950	46,374,070	0.07	0.06	0.06	0.01	0.01	0.01	1.18 [1.04-1.33]*	1.13 [0.99-1.28]
S40	3,090	46,380,130	0.06	0.07	0.07	0.00	−0.01	−0.01	0.92 [0.81-1.04]	0.90 [0.79-1.02]
S41	2,180	46,394,730	0.05	0.05	0.05	0.00	0.00	0.00	0.98 [0.85-1.14]	0.98 [0.84-1.14]
S42	20,750	46,090,680	0.47	0.44	0.45	0.03	0.02	0.02	1.05 [1.00-1.10]	1.04 [0.99-1.09]
S43	1,080	46,408,230	0.02	0.02	0.02	0.00	0.00	0.00	1.02 [0.82-1.26]	1.01 [0.81-1.26]
S49	610	46,410,090	0.01	0.01	0.01	0.00	0.00	0.00	1.17 [0.89-1.53]	1.14 [0.86-1.51]
S50	4,550	46,370,800	0.10	0.10	0.10	0.00	−0.01	−0.01	0.93 [0.84-1.03]	0.90 [0.81-1.00]
S51	6,390	46,357,540	0.13	0.14	0.14	0.00	0.00	0.00	0.98 [0.90-1.07]	0.97 [0.89-1.06]
S52	54,280	45,503,840	1.22	1.19	1.21	0.04	0.01	0.00	1.01 [0.98-1.04]	1.00 [0.97-1.03]
S53	1,750	46,389,450	0.04	0.04	0.04	0.00	0.00	0.00	0.96 [0.82-1.13]	0.96 [0.81-1.13]
S54	610	46,411,580	0.01	0.01	0.01	0.00	0.00	0.00	1.00 [0.75-1.33]	1.00 [0.75-1.34]
S56	2,010	46,399,110	0.04	0.04	0.04	0.00	0.00	0.00	0.97 [0.83-1.14]	0.96 [0.82-1.14]
S59	580	46,408,360	0.01	0.01	0.01	0.00	0.00	0.00	1.02 [0.77-1.35]	0.95 [0.71-1.27]
S60	4,580	46,366,140	0.10	0.10	0.10	0.00	0.00	0.00	1.00 [0.91-1.11]	0.99 [0.89-1.10]
S61	30,870	46,031,600	0.64	0.68	0.66	−0.04	−0.03	−0.03	0.96 [0.92-1.00]	0.96 [0.92-1.00]
S62	23,030	46,159,790	0.49	0.50	0.49	−0.01	0.00	0.00	1.01 [0.97-1.06]	1.00 [0.95-1.04]
S63	2,310	46,392,870	0.05	0.05	0.05	0.00	0.01	0.00	1.14 [0.99-1.31]	1.10 [0.95-1.27]
S64	4,030	46,373,980	0.08	0.09	0.08	−0.01	0.00	0.00	0.94 [0.84-1.05]	0.94 [0.84-1.06]
S65	1,060	46,406,780	0.02	0.02	0.02	0.00	0.00	0.00	1.03 [0.83-1.27]	1.04 [0.83-1.30]
S66	7,300	46,339,330	0.15	0.16	0.15	−0.01	0.00	0.00	0.99 [0.91-1.07]	0.98 [0.90-1.07]
S67	1,790	46,383,930	0.04	0.04	0.04	0.00	0.00	0.00	0.92 [0.79-1.09]	0.92 [0.78-1.09]
S68	3,160	46,362,860	0.07	0.07	0.07	0.00	0.00	0.00	0.99 [0.88-1.12]	0.98 [0.87-1.11]
S69	2,140	46,389,480	0.05	0.05	0.05	0.00	0.00	0.00	1.02 [0.88-1.18]	1.01 [0.87-1.17]
S70	2,750	46,384,640	0.06	0.06	0.06	0.00	0.00	0.00	0.94 [0.82-1.07]	0.94 [0.82-1.07]
S71	2,670	46,390,560	0.06	0.06	0.06	0.00	0.00	0.00	0.99 [0.86-1.13]	0.95 [0.82-1.09]
S72	5,860	46,300,920	0.12	0.13	0.12	−0.01	0.00	0.00	0.98 [0.90-1.07]	0.97 [0.89-1.06]
S76	710	46,412,370	0.02	0.01	0.02	0.00	0.00	0.00	1.09 [0.84-1.40]	1.05 [0.80-1.36]
S79	760	46,408,100	0.02	0.02	0.01	0.00	0.00	0.00	1.17 [0.92-1.49]	1.21 [0.94-1.55]
S80	5,830	46,344,620	0.12	0.13	0.13	−0.01	−0.01	−0.01	0.93 [0.85-1.02]	0.91 [0.83-0.99]
S81	5,340	46,352,400	0.12	0.11	0.12	0.01	0.01	0.00	1.06 [0.97-1.17]	1.04 [0.95-1.14]
S82	23,290	46,089,130	0.51	0.51	0.49	0.00	0.02	0.01	1.04 [0.99-1.08]	1.02 [0.97-1.07]

S83	4,140	46,379,270	0.09	0.09	0.08	0.00	0.01	0.01	1.11 [1.00-1.24]	1.07 [0.96-1.19]
S86	870	46,409,480	0.02	0.02	0.02	0.00	0.00	0.00	1.04 [0.82-1.31]	1.01 [0.79-1.28]
S89	1,300	46,401,060	0.03	0.03	0.03	0.00	0.00	0.00	1.05 [0.87-1.26]	1.03 [0.85-1.25]
S90	2,310	46,385,620	0.05	0.05	0.05	0.00	0.00	0.00	1.02 [0.88-1.17]	1.01 [0.88-1.17]
S91	4,910	46,345,950	0.11	0.11	0.11	0.00	0.00	0.00	0.99 [0.90-1.09]	0.99 [0.89-1.09]
S92	4,000	46,373,850	0.08	0.09	0.08	−0.01	0.00	0.00	0.99 [0.88-1.10]	0.97 [0.86-1.08]
S93	1,250	46,405,770	0.03	0.03	0.03	0.00	0.01	0.00	1.20 [1.00-1.45]	1.16 [0.96-1.41]
S96	460	46,412,210	0.01	0.01	0.01	0.00	0.00	0.00	1.22 [0.90-1.65]	1.21 [0.88-1.65]
S99	970	46,405,130	0.02	0.02	0.02	0.00	0.00	0.00	1.17 [0.94-1.45]	1.14 [0.91-1.42]
T00	2,150	46,391,690	0.05	0.05	0.05	0.00	0.00	0.00	0.98 [0.84-1.13]	0.98 [0.84-1.14]
T01	1,180	46,409,690	0.03	0.02	0.03	0.00	0.00	0.00	1.07 [0.87-1.30]	1.05 [0.86-1.29]
T02	780	46,409,320	0.02	0.02	0.02	0.00	0.00	0.00	0.97 [0.76-1.24]	0.94 [0.73-1.20]
T11	1,230	46,403,130	0.03	0.03	0.03	0.00	0.00	0.00	0.95 [0.78-1.16]	0.93 [0.76-1.15]
T13	660	46,407,050	0.01	0.01	0.01	0.00	0.00	0.00	0.83 [0.63-1.10]	0.83 [0.62-1.10]
T14	920	46,390,750	0.02	0.02	0.02	0.00	0.00	0.00	0.89 [0.71-1.13]	0.86 [0.68-1.09]
T15	510	46,408,110	0.01	0.01	0.01	0.00	0.00	0.00	1.00 [0.74-1.35]	0.98 [0.72-1.32]
T16	8,310	46,276,250	0.19	0.18	0.18	0.01	0.01	0.01	1.05 [0.97-1.12]	1.03 [0.96-1.11]
T17	7,190	46,262,300	0.15	0.16	0.16	0.00	0.00	−0.01	0.99 [0.91-1.07]	0.97 [0.89-1.05]
T18	4,880	46,333,000	0.11	0.10	0.11	0.00	0.00	0.00	1.01 [0.91-1.11]	0.98 [0.89-1.08]
T20	2,960	46,348,910	0.06	0.07	0.06	−0.01	−0.01	−0.01	0.90 [0.80-1.02]	0.90 [0.79-1.02]
T21	3,960	46,322,580	0.08	0.09	0.09	−0.01	−0.01	−0.01	0.91 [0.82-1.01]	0.91 [0.81-1.01]
T22	3,380	46,342,080	0.07	0.07	0.07	−0.01	0.00	0.00	0.94 [0.84-1.06]	0.94 [0.84-1.06]
T23	3,600	46,342,650	0.08	0.08	0.08	0.00	0.00	0.00	0.97 [0.87-1.09]	0.95 [0.85-1.07]
T24	2,310	46,367,560	0.05	0.05	0.05	0.00	−0.01	0.00	0.90 [0.78-1.04]	0.91 [0.79-1.05]
T25	1,480	46,385,470	0.03	0.03	0.03	0.00	0.00	0.00	0.92 [0.77-1.10]	0.90 [0.75-1.08]
T29	590	46,404,510	0.01	0.01	0.01	0.00	0.00	0.00	0.81 [0.61-1.08]	0.80 [0.60-1.07]
T31	9,970	46,212,710	0.20	0.22	0.22	−0.02	−0.02	−0.02	0.92 [0.86-0.99]	0.92 [0.86-0.98]
T36	1,850	46,392,570	0.04	0.04	0.04	0.00	0.01	0.01	1.18 [1.01-1.38]	1.17 [1.00-1.37]
T37	580	46,409,190	0.01	0.01	0.01	0.00	0.00	0.00	1.14 [0.86-1.50]	1.17 [0.88-1.55]
T38	1,840	46,394,820	0.04	0.04	0.04	0.00	0.00	0.00	1.05 [0.89-1.22]	1.02 [0.87-1.20]
T39	35,760	46,003,750	0.78	0.78	0.77	0.01	0.02	0.01	1.02 [0.98-1.06]	1.01 [0.97-1.05]
T40	9,850	46,326,630	0.20	0.22	0.21	−0.01	−0.01	0.00	0.97 [0.90-1.04]	0.98 [0.91-1.05]
T42	5,430	46,358,520	0.11	0.12	0.11	−0.01	0.00	0.00	0.96 [0.87-1.05]	0.98 [0.88-1.08]
T43	13,380	46,284,310	0.28	0.29	0.28	−0.02	−0.01	−0.01	0.97 [0.91-1.03]	0.98 [0.92-1.04]
T44	2,770	46,383,170	0.06	0.06	0.06	0.00	0.00	0.00	1.01 [0.89-1.15]	1.00 [0.88-1.15]
T45	4,250	46,363,010	0.10	0.09	0.09	0.02	0.02	0.01	1.18 [1.07-1.31]*	1.14 [1.03-1.27]
T46	1,240	46,397,710	0.03	0.03	0.03	0.00	0.00	0.00	1.00 [0.83-1.21]	0.98 [0.81-1.19]
T47	1,380	46,402,500	0.03	0.03	0.03	0.00	0.00	0.00	0.95 [0.79-1.15]	0.94 [0.77-1.14]
T48	1,280	46,389,260	0.03	0.03	0.03	0.00	0.00	0.00	1.05 [0.87-1.26]	1.01 [0.83-1.22]
T49	1,210	46,389,840	0.02	0.03	0.03	0.00	0.00	0.00	0.95 [0.79-1.16]	0.92 [0.76-1.12]
T50	2,970	46,372,590	0.06	0.07	0.06	−0.01	0.00	0.00	0.96 [0.85-1.09]	0.95 [0.83-1.08]
T51	9,260	46,327,950	0.18	0.20	0.19	−0.02	0.00	0.00	0.98 [0.91-1.05]	1.00 [0.93-1.08]
T52	1,260	46,384,930	0.02	0.03	0.03	−0.01	0.00	0.00	0.83 [0.68-1.01]	0.83 [0.68-1.01]
T54	1,060	46,403,010	0.02	0.02	0.03	0.00	0.00	0.00	0.86 [0.70-1.07]	0.85 [0.68-1.06]
T59	500	46,405,480	0.01	0.01	0.01	0.00	0.00	0.00	1.04 [0.77-1.41]	1.05 [0.77-1.43]
T62	500	46,405,960	0.01	0.01	0.01	0.00	0.00	0.00	1.09 [0.82-1.46]	1.06 [0.79-1.43]
T65	1,050	46,394,280	0.02	0.02	0.02	0.00	0.00	0.00	0.99 [0.80-1.21]	0.97 [0.78-1.19]
T74	1,040	46,394,520	0.02	0.02	0.02	0.00	0.00	0.00	0.85 [0.68-1.06]	0.82 [0.66-1.03]
T75	610	46,404,280	0.01	0.01	0.01	0.00	0.00	0.00	1.01 [0.76-1.32]	0.97 [0.73-1.28]
T78	12,590	46,119,040	0.71	0.15	0.15	0.56	0.57	0.53	4.81 [4.57-5.06]*	3.86 [3.66-4.07]*
T79	2,030	46,388,240	0.04	0.04	0.04	0.00	0.00	0.00	1.05 [0.90-1.22]	1.03 [0.89-1.21]
T80	1,160	46,390,000	0.03	0.02	0.03	0.00	0.00	0.00	1.09 [0.90-1.33]	1.08 [0.89-1.32]
T81	26,500	46,025,840	0.66	0.55	0.54	0.11	0.12	0.10	1.22 [1.17-1.27]*	1.17 [1.12-1.22]*
T82	4,550	46,338,940	0.10	0.10	0.10	0.00	0.00	0.00	1.01 [0.91-1.11]	0.99 [0.89-1.09]
T83	1,840	46,400,870	0.04	0.04	0.04	0.00	0.00	0.00	1.02 [0.87-1.20]	0.96 [0.81-1.14]
T84	5,300	46,367,010	0.12	0.11	0.11	0.01	0.01	0.01	1.14 [1.04-1.25]*	1.11 [1.01-1.22]
T85	6,280	46,316,080	0.15	0.13	0.13	0.02	0.02	0.02	1.17 [1.08-1.27]*	1.12 [1.03-1.22]
T86	940	46,406,040	0.02	0.02	0.02	0.00	0.00	0.00	1.02 [0.82-1.28]	1.01 [0.81-1.27]
T88	2,250	46,354,530	0.06	0.04	0.04	0.02	0.02	0.01	1.40 [1.23-1.60]*	1.29 [1.13-1.48]*
T90	3,150	46,378,010	0.07	0.07	0.06	0.01	0.01	0.01	1.15 [1.02-1.29]	1.13 [1.00-1.27]
T91	540	46,412,570	0.01	0.01	0.01	0.00	0.00	0.00	1.07 [0.79-1.43]	1.08 [0.79-1.46]
T92	1,840	46,393,390	0.04	0.04	0.04	0.00	0.00	0.00	1.07 [0.91-1.26]	1.04 [0.88-1.23]
T93	2,520	46,389,440	0.05	0.05	0.05	0.00	0.00	0.00	1.10 [0.96-1.26]	1.05 [0.91-1.21]
T95	580	46,406,960	0.01	0.01	0.01	0.00	0.00	0.00	0.80 [0.60-1.07]	0.82 [0.61-1.11]
<18 - U00-U89										
U07	3,600	46,412,830	0.08	0.08	0.07	0.01	0.01	0.01	1.14 [1.02-1.29]	1.09 [0.96-1.23]
U80	1,500	46,390,800	0.04	0.03	0.03	0.01	0.01	0.01	1.41 [1.20-1.65]*	1.33 [1.12-1.57]*
U82	3,720	46,397,420	0.10	0.07	0.08	0.02	0.02	0.02	1.30 [1.17-1.45]*	1.26 [1.13-1.41]*
U83	2,190	46,406,430	0.06	0.04	0.05	0.01	0.01	0.01	1.22 [1.06-1.41]*	1.17 [1.01-1.35]
U89	480	46,410,560	0.01	0.01	0.01	0.00	0.00	0.00	1.33 [1.00-1.77]	1.28 [0.96-1.72]
<18 - V00-Y99										
V03	6,080	46,309,040	0.12	0.13	0.13	−0.01	−0.01	−0.01	0.93 [0.85-1.02]	0.93 [0.85-1.02]
V09	910	46,404,240	0.02	0.02	0.02	0.00	0.00	0.00	1.12 [0.90-1.39]	1.08 [0.87-1.35]
V13	1,430	46,396,740	0.03	0.03	0.03	0.00	0.00	0.00	0.94 [0.78-1.13]	0.97 [0.80-1.17]
V17	740	46,408,690	0.02	0.02	0.02	0.00	0.00	0.00	0.97 [0.75-1.25]	0.96 [0.75-1.24]
V18	12,720	46,208,550	0.28	0.27	0.27	0.01	0.01	0.00	1.03 [0.97-1.09]	1.00 [0.94-1.07]
V19	1,880	46,390,410	0.04	0.04	0.04	0.00	0.00	0.00	1.02 [0.87-1.19]	1.01 [0.87-1.19]
V23	1,890	46,402,640	0.04	0.04	0.04	−0.01	0.00	0.00	0.93 [0.79-1.10]	0.95 [0.80-1.13]
V27	570	46,412,740	0.01	0.01	0.01	0.00	0.00	0.00	0.91 [0.67-1.23]	0.89 [0.65-1.22]
V28	2,710	46,389,470	0.05	0.06	0.05	−0.01	0.00	0.00	1.01 [0.88-1.15]	0.98 [0.85-1.12]
V29	660	46,410,560	0.01	0.01	0.01	0.00	0.00	0.00	0.91 [0.69-1.20]	0.91 [0.69-1.21]
V43	2,640	46,386,510	0.05	0.06	0.05	0.00	0.00	0.00	1.04 [0.91-1.19]	1.02 [0.88-1.17]
V47	1,670	46,401,250	0.04	0.04	0.03	0.00	0.00	0.00	1.04 [0.88-1.23]	1.02 [0.86-1.22]
V48	1,300	46,402,250	0.03	0.03	0.02	0.00	0.00	0.00	1.13 [0.94-1.36]	1.12 [0.92-1.36]
V49	1,300	46,399,510	0.03	0.03	0.03	0.00	0.00	0.00	1.06 [0.88-1.28]	1.06 [0.87-1.29]
V80	2,920	46,378,460	0.06	0.06	0.06	0.00	0.00	0.00	1.03 [0.90-1.17]	1.01 [0.88-1.15]
V86	920	46,409,230	0.02	0.02	0.02	0.00	0.00	0.00	1.14 [0.91-1.42]	1.10 [0.87-1.38]
V89	610	46,404,490	0.01	0.01	0.01	0.00	0.00	0.00	0.90 [0.67-1.20]	0.85 [0.63-1.15]
W00	820	46,407,680	0.02	0.02	0.02	0.00	0.00	0.00	1.19 [0.94-1.50]	1.19 [0.93-1.51]
W01	23,640	46,019,140	0.53	0.51	0.51	0.02	0.02	0.01	1.03 [0.99-1.08]	1.02 [0.97-1.06]
W02	6,950	46,315,910	0.16	0.15	0.15	0.01	0.00	0.00	1.00 [0.92-1.09]	1.00 [0.92-1.08]
W03	9,120	46,299,380	0.20	0.20	0.20	0.01	0.00	0.00	1.02 [0.95-1.10]	1.00 [0.93-1.07]
W04	1,570	46,307,360	0.03	0.03	0.03	0.00	0.00	0.00	0.97 [0.82-1.15]	0.97 [0.81-1.15]
W06	5,250	46,257,500	0.11	0.11	0.11	0.00	0.00	0.00	1.03 [0.94-1.13]	1.02 [0.93-1.12]
W07	5,040	46,287,490	0.11	0.11	0.11	0.00	0.00	0.00	0.97 [0.88-1.06]	0.96 [0.87-1.06]
W08	3,700	46,322,300	0.07	0.08	0.08	−0.01	−0.01	−0.01	0.89 [0.80-1.00]	0.89 [0.80-1.00]
W09	22,650	46,068,570	0.53	0.48	0.50	0.05	0.03	0.02	1.05 [1.01-1.10]	1.04 [0.99-1.09]
W10	10,850	46,193,640	0.23	0.24	0.23	−0.01	0.00	0.00	1.02 [0.95-1.08]	1.00 [0.94-1.07]
W11	620	46,409,890	0.01	0.01	0.01	0.00	0.00	0.00	1.05 [0.80-1.38]	1.06 [0.80-1.40]
W13	5,180	46,322,050	0.11	0.11	0.11	−0.01	−0.01	−0.01	0.95 [0.87-1.05]	0.94 [0.85-1.04]
W14	2,640	46,368,580	0.06	0.06	0.06	0.00	0.00	0.00	0.95 [0.83-1.08]	0.94 [0.82-1.08]
W17	9,320	46,235,120	0.20	0.20	0.20	0.00	−0.01	−0.01	0.97 [0.91-1.04]	0.95 [0.88-1.02]
W18	14,030	46,200,880	0.32	0.30	0.30	0.02	0.02	0.01	1.06 [1.00-1.12]	1.05 [0.99-1.11]
W19	22,970	46,019,900	0.51	0.50	0.49	0.01	0.02	0.02	1.04 [1.00-1.09]	1.03 [0.98-1.08]
W20	3,410	46,359,720	0.07	0.07	0.07	0.00	0.00	0.00	0.99 [0.88-1.11]	0.96 [0.85-1.08]
W21	5,110	46,348,870	0.12	0.11	0.11	0.01	0.01	0.01	1.12 [1.02-1.23]	1.10 [1.00-1.21]
W22	17,590	46,139,140	0.39	0.38	0.37	0.01	0.02	0.01	1.05 [1.00-1.10]	1.03 [0.98-1.09]

W23	15,360	46,171,690	0.33	0.33	0.33	0.00	0.00	0.00	1.00 [0.95-1.05]	0.99 [0.94-1.05]
W25	6,660	46,323,140	0.13	0.15	0.14	−0.01	−0.01	−0.01	0.95 [0.87-1.03]	0.94 [0.86-1.02]
W26	4,540	46,384,190	0.10	0.10	0.10	0.00	0.00	0.00	1.04 [0.93-1.15]	1.03 [0.93-1.14]
W27	1,590	46,395,240	0.03	0.04	0.03	−0.01	0.00	0.00	0.89 [0.74-1.06]	0.89 [0.74-1.06]
W29	1,090	46,407,250	0.02	0.02	0.02	0.00	0.00	0.00	0.98 [0.79-1.21]	0.98 [0.79-1.23]
W31	1,170	46,406,300	0.03	0.02	0.02	0.00	0.00	0.00	1.17 [0.96-1.42]	1.16 [0.95-1.43]
W34	530	46,411,290	0.01	0.01	0.01	0.00	0.00	0.00	1.04 [0.77-1.41]	1.04 [0.76-1.43]
W44	17,070	46,123,250	0.38	0.37	0.37	0.01	0.01	0.00	1.02 [0.97-1.08]	1.01 [0.96-1.06]
W45	4,220	46,350,020	0.10	0.09	0.09	0.01	0.01	0.00	1.06 [0.96-1.18]	1.05 [0.94-1.16]
W49	980	46,399,910	0.02	0.02	0.02	0.00	0.00	0.00	1.01 [0.81-1.26]	1.01 [0.81-1.26]
W50	8,540	46,310,840	0.19	0.18	0.18	0.01	0.01	0.01	1.08 [1.00-1.16]	1.06 [0.98-1.14]
W51	6,930	46,320,010	0.16	0.15	0.15	0.01	0.01	0.00	1.04 [0.96-1.13]	1.01 [0.93-1.10]
W54	5,490	46,339,890	0.11	0.12	0.12	−0.01	−0.01	−0.02	0.88 [0.80-0.97]*	0.86 [0.78-0.95]*
W55	1,240	46,403,170	0.03	0.03	0.03	0.00	0.00	0.00	0.99 [0.81-1.20]	0.98 [0.80-1.20]
W57	2,140	46,394,660	0.05	0.04	0.04	0.01	0.01	0.01	1.26 [1.09-1.45]*	1.24 [1.08-1.44]*
W79	670	46,403,460	0.02	0.01	0.01	0.01	0.01	0.01	1.96 [1.56-2.47]*	1.68 [1.32-2.14]*
X10	3,280	46,339,990	0.07	0.07	0.07	0.00	0.00	0.00	0.98 [0.87-1.10]	0.98 [0.87-1.10]
X11	870	46,391,750	0.02	0.02	0.02	0.00	0.00	0.00	1.01 [0.81-1.26]	0.99 [0.79-1.24]
X12	1,660	46,368,680	0.03	0.04	0.04	0.00	0.00	−0.01	0.87 [0.74-1.03]	0.87 [0.73-1.03]
X15	1,560	46,386,830	0.03	0.03	0.03	0.00	0.00	0.00	0.99 [0.83-1.17]	0.99 [0.83-1.17]
X40	8,890	46,270,870	0.19	0.19	0.19	−0.01	0.00	0.00	1.00 [0.94-1.08]	1.00 [0.93-1.08]
X41	3,680	46,353,390	0.08	0.08	0.08	0.00	0.00	0.00	0.98 [0.88-1.10]	0.99 [0.88-1.11]
X42	2,200	46,393,440	0.04	0.05	0.05	0.00	0.00	0.00	0.98 [0.85-1.14]	0.98 [0.84-1.14]
X43	780	46,399,740	0.02	0.02	0.02	0.00	0.00	0.00	0.95 [0.75-1.22]	0.92 [0.71-1.18]
X44	5,840	46,282,630	0.13	0.13	0.12	0.00	0.00	0.00	1.03 [0.94-1.12]	1.01 [0.92-1.10]
X45	1,440	46,398,890	0.03	0.03	0.03	0.00	0.00	0.00	0.92 [0.76-1.11]	0.94 [0.78-1.14]
X46	910	46,391,450	0.02	0.02	0.02	0.00	0.00	0.00	0.83 [0.66-1.04]	0.82 [0.65-1.04]
X49	3,310	46,340,160	0.09	0.07	0.07	0.02	0.02	0.02	1.37 [1.23-1.52]*	1.28 [1.14-1.43]*
X50	5,630	46,362,320	0.12	0.12	0.12	0.00	0.01	0.00	1.05 [0.96-1.15]	1.01 [0.92-1.11]
X58	1,720	46,387,930	0.06	0.03	0.03	0.03	0.03	0.03	2.06 [1.78-2.38]*	1.79 [1.54-2.08]*
X59	24,560	45,922,830	0.55	0.53	0.51	0.02	0.04	0.03	1.08 [1.03-1.12]*	1.05 [1.00-1.09]
X60	27,590	46,146,220	0.61	0.60	0.59	0.01	0.01	0.01	1.02 [0.98-1.07]	1.01 [0.97-1.06]
X61	13,160	46,306,860	0.27	0.29	0.28	−0.01	−0.01	0.00	0.97 [0.92-1.04]	0.98 [0.92-1.05]
X62	7,750	46,351,590	0.16	0.17	0.16	−0.01	0.00	0.00	0.98 [0.90-1.06]	0.98 [0.91-1.07]
X63	1,970	46,402,370	0.04	0.04	0.04	0.00	0.00	0.00	1.05 [0.90-1.23]	1.06 [0.90-1.24]
X64	8,140	46,336,520	0.18	0.17	0.17	0.01	0.01	0.01	1.05 [0.98-1.14]	1.03 [0.95-1.11]
X65	7,880	46,347,290	0.16	0.17	0.16	−0.02	0.00	0.00	0.99 [0.92-1.08]	1.02 [0.94-1.11]
X69	1,000	46,409,450	0.02	0.02	0.02	0.00	0.00	0.00	1.03 [0.83-1.27]	1.01 [0.81-1.26]
X70	700	46,413,150	0.01	0.02	0.01	0.00	0.00	0.00	0.98 [0.75-1.27]	1.04 [0.80-1.37]
X78	7,320	46,361,400	0.17	0.16	0.16	0.01	0.00	0.00	1.03 [0.95-1.11]	1.01 [0.93-1.09]
X83	570	46,414,240	0.01	0.01	0.01	0.00	0.00	0.00	0.87 [0.65-1.17]	0.84 [0.62-1.13]
X84	860	46,411,570	0.02	0.02	0.02	0.00	0.00	0.00	0.95 [0.75-1.22]	0.95 [0.74-1.22]
X99	3,030	46,394,160	0.06	0.07	0.06	0.00	0.00	0.00	1.02 [0.90-1.16]	1.02 [0.90-1.17]
Y00	1,070	46,407,070	0.02	0.02	0.02	0.00	0.00	0.00	0.91 [0.73-1.13]	0.89 [0.71-1.11]
Y04	11,900	46,284,710	0.23	0.26	0.23	−0.03	0.00	0.00	1.00 [0.94-1.07]	0.99 [0.93-1.06]
Y07	1,020	46,393,990	0.02	0.02	0.02	0.00	0.00	0.00	0.92 [0.74-1.15]	0.92 [0.74-1.15]
Y09	1,280	46,402,880	0.02	0.03	0.02	0.00	0.00	0.00	1.08 [0.89-1.32]	1.06 [0.86-1.30]
Y40	2,930	46,371,570	0.08	0.06	0.06	0.02	0.02	0.01	1.30 [1.15-1.47]*	1.25 [1.10-1.41]*
Y41	430	46,412,940	0.01	0.01	0.01	0.00	0.00	0.00	1.17 [0.85-1.62]	1.09 [0.78-1.54]
Y42	1,290	46,405,360	0.03	0.03	0.02	0.01	0.01	0.01	1.36 [1.14-1.63]*	1.30 [1.08-1.57]*
Y43	3,240	46,380,350	0.07	0.07	0.07	0.01	0.01	0.01	1.11 [0.99-1.24]	1.08 [0.96-1.22]
Y44	400	46,414,730	0.01	0.01	0.01	0.00	0.00	0.00	1.40 [1.03-1.92]	1.38 [1.00-1.90]
Y45	1,620	46,402,410	0.04	0.03	0.03	0.01	0.01	0.01	1.29 [1.10-1.52]*	1.22 [1.03-1.45]
Y46	440	46,412,890	0.01	0.01	0.01	0.00	0.00	0.00	1.20 [0.88-1.65]	1.16 [0.84-1.60]
Y55	860	46,411,760	0.02	0.02	0.02	0.00	0.01	0.00	1.31 [1.05-1.64]	1.22 [0.96-1.55]
Y57	650	46,407,380	0.02	0.01	0.01	0.01	0.01	0.01	1.84 [1.44-2.33]*	1.65 [1.28-2.13]*
Y59	490	46,399,430	0.02	0.01	0.01	0.01	0.01	0.01	1.62 [1.23-2.13]*	1.50 [1.13-1.99]*
Y60	850	46,410,280	0.02	0.02	0.02	0.00	0.00	0.00	1.10 [0.87-1.39]	1.07 [0.85-1.36]
Y83	32,540	45,969,670	0.81	0.68	0.66	0.13	0.15	0.12	1.22 [1.18-1.27]*	1.17 [1.13-1.22]*
Y84	3,140	46,367,110	0.08	0.07	0.06	0.01	0.01	0.01	1.21 [1.08-1.36]*	1.16 [1.03-1.31]
Y85	980	46,407,380	0.02	0.02	0.02	0.00	0.00	0.00	1.16 [0.93-1.44]	1.16 [0.93-1.46]
Y86	2,910	46,381,050	0.06	0.06	0.06	0.00	0.00	0.00	0.99 [0.87-1.13]	0.97 [0.85-1.11]
Y87	580	46,412,060	0.01	0.01	0.01	0.00	0.00	0.00	1.05 [0.78-1.40]	0.98 [0.72-1.33]
Y95	930	46,411,760	0.02	0.02	0.02	0.01	0.01	0.01	1.39 [1.12-1.73]*	1.43 [1.14-1.79]*
<18 - Z00-Z99										
Z00	920	46,355,600	0.02	0.02	0.02	0.00	0.00	0.00	1.18 [0.95-1.46]	1.17 [0.94-1.46]
Z01	19,200	45,677,820	0.90	0.29	0.29	0.61	0.61	0.54	3.12 [3.00-3.25]*	2.48 [2.38-2.59]*
Z02	1,810	46,400,720	0.03	0.04	0.03	−0.01	0.00	0.00	1.06 [0.89-1.25]	1.04 [0.87-1.24]
Z03	33,510	44,686,530	0.94	0.70	0.68	0.24	0.26	0.22	1.38 [1.34-1.43]*	1.30 [1.25-1.34]*
Z04	11,460	45,929,180	0.26	0.25	0.23	0.01	0.03	0.03	1.14 [1.07-1.21]*	1.11 [1.04-1.18]*
Z08	1,400	46,400,340	0.03	0.03	0.03	0.00	0.00	0.00	0.98 [0.82-1.18]	1.01 [0.84-1.22]
Z09	10,380	46,205,250	0.25	0.22	0.21	0.03	0.04	0.03	1.18 [1.11-1.26]*	1.14 [1.06-1.21]*
Z11	57,710	46,286,310	1.39	1.21	1.19	0.19	0.21	0.17	1.17 [1.14-1.21]*	1.14 [1.11-1.17]*
Z12	550	46,409,370	0.01	0.01	0.01	0.00	0.00	0.00	1.30 [0.98-1.74]	1.23 [0.91-1.65]
Z13	33,760	45,650,510	0.89	0.70	0.65	0.20	0.24	0.20	1.37 [1.32-1.42]*	1.29 [1.24-1.33]*
Z20	1,950	46,370,790	0.05	0.04	0.04	0.01	0.01	0.01	1.22 [1.06-1.42]*	1.19 [1.03-1.39]
Z22	13,220	46,267,650	0.29	0.28	0.26	0.00	0.03	0.03	1.13 [1.06-1.20]*	1.10 [1.03-1.17]*
Z24	810	46,349,990	0.02	0.02	0.02	0.00	0.00	0.00	1.02 [0.80-1.31]	0.98 [0.76-1.27]
Z25	640	46,398,460	0.03	0.01	0.01	0.02	0.02	0.02	3.36 [2.69-4.21]*	2.52 [1.98-3.21]*
Z27	3,260	46,278,730	0.14	0.05	0.04	0.09	0.10	0.09	3.25 [2.94-3.59]*	2.71 [2.44-3.01]*
Z29	8,640	45,598,000	0.20	0.19	0.17	0.02	0.03	0.03	1.17 [1.09-1.26]*	1.14 [1.06-1.23]*
Z30	5,960	46,348,660	0.11	0.13	0.11	−0.02	0.00	0.00	1.03 [0.94-1.13]	1.03 [0.94-1.13]
Z32	1,150	46,404,670	0.02	0.03	0.02	0.00	0.00	0.00	1.06 [0.86-1.31]	1.07 [0.86-1.34]
Z33	12,380	46,309,960	0.26	0.27	0.23	−0.01	0.03	0.02	1.12 [1.05-1.20]*	1.10 [1.03-1.18]*
Z34	32,090	46,123,090	0.63	0.71	0.63	−0.09	−0.01	−0.01	0.99 [0.95-1.03]	0.99 [0.95-1.03]
Z35	20,940	46,284,360	0.40	0.47	0.41	−0.07	−0.01	−0.02	0.97 [0.92-1.02]	0.96 [0.91-1.01]
Z36	3,280	46,372,610	0.06	0.07	0.06	−0.01	0.00	0.00	0.99 [0.87-1.12]	0.97 [0.85-1.10]
Z37	138,770	45,098,260	2.81	3.15	2.87	−0.34	−0.06	−0.06	0.98 [0.96-1.00]	0.98 [0.96-1.00]
Z39	7,720	46,344,620	0.15	0.17	0.14	−0.02	0.01	0.01	1.05 [0.97-1.14]	1.06 [0.97-1.15]
Z40	840	46,412,250	0.02	0.02	0.02	0.00	0.00	0.00	1.07 [0.85-1.35]	1.05 [0.83-1.33]
Z41	2,760	46,314,530	0.08	0.06	0.05	0.02	0.02	0.02	1.39 [1.24-1.57]*	1.39 [1.23-1.57]*
Z42	1,680	46,391,800	0.04	0.04	0.03	0.00	0.01	0.00	1.15 [0.98-1.36]	1.15 [0.97-1.36]
Z43	3,640	46,342,440	0.07	0.08	0.08	−0.01	−0.01	−0.01	0.93 [0.83-1.04]	0.87 [0.77-0.98]
Z45	5,990	46,312,530	0.14	0.13	0.13	0.01	0.01	0.01	1.08 [0.99-1.18]	1.04 [0.96-1.14]
Z46	4,270	46,345,420	0.09	0.09	0.09	0.00	0.00	0.00	1.05 [0.95-1.17]	1.02 [0.92-1.14]
Z47	24,700	46,002,290	0.55	0.53	0.53	0.02	0.02	0.01	1.03 [0.99-1.08]	1.02 [0.97-1.06]
Z48	5,650	46,265,370	0.13	0.12	0.12	0.01	0.01	0.01	1.10 [1.01-1.20]	1.06 [0.97-1.16]
Z50	16,820	46,298,690	0.40	0.35	0.36	0.04	0.04	0.03	1.12 [1.06-1.18]*	1.07 [1.02-1.13]
Z51	24,270	46,015,510	0.60	0.51	0.49	0.09	0.11	0.08	1.22 [1.17-1.27]*	1.17 [1.12-1.22]*
Z53	54,150	45,537,250	1.38	1.14	1.11	0.25	0.27	0.22	1.25 [1.21-1.28]*	1.19 [1.16-1.23]*
Z55	1,380	46,405,520	0.04	0.03	0.03	0.01	0.01	0.01	1.23 [1.03-1.46]	1.19 [0.99-1.42]
Z56	820	46,413,400	0.02	0.02	0.02	0.00	0.00	0.00	1.00 [0.78-1.29]	0.96 [0.73-1.25]
Z58	2,680	46,376,250	0.08	0.05	0.05	0.02	0.02	0.02	1.44 [1.27-1.62]*	1.36 [1.20-1.54]*
Z59	1,780	46,399,370	0.04	0.04	0.04	0.00	0.00	0.00	1.02 [0.87-1.21]	1.08 [0.90-1.28]
Z60	2,590	46,363,180	0.05	0.06	0.06	0.00	0.00	0.00	0.92 [0.80-1.06]	0.92 [0.80-1.06]
Z61	3,240	46,379,370	0.07	0.07	0.07	0.00	0.00	0.00	0.98 [0.87-1.11]	0.97 [0.86-1.10]
Z62	890	46,398,870	0.02	0.02	0.02	0.00	0.00	0.00	0.96 [0.77-1.21]	0.94 [0.75-1.19]
Z63	12,700	46,233,570	0.27	0.28	0.29	−0.01	−0.02	−0.02	0.94 [0.88-1.00]	0.92 [0.86-0.98]

Z65	1,190	46,404,610	0.02	0.03	0.03	0.00	0.00	0.00	0.96 [0.78-1.17]	0.99 [0.80-1.23]
Z71	10,530	46,194,010	0.47	0.16	0.16	0.31	0.31	0.27	2.95 [2.79-3.12]*	2.36 [2.22-2.50]*
Z72	31,140	46,088,140	0.61	0.69	0.59	−0.09	0.01	0.01	1.02 [0.98-1.06]	1.02 [0.98-1.07]
Z73	1,840	46,404,950	0.04	0.04	0.04	0.00	0.00	0.00	1.08 [0.91-1.27]	1.05 [0.88-1.24]
Z75	1,700	46,387,370	0.04	0.04	0.04	0.00	0.00	0.00	1.13 [0.96-1.32]	1.11 [0.94-1.31]
Z76	1,810	45,502,160	0.04	0.04	0.03	0.00	0.01	0.00	1.19 [1.01-1.39]	1.13 [0.95-1.33]
Z80	5,360	46,371,520	0.12	0.11	0.10	0.01	0.02	0.02	1.22 [1.11-1.34]*	1.17 [1.06-1.29]*
Z81	9,030	46,231,030	0.22	0.19	0.19	0.03	0.03	0.02	1.13 [1.05-1.21]*	1.09 [1.02-1.18]
Z82	41,460	45,416,680	1.24	0.83	0.79	0.41	0.44	0.41	1.56 [1.51-1.61]*	1.50 [1.45-1.54]*
Z83	25,420	45,277,060	0.65	0.54	0.53	0.12	0.12	0.10	1.24 [1.19-1.29]*	1.19 [1.14-1.24]*
Z84	7,180	46,195,140	0.25	0.13	0.13	0.12	0.12	0.12	1.96 [1.83-2.10]*	1.88 [1.75-2.02]*
Z85	5,130	46,365,630	0.11	0.11	0.11	0.00	0.01	0.01	1.05 [0.96-1.16]	1.06 [0.96-1.17]
Z86	88,100	45,725,420	1.97	1.92	1.78	0.05	0.18	0.16	1.10 [1.08-1.13]*	1.09 [1.06-1.11]*
Z87	99,180	45,070,060	2.57	2.10	2.05	0.47	0.52	0.41	1.25 [1.23-1.28]*	1.19 [1.17-1.22]*
Z88	69,650	45,689,740	1.95	1.41	1.34	0.55	0.61	0.49	1.46 [1.42-1.49]*	1.34 [1.30-1.37]*
Z90	13,130	46,324,620	0.31	0.28	0.26	0.04	0.05	0.04	1.21 [1.14-1.28]*	1.16 [1.09-1.23]*
Z91	74,680	45,741,780	2.78	1.32	1.30	1.46	1.48	1.28	2.13 [2.08-2.18]*	1.86 [1.81-1.90]*
Z92	19,570	46,208,820	0.48	0.41	0.40	0.07	0.08	0.06	1.19 [1.13-1.25]*	1.14 [1.08-1.19]*
Z93	5,700	46,310,300	0.12	0.12	0.12	−0.01	−0.01	−0.01	0.96 [0.87-1.05]	0.90 [0.82-0.99]
Z94	2,000	46,390,380	0.05	0.04	0.04	0.00	0.00	0.00	1.09 [0.94-1.26]	1.06 [0.91-1.24]
Z95	4,930	46,356,700	0.10	0.11	0.11	0.00	−0.01	−0.01	0.95 [0.86-1.05]	0.92 [0.83-1.02]
Z96	8,770	46,337,410	0.20	0.19	0.18	0.02	0.02	0.02	1.12 [1.04-1.20]*	1.08 [1.01-1.17]
Z97	4,970	46,380,650	0.11	0.11	0.10	0.01	0.01	0.01	1.12 [1.01-1.23]	1.07 [0.97-1.18]
Z98	4,700	46,335,100	0.10	0.10	0.10	0.00	0.01	0.00	1.08 [0.98-1.19]	1.03 [0.93-1.14]
Z99	4,940	46,357,320	0.10	0.11	0.11	0.00	−0.01	−0.01	0.94 [0.86-1.04]	0.91 [0.82-1.01]
hospitalised - A00-B99										
A02	1,530	81,557,590	0.02	0.02	0.02	0.00	0.00	0.00	1.11 [0.93-1.31]	1.05 [0.89-1.25]
A04	46,880	80,951,040	0.62	0.57	0.52	0.05	0.10	0.08	1.19 [1.16-1.23]*	1.15 [1.12-1.19]*
A05	490	81,603,180	0.01	0.01	0.01	0.00	0.00	0.00	1.19 [0.87-1.62]	1.20 [0.87-1.64]
A07	860	81,590,390	0.01	0.01	0.01	0.00	0.00	0.00	1.17 [0.93-1.47]	1.14 [0.90-1.44]
A08	57,140	79,844,050	0.83	0.69	0.72	0.14	0.11	0.09	1.15 [1.12-1.18]*	1.12 [1.09-1.16]*
A09	212,790	79,837,570	2.98	2.59	2.45	0.39	0.52	0.41	1.21 [1.20-1.23]*	1.16 [1.14-1.18]*
A15	1,090	81,580,310	0.01	0.01	0.01	0.00	0.00	0.00	1.15 [0.94-1.42]	1.12 [0.90-1.38]
A16	2,140	81,548,890	0.03	0.03	0.02	0.01	0.01	0.01	1.30 [1.13-1.50]*	1.24 [1.07-1.44]*
A18	1,070	81,568,590	0.01	0.01	0.01	0.00	0.00	0.00	1.22 [1.00-1.50]	1.18 [0.96-1.45]
A31	1,090	81,599,450	0.02	0.01	0.01	0.00	0.00	0.00	1.35 [1.11-1.65]*	1.21 [0.99-1.48]
A38	1,840	81,585,520	0.03	0.02	0.03	0.01	0.00	0.00	1.03 [0.89-1.20]	1.02 [0.87-1.19]
A39	1,850	81,503,120	0.03	0.02	0.02	0.00	0.00	0.00	1.05 [0.90-1.22]	1.03 [0.88-1.21]
A40	9,810	81,514,230	0.13	0.12	0.11	0.02	0.03	0.03	1.26 [1.18-1.35]*	1.24 [1.16-1.33]*
A41	184,770	80,642,010	2.47	2.25	2.07	0.23	0.40	0.38	1.19 [1.18-1.21]*	1.18 [1.16-1.20]*
A46	580	81,589,290	0.01	0.01	0.01	0.00	0.01	0.01	1.97 [1.53-2.53]*	1.93 [1.50-2.49]*
A48	870	81,594,320	0.01	0.01	0.01	0.00	0.00	0.00	1.23 [0.98-1.54]	1.27 [1.01-1.60]
A49	26,570	81,284,930	0.36	0.32	0.29	0.05	0.08	0.07	1.27 [1.22-1.32]*	1.24 [1.19-1.29]*
A63	620	81,506,810	0.01	0.01	0.01	0.00	0.00	0.00	1.45 [1.12-1.86]*	1.38 [1.07-1.80]
A69	700	81,599,440	0.01	0.01	0.01	0.00	0.00	0.00	1.14 [0.89-1.47]	1.07 [0.82-1.38]
A86	1,260	81,589,510	0.02	0.02	0.01	0.00	0.00	0.00	1.03 [0.84-1.25]	0.99 [0.81-1.21]
A87	3,260	81,483,260	0.04	0.04	0.04	0.00	0.01	0.00	1.16 [1.03-1.30]	1.09 [0.96-1.22]
B00	9,070	81,373,920	0.20	0.09	0.09	0.11	0.11	0.11	2.31 [2.17-2.46]*	2.18 [2.05-2.33]*
B01	8,220	81,337,150	0.15	0.09	0.10	0.06	0.05	0.05	1.53 [1.44-1.64]*	1.49 [1.40-1.60]*
B02	13,560	81,467,790	0.20	0.16	0.15	0.04	0.05	0.04	1.32 [1.24-1.40]*	1.26 [1.19-1.33]*
B07	5,490	81,435,720	0.08	0.06	0.06	0.02	0.02	0.02	1.32 [1.21-1.45]*	1.25 [1.14-1.37]*
B08	3,000	81,528,610	0.05	0.03	0.03	0.02	0.02	0.02	1.54 [1.38-1.72]*	1.49 [1.33-1.67]*
B09	1,310	81,561,170	0.02	0.02	0.02	0.00	0.00	0.00	1.17 [0.98-1.40]	1.16 [0.97-1.39]
B15	660	81,592,280	0.01	0.01	0.01	0.00	0.00	0.00	1.06 [0.82-1.39]	1.03 [0.79-1.35]
B16	1,290	81,566,860	0.02	0.02	0.01	0.00	0.00	0.00	1.17 [0.97-1.41]	1.17 [0.97-1.42]
B17	1,990	81,558,400	0.03	0.02	0.02	0.00	0.01	0.00	1.24 [1.07-1.44]*	1.20 [1.03-1.40]
B18	8,230	81,428,980	0.10	0.10	0.09	−0.01	0.01	0.01	1.07 [0.99-1.16]	1.09 [1.01-1.18]
B19	470	81,599,640	0.01	0.01	0.01	0.00	0.00	0.00	1.18 [0.87-1.60]	1.14 [0.84-1.57]
B25	1,690	81,585,200	0.02	0.02	0.02	0.00	0.00	0.00	1.23 [1.05-1.44]	1.18 [1.01-1.40]
B26	500	81,595,840	0.01	0.01	0.01	0.00	0.00	0.00	1.13 [0.84-1.52]	1.08 [0.79-1.46]
B27	4,620	81,485,940	0.07	0.05	0.06	0.01	0.01	0.00	1.08 [0.98-1.19]	1.04 [0.94-1.15]
B30	620	81,596,460	0.01	0.01	0.01	0.00	0.00	0.00	1.16 [0.90-1.51]	1.09 [0.83-1.43]
B34	124,310	77,872,300	2.32	1.42	1.56	0.90	0.76	0.70	1.49 [1.46-1.51]*	1.44 [1.41-1.46]*
B35	5,770	81,534,900	0.10	0.06	0.06	0.03	0.04	0.03	1.65 [1.51-1.79]*	1.55 [1.42-1.69]*
B36	2,230	81,590,440	0.04	0.02	0.02	0.01	0.01	0.01	1.68 [1.46-1.92]*	1.60 [1.39-1.84]*
B37	77,600	80,744,150	1.08	0.93	0.86	0.15	0.22	0.17	1.26 [1.23-1.29]*	1.19 [1.16-1.22]*
B44	3,100	81,579,850	0.05	0.04	0.03	0.01	0.02	0.01	1.51 [1.35-1.70]*	1.27 [1.13-1.43]*
B49	1,810	81,594,820	0.03	0.02	0.02	0.01	0.01	0.01	1.51 [1.30-1.76]*	1.45 [1.25-1.69]*
B50	690	81,574,190	0.01	0.01	0.01	0.00	0.00	0.00	0.83 [0.63-1.10]	0.86 [0.65-1.14]
B59	920	81,603,630	0.01	0.01	0.01	0.00	0.00	0.00	1.36 [1.09-1.68]*	1.30 [1.04-1.62]
B80	2,300	81,565,690	0.03	0.03	0.03	0.01	0.00	0.00	1.16 [1.01-1.33]	1.11 [0.96-1.27]
B85	930	81,590,850	0.01	0.01	0.01	0.00	0.00	0.00	1.04 [0.83-1.30]	1.03 [0.82-1.29]
B86	1,590	81,570,060	0.03	0.02	0.01	0.02	0.02	0.02	2.19 [1.87-2.55]*	2.20 [1.88-2.58]*
B90	720	81,601,060	0.01	0.01	0.01	0.00	0.00	0.00	1.41 [1.11-1.81]*	1.31 [1.02-1.68]
B91	1,070	81,590,030	0.01	0.01	0.01	0.00	0.00	0.00	1.14 [0.92-1.41]	1.16 [0.94-1.44]
B94	1,940	81,584,660	0.03	0.02	0.02	0.00	0.01	0.00	1.24 [1.07-1.44]*	1.18 [1.01-1.37]
B95	107,700	79,824,640	1.66	1.27	1.17	0.39	0.48	0.46	1.41 [1.38-1.44]*	1.38 [1.35-1.41]*
B96	216,040	78,712,320	2.94	2.70	2.52	0.24	0.42	0.36	1.17 [1.15-1.18]*	1.14 [1.12-1.16]*
B97	60,280	80,666,480	0.89	0.71	0.71	0.18	0.18	0.14	1.25 [1.22-1.29]*	1.19 [1.16-1.23]*
B98	21,450	81,435,090	0.27	0.26	0.24	0.01	0.03	0.02	1.13 [1.08-1.18]*	1.10 [1.04-1.15]*
B99	6,270	81,575,560	0.08	0.08	0.07	0.01	0.01	0.01	1.20 [1.10-1.32]*	1.19 [1.09-1.31]*
hospitalised - C00-D49										
C00	700	81,598,920	0.01	0.01	0.01	0.00	0.00	0.00	1.04 [0.80-1.36]	1.03 [0.79-1.35]
C01	1,420	81,596,880	0.02	0.02	0.01	0.00	0.00	0.00	1.12 [0.93-1.35]	1.17 [0.97-1.41]
C02	2,190	81,580,900	0.03	0.03	0.02	0.00	0.01	0.01	1.30 [1.13-1.50]*	1.31 [1.13-1.51]*
C03	510	81,605,650	0.01	0.01	0.01	0.00	0.00	0.00	1.32 [0.99-1.76]	1.32 [0.98-1.77]
C04	690	81,600,140	0.01	0.01	0.01	0.00	0.00	0.00	1.10 [0.84-1.43]	1.13 [0.86-1.48]
C05	690	81,601,330	0.01	0.01	0.01	0.00	0.00	0.00	1.14 [0.89-1.47]	1.20 [0.93-1.56]
C06	1,030	81,599,480	0.01	0.01	0.01	0.00	0.00	0.00	1.18 [0.96-1.46]	1.19 [0.96-1.47]
C07	1,000	81,596,020	0.01	0.01	0.01	0.00	0.00	0.00	1.04 [0.83-1.31]	1.03 [0.82-1.30]
C09	1,740	81,586,910	0.02	0.02	0.02	0.00	0.00	0.00	1.07 [0.90-1.26]	1.08 [0.91-1.28]
C10	950	81,603,520	0.01	0.01	0.01	0.00	0.00	0.00	1.08 [0.86-1.36]	1.09 [0.87-1.38]
C12	500	81,606,400	0.01	0.01	0.01	0.00	0.00	0.00	1.26 [0.93-1.70]	1.33 [0.98-1.81]
C13	570	81,607,400	0.01	0.01	0.01	0.00	0.00	0.00	1.19 [0.89-1.58]	1.30 [0.97-1.75]
C14	1,120	81,603,030	0.01	0.01	0.01	0.00	0.00	0.00	0.96 [0.77-1.18]	0.97 [0.78-1.20]
C15	13,720	81,546,480	0.17	0.17	0.15	0.00	0.02	0.02	1.13 [1.07-1.20]*	1.14 [1.07-1.21]*
C16	11,120	81,548,420	0.13	0.14	0.12	−0.01	0.01	0.01	1.06 [0.99-1.14]	1.06 [0.99-1.14]
C17	2,410	81,593,690	0.03	0.03	0.03	0.00	0.00	0.00	1.08 [0.94-1.24]	1.07 [0.93-1.23]
C18	37,720	81,181,490	0.42	0.47	0.43	−0.05	0.00	0.00	1.00 [0.96-1.04]	1.00 [0.96-1.03]
C19	7,770	81,530,200	0.08	0.10	0.09	−0.02	−0.01	0.00	0.94 [0.87-1.02]	0.95 [0.88-1.04]
C20	15,290	81,389,270	0.16	0.19	0.17	−0.03	−0.01	−0.01	0.95 [0.90-1.01]	0.97 [0.91-1.03]
C21	2,100	81,583,730	0.02	0.03	0.02	0.00	0.00	0.00	1.04 [0.89-1.21]	1.04 [0.89-1.21]
C22	8,170	81,586,170	0.11	0.10	0.09	0.01	0.02	0.02	1.18 [1.09-1.27]*	1.17 [1.08-1.26]*
C23	1,100	81,607,650	0.01	0.01	0.01	0.00	0.00	0.00	1.03 [0.83-1.28]	1.04 [0.84-1.29]
C24	2,140	81,600,390	0.02	0.03	0.02	0.00	0.00	0.00	1.04 [0.89-1.22]	1.03 [0.88-1.20]
C25	12,960	81,580,950	0.16	0.16	0.14	0.00	0.02	0.02	1.13 [1.07-1.20]*	1.13 [1.07-1.21]*
C26	3,170	81,594,500	0.04	0.04	0.04	0.00	0.00	0.00	1.06 [0.93-1.20]	1.05 [0.92-1.19]

C32	3,070	81,566,270	0.04	0.04	0.03	0.00	0.00	0.00	1.08 [0.95-1.22]	1.11 [0.98-1.26]
C34	56,780	81,413,950	0.70	0.70	0.63	0.00	0.07	0.07	1.11 [1.08-1.14]*	1.12 [1.09-1.15]*
C38	780	81,606,300	0.01	0.01	0.01	0.00	0.00	0.00	1.02 [0.80-1.32]	1.04 [0.80-1.34]
C40	550	81,600,130	0.01	0.01	0.01	0.00	0.00	0.00	1.02 [0.75-1.37]	1.04 [0.77-1.41]
C41	1,600	81,592,870	0.02	0.02	0.02	0.00	0.00	0.00	1.18 [1.00-1.40]	1.19 [1.00-1.41]
C43	15,430	81,375,510	0.17	0.20	0.17	−0.03	−0.01	−0.01	0.97 [0.91-1.02]	0.96 [0.90-1.02]
C44	110,000	79,937,130	1.34	1.39	1.27	−0.05	0.07	0.04	1.06 [1.03-1.08]*	1.03 [1.01-1.06]*
C45	3,400	81,601,410	0.04	0.04	0.04	0.00	0.00	0.00	1.02 [0.91-1.16]	1.04 [0.92-1.18]
C48	1,890	81,598,880	0.02	0.02	0.02	0.00	0.00	0.00	1.04 [0.89-1.22]	1.03 [0.88-1.21]
C49	3,580	81,565,410	0.04	0.04	0.04	0.00	0.00	0.00	1.10 [0.98-1.24]	1.09 [0.97-1.23]
C50	61,740	80,280,890	0.72	0.78	0.70	−0.06	0.02	0.02	1.02 [0.99-1.05]	1.03 [1.00-1.06]
C51	1,730	81,584,860	0.02	0.02	0.02	0.00	0.00	0.00	1.21 [1.03-1.43]	1.20 [1.02-1.42]
C52	590	81,605,690	0.01	0.01	0.01	0.00	0.00	0.00	0.93 [0.69-1.24]	0.93 [0.69-1.25]
C53	3,410	81,539,120	0.04	0.04	0.04	0.00	0.00	0.00	1.02 [0.90-1.15]	1.05 [0.93-1.19]
C54	9,850	81,431,870	0.11	0.12	0.11	−0.01	0.00	0.00	1.03 [0.96-1.10]	1.04 [0.97-1.12]
C55	1,470	81,594,570	0.02	0.02	0.02	0.00	0.00	0.00	0.96 [0.80-1.15]	0.95 [0.79-1.15]
C56	9,020	81,499,100	0.10	0.11	0.10	−0.01	0.00	0.00	1.00 [0.93-1.08]	1.01 [0.94-1.09]
C57	1,070	81,603,150	0.01	0.01	0.01	0.00	0.00	0.00	1.15 [0.94-1.42]	1.17 [0.95-1.44]
C60	810	81,597,070	0.01	0.01	0.01	0.00	0.00	0.00	1.17 [0.92-1.49]	1.21 [0.94-1.55]
C61	61,890	80,881,010	0.72	0.78	0.71	−0.06	0.00	0.00	1.01 [0.98-1.04]	1.00 [0.97-1.03]
C62	1,320	81,554,190	0.02	0.02	0.01	0.00	0.00	0.00	1.02 [0.84-1.24]	1.03 [0.84-1.25]
C64	13,300	81,477,050	0.16	0.17	0.15	−0.01	0.01	0.01	1.06 [0.99-1.12]	1.05 [0.99-1.12]
C65	1,240	81,598,470	0.01	0.02	0.01	0.00	0.00	0.00	1.11 [0.91-1.36]	1.15 [0.94-1.41]
C66	1,750	81,595,990	0.02	0.02	0.02	0.00	0.00	0.00	0.99 [0.83-1.17]	1.00 [0.84-1.18]
C67	28,770	81,173,620	0.34	0.36	0.33	−0.02	0.01	0.01	1.03 [0.99-1.07]	1.03 [0.99-1.08]
C68	700	81,604,130	0.01	0.01	0.01	0.00	0.00	0.00	0.94 [0.71-1.23]	0.95 [0.72-1.26]
C69	1,210	81,583,290	0.01	0.02	0.01	0.00	0.00	0.00	0.96 [0.79-1.18]	0.97 [0.79-1.19]
C71	6,810	81,552,500	0.08	0.08	0.08	0.00	0.01	0.01	1.09 [1.00-1.19]	1.09 [1.00-1.18]
C73	3,670	81,538,910	0.04	0.05	0.04	0.00	0.00	0.00	1.02 [0.91-1.15]	1.01 [0.90-1.14]
C74	620	81,601,690	0.01	0.01	0.01	0.00	0.00	0.00	1.09 [0.83-1.42]	1.08 [0.82-1.42]
C75	660	81,602,410	0.01	0.01	0.01	0.00	0.00	0.00	0.99 [0.75-1.30]	1.00 [0.76-1.32]
C76	2,110	81,590,460	0.03	0.03	0.02	0.00	0.00	0.00	1.09 [0.93-1.26]	1.09 [0.94-1.28]
C77	64,290	81,010,120	0.74	0.81	0.72	−0.06	0.03	0.04	1.04 [1.01-1.07]*	1.05 [1.02-1.08]*
C78	97,790	81,367,710	1.14	1.22	1.09	−0.08	0.05	0.07	1.05 [1.02-1.07]*	1.07 [1.04-1.09]*
C79	84,090	81,397,000	0.98	1.05	0.93	−0.06	0.05	0.07	1.05 [1.03-1.08]*	1.08 [1.05-1.11]*
C80	27,990	81,516,900	0.33	0.35	0.31	−0.01	0.03	0.03	1.09 [1.04-1.13]*	1.09 [1.05-1.14]*
C81	2,800	81,539,400	0.04	0.03	0.03	0.01	0.01	0.01	1.46 [1.30-1.65]*	1.43 [1.26-1.61]*
C82	3,520	81,555,970	0.04	0.04	0.04	0.00	0.00	0.00	1.04 [0.93-1.17]	1.02 [0.90-1.15]
C83	10,050	81,512,390	0.13	0.12	0.11	0.01	0.02	0.02	1.19 [1.11-1.27]*	1.17 [1.09-1.25]*
C84	1,330	81,592,440	0.03	0.01	0.01	0.01	0.01	0.01	2.15 [1.83-2.53]*	2.17 [1.84-2.57]*
C85	12,520	81,430,380	0.16	0.15	0.13	0.01	0.03	0.03	1.23 [1.16-1.30]*	1.21 [1.13-1.28]*
C88	1,760	81,596,430	0.02	0.02	0.02	0.00	0.00	0.00	1.14 [0.96-1.34]	1.12 [0.95-1.33]
C90	8,640	81,537,510	0.10	0.11	0.10	0.00	0.01	0.01	1.10 [1.02-1.19]	1.08 [1.00-1.17]
C91	9,190	81,491,170	0.11	0.11	0.11	0.00	0.00	0.00	1.04 [0.97-1.12]	1.03 [0.95-1.11]
C92	6,960	81,553,870	0.09	0.08	0.07	0.01	0.02	0.02	1.24 [1.15-1.35]*	1.24 [1.14-1.35]*
C93	980	81,607,480	0.01	0.01	0.01	0.00	0.00	0.00	1.11 [0.89-1.40]	1.15 [0.91-1.44]
C95	1,690	81,596,680	0.02	0.02	0.02	0.00	0.00	0.00	1.17 [0.99-1.38]	1.16 [0.98-1.37]
C97	8,170	81,583,760	0.09	0.10	0.09	−0.01	0.00	0.00	1.02 [0.94-1.10]	1.01 [0.93-1.09]
D00	900	81,600,100	0.01	0.01	0.01	0.00	0.00	0.00	1.02 [0.81-1.29]	1.01 [0.80-1.28]
D01	900	81,598,370	0.01	0.01	0.01	0.00	0.00	0.00	1.00 [0.79-1.27]	0.96 [0.76-1.22]
D02	890	81,599,930	0.01	0.01	0.01	0.00	0.00	0.00	1.14 [0.91-1.43]	1.13 [0.90-1.43]
D03	5,970	81,516,900	0.07	0.07	0.07	−0.01	0.00	0.00	1.02 [0.93-1.12]	1.00 [0.91-1.09]
D04	13,490	81,458,650	0.18	0.16	0.15	0.01	0.03	0.02	1.20 [1.13-1.28]*	1.16 [1.09-1.23]*
D05	10,490	81,358,630	0.11	0.13	0.12	−0.02	0.00	0.00	0.96 [0.90-1.03]	0.96 [0.89-1.03]
D06	9,040	81,199,100	0.10	0.11	0.10	−0.01	0.00	0.00	0.99 [0.92-1.07]	1.00 [0.93-1.08]
D07	3,510	81,538,570	0.04	0.04	0.04	0.00	0.01	0.00	1.13 [1.01-1.27]	1.10 [0.98-1.24]
D09	3,380	81,570,810	0.04	0.04	0.04	0.00	0.00	0.00	1.05 [0.93-1.19]	1.05 [0.93-1.19]
D10	3,320	81,512,670	0.05	0.04	0.04	0.01	0.01	0.01	1.25 [1.12-1.40]*	1.20 [1.07-1.35]*
D11	3,690	81,518,610	0.04	0.05	0.04	0.00	0.00	0.00	1.02 [0.91-1.15]	1.02 [0.91-1.15]
D12	111,980	79,893,120	1.40	1.40	1.24	0.00	0.17	0.12	1.13 [1.11-1.16]*	1.09 [1.07-1.12]*
D13	7,610	81,376,500	0.09	0.09	0.08	0.00	0.01	0.00	1.10 [1.02-1.19]	1.04 [0.96-1.13]
D14	2,380	81,545,640	0.03	0.03	0.03	0.00	0.00	0.00	1.16 [1.01-1.33]	1.12 [0.97-1.29]
D15	940	81,596,760	0.01	0.01	0.01	0.00	0.00	0.00	1.07 [0.85-1.34]	1.06 [0.84-1.33]
D16	3,980	81,505,160	0.05	0.05	0.05	0.01	0.00	0.00	1.08 [0.98-1.21]	1.05 [0.94-1.17]
D17	32,970	80,452,790	0.39	0.41	0.36	−0.02	0.03	0.01	1.07 [1.03-1.11]*	1.03 [0.99-1.07]
D18	16,030	81,207,280	0.20	0.20	0.18	0.01	0.02	0.01	1.11 [1.05-1.17]*	1.07 [1.01-1.13]
D21	3,680	81,509,190	0.05	0.04	0.04	0.00	0.01	0.01	1.21 [1.08-1.35]*	1.16 [1.04-1.30]*
D22	31,790	80,076,170	0.40	0.40	0.37	0.00	0.03	0.02	1.07 [1.03-1.12]*	1.04 [1.00-1.08]
D23	21,570	80,752,420	0.28	0.26	0.25	0.01	0.03	0.02	1.13 [1.08-1.19]*	1.09 [1.04-1.15]*
D24	10,600	81,228,790	0.12	0.13	0.12	−0.01	0.00	0.00	0.99 [0.93-1.06]	0.97 [0.90-1.04]
D25	51,800	79,878,700	0.62	0.65	0.59	−0.03	0.03	0.02	1.06 [1.03-1.09]*	1.03 [1.00-1.06]
D26	1,800	81,543,660	0.02	0.02	0.02	0.00	0.00	0.00	1.09 [0.93-1.29]	1.07 [0.90-1.26]
D27	12,610	81,291,980	0.15	0.16	0.14	−0.01	0.00	0.00	1.03 [0.96-1.09]	1.01 [0.95-1.07]
D28	1,400	81,565,610	0.02	0.02	0.01	0.00	0.00	0.00	1.20 [1.00-1.44]	1.17 [0.97-1.40]
D29	1,100	81,585,520	0.01	0.01	0.01	0.00	0.00	0.00	1.17 [0.95-1.44]	1.14 [0.92-1.40]
D30	2,160	81,577,330	0.02	0.03	0.02	0.00	0.00	0.00	1.03 [0.88-1.20]	1.01 [0.87-1.17]
D31	2,160	81,570,730	0.03	0.03	0.02	0.01	0.01	0.01	1.28 [1.11-1.48]*	1.26 [1.09-1.46]*
D32	7,880	81,523,740	0.09	0.10	0.09	0.00	0.01	0.01	1.09 [1.01-1.18]	1.07 [0.99-1.17]
D33	3,070	81,555,970	0.04	0.04	0.04	0.00	0.00	0.00	1.03 [0.91-1.17]	0.99 [0.87-1.12]
D34	1,990	81,562,410	0.02	0.02	0.02	0.00	0.00	0.00	1.06 [0.91-1.24]	1.01 [0.86-1.19]
D35	11,720	81,442,730	0.13	0.15	0.13	−0.01	0.00	0.00	1.04 [0.97-1.11]	1.01 [0.95-1.08]
D36	5,360	81,460,900	0.07	0.07	0.06	0.00	0.01	0.01	1.12 [1.02-1.23]	1.08 [0.98-1.19]
D37	14,600	81,421,620	0.17	0.18	0.16	−0.01	0.02	0.01	1.09 [1.03-1.16]*	1.09 [1.03-1.15]*
D38	3,480	81,577,490	0.04	0.04	0.04	0.00	0.01	0.01	1.15 [1.03-1.29]	1.17 [1.04-1.31]*
D39	1,960	81,564,350	0.02	0.02	0.02	0.00	0.00	0.00	1.09 [0.93-1.28]	1.12 [0.95-1.31]
D40	670	81,592,810	0.01	0.01	0.01	0.00	0.00	0.00	1.13 [0.86-1.47]	1.11 [0.85-1.46]
D41	10,510	81,461,220	0.12	0.13	0.12	−0.01	0.00	0.00	1.02 [0.95-1.09]	1.02 [0.95-1.09]
D43	3,550	81,572,440	0.04	0.04	0.04	0.00	0.00	0.00	1.09 [0.97-1.22]	1.08 [0.96-1.22]
D44	2,210	81,568,090	0.03	0.03	0.03	0.00	0.00	0.00	1.08 [0.94-1.26]	1.06 [0.91-1.23]
D45	3,850	81,531,490	0.04	0.05	0.04	0.00	0.00	0.00	1.06 [0.95-1.19]	1.04 [0.93-1.17]
D46	9,430	81,553,650	0.13	0.11	0.10	0.02	0.03	0.03	1.26 [1.17-1.35]*	1.24 [1.16-1.34]*
D47	15,950	81,504,200	0.21	0.19	0.18	0.02	0.03	0.03	1.19 [1.13-1.26]*	1.16 [1.09-1.22]*
D48	7,410	81,439,490	0.09	0.09	0.08	0.00	0.00	0.00	1.03 [0.95-1.11]	1.01 [0.93-1.09]
hospitalised - D50-D89										
D50	215,590	79,258,590	3.05	2.64	2.43	0.41	0.62	0.52	1.25 [1.23-1.27]*	1.20 [1.19-1.22]*
D51	19,160	81,384,470	0.26	0.23	0.20	0.03	0.06	0.05	1.29 [1.23-1.35]*	1.23 [1.17-1.29]*
D52	14,790	81,528,460	0.20	0.18	0.16	0.02	0.04	0.04	1.25 [1.18-1.32]*	1.24 [1.17-1.31]*
D53	4,040	81,578,680	0.06	0.05	0.04	0.01	0.01	0.01	1.35 [1.21-1.50]*	1.30 [1.17-1.45]*
D55	620	81,597,900	0.01	0.01	0.01	0.00	0.00	0.00	1.40 [1.08-1.82]	1.41 [1.08-1.83]
D56	7,820	81,445,640	0.11	0.09	0.09	0.02	0.02	0.02	1.27 [1.18-1.37]*	1.25 [1.16-1.35]*
D57	8,570	81,368,410	0.12	0.10	0.10	0.02	0.02	0.02	1.22 [1.13-1.31]*	1.21 [1.12-1.30]*
D58	2,230	81,567,830	0.03	0.03	0.02	0.00	0.00	0.00	1.17 [1.01-1.35]	1.17 [1.01-1.35]
D59	2,730	81,568,660	0.04	0.03	0.03	0.01	0.01	0.01	1.36 [1.20-1.54]*	1.36 [1.20-1.55]*
D61	13,800	81,526,800	0.19	0.17	0.15	0.02	0.04	0.04	1.25 [1.18-1.32]*	1.24 [1.17-1.31]*
D62	5,860	81,472,720	0.07	0.07	0.07	0.00	0.01	0.01	1.07 [0.98-1.18]	1.08 [0.99-1.19]
D63	20,860	81,470,620	0.28	0.25	0.23	0.02	0.05	0.05	1.20 [1.15-1.26]*	1.20 [1.14-1.26]*
D64	282,260	78,408,510	3.97	3.51	3.24	0.47	0.74	0.66	1.23 [1.21-1.24]*	1.20 [1.18-1.21]*
D65	940	81,604,210	0.01	0.01	0.01	0.00	0.00	0.00	1.16 [0.93-1.45]	1.17 [0.93-1.46]

D66	960	81,581,630	0.01	0.01	0.01	0.00	0.00	0.00	1.20 [0.97-1.50]	1.17 [0.94-1.47]
D68	16,700	81,332,350	0.22	0.20	0.18	0.02	0.03	0.02	1.19 [1.13-1.25]*	1.13 [1.07-1.19]*
D69	48,690	81,047,650	0.65	0.59	0.56	0.07	0.10	0.09	1.18 [1.14-1.21]*	1.17 [1.13-1.20]*
D70	40,740	81,194,780	0.53	0.49	0.45	0.04	0.08	0.08	1.18 [1.14-1.22]*	1.17 [1.13-1.21]*
D72	9,300	81,527,550	0.14	0.11	0.10	0.03	0.04	0.03	1.35 [1.26-1.45]*	1.26 [1.17-1.35]*
D73	9,950	81,507,860	0.13	0.12	0.11	0.01	0.02	0.02	1.16 [1.08-1.24]*	1.14 [1.07-1.23]*
D75	11,780	81,468,040	0.15	0.14	0.13	0.01	0.02	0.02	1.16 [1.09-1.24]*	1.15 [1.07-1.22]*
D76	790	81,588,920	0.01	0.01	0.01	0.00	0.00	0.00	1.28 [1.01-1.61]	1.21 [0.96-1.54]
D80	2,940	81,567,680	0.05	0.03	0.03	0.01	0.01	0.01	1.44 [1.28-1.62]*	1.30 [1.15-1.47]*
D82	720	81,595,290	0.01	0.01	0.01	0.00	0.00	0.00	1.27 [1.00-1.62]	1.19 [0.92-1.53]
D83	390	81,603,620	0.01	0.00	0.00	0.00	0.00	0.00	1.74 [1.28-2.37]*	1.55 [1.12-2.13]*
D84	1,580	81,586,570	0.03	0.02	0.02	0.01	0.01	0.01	1.55 [1.32-1.81]*	1.33 [1.13-1.56]*
D86	5,880	81,501,790	0.09	0.07	0.06	0.02	0.03	0.02	1.43 [1.32-1.56]*	1.34 [1.23-1.46]*
D89	4,920	81,558,150	0.07	0.06	0.05	0.01	0.02	0.01	1.32 [1.20-1.45]*	1.26 [1.14-1.38]*
hospitalised - E00-E89										
E02	1,770	81,600,700	0.02	0.02	0.02	0.00	0.00	0.00	1.11 [0.93-1.31]	1.11 [0.93-1.31]
E03	188,000	78,608,480	2.65	2.33	2.10	0.32	0.55	0.47	1.26 [1.24-1.28]*	1.22 [1.20-1.24]*
E04	20,750	81,261,940	0.25	0.26	0.23	0.00	0.02	0.02	1.09 [1.04-1.15]*	1.06 [1.01-1.12]
E05	34,380	81,111,240	0.45	0.42	0.38	0.04	0.08	0.06	1.21 [1.17-1.25]*	1.16 [1.12-1.21]*
E06	3,700	81,547,400	0.05	0.04	0.04	0.00	0.01	0.01	1.20 [1.07-1.34]*	1.14 [1.02-1.28]
E07	6,000	81,529,610	0.08	0.07	0.07	0.01	0.01	0.01	1.18 [1.08-1.29]*	1.11 [1.01-1.21]
E10	41,150	80,467,140	0.54	0.50	0.47	0.04	0.07	0.05	1.14 [1.11-1.18]*	1.11 [1.07-1.15]*
E11	275,400	76,698,820	3.84	3.53	3.21	0.31	0.64	0.53	1.20 [1.18-1.21]*	1.16 [1.15-1.18]*
E13	4,460	81,572,120	0.06	0.05	0.05	0.01	0.01	0.01	1.29 [1.16-1.42]*	1.19 [1.08-1.32]*
E14	45,900	80,960,140	0.63	0.55	0.49	0.07	0.13	0.11	1.27 [1.23-1.31]*	1.21 [1.17-1.25]*
E16	56,760	81,191,820	0.75	0.69	0.64	0.06	0.10	0.09	1.16 [1.13-1.20]*	1.14 [1.10-1.17]*
E20	1,520	81,593,770	0.02	0.02	0.02	0.00	0.01	0.00	1.32 [1.11-1.56]*	1.23 [1.03-1.46]
E21	16,730	81,461,500	0.20	0.21	0.19	0.00	0.02	0.01	1.08 [1.03-1.15]*	1.06 [1.00-1.12]
E22	10,920	81,510,590	0.16	0.13	0.12	0.04	0.05	0.04	1.39 [1.30-1.48]*	1.34 [1.26-1.43]*
E23	6,370	81,498,450	0.09	0.07	0.07	0.02	0.02	0.02	1.25 [1.15-1.36]*	1.20 [1.10-1.30]*
E24	1,360	81,585,690	0.02	0.02	0.01	0.00	0.01	0.00	1.40 [1.17-1.67]*	1.21 [1.01-1.45]
E26	720	81,596,600	0.01	0.01	0.01	0.00	0.00	0.00	1.16 [0.90-1.50]	1.14 [0.88-1.48]
E27	12,390	81,486,410	0.18	0.14	0.13	0.04	0.05	0.04	1.37 [1.29-1.45]*	1.27 [1.19-1.35]*
E28	27,200	81,137,860	0.36	0.33	0.31	0.04	0.05	0.04	1.18 [1.13-1.23]*	1.11 [1.06-1.16]*
E29	1,790	81,584,470	0.03	0.02	0.02	0.01	0.01	0.01	1.45 [1.25-1.69]*	1.32 [1.13-1.55]*
E30	2,720	81,563,210	0.05	0.03	0.04	0.01	0.01	0.01	1.15 [1.02-1.30]	1.13 [0.99-1.28]
E34	5,390	81,514,690	0.08	0.06	0.07	0.02	0.01	0.01	1.16 [1.06-1.27]*	1.11 [1.02-1.22]
E41	580	81,608,220	0.01	0.01	0.01	0.00	0.00	0.00	1.15 [0.86-1.55]	1.20 [0.89-1.61]
E43	750	81,606,700	0.01	0.01	0.01	0.00	0.00	0.00	1.11 [0.86-1.43]	1.13 [0.87-1.47]
E46	9,190	81,561,900	0.12	0.11	0.10	0.00	0.02	0.02	1.16 [1.08-1.25]*	1.17 [1.08-1.26]*
E51	900	81,600,720	0.01	0.01	0.01	0.00	0.00	0.00	1.36 [1.09-1.70]*	1.38 [1.10-1.73]*
E53	52,200	81,301,980	0.71	0.63	0.57	0.09	0.14	0.12	1.24 [1.20-1.28]*	1.19 [1.16-1.23]*
E55	72,780	81,242,350	1.05	0.86	0.81	0.20	0.24	0.19	1.30 [1.26-1.33]*	1.23 [1.19-1.26]*
E56	760	81,604,260	0.01	0.01	0.01	0.00	0.00	0.00	1.21 [0.94-1.54]	1.18 [0.92-1.52]
E58	530	81,605,810	0.01	0.01	0.01	0.00	0.00	0.00	1.55 [1.17-2.06]*	1.50 [1.13-1.99]*
E61	11,430	81,531,550	0.16	0.13	0.13	0.03	0.03	0.03	1.27 [1.19-1.35]*	1.19 [1.12-1.27]*
E63	700	81,604,840	0.01	0.01	0.01	0.00	0.00	0.00	1.23 [0.95-1.59]	1.18 [0.91-1.53]
E66	358,810	77,812,920	5.01	4.51	4.09	0.50	0.92	0.67	1.22 [1.21-1.24]*	1.15 [1.14-1.17]*
E70	420	81,595,160	0.01	0.00	0.01	0.00	0.00	0.00	1.25 [0.91-1.72]	1.22 [0.88-1.69]
E72	840	81,594,490	0.01	0.01	0.01	0.00	0.00	0.00	1.13 [0.90-1.43]	1.12 [0.89-1.43]
E73	4,060	81,531,880	0.06	0.05	0.05	0.02	0.02	0.01	1.32 [1.19-1.45]*	1.16 [1.05-1.29]*
E74	1,420	81,585,670	0.02	0.02	0.02	0.00	0.00	0.00	1.29 [1.08-1.53]*	1.23 [1.03-1.47]
E75	600	81,599,130	0.01	0.01	0.01	0.00	0.00	0.00	1.11 [0.84-1.47]	1.12 [0.85-1.48]
E78	386,100	75,439,450	5.43	5.04	4.71	0.39	0.72	0.59	1.15 [1.14-1.17]*	1.12 [1.11-1.14]*
E79	590	81,604,050	0.01	0.01	0.01	0.00	0.00	0.00	1.19 [0.90-1.58]	1.13 [0.85-1.50]
E80	5,630	81,524,740	0.07	0.07	0.06	0.00	0.01	0.00	1.08 [0.99-1.19]	1.03 [0.94-1.13]
E83	79,560	81,168,710	1.02	0.97	0.89	0.05	0.13	0.12	1.15 [1.12-1.18]*	1.13 [1.10-1.16]*
E84	630	81,573,470	0.01	0.01	0.01	0.00	0.00	0.00	1.14 [0.87-1.50]	1.03 [0.78-1.36]
E85	2,880	81,591,010	0.04	0.03	0.03	0.00	0.01	0.00	1.18 [1.04-1.34]	1.14 [1.00-1.30]
E86	208,290	80,284,600	2.77	2.55	2.43	0.21	0.34	0.32	1.14 [1.12-1.16]*	1.13 [1.11-1.15]*
E87	310,120	79,913,950	4.28	3.78	3.51	0.50	0.77	0.70	1.22 [1.21-1.24]*	1.20 [1.18-1.21]*
E88	15,120	81,513,790	0.21	0.18	0.17	0.03	0.04	0.03	1.23 [1.16-1.30]*	1.20 [1.13-1.27]*
E89	20,670	81,369,430	0.26	0.25	0.23	0.01	0.03	0.03	1.15 [1.10-1.21]*	1.11 [1.06-1.16]*
hospitalised - F01-F99										
F00	47,170	81,452,030	0.58	0.58	0.56	0.01	0.03	0.03	1.05 [1.02-1.09]*	1.05 [1.01-1.08]*
F01	55,520	81,418,320	0.69	0.68	0.65	0.01	0.04	0.04	1.06 [1.03-1.10]*	1.06 [1.03-1.09]*
F02	9,690	81,576,050	0.11	0.12	0.11	−0.01	−0.01	0.00	0.95 [0.88-1.03]	0.97 [0.90-1.05]
F03	129,530	81,128,370	1.62	1.59	1.55	0.02	0.06	0.07	1.04 [1.02-1.06]*	1.04 [1.02-1.07]*
F05	123,790	81,281,940	1.62	1.50	1.42	0.13	0.20	0.19	1.14 [1.12-1.16]*	1.13 [1.11-1.16]*
F06	19,490	81,507,640	0.25	0.24	0.22	0.01	0.02	0.02	1.10 [1.04-1.16]*	1.09 [1.04-1.15]*
F07	1,320	81,585,890	0.02	0.02	0.02	0.00	0.00	0.00	1.09 [0.90-1.32]	1.08 [0.89-1.31]
F10	91,770	79,520,110	1.18	1.15	1.03	0.03	0.14	0.15	1.14 [1.11-1.16]*	1.15 [1.12-1.18]*
F11	7,480	81,345,860	0.09	0.09	0.08	−0.01	0.01	0.00	1.07 [0.99-1.16]	1.06 [0.97-1.15]
F12	16,290	81,375,920	0.19	0.20	0.20	−0.01	−0.01	−0.01	0.95 [0.90-1.01]	0.96 [0.91-1.02]
F13	1,830	81,577,800	0.02	0.02	0.02	0.00	0.00	0.00	1.10 [0.93-1.29]	1.06 [0.89-1.24]
F14	6,700	81,501,640	0.08	0.08	0.08	−0.01	0.00	0.00	0.98 [0.90-1.07]	0.99 [0.91-1.08]
F15	2,280	81,555,960	0.03	0.03	0.03	0.00	0.00	0.00	0.93 [0.80-1.08]	0.94 [0.81-1.10]
F16	680	81,589,220	0.01	0.01	0.01	0.00	0.00	0.00	0.87 [0.66-1.14]	0.88 [0.66-1.17]
F17	459,920	76,318,170	5.87	6.07	5.53	−0.19	0.34	0.49	1.06 [1.05-1.07]*	1.09 [1.08-1.10]*
F19	7,480	81,462,220	0.08	0.09	0.09	−0.01	0.00	0.00	0.96 [0.89-1.05]	0.98 [0.90-1.07]
F20	13,370	81,160,100	0.17	0.16	0.15	0.00	0.02	0.02	1.15 [1.08-1.22]*	1.17 [1.10-1.24]*
F22	6,480	81,515,060	0.09	0.08	0.07	0.01	0.01	0.01	1.19 [1.10-1.30]*	1.19 [1.09-1.29]*
F23	3,690	81,508,600	0.04	0.05	0.04	0.00	0.00	0.00	1.07 [0.96-1.20]	1.08 [0.96-1.21]
F25	3,420	81,513,540	0.04	0.04	0.04	0.00	0.00	0.01	1.11 [0.99-1.25]	1.15 [1.02-1.30]
F29	7,550	81,501,580	0.10	0.09	0.09	0.01	0.01	0.01	1.11 [1.03-1.20]*	1.11 [1.02-1.20]
F30	1,870	81,545,300	0.02	0.02	0.02	0.00	0.00	0.00	1.19 [1.02-1.39]	1.18 [1.01-1.38]
F31	18,490	81,255,810	0.23	0.23	0.20	0.01	0.03	0.03	1.16 [1.10-1.22]*	1.12 [1.06-1.18]*
F32	328,920	77,616,790	4.74	4.11	3.76	0.63	0.99	0.78	1.26 [1.25-1.28]*	1.20 [1.18-1.21]*
F33	10,430	81,330,010	0.13	0.13	0.11	0.00	0.02	0.02	1.19 [1.11-1.27]*	1.15 [1.07-1.23]*
F34	1,800	81,578,730	0.02	0.02	0.02	0.00	0.00	0.00	1.23 [1.05-1.44]*	1.13 [0.96-1.32]
F38	820	81,600,480	0.01	0.01	0.01	0.00	0.00	0.00	1.16 [0.92-1.47]	1.10 [0.86-1.41]
F39	1,500	81,590,650	0.02	0.02	0.02	0.00	0.00	0.00	1.24 [1.04-1.47]	1.17 [0.98-1.40]
F40	30,890	81,275,260	0.43	0.37	0.36	0.06	0.07	0.05	1.19 [1.14-1.24]*	1.13 [1.09-1.18]*
F41	281,700	79,236,760	4.01	3.44	3.22	0.56	0.79	0.58	1.25 [1.23-1.26]*	1.17 [1.16-1.19]*
F42	6,960	81,521,290	0.11	0.08	0.08	0.03	0.04	0.03	1.47 [1.36-1.58]*	1.34 [1.24-1.46]*
F43	16,460	81,327,920	0.21	0.20	0.19	0.02	0.03	0.02	1.16 [1.10-1.22]*	1.09 [1.04-1.15]*
F44	4,100	81,555,090	0.06	0.05	0.05	0.01	0.01	0.01	1.23 [1.11-1.36]*	1.11 [1.00-1.24]
F45	4,700	81,515,050	0.07	0.06	0.05	0.01	0.02	0.01	1.29 [1.17-1.42]*	1.17 [1.06-1.29]*
F48	1,350	81,589,030	0.02	0.02	0.01	0.00	0.00	0.00	1.24 [1.04-1.48]	1.12 [0.93-1.35]
F50	7,180	81,486,360	0.11	0.08	0.09	0.02	0.02	0.01	1.19 [1.10-1.28]*	1.13 [1.04-1.22]*
F51	1,650	81,588,390	0.03	0.02	0.02	0.01	0.01	0.01	1.37 [1.17-1.60]*	1.27 [1.08-1.50]*
F52	2,630	81,558,310	0.04	0.03	0.03	0.00	0.01	0.01	1.32 [1.16-1.50]*	1.24 [1.09-1.42]*
F53	2,750	81,558,040	0.04	0.03	0.03	0.00	0.01	0.00	1.20 [1.06-1.36]*	1.15 [1.01-1.31]
F60	14,290	81,347,590	0.19	0.17	0.16	0.02	0.03	0.02	1.21 [1.14-1.27]*	1.13 [1.07-1.20]*
F69	1,130	81,585,390	0.02	0.01	0.01	0.00	0.00	0.00	1.39 [1.15-1.68]*	1.29 [1.06-1.57]
F70	2,060	81,563,140	0.03	0.02	0.02	0.01	0.01	0.01	1.50 [1.30-1.72]*	1.39 [1.20-1.61]*
F71	820	81,589,730	0.01	0.01	0.01	0.01	0.01	0.01	1.73 [1.39-2.15]*	1.67 [1.34-2.08]*

F72	1,640	81,576,620	0.03	0.02	0.02	0.01	0.01	0.01	1.42 [1.21-1.66]*	1.34 [1.14-1.57]*
F79	9,790	81,460,770	0.16	0.11	0.12	0.05	0.04	0.04	1.38 [1.30-1.48]*	1.31 [1.23-1.40]*
F80	11,110	81,474,720	0.16	0.13	0.14	0.03	0.02	0.02	1.14 [1.08-1.22]*	1.12 [1.05-1.19]*
F81	26,620	81,066,040	0.43	0.30	0.31	0.12	0.11	0.10	1.36 [1.31-1.42]*	1.29 [1.24-1.35]*
F82	1,570	81,584,850	0.02	0.02	0.02	0.00	0.00	0.00	1.06 [0.90-1.25]	1.02 [0.86-1.20]
F83	1,490	81,593,500	0.02	0.02	0.02	0.00	0.00	0.00	0.88 [0.74-1.06]	0.84 [0.70-1.01]
F84	25,430	81,309,560	0.43	0.28	0.35	0.14	0.08	0.05	1.23 [1.18-1.28]*	1.14 [1.09-1.19]*
F89	7,210	81,549,550	0.10	0.09	0.10	0.01	−0.01	−0.01	0.94 [0.87-1.01]	0.88 [0.82-0.96]*
F90	13,160	81,467,110	0.21	0.15	0.19	0.06	0.03	0.02	1.14 [1.08-1.21]*	1.11 [1.04-1.17]*
F91	2,870	81,547,410	0.04	0.03	0.04	0.01	0.01	0.00	1.16 [1.03-1.31]	1.12 [0.99-1.26]
F94	520	81,604,790	0.01	0.01	0.01	0.00	0.00	0.00	1.07 [0.81-1.43]	1.04 [0.77-1.39]
F95	1,520	81,591,710	0.03	0.02	0.02	0.01	0.01	0.00	1.27 [1.08-1.50]*	1.20 [1.01-1.42]
F98	1,990	81,577,510	0.03	0.02	0.03	0.01	0.01	0.00	1.22 [1.05-1.41]*	1.15 [0.99-1.34]
F99	5,580	81,473,030	0.07	0.07	0.06	0.00	0.01	0.01	1.12 [1.02-1.23]	1.09 [0.99-1.19]
hospitalised - G00-G99										
G00	1,450	81,545,990	0.02	0.02	0.02	0.00	0.00	0.00	1.02 [0.85-1.22]	0.98 [0.81-1.18]
G01	700	81,569,010	0.01	0.01	0.01	0.00	0.00	0.00	1.10 [0.85-1.41]	1.08 [0.84-1.39]
G02	690	81,584,660	0.01	0.01	0.01	0.00	0.00	0.00	1.05 [0.81-1.37]	1.00 [0.76-1.30]
G03	1,960	81,549,040	0.02	0.02	0.02	0.00	0.00	0.00	1.05 [0.90-1.23]	1.02 [0.87-1.19]
G04	4,270	81,552,860	0.06	0.05	0.05	0.01	0.01	0.01	1.15 [1.04-1.28]*	1.12 [1.01-1.24]
G05	1,030	81,593,120	0.02	0.01	0.01	0.00	0.00	0.00	1.44 [1.18-1.77]*	1.38 [1.12-1.70]*
G06	1,840	81,581,090	0.02	0.02	0.02	0.00	0.00	0.00	1.20 [1.03-1.41]	1.20 [1.02-1.41]
G08	950	81,595,360	0.01	0.01	0.01	0.00	0.00	0.00	1.29 [1.04-1.60]	1.29 [1.03-1.61]
G09	1,280	81,585,170	0.02	0.02	0.01	0.00	0.00	0.00	1.17 [0.98-1.41]	1.14 [0.95-1.38]
G10	470	81,600,980	0.01	0.01	0.01	0.00	0.00	0.00	1.25 [0.92-1.69]	1.27 [0.93-1.73]
G11	1,950	81,579,880	0.02	0.02	0.02	0.00	0.00	0.00	1.15 [0.99-1.34]	1.14 [0.97-1.33]
G12	3,840	81,583,480	0.05	0.05	0.04	0.00	0.00	0.00	1.06 [0.95-1.19]	1.05 [0.94-1.18]
G20	33,700	81,335,820	0.40	0.42	0.39	−0.02	0.01	0.01	1.03 [0.99-1.07]	1.03 [0.99-1.07]
G21	3,370	81,590,570	0.05	0.04	0.04	0.01	0.01	0.01	1.22 [1.08-1.38]*	1.24 [1.10-1.40]*
G23	2,030	81,600,540	0.02	0.03	0.02	0.00	0.00	0.00	1.02 [0.87-1.19]	1.01 [0.86-1.18]
G24	4,670	81,540,800	0.06	0.06	0.06	0.00	0.00	0.00	1.04 [0.94-1.15]	1.02 [0.92-1.13]
G25	21,460	81,434,560	0.29	0.26	0.24	0.03	0.05	0.03	1.20 [1.15-1.26]*	1.13 [1.07-1.18]*
G30	62,200	81,361,150	0.77	0.76	0.73	0.01	0.04	0.04	1.06 [1.03-1.09]*	1.05 [1.02-1.09]*
G31	46,520	81,362,890	0.59	0.57	0.52	0.03	0.07	0.07	1.13 [1.10-1.17]*	1.13 [1.09-1.17]*
G32	760	81,602,140	0.01	0.01	0.01	0.00	0.00	0.00	1.29 [1.01-1.64]	1.29 [1.01-1.65]
G35	8,460	81,328,400	0.10	0.10	0.09	0.00	0.01	0.01	1.14 [1.06-1.22]*	1.12 [1.04-1.21]*
G37	2,540	81,543,150	0.03	0.03	0.03	0.00	0.01	0.01	1.22 [1.07-1.40]*	1.19 [1.04-1.36]
G40	65,210	79,984,660	0.89	0.80	0.75	0.09	0.14	0.11	1.19 [1.16-1.22]*	1.14 [1.11-1.17]*
G41	5,580	81,518,390	0.07	0.07	0.07	0.01	0.01	0.01	1.09 [1.00-1.20]	1.07 [0.98-1.18]
G43	65,590	80,732,890	0.91	0.79	0.75	0.12	0.16	0.09	1.21 [1.18-1.25]*	1.12 [1.09-1.15]*
G44	9,500	81,449,610	0.13	0.11	0.11	0.02	0.02	0.02	1.23 [1.15-1.32]*	1.15 [1.07-1.23]*
G45	43,040	81,007,210	0.54	0.53	0.48	0.01	0.06	0.05	1.12 [1.08-1.15]*	1.09 [1.06-1.13]*
G46	2,910	81,589,260	0.04	0.04	0.03	0.00	0.00	0.00	1.15 [1.01-1.30]	1.13 [0.99-1.29]
G47	85,780	80,393,660	1.34	1.00	0.97	0.34	0.37	0.28	1.38 [1.35-1.41]*	1.27 [1.24-1.30]*
G50	7,350	81,509,750	0.10	0.09	0.08	0.02	0.02	0.02	1.32 [1.22-1.42]*	1.22 [1.13-1.32]*
G51	15,430	81,363,460	0.20	0.19	0.17	0.01	0.03	0.02	1.15 [1.09-1.21]*	1.11 [1.05-1.17]*
G52	1,180	81,593,630	0.02	0.01	0.01	0.00	0.00	0.00	1.26 [1.03-1.53]	1.20 [0.98-1.46]
G53	2,550	81,586,630	0.04	0.03	0.03	0.00	0.01	0.01	1.26 [1.10-1.44]*	1.21 [1.05-1.39]*
G54	4,830	81,538,200	0.06	0.06	0.05	0.00	0.01	0.01	1.24 [1.13-1.37]*	1.18 [1.07-1.30]*
G55	52,950	80,844,380	0.67	0.65	0.57	0.02	0.10	0.07	1.18 [1.14-1.21]*	1.11 [1.08-1.15]*
G56	74,220	79,648,020	1.00	0.91	0.81	0.09	0.19	0.14	1.24 [1.21-1.27]*	1.16 [1.13-1.19]*
G57	9,170	81,419,860	0.12	0.11	0.10	0.01	0.02	0.02	1.25 [1.17-1.34]*	1.17 [1.09-1.26]*
G58	4,820	81,529,180	0.06	0.06	0.05	0.01	0.01	0.01	1.24 [1.12-1.36]*	1.16 [1.05-1.28]*
G59	3,930	81,559,050	0.05	0.05	0.04	0.01	0.01	0.01	1.27 [1.14-1.41]*	1.20 [1.07-1.33]*
G60	2,250	81,575,250	0.03	0.03	0.03	0.00	0.00	0.00	1.17 [1.01-1.35]	1.07 [0.93-1.24]
G61	2,540	81,556,420	0.03	0.03	0.03	0.00	0.00	0.00	1.14 [0.99-1.30]	1.10 [0.96-1.26]
G62	30,530	81,359,790	0.41	0.37	0.33	0.04	0.08	0.06	1.23 [1.19-1.28]*	1.17 [1.12-1.22]*
G63	16,580	81,494,480	0.23	0.20	0.17	0.03	0.06	0.04	1.32 [1.26-1.39]*	1.24 [1.18-1.31]*
G70	2,550	81,567,030	0.03	0.03	0.03	0.00	0.00	0.00	1.14 [0.99-1.30]	1.10 [0.95-1.26]
G71	2,280	81,559,740	0.03	0.03	0.03	0.00	0.00	0.00	1.04 [0.90-1.20]	1.03 [0.89-1.19]
G72	3,320	81,570,070	0.05	0.04	0.04	0.01	0.01	0.01	1.28 [1.14-1.43]*	1.23 [1.09-1.38]*
G80	7,370	81,371,710	0.09	0.09	0.10	0.00	−0.01	−0.01	0.92 [0.85-1.00]	0.90 [0.83-0.97]*
G81	52,100	81,045,370	0.66	0.64	0.59	0.02	0.07	0.07	1.12 [1.08-1.15]*	1.11 [1.08-1.15]*
G82	5,750	81,488,740	0.07	0.07	0.07	0.00	0.00	0.00	1.04 [0.95-1.14]	1.03 [0.94-1.13]
G83	8,530	81,509,330	0.10	0.11	0.09	0.00	0.01	0.01	1.09 [1.01-1.18]	1.06 [0.98-1.15]
G90	4,510	81,559,250	0.06	0.05	0.05	0.00	0.01	0.00	1.13 [1.02-1.25]	1.06 [0.95-1.18]
G91	9,760	81,457,550	0.12	0.12	0.11	0.01	0.02	0.02	1.14 [1.06-1.22]*	1.14 [1.06-1.22]*
G93	42,270	81,167,670	0.56	0.51	0.48	0.05	0.08	0.06	1.17 [1.14-1.21]*	1.12 [1.08-1.16]*
G94	1,450	81,595,890	0.02	0.02	0.02	0.00	0.00	0.00	1.11 [0.93-1.32]	1.13 [0.94-1.36]
G95	11,170	81,485,510	0.14	0.14	0.12	0.00	0.01	0.01	1.11 [1.04-1.19]*	1.09 [1.02-1.17]
G96	3,680	81,552,160	0.05	0.04	0.04	0.00	0.01	0.01	1.20 [1.08-1.34]*	1.15 [1.03-1.29]
G97	3,480	81,544,530	0.05	0.04	0.04	0.00	0.00	0.00	1.11 [0.99-1.24]	1.05 [0.94-1.19]
G98	1,320	81,600,510	0.02	0.02	0.01	0.00	0.01	0.00	1.41 [1.18-1.69]*	1.29 [1.07-1.55]*
G99	17,400	81,413,380	0.22	0.21	0.19	0.01	0.04	0.03	1.19 [1.13-1.26]*	1.15 [1.09-1.21]*
hospitalised - H00-H59										
H00	13,330	81,108,730	0.18	0.16	0.16	0.02	0.03	0.02	1.17 [1.11-1.24]*	1.14 [1.08-1.21]*
H01	10,440	81,456,960	0.15	0.12	0.11	0.03	0.04	0.03	1.32 [1.24-1.41]*	1.26 [1.18-1.34]*
H02	53,490	80,471,540	0.72	0.65	0.59	0.07	0.13	0.11	1.22 [1.19-1.26]*	1.17 [1.14-1.21]*
H03	790	81,591,260	0.01	0.01	0.01	0.00	0.00	0.00	1.34 [1.06-1.68]	1.23 [0.97-1.57]
H04	27,450	81,046,430	0.39	0.32	0.30	0.07	0.09	0.08	1.31 [1.26-1.37]*	1.24 [1.19-1.29]*
H05	9,520	81,380,630	0.15	0.11	0.11	0.04	0.04	0.03	1.32 [1.24-1.41]*	1.29 [1.21-1.38]*
H06	1,100	81,588,950	0.02	0.01	0.01	0.00	0.00	0.00	1.37 [1.13-1.67]*	1.34 [1.10-1.63]*
H10	16,230	81,269,940	0.25	0.19	0.18	0.06	0.06	0.05	1.35 [1.28-1.42]*	1.28 [1.22-1.35]*
H11	11,120	81,396,020	0.14	0.13	0.12	0.01	0.02	0.02	1.17 [1.10-1.25]*	1.15 [1.08-1.23]*
H13	890	81,589,830	0.01	0.01	0.01	0.00	0.00	0.00	1.36 [1.10-1.69]*	1.29 [1.03-1.61]
H15	1,070	81,591,570	0.01	0.01	0.01	0.00	0.00	0.00	1.32 [1.08-1.62]*	1.24 [1.01-1.53]
H16	4,970	81,516,860	0.08	0.06	0.05	0.02	0.03	0.03	1.59 [1.45-1.74]*	1.53 [1.39-1.68]*
H17	2,940	81,567,980	0.04	0.03	0.03	0.01	0.01	0.01	1.39 [1.23-1.57]*	1.33 [1.18-1.50]*
H18	19,510	81,388,660	0.28	0.23	0.21	0.05	0.06	0.05	1.29 [1.23-1.36]*	1.24 [1.18-1.30]*
H19	1,250	81,589,910	0.02	0.01	0.01	0.01	0.01	0.01	1.67 [1.40-1.99]*	1.51 [1.26-1.81]*
H20	5,370	81,513,290	0.08	0.06	0.06	0.02	0.02	0.02	1.38 [1.26-1.50]*	1.34 [1.23-1.47]*
H21	8,490	81,512,120	0.11	0.10	0.09	0.01	0.01	0.01	1.15 [1.06-1.24]*	1.12 [1.04-1.21]*
H22	430	81,604,970	0.01	0.01	0.00	0.00	0.00	0.00	1.36 [0.99-1.87]	1.33 [0.96-1.84]
H25	188,180	79,053,140	2.51	2.35	2.23	0.16	0.28	0.22	1.12 [1.11-1.14]*	1.10 [1.08-1.12]*
H26	262,240	77,501,040	3.67	3.31	3.18	0.36	0.49	0.41	1.16 [1.14-1.17]*	1.13 [1.11-1.14]*
H27	3,970	81,535,230	0.05	0.05	0.04	0.00	0.01	0.01	1.15 [1.03-1.28]	1.13 [1.01-1.27]
H28	1,520	81,590,210	0.02	0.02	0.02	0.00	0.00	0.00	1.27 [1.07-1.51]*	1.24 [1.04-1.48]
H30	480	81,600,150	0.01	0.01	0.01	0.00	0.00	0.00	1.34 [1.00-1.81]	1.30 [0.96-1.76]
H31	4,820	81,557,210	0.06	0.06	0.06	0.00	0.00	0.00	1.05 [0.95-1.16]	1.04 [0.94-1.15]
H33	17,000	81,178,500	0.19	0.21	0.19	−0.02	0.01	0.01	1.03 [0.98-1.09]	1.03 [0.97-1.09]
H34	11,420	81,476,640	0.14	0.14	0.13	0.00	0.01	0.01	1.11 [1.04-1.18]*	1.09 [1.02-1.17]
H35	102,790	80,493,180	1.33	1.26	1.18	0.06	0.15	0.12	1.12 [1.10-1.15]*	1.10 [1.08-1.13]*
H36	43,800	81,114,960	0.56	0.53	0.48	0.03	0.08	0.07	1.17 [1.13-1.21]*	1.15 [1.11-1.19]*
H40	91,610	80,457,410	1.18	1.13	1.04	0.05	0.14	0.12	1.13 [1.11-1.16]*	1.11 [1.08-1.14]*
H43	19,620	81,340,400	0.24	0.24	0.22	−0.01	0.02	0.01	1.08 [1.03-1.13]*	1.06 [1.01-1.11]
H44	6,430	81,493,780	0.09	0.08	0.07	0.01	0.02	0.01	1.24 [1.14-1.35]*	1.19 [1.09-1.30]*
H46	1,920	81,575,540	0.03	0.02	0.02	0.00	0.00	0.00	1.22 [1.05-1.42]	1.18 [1.01-1.38]

H47	8,370	81,497,530	0.11	0.10	0.10	0.01	0.01	0.01	1.15 [1.07-1.24]*	1.12 [1.04-1.21]*
H49	5,380	81,529,730	0.06	0.07	0.06	0.00	0.00	0.00	1.02 [0.93-1.13]	1.00 [0.91-1.10]
H50	23,520	80,971,190	0.32	0.28	0.31	0.04	0.01	0.00	1.03 [0.99-1.08]	1.01 [0.97-1.06]
H51	1,050	81,592,920	0.01	0.01	0.01	0.00	0.00	0.00	1.05 [0.85-1.30]	1.00 [0.81-1.24]
H52	33,260	81,265,310	0.42	0.41	0.38	0.01	0.04	0.03	1.09 [1.05-1.13]*	1.07 [1.02-1.11]*
H53	68,880	80,602,830	0.92	0.84	0.79	0.08	0.13	0.09	1.16 [1.13-1.19]*	1.11 [1.08-1.14]*
H54	50,950	81,220,010	0.66	0.62	0.58	0.04	0.08	0.07	1.14 [1.11-1.18]*	1.12 [1.09-1.16]*
H55	5,510	81,533,360	0.07	0.07	0.07	0.00	0.00	0.00	1.06 [0.97-1.17]	1.04 [0.94-1.14]
H57	11,680	81,449,570	0.16	0.14	0.13	0.02	0.03	0.02	1.20 [1.12-1.27]*	1.14 [1.07-1.21]*
H58	2,210	81,586,400	0.03	0.03	0.02	0.00	0.00	0.00	1.16 [1.00-1.35]	1.13 [0.98-1.32]
H59	3,880	81,542,340	0.05	0.05	0.04	0.00	0.00	0.00	1.12 [1.00-1.25]	1.11 [0.99-1.25]
hospitalised - H60-H95										
H60	11,080	81,348,560	0.18	0.13	0.12	0.05	0.06	0.05	1.48 [1.39-1.57]*	1.42 [1.34-1.51]*
H61	21,580	81,159,580	0.33	0.25	0.26	0.08	0.07	0.06	1.27 [1.22-1.33]*	1.24 [1.18-1.30]*
H65	55,860	79,788,940	0.87	0.66	0.75	0.22	0.12	0.10	1.17 [1.14-1.20]*	1.14 [1.11-1.17]*
H66	29,670	80,658,090	0.45	0.35	0.37	0.10	0.08	0.07	1.21 [1.16-1.25]*	1.18 [1.13-1.22]*
H69	2,250	81,558,720	0.03	0.03	0.03	0.01	0.01	0.01	1.32 [1.16-1.52]*	1.25 [1.08-1.43]*
H70	2,840	81,551,950	0.04	0.03	0.03	0.00	0.00	0.00	1.10 [0.97-1.24]	1.07 [0.94-1.22]
H71	6,110	81,436,460	0.08	0.07	0.08	0.01	0.00	0.00	1.04 [0.95-1.13]	1.05 [0.96-1.15]
H72	12,800	81,225,210	0.18	0.15	0.16	0.03	0.02	0.02	1.13 [1.07-1.20]*	1.11 [1.05-1.18]*
H73	9,050	81,384,620	0.13	0.11	0.12	0.03	0.02	0.01	1.14 [1.06-1.22]*	1.12 [1.05-1.20]*
H74	6,640	81,428,060	0.09	0.08	0.08	0.01	0.01	0.01	1.12 [1.03-1.21]*	1.09 [1.00-1.18]
H80	1,230	81,569,180	0.01	0.02	0.01	0.00	0.00	0.00	1.11 [0.92-1.35]	1.09 [0.89-1.33]
H81	20,710	81,392,500	0.27	0.25	0.23	0.02	0.04	0.03	1.17 [1.11-1.23]*	1.11 [1.06-1.16]*
H83	8,560	81,452,320	0.11	0.10	0.09	0.01	0.02	0.01	1.17 [1.09-1.26]*	1.11 [1.03-1.19]*
H90	27,840	81,256,260	0.40	0.33	0.33	0.07	0.07	0.05	1.20 [1.16-1.25]*	1.15 [1.11-1.20]*
H91	130,360	80,566,200	1.77	1.58	1.51	0.19	0.26	0.22	1.18 [1.15-1.20]*	1.14 [1.12-1.16]*
H92	10,520	81,366,910	0.16	0.12	0.12	0.03	0.03	0.03	1.26 [1.18-1.34]*	1.20 [1.13-1.28]*
H93	12,160	81,457,620	0.17	0.14	0.13	0.02	0.04	0.03	1.28 [1.21-1.36]*	1.21 [1.14-1.29]*
H95	1,450	81,571,870	0.02	0.02	0.02	0.00	0.00	0.00	1.12 [0.94-1.33]	1.13 [0.94-1.35]
hospitalised - I00-I99										
I05	6,750	81,517,090	0.09	0.08	0.07	0.01	0.01	0.01	1.19 [1.10-1.30]*	1.16 [1.07-1.26]*
I06	670	81,596,240	0.01	0.01	0.01	0.00	0.00	0.00	1.18 [0.91-1.52]	1.12 [0.86-1.45]
I07	17,640	81,520,100	0.24	0.21	0.19	0.03	0.05	0.04	1.23 [1.17-1.30]*	1.20 [1.14-1.27]*
I08	88,830	81,118,690	1.16	1.08	1.01	0.08	0.15	0.13	1.15 [1.12-1.18]*	1.12 [1.10-1.15]*
I09	1,030	81,596,700	0.01	0.01	0.01	0.00	0.00	0.00	1.37 [1.11-1.68]*	1.33 [1.07-1.64]*
I10	652,690	67,379,090	10.99	9.35	9.37	1.65	1.62	1.38	1.17 [1.16-1.18]*	1.14 [1.13-1.15]*
I11	4,980	81,543,760	0.06	0.06	0.06	0.00	0.00	0.00	1.06 [0.96-1.17]	1.04 [0.94-1.15]
I12	50,020	81,093,880	0.64	0.61	0.54	0.03	0.10	0.10	1.19 [1.16-1.23]*	1.18 [1.14-1.22]*
I13	2,620	81,601,500	0.03	0.03	0.03	0.00	0.01	0.00	1.17 [1.02-1.35]	1.14 [0.99-1.31]
I15	3,920	81,555,910	0.05	0.05	0.04	0.00	0.00	0.00	1.11 [1.00-1.24]	1.09 [0.98-1.22]
I20	176,160	77,429,990	2.43	2.24	2.08	0.20	0.35	0.26	1.17 [1.15-1.19]*	1.12 [1.10-1.14]*
I21	117,600	79,809,470	1.50	1.47	1.35	0.03	0.15	0.13	1.11 [1.09-1.13]*	1.10 [1.07-1.12]*
I22	11,880	81,420,530	0.15	0.14	0.13	0.01	0.02	0.02	1.18 [1.11-1.25]*	1.15 [1.08-1.23]*
I23	650	81,598,080	0.01	0.01	0.01	0.00	0.00	0.00	1.31 [1.02-1.70]	1.29 [1.00-1.68]
I24	37,560	81,297,100	0.49	0.46	0.42	0.03	0.07	0.06	1.17 [1.13-1.21]*	1.14 [1.10-1.18]*
I25	296,940	76,169,720	4.19	3.82	3.67	0.36	0.52	0.41	1.14 [1.13-1.16]*	1.11 [1.10-1.12]*
I26	63,850	80,929,310	0.77	0.79	0.72	−0.02	0.05	0.03	1.07 [1.04-1.10]*	1.04 [1.01-1.07]*
I27	41,440	81,375,400	0.56	0.50	0.45	0.07	0.11	0.09	1.25 [1.21-1.29]*	1.20 [1.16-1.24]*
I28	1,290	81,596,300	0.02	0.02	0.02	0.00	0.00	0.00	0.99 [0.82-1.21]	0.94 [0.77-1.15]
I30	1,900	81,562,200	0.02	0.02	0.02	0.00	0.00	0.00	1.18 [1.01-1.37]	1.15 [0.98-1.34]
I31	25,010	81,357,660	0.31	0.31	0.28	0.01	0.04	0.03	1.13 [1.08-1.18]*	1.10 [1.05-1.15]*
I33	4,130	81,566,040	0.06	0.05	0.04	0.01	0.01	0.01	1.25 [1.13-1.39]*	1.22 [1.10-1.36]*
I34	56,820	81,068,350	0.72	0.70	0.64	0.02	0.08	0.06	1.13 [1.09-1.16]*	1.10 [1.07-1.13]*
I35	82,070	80,919,520	1.08	1.00	0.93	0.08	0.16	0.13	1.17 [1.14-1.20]*	1.13 [1.10-1.16]*
I36	6,360	81,576,210	0.08	0.08	0.07	0.01	0.01	0.01	1.17 [1.07-1.28]*	1.13 [1.04-1.24]*
I37	6,970	81,564,250	0.09	0.08	0.08	0.01	0.01	0.01	1.13 [1.04-1.23]*	1.10 [1.01-1.20]
I38	9,400	81,489,180	0.12	0.11	0.10	0.01	0.02	0.02	1.21 [1.13-1.30]*	1.17 [1.09-1.26]*
I40	620	81,600,160	0.01	0.01	0.01	0.00	0.00	0.00	1.35 [1.04-1.76]	1.33 [1.02-1.75]
I42	21,160	81,333,670	0.27	0.26	0.23	0.01	0.03	0.03	1.15 [1.10-1.20]*	1.11 [1.06-1.16]*
I43	920	81,605,810	0.01	0.01	0.01	0.00	0.00	0.00	0.97 [0.76-1.23]	0.93 [0.73-1.18]
I44	129,810	80,634,060	1.70	1.59	1.47	0.11	0.22	0.19	1.15 [1.13-1.17]*	1.13 [1.10-1.15]*
I45	82,070	80,977,230	1.06	1.00	0.92	0.06	0.14	0.11	1.15 [1.12-1.18]*	1.12 [1.09-1.15]*
I46	41,380	81,459,640	0.53	0.50	0.45	0.03	0.08	0.08	1.18 [1.14-1.22]*	1.17 [1.13-1.21]*
I47	46,510	80,859,610	0.59	0.57	0.52	0.02	0.07	0.05	1.14 [1.10-1.18]*	1.10 [1.06-1.13]*
I48	331,760	78,203,070	4.50	4.18	4.02	0.32	0.48	0.40	1.12 [1.11-1.13]*	1.10 [1.09-1.11]*
I49	56,460	81,011,750	0.72	0.69	0.64	0.02	0.08	0.05	1.12 [1.09-1.15]*	1.08 [1.05-1.11]*
I50	269,500	79,775,020	3.72	3.29	3.09	0.43	0.63	0.54	1.20 [1.19-1.22]*	1.17 [1.15-1.19]*
I51	156,150	80,532,850	2.07	1.91	1.75	0.16	0.31	0.26	1.18 [1.16-1.20]*	1.14 [1.12-1.16]*
I60	8,000	81,472,570	0.10	0.10	0.09	0.00	0.01	0.01	1.10 [1.02-1.18]	1.10 [1.02-1.20]
I61	20,910	81,439,670	0.26	0.26	0.23	0.01	0.03	0.03	1.14 [1.09-1.20]*	1.14 [1.09-1.20]*
I62	12,680	81,515,050	0.16	0.15	0.14	0.00	0.01	0.01	1.10 [1.03-1.17]*	1.10 [1.04-1.18]*
I63	99,330	80,740,600	1.21	1.23	1.14	−0.02	0.08	0.08	1.07 [1.04-1.09]*	1.07 [1.04-1.09]*
I64	24,590	81,239,710	0.31	0.30	0.27	0.00	0.03	0.03	1.12 [1.07-1.17]*	1.12 [1.07-1.17]*
I65	25,030	81,324,150	0.31	0.31	0.28	0.00	0.03	0.03	1.11 [1.06-1.16]*	1.10 [1.05-1.15]*
I66	1,610	81,594,550	0.02	0.02	0.02	0.00	0.00	0.00	1.12 [0.94-1.34]	1.09 [0.91-1.30]
I67	160,410	80,761,490	2.08	1.96	1.83	0.12	0.25	0.23	1.14 [1.12-1.16]*	1.13 [1.11-1.15]*
I68	950	81,606,610	0.01	0.01	0.01	0.00	0.00	0.00	1.25 [1.00-1.56]	1.22 [0.97-1.53]
I69	48,360	81,129,260	0.60	0.60	0.54	0.00	0.05	0.05	1.10 [1.06-1.13]*	1.09 [1.06-1.13]*
I70	40,220	81,273,150	0.53	0.49	0.45	0.04	0.08	0.08	1.18 [1.14-1.22]*	1.17 [1.13-1.21]*
I71	47,430	81,217,710	0.59	0.58	0.53	0.01	0.06	0.06	1.11 [1.07-1.15]*	1.11 [1.08-1.15]*
I72	11,840	81,486,870	0.14	0.15	0.13	0.00	0.01	0.01	1.11 [1.04-1.18]*	1.10 [1.03-1.17]*
I73	87,780	80,614,700	1.24	1.05	0.96	0.18	0.28	0.25	1.29 [1.26-1.32]*	1.25 [1.22-1.28]*
I74	22,460	81,355,910	0.28	0.28	0.25	0.00	0.03	0.03	1.11 [1.06-1.17]*	1.11 [1.06-1.17]*
I77	30,020	81,232,370	0.40	0.36	0.33	0.03	0.07	0.07	1.22 [1.17-1.27]*	1.21 [1.16-1.26]*
I78	7,780	81,493,780	0.10	0.09	0.08	0.01	0.02	0.02	1.24 [1.15-1.34]*	1.21 [1.12-1.31]*
I79	3,170	81,578,080	0.04	0.04	0.03	0.00	0.01	0.01	1.19 [1.06-1.35]*	1.15 [1.02-1.30]
I80	56,610	80,660,280	0.74	0.69	0.63	0.05	0.12	0.10	1.19 [1.15-1.22]*	1.15 [1.12-1.19]*
I81	3,460	81,591,460	0.04	0.04	0.04	0.00	0.00	0.00	1.04 [0.92-1.17]	1.03 [0.91-1.16]
I82	7,740	81,536,300	0.10	0.09	0.09	0.00	0.01	0.01	1.13 [1.04-1.22]*	1.10 [1.02-1.19]
I83	63,260	79,639,390	1.00	0.74	0.66	0.26	0.34	0.33	1.52 [1.48-1.56]*	1.48 [1.45-1.52]*
I84	100,420	78,371,030	1.37	1.26	1.09	0.11	0.28	0.22	1.25 [1.23-1.28]*	1.19 [1.17-1.22]*
I85	9,510	81,505,490	0.13	0.11	0.10	0.02	0.04	0.03	1.37 [1.28-1.47]*	1.34 [1.25-1.43]*
I86	7,570	81,471,110	0.10	0.09	0.08	0.01	0.02	0.02	1.24 [1.15-1.34]*	1.21 [1.12-1.31]*
I87	15,640	81,481,180	0.24	0.18	0.16	0.06	0.08	0.08	1.49 [1.42-1.57]*	1.45 [1.38-1.53]*
I88	11,850	81,361,660	0.19	0.13	0.16	0.06	0.04	0.03	1.23 [1.16-1.31]*	1.20 [1.13-1.28]*
I89	21,140	81,449,470	0.31	0.25	0.22	0.07	0.09	0.08	1.42 [1.35-1.48]*	1.37 [1.31-1.43]*
I95	197,290	80,402,540	2.58	2.42	2.25	0.15	0.33	0.29	1.15 [1.13-1.16]*	1.13 [1.11-1.15]*
I97	2,450	81,550,980	0.03	0.03	0.03	0.00	0.00	0.00	1.17 [1.02-1.34]	1.17 [1.02-1.34]
I98	6,550	81,556,540	0.09	0.08	0.07	0.01	0.02	0.02	1.36 [1.26-1.48]*	1.31 [1.21-1.43]*
I99	3,590	81,568,850	0.04	0.04	0.04	0.00	0.01	0.00	1.13 [1.00-1.27]	1.11 [0.99-1.25]
hospitalised - J00-J99										
J00	12,330	81,270,110	0.20	0.14	0.15	0.06	0.05	0.05	1.36 [1.28-1.44]*	1.31 [1.23-1.39]*
J01	3,070	81,543,010	0.04	0.04	0.03	0.01	0.01	0.01	1.28 [1.14-1.44]*	1.19 [1.05-1.34]*
J02	22,430	81,142,530	0.33	0.26	0.27	0.07	0.06	0.05	1.23 [1.18-1.28]*	1.18 [1.13-1.23]*
J03	108,380	79,148,740	1.70	1.29	1.46	0.42	0.25	0.20	1.17 [1.15-1.19]*	1.14 [1.11-1.16]*

J04	2,750	81,550,670	0.04	0.03	0.03	0.01	0.01	0.01	1.33 [1.17-1.50]*	1.23 [1.08-1.40]*
J05	20,010	80,887,380	0.27	0.24	0.27	0.02	0.00	−0.01	0.98 [0.94-1.03]	0.97 [0.92-1.02]
J06	76,210	78,184,710	1.23	0.91	1.00	0.32	0.24	0.20	1.24 [1.21-1.27]*	1.19 [1.17-1.22]*
J10	19,480	81,483,750	0.28	0.23	0.22	0.05	0.06	0.04	1.28 [1.22-1.34]*	1.18 [1.13-1.24]*
J11	3,970	81,536,410	0.06	0.05	0.04	0.01	0.01	0.01	1.26 [1.13-1.40]*	1.18 [1.06-1.32]*
J12	29,960	81,533,820	0.41	0.36	0.34	0.06	0.08	0.06	1.23 [1.18-1.28]*	1.17 [1.12-1.22]*
J13	6,360	81,528,740	0.09	0.08	0.07	0.01	0.02	0.01	1.27 [1.17-1.38]*	1.19 [1.09-1.29]*
J14	2,750	81,581,780	0.04	0.03	0.03	0.00	0.01	0.00	1.23 [1.08-1.40]*	1.11 [0.97-1.26]
J15	14,180	81,453,670	0.20	0.17	0.15	0.04	0.05	0.04	1.32 [1.24-1.39]*	1.23 [1.16-1.30]*
J17	2,820	81,583,460	0.04	0.03	0.03	0.01	0.01	0.01	1.35 [1.20-1.53]*	1.28 [1.13-1.45]*
J18	402,130	78,500,770	5.65	4.99	4.67	0.66	0.98	0.83	1.21 [1.20-1.22]*	1.17 [1.16-1.18]*
J20	5,620	81,499,840	0.08	0.07	0.06	0.01	0.01	0.01	1.22 [1.12-1.34]*	1.15 [1.05-1.26]*
J21	4,670	79,843,560	0.07	0.06	0.06	0.01	0.01	0.00	1.09 [0.99-1.20]	1.03 [0.93-1.13]
J22	263,300	77,974,730	3.95	3.23	3.09	0.72	0.87	0.71	1.28 [1.26-1.30]*	1.22 [1.20-1.23]*
J30	45,100	81,034,840	0.95	0.46	0.47	0.49	0.48	0.38	2.03 [1.98-2.09]*	1.66 [1.61-1.71]*
J31	10,340	81,371,670	0.18	0.11	0.11	0.06	0.07	0.05	1.62 [1.52-1.72]*	1.34 [1.26-1.43]*
J32	24,280	81,027,590	0.35	0.29	0.26	0.06	0.09	0.05	1.32 [1.27-1.38]*	1.17 [1.12-1.22]*
J33	14,260	81,018,130	0.21	0.17	0.15	0.04	0.06	0.04	1.42 [1.35-1.50]*	1.20 [1.14-1.27]*
J34	51,120	79,591,450	0.80	0.60	0.57	0.19	0.22	0.17	1.39 [1.35-1.43]*	1.26 [1.23-1.30]*
J35	79,650	78,709,280	1.33	0.93	1.07	0.39	0.26	0.22	1.24 [1.21-1.27]*	1.20 [1.17-1.23]*
J36	8,570	81,334,380	0.11	0.10	0.10	0.01	0.01	0.01	1.07 [1.00-1.15]	1.06 [0.99-1.15]
J37	580	81,589,640	0.01	0.01	0.01	0.00	0.00	0.00	1.19 [0.90-1.58]	1.09 [0.82-1.45]
J38	18,970	81,182,600	0.25	0.23	0.21	0.02	0.04	0.03	1.21 [1.15-1.27]*	1.15 [1.09-1.21]*
J39	10,390	81,428,470	0.14	0.12	0.12	0.02	0.02	0.02	1.20 [1.12-1.28]*	1.12 [1.05-1.20]*
J40	11,310	81,436,530	0.16	0.13	0.12	0.02	0.04	0.03	1.30 [1.23-1.39]*	1.19 [1.12-1.27]*
J41	540	81,602,340	0.01	0.01	0.01	0.00	0.00	0.00	1.18 [0.88-1.57]	1.13 [0.84-1.52]
J42	4,580	81,539,790	0.06	0.05	0.05	0.01	0.02	0.01	1.31 [1.19-1.44]*	1.17 [1.05-1.29]*
J43	62,250	81,136,800	0.85	0.75	0.67	0.11	0.19	0.15	1.28 [1.25-1.32]*	1.21 [1.18-1.25]*
J44	237,140	79,075,120	3.51	2.87	2.59	0.64	0.93	0.64	1.36 [1.34-1.38]*	1.22 [1.21-1.24]*
J45	385,090	69,544,600	8.73	4.73	4.51	3.99	4.21	2.96	1.93 [1.91-1.95]*	1.51 [1.50-1.53]*
J46	8,330	81,244,880	0.21	0.08	0.08	0.13	0.13	0.10	2.59 [2.43-2.76]*	2.01 [1.88-2.15]*
J47	46,340	81,234,330	0.69	0.54	0.49	0.15	0.19	0.12	1.39 [1.35-1.44]*	1.22 [1.18-1.26]*
J61	6,350	81,569,760	0.09	0.08	0.07	0.01	0.02	0.02	1.32 [1.21-1.43]*	1.25 [1.15-1.37]*
J67	1,780	81,594,100	0.03	0.02	0.02	0.01	0.01	0.01	1.44 [1.24-1.67]*	1.30 [1.11-1.52]*
J69	61,760	81,434,640	0.79	0.75	0.70	0.04	0.09	0.09	1.12 [1.09-1.16]*	1.13 [1.10-1.16]*
J70	1,280	81,602,840	0.02	0.01	0.01	0.00	0.00	0.00	1.35 [1.13-1.62]*	1.28 [1.06-1.54]*
J80	4,710	81,571,130	0.06	0.06	0.05	0.01	0.01	0.01	1.27 [1.15-1.40]*	1.25 [1.13-1.38]*
J81	20,970	81,448,390	0.27	0.25	0.23	0.02	0.04	0.04	1.19 [1.14-1.25]*	1.17 [1.11-1.23]*
J82	1,730	81,598,430	0.04	0.02	0.02	0.02	0.02	0.01	2.24 [1.93-2.59]*	1.69 [1.44-1.98]*
J84	38,840	81,407,170	0.57	0.46	0.41	0.11	0.15	0.14	1.37 [1.32-1.42]*	1.32 [1.27-1.36]*
J85	2,070	81,589,020	0.03	0.02	0.02	0.00	0.01	0.00	1.23 [1.06-1.42]*	1.18 [1.01-1.37]
J86	7,140	81,514,590	0.09	0.09	0.08	0.00	0.01	0.01	1.13 [1.05-1.23]*	1.13 [1.04-1.23]*
J90	193,490	80,481,220	2.51	2.38	2.21	0.13	0.31	0.28	1.14 [1.12-1.16]*	1.12 [1.11-1.14]*
J91	4,560	81,588,560	0.06	0.05	0.05	0.01	0.01	0.01	1.20 [1.09-1.34]*	1.19 [1.08-1.32]*
J92	22,160	81,485,720	0.29	0.27	0.24	0.03	0.05	0.05	1.21 [1.15-1.27]*	1.19 [1.13-1.25]*
J93	17,710	81,364,400	0.22	0.22	0.20	0.00	0.02	0.01	1.08 [1.02-1.14]*	1.07 [1.01-1.12]
J94	7,400	81,556,750	0.09	0.09	0.08	0.00	0.01	0.01	1.09 [1.01-1.18]	1.08 [0.99-1.17]
J95	5,870	81,509,350	0.07	0.07	0.06	0.00	0.01	0.01	1.11 [1.01-1.21]	1.10 [1.00-1.20]
J96	127,850	81,090,280	1.74	1.54	1.40	0.20	0.34	0.27	1.24 [1.22-1.26]*	1.18 [1.16-1.21]*
J98	122,090	80,669,870	1.62	1.49	1.37	0.13	0.25	0.19	1.18 [1.16-1.21]*	1.14 [1.11-1.16]*
J99	3,880	81,575,380	0.06	0.04	0.04	0.01	0.02	0.01	1.44 [1.30-1.60]*	1.28 [1.15-1.42]*
hospitalised - K00-K95										
K00	20,070	81,116,610	0.31	0.23	0.28	0.08	0.03	0.02	1.09 [1.05-1.15]*	1.07 [1.02-1.13]*
K01	44,940	79,720,850	0.65	0.54	0.57	0.10	0.08	0.06	1.13 [1.10-1.17]*	1.10 [1.06-1.13]*
K02	155,560	77,937,500	2.23	1.94	2.12	0.29	0.10	0.06	1.05 [1.03-1.07]*	1.03 [1.01-1.05]*
K03	4,190	81,543,490	0.06	0.05	0.06	0.01	0.01	0.00	1.11 [1.00-1.23]	1.07 [0.97-1.19]
K04	35,360	80,753,310	0.46	0.43	0.43	0.03	0.03	0.02	1.07 [1.03-1.11]*	1.04 [1.00-1.08]
K05	29,470	80,848,610	0.40	0.36	0.34	0.04	0.06	0.05	1.19 [1.15-1.24]*	1.15 [1.10-1.19]*
K06	4,340	81,528,490	0.06	0.05	0.05	0.01	0.01	0.01	1.24 [1.12-1.37]*	1.19 [1.07-1.32]*
K07	28,280	80,772,930	0.42	0.33	0.38	0.09	0.05	0.04	1.12 [1.08-1.17]*	1.09 [1.05-1.14]*
K08	49,670	80,505,860	0.66	0.61	0.59	0.06	0.07	0.05	1.12 [1.08-1.15]*	1.08 [1.05-1.12]*
K09	3,380	81,489,100	0.05	0.04	0.04	0.00	0.01	0.01	1.17 [1.05-1.32]*	1.15 [1.02-1.29]
K10	5,280	81,530,860	0.07	0.06	0.06	0.00	0.01	0.01	1.15 [1.04-1.26]*	1.10 [1.00-1.21]
K11	11,490	81,351,840	0.16	0.14	0.13	0.02	0.03	0.03	1.24 [1.16-1.32]*	1.19 [1.12-1.27]*
K12	18,940	81,355,520	0.27	0.22	0.22	0.04	0.05	0.04	1.23 [1.17-1.29]*	1.20 [1.14-1.26]*
K13	23,250	81,082,660	0.34	0.27	0.26	0.06	0.08	0.07	1.30 [1.25-1.36]*	1.26 [1.20-1.31]*
K14	12,090	81,387,660	0.17	0.14	0.13	0.02	0.04	0.03	1.29 [1.21-1.37]*	1.21 [1.14-1.29]*
K20	74,210	80,000,990	1.00	0.91	0.82	0.09	0.19	0.13	1.23 [1.20-1.26]*	1.15 [1.12-1.18]*
K21	293,970	76,908,000	4.20	3.73	3.37	0.48	0.84	0.54	1.25 [1.23-1.26]*	1.15 [1.13-1.16]*
K22	108,790	79,821,530	1.45	1.34	1.20	0.11	0.25	0.19	1.21 [1.18-1.23]*	1.15 [1.13-1.17]*
K23	1,370	81,599,500	0.02	0.02	0.01	0.00	0.01	0.01	1.47 [1.23-1.75]*	1.33 [1.11-1.60]*
K25	53,830	80,693,520	0.71	0.66	0.59	0.05	0.12	0.10	1.21 [1.17-1.24]*	1.16 [1.13-1.20]*
K26	37,060	80,917,980	0.47	0.46	0.41	0.01	0.06	0.06	1.15 [1.11-1.19]*	1.14 [1.10-1.18]*
K27	7,060	81,498,750	0.09	0.09	0.08	0.00	0.01	0.01	1.18 [1.09-1.28]*	1.13 [1.04-1.23]*
K28	1,050	81,597,060	0.02	0.01	0.01	0.00	0.00	0.00	1.43 [1.17-1.75]*	1.29 [1.05-1.59]
K29	274,710	76,266,830	3.92	3.52	3.21	0.40	0.71	0.54	1.22 [1.21-1.24]*	1.16 [1.15-1.18]*
K30	65,390	79,576,250	0.89	0.81	0.71	0.08	0.18	0.13	1.25 [1.22-1.28]*	1.16 [1.13-1.20]*
K31	97,970	80,488,750	1.30	1.20	1.08	0.10	0.22	0.15	1.21 [1.18-1.23]*	1.13 [1.10-1.15]*
K35	47,670	80,165,410	0.62	0.59	0.61	0.03	0.01	0.00	1.01 [0.98-1.04]	0.99 [0.96-1.03]
K36	1,100	81,581,340	0.01	0.01	0.01	0.00	0.00	0.00	1.15 [0.94-1.40]	1.12 [0.91-1.37]
K37	9,380	81,349,570	0.13	0.11	0.12	0.02	0.01	0.00	1.06 [0.99-1.13]	1.02 [0.95-1.09]
K38	6,680	81,476,850	0.09	0.08	0.08	0.01	0.00	0.00	1.06 [0.98-1.15]	1.04 [0.95-1.13]
K40	93,390	78,560,130	1.19	1.19	1.12	0.00	0.07	0.04	1.06 [1.04-1.09]*	1.04 [1.02-1.06]*
K41	7,330	81,459,960	0.09	0.09	0.08	0.00	0.01	0.01	1.12 [1.03-1.21]*	1.07 [0.98-1.16]
K42	48,630	80,553,380	0.65	0.59	0.54	0.05	0.11	0.07	1.20 [1.17-1.24]*	1.13 [1.09-1.16]*
K43	40,740	80,887,220	0.52	0.50	0.44	0.02	0.08	0.06	1.18 [1.14-1.22]*	1.12 [1.09-1.16]*
K44	264,170	76,819,440	3.71	3.37	3.05	0.34	0.66	0.45	1.22 [1.20-1.23]*	1.14 [1.12-1.15]*
K45	1,890	81,584,640	0.02	0.02	0.02	0.00	0.00	0.00	1.12 [0.96-1.32]	1.04 [0.88-1.23]
K46	7,950	81,542,930	0.10	0.10	0.09	0.01	0.02	0.01	1.20 [1.11-1.30]*	1.12 [1.04-1.21]*
K50	20,250	81,049,270	0.33	0.23	0.21	0.09	0.12	0.09	1.55 [1.48-1.62]*	1.38 [1.32-1.45]*
K51	28,680	80,863,330	0.42	0.34	0.31	0.08	0.11	0.08	1.36 [1.31-1.42]*	1.25 [1.20-1.30]*
K52	157,320	76,930,080	2.26	1.99	1.81	0.26	0.45	0.37	1.25 [1.23-1.27]*	1.20 [1.18-1.22]*
K55	24,820	81,412,770	0.32	0.30	0.28	0.02	0.04	0.04	1.15 [1.10-1.21]*	1.13 [1.08-1.19]*
K56	75,850	80,772,830	0.97	0.93	0.85	0.03	0.12	0.09	1.14 [1.11-1.17]*	1.10 [1.07-1.13]*
K57	304,890	77,511,870	4.12	3.89	3.53	0.23	0.59	0.42	1.17 [1.15-1.18]*	1.11 [1.10-1.13]*
K58	92,310	80,313,960	1.38	1.09	0.99	0.28	0.38	0.25	1.39 [1.36-1.42]*	1.22 [1.19-1.25]*
K59	309,970	78,138,110	4.39	3.86	3.67	0.53	0.73	0.58	1.20 [1.18-1.21]*	1.15 [1.14-1.17]*
K60	25,530	80,789,230	0.38	0.30	0.27	0.08	0.11	0.10	1.42 [1.37-1.48]*	1.35 [1.30-1.41]*
K61	15,240	81,109,590	0.21	0.18	0.17	0.03	0.05	0.04	1.29 [1.22-1.36]*	1.26 [1.19-1.33]*
K62	157,110	77,845,850	2.16	1.98	1.76	0.17	0.40	0.32	1.22 [1.20-1.25]*	1.17 [1.15-1.19]*
K63	166,330	79,651,920	2.18	2.07	1.83	0.11	0.34	0.26	1.19 [1.17-1.21]*	1.14 [1.12-1.16]*
K64	121,010	81,082,530	1.61	1.46	1.31	0.15	0.30	0.22	1.22 [1.20-1.25]*	1.16 [1.14-1.18]*
K65	17,640	81,428,450	0.22	0.22	0.19	0.00	0.02	0.02	1.12 [1.07-1.18]*	1.11 [1.06-1.17]*
K66	64,960	80,792,520	0.81	0.80	0.73	0.01	0.08	0.06	1.11 [1.08-1.14]*	1.08 [1.05-1.11]*
K70	20,580	81,331,930	0.28	0.25	0.21	0.04	0.07	0.07	1.35 [1.29-1.41]*	1.34 [1.28-1.41]*
K71	1,420	81,591,530	0.02	0.02	0.02	0.00	0.00	0.00	1.18 [0.98-1.41]	1.16 [0.97-1.39]
K72	13,290	81,546,100	0.18	0.16	0.14	0.02	0.04	0.04	1.30 [1.23-1.38]*	1.27 [1.20-1.35]*
K73	1,310	81,577,450	0.02	0.02	0.01	0.00	0.01	0.00	1.40 [1.17-1.67]*	1.32 [1.10-1.59]*

K74	20,290	81,438,270	0.29	0.24	0.21	0.05	0.08	0.07	1.37 [1.31-1.43]*	1.32 [1.26-1.38]*
K75	13,090	81,470,520	0.19	0.15	0.14	0.03	0.05	0.04	1.33 [1.26-1.41]*	1.28 [1.20-1.35]*
K76	96,960	80,852,320	1.31	1.17	1.05	0.13	0.26	0.20	1.25 [1.22-1.28]*	1.19 [1.16-1.21]*
K80	172,840	78,512,740	2.19	2.20	2.00	−0.01	0.19	0.13	1.10 [1.08-1.12]*	1.06 [1.04-1.08]*
K81	32,140	81,008,170	0.40	0.40	0.35	0.01	0.05	0.04	1.13 [1.09-1.18]*	1.10 [1.05-1.14]*
K82	24,800	81,230,060	0.30	0.31	0.27	0.00	0.03	0.02	1.11 [1.06-1.16]*	1.07 [1.02-1.12]*
K83	46,670	81,107,480	0.58	0.57	0.52	0.01	0.06	0.05	1.12 [1.09-1.16]*	1.10 [1.06-1.14]*
K85	30,510	81,088,150	0.38	0.38	0.33	0.00	0.04	0.04	1.13 [1.09-1.18]*	1.10 [1.06-1.15]*
K86	26,820	81,342,850	0.34	0.33	0.30	0.01	0.04	0.03	1.14 [1.10-1.19]*	1.11 [1.07-1.16]*
K90	25,860	81,075,820	0.46	0.28	0.28	0.18	0.18	0.15	1.67 [1.61-1.73]*	1.49 [1.43-1.55]*
K91	24,720	81,262,820	0.32	0.30	0.27	0.02	0.05	0.04	1.19 [1.14-1.24]*	1.13 [1.09-1.18]*
K92	207,230	79,207,000	2.80	2.57	2.34	0.23	0.46	0.38	1.20 [1.18-1.22]*	1.16 [1.14-1.17]*
hospitalised - L00-L99										
L00	540	81,568,910	0.01	0.01	0.01	0.00	0.00	0.00	1.36 [1.04-1.77]	1.33 [1.01-1.74]
L01	3,140	81,487,780	0.07	0.03	0.03	0.04	0.04	0.04	2.16 [1.95-2.39]*	2.01 [1.81-2.23]*
L02	43,330	80,534,190	0.61	0.52	0.49	0.09	0.13	0.12	1.26 [1.22-1.30]*	1.23 [1.19-1.27]*
L03	158,270	79,318,120	2.54	1.86	1.70	0.68	0.84	0.78	1.49 [1.47-1.52]*	1.44 [1.42-1.46]*
L04	4,470	81,501,380	0.07	0.05	0.06	0.01	0.01	0.01	1.16 [1.06-1.28]*	1.15 [1.05-1.27]*
L05	12,140	81,015,590	0.16	0.15	0.15	0.02	0.01	0.01	1.09 [1.02-1.16]*	1.09 [1.02-1.16]
L08	44,280	81,095,370	0.68	0.51	0.47	0.17	0.21	0.20	1.45 [1.41-1.50]*	1.42 [1.38-1.47]*
L10	500	81,603,670	0.01	0.01	0.00	0.00	0.00	0.00	2.00 [1.52-2.64]*	1.86 [1.40-2.46]*
L11	410	81,603,630	0.01	0.00	0.00	0.00	0.00	0.00	1.64 [1.20-2.25]*	1.58 [1.15-2.17]*
L12	3,500	81,583,340	0.08	0.03	0.03	0.04	0.05	0.05	2.64 [2.37-2.93]*	2.56 [2.30-2.85]*
L13	610	81,598,590	0.01	0.01	0.01	0.01	0.01	0.01	2.83 [2.23-3.59]*	2.63 [2.06-3.35]*
L20	6,010	81,313,990	0.32	0.01	0.01	0.31	0.31	0.30	22.58 [20.37-25.03]*	19.70 [17.74-21.86]*
L21	1,740	81,550,310	0.04	0.02	0.01	0.03	0.03	0.03	2.86 [2.48-3.29]*	2.72 [2.36-3.15]*
L22	1,990	81,502,350	0.03	0.02	0.02	0.00	0.00	0.00	1.01 [0.86-1.18]	0.99 [0.84-1.16]
L23	2,010	81,557,180	0.06	0.02	0.02	0.04	0.04	0.04	3.34 [2.94-3.78]*	3.01 [2.64-3.42]*
L24	10,460	81,588,620	0.16	0.12	0.11	0.03	0.04	0.04	1.36 [1.27-1.45]*	1.34 [1.25-1.44]*
L25	1,880	81,576,020	0.04	0.02	0.02	0.03	0.03	0.03	2.69 [2.35-3.08]*	2.61 [2.27-2.99]*
L26	190	81,607,090	0.01	0.00	0.00	0.01	0.01	0.01	6.43 [4.17-9.91]*	6.03 [3.88-9.38]*
L27	9,970	81,459,700	0.17	0.11	0.10	0.06	0.07	0.06	1.62 [1.52-1.73]*	1.53 [1.43-1.63]*
L28	3,240	81,544,760	0.09	0.03	0.02	0.07	0.07	0.07	3.75 [3.39-4.14]*	3.51 [3.17-3.89]*
L29	16,050	81,299,320	0.27	0.18	0.17	0.09	0.10	0.09	1.59 [1.51-1.67]*	1.50 [1.42-1.58]*
L30	95,050	79,174,070	4.24	0.47	0.46	3.78	3.78	3.74	9.24 [9.06-9.43]*	8.55 [8.37-8.72]*
L40	37,810	81,058,980	0.74	0.40	0.35	0.34	0.39	0.38	2.11 [2.04-2.17]*	2.04 [1.98-2.11]*
L42	660	81,600,970	0.01	0.01	0.01	0.00	0.00	0.00	1.42 [1.10-1.83]*	1.32 [1.02-1.72]
L43	4,160	81,526,160	0.08	0.04	0.04	0.03	0.04	0.04	1.99 [1.81-2.18]*	1.89 [1.72-2.08]*
L50	11,590	81,366,990	0.23	0.12	0.12	0.11	0.11	0.09	1.89 [1.79-2.00]*	1.60 [1.51-1.70]*
L51	2,740	81,533,400	0.04	0.03	0.03	0.01	0.01	0.01	1.24 [1.10-1.40]*	1.18 [1.05-1.34]*
L52	1,060	81,581,960	0.02	0.01	0.01	0.00	0.00	0.00	1.36 [1.12-1.66]*	1.24 [1.01-1.52]
L53	14,760	81,409,400	0.25	0.17	0.15	0.08	0.09	0.09	1.61 [1.53-1.69]*	1.56 [1.47-1.64]*
L55	460	81,600,140	0.01	0.01	0.01	0.00	0.00	0.00	1.12 [0.81-1.53]	1.10 [0.79-1.52]
L56	560	81,598,890	0.01	0.01	0.01	0.01	0.01	0.01	2.36 [1.85-3.00]*	2.22 [1.73-2.86]*
L57	31,910	81,190,610	0.43	0.38	0.35	0.05	0.09	0.07	1.25 [1.20-1.30]*	1.20 [1.15-1.24]*
L59	470	81,603,390	0.01	0.01	0.01	0.00	0.00	0.00	1.21 [0.89-1.65]	1.25 [0.91-1.72]
L60	11,930	81,135,920	0.18	0.14	0.14	0.04	0.05	0.04	1.33 [1.25-1.41]*	1.24 [1.17-1.32]*
L63	630	81,599,290	0.02	0.01	0.01	0.01	0.01	0.01	2.70 [2.15-3.40]*	2.19 [1.71-2.79]*
L65	2,280	81,578,110	0.04	0.02	0.02	0.02	0.02	0.02	1.89 [1.66-2.15]*	1.68 [1.47-1.92]*
L66	460	81,601,690	0.01	0.00	0.00	0.00	0.00	0.00	1.72 [1.29-2.29]*	1.62 [1.21-2.17]*
L68	760	81,588,530	0.01	0.01	0.01	0.00	0.00	0.00	1.10 [0.86-1.41]	1.05 [0.82-1.35]
L70	2,830	81,569,000	0.04	0.03	0.03	0.01	0.01	0.01	1.29 [1.14-1.46]*	1.21 [1.07-1.37]*
L71	3,200	81,570,380	0.05	0.04	0.03	0.01	0.02	0.01	1.50 [1.34-1.68]*	1.43 [1.27-1.60]*
L72	43,240	79,909,640	0.53	0.54	0.49	−0.01	0.04	0.03	1.09 [1.06-1.13]*	1.07 [1.03-1.10]*
L73	6,240	81,455,450	0.09	0.07	0.07	0.02	0.03	0.02	1.37 [1.26-1.49]*	1.31 [1.20-1.42]*
L80	1,440	81,591,570	0.03	0.02	0.01	0.01	0.01	0.01	1.83 [1.56-2.15]*	1.73 [1.46-2.04]*
L81	7,390	81,450,080	0.10	0.09	0.08	0.01	0.02	0.02	1.24 [1.15-1.34]*	1.19 [1.10-1.29]*
L82	20,650	81,194,070	0.26	0.25	0.22	0.01	0.04	0.03	1.18 [1.13-1.24]*	1.13 [1.07-1.18]*
L83	1,400	81,583,830	0.02	0.02	0.02	0.01	0.01	0.01	1.55 [1.31-1.83]*	1.46 [1.23-1.74]*
L84	2,480	81,563,320	0.04	0.03	0.03	0.01	0.01	0.01	1.32 [1.16-1.51]*	1.26 [1.10-1.44]*
L85	9,000	81,475,470	0.14	0.10	0.09	0.04	0.05	0.05	1.56 [1.46-1.67]*	1.51 [1.41-1.62]*
L88	590	81,602,150	0.01	0.01	0.01	0.00	0.00	0.00	1.33 [1.02-1.74]	1.21 [0.92-1.60]
L89	115,450	81,284,880	1.54	1.39	1.30	0.14	0.24	0.24	1.18 [1.16-1.21]*	1.18 [1.16-1.21]*
L90	23,090	81,008,110	0.31	0.28	0.25	0.03	0.05	0.04	1.20 [1.15-1.26]*	1.16 [1.11-1.21]*
L91	7,930	81,322,600	0.11	0.09	0.09	0.02	0.02	0.02	1.22 [1.13-1.32]*	1.18 [1.09-1.27]*
L92	6,690	81,429,620	0.09	0.08	0.08	0.01	0.02	0.01	1.21 [1.11-1.31]*	1.15 [1.06-1.25]*
L93	2,610	81,568,110	0.04	0.03	0.03	0.01	0.02	0.02	1.68 [1.49-1.90]*	1.55 [1.36-1.75]*
L94	1,870	81,577,330	0.03	0.02	0.02	0.01	0.01	0.01	1.46 [1.26-1.69]*	1.37 [1.18-1.59]*
L95	2,240	81,575,110	0.04	0.03	0.02	0.01	0.01	0.01	1.51 [1.32-1.73]*	1.42 [1.23-1.63]*
L97	81,580	81,066,480	1.24	0.95	0.85	0.29	0.39	0.37	1.45 [1.42-1.49]*	1.43 [1.40-1.46]*
L98	66,300	80,323,000	0.94	0.80	0.72	0.15	0.22	0.20	1.30 [1.27-1.34]*	1.26 [1.23-1.29]*
hospitalised - M00-M99										
M00	8,550	81,467,400	0.12	0.10	0.09	0.02	0.03	0.03	1.35 [1.26-1.45]*	1.31 [1.22-1.41]*
M02	2,050	81,569,700	0.03	0.02	0.02	0.00	0.00	0.00	1.17 [1.01-1.35]	1.14 [0.98-1.32]
M05	9,850	81,467,650	0.13	0.12	0.11	0.01	0.03	0.02	1.24 [1.16-1.32]*	1.21 [1.13-1.29]*
M06	68,390	80,587,150	0.96	0.82	0.73	0.14	0.23	0.18	1.31 [1.28-1.34]*	1.24 [1.21-1.27]*
M07	8,350	81,482,310	0.13	0.09	0.08	0.04	0.05	0.04	1.60 [1.49-1.71]*	1.50 [1.40-1.61]*
M08	2,740	81,540,120	0.04	0.03	0.04	0.01	0.01	0.01	1.25 [1.10-1.41]*	1.23 [1.08-1.39]*
M10	79,130	80,936,360	1.08	0.95	0.85	0.13	0.23	0.20	1.27 [1.24-1.30]*	1.22 [1.19-1.25]*
M11	9,170	81,519,410	0.12	0.11	0.10	0.01	0.03	0.02	1.26 [1.17-1.35]*	1.20 [1.12-1.29]*
M12	1,980	81,575,720	0.03	0.02	0.02	0.00	0.01	0.00	1.25 [1.08-1.45]*	1.21 [1.04-1.40]
M13	201,300	79,359,340	2.82	2.47	2.24	0.35	0.57	0.44	1.26 [1.24-1.27]*	1.18 [1.17-1.20]*
M14	2,380	81,580,580	0.03	0.03	0.02	0.00	0.01	0.01	1.32 [1.15-1.51]*	1.25 [1.09-1.43]*
M15	76,670	80,825,760	1.05	0.92	0.83	0.13	0.22	0.17	1.27 [1.24-1.30]*	1.19 [1.16-1.22]*
M16	114,100	79,576,170	1.40	1.44	1.31	−0.04	0.09	0.06	1.07 [1.05-1.09]*	1.05 [1.03-1.07]*
M17	188,250	78,007,630	2.52	2.39	2.15	0.14	0.37	0.27	1.17 [1.15-1.19]*	1.12 [1.10-1.14]*
M18	11,450	81,444,030	0.16	0.14	0.12	0.02	0.03	0.02	1.28 [1.20-1.36]*	1.17 [1.10-1.25]*
M19	315,810	78,559,980	4.49	3.90	3.57	0.59	0.93	0.71	1.26 [1.24-1.27]*	1.19 [1.17-1.20]*
M20	43,580	80,353,420	0.58	0.53	0.48	0.04	0.09	0.07	1.19 [1.16-1.23]*	1.14 [1.10-1.17]*
M21	28,510	81,198,690	0.37	0.35	0.33	0.02	0.04	0.03	1.11 [1.07-1.16]*	1.07 [1.03-1.12]*
M22	8,240	81,310,780	0.11	0.10	0.09	0.01	0.02	0.02	1.26 [1.17-1.36]*	1.17 [1.09-1.26]*
M23	78,520	78,921,090	0.99	1.00	0.88	0.00	0.11	0.06	1.12 [1.10-1.15]*	1.07 [1.04-1.09]*
M24	35,360	80,836,880	0.46	0.43	0.40	0.03	0.06	0.04	1.15 [1.11-1.19]*	1.10 [1.06-1.14]*
M25	209,400	78,278,930	2.91	2.62	2.43	0.29	0.48	0.37	1.20 [1.18-1.22]*	1.14 [1.13-1.16]*
M30	1,800	81,567,190	0.03	0.02	0.02	0.01	0.01	0.01	1.32 [1.13-1.53]*	1.23 [1.06-1.43]*
M31	11,030	81,477,980	0.15	0.13	0.12	0.02	0.03	0.02	1.25 [1.17-1.33]*	1.18 [1.10-1.26]*
M32	5,260	81,502,600	0.09	0.06	0.05	0.03	0.03	0.03	1.62 [1.49-1.76]*	1.49 [1.36-1.63]*
M33	1,060	81,588,360	0.02	0.01	0.01	0.01	0.01	0.01	1.90 [1.58-2.29]*	1.81 [1.50-2.19]*
M34	2,070	81,574,960	0.03	0.02	0.02	0.01	0.01	0.01	1.55 [1.35-1.78]*	1.46 [1.26-1.68]*
M35	52,950	81,063,260	0.78	0.62	0.58	0.16	0.20	0.16	1.35 [1.31-1.39]*	1.27 [1.23-1.30]*
M36	530	81,600,460	0.01	0.01	0.01	0.00	0.00	0.00	1.29 [0.97-1.73]	1.24 [0.92-1.67]
M40	13,350	81,531,760	0.18	0.16	0.15	0.02	0.03	0.02	1.18 [1.11-1.25]*	1.13 [1.06-1.20]*
M41	37,300	81,254,980	0.50	0.45	0.44	0.05	0.06	0.04	1.14 [1.10-1.18]*	1.09 [1.05-1.13]*
M42	730	81,600,300	0.01	0.01	0.01	0.00	0.00	0.00	1.14 [0.89-1.46]	1.02 [0.79-1.32]
M43	30,620	81,228,660	0.40	0.37	0.34	0.03	0.06	0.04	1.18 [1.13-1.22]*	1.10 [1.06-1.15]*
M45	6,740	81,493,240	0.10	0.08	0.07	0.02	0.03	0.02	1.42 [1.31-1.54]*	1.33 [1.23-1.44]*

M46	24,550	81,341,170	0.34	0.29	0.26	0.05	0.08	0.06	1.29 [1.24-1.35]*	1.20 [1.15-1.26]*
M47	136,980	80,068,320	1.88	1.67	1.50	0.21	0.38	0.29	1.26 [1.23-1.28]*	1.18 [1.16-1.20]*
M48	80,880	80,800,370	1.06	0.99	0.89	0.07	0.17	0.13	1.20 [1.17-1.23]*	1.14 [1.11-1.16]*
M49	2,660	81,594,130	0.03	0.03	0.03	0.00	0.00	0.00	1.08 [0.94-1.24]	1.09 [0.95-1.25]
M50	21,390	81,338,810	0.28	0.26	0.23	0.02	0.05	0.04	1.21 [1.16-1.27]*	1.15 [1.09-1.20]*
M51	100,560	80,026,400	1.32	1.24	1.10	0.08	0.22	0.15	1.20 [1.18-1.23]*	1.13 [1.11-1.16]*
M53	6,530	81,492,480	0.08	0.08	0.07	0.00	0.01	0.00	1.15 [1.06-1.25]*	1.06 [0.97-1.16]
M54	250,260	77,375,330	3.46	3.18	2.91	0.28	0.55	0.38	1.19 [1.17-1.20]*	1.12 [1.11-1.14]*
M60	5,040	81,536,490	0.08	0.06	0.05	0.02	0.02	0.02	1.38 [1.26-1.52]*	1.35 [1.23-1.49]*
M62	21,830	81,363,300	0.28	0.26	0.25	0.02	0.03	0.03	1.14 [1.09-1.19]*	1.11 [1.06-1.17]*
M65	43,470	80,681,090	0.59	0.53	0.47	0.07	0.12	0.09	1.26 [1.22-1.30]*	1.19 [1.15-1.22]*
M66	4,260	81,520,580	0.05	0.05	0.05	0.00	0.01	0.01	1.16 [1.05-1.29]*	1.11 [1.00-1.24]
M67	29,840	80,756,360	0.39	0.36	0.34	0.03	0.06	0.04	1.17 [1.12-1.21]*	1.11 [1.07-1.16]*
M70	16,970	81,292,580	0.23	0.20	0.18	0.03	0.05	0.04	1.30 [1.23-1.37]*	1.21 [1.15-1.27]*
M71	10,840	81,450,590	0.15	0.13	0.11	0.01	0.03	0.02	1.26 [1.18-1.35]*	1.19 [1.11-1.27]*
M72	22,100	81,074,170	0.26	0.28	0.24	−0.02	0.01	0.01	1.06 [1.01-1.11]	1.04 [0.99-1.09]
M75	67,990	80,184,840	0.89	0.84	0.74	0.05	0.15	0.10	1.20 [1.17-1.23]*	1.13 [1.10-1.16]*
M76	5,770	81,524,080	0.08	0.07	0.06	0.01	0.02	0.01	1.32 [1.21-1.44]*	1.22 [1.11-1.33]*
M77	13,560	81,312,190	0.19	0.16	0.14	0.03	0.05	0.04	1.34 [1.26-1.41]*	1.24 [1.17-1.31]*
M79	209,940	78,285,910	3.05	2.59	2.38	0.46	0.68	0.52	1.28 [1.27-1.30]*	1.20 [1.19-1.22]*
M80	24,740	81,395,890	0.33	0.30	0.27	0.04	0.06	0.05	1.22 [1.17-1.28]*	1.17 [1.12-1.22]*
M81	151,170	80,350,480	2.08	1.83	1.70	0.25	0.39	0.29	1.23 [1.21-1.25]*	1.16 [1.14-1.18]*
M83	960	81,595,120	0.01	0.01	0.01	0.00	0.00	0.00	1.29 [1.04-1.60]	1.22 [0.98-1.52]
M84	21,330	81,190,800	0.27	0.26	0.24	0.01	0.04	0.03	1.15 [1.10-1.20]*	1.13 [1.07-1.18]*
M85	35,070	81,372,430	0.48	0.42	0.39	0.06	0.09	0.07	1.24 [1.19-1.28]*	1.16 [1.12-1.20]*
M86	15,880	81,411,250	0.22	0.19	0.17	0.03	0.05	0.04	1.28 [1.21-1.35]*	1.26 [1.19-1.33]*
M87	6,730	81,497,510	0.09	0.08	0.07	0.00	0.01	0.01	1.16 [1.07-1.26]*	1.13 [1.04-1.23]*
M88	3,790	81,528,080	0.05	0.05	0.04	0.00	0.00	0.00	1.07 [0.96-1.21]	1.05 [0.93-1.18]
M89	17,420	81,246,350	0.23	0.21	0.20	0.02	0.03	0.03	1.18 [1.12-1.24]*	1.13 [1.07-1.19]*
M90	6,560	81,581,130	0.07	0.08	0.07	−0.01	0.00	0.00	1.02 [0.93-1.11]	1.04 [0.95-1.14]
M91	1,450	81,565,990	0.02	0.02	0.02	0.00	0.00	0.00	1.13 [0.95-1.34]	1.11 [0.93-1.32]
M92	2,200	81,562,280	0.03	0.03	0.03	0.01	0.01	0.01	1.31 [1.14-1.51]*	1.18 [1.02-1.36]
M93	4,210	81,493,300	0.06	0.05	0.05	0.01	0.01	0.01	1.18 [1.07-1.31]*	1.12 [1.00-1.24]
M94	12,620	81,403,730	0.18	0.15	0.14	0.03	0.04	0.03	1.31 [1.23-1.39]*	1.21 [1.14-1.29]*
M95	2,960	81,491,560	0.04	0.04	0.03	0.01	0.01	0.01	1.21 [1.07-1.37]*	1.16 [1.03-1.32]
M96	9,030	81,516,410	0.12	0.11	0.10	0.01	0.02	0.02	1.20 [1.11-1.29]*	1.17 [1.08-1.26]*
M99	2,560	81,586,100	0.03	0.03	0.03	0.00	0.00	0.00	1.16 [1.01-1.33]	1.09 [0.95-1.25]
hospitalised - N00-N99										
N00	530	81,599,820	0.01	0.01	0.01	0.00	0.00	0.00	1.15 [0.87-1.53]	1.14 [0.85-1.54]
N02	6,600	81,456,850	0.09	0.08	0.07	0.01	0.01	0.01	1.19 [1.10-1.30]*	1.17 [1.07-1.27]*
N03	26,170	81,385,950	0.35	0.31	0.28	0.04	0.07	0.06	1.26 [1.21-1.32]*	1.22 [1.17-1.28]*
N04	4,280	81,526,240	0.06	0.05	0.05	0.01	0.01	0.01	1.23 [1.12-1.37]*	1.20 [1.08-1.33]*
N05	4,380	81,527,020	0.06	0.05	0.05	0.01	0.02	0.02	1.37 [1.24-1.51]*	1.32 [1.19-1.46]*
N08	12,730	81,491,740	0.17	0.15	0.14	0.02	0.04	0.04	1.28 [1.21-1.36]*	1.26 [1.19-1.34]*
N10	5,320	81,507,240	0.07	0.06	0.06	0.00	0.00	0.00	1.06 [0.97-1.17]	1.03 [0.93-1.13]
N11	2,580	81,553,650	0.03	0.03	0.03	0.00	0.00	0.00	1.08 [0.94-1.24]	1.09 [0.95-1.25]
N12	29,720	81,114,050	0.38	0.36	0.35	0.02	0.03	0.02	1.10 [1.05-1.14]*	1.06 [1.02-1.11]*
N13	61,440	80,899,320	0.72	0.77	0.71	−0.05	0.01	0.01	1.01 [0.99-1.04]	1.02 [0.99-1.05]
N14	930	81,603,530	0.01	0.01	0.01	0.00	0.00	0.00	1.25 [1.00-1.58]	1.22 [0.98-1.53]
N15	1,710	81,587,830	0.02	0.02	0.02	0.00	0.00	0.00	1.08 [0.91-1.28]	1.06 [0.90-1.26]
N16	870	81,598,350	0.01	0.01	0.01	0.00	0.00	0.00	1.29 [1.03-1.62]	1.30 [1.03-1.64]
N17	338,580	80,306,460	4.52	4.14	3.85	0.37	0.67	0.63	1.17 [1.16-1.19]*	1.16 [1.15-1.18]*
N18	289,170	80,151,460	3.88	3.54	3.26	0.34	0.61	0.54	1.19 [1.17-1.20]*	1.16 [1.15-1.18]*
N19	40,720	81,185,420	0.54	0.49	0.44	0.05	0.10	0.09	1.23 [1.19-1.27]*	1.20 [1.16-1.24]*
N20	48,820	80,605,500	0.58	0.61	0.54	−0.03	0.04	0.03	1.07 [1.04-1.10]*	1.05 [1.02-1.08]*
N21	12,260	81,422,080	0.13	0.16	0.14	−0.02	−0.01	−0.01	0.95 [0.89-1.02]	0.96 [0.90-1.02]
N23	11,140	81,174,260	0.13	0.14	0.12	−0.01	0.01	0.00	1.05 [0.98-1.12]	1.01 [0.94-1.08]
N25	2,650	81,575,090	0.03	0.03	0.03	0.00	0.00	0.00	1.15 [1.00-1.31]	1.14 [1.00-1.31]
N26	5,190	81,575,220	0.06	0.06	0.06	0.00	0.01	0.01	1.10 [1.00-1.21]	1.09 [0.99-1.21]
N27	2,770	81,573,760	0.04	0.03	0.03	0.00	0.00	0.00	1.12 [0.98-1.28]	1.12 [0.98-1.27]
N28	69,620	80,965,660	0.87	0.86	0.79	0.02	0.09	0.07	1.11 [1.08-1.14]*	1.09 [1.06-1.12]*
N29	660	81,598,300	0.01	0.01	0.01	0.00	0.00	0.00	1.18 [0.91-1.55]	1.16 [0.88-1.52]
N30	32,110	80,992,360	0.41	0.39	0.35	0.02	0.06	0.05	1.16 [1.12-1.21]*	1.13 [1.09-1.17]*
N31	10,300	81,363,560	0.14	0.12	0.11	0.01	0.02	0.02	1.19 [1.12-1.27]*	1.13 [1.06-1.21]*
N32	91,150	79,970,370	1.18	1.13	1.03	0.05	0.15	0.12	1.14 [1.12-1.17]*	1.11 [1.09-1.14]*
N34	1,580	81,565,930	0.02	0.02	0.02	0.00	0.00	0.00	1.12 [0.94-1.33]	1.09 [0.91-1.29]
N35	31,140	80,880,560	0.41	0.38	0.34	0.03	0.07	0.06	1.20 [1.16-1.25]*	1.17 [1.12-1.22]*
N36	9,020	81,421,600	0.11	0.11	0.10	0.00	0.01	0.01	1.14 [1.06-1.23]*	1.11 [1.03-1.19]*
N39	403,030	76,126,900	5.63	5.21	4.96	0.42	0.67	0.58	1.14 [1.12-1.15]*	1.11 [1.10-1.13]*
N40	124,410	79,729,070	1.64	1.54	1.49	0.10	0.15	0.11	1.10 [1.08-1.12]*	1.07 [1.05-1.09]*
N41	9,450	81,405,900	0.11	0.12	0.10	−0.01	0.00	0.00	1.04 [0.97-1.12]	1.01 [0.93-1.08]
N42	12,520	81,381,370	0.14	0.16	0.14	−0.01	0.00	0.00	1.03 [0.97-1.10]	1.01 [0.95-1.08]
N43	17,630	81,128,920	0.23	0.21	0.21	0.02	0.02	0.02	1.10 [1.05-1.16]*	1.09 [1.03-1.15]*
N44	5,530	81,482,120	0.09	0.06	0.08	0.03	0.01	0.01	1.15 [1.06-1.26]*	1.14 [1.04-1.25]*
N45	13,290	81,316,900	0.17	0.16	0.15	0.01	0.02	0.01	1.11 [1.04-1.18]*	1.09 [1.02-1.15]*
N47	44,480	80,256,610	0.74	0.51	0.54	0.23	0.20	0.19	1.37 [1.33-1.42]*	1.34 [1.30-1.38]*
N48	26,020	80,994,840	0.39	0.30	0.30	0.08	0.09	0.08	1.28 [1.23-1.34]*	1.26 [1.21-1.31]*
N49	3,220	81,547,360	0.04	0.04	0.04	0.00	0.01	0.01	1.18 [1.05-1.33]*	1.19 [1.05-1.34]*
N50	27,610	80,905,870	0.38	0.33	0.33	0.04	0.05	0.04	1.15 [1.10-1.19]*	1.12 [1.08-1.17]*
N60	9,260	81,273,960	0.11	0.11	0.10	0.00	0.01	0.01	1.10 [1.02-1.18]*	1.09 [1.01-1.17]
N61	6,460	81,427,950	0.09	0.08	0.07	0.01	0.02	0.02	1.27 [1.17-1.38]*	1.26 [1.15-1.37]*
N62	5,140	81,377,660	0.07	0.06	0.06	0.00	0.01	0.01	1.16 [1.05-1.27]*	1.12 [1.01-1.23]
N63	9,880	81,264,630	0.12	0.12	0.11	0.00	0.01	0.01	1.10 [1.02-1.18]*	1.08 [1.00-1.16]
N64	9,960	81,355,960	0.13	0.12	0.11	0.01	0.02	0.02	1.18 [1.11-1.27]*	1.15 [1.08-1.24]*
N70	6,970	81,401,480	0.08	0.09	0.08	−0.01	0.00	0.00	1.00 [0.92-1.09]	0.98 [0.90-1.07]
N71	3,460	81,512,870	0.04	0.04	0.04	0.00	0.01	0.00	1.14 [1.02-1.28]	1.08 [0.96-1.22]
N72	6,140	81,302,250	0.07	0.08	0.07	−0.01	0.00	0.00	1.04 [0.96-1.14]	1.02 [0.93-1.12]
N73	38,640	80,480,810	0.47	0.48	0.44	−0.01	0.03	0.02	1.07 [1.03-1.11]*	1.03 [1.00-1.07]
N75	6,090	81,377,660	0.07	0.08	0.07	−0.01	0.00	0.00	1.01 [0.92-1.10]	1.00 [0.91-1.10]
N76	10,140	81,313,920	0.15	0.12	0.11	0.03	0.04	0.03	1.32 [1.24-1.41]*	1.27 [1.19-1.36]*
N77	12,940	81,328,630	0.18	0.16	0.15	0.02	0.03	0.02	1.19 [1.12-1.26]*	1.13 [1.06-1.20]*
N80	36,000	80,412,160	0.45	0.45	0.41	0.00	0.03	0.01	1.08 [1.04-1.12]*	1.03 [0.99-1.07]
N81	59,120	79,925,540	0.76	0.73	0.66	0.02	0.10	0.07	1.15 [1.12-1.18]*	1.10 [1.07-1.13]*
N82	3,080	81,559,930	0.04	0.04	0.03	0.00	0.01	0.01	1.23 [1.09-1.38]*	1.19 [1.05-1.34]*
N83	60,910	79,951,970	0.77	0.76	0.71	0.01	0.06	0.03	1.08 [1.05-1.11]*	1.04 [1.01-1.07]*
N84	45,630	80,062,610	0.56	0.57	0.51	−0.02	0.04	0.03	1.08 [1.05-1.12]*	1.07 [1.03-1.10]*
N85	39,540	80,593,480	0.50	0.49	0.44	0.01	0.05	0.04	1.12 [1.09-1.16]*	1.09 [1.05-1.13]*
N86	11,210	81,174,440	0.14	0.14	0.13	0.01	0.02	0.01	1.13 [1.06-1.20]*	1.08 [1.01-1.15]
N87	12,780	80,802,690	0.15	0.16	0.14	−0.01	0.01	0.01	1.05 [0.99-1.12]	1.05 [0.98-1.11]
N88	11,860	81,269,870	0.15	0.15	0.13	0.00	0.02	0.01	1.12 [1.05-1.19]*	1.10 [1.03-1.17]*
N89	20,720	80,955,580	0.27	0.25	0.23	0.02	0.04	0.03	1.17 [1.12-1.23]*	1.12 [1.07-1.18]*
N90	19,020	81,089,870	0.26	0.23	0.21	0.03	0.06	0.05	1.27 [1.21-1.34]*	1.22 [1.16-1.28]*
N91	2,230	81,534,480	0.03	0.03	0.03	0.00	0.00	0.00	1.15 [1.00-1.32]	1.10 [0.95-1.27]
N92	79,150	78,677,320	1.05	1.00	0.92	0.05	0.13	0.09	1.15 [1.12-1.17]*	1.10 [1.07-1.13]*
N93	38,310	80,244,560	0.49	0.48	0.44	0.01	0.05	0.03	1.11 [1.07-1.14]*	1.07 [1.04-1.11]*
N94	30,520	80,349,940	0.40	0.37	0.34	0.03	0.06	0.04	1.17 [1.13-1.22]*	1.11 [1.07-1.16]*
N95	40,150	80,260,210	0.53	0.49	0.44	0.04	0.09	0.07	1.19 [1.15-1.23]*	1.16 [1.12-1.20]*

N96	670	81,593,170	0.01	0.01	0.01	0.00	0.00	0.00	0.99 [0.75-1.30]	1.00 [0.76-1.33]
N97	4,700	80,991,400	0.05	0.06	0.05	−0.01	0.00	0.00	0.97 [0.88-1.08]	0.96 [0.86-1.07]
N98	730	81,578,260	0.01	0.01	0.01	0.00	0.00	0.00	0.80 [0.60-1.06]	0.77 [0.57-1.02]
N99	11,620	81,356,340	0.14	0.14	0.13	0.00	0.02	0.01	1.13 [1.06-1.20]*	1.11 [1.04-1.18]*
hospitalised - O00-O9A										
O00	11,450	81,201,520	0.13	0.14	0.13	−0.01	0.00	0.00	0.99 [0.92-1.05]	0.98 [0.92-1.05]
O01	650	81,584,750	0.01	0.01	0.01	0.00	0.00	0.00	1.14 [0.87-1.48]	1.21 [0.92-1.59]
O02	25,360	80,417,910	0.30	0.32	0.29	−0.02	0.01	0.01	1.04 [1.00-1.09]	1.03 [0.99-1.08]
O03	38,390	79,946,380	0.46	0.49	0.45	−0.03	0.00	0.00	1.01 [0.97-1.05]	1.00 [0.97-1.04]
O04	43,540	79,428,140	0.54	0.55	0.57	−0.01	−0.03	−0.03	0.94 [0.91-0.98]*	0.95 [0.92-0.98]*
O08	4,280	81,483,310	0.05	0.05	0.05	0.00	0.00	0.00	0.97 [0.87-1.09]	0.97 [0.87-1.09]
O10	4,440	81,487,960	0.05	0.05	0.05	0.00	0.00	0.00	1.09 [0.98-1.21]	1.06 [0.96-1.19]
O11	620	81,591,780	0.01	0.01	0.01	0.00	0.00	0.00	1.12 [0.85-1.48]	1.09 [0.82-1.44]
O12	8,260	81,213,360	0.10	0.10	0.09	0.00	0.01	0.00	1.09 [1.01-1.17]	1.05 [0.97-1.14]
O13	20,670	80,566,070	0.25	0.26	0.24	0.00	0.01	0.01	1.05 [1.00-1.10]	1.03 [0.98-1.08]
O14	14,620	80,888,980	0.18	0.18	0.17	0.00	0.01	0.01	1.06 [1.00-1.13]	1.03 [0.97-1.09]
O15	580	81,580,460	0.01	0.01	0.01	0.00	0.00	0.00	1.05 [0.79-1.40]	1.00 [0.74-1.35]
O16	16,400	80,530,040	0.20	0.20	0.19	0.00	0.02	0.01	1.08 [1.02-1.14]*	1.06 [1.01-1.12]
O20	33,410	80,135,800	0.41	0.42	0.41	−0.01	0.00	0.00	1.00 [0.96-1.04]	0.99 [0.95-1.03]
O21	24,680	80,711,630	0.30	0.31	0.30	0.00	0.01	0.00	1.02 [0.97-1.06]	0.99 [0.94-1.03]
O22	4,390	81,489,790	0.05	0.05	0.05	0.00	0.00	0.00	1.00 [0.90-1.12]	1.00 [0.90-1.12]
O23	26,820	80,563,170	0.34	0.33	0.32	0.01	0.03	0.02	1.08 [1.04-1.13]*	1.06 [1.02-1.11]*
O24	33,740	80,897,110	0.40	0.42	0.39	−0.02	0.01	0.00	1.02 [0.98-1.06]	1.01 [0.97-1.05]
O26	91,380	77,302,320	1.27	1.16	1.18	0.11	0.09	0.05	1.07 [1.05-1.10]*	1.04 [1.02-1.07]*
O28	10,310	81,388,470	0.12	0.13	0.12	−0.01	0.00	0.00	0.99 [0.92-1.06]	0.96 [0.90-1.03]
O30	12,370	81,191,910	0.14	0.16	0.14	−0.02	−0.01	−0.01	0.95 [0.89-1.02]	0.95 [0.88-1.01]
O31	720	81,587,380	0.01	0.01	0.01	0.00	0.00	0.00	1.08 [0.84-1.39]	1.07 [0.82-1.39]
O32	45,580	79,917,320	0.54	0.58	0.54	−0.04	−0.01	−0.01	0.99 [0.96-1.02]	0.98 [0.95-1.02]
O33	1,140	81,534,310	0.01	0.01	0.01	0.00	0.00	0.00	1.02 [0.83-1.26]	1.01 [0.82-1.24]
O34	90,710	79,146,910	1.09	1.16	1.07	−0.07	0.02	0.02	1.02 [1.00-1.04]	1.02 [0.99-1.04]
O35	12,260	81,259,790	0.14	0.15	0.14	−0.01	0.00	0.00	0.99 [0.93-1.05]	0.98 [0.92-1.05]
O36	119,100	77,225,730	1.65	1.52	1.58	0.13	0.07	0.05	1.04 [1.02-1.07]*	1.03 [1.01-1.05]*
O40	11,950	81,352,870	0.14	0.15	0.14	−0.01	0.01	0.00	1.04 [0.98-1.11]	1.02 [0.96-1.09]
O41	13,900	81,005,190	0.17	0.17	0.16	0.00	0.01	0.01	1.04 [0.98-1.11]	1.04 [0.98-1.10]
O42	68,050	78,812,600	0.86	0.87	0.85	−0.01	0.01	0.01	1.01 [0.98-1.04]	1.02 [0.99-1.05]
O43	10,540	81,165,940	0.12	0.13	0.12	−0.01	0.00	0.00	1.01 [0.94-1.08]	1.00 [0.94-1.08]
O44	7,850	81,352,440	0.09	0.10	0.09	−0.01	0.00	0.00	1.01 [0.94-1.10]	1.00 [0.92-1.08]
O45	3,300	81,489,740	0.04	0.04	0.04	0.00	0.00	0.00	1.00 [0.88-1.13]	0.99 [0.88-1.13]
O46	35,020	80,086,420	0.44	0.44	0.41	0.00	0.03	0.02	1.06 [1.02-1.10]*	1.05 [1.01-1.09]
O47	23,500	79,470,910	0.30	0.30	0.28	0.00	0.02	0.01	1.07 [1.02-1.12]*	1.05 [1.00-1.10]
O48	40,410	79,270,940	0.48	0.52	0.49	−0.04	−0.02	−0.01	0.97 [0.93-1.00]	0.97 [0.94-1.01]
O60	39,610	80,086,560	0.48	0.50	0.47	−0.02	0.01	0.01	1.03 [0.99-1.06]	1.02 [0.98-1.06]
O61	6,700	81,288,040	0.08	0.08	0.08	0.00	0.00	0.00	1.05 [0.97-1.15]	1.02 [0.93-1.11]
O62	27,980	80,578,560	0.34	0.35	0.33	−0.01	0.01	0.01	1.02 [0.98-1.07]	1.03 [0.98-1.07]
O63	50,500	78,365,170	0.64	0.65	0.63	−0.01	0.01	0.00	1.01 [0.98-1.04]	1.00 [0.97-1.03]
O64	11,000	80,863,800	0.13	0.14	0.13	−0.01	0.00	0.00	1.01 [0.94-1.07]	1.00 [0.94-1.07]
O65	820	81,531,580	0.01	0.01	0.01	0.00	0.00	0.00	0.99 [0.78-1.26]	1.02 [0.79-1.31]
O66	11,740	81,025,240	0.13	0.15	0.14	−0.01	0.00	0.00	0.99 [0.93-1.06]	0.99 [0.93-1.06]
O67	3,230	81,445,820	0.04	0.04	0.04	0.00	0.00	0.00	1.01 [0.89-1.14]	0.99 [0.88-1.13]
O68	113,140	75,488,680	1.58	1.48	1.57	0.10	0.01	0.00	1.01 [0.99-1.03]	1.00 [0.98-1.02]
O69	12,920	80,716,360	0.15	0.16	0.15	−0.01	0.01	0.01	1.03 [0.97-1.10]	1.04 [0.98-1.11]
O70	149,700	73,066,490	2.23	2.00	2.28	0.23	−0.05	−0.02	0.98 [0.96-0.99]	0.99 [0.97-1.01]
O71	11,070	81,128,280	0.13	0.14	0.13	−0.01	0.00	0.00	1.00 [0.93-1.06]	1.00 [0.94-1.07]
O72	88,670	78,684,440	1.13	1.13	1.10	0.00	0.02	0.02	1.02 [1.00-1.04]	1.02 [0.99-1.04]
O73	6,700	81,269,990	0.08	0.08	0.07	−0.01	0.00	0.00	1.04 [0.95-1.13]	1.03 [0.95-1.13]
O74	1,850	81,544,010	0.02	0.02	0.02	0.00	0.00	0.00	1.12 [0.96-1.31]	1.10 [0.94-1.30]
O75	45,770	79,886,760	0.55	0.58	0.55	−0.03	0.00	0.01	1.01 [0.97-1.04]	1.01 [0.98-1.05]
O80	52,830	78,240,240	0.64	0.68	0.66	−0.04	−0.02	0.00	0.97 [0.94-1.00]	1.00 [0.97-1.03]
O82	7,740	81,056,600	0.09	0.10	0.09	−0.01	0.00	0.00	1.02 [0.94-1.10]	1.01 [0.93-1.09]
O85	4,170	81,530,810	0.05	0.05	0.05	0.00	0.00	0.00	1.03 [0.93-1.15]	1.01 [0.90-1.12]
O86	14,470	81,111,350	0.18	0.18	0.16	0.01	0.02	0.01	1.11 [1.05-1.18]*	1.08 [1.02-1.15]*
O87	2,160	81,502,560	0.03	0.03	0.02	0.00	0.00	0.00	1.17 [1.01-1.35]	1.16 [0.99-1.35]
O88	900	81,589,330	0.01	0.01	0.01	0.00	0.00	0.00	1.14 [0.91-1.44]	1.09 [0.86-1.38]
O89	640	81,583,300	0.01	0.01	0.01	0.00	0.00	0.00	1.17 [0.90-1.53]	1.14 [0.87-1.49]
O90	17,470	81,144,550	0.22	0.21	0.20	0.01	0.02	0.02	1.11 [1.05-1.17]*	1.08 [1.02-1.14]*
O91	1,740	81,561,350	0.02	0.02	0.02	0.00	0.00	0.00	0.98 [0.82-1.16]	0.95 [0.80-1.14]
O92	2,610	81,508,320	0.03	0.03	0.03	0.00	0.00	0.00	1.04 [0.91-1.19]	1.03 [0.89-1.18]
O94	660	81,603,890	0.01	0.01	0.01	0.00	0.00	0.00	1.15 [0.88-1.50]	1.11 [0.84-1.46]
O98	6,820	81,400,060	0.09	0.08	0.08	0.00	0.01	0.01	1.10 [1.01-1.19]	1.06 [0.98-1.16]
O99	104,040	77,881,300	1.43	1.31	1.31	0.11	0.11	0.08	1.08 [1.06-1.11]*	1.06 [1.04-1.08]*
hospitalised - P00-P96										
P83	1,370	80,594,280	0.02	0.02	0.02	0.00	0.00	0.00	1.00 [0.84-1.20]	1.01 [0.84-1.21]
hospitalised - Q00-Q99										
Q02	1,950	81,557,430	0.03	0.02	0.03	0.00	0.00	0.00	1.02 [0.88-1.18]	1.00 [0.86-1.16]
Q03	760	81,567,870	0.01	0.01	0.01	0.00	0.00	0.00	1.22 [0.96-1.55]	1.21 [0.95-1.55]
Q04	2,090	81,536,420	0.03	0.03	0.03	0.00	0.00	0.00	1.02 [0.88-1.18]	0.98 [0.84-1.14]
Q05	1,870	81,544,140	0.02	0.02	0.02	0.00	0.00	0.00	1.18 [1.01-1.37]	1.10 [0.94-1.28]
Q06	600	81,597,540	0.01	0.01	0.01	0.00	0.00	0.00	0.85 [0.63-1.13]	0.81 [0.60-1.08]
Q07	2,370	81,573,650	0.03	0.03	0.03	0.00	0.00	0.00	1.11 [0.97-1.27]	1.04 [0.90-1.19]
Q10	2,280	81,541,940	0.03	0.03	0.03	0.01	0.00	0.00	1.14 [1.00-1.31]	1.12 [0.98-1.29]
Q12	1,180	81,570,370	0.02	0.01	0.01	0.00	0.00	0.00	1.16 [0.95-1.40]	1.13 [0.93-1.38]
Q13	480	81,593,240	0.01	0.01	0.01	0.00	0.00	0.00	1.22 [0.90-1.64]	1.14 [0.84-1.55]
Q14	620	81,597,600	0.01	0.01	0.01	0.00	0.00	0.00	1.08 [0.82-1.42]	1.08 [0.82-1.43]
Q17	4,170	81,342,050	0.06	0.05	0.06	0.01	0.00	0.00	1.06 [0.96-1.17]	1.06 [0.95-1.17]
Q18	2,770	81,506,030	0.04	0.03	0.03	0.01	0.00	0.00	1.14 [1.01-1.29]	1.13 [1.00-1.29]
Q20	570	81,565,000	0.01	0.01	0.01	0.00	0.00	0.00	1.03 [0.77-1.37]	1.00 [0.75-1.34]
Q21	9,280	81,167,760	0.12	0.11	0.11	0.01	0.01	0.01	1.12 [1.04-1.20]*	1.07 [1.00-1.15]
Q22	1,220	81,544,750	0.01	0.02	0.02	0.00	0.00	0.00	0.94 [0.77-1.15]	0.95 [0.78-1.17]
Q23	4,210	81,516,460	0.05	0.05	0.05	0.00	0.00	0.00	1.04 [0.94-1.16]	1.02 [0.92-1.14]
Q24	3,430	81,491,750	0.04	0.04	0.04	0.00	0.00	0.00	1.08 [0.96-1.22]	1.03 [0.92-1.16]
Q25	3,480	81,285,750	0.04	0.04	0.04	0.00	0.00	0.00	1.02 [0.91-1.14]	0.98 [0.88-1.11]
Q26	620	81,591,250	0.01	0.01	0.01	0.00	0.00	0.00	0.97 [0.74-1.27]	0.93 [0.71-1.23]
Q27	2,950	81,545,950	0.04	0.04	0.04	0.00	0.00	0.00	1.03 [0.91-1.17]	1.02 [0.90-1.16]
Q28	1,440	81,574,030	0.02	0.02	0.02	0.00	0.00	0.00	1.18 [0.99-1.40]	1.17 [0.98-1.39]
Q31	1,320	81,524,350	0.02	0.02	0.02	0.00	0.00	0.00	0.92 [0.77-1.11]	0.84 [0.69-1.02]
Q32	540	81,592,100	0.01	0.01	0.01	0.00	0.00	0.00	0.92 [0.68-1.23]	0.80 [0.59-1.09]
Q33	520	81,575,570	0.01	0.01	0.01	0.00	0.00	0.00	1.01 [0.75-1.36]	0.90 [0.66-1.23]
Q35	1,350	81,537,590	0.02	0.02	0.02	0.00	0.00	0.00	1.04 [0.87-1.24]	1.01 [0.84-1.22]
Q38	8,660	80,950,640	0.12	0.10	0.11	0.02	0.01	0.01	1.10 [1.02-1.18]	1.07 [1.00-1.15]
Q39	1,650	81,575,410	0.02	0.02	0.02	0.00	0.00	0.00	1.11 [0.93-1.31]	1.03 [0.87-1.23]
Q40	1,010	81,513,210	0.01	0.01	0.01	0.00	0.00	0.00	1.20 [0.97-1.48]	1.12 [0.90-1.39]
Q43	3,920	81,489,790	0.05	0.05	0.04	0.00	0.01	0.00	1.13 [1.01-1.26]	1.07 [0.96-1.19]
Q44	1,320	81,582,930	0.02	0.02	0.01	0.00	0.00	0.00	1.08 [0.89-1.31]	1.05 [0.86-1.27]
Q45	540	81,600,990	0.01	0.01	0.01	0.00	0.00	0.00	1.37 [1.03-1.81]	1.29 [0.97-1.72]
Q50	2,630	81,529,660	0.03	0.03	0.03	0.00	0.00	0.00	1.13 [0.99-1.29]	1.11 [0.97-1.27]
Q51	2,580	81,544,310	0.03	0.03	0.03	0.00	0.00	0.00	1.06 [0.93-1.22]	1.03 [0.90-1.18]

Q52	1,260	81,558,060	0.02	0.01	0.02	0.00	0.00	0.00	1.23 [1.03-1.48]	1.20 [0.99-1.44]
Q53	11,030	81,025,560	0.15	0.13	0.16	0.02	−0.01	−0.01	0.96 [0.90-1.02]	0.96 [0.90-1.02]
Q54	3,240	81,360,470	0.04	0.04	0.04	0.00	0.00	0.00	1.04 [0.93-1.17]	1.06 [0.94-1.19]
Q55	7,100	81,403,860	0.11	0.08	0.10	0.03	0.01	0.01	1.13 [1.04-1.22]*	1.10 [1.02-1.19]
Q60	2,720	81,554,670	0.03	0.03	0.03	0.00	0.00	0.00	1.07 [0.94-1.23]	1.06 [0.92-1.21]
Q61	6,690	81,470,580	0.08	0.08	0.08	0.00	0.01	0.00	1.07 [0.99-1.17]	1.05 [0.96-1.15]
Q62	1,520	81,409,110	0.02	0.02	0.02	0.00	0.00	0.00	0.96 [0.81-1.15]	0.96 [0.80-1.15]
Q63	5,300	81,470,370	0.07	0.06	0.06	0.00	0.00	0.00	1.05 [0.96-1.16]	1.00 [0.91-1.10]
Q64	1,140	81,551,510	0.02	0.01	0.02	0.00	0.00	0.00	1.03 [0.85-1.26]	1.01 [0.82-1.24]
Q65	4,290	81,417,150	0.05	0.05	0.05	0.00	0.00	0.00	0.93 [0.84-1.04]	0.91 [0.82-1.02]
Q66	4,730	80,756,330	0.07	0.06	0.06	0.01	0.01	0.01	1.12 [1.02-1.23]	1.09 [0.99-1.20]
Q67	3,130	81,501,880	0.04	0.04	0.04	0.01	0.00	0.00	1.10 [0.98-1.24]	1.07 [0.95-1.21]
Q68	1,080	81,562,170	0.01	0.01	0.01	0.00	0.00	0.00	1.03 [0.84-1.27]	1.00 [0.81-1.24]
Q72	480	81,592,300	0.01	0.01	0.01	0.00	0.00	0.00	1.06 [0.78-1.43]	1.04 [0.76-1.42]
Q74	3,260	81,517,730	0.05	0.04	0.04	0.01	0.00	0.00	1.11 [0.98-1.24]	1.08 [0.96-1.21]
Q75	1,830	81,521,550	0.02	0.02	0.03	0.00	0.00	0.00	0.98 [0.84-1.14]	0.95 [0.81-1.11]
Q76	2,710	81,540,610	0.03	0.03	0.03	0.00	0.00	0.00	1.07 [0.94-1.21]	1.02 [0.89-1.16]
Q78	1,760	81,573,860	0.02	0.02	0.02	0.00	0.00	0.00	1.12 [0.95-1.31]	1.07 [0.91-1.26]
Q79	3,540	81,523,670	0.05	0.04	0.04	0.01	0.01	0.00	1.19 [1.07-1.33]*	1.04 [0.93-1.17]
Q80	250	81,602,560	0.01	0.00	0.00	0.00	0.00	0.00	2.74 [1.90-3.95]*	2.50 [1.71-3.64]*
Q82	2,680	80,900,600	0.04	0.03	0.03	0.01	0.01	0.01	1.32 [1.17-1.49]*	1.26 [1.11-1.43]*
Q83	1,260	81,537,110	0.02	0.02	0.01	0.00	0.00	0.00	1.04 [0.86-1.26]	0.99 [0.82-1.21]
Q85	2,330	81,551,960	0.03	0.03	0.03	0.00	0.00	0.00	0.94 [0.81-1.08]	0.92 [0.79-1.06]
Q87	3,320	81,507,090	0.05	0.04	0.04	0.01	0.01	0.00	1.14 [1.01-1.28]	1.10 [0.98-1.24]
Q89	2,890	81,526,250	0.04	0.03	0.04	0.00	0.00	0.00	1.06 [0.93-1.20]	1.04 [0.91-1.18]
Q90	1,160	81,459,150	0.02	0.01	0.01	0.01	0.01	0.01	2.00 [1.68-2.37]*	1.90 [1.59-2.27]*
Q92	620	81,601,510	0.01	0.01	0.01	0.00	0.00	0.00	1.18 [0.91-1.54]	1.15 [0.88-1.51]
Q93	1,690	81,582,690	0.03	0.02	0.02	0.01	0.01	0.00	1.25 [1.07-1.46]*	1.17 [1.00-1.38]
Q98	460	81,596,960	0.01	0.01	0.00	0.00	0.00	0.00	1.63 [1.22-2.17]*	1.50 [1.12-2.02]*
Q99	2,040	81,574,330	0.03	0.02	0.03	0.01	0.00	0.00	1.10 [0.95-1.27]	1.05 [0.90-1.21]
hospitalised - R00-R99										
R00	188,760	79,371,840	2.50	2.35	2.20	0.15	0.30	0.22	1.14 [1.12-1.16]*	1.10 [1.08-1.12]*
R01	30,680	81,007,020	0.42	0.37	0.36	0.05	0.06	0.05	1.17 [1.13-1.22]*	1.13 [1.09-1.18]*
R02	28,210	81,413,330	0.36	0.34	0.31	0.02	0.05	0.05	1.16 [1.11-1.21]*	1.16 [1.11-1.21]*
R03	64,760	80,822,970	0.83	0.79	0.74	0.03	0.09	0.07	1.12 [1.09-1.15]*	1.10 [1.07-1.13]*
R04	73,250	80,377,220	1.01	0.89	0.82	0.13	0.19	0.16	1.23 [1.20-1.26]*	1.19 [1.16-1.22]*
R05	79,910	80,300,020	1.18	0.95	0.92	0.23	0.27	0.21	1.29 [1.26-1.32]*	1.21 [1.18-1.24]*
R06	260,570	76,492,040	4.42	3.16	3.10	1.26	1.32	1.18	1.43 [1.41-1.44]*	1.36 [1.35-1.38]*
R07	301,820	74,726,870	4.31	3.97	3.64	0.34	0.67	0.48	1.19 [1.17-1.20]*	1.13 [1.11-1.14]*
R09	28,890	81,267,600	0.39	0.35	0.32	0.04	0.07	0.05	1.22 [1.17-1.27]*	1.16 [1.12-1.21]*
R10	408,530	69,657,160	6.53	5.69	5.54	0.83	0.99	0.71	1.18 [1.17-1.19]*	1.12 [1.11-1.13]*
R11	263,030	76,697,280	3.74	3.35	3.25	0.39	0.49	0.36	1.15 [1.13-1.17]*	1.11 [1.09-1.12]*
R12	16,740	81,258,260	0.22	0.20	0.18	0.02	0.04	0.03	1.23 [1.17-1.30]*	1.14 [1.08-1.20]*
R13	126,880	80,364,500	1.73	1.54	1.41	0.19	0.32	0.25	1.23 [1.21-1.25]*	1.17 [1.15-1.19]*
R14	29,600	81,168,960	0.40	0.36	0.33	0.04	0.07	0.05	1.22 [1.17-1.26]*	1.14 [1.10-1.19]*
R15	54,570	81,298,980	0.71	0.66	0.62	0.04	0.09	0.08	1.14 [1.10-1.17]*	1.13 [1.09-1.16]*
R16	20,570	81,386,920	0.29	0.24	0.23	0.04	0.06	0.05	1.27 [1.21-1.33]*	1.23 [1.17-1.29]*
R17	15,740	81,357,570	0.20	0.19	0.17	0.01	0.03	0.03	1.19 [1.12-1.25]*	1.18 [1.12-1.25]*
R18	40,870	81,331,060	0.52	0.50	0.44	0.02	0.08	0.08	1.17 [1.13-1.21]*	1.17 [1.13-1.21]*
R19	158,450	79,215,830	2.19	1.95	1.76	0.24	0.44	0.33	1.25 [1.23-1.27]*	1.18 [1.16-1.20]*
R20	45,150	80,884,180	0.59	0.55	0.50	0.03	0.08	0.05	1.16 [1.13-1.20]*	1.10 [1.06-1.13]*
R21	68,450	79,860,330	1.10	0.80	0.81	0.30	0.30	0.27	1.37 [1.34-1.40]*	1.32 [1.29-1.35]*
R22	33,100	80,883,670	0.48	0.39	0.37	0.09	0.11	0.10	1.31 [1.27-1.36]*	1.25 [1.20-1.29]*
R23	27,980	81,023,600	0.40	0.33	0.34	0.07	0.06	0.05	1.18 [1.14-1.23]*	1.14 [1.10-1.19]*
R25	26,650	81,075,410	0.35	0.32	0.30	0.03	0.05	0.04	1.16 [1.12-1.21]*	1.11 [1.07-1.16]*
R26	199,250	80,702,410	2.66	2.42	2.31	0.24	0.34	0.31	1.15 [1.13-1.17]*	1.13 [1.11-1.15]*
R27	8,000	81,506,630	0.11	0.10	0.09	0.01	0.01	0.01	1.12 [1.04-1.21]*	1.09 [1.01-1.18]
R29	261,880	80,059,520	3.49	3.22	3.05	0.27	0.44	0.38	1.14 [1.13-1.16]*	1.12 [1.11-1.14]*
R30	20,910	81,210,550	0.29	0.25	0.24	0.04	0.05	0.04	1.21 [1.16-1.27]*	1.14 [1.09-1.20]*
R31	129,590	78,761,260	1.68	1.64	1.51	0.04	0.17	0.14	1.11 [1.09-1.13]*	1.09 [1.07-1.11]*
R32	104,490	80,866,240	1.36	1.27	1.21	0.09	0.15	0.14	1.13 [1.10-1.15]*	1.11 [1.09-1.14]*
R33	188,060	79,946,970	2.46	2.32	2.20	0.14	0.27	0.23	1.12 [1.10-1.14]*	1.10 [1.09-1.12]*
R34	5,190	81,574,200	0.07	0.06	0.06	0.01	0.01	0.01	1.23 [1.11-1.35]*	1.20 [1.09-1.32]*
R35	31,880	80,787,160	0.44	0.38	0.35	0.05	0.08	0.06	1.23 [1.19-1.28]*	1.17 [1.12-1.21]*
R36	700	81,597,920	0.01	0.01	0.01	0.00	0.00	0.00	1.19 [0.92-1.54]	1.15 [0.88-1.48]
R39	37,540	80,759,680	0.50	0.46	0.41	0.04	0.09	0.07	1.22 [1.18-1.26]*	1.16 [1.12-1.20]*
R40	31,520	81,326,580	0.42	0.38	0.36	0.04	0.06	0.05	1.15 [1.11-1.20]*	1.13 [1.08-1.17]*
R41	183,140	80,526,070	2.41	2.24	2.12	0.17	0.29	0.27	1.14 [1.12-1.16]*	1.13 [1.11-1.15]*
R42	104,580	80,284,090	1.38	1.28	1.20	0.10	0.19	0.13	1.16 [1.13-1.18]*	1.10 [1.08-1.13]*
R43	1,410	81,595,460	0.02	0.02	0.02	0.00	0.00	0.00	1.24 [1.04-1.49]	1.17 [0.98-1.41]
R44	27,780	81,405,630	0.36	0.34	0.32	0.03	0.05	0.04	1.15 [1.10-1.19]*	1.12 [1.08-1.17]*
R45	63,000	81,172,120	0.85	0.76	0.76	0.09	0.09	0.08	1.12 [1.09-1.15]*	1.10 [1.07-1.13]*
R46	18,010	81,493,330	0.23	0.22	0.21	0.01	0.02	0.02	1.10 [1.04-1.16]*	1.11 [1.05-1.17]*
R47	52,310	81,169,530	0.66	0.64	0.59	0.02	0.07	0.06	1.11 [1.08-1.15]*	1.10 [1.06-1.13]*
R48	2,070	81,591,160	0.03	0.02	0.03	0.01	0.01	0.00	1.23 [1.06-1.42]*	1.16 [1.00-1.35]
R49	10,460	81,381,110	0.14	0.13	0.12	0.02	0.03	0.02	1.23 [1.15-1.31]*	1.15 [1.08-1.23]*
R50	112,410	79,302,880	1.60	1.37	1.38	0.23	0.22	0.18	1.16 [1.14-1.18]*	1.13 [1.11-1.15]*
R51	148,830	78,643,690	2.10	1.84	1.77	0.25	0.33	0.23	1.19 [1.17-1.21]*	1.12 [1.10-1.14]*
R52	25,280	81,315,440	0.35	0.30	0.27	0.05	0.08	0.06	1.28 [1.23-1.34]*	1.20 [1.15-1.25]*
R53	100,410	80,687,600	1.35	1.22	1.16	0.13	0.19	0.15	1.17 [1.14-1.19]*	1.13 [1.10-1.16]*
R54	118,780	81,141,370	1.55	1.44	1.38	0.11	0.17	0.17	1.13 [1.10-1.15]*	1.12 [1.10-1.14]*
R55	181,620	79,085,190	2.42	2.27	2.13	0.15	0.29	0.25	1.14 [1.12-1.16]*	1.11 [1.09-1.13]*
R56	85,100	79,525,880	1.13	1.05	1.06	0.08	0.08	0.07	1.07 [1.05-1.10]*	1.06 [1.04-1.09]*
R57	16,150	81,556,560	0.20	0.20	0.18	0.01	0.03	0.03	1.15 [1.09-1.21]*	1.15 [1.08-1.21]*
R58	2,600	81,584,790	0.04	0.03	0.03	0.01	0.01	0.01	1.30 [1.14-1.48]*	1.29 [1.13-1.48]*
R59	52,360	80,851,360	0.75	0.62	0.59	0.13	0.16	0.15	1.27 [1.24-1.31]*	1.26 [1.22-1.29]*
R60	87,450	81,069,580	1.21	1.05	0.95	0.16	0.25	0.22	1.27 [1.24-1.30]*	1.22 [1.19-1.25]*
R61	9,720	81,434,020	0.13	0.12	0.11	0.02	0.03	0.02	1.23 [1.15-1.32]*	1.16 [1.09-1.25]*
R62	15,270	80,998,400	0.21	0.18	0.21	0.02	0.00	−0.01	0.99 [0.94-1.05]	0.97 [0.92-1.02]
R63	183,790	79,277,050	2.50	2.27	2.12	0.23	0.38	0.32	1.18 [1.16-1.20]*	1.14 [1.13-1.16]*
R64	4,830	81,600,160	0.06	0.06	0.06	0.00	0.01	0.01	1.09 [0.99-1.21]	1.12 [1.01-1.25]
R65	11,520	81,579,380	0.15	0.14	0.13	0.01	0.02	0.02	1.18 [1.11-1.26]*	1.15 [1.08-1.23]*
R68	18,200	81,260,350	0.24	0.22	0.21	0.02	0.03	0.03	1.17 [1.11-1.23]*	1.16 [1.10-1.23]*
R69	139,970	73,746,920	2.01	1.87	1.73	0.14	0.28	0.24	1.16 [1.14-1.18]*	1.13 [1.11-1.15]*
R70	1,240	81,577,230	0.02	0.01	0.01	0.00	0.00	0.00	1.35 [1.12-1.62]*	1.28 [1.06-1.55]*
R71	1,080	81,596,870	0.01	0.01	0.01	0.00	0.00	0.00	1.11 [0.90-1.36]	1.09 [0.88-1.35]
R72	5,980	81,544,140	0.08	0.07	0.07	0.00	0.01	0.01	1.15 [1.05-1.26]*	1.13 [1.03-1.23]
R73	22,880	81,348,630	0.31	0.27	0.25	0.04	0.06	0.05	1.25 [1.20-1.31]*	1.19 [1.14-1.25]*
R74	10,260	81,511,670	0.13	0.12	0.12	0.01	0.02	0.01	1.15 [1.08-1.23]*	1.12 [1.04-1.20]*
R76	2,720	81,563,050	0.04	0.03	0.03	0.01	0.01	0.01	1.29 [1.14-1.46]*	1.23 [1.08-1.40]*
R77	6,370	81,565,830	0.09	0.08	0.07	0.01	0.02	0.01	1.23 [1.13-1.34]*	1.18 [1.08-1.29]*
R78	820	81,593,170	0.01	0.01	0.01	0.00	0.00	0.00	1.04 [0.82-1.33]	1.05 [0.82-1.35]
R79	122,040	80,488,140	1.60	1.50	1.37	0.11	0.23	0.19	1.17 [1.15-1.19]*	1.14 [1.11-1.16]*
R80	4,940	81,504,550	0.07	0.06	0.06	0.01	0.01	0.01	1.23 [1.12-1.36]*	1.20 [1.09-1.32]*
R81	1,250	81,508,030	0.02	0.02	0.01	0.00	0.00	0.00	1.20 [0.99-1.44]	1.17 [0.97-1.42]
R82	10,600	81,465,750	0.14	0.13	0.12	0.01	0.01	0.01	1.12 [1.05-1.20]*	1.10 [1.02-1.17]*
R84	3,530	81,583,100	0.05	0.04	0.04	0.01	0.01	0.00	1.19 [1.06-1.33]*	1.08 [0.96-1.21]
R85	1,780	81,557,380	0.03	0.02	0.02	0.00	0.01	0.00	1.32 [1.13-1.54]*	1.22 [1.04-1.43]

R87	7,550	81,001,980	0.09	0.09	0.08	0.00	0.01	0.00	1.07 [0.99-1.16]	1.06 [0.97-1.15]
R89	1,720	81,588,300	0.02	0.02	0.02	0.00	0.01	0.01	1.31 [1.11-1.53]*	1.26 [1.07-1.49]*
R90	5,490	81,545,570	0.07	0.07	0.06	0.00	0.01	0.01	1.17 [1.07-1.28]*	1.14 [1.04-1.26]*
R91	57,990	81,118,200	0.77	0.70	0.64	0.06	0.13	0.11	1.20 [1.17-1.24]*	1.17 [1.14-1.21]*
R93	60,270	80,965,800	0.77	0.74	0.67	0.03	0.10	0.08	1.14 [1.11-1.18]*	1.12 [1.08-1.15]*
R94	106,370	80,560,420	1.42	1.30	1.19	0.12	0.22	0.20	1.19 [1.16-1.21]*	1.16 [1.14-1.19]*
hospitalised - S00-T88										
S00	114,120	80,202,090	1.50	1.40	1.38	0.09	0.11	0.10	1.08 [1.06-1.11]*	1.07 [1.05-1.09]*
S01	118,230	79,884,540	1.57	1.46	1.45	0.11	0.12	0.11	1.08 [1.06-1.11]*	1.07 [1.05-1.09]*
S02	40,280	80,453,570	0.51	0.50	0.49	0.01	0.02	0.01	1.04 [1.00-1.07]	1.02 [0.99-1.06]
S03	1,880	81,578,420	0.03	0.02	0.02	0.00	0.00	0.00	1.07 [0.92-1.25]	1.06 [0.90-1.24]
S05	9,820	81,429,500	0.12	0.12	0.11	0.00	0.01	0.01	1.10 [1.02-1.18]	1.09 [1.02-1.17]
S06	31,970	81,170,680	0.40	0.39	0.38	0.01	0.02	0.02	1.06 [1.01-1.10]*	1.05 [1.01-1.09]
S09	62,400	80,015,610	0.84	0.77	0.76	0.07	0.08	0.07	1.11 [1.08-1.14]*	1.08 [1.05-1.12]*
S10	3,500	81,547,010	0.04	0.04	0.04	0.00	0.00	0.00	0.97 [0.86-1.10]	0.96 [0.85-1.09]
S11	1,770	81,575,210	0.02	0.02	0.02	0.00	0.00	0.00	1.16 [0.98-1.36]	1.16 [0.98-1.38]
S12	7,770	81,525,610	0.10	0.09	0.09	0.01	0.01	0.01	1.15 [1.06-1.25]*	1.15 [1.06-1.24]*
S13	1,940	81,551,570	0.03	0.02	0.02	0.00	0.00	0.00	1.14 [0.98-1.33]	1.10 [0.94-1.29]
S14	1,440	81,583,080	0.02	0.02	0.02	0.00	0.00	0.00	1.01 [0.84-1.22]	1.02 [0.84-1.24]
S19	2,620	81,541,410	0.03	0.03	0.03	0.00	0.00	0.00	1.16 [1.02-1.32]	1.08 [0.95-1.24]
S20	12,180	81,464,340	0.16	0.15	0.14	0.01	0.02	0.01	1.12 [1.05-1.19]*	1.08 [1.02-1.16]
S21	2,320	81,560,180	0.03	0.03	0.03	0.00	0.00	0.00	1.05 [0.91-1.22]	1.06 [0.91-1.23]
S22	38,960	81,151,780	0.50	0.48	0.43	0.02	0.06	0.05	1.15 [1.11-1.19]*	1.12 [1.08-1.16]*
S27	11,590	81,429,970	0.14	0.14	0.13	−0.01	0.01	0.01	1.04 [0.97-1.11]	1.04 [0.97-1.11]
S29	3,150	81,566,220	0.04	0.04	0.03	0.01	0.01	0.01	1.32 [1.17-1.48]*	1.24 [1.10-1.41]*
S30	23,110	81,302,490	0.30	0.28	0.27	0.02	0.03	0.02	1.10 [1.05-1.16]*	1.07 [1.02-1.12]*
S31	6,600	81,456,140	0.09	0.08	0.08	0.01	0.00	0.00	1.05 [0.97-1.15]	1.05 [0.96-1.14]
S32	42,660	81,179,250	0.53	0.52	0.49	0.01	0.04	0.03	1.08 [1.04-1.12]*	1.06 [1.02-1.09]*
S33	530	81,598,500	0.01	0.01	0.01	0.00	0.00	0.00	1.00 [0.74-1.36]	0.92 [0.68-1.26]
S36	4,790	81,520,250	0.06	0.06	0.06	0.00	0.00	0.00	1.05 [0.95-1.16]	1.05 [0.95-1.16]
S37	3,730	81,553,990	0.04	0.05	0.04	−0.01	0.00	0.00	0.96 [0.85-1.08]	0.95 [0.84-1.08]
S39	8,850	81,430,610	0.12	0.11	0.10	0.01	0.01	0.01	1.12 [1.04-1.20]*	1.07 [0.99-1.15]
S40	17,780	81,454,700	0.23	0.22	0.21	0.01	0.02	0.01	1.10 [1.04-1.16]*	1.07 [1.01-1.13]
S41	6,940	81,526,630	0.09	0.08	0.08	0.01	0.01	0.01	1.14 [1.05-1.24]*	1.10 [1.01-1.20]
S42	58,220	80,647,580	0.76	0.71	0.70	0.04	0.05	0.05	1.08 [1.05-1.11]*	1.07 [1.04-1.10]*
S43	8,340	81,465,720	0.10	0.10	0.10	0.00	0.01	0.00	1.06 [0.98-1.14]	1.05 [0.97-1.13]
S46	3,610	81,563,490	0.04	0.04	0.04	0.00	0.00	0.00	1.06 [0.95-1.20]	1.02 [0.90-1.15]
S49	5,290	81,551,120	0.07	0.06	0.06	0.01	0.01	0.01	1.18 [1.07-1.30]*	1.14 [1.03-1.26]*
S50	19,430	81,446,070	0.25	0.23	0.23	0.02	0.02	0.01	1.09 [1.03-1.14]*	1.06 [1.01-1.12]
S51	27,290	81,320,790	0.36	0.33	0.32	0.03	0.04	0.03	1.13 [1.09-1.18]*	1.10 [1.05-1.14]*
S52	87,620	79,385,340	1.17	1.09	1.14	0.08	0.03	0.01	1.02 [1.00-1.05]	1.01 [0.99-1.04]
S53	2,490	81,542,770	0.03	0.03	0.03	0.00	0.00	0.00	1.03 [0.90-1.18]	1.01 [0.88-1.16]
S54	1,060	81,574,880	0.01	0.01	0.01	0.00	0.00	0.00	1.20 [0.98-1.47]	1.15 [0.93-1.43]
S55	450	81,595,920	0.01	0.01	0.00	0.00	0.00	0.00	1.35 [0.99-1.84]	1.43 [1.04-1.97]
S56	3,610	81,506,350	0.04	0.05	0.04	0.00	0.00	0.00	0.92 [0.82-1.04]	0.93 [0.82-1.05]
S59	2,260	81,580,010	0.03	0.03	0.03	0.00	0.00	0.00	1.15 [0.99-1.33]	1.11 [0.95-1.29]
S60	15,390	81,432,450	0.20	0.19	0.19	0.01	0.01	0.01	1.07 [1.01-1.13]	1.05 [0.99-1.11]
S61	58,400	80,262,880	0.72	0.73	0.73	−0.01	0.00	−0.01	0.99 [0.97-1.02]	0.98 [0.95-1.01]
S62	39,560	80,638,870	0.49	0.49	0.49	0.00	−0.01	−0.01	0.99 [0.95-1.02]	0.98 [0.95-1.02]
S63	6,140	81,459,670	0.08	0.07	0.07	0.00	0.01	0.01	1.13 [1.03-1.23]*	1.09 [1.00-1.19]
S64	7,530	81,354,260	0.08	0.09	0.09	−0.01	−0.01	−0.01	0.90 [0.83-0.98]	0.90 [0.83-0.98]
S65	2,080	81,546,380	0.02	0.03	0.02	0.00	0.00	0.00	0.96 [0.83-1.13]	0.98 [0.84-1.15]
S66	13,460	81,129,770	0.15	0.17	0.16	−0.02	−0.01	−0.01	0.95 [0.89-1.00]	0.95 [0.89-1.01]
S67	2,110	81,533,390	0.02	0.03	0.03	0.00	0.00	0.00	0.87 [0.75-1.02]	0.88 [0.75-1.03]
S68	4,860	81,455,230	0.06	0.06	0.06	0.00	0.00	0.00	0.92 [0.83-1.02]	0.92 [0.84-1.02]
S69	5,950	81,509,390	0.08	0.07	0.07	0.01	0.01	0.01	1.17 [1.07-1.28]*	1.13 [1.03-1.24]*
S70	17,690	81,459,540	0.23	0.21	0.21	0.01	0.02	0.01	1.08 [1.02-1.14]*	1.06 [1.00-1.12]
S71	3,880	81,533,700	0.05	0.05	0.05	0.00	0.00	0.00	1.03 [0.92-1.15]	1.01 [0.90-1.13]
S72	110,390	80,501,790	1.42	1.36	1.31	0.06	0.11	0.11	1.08 [1.06-1.11]*	1.09 [1.06-1.11]*
S73	1,700	81,580,490	0.02	0.02	0.02	0.00	0.00	0.00	1.05 [0.88-1.24]	1.02 [0.86-1.22]
S76	3,310	81,555,670	0.04	0.04	0.04	0.00	0.00	0.00	1.09 [0.96-1.23]	1.05 [0.92-1.18]
S79	9,110	81,530,370	0.12	0.11	0.10	0.01	0.02	0.02	1.17 [1.09-1.27]*	1.15 [1.07-1.24]*
S80	34,070	81,288,440	0.44	0.42	0.40	0.02	0.03	0.02	1.09 [1.04-1.13]*	1.04 [1.00-1.08]
S81	25,060	81,310,840	0.33	0.30	0.29	0.03	0.04	0.03	1.15 [1.10-1.20]*	1.08 [1.03-1.13]*
S82	63,730	79,877,290	0.79	0.80	0.76	0.00	0.03	0.02	1.04 [1.02-1.07]*	1.03 [1.00-1.06]
S83	8,470	81,365,050	0.11	0.10	0.10	0.00	0.01	0.00	1.06 [0.98-1.14]	1.02 [0.94-1.10]
S86	4,080	81,463,130	0.05	0.05	0.05	0.00	0.00	0.00	1.10 [0.99-1.22]	1.04 [0.93-1.16]
S89	7,170	81,512,030	0.09	0.09	0.08	0.01	0.01	0.01	1.12 [1.04-1.22]*	1.08 [0.99-1.17]
S90	8,850	81,502,400	0.11	0.11	0.10	0.00	0.00	0.00	1.04 [0.96-1.12]	1.01 [0.93-1.09]
S91	9,090	81,433,920	0.12	0.11	0.11	0.01	0.01	0.01	1.08 [1.01-1.17]	1.06 [0.98-1.14]
S92	13,660	81,303,660	0.17	0.17	0.16	0.01	0.02	0.01	1.10 [1.04-1.17]*	1.08 [1.01-1.14]
S93	4,540	81,523,720	0.06	0.06	0.05	0.00	0.01	0.00	1.11 [1.00-1.23]	1.05 [0.95-1.17]
S96	1,010	81,585,440	0.01	0.01	0.01	0.00	0.00	0.00	1.16 [0.94-1.43]	1.06 [0.85-1.32]
S99	3,650	81,557,000	0.05	0.04	0.04	0.00	0.00	0.00	1.08 [0.97-1.21]	1.03 [0.92-1.16]
T00	7,860	81,536,810	0.10	0.10	0.09	0.01	0.01	0.00	1.08 [0.99-1.17]	1.04 [0.96-1.13]
T01	3,780	81,574,850	0.05	0.05	0.05	0.00	0.00	0.00	1.09 [0.97-1.22]	1.05 [0.93-1.18]
T02	2,340	81,571,610	0.03	0.03	0.03	0.00	0.00	0.00	1.05 [0.91-1.21]	1.05 [0.90-1.21]
T09	1,270	81,586,350	0.02	0.02	0.01	0.00	0.00	0.00	1.11 [0.92-1.35]	1.09 [0.89-1.33]
T11	5,270	81,557,050	0.07	0.06	0.06	0.01	0.01	0.01	1.15 [1.05-1.27]*	1.11 [1.00-1.23]
T13	3,300	81,565,470	0.04	0.04	0.04	0.00	0.00	0.00	1.11 [0.98-1.26]	1.06 [0.94-1.21]
T14	3,080	81,493,750	0.04	0.04	0.04	0.00	0.00	0.00	1.02 [0.90-1.16]	0.98 [0.87-1.12]
T15	710	81,586,950	0.01	0.01	0.01	0.00	0.00	0.00	0.95 [0.74-1.23]	0.95 [0.74-1.23]
T16	7,430	81,446,920	0.10	0.09	0.10	0.02	0.00	0.00	1.02 [0.94-1.10]	1.00 [0.93-1.08]
T17	11,950	81,369,370	0.16	0.14	0.15	0.01	0.01	0.00	1.07 [1.00-1.14]	1.03 [0.97-1.10]
T18	9,140	81,411,020	0.12	0.11	0.11	0.01	0.01	0.00	1.07 [0.99-1.14]	1.02 [0.95-1.10]
T19	870	81,590,050	0.01	0.01	0.01	0.00	0.00	0.00	1.17 [0.93-1.47]	1.15 [0.91-1.45]
T20	3,920	81,500,220	0.05	0.05	0.05	0.00	0.00	0.00	0.99 [0.88-1.10]	0.99 [0.89-1.11]
T21	5,800	81,472,200	0.07	0.07	0.07	0.00	0.00	0.00	0.99 [0.91-1.09]	1.00 [0.91-1.09]
T22	4,860	81,487,670	0.06	0.06	0.06	0.00	0.00	0.00	0.94 [0.85-1.04]	0.95 [0.85-1.05]
T23	4,790	81,482,320	0.06	0.06	0.06	0.00	0.00	0.00	0.97 [0.88-1.07]	0.97 [0.88-1.08]
T24	4,100	81,517,750	0.05	0.05	0.05	0.00	−0.01	−0.01	0.89 [0.79-0.99]	0.90 [0.80-1.00]
T25	2,120	81,560,260	0.02	0.03	0.03	0.00	0.00	0.00	0.89 [0.76-1.03]	0.87 [0.75-1.02]
T29	920	81,586,850	0.01	0.01	0.01	0.00	0.00	0.00	0.94 [0.75-1.19]	0.94 [0.74-1.19]
T31	13,100	81,297,810	0.16	0.16	0.17	0.00	−0.01	−0.01	0.94 [0.89-1.00]	0.94 [0.89-1.00]
T36	2,880	81,521,450	0.04	0.03	0.03	0.01	0.00	0.00	1.14 [1.00-1.29]	1.11 [0.98-1.26]
T37	1,210	81,578,700	0.02	0.01	0.01	0.00	0.00	0.00	1.21 [1.00-1.47]	1.20 [0.98-1.46]
T38	5,210	81,516,650	0.07	0.06	0.06	0.01	0.01	0.01	1.14 [1.04-1.25]*	1.09 [0.99-1.20]
T39	51,500	80,036,120	0.67	0.64	0.66	0.04	0.01	0.01	1.02 [0.99-1.05]	1.01 [0.98-1.04]
T40	25,930	81,110,930	0.33	0.32	0.30	0.01	0.03	0.02	1.08 [1.04-1.13]*	1.06 [1.01-1.10]
T41	620	81,599,690	0.01	0.01	0.01	0.00	0.00	0.00	1.14 [0.87-1.49]	1.11 [0.84-1.47]
T42	18,920	81,057,790	0.23	0.23	0.22	−0.01	0.01	0.01	1.05 [1.00-1.10]	1.03 [0.98-1.08]
T43	28,310	80,786,820	0.36	0.35	0.34	0.01	0.02	0.01	1.06 [1.01-1.10]*	1.04 [1.00-1.09]
T44	6,370	81,468,970	0.08	0.08	0.07	0.00	0.01	0.00	1.07 [0.98-1.16]	1.06 [0.97-1.16]
T45	8,230	81,438,110	0.11	0.10	0.10	0.02	0.01	0.01	1.15 [1.07-1.24]*	1.11 [1.03-1.20]*
T46	5,970	81,519,360	0.08	0.07	0.07	0.00	0.01	0.01	1.12 [1.02-1.23]	1.09 [0.99-1.19]
T47	3,370	81,548,510	0.04	0.04	0.04	0.00	0.00	0.00	1.08 [0.96-1.22]	1.04 [0.92-1.17]
T48	1,680	81,562,120	0.02	0.02	0.02	0.00	0.00	0.00	1.11 [0.95-1.30]	1.01 [0.85-1.19]

T49	1,410	81,564,820	0.02	0.02	0.02	0.00	0.00	0.00	0.99 [0.82-1.18]	0.97 [0.81-1.16]
T50	7,580	81,350,760	0.09	0.09	0.09	0.00	0.00	0.00	1.05 [0.97-1.14]	1.02 [0.94-1.11]
T51	23,490	80,912,660	0.29	0.29	0.27	−0.01	0.02	0.02	1.06 [1.01-1.11]	1.06 [1.01-1.11]
T52	1,350	81,568,250	0.02	0.02	0.02	0.00	0.00	0.00	0.87 [0.72-1.05]	0.86 [0.71-1.04]
T54	1,360	81,579,330	0.02	0.02	0.02	0.00	0.00	0.00	0.95 [0.79-1.15]	0.94 [0.78-1.14]
T56	700	81,594,260	0.01	0.01	0.01	0.00	0.00	0.00	1.10 [0.85-1.43]	1.12 [0.86-1.45]
T59	1,860	81,559,170	0.02	0.02	0.02	0.00	0.00	0.00	1.04 [0.89-1.23]	1.03 [0.87-1.21]
T62	630	81,587,760	0.01	0.01	0.01	0.00	0.00	0.00	1.21 [0.93-1.56]	1.15 [0.88-1.50]
T63	820	81,583,150	0.01	0.01	0.01	0.00	0.00	0.00	1.00 [0.78-1.28]	0.95 [0.74-1.22]
T65	1,480	81,571,230	0.02	0.02	0.02	0.00	0.00	0.00	1.01 [0.85-1.20]	0.99 [0.83-1.18]
T68	8,650	81,569,910	0.11	0.11	0.10	0.00	0.01	0.01	1.07 [0.99-1.16]	1.11 [1.03-1.21]*
T71	760	81,597,110	0.01	0.01	0.01	0.00	0.00	0.00	0.88 [0.68-1.14]	0.89 [0.68-1.16]
T74	1,400	81,578,440	0.02	0.02	0.02	0.00	0.00	0.00	0.91 [0.75-1.09]	0.89 [0.73-1.07]
T75	900	81,579,060	0.01	0.01	0.01	0.00	0.00	0.00	1.00 [0.80-1.26]	0.96 [0.76-1.22]
T78	20,970	81,000,580	0.55	0.19	0.19	0.36	0.36	0.32	2.90 [2.78-3.01]*	2.42 [2.32-2.52]*
T79	11,120	81,432,590	0.13	0.14	0.13	0.00	0.01	0.01	1.04 [0.98-1.12]	1.04 [0.97-1.12]
T80	5,420	81,518,400	0.08	0.06	0.06	0.01	0.02	0.01	1.26 [1.15-1.38]*	1.24 [1.13-1.36]*
T81	161,790	78,353,140	2.23	2.02	1.85	0.20	0.38	0.31	1.20 [1.18-1.22]*	1.16 [1.14-1.18]*
T82	42,020	81,058,860	0.55	0.51	0.46	0.04	0.09	0.08	1.19 [1.16-1.23]*	1.17 [1.13-1.21]*
T83	40,610	81,279,850	0.49	0.50	0.46	−0.01	0.04	0.03	1.08 [1.04-1.12]*	1.07 [1.03-1.11]*
T84	55,170	80,604,440	0.73	0.67	0.60	0.05	0.12	0.10	1.20 [1.17-1.24]*	1.17 [1.13-1.20]*
T85	36,850	81,075,090	0.49	0.45	0.41	0.04	0.08	0.07	1.19 [1.15-1.23]*	1.15 [1.11-1.20]*
T86	5,300	81,521,380	0.07	0.06	0.06	0.00	0.01	0.01	1.17 [1.07-1.28]*	1.17 [1.06-1.29]*
T87	3,180	81,563,140	0.04	0.04	0.03	0.00	0.01	0.01	1.21 [1.07-1.36]*	1.17 [1.04-1.32]
T88	10,810	81,338,860	0.16	0.13	0.12	0.03	0.04	0.03	1.36 [1.28-1.45]*	1.27 [1.19-1.35]*
T90	8,670	81,367,960	0.11	0.10	0.10	0.01	0.02	0.01	1.16 [1.08-1.25]*	1.14 [1.06-1.23]*
T91	4,180	81,515,540	0.05	0.05	0.04	0.00	0.01	0.00	1.14 [1.02-1.26]	1.08 [0.97-1.20]
T92	6,780	81,397,600	0.08	0.08	0.08	0.00	0.01	0.01	1.09 [1.00-1.18]	1.07 [0.98-1.16]
T93	8,770	81,354,830	0.11	0.11	0.10	0.00	0.01	0.01	1.14 [1.06-1.22]*	1.09 [1.01-1.18]
T94	830	81,591,120	0.01	0.01	0.01	0.00	0.00	0.00	1.11 [0.87-1.41]	1.06 [0.83-1.35]
T95	970	81,580,890	0.01	0.01	0.01	0.00	0.00	0.00	1.06 [0.85-1.31]	1.08 [0.86-1.34]
T98	1,560	81,579,630	0.02	0.02	0.02	0.00	0.00	0.00	1.08 [0.91-1.29]	1.04 [0.87-1.24]
hospitalised - U00-U89										
U07	51,060	81,559,080	0.69	0.61	0.57	0.08	0.12	0.09	1.21 [1.17-1.25]*	1.15 [1.12-1.19]*
U80	18,360	81,477,260	0.27	0.21	0.19	0.06	0.08	0.07	1.40 [1.33-1.47]*	1.35 [1.29-1.42]*
U81	1,070	81,604,370	0.01	0.01	0.01	0.00	0.00	0.00	1.16 [0.94-1.44]	1.14 [0.92-1.42]
U82	36,620	81,498,750	0.53	0.43	0.40	0.10	0.13	0.12	1.32 [1.27-1.37]*	1.28 [1.23-1.33]*
U83	21,740	81,548,800	0.31	0.26	0.24	0.06	0.07	0.06	1.29 [1.23-1.35]*	1.25 [1.20-1.31]*
U84	460	81,609,200	0.01	0.01	0.01	0.00	0.00	0.00	1.20 [0.87-1.66]	1.19 [0.86-1.66]
U88	4,470	81,582,070	0.06	0.05	0.05	0.01	0.01	0.01	1.16 [1.05-1.29]*	1.12 [1.01-1.24]
U89	5,130	81,575,890	0.07	0.06	0.06	0.01	0.02	0.01	1.29 [1.17-1.42]*	1.25 [1.13-1.38]*
hospitalised - V00-Y99										
V01	560	81,597,380	0.01	0.01	0.01	0.00	0.00	0.00	1.02 [0.76-1.36]	1.00 [0.74-1.33]
V03	8,530	81,340,650	0.11	0.10	0.11	0.00	0.00	0.00	0.98 [0.91-1.06]	0.97 [0.90-1.05]
V04	540	81,592,510	0.01	0.01	0.01	0.00	0.00	0.00	1.34 [1.00-1.78]	1.33 [0.99-1.78]
V09	2,220	81,572,890	0.03	0.03	0.03	0.00	0.00	0.00	1.19 [1.03-1.37]	1.13 [0.98-1.31]
V11	490	81,600,550	0.01	0.01	0.01	0.00	0.00	0.00	0.97 [0.71-1.31]	0.96 [0.71-1.32]
V13	2,270	81,539,150	0.02	0.03	0.03	0.00	−0.01	−0.01	0.81 [0.70-0.95]*	0.82 [0.70-0.96]
V17	1,010	81,588,190	0.01	0.01	0.01	0.00	0.00	0.00	0.87 [0.70-1.09]	0.88 [0.70-1.11]
V18	13,980	81,195,160	0.18	0.17	0.18	0.01	0.00	0.00	0.99 [0.93-1.05]	0.98 [0.92-1.04]
V19	2,200	81,550,890	0.03	0.03	0.03	0.00	0.00	0.00	0.95 [0.82-1.10]	0.94 [0.81-1.09]
V23	2,820	81,508,900	0.03	0.04	0.03	−0.01	0.00	0.00	0.89 [0.77-1.01]	0.91 [0.79-1.05]
V27	720	81,585,660	0.01	0.01	0.01	0.00	0.00	0.00	0.80 [0.61-1.06]	0.78 [0.59-1.04]
V28	4,050	81,478,400	0.04	0.05	0.05	−0.01	0.00	0.00	0.92 [0.82-1.03]	0.91 [0.81-1.03]
V29	1,010	81,567,890	0.01	0.01	0.01	0.00	0.00	0.00	1.08 [0.87-1.33]	1.06 [0.85-1.32]
V43	7,300	81,366,180	0.09	0.09	0.08	0.00	0.01	0.00	1.07 [0.99-1.16]	1.04 [0.95-1.12]
V44	660	81,585,990	0.01	0.01	0.01	0.00	0.00	0.00	1.15 [0.88-1.49]	1.13 [0.86-1.48]
V47	3,130	81,513,390	0.04	0.04	0.04	0.00	0.01	0.01	1.18 [1.05-1.33]*	1.16 [1.02-1.31]
V48	4,310	81,517,960	0.05	0.05	0.05	0.00	0.00	0.00	1.08 [0.97-1.20]	1.05 [0.94-1.17]
V49	3,100	81,492,160	0.04	0.04	0.04	0.00	0.00	0.00	1.06 [0.94-1.21]	1.06 [0.93-1.20]
V78	1,750	81,587,700	0.02	0.02	0.02	0.00	0.00	0.00	1.16 [0.98-1.37]	1.13 [0.96-1.34]
V79	570	81,598,950	0.01	0.01	0.01	0.00	0.00	0.00	1.07 [0.80-1.44]	1.00 [0.74-1.36]
V80	4,290	81,476,150	0.05	0.05	0.05	−0.01	−0.01	−0.01	0.88 [0.79-0.98]	0.87 [0.78-0.98]
V82	440	81,603,210	0.01	0.01	0.00	0.00	0.00	0.00	1.39 [1.02-1.90]	1.41 [1.02-1.93]
V86	1,110	81,589,010	0.01	0.01	0.01	0.00	0.00	0.00	1.05 [0.85-1.29]	1.03 [0.84-1.27]
V89	1,290	81,523,200	0.02	0.02	0.01	0.00	0.00	0.00	1.15 [0.96-1.39]	1.10 [0.91-1.34]
W00	6,310	81,465,120	0.07	0.08	0.07	−0.01	0.00	0.00	1.04 [0.96-1.14]	1.03 [0.95-1.13]
W01	140,330	79,568,580	1.85	1.74	1.68	0.10	0.17	0.14	1.10 [1.08-1.12]*	1.08 [1.06-1.10]*
W02	6,510	81,410,890	0.09	0.08	0.09	0.01	0.00	0.00	0.99 [0.91-1.07]	0.97 [0.89-1.06]
W03	8,960	81,344,760	0.12	0.11	0.12	0.01	0.00	−0.01	0.98 [0.91-1.05]	0.95 [0.88-1.02]
W04	1,660	81,492,410	0.02	0.02	0.02	0.00	0.00	0.00	1.01 [0.86-1.20]	1.03 [0.88-1.22]
W05	3,340	81,581,160	0.04	0.04	0.04	0.00	0.01	0.01	1.15 [1.02-1.30]	1.14 [1.01-1.29]
W06	36,400	81,292,760	0.48	0.44	0.43	0.04	0.05	0.04	1.12 [1.08-1.16]*	1.10 [1.06-1.14]*
W07	21,230	81,352,910	0.27	0.26	0.25	0.02	0.02	0.02	1.08 [1.03-1.13]*	1.06 [1.01-1.12]
W08	7,150	81,474,430	0.09	0.09	0.09	0.01	0.00	0.00	1.04 [0.96-1.13]	1.03 [0.95-1.12]
W09	18,400	81,204,130	0.27	0.22	0.27	0.06	0.01	0.00	1.02 [0.97-1.07]	1.01 [0.96-1.06]
W10	58,540	80,599,250	0.75	0.72	0.67	0.02	0.07	0.06	1.11 [1.08-1.14]*	1.09 [1.06-1.12]*
W11	7,720	81,390,510	0.08	0.10	0.09	−0.01	0.00	0.00	0.99 [0.91-1.07]	0.97 [0.89-1.05]
W13	6,440	81,394,090	0.08	0.08	0.08	0.00	0.00	0.00	0.97 [0.89-1.06]	0.97 [0.89-1.06]
W14	2,090	81,536,070	0.03	0.02	0.03	0.01	0.00	0.00	1.00 [0.87-1.16]	1.01 [0.87-1.17]
W17	12,640	81,258,120	0.16	0.15	0.16	0.01	0.00	0.00	1.00 [0.94-1.06]	0.99 [0.93-1.05]
W18	98,890	80,698,910	1.31	1.20	1.15	0.11	0.16	0.14	1.14 [1.11-1.16]*	1.12 [1.09-1.14]*
W19	215,560	79,286,030	2.92	2.67	2.57	0.24	0.34	0.31	1.13 [1.12-1.15]*	1.12 [1.10-1.14]*
W20	5,830	81,449,950	0.07	0.07	0.07	0.00	0.00	0.00	0.99 [0.90-1.08]	0.97 [0.89-1.07]
W21	4,730	81,466,520	0.07	0.06	0.07	0.01	0.00	0.00	1.04 [0.95-1.15]	1.02 [0.92-1.13]
W22	30,700	81,000,470	0.41	0.37	0.38	0.03	0.03	0.02	1.07 [1.03-1.12]*	1.05 [1.01-1.09]
W23	19,030	81,166,550	0.24	0.23	0.25	0.01	−0.01	−0.01	0.97 [0.92-1.02]	0.96 [0.92-1.01]
W25	8,820	81,258,060	0.11	0.11	0.11	0.00	0.00	0.00	0.98 [0.91-1.05]	0.97 [0.90-1.05]
W26	8,380	81,424,060	0.10	0.10	0.10	0.00	0.00	0.00	0.99 [0.92-1.07]	0.98 [0.91-1.06]
W27	3,770	81,487,560	0.04	0.05	0.04	0.00	0.00	0.00	0.97 [0.87-1.09]	0.96 [0.85-1.08]
W29	3,910	81,509,180	0.04	0.05	0.04	−0.01	0.00	0.00	0.90 [0.80-1.01]	0.91 [0.81-1.02]
W31	3,430	81,487,010	0.04	0.04	0.04	−0.01	0.00	0.00	0.93 [0.82-1.05]	0.92 [0.81-1.04]
W44	21,220	81,150,610	0.29	0.26	0.27	0.03	0.01	0.00	1.05 [1.00-1.10]	1.02 [0.97-1.06]
W45	6,550	81,410,540	0.08	0.08	0.08	0.00	0.00	0.00	1.01 [0.93-1.10]	0.99 [0.90-1.07]
W46	1,020	81,603,260	0.01	0.01	0.01	0.00	0.00	0.00	0.86 [0.69-1.09]	0.85 [0.67-1.08]
W49	1,660	81,551,350	0.02	0.02	0.02	0.00	0.00	0.00	1.20 [1.02-1.42]	1.18 [1.00-1.39]
W50	8,910	81,359,480	0.12	0.11	0.11	0.02	0.01	0.01	1.06 [0.99-1.14]	1.05 [0.98-1.13]
W51	6,820	81,403,420	0.09	0.08	0.09	0.01	0.00	0.00	1.05 [0.97-1.13]	1.02 [0.94-1.11]
W54	9,950	81,409,710	0.12	0.12	0.13	−0.01	−0.01	−0.01	0.93 [0.86-0.99]	0.91 [0.85-0.98]*
W55	4,440	81,522,680	0.05	0.06	0.05	−0.01	0.00	−0.01	0.92 [0.83-1.03]	0.88 [0.79-0.98]
W57	5,650	81,503,830	0.08	0.07	0.06	0.01	0.02	0.02	1.29 [1.19-1.41]*	1.24 [1.13-1.36]*
W60	510	81,600,230	0.01	0.01	0.01	0.00	0.00	0.00	1.29 [0.97-1.71]	1.24 [0.93-1.66]
W64	1,160	81,595,720	0.01	0.01	0.01	0.00	0.00	0.00	1.11 [0.91-1.36]	1.11 [0.91-1.37]
W78	3,580	81,596,640	0.04	0.04	0.04	0.00	0.00	0.00	1.05 [0.93-1.19]	1.06 [0.94-1.20]
W79	5,380	81,565,780	0.07	0.06	0.06	0.01	0.01	0.01	1.22 [1.11-1.34]*	1.18 [1.07-1.30]*
W80	2,250	81,596,270	0.03	0.03	0.03	0.00	0.00	0.00	1.10 [0.95-1.29]	1.06 [0.91-1.24]
W84	2,100	81,604,260	0.03	0.03	0.02	0.00	0.00	0.00	1.02 [0.87-1.20]	1.01 [0.86-1.19]

X08	600	81,596,800	0.01	0.01	0.01	0.00	0.00	0.00	0.98 [0.74-1.31]	0.96 [0.72-1.29]
X09	830	81,588,930	0.01	0.01	0.01	0.00	0.00	0.00	0.96 [0.75-1.24]	0.94 [0.73-1.21]
X10	3,920	81,512,100	0.05	0.05	0.05	0.00	0.00	0.00	0.99 [0.89-1.11]	0.99 [0.89-1.11]
X11	1,130	81,575,610	0.01	0.01	0.01	0.00	0.00	0.00	0.95 [0.77-1.17]	0.93 [0.75-1.15]
X12	2,280	81,545,120	0.03	0.03	0.03	0.00	0.00	0.00	0.92 [0.80-1.06]	0.91 [0.78-1.05]
X15	1,720	81,573,520	0.02	0.02	0.02	0.00	0.00	0.00	1.00 [0.85-1.17]	1.00 [0.85-1.18]
X16	1,180	81,588,880	0.01	0.01	0.01	0.00	0.00	0.00	0.95 [0.77-1.17]	0.95 [0.77-1.18]
X19	740	81,594,220	0.01	0.01	0.01	0.00	0.00	0.00	1.02 [0.80-1.31]	0.99 [0.77-1.28]
X23	1,070	81,584,320	0.01	0.01	0.01	0.00	0.00	0.00	1.24 [1.01-1.52]	1.18 [0.96-1.46]
X31	1,180	81,602,500	0.01	0.01	0.01	0.00	0.00	0.00	0.97 [0.78-1.21]	1.01 [0.80-1.26]
X40	13,870	81,251,810	0.17	0.17	0.17	0.00	0.00	0.00	1.02 [0.96-1.08]	1.00 [0.94-1.06]
X41	8,480	81,375,840	0.11	0.10	0.10	0.00	0.01	0.00	1.06 [0.99-1.14]	1.05 [0.97-1.13]
X42	7,960	81,495,760	0.10	0.10	0.09	0.01	0.01	0.01	1.13 [1.05-1.22]*	1.10 [1.02-1.19]
X43	1,800	81,573,040	0.02	0.02	0.02	0.00	0.00	0.00	1.02 [0.86-1.21]	1.02 [0.86-1.20]
X44	12,040	81,346,450	0.16	0.15	0.14	0.01	0.01	0.01	1.09 [1.03-1.16]*	1.06 [1.00-1.13]
X45	3,180	81,509,430	0.04	0.04	0.04	0.00	0.00	0.00	1.01 [0.89-1.14]	1.00 [0.88-1.14]
X46	920	81,579,010	0.01	0.01	0.01	0.00	0.00	0.00	0.91 [0.72-1.14]	0.90 [0.71-1.12]
X47	1,120	81,581,920	0.01	0.01	0.01	0.00	0.00	0.00	1.08 [0.88-1.33]	1.06 [0.86-1.31]
X49	4,490	81,478,370	0.07	0.05	0.05	0.02	0.02	0.01	1.31 [1.19-1.44]*	1.24 [1.12-1.36]*
X50	15,540	81,285,960	0.19	0.19	0.18	0.00	0.01	0.00	1.05 [0.99-1.11]	1.01 [0.95-1.07]
X51	1,030	81,587,710	0.01	0.01	0.01	0.00	0.00	0.00	1.06 [0.86-1.32]	1.04 [0.84-1.30]
X58	4,900	81,528,870	0.08	0.06	0.05	0.03	0.03	0.02	1.50 [1.36-1.64]*	1.38 [1.26-1.52]*
X59	65,630	80,132,300	0.89	0.80	0.78	0.09	0.11	0.09	1.14 [1.11-1.17]*	1.11 [1.08-1.14]*
X60	40,040	80,374,800	0.52	0.49	0.51	0.03	0.01	0.01	1.03 [0.99-1.06]	1.01 [0.98-1.05]
X61	30,720	80,704,550	0.38	0.38	0.37	0.00	0.02	0.01	1.05 [1.01-1.09]	1.04 [1.00-1.08]
X62	18,130	81,234,150	0.23	0.22	0.21	0.00	0.01	0.01	1.07 [1.01-1.12]	1.04 [0.98-1.09]
X63	4,510	81,512,000	0.06	0.05	0.05	0.00	0.00	0.00	1.06 [0.95-1.17]	1.05 [0.95-1.17]
X64	15,320	81,181,840	0.20	0.19	0.19	0.02	0.02	0.01	1.09 [1.03-1.15]*	1.07 [1.01-1.13]
X65	21,240	81,021,790	0.26	0.26	0.24	0.00	0.02	0.02	1.08 [1.03-1.13]*	1.07 [1.02-1.13]*
X69	1,580	81,574,240	0.02	0.02	0.02	0.00	0.00	0.00	0.95 [0.79-1.13]	0.94 [0.79-1.13]
X70	1,210	81,591,880	0.01	0.02	0.02	0.00	0.00	0.00	0.88 [0.71-1.08]	0.89 [0.72-1.09]
X78	10,230	81,414,310	0.13	0.12	0.13	0.01	0.00	0.00	1.01 [0.95-1.09]	0.99 [0.92-1.06]
X83	980	81,597,870	0.01	0.01	0.01	0.00	0.00	0.00	0.85 [0.67-1.07]	0.82 [0.65-1.04]
X84	1,430	81,585,310	0.02	0.02	0.02	0.00	0.00	0.00	0.97 [0.80-1.17]	0.91 [0.75-1.10]
X99	3,780	81,475,740	0.04	0.05	0.05	0.00	0.00	0.00	0.96 [0.85-1.07]	0.97 [0.87-1.10]
Y00	2,170	81,526,480	0.02	0.03	0.03	0.00	0.00	0.00	0.95 [0.81-1.11]	0.97 [0.83-1.14]
Y04	17,110	80,902,910	0.20	0.21	0.21	−0.01	−0.01	−0.01	0.97 [0.92-1.03]	0.97 [0.92-1.03]
Y07	1,500	81,573,720	0.02	0.02	0.02	0.00	0.00	0.00	0.88 [0.73-1.05]	0.87 [0.72-1.04]
Y08	850	81,581,940	0.01	0.01	0.01	0.00	0.00	0.00	0.86 [0.67-1.11]	0.89 [0.68-1.16]
Y09	2,510	81,489,410	0.03	0.03	0.03	0.00	0.00	0.00	1.02 [0.88-1.17]	1.02 [0.88-1.17]
Y28	640	81,587,680	0.01	0.01	0.01	0.00	0.00	0.00	0.93 [0.71-1.24]	0.87 [0.65-1.16]
Y34	560	81,584,930	0.01	0.01	0.01	0.00	0.00	0.00	1.02 [0.77-1.37]	0.99 [0.74-1.34]
Y40	18,560	81,367,390	0.28	0.22	0.20	0.06	0.08	0.07	1.40 [1.33-1.47]*	1.32 [1.26-1.39]*
Y41	3,870	81,559,070	0.06	0.05	0.04	0.01	0.01	0.01	1.29 [1.16-1.44]*	1.21 [1.08-1.35]*
Y42	15,240	81,442,400	0.22	0.18	0.16	0.04	0.05	0.04	1.33 [1.26-1.40]*	1.22 [1.15-1.28]*
Y43	30,430	81,341,340	0.39	0.37	0.34	0.02	0.05	0.05	1.15 [1.11-1.19]*	1.14 [1.10-1.19]*
Y44	10,610	81,515,460	0.14	0.13	0.12	0.01	0.02	0.02	1.19 [1.11-1.27]*	1.15 [1.07-1.23]*
Y45	24,130	81,343,150	0.33	0.29	0.27	0.04	0.07	0.05	1.25 [1.19-1.30]*	1.19 [1.14-1.25]*
Y46	3,680	81,563,690	0.05	0.04	0.04	0.01	0.01	0.01	1.23 [1.11-1.38]*	1.19 [1.06-1.33]*
Y47	1,840	81,588,900	0.02	0.02	0.02	0.00	0.00	0.00	1.17 [0.99-1.37]	1.13 [0.96-1.33]
Y48	2,100	81,579,960	0.03	0.03	0.02	0.00	0.00	0.00	1.20 [1.04-1.39]	1.14 [0.98-1.32]
Y49	5,880	81,544,440	0.08	0.07	0.06	0.01	0.02	0.02	1.28 [1.18-1.40]*	1.27 [1.16-1.39]*
Y51	8,950	81,520,030	0.12	0.11	0.10	0.01	0.02	0.02	1.19 [1.10-1.28]*	1.18 [1.10-1.27]*
Y52	21,640	81,430,220	0.29	0.26	0.23	0.03	0.06	0.05	1.24 [1.18-1.30]*	1.21 [1.15-1.27]*
Y53	4,150	81,577,550	0.06	0.05	0.05	0.01	0.01	0.01	1.31 [1.18-1.46]*	1.22 [1.10-1.36]*
Y54	16,690	81,501,780	0.23	0.20	0.18	0.04	0.05	0.04	1.29 [1.22-1.36]*	1.24 [1.17-1.30]*
Y55	2,280	81,583,440	0.03	0.03	0.03	0.01	0.01	0.00	1.32 [1.15-1.51]*	1.16 [1.01-1.34]
Y56	1,880	81,570,290	0.03	0.02	0.02	0.01	0.01	0.01	1.65 [1.43-1.90]*	1.51 [1.31-1.75]*
Y57	10,020	81,514,730	0.14	0.12	0.11	0.02	0.03	0.03	1.33 [1.24-1.42]*	1.25 [1.17-1.34]*
Y59	1,260	81,583,470	0.02	0.01	0.01	0.01	0.01	0.00	1.41 [1.18-1.69]*	1.30 [1.08-1.57]*
Y60	12,010	81,487,110	0.16	0.15	0.13	0.01	0.02	0.02	1.17 [1.10-1.24]*	1.13 [1.06-1.21]*
Y65	1,660	81,587,730	0.02	0.02	0.02	0.00	0.00	0.00	1.10 [0.93-1.30]	1.08 [0.91-1.28]
Y71	800	81,598,670	0.01	0.01	0.01	0.00	0.00	0.00	1.38 [1.09-1.74]*	1.32 [1.04-1.67]
Y73	740	81,600,600	0.01	0.01	0.01	0.00	0.00	0.00	0.92 [0.70-1.21]	0.89 [0.67-1.18]
Y77	760	81,601,740	0.01	0.01	0.01	0.00	0.00	0.00	1.05 [0.81-1.37]	1.03 [0.79-1.35]
Y79	1,690	81,577,640	0.02	0.02	0.02	0.00	0.00	0.00	1.15 [0.97-1.36]	1.10 [0.93-1.31]
Y82	650	81,598,200	0.01	0.01	0.01	0.00	0.00	0.00	1.20 [0.92-1.56]	1.19 [0.91-1.55]
Y83	218,840	77,712,220	3.01	2.77	2.54	0.24	0.47	0.38	1.18 [1.17-1.20]*	1.15 [1.13-1.16]*
Y84	49,800	81,143,380	0.64	0.61	0.55	0.04	0.09	0.08	1.16 [1.13-1.20]*	1.14 [1.10-1.18]*
Y85	5,210	81,479,470	0.07	0.06	0.06	0.00	0.01	0.01	1.18 [1.07-1.29]*	1.13 [1.02-1.24]
Y86	9,340	81,348,370	0.12	0.11	0.11	0.00	0.01	0.01	1.09 [1.02-1.17]	1.06 [0.98-1.14]
Y87	1,570	81,564,890	0.02	0.02	0.02	0.00	0.00	0.00	1.16 [0.98-1.37]	1.14 [0.96-1.36]
Y88	1,990	81,577,140	0.02	0.02	0.02	0.00	0.00	0.00	1.02 [0.87-1.20]	0.97 [0.83-1.14]
Y89	590	81,594,950	0.01	0.01	0.01	0.00	0.00	0.00	1.06 [0.80-1.40]	1.06 [0.80-1.42]
Y90	1,080	81,594,690	0.01	0.01	0.01	0.00	0.00	0.00	0.98 [0.80-1.21]	0.99 [0.80-1.23]
Y91	1,970	81,555,200	0.02	0.02	0.02	0.00	0.00	0.00	1.10 [0.94-1.29]	1.11 [0.95-1.31]
Y95	113,580	81,310,160	1.52	1.37	1.27	0.15	0.25	0.22	1.20 [1.17-1.22]*	1.17 [1.14-1.19]*
hospitalised - Z00-Z99										
Z00	4,520	81,426,880	0.06	0.06	0.05	0.00	0.01	0.00	1.10 [1.00-1.22]	1.07 [0.97-1.19]
Z01	26,600	80,106,450	0.63	0.26	0.27	0.37	0.35	0.30	2.28 [2.20-2.36]*	1.90 [1.83-1.98]*
Z02	17,230	81,402,100	0.21	0.21	0.19	0.00	0.02	0.01	1.10 [1.04-1.16]*	1.07 [1.01-1.13]
Z03	140,810	77,422,570	2.08	1.75	1.65	0.32	0.42	0.34	1.26 [1.23-1.28]*	1.20 [1.17-1.22]*
Z04	24,330	80,370,990	0.32	0.30	0.29	0.03	0.04	0.03	1.12 [1.08-1.17]*	1.09 [1.04-1.14]*
Z08	41,140	80,740,920	0.48	0.52	0.46	−0.03	0.02	0.02	1.05 [1.01-1.09]*	1.05 [1.01-1.09]*
Z09	83,650	79,753,210	1.10	1.04	0.94	0.07	0.17	0.12	1.18 [1.15-1.20]*	1.12 [1.10-1.15]*
Z11	260,640	81,037,100	3.49	3.15	3.04	0.34	0.46	0.37	1.15 [1.13-1.17]*	1.12 [1.10-1.13]*
Z12	53,890	80,923,370	0.64	0.67	0.58	−0.04	0.06	0.04	1.10 [1.06-1.13]*	1.07 [1.04-1.11]*
Z13	152,810	78,842,970	2.13	1.89	1.79	0.24	0.34	0.27	1.19 [1.17-1.21]*	1.14 [1.12-1.17]*
Z20	9,230	81,532,300	0.12	0.11	0.11	0.01	0.02	0.01	1.16 [1.08-1.25]*	1.12 [1.04-1.21]*
Z22	77,930	80,655,920	1.12	0.93	0.86	0.19	0.26	0.23	1.30 [1.27-1.33]*	1.26 [1.23-1.29]*
Z24	1,350	81,489,410	0.02	0.02	0.02	0.00	0.00	0.00	1.07 [0.89-1.29]	1.09 [0.90-1.32]
Z25	800	81,588,150	0.02	0.01	0.01	0.01	0.01	0.01	2.63 [2.15-3.22]*	2.17 [1.75-2.69]*
Z27	3,890	81,426,870	0.08	0.04	0.04	0.05	0.05	0.04	2.26 [2.06-2.48]*	2.04 [1.85-2.25]*
Z29	42,620	80,382,840	0.58	0.52	0.48	0.06	0.10	0.08	1.20 [1.16-1.24]*	1.16 [1.12-1.20]*
Z30	42,980	78,753,240	0.52	0.55	0.49	−0.03	0.03	0.02	1.06 [1.02-1.09]*	1.05 [1.01-1.08]
Z31	590	81,484,240	0.01	0.01	0.01	0.00	0.00	0.00	1.20 [0.91-1.57]	1.15 [0.86-1.53]
Z32	3,340	81,416,190	0.04	0.04	0.04	0.00	0.00	0.00	1.09 [0.97-1.23]	1.07 [0.95-1.21]
Z33	31,360	80,365,070	0.41	0.38	0.37	0.03	0.05	0.03	1.12 [1.08-1.17]*	1.08 [1.04-1.12]*
Z34	73,730	78,565,720	0.96	0.93	0.94	0.03	0.03	0.02	1.03 [1.00-1.05]	1.02 [0.99-1.04]
Z35	79,400	79,883,840	0.96	1.00	0.96	−0.05	0.00	0.00	1.00 [0.98-1.02]	1.00 [0.98-1.03]
Z36	12,550	80,304,960	0.16	0.16	0.15	0.00	0.01	0.01	1.05 [0.99-1.11]	1.05 [0.98-1.11]
Z37	153,410	66,916,600	2.98	2.11	2.99	0.87	−0.01	0.01	1.00 [0.98-1.01]	1.00 [0.99-1.02]
Z38	800	57,877,130	0.01	0.01	0.01	0.00	0.00	0.00	1.02 [0.80-1.31]	1.01 [0.79-1.29]
Z39	21,870	80,456,560	0.27	0.27	0.26	0.00	0.01	0.01	1.03 [0.98-1.08]	1.03 [0.98-1.08]
Z40	5,470	81,522,190	0.06	0.07	0.06	0.00	0.00	0.00	1.02 [0.93-1.12]	1.01 [0.92-1.12]
Z41	5,670	81,358,940	0.08	0.07	0.07	0.01	0.01	0.01	1.22 [1.12-1.33]*	1.21 [1.11-1.32]*
Z42	14,460	81,276,510	0.18	0.18	0.16	0.00	0.02	0.02	1.11 [1.05-1.17]*	1.11 [1.05-1.18]*
Z43	20,920	81,181,220	0.26	0.26	0.24	0.00	0.02	0.02	1.09 [1.04-1.14]*	1.08 [1.03-1.13]*

Z45	45,600	80,955,240	0.56	0.56	0.51	0.00	0.05	0.04	1.10 [1.06-1.13]*	1.07 [1.04-1.11]*
Z46	51,820	80,642,250	0.63	0.65	0.59	−0.02	0.04	0.04	1.07 [1.04-1.10]*	1.06 [1.03-1.09]*
Z47	38,390	80,279,350	0.52	0.47	0.48	0.05	0.04	0.03	1.08 [1.05-1.12]*	1.06 [1.03-1.10]*
Z48	14,840	81,121,260	0.20	0.18	0.17	0.02	0.03	0.02	1.15 [1.08-1.21]*	1.12 [1.06-1.19]*
Z49	8,790	81,492,870	0.12	0.11	0.09	0.01	0.02	0.02	1.25 [1.17-1.34]*	1.26 [1.17-1.36]*
Z50	303,650	79,443,320	4.05	3.77	3.56	0.28	0.49	0.42	1.14 [1.12-1.15]*	1.12 [1.10-1.13]*
Z51	356,290	78,073,660	4.78	4.51	4.14	0.27	0.64	0.56	1.16 [1.14-1.17]*	1.13 [1.12-1.15]*
Z52	1,590	81,560,440	0.02	0.02	0.02	0.00	0.00	0.00	0.94 [0.79-1.13]	0.96 [0.80-1.15]
Z53	305,670	75,087,910	4.45	3.97	3.66	0.48	0.79	0.64	1.21 [1.20-1.23]*	1.17 [1.15-1.18]*
Z54	2,320	81,529,700	0.03	0.03	0.03	0.00	0.01	0.01	1.27 [1.11-1.47]*	1.27 [1.10-1.46]*
Z55	1,320	81,588,450	0.02	0.01	0.02	0.01	0.00	0.00	1.18 [0.99-1.41]	1.15 [0.96-1.38]
Z56	3,760	81,549,650	0.04	0.05	0.04	0.00	0.00	0.00	1.07 [0.96-1.20]	1.04 [0.92-1.16]
Z57	11,140	81,532,410	0.14	0.13	0.12	0.01	0.03	0.02	1.21 [1.14-1.29]*	1.16 [1.09-1.24]*
Z58	8,480	81,532,670	0.12	0.10	0.10	0.02	0.02	0.02	1.24 [1.15-1.33]*	1.16 [1.08-1.25]*
Z59	8,840	81,488,960	0.10	0.11	0.10	−0.01	0.00	0.00	1.00 [0.93-1.08]	1.03 [0.96-1.12]
Z60	93,120	80,346,040	1.19	1.15	1.07	0.04	0.13	0.11	1.12 [1.09-1.15]*	1.10 [1.08-1.13]*
Z61	4,270	81,538,500	0.06	0.05	0.06	0.00	0.00	0.00	0.95 [0.86-1.06]	0.94 [0.85-1.05]
Z62	930	81,585,740	0.01	0.01	0.01	0.00	0.00	0.00	0.87 [0.69-1.08]	0.84 [0.67-1.06]
Z63	32,370	81,131,070	0.41	0.40	0.39	0.02	0.02	0.01	1.05 [1.01-1.09]	1.03 [0.99-1.07]
Z64	670	81,563,980	0.01	0.01	0.01	0.00	0.00	0.00	0.99 [0.75-1.30]	0.98 [0.74-1.30]
Z65	2,730	81,564,730	0.03	0.03	0.03	0.00	0.00	0.00	1.00 [0.87-1.14]	1.01 [0.88-1.16]
Z71	32,190	81,070,080	0.55	0.36	0.35	0.19	0.20	0.16	1.57 [1.51-1.62]*	1.43 [1.38-1.48]*
Z72	180,940	75,101,660	2.38	2.42	2.15	−0.03	0.23	0.25	1.11 [1.09-1.12]*	1.11 [1.10-1.13]*
Z73	51,680	81,334,090	0.67	0.63	0.59	0.04	0.08	0.06	1.13 [1.09-1.16]*	1.11 [1.07-1.14]*
Z74	39,090	81,339,670	0.49	0.48	0.44	0.01	0.05	0.05	1.12 [1.08-1.16]*	1.12 [1.08-1.16]*
Z75	77,150	81,182,540	0.98	0.94	0.89	0.04	0.09	0.09	1.10 [1.07-1.13]*	1.10 [1.07-1.13]*
Z76	5,500	80,573,310	0.07	0.07	0.06	0.00	0.01	0.01	1.15 [1.04-1.26]*	1.14 [1.04-1.25]*
Z80	80,340	80,228,440	0.98	1.01	0.90	−0.03	0.08	0.05	1.09 [1.06-1.12]*	1.06 [1.03-1.08]*
Z81	13,500	81,348,130	0.19	0.16	0.17	0.02	0.01	0.01	1.07 [1.01-1.13]	1.05 [0.99-1.11]
Z82	173,450	77,952,170	2.45	2.17	2.02	0.28	0.43	0.36	1.21 [1.19-1.23]*	1.17 [1.15-1.19]*
Z83	66,650	79,761,760	0.93	0.81	0.80	0.12	0.14	0.11	1.17 [1.14-1.20]*	1.13 [1.10-1.16]*
Z84	10,020	81,317,680	0.19	0.11	0.12	0.08	0.07	0.07	1.61 [1.52-1.71]*	1.57 [1.47-1.67]*
Z85	298,230	77,867,340	3.87	3.82	3.53	0.05	0.34	0.32	1.10 [1.08-1.11]*	1.09 [1.08-1.10]*
Z86	903,550	70,578,800	13.69	12.57	11.69	1.12	1.99	1.68	1.17 [1.16-1.18]*	1.14 [1.13-1.15]*
Z87	511,880	74,515,470	7.47	6.72	6.29	0.76	1.18	0.89	1.19 [1.18-1.20]*	1.13 [1.12-1.15]*
Z88	464,510	74,024,570	7.54	5.96	5.50	1.58	2.04	1.59	1.37 [1.36-1.38]*	1.27 [1.25-1.28]*
Z89	14,930	81,465,650	0.19	0.18	0.16	0.01	0.03	0.03	1.18 [1.11-1.24]*	1.17 [1.11-1.24]*
Z90	272,590	78,940,640	3.53	3.43	3.12	0.10	0.41	0.31	1.13 [1.12-1.15]*	1.10 [1.08-1.11]*
Z91	212,370	78,905,790	3.80	2.42	2.35	1.38	1.45	1.20	1.61 [1.59-1.64]*	1.46 [1.44-1.48]*
Z92	608,550	75,665,020	8.47	7.93	7.39	0.54	1.08	0.88	1.15 [1.14-1.16]*	1.12 [1.11-1.13]*
Z93	61,670	80,925,830	0.77	0.76	0.69	0.01	0.08	0.08	1.12 [1.09-1.15]*	1.11 [1.08-1.14]*
Z94	12,980	81,377,700	0.17	0.16	0.14	0.01	0.03	0.03	1.21 [1.14-1.28]*	1.20 [1.13-1.27]*
Z95	206,260	78,942,430	2.69	2.59	2.40	0.09	0.29	0.25	1.12 [1.10-1.14]*	1.10 [1.08-1.12]*
Z96	390,130	77,603,750	5.27	4.97	4.63	0.31	0.64	0.54	1.14 [1.13-1.15]*	1.11 [1.10-1.13]*
Z97	39,920	81,226,280	0.53	0.48	0.45	0.05	0.08	0.06	1.18 [1.14-1.22]*	1.14 [1.10-1.18]*
Z98	55,790	80,908,410	0.73	0.68	0.61	0.05	0.12	0.08	1.19 [1.16-1.23]*	1.13 [1.10-1.16]*
Z99	69,870	81,074,510	0.96	0.84	0.77	0.12	0.19	0.15	1.25 [1.22-1.28]*	1.19 [1.16-1.22]*

[1] Hazard ratios (99% confidence intervals) estimated from Cox models (stratifying on matched set) comparing people with eczema to those without eczema. Results are implicitly adjusted through matching on age, sex and general practice, and calendar time, as comparators entered the cohort on the same day as exposed individuals. * indicates that the result is significant under Bonferroni-correction.

[2] Estimated as the rate in the exposed * (1/crude hazard ratio)

[3] Rate in the exposed - observed rate in the unexposed

[4] Rate in the exposed - rate in the exposed * (1/crude hazard ratio)

[5] Rate in the exposed - rate in the exposed * (1/comorbidity-adjusted hazard ratio)

Supplementary Table 3: Hazard ratios and events from all cohorts

	Hazard ratio (99% confidence interval)										Events (in exposed)				
	crude					adjusted									
Outcome	any age	18+	40+	<18	hosp.	any age	18+	40+	<18	hosp.	any age	18+	40+	<18	hosp.
Certain infectious and parasitic diseases (A00-B99)															
A02	1.23 [1.05-1.44]	1.17 [0.97-1.42]	1.27 [1.02-1.58]	1.25 [0.97-1.61]	1.11 [0.93-1.31]	1.15 [0.98-1.36]	1.04 [0.86-1.27]	1.12 [0.90-1.41]	1.26 [0.97-1.63]	1.05 [0.89-1.25]	395	259	203	160	324
A04	1.27 [1.23-1.31]	1.28 [1.24-1.32]	1.28 [1.24-1.32]	1.08 [0.97-1.21]	1.19 [1.16-1.23]	1.21 [1.17-1.24]	1.18 [1.14-1.22]	1.17 [1.14-1.21]	1.03 [0.91-1.15]	1.15 [1.12-1.19]	11,231	10,718	9,774	814	9,899
A05	1.26 [0.93-1.69]	1.31 [0.96-1.80]			1.19 [0.87-1.62]	1.20 [0.89-1.63]	1.23 [0.89-1.70]			1.20 [0.87-1.64]	118	102	NA	NA	104
A07	1.21 [0.98-1.50]	1.19 [0.91-1.56]		1.52 [1.10-2.10]	1.17 [0.93-1.47]	1.14 [0.92-1.42]	1.04 [0.79-1.38]		1.44 [1.03-2.00]	1.14 [0.90-1.44]	216	132	NA	102	183
A08	1.20 [1.17-1.23]	1.31 [1.26-1.37]	1.31 [1.25-1.38]	1.14 [1.10-1.17]	1.15 [1.12-1.18]	1.15 [1.12-1.18]	1.17 [1.12-1.22]	1.17 [1.12-1.23]	1.11 [1.08-1.15]	1.12 [1.09-1.16]	14,673	5,645	4,555	9,469	13,130
A09	1.29 [1.28-1.31]	1.32 [1.30-1.34]	1.30 [1.27-1.32]	1.19 [1.15-1.22]	1.21 [1.20-1.23]	1.20 [1.18-1.22]	1.17 [1.15-1.19]	1.15 [1.13-1.17]	1.14 [1.10-1.17]	1.16 [1.14-1.18]	51,508	44,465	35,930	10,685	47,052
A15	1.09 [0.90-1.32]	1.13 [0.93-1.37]	1.29 [1.03-1.61]		1.15 [0.94-1.42]	1.06 [0.87-1.29]	1.08 [0.88-1.32]	1.17 [0.93-1.48]		1.12 [0.90-1.38]	261	240	186	NA	217
A16	1.33 [1.17-1.51]	1.41 [1.23-1.61]	1.43 [1.23-1.67]		1.30 [1.13-1.50]	1.28 [1.12-1.46]	1.30 [1.14-1.50]	1.30 [1.11-1.52]		1.24 [1.07-1.44]	610	554	429	NA	497
A18	1.19 [0.99-1.42]	1.35 [1.11-1.63]	1.35 [1.08-1.68]		1.22 [1.00-1.50]	1.14 [0.95-1.38]	1.31 [1.08-1.60]	1.28 [1.02-1.61]		1.18 [0.96-1.45]	286	263	193	NA	228
A31	1.41 [1.16-1.71]	1.63 [1.32-2.01]	1.69 [1.36-2.12]		1.35 [1.11-1.65]	1.26 [1.03-1.54]	1.39 [1.12-1.73]	1.43 [1.13-1.80]		1.21 [0.99-1.48]	266	235	208	NA	251
A38	1.08 [0.93-1.25]			1.05 [0.91-1.22]	1.03 [0.89-1.20]	1.06 [0.91-1.22]			1.03 [0.89-1.20]	1.02 [0.87-1.19]	469	NA	NA	456	430
A39	1.06 [0.92-1.21]	1.10 [0.82-1.46]		1.05 [0.91-1.22]	1.05 [0.90-1.22]	1.03 [0.90-1.18]	1.06 [0.79-1.42]		1.04 [0.89-1.21]	1.03 [0.88-1.21]	511	116	NA	421	415
A40	1.33 [1.25-1.43]	1.35 [1.26-1.45]	1.39 [1.29-1.49]	1.11 [0.90-1.35]	1.26 [1.18-1.35]	1.28 [1.20-1.37]	1.27 [1.18-1.36]	1.29 [1.20-1.39]	1.05 [0.86-1.30]	1.24 [1.16-1.33]	2,387	2,187	2,041	247	2,169
A41	1.25 [1.23-1.27]	1.26 [1.24-1.28]	1.25 [1.23-1.27]	1.10 [1.03-1.18]	1.19 [1.18-1.21]	1.21 [1.19-1.23]	1.17 [1.15-1.19]	1.17 [1.15-1.19]	1.08 [1.01-1.16]	1.18 [1.16-1.20]	42,650	41,175	38,875	2,275	39,444
A46	2.16 [1.72-2.72]	2.10 [1.67-2.65]	2.09 [1.64-2.66]		1.97 [1.53-2.53]	2.11 [1.67-2.66]	1.95 [1.54-2.48]	1.97 [1.54-2.53]		1.93 [1.50-2.49]	211	204	182	NA	171
A48	1.21 [0.98-1.49]	1.26 [1.00-1.59]	1.21 [0.95-1.55]		1.23 [0.98-1.54]	1.22 [0.99-1.51]	1.20 [0.95-1.52]	1.16 [0.91-1.49]		1.27 [1.01-1.60]	217	179	164	NA	190
A49	1.34 [1.29-1.40]	1.36 [1.31-1.42]	1.34 [1.28-1.40]	1.19 [1.06-1.34]	1.27 [1.22-1.32]	1.28 [1.23-1.34]	1.25 [1.20-1.31]	1.23 [1.18-1.29]	1.17 [1.04-1.32]	1.24 [1.19-1.29]	6,459	5,849	5,422	740	5,854
A63	1.49 [1.21-1.83]	1.47 [1.19-1.81]			1.45 [1.12-1.86]	1.35 [1.09-1.67]	1.31 [1.06-1.63]			1.38 [1.07-1.80]	216	205	NA	NA	141
A69	1.25 [0.99-1.59]	1.23 [0.94-1.61]			1.14 [0.89-1.47]	1.15 [0.90-1.47]	1.06 [0.80-1.41]			1.07 [0.82-1.38]	169	129	NA	NA	146
A86	1.01 [0.84-1.21]	1.09 [0.89-1.33]	1.18 [0.95-1.47]		1.03 [0.84-1.25]	0.98 [0.81-1.17]	1.01 [0.82-1.23]	1.08 [0.86-1.35]		0.99 [0.81-1.21]	291	238	199	NA	243
A87	1.31 [1.17-1.45]	1.40 [1.25-1.58]	1.38 [1.12-1.71]	1.27 [1.06-1.51]	1.16 [1.03-1.30]	1.20 [1.08-1.34]	1.22 [1.08-1.38]	1.19 [0.96-1.49]	1.24 [1.03-1.49]	1.09 [0.96-1.22]	873	732	200	342	707
B00	2.52 [2.38-2.67]	2.06 [1.91-2.24]	1.73 [1.58-1.91]	3.13 [2.90-3.38]	2.31 [2.17-2.46]	2.34 [2.21-2.49]	1.80 [1.66-1.96]	1.54 [1.40-1.70]	2.97 [2.74-3.22]	2.18 [2.05-2.33]	3,846	1,786	1,174	2,331	3,252
B01	1.61 [1.52-1.72]	1.39 [1.14-1.68]	1.43 [1.10-1.87]	1.63 [1.53-1.74]	1.53 [1.44-1.64]	1.56 [1.46-1.66]	1.27 [1.04-1.55]	1.29 [0.99-1.70]	1.58 [1.48-1.69]	1.49 [1.40-1.60]	2,710	260	139	2,482	2,395
B02	1.42 [1.34-1.50]	1.41 [1.33-1.49]	1.40 [1.32-1.49]	1.41 [1.20-1.67]	1.32 [1.24-1.40]	1.34 [1.26-1.42]	1.29 [1.21-1.37]	1.29 [1.21-1.37]	1.35 [1.13-1.60]	1.26 [1.19-1.33]	3,475	3,158	2,946	394	3,139
B07	1.41 [1.30-1.53]	1.40 [1.28-1.53]	1.39 [1.26-1.54]	1.45 [1.22-1.71]	1.32 [1.21-1.45]	1.30 [1.20-1.42]	1.25 [1.14-1.37]	1.28 [1.15-1.42]	1.32 [1.11-1.58]	1.25 [1.14-1.37]	1,541	1,240	948	379	1,278
B08	1.60 [1.44-1.77]	1.95 [1.48-2.56]		1.53 [1.37-1.71]	1.54 [1.38-1.72]	1.52 [1.37-1.69]	1.70 [1.27-2.27]		1.48 [1.32-1.66]	1.49 [1.33-1.67]	951	143	NA	826	862
B09	1.19 [1.01-1.41]			1.16 [0.97-1.38]	1.17 [0.98-1.40]	1.18 [0.99-1.40]			1.14 [0.95-1.36]	1.16 [0.97-1.39]	351	NA	NA	326	315
B15	1.10 [0.86-1.39]	1.24 [0.95-1.60]	1.35 [1.00-1.82]		1.06 [0.82-1.39]	1.06 [0.83-1.36]	1.17 [0.89-1.53]	1.26 [0.93-1.72]		1.03 [0.79-1.35]	171	142	108	NA	139
B16	1.34 [1.12-1.59]	1.39 [1.17-1.66]	1.54 [1.25-1.90]		1.17 [0.97-1.41]	1.31 [1.09-1.56]	1.37 [1.14-1.64]	1.45 [1.17-1.81]		1.17 [0.97-1.42]	314	309	217	NA	256
B17	1.39 [1.21-1.60]	1.41 [1.22-1.62]	1.52 [1.29-1.80]		1.24 [1.07-1.44]	1.33 [1.15-1.53]	1.26 [1.08-1.46]	1.35 [1.13-1.60]		1.20 [1.03-1.40]	499	463	353	NA	421
B18	1.19 [1.11-1.28]	1.23 [1.14-1.32]	1.30 [1.20-1.41]		1.07 [0.99-1.16]	1.16 [1.08-1.25]	1.13 [1.05-1.22]	1.18 [1.08-1.29]		1.09 [1.01-1.18]	1,820	1,807	1,396	NA	1,544
B19	1.16 [0.87-1.54]				1.18 [0.87-1.60]	1.07 [0.80-1.43]				1.14 [0.84-1.57]	123	NA	NA	NA	102
B25	1.41 [1.21-1.64]	1.40 [1.19-1.65]	1.45 [1.19-1.75]		1.23 [1.05-1.44]	1.35 [1.15-1.58]	1.27 [1.07-1.50]	1.32 [1.09-1.61]		1.18 [1.01-1.40]	413	357	264	NA	367
B26	1.20 [0.92-1.56]				1.13 [0.84-1.52]	1.21 [0.92-1.58]				1.08 [0.79-1.46]	144	NA	NA	NA	109
B27	1.15 [1.06-1.24]	1.11 [1.00-1.24]		1.16 [1.06-1.26]	1.08 [0.98-1.19]	1.10 [1.01-1.19]	1.02 [0.91-1.14]		1.10 [1.01-1.21]	1.04 [0.94-1.15]	1,551	787	NA	1,319	1,099
B30	1.26 [0.98-1.62]				1.16 [0.90-1.51]	1.16 [0.89-1.50]				1.09 [0.83-1.43]	156	NA	NA	NA	144
B33	1.24 [0.93-1.66]					1.22 [0.91-1.64]					109	NA	NA	NA	NA
B34	1.54 [1.52-1.57]	1.33 [1.28-1.38]	1.33 [1.27-1.40]	1.57 [1.55-1.60]	1.49 [1.46-1.51]	1.48 [1.45-1.50]	1.15 [1.11-1.20]	1.16 [1.10-1.22]	1.53 [1.50-1.55]	1.44 [1.41-1.46]	40,130	6,707	3,879	34,560	35,691
B35	1.72 [1.58-1.86]	1.77 [1.62-1.93]	1.71 [1.57-1.87]	1.47 [1.19-1.82]	1.65 [1.51-1.79]	1.58 [1.45-1.71]	1.56 [1.43-1.71]	1.52 [1.39-1.67]	1.38 [1.10-1.72]	1.55 [1.42-1.69]	1,735	1,527	1,376	248	1,546
B36	1.87 [1.63-2.14]	1.86 [1.62-2.14]	1.77 [1.53-2.05]		1.68 [1.46-1.92]	1.72 [1.50-1.97]	1.66 [1.44-1.92]	1.58 [1.36-1.84]		1.60 [1.39-1.84]	651	592	538	NA	596
B37	1.36 [1.33-1.39]	1.37 [1.34-1.40]	1.38 [1.34-1.41]	1.11 [1.04-1.20]	1.26 [1.23-1.29]	1.25 [1.22-1.28]	1.20 [1.17-1.23]	1.21 [1.18-1.24]	1.06 [0.99-1.14]	1.19 [1.16-1.22]	18,911	17,959	15,080	1,965	17,282
B44	1.69 [1.51-1.88]	1.84 [1.64-2.06]	1.90 [1.68-2.14]		1.51 [1.35-1.70]	1.40 [1.24-1.57]	1.47 [1.30-1.66]	1.50 [1.32-1.70]		1.27 [1.13-1.43]	857	811	745	NA	784
B49	1.63 [1.41-1.88]	1.53 [1.30-1.78]	1.51 [1.29-1.78]		1.51 [1.30-1.76]	1.54 [1.33-1.79]	1.40 [1.19-1.65]	1.39 [1.18-1.64]		1.45 [1.25-1.69]	500	418	376	NA	461
B50	0.95 [0.76-1.19]	0.98 [0.76-1.26]			0.83 [0.63-1.10]	0.97 [0.77-1.22]	1.05 [0.81-1.37]			0.86 [0.65-1.14]	194	143	NA	NA	116
B59	1.53 [1.24-1.88]	1.55 [1.26-1.91]	1.39 [1.12-1.73]		1.36 [1.09-1.68]	1.44 [1.16-1.78]	1.44 [1.16-1.78]	1.30 [1.04-1.62]		1.30 [1.04-1.62]	236	228	205	NA	210
B80	1.21 [1.06-1.37]	1.24 [1.03-1.50]	1.21 [0.95-1.54]	1.18 [1.00-1.38]	1.16 [1.01-1.33]	1.14 [1.00-1.30]	1.13 [0.93-1.37]	1.14 [0.88-1.47]	1.12 [0.95-1.32]	1.11 [0.96-1.27]	614	261	148	405	532
B85	1.12 [0.91-1.37]	1.19 [0.89-1.59]		0.95 [0.71-1.26]	1.04 [0.83-1.30]	1.11 [0.90-1.36]	1.09 [0.81-1.47]		0.95 [0.71-1.26]	1.03 [0.82-1.29]	228	117	NA	115	189
B86	2.53 [2.19-2.93]	2.59 [2.22-3.04]	2.65 [2.26-3.12]		2.19 [1.87-2.55]	2.49 [2.15-2.89]	2.43 [2.07-2.86]	2.49 [2.11-2.94]		2.20 [1.88-2.58]	607	519	496	NA	516
B90	1.60 [1.25-2.03]	1.43 [1.12-1.82]	1.39 [1.08-1.79]		1.41 [1.11-1.81]	1.46 [1.14-1.87]	1.29 [1.00-1.65]	1.25 [0.97-1.62]		1.31 [1.02-1.68]	184	182	167	NA	171
B91	1.30 [1.07-1.58]	1.29 [1.06-1.57]	1.33 [1.09-1.62]		1.14 [0.92-1.41]	1.30 [1.06-1.58]	1.27 [1.04-1.56]	1.29 [1.06-1.59]		1.16 [0.94-1.44]	257	256	250	NA	210
B94	1.32 [1.14-1.52]	1.31 [1.11-1.55]	1.33 [1.11-1.60]	1.23 [0.93-1.61]	1.24 [1.07-1.44]	1.19 [1.02-1.39]	1.14 [0.96-1.36]	1.17 [0.97-1.41]	1.25 [0.94-1.65]	1.18 [1.01-1.37]	468	348	296	137	438
B95	1.51 [1.48-1.54]	1.50 [1.47-1.54]	1.50 [1.46-1.53]	1.50 [1.43-1.57]	1.41 [1.38-1.44]	1.44 [1.42-1.47]	1.39 [1.36-1.42]	1.38 [1.35-1.41]	1.45 [1.39-1.52]	1.38 [1.35-1.41]	29,775	25,667	21,484	5,462	26,256
B96	1.23 [1.21-1.24]	1.24 [1.22-1.26]	1.24 [1.22-1.26]	1.07 [1.02-1.12]	1.17 [1.15-1.18]	1.17 [1.16-1.19]	1.14 [1.13-1.16]	1.15 [1.13-1.17]	1.04 [1.00-1.09]	1.14 [1.12-1.16]	51,288	47,700	43,442	5,099	46,091
B97	1.32 [1.29-1.35]	1.29 [1.25-1.34]	1.30 [1.25-1.35]	1.34 [1.29-1.39]	1.25 [1.22-1.29]	1.23 [1.20-1.27]	1.15 [1.11-1.19]	1.14 [1.10-1.19]	1.28 [1.23-1.34]	1.19 [1.16-1.23]	15,607	9,623	7,862	6,512	14,153
B98	1.20 [1.14-1.25]	1.22 [1.16-1.27]	1.21 [1.15-1.27]	1.01 [0.82-1.25]	1.13 [1.08-1.18]	1.14 [1.09-1.19]	1.13 [1.08-1.18]	1.12 [1.07-1.18]	1.00 [0.80-1.24]	1.10 [1.04-1.15]	4,905	4,763	4,267	248	4,320
B99	1.24 [1.14-1.36]	1.26 [1.16-1.38]	1.21 [1.10-1.33]	1.25 [0.95-1.63]	1.20 [1.10-1.32]	1.20 [1.10-1.31]	1.17 [1.07-1.28]	1.12 [1.02-1.24]	1.18 [0.89-1.56]	1.19 [1.09-1.31]	1,443	1,335	1,229	144	1,356
Neoplasms (C00-D49)															
C00	1.04 [0.81-1.35]	1.08 [0.84-1.40]	1.10 [0.85-1.42]		1.04 [0.80-1.36]	1.03 [0.80-1.33]	1.05 [0.81-1.36]	1.05 [0.81-1.37]		1.03 [0.79-1.35]	146	146	144	NA	128
C01	1.08 [0.91-1.28]	1.09 [0.92-1.30]	1.07 [0.90-1.27]		1.12 [0.93-1.35]	1.08 [0.90-1.28]	1.05 [0.88-1.26]	1.04 [0.87-1.24]		1.17 [0.97-1.41]	306	306	303	NA	266
C02	1.28 [1.13-1.46]	1.31 [1.15-1.50]	1.32 [1.16-1.51]		1.30 [1.13-1.50]	1.27 [1.11-1.45]	1.24 [1.08-1.42]	1.27 [1.11-1.46]		1.31 [1.13-1.51]	548	548	530	NA	473
C03	1.47 [1.12-1.92]	1.38 [1.06-1.81]	1.44 [1.09-1.91]		1.32 [0.99-1.76]	1.46 [1.11-1.91]	1.38 [1.05-1.82]	1.42 [1.07-1.88]		1.32 [0.98-1.77]	139	139	132	NA	118
C04	1.13 [0.88-1.45]	1.15 [0.90-1.48]	1.12 [0.87-1.44]		1.10 [0.84-1.43]	1.14 [0.89-1.47]	1.08 [0.84-1.39]	1.06 [0.82-1.37]		1.13 [0.86-1.48]	151	150	145	NA	128
C05	1.28 [1.01-1.62]	1.32 [1.05-1.67]	1.32 [1.04-1.68]		1.14 [0.89-1.47]	1.29 [1.02-1.64]	1.29 [1.01-1.63]	1.27 [0.99-1.62]		1.20 [0.93-1.56]	176	177	168	NA	147
C06	1.25 [1.03-1.52]	1.24 [1.02-1.52]	1.21 [0.99-1.48]		1.18 [0.96-1.46]	1.24 [1.01-1.51]	1.18 [0.96-1.44]	1.13 [0.92-1.39]		1.19 [0.96-1.47]	244	246	233	NA	214
C07	1.03 [0.84-1.27]	1.05 [0.85-1.29]	1.01 [0.81-1.25]		1.04 [0.83-1.31]	1.02 [0.83-1.25]	1.00 [0.80-1.24]	0.97 [0.78-1.21]		1.03 [0.82-1.30]	219	212	192	NA	184
C09	1.07 [0.92-1.25]	1.12 [0.97-1.31]	1.15 [0.99-1.33]		1.07 [0.90-1.26]	1.04 [0.89-1.22]	1.06 [0.91-1.24]	1.09 [0.93-1.27]		1.08 [0.91-1.28]	391	391	391	NA	316
C10	1.11 [0.90-1.37]	1.18 [0.95-1.45]	1.16 [0.94-1.44]		1.08 [0.86-1.36]	1.09 [0.88-1.35]	1.10 [0.88-1.36]	1.09 [0.87-1.35]		1.09 [0.87-1.38]	204	204	203	NA	176
C11	1.19 [0.90-1.58]	1.20 [0.90-1.60]	1.36 [1.01-1.83]			1.19 [0.89-1.58]	1.17 [0.87-1.56]	1.31 [0.97-1.77]			115	111	108	NA	NA
C12	1.24 [0.94-1.63]	1.29 [0.98-1.70]	1.35 [1.02-1.78]		1.26 [0.93-1.70]	1.25 [0.94-1.65]	1.24 [0.93-1.65]	1.31 [0.99-1.74]		1.33 [0.98-1.81]	122	122	122	NA	103
C13	1.16 [0.89-1.52]	1.25 [0.95-1.63]	1.22 [0.93-1.60]		1.19 [0.89-1.58]	1.20 [0.91-1.57]	1.24 [0.94-1.63]	1.20 [0.91-1.58]		1.30 [0.97-1.75]	132	132	132	NA	116
C14	1.10 [0.90-1.34]	1.12 [0.91-1.36]	1.06 [0.87-1.30]		0.96 [0.77-1.18]	1.08 [0.88-1.33]	1.06 [0.87-1.31]	1.00 [0.81-1.23]		0.97 [0.78-1.20]	228	228	227	NA	196
C15	1.18 [1.12-1.25]	1.15 [1.09-1.22]	1.17 [1.10-1.23]		1.13 [1.07-1.20]	1.17 [1.11-1.24]	1.12 [1.06-1.18]	1.13 [1.07-1.19]		1.14 [1.07-1.21]	3,259	3,259	3,242	NA	2,722
C16	1.08 [1.01-1.15]	1.07 [1.00-1.13]	1.09 [1.02-1.16]		1.06 [0.99-1.14]	1.07 [1.01-1.14]	1.04 [0.98-1.11]	1.06 [0.99-1.13]		1.06 [0.99-1.14]	2,508	2,508	2,476	NA	2,102
C17	1.11 [0.98-1.27]	1.15 [1.01-1.32]	1.13 [0.99-1.29]		1.08 [0.94-1.24]	1.10 [0.96-1.26]	1.09 [0.95-1.25]	1.10 [0.96-1.26]		1.07 [0.93-1.23]	528	527	520	NA	468
C18	1.01 [0.98-1.05]	1.01 [0.98-1.05]	1.01 [0.98-1.05]		1.00 [0.96-1.04]	1.00 [0.97-1.04]	1.00 [0.97-1.03]	1.00 [0.96-1.03]		1.00 [0.96-1.03]	8,255	8,246	8,116	NA	6,819
C19	0.95 [0.88-1.02]	0.96 [0.89-1.04]	0.96 [0.89-1.04]		0.94 [0.87-1.02]	0.95 [0.88-1.02]	0.96 [0.89-1.04]	0.96 [0.89-1.04]		0.95 [0.88-1.04]	1,621	1,619	1,583	NA	1,334
C20	0.97 [0.92-1.02]	0.97 [0.92-1.02]	0.97 [0.92-1.02]		0.95 [0.90-1.01]	0.98 [0.93-1.03]	0.98 [0.93-1.03]	0.97 [0.92-1.03]		0.97 [0.91-1.03]	3,365	3,365	3,318	NA	2,627
C21	1.08 [0.94-1.24]	1.08 [0.94-1.25]	1.09 [0.94-1.25]		1.04 [0.89-1.21]	1.07 [0.93-1.23]	1.05 [0.91-1.21]	1.05 [0.91-1.21]		1.04 [0.89-1.21]	458	458	451	NA	390
C22	1.25 [1.16-1.34]	1.25 [1.16-1.34]	1.23 [1.14-1.32]		1.18 [1.09-1.27]	1.21 [1.13-1.31]	1.18 [1.10-1.27]	1.17 [1.08-1.26]		1.17 [1.08-1.26]	1,925	1,919	1,894	NA	1,708
C23	1.07 [0.88-1.31]	1.06 [0.87-1.29]	1.06 [0.87-1.29]		1.03 [0.83-1.28]	1.07 [0.88-1.30]	1.04 [0.85-1.26]	1.05 [0.86-1.28]		1.04 [0.84-1.29]	242	242	242	NA	204
C24	1.09 [0.94-1.25]	1.07 [0.93-1.24]	1.07 [0.92-1.23]		1.04 [0.89-1.22]	1.08 [0.94-1.25]	1.04 [0.90-1.20]	1.02 [0.88-1.18]		1.03 [0.88-1.20]	464	465	458	NA	389
C25	1.16 [1.10-1.23]	1.14 [1.08-1.21]	1.15 [1.09-1.22]		1.13 [1.07-1.20]	1.15 [1.09-1.22]	1.10 [1.04-1.17]	1.11 [1.05-1.18]		1.13 [1.07-1.21]	3,016	3,008	2,998	NA	2,573
C26	1.13 [1.00-1.28]	1.10 [0.97-1.24]	1.12 [0.99-1.26]		1.06 [0.93-1.20]	1.10 [0.98-1.25]	1.05 [0.93-1.19]	1.08 [0.95-1.22]		1.05 [0.92-1.19]	678	677	659	NA	626
C30	1.07 [0.80-1.43]	1.12 [0.84-1.51]	1.16 [0.86-1.56]			1.06 [0.79-1.42]	1.09 [0.81-1.46]	1.12 [0.83-1.52]			109	107	107	NA	NA
C32	1.14 [1.02-1.28]	1.17 [1.04-1.32]	1.16 [1.03-1.30]		1.08 [0.95-1.22]	1.16 [1.03-1.30]	1.14 [1.01-1.28]	1.12 [0.99-1.26]		1.11 [0.98-1.26]	690	689	680	NA	586
C34	1.15 [1.12-1.18]	1.15 [1.12-1.18]	1.15 [1.12-1.18]		1.11 [1.08-1.14]	1.14 [1.11-1.17]	1.09 [1.06-1.12]	1.09 [1.06-1.12]		1.12 [1.09-1.15]	13,074	13,057	13,024	NA	11,181
C38	1.07 [0.85-1.35]	0.97 [0.76-1.22]	1.00 [0.79-1.27]		1.02 [0.80-1.32]	1.08 [0.85-1.37]	0.95 [0.74-1.20]	0.99 [0.77-1.26]		1.04 [0.80-1.34]	171	165	157	NA	144
C40	1.05 [0.80-1.37]				1.02 [0.75-1.37]	1.02 [0.78-1.34]				1.04 [0.77-1.41]	134	NA	NA	NA	104
C41	1.25 [1.07-1.46]	1.23 [1.04-1.45]	1.21 [1.02-1.44]		1.18 [1.00-1.40]	1.24 [1.06-1.46]	1.19 [1.00-1.40]	1.17 [0.98-1.39]		1.19 [1.00-1.41]	397	353	316	NA	332
C43	0.95 [0.90-1.01]	0.97 [0.92-1.02]	0.96 [0.90-1.01]		0.97 [0.91-1.02]	0.94 [0.89-1.00]	0.95 [0.90-1.01]	0.94 [0.89-0.99]		0.96 [0.90-1.02]	3,170	3,159	2,886	NA	2,682
C44	1.10 [1.08-1.12]	1.11 [1.08-1.13]	1.10 [1.08-1.13]		1.06 [1.03-1.08]	1.07 [1.05-1.09]	1.07 [1.04-1.09]	1.06 [1.04-1.08]		1.03 [1.01-1.06]	25,094	25,077	24,709	NA	21,264
C45	0.98 [0.88-1.10]	1.00 [0.89-1.12]	1.01 [0.90-1.13]		1.02 [0.91-1.16]	0.99 [0.89-1.11]	1.01 [0.90-1.13]	1.02 [0.91-1.14]		1.04 [0.92-1.18]	725	723	725	NA	614
C48	1.05 [0.90-1.21]	1.07 [0.93-1.24]	1.06 [0.91-1.23]		1.04 [0.89-1.22]	1.03 [0.89-1.20]	1.07 [0.92-1.24]	1.04 [0.90-1.21]		1.03 [0.88-1.21]	420	413	409	NA	351
C49	1.08 [0.97-1.20]	1.13 [1.01-1.26]	1.13 [1.00-1.27]		1.10 [0.98-1.24]	1.06 [0.95-1.18]	1.10 [0.99-1.24]	1.09 [0.96-1.23]		1.09 [0.97-1.23]	820	758	682	NA	697
C50	1.04 [1.02-1.07]	1.04 [1.01-1.06]	1.03 [1.01-1.06]		1.02 [0.99-1.05]	1.04 [1.01-1.07]	1.03 [1.00-1.05]	1.02 [0.99-1.05]		1.03 [1.00-1.06]	14,359	14,360	13,755	NA	11,473
C51	1.20 [1.04-1.39]	1.24 [1.07-1.44]	1.27 [1.09-1.47]		1.21 [1.03-1.43]	1.18 [1.02-1.38]	1.22 [1.05-1.42]	1.22 [1.05-1.42]		1.20 [1.02-1.42]	434	433	424	NA	361
C52	0.98 [0.74-1.29]	0.90 [0.68-1.19]	0.92 [0.70-1.22]		0.93 [0.69-1.24]	1.00 [0.76-1.33]	0.90 [0.68-1.19]	0.93 [0.70-1.23]		0.93 [0.69-1.25]	117	116	114	NA	103
C53	0.96 [0.86-1.07]	0.96 [0.86-1.07]	0.96 [0.84-1.09]		1.02 [0.90-1.15]	0.97 [0.87-1.09]	0.97 [0.87-1.08]	0.98 [0.86-1.12]		1.05 [0.93-1.19]	755	752	523	NA	615
C54	1.04 [0.98-1.11]	1.04 [0.98-1.11]	1.03 [0.97-1.09]		1.03 [0.96-1.10]	1.06 [0.99-1.12]	1.06 [1.00-1.13]	1.05 [0.98-1.11]		1.04 [0.97-1.12]	2,358	2,355	2,327	NA	1,822
C55	1.06 [0.90-1.25]	1.02 [0.86-1.20]	1.01 [0.85-1.19]		0.96 [0.80-1.15]	1.05 [0.88-1.24]	1.01 [0.85-1.19]	1.01 [0.85-1.20]		0.95 [0.79-1.15]	325	323	315	NA	263
C56	1.03 [0.97-1.10]	1.01 [0.95-1.08]	1.02 [0.96-1.09]		1.00 [0.93-1.08]	1.04 [0.97-1.11]	1.02 [0.95-1.09]	1.02 [0.96-1.10]		1.01 [0.94-1.09]	2,094	2,078	1,979	NA	1,630
C57	1.18 [0.97-1.42]	1.19 [0.99-1.44]	1.19 [0.99-1.44]		1.15 [0.94-1.42]	1.20 [0.99-1.46]	1.22 [1.00-1.47]	1.21 [1.00-1.47]		1.17 [0.95-1.44]	262	262	260	NA	215
C60	1.19 [0.95-1.48]	1.30 [1.04-1.62]	1.21 [0.97-1.51]		1.17 [0.92-1.49]	1.20 [0.96-1.50]	1.29 [1.03-1.62]	1.24 [0.99-1.56]		1.21 [0.94-1.55]	195	195	190	NA	162
C61	1.06 [1.03-1.09]	1.06 [1.03-1.09]	1.05 [1.03-1.08]		1.01 [0.98-1.04]	1.04 [1.01-1.06]	1.03 [1.00-1.06]	1.02 [1.00-1.05]		1.00 [0.97-1.03]	13,721	13,725	13,723	NA	11,486
C62	1.04 [0.89-1.22]	1.02 [0.87-1.20]	0.99 [0.78-1.26]		1.02 [0.84-1.24]	1.02 [0.87-1.20]	0.99 [0.84-1.17]	0.95 [0.74-1.22]		1.03 [0.84-1.25]	362	345	146	NA	242
C64	1.11 [1.05-1.18]	1.10 [1.04-1.17]	1.11 [1.05-1.18]		1.06 [0.99-1.12]	1.09 [1.03-1.15]	1.05 [0.99-1.11]	1.06 [1.00-1.12]		1.05 [0.99-1.12]	2,914	2,867	2,824	NA	2,501
C65	1.22 [1.01-1.47]	1.22 [1.01-1.47]	1.22 [1.01-1.48]		1.11 [0.91-1.36]	1.24 [1.02-1.50]	1.18 [0.98-1.43]	1.21 [1.00-1.47]		1.15 [0.94-1.41]	282	280	277	NA	241
C66	1.10 [0.93-1.29]	1.09 [0.92-1.28]	1.08 [0.92-1.28]		0.99 [0.83-1.17]	1.09 [0.93-1.29]	1.05 [0.89-1.24]	1.04 [0.88-1.23]		1.00 [0.84-1.18]	357	357	357	NA	316
C67	1.08 [1.04-1.12]	1.09 [1.05-1.13]	1.08 [1.04-1.13]		1.03 [0.99-1.07]	1.07 [1.03-1.12]	1.06 [1.02-1.10]	1.06 [1.02-1.10]		1.03 [0.99-1.08]	6,425	6,415	6,366	NA	5,376
C68	1.03 [0.79-1.35]	1.05 [0.81-1.37]	1.11 [0.85-1.44]		0.94 [0.71-1.23]	1.02 [0.78-1.33]	1.04 [0.80-1.36]	1.10 [0.84-1.43]		0.95 [0.72-1.26]	134	135	132	NA	118
C69	0.98 [0.81-1.18]	0.97 [0.80-1.17]	1.03 [0.84-1.26]		0.96 [0.79-1.18]	0.96 [0.79-1.16]	0.95 [0.78-1.16]	1.00 [0.81-1.23]		0.97 [0.79-1.19]	261	238	222	NA	221
C71	1.12 [1.04-1.21]	1.15 [1.06-1.24]	1.13 [1.04-1.23]	0.99 [0.81-1.21]	1.09 [1.00-1.19]	1.11 [1.03-1.19]	1.12 [1.03-1.21]	1.10 [1.01-1.20]	1.02 [0.83-1.24]	1.09 [1.00-1.18]	1,672	1,469	1,338	243	1,326
C72	1.20 [0.90-1.59]					1.11 [0.82-1.49]					120	NA	NA	NA	NA
C73	1.06 [0.96-1.18]	1.06 [0.95-1.18]	1.06 [0.94-1.20]		1.02 [0.91-1.15]	1.04 [0.93-1.16]	1.02 [0.92-1.14]	1.03 [0.92-1.17]		1.01 [0.90-1.14]	819	798	618	NA	675
C74	1.20 [0.93-1.55]	1.13 [0.83-1.52]			1.09 [0.83-1.42]	1.19 [0.92-1.54]	1.06 [0.77-1.44]			1.08 [0.82-1.42]	145	107	NA	NA	129
C75	1.07 [0.83-1.39]	1.00 [0.76-1.32]	1.07 [0.81-1.41]		0.99 [0.75-1.30]	1.04 [0.80-1.36]	0.96 [0.73-1.27]	1.00 [0.75-1.33]		1.00 [0.76-1.32]	137	121	112	NA	121
C76	1.10 [0.96-1.27]	1.11 [0.95-1.28]	1.07 [0.92-1.25]		1.09 [0.93-1.26]	1.11 [0.96-1.28]	1.09 [0.94-1.27]	1.06 [0.91-1.24]		1.09 [0.94-1.28]	472	431	415	NA	412
C77	1.06 [1.03-1.09]	1.05 [1.03-1.08]	1.05 [1.03-1.08]	1.14 [0.86-1.51]	1.04 [1.01-1.07]	1.06 [1.04-1.09]	1.04 [1.01-1.07]	1.04 [1.01-1.07]	1.14 [0.86-1.53]	1.05 [1.02-1.08]	14,180	14,120	13,563	140	11,924
C78	1.06 [1.04-1.09]	1.05 [1.03-1.08]	1.06 [1.04-1.08]	1.01 [0.78-1.32]	1.05 [1.02-1.07]	1.07 [1.04-1.09]	1.04 [1.01-1.06]	1.04 [1.02-1.07]	1.03 [0.78-1.35]	1.07 [1.04-1.09]	20,975	20,875	20,532	147	18,290
C79	1.08 [1.05-1.10]	1.07 [1.05-1.10]	1.08 [1.05-1.10]	0.98 [0.77-1.25]	1.05 [1.03-1.08]	1.08 [1.06-1.11]	1.05 [1.03-1.08]	1.06 [1.03-1.08]	0.99 [0.77-1.27]	1.08 [1.05-1.11]	17,880	17,755	17,443	168	15,788
C80	1.10 [1.06-1.15]	1.09 [1.05-1.13]	1.10 [1.06-1.14]		1.09 [1.04-1.13]	1.10 [1.06-1.14]	1.06 [1.02-1.10]	1.07 [1.03-1.12]		1.09 [1.05-1.14]	6,357	6,332	6,242	NA	5,385
C81	1.71 [1.54-1.90]	1.72 [1.54-1.91]	1.62 [1.42-1.85]	1.41 [1.11-1.79]	1.46 [1.30-1.65]	1.64 [1.48-1.83]	1.65 [1.48-1.85]	1.52 [1.33-1.74]	1.33 [1.04-1.71]	1.43 [1.26-1.61]	969	870	588	185	701
C82	1.16 [1.04-1.29]	1.10 [0.99-1.23]	1.10 [0.98-1.23]		1.04 [0.93-1.17]	1.10 [0.99-1.23]	1.04 [0.93-1.16]	1.05 [0.94-1.17]		1.02 [0.90-1.15]	790	782	744	NA	658
C83	1.24 [1.17-1.32]	1.26 [1.18-1.34]	1.23 [1.15-1.31]		1.19 [1.11-1.27]	1.21 [1.13-1.29]	1.22 [1.14-1.30]	1.19 [1.11-1.27]		1.17 [1.09-1.25]	2,429	2,371	2,250	NA	2,081
C84	2.44 [2.11-2.83]	2.71 [2.33-3.15]	2.56 [2.19-2.99]		2.15 [1.83-2.53]	2.41 [2.08-2.80]	2.64 [2.26-3.08]	2.48 [2.11-2.90]		2.17 [1.84-2.57]	545	530	489	NA	437
C85	1.33 [1.25-1.40]	1.31 [1.24-1.39]	1.30 [1.23-1.38]		1.23 [1.16-1.30]	1.28 [1.21-1.35]	1.26 [1.19-1.33]	1.23 [1.16-1.31]		1.21 [1.13-1.28]	3,161	3,108	2,917	NA	2,644
C88	1.28 [1.10-1.50]	1.23 [1.06-1.44]	1.28 [1.10-1.51]		1.14 [0.96-1.34]	1.26 [1.08-1.48]	1.21 [1.03-1.42]	1.24 [1.06-1.46]		1.12 [0.95-1.33]	410	407	395	NA	365
C90	1.13 [1.06-1.22]	1.15 [1.07-1.23]	1.14 [1.06-1.22]		1.10 [1.02-1.19]	1.11 [1.03-1.19]	1.13 [1.05-1.21]	1.10 [1.03-1.18]		1.08 [1.00-1.17]	1,995	1,988	1,969	NA	1,686
C91	1.07 [1.00-1.15]	1.12 [1.04-1.21]	1.09 [1.02-1.18]	0.97 [0.81-1.15]	1.04 [0.97-1.12]	1.05 [0.99-1.13]	1.08 [1.01-1.17]	1.05 [0.98-1.13]	0.97 [0.81-1.15]	1.03 [0.95-1.11]	2,123	1,828	1,763	321	1,756
C92	1.28 [1.18-1.38]	1.29 [1.20-1.39]	1.28 [1.19-1.39]	1.09 [0.81-1.49]	1.24 [1.15-1.35]	1.25 [1.16-1.35]	1.26 [1.16-1.36]	1.25 [1.15-1.35]	1.10 [0.80-1.51]	1.24 [1.14-1.35]	1,752	1,680	1,574	107	1,488
C93	1.17 [0.94-1.46]	1.18 [0.95-1.47]	1.10 [0.88-1.37]		1.11 [0.89-1.40]	1.19 [0.95-1.48]	1.14 [0.91-1.42]	1.07 [0.85-1.34]		1.15 [0.91-1.44]	216	213	211	NA	199
C94	1.25 [0.92-1.70]	1.31 [0.96-1.80]	1.23 [0.90-1.68]			1.24 [0.91-1.70]	1.27 [0.92-1.74]	1.17 [0.85-1.61]			105	104	103	NA	NA
C95	1.22 [1.04-1.43]	1.34 [1.13-1.60]	1.32 [1.11-1.57]		1.17 [0.99-1.38]	1.19 [1.02-1.40]	1.27 [1.06-1.52]	1.24 [1.03-1.48]		1.16 [0.98-1.37]	395	351	336	NA	357
C97	1.06 [0.98-1.15]	1.08 [1.00-1.17]	1.05 [0.97-1.14]		1.02 [0.94-1.10]	1.04 [0.96-1.12]	1.04 [0.96-1.13]	1.02 [0.94-1.10]		1.01 [0.93-1.09]	1,611	1,610	1,596	NA	1,510
D00	1.09 [0.87-1.36]	1.05 [0.84-1.32]	1.07 [0.85-1.34]		1.02 [0.81-1.29]	1.05 [0.84-1.32]	0.99 [0.79-1.24]	0.98 [0.77-1.23]		1.01 [0.80-1.28]	181	181	179	NA	168
D01	1.10 [0.88-1.38]	1.11 [0.89-1.40]	1.13 [0.89-1.42]		1.00 [0.79-1.27]	1.04 [0.82-1.30]	1.02 [0.81-1.29]	1.04 [0.82-1.31]		0.96 [0.76-1.22]	180	180	170	NA	162
D02	1.27 [1.02-1.57]	1.20 [0.97-1.49]	1.22 [0.98-1.52]		1.14 [0.91-1.43]	1.23 [0.99-1.54]	1.13 [0.91-1.41]	1.14 [0.92-1.43]		1.13 [0.90-1.43]	209	208	203	NA	184
D03	1.06 [0.97-1.15]	1.08 [0.99-1.18]	1.08 [0.99-1.18]		1.02 [0.93-1.12]	1.02 [0.94-1.11]	1.04 [0.95-1.13]	1.04 [0.95-1.14]		1.00 [0.91-1.09]	1,295	1,292	1,226	NA	1,095
D04	1.25 [1.18-1.33]	1.26 [1.19-1.34]	1.26 [1.19-1.34]		1.20 [1.13-1.28]	1.19 [1.12-1.27]	1.18 [1.11-1.25]	1.17 [1.10-1.24]		1.16 [1.09-1.23]	3,098	3,093	3,069	NA	2,827
D05	1.01 [0.95-1.07]	1.01 [0.95-1.07]	1.00 [0.94-1.06]		0.96 [0.90-1.03]	0.99 [0.93-1.06]	1.00 [0.93-1.06]	0.98 [0.92-1.05]		0.96 [0.89-1.03]	2,263	2,265	2,184	NA	1,812
D06	1.05 [0.98-1.12]	1.06 [0.99-1.13]	1.03 [0.90-1.18]	0.92 [0.77-1.10]	0.99 [0.92-1.07]	1.03 [0.96-1.11]	1.00 [0.93-1.07]	0.99 [0.86-1.14]	0.92 [0.77-1.11]	1.00 [0.93-1.08]	2,060	2,068	455	308	1,642
D07	1.20 [1.08-1.33]	1.28 [1.15-1.42]	1.22 [1.09-1.37]		1.13 [1.01-1.27]	1.14 [1.02-1.28]	1.20 [1.07-1.34]	1.16 [1.03-1.30]		1.10 [0.98-1.24]	799	797	738	NA	700
D09	1.12 [1.00-1.26]	1.13 [1.00-1.27]	1.13 [1.01-1.27]		1.05 [0.93-1.19]	1.11 [0.98-1.25]	1.08 [0.96-1.21]	1.10 [0.97-1.23]		1.05 [0.93-1.19]	694	697	693	NA	635

Supplementary Table 3: Hazard ratios and events from all cohorts

	Hazard ratio (99% confidence interval)										Events (in exposed)				
	crude					adjusted									
Outcome	any age	18+	40+	<18	hosp.	any age	18+	40+	<18	hosp.	any age	18+	40+	<18	hosp.
D10	1.27 [1.15-1.41]	1.33 [1.20-1.49]	1.35 [1.19-1.54]	1.14 [0.90-1.45]	1.25 [1.12-1.40]	1.21 [1.09-1.35]	1.19 [1.06-1.33]	1.21 [1.06-1.38]	1.13 [0.88-1.44]	1.20 [1.07-1.35]	906	794	577	181	728
D11	1.04 [0.94-1.16]	1.04 [0.94-1.16]	1.04 [0.93-1.16]		1.02 [0.91-1.15]	1.04 [0.93-1.16]	1.00 [0.90-1.12]	0.99 [0.88-1.11]		1.02 [0.91-1.15]	812	795	689	NA	664
D12	1.21 [1.19-1.24]	1.21 [1.19-1.24]	1.21 [1.18-1.23]	1.26 [0.96-1.64]	1.13 [1.11-1.16]	1.14 [1.12-1.16]	1.11 [1.09-1.13]	1.11 [1.08-1.13]	1.22 [0.93-1.61]	1.09 [1.07-1.12]	25,696	25,649	24,951	150	22,280
D13	1.22 [1.13-1.31]	1.21 [1.12-1.30]	1.21 [1.12-1.31]		1.10 [1.02-1.19]	1.12 [1.04-1.21]	1.09 [1.01-1.17]	1.09 [1.01-1.18]		1.04 [0.96-1.13]	1,755	1,739	1,613	NA	1,479
D14	1.31 [1.15-1.48]	1.30 [1.14-1.48]	1.32 [1.14-1.53]		1.16 [1.01-1.33]	1.23 [1.08-1.40]	1.16 [1.02-1.33]	1.22 [1.05-1.42]		1.12 [0.97-1.29]	609	560	433	NA	489
D15	1.15 [0.93-1.42]	1.15 [0.92-1.43]	1.16 [0.93-1.45]		1.07 [0.85-1.34]	1.14 [0.92-1.41]	1.11 [0.89-1.38]	1.12 [0.89-1.40]		1.06 [0.84-1.33]	214	200	186	NA	176
D16	1.18 [1.07-1.29]	1.27 [1.13-1.43]	1.20 [1.03-1.40]	1.07 [0.94-1.22]	1.08 [0.98-1.21]	1.14 [1.03-1.25]	1.18 [1.04-1.33]	1.09 [0.93-1.28]	1.06 [0.92-1.21]	1.05 [0.94-1.17]	1,123	647	390	604	878
D17	1.16 [1.12-1.20]	1.16 [1.12-1.20]	1.13 [1.09-1.18]	1.13 [0.98-1.30]	1.07 [1.03-1.11]	1.10 [1.07-1.14]	1.08 [1.05-1.12]	1.06 [1.02-1.10]	1.07 [0.93-1.23]	1.03 [0.99-1.07]	7,797	7,547	6,147	516	6,248
D18	1.17 [1.11-1.23]	1.16 [1.10-1.23]	1.19 [1.12-1.26]	1.14 [1.02-1.28]	1.11 [1.05-1.17]	1.11 [1.05-1.17]	1.07 [1.01-1.13]	1.09 [1.02-1.16]	1.13 [1.01-1.27]	1.07 [1.01-1.13]	3,729	3,083	2,567	790	3,237
D21	1.28 [1.16-1.42]	1.34 [1.21-1.49]	1.25 [1.12-1.40]		1.21 [1.08-1.35]	1.23 [1.11-1.36]	1.24 [1.11-1.38]	1.17 [1.04-1.31]		1.16 [1.04-1.30]	949	880	712	NA	765
D22	1.14 [1.10-1.18]	1.15 [1.11-1.20]	1.11 [1.06-1.17]	1.15 [1.08-1.22]	1.07 [1.03-1.12]	1.09 [1.05-1.13]	1.09 [1.05-1.13]	1.05 [0.99-1.10]	1.11 [1.04-1.18]	1.04 [1.00-1.08]	8,229	6,589	3,685	2,539	6,285
D23	1.23 [1.18-1.28]	1.26 [1.20-1.32]	1.26 [1.20-1.32]	1.14 [1.05-1.24]	1.13 [1.08-1.19]	1.17 [1.13-1.22]	1.17 [1.12-1.23]	1.17 [1.11-1.24]	1.11 [1.02-1.21]	1.09 [1.04-1.15]	5,653	4,444	3,532	1,395	4,449
D24	1.05 [0.99-1.11]	1.04 [0.98-1.11]	1.02 [0.94-1.11]	1.13 [1.00-1.28]	0.99 [0.93-1.06]	1.01 [0.95-1.07]	0.99 [0.93-1.06]	0.98 [0.90-1.07]	1.12 [0.98-1.27]	0.97 [0.90-1.04]	2,632	2,432	1,299	675	1,975
D25	1.07 [1.04-1.10]	1.08 [1.05-1.11]	1.09 [1.06-1.12]	1.14 [0.89-1.44]	1.06 [1.03-1.09]	1.04 [1.01-1.07]	1.05 [1.02-1.08]	1.05 [1.02-1.08]	1.12 [0.87-1.44]	1.03 [1.00-1.06]	12,552	12,549	10,372	190	9,894
D26	1.12 [0.96-1.29]	1.10 [0.95-1.27]	1.08 [0.92-1.27]		1.09 [0.93-1.29]	1.09 [0.94-1.27]	1.08 [0.93-1.25]	1.04 [0.88-1.23]		1.07 [0.90-1.26]	424	420	330	NA	342
D27	1.04 [0.98-1.10]	1.06 [1.00-1.12]	1.04 [0.97-1.11]	1.07 [0.90-1.28]	1.03 [0.96-1.09]	1.02 [0.96-1.08]	1.01 [0.96-1.07]	0.99 [0.93-1.07]	1.04 [0.87-1.25]	1.01 [0.95-1.07]	2,900	2,818	1,877	349	2,332
D28	1.25 [1.06-1.47]	1.26 [1.06-1.49]	1.14 [0.93-1.40]		1.20 [1.00-1.44]	1.19 [1.00-1.41]	1.19 [1.00-1.41]	1.11 [0.90-1.37]		1.17 [0.97-1.40]	347	332	218	NA	287
D29	1.23 [1.02-1.49]	1.21 [0.99-1.49]	1.14 [0.92-1.42]		1.17 [0.95-1.44]	1.19 [0.98-1.45]	1.16 [0.95-1.43]	1.10 [0.89-1.38]		1.14 [0.92-1.40]	263	232	208	NA	225
D30	1.11 [0.96-1.28]	1.08 [0.94-1.25]	1.08 [0.94-1.26]		1.03 [0.88-1.20]	1.08 [0.93-1.25]	1.02 [0.88-1.18]	1.03 [0.89-1.20]		1.01 [0.87-1.17]	450	443	422	NA	400
D31	1.28 [1.12-1.46]	1.30 [1.12-1.50]	1.26 [1.07-1.47]	1.33 [1.01-1.74]	1.28 [1.11-1.48]	1.25 [1.09-1.43]	1.23 [1.06-1.43]	1.20 [1.02-1.41]	1.29 [0.98-1.70]	1.26 [1.09-1.46]	571	459	394	143	495
D32	1.13 [1.05-1.22]	1.09 [1.01-1.18]	1.11 [1.03-1.21]		1.09 [1.01-1.18]	1.09 [1.01-1.18]	1.03 [0.95-1.11]	1.05 [0.97-1.14]		1.07 [0.99-1.17]	1,701	1,692	1,611	NA	1,525
D33	1.13 [1.00-1.27]	1.08 [0.96-1.22]	1.11 [0.97-1.27]		1.03 [0.91-1.17]	1.05 [0.93-1.18]	0.98 [0.87-1.11]	1.02 [0.89-1.17]		0.99 [0.87-1.12]	685	612	539	NA	594
D34	1.07 [0.93-1.24]	1.04 [0.90-1.21]	1.04 [0.89-1.23]		1.06 [0.91-1.24]	1.03 [0.88-1.19]	0.98 [0.84-1.14]	1.00 [0.85-1.19]		1.01 [0.86-1.19]	432	419	333	NA	381
D35	1.13 [1.06-1.20]	1.13 [1.06-1.20]	1.13 [1.05-1.20]		1.04 [0.97-1.11]	1.08 [1.02-1.15]	1.05 [0.99-1.12]	1.04 [0.98-1.12]		1.01 [0.95-1.08]	2,516	2,486	2,196	NA	2,170
D36	1.22 [1.12-1.33]	1.23 [1.12-1.34]	1.24 [1.12-1.37]	1.04 [0.84-1.29]	1.12 [1.02-1.23]	1.14 [1.04-1.24]	1.13 [1.03-1.24]	1.13 [1.02-1.26]	0.99 [0.79-1.24]	1.08 [0.98-1.19]	1,284	1,144	903	213	1,064
D37	1.10 [1.04-1.16]	1.10 [1.05-1.16]	1.11 [1.05-1.17]		1.09 [1.03-1.16]	1.07 [1.01-1.13]	1.06 [1.00-1.12]	1.06 [1.00-1.12]		1.09 [1.03-1.15]	3,261	3,236	3,142	NA	2,802
D38	1.22 [1.10-1.36]	1.21 [1.09-1.35]	1.20 [1.08-1.34]		1.15 [1.03-1.29]	1.20 [1.08-1.34]	1.15 [1.03-1.28]	1.14 [1.02-1.27]		1.17 [1.04-1.31]	833	823	792	NA	707
D39	1.06 [0.92-1.22]	1.02 [0.88-1.18]	0.97 [0.83-1.14]		1.09 [0.93-1.28]	1.06 [0.92-1.22]	1.02 [0.88-1.18]	0.94 [0.79-1.11]		1.12 [0.95-1.31]	459	438	329	NA	373
D40	1.12 [0.88-1.43]	1.13 [0.88-1.45]	1.21 [0.92-1.59]		1.13 [0.86-1.47]	1.11 [0.87-1.43]	1.11 [0.86-1.43]	1.17 [0.88-1.54]		1.11 [0.85-1.46]	158	151	122	NA	130
D41	1.09 [1.02-1.16]	1.09 [1.02-1.17]	1.10 [1.03-1.18]		1.02 [0.95-1.09]	1.07 [1.00-1.15]	1.06 [0.99-1.13]	1.06 [1.00-1.14]		1.02 [0.95-1.09]	2,275	2,266	2,225	NA	1,938
D43	1.13 [1.02-1.26]	1.11 [0.99-1.25]	1.06 [0.94-1.20]	1.22 [0.95-1.57]	1.09 [0.97-1.22]	1.11 [0.99-1.23]	1.07 [0.95-1.21]	1.01 [0.89-1.14]	1.23 [0.95-1.59]	1.08 [0.96-1.22]	833	701	611	164	702
D44	1.17 [1.02-1.34]	1.16 [1.01-1.34]	1.26 [1.08-1.47]		1.08 [0.94-1.26]	1.10 [0.96-1.27]	1.07 [0.92-1.24]	1.16 [0.99-1.35]		1.06 [0.91-1.23]	512	465	395	NA	439
D45	1.10 [0.99-1.23]	1.13 [1.01-1.25]	1.06 [0.96-1.19]		1.06 [0.95-1.19]	1.09 [0.98-1.21]	1.06 [0.95-1.18]	1.00 [0.89-1.11]		1.04 [0.93-1.17]	839	837	776	NA	704
D46	1.32 [1.24-1.42]	1.34 [1.26-1.44]	1.33 [1.24-1.42]		1.26 [1.17-1.35]	1.29 [1.20-1.38]	1.28 [1.19-1.37]	1.26 [1.18-1.35]		1.24 [1.16-1.34]	2,285	2,273	2,247	NA	2,056
D47	1.26 [1.20-1.33]	1.27 [1.20-1.34]	1.25 [1.19-1.32]	1.36 [0.98-1.88]	1.19 [1.13-1.26]	1.20 [1.13-1.26]	1.18 [1.11-1.24]	1.16 [1.10-1.23]	1.30 [0.92-1.82]	1.16 [1.09-1.22]	3,677	3,627	3,488	101	3,408
D48	1.09 [1.01-1.17]	1.12 [1.03-1.21]	1.11 [1.02-1.20]	0.93 [0.75-1.15]	1.03 [0.95-1.11]	1.05 [0.97-1.13]	1.06 [0.98-1.15]	1.05 [0.96-1.15]	0.92 [0.74-1.15]	1.01 [0.93-1.09]	1,721	1,557	1,319	229	1,400
Diseases of the blood and blood-forming organs and certain disorders involving the immune mechanism (D50-D89)															
D50	1.32 [1.31-1.34]	1.32 [1.30-1.34]	1.32 [1.30-1.34]	1.28 [1.21-1.35]	1.25 [1.23-1.27]	1.25 [1.23-1.27]	1.20 [1.18-1.22]	1.20 [1.18-1.22]	1.24 [1.18-1.31]	1.20 [1.19-1.22]	53,162	50,841	45,714	4,007	47,985
D51	1.38 [1.32-1.44]	1.35 [1.29-1.42]	1.36 [1.30-1.43]	1.43 [1.07-1.91]	1.29 [1.23-1.35]	1.29 [1.23-1.35]	1.23 [1.17-1.29]	1.24 [1.18-1.31]	1.33 [0.99-1.80]	1.23 [1.17-1.29]	4,681	4,667	4,277	132	4,210
D52	1.28 [1.21-1.35]	1.31 [1.24-1.38]	1.30 [1.23-1.38]		1.25 [1.18-1.32]	1.23 [1.17-1.31]	1.22 [1.15-1.29]	1.20 [1.14-1.28]		1.24 [1.17-1.31]	3,437	3,417	3,254	NA	3,181
D53	1.42 [1.28-1.58]	1.43 [1.28-1.59]	1.53 [1.37-1.71]		1.35 [1.21-1.50]	1.34 [1.20-1.49]	1.30 [1.16-1.45]	1.37 [1.23-1.54]		1.30 [1.17-1.45]	1,003	976	937	NA	928
D55	1.50 [1.17-1.91]	1.61 [1.18-2.18]			1.40 [1.08-1.82]	1.47 [1.14-1.89]	1.50 [1.09-2.06]			1.41 [1.08-1.83]	181	108	NA	NA	157
D56	1.40 [1.31-1.50]	1.42 [1.32-1.53]	1.54 [1.40-1.70]	1.34 [1.16-1.56]	1.27 [1.18-1.37]	1.37 [1.27-1.47]	1.37 [1.27-1.48]	1.42 [1.28-1.57]	1.31 [1.12-1.53]	1.25 [1.16-1.35]	2,201	1,898	1,101	518	1,811
D57	1.31 [1.23-1.40]	1.37 [1.27-1.47]	1.30 [1.17-1.44]	1.36 [1.23-1.51]	1.22 [1.13-1.31]	1.28 [1.20-1.37]	1.32 [1.22-1.43]	1.23 [1.10-1.37]	1.36 [1.22-1.51]	1.21 [1.12-1.30]	2,519	1,759	859	1,054	1,979
D58	1.21 [1.05-1.38]	1.24 [1.07-1.44]	1.18 [0.99-1.40]	1.21 [0.92-1.59]	1.17 [1.01-1.35]	1.19 [1.04-1.37]	1.19 [1.02-1.39]	1.12 [0.94-1.34]	1.20 [0.90-1.58]	1.17 [1.01-1.35]	534	433	317	137	466
D59	1.44 [1.28-1.62]	1.47 [1.30-1.67]	1.43 [1.25-1.64]	1.27 [0.96-1.67]	1.36 [1.20-1.54]	1.42 [1.26-1.59]	1.43 [1.25-1.63]	1.38 [1.20-1.58]	1.25 [0.94-1.67]	1.36 [1.20-1.55]	745	639	566	133	654
D61	1.31 [1.24-1.39]	1.33 [1.25-1.41]	1.30 [1.22-1.38]	1.07 [0.87-1.30]	1.25 [1.18-1.32]	1.27 [1.20-1.35]	1.26 [1.19-1.34]	1.22 [1.15-1.30]	1.04 [0.84-1.27]	1.24 [1.17-1.31]	3,263	3,099	2,850	249	2,999
D62	1.13 [1.04-1.23]	1.13 [1.04-1.23]	1.18 [1.05-1.33]	0.99 [0.79-1.23]	1.07 [0.98-1.18]	1.12 [1.03-1.22]	1.10 [1.01-1.20]	1.12 [0.99-1.26]	0.98 [0.78-1.23]	1.08 [0.99-1.19]	1,430	1,401	686	211	1,170
D63	1.28 [1.23-1.34]	1.29 [1.23-1.35]	1.27 [1.21-1.33]	1.18 [0.86-1.62]	1.20 [1.15-1.26]	1.24 [1.19-1.30]	1.21 [1.15-1.27]	1.18 [1.13-1.24]	1.16 [0.83-1.60]	1.20 [1.14-1.26]	4,840	4,774	4,591	103	4,427
D64	1.30 [1.28-1.31]	1.30 [1.29-1.32]	1.30 [1.29-1.32]	1.17 [1.11-1.23]	1.23 [1.21-1.24]	1.24 [1.23-1.26]	1.21 [1.19-1.22]	1.21 [1.19-1.22]	1.14 [1.08-1.21]	1.20 [1.18-1.21]	69,443	67,794	61,959	3,476	62,148
D65	1.19 [0.96-1.46]	1.25 [1.00-1.57]	1.21 [0.95-1.53]		1.16 [0.93-1.45]	1.17 [0.94-1.44]	1.21 [0.96-1.51]	1.17 [0.92-1.49]		1.17 [0.93-1.46]	221	199	174	NA	190
D66	1.15 [0.94-1.40]	1.13 [0.90-1.43]	1.18 [0.90-1.57]		1.20 [0.97-1.50]	1.11 [0.90-1.36]	1.03 [0.81-1.31]	1.09 [0.82-1.45]		1.17 [0.94-1.47]	242	177	121	NA	204
D68	1.29 [1.22-1.35]	1.32 [1.25-1.39]	1.34 [1.26-1.42]	1.09 [0.96-1.23]	1.19 [1.13-1.25]	1.19 [1.13-1.25]	1.18 [1.11-1.24]	1.18 [1.11-1.26]	1.03 [0.90-1.17]	1.13 [1.07-1.19]	3,974	3,599	2,602	650	3,500
D69	1.23 [1.20-1.27]	1.27 [1.23-1.31]	1.28 [1.24-1.33]	1.06 [1.00-1.13]	1.18 [1.14-1.21]	1.20 [1.16-1.23]	1.20 [1.16-1.24]	1.20 [1.16-1.25]	1.06 [1.00-1.13]	1.17 [1.13-1.20]	11,780	9,406	8,184	2,747	10,458
D70	1.23 [1.19-1.27]	1.24 [1.20-1.28]	1.23 [1.19-1.28]	1.14 [1.04-1.24]	1.18 [1.14-1.22]	1.20 [1.16-1.24]	1.19 [1.15-1.23]	1.19 [1.15-1.23]	1.11 [1.02-1.22]	1.17 [1.13-1.21]	9,777	8,724	7,840	1,334	8,508
D72	1.43 [1.34-1.53]	1.44 [1.35-1.55]	1.40 [1.30-1.51]	1.41 [1.18-1.68]	1.35 [1.26-1.45]	1.31 [1.22-1.40]	1.27 [1.18-1.37]	1.25 [1.16-1.35]	1.31 [1.10-1.58]	1.26 [1.17-1.35]	2,421	2,176	1,866	358	2,199
D73	1.25 [1.17-1.34]	1.24 [1.16-1.32]	1.26 [1.17-1.35]	0.92 [0.72-1.18]	1.16 [1.08-1.24]	1.20 [1.12-1.28]	1.15 [1.07-1.23]	1.16 [1.08-1.25]	0.95 [0.74-1.22]	1.14 [1.07-1.23]	2,222	2,116	1,898	169	2,030
D75	1.21 [1.14-1.29]	1.22 [1.15-1.30]	1.21 [1.14-1.29]	1.18 [0.86-1.62]	1.16 [1.09-1.24]	1.17 [1.10-1.24]	1.12 [1.05-1.19]	1.12 [1.05-1.19]	1.12 [0.81-1.55]	1.15 [1.07-1.22]	2,692	2,637	2,448	107	2,406
D76	1.27 [1.02-1.57]	1.37 [1.06-1.78]	1.38 [1.03-1.85]		1.28 [1.01-1.61]	1.20 [0.97-1.50]	1.26 [0.96-1.65]	1.27 [0.94-1.72]		1.21 [0.96-1.54]	213	141	108	NA	178
D80	1.54 [1.37-1.73]	1.56 [1.36-1.77]	1.46 [1.27-1.69]	1.47 [1.18-1.84]	1.44 [1.28-1.62]	1.36 [1.20-1.53]	1.32 [1.15-1.52]	1.21 [1.04-1.41]	1.33 [1.06-1.68]	1.30 [1.15-1.47]	784	597	494	223	735
D82	1.31 [1.04-1.65]	1.51 [1.12-2.02]			1.27 [1.00-1.62]	1.19 [0.94-1.51]	1.33 [0.98-1.80]			1.19 [0.92-1.53]	188	116	NA	NA	169
D83	2.01 [1.46-2.75]	2.05 [1.48-2.85]			1.74 [1.28-2.37]	1.76 [1.27-2.43]	1.75 [1.24-2.46]			1.55 [1.12-2.13]	119	104	NA	NA	114
D84	1.71 [1.47-1.99]	1.70 [1.41-2.05]	1.75 [1.42-2.16]	1.76 [1.37-2.27]	1.55 [1.32-1.81]	1.43 [1.22-1.69]	1.35 [1.10-1.65]	1.38 [1.10-1.72]	1.59 [1.22-2.06]	1.33 [1.13-1.56]	464	296	234	186	433
D86	1.58 [1.46-1.71]	1.56 [1.44-1.69]	1.51 [1.39-1.64]		1.43 [1.32-1.56]	1.47 [1.35-1.59]	1.41 [1.30-1.54]	1.35 [1.23-1.47]		1.34 [1.23-1.46]	1,617	1,586	1,381	NA	1,402
D89	1.43 [1.31-1.57]	1.42 [1.29-1.56]	1.41 [1.28-1.55]	1.53 [1.13-2.08]	1.32 [1.20-1.45]	1.33 [1.21-1.46]	1.29 [1.17-1.42]	1.28 [1.16-1.41]	1.37 [1.00-1.89]	1.26 [1.14-1.38]	1,274	1,180	1,098	120	1,133
Endocrine, nutritional and metabolic diseases (E00-E89)															
E02	1.14 [0.97-1.35]	1.20 [1.02-1.41]	1.11 [0.93-1.32]		1.11 [0.93-1.31]	1.12 [0.95-1.33]	1.16 [0.98-1.37]	1.07 [0.89-1.28]		1.11 [0.93-1.31]	395	392	347	NA	364
E03	1.34 [1.32-1.36]	1.34 [1.32-1.36]	1.33 [1.31-1.35]	1.20 [1.11-1.31]	1.26 [1.24-1.28]	1.28 [1.26-1.30]	1.24 [1.23-1.26]	1.24 [1.22-1.26]	1.18 [1.09-1.29]	1.22 [1.20-1.24]	50,515	49,910	44,793	1,612	41,507
E04	1.15 [1.10-1.20]	1.13 [1.08-1.18]	1.12 [1.07-1.17]	1.24 [0.98-1.58]	1.09 [1.04-1.15]	1.09 [1.04-1.14]	1.05 [1.01-1.10]	1.04 [0.99-1.09]	1.24 [0.97-1.59]	1.06 [1.01-1.12]	4,652	4,564	4,023	181	4,054
E05	1.29 [1.25-1.34]	1.29 [1.24-1.33]	1.27 [1.22-1.32]	1.28 [1.09-1.50]	1.21 [1.17-1.25]	1.23 [1.18-1.27]	1.18 [1.14-1.22]	1.17 [1.12-1.21]	1.24 [1.05-1.46]	1.16 [1.12-1.21]	8,314	8,168	6,931	419	7,289
E06	1.22 [1.10-1.35]	1.22 [1.10-1.36]	1.24 [1.10-1.40]		1.20 [1.07-1.34]	1.17 [1.05-1.30]	1.13 [1.01-1.26]	1.16 [1.02-1.31]		1.14 [1.02-1.28]	883	841	636	NA	787
E07	1.26 [1.16-1.37]	1.29 [1.19-1.41]	1.27 [1.16-1.39]		1.18 [1.08-1.29]	1.17 [1.07-1.27]	1.17 [1.08-1.28]	1.15 [1.05-1.26]		1.11 [1.01-1.21]	1,392	1,364	1,204	NA	1,266
E10	1.23 [1.19-1.26]	1.30 [1.25-1.34]	1.32 [1.27-1.36]	0.97 [0.91-1.04]	1.14 [1.11-1.18]	1.15 [1.12-1.19]	1.12 [1.09-1.16]	1.13 [1.09-1.17]	0.96 [0.90-1.03]	1.11 [1.07-1.15]	10,587	8,646	7,458	2,324	8,645
E11	1.26 [1.24-1.27]	1.26 [1.24-1.27]	1.25 [1.23-1.26]	1.25 [1.09-1.43]	1.20 [1.18-1.21]	1.19 [1.18-1.20]	1.12 [1.11-1.14]	1.11 [1.10-1.12]	1.23 [1.08-1.41]	1.16 [1.15-1.18]	74,974	74,783	72,124	622	59,224
E13	1.41 [1.28-1.55]	1.39 [1.26-1.54]	1.43 [1.29-1.59]		1.29 [1.16-1.42]	1.25 [1.13-1.39]	1.18 [1.06-1.31]	1.21 [1.09-1.35]		1.19 [1.08-1.32]	1,051	1,010	890	NA	972
E14	1.37 [1.33-1.41]	1.38 [1.34-1.42]	1.37 [1.33-1.41]	0.92 [0.75-1.14]	1.27 [1.23-1.31]	1.25 [1.22-1.29]	1.19 [1.15-1.23]	1.17 [1.13-1.21]	0.92 [0.74-1.14]	1.21 [1.17-1.25]	11,113	11,014	10,369	228	10,005
E16	1.25 [1.22-1.29]	1.27 [1.24-1.31]	1.27 [1.23-1.31]	1.07 [0.99-1.15]	1.16 [1.13-1.20]	1.18 [1.14-1.21]	1.11 [1.08-1.14]	1.10 [1.07-1.14]	1.05 [0.97-1.14]	1.14 [1.10-1.17]	12,636	11,294	10,504	1,633	11,955
E20	1.44 [1.21-1.70]	1.49 [1.25-1.77]	1.53 [1.27-1.84]		1.32 [1.11-1.56]	1.26 [1.06-1.50]	1.30 [1.09-1.56]	1.33 [1.10-1.62]		1.23 [1.03-1.46]	372	343	302	NA	349
E21	1.17 [1.11-1.23]	1.17 [1.11-1.23]	1.16 [1.10-1.22]		1.08 [1.03-1.15]	1.12 [1.06-1.18]	1.08 [1.02-1.14]	1.07 [1.02-1.13]		1.06 [1.00-1.12]	3,577	3,548	3,397	NA	3,267
E22	1.48 [1.39-1.57]	1.47 [1.38-1.56]	1.51 [1.41-1.61]	1.35 [1.06-1.71]	1.39 [1.30-1.48]	1.41 [1.32-1.50]	1.37 [1.28-1.46]	1.41 [1.32-1.50]	1.28 [1.00-1.63]	1.34 [1.26-1.43]	2,906	2,778	2,574	199	2,627
E23	1.39 [1.28-1.50]	1.43 [1.30-1.56]	1.49 [1.34-1.65]	1.24 [1.07-1.43]	1.25 [1.15-1.36]	1.29 [1.19-1.40]	1.27 [1.15-1.39]	1.33 [1.20-1.48]	1.18 [1.02-1.37]	1.20 [1.10-1.30]	1,650	1,222	958	519	1,470
E24	1.60 [1.35-1.90]	1.46 [1.22-1.74]	1.61 [1.33-1.96]		1.40 [1.17-1.67]	1.32 [1.10-1.58]	1.12 [0.93-1.36]	1.24 [1.01-1.52]		1.21 [1.01-1.45]	357	319	263	NA	327
E26	1.21 [0.95-1.54]	1.16 [0.91-1.49]	1.14 [0.87-1.50]		1.16 [0.90-1.50]	1.13 [0.88-1.44]	1.04 [0.80-1.34]	0.97 [0.73-1.29]		1.14 [0.88-1.48]	163	150	124	NA	142
E27	1.49 [1.40-1.57]	1.48 [1.39-1.57]	1.45 [1.36-1.54]	1.46 [1.24-1.71]	1.37 [1.29-1.45]	1.34 [1.26-1.42]	1.28 [1.20-1.36]	1.25 [1.17-1.34]	1.32 [1.12-1.55]	1.27 [1.19-1.35]	3,166	2,827	2,446	456	2,935
E28	1.21 [1.17-1.26]	1.24 [1.20-1.29]	1.26 [1.15-1.39]	1.13 [1.06-1.21]	1.18 [1.13-1.23]	1.11 [1.07-1.15]	1.08 [1.04-1.12]	1.05 [0.95-1.16]	1.10 [1.03-1.18]	1.11 [1.06-1.16]	7,155	6,884	991	2,338	5,846
E29	1.71 [1.47-1.98]	1.76 [1.51-2.05]	1.76 [1.50-2.08]		1.45 [1.25-1.69]	1.50 [1.28-1.75]	1.42 [1.21-1.67]	1.40 [1.18-1.66]		1.32 [1.13-1.55]	466	445	385	NA	430
E30	1.24 [1.11-1.39]			1.22 [1.09-1.37]	1.15 [1.02-1.30]	1.19 [1.06-1.34]			1.17 [1.04-1.32]	1.13 [0.99-1.28]	843	NA	NA	827	725
E34	1.23 [1.13-1.34]	1.31 [1.12-1.53]	1.39 [1.14-1.70]	1.19 [1.08-1.31]	1.16 [1.06-1.27]	1.14 [1.05-1.24]	1.13 [0.96-1.34]	1.23 [1.00-1.50]	1.11 [1.01-1.22]	1.11 [1.02-1.22]	1,485	395	242	1,140	1,328
E41	1.07 [0.80-1.42]	1.05 [0.78-1.41]	1.10 [0.82-1.48]		1.15 [0.86-1.55]	1.10 [0.82-1.47]	1.06 [0.78-1.42]	1.09 [0.81-1.48]		1.20 [0.89-1.61]	123	121	113	NA	118
E43	1.20 [0.94-1.54]	1.09 [0.84-1.40]	1.24 [0.94-1.63]		1.11 [0.86-1.43]	1.18 [0.92-1.53]	1.00 [0.77-1.30]	1.20 [0.91-1.60]		1.13 [0.87-1.47]	158	148	130	NA	149
E46	1.22 [1.13-1.31]	1.18 [1.10-1.27]	1.16 [1.07-1.25]	1.29 [0.97-1.73]	1.16 [1.08-1.25]	1.19 [1.11-1.28]	1.10 [1.02-1.18]	1.09 [1.01-1.18]	1.25 [0.93-1.69]	1.17 [1.08-1.26]	2,023	1,956	1,779	135	1,874
E51	1.47 [1.19-1.82]	1.41 [1.14-1.74]	1.48 [1.19-1.84]		1.36 [1.09-1.70]	1.39 [1.12-1.73]	1.23 [0.99-1.54]	1.28 [1.02-1.61]		1.38 [1.10-1.73]	216	213	200	NA	197
E53	1.30 [1.27-1.34]	1.31 [1.27-1.35]	1.28 [1.24-1.33]	1.27 [1.11-1.45]	1.24 [1.20-1.28]	1.23 [1.20-1.27]	1.18 [1.15-1.22]	1.17 [1.13-1.21]	1.19 [1.04-1.37]	1.19 [1.16-1.23]	12,236	12,126	10,701	648	11,410
E55	1.37 [1.34-1.40]	1.38 [1.34-1.42]	1.35 [1.32-1.39]	1.34 [1.25-1.45]	1.30 [1.26-1.33]	1.26 [1.23-1.29]	1.22 [1.19-1.25]	1.20 [1.17-1.24]	1.26 [1.17-1.35]	1.23 [1.19-1.26]	18,051	16,575	14,208	2,279	16,868
E56	1.27 [1.00-1.62]	1.29 [1.00-1.67]	1.21 [0.92-1.60]		1.21 [0.94-1.54]	1.19 [0.93-1.53]	1.14 [0.87-1.48]	1.10 [0.83-1.46]		1.18 [0.92-1.52]	176	155	132	NA	165
E58	1.55 [1.18-2.04]	1.41 [1.06-1.88]			1.55 [1.17-2.06]	1.48 [1.11-1.95]	1.25 [0.93-1.68]			1.50 [1.13-1.99]	141	124	NA	NA	134
E61	1.38 [1.30-1.47]	1.39 [1.30-1.48]	1.39 [1.29-1.49]	1.35 [1.16-1.57]	1.27 [1.19-1.35]	1.26 [1.18-1.34]	1.21 [1.14-1.30]	1.22 [1.13-1.31]	1.27 [1.09-1.48]	1.19 [1.12-1.27]	2,835	2,508	2,090	509	2,635
E63	1.30 [1.01-1.67]	1.17 [0.89-1.52]	1.46 [1.11-1.94]		1.23 [0.95-1.59]	1.24 [0.96-1.60]	1.08 [0.82-1.41]	1.32 [0.99-1.77]		1.18 [0.91-1.53]	165	143	134	NA	157
E65	1.45 [1.08-1.95]	1.54 [1.14-2.08]				1.33 [0.98-1.80]	1.39 [1.02-1.90]				113	107	NA	NA	NA
E66	1.30 [1.29-1.32]	1.32 [1.30-1.33]	1.32 [1.31-1.34]	1.18 [1.14-1.22]	1.22 [1.21-1.24]	1.20 [1.19-1.21]	1.15 [1.14-1.17]	1.15 [1.13-1.16]	1.13 [1.10-1.17]	1.15 [1.14-1.17]	88,230	86,031	60,487	11,250	77,698
E70	1.35 [1.02-1.79]				1.25 [0.91-1.72]	1.30 [0.98-1.74]				1.22 [0.88-1.69]	128	NA	NA	NA	101
E72	1.17 [0.93-1.46]	1.39 [1.07-1.81]	1.52 [1.12-2.08]		1.13 [0.90-1.43]	1.09 [0.87-1.37]	1.24 [0.94-1.62]	1.39 [1.01-1.90]		1.12 [0.89-1.43]	189	139	104	NA	175
E73	1.41 [1.28-1.55]	1.38 [1.21-1.57]	1.35 [1.14-1.59]	1.36 [1.20-1.55]	1.32 [1.19-1.45]	1.22 [1.11-1.35]	1.11 [0.96-1.27]	1.10 [0.92-1.30]	1.23 [1.08-1.41]	1.16 [1.05-1.29]	1,148	578	356	660	1,014
E74	1.35 [1.14-1.60]	1.34 [1.10-1.63]	1.37 [1.10-1.70]	1.32 [0.98-1.78]	1.29 [1.08-1.53]	1.24 [1.05-1.48]	1.22 [1.00-1.49]	1.25 [1.01-1.56]	1.22 [0.89-1.66]	1.23 [1.03-1.47]	365	257	210	126	324
E75	1.20 [0.93-1.55]				1.11 [0.84-1.47]	1.17 [0.90-1.52]				1.12 [0.85-1.48]	144	NA	NA	NA	125
E78	1.23 [1.22-1.24]	1.23 [1.22-1.24]	1.23 [1.22-1.24]	1.18 [0.98-1.43]	1.15 [1.14-1.17]	1.17 [1.16-1.18]	1.12 [1.10-1.13]	1.11 [1.10-1.12]	1.16 [0.95-1.41]	1.12 [1.11-1.14]	97,183	97,116	95,613	285	82,546
E79	1.32 [1.01-1.72]	1.35 [1.02-1.77]	1.43 [1.07-1.92]		1.19 [0.90-1.58]	1.25 [0.95-1.65]	1.21 [0.91-1.60]	1.25 [0.92-1.69]		1.13 [0.85-1.50]	141	132	115	NA	123
E80	1.17 [1.07-1.28]	1.21 [1.11-1.33]	1.21 [1.09-1.35]	1.12 [0.92-1.36]	1.08 [0.99-1.19]	1.09 [1.00-1.20]	1.11 [1.01-1.22]	1.13 [1.01-1.26]	1.05 [0.86-1.29]	1.03 [0.94-1.13]	1,292	1,155	824	270	1,115
E83	1.21 [1.18-1.24]	1.22 [1.19-1.25]	1.20 [1.17-1.23]	1.08 [0.96-1.20]	1.15 [1.12-1.18]	1.16 [1.13-1.19]	1.12 [1.09-1.15]	1.10 [1.08-1.13]	1.06 [0.95-1.19]	1.13 [1.10-1.16]	17,831	17,352	16,193	875	16,376
E84	1.23 [0.96-1.58]				1.14 [0.87-1.50]	1.11 [0.86-1.43]				1.03 [0.78-1.36]	160	NA	NA	NA	134
E85	1.25 [1.10-1.41]	1.22 [1.07-1.38]	1.18 [1.04-1.35]		1.18 [1.04-1.34]	1.20 [1.06-1.36]	1.14 [1.00-1.29]	1.10 [0.96-1.25]		1.14 [1.00-1.30]	655	623	590	NA	596
E86	1.20 [1.18-1.21]	1.19 [1.17-1.21]	1.19 [1.17-1.21]	1.13 [1.08-1.18]	1.14 [1.12-1.16]	1.16 [1.14-1.18]	1.12 [1.10-1.14]	1.12 [1.10-1.14]	1.10 [1.05-1.15]	1.13 [1.11-1.15]	47,668	43,859	41,005	4,893	43,955
E87	1.28 [1.27-1.30]	1.29 [1.27-1.30]	1.28 [1.27-1.30]	1.09 [1.02-1.15]	1.22 [1.21-1.24]	1.23 [1.22-1.25]	1.18 [1.17-1.20]	1.18 [1.17-1.20]	1.06 [1.00-1.13]	1.20 [1.18-1.21]	73,651	71,910	69,111	2,751	67,831
E88	1.29 [1.23-1.36]	1.32 [1.24-1.39]	1.31 [1.23-1.39]	1.16 [0.99-1.36]	1.23 [1.16-1.30]	1.23 [1.17-1.30]	1.21 [1.14-1.28]	1.20 [1.13-1.27]	1.12 [0.95-1.32]	1.20 [1.13-1.27]	3,596	3,299	3,016	408	3,317
E89	1.25 [1.19-1.31]	1.26 [1.20-1.32]	1.25 [1.19-1.31]	1.11 [0.84-1.47]	1.15 [1.10-1.21]	1.17 [1.12-1.23]	1.14 [1.09-1.20]	1.13 [1.08-1.19]	1.09 [0.81-1.47]	1.11 [1.06-1.16]	4,522	4,479	4,023	133	4,194
Mental, Behavioral and Neurodevelopmental disorders (F01-F99)															
F00	1.06 [1.03-1.10]	1.06 [1.03-1.10]	1.07 [1.03-1.10]		1.05 [1.02-1.09]	1.06 [1.03-1.10]	1.04 [1.01-1.08]	1.05 [1.01-1.08]		1.05 [1.01-1.08]	10,162	10,154	10,158	NA	9,384
F01	1.10 [1.07-1.13]	1.10 [1.06-1.13]	1.09 [1.06-1.12]		1.06 [1.03-1.10]	1.08 [1.05-1.12]	1.05 [1.02-1.08]	1.04 [1.00-1.07]		1.06 [1.03-1.09]	11,780	11,779	11,773	NA	11,049
F02	1.01 [0.94-1.09]	1.01 [0.94-1.09]	1.02 [0.95-1.10]		0.95 [0.88-1.03]	1.02 [0.95-1.10]	0.99 [0.92-1.07]	1.00 [0.93-1.08]		0.97 [0.90-1.05]	1,911	1,907	1,898	NA	1,762
F03	1.07 [1.05-1.09]	1.06 [1.04-1.09]	1.07 [1.05-1.09]		1.04 [1.02-1.06]	1.07 [1.05-1.09]	1.04 [1.02-1.06]	1.04 [1.02-1.06]		1.04 [1.02-1.07]	28,174	28,162	28,133	NA	25,894
F05	1.17 [1.15-1.20]	1.17 [1.15-1.19]	1.17 [1.15-1.19]	1.00 [0.77-1.30]	1.14 [1.12-1.16]	1.14 [1.12-1.17]	1.10 [1.08-1.13]	1.10 [1.08-1.13]	1.01 [0.78-1.32]	1.13 [1.11-1.16]	27,443	27,341	27,145	161	26,053
F06	1.16 [1.10-1.22]	1.16 [1.10-1.22]	1.17 [1.11-1.23]		1.10 [1.04-1.16]	1.13 [1.07-1.19]	1.10 [1.04-1.16]	1.11 [1.05-1.16]		1.09 [1.04-1.15]	4,231	4,198	4,118	NA	3,968
F07	1.28 [1.07-1.53]	1.29 [1.07-1.55]	1.35 [1.09-1.68]		1.09 [0.90-1.32]	1.18 [0.99-1.42]	1.16 [0.96-1.41]	1.23 [0.98-1.53]		1.08 [0.89-1.31]	319	278	207	NA	271
F10	1.20 [1.18-1.23]	1.23 [1.20-1.25]	1.26 [1.23-1.29]	0.98 [0.93-1.04]	1.14 [1.11-1.16]	1.17 [1.15-1.19]	1.11 [1.09-1.14]	1.15 [1.12-1.18]	0.97 [0.92-1.02]	1.15 [1.12-1.18]	23,382	22,021	16,404	3,621	18,640
F11	1.17 [1.08-1.26]	1.18 [1.10-1.28]	1.30 [1.17-1.45]	1.00 [0.81-1.25]	1.07 [0.99-1.16]	1.08 [1.00-1.17]	0.95 [0.88-1.03]	1.05 [0.94-1.17]	1.07 [0.85-1.35]	1.06 [0.97-1.15]	1,667	1,649	834	200	1,382
F12	1.08 [1.03-1.14]	1.12 [1.06-1.18]	1.28 [1.17-1.41]	0.96 [0.89-1.04]	0.95 [0.90-1.01]	1.01 [0.96-1.07]	0.95 [0.90-1.00]	1.06 [0.96-1.17]	0.97 [0.89-1.05]	0.96 [0.91-1.02]	3,794	3,351	1,033	1,749	3,051
F13	1.26 [1.08-1.47]	1.24 [1.06-1.45]	1.42 [1.16-1.74]		1.10 [0.93-1.29]	1.14 [0.97-1.33]	0.98 [0.83-1.15]	1.14 [0.92-1.41]		1.06 [0.89-1.24]	416	387	236	NA	367
F14	1.12 [1.03-1.21]	1.16 [1.07-1.26]	1.42 [1.22-1.65]	1.02 [0.89-1.16]	0.98 [0.90-1.07]	1.04 [0.95-1.13]	0.97 [0.89-1.05]	1.13 [0.97-1.33]	1.03 [0.89-1.18]	0.99 [0.91-1.08]	1,455	1,412	404	530	1,208
F15	1.03 [0.90-1.19]	1.04 [0.90-1.20]	1.16 [0.91-1.48]	0.95 [0.76-1.18]	0.93 [0.80-1.08]	0.97 [0.85-1.12]	0.93 [0.80-1.08]	0.98 [0.76-1.27]	0.92 [0.73-1.16]	0.94 [0.81-1.10]	503	438	148	200	403
F16	0.95 [0.73-1.22]	0.87 [0.66-1.15]			0.87 [0.66-1.14]	0.91 [0.70-1.19]	0.78 [0.59-1.03]			0.88 [0.66-1.17]	153	114	NA	NA	120
F17	1.13 [1.12-1.14]	1.15 [1.14-1.16]	1.15 [1.14-1.17]	0.99 [0.97-1.01]	1.06 [1.05-1.07]	1.11 [1.10-1.12]	1.02 [1.01-1.03]	1.04 [1.03-1.05]	1.00 [0.98-1.02]	1.09 [1.08-1.10]	108,659	105,861	64,812	21,247	90,044
F19	1.07 [0.99-1.16]	1.08 [1.00-1.17]	1.22 [1.07-1.39]	0.93 [0.81-1.06]	0.96 [0.89-1.05]	1.00 [0.92-1.09]	0.89 [0.82-0.97]	0.96 [0.84-1.10]	0.96 [0.83-1.10]	0.98 [0.90-1.07]	1,561	1,442	535	525	1,355
F20	1.28 [1.21-1.34]	1.27 [1.20-1.34]	1.32 [1.24-1.40]	1.00 [0.84-1.20]	1.15 [1.08-1.22]	1.26 [1.19-1.33]	1.17 [1.11-1.24]	1.25 [1.18-1.34]	1.04 [0.86-1.25]	1.17 [1.10-1.24]	3,454	3,406	2,516	313	2,702
F22	1.29 [1.19-1.40]	1.28 [1.18-1.39]	1.32 [1.21-1.45]	1.00 [0.74-1.36]	1.19 [1.10-1.30]	1.23 [1.14-1.34]	1.18 [1.08-1.28]	1.22 [1.11-1.33]	0.99 [0.72-1.35]	1.19 [1.09-1.29]	1,560	1,515	1,291	113	1,372
F23	1.16 [1.05-1.29]	1.12 [1.01-1.24]	1.28 [1.12-1.48]	0.94 [0.75-1.17]	1.07 [0.96-1.20]	1.13 [1.02-1.26]	1.05 [0.94-1.17]	1.20 [1.04-1.38]	0.93 [0.74-1.17]	1.08 [0.96-1.21]	916	838	494	194	720
F25	1.36 [1.21-1.52]	1.34 [1.20-1.50]	1.38 [1.20-1.58]		1.11 [0.99-1.25]	1.32 [1.17-1.49]	1.19 [1.06-1.34]	1.27 [1.10-1.46]		1.15 [1.02-1.30]	747	741	510	NA	670
F29	1.22 [1.13-1.32]	1.26 [1.17-1.36]	1.40 [1.28-1.54]	0.91 [0.77-1.07]	1.11 [1.03-1.20]	1.16 [1.07-1.25]	1.11 [1.03-1.20]	1.24 [1.13-1.37]	0.92 [0.77-1.09]	1.11 [1.02-1.20]	1,809	1,696	1,103	375	1,564
F30	1.34 [1.16-1.54]	1.33 [1.15-1.54]	1.42 [1.21-1.68]		1.19 [1.02-1.39]	1.28 [1.11-1.48]	1.21 [1.05-1.41]	1.31 [1.11-1.55]		1.18 [1.01-1.38]	482	465	350	NA	391
F31	1.31 [1.24-1.37]	1.32 [1.26-1.39]	1.33 [1.25-1.41]	1.07 [0.93-1.23]	1.16 [1.10-1.22]	1.19 [1.13-1.25]	1.08 [1.03-1.14]	1.12 [1.06-1.20]	1.08 [0.93-1.24]	1.12 [1.06-1.18]	4,288	4,215	2,717	559	3,768
F32	1.37 [1.35-1.38]	1.38 [1.36-1.40]	1.39 [1.37-1.41]	1.14 [1.10-1.17]	1.26 [1.25-1.28]	1.23 [1.22-1.25]	1.13 [1.12-1.14]	1.15 [1.14-1.17]	1.13 [1.09-1.16]	1.20 [1.18-1.21]	83,130	80,435	58,205	11,060	73,576
F33	1.31 [1.23-1.39]	1.30 [1.22-1.39]	1.28 [1.19-1.37]	1.10 [0.84-1.44]	1.19 [1.11-1.27]	1.18 [1.10-1.26]	1.07 [1.00-1.14]	1.05 [0.98-1.14]	1.18 [0.89-1.56]	1.15 [1.07-1.23]	2,416	2,378	1,853	139	2,125
F34	1.44 [1.24-1.68]	1.47 [1.26-1.72]	1.52 [1.27-1.83]		1.23 [1.05-1.44]	1.25 [1.06-1.46]	1.18 [1.00-1.39]	1.24 [1.02-1.50]		1.13 [0.96-1.32]	448	416	299	NA	392
F38	1.30 [1.04-1.64]	1.27 [1.00-1.62]	1.21 [0.92-1.61]		1.16 [0.92-1.47]	1.19 [0.94-1.51]	1.09 [0.85-1.40]	1.04 [0.78-1.39]		1.10 [0.86-1.41]	199	176	126	NA	179
F39	1.33 [1.12-1.57]	1.35 [1.13-1.61]	1.33 [1.08-1.64]		1.24 [1.04-1.47]	1.20 [1.00-1.42]	1.10 [0.92-1.33]	1.15 [0.93-1.43]		1.17 [0.98-1.40]	368	318	237	NA	337
F40	1.26 [1.21-1.30]	1.30 [1.25-1.35]	1.30 [1.24-1.36]	1.13 [1.05-1.21]	1.19 [1.14-1.24]	1.16 [1.12-1.21]	1.13 [1.09-1.18]	1.14 [1.08-1.19]	1.08 [1.01-1.16]	1.13 [1.09-1.18]	7,877	6,569	4,366	2,284	6,831
F41	1.34 [1.32-1.36]	1.35 [1.33-1.37]	1.36 [1.34-1.38]	1.16 [1.13-1.20]	1.25 [1.23-1.26]	1.20 [1.19-1.22]	1.11 [1.10-1.13]	1.13 [1.12-1.15]	1.14 [1.10-1.17]	1.17 [1.16-1.19]	70,774	66,825	45,718	12,693	62,988

Supplementary Table 3: Hazard ratios and events from all cohorts

	Hazard ratio (99% confidence interval)										Events (in exposed)				
	crude					adjusted									
Outcome	any age	18+	40+	<18	hosp.	any age	18+	40+	<18	hosp.	any age	18+	40+	<18	hosp.
F42	1.64 [1.52-1.76]	1.67 [1.54-1.80]	1.84 [1.65-2.06]	1.25 [1.09-1.43]	1.47 [1.36-1.58]	1.43 [1.32-1.54]	1.33 [1.22-1.44]	1.53 [1.37-1.72]	1.15 [1.00-1.32]	1.34 [1.24-1.46]	2,087	1,795	888	635	1,802
F43	1.26 [1.20-1.33]	1.28 [1.21-1.35]	1.35 [1.26-1.45]	1.00 [0.90-1.12]	1.16 [1.10-1.22]	1.11 [1.05-1.17]	1.01 [0.95-1.06]	1.07 [0.99-1.15]	0.99 [0.89-1.11]	1.09 [1.04-1.15]	3,877	3,597	1,949	901	3,448
F44	1.40 [1.26-1.55]	1.43 [1.28-1.59]	1.45 [1.27-1.67]	1.27 [1.04-1.55]	1.23 [1.11-1.36]	1.21 [1.09-1.34]	1.11 [1.00-1.25]	1.15 [0.99-1.33]	1.19 [0.97-1.47]	1.11 [1.00-1.24]	1,011	905	522	279	924
F45	1.45 [1.32-1.59]	1.40 [1.27-1.54]	1.37 [1.23-1.53]	1.27 [1.02-1.60]	1.29 [1.17-1.42]	1.28 [1.16-1.41]	1.15 [1.04-1.27]	1.16 [1.04-1.30]	1.16 [0.91-1.46]	1.17 [1.06-1.29]	1,216	1,079	802	215	1,083
F48	1.55 [1.30-1.84]	1.56 [1.30-1.87]	1.51 [1.21-1.87]		1.24 [1.04-1.48]	1.35 [1.12-1.62]	1.27 [1.05-1.54]	1.20 [0.95-1.51]		1.12 [0.93-1.35]	346	306	206	NA	298
F50	1.28 [1.19-1.37]	1.29 [1.18-1.41]	1.42 [1.22-1.64]	1.13 [1.02-1.24]	1.19 [1.10-1.28]	1.17 [1.08-1.26]	1.08 [0.99-1.18]	1.19 [1.02-1.39]	1.09 [0.99-1.20]	1.13 [1.04-1.22]	2,058	1,258	443	1,189	1,711
F51	1.43 [1.23-1.67]	1.57 [1.30-1.89]	1.50 [1.20-1.88]	1.33 [1.05-1.68]	1.37 [1.17-1.60]	1.30 [1.11-1.52]	1.32 [1.09-1.60]	1.27 [1.00-1.60]	1.31 [1.03-1.67]	1.27 [1.08-1.50]	458	302	201	193	411
F52	1.49 [1.32-1.69]	1.48 [1.30-1.67]	1.48 [1.30-1.68]		1.32 [1.16-1.50]	1.35 [1.19-1.53]	1.25 [1.10-1.42]	1.26 [1.10-1.44]		1.24 [1.09-1.42]	643	635	577	NA	569
F53	1.35 [1.19-1.53]	1.34 [1.18-1.52]		0.96 [0.73-1.26]	1.20 [1.06-1.36]	1.24 [1.08-1.41]	1.14 [0.99-1.30]		0.95 [0.71-1.26]	1.15 [1.01-1.31]	619	613	NA	135	586
F60	1.34 [1.27-1.42]	1.39 [1.31-1.47]	1.59 [1.46-1.72]	1.02 [0.92-1.14]	1.21 [1.14-1.27]	1.15 [1.09-1.22]	1.05 [0.99-1.11]	1.23 [1.13-1.35]	1.02 [0.91-1.14]	1.13 [1.07-1.20]	3,390	3,274	1,412	915	3,113
F61	1.13 [0.85-1.51]	1.17 [0.88-1.56]				0.99 [0.73-1.35]	0.97 [0.71-1.32]				112	111	NA	NA	NA
F69	1.47 [1.22-1.77]	1.62 [1.31-1.99]	1.81 [1.39-2.36]		1.39 [1.15-1.68]	1.27 [1.05-1.55]	1.27 [1.01-1.58]	1.49 [1.13-1.97]		1.29 [1.06-1.57]	302	234	147	NA	275
F70	1.72 [1.51-1.97]	1.87 [1.62-2.16]	2.17 [1.80-2.61]	1.19 [0.94-1.51]	1.50 [1.30-1.72]	1.54 [1.34-1.76]	1.65 [1.42-1.92]	1.81 [1.49-2.20]	1.14 [0.89-1.46]	1.39 [1.20-1.61]	625	518	317	183	533
F71	2.16 [1.76-2.65]	2.40 [1.92-3.00]	2.90 [2.15-3.91]		1.73 [1.39-2.15]	1.93 [1.56-2.39]	2.23 [1.77-2.82]	2.62 [1.92-3.56]		1.67 [1.34-2.08]	280	231	137	NA	237
F72	1.67 [1.43-1.94]	2.15 [1.80-2.57]	2.79 [2.21-3.52]	1.03 [0.82-1.29]	1.42 [1.21-1.66]	1.51 [1.29-1.76]	2.09 [1.74-2.52]	2.58 [2.02-3.29]	1.01 [0.80-1.29]	1.34 [1.14-1.57]	485	355	219	197	439
F79	1.56 [1.47-1.66]	1.89 [1.76-2.04]	2.13 [1.94-2.33]	1.06 [0.96-1.16]	1.38 [1.30-1.48]	1.44 [1.35-1.54]	1.74 [1.61-1.88]	1.93 [1.76-2.12]	1.03 [0.93-1.13]	1.31 [1.23-1.40]	2,891	2,009	1,368	1,151	2,559
F80	1.22 [1.15-1.29]	1.38 [1.22-1.56]	1.41 [1.24-1.61]	1.16 [1.09-1.24]	1.14 [1.08-1.22]	1.16 [1.10-1.24]	1.29 [1.13-1.46]	1.31 [1.14-1.49]	1.11 [1.04-1.19]	1.12 [1.05-1.19]	2,868	666	603	2,234	2,631
F81	1.56 [1.50-1.61]	1.92 [1.83-2.00]	2.15 [2.03-2.27]	1.13 [1.07-1.19]	1.36 [1.31-1.42]	1.44 [1.39-1.50]	1.79 [1.71-1.88]	1.99 [1.88-2.11]	1.08 [1.03-1.14]	1.29 [1.24-1.35]	8,353	5,304	3,443	3,825	6,839
F82	1.19 [1.02-1.39]			1.12 [0.95-1.33]	1.06 [0.90-1.25]	1.11 [0.95-1.31]			1.07 [0.90-1.26]	1.02 [0.86-1.20]	403	NA	NA	372	363
F83	0.95 [0.80-1.13]			0.91 [0.76-1.09]	0.88 [0.74-1.06]	0.89 [0.74-1.06]			0.88 [0.73-1.05]	0.84 [0.70-1.01]	326	NA	NA	311	311
F84	1.32 [1.28-1.38]	1.54 [1.44-1.65]	2.15 [1.90-2.44]	1.22 [1.17-1.27]	1.23 [1.18-1.28]	1.18 [1.14-1.23]	1.34 [1.24-1.44]	1.73 [1.51-1.97]	1.15 [1.11-1.20]	1.14 [1.09-1.19]	7,896	2,095	695	6,653	6,860
F89	1.02 [0.95-1.10]	1.53 [1.20-1.96]		0.97 [0.90-1.05]	0.94 [0.87-1.01]	0.94 [0.87-1.02]	1.33 [1.02-1.73]		0.92 [0.85-1.00]	0.88 [0.82-0.96]	1,669	166	NA	1,560	1,570
F90	1.22 [1.15-1.28]	1.23 [1.12-1.35]	1.75 [1.34-2.29]	1.16 [1.09-1.22]	1.14 [1.08-1.21]	1.12 [1.06-1.19]	1.09 [0.99-1.21]	1.26 [0.94-1.68]	1.12 [1.06-1.19]	1.11 [1.04-1.17]	3,883	1,020	139	3,444	3,393
F91	1.27 [1.13-1.42]	1.19 [0.93-1.52]	1.09 [0.81-1.47]	1.30 [1.15-1.48]	1.16 [1.03-1.31]	1.19 [1.06-1.34]	1.08 [0.84-1.39]	1.01 [0.74-1.37]	1.24 [1.09-1.41]	1.12 [0.99-1.26]	820	160	106	694	709
F94	1.22 [0.94-1.58]			1.19 [0.90-1.56]	1.07 [0.81-1.43]	1.15 [0.88-1.51]			1.14 [0.86-1.51]	1.04 [0.77-1.39]	161	NA	NA	148	136
F95	1.38 [1.18-1.61]	1.52 [1.16-1.98]		1.30 [1.10-1.55]	1.27 [1.08-1.50]	1.23 [1.05-1.45]	1.23 [0.93-1.64]		1.22 [1.02-1.45]	1.20 [1.01-1.42]	474	137	NA	380	414
F98	1.32 [1.15-1.52]	1.26 [0.95-1.66]		1.30 [1.12-1.51]	1.22 [1.05-1.41]	1.22 [1.06-1.41]	1.08 [0.81-1.44]		1.25 [1.08-1.46]	1.15 [0.99-1.34]	567	128	NA	495	513
F99	1.24 [1.14-1.36]	1.27 [1.16-1.39]	1.30 [1.16-1.46]	1.10 [0.91-1.32]	1.12 [1.02-1.23]	1.16 [1.06-1.27]	1.08 [0.99-1.19]	1.12 [0.99-1.26]	1.09 [0.90-1.33]	1.09 [0.99-1.19]	1,303	1,179	715	299	1,144
Diseases of the nervous system (G00-G99)															
G00	1.12 [0.95-1.31]	1.20 [1.00-1.44]	1.25 [1.01-1.55]	0.92 [0.69-1.24]	1.02 [0.85-1.22]	1.07 [0.90-1.26]	1.12 [0.92-1.35]	1.20 [0.96-1.49]	0.90 [0.66-1.21]	0.98 [0.81-1.18]	351	275	200	108	283
G01	1.11 [0.89-1.39]			1.09 [0.82-1.45]	1.10 [0.85-1.41]	1.06 [0.84-1.33]			1.11 [0.83-1.49]	1.08 [0.84-1.39]	193	NA	NA	116	157
G02	1.08 [0.85-1.39]	1.19 [0.90-1.56]			1.05 [0.81-1.37]	0.98 [0.76-1.27]	1.04 [0.78-1.39]			1.00 [0.76-1.30]	165	130	NA	NA	143
G03	1.24 [1.07-1.43]	1.17 [0.98-1.39]	1.13 [0.92-1.40]	1.32 [1.06-1.65]	1.05 [0.90-1.23]	1.15 [1.00-1.34]	1.03 [0.86-1.24]	1.00 [0.80-1.24]	1.27 [1.01-1.60]	1.02 [0.87-1.19]	464	307	200	205	396
G04	1.25 [1.13-1.38]	1.28 [1.15-1.43]	1.23 [1.09-1.39]	1.10 [0.91-1.33]	1.15 [1.04-1.28]	1.19 [1.08-1.32]	1.17 [1.04-1.30]	1.12 [0.99-1.27]	1.08 [0.89-1.31]	1.12 [1.01-1.24]	1,050	822	674	279	908
G05	1.42 [1.17-1.71]	1.55 [1.26-1.91]	1.60 [1.28-2.00]		1.44 [1.18-1.77]	1.37 [1.14-1.66]	1.46 [1.18-1.81]	1.48 [1.18-1.86]		1.38 [1.12-1.70]	290	232	204	NA	251
G06	1.25 [1.08-1.45]	1.20 [1.02-1.41]	1.18 [1.00-1.41]		1.20 [1.03-1.41]	1.20 [1.03-1.40]	1.14 [0.97-1.34]	1.11 [0.93-1.33]		1.20 [1.02-1.41]	446	365	319	NA	385
G08	1.16 [0.95-1.42]	1.32 [1.06-1.65]	1.14 [0.86-1.51]		1.29 [1.04-1.60]	1.12 [0.92-1.38]	1.26 [1.00-1.58]	1.06 [0.79-1.42]		1.29 [1.03-1.61]	239	199	116	NA	206
G09	1.36 [1.14-1.63]	1.38 [1.12-1.70]	1.24 [0.98-1.56]		1.17 [0.98-1.41]	1.28 [1.06-1.54]	1.27 [1.03-1.57]	1.11 [0.87-1.41]		1.14 [0.95-1.38]	311	232	176	NA	280
G10	1.40 [1.07-1.84]	1.33 [1.02-1.74]	1.26 [0.94-1.67]		1.25 [0.92-1.69]	1.42 [1.08-1.87]	1.39 [1.05-1.83]	1.29 [0.97-1.73]		1.27 [0.93-1.73]	135	132	113	NA	101
G11	1.17 [1.01-1.36]	1.25 [1.07-1.46]	1.20 [1.02-1.43]		1.15 [0.99-1.34]	1.13 [0.97-1.31]	1.16 [0.99-1.36]	1.10 [0.93-1.31]		1.14 [0.97-1.33]	454	380	328	NA	400
G12	1.08 [0.97-1.20]	1.12 [1.00-1.24]	1.10 [0.99-1.22]		1.06 [0.95-1.19]	1.04 [0.93-1.15]	1.07 [0.96-1.20]	1.06 [0.95-1.18]		1.05 [0.94-1.18]	857	824	796	NA	732
G20	1.06 [1.02-1.09]	1.05 [1.01-1.09]	1.06 [1.02-1.10]		1.03 [0.99-1.07]	1.05 [1.01-1.09]	1.02 [0.99-1.06]	1.03 [0.99-1.07]		1.03 [0.99-1.07]	7,432	7,435	7,409	NA	6,407
G21	1.34 [1.19-1.50]	1.27 [1.13-1.43]	1.34 [1.19-1.51]		1.22 [1.08-1.38]	1.31 [1.17-1.48]	1.20 [1.06-1.35]	1.28 [1.13-1.44]		1.24 [1.10-1.40]	774	773	752	NA	736
G23	1.17 [1.00-1.36]	1.12 [0.96-1.30]	1.08 [0.93-1.27]		1.02 [0.87-1.19]	1.14 [0.97-1.33]	1.07 [0.92-1.25]	1.03 [0.88-1.21]		1.01 [0.86-1.18]	428	418	405	NA	393
G24	1.14 [1.03-1.25]	1.20 [1.08-1.34]	1.21 [1.08-1.37]	0.82 [0.67-1.00]	1.04 [0.94-1.15]	1.09 [0.98-1.20]	1.10 [0.98-1.23]	1.11 [0.99-1.26]	0.79 [0.64-0.96]	1.02 [0.92-1.13]	1,028	821	698	250	933
G25	1.28 [1.23-1.34]	1.30 [1.24-1.36]	1.28 [1.22-1.35]	1.07 [0.91-1.27]	1.20 [1.15-1.26]	1.16 [1.11-1.22]	1.12 [1.07-1.17]	1.11 [1.06-1.17]	1.04 [0.87-1.24]	1.13 [1.07-1.18]	4,923	4,667	4,349	353	4,613
G30	1.07 [1.04-1.10]	1.07 [1.04-1.10]	1.08 [1.05-1.11]		1.06 [1.03-1.09]	1.06 [1.03-1.09]	1.05 [1.02-1.08]	1.06 [1.03-1.09]		1.05 [1.02-1.09]	13,635	13,626	13,622	NA	12,389
G31	1.17 [1.13-1.21]	1.18 [1.14-1.22]	1.17 [1.13-1.20]	1.04 [0.78-1.37]	1.13 [1.10-1.17]	1.14 [1.10-1.18]	1.12 [1.08-1.16]	1.10 [1.07-1.14]	1.01 [0.76-1.35]	1.13 [1.09-1.17]	10,278	10,176	10,013	126	9,507
G32	1.39 [1.10-1.76]	1.29 [1.02-1.63]	1.47 [1.15-1.89]		1.29 [1.01-1.64]	1.30 [1.02-1.66]	1.12 [0.88-1.43]	1.30 [1.01-1.68]		1.29 [1.01-1.65]	174	171	160	NA	161
G35	1.23 [1.15-1.31]	1.22 [1.15-1.30]	1.23 [1.14-1.32]	1.14 [0.85-1.53]	1.14 [1.06-1.22]	1.16 [1.08-1.24]	1.08 [1.01-1.15]	1.09 [1.01-1.18]	1.15 [0.85-1.55]	1.12 [1.04-1.21]	2,212	2,199	1,684	125	1,677
G37	1.28 [1.14-1.45]	1.30 [1.14-1.47]	1.36 [1.17-1.59]	1.04 [0.77-1.40]	1.22 [1.07-1.40]	1.22 [1.07-1.38]	1.16 [1.02-1.33]	1.24 [1.06-1.46]	1.02 [0.75-1.39]	1.19 [1.04-1.36]	646	580	389	113	544
G40	1.31 [1.28-1.34]	1.36 [1.32-1.40]	1.38 [1.34-1.42]	1.08 [1.02-1.13]	1.19 [1.16-1.22]	1.21 [1.18-1.24]	1.19 [1.15-1.22]	1.20 [1.16-1.23]	1.06 [1.00-1.11]	1.14 [1.11-1.17]	17,275	14,171	11,140	4,096	14,157
G41	1.20 [1.10-1.31]	1.33 [1.20-1.48]	1.29 [1.15-1.46]	0.96 [0.84-1.11]	1.09 [1.00-1.20]	1.13 [1.04-1.24]	1.20 [1.07-1.34]	1.15 [1.02-1.30]	0.95 [0.83-1.10]	1.07 [0.98-1.18]	1,276	866	693	479	1,177
G43	1.32 [1.28-1.35]	1.32 [1.29-1.36]	1.33 [1.29-1.38]	1.20 [1.14-1.26]	1.21 [1.18-1.25]	1.16 [1.13-1.19]	1.08 [1.05-1.11]	1.10 [1.06-1.14]	1.13 [1.07-1.20]	1.12 [1.09-1.15]	16,417	14,623	8,600	3,948	14,478
G44	1.33 [1.24-1.42]	1.31 [1.23-1.40]	1.37 [1.26-1.49]	1.22 [1.05-1.42]	1.23 [1.15-1.32]	1.20 [1.12-1.28]	1.12 [1.04-1.20]	1.15 [1.06-1.26]	1.19 [1.02-1.39]	1.15 [1.07-1.23]	2,317	2,109	1,353	470	2,084
G45	1.18 [1.15-1.22]	1.18 [1.15-1.22]	1.18 [1.14-1.22]		1.12 [1.08-1.15]	1.14 [1.10-1.17]	1.10 [1.07-1.14]	1.10 [1.06-1.13]		1.09 [1.06-1.13]	9,727	9,696	9,541	NA	8,585
G46	1.22 [1.07-1.38]	1.18 [1.04-1.34]	1.19 [1.05-1.36]		1.15 [1.01-1.30]	1.19 [1.04-1.35]	1.12 [0.98-1.27]	1.12 [0.98-1.28]		1.13 [0.99-1.29]	647	644	635	NA	596
G47	1.48 [1.45-1.51]	1.58 [1.54-1.62]	1.58 [1.54-1.63]	1.31 [1.27-1.36]	1.38 [1.35-1.41]	1.32 [1.30-1.35]	1.28 [1.24-1.31]	1.27 [1.24-1.31]	1.25 [1.21-1.30]	1.27 [1.24-1.30]	23,676	15,345	13,168	8,935	21,280
G50	1.42 [1.32-1.53]	1.43 [1.33-1.55]	1.44 [1.33-1.56]		1.32 [1.22-1.42]	1.29 [1.20-1.39]	1.22 [1.13-1.32]	1.24 [1.15-1.35]		1.22 [1.13-1.32]	1,837	1,821	1,646	NA	1,651
G51	1.21 [1.15-1.28]	1.24 [1.17-1.31]	1.21 [1.14-1.29]	1.09 [0.95-1.24]	1.15 [1.09-1.21]	1.15 [1.09-1.21]	1.13 [1.06-1.19]	1.10 [1.04-1.17]	1.09 [0.95-1.24]	1.11 [1.05-1.17]	3,683	3,203	2,781	595	3,191
G52	1.23 [1.02-1.49]	1.32 [1.08-1.60]	1.28 [1.04-1.57]		1.26 [1.03-1.53]	1.17 [0.97-1.42]	1.18 [0.97-1.45]	1.15 [0.93-1.43]		1.20 [0.98-1.46]	276	259	230	NA	254
G53	1.33 [1.17-1.52]	1.32 [1.15-1.51]	1.35 [1.18-1.55]		1.26 [1.10-1.44]	1.26 [1.10-1.44]	1.21 [1.06-1.39]	1.25 [1.09-1.44]		1.21 [1.05-1.39]	615	595	570	NA	564
G54	1.33 [1.21-1.46]	1.29 [1.17-1.42]	1.35 [1.22-1.50]		1.24 [1.13-1.37]	1.21 [1.10-1.33]	1.10 [1.00-1.22]	1.16 [1.04-1.29]		1.18 [1.07-1.30]	1,109	1,087	912	NA	1,017
G55	1.27 [1.24-1.31]	1.27 [1.23-1.31]	1.28 [1.24-1.32]	1.12 [0.96-1.30]	1.18 [1.14-1.21]	1.17 [1.14-1.21]	1.11 [1.07-1.14]	1.11 [1.08-1.15]	1.06 [0.90-1.23]	1.11 [1.08-1.15]	11,907	11,866	9,868	458	10,751
G56	1.35 [1.32-1.38]	1.34 [1.31-1.37]	1.33 [1.30-1.36]	1.23 [1.06-1.44]	1.24 [1.21-1.27]	1.24 [1.21-1.27]	1.18 [1.15-1.21]	1.17 [1.14-1.20]	1.16 [0.98-1.36]	1.16 [1.13-1.19]	18,507	18,382	16,157	428	15,834
G57	1.39 [1.30-1.48]	1.40 [1.31-1.50]	1.35 [1.26-1.45]		1.25 [1.17-1.34]	1.28 [1.19-1.37]	1.25 [1.17-1.34]	1.19 [1.11-1.28]		1.17 [1.09-1.26]	2,204	2,164	1,841	NA	1,944
G58	1.32 [1.20-1.45]	1.40 [1.27-1.54]	1.41 [1.27-1.56]		1.24 [1.12-1.36]	1.20 [1.09-1.32]	1.21 [1.09-1.33]	1.22 [1.10-1.36]		1.16 [1.05-1.28]	1,125	1,087	932	NA	1,032
G59	1.49 [1.34-1.65]	1.47 [1.33-1.64]	1.49 [1.34-1.66]		1.27 [1.14-1.41]	1.31 [1.18-1.47]	1.18 [1.05-1.32]	1.18 [1.05-1.32]		1.20 [1.07-1.33]	888	886	856	NA	843
G60	1.28 [1.11-1.46]	1.26 [1.10-1.46]	1.26 [1.09-1.47]		1.17 [1.01-1.35]	1.17 [1.02-1.34]	1.12 [0.97-1.30]	1.11 [0.95-1.30]		1.07 [0.93-1.24]	529	483	419	NA	473
G61	1.25 [1.11-1.41]	1.26 [1.11-1.43]	1.24 [1.08-1.43]		1.14 [0.99-1.30]	1.21 [1.07-1.37]	1.19 [1.05-1.36]	1.17 [1.02-1.35]		1.10 [0.96-1.26]	627	574	494	NA	517
G62	1.35 [1.30-1.40]	1.36 [1.30-1.41]	1.35 [1.30-1.40]	0.97 [0.78-1.22]	1.23 [1.19-1.28]	1.23 [1.18-1.28]	1.16 [1.12-1.21]	1.15 [1.11-1.20]	0.94 [0.74-1.19]	1.17 [1.12-1.22]	6,954	6,849	6,481	198	6,516
G63	1.45 [1.38-1.53]	1.44 [1.37-1.52]	1.46 [1.38-1.54]		1.32 [1.26-1.39]	1.30 [1.23-1.37]	1.16 [1.10-1.23]	1.17 [1.11-1.24]		1.24 [1.18-1.31]	3,841	3,821	3,700	NA	3,718
G70	1.12 [0.99-1.28]	1.17 [1.02-1.33]	1.17 [1.02-1.35]		1.14 [0.99-1.30]	1.07 [0.94-1.22]	1.09 [0.95-1.25]	1.09 [0.94-1.26]		1.10 [0.95-1.26]	582	535	478	NA	511
G71	1.18 [1.04-1.35]	1.22 [1.04-1.43]	1.14 [0.95-1.36]	1.00 [0.81-1.25]	1.04 [0.90-1.20]	1.15 [1.00-1.31]	1.16 [0.98-1.36]	1.08 [0.89-1.30]	1.01 [0.81-1.26]	1.03 [0.89-1.19]	555	387	271	201	459
G72	1.41 [1.26-1.58]	1.42 [1.27-1.60]	1.41 [1.25-1.59]		1.28 [1.14-1.43]	1.32 [1.18-1.48]	1.28 [1.13-1.44]	1.29 [1.14-1.46]		1.23 [1.09-1.38]	815	748	682	NA	743
G80	1.01 [0.94-1.09]	1.39 [1.25-1.55]	1.66 [1.46-1.90]	0.79 [0.72-0.87]	0.92 [0.85-1.00]	0.96 [0.89-1.04]	1.33 [1.19-1.49]	1.59 [1.38-1.82]	0.77 [0.70-0.85]	0.90 [0.83-0.97]	1,750	801	552	1,019	1,431
G81	1.17 [1.14-1.20]	1.19 [1.15-1.23]	1.17 [1.14-1.21]	0.98 [0.86-1.13]	1.12 [1.08-1.15]	1.14 [1.11-1.18]	1.12 [1.08-1.15]	1.10 [1.07-1.13]	0.98 [0.85-1.12]	1.11 [1.08-1.15]	11,632	11,192	10,789	541	10,542
G82	1.15 [1.05-1.25]	1.22 [1.11-1.34]	1.21 [1.10-1.34]	0.75 [0.61-0.93]	1.04 [0.95-1.14]	1.10 [1.01-1.20]	1.14 [1.04-1.25]	1.13 [1.03-1.26]	0.73 [0.59-0.91]	1.03 [0.94-1.13]	1,261	1,119	951	198	1,098
G83	1.21 [1.12-1.30]	1.20 [1.12-1.30]	1.23 [1.13-1.33]	0.97 [0.76-1.24]	1.09 [1.01-1.18]	1.15 [1.06-1.23]	1.09 [1.01-1.17]	1.12 [1.03-1.21]	0.95 [0.74-1.22]	1.06 [0.98-1.15]	1,842	1,735	1,491	165	1,652
G90	1.26 [1.15-1.40]	1.23 [1.11-1.36]	1.22 [1.08-1.36]	1.01 [0.77-1.33]	1.13 [1.02-1.25]	1.17 [1.05-1.29]	1.07 [0.97-1.19]	1.08 [0.96-1.22]	0.94 [0.71-1.25]	1.06 [0.95-1.18]	1,018	945	747	143	926
G91	1.17 [1.10-1.25]	1.21 [1.13-1.30]	1.17 [1.09-1.26]	0.97 [0.82-1.14]	1.14 [1.06-1.22]	1.15 [1.08-1.23]	1.16 [1.08-1.25]	1.13 [1.04-1.21]	0.97 [0.82-1.14]	1.14 [1.06-1.22]	2,341	2,035	1,777	376	2,007
G93	1.26 [1.22-1.30]	1.27 [1.23-1.31]	1.25 [1.21-1.30]	1.13 [1.05-1.22]	1.17 [1.14-1.21]	1.17 [1.13-1.21]	1.12 [1.08-1.16]	1.11 [1.07-1.16]	1.08 [1.01-1.17]	1.12 [1.08-1.16]	10,133	8,873	6,837	2,055	9,031
G94	1.18 [1.00-1.39]	1.25 [1.04-1.50]	1.19 [0.98-1.45]		1.11 [0.93-1.32]	1.17 [0.99-1.38]	1.22 [1.01-1.47]	1.16 [0.95-1.42]		1.13 [0.94-1.36]	361	272	245	NA	295
G95	1.19 [1.12-1.27]	1.22 [1.14-1.29]	1.22 [1.14-1.31]	1.08 [0.87-1.35]	1.11 [1.04-1.19]	1.14 [1.07-1.21]	1.13 [1.06-1.21]	1.14 [1.06-1.22]	1.02 [0.82-1.28]	1.09 [1.02-1.17]	2,508	2,358	2,173	207	2,215
G96	1.30 [1.17-1.44]	1.35 [1.20-1.50]	1.37 [1.21-1.55]	1.03 [0.79-1.33]	1.20 [1.08-1.34]	1.20 [1.07-1.34]	1.18 [1.05-1.32]	1.21 [1.06-1.37]	0.97 [0.74-1.26]	1.15 [1.03-1.29]	875	780	621	145	781
G97	1.22 [1.09-1.36]	1.26 [1.12-1.42]	1.24 [1.06-1.45]	1.12 [0.91-1.39]	1.11 [0.99-1.24]	1.11 [0.99-1.24]	1.09 [0.97-1.23]	1.09 [0.93-1.28]	1.09 [0.88-1.35]	1.05 [0.94-1.19]	818	695	378	237	726
G98	1.49 [1.25-1.79]	1.46 [1.20-1.76]	1.53 [1.24-1.88]		1.41 [1.18-1.69]	1.33 [1.10-1.60]	1.18 [0.97-1.45]	1.22 [0.98-1.52]		1.29 [1.07-1.55]	323	279	243	NA	314
G99	1.26 [1.20-1.33]	1.28 [1.21-1.34]	1.26 [1.19-1.32]		1.19 [1.13-1.26]	1.18 [1.12-1.24]	1.13 [1.08-1.19]	1.12 [1.06-1.18]		1.15 [1.09-1.21]	3,910	3,886	3,580	NA	3,567
Diseases of the eye and adnexa (H00-H59)															
H00	1.25 [1.19-1.31]	1.27 [1.19-1.35]	1.25 [1.15-1.34]	1.20 [1.11-1.29]	1.17 [1.11-1.24]	1.20 [1.14-1.27]	1.22 [1.14-1.29]	1.18 [1.10-1.28]	1.17 [1.09-1.26]	1.14 [1.08-1.21]	3,999	2,448	1,574	1,871	2,955
H01	1.42 [1.33-1.51]	1.39 [1.30-1.48]	1.40 [1.31-1.50]	1.49 [1.23-1.81]	1.32 [1.24-1.41]	1.33 [1.25-1.42]	1.29 [1.21-1.38]	1.31 [1.22-1.40]	1.39 [1.14-1.70]	1.26 [1.18-1.34]	2,757	2,521	2,368	291	2,435
H02	1.31 [1.27-1.34]	1.32 [1.28-1.35]	1.31 [1.28-1.35]	1.22 [1.10-1.35]	1.22 [1.19-1.26]	1.24 [1.21-1.28]	1.22 [1.19-1.26]	1.22 [1.19-1.26]	1.18 [1.06-1.31]	1.17 [1.14-1.21]	13,523	12,689	11,932	1,036	11,492
H03	1.61 [1.30-1.98]	1.41 [1.07-1.84]	1.30 [0.96-1.76]	1.74 [1.27-2.38]	1.34 [1.06-1.68]	1.50 [1.20-1.86]	1.25 [0.95-1.65]	1.18 [0.87-1.61]	1.60 [1.16-2.21]	1.23 [0.97-1.57]	247	137	107	122	202
H04	1.40 [1.35-1.45]	1.44 [1.38-1.50]	1.44 [1.39-1.51]	1.20 [1.09-1.32]	1.31 [1.26-1.37]	1.30 [1.25-1.35]	1.32 [1.27-1.37]	1.32 [1.26-1.37]	1.15 [1.04-1.26]	1.24 [1.19-1.29]	7,312	6,384	6,029	1,027	6,318
H05	1.40 [1.32-1.49]	1.43 [1.30-1.57]	1.47 [1.33-1.63]	1.33 [1.23-1.44]	1.32 [1.24-1.41]	1.36 [1.28-1.44]	1.31 [1.19-1.45]	1.35 [1.21-1.50]	1.29 [1.19-1.39]	1.29 [1.21-1.38]	2,893	1,170	926	1,807	2,409
H06	1.49 [1.23-1.79]	1.45 [1.21-1.75]	1.47 [1.20-1.80]		1.37 [1.13-1.67]	1.43 [1.18-1.73]	1.28 [1.05-1.55]	1.36 [1.11-1.68]		1.34 [1.10-1.63]	292	288	242	NA	250
H10	1.43 [1.36-1.50]	1.43 [1.34-1.52]	1.35 [1.26-1.45]	1.42 [1.32-1.54]	1.35 [1.28-1.42]	1.34 [1.27-1.41]	1.29 [1.21-1.38]	1.24 [1.16-1.33]	1.32 [1.22-1.43]	1.28 [1.22-1.35]	4,406	2,770	2,435	1,760	3,999
H11	1.24 [1.16-1.31]	1.24 [1.16-1.32]	1.27 [1.19-1.36]	1.10 [0.91-1.32]	1.17 [1.10-1.25]	1.19 [1.12-1.26]	1.18 [1.11-1.26]	1.21 [1.13-1.29]	1.07 [0.88-1.30]	1.15 [1.08-1.23]	2,738	2,541	2,263	289	2,305
H13	1.47 [1.20-1.80]	1.59 [1.24-2.03]	1.78 [1.35-2.34]		1.36 [1.10-1.69]	1.37 [1.11-1.69]	1.50 [1.16-1.93]	1.63 [1.23-2.16]		1.29 [1.03-1.61]	252	172	143	NA	216
H15	1.41 [1.17-1.71]	1.45 [1.19-1.77]	1.30 [1.04-1.62]		1.32 [1.08-1.62]	1.31 [1.08-1.60]	1.28 [1.05-1.58]	1.18 [0.94-1.48]		1.24 [1.01-1.53]	277	260	208	NA	241
H16	1.71 [1.57-1.86]	1.64 [1.50-1.80]	1.58 [1.44-1.75]	1.92 [1.54-2.41]	1.59 [1.45-1.74]	1.60 [1.47-1.75]	1.52 [1.39-1.67]	1.48 [1.34-1.64]	1.75 [1.39-2.21]	1.53 [1.39-1.68]	1,515	1,344	1,111	243	1,297
H17	1.41 [1.26-1.59]	1.40 [1.24-1.57]	1.37 [1.21-1.56]		1.39 [1.23-1.57]	1.34 [1.19-1.51]	1.30 [1.15-1.48]	1.28 [1.12-1.46]		1.33 [1.18-1.50]	774	722	635	NA	694
H18	1.38 [1.32-1.44]	1.36 [1.30-1.43]	1.24 [1.18-1.31]	1.91 [1.67-2.18]	1.29 [1.23-1.36]	1.31 [1.25-1.37]	1.27 [1.22-1.34]	1.19 [1.13-1.25]	1.70 [1.48-1.95]	1.24 [1.18-1.30]	5,208	4,964	3,982	735	4,460
H19	1.81 [1.53-2.14]	1.85 [1.55-2.19]	1.77 [1.47-2.14]		1.67 [1.40-1.99]	1.60 [1.34-1.90]	1.59 [1.33-1.90]	1.54 [1.27-1.88]		1.51 [1.26-1.81]	401	366	307	NA	339
H20	1.43 [1.32-1.56]	1.38 [1.26-1.50]	1.38 [1.25-1.51]	1.40 [1.12-1.74]	1.38 [1.26-1.50]	1.38 [1.27-1.50]	1.29 [1.18-1.41]	1.30 [1.18-1.43]	1.35 [1.08-1.70]	1.34 [1.23-1.47]	1,446	1,262	1,077	233	1,273
H21	1.18 [1.10-1.27]	1.18 [1.09-1.26]	1.15 [1.07-1.24]		1.15 [1.06-1.24]	1.14 [1.06-1.23]	1.12 [1.04-1.21]	1.09 [1.01-1.18]		1.12 [1.04-1.21]	1,915	1,866	1,792	NA	1,746
H22	1.46 [1.07-1.99]	1.45 [1.06-1.98]			1.36 [0.99-1.87]	1.40 [1.02-1.91]	1.28 [0.92-1.77]			1.33 [0.96-1.84]	112	103	NA	NA	102
H25	1.17 [1.15-1.19]	1.17 [1.16-1.19]	1.18 [1.17-1.20]		1.12 [1.11-1.14]	1.13 [1.12-1.15]	1.12 [1.10-1.13]	1.13 [1.11-1.14]		1.10 [1.08-1.12]	46,074	46,065	45,897	NA	39,385
H26	1.21 [1.19-1.23]	1.21 [1.19-1.22]	1.21 [1.19-1.22]	1.60 [1.36-1.87]	1.16 [1.14-1.17]	1.17 [1.15-1.18]	1.14 [1.13-1.16]	1.14 [1.13-1.16]	1.56 [1.32-1.83]	1.13 [1.11-1.14]	67,194	66,929	66,284	444	56,865
H27	1.19 [1.08-1.32]	1.22 [1.09-1.35]	1.19 [1.07-1.33]		1.15 [1.03-1.28]	1.16 [1.05-1.29]	1.16 [1.04-1.29]	1.14 [1.01-1.27]		1.13 [1.01-1.27]	935	877	810	NA	824
H28	1.32 [1.12-1.55]	1.35 [1.14-1.59]	1.28 [1.08-1.51]		1.27 [1.07-1.51]	1.24 [1.05-1.47]	1.22 [1.03-1.44]	1.13 [0.95-1.34]		1.24 [1.04-1.48]	365	361	350	NA	329
H30	1.33 [1.00-1.76]	1.29 [0.96-1.74]			1.34 [1.00-1.81]	1.26 [0.94-1.68]	1.17 [0.87-1.59]			1.30 [0.96-1.76]	125	109	NA	NA	109
H31	1.11 [1.01-1.22]	1.13 [1.02-1.24]	1.10 [1.00-1.22]		1.05 [0.95-1.16]	1.09 [0.99-1.20]	1.09 [0.98-1.20]	1.06 [0.96-1.17]		1.04 [0.94-1.15]	1,062	1,040	999	NA	953
H33	1.05 [1.00-1.10]	1.08 [1.02-1.13]	1.04 [0.99-1.10]	1.10 [0.89-1.35]	1.03 [0.98-1.09]	1.03 [0.98-1.08]	1.05 [1.00-1.11]	1.02 [0.97-1.08]	1.08 [0.87-1.34]	1.03 [0.97-1.09]	3,916	3,790	3,415	243	3,116
H34	1.17 [1.10-1.24]	1.17 [1.10-1.24]	1.15 [1.08-1.22]		1.11 [1.04-1.18]	1.14 [1.07-1.21]	1.11 [1.04-1.18]	1.09 [1.02-1.16]		1.09 [1.02-1.17]	2,582	2,577	2,534	NA	2,300
H35	1.17 [1.14-1.19]	1.17 [1.15-1.20]	1.16 [1.14-1.19]	1.16 [0.96-1.39]	1.12 [1.10-1.15]	1.13 [1.11-1.16]	1.12 [1.10-1.15]	1.11 [1.09-1.13]	1.14 [0.94-1.38]	1.10 [1.08-1.13]	23,935	23,741	23,343	308	21,152
H36	1.25 [1.21-1.29]	1.25 [1.21-1.29]	1.24 [1.20-1.28]	1.09 [0.81-1.48]	1.17 [1.13-1.21]	1.18 [1.14-1.22]	1.09 [1.06-1.13]	1.08 [1.05-1.12]	1.14 [0.83-1.57]	1.15 [1.11-1.19]	9,878	9,864	9,452	111	9,023
H40	1.18 [1.15-1.20]	1.18 [1.15-1.20]	1.18 [1.15-1.20]	1.29 [1.00-1.67]	1.13 [1.11-1.16]	1.14 [1.12-1.17]	1.13 [1.11-1.16]	1.13 [1.11-1.16]	1.26 [0.97-1.64]	1.11 [1.08-1.14]	21,887	21,771	21,555	166	18,784
H43	1.13 [1.07-1.18]	1.13 [1.07-1.18]	1.14 [1.09-1.20]		1.08 [1.03-1.13]	1.08 [1.03-1.14]	1.07 [1.01-1.12]	1.08 [1.03-1.13]		1.06 [1.01-1.11]	4,302	4,261	4,076	NA	3,808
H44	1.30 [1.20-1.40]	1.32 [1.22-1.43]	1.29 [1.19-1.41]	1.25 [0.95-1.64]	1.24 [1.14-1.35]	1.24 [1.14-1.34]	1.25 [1.15-1.36]	1.22 [1.12-1.33]	1.14 [0.87-1.51]	1.19 [1.09-1.30]	1,563	1,446	1,338	145	1,383
H46	1.37 [1.19-1.58]	1.41 [1.22-1.63]	1.33 [1.12-1.58]	1.43 [1.04-1.97]	1.22 [1.05-1.42]	1.33 [1.15-1.54]	1.27 [1.09-1.48]	1.20 [1.01-1.44]	1.45 [1.04-2.02]	1.18 [1.01-1.38]	513	464	327	108	429
H47	1.18 [1.10-1.26]	1.23 [1.13-1.33]	1.24 [1.13-1.36]	1.10 [0.97-1.24]	1.15 [1.07-1.24]	1.13 [1.06-1.22]	1.13 [1.04-1.23]	1.18 [1.08-1.30]	1.06 [0.93-1.20]	1.12 [1.04-1.21]	2,095	1,584	1,118	730	1,845
H49	1.06 [0.97-1.16]	1.10 [1.00-1.21]	1.13 [1.02-1.26]	0.97 [0.80-1.18]	1.02 [0.93-1.13]	1.01 [0.92-1.11]	1.02 [0.92-1.13]	1.05 [0.94-1.17]	0.92 [0.75-1.12]	1.00 [0.91-1.10]	1,185	965	849	258	1,024
H50	1.07 [1.03-1.12]	1.27 [1.18-1.37]	1.29 [1.18-1.40]	1.01 [0.96-1.05]	1.03 [0.99-1.08]	1.04 [1.00-1.08]	1.19 [1.11-1.28]	1.20 [1.10-1.31]	0.99 [0.94-1.04]	1.01 [0.97-1.06]	6,189	1,873	1,274	4,525	5,152
H51	1.20 [0.98-1.46]	1.25 [0.98-1.59]	1.11 [0.84-1.48]		1.05 [0.85-1.30]	1.12 [0.91-1.38]	1.12 [0.88-1.44]	1.01 [0.76-1.35]		1.00 [0.81-1.24]	240	170	124	NA	223
H52	1.13 [1.09-1.17]	1.13 [1.09-1.17]	1.12 [1.08-1.17]	1.13 [1.02-1.25]	1.09 [1.05-1.13]	1.10 [1.06-1.14]	1.09 [1.05-1.13]	1.08 [1.04-1.12]	1.10 [0.99-1.21]	1.07 [1.02-1.11]	7,679	6,786	6,490	1,014	6,731
H53	1.24 [1.21-1.27]	1.25 [1.22-1.28]	1.25 [1.21-1.29]	1.18 [1.12-1.24]	1.16 [1.13-1.19]	1.16 [1.13-1.18]	1.11 [1.08-1.14]	1.13 [1.09-1.16]	1.13 [1.07-1.19]	1.11 [1.08-1.14]	16,638	14,327	10,510	3,753	14,631
H54	1.20 [1.16-1.24]	1.22 [1.18-1.25]	1.21 [1.17-1.25]	1.00 [0.89-1.12]	1.14 [1.11-1.18]	1.16 [1.12-1.20]	1.14 [1.11-1.18]	1.14 [1.10-1.17]	0.96 [0.86-1.08]	1.12 [1.09-1.16]	11,421	10,807	10,273	773	10,552
H55	1.12 [1.03-1.23]	1.19 [1.07-1.32]	1.15 [1.03-1.29]	0.95 [0.80-1.11]	1.06 [0.97-1.17]	1.07 [0.98-1.17]	1.09 [0.98-1.21]	1.04 [0.93-1.17]	0.92 [0.78-1.09]	1.04 [0.94-1.14]	1,222	903	763	368	1,120
H57	1.27 [1.20-1.35]	1.27 [1.19-1.36]	1.28 [1.19-1.37]	1.28 [1.13-1.46]	1.20 [1.12-1.27]	1.19 [1.12-1.27]	1.15 [1.07-1.23]	1.16 [1.08-1.25]	1.22 [1.07-1.40]	1.14 [1.07-1.21]	2,834	2,314	2,013	631	2,526
H58	1.23 [1.07-1.42]	1.26 [1.09-1.46]	1.17 [1.01-1.36]		1.16 [1.00-1.35]	1.18 [1.02-1.36]	1.15 [1.00-1.34]	1.06 [0.91-1.23]		1.13 [0.98-1.32]	515	483	451	NA	455
H59	1.17 [1.05-1.30]	1.16 [1.05-1.30]	1.10 [0.98-1.22]		1.12 [1.00-1.25]	1.14 [1.03-1.27]	1.13 [1.01-1.26]	1.06 [0.95-1.18]		1.11 [0.99-1.25]	842	811	779	NA	758
Diseases of the ear and mastoid process (H60-H95)															
H60	1.60 [1.51-1.69]	1.71 [1.60-1.83]	1.85 [1.71-2.00]	1.32 [1.20-1.46]	1.48 [1.39-1.57]	1.50 [1.42-1.59]	1.54 [1.43-1.64]	1.67 [1.54-1.81]	1.28 [1.16-1.41]	1.42 [1.34-1.51]	3,336	2,433	1,757	1,163	2,866
H61	1.35 [1.29-1.40]	1.45 [1.37-1.54]	1.45 [1.36-1.55]	1.26 [1.19-1.33]	1.27 [1.22-1.33]	1.30 [1.24-1.35]	1.35 [1.27-1.43]	1.34 [1.26-1.43]	1.22 [1.15-1.29]	1.24 [1.18-1.30]	5,991	3,047	2,555	3,129	5,243
H65	1.21 [1.18-1.24]	1.35 [1.25-1.46]	1.35 [1.24-1.48]	1.20 [1.17-1.23]	1.17 [1.14-1.20]	1.17 [1.14-1.20]	1.22 [1.12-1.32]	1.24 [1.13-1.35]	1.16 [1.13-1.19]	1.14 [1.11-1.17]	16,730	1,582	1,177	15,291	13,784
H66	1.26 [1.22-1.31]	1.53 [1.43-1.64]	1.52 [1.40-1.65]	1.21 [1.16-1.25]	1.21 [1.16-1.25]	1.21 [1.17-1.26]	1.39 [1.29-1.49]	1.37 [1.26-1.50]	1.17 [1.13-1.22]	1.18 [1.13-1.22]	8,169	2,125	1,460	6,303	7,174
H69	1.46 [1.28-1.66]	1.49 [1.27-1.74]	1.47 [1.21-1.79]	1.43 [1.18-1.73]	1.32 [1.16-1.52]	1.36 [1.19-1.56]	1.28 [1.09-1.51]	1.27 [1.04-1.55]	1.44 [1.18-1.76]	1.25 [1.08-1.43]	634	400	254	297	544
H70	1.18 [1.05-1.33]	1.38 [1.17-1.63]	1.24 [1.03-1.50]	1.04 [0.88-1.23]	1.10 [0.97-1.24]	1.13 [1.00-1.28]	1.23 [1.04-1.46]	1.13 [0.93-1.38]	1.01 [0.85-1.20]	1.07 [0.94-1.22]	696	376	280	350	607
H71	1.10 [1.02-1.19]	1.12 [1.01-1.24]	1.12 [0.98-1.28]	1.07 [0.95-1.19]	1.04 [0.95-1.13]	1.08 [0.99-1.17]	1.08 [0.98-1.21]	1.07 [0.93-1.22]	1.05 [0.94-1.18]	1.05 [0.96-1.15]	1,527	865	512	792	1,274
H72	1.24 [1.18-1.31]	1.25 [1.16-1.35]	1.31 [1.19-1.43]	1.17 [1.09-1.26]	1.13 [1.07-1.20]	1.19 [1.13-1.26]	1.18 [1.09-1.27]	1.20 [1.10-1.32]	1.14 [1.06-1.23]	1.11 [1.05-1.18]	3,386	1,686	1,115	1,886	2,890
H73	1.22 [1.14-1.30]	1.32 [1.18-1.48]	1.34 [1.16-1.55]	1.19 [1.10-1.28]	1.14 [1.06-1.22]	1.17 [1.10-1.25]	1.23 [1.09-1.38]	1.23 [1.06-1.42]	1.16 [1.07-1.25]	1.12 [1.05-1.20]	2,421	755	449	1,806	2,153
H74	1.19 [1.10-1.29]	1.24 [1.12-1.37]	1.33 [1.18-1.51]	1.10 [0.99-1.23]	1.12 [1.03-1.21]	1.15 [1.06-1.24]	1.16 [1.04-1.29]	1.24 [1.09-1.41]	1.06 [0.95-1.19]	1.09 [1.00-1.18]	1,687	906	622	880	1,471
H80	1.09 [0.91-1.30]	1.12 [0.93-1.34]	1.14 [0.93-1.40]		1.11 [0.92-1.35]	1.07 [0.90-1.29]	1.09 [0.91-1.32]	1.12 [0.90-1.38]		1.09 [0.89-1.33]	291	286	209	NA	238
H81	1.26 [1.20-1.32]	1.26 [1.20-1.32]	1.26 [1.20-1.32]	1.31 [0.94-1.81]	1.17 [1.11-1.23]	1.17 [1.12-1.23]	1.14 [1.09-1.20]	1.14 [1.09-1.20]	1.23 [0.87-1.73]	1.11 [1.06-1.16]	4,814	4,767	4,531	103	4,323
H83	1.26 [1.18-1.35]	1.28 [1.19-1.38]	1.26 [1.17-1.36]	1.18 [0.95-1.47]	1.17 [1.09-1.26]	1.17 [1.09-1.25]	1.16 [1.08-1.25]	1.13 [1.04-1.22]	1.12 [0.89-1.40]	1.11 [1.03-1.19]	2,065	1,914	1,663	213	1,781

Supplementary Table 3: Hazard ratios and events from all cohorts

	Hazard ratio (99% confidence interval)										Events (in exposed)				
	crude					adjusted									
Outcome	any age	18+	40+	<18	hosp.	any age	18+	40+	<18	hosp.	any age	18+	40+	<18	hosp.
H90	1.29 [1.24-1.34]	1.36 [1.30-1.43]	1.37 [1.30-1.44]	1.16 [1.09-1.23]	1.20 [1.16-1.25]	1.22 [1.17-1.27]	1.23 [1.17-1.29]	1.24 [1.18-1.31]	1.12 [1.05-1.19]	1.15 [1.11-1.20]	6,956	4,601	3,963	2,583	6,392
H91	1.23 [1.20-1.25]	1.24 [1.21-1.26]	1.23 [1.21-1.26]	1.15 [1.08-1.21]	1.18 [1.15-1.20]	1.17 [1.15-1.20]	1.16 [1.14-1.19]	1.16 [1.13-1.18]	1.11 [1.05-1.17]	1.14 [1.12-1.16]	30,611	27,774	26,657	3,227	28,186
H92	1.34 [1.26-1.42]	1.49 [1.37-1.61]	1.43 [1.30-1.57]	1.23 [1.13-1.33]	1.26 [1.18-1.34]	1.25 [1.18-1.33]	1.31 [1.20-1.42]	1.26 [1.14-1.39]	1.18 [1.08-1.29]	1.20 [1.13-1.28]	2,840	1,565	1,090	1,443	2,500
H93	1.35 [1.27-1.43]	1.37 [1.29-1.46]	1.36 [1.27-1.44]	1.20 [1.01-1.43]	1.28 [1.21-1.36]	1.26 [1.19-1.34]	1.23 [1.16-1.31]	1.22 [1.14-1.30]	1.16 [0.98-1.39]	1.21 [1.14-1.29]	3,029	2,778	2,419	378	2,708
H95	1.27 [1.07-1.50]	1.36 [1.10-1.67]	1.36 [1.04-1.79]	1.20 [0.93-1.54]	1.12 [0.94-1.33]	1.24 [1.05-1.48]	1.27 [1.03-1.58]	1.30 [0.99-1.72]	1.17 [0.90-1.51]	1.13 [0.94-1.35]	349	219	121	168	319
Diseases of the circulatory system (I00-I99)															
I05	1.30 [1.20-1.40]	1.27 [1.17-1.37]	1.32 [1.21-1.43]		1.19 [1.10-1.30]	1.22 [1.12-1.32]	1.17 [1.07-1.27]	1.21 [1.11-1.32]		1.16 [1.07-1.26]	1,575	1,563	1,520	NA	1,409
I06	1.34 [1.05-1.73]	1.25 [0.97-1.60]	1.36 [1.06-1.75]		1.18 [0.91-1.52]	1.26 [0.98-1.63]	1.14 [0.89-1.48]	1.22 [0.95-1.58]		1.12 [0.86-1.45]	155	153	153	NA	141
I07	1.31 [1.24-1.38]	1.30 [1.23-1.37]	1.31 [1.25-1.38]	0.89 [0.67-1.18]	1.23 [1.17-1.30]	1.25 [1.19-1.32]	1.20 [1.14-1.26]	1.20 [1.14-1.27]	0.89 [0.67-1.20]	1.20 [1.14-1.27]	4,088	4,013	3,880	118	3,843
I08	1.21 [1.19-1.24]	1.22 [1.19-1.25]	1.22 [1.19-1.25]	1.08 [0.84-1.37]	1.15 [1.12-1.18]	1.17 [1.14-1.20]	1.13 [1.11-1.16]	1.13 [1.10-1.16]	1.06 [0.83-1.37]	1.12 [1.10-1.15]	19,877	19,808	19,471	177	18,578
I09	1.42 [1.17-1.74]	1.43 [1.17-1.74]	1.52 [1.24-1.86]		1.37 [1.11-1.68]	1.36 [1.11-1.67]	1.35 [1.10-1.65]	1.39 [1.13-1.71]		1.33 [1.07-1.64]	249	247	239	NA	230
I10	1.24 [1.23-1.25]	1.24 [1.23-1.25]	1.24 [1.23-1.25]	1.21 [1.12-1.31]	1.17 [1.16-1.18]	1.19 [1.18-1.20]	1.13 [1.12-1.14]	1.12 [1.11-1.13]	1.17 [1.08-1.27]	1.14 [1.13-1.15]	207,382	206,548	200,764	1,786	153,264
I11	1.21 [1.10-1.33]	1.21 [1.10-1.33]	1.22 [1.11-1.35]		1.06 [0.96-1.17]	1.15 [1.04-1.27]	1.08 [0.98-1.19]	1.10 [0.99-1.21]		1.04 [0.94-1.15]	1,103	1,092	1,084	NA	972
I12	1.29 [1.26-1.33]	1.30 [1.27-1.34]	1.31 [1.27-1.35]		1.19 [1.16-1.23]	1.25 [1.21-1.29]	1.19 [1.15-1.23]	1.19 [1.16-1.23]		1.18 [1.14-1.22]	11,437	11,390	11,255	NA	10,333
I13	1.30 [1.13-1.49]	1.29 [1.12-1.48]	1.28 [1.11-1.47]		1.17 [1.02-1.35]	1.22 [1.06-1.40]	1.15 [1.00-1.33]	1.14 [0.99-1.31]		1.14 [0.99-1.31]	579	577	573	NA	561
I15	1.28 [1.15-1.42]	1.31 [1.17-1.47]	1.26 [1.12-1.42]	1.07 [0.84-1.38]	1.11 [1.00-1.24]	1.22 [1.09-1.36]	1.17 [1.04-1.32]	1.11 [0.98-1.26]	1.08 [0.84-1.39]	1.09 [0.98-1.22]	875	727	675	163	797
I20	1.28 [1.26-1.29]	1.27 [1.26-1.29]	1.28 [1.26-1.30]		1.17 [1.15-1.19]	1.20 [1.18-1.21]	1.14 [1.12-1.15]	1.14 [1.12-1.15]		1.12 [1.10-1.14]	44,789	44,808	44,361	NA	37,819
I21	1.16 [1.13-1.18]	1.15 [1.13-1.18]	1.15 [1.13-1.18]		1.11 [1.09-1.13]	1.13 [1.11-1.15]	1.08 [1.06-1.11]	1.08 [1.06-1.10]		1.10 [1.07-1.12]	28,070	28,078	27,805	NA	23,835
I22	1.28 [1.20-1.36]	1.28 [1.21-1.36]	1.29 [1.22-1.37]		1.18 [1.11-1.25]	1.22 [1.15-1.30]	1.16 [1.09-1.23]	1.16 [1.09-1.24]		1.15 [1.08-1.23]	2,692	2,699	2,683	NA	2,441
I23	1.24 [0.98-1.57]	1.23 [0.97-1.56]	1.30 [1.02-1.66]		1.31 [1.02-1.70]	1.22 [0.95-1.55]	1.15 [0.90-1.47]	1.29 [1.00-1.65]		1.29 [1.00-1.68]	167	166	162	NA	144
I24	1.24 [1.20-1.28]	1.25 [1.21-1.29]	1.24 [1.20-1.28]		1.17 [1.13-1.21]	1.18 [1.13-1.22]	1.13 [1.09-1.17]	1.11 [1.07-1.15]		1.14 [1.10-1.18]	8,471	8,469	8,361	NA	7,812
I25	1.22 [1.21-1.23]	1.22 [1.21-1.24]	1.22 [1.21-1.24]	1.06 [0.81-1.37]	1.14 [1.13-1.16]	1.16 [1.15-1.18]	1.11 [1.10-1.13]	1.11 [1.10-1.12]	1.08 [0.83-1.41]	1.11 [1.10-1.12]	75,749	75,716	75,062	144	64,357
I26	1.13 [1.10-1.16]	1.12 [1.09-1.15]	1.12 [1.09-1.15]	1.07 [0.88-1.29]	1.07 [1.04-1.10]	1.08 [1.05-1.11]	1.05 [1.02-1.08]	1.04 [1.01-1.07]	1.03 [0.84-1.26]	1.04 [1.01-1.07]	14,114	14,073	13,190	277	12,322
I27	1.32 [1.28-1.37]	1.32 [1.28-1.37]	1.31 [1.27-1.36]	0.85 [0.63-1.15]	1.25 [1.21-1.29]	1.25 [1.21-1.29]	1.19 [1.15-1.23]	1.18 [1.14-1.22]	0.77 [0.57-1.05]	1.20 [1.16-1.24]	9,635	9,575	9,398	106	9,065
I28	1.16 [0.96-1.41]	1.19 [0.97-1.46]	1.16 [0.92-1.45]		0.99 [0.82-1.21]	1.07 [0.88-1.30]	1.06 [0.86-1.31]	1.01 [0.80-1.28]		0.94 [0.77-1.15]	269	232	190	NA	246
I30	1.24 [1.07-1.42]	1.20 [1.04-1.39]	1.26 [1.07-1.49]		1.18 [1.01-1.37]	1.19 [1.03-1.37]	1.13 [0.98-1.31]	1.19 [1.00-1.41]		1.15 [0.98-1.34]	475	445	333	NA	388
I31	1.22 [1.17-1.28]	1.23 [1.18-1.28]	1.22 [1.17-1.28]	1.02 [0.88-1.19]	1.13 [1.08-1.18]	1.17 [1.12-1.22]	1.13 [1.08-1.18]	1.13 [1.08-1.18]	0.99 [0.85-1.16]	1.10 [1.05-1.15]	5,730	5,512	4,908	442	5,048
I33	1.35 [1.23-1.50]	1.36 [1.23-1.51]	1.34 [1.20-1.49]		1.25 [1.13-1.39]	1.30 [1.17-1.44]	1.26 [1.13-1.40]	1.25 [1.12-1.39]		1.22 [1.10-1.36]	1,011	982	902	NA	891
I34	1.18 [1.14-1.21]	1.19 [1.16-1.22]	1.19 [1.16-1.23]	0.99 [0.81-1.21]	1.13 [1.09-1.16]	1.13 [1.10-1.16]	1.11 [1.08-1.14]	1.10 [1.07-1.14]	0.93 [0.76-1.15]	1.10 [1.07-1.13]	12,668	12,517	12,151	250	11,507
I35	1.23 [1.20-1.26]	1.23 [1.20-1.26]	1.23 [1.20-1.26]	0.95 [0.78-1.17]	1.17 [1.14-1.20]	1.17 [1.15-1.20]	1.14 [1.11-1.17]	1.14 [1.11-1.17]	0.96 [0.78-1.18]	1.13 [1.10-1.16]	19,023	18,862	18,630	238	17,300
I36	1.23 [1.12-1.34]	1.21 [1.11-1.32]	1.24 [1.13-1.35]		1.17 [1.07-1.28]	1.16 [1.06-1.26]	1.12 [1.02-1.22]	1.13 [1.04-1.24]		1.13 [1.04-1.24]	1,417	1,373	1,313	NA	1,334
I37	1.22 [1.12-1.32]	1.26 [1.15-1.37]	1.29 [1.18-1.41]	0.95 [0.75-1.20]	1.13 [1.04-1.23]	1.15 [1.06-1.25]	1.17 [1.07-1.27]	1.17 [1.07-1.28]	0.94 [0.74-1.20]	1.10 [1.01-1.20]	1,558	1,448	1,318	167	1,463
I38	1.29 [1.21-1.38]	1.30 [1.21-1.40]	1.33 [1.24-1.43]	1.09 [0.84-1.40]	1.21 [1.13-1.30]	1.23 [1.15-1.32]	1.20 [1.11-1.29]	1.22 [1.14-1.32]	1.06 [0.82-1.38]	1.17 [1.09-1.26]	2,228	2,082	2,008	158	2,005
I40	1.29 [1.01-1.64]	1.23 [0.96-1.58]	1.50 [1.10-2.04]		1.35 [1.04-1.76]	1.23 [0.96-1.59]	1.19 [0.92-1.54]	1.39 [1.01-1.91]		1.33 [1.02-1.75]	173	156	108	NA	152
I42	1.21 [1.16-1.27]	1.23 [1.18-1.29]	1.22 [1.16-1.28]	1.17 [0.96-1.43]	1.15 [1.10-1.20]	1.15 [1.10-1.20]	1.12 [1.07-1.17]	1.10 [1.05-1.16]	1.18 [0.95-1.45]	1.11 [1.06-1.16]	4,772	4,639	4,297	242	4,273
I43	0.98 [0.78-1.25]	1.07 [0.85-1.36]	0.98 [0.77-1.25]		0.97 [0.76-1.23]	0.94 [0.74-1.20]	0.99 [0.78-1.27]	0.92 [0.72-1.18]		0.93 [0.73-1.18]	178	172	160	NA	167
I44	1.20 [1.18-1.22]	1.20 [1.18-1.23]	1.20 [1.18-1.23]	1.29 [1.04-1.58]	1.15 [1.13-1.17]	1.16 [1.13-1.18]	1.13 [1.10-1.15]	1.12 [1.10-1.14]	1.26 [1.02-1.56]	1.13 [1.10-1.15]	29,534	29,415	29,080	256	27,092
I45	1.20 [1.18-1.23]	1.21 [1.18-1.24]	1.20 [1.17-1.23]	1.03 [0.91-1.16]	1.15 [1.12-1.18]	1.15 [1.12-1.18]	1.12 [1.09-1.15]	1.11 [1.08-1.14]	0.99 [0.87-1.12]	1.12 [1.09-1.15]	18,619	18,306	17,448	697	17,020
I46	1.23 [1.19-1.27]	1.24 [1.20-1.28]	1.23 [1.19-1.27]	1.08 [0.89-1.30]	1.18 [1.14-1.22]	1.19 [1.15-1.23]	1.15 [1.11-1.19]	1.14 [1.10-1.18]	1.04 [0.85-1.26]	1.17 [1.13-1.21]	9,596	9,458	9,070	275	8,594
I47	1.22 [1.19-1.26]	1.22 [1.18-1.26]	1.22 [1.18-1.26]	1.16 [1.03-1.31]	1.14 [1.10-1.18]	1.15 [1.12-1.19]	1.11 [1.08-1.15]	1.11 [1.07-1.15]	1.11 [0.98-1.25]	1.10 [1.06-1.13]	10,826	10,396	9,373	764	9,433
I48	1.16 [1.15-1.18]	1.16 [1.15-1.18]	1.16 [1.15-1.18]	1.06 [0.85-1.32]	1.12 [1.11-1.13]	1.13 [1.11-1.14]	1.10 [1.09-1.11]	1.09 [1.08-1.11]	1.01 [0.80-1.27]	1.10 [1.09-1.11]	81,212	81,194	80,479	207	70,255
I49	1.18 [1.15-1.22]	1.19 [1.15-1.22]	1.18 [1.15-1.22]	1.16 [1.03-1.31]	1.12 [1.09-1.15]	1.12 [1.09-1.15]	1.09 [1.06-1.12]	1.09 [1.06-1.12]	1.10 [0.96-1.24]	1.08 [1.05-1.11]	12,655	12,285	11,350	733	11,482
I50	1.26 [1.25-1.28]	1.26 [1.24-1.27]	1.26 [1.25-1.28]	1.09 [0.90-1.33]	1.20 [1.19-1.22]	1.20 [1.19-1.22]	1.15 [1.14-1.17]	1.15 [1.14-1.17]	1.10 [0.90-1.35]	1.17 [1.15-1.19]	64,824	64,744	64,197	270	58,996
I51	1.25 [1.23-1.27]	1.25 [1.23-1.27]	1.25 [1.23-1.27]	1.09 [0.97-1.23]	1.18 [1.16-1.20]	1.18 [1.16-1.21]	1.14 [1.12-1.16]	1.13 [1.11-1.16]	1.07 [0.95-1.22]	1.14 [1.12-1.16]	35,441	35,043	34,064	721	32,954
I60	1.16 [1.08-1.24]	1.16 [1.08-1.25]	1.14 [1.06-1.22]		1.10 [1.02-1.18]	1.15 [1.07-1.24]	1.13 [1.05-1.21]	1.11 [1.03-1.19]		1.10 [1.02-1.20]	1,910	1,880	1,738	NA	1,556
I61	1.17 [1.12-1.23]	1.17 [1.12-1.23]	1.17 [1.12-1.22]	0.88 [0.65-1.19]	1.14 [1.09-1.20]	1.16 [1.11-1.22]	1.14 [1.09-1.20]	1.13 [1.08-1.19]	0.88 [0.64-1.20]	1.14 [1.09-1.20]	4,912	4,837	4,715	104	4,230
I62	1.16 [1.09-1.23]	1.18 [1.11-1.25]	1.17 [1.10-1.25]		1.10 [1.03-1.17]	1.14 [1.07-1.21]	1.13 [1.07-1.21]	1.12 [1.05-1.19]		1.10 [1.04-1.18]	2,840	2,794	2,737	NA	2,549
I63	1.09 [1.07-1.11]	1.09 [1.07-1.12]	1.09 [1.07-1.11]	1.10 [0.86-1.42]	1.07 [1.04-1.09]	1.08 [1.06-1.10]	1.05 [1.03-1.07]	1.04 [1.02-1.07]	1.17 [0.90-1.51]	1.07 [1.04-1.09]	22,057	21,969	21,680	159	19,420
I64	1.15 [1.10-1.19]	1.14 [1.09-1.19]	1.15 [1.10-1.19]		1.12 [1.07-1.17]	1.13 [1.08-1.18]	1.09 [1.04-1.13]	1.09 [1.05-1.14]		1.12 [1.07-1.17]	5,844	5,845	5,801	NA	4,912
I65	1.17 [1.13-1.22]	1.17 [1.12-1.22]	1.17 [1.12-1.22]		1.11 [1.06-1.16]	1.14 [1.10-1.19]	1.09 [1.04-1.14]	1.08 [1.04-1.13]		1.10 [1.05-1.15]	5,593	5,582	5,554	NA	4,973
I66	1.15 [0.97-1.36]	1.14 [0.96-1.36]	1.08 [0.90-1.28]		1.12 [0.94-1.34]	1.11 [0.94-1.32]	1.08 [0.91-1.29]	1.00 [0.84-1.20]		1.09 [0.91-1.30]	358	333	309	NA	327
I67	1.18 [1.16-1.20]	1.18 [1.16-1.20]	1.18 [1.16-1.20]	1.08 [0.82-1.41]	1.14 [1.12-1.16]	1.15 [1.13-1.17]	1.11 [1.09-1.13]	1.11 [1.09-1.13]	1.08 [0.82-1.43]	1.13 [1.11-1.15]	35,644	35,555	35,303	135	33,238
I68	1.22 [0.99-1.52]	1.24 [1.00-1.54]	1.22 [0.98-1.52]		1.25 [1.00-1.56]	1.20 [0.96-1.49]	1.19 [0.95-1.49]	1.16 [0.92-1.45]		1.22 [0.97-1.53]	216	214	208	NA	202
I69	1.18 [1.15-1.22]	1.19 [1.15-1.23]	1.18 [1.15-1.22]	0.94 [0.73-1.21]	1.10 [1.06-1.13]	1.15 [1.12-1.19]	1.11 [1.07-1.14]	1.10 [1.06-1.13]	0.91 [0.70-1.17]	1.09 [1.06-1.13]	10,401	10,290	10,123	153	9,596
I70	1.26 [1.21-1.30]	1.25 [1.21-1.30]	1.27 [1.22-1.31]		1.18 [1.14-1.22]	1.21 [1.17-1.26]	1.14 [1.10-1.18]	1.15 [1.11-1.19]		1.17 [1.13-1.21]	9,179	9,177	9,126	NA	8,437
I71	1.17 [1.13-1.20]	1.17 [1.13-1.21]	1.18 [1.14-1.21]		1.11 [1.07-1.15]	1.15 [1.12-1.19]	1.11 [1.07-1.14]	1.11 [1.08-1.15]		1.11 [1.08-1.15]	10,600	10,544	10,459	NA	9,458
I72	1.17 [1.10-1.25]	1.18 [1.11-1.25]	1.18 [1.10-1.25]		1.11 [1.04-1.18]	1.14 [1.07-1.21]	1.09 [1.02-1.16]	1.09 [1.02-1.16]		1.10 [1.03-1.17]	2,555	2,547	2,443	NA	2,312
I73	1.40 [1.37-1.43]	1.40 [1.37-1.43]	1.39 [1.36-1.42]	1.44 [1.24-1.68]	1.29 [1.26-1.32]	1.32 [1.29-1.35]	1.24 [1.21-1.27]	1.23 [1.20-1.26]	1.35 [1.15-1.58]	1.25 [1.22-1.28]	21,801	21,629	20,652	481	19,729
I74	1.18 [1.13-1.23]	1.17 [1.12-1.22]	1.19 [1.14-1.25]		1.11 [1.06-1.17]	1.14 [1.09-1.19]	1.07 [1.02-1.12]	1.09 [1.04-1.14]		1.11 [1.06-1.17]	4,919	4,896	4,775	NA	4,434
I77	1.32 [1.27-1.37]	1.32 [1.27-1.37]	1.33 [1.28-1.39]	1.19 [0.91-1.56]	1.22 [1.17-1.27]	1.26 [1.21-1.31]	1.20 [1.15-1.24]	1.20 [1.16-1.25]	1.13 [0.85-1.49]	1.21 [1.16-1.26]	7,078	7,005	6,801	142	6,373
I78	1.30 [1.21-1.40]	1.35 [1.25-1.45]	1.31 [1.21-1.42]	1.03 [0.77-1.36]	1.24 [1.15-1.34]	1.24 [1.15-1.34]	1.24 [1.14-1.34]	1.21 [1.12-1.31]	1.01 [0.76-1.35]	1.21 [1.12-1.31]	1,829	1,727	1,615	121	1,655
I79	1.34 [1.19-1.51]	1.35 [1.20-1.52]	1.35 [1.20-1.52]		1.19 [1.06-1.35]	1.24 [1.10-1.40]	1.13 [1.00-1.28]	1.13 [1.00-1.28]		1.15 [1.02-1.30]	702	702	687	NA	651
I80	1.25 [1.22-1.29]	1.24 [1.21-1.28]	1.25 [1.21-1.28]	1.15 [0.98-1.36]	1.19 [1.15-1.22]	1.20 [1.17-1.23]	1.16 [1.12-1.19]	1.16 [1.13-1.20]	1.11 [0.93-1.32]	1.15 [1.12-1.19]	13,733	13,610	12,516	371	11,862
I81	1.14 [1.01-1.28]	1.17 [1.04-1.31]	1.14 [1.01-1.28]		1.04 [0.92-1.17]	1.08 [0.96-1.22]	1.08 [0.96-1.22]	1.06 [0.94-1.19]		1.03 [0.91-1.16]	730	720	670	NA	649
I82	1.21 [1.12-1.30]	1.21 [1.12-1.31]	1.20 [1.11-1.30]	1.00 [0.77-1.29]	1.13 [1.04-1.22]	1.15 [1.07-1.25]	1.12 [1.04-1.21]	1.11 [1.02-1.20]	0.98 [0.75-1.29]	1.10 [1.02-1.19]	1,750	1,683	1,445	141	1,556
I83	1.63 [1.59-1.67]	1.63 [1.59-1.67]	1.68 [1.64-1.72]	1.05 [0.86-1.27]	1.52 [1.48-1.56]	1.57 [1.54-1.61]	1.55 [1.52-1.59]	1.59 [1.55-1.63]	1.01 [0.82-1.24]	1.48 [1.45-1.52]	19,256	19,220	17,136	254	15,892
I84	1.37 [1.34-1.39]	1.37 [1.34-1.39]	1.34 [1.32-1.37]	1.21 [1.08-1.36]	1.25 [1.23-1.28]	1.28 [1.25-1.30]	1.24 [1.22-1.27]	1.22 [1.20-1.25]	1.17 [1.04-1.31]	1.19 [1.17-1.22]	26,186	25,999	21,897	783	21,420
I85	1.46 [1.37-1.55]	1.51 [1.41-1.61]	1.47 [1.38-1.57]		1.37 [1.28-1.47]	1.37 [1.29-1.46]	1.35 [1.26-1.44]	1.31 [1.22-1.40]		1.34 [1.25-1.43]	2,382	2,351	2,208	NA	2,141
I86	1.31 [1.22-1.41]	1.36 [1.26-1.47]	1.36 [1.24-1.49]	1.03 [0.88-1.21]	1.24 [1.15-1.34]	1.22 [1.14-1.32]	1.22 [1.13-1.32]	1.21 [1.10-1.33]	0.99 [0.84-1.17]	1.21 [1.12-1.31]	1,918	1,691	1,175	412	1,611
I87	1.60 [1.52-1.69]	1.61 [1.53-1.69]	1.65 [1.56-1.74]	1.06 [0.79-1.42]	1.49 [1.42-1.57]	1.52 [1.44-1.60]	1.48 [1.40-1.55]	1.51 [1.43-1.60]	1.03 [0.76-1.39]	1.45 [1.38-1.53]	4,228	4,167	3,906	125	3,889
I88	1.28 [1.22-1.36]	1.34 [1.18-1.52]	1.53 [1.29-1.82]	1.26 [1.19-1.33]	1.23 [1.16-1.31]	1.25 [1.18-1.32]	1.22 [1.07-1.39]	1.45 [1.22-1.74]	1.22 [1.15-1.29]	1.20 [1.13-1.28]	3,739	602	327	3,277	3,123
I89	1.53 [1.46-1.59]	1.52 [1.46-1.59]	1.51 [1.44-1.58]	1.16 [0.91-1.47]	1.42 [1.35-1.48]	1.43 [1.37-1.50]	1.37 [1.31-1.44]	1.36 [1.30-1.43]	1.08 [0.84-1.39]	1.37 [1.31-1.43]	5,461	5,350	5,091	177	5,058
I95	1.20 [1.18-1.22]	1.21 [1.19-1.23]	1.20 [1.19-1.22]	1.06 [0.97-1.16]	1.15 [1.13-1.16]	1.16 [1.14-1.17]	1.12 [1.11-1.14]	1.12 [1.10-1.14]	1.04 [0.95-1.14]	1.13 [1.11-1.15]	44,151	43,521	41,490	1,343	41,026
I97	1.33 [1.17-1.51]	1.34 [1.18-1.53]	1.28 [1.12-1.46]		1.17 [1.02-1.34]	1.26 [1.10-1.43]	1.25 [1.09-1.43]	1.19 [1.04-1.36]		1.17 [1.02-1.34]	573	563	546	NA	510
I98	1.45 [1.34-1.56]	1.49 [1.38-1.61]	1.45 [1.34-1.58]		1.36 [1.26-1.48]	1.34 [1.24-1.45]	1.31 [1.21-1.42]	1.27 [1.17-1.38]		1.31 [1.21-1.43]	1,591	1,586	1,468	NA	1,455
I99	1.25 [1.11-1.39]	1.22 [1.09-1.37]	1.24 [1.11-1.39]		1.13 [1.00-1.27]	1.20 [1.07-1.34]	1.12 [1.00-1.26]	1.14 [1.02-1.28]		1.11 [0.99-1.25]	808	790	767	NA	718
Diseases of the respiratory system (J00-J99)															
J00	1.41 [1.33-1.48]	1.35 [1.23-1.48]	1.38 [1.23-1.55]	1.41 [1.32-1.50]	1.36 [1.28-1.44]	1.33 [1.26-1.40]	1.22 [1.11-1.34]	1.25 [1.11-1.41]	1.35 [1.27-1.44]	1.31 [1.23-1.39]	3,524	1,113	728	2,539	3,159
J01	1.46 [1.31-1.63]	1.38 [1.21-1.56]	1.38 [1.18-1.62]	1.44 [1.19-1.74]	1.28 [1.14-1.44]	1.31 [1.17-1.47]	1.18 [1.03-1.35]	1.16 [0.99-1.37]	1.36 [1.12-1.66]	1.19 [1.05-1.34]	851	617	388	306	714
J02	1.32 [1.27-1.38]	1.39 [1.31-1.46]	1.44 [1.34-1.54]	1.19 [1.13-1.26]	1.23 [1.18-1.28]	1.24 [1.19-1.29]	1.24 [1.17-1.31]	1.26 [1.18-1.36]	1.15 [1.09-1.22]	1.18 [1.13-1.23]	6,206	3,525	2,082	3,263	5,339
J03	1.22 [1.20-1.24]	1.31 [1.27-1.36]	1.25 [1.14-1.36]	1.20 [1.17-1.22]	1.17 [1.15-1.19]	1.17 [1.15-1.19]	1.17 [1.13-1.21]	1.08 [0.98-1.19]	1.16 [1.14-1.18]	1.14 [1.11-1.16]	32,053	8,122	1,121	27,861	26,714
J04	1.34 [1.19-1.51]	1.47 [1.28-1.68]	1.41 [1.21-1.65]	1.32 [1.07-1.63]	1.33 [1.17-1.50]	1.23 [1.09-1.39]	1.29 [1.12-1.48]	1.19 [1.01-1.40]	1.29 [1.05-1.60]	1.23 [1.08-1.40]	724	538	396	242	633
J05	1.02 [0.97-1.06]	1.04 [0.84-1.28]	1.12 [0.88-1.43]	1.01 [0.96-1.05]	0.98 [0.94-1.03]	0.99 [0.95-1.04]	0.97 [0.78-1.21]	1.03 [0.80-1.32]	0.98 [0.94-1.03]	0.97 [0.92-1.02]	4,821	201	157	4,640	4,236
J06	1.30 [1.27-1.33]	1.38 [1.31-1.45]	1.36 [1.28-1.45]	1.29 [1.26-1.32]	1.24 [1.21-1.27]	1.24 [1.21-1.27]	1.18 [1.12-1.25]	1.18 [1.11-1.26]	1.25 [1.22-1.27]	1.19 [1.17-1.22]	22,016	3,769	2,499	18,724	19,142
J10	1.35 [1.28-1.41]	1.40 [1.33-1.47]	1.36 [1.28-1.44]	1.23 [1.12-1.35]	1.28 [1.22-1.34]	1.22 [1.16-1.28]	1.19 [1.13-1.26]	1.17 [1.10-1.24]	1.15 [1.04-1.26]	1.18 [1.13-1.24]	4,807	3,923	3,220	1,161	4,498
J11	1.35 [1.22-1.49]	1.34 [1.20-1.50]	1.35 [1.19-1.54]	1.18 [0.97-1.44]	1.26 [1.13-1.40]	1.24 [1.12-1.38]	1.17 [1.04-1.32]	1.20 [1.05-1.37]	1.13 [0.92-1.38]	1.18 [1.06-1.32]	1,009	809	587	269	902
J12	1.28 [1.23-1.33]	1.29 [1.23-1.34]	1.31 [1.25-1.36]	1.16 [1.02-1.32]	1.23 [1.18-1.28]	1.19 [1.14-1.24]	1.15 [1.10-1.20]	1.16 [1.11-1.21]	1.10 [0.96-1.25]	1.17 [1.12-1.22]	7,080	6,617	6,240	592	6,651
J13	1.32 [1.22-1.43]	1.37 [1.26-1.48]	1.36 [1.24-1.48]	1.18 [0.90-1.53]	1.27 [1.17-1.38]	1.21 [1.12-1.32]	1.20 [1.10-1.31]	1.19 [1.09-1.30]	1.09 [0.83-1.43]	1.19 [1.09-1.29]	1,584	1,485	1,350	141	1,388
J14	1.33 [1.17-1.50]	1.38 [1.21-1.56]	1.35 [1.19-1.54]		1.23 [1.08-1.40]	1.17 [1.03-1.33]	1.14 [0.99-1.30]	1.11 [0.96-1.27]		1.11 [0.97-1.26]	656	626	583	NA	596
J15	1.38 [1.31-1.45]	1.42 [1.34-1.50]	1.43 [1.35-1.52]	1.22 [1.05-1.41]	1.32 [1.24-1.39]	1.26 [1.19-1.33]	1.24 [1.17-1.32]	1.23 [1.16-1.31]	1.16 [1.00-1.35]	1.23 [1.16-1.30]	3,592	3,195	2,976	465	3,254
J17	1.49 [1.32-1.67]	1.49 [1.32-1.68]	1.46 [1.29-1.65]		1.35 [1.20-1.53]	1.38 [1.23-1.56]	1.34 [1.18-1.51]	1.31 [1.15-1.50]		1.28 [1.13-1.45]	737	687	628	NA	661
J18	1.27 [1.26-1.28]	1.27 [1.26-1.28]	1.26 [1.25-1.28]	1.30 [1.26-1.35]	1.21 [1.20-1.22]	1.21 [1.20-1.22]	1.16 [1.15-1.18]	1.16 [1.14-1.17]	1.25 [1.21-1.30]	1.17 [1.16-1.18]	97,392	90,121	86,034	8,531	88,216
J20	1.29 [1.18-1.40]	1.28 [1.16-1.41]	1.26 [1.14-1.40]	1.30 [1.09-1.56]	1.22 [1.12-1.34]	1.18 [1.08-1.29]	1.12 [1.02-1.24]	1.12 [1.00-1.24]	1.25 [1.04-1.50]	1.15 [1.05-1.26]	1,357	1,100	960	309	1,246
J21	1.14 [1.05-1.25]	1.46 [1.22-1.75]	1.47 [1.22-1.78]	1.04 [0.94-1.16]	1.09 [0.99-1.20]	1.07 [0.97-1.17]	1.21 [1.00-1.46]	1.25 [1.03-1.53]	1.00 [0.90-1.11]	1.03 [0.93-1.13]	1,139	318	287	830	1,033
J22	1.36 [1.35-1.38]	1.35 [1.33-1.37]	1.32 [1.30-1.34]	1.43 [1.39-1.46]	1.28 [1.26-1.30]	1.27 [1.26-1.29]	1.21 [1.19-1.23]	1.19 [1.17-1.21]	1.35 [1.31-1.38]	1.22 [1.20-1.23]	68,041	53,382	47,758	16,442	61,394
J30	2.14 [2.08-2.20]	1.76 [1.71-1.82]	1.67 [1.60-1.74]	2.83 [2.72-2.95]	2.03 [1.98-2.09]	1.73 [1.68-1.78]	1.32 [1.27-1.36]	1.29 [1.23-1.35]	2.38 [2.28-2.48]	1.66 [1.61-1.71]	17,750	10,536	6,066	9,124	15,193
J31	1.75 [1.66-1.86]	1.70 [1.59-1.81]	1.59 [1.47-1.72]	1.96 [1.78-2.17]	1.62 [1.52-1.72]	1.44 [1.35-1.53]	1.33 [1.24-1.43]	1.24 [1.14-1.34]	1.68 [1.52-1.87]	1.34 [1.26-1.43]	3,383	2,421	1,625	1,259	2,875
J32	1.42 [1.36-1.47]	1.46 [1.40-1.52]	1.42 [1.35-1.49]	1.31 [1.20-1.44]	1.32 [1.27-1.38]	1.22 [1.18-1.28]	1.20 [1.15-1.26]	1.18 [1.13-1.25]	1.21 [1.10-1.33]	1.17 [1.12-1.22]	6,587	5,843	4,146	1,286	5,596
J33	1.56 [1.49-1.64]	1.54 [1.47-1.62]	1.52 [1.44-1.61]	1.41 [1.21-1.64]	1.42 [1.35-1.50]	1.29 [1.23-1.36]	1.25 [1.19-1.32]	1.25 [1.18-1.32]	1.21 [1.03-1.42]	1.20 [1.14-1.27]	4,446	4,224	3,253	471	3,366
J34	1.51 [1.47-1.54]	1.51 [1.46-1.55]	1.48 [1.43-1.54]	1.48 [1.42-1.54]	1.39 [1.35-1.43]	1.35 [1.31-1.38]	1.29 [1.25-1.33]	1.28 [1.23-1.33]	1.37 [1.31-1.43]	1.26 [1.23-1.30]	16,429	12,145	6,606	6,409	12,605
J35	1.28 [1.26-1.31]	1.30 [1.24-1.36]	1.23 [1.13-1.35]	1.28 [1.25-1.30]	1.24 [1.21-1.27]	1.23 [1.21-1.26]	1.17 [1.11-1.23]	1.10 [1.01-1.21]	1.23 [1.20-1.26]	1.20 [1.17-1.23]	25,871	4,000	1,120	23,193	20,766
J36	1.14 [1.07-1.22]	1.16 [1.08-1.24]	1.14 [1.00-1.30]	1.11 [1.01-1.22]	1.07 [1.00-1.15]	1.11 [1.04-1.18]	1.11 [1.03-1.19]	1.09 [0.95-1.25]	1.10 [1.00-1.21]	1.06 [0.99-1.15]	2,461	1,982	525	1,193	1,763
J37	1.39 [1.07-1.81]	1.40 [1.07-1.84]	1.31 [0.98-1.75]		1.19 [0.90-1.58]	1.24 [0.95-1.63]	1.15 [0.86-1.53]	1.10 [0.81-1.49]		1.09 [0.82-1.45]	143	129	108	NA	118
J38	1.30 [1.24-1.36]	1.31 [1.25-1.38]	1.30 [1.24-1.37]	1.23 [1.08-1.39]	1.21 [1.15-1.27]	1.21 [1.15-1.27]	1.15 [1.10-1.21]	1.16 [1.10-1.22]	1.12 [0.99-1.28]	1.15 [1.09-1.21]	4,666	4,151	3,615	666	4,048
J39	1.33 [1.25-1.42]	1.34 [1.25-1.44]	1.32 [1.23-1.43]	1.27 [1.11-1.45]	1.20 [1.12-1.28]	1.23 [1.15-1.31]	1.18 [1.10-1.26]	1.18 [1.09-1.27]	1.17 [1.02-1.34]	1.12 [1.05-1.20]	2,592	2,111	1,790	588	2,276
J40	1.44 [1.36-1.53]	1.44 [1.36-1.53]	1.45 [1.36-1.54]	1.32 [0.96-1.82]	1.30 [1.23-1.39]	1.29 [1.21-1.37]	1.23 [1.16-1.31]	1.25 [1.17-1.33]	1.24 [0.89-1.73]	1.19 [1.12-1.27]	2,869	2,824	2,580	108	2,545
J41	1.44 [1.09-1.89]	1.38 [1.05-1.81]	1.36 [1.03-1.81]		1.18 [0.88-1.57]	1.32 [0.99-1.74]	1.18 [0.88-1.57]	1.18 [0.88-1.58]		1.13 [0.84-1.52]	131	130	120	NA	112
J42	1.45 [1.32-1.60]	1.42 [1.29-1.56]	1.42 [1.29-1.56]		1.31 [1.19-1.44]	1.28 [1.16-1.41]	1.19 [1.08-1.32]	1.18 [1.07-1.31]		1.17 [1.05-1.29]	1,161	1,122	1,077	NA	1,039
J43	1.35 [1.32-1.39]	1.35 [1.32-1.39]	1.36 [1.32-1.39]	1.21 [0.93-1.57]	1.28 [1.25-1.32]	1.25 [1.22-1.29]	1.18 [1.15-1.21]	1.18 [1.15-1.22]	1.17 [0.89-1.53]	1.21 [1.18-1.25]	15,001	14,949	14,688	151	13,692
J44	1.46 [1.44-1.48]	1.45 [1.44-1.47]	1.45 [1.43-1.47]	1.81 [1.49-2.20]	1.36 [1.34-1.38]	1.29 [1.27-1.31]	1.20 [1.19-1.22]	1.21 [1.19-1.22]	1.54 [1.26-1.89]	1.22 [1.21-1.24]	63,230	63,040	62,368	310	55,206
J45	2.12 [2.11-2.14]	1.98 [1.96-2.00]	1.84 [1.82-1.87]	2.50 [2.46-2.53]	1.93 [1.91-1.95]	1.61 [1.59-1.62]	1.34 [1.33-1.36]	1.31 [1.29-1.33]	2.06 [2.02-2.09]	1.51 [1.50-1.53]	165,066	117,603	75,502	63,223	122,304
J46	2.95 [2.79-3.13]	2.46 [2.25-2.68]	2.08 [1.85-2.33]	3.34 [3.10-3.60]	2.59 [2.43-2.76]	2.29 [2.15-2.44]	1.55 [1.40-1.72]	1.35 [1.18-1.54]	2.76 [2.55-2.99]	2.01 [1.88-2.15]	3,968	1,559	809	2,673	3,324
J47	1.49 [1.45-1.54]	1.51 [1.47-1.56]	1.50 [1.45-1.55]	1.29 [1.06-1.58]	1.39 [1.35-1.44]	1.29 [1.25-1.33]	1.27 [1.23-1.31]	1.26 [1.22-1.30]	1.15 [0.93-1.42]	1.22 [1.18-1.26]	11,900	11,734	11,381	269	11,013
J61	1.39 [1.28-1.52]	1.38 [1.27-1.50]	1.40 [1.28-1.52]		1.32 [1.21-1.43]	1.32 [1.21-1.43]	1.27 [1.16-1.38]	1.27 [1.17-1.38]		1.25 [1.15-1.37]	1,512	1,513	1,512	NA	1,416
J67	1.61 [1.39-1.86]	1.60 [1.38-1.85]	1.59 [1.37-1.84]		1.44 [1.24-1.67]	1.44 [1.24-1.67]	1.38 [1.19-1.61]	1.40 [1.20-1.63]		1.30 [1.11-1.52]	497	492	464	NA	442
J69	1.17 [1.14-1.20]	1.18 [1.14-1.21]	1.18 [1.14-1.21]	0.93 [0.78-1.10]	1.12 [1.09-1.16]	1.15 [1.12-1.18]	1.12 [1.09-1.16]	1.12 [1.09-1.15]	0.90 [0.76-1.07]	1.13 [1.10-1.16]	13,535	13,347	12,953	323	12,687
J70	1.52 [1.27-1.82]	1.44 [1.21-1.73]	1.51 [1.25-1.81]		1.35 [1.13-1.62]	1.42 [1.18-1.70]	1.27 [1.06-1.53]	1.36 [1.13-1.64]		1.28 [1.06-1.54]	321	309	303	NA	300
J80	1.37 [1.25-1.51]	1.40 [1.27-1.54]	1.45 [1.31-1.60]		1.27 [1.15-1.40]	1.31 [1.19-1.44]	1.27 [1.15-1.40]	1.31 [1.18-1.45]		1.25 [1.13-1.38]	1,168	1,119	1,008	NA	1,042
J81	1.30 [1.24-1.36]	1.29 [1.23-1.35]	1.31 [1.25-1.37]	0.97 [0.72-1.31]	1.19 [1.14-1.25]	1.25 [1.20-1.31]	1.18 [1.13-1.24]	1.21 [1.15-1.27]	0.96 [0.70-1.30]	1.17 [1.11-1.23]	4,916	4,853	4,709	107	4,416
J82	2.40 [2.08-2.78]	2.31 [2.00-2.68]	2.11 [1.80-2.47]		2.24 [1.93-2.59]	1.72 [1.47-2.02]	1.55 [1.31-1.82]	1.49 [1.25-1.77]		1.69 [1.44-1.98]	600	574	451	NA	570
J84	1.44 [1.39-1.49]	1.45 [1.41-1.50]	1.44 [1.40-1.49]		1.37 [1.32-1.42]	1.36 [1.31-1.41]	1.34 [1.29-1.38]	1.33 [1.28-1.37]		1.32 [1.27-1.36]	9,901	9,866	9,723	NA	9,106
J85	1.31 [1.14-1.51]	1.37 [1.19-1.59]	1.37 [1.18-1.59]		1.23 [1.06-1.42]	1.21 [1.05-1.39]	1.23 [1.06-1.43]	1.22 [1.05-1.43]		1.18 [1.01-1.37]	509	469	429	NA	442
J86	1.20 [1.12-1.30]	1.24 [1.14-1.35]	1.25 [1.15-1.36]	1.07 [0.89-1.30]	1.13 [1.05-1.23]	1.17 [1.08-1.26]	1.16 [1.07-1.27]	1.16 [1.07-1.27]	1.10 [0.91-1.33]	1.13 [1.04-1.23]	1,711	1,474	1,351	269	1,449
J90	1.19 [1.17-1.21]	1.19 [1.17-1.21]	1.20 [1.18-1.22]	1.11 [1.01-1.21]	1.14 [1.12-1.16]	1.16 [1.14-1.18]	1.12 [1.10-1.14]	1.12 [1.10-1.14]	1.11 [1.02-1.22]	1.12 [1.11-1.14]	44,263	43,369	41,910	1,336	40,046
J91	1.24 [1.12-1.37]	1.25 [1.13-1.38]	1.21 [1.09-1.34]		1.20 [1.09-1.34]	1.21 [1.09-1.33]	1.17 [1.06-1.30]	1.13 [1.02-1.25]		1.19 [1.08-1.32]	1,085	1,059	1,021	NA	984
J92	1.26 [1.20-1.31]	1.28 [1.22-1.34]	1.26 [1.20-1.31]		1.21 [1.15-1.27]	1.22 [1.16-1.27]	1.21 [1.15-1.26]	1.18 [1.13-1.24]		1.19 [1.13-1.25]	5,072	5,066	5,035	NA	4,697
J93	1.15 [1.09-1.20]	1.14 [1.08-1.20]	1.17 [1.10-1.23]	1.05 [0.93-1.20]	1.08 [1.02-1.14]	1.12 [1.06-1.17]	1.07 [1.01-1.12]	1.10 [1.04-1.16]	1.06 [0.93-1.21]	1.07 [1.01-1.12]	4,150	3,817	3,221	627	3,472
J94	1.18 [1.09-1.28]	1.18 [1.09-1.28]	1.20 [1.11-1.30]		1.09 [1.01-1.18]	1.15 [1.07-1.25]	1.10 [1.02-1.19]	1.12 [1.03-1.22]		1.08 [0.99-1.17]	1,641	1,604	1,549	NA	1,469
J95	1.21 [1.11-1.31]	1.20 [1.10-1.31]	1.24 [1.13-1.35]	0.98 [0.75-1.27]	1.11 [1.01-1.21]	1.17 [1.08-1.27]	1.11 [1.02-1.22]	1.16 [1.06-1.27]	0.91 [0.70-1.20]	1.10 [1.00-1.20]	1,356	1,249	1,173	134	1,156
J96	1.32 [1.30-1.34]	1.31 [1.29-1.34]	1.30 [1.28-1.33]	1.21 [1.07-1.36]	1.24 [1.22-1.26]	1.23 [1.21-1.25]	1.17 [1.14-1.19]	1.16 [1.13-1.18]	1.12 [0.99-1.26]	1.18 [1.16-1.21]	30,130	29,782	28,727	709	27,859
J98	1.25 [1.23-1.28]	1.24 [1.22-1.26]	1.23 [1.21-1.26]	1.25 [1.17-1.33]	1.18 [1.16-1.21]	1.17 [1.15-1.20]	1.12 [1.09-1.14]	1.11 [1.09-1.13]	1.17 [1.10-1.25]	1.14 [1.11-1.16]	28,140	26,101	24,543	2,583	25,833
J99	1.60 [1.45-1.77]	1.66 [1.50-1.83]	1.71 [1.54-1.91]		1.44 [1.30-1.60]	1.39 [1.25-1.54]	1.41 [1.27-1.57]	1.47 [1.32-1.64]		1.28 [1.15-1.42]	1,026	987	897	NA	950
Diseases of the digestive system (K00-K95)															
K00	1.16 [1.12-1.21]	1.15 [1.06-1.25]	1.22 [1.02-1.47]	1.14 [1.09-1.19]	1.09 [1.05-1.15]	1.12 [1.08-1.17]	1.05 [0.96-1.14]	1.12 [0.93-1.35]	1.11 [1.06-1.16]	1.07 [1.02-1.13]	6,500	1,359	276	5,703	4,997
K01	1.21 [1.18-1.25]	1.23 [1.19-1.27]	1.15 [1.06-1.24]	1.18 [1.14-1.22]	1.13 [1.10-1.17]	1.16 [1.13-1.19]	1.13 [1.10-1.17]	1.10 [1.02-1.19]	1.14 [1.10-1.18]	1.10 [1.06-1.13]	14,936	9,240	1,484	8,671	10,238
K02	1.11 [1.09-1.13]	1.25 [1.22-1.28]	1.28 [1.24-1.32]	1.02 [1.00-1.04]	1.05 [1.03-1.07]	1.07 [1.05-1.09]	1.12 [1.10-1.15]	1.15 [1.11-1.19]	1.01 [0.99-1.03]	1.03 [1.01-1.05]	42,689	17,603	8,465	28,068	34,584
K03	1.17 [1.07-1.29]	1.46 [1.27-1.68]	1.42 [1.18-1.72]	1.00 [0.89-1.13]	1.11 [1.00-1.23]	1.12 [1.01-1.23]	1.34 [1.16-1.56]	1.27 [1.05-1.54]	0.98 [0.86-1.11]	1.07 [0.97-1.19]	1,129	494	271	736	995
K04	1.14 [1.10-1.18]	1.22 [1.17-1.27]	1.25 [1.19-1.32]	1.00 [0.95-1.05]	1.07 [1.03-1.11]	1.09 [1.05-1.13]	1.10 [1.05-1.15]	1.13 [1.07-1.19]	0.99 [0.94-1.05]	1.04 [1.00-1.08]	8,999	5,838	3,471	3,950	7,404
K05	1.29 [1.25-1.34]	1.33 [1.28-1.38]	1.33 [1.24-1.42]	1.21 [1.14-1.29]	1.19 [1.15-1.24]	1.22 [1.17-1.26]	1.20 [1.15-1.24]	1.21 [1.13-1.29]	1.16 [1.09-1.24]	1.15 [1.10-1.19]	8,466	7,996	2,186	2,842	6,400
K06	1.38 [1.26-1.52]	1.37 [1.23-1.51]	1.37 [1.22-1.54]	1.19 [0.98-1.44]	1.24 [1.12-1.37]	1.31 [1.19-1.44]	1.27 [1.15-1.42]	1.25 [1.11-1.40]	1.12 [0.92-1.37]	1.19 [1.07-1.32]	1,145	937	728	279	970
K07	1.19 [1.15-1.23]	1.22 [1.16-1.28]	1.29 [1.17-1.42]	1.15 [1.10-1.19]	1.12 [1.08-1.17]	1.14 [1.10-1.18]	1.11 [1.05-1.17]	1.14 [1.03-1.27]	1.11 [1.07-1.16]	1.09 [1.05-1.14]	9,242	3,771	948	6,972	6,750
K08	1.19 [1.15-1.22]	1.25 [1.21-1.29]	1.29 [1.24-1.34]	1.04 [0.99-1.09]	1.12 [1.08-1.15]	1.13 [1.10-1.16]	1.14 [1.10-1.17]	1.15 [1.11-1.20]	1.02 [0.98-1.07]	1.08 [1.05-1.12]	12,768	9,116	5,826	4,744	10,589
K09	1.26 [1.14-1.40]	1.26 [1.13-1.41]	1.13 [0.99-1.30]	1.23 [1.02-1.48]	1.17 [1.05-1.32]	1.24 [1.12-1.38]	1.18 [1.05-1.33]	1.08 [0.94-1.24]	1.19 [0.98-1.44]	1.15 [1.02-1.29]	971	742	489	311	725
K10	1.25 [1.14-1.36]	1.27 [1.15-1.39]	1.27 [1.15-1.41]	1.26 [1.02-1.57]	1.15 [1.04-1.26]	1.15 [1.05-1.26]	1.11 [1.01-1.22]	1.12 [1.01-1.25]	1.21 [0.97-1.52]	1.10 [1.00-1.21]	1,235	1,114	877	227	1,102
K11	1.31 [1.24-1.39]	1.36 [1.28-1.45]	1.36 [1.27-1.46]	1.16 [1.04-1.30]	1.24 [1.16-1.32]	1.24 [1.17-1.32]	1.25 [1.17-1.33]	1.23 [1.15-1.33]	1.12 [1.00-1.25]	1.19 [1.12-1.27]	3,061	2,405	1,914	848	2,583
K12	1.29 [1.24-1.35]	1.33 [1.26-1.40]	1.35 [1.28-1.44]	1.19 [1.10-1.29]	1.23 [1.17-1.29]	1.24 [1.19-1.30]	1.22 [1.16-1.29]	1.25 [1.18-1.33]	1.17 [1.08-1.27]	1.20 [1.14-1.26]	4,859	3,595	2,811	1,596	4,287

Supplementary Table 3: Hazard ratios and events from all cohorts

	Hazard ratio (99% confidence interval)										Events (in exposed)				
	crude					adjusted									
Outcome	any age	18+	40+	<18	hosp.	any age	18+	40+	<18	hosp.	any age	18+	40+	<18	hosp.
K13	1.37 [1.31-1.42]	1.38 [1.32-1.44]	1.38 [1.32-1.45]	1.30 [1.20-1.41]	1.30 [1.25-1.36]	1.30 [1.25-1.35]	1.26 [1.21-1.32]	1.26 [1.20-1.32]	1.26 [1.16-1.37]	1.26 [1.20-1.31]	6,539	5,201	4,172	1,657	5,395
K14	1.39 [1.31-1.47]	1.40 [1.32-1.49]	1.37 [1.29-1.46]	1.32 [1.14-1.53]	1.29 [1.21-1.37]	1.28 [1.21-1.36]	1.24 [1.17-1.32]	1.23 [1.15-1.31]	1.26 [1.09-1.47]	1.21 [1.14-1.29]	3,114	2,746	2,364	495	2,705
K20	1.33 [1.30-1.36]	1.32 [1.29-1.36]	1.30 [1.27-1.34]	1.49 [1.35-1.64]	1.23 [1.20-1.26]	1.22 [1.19-1.25]	1.18 [1.15-1.20]	1.16 [1.13-1.19]	1.34 [1.21-1.49]	1.15 [1.12-1.18]	18,438	17,762	16,047	1,158	15,910
K21	1.36 [1.34-1.37]	1.36 [1.35-1.38]	1.36 [1.34-1.37]	1.23 [1.18-1.29]	1.25 [1.23-1.26]	1.21 [1.20-1.23]	1.18 [1.17-1.19]	1.17 [1.16-1.19]	1.11 [1.06-1.17]	1.15 [1.13-1.16]	73,030	70,858	62,008	4,889	64,783
K22	1.29 [1.27-1.32]	1.30 [1.27-1.32]	1.29 [1.26-1.32]	1.21 [1.10-1.33]	1.21 [1.18-1.23]	1.21 [1.18-1.23]	1.18 [1.16-1.20]	1.17 [1.15-1.20]	1.12 [1.01-1.24]	1.15 [1.13-1.17]	26,465	25,926	24,113	1,101	23,013
K23	1.54 [1.30-1.84]	1.50 [1.26-1.78]	1.49 [1.24-1.78]		1.47 [1.23-1.75]	1.38 [1.16-1.65]	1.23 [1.02-1.47]	1.27 [1.05-1.52]		1.33 [1.11-1.60]	347	345	330	NA	325
K25	1.29 [1.26-1.33]	1.30 [1.26-1.33]	1.29 [1.25-1.32]	1.30 [1.10-1.54]	1.21 [1.17-1.24]	1.22 [1.18-1.25]	1.17 [1.14-1.20]	1.17 [1.13-1.20]	1.27 [1.07-1.52]	1.16 [1.13-1.20]	13,006	12,851	12,008	368	11,348
K26	1.19 [1.15-1.23]	1.21 [1.17-1.25]	1.20 [1.16-1.24]	1.21 [0.99-1.48]	1.15 [1.11-1.19]	1.16 [1.12-1.20]	1.14 [1.10-1.18]	1.13 [1.09-1.17]	1.18 [0.95-1.46]	1.14 [1.10-1.18]	8,838	8,712	8,214	259	7,495
K27	1.29 [1.20-1.40]	1.28 [1.19-1.39]	1.29 [1.19-1.40]		1.18 [1.09-1.28]	1.20 [1.10-1.30]	1.13 [1.04-1.22]	1.15 [1.05-1.25]		1.13 [1.04-1.23]	1,584	1,563	1,448	NA	1,439
K28	1.47 [1.20-1.79]	1.46 [1.19-1.79]	1.38 [1.11-1.72]		1.43 [1.17-1.75]	1.24 [1.00-1.52]	1.19 [0.96-1.47]	1.07 [0.84-1.35]		1.29 [1.05-1.59]	255	248	201	NA	247
K29	1.32 [1.31-1.34]	1.33 [1.31-1.35]	1.33 [1.31-1.35]	1.23 [1.17-1.28]	1.22 [1.21-1.24]	1.22 [1.21-1.24]	1.19 [1.17-1.20]	1.19 [1.17-1.20]	1.16 [1.11-1.22]	1.16 [1.15-1.18]	69,990	66,962	59,204	5,411	60,277
K30	1.36 [1.33-1.39]	1.36 [1.33-1.39]	1.36 [1.32-1.39]	1.19 [1.06-1.34]	1.25 [1.22-1.28]	1.24 [1.21-1.27]	1.20 [1.17-1.23]	1.21 [1.17-1.24]	1.12 [0.99-1.26]	1.16 [1.13-1.20]	16,797	16,597	14,037	731	14,039
K31	1.30 [1.27-1.33]	1.31 [1.28-1.33]	1.30 [1.27-1.33]	1.11 [0.97-1.26]	1.21 [1.18-1.23]	1.18 [1.16-1.21]	1.15 [1.12-1.17]	1.14 [1.12-1.17]	1.04 [0.91-1.19]	1.13 [1.10-1.15]	22,728	22,438	21,097	633	20,728
K35	1.08 [1.05-1.11]	1.09 [1.05-1.13]	1.05 [1.00-1.11]	1.04 [1.00-1.08]	1.01 [0.98-1.04]	1.04 [1.01-1.07]	1.03 [1.00-1.07]	1.01 [0.95-1.06]	1.02 [0.98-1.06]	0.99 [0.96-1.03]	13,415	7,735	3,340	7,734	9,842
K36	1.17 [0.98-1.40]	1.20 [0.97-1.48]		1.35 [1.03-1.76]	1.15 [0.94-1.40]	1.15 [0.96-1.38]	1.11 [0.89-1.38]		1.36 [1.03-1.79]	1.12 [0.91-1.37]	297	201	NA	148	237
K37	1.14 [1.07-1.21]	1.13 [1.04-1.22]	1.15 [1.01-1.32]	1.08 [0.99-1.17]	1.06 [0.99-1.13]	1.07 [1.01-1.14]	1.01 [0.93-1.10]	1.07 [0.93-1.23]	1.04 [0.95-1.13]	1.02 [0.95-1.09]	2,653	1,504	518	1,617	2,057
K38	1.15 [1.07-1.24]	1.16 [1.06-1.27]	1.10 [0.97-1.25]	1.08 [0.97-1.21]	1.06 [0.98-1.15]	1.10 [1.01-1.18]	1.06 [0.97-1.16]	1.05 [0.92-1.19]	1.05 [0.94-1.18]	1.04 [0.95-1.13]	1,735	1,171	581	837	1,394
K40	1.10 [1.07-1.12]	1.11 [1.09-1.14]	1.11 [1.08-1.13]	1.02 [0.96-1.08]	1.06 [1.04-1.09]	1.06 [1.04-1.08]	1.08 [1.06-1.10]	1.07 [1.05-1.10]	0.98 [0.93-1.04]	1.04 [1.02-1.06]	23,888	21,407	19,508	3,081	18,644
K41	1.19 [1.10-1.28]	1.18 [1.09-1.27]	1.16 [1.08-1.26]		1.12 [1.03-1.21]	1.13 [1.05-1.22]	1.12 [1.04-1.21]	1.10 [1.02-1.19]		1.07 [0.98-1.16]	1,699	1,670	1,555	NA	1,454
K42	1.29 [1.25-1.32]	1.29 [1.26-1.33]	1.29 [1.25-1.34]	1.19 [1.10-1.28]	1.20 [1.17-1.24]	1.17 [1.14-1.21]	1.14 [1.11-1.18]	1.14 [1.10-1.17]	1.09 [1.00-1.18]	1.13 [1.09-1.16]	11,993	10,583	8,923	1,665	10,301
K43	1.26 [1.22-1.30]	1.27 [1.23-1.32]	1.28 [1.24-1.33]	1.13 [1.00-1.28]	1.18 [1.14-1.22]	1.16 [1.12-1.20]	1.14 [1.10-1.18]	1.14 [1.10-1.18]	1.05 [0.92-1.20]	1.12 [1.09-1.16]	8,989	8,524	7,619	615	8,303
K44	1.31 [1.29-1.32]	1.32 [1.30-1.33]	1.30 [1.29-1.32]	1.27 [1.17-1.38]	1.22 [1.20-1.23]	1.20 [1.18-1.21]	1.17 [1.16-1.19]	1.16 [1.15-1.18]	1.17 [1.08-1.28]	1.14 [1.12-1.15]	66,300	66,090	60,673	1,682	57,225
K45	1.22 [1.04-1.42]	1.18 [1.01-1.38]	1.20 [1.02-1.41]		1.12 [0.96-1.32]	1.12 [0.95-1.31]	1.04 [0.88-1.22]	1.07 [0.90-1.26]		1.04 [0.88-1.23]	407	401	361	NA	378
K46	1.31 [1.21-1.41]	1.34 [1.24-1.45]	1.35 [1.25-1.46]		1.20 [1.11-1.30]	1.18 [1.09-1.28]	1.16 [1.07-1.25]	1.17 [1.08-1.27]		1.12 [1.04-1.21]	1,772	1,736	1,636	NA	1,684
K50	1.70 [1.63-1.77]	1.65 [1.58-1.72]	1.56 [1.47-1.64]	1.80 [1.65-1.96]	1.55 [1.48-1.62]	1.47 [1.41-1.54]	1.35 [1.29-1.42]	1.29 [1.22-1.36]	1.67 [1.53-1.82]	1.38 [1.32-1.45]	6,324	5,403	3,344	1,728	5,217
K51	1.51 [1.46-1.56]	1.51 [1.46-1.57]	1.45 [1.39-1.51]	1.44 [1.31-1.58]	1.36 [1.31-1.42]	1.35 [1.30-1.40]	1.29 [1.24-1.34]	1.24 [1.19-1.30]	1.36 [1.23-1.50]	1.25 [1.20-1.30]	8,057	7,573	5,375	1,254	6,659
K52	1.35 [1.33-1.38]	1.39 [1.36-1.41]	1.36 [1.34-1.39]	1.22 [1.18-1.27]	1.25 [1.23-1.27]	1.27 [1.25-1.29]	1.24 [1.22-1.26]	1.23 [1.21-1.25]	1.19 [1.14-1.23]	1.20 [1.18-1.22]	41,353	35,695	29,410	7,425	34,827
K55	1.25 [1.20-1.30]	1.25 [1.20-1.30]	1.25 [1.20-1.31]		1.15 [1.10-1.21]	1.19 [1.14-1.24]	1.14 [1.10-1.20]	1.14 [1.09-1.19]		1.13 [1.08-1.19]	5,570	5,532	5,358	NA	5,112
K56	1.21 [1.18-1.24]	1.21 [1.18-1.24]	1.20 [1.17-1.23]	1.08 [0.99-1.18]	1.14 [1.11-1.17]	1.15 [1.12-1.17]	1.11 [1.09-1.14]	1.11 [1.08-1.14]	1.04 [0.95-1.14]	1.10 [1.07-1.13]	17,069	16,153	14,775	1,354	15,433
K57	1.25 [1.24-1.27]	1.25 [1.23-1.26]	1.25 [1.23-1.26]	1.15 [0.93-1.43]	1.17 [1.15-1.18]	1.17 [1.16-1.19]	1.14 [1.12-1.15]	1.14 [1.12-1.15]	1.06 [0.84-1.32]	1.11 [1.10-1.13]	72,988	72,950	71,810	235	63,834
K58	1.50 [1.47-1.53]	1.52 [1.49-1.55]	1.53 [1.49-1.56]	1.29 [1.21-1.37]	1.39 [1.36-1.42]	1.27 [1.24-1.30]	1.19 [1.17-1.22]	1.20 [1.17-1.23]	1.21 [1.14-1.29]	1.22 [1.19-1.25]	24,237	23,632	16,259	3,011	21,882
K59	1.27 [1.26-1.29]	1.29 [1.27-1.30]	1.27 [1.26-1.29]	1.19 [1.16-1.23]	1.20 [1.18-1.21]	1.20 [1.18-1.21]	1.17 [1.15-1.18]	1.16 [1.14-1.18]	1.15 [1.11-1.18]	1.15 [1.14-1.17]	75,465	65,892	58,307	12,257	68,411
K60	1.52 [1.47-1.58]	1.54 [1.48-1.60]	1.53 [1.46-1.60]	1.40 [1.28-1.53]	1.42 [1.37-1.48]	1.41 [1.36-1.46]	1.38 [1.32-1.43]	1.37 [1.30-1.43]	1.34 [1.22-1.47]	1.35 [1.30-1.41]	7,311	6,701	4,241	1,289	6,070
K61	1.39 [1.33-1.46]	1.39 [1.33-1.46]	1.39 [1.31-1.49]	1.32 [1.20-1.47]	1.29 [1.22-1.36]	1.32 [1.26-1.39]	1.26 [1.19-1.32]	1.25 [1.17-1.34]	1.32 [1.19-1.47]	1.26 [1.19-1.33]	4,318	3,877	2,232	1,034	3,412
K62	1.33 [1.31-1.35]	1.34 [1.31-1.36]	1.31 [1.29-1.33]	1.34 [1.26-1.42]	1.22 [1.20-1.25]	1.25 [1.23-1.27]	1.21 [1.19-1.23]	1.19 [1.17-1.21]	1.26 [1.18-1.35]	1.17 [1.15-1.19]	39,989	38,779	32,850	2,822	33,631
K63	1.27 [1.25-1.30]	1.27 [1.25-1.29]	1.25 [1.23-1.27]	1.40 [1.29-1.53]	1.19 [1.17-1.21]	1.19 [1.17-1.21]	1.14 [1.12-1.16]	1.13 [1.11-1.15]	1.30 [1.19-1.42]	1.14 [1.12-1.16]	38,712	38,142	34,930	1,491	34,387
K64	1.31 [1.29-1.34]	1.31 [1.29-1.34]	1.29 [1.27-1.32]	1.34 [1.23-1.46]	1.22 [1.20-1.25]	1.21 [1.19-1.23]	1.18 [1.15-1.20]	1.16 [1.14-1.19]	1.27 [1.16-1.39]	1.16 [1.14-1.18]	28,431	28,219	24,209	1,558	25,763
K65	1.19 [1.13-1.25]	1.18 [1.12-1.24]	1.17 [1.11-1.23]	1.05 [0.88-1.26]	1.12 [1.07-1.18]	1.16 [1.10-1.21]	1.10 [1.05-1.16]	1.10 [1.04-1.16]	1.06 [0.88-1.28]	1.11 [1.06-1.17]	4,002	3,821	3,397	319	3,510
K66	1.19 [1.16-1.22]	1.20 [1.17-1.23]	1.18 [1.14-1.21]	1.11 [1.02-1.21]	1.11 [1.08-1.14]	1.12 [1.09-1.15]	1.09 [1.06-1.12]	1.07 [1.04-1.11]	1.07 [0.99-1.17]	1.08 [1.05-1.11]	14,367	13,909	10,436	1,499	12,929
K70	1.44 [1.38-1.51]	1.46 [1.40-1.52]	1.46 [1.40-1.53]		1.35 [1.29-1.41]	1.37 [1.31-1.43]	1.29 [1.23-1.35]	1.28 [1.23-1.34]		1.34 [1.28-1.41]	5,266	5,262	4,799	NA	4,536
K71	1.37 [1.15-1.62]	1.40 [1.17-1.67]	1.52 [1.25-1.85]		1.18 [0.98-1.41]	1.29 [1.08-1.54]	1.28 [1.07-1.54]	1.39 [1.14-1.70]		1.16 [0.97-1.39]	337	324	261	NA	300
K72	1.36 [1.28-1.43]	1.35 [1.28-1.43]	1.40 [1.32-1.48]	0.89 [0.67-1.18]	1.30 [1.23-1.38]	1.28 [1.21-1.36]	1.22 [1.15-1.29]	1.25 [1.18-1.33]	0.88 [0.66-1.17]	1.27 [1.20-1.35]	3,232	3,161	2,960	125	2,923
K73	1.48 [1.25-1.75]	1.55 [1.30-1.84]	1.51 [1.25-1.82]		1.40 [1.17-1.67]	1.39 [1.16-1.65]	1.41 [1.18-1.69]	1.36 [1.12-1.64]		1.32 [1.10-1.59]	334	319	276	NA	299
K74	1.46 [1.39-1.52]	1.49 [1.42-1.55]	1.49 [1.42-1.56]		1.37 [1.31-1.43]	1.35 [1.29-1.42]	1.31 [1.25-1.37]	1.30 [1.24-1.37]		1.32 [1.26-1.38]	5,049	5,005	4,744	NA	4,649
K75	1.40 [1.33-1.48]	1.42 [1.34-1.50]	1.41 [1.33-1.49]	1.33 [1.07-1.66]	1.33 [1.26-1.41]	1.30 [1.23-1.38]	1.26 [1.19-1.33]	1.25 [1.18-1.33]	1.29 [1.03-1.63]	1.28 [1.20-1.35]	3,307	3,184	2,850	231	3,003
K76	1.33 [1.31-1.36]	1.34 [1.31-1.36]	1.33 [1.30-1.36]	1.12 [1.00-1.24]	1.25 [1.22-1.28]	1.23 [1.20-1.25]	1.17 [1.14-1.19]	1.16 [1.14-1.19]	1.07 [0.96-1.20]	1.19 [1.16-1.21]	22,874	22,511	20,279	956	20,897
K80	1.16 [1.14-1.18]	1.17 [1.15-1.19]	1.15 [1.13-1.17]	1.05 [0.97-1.12]	1.10 [1.08-1.12]	1.10 [1.09-1.12]	1.08 [1.06-1.09]	1.07 [1.05-1.08]	1.02 [0.95-1.10]	1.06 [1.04-1.08]	39,352	39,031	32,181	1,957	34,401
K81	1.19 [1.15-1.23]	1.19 [1.15-1.24]	1.19 [1.14-1.24]	1.06 [0.89-1.26]	1.13 [1.09-1.18]	1.12 [1.08-1.17]	1.09 [1.05-1.13]	1.09 [1.04-1.13]	1.03 [0.86-1.24]	1.10 [1.05-1.14]	7,288	7,250	6,041	326	6,430
K82	1.18 [1.13-1.23]	1.19 [1.14-1.24]	1.18 [1.13-1.24]	0.99 [0.84-1.17]	1.11 [1.06-1.16]	1.11 [1.07-1.16]	1.09 [1.04-1.14]	1.08 [1.03-1.14]	0.96 [0.81-1.15]	1.07 [1.02-1.12]	5,528	5,479	4,323	344	4,861
K83	1.16 [1.12-1.19]	1.17 [1.13-1.20]	1.16 [1.12-1.20]	1.05 [0.89-1.23]	1.12 [1.09-1.16]	1.11 [1.08-1.15]	1.08 [1.05-1.12]	1.07 [1.04-1.11]	1.01 [0.85-1.19]	1.10 [1.06-1.14]	10,270	10,156	9,035	390	9,314
K85	1.20 [1.16-1.25]	1.21 [1.17-1.26]	1.19 [1.14-1.24]	1.03 [0.89-1.18]	1.13 [1.09-1.18]	1.14 [1.10-1.18]	1.11 [1.07-1.15]	1.08 [1.04-1.13]	1.02 [0.88-1.18]	1.10 [1.06-1.15]	7,005	6,863	5,652	497	6,079
K86	1.21 [1.17-1.27]	1.24 [1.19-1.29]	1.23 [1.18-1.29]	0.86 [0.70-1.05]	1.14 [1.10-1.19]	1.15 [1.10-1.19]	1.12 [1.07-1.17]	1.11 [1.06-1.16]	0.84 [0.69-1.03]	1.11 [1.07-1.16]	5,937	5,821	5,358	244	5,452
K90	1.78 [1.72-1.85]	1.60 [1.53-1.67]	1.61 [1.53-1.70]	2.04 [1.93-2.17]	1.67 [1.61-1.73]	1.56 [1.50-1.62]	1.36 [1.30-1.42]	1.37 [1.30-1.44]	1.76 [1.65-1.87]	1.49 [1.43-1.55]	8,443	5,443	3,833	3,599	7,356
K91	1.29 [1.24-1.35]	1.32 [1.26-1.37]	1.28 [1.22-1.34]	1.06 [0.93-1.21]	1.19 [1.14-1.24]	1.20 [1.15-1.25]	1.18 [1.13-1.23]	1.16 [1.11-1.22]	0.99 [0.86-1.13]	1.13 [1.09-1.18]	5,721	5,347	4,601	581	5,203
K92	1.28 [1.27-1.30]	1.30 [1.28-1.31]	1.28 [1.26-1.30]	1.19 [1.14-1.25]	1.20 [1.18-1.22]	1.21 [1.19-1.23]	1.17 [1.16-1.19]	1.16 [1.14-1.18]	1.13 [1.08-1.19]	1.16 [1.14-1.17]	49,810	47,775	40,587	4,646	44,150
Diseases of the skin and subcutaneous tissue (L00-L99)															
L00	1.54 [1.22-1.93]			1.55 [1.23-1.94]	1.36 [1.04-1.77]	1.54 [1.22-1.93]			1.54 [1.22-1.94]	1.33 [1.01-1.74]	205	NA	NA	195	145
L01	2.28 [2.08-2.51]	2.65 [2.21-3.17]	2.27 [1.83-2.82]	2.22 [2.00-2.48]	2.16 [1.95-2.39]	2.10 [1.91-2.32]	2.19 [1.81-2.65]	1.96 [1.57-2.46]	2.09 [1.87-2.33]	2.01 [1.81-2.23]	1,345	383	245	1,019	1,124
L02	1.38 [1.34-1.42]	1.38 [1.34-1.43]	1.37 [1.32-1.43]	1.29 [1.22-1.36]	1.26 [1.22-1.30]	1.30 [1.26-1.34]	1.23 [1.19-1.27]	1.23 [1.18-1.28]	1.26 [1.19-1.33]	1.23 [1.19-1.27]	12,020	9,781	6,084	3,557	9,776
L03	1.61 [1.58-1.63]	1.64 [1.62-1.67]	1.64 [1.61-1.66]	1.38 [1.32-1.44]	1.49 [1.47-1.52]	1.53 [1.50-1.55]	1.50 [1.48-1.53]	1.49 [1.47-1.52]	1.34 [1.28-1.40]	1.44 [1.42-1.46]	45,791	41,851	37,940	5,185	40,007
L04	1.21 [1.11-1.33]	1.29 [1.07-1.55]	1.37 [1.09-1.71]	1.14 [1.03-1.26]	1.16 [1.06-1.28]	1.19 [1.08-1.30]	1.21 [1.00-1.46]	1.32 [1.05-1.66]	1.12 [1.01-1.25]	1.15 [1.05-1.27]	1,202	280	190	947	1,062
L05	1.15 [1.09-1.21]	1.17 [1.11-1.24]	1.30 [1.15-1.48]	1.09 [1.02-1.17]	1.09 [1.02-1.16]	1.11 [1.05-1.17]	1.08 [1.02-1.14]	1.17 [1.03-1.34]	1.06 [0.98-1.14]	1.09 [1.02-1.16]	3,813	3,135	557	2,154	2,607
L08	1.54 [1.50-1.59]	1.51 [1.46-1.56]	1.51 [1.46-1.56]	1.72 [1.59-1.86]	1.45 [1.41-1.50]	1.47 [1.43-1.52]	1.38 [1.34-1.43]	1.37 [1.32-1.42]	1.68 [1.55-1.82]	1.42 [1.38-1.47]	12,090	10,568	9,505	1,865	10,924
L10	2.28 [1.75-2.95]	2.25 [1.74-2.92]	2.27 [1.74-2.97]		2.00 [1.52-2.64]	2.11 [1.62-2.76]	2.06 [1.57-2.69]	2.01 [1.53-2.66]		1.86 [1.40-2.46]	180	175	164	NA	156
L11	1.67 [1.24-2.25]	1.64 [1.21-2.22]	1.81 [1.33-2.46]		1.64 [1.20-2.25]	1.57 [1.16-2.13]	1.56 [1.15-2.13]	1.77 [1.29-2.42]		1.58 [1.15-2.17]	118	117	114	NA	107
L12	2.86 [2.59-3.16]	2.81 [2.54-3.11]	2.80 [2.54-3.10]		2.64 [2.37-2.93]	2.75 [2.48-3.04]	2.65 [2.39-2.93]	2.63 [2.37-2.91]		2.56 [2.30-2.85]	1,416	1,403	1,391	NA	1,268
L13	3.14 [2.51-3.92]	3.31 [2.62-4.18]	3.00 [2.34-3.86]		2.83 [2.23-3.59]	2.84 [2.26-3.58]	2.99 [2.35-3.79]	2.70 [2.08-3.49]		2.63 [2.06-3.35]	282	253	215	NA	239
L20	24.72 [22.44-27.23]	31.99 [27.28-37.50]	35.83 [29.13-44.08]	18.85 [16.88-21.04]	22.58 [20.37-25.03]	21.74 [19.72-23.97]	27.25 [23.19-32.01]	31.58 [25.62-38.94]	16.21 [14.49-18.13]	19.70 [17.74-21.86]	6,205	2,770	1,797	3,853	5,147
L21	3.29 [2.87-3.77]	3.13 [2.71-3.62]	3.22 [2.77-3.75]	3.22 [2.24-4.64]	2.86 [2.48-3.29]	3.09 [2.69-3.56]	2.85 [2.45-3.30]	2.91 [2.49-3.40]	3.12 [2.12-4.57]	2.72 [2.36-3.15]	776	682	616	114	678
L22	1.05 [0.90-1.22]	1.08 [0.83-1.40]	1.00 [0.77-1.31]	1.01 [0.84-1.21]	1.01 [0.86-1.18]	1.01 [0.87-1.18]	1.02 [0.79-1.33]	0.96 [0.73-1.25]	0.95 [0.78-1.15]	0.99 [0.84-1.16]	424	164	160	264	405
L23	3.75 [3.33-4.22]	3.56 [3.07-4.12]	3.72 [3.14-4.40]	4.13 [3.41-5.01]	3.34 [2.94-3.78]	3.33 [2.94-3.76]	3.19 [2.74-3.71]	3.39 [2.86-4.03]	3.36 [2.75-4.12]	3.01 [2.64-3.42]	1,066	656	492	460	893
L24	1.45 [1.35-1.55]	1.42 [1.32-1.52]	1.41 [1.32-1.51]		1.36 [1.27-1.45]	1.39 [1.30-1.49]	1.31 [1.22-1.41]	1.29 [1.20-1.39]		1.34 [1.25-1.44]	2,601	2,559	2,515	NA	2,507
L25	2.99 [2.63-3.39]	3.18 [2.77-3.66]	3.33 [2.85-3.90]	1.95 [1.49-2.56]	2.69 [2.35-3.08]	2.84 [2.49-3.23]	2.96 [2.57-3.41]	3.10 [2.64-3.64]	1.80 [1.36-2.37]	2.61 [2.27-2.99]	847	730	573	164	713
L26	6.48 [4.30-9.76]	6.67 [4.43-10.04]			6.43 [4.17-9.91]	6.06 [3.99-9.21]	5.73 [3.75-8.75]			6.03 [3.88-9.38]	121	118	NA	NA	102
L27	1.75 [1.65-1.86]	1.60 [1.50-1.71]	1.60 [1.49-1.72]	2.18 [1.93-2.47]	1.62 [1.52-1.73]	1.61 [1.51-1.71]	1.45 [1.36-1.56]	1.46 [1.36-1.58]	1.91 [1.68-2.18]	1.53 [1.43-1.63]	3,074	2,429	2,008	807	2,727
L28	4.46 [4.06-4.91]	4.18 [3.79-4.61]	3.99 [3.59-4.42]	6.73 [4.77-9.50]	3.75 [3.39-4.14]	4.14 [3.75-4.56]	3.82 [3.45-4.22]	3.61 [3.25-4.01]	6.04 [4.24-8.60]	3.51 [3.17-3.89]	1,757	1,585	1,387	208	1,480
L29	1.72 [1.64-1.80]	1.68 [1.60-1.76]	1.99 [1.87-2.12]	1.46 [1.31-1.62]	1.59 [1.51-1.67]	1.60 [1.52-1.68]	1.50 [1.42-1.58]	1.78 [1.67-1.90]	1.36 [1.21-1.51]	1.50 [1.42-1.58]	4,866	4,324	2,764	1,002	4,307
L30	10.30 [10.10-10.50]	10.25 [9.99-10.52]	9.44 [9.16-9.73]	9.92 [9.66-10.19]	9.24 [9.06-9.43]	9.48 [9.30-9.67]	9.13 [8.89-9.38]	8.61 [8.35-8.89]	9.12 [8.88-9.37]	8.55 [8.37-8.72]	77,273	40,673	27,658	41,677	65,364
L40	2.23 [2.17-2.30]	2.19 [2.13-2.26]	2.21 [2.14-2.28]	2.04 [1.84-2.26]	2.11 [2.04-2.17]	2.13 [2.07-2.20]	2.02 [1.96-2.08]	2.03 [1.97-2.10]	2.01 [1.80-2.23]	2.04 [1.98-2.11]	14,006	13,506	11,116	1,195	11,883
L42	1.54 [1.22-1.95]	1.63 [1.27-2.11]	1.79 [1.32-2.43]		1.42 [1.10-1.83]	1.44 [1.13-1.83]	1.40 [1.07-1.83]	1.61 [1.18-2.22]		1.32 [1.02-1.72]	194	166	121	NA	163
L43	2.18 [1.99-2.38]	2.15 [1.97-2.35]	2.15 [1.96-2.36]		1.99 [1.81-2.18]	2.03 [1.86-2.23]	1.98 [1.81-2.17]	1.99 [1.81-2.18]		1.89 [1.72-2.08]	1,468	1,442	1,332	NA	1,256
L44	3.12 [2.21-4.41]					2.92 [2.05-4.16]					109	NA	NA	NA	NA
L50	1.93 [1.83-2.03]	1.78 [1.65-1.92]	1.86 [1.70-2.03]	2.03 [1.89-2.17]	1.89 [1.79-2.00]	1.64 [1.55-1.73]	1.45 [1.34-1.56]	1.50 [1.37-1.65]	1.75 [1.62-1.88]	1.60 [1.51-1.70]	4,198	1,906	1,317	2,516	3,728
L51	1.28 [1.14-1.43]	1.83 [1.54-2.16]	2.03 [1.67-2.46]	0.97 [0.83-1.13]	1.24 [1.10-1.40]	1.21 [1.07-1.35]	1.60 [1.35-1.91]	1.77 [1.45-2.16]	0.95 [0.82-1.11]	1.18 [1.05-1.34]	767	378	296	416	656
L52	1.49 [1.25-1.79]	1.50 [1.22-1.84]	1.49 [1.12-1.96]	1.47 [1.11-1.96]	1.36 [1.12-1.66]	1.38 [1.15-1.66]	1.33 [1.07-1.64]	1.28 [0.96-1.71]	1.36 [1.01-1.82]	1.24 [1.01-1.52]	329	233	127	139	264
L53	1.75 [1.66-1.84]	1.81 [1.71-1.91]	1.82 [1.72-1.93]	1.45 [1.29-1.63]	1.61 [1.53-1.69]	1.66 [1.58-1.75]	1.65 [1.56-1.75]	1.66 [1.57-1.77]	1.37 [1.21-1.54]	1.56 [1.47-1.64]	4,468	3,852	3,354	791	3,949
L55	1.15 [0.86-1.53]				1.12 [0.81-1.53]	1.10 [0.82-1.48]				1.10 [0.79-1.52]	118	NA	NA	NA	101
L56	2.80 [2.22-3.53]	2.85 [2.23-3.63]	2.99 [2.29-3.90]		2.36 [1.85-3.00]	2.65 [2.09-3.36]	2.58 [2.01-3.31]	2.67 [2.03-3.51]		2.22 [1.73-2.86]	238	212	176	NA	203
L57	1.32 [1.27-1.37]	1.31 [1.26-1.35]	1.32 [1.28-1.37]		1.25 [1.20-1.30]	1.25 [1.21-1.30]	1.22 [1.18-1.27]	1.24 [1.19-1.28]		1.20 [1.15-1.24]	7,794	7,772	7,653	NA	6,959
L59	1.42 [1.07-1.89]	1.44 [1.07-1.93]	1.40 [1.03-1.91]		1.21 [0.89-1.65]	1.38 [1.02-1.85]	1.41 [1.04-1.91]	1.40 [1.01-1.92]		1.25 [0.91-1.72]	126	117	104	NA	104
L60	1.41 [1.34-1.49]	1.48 [1.39-1.58]	1.51 [1.39-1.63]	1.32 [1.22-1.43]	1.33 [1.25-1.41]	1.30 [1.23-1.38]	1.33 [1.24-1.42]	1.32 [1.22-1.44]	1.24 [1.14-1.34]	1.24 [1.17-1.32]	3,703	2,359	1,553	1,740	2,908
L63	2.87 [2.32-3.56]	2.84 [2.24-3.59]	2.62 [1.97-3.48]		2.70 [2.15-3.40]	2.27 [1.80-2.85]	2.08 [1.61-2.68]	2.06 [1.53-2.79]		2.19 [1.71-2.79]	290	234	151	NA	243
L65	1.99 [1.76-2.25]	2.04 [1.78-2.33]	2.09 [1.78-2.45]	1.78 [1.41-2.24]	1.89 [1.66-2.15]	1.75 [1.54-1.99]	1.73 [1.50-1.99]	1.77 [1.50-2.08]	1.64 [1.29-2.08]	1.68 [1.47-1.92]	805	634	451	229	693
L66	1.73 [1.31-2.27]	1.93 [1.44-2.57]	1.82 [1.32-2.51]		1.72 [1.29-2.29]	1.62 [1.22-2.14]	1.79 [1.33-2.42]	1.64 [1.18-2.28]		1.62 [1.21-2.17]	151	135	105	NA	132
L68	1.25 [1.00-1.56]	1.28 [1.00-1.64]			1.10 [0.86-1.41]	1.19 [0.95-1.49]	1.12 [0.87-1.45]			1.05 [0.82-1.35]	211	157	NA	NA	167
L70	1.45 [1.30-1.62]	1.42 [1.25-1.61]	1.44 [1.13-1.83]	1.37 [1.17-1.59]	1.29 [1.14-1.46]	1.32 [1.18-1.49]	1.22 [1.07-1.39]	1.24 [0.97-1.60]	1.27 [1.08-1.49]	1.21 [1.07-1.37]	887	622	168	512	718
L71	1.55 [1.39-1.72]	1.59 [1.43-1.78]	1.52 [1.36-1.70]		1.50 [1.34-1.68]	1.44 [1.29-1.61]	1.49 [1.33-1.66]	1.40 [1.25-1.57]		1.43 [1.27-1.60]	890	873	798	NA	784
L72	1.16 [1.13-1.19]	1.17 [1.14-1.21]	1.17 [1.13-1.21]	1.10 [1.02-1.19]	1.09 [1.06-1.13]	1.11 [1.08-1.14]	1.09 [1.06-1.12]	1.09 [1.06-1.14]	1.07 [0.99-1.16]	1.07 [1.03-1.10]	10,923	10,002	7,136	1,763	8,434
L73	1.57 [1.46-1.70]	1.56 [1.44-1.68]	1.58 [1.42-1.75]	1.32 [1.12-1.55]	1.37 [1.26-1.49]	1.44 [1.33-1.56]	1.35 [1.24-1.46]	1.38 [1.24-1.55]	1.24 [1.05-1.47]	1.31 [1.20-1.42]	1,777	1,630	848	404	1,497
L80	1.97 [1.69-2.29]	1.85 [1.57-2.18]	1.90 [1.59-2.27]	2.46 [1.72-3.53]	1.83 [1.56-2.15]	1.80 [1.54-2.11]	1.65 [1.40-1.96]	1.67 [1.39-2.01]	2.34 [1.60-3.42]	1.73 [1.46-2.04]	492	410	343	106	427
L81	1.29 [1.20-1.38]	1.29 [1.19-1.39]	1.27 [1.17-1.38]	1.50 [1.26-1.78]	1.24 [1.15-1.34]	1.23 [1.14-1.32]	1.20 [1.11-1.30]	1.19 [1.09-1.30]	1.45 [1.21-1.72]	1.19 [1.10-1.29]	1,903	1,576	1,340	395	1,627
L82	1.22 [1.17-1.28]	1.25 [1.19-1.31]	1.26 [1.20-1.31]		1.18 [1.13-1.24]	1.16 [1.11-1.21]	1.17 [1.12-1.22]	1.17 [1.12-1.23]		1.13 [1.07-1.18]	4,915	4,889	4,688	NA	4,220
L83	1.67 [1.43-1.96]	1.82 [1.51-2.18]	1.71 [1.41-2.08]	1.58 [1.18-2.12]	1.55 [1.31-1.83]	1.56 [1.33-1.84]	1.60 [1.32-1.93]	1.56 [1.27-1.90]	1.51 [1.11-2.04]	1.46 [1.23-1.74]	456	322	278	150	387
L84	1.49 [1.31-1.69]	1.47 [1.29-1.67]	1.50 [1.31-1.72]		1.32 [1.16-1.51]	1.39 [1.22-1.58]	1.33 [1.16-1.51]	1.35 [1.18-1.55]		1.26 [1.10-1.44]	634	609	550	NA	566
L85	1.62 [1.52-1.73]	1.57 [1.47-1.68]	1.55 [1.45-1.66]	2.18 [1.67-2.84]	1.56 [1.46-1.67]	1.54 [1.44-1.65]	1.46 [1.37-1.57]	1.45 [1.35-1.55]	2.18 [1.66-2.87]	1.51 [1.41-1.62]	2,552	2,405	2,271	183	2,257
L88	1.56 [1.20-2.04]	1.68 [1.29-2.19]	1.68 [1.25-2.26]		1.33 [1.02-1.74]	1.37 [1.04-1.80]	1.40 [1.07-1.85]	1.45 [1.07-1.97]		1.21 [0.92-1.60]	151	150	119	NA	139
L89	1.22 [1.20-1.25]	1.21 [1.19-1.24]	1.22 [1.19-1.24]	0.99 [0.82-1.20]	1.18 [1.16-1.21]	1.20 [1.17-1.22]	1.15 [1.13-1.18]	1.15 [1.13-1.17]	0.97 [0.80-1.18]	1.18 [1.16-1.21]	26,249	26,064	25,754	284	24,650
L90	1.29 [1.24-1.34]	1.33 [1.28-1.39]	1.32 [1.25-1.38]	1.04 [0.94-1.16]	1.20 [1.15-1.26]	1.22 [1.17-1.27]	1.24 [1.18-1.29]	1.21 [1.15-1.28]	1.02 [0.92-1.14]	1.16 [1.11-1.21]	5,613	5,036	3,915	880	4,909
L91	1.35 [1.26-1.44]	1.35 [1.25-1.46]	1.36 [1.23-1.50]	1.26 [1.12-1.40]	1.22 [1.13-1.32]	1.27 [1.19-1.36]	1.26 [1.16-1.36]	1.25 [1.13-1.38]	1.25 [1.11-1.40]	1.18 [1.09-1.27]	2,210	1,609	978	826	1,765
L92	1.30 [1.20-1.40]	1.40 [1.28-1.52]	1.37 [1.24-1.52]	1.11 [0.97-1.28]	1.21 [1.11-1.31]	1.21 [1.12-1.31]	1.26 [1.15-1.37]	1.24 [1.12-1.38]	1.04 [0.90-1.20]	1.15 [1.06-1.25]	1,723	1,322	957	531	1,468
L93	1.86 [1.66-2.10]	1.86 [1.66-2.10]	1.84 [1.62-2.10]		1.68 [1.49-1.90]	1.67 [1.48-1.88]	1.61 [1.42-1.82]	1.58 [1.38-1.81]		1.55 [1.36-1.75]	786	768	620	NA	700
L94	1.55 [1.35-1.78]	1.55 [1.33-1.81]	1.81 [1.53-2.14]	1.53 [1.14-2.06]	1.46 [1.26-1.69]	1.45 [1.26-1.68]	1.41 [1.20-1.65]	1.60 [1.34-1.90]	1.54 [1.13-2.09]	1.37 [1.18-1.59]	541	426	368	136	472
L95	1.61 [1.42-1.83]	1.78 [1.56-2.04]	1.70 [1.47-1.96]		1.51 [1.32-1.73]	1.50 [1.31-1.71]	1.63 [1.42-1.87]	1.53 [1.32-1.77]		1.42 [1.23-1.63]	663	606	526	NA	572
L97	1.54 [1.51-1.58]	1.53 [1.50-1.57]	1.53 [1.50-1.57]	1.28 [0.94-1.75]	1.45 [1.42-1.49]	1.49 [1.46-1.53]	1.42 [1.39-1.46]	1.42 [1.38-1.45]	1.34 [0.97-1.85]	1.43 [1.40-1.46]	21,630	21,576	21,276	108	19,873
L98	1.39 [1.36-1.42]	1.40 [1.36-1.44]	1.39 [1.35-1.43]	1.28 [1.19-1.38]	1.30 [1.27-1.34]	1.32 [1.29-1.36]	1.30 [1.27-1.33]	1.29 [1.25-1.33]	1.25 [1.16-1.35]	1.26 [1.23-1.29]	17,334	15,883	14,075	1,917	15,018
Diseases of the musculoskeletal system and connective tissue (M00-M99)															
M00	1.48 [1.39-1.59]	1.50 [1.39-1.61]	1.54 [1.42-1.66]	1.40 [1.20-1.64]	1.35 [1.26-1.45]	1.41 [1.32-1.51]	1.37 [1.27-1.48]	1.41 [1.30-1.52]	1.37 [1.16-1.61]	1.31 [1.22-1.41]	2,261	1,904	1,742	421	1,990
M02	1.22 [1.07-1.40]	1.42 [1.18-1.72]	1.34 [1.07-1.67]	1.13 [0.94-1.34]	1.17 [1.01-1.35]	1.18 [1.03-1.35]	1.29 [1.06-1.57]	1.23 [0.97-1.54]	1.11 [0.93-1.33]	1.14 [0.98-1.32]	556	279	191	313	464
M05	1.40 [1.32-1.50]	1.39 [1.30-1.48]	1.37 [1.28-1.47]		1.24 [1.16-1.32]	1.35 [1.27-1.44]	1.30 [1.22-1.39]	1.29 [1.21-1.38]		1.21 [1.13-1.29]	2,360	2,359	2,210	NA	2,108
M06	1.40 [1.37-1.44]	1.40 [1.36-1.43]	1.39 [1.36-1.42]	1.29 [1.06-1.57]	1.31 [1.28-1.34]	1.31 [1.28-1.34]	1.27 [1.23-1.30]	1.26 [1.23-1.29]	1.21 [0.99-1.49]	1.24 [1.21-1.27]	17,481	17,434	16,286	282	15,317
M07	1.75 [1.64-1.87]	1.70 [1.59-1.82]	1.70 [1.59-1.83]	1.52 [1.14-2.03]	1.60 [1.49-1.71]	1.62 [1.52-1.74]	1.51 [1.41-1.61]	1.50 [1.40-1.62]	1.46 [1.08-1.97]	1.50 [1.40-1.61]	2,456	2,412	1,965	138	2,147
M08	1.33 [1.19-1.48]	1.38 [1.09-1.73]		1.34 [1.19-1.51]	1.25 [1.10-1.41]	1.28 [1.14-1.43]	1.26 [1.00-1.60]		1.30 [1.15-1.47]	1.23 [1.08-1.39]	852	183	NA	723	706
M10	1.36 [1.33-1.39]	1.36 [1.33-1.39]	1.36 [1.33-1.39]		1.27 [1.24-1.30]	1.29 [1.26-1.32]	1.24 [1.21-1.27]	1.23 [1.20-1.26]		1.22 [1.19-1.25]	19,181	19,179	18,861	NA	17,324
M11	1.29 [1.20-1.39]	1.30 [1.21-1.40]	1.29 [1.20-1.38]		1.26 [1.17-1.35]	1.21 [1.12-1.30]	1.19 [1.11-1.28]	1.17 [1.09-1.26]		1.20 [1.12-1.29]	2,128	2,129	2,100	NA	1,994
M12	1.40 [1.21-1.61]	1.33 [1.15-1.54]	1.36 [1.16-1.61]		1.25 [1.08-1.45]	1.27 [1.10-1.48]	1.17 [1.01-1.37]	1.16 [0.98-1.38]		1.21 [1.04-1.40]	475	439	350	NA	430
M13	1.34 [1.32-1.36]	1.33 [1.31-1.35]	1.33 [1.31-1.35]	1.36 [1.19-1.55]	1.26 [1.24-1.27]	1.24 [1.22-1.26]	1.19 [1.17-1.21]	1.19 [1.17-1.21]	1.27 [1.11-1.45]	1.18 [1.17-1.20]	48,665	48,345	46,962	640	44,324
M14	1.61 [1.41-1.85]	1.62 [1.42-1.86]	1.62 [1.41-1.87]		1.32 [1.15-1.51]	1.46 [1.28-1.68]	1.33 [1.16-1.54]	1.31 [1.14-1.52]		1.25 [1.09-1.43]	567	557	526	NA	528
M15	1.38 [1.34-1.41]	1.37 [1.34-1.41]	1.36 [1.33-1.40]		1.27 [1.24-1.30]	1.26 [1.23-1.29]	1.22 [1.19-1.25]	1.21 [1.18-1.24]		1.19 [1.16-1.22]	18,016	18,006	17,845	NA	16,796
M16	1.13 [1.10-1.15]	1.12 [1.10-1.14]	1.11 [1.09-1.14]	1.12 [0.84-1.49]	1.07 [1.05-1.09]	1.09 [1.07-1.11]	1.07 [1.05-1.09]	1.06 [1.04-1.08]	1.08 [0.80-1.46]	1.05 [1.03-1.07]	25,902	25,881	25,397	127	22,202
M17	1.25 [1.23-1.27]	1.25 [1.23-1.27]	1.25 [1.23-1.27]	1.14 [0.96-1.35]	1.17 [1.15-1.19]	1.18 [1.16-1.20]	1.16 [1.14-1.18]	1.15 [1.13-1.17]	1.09 [0.91-1.30]	1.12 [1.10-1.14]	45,645	45,589	44,018	348	39,337
M18	1.43 [1.35-1.52]	1.42 [1.33-1.50]	1.44 [1.35-1.53]		1.28 [1.20-1.36]	1.27 [1.19-1.35]	1.22 [1.14-1.29]	1.24 [1.17-1.32]		1.17 [1.10-1.25]	2,713	2,711	2,671	NA	2,503
M19	1.36 [1.34-1.37]	1.36 [1.34-1.38]	1.35 [1.34-1.37]	1.26 [1.05-1.51]	1.26 [1.24-1.27]	1.25 [1.24-1.27]	1.21 [1.20-1.23]	1.20 [1.19-1.22]	1.14 [0.94-1.39]	1.19 [1.17-1.20]	76,155	76,146	74,683	319	70,181
M20	1.30 [1.26-1.34]	1.30 [1.26-1.34]	1.31 [1.26-1.35]	1.19 [1.08-1.32]	1.19 [1.16-1.23]	1.22 [1.18-1.26]	1.20 [1.16-1.24]	1.21 [1.17-1.25]	1.15 [1.04-1.27]	1.14 [1.10-1.17]	11,087	10,431	8,993	1,070	9,169
M21	1.19 [1.15-1.24]	1.24 [1.19-1.29]	1.23 [1.18-1.29]	1.00 [0.92-1.09]	1.11 [1.07-1.16]	1.12 [1.07-1.16]	1.14 [1.09-1.19]	1.13 [1.08-1.19]	0.97 [0.88-1.06]	1.07 [1.03-1.12]	6,537	5,393	4,757	1,371	5,909
M22	1.32 [1.24-1.41]	1.35 [1.26-1.44]	1.33 [1.21-1.46]	1.26 [1.10-1.44]	1.26 [1.17-1.36]	1.20 [1.12-1.29]	1.18 [1.10-1.27]	1.18 [1.07-1.29]	1.18 [1.03-1.35]	1.17 [1.09-1.26]	2,218	1,919	1,042	618	1,797
M23	1.22 [1.19-1.25]	1.22 [1.20-1.25]	1.23 [1.20-1.27]	1.14 [1.08-1.21]	1.12 [1.10-1.15]	1.14 [1.12-1.17]	1.13 [1.10-1.15]	1.13 [1.10-1.16]	1.09 [1.03-1.16]	1.07 [1.04-1.09]	20,542	19,394	14,216	3,153	15,635
M24	1.26 [1.22-1.30]	1.29 [1.24-1.33]	1.29 [1.23-1.35]	1.15 [1.08-1.23]	1.15 [1.11-1.19]	1.17 [1.13-1.21]	1.16 [1.11-1.20]	1.16 [1.11-1.21]	1.10 [1.03-1.18]	1.10 [1.06-1.14]	8,753	7,438	4,819	2,395	7,397
M25	1.29 [1.27-1.31]	1.30 [1.28-1.32]	1.29 [1.26-1.31]	1.21 [1.16-1.25]	1.20 [1.18-1.22]	1.20 [1.18-1.22]	1.17 [1.15-1.18]	1.15 [1.14-1.17]	1.15 [1.10-1.19]	1.14 [1.13-1.16]	51,040	45,901	39,263	7,314	45,475
M30	1.39 [1.21-1.59]	1.47 [1.20-1.82]	1.42 [1.13-1.78]	1.28 [1.07-1.53]	1.32 [1.13-1.53]	1.28 [1.11-1.48]	1.23 [0.99-1.53]	1.18 [0.93-1.50]	1.29 [1.08-1.55]	1.23 [1.06-1.43]	523	227	192	309	454
M31	1.34 [1.26-1.42]	1.34 [1.26-1.43]	1.33 [1.25-1.42]		1.25 [1.17-1.33]	1.26 [1.18-1.34]	1.24 [1.16-1.32]	1.22 [1.15-1.30]		1.18 [1.10-1.26]	2,711	2,681	2,594	NA	2,394
M32	1.79 [1.65-1.95]	1.75 [1.61-1.90]	1.69 [1.54-1.86]	1.73 [1.30-2.30]	1.62 [1.49-1.76]	1.63 [1.49-1.77]	1.52 [1.39-1.65]	1.48 [1.34-1.63]	1.73 [1.29-2.32]	1.49 [1.36-1.63]	1,578	1,517	1,106	150	1,379
M33	2.07 [1.75-2.45]	2.10 [1.76-2.50]	2.02 [1.67-2.43]		1.90 [1.58-2.29]	2.00 [1.69-2.38]	1.98 [1.65-2.37]	1.92 [1.58-2.32]		1.81 [1.50-2.19]	403	354	306	NA	315
M34	1.75 [1.53-2.00]	1.66 [1.45-1.89]	1.65 [1.44-1.90]		1.55 [1.35-1.78]	1.62 [1.41-1.85]	1.49 [1.30-1.71]	1.49 [1.29-1.72]		1.46 [1.26-1.68]	605	585	529	NA	525
M35	1.44 [1.40-1.48]	1.44 [1.40-1.49]	1.43 [1.39-1.47]	1.43 [1.31-1.56]	1.35 [1.31-1.39]	1.33 [1.29-1.36]	1.31 [1.27-1.34]	1.30 [1.26-1.34]	1.32 [1.21-1.45]	1.27 [1.23-1.30]	14,083	13,063	11,863	1,478	12,463
M36	1.43 [1.07-1.89]	1.41 [1.03-1.93]			1.29 [0.97-1.73]	1.30 [0.97-1.74]	1.27 [0.92-1.76]			1.24 [0.92-1.67]	123	102	NA	NA	118

Supplementary Table 3: Hazard ratios and events from all cohorts

	Hazard ratio (99% confidence interval)										Events (in exposed)				
	crude					adjusted									
Outcome	any age	18+	40+	<18	hosp.	any age	18+	40+	<18	hosp.	any age	18+	40+	<18	hosp.
M40	1.25 [1.18-1.33]	1.24 [1.17-1.32]	1.24 [1.17-1.32]	1.04 [0.82-1.32]	1.18 [1.11-1.25]	1.18 [1.11-1.25]	1.14 [1.07-1.21]	1.15 [1.08-1.22]	0.98 [0.77-1.26]	1.13 [1.06-1.20]	3,017	2,897	2,744	180	2,846
M41	1.24 [1.20-1.28]	1.27 [1.23-1.32]	1.27 [1.22-1.32]	1.07 [0.99-1.15]	1.14 [1.10-1.18]	1.16 [1.12-1.20]	1.16 [1.12-1.21]	1.16 [1.11-1.21]	1.03 [0.96-1.12]	1.09 [1.05-1.13]	8,862	7,531	6,535	1,747	8,018
M42	1.13 [0.89-1.44]	1.22 [0.94-1.57]			1.14 [0.89-1.46]	0.98 [0.76-1.26]	1.01 [0.77-1.32]			1.02 [0.79-1.32]	167	144	NA	NA	151
M43	1.29 [1.24-1.34]	1.29 [1.24-1.34]	1.30 [1.25-1.35]	1.02 [0.90-1.17]	1.18 [1.13-1.22]	1.18 [1.14-1.23]	1.14 [1.09-1.19]	1.15 [1.10-1.19]	0.98 [0.85-1.12]	1.10 [1.06-1.15]	7,046	6,623	6,121	575	6,468
M45	1.50 [1.40-1.62]	1.56 [1.45-1.69]	1.48 [1.37-1.61]		1.42 [1.31-1.54]	1.39 [1.29-1.50]	1.39 [1.29-1.50]	1.31 [1.20-1.42]		1.33 [1.23-1.44]	1,799	1,796	1,542	NA	1,578
M46	1.42 [1.36-1.48]	1.41 [1.36-1.48]	1.41 [1.35-1.47]	1.10 [0.83-1.45]	1.29 [1.24-1.35]	1.28 [1.22-1.33]	1.22 [1.17-1.28]	1.21 [1.16-1.27]	1.08 [0.81-1.44]	1.20 [1.15-1.26]	5,856	5,775	5,440	135	5,426
M47	1.35 [1.33-1.38]	1.36 [1.34-1.38]	1.35 [1.32-1.37]	1.07 [0.85-1.35]	1.26 [1.23-1.28]	1.23 [1.21-1.26]	1.19 [1.17-1.21]	1.18 [1.16-1.20]	1.00 [0.79-1.27]	1.18 [1.16-1.20]	32,121	32,101	30,887	193	29,828
M48	1.29 [1.26-1.32]	1.29 [1.26-1.32]	1.29 [1.26-1.32]	1.12 [0.88-1.41]	1.20 [1.17-1.23]	1.19 [1.16-1.22]	1.15 [1.12-1.17]	1.14 [1.11-1.16]	1.05 [0.82-1.33]	1.14 [1.11-1.16]	18,247	18,194	17,646	194	16,947
M49	1.08 [0.95-1.23]	1.07 [0.94-1.21]	1.16 [1.01-1.32]		1.08 [0.94-1.24]	1.06 [0.93-1.21]	1.03 [0.90-1.17]	1.12 [0.98-1.28]		1.09 [0.95-1.25]	585	575	548	NA	515
M50	1.33 [1.27-1.40]	1.32 [1.26-1.38]	1.33 [1.27-1.39]		1.21 [1.16-1.27]	1.22 [1.17-1.28]	1.14 [1.09-1.20]	1.15 [1.10-1.21]		1.15 [1.09-1.20]	4,856	4,851	4,448	NA	4,460
M51	1.30 [1.28-1.33]	1.30 [1.27-1.33]	1.32 [1.29-1.34]	1.24 [1.11-1.38]	1.20 [1.18-1.23]	1.19 [1.16-1.21]	1.13 [1.11-1.15]	1.14 [1.12-1.17]	1.17 [1.04-1.30]	1.13 [1.11-1.16]	23,386	23,257	19,588	925	21,018
M53	1.26 [1.16-1.36]	1.27 [1.17-1.38]	1.30 [1.18-1.42]	1.03 [0.78-1.36]	1.15 [1.06-1.25]	1.12 [1.03-1.22]	1.08 [0.99-1.18]	1.09 [0.99-1.21]	0.99 [0.75-1.31]	1.06 [0.97-1.16]	1,485	1,418	1,097	137	1,341
M54	1.30 [1.28-1.32]	1.30 [1.28-1.32]	1.30 [1.28-1.32]	1.15 [1.10-1.19]	1.19 [1.17-1.20]	1.19 [1.17-1.21]	1.13 [1.12-1.15]	1.14 [1.12-1.16]	1.09 [1.04-1.14]	1.12 [1.11-1.14]	60,233	57,895	46,321	5,857	53,676
M60	1.45 [1.33-1.58]	1.48 [1.34-1.63]	1.48 [1.33-1.65]	1.35 [1.15-1.59]	1.38 [1.26-1.52]	1.38 [1.27-1.51]	1.36 [1.23-1.51]	1.38 [1.24-1.54]	1.31 [1.11-1.55]	1.35 [1.23-1.49]	1,388	1,020	865	410	1,221
M62	1.22 [1.17-1.28]	1.26 [1.20-1.33]	1.25 [1.19-1.32]	0.99 [0.88-1.12]	1.14 [1.09-1.19]	1.16 [1.11-1.22]	1.16 [1.10-1.22]	1.16 [1.10-1.23]	0.94 [0.83-1.06]	1.11 [1.06-1.17]	5,043	4,523	3,883	738	4,560
M65	1.36 [1.32-1.40]	1.35 [1.31-1.39]	1.35 [1.30-1.39]	1.26 [1.17-1.37]	1.26 [1.22-1.30]	1.25 [1.21-1.29]	1.21 [1.17-1.25]	1.20 [1.16-1.24]	1.23 [1.13-1.33]	1.19 [1.15-1.22]	10,874	9,568	8,130	1,723	9,468
M66	1.29 [1.17-1.42]	1.31 [1.18-1.44]	1.30 [1.17-1.44]		1.16 [1.05-1.29]	1.21 [1.10-1.34]	1.21 [1.09-1.34]	1.18 [1.06-1.31]		1.11 [1.00-1.24]	1,022	1,012	919	NA	866
M67	1.27 [1.22-1.31]	1.30 [1.25-1.35]	1.33 [1.27-1.39]	1.13 [1.05-1.21]	1.17 [1.12-1.21]	1.19 [1.14-1.23]	1.19 [1.14-1.24]	1.21 [1.15-1.26]	1.08 [1.01-1.16]	1.11 [1.07-1.16]	7,633	6,111	4,364	2,036	6,296
M70	1.44 [1.37-1.51]	1.44 [1.37-1.52]	1.43 [1.36-1.51]	1.16 [0.93-1.46]	1.30 [1.23-1.37]	1.31 [1.25-1.38]	1.28 [1.21-1.34]	1.26 [1.20-1.33]	1.13 [0.89-1.43]	1.21 [1.15-1.27]	4,283	4,180	3,719	208	3,753
M71	1.34 [1.26-1.42]	1.33 [1.25-1.41]	1.32 [1.24-1.41]	1.51 [1.09-2.08]	1.26 [1.18-1.35]	1.24 [1.16-1.32]	1.20 [1.13-1.28]	1.19 [1.11-1.27]	1.44 [1.04-2.02]	1.19 [1.11-1.27]	2,600	2,544	2,355	107	2,333
M72	1.12 [1.07-1.17]	1.12 [1.07-1.17]	1.12 [1.07-1.17]	1.23 [0.91-1.67]	1.06 [1.01-1.11]	1.08 [1.03-1.13]	1.07 [1.02-1.11]	1.07 [1.02-1.12]	1.18 [0.85-1.62]	1.04 [0.99-1.09]	5,021	4,955	4,699	114	4,137
M75	1.32 [1.28-1.35]	1.32 [1.29-1.35]	1.32 [1.29-1.36]	1.18 [1.01-1.37]	1.20 [1.17-1.23]	1.20 [1.17-1.23]	1.17 [1.14-1.20]	1.17 [1.14-1.20]	1.12 [0.96-1.31]	1.13 [1.10-1.16]	16,137	16,023	14,523	440	14,089
M76	1.42 [1.31-1.54]	1.41 [1.29-1.53]	1.41 [1.28-1.55]	1.24 [0.95-1.63]	1.32 [1.21-1.44]	1.28 [1.18-1.40]	1.23 [1.12-1.34]	1.21 [1.10-1.33]	1.13 [0.85-1.50]	1.22 [1.11-1.33]	1,443	1,367	1,085	151	1,302
M77	1.47 [1.40-1.56]	1.48 [1.40-1.56]	1.47 [1.39-1.55]	0.95 [0.73-1.26]	1.34 [1.26-1.41]	1.33 [1.26-1.40]	1.29 [1.22-1.36]	1.27 [1.19-1.35]	0.88 [0.66-1.17]	1.24 [1.17-1.31]	3,405	3,360	2,884	136	3,032
M79	1.39 [1.37-1.41]	1.41 [1.39-1.43]	1.41 [1.39-1.44]	1.20 [1.15-1.25]	1.28 [1.27-1.30]	1.27 [1.25-1.29]	1.22 [1.21-1.24]	1.23 [1.21-1.25]	1.15 [1.10-1.20]	1.20 [1.19-1.22]	53,229	49,622	41,362	5,874	47,643
M80	1.28 [1.23-1.34]	1.29 [1.23-1.34]	1.30 [1.24-1.35]		1.22 [1.17-1.28]	1.22 [1.16-1.27]	1.18 [1.13-1.23]	1.19 [1.14-1.24]		1.17 [1.12-1.22]	5,833	5,806	5,748	NA	5,339
M81	1.31 [1.28-1.33]	1.30 [1.28-1.32]	1.30 [1.28-1.32]	1.38 [1.09-1.75]	1.23 [1.21-1.25]	1.22 [1.20-1.24]	1.17 [1.15-1.19]	1.18 [1.16-1.20]	1.30 [1.02-1.66]	1.16 [1.14-1.18]	35,991	35,913	35,416	198	33,098
M83	1.34 [1.09-1.65]	1.23 [1.00-1.52]	1.28 [1.02-1.59]		1.29 [1.04-1.60]	1.22 [0.98-1.51]	1.13 [0.91-1.39]	1.16 [0.92-1.45]		1.22 [0.98-1.52]	232	231	208	NA	216
M84	1.23 [1.17-1.28]	1.25 [1.19-1.31]	1.26 [1.20-1.33]	1.11 [1.00-1.23]	1.15 [1.10-1.20]	1.16 [1.11-1.21]	1.14 [1.09-1.20]	1.16 [1.10-1.22]	1.08 [0.96-1.20]	1.13 [1.07-1.18]	5,167	4,683	3,741	886	4,392
M85	1.31 [1.27-1.36]	1.32 [1.27-1.37]	1.29 [1.25-1.34]	1.18 [1.01-1.39]	1.24 [1.19-1.28]	1.20 [1.16-1.25]	1.17 [1.13-1.22]	1.16 [1.11-1.20]	1.13 [0.96-1.33]	1.16 [1.12-1.20]	8,162	7,888	7,406	435	7,679
M86	1.37 [1.30-1.44]	1.38 [1.31-1.46]	1.37 [1.30-1.45]	1.36 [1.19-1.56]	1.28 [1.21-1.35]	1.31 [1.25-1.38]	1.25 [1.18-1.32]	1.23 [1.16-1.30]	1.33 [1.16-1.53]	1.26 [1.19-1.33]	3,878	3,351	3,129	604	3,536
M87	1.28 [1.18-1.39]	1.27 [1.17-1.38]	1.28 [1.17-1.39]	1.22 [0.93-1.59]	1.16 [1.07-1.26]	1.21 [1.11-1.31]	1.16 [1.07-1.26]	1.16 [1.06-1.27]	1.16 [0.88-1.53]	1.13 [1.04-1.23]	1,542	1,451	1,285	146	1,373
M88	1.17 [1.05-1.30]	1.15 [1.03-1.28]	1.18 [1.05-1.31]		1.07 [0.96-1.21]	1.13 [1.01-1.26]	1.11 [0.99-1.24]	1.13 [1.01-1.27]		1.05 [0.93-1.18]	890	887	851	NA	738
M89	1.29 [1.23-1.35]	1.30 [1.24-1.37]	1.32 [1.24-1.40]	1.12 [1.01-1.25]	1.18 [1.12-1.24]	1.20 [1.14-1.26]	1.17 [1.11-1.24]	1.18 [1.11-1.25]	1.09 [0.97-1.22]	1.13 [1.07-1.19]	4,230	3,617	2,882	849	3,693
M90	1.06 [0.97-1.15]	1.03 [0.95-1.13]	1.04 [0.96-1.13]		1.02 [0.93-1.11]	1.06 [0.97-1.15]	1.02 [0.94-1.11]	1.02 [0.94-1.12]		1.04 [0.95-1.14]	1,351	1,318	1,268	NA	1,201
M91	1.19 [1.02-1.39]	1.27 [0.99-1.64]		1.16 [0.96-1.39]	1.13 [0.95-1.34]	1.16 [0.99-1.36]	1.21 [0.93-1.56]		1.12 [0.92-1.35]	1.11 [0.93-1.32]	412	151	NA	281	337
M92	1.45 [1.28-1.64]	1.43 [1.22-1.67]	1.45 [1.17-1.78]	1.31 [1.10-1.56]	1.31 [1.14-1.51]	1.31 [1.16-1.50]	1.27 [1.08-1.49]	1.27 [1.02-1.58]	1.19 [0.99-1.43]	1.18 [1.02-1.36]	680	406	214	360	533
M93	1.31 [1.20-1.44]	1.30 [1.16-1.46]	1.30 [1.13-1.50]	1.25 [1.09-1.43]	1.18 [1.07-1.31]	1.22 [1.11-1.34]	1.15 [1.02-1.29]	1.15 [0.99-1.33]	1.18 [1.03-1.36]	1.12 [1.00-1.24]	1,199	751	441	579	938
M94	1.41 [1.33-1.49]	1.41 [1.33-1.50]	1.49 [1.39-1.60]	1.24 [1.09-1.40]	1.31 [1.23-1.39]	1.26 [1.19-1.34]	1.21 [1.14-1.28]	1.27 [1.18-1.36]	1.18 [1.04-1.35]	1.21 [1.14-1.29]	3,261	2,901	2,006	715	2,911
M95	1.36 [1.21-1.51]	1.34 [1.19-1.51]	1.31 [1.10-1.56]	1.21 [1.00-1.46]	1.21 [1.07-1.37]	1.27 [1.13-1.42]	1.20 [1.06-1.35]	1.14 [0.95-1.37]	1.20 [0.98-1.46]	1.16 [1.03-1.32]	806	662	311	284	654
M96	1.28 [1.19-1.38]	1.31 [1.21-1.41]	1.35 [1.25-1.46]		1.20 [1.11-1.29]	1.20 [1.12-1.30]	1.18 [1.10-1.28]	1.23 [1.14-1.33]		1.17 [1.08-1.26]	1,995	1,962	1,855	NA	1,905
M99	1.34 [1.17-1.53]	1.32 [1.15-1.51]	1.32 [1.15-1.52]		1.16 [1.01-1.33]	1.20 [1.05-1.38]	1.13 [0.98-1.30]	1.11 [0.97-1.29]		1.09 [0.95-1.25]	559	555	521	NA	519
Diseases of the genitourinary system (N00-N99)															
N00	1.32 [1.01-1.73]				1.15 [0.87-1.53]	1.30 [0.98-1.71]				1.14 [0.85-1.54]	140	NA	NA	NA	128
N02	1.32 [1.22-1.43]	1.36 [1.25-1.47]	1.36 [1.25-1.48]	0.86 [0.66-1.12]	1.19 [1.10-1.30]	1.27 [1.17-1.37]	1.28 [1.17-1.38]	1.27 [1.17-1.39]	0.84 [0.64-1.10]	1.17 [1.07-1.27]	1,604	1,516	1,301	139	1,399
N03	1.34 [1.28-1.39]	1.34 [1.29-1.40]	1.35 [1.29-1.41]		1.26 [1.21-1.32]	1.27 [1.22-1.32]	1.22 [1.17-1.27]	1.22 [1.17-1.28]		1.22 [1.17-1.28]	6,043	6,015	5,937	NA	5,629
N04	1.34 [1.22-1.48]	1.50 [1.35-1.67]	1.47 [1.31-1.65]	0.99 [0.82-1.20]	1.23 [1.12-1.37]	1.28 [1.16-1.41]	1.37 [1.23-1.52]	1.33 [1.19-1.50]	0.98 [0.81-1.19]	1.20 [1.08-1.33]	1,140	901	762	273	962
N05	1.48 [1.35-1.63]	1.49 [1.34-1.66]	1.49 [1.33-1.67]	1.36 [1.13-1.63]	1.37 [1.24-1.51]	1.42 [1.29-1.56]	1.36 [1.22-1.51]	1.36 [1.21-1.53]	1.32 [1.10-1.60]	1.32 [1.19-1.46]	1,170	893	754	319	1,039
N08	1.43 [1.35-1.52]	1.45 [1.36-1.53]	1.43 [1.35-1.52]		1.28 [1.21-1.36]	1.35 [1.27-1.43]	1.26 [1.18-1.33]	1.22 [1.15-1.31]		1.26 [1.19-1.34]	2,958	2,908	2,705	NA	2,814
N10	1.17 [1.07-1.28]	1.18 [1.07-1.29]	1.21 [1.07-1.37]	1.07 [0.92-1.26]	1.06 [0.97-1.17]	1.10 [1.01-1.21]	1.04 [0.94-1.15]	1.09 [0.96-1.24]	1.05 [0.89-1.24]	1.03 [0.93-1.13]	1,271	1,059	594	411	1,091
N11	1.25 [1.10-1.42]	1.24 [1.08-1.41]	1.29 [1.11-1.51]		1.08 [0.94-1.24]	1.20 [1.05-1.37]	1.14 [1.00-1.31]	1.18 [1.01-1.38]		1.09 [0.95-1.25]	572	519	401	NA	508
N12	1.18 [1.13-1.22]	1.21 [1.16-1.25]	1.28 [1.21-1.35]	1.05 [0.98-1.12]	1.10 [1.05-1.14]	1.10 [1.06-1.15]	1.07 [1.03-1.12]	1.13 [1.07-1.19]	1.02 [0.95-1.10]	1.06 [1.02-1.11]	7,028	6,129	3,401	2,174	6,119
N13	1.06 [1.03-1.09]	1.07 [1.04-1.10]	1.07 [1.04-1.10]	1.05 [0.97-1.15]	1.01 [0.99-1.04]	1.05 [1.02-1.08]	1.03 [1.01-1.06]	1.04 [1.01-1.07]	1.02 [0.94-1.12]	1.02 [0.99-1.05]	13,110	12,270	10,762	1,400	11,521
N14	1.34 [1.08-1.66]	1.31 [1.05-1.63]	1.41 [1.12-1.77]		1.25 [1.00-1.55]	1.29 [1.03-1.61]	1.20 [0.96-1.51]	1.26 [1.00-1.59]		1.22 [0.98-1.53]	220	210	202	NA	212
N15	1.20 [1.02-1.41]	1.21 [1.03-1.43]	1.21 [1.00-1.46]		1.08 [0.91-1.28]	1.13 [0.96-1.33]	1.08 [0.91-1.29]	1.10 [0.91-1.34]		1.06 [0.90-1.26]	372	346	272	NA	338
N16	1.61 [1.29-2.00]	1.65 [1.32-2.06]	1.51 [1.17-1.95]		1.29 [1.03-1.62]	1.53 [1.22-1.92]	1.51 [1.20-1.89]	1.34 [1.03-1.75]		1.30 [1.03-1.64]	221	212	155	NA	195
N17	1.23 [1.21-1.24]	1.22 [1.21-1.23]	1.23 [1.21-1.24]	1.07 [0.98-1.17]	1.17 [1.16-1.19]	1.19 [1.17-1.20]	1.13 [1.12-1.15]	1.14 [1.12-1.15]	1.07 [0.97-1.17]	1.16 [1.15-1.18]	76,985	76,432	74,248	1,369	71,764
N18	1.26 [1.24-1.28]	1.26 [1.24-1.28]	1.25 [1.24-1.27]	1.14 [0.97-1.34]	1.19 [1.17-1.20]	1.21 [1.19-1.22]	1.15 [1.14-1.17]	1.15 [1.13-1.16]	1.12 [0.95-1.33]	1.16 [1.15-1.18]	65,241	65,046	64,301	393	61,464
N19	1.30 [1.26-1.34]	1.31 [1.27-1.36]	1.29 [1.25-1.34]	1.08 [0.91-1.29]	1.23 [1.19-1.27]	1.24 [1.20-1.28]	1.21 [1.17-1.25]	1.19 [1.15-1.23]	1.05 [0.87-1.26]	1.20 [1.16-1.24]	9,785	9,554	9,235	316	8,723
N20	1.15 [1.11-1.18]	1.15 [1.12-1.19]	1.15 [1.11-1.19]	0.94 [0.84-1.05]	1.07 [1.04-1.10]	1.10 [1.06-1.13]	1.07 [1.04-1.10]	1.07 [1.03-1.10]	0.92 [0.81-1.03]	1.05 [1.02-1.08]	10,970	10,810	8,690	738	9,284
N21	1.02 [0.96-1.08]	1.01 [0.95-1.08]	1.03 [0.96-1.10]		0.95 [0.89-1.02]	1.00 [0.94-1.07]	0.98 [0.92-1.05]	1.00 [0.94-1.07]		0.96 [0.90-1.02]	2,439	2,414	2,229	NA	2,124
N23	1.17 [1.11-1.25]	1.19 [1.12-1.26]	1.14 [1.05-1.23]	1.03 [0.87-1.22]	1.05 [0.98-1.12]	1.08 [1.02-1.15]	1.06 [1.00-1.13]	1.01 [0.94-1.09]	0.98 [0.83-1.18]	1.01 [0.94-1.08]	2,585	2,517	1,569	334	2,068
N25	1.31 [1.15-1.48]	1.39 [1.22-1.59]	1.39 [1.21-1.60]		1.15 [1.00-1.31]	1.25 [1.10-1.43]	1.29 [1.12-1.48]	1.28 [1.11-1.48]		1.14 [1.00-1.31]	592	557	509	NA	546
N26	1.20 [1.09-1.32]	1.18 [1.07-1.30]	1.18 [1.06-1.31]		1.10 [1.00-1.21]	1.14 [1.03-1.26]	1.07 [0.97-1.19]	1.07 [0.96-1.18]		1.09 [0.99-1.21]	1,094	1,042	952	NA	1,040
N27	1.30 [1.15-1.47]	1.34 [1.17-1.53]	1.37 [1.19-1.58]	0.96 [0.73-1.27]	1.12 [0.98-1.28]	1.27 [1.12-1.44]	1.25 [1.09-1.43]	1.29 [1.11-1.49]	0.94 [0.71-1.25]	1.12 [0.98-1.27]	659	560	496	129	591
N28	1.18 [1.15-1.22]	1.19 [1.16-1.22]	1.19 [1.16-1.22]	0.99 [0.89-1.11]	1.11 [1.08-1.14]	1.13 [1.11-1.16]	1.10 [1.07-1.13]	1.10 [1.07-1.13]	0.98 [0.88-1.10]	1.09 [1.06-1.12]	15,323	14,766	13,912	808	13,992
N29	1.32 [1.02-1.71]	1.48 [1.11-1.97]			1.18 [0.91-1.55]	1.30 [1.00-1.68]	1.35 [1.01-1.82]			1.16 [0.88-1.52]	156	121	NA	NA	139
N30	1.27 [1.22-1.32]	1.27 [1.22-1.31]	1.23 [1.18-1.28]	1.40 [1.21-1.62]	1.16 [1.12-1.21]	1.20 [1.16-1.25]	1.17 [1.12-1.21]	1.14 [1.09-1.18]	1.33 [1.15-1.55]	1.13 [1.09-1.17]	7,370	7,189	6,222	517	6,559
N31	1.35 [1.26-1.43]	1.39 [1.30-1.48]	1.40 [1.30-1.51]	0.98 [0.82-1.16]	1.19 [1.12-1.27]	1.24 [1.16-1.32]	1.20 [1.12-1.28]	1.22 [1.13-1.31]	0.95 [0.79-1.13]	1.13 [1.06-1.21]	2,392	2,148	1,849	319	2,190
N32	1.23 [1.20-1.26]	1.22 [1.20-1.25]	1.22 [1.19-1.25]	1.16 [1.05-1.27]	1.14 [1.12-1.17]	1.17 [1.14-1.19]	1.13 [1.10-1.16]	1.14 [1.11-1.16]	1.10 [0.99-1.21]	1.11 [1.09-1.14]	21,199	20,497	18,959	1,124	18,793
N34	1.35 [1.16-1.58]	1.34 [1.13-1.59]	1.25 [1.03-1.52]		1.12 [0.94-1.33]	1.30 [1.11-1.53]	1.25 [1.05-1.49]	1.19 [0.97-1.45]		1.09 [0.91-1.29]	404	334	251	NA	327
N35	1.31 [1.27-1.36]	1.31 [1.26-1.36]	1.30 [1.25-1.35]	1.33 [1.19-1.49]	1.20 [1.16-1.25]	1.25 [1.20-1.30]	1.21 [1.16-1.26]	1.21 [1.16-1.26]	1.29 [1.15-1.45]	1.17 [1.12-1.22]	7,679	7,156	6,216	840	6,589
N36	1.23 [1.15-1.32]	1.29 [1.20-1.38]	1.23 [1.14-1.32]	1.03 [0.85-1.24]	1.14 [1.06-1.23]	1.17 [1.10-1.26]	1.21 [1.12-1.30]	1.15 [1.06-1.24]	0.99 [0.82-1.21]	1.11 [1.03-1.19]	2,100	1,897	1,635	285	1,834
N39	1.20 [1.19-1.21]	1.20 [1.19-1.21]	1.20 [1.19-1.21]	1.12 [1.09-1.16]	1.14 [1.12-1.15]	1.15 [1.14-1.17]	1.12 [1.11-1.13]	1.12 [1.11-1.13]	1.09 [1.05-1.13]	1.11 [1.10-1.13]	96,916	90,817	82,908	8,906	86,335
N40	1.16 [1.14-1.19]	1.16 [1.14-1.18]	1.16 [1.14-1.18]		1.10 [1.08-1.12]	1.12 [1.10-1.14]	1.10 [1.08-1.12]	1.10 [1.08-1.12]		1.07 [1.05-1.09]	30,069	30,057	29,975	NA	26,000
N41	1.15 [1.08-1.23]	1.14 [1.07-1.22]	1.11 [1.03-1.18]		1.04 [0.97-1.12]	1.10 [1.02-1.17]	1.08 [1.00-1.15]	1.05 [0.98-1.12]		1.01 [0.93-1.08]	2,047	2,045	1,963	NA	1,726
N42	1.11 [1.05-1.18]	1.11 [1.05-1.18]	1.13 [1.06-1.20]		1.03 [0.97-1.10]	1.07 [1.00-1.13]	1.06 [1.00-1.13]	1.08 [1.01-1.14]		1.01 [0.95-1.08]	2,640	2,638	2,612	NA	2,322
N43	1.13 [1.08-1.18]	1.21 [1.14-1.28]	1.19 [1.12-1.27]	1.05 [0.98-1.12]	1.10 [1.05-1.16]	1.10 [1.05-1.15]	1.16 [1.09-1.23]	1.14 [1.07-1.22]	1.02 [0.96-1.10]	1.09 [1.03-1.15]	4,566	2,712	2,271	2,081	3,718
N44	1.18 [1.09-1.27]	1.10 [0.92-1.32]		1.14 [1.05-1.23]	1.15 [1.06-1.26]	1.16 [1.07-1.25]	1.02 [0.85-1.24]		1.11 [1.03-1.21]	1.14 [1.04-1.25]	1,858	267	NA	1,759	1,460
N45	1.21 [1.15-1.28]	1.26 [1.18-1.33]	1.25 [1.16-1.35]	1.12 [1.02-1.23]	1.11 [1.04-1.18]	1.15 [1.09-1.22]	1.15 [1.08-1.22]	1.15 [1.07-1.24]	1.09 [0.99-1.20]	1.09 [1.02-1.15]	3,388	2,598	1,768	1,211	2,758
N47	1.46 [1.42-1.50]	1.43 [1.38-1.48]	1.38 [1.32-1.45]	1.45 [1.41-1.50]	1.37 [1.33-1.42]	1.40 [1.37-1.44]	1.32 [1.27-1.38]	1.28 [1.22-1.35]	1.41 [1.36-1.46]	1.34 [1.30-1.38]	15,415	6,884	4,410	9,851	11,705
N48	1.40 [1.35-1.46]	1.44 [1.37-1.50]	1.40 [1.33-1.48]	1.35 [1.28-1.43]	1.28 [1.23-1.34]	1.34 [1.29-1.40]	1.32 [1.26-1.39]	1.29 [1.23-1.36]	1.32 [1.24-1.40]	1.26 [1.21-1.31]	7,614	4,656	3,736	3,369	6,219
N49	1.35 [1.21-1.50]	1.38 [1.23-1.56]	1.41 [1.23-1.60]	1.13 [0.88-1.44]	1.18 [1.05-1.33]	1.29 [1.16-1.45]	1.27 [1.13-1.44]	1.28 [1.12-1.47]	1.11 [0.87-1.43]	1.19 [1.05-1.34]	822	691	574	171	680
N50	1.22 [1.17-1.26]	1.25 [1.20-1.31]	1.26 [1.20-1.33]	1.12 [1.05-1.19]	1.15 [1.10-1.19]	1.16 [1.12-1.21]	1.16 [1.11-1.21]	1.17 [1.11-1.23]	1.08 [1.02-1.15]	1.12 [1.08-1.17]	7,328	5,008	3,731	2,956	6,009
N51	1.48 [1.08-2.03]					1.37 [0.99-1.90]					108	NA	NA	NA	NA
N60	1.17 [1.10-1.25]	1.14 [1.07-1.22]	1.11 [1.03-1.19]	1.37 [1.01-1.85]	1.10 [1.02-1.18]	1.13 [1.06-1.21]	1.09 [1.02-1.16]	1.05 [0.97-1.13]	1.34 [0.98-1.84]	1.09 [1.01-1.17]	2,183	2,148	1,645	118	1,789
N61	1.34 [1.24-1.44]	1.38 [1.27-1.49]	1.39 [1.25-1.54]	1.26 [1.03-1.54]	1.27 [1.17-1.38]	1.28 [1.18-1.38]	1.24 [1.14-1.35]	1.25 [1.12-1.39]	1.22 [0.99-1.51]	1.26 [1.15-1.37]	1,671	1,585	906	262	1,429
N62	1.28 [1.18-1.39]	1.28 [1.17-1.39]	1.27 [1.13-1.42]	1.30 [1.09-1.55]	1.16 [1.05-1.27]	1.21 [1.11-1.32]	1.15 [1.05-1.26]	1.11 [0.99-1.25]	1.26 [1.05-1.51]	1.12 [1.01-1.23]	1,388	1,242	739	339	1,060
N63	1.16 [1.09-1.23]	1.17 [1.09-1.24]	1.16 [1.08-1.25]	1.19 [0.93-1.53]	1.10 [1.02-1.18]	1.11 [1.04-1.18]	1.09 [1.02-1.16]	1.08 [1.00-1.16]	1.16 [0.90-1.50]	1.08 [1.00-1.16]	2,320	2,264	1,839	163	1,932
N64	1.25 [1.18-1.34]	1.25 [1.17-1.33]	1.21 [1.12-1.30]	1.37 [1.09-1.71]	1.18 [1.11-1.27]	1.19 [1.12-1.27]	1.15 [1.07-1.23]	1.11 [1.03-1.20]	1.35 [1.07-1.70]	1.15 [1.08-1.24]	2,374	2,310	1,728	217	2,070
N70	1.07 [0.99-1.15]	1.08 [1.00-1.17]	1.06 [0.95-1.18]	1.08 [0.85-1.37]	1.00 [0.92-1.09]	1.03 [0.95-1.11]	1.01 [0.93-1.09]	1.00 [0.89-1.11]	1.06 [0.83-1.35]	0.98 [0.90-1.07]	1,521	1,491	779	181	1,282
N71	1.17 [1.05-1.31]	1.23 [1.10-1.37]	1.25 [1.09-1.42]		1.14 [1.02-1.28]	1.08 [0.97-1.21]	1.12 [1.01-1.26]	1.13 [0.99-1.30]		1.08 [0.96-1.22]	792	791	507	NA	699
N72	1.09 [1.01-1.18]	1.12 [1.04-1.21]	1.12 [1.01-1.24]	0.95 [0.72-1.26]	1.04 [0.96-1.14]	1.04 [0.96-1.13]	1.06 [0.98-1.15]	1.07 [0.96-1.19]	0.96 [0.72-1.28]	1.02 [0.93-1.12]	1,444	1,441	782	126	1,136
N73	1.14 [1.10-1.17]	1.16 [1.12-1.20]	1.13 [1.08-1.18]	1.11 [1.01-1.21]	1.07 [1.03-1.11]	1.07 [1.04-1.11]	1.06 [1.02-1.09]	1.04 [0.99-1.09]	1.08 [0.98-1.18]	1.03 [1.00-1.07]	8,766	8,580	4,006	1,360	7,511
N75	1.06 [0.97-1.15]	1.04 [0.96-1.13]	1.06 [0.93-1.20]	0.93 [0.77-1.11]	1.01 [0.92-1.10]	1.04 [0.95-1.12]	0.99 [0.91-1.07]	1.02 [0.89-1.16]	0.93 [0.77-1.12]	1.00 [0.91-1.10]	1,430	1,376	516	298	1,118
N76	1.39 [1.31-1.47]	1.44 [1.35-1.53]	1.51 [1.39-1.64]	1.22 [1.09-1.38]	1.32 [1.24-1.41]	1.31 [1.23-1.39]	1.29 [1.21-1.38]	1.37 [1.26-1.49]	1.17 [1.04-1.32]	1.27 [1.19-1.36]	2,790	2,348	1,438	759	2,369
N77	1.28 [1.21-1.36]	1.30 [1.23-1.38]	1.40 [1.27-1.54]	1.10 [0.99-1.22]	1.19 [1.12-1.26]	1.19 [1.12-1.26]	1.13 [1.06-1.20]	1.19 [1.07-1.31]	1.06 [0.95-1.18]	1.13 [1.06-1.20]	3,197	2,984	1,073	924	2,821
N80	1.13 [1.09-1.17]	1.15 [1.11-1.18]	1.09 [1.03-1.14]	1.16 [1.06-1.27]	1.08 [1.04-1.12]	1.06 [1.02-1.10]	1.04 [1.00-1.07]	1.00 [0.95-1.05]	1.12 [1.02-1.22]	1.03 [0.99-1.07]	8,651	8,539	3,717	1,438	7,115
N81	1.21 [1.18-1.24]	1.20 [1.17-1.23]	1.20 [1.17-1.23]	1.27 [0.95-1.69]	1.15 [1.12-1.18]	1.14 [1.12-1.17]	1.13 [1.10-1.16]	1.12 [1.09-1.15]	1.26 [0.94-1.70]	1.10 [1.07-1.13]	14,255	14,238	12,975	129	12,058
N82	1.32 [1.18-1.49]	1.32 [1.17-1.48]	1.30 [1.15-1.48]		1.23 [1.09-1.38]	1.24 [1.11-1.40]	1.21 [1.07-1.36]	1.19 [1.05-1.36]		1.19 [1.05-1.34]	730	718	593	NA	667
N83	1.13 [1.10-1.16]	1.15 [1.12-1.18]	1.12 [1.08-1.16]	1.11 [1.05-1.17]	1.08 [1.05-1.11]	1.07 [1.04-1.10]	1.04 [1.01-1.07]	1.04 [1.00-1.07]	1.06 [1.00-1.13]	1.04 [1.01-1.07]	14,639	13,619	6,998	3,120	12,225
N84	1.12 [1.09-1.16]	1.13 [1.09-1.16]	1.12 [1.09-1.16]	1.17 [0.93-1.48]	1.08 [1.05-1.12]	1.10 [1.07-1.13]	1.09 [1.06-1.12]	1.09 [1.06-1.13]	1.09 [0.86-1.39]	1.07 [1.03-1.10]	11,263	11,250	9,786	199	8,858
N85	1.17 [1.14-1.21]	1.19 [1.16-1.23]	1.18 [1.14-1.22]	1.17 [1.01-1.35]	1.12 [1.09-1.16]	1.13 [1.09-1.16]	1.11 [1.08-1.15]	1.11 [1.07-1.15]	1.13 [0.97-1.32]	1.09 [1.05-1.13]	9,361	9,313	7,312	516	7,912
N86	1.19 [1.12-1.26]	1.20 [1.13-1.27]	1.23 [1.10-1.37]	1.07 [0.94-1.22]	1.13 [1.06-1.20]	1.13 [1.07-1.20]	1.12 [1.05-1.19]	1.17 [1.04-1.31]	1.04 [0.91-1.19]	1.08 [1.01-1.15]	2,864	2,823	716	627	2,300
N87	1.10 [1.05-1.17]	1.13 [1.07-1.19]	1.06 [0.97-1.17]	1.13 [0.98-1.31]	1.05 [0.99-1.12]	1.06 [1.01-1.12]	1.06 [1.00-1.12]	1.01 [0.91-1.11]	1.15 [0.98-1.34]	1.05 [0.98-1.11]	3,251	3,250	962	461	2,421
N88	1.21 [1.14-1.28]	1.21 [1.14-1.28]	1.19 [1.12-1.27]	1.08 [0.78-1.49]	1.12 [1.05-1.19]	1.16 [1.10-1.23]	1.15 [1.08-1.22]	1.12 [1.05-1.20]	1.10 [0.78-1.55]	1.10 [1.03-1.17]	2,805	2,806	2,255	101	2,351
N89	1.29 [1.23-1.34]	1.30 [1.25-1.36]	1.30 [1.22-1.39]	1.10 [1.00-1.21]	1.17 [1.12-1.23]	1.21 [1.16-1.27]	1.18 [1.13-1.24]	1.21 [1.13-1.29]	1.07 [0.97-1.17]	1.12 [1.07-1.18]	5,156	4,822	2,065	1,179	4,386
N90	1.38 [1.32-1.45]	1.37 [1.31-1.43]	1.43 [1.36-1.51]	1.23 [1.08-1.41]	1.27 [1.21-1.34]	1.31 [1.25-1.37]	1.26 [1.20-1.32]	1.32 [1.25-1.39]	1.22 [1.06-1.41]	1.22 [1.16-1.28]	4,972	4,765	3,303	553	4,201
N91	1.29 [1.13-1.47]	1.30 [1.13-1.49]	1.52 [1.19-1.93]	1.14 [0.92-1.41]	1.15 [1.00-1.32]	1.22 [1.06-1.39]	1.14 [0.99-1.32]	1.41 [1.10-1.81]	1.11 [0.89-1.38]	1.10 [0.95-1.27]	609	491	160	239	485
N92	1.21 [1.18-1.23]	1.21 [1.18-1.24]	1.18 [1.15-1.21]	1.21 [1.13-1.30]	1.15 [1.12-1.17]	1.13 [1.11-1.16]	1.10 [1.08-1.13]	1.09 [1.06-1.12]	1.16 [1.08-1.25]	1.10 [1.07-1.13]	19,969	19,184	12,084	2,231	16,515
N93	1.20 [1.16-1.24]	1.21 [1.17-1.25]	1.18 [1.13-1.24]	1.14 [1.06-1.23]	1.11 [1.07-1.14]	1.14 [1.10-1.17]	1.10 [1.06-1.13]	1.09 [1.04-1.14]	1.11 [1.02-1.20]	1.07 [1.04-1.11]	9,184	8,788	4,249	1,741	7,743
N94	1.26 [1.21-1.30]	1.28 [1.24-1.33]	1.27 [1.20-1.34]	1.24 [1.15-1.34]	1.17 [1.13-1.22]	1.16 [1.12-1.20]	1.13 [1.09-1.17]	1.14 [1.08-1.21]	1.18 [1.09-1.28]	1.11 [1.07-1.16]	7,792	7,307	2,895	1,794	6,450
N95	1.25 [1.21-1.29]	1.25 [1.21-1.29]	1.24 [1.21-1.28]		1.19 [1.15-1.23]	1.20 [1.16-1.24]	1.18 [1.15-1.22]	1.17 [1.14-1.21]		1.16 [1.12-1.20]	10,449	10,449	10,388	NA	8,443
N96	1.08 [0.83-1.41]	1.11 [0.85-1.44]			0.99 [0.75-1.30]	1.07 [0.81-1.41]	1.07 [0.81-1.41]			1.00 [0.76-1.33]	134	134	NA	NA	120
N97	0.93 [0.86-1.01]	0.95 [0.87-1.03]	0.97 [0.75-1.26]		0.97 [0.88-1.08]	0.92 [0.85-1.00]	0.93 [0.86-1.01]	0.98 [0.76-1.27]		0.96 [0.86-1.07]	1,306	1,306	125	NA	800
N98	0.80 [0.63-1.03]	0.81 [0.64-1.04]			0.80 [0.60-1.06]	0.76 [0.59-0.98]	0.76 [0.59-0.98]			0.77 [0.57-1.02]	143	143	NA	NA	105
N99	1.22 [1.15-1.30]	1.25 [1.18-1.33]	1.22 [1.14-1.31]	1.01 [0.80-1.26]	1.13 [1.06-1.20]	1.17 [1.10-1.25]	1.17 [1.10-1.25]	1.14 [1.06-1.22]	0.99 [0.79-1.25]	1.11 [1.04-1.18]	2,593	2,456	2,020	199	2,309
Pregnancy, childbirth and the puerperium (O00-O9A)															
O00	1.04 [0.98-1.11]	1.09 [1.03-1.16]	1.03 [0.81-1.31]	1.02 [0.90-1.16]	0.99 [0.92-1.05]	1.02 [0.95-1.08]	1.03 [0.97-1.10]	1.02 [0.80-1.30]	0.99 [0.87-1.13]	0.98 [0.92-1.05]	2,598	2,578	146	620	2,122
O01	1.17 [0.92-1.49]	1.18 [0.93-1.51]			1.14 [0.87-1.48]	1.19 [0.92-1.53]	1.17 [0.91-1.50]			1.21 [0.92-1.59]	165	161	NA	NA	132
O02	1.10 [1.05-1.14]	1.11 [1.06-1.15]	1.02 [0.91-1.15]	1.05 [0.95-1.15]	1.04 [1.00-1.09]	1.07 [1.02-1.11]	1.07 [1.03-1.11]	1.00 [0.89-1.13]	1.04 [0.94-1.14]	1.03 [0.99-1.08]	6,033	5,967	628	1,190	4,823
O03	1.06 [1.02-1.09]	1.07 [1.04-1.11]	1.04 [0.95-1.14]	1.00 [0.94-1.07]	1.01 [0.97-1.05]	1.04 [1.00-1.07]	1.03 [1.00-1.07]	1.03 [0.94-1.14]	0.99 [0.92-1.06]	1.00 [0.97-1.04]	9,004	8,798	949	2,142	7,259
O04	1.02 [1.00-1.05]	1.04 [1.01-1.07]	0.92 [0.81-1.04]	0.98 [0.94-1.03]	0.94 [0.91-0.98]	1.01 [0.98-1.04]	0.99 [0.96-1.03]	0.90 [0.79-1.03]	0.98 [0.94-1.03]	0.95 [0.92-0.98]	11,751	10,339	521	5,427	8,510
O08	1.05 [0.95-1.16]	1.09 [0.98-1.20]		0.99 [0.83-1.18]	0.97 [0.87-1.09]	1.01 [0.91-1.12]	1.01 [0.91-1.12]		0.99 [0.83-1.18]	0.97 [0.87-1.09]	942	916	NA	336	792
O10	1.13 [1.02-1.25]	1.16 [1.05-1.28]	1.27 [1.01-1.59]	0.95 [0.72-1.25]	1.09 [0.98-1.21]	1.10 [0.99-1.21]	1.07 [0.97-1.19]	1.06 [0.83-1.34]	0.95 [0.71-1.27]	1.06 [0.96-1.19]	994	995	170	132	851
O11	1.17 [0.90-1.51]	1.26 [0.98-1.63]			1.12 [0.85-1.48]	1.12 [0.86-1.45]	1.16 [0.88-1.52]			1.09 [0.82-1.44]	149	148	NA	NA	127
O12	1.13 [1.06-1.21]	1.15 [1.08-1.23]		1.10 [0.97-1.25]	1.09 [1.01-1.17]	1.08 [1.01-1.16]	1.06 [0.99-1.14]		1.08 [0.95-1.23]	1.05 [0.97-1.14]	2,093	2,054	NA	628	1,647
O13	1.08 [1.04-1.13]	1.11 [1.07-1.16]	0.96 [0.81-1.14]	1.04 [0.96-1.13]	1.05 [1.00-1.10]	1.06 [1.01-1.11]	1.08 [1.04-1.13]	0.96 [0.81-1.14]	1.02 [0.93-1.11]	1.03 [0.98-1.08]	5,577	5,523	288	1,424	4,037
O14	1.10 [1.05-1.16]	1.12 [1.07-1.18]	0.98 [0.80-1.20]	1.12 [1.02-1.22]	1.06 [1.00-1.13]	1.07 [1.02-1.13]	1.09 [1.03-1.14]	0.96 [0.78-1.18]	1.12 [1.02-1.22]	1.03 [0.97-1.09]	4,133	4,062	205	1,285	2,898
O15	1.10 [0.86-1.41]	1.12 [0.88-1.44]			1.05 [0.79-1.40]	1.08 [0.84-1.39]	1.08 [0.83-1.39]			1.00 [0.74-1.35]	159	153	NA	NA	108
O16	1.12 [1.07-1.17]	1.12 [1.07-1.18]	1.03 [0.86-1.24]	1.10 [1.00-1.21]	1.08 [1.02-1.14]	1.09 [1.04-1.14]	1.08 [1.03-1.14]	1.02 [0.84-1.24]	1.08 [0.98-1.19]	1.06 [1.01-1.12]	4,610	4,502	243	1,090	3,230
O20	1.06 [1.03-1.10]	1.10 [1.06-1.14]	0.97 [0.83-1.13]	1.00 [0.94-1.07]	1.00 [0.96-1.04]	1.03 [0.99-1.07]	1.03 [1.00-1.07]	0.98 [0.84-1.15]	1.00 [0.93-1.06]	0.99 [0.95-1.03]	8,064	7,869	356	2,475	6,494
O21	1.11 [1.06-1.15]	1.16 [1.11-1.21]		1.03 [0.97-1.10]	1.02 [0.97-1.06]	1.04 [1.00-1.09]	1.05 [1.00-1.09]		1.01 [0.94-1.08]	0.99 [0.94-1.03]	5,975	5,800	NA	2,422	4,869
O22	1.11 [1.00-1.23]	1.10 [1.00-1.22]		0.95 [0.74-1.22]	1.00 [0.90-1.12]	1.09 [0.98-1.21]	1.07 [0.96-1.19]		0.90 [0.69-1.17]	1.00 [0.90-1.12]	915	913	NA	157	805
O23	1.16 [1.12-1.20]	1.20 [1.15-1.24]	0.93 [0.73-1.19]	1.03 [0.97-1.10]	1.08 [1.04-1.13]	1.11 [1.07-1.15]	1.09 [1.05-1.14]	0.89 [0.69-1.14]	1.02 [0.96-1.09]	1.06 [1.02-1.11]	6,682	6,472	135	2,480	5,486
O24	1.07 [1.03-1.11]	1.09 [1.06-1.13]	1.04 [0.93-1.16]	1.00 [0.92-1.09]	1.02 [0.98-1.06]	1.05 [1.01-1.09]	1.07 [1.03-1.11]	1.03 [0.92-1.16]	1.02 [0.94-1.11]	1.01 [0.97-1.05]	7,581	7,552	683	1,507	6,382
O26	1.16 [1.13-1.18]	1.19 [1.17-1.22]	0.95 [0.85-1.05]	1.05 [1.01-1.08]	1.07 [1.05-1.10]	1.10 [1.08-1.13]	1.10 [1.07-1.12]	0.92 [0.83-1.03]	1.02 [0.99-1.06]	1.04 [1.02-1.07]	25,422	24,733	756	8,267	19,725
O28	1.06 [0.99-1.13]	1.09 [1.02-1.16]	0.97 [0.75-1.25]	0.98 [0.87-1.10]	0.99 [0.92-1.06]	1.01 [0.95-1.08]	1.01 [0.94-1.08]	0.94 [0.72-1.23]	0.97 [0.86-1.09]	0.96 [0.90-1.03]	2,337	2,318	122	778	1,957
O30	1.00 [0.94-1.06]	1.01 [0.96-1.08]	0.86 [0.69-1.07]	1.08 [0.94-1.23]	0.95 [0.89-1.02]	0.98 [0.92-1.04]	0.98 [0.92-1.04]	0.88 [0.70-1.10]	1.08 [0.94-1.24]	0.95 [0.88-1.01]	2,655	2,647	170	573	2,178
O31	1.05 [0.82-1.34]	1.10 [0.86-1.41]			1.08 [0.84-1.39]	1.01 [0.78-1.30]	1.01 [0.78-1.31]			1.07 [0.82-1.39]	158	157	NA	NA	140
O32	1.03 [1.00-1.06]	1.06 [1.03-1.09]	0.96 [0.85-1.08]	0.96 [0.90-1.02]	0.99 [0.96-1.02]	1.02 [0.99-1.05]	1.04 [1.01-1.07]	0.98 [0.87-1.11]	0.95 [0.89-1.01]	0.98 [0.95-1.02]	11,077	11,025	591	2,583	8,577
O33	1.15 [0.95-1.39]	1.19 [0.99-1.44]			1.02 [0.83-1.26]	1.13 [0.93-1.37]	1.16 [0.96-1.40]			1.01 [0.82-1.24]	265	266	NA	NA	209
O34	1.09 [1.06-1.11]	1.10 [1.08-1.13]	1.00 [0.93-1.08]	0.98 [0.92-1.04]	1.02 [1.00-1.04]	1.07 [1.05-1.10]	1.08 [1.06-1.11]	1.03 [0.95-1.12]	0.97 [0.91-1.03]	1.02 [0.99-1.04]	18,898	18,872	1,365	3,020	17,262
O35	1.02 [0.96-1.08]	1.06 [1.00-1.13]	0.97 [0.80-1.16]	1.00 [0.89-1.13]	0.99 [0.93-1.05]	1.00 [0.94-1.06]	1.03 [0.97-1.10]	0.95 [0.78-1.15]	0.99 [0.88-1.13]	0.98 [0.92-1.05]	2,724	2,691	243	688	2,259
O36	1.12 [1.10-1.14]	1.15 [1.13-1.17]	1.02 [0.93-1.11]	1.03 [1.00-1.06]	1.04 [1.02-1.07]	1.08 [1.06-1.10]	1.09 [1.07-1.11]	1.02 [0.93-1.11]	1.02 [0.99-1.05]	1.03 [1.01-1.05]	33,543	32,909	1,131	11,623	25,591
O40	1.09 [1.03-1.16]	1.10 [1.04-1.17]	0.95 [0.76-1.19]	1.04 [0.92-1.18]	1.04 [0.98-1.11]	1.05 [0.99-1.12]	1.04 [0.98-1.11]	0.96 [0.76-1.22]	1.06 [0.93-1.20]	1.02 [0.96-1.09]	2,620	2,607	158	685	2,291
O41	1.10 [1.04-1.16]	1.12 [1.06-1.18]	0.99 [0.77-1.27]	0.99 [0.89-1.09]	1.04 [0.98-1.11]	1.08 [1.02-1.14]	1.08 [1.02-1.14]	1.05 [0.81-1.36]	1.00 [0.90-1.10]	1.04 [0.98-1.10]	3,493	3,440	132	985	2,693

Supplementary Table 3: Hazard ratios and events from all cohorts

	Hazard ratio (99% confidence interval)										Events (in exposed)				
	crude					adjusted									
Outcome	any age	18+	40+	<18	hosp.	any age	18+	40+	<18	hosp.	any age	18+	40+	<18	hosp.
O42	1.03 [1.01-1.06]	1.06 [1.04-1.08]	0.84 [0.75-0.95]	1.00 [0.96-1.05]	1.01 [0.98-1.04]	1.03 [1.01-1.05]	1.04 [1.02-1.07]	0.89 [0.78-1.00]	1.00 [0.95-1.04]	1.02 [0.99-1.05]	18,440	18,245	557	5,216	13,509
O43	1.03 [0.97-1.10]	1.06 [0.99-1.12]	0.94 [0.71-1.25]	0.94 [0.83-1.06]	1.01 [0.94-1.08]	1.02 [0.96-1.09]	1.03 [0.97-1.10]	0.92 [0.69-1.23]	0.96 [0.84-1.09]	1.00 [0.94-1.08]	2,479	2,451	103	627	1,950
O44	1.03 [0.95-1.11]	1.04 [0.96-1.12]	0.98 [0.76-1.25]	0.90 [0.74-1.09]	1.01 [0.94-1.10]	0.99 [0.91-1.07]	0.99 [0.92-1.07]	0.97 [0.75-1.26]	0.87 [0.71-1.06]	1.00 [0.92-1.08]	1,648	1,640	134	264	1,419
O45	1.02 [0.91-1.14]	1.05 [0.94-1.17]		1.01 [0.80-1.26]	1.00 [0.88-1.13]	1.00 [0.89-1.12]	1.01 [0.90-1.14]		1.03 [0.82-1.31]	0.99 [0.88-1.13]	734	733	NA	192	605
O46	1.12 [1.09-1.16]	1.16 [1.12-1.19]	1.04 [0.89-1.22]	1.04 [0.97-1.10]	1.06 [1.02-1.10]	1.09 [1.05-1.13]	1.09 [1.06-1.13]	1.04 [0.88-1.22]	1.02 [0.96-1.09]	1.05 [1.01-1.09]	8,965	8,804	333	2,539	6,973
O47	1.16 [1.12-1.21]	1.17 [1.13-1.22]	0.95 [0.75-1.20]	1.05 [0.97-1.14]	1.07 [1.02-1.12]	1.12 [1.07-1.16]	1.10 [1.06-1.14]	0.91 [0.71-1.16]	1.04 [0.95-1.13]	1.05 [1.00-1.10]	6,347	6,178	146	1,471	4,703
O48	1.02 [0.99-1.05]	1.03 [1.00-1.06]	0.84 [0.71-1.00]	0.97 [0.92-1.03]	0.97 [0.93-1.00]	1.02 [0.99-1.05]	1.03 [1.00-1.06]	0.89 [0.75-1.06]	0.97 [0.91-1.03]	0.97 [0.94-1.01]	10,930	10,772	279	2,881	7,536
O60	1.08 [1.04-1.11]	1.11 [1.07-1.14]	1.06 [0.93-1.21]	1.02 [0.96-1.08]	1.03 [0.99-1.06]	1.05 [1.01-1.08]	1.05 [1.02-1.09]	1.03 [0.90-1.18]	1.02 [0.96-1.09]	1.02 [0.98-1.06]	9,664	9,541	479	2,675	7,666
O61	1.08 [1.01-1.17]	1.11 [1.03-1.19]	0.98 [0.75-1.27]	1.10 [0.94-1.27]	1.05 [0.97-1.15]	1.05 [0.97-1.13]	1.06 [0.98-1.14]	0.92 [0.70-1.21]	1.05 [0.90-1.23]	1.02 [0.93-1.11]	1,800	1,784	120	471	1,298
O62	1.05 [1.01-1.09]	1.08 [1.04-1.12]	0.96 [0.81-1.15]	0.97 [0.91-1.04]	1.02 [0.98-1.07]	1.05 [1.01-1.09]	1.06 [1.02-1.10]	0.99 [0.83-1.19]	0.97 [0.90-1.04]	1.03 [0.98-1.07]	7,213	7,138	271	2,058	5,453
O63	1.02 [0.99-1.04]	1.03 [1.01-1.06]	0.91 [0.80-1.03]	1.00 [0.95-1.05]	1.01 [0.98-1.04]	1.01 [0.98-1.03]	1.02 [1.00-1.05]	0.96 [0.84-1.10]	0.98 [0.93-1.03]	1.00 [0.97-1.03]	15,999	15,795	487	4,449	10,045
O64	1.02 [0.97-1.08]	1.03 [0.97-1.09]		1.08 [0.96-1.22]	1.01 [0.94-1.07]	1.02 [0.96-1.08]	1.03 [0.97-1.09]		1.07 [0.94-1.20]	1.00 [0.94-1.07]	3,014	2,962	NA	700	2,033
O65	0.98 [0.80-1.21]	1.01 [0.82-1.24]			0.99 [0.78-1.26]	0.99 [0.80-1.22]	1.04 [0.84-1.29]			1.02 [0.79-1.31]	207	205	NA	NA	144
O66	1.01 [0.96-1.07]	1.03 [0.97-1.09]	0.95 [0.73-1.24]	0.90 [0.80-1.01]	0.99 [0.93-1.06]	1.00 [0.94-1.06]	1.01 [0.96-1.07]	0.97 [0.74-1.28]	0.90 [0.80-1.01]	0.99 [0.93-1.06]	2,954	2,917	115	705	2,146
O67	1.02 [0.91-1.13]	1.07 [0.96-1.19]		1.07 [0.85-1.36]	1.01 [0.89-1.14]	1.01 [0.90-1.13]	1.06 [0.95-1.19]		1.10 [0.86-1.41]	0.99 [0.88-1.13]	776	771	NA	178	596
O68	1.04 [1.03-1.06]	1.08 [1.06-1.09]	0.93 [0.85-1.01]	1.00 [0.97-1.03]	1.01 [0.99-1.03]	1.03 [1.02-1.05]	1.06 [1.04-1.07]	0.95 [0.87-1.04]	1.00 [0.97-1.03]	1.00 [0.98-1.02]	35,814	35,282	1,175	10,799	24,226
O69	1.09 [1.03-1.15]	1.10 [1.04-1.16]	1.18 [0.93-1.49]	0.99 [0.88-1.11]	1.03 [0.97-1.10]	1.08 [1.02-1.14]	1.10 [1.04-1.16]	1.22 [0.95-1.56]	0.99 [0.88-1.12]	1.04 [0.98-1.11]	3,144	3,121	152	714	2,453
O70	1.03 [1.02-1.05]	1.06 [1.04-1.08]	0.93 [0.85-1.01]	0.97 [0.95-1.00]	0.98 [0.96-0.99]	1.04 [1.02-1.05]	1.06 [1.04-1.07]	1.00 [0.92-1.10]	0.98 [0.95-1.00]	0.99 [0.97-1.01]	48,711	47,702	1,170	15,359	33,463
O71	1.01 [0.96-1.08]	1.05 [0.99-1.11]		1.00 [0.90-1.12]	1.00 [0.93-1.06]	1.01 [0.95-1.07]	1.05 [0.98-1.11]		1.02 [0.91-1.14]	1.00 [0.94-1.07]	2,697	2,647	NA	802	2,035
O72	1.05 [1.03-1.08]	1.08 [1.06-1.10]	0.97 [0.89-1.06]	0.98 [0.94-1.02]	1.02 [1.00-1.04]	1.05 [1.02-1.07]	1.07 [1.05-1.09]	1.00 [0.92-1.10]	0.97 [0.93-1.02]	1.02 [0.99-1.04]	23,598	23,373	1,079	6,369	17,714
O73	1.10 [1.02-1.19]	1.11 [1.03-1.20]		1.10 [0.93-1.31]	1.04 [0.95-1.13]	1.09 [1.01-1.18]	1.08 [1.00-1.17]		1.11 [0.93-1.32]	1.03 [0.95-1.13]	1,593	1,576	NA	333	1,234
O74	1.16 [1.00-1.34]	1.15 [1.00-1.34]		0.88 [0.66-1.16]	1.12 [0.96-1.31]	1.12 [0.96-1.30]	1.10 [0.95-1.28]		0.87 [0.65-1.16]	1.10 [0.94-1.30]	464	453	NA	122	378
O75	1.04 [1.01-1.07]	1.06 [1.03-1.09]	0.91 [0.79-1.05]	0.95 [0.90-1.01]	1.01 [0.97-1.04]	1.04 [1.01-1.07]	1.06 [1.03-1.09]	0.97 [0.83-1.12]	0.95 [0.89-1.01]	1.01 [0.98-1.05]	11,471	11,349	410	3,085	8,786
O80	1.08 [1.05-1.11]	1.09 [1.06-1.12]	1.05 [0.89-1.24]	0.94 [0.89-1.00]	0.97 [0.94-1.00]	1.09 [1.06-1.12]	1.09 [1.06-1.12]	1.11 [0.94-1.32]	0.96 [0.91-1.02]	1.00 [0.97-1.03]	12,482	12,266	312	2,989	10,079
O81	1.07 [0.86-1.34]	1.05 [0.84-1.32]				1.06 [0.84-1.33]	1.04 [0.83-1.31]				177	173	NA	NA	NA
O82	1.10 [1.03-1.19]	1.08 [1.00-1.16]	0.86 [0.67-1.11]	1.07 [0.86-1.33]	1.02 [0.94-1.10]	1.08 [1.00-1.16]	1.05 [0.98-1.13]	0.91 [0.69-1.18]	1.07 [0.85-1.34]	1.01 [0.93-1.09]	1,727	1,717	120	211	1,404
O85	1.07 [0.97-1.19]	1.08 [0.98-1.20]		0.93 [0.78-1.11]	1.03 [0.93-1.15]	1.03 [0.93-1.15]	1.02 [0.92-1.13]		0.90 [0.74-1.08]	1.01 [0.90-1.12]	988	977	NA	328	816
O86	1.15 [1.09-1.21]	1.18 [1.12-1.24]	1.20 [0.97-1.47]	1.03 [0.93-1.13]	1.11 [1.05-1.18]	1.12 [1.06-1.18]	1.13 [1.08-1.20]	1.16 [0.93-1.43]	1.02 [0.92-1.13]	1.08 [1.02-1.15]	3,748	3,716	201	1,055	2,931
O87	1.19 [1.04-1.36]	1.24 [1.09-1.42]			1.17 [1.01-1.35]	1.17 [1.02-1.34]	1.21 [1.06-1.39]			1.16 [0.99-1.35]	542	540	NA	NA	440
O88	1.15 [0.92-1.44]	1.20 [0.96-1.50]			1.14 [0.91-1.44]	1.07 [0.85-1.35]	1.10 [0.87-1.39]			1.09 [0.86-1.38]	201	200	NA	NA	184
O89	1.32 [1.03-1.68]	1.34 [1.06-1.71]			1.17 [0.90-1.53]	1.25 [0.97-1.62]	1.30 [1.02-1.67]			1.14 [0.87-1.49]	174	174	NA	NA	133
O90	1.18 [1.12-1.24]	1.20 [1.14-1.25]	1.10 [0.91-1.33]	1.06 [0.97-1.17]	1.11 [1.05-1.17]	1.14 [1.08-1.19]	1.13 [1.08-1.19]	1.08 [0.89-1.31]	1.04 [0.95-1.15]	1.08 [1.02-1.14]	4,483	4,439	242	1,218	3,558
O91	1.02 [0.87-1.18]	1.06 [0.91-1.23]		1.07 [0.78-1.46]	0.98 [0.82-1.16]	0.99 [0.84-1.16]	1.02 [0.87-1.20]		1.10 [0.79-1.53]	0.95 [0.80-1.14]	413	407	NA	104	311
O92	1.11 [0.98-1.25]	1.12 [0.99-1.26]		0.96 [0.74-1.24]	1.04 [0.91-1.19]	1.07 [0.94-1.21]	1.07 [0.95-1.22]		0.91 [0.69-1.20]	1.03 [0.89-1.18]	638	631	NA	146	493
O94	1.26 [0.97-1.63]	1.18 [0.92-1.53]			1.15 [0.88-1.50]	1.14 [0.86-1.50]	1.04 [0.79-1.36]			1.11 [0.84-1.46]	149	149	NA	NA	138
O98	1.15 [1.07-1.25]	1.21 [1.12-1.31]		1.06 [0.92-1.22]	1.10 [1.01-1.19]	1.11 [1.02-1.20]	1.14 [1.05-1.23]		1.04 [0.90-1.20]	1.06 [0.98-1.16]	1,674	1,653	NA	524	1,404
O99	1.16 [1.14-1.19]	1.19 [1.17-1.22]	1.06 [0.97-1.15]	1.05 [1.01-1.08]	1.08 [1.06-1.11]	1.11 [1.09-1.13]	1.11 [1.08-1.13]	1.02 [0.93-1.11]	1.03 [0.99-1.07]	1.06 [1.04-1.08]	27,987	27,633	1,210	8,440	22,273
Certain conditions originating in the perinatal period (P00-P96)															
P83	1.02 [0.87-1.20]			1.03 [0.88-1.21]	1.00 [0.84-1.20]	1.02 [0.86-1.20]			1.01 [0.86-1.19]	1.01 [0.84-1.21]	369	NA	NA	366	299
Congenital malformations, deformations and chromosomal abnormalities (Q00-Q99)															
Q02	1.10 [0.96-1.27]			1.08 [0.93-1.25]	1.02 [0.88-1.18]	1.05 [0.91-1.21]			1.05 [0.90-1.22]	1.00 [0.86-1.16]	474	NA	NA	436	438
Q03	1.20 [0.96-1.50]	1.58 [1.17-2.12]			1.22 [0.96-1.55]	1.15 [0.91-1.45]	1.47 [1.08-2.00]			1.21 [0.95-1.55]	187	112	NA	NA	164
Q04	1.08 [0.94-1.25]	1.21 [0.96-1.53]		1.01 [0.85-1.20]	1.02 [0.88-1.18]	1.01 [0.87-1.17]	1.07 [0.84-1.36]		0.97 [0.81-1.16]	0.98 [0.84-1.14]	468	178	NA	324	430
Q05	1.29 [1.12-1.49]	1.33 [1.14-1.55]	1.32 [1.10-1.60]	1.08 [0.81-1.45]	1.18 [1.01-1.37]	1.18 [1.02-1.37]	1.20 [1.02-1.40]	1.16 [0.96-1.41]	1.03 [0.76-1.39]	1.10 [0.94-1.28]	474	403	268	114	393
Q06	0.91 [0.70-1.20]				0.85 [0.63-1.13]	0.88 [0.66-1.16]				0.81 [0.60-1.08]	126	NA	NA	NA	112
Q07	1.20 [1.05-1.36]	1.34 [1.14-1.58]	1.31 [1.02-1.69]	1.02 [0.85-1.23]	1.11 [0.97-1.27]	1.09 [0.95-1.24]	1.12 [0.94-1.34]	1.10 [0.84-1.43]	0.96 [0.80-1.16]	1.04 [0.90-1.19]	582	360	142	309	524
Q10	1.19 [1.05-1.35]	1.46 [1.08-1.97]		1.14 [1.00-1.31]	1.14 [1.00-1.31]	1.18 [1.04-1.34]	1.40 [1.02-1.90]		1.12 [0.98-1.29]	1.12 [0.98-1.29]	610	107	NA	520	530
Q12	1.22 [1.02-1.46]	1.26 [1.00-1.58]	1.23 [0.93-1.62]	1.25 [0.97-1.63]	1.16 [0.95-1.40]	1.17 [0.98-1.41]	1.19 [0.94-1.51]	1.18 [0.88-1.56]	1.21 [0.93-1.57]	1.13 [0.93-1.38]	313	185	123	150	261
Q13	1.33 [1.00-1.77]				1.22 [0.90-1.64]	1.21 [0.90-1.62]				1.14 [0.84-1.55]	120	NA	NA	NA	106
Q14	1.11 [0.86-1.44]				1.08 [0.82-1.42]	1.07 [0.82-1.39]				1.08 [0.82-1.43]	142	NA	NA	NA	126
Q16	1.28 [0.93-1.75]					1.24 [0.90-1.70]					104	NA	NA	NA	NA
Q17	1.08 [0.99-1.17]			1.07 [0.98-1.16]	1.06 [0.96-1.17]	1.06 [0.97-1.15]			1.06 [0.97-1.15]	1.06 [0.95-1.17]	1,396	NA	NA	1,359	980
Q18	1.16 [1.04-1.30]	1.10 [0.93-1.31]	1.18 [0.92-1.50]	1.19 [1.04-1.36]	1.14 [1.01-1.29]	1.13 [1.01-1.27]	1.01 [0.84-1.20]	1.10 [0.86-1.42]	1.17 [1.02-1.34]	1.13 [1.00-1.29]	788	305	147	538	631
Q20	1.01 [0.77-1.32]				1.03 [0.77-1.37]	0.98 [0.75-1.30]				1.00 [0.75-1.34]	128	NA	NA	NA	116
Q21	1.16 [1.09-1.24]	1.24 [1.14-1.34]	1.28 [1.16-1.40]	1.01 [0.91-1.12]	1.12 [1.04-1.20]	1.10 [1.03-1.18]	1.14 [1.06-1.24]	1.16 [1.06-1.28]	0.98 [0.88-1.09]	1.07 [1.00-1.15]	2,318	1,521	1,110	923	1,960
Q22	0.96 [0.79-1.15]			0.87 [0.69-1.09]	0.94 [0.77-1.15]	0.96 [0.79-1.17]			0.87 [0.70-1.10]	0.95 [0.78-1.17]	259	NA	NA	181	231
Q23	1.15 [1.04-1.26]	1.17 [1.04-1.31]	1.14 [1.00-1.30]	1.07 [0.91-1.27]	1.04 [0.94-1.16]	1.11 [1.01-1.23]	1.10 [0.98-1.24]	1.10 [0.96-1.25]	1.04 [0.87-1.23]	1.02 [0.92-1.14]	998	703	552	354	846
Q24	1.17 [1.05-1.31]	1.36 [1.19-1.55]	1.40 [1.19-1.66]	0.94 [0.79-1.11]	1.08 [0.96-1.22]	1.10 [0.99-1.23]	1.23 [1.07-1.41]	1.24 [1.04-1.47]	0.92 [0.77-1.09]	1.03 [0.92-1.16]	815	538	344	347	722
Q25	1.06 [0.95-1.18]	1.19 [1.01-1.40]	1.19 [0.97-1.46]	0.96 [0.84-1.10]	1.02 [0.91-1.14]	1.03 [0.92-1.14]	1.11 [0.94-1.31]	1.08 [0.87-1.33]	0.93 [0.81-1.07]	0.98 [0.88-1.11]	821	365	217	512	717
Q26	1.11 [0.85-1.44]				0.97 [0.74-1.27]	1.04 [0.80-1.37]				0.93 [0.71-1.23]	141	NA	NA	NA	129
Q27	1.08 [0.96-1.22]	1.20 [1.03-1.40]	1.19 [0.99-1.43]	1.01 [0.84-1.20]	1.03 [0.91-1.17]	1.04 [0.92-1.18]	1.13 [0.96-1.32]	1.11 [0.91-1.34]	1.00 [0.83-1.19]	1.02 [0.90-1.16]	668	394	273	324	608
Q28	1.32 [1.12-1.54]	1.46 [1.23-1.74]	1.36 [1.11-1.66]	0.98 [0.71-1.35]	1.18 [0.99-1.40]	1.27 [1.08-1.49]	1.36 [1.14-1.63]	1.25 [1.02-1.54]	0.98 [0.71-1.36]	1.17 [0.98-1.39]	391	323	235	101	314
Q31	1.03 [0.86-1.24]			0.98 [0.80-1.21]	0.92 [0.77-1.11]	0.94 [0.78-1.14]			0.87 [0.70-1.08]	0.84 [0.69-1.02]	274	NA	NA	210	254
Q32	0.96 [0.73-1.27]				0.92 [0.68-1.23]	0.85 [0.64-1.13]				0.80 [0.59-1.09]	118	NA	NA	NA	109
Q33	1.05 [0.79-1.41]				1.01 [0.75-1.36]	0.93 [0.69-1.26]				0.90 [0.66-1.23]	112	NA	NA	NA	105
Q35	1.11 [0.94-1.32]			1.10 [0.92-1.32]	1.04 [0.87-1.24]	1.06 [0.89-1.26]			1.04 [0.87-1.26]	1.01 [0.84-1.22]	344	NA	NA	302	304
Q38	1.14 [1.07-1.21]	1.16 [1.02-1.31]	1.20 [1.05-1.37]	1.11 [1.03-1.20]	1.10 [1.02-1.18]	1.11 [1.04-1.18]	1.08 [0.95-1.22]	1.10 [0.97-1.26]	1.09 [1.01-1.18]	1.07 [1.00-1.15]	2,375	673	605	1,736	1,978
Q39	1.27 [1.08-1.49]	1.30 [1.09-1.53]	1.29 [1.08-1.53]		1.11 [0.93-1.31]	1.17 [0.99-1.39]	1.16 [0.98-1.38]	1.16 [0.97-1.39]		1.03 [0.87-1.23]	376	352	326	NA	334
Q40	1.24 [1.01-1.52]	1.20 [0.97-1.48]	1.17 [0.94-1.47]		1.20 [0.97-1.48]	1.11 [0.90-1.38]	1.03 [0.82-1.28]	1.03 [0.82-1.30]		1.12 [0.90-1.39]	227	207	185	NA	211
Q43	1.17 [1.06-1.29]	1.23 [1.10-1.38]	1.22 [1.07-1.39]	0.98 [0.81-1.17]	1.13 [1.01-1.26]	1.10 [0.99-1.22]	1.10 [0.98-1.24]	1.10 [0.96-1.26]	0.95 [0.79-1.14]	1.07 [0.96-1.19]	950	723	519	301	815
Q44	1.15 [0.95-1.38]	1.21 [1.00-1.47]	1.11 [0.90-1.38]		1.08 [0.89-1.31]	1.09 [0.90-1.31]	1.10 [0.91-1.34]	1.01 [0.81-1.26]		1.05 [0.86-1.27]	289	254	200	NA	253
Q45	1.34 [1.02-1.76]	1.36 [1.01-1.83]			1.37 [1.03-1.81]	1.24 [0.93-1.64]	1.13 [0.82-1.54]			1.29 [0.97-1.72]	135	111	NA	NA	126
Q50	1.25 [1.11-1.41]	1.30 [1.14-1.47]	1.12 [0.91-1.37]	1.41 [1.12-1.77]	1.13 [0.99-1.29]	1.19 [1.06-1.35]	1.19 [1.04-1.35]	1.04 [0.84-1.28]	1.28 [1.00-1.63]	1.11 [0.97-1.27]	682	605	206	214	550
Q51	1.06 [0.94-1.21]	1.10 [0.97-1.25]	1.20 [1.00-1.44]	1.09 [0.83-1.44]	1.06 [0.93-1.22]	1.01 [0.89-1.15]	1.02 [0.89-1.16]	1.08 [0.89-1.31]	1.08 [0.81-1.43]	1.03 [0.90-1.18]	599	567	260	135	499
Q52	1.21 [1.03-1.42]	1.22 [0.97-1.52]	1.45 [1.08-1.96]	1.13 [0.91-1.40]	1.23 [1.03-1.48]	1.12 [0.94-1.32]	1.14 [0.90-1.44]	1.32 [0.97-1.80]	1.07 [0.86-1.33]	1.20 [0.99-1.44]	380	187	108	225	302
Q53	1.00 [0.94-1.06]	1.12 [0.85-1.46]		0.99 [0.93-1.05]	0.96 [0.90-1.02]	0.98 [0.92-1.04]	1.05 [0.79-1.38]		0.97 [0.91-1.03]	0.96 [0.90-1.02]	3,035	126	NA	2,935	2,479
Q54	1.11 [1.00-1.23]	1.24 [1.03-1.49]	1.17 [0.96-1.41]	1.07 [0.95-1.22]	1.04 [0.93-1.17]	1.11 [0.99-1.23]	1.17 [0.97-1.41]	1.12 [0.92-1.36]	1.07 [0.94-1.22]	1.06 [0.94-1.19]	866	292	262	588	694
Q55	1.13 [1.06-1.22]	1.22 [0.96-1.55]		1.11 [1.03-1.19]	1.13 [1.04-1.22]	1.11 [1.03-1.19]	1.10 [0.86-1.42]		1.08 [1.01-1.16]	1.10 [1.02-1.19]	2,125	159	NA	2,015	1,779
Q60	1.17 [1.03-1.33]	1.20 [1.05-1.38]	1.23 [1.05-1.44]	0.92 [0.72-1.18]	1.07 [0.94-1.23]	1.12 [0.99-1.28]	1.08 [0.94-1.25]	1.13 [0.96-1.32]	0.92 [0.72-1.17]	1.06 [0.92-1.21]	628	502	394	166	552
Q61	1.16 [1.07-1.26]	1.14 [1.05-1.24]	1.19 [1.08-1.30]	1.05 [0.86-1.29]	1.07 [0.99-1.17]	1.11 [1.03-1.21]	1.04 [0.95-1.14]	1.10 [1.01-1.21]	1.00 [0.82-1.23]	1.05 [0.96-1.15]	1,502	1,317	1,141	239	1,307
Q62	1.00 [0.85-1.18]	1.01 [0.77-1.31]		0.99 [0.81-1.21]	0.96 [0.81-1.15]	0.98 [0.83-1.16]	0.98 [0.75-1.29]		0.97 [0.79-1.19]	0.96 [0.80-1.15]	338	130	NA	228	300
Q63	1.16 [1.06-1.27]	1.17 [1.05-1.30]	1.09 [0.96-1.24]	1.16 [1.00-1.34]	1.05 [0.96-1.16]	1.09 [0.99-1.19]	1.05 [0.94-1.17]	0.97 [0.85-1.10]	1.12 [0.96-1.30]	1.00 [0.91-1.10]	1,233	857	567	497	1,083
Q64	1.15 [0.95-1.38]	1.31 [0.99-1.74]		1.06 [0.84-1.33]	1.03 [0.85-1.26]	1.09 [0.90-1.32]	1.21 [0.91-1.62]		1.02 [0.80-1.29]	1.01 [0.82-1.24]	290	116	NA	192	253
Q65	1.04 [0.94-1.15]	1.17 [1.02-1.34]	1.18 [0.97-1.43]	0.89 [0.78-1.02]	0.93 [0.84-1.04]	0.99 [0.89-1.09]	1.07 [0.93-1.23]	1.08 [0.88-1.32]	0.86 [0.75-0.99]	0.91 [0.82-1.02]	947	502	238	525	815
Q66	1.26 [1.15-1.37]	1.38 [1.20-1.57]	1.60 [1.36-1.89]	1.12 [1.01-1.25]	1.12 [1.02-1.23]	1.19 [1.09-1.30]	1.25 [1.09-1.44]	1.44 [1.21-1.71]	1.08 [0.97-1.21]	1.09 [0.99-1.20]	1,328	528	363	871	1,111
Q67	1.20 [1.07-1.33]	1.41 [1.19-1.68]	1.56 [1.23-1.99]	1.08 [0.95-1.23]	1.10 [0.98-1.24]	1.14 [1.02-1.28]	1.30 [1.09-1.56]	1.47 [1.15-1.87]	1.01 [0.89-1.16]	1.07 [0.95-1.21]	831	319	177	598	720
Q68	1.21 [1.00-1.46]			1.11 [0.90-1.39]	1.03 [0.84-1.27]	1.16 [0.96-1.40]			1.06 [0.85-1.33]	1.00 [0.81-1.24]	288	NA	NA	211	241
Q69	0.90 [0.69-1.18]			0.91 [0.69-1.20]		0.88 [0.67-1.15]			0.89 [0.67-1.18]		125	NA	NA	118	NA
Q70	0.87 [0.65-1.16]					0.87 [0.65-1.16]					113	NA	NA	NA	NA
Q72	1.18 [0.88-1.57]				1.06 [0.78-1.43]	1.15 [0.86-1.54]				1.04 [0.76-1.42]	128	NA	NA	NA	113
Q74	1.21 [1.09-1.35]	1.28 [1.08-1.53]	1.35 [1.06-1.72]	1.17 [1.03-1.33]	1.11 [0.98-1.24]	1.17 [1.05-1.31]	1.16 [0.97-1.40]	1.18 [0.91-1.52]	1.15 [1.01-1.30]	1.08 [0.96-1.21]	889	316	160	646	741
Q75	1.03 [0.89-1.19]			1.00 [0.86-1.17]	0.98 [0.84-1.14]	0.99 [0.85-1.15]			0.97 [0.83-1.13]	0.95 [0.81-1.11]	433	NA	NA	401	396
Q76	1.15 [1.02-1.31]	1.23 [1.06-1.42]	1.35 [1.13-1.61]	0.91 [0.75-1.12]	1.07 [0.94-1.21]	1.06 [0.94-1.21]	1.05 [0.90-1.22]	1.11 [0.92-1.34]	0.87 [0.71-1.07]	1.02 [0.89-1.16]	639	444	293	242	556
Q78	1.22 [1.05-1.42]	1.31 [1.07-1.61]	1.30 [1.03-1.63]	1.06 [0.85-1.32]	1.12 [0.95-1.31]	1.15 [0.99-1.35]	1.17 [0.95-1.45]	1.12 [0.88-1.42]	1.03 [0.82-1.29]	1.07 [0.91-1.26]	429	237	179	205	385
Q79	1.35 [1.22-1.51]	1.34 [1.18-1.52]	1.19 [0.98-1.45]	1.24 [1.06-1.45]	1.19 [1.07-1.33]	1.13 [1.01-1.26]	1.01 [0.88-1.16]	0.91 [0.74-1.12]	1.12 [0.95-1.32]	1.04 [0.93-1.17]	920	629	242	451	807
Q80	2.79 [1.97-3.95]				2.74 [1.90-3.95]	2.58 [1.81-3.68]				2.50 [1.71-3.64]	119	NA	NA	NA	105
Q82	1.36 [1.21-1.52]	1.75 [1.47-2.07]	1.81 [1.49-2.21]	1.19 [1.03-1.38]	1.32 [1.17-1.49]	1.28 [1.14-1.44]	1.56 [1.31-1.86]	1.58 [1.29-1.94]	1.16 [1.00-1.34]	1.26 [1.11-1.43]	802	360	273	475	672
Q83	1.13 [0.96-1.33]	1.15 [0.96-1.38]		0.97 [0.76-1.25]	1.04 [0.86-1.26]	1.06 [0.90-1.26]	1.05 [0.87-1.26]		0.93 [0.73-1.20]	0.99 [0.82-1.21]	352	278	NA	154	250
Q85	1.02 [0.89-1.16]	1.05 [0.89-1.24]	1.08 [0.88-1.32]	0.96 [0.79-1.17]	0.94 [0.81-1.08]	0.97 [0.85-1.12]	0.95 [0.80-1.14]	1.00 [0.81-1.24]	0.94 [0.76-1.15]	0.92 [0.79-1.06]	515	317	215	240	444
Q87	1.23 [1.10-1.37]	1.44 [1.24-1.69]	1.59 [1.29-1.97]	1.05 [0.91-1.21]	1.14 [1.01-1.28]	1.15 [1.03-1.29]	1.30 [1.10-1.52]	1.42 [1.14-1.77]	1.02 [0.88-1.17]	1.10 [0.98-1.24]	847	406	215	512	751
Q89	1.09 [0.97-1.22]	1.20 [1.01-1.43]	1.15 [0.93-1.43]	1.02 [0.88-1.18]	1.06 [0.93-1.20]	1.06 [0.94-1.19]	1.09 [0.91-1.30]	1.08 [0.87-1.34]	1.01 [0.87-1.17]	1.04 [0.91-1.18]	716	315	199	451	615
Q90	2.77 [2.46-3.12]	3.76 [3.28-4.32]	4.16 [3.54-4.89]	1.03 [0.81-1.31]	2.00 [1.68-2.37]	2.68 [2.37-3.02]	3.86 [3.35-4.45]	4.05 [3.44-4.78]	1.00 [0.79-1.29]	1.90 [1.59-2.27]	855	725	526	161	367
Q92	1.12 [0.87-1.45]			0.98 [0.73-1.31]	1.18 [0.91-1.54]	1.07 [0.83-1.40]			0.95 [0.71-1.29]	1.15 [0.88-1.51]	151	NA	NA	113	143
Q93	1.27 [1.09-1.47]			1.19 [1.01-1.41]	1.25 [1.07-1.46]	1.17 [1.00-1.36]			1.12 [0.95-1.32]	1.17 [1.00-1.38]	466	NA	NA	398	436
Q96	1.62 [1.23-2.13]					1.65 [1.24-2.19]					140	NA	NA	NA	NA
Q98	1.77 [1.35-2.32]				1.63 [1.22-2.17]	1.60 [1.21-2.11]				1.50 [1.12-2.02]	149	NA	NA	NA	129
Q99	1.16 [1.00-1.33]	1.28 [1.00-1.65]		1.12 [0.95-1.31]	1.10 [0.95-1.27]	1.08 [0.94-1.25]	1.14 [0.88-1.47]		1.07 [0.91-1.26]	1.05 [0.90-1.21]	517	153	NA	395	472
Symptoms, signs and abnormal clinical and laboratory findings, not elsewhere classified (R00-R99)															
R00	1.21 [1.19-1.23]	1.22 [1.20-1.24]	1.22 [1.20-1.24]	1.14 [1.10-1.18]	1.14 [1.12-1.16]	1.14 [1.13-1.16]	1.11 [1.09-1.13]	1.11 [1.09-1.13]	1.10 [1.06-1.15]	1.10 [1.08-1.12]	44,637	40,927	32,645	7,228	39,435
R01	1.21 [1.17-1.26]	1.22 [1.17-1.27]	1.23 [1.18-1.29]	1.21 [1.13-1.29]	1.17 [1.13-1.22]	1.15 [1.11-1.19]	1.12 [1.07-1.17]	1.14 [1.08-1.19]	1.16 [1.09-1.24]	1.13 [1.09-1.18]	7,450	5,428	4,624	2,324	6,736
R02	1.22 [1.17-1.27]	1.23 [1.18-1.28]	1.22 [1.17-1.28]	1.08 [0.92-1.28]	1.16 [1.11-1.21]	1.19 [1.14-1.24]	1.13 [1.08-1.18]	1.12 [1.07-1.17]	1.07 [0.91-1.27]	1.16 [1.11-1.21]	6,432	6,203	5,777	376	5,857
R03	1.18 [1.15-1.22]	1.19 [1.16-1.22]	1.20 [1.17-1.24]	1.13 [1.04-1.22]	1.12 [1.09-1.15]	1.14 [1.11-1.17]	1.12 [1.09-1.15]	1.13 [1.10-1.16]	1.10 [1.02-1.20]	1.10 [1.07-1.13]	15,117	14,446	11,408	1,719	13,251
R04	1.33 [1.30-1.36]	1.35 [1.31-1.38]	1.34 [1.31-1.38]	1.24 [1.17-1.31]	1.23 [1.20-1.26]	1.25 [1.22-1.28]	1.22 [1.18-1.25]	1.21 [1.18-1.24]	1.19 [1.12-1.26]	1.19 [1.16-1.22]	18,561	15,989	14,425	3,187	16,162
R05	1.38 [1.35-1.41]	1.35 [1.32-1.39]	1.36 [1.32-1.39]	1.37 [1.32-1.43]	1.29 [1.26-1.32]	1.27 [1.24-1.30]	1.19 [1.15-1.22]	1.19 [1.15-1.22]	1.30 [1.25-1.35]	1.21 [1.18-1.24]	20,701	14,452	12,383	6,900	18,750
R06	1.52 [1.50-1.54]	1.34 [1.32-1.36]	1.33 [1.31-1.35]	1.76 [1.72-1.79]	1.43 [1.41-1.44]	1.43 [1.41-1.44]	1.18 [1.16-1.20]	1.18 [1.16-1.20]	1.70 [1.67-1.73]	1.36 [1.35-1.38]	74,870	41,003	34,545	35,908	67,136
R07	1.29 [1.28-1.31]	1.30 [1.29-1.32]	1.31 [1.29-1.33]	1.14 [1.10-1.18]	1.19 [1.17-1.20]	1.20 [1.18-1.21]	1.16 [1.14-1.17]	1.16 [1.14-1.17]	1.10 [1.06-1.15]	1.13 [1.11-1.14]	77,085	74,192	60,170	7,686	65,437
R09	1.31 [1.26-1.36]	1.32 [1.27-1.37]	1.31 [1.25-1.36]	1.21 [1.08-1.34]	1.22 [1.17-1.27]	1.22 [1.17-1.26]	1.17 [1.12-1.22]	1.17 [1.11-1.22]	1.15 [1.03-1.29]	1.16 [1.12-1.21]	6,918	6,381	5,412	887	6,244
R10	1.27 [1.26-1.28]	1.28 [1.27-1.29]	1.30 [1.29-1.32]	1.19 [1.17-1.21]	1.18 [1.17-1.19]	1.18 [1.17-1.19]	1.14 [1.13-1.15]	1.16 [1.14-1.18]	1.14 [1.12-1.16]	1.12 [1.11-1.13]	116,393	93,858	55,106	37,214	94,513
R11	1.23 [1.22-1.25]	1.25 [1.23-1.27]	1.25 [1.23-1.27]	1.17 [1.14-1.19]	1.15 [1.13-1.17]	1.16 [1.14-1.17]	1.12 [1.10-1.14]	1.13 [1.11-1.15]	1.13 [1.10-1.15]	1.11 [1.09-1.12]	66,001	49,305	37,038	21,941	57,542
R12	1.35 [1.29-1.42]	1.35 [1.28-1.42]	1.34 [1.27-1.42]	1.08 [0.90-1.28]	1.23 [1.17-1.30]	1.22 [1.16-1.28]	1.18 [1.12-1.24]	1.17 [1.11-1.24]	1.00 [0.83-1.20]	1.14 [1.08-1.20]	4,130	4,046	3,156	336	3,611
R13	1.31 [1.29-1.34]	1.32 [1.29-1.34]	1.31 [1.29-1.34]	1.21 [1.11-1.32]	1.23 [1.21-1.25]	1.22 [1.19-1.24]	1.18 [1.16-1.21]	1.18 [1.16-1.21]	1.12 [1.02-1.23]	1.17 [1.15-1.19]	30,132	29,322	27,273	1,407	27,584
R14	1.31 [1.27-1.36]	1.32 [1.27-1.37]	1.30 [1.25-1.36]	1.16 [1.04-1.30]	1.22 [1.17-1.26]	1.20 [1.16-1.25]	1.15 [1.11-1.20]	1.15 [1.10-1.20]	1.11 [0.99-1.24]	1.14 [1.10-1.19]	7,064	6,608	5,225	903	6,356
R15	1.20 [1.16-1.24]	1.20 [1.17-1.24]	1.19 [1.15-1.23]	1.14 [0.99-1.31]	1.14 [1.10-1.17]	1.16 [1.13-1.20]	1.13 [1.09-1.16]	1.12 [1.09-1.16]	1.09 [0.95-1.25]	1.13 [1.09-1.16]	12,111	11,699	11,132	560	11,344
R16	1.38 [1.32-1.45]	1.44 [1.38-1.51]	1.41 [1.34-1.49]	1.11 [0.99-1.24]	1.27 [1.21-1.33]	1.30 [1.24-1.36]	1.28 [1.22-1.35]	1.27 [1.20-1.34]	1.08 [0.96-1.21]	1.23 [1.17-1.29]	5,156	4,624	3,839	829	4,592
R17	1.22 [1.16-1.28]	1.24 [1.18-1.31]	1.23 [1.17-1.30]	1.12 [0.94-1.33]	1.19 [1.12-1.25]	1.19 [1.13-1.25]	1.18 [1.12-1.24]	1.17 [1.11-1.24]	1.13 [0.95-1.34]	1.18 [1.12-1.25]	3,810	3,566	3,200	362	3,246
R18	1.20 [1.17-1.24]	1.22 [1.18-1.26]	1.22 [1.18-1.26]	0.94 [0.81-1.10]	1.17 [1.13-1.21]	1.17 [1.14-1.21]	1.15 [1.11-1.19]	1.15 [1.11-1.19]	0.93 [0.80-1.09]	1.17 [1.13-1.21]	9,491	9,236	8,539	441	8,376
R19	1.34 [1.31-1.36]	1.35 [1.33-1.37]	1.33 [1.31-1.36]	1.19 [1.13-1.26]	1.25 [1.23-1.27]	1.23 [1.21-1.25]	1.21 [1.19-1.23]	1.19 [1.17-1.21]	1.14 [1.08-1.20]	1.18 [1.16-1.20]	39,491	37,905	31,773	3,664	34,526
R20	1.27 [1.23-1.30]	1.27 [1.23-1.31]	1.27 [1.23-1.32]	1.15 [1.06-1.24]	1.16 [1.13-1.20]	1.16 [1.12-1.20]	1.11 [1.07-1.14]	1.11 [1.07-1.15]	1.09 [1.00-1.18]	1.10 [1.06-1.13]	10,595	9,813	7,028	1,678	9,380
R21	1.46 [1.42-1.49]	1.70 [1.65-1.75]	1.78 [1.72-1.84]	1.17 [1.14-1.21]	1.37 [1.34-1.40]	1.39 [1.35-1.42]	1.55 [1.50-1.60]	1.63 [1.58-1.69]	1.13 [1.10-1.17]	1.32 [1.29-1.35]	19,887	11,833	9,506	8,865	17,409
R22	1.38 [1.34-1.43]	1.37 [1.31-1.42]	1.36 [1.31-1.42]	1.42 [1.33-1.52]	1.31 [1.27-1.36]	1.29 [1.25-1.34]	1.22 [1.17-1.27]	1.23 [1.18-1.29]	1.36 [1.27-1.45]	1.25 [1.20-1.29]	8,990	6,898	5,433	2,598	7,743
R23	1.25 [1.20-1.29]	1.33 [1.26-1.40]	1.33 [1.26-1.41]	1.16 [1.10-1.22]	1.18 [1.14-1.23]	1.19 [1.14-1.24]	1.21 [1.15-1.28]	1.21 [1.14-1.28]	1.12 [1.06-1.18]	1.14 [1.10-1.19]	7,127	3,782	3,302	3,510	6,405
R25	1.24 [1.19-1.29]	1.28 [1.23-1.34]	1.28 [1.22-1.35]	1.10 [1.01-1.20]	1.16 [1.12-1.21]	1.15 [1.10-1.20]	1.14 [1.09-1.19]	1.14 [1.09-1.20]	1.06 [0.97-1.16]	1.11 [1.07-1.16]	6,270	5,246	4,471	1,304	5,683
R26	1.21 [1.19-1.23]	1.20 [1.19-1.22]	1.20 [1.18-1.22]	1.10 [1.03-1.17]	1.15 [1.13-1.17]	1.16 [1.15-1.18]	1.12 [1.10-1.14]	1.12 [1.10-1.14]	1.07 [1.00-1.15]	1.13 [1.11-1.15]	45,075	43,012	42,038	2,374	42,416
R27	1.18 [1.10-1.27]	1.18 [1.08-1.29]	1.20 [1.09-1.31]	1.17 [1.03-1.33]	1.12 [1.04-1.21]	1.12 [1.04-1.21]	1.07 [0.98-1.16]	1.09 [0.99-1.20]	1.12 [0.98-1.28]	1.09 [1.01-1.18]	1,904	1,344	1,171	629	1,698
R29	1.19 [1.18-1.21]	1.19 [1.18-1.21]	1.19 [1.17-1.21]	1.07 [0.99-1.17]	1.14 [1.13-1.16]	1.15 [1.13-1.16]	1.10 [1.09-1.12]	1.11 [1.09-1.12]	1.02 [0.94-1.11]	1.12 [1.11-1.14]	58,332	57,338	55,769	1,516	55,239
R30	1.35 [1.29-1.41]	1.34 [1.28-1.41]	1.36 [1.29-1.44]	1.18 [1.08-1.29]	1.21 [1.16-1.27]	1.24 [1.19-1.30]	1.17 [1.11-1.23]	1.18 [1.11-1.25]	1.14 [1.05-1.25]	1.14 [1.09-1.20]	5,315	4,379	3,136	1,412	4,639
R31	1.19 [1.17-1.22]	1.19 [1.17-1.21]	1.18 [1.16-1.21]	1.14 [1.06-1.22]	1.11 [1.09-1.13]	1.15 [1.13-1.17]	1.12 [1.10-1.14]	1.11 [1.09-1.13]	1.09 [1.02-1.17]	1.09 [1.07-1.11]	31,241	29,856	26,969	2,259	26,387
R32	1.19 [1.17-1.22]	1.18 [1.16-1.21]	1.17 [1.15-1.20]	1.15 [1.07-1.25]	1.13 [1.10-1.15]	1.15 [1.13-1.18]	1.10 [1.08-1.13]	1.10 [1.08-1.13]	1.10 [1.02-1.20]	1.11 [1.09-1.14]	23,400	22,061	20,863	1,701	21,813
R33	1.17 [1.15-1.19]	1.16 [1.14-1.18]	1.16 [1.14-1.18]	1.16 [1.08-1.25]	1.12 [1.10-1.14]	1.13 [1.11-1.15]	1.10 [1.08-1.11]	1.10 [1.08-1.12]	1.10 [1.02-1.19]	1.10 [1.09-1.12]	43,140	42,266	39,647	1,817	39,109
R34	1.21 [1.11-1.33]	1.30 [1.18-1.43]	1.26 [1.14-1.39]	0.95 [0.73-1.23]	1.23 [1.11-1.35]	1.15 [1.05-1.27]	1.17 [1.06-1.30]	1.15 [1.03-1.27]	0.92 [0.71-1.21]	1.20 [1.09-1.32]	1,225	1,113	1,027	145	1,131
R35	1.35 [1.30-1.40]	1.33 [1.29-1.38]	1.32 [1.27-1.38]	1.22 [1.10-1.35]	1.23 [1.19-1.28]	1.26 [1.21-1.30]	1.20 [1.15-1.25]	1.19 [1.14-1.24]	1.19 [1.07-1.32]	1.17 [1.12-1.21]	7,985	7,287	6,145	1,046	6,959
R36	1.28 [1.00-1.63]	1.35 [1.04-1.75]	1.39 [1.05-1.85]		1.19 [0.92-1.54]	1.25 [0.97-1.60]	1.23 [0.94-1.61]	1.24 [0.92-1.66]		1.15 [0.88-1.48]	176	156	132	NA	155
R39	1.35 [1.30-1.39]	1.35 [1.31-1.40]	1.33 [1.28-1.38]	1.31 [1.19-1.45]	1.22 [1.18-1.26]	1.25 [1.21-1.29]	1.21 [1.16-1.25]	1.19 [1.15-1.23]	1.25 [1.13-1.39]	1.16 [1.12-1.20]	9,110	8,523	7,284	1,019	7,999
R40	1.21 [1.17-1.26]	1.23 [1.18-1.28]	1.24 [1.18-1.29]	1.05 [0.95-1.16]	1.15 [1.11-1.20]	1.15 [1.11-1.20]	1.12 [1.07-1.16]	1.12 [1.08-1.17]	1.02 [0.92-1.13]	1.13 [1.08-1.17]	7,295	6,480	5,786	1,095	6,730
R41	1.17 [1.16-1.19]	1.18 [1.16-1.20]	1.17 [1.15-1.19]	1.09 [1.01-1.18]	1.14 [1.12-1.16]	1.14 [1.12-1.16]	1.11 [1.09-1.13]	1.11 [1.09-1.13]	1.04 [0.96-1.13]	1.13 [1.11-1.15]	41,977	40,673	39,528	1,764	38,419
R42	1.24 [1.21-1.26]	1.25 [1.22-1.27]	1.24 [1.22-1.27]	1.11 [1.05-1.18]	1.16 [1.13-1.18]	1.15 [1.13-1.18]	1.12 [1.09-1.14]	1.12 [1.09-1.15]	1.07 [1.01-1.14]	1.10 [1.08-1.13]	24,441	22,855	19,322	2,819	22,003

Supplementary Table 3: Hazard ratios and events from all cohorts

	Hazard ratio (99% confidence interval)														
	crude					adjusted					Events (in exposed)				
Outcome	any age	18+	40+	<18	hosp.	any age	18+	40+	<18	hosp.	any age	18+	40+	<18	hosp.
R43	1.35 [1.14-1.61]	1.28 [1.07-1.52]	1.36 [1.11-1.66]		1.24 [1.04-1.49]	1.23 [1.03-1.47]	1.12 [0.93-1.34]	1.14 [0.92-1.41]		1.17 [0.98-1.41]	348	318	243	NA	305
R44	1.21 [1.16-1.26]	1.22 [1.17-1.28]	1.22 [1.17-1.28]	1.14 [1.03-1.26]	1.15 [1.10-1.19]	1.15 [1.11-1.20]	1.11 [1.07-1.17]	1.12 [1.07-1.17]	1.10 [1.00-1.22]	1.12 [1.08-1.17]	6,419	5,617	4,922	1,109	5,851
R45	1.19 [1.16-1.22]	1.19 [1.15-1.22]	1.24 [1.20-1.28]	1.06 [1.02-1.12]	1.12 [1.09-1.15]	1.13 [1.10-1.16]	1.06 [1.03-1.09]	1.11 [1.07-1.15]	1.04 [0.99-1.10]	1.10 [1.07-1.13]	15,065	11,627	8,843	4,850	13,574
R46	1.13 [1.08-1.19]	1.13 [1.07-1.19]	1.12 [1.06-1.19]	1.06 [0.94-1.19]	1.10 [1.04-1.16]	1.11 [1.06-1.17]	1.07 [1.01-1.13]	1.07 [1.01-1.13]	1.00 [0.89-1.14]	1.11 [1.05-1.17]	3,990	3,358	3,052	731	3,662
R47	1.17 [1.13-1.20]	1.17 [1.13-1.20]	1.16 [1.13-1.20]	1.09 [0.97-1.22]	1.11 [1.08-1.15]	1.13 [1.09-1.16]	1.08 [1.05-1.12]	1.08 [1.04-1.11]	1.04 [0.92-1.18]	1.10 [1.06-1.13]	11,628	11,115	10,439	719	10,584
R48	1.29 [1.12-1.48]	1.32 [1.11-1.56]	1.31 [1.03-1.66]	1.16 [0.96-1.40]	1.23 [1.06-1.42]	1.15 [1.00-1.33]	1.10 [0.92-1.32]	1.13 [0.88-1.45]	1.09 [0.90-1.32]	1.16 [1.00-1.35]	570	352	175	309	512
R49	1.35 [1.27-1.44]	1.35 [1.27-1.44]	1.32 [1.24-1.42]	1.25 [1.05-1.48]	1.23 [1.15-1.31]	1.23 [1.16-1.32]	1.17 [1.10-1.26]	1.14 [1.07-1.23]	1.20 [1.01-1.42]	1.15 [1.08-1.23]	2,591	2,269	2,050	364	2,279
R50	1.22 [1.20-1.25]	1.27 [1.24-1.30]	1.27 [1.24-1.31]	1.15 [1.12-1.18]	1.16 [1.14-1.18]	1.17 [1.15-1.19]	1.17 [1.14-1.20]	1.17 [1.14-1.21]	1.12 [1.09-1.15]	1.13 [1.11-1.15]	28,721	16,386	12,681	13,748	25,177
R51	1.28 [1.26-1.30]	1.29 [1.27-1.32]	1.32 [1.29-1.35]	1.20 [1.17-1.24]	1.19 [1.17-1.21]	1.18 [1.16-1.20]	1.13 [1.11-1.15]	1.15 [1.13-1.18]	1.15 [1.12-1.19]	1.12 [1.10-1.14]	38,088	30,723	19,242	11,486	32,875
R52	1.40 [1.34-1.46]	1.42 [1.36-1.48]	1.42 [1.35-1.48]	1.12 [0.98-1.28]	1.28 [1.23-1.34]	1.26 [1.21-1.31]	1.20 [1.15-1.25]	1.21 [1.15-1.27]	1.06 [0.93-1.22]	1.20 [1.15-1.25]	6,030	5,669	4,672	610	5,631
R53	1.24 [1.21-1.27]	1.23 [1.20-1.26]	1.22 [1.19-1.25]	1.22 [1.16-1.29]	1.17 [1.14-1.19]	1.18 [1.15-1.20]	1.12 [1.10-1.15]	1.12 [1.09-1.14]	1.18 [1.11-1.25]	1.13 [1.10-1.16]	23,449	20,786	18,510	3,424	21,508
R54	1.17 [1.14-1.19]	1.17 [1.15-1.19]	1.17 [1.14-1.19]		1.13 [1.10-1.15]	1.14 [1.12-1.17]	1.11 [1.09-1.14]	1.11 [1.08-1.13]		1.12 [1.10-1.14]	26,745	26,733	26,699	NA	24,911
R55	1.20 [1.18-1.21]	1.20 [1.18-1.22]	1.20 [1.18-1.22]	1.10 [1.05-1.14]	1.14 [1.12-1.16]	1.15 [1.13-1.17]	1.12 [1.10-1.14]	1.12 [1.10-1.14]	1.05 [1.01-1.10]	1.11 [1.09-1.13]	43,614	40,258	35,478	5,290	38,136
R56	1.14 [1.11-1.16]	1.26 [1.22-1.29]	1.26 [1.22-1.30]	1.02 [0.99-1.05]	1.07 [1.05-1.10]	1.10 [1.08-1.13]	1.15 [1.12-1.19]	1.16 [1.13-1.20]	1.01 [0.98-1.04]	1.06 [1.04-1.09]	20,741	11,494	9,214	10,145	17,909
R57	1.18 [1.12-1.25]	1.20 [1.13-1.26]	1.18 [1.11-1.24]	1.01 [0.80-1.29]	1.15 [1.09-1.21]	1.15 [1.09-1.22]	1.12 [1.06-1.18]	1.10 [1.04-1.16]	1.03 [0.80-1.32]	1.15 [1.08-1.21]	3,599	3,468	3,306	179	3,298
R58	1.35 [1.19-1.53]	1.34 [1.17-1.52]	1.34 [1.17-1.54]		1.30 [1.14-1.48]	1.30 [1.14-1.48]	1.22 [1.07-1.40]	1.22 [1.06-1.41]		1.29 [1.13-1.48]	644	582	533	NA	597
R59	1.35 [1.32-1.39]	1.36 [1.32-1.40]	1.32 [1.28-1.37]	1.29 [1.22-1.36]	1.27 [1.24-1.31]	1.31 [1.28-1.35]	1.27 [1.23-1.31]	1.24 [1.20-1.29]	1.25 [1.18-1.32]	1.26 [1.22-1.29]	13,940	11,011	9,187	3,575	11,969
R60	1.33 [1.30-1.36]	1.33 [1.30-1.36]	1.34 [1.31-1.37]	1.21 [1.05-1.40]	1.27 [1.24-1.30]	1.26 [1.23-1.29]	1.22 [1.19-1.24]	1.22 [1.19-1.25]	1.16 [1.00-1.35]	1.22 [1.19-1.25]	20,865	20,570	19,881	481	19,325
R61	1.36 [1.27-1.45]	1.38 [1.29-1.48]	1.34 [1.24-1.45]	1.24 [1.07-1.45]	1.23 [1.15-1.32]	1.24 [1.16-1.32]	1.21 [1.13-1.30]	1.17 [1.08-1.27]	1.18 [1.01-1.37]	1.16 [1.09-1.25]	2,502	2,240	1,590	476	2,119
R62	1.07 [1.01-1.12]	1.09 [0.93-1.27]	1.26 [1.02-1.56]	1.06 [1.00-1.12]	0.99 [0.94-1.05]	1.02 [0.97-1.07]	1.00 [0.85-1.17]	1.18 [0.95-1.46]	1.02 [0.96-1.07]	0.97 [0.92-1.02]	3,726	407	228	3,386	3,335
R63	1.24 [1.22-1.26]	1.25 [1.23-1.27]	1.24 [1.22-1.27]	1.14 [1.10-1.19]	1.18 [1.16-1.20]	1.18 [1.16-1.20]	1.14 [1.12-1.16]	1.14 [1.12-1.16]	1.11 [1.06-1.15]	1.14 [1.13-1.16]	44,131	39,308	35,255	6,420	39,412
R64	1.16 [1.05-1.28]	1.12 [1.01-1.24]	1.10 [1.00-1.22]		1.09 [0.99-1.21]	1.17 [1.06-1.30]	1.09 [0.99-1.21]	1.08 [0.97-1.20]		1.12 [1.01-1.25]	1,044	1,034	1,014	NA	970
R65	1.23 [1.16-1.31]	1.22 [1.14-1.30]	1.23 [1.15-1.32]	1.21 [0.90-1.63]	1.18 [1.11-1.26]	1.18 [1.10-1.26]	1.12 [1.05-1.20]	1.13 [1.05-1.21]	1.20 [0.88-1.63]	1.15 [1.08-1.23]	2,566	2,506	2,352	123	2,426
R68	1.21 [1.15-1.27]	1.24 [1.18-1.31]	1.24 [1.17-1.31]	1.03 [0.91-1.17]	1.17 [1.11-1.23]	1.19 [1.13-1.25]	1.18 [1.12-1.25]	1.17 [1.11-1.24]	1.01 [0.89-1.15]	1.16 [1.10-1.23]	4,208	3,725	3,469	576	3,872
R69	1.25 [1.23-1.27]	1.24 [1.22-1.27]	1.24 [1.22-1.27]	1.20 [1.15-1.26]	1.16 [1.14-1.18]	1.19 [1.18-1.22]	1.15 [1.13-1.17]	1.16 [1.14-1.18]	1.17 [1.11-1.22]	1.13 [1.11-1.15]	37,013	33,821	27,560	4,814	29,988
R70	1.50 [1.27-1.78]	1.46 [1.22-1.75]	1.48 [1.23-1.77]		1.35 [1.12-1.62]	1.43 [1.20-1.70]	1.35 [1.13-1.62]	1.37 [1.13-1.65]		1.28 [1.06-1.55]	359	318	293	NA	297
R71	1.22 [1.00-1.49]	1.11 [0.90-1.36]	1.13 [0.91-1.40]		1.11 [0.90-1.36]	1.18 [0.97-1.45]	1.05 [0.85-1.30]	1.06 [0.85-1.33]		1.09 [0.88-1.35]	249	223	200	NA	217
R72	1.23 [1.13-1.34]	1.20 [1.10-1.31]	1.20 [1.09-1.32]	1.22 [0.97-1.52]	1.15 [1.05-1.26]	1.18 [1.09-1.29]	1.10 [1.01-1.21]	1.13 [1.03-1.25]	1.16 [0.92-1.46]	1.13 [1.03-1.23]	1,398	1,274	1,059	214	1,232
R73	1.31 [1.26-1.37]	1.32 [1.26-1.38]	1.31 [1.25-1.38]	1.20 [1.04-1.40]	1.25 [1.20-1.31]	1.21 [1.16-1.27]	1.17 [1.12-1.23]	1.16 [1.11-1.22]	1.13 [0.97-1.32]	1.19 [1.14-1.25]	5,465	5,101	4,622	481	4,997
R74	1.21 [1.14-1.30]	1.17 [1.09-1.26]	1.21 [1.12-1.30]	1.24 [1.05-1.46]	1.15 [1.08-1.23]	1.15 [1.07-1.23]	1.06 [0.98-1.14]	1.10 [1.02-1.19]	1.14 [0.96-1.36]	1.12 [1.04-1.20]	2,353	2,104	1,804	390	2,149
R76	1.38 [1.23-1.56]	1.37 [1.21-1.56]	1.38 [1.20-1.60]	1.29 [1.02-1.64]	1.29 [1.14-1.46]	1.31 [1.16-1.47]	1.26 [1.11-1.44]	1.26 [1.09-1.46]	1.24 [0.97-1.59]	1.23 [1.08-1.40]	749	608	480	189	641
R77	1.26 [1.16-1.37]	1.29 [1.18-1.40]	1.27 [1.16-1.40]	1.12 [0.87-1.45]	1.23 [1.13-1.34]	1.18 [1.09-1.29]	1.16 [1.07-1.27]	1.17 [1.06-1.28]	1.12 [0.86-1.46]	1.18 [1.08-1.29]	1,496	1,395	1,220	167	1,385
R78	1.19 [0.95-1.49]	1.16 [0.91-1.46]	1.12 [0.88-1.45]		1.04 [0.82-1.33]	1.15 [0.91-1.45]	1.04 [0.81-1.32]	1.02 [0.79-1.32]		1.05 [0.82-1.35]	186	174	153	NA	155
R79	1.24 [1.22-1.26]	1.24 [1.21-1.26]	1.24 [1.22-1.26]	1.18 [1.09-1.28]	1.17 [1.15-1.19]	1.18 [1.15-1.20]	1.14 [1.11-1.16]	1.14 [1.11-1.16]	1.14 [1.05-1.25]	1.14 [1.11-1.16]	28,047	27,092	25,510	1,523	25,528
R80	1.33 [1.22-1.45]	1.38 [1.25-1.53]	1.45 [1.29-1.62]	1.18 [0.99-1.40]	1.23 [1.12-1.36]	1.28 [1.17-1.40]	1.26 [1.14-1.40]	1.31 [1.17-1.47]	1.17 [0.98-1.40]	1.20 [1.09-1.32]	1,292	1,006	788	366	1,101
R81	1.20 [1.02-1.42]	1.15 [0.96-1.38]		1.24 [0.92-1.67]	1.20 [0.99-1.44]	1.15 [0.97-1.36]	1.09 [0.90-1.31]		1.24 [0.91-1.69]	1.17 [0.97-1.42]	340	262	NA	113	265
R82	1.20 [1.13-1.28]	1.21 [1.13-1.30]	1.16 [1.07-1.25]	1.18 [1.03-1.35]	1.12 [1.05-1.20]	1.15 [1.07-1.22]	1.10 [1.03-1.18]	1.06 [0.98-1.15]	1.15 [1.00-1.32]	1.10 [1.02-1.17]	2,432	2,073	1,563	562	2,206
R83	1.20 [0.89-1.62]					1.14 [0.84-1.56]					101	NA	NA	NA	NA
R84	1.29 [1.15-1.44]	1.36 [1.20-1.54]	1.45 [1.28-1.65]	1.05 [0.84-1.30]	1.19 [1.06-1.33]	1.16 [1.03-1.30]	1.15 [1.01-1.31]	1.24 [1.09-1.42]	0.99 [0.80-1.24]	1.08 [0.96-1.21]	858	668	615	216	782
R85	1.32 [1.14-1.53]	1.39 [1.19-1.62]	1.32 [1.11-1.58]		1.32 [1.13-1.54]	1.21 [1.04-1.40]	1.25 [1.06-1.46]	1.16 [0.97-1.39]		1.22 [1.04-1.43]	460	417	314	NA	404
R87	1.16 [1.08-1.24]	1.17 [1.09-1.25]	1.08 [0.97-1.21]	1.09 [0.87-1.37]	1.07 [0.99-1.16]	1.12 [1.05-1.20]	1.11 [1.03-1.19]	1.03 [0.92-1.15]	1.08 [0.86-1.37]	1.06 [0.97-1.15]	1,971	1,966	747	186	1,436
R89	1.48 [1.27-1.73]	1.44 [1.22-1.70]	1.47 [1.23-1.75]	1.53 [1.10-2.14]	1.31 [1.11-1.53]	1.41 [1.21-1.65]	1.31 [1.11-1.55]	1.35 [1.13-1.62]	1.40 [0.99-1.97]	1.26 [1.07-1.49]	452	376	326	101	401
R90	1.21 [1.11-1.32]	1.19 [1.08-1.30]	1.15 [1.04-1.27]	1.17 [0.95-1.45]	1.17 [1.07-1.28]	1.16 [1.06-1.27]	1.10 [1.00-1.21]	1.08 [0.98-1.20]	1.14 [0.92-1.41]	1.14 [1.04-1.26]	1,302	1,132	949	229	1,147
R91	1.26 [1.22-1.29]	1.25 [1.22-1.29]	1.25 [1.22-1.29]	1.29 [1.09-1.53]	1.20 [1.17-1.24]	1.20 [1.17-1.24]	1.15 [1.12-1.18]	1.15 [1.12-1.18]	1.26 [1.06-1.50]	1.17 [1.14-1.21]	13,726	13,469	13,019	377	12,284
R92	1.09 [0.81-1.46]	1.09 [0.81-1.46]	1.07 [0.80-1.44]			1.06 [0.78-1.43]	0.97 [0.72-1.32]	1.01 [0.75-1.37]			109	110	108	NA	NA
R93	1.21 [1.18-1.25]	1.21 [1.18-1.25]	1.20 [1.17-1.24]	1.07 [0.96-1.19]	1.14 [1.11-1.18]	1.16 [1.13-1.19]	1.12 [1.09-1.15]	1.11 [1.08-1.15]	1.04 [0.93-1.16]	1.12 [1.08-1.15]	13,695	13,179	11,961	913	12,289
R94	1.26 [1.24-1.29]	1.26 [1.24-1.29]	1.26 [1.23-1.29]	1.16 [1.08-1.24]	1.19 [1.16-1.21]	1.21 [1.18-1.23]	1.17 [1.14-1.19]	1.16 [1.14-1.19]	1.12 [1.04-1.21]	1.16 [1.14-1.19]	25,164	24,148	21,195	2,130	22,573
Injury, poisoning and certain other consequences of external causes (S00-T88)															
S00	1.13 [1.11-1.15]	1.19 [1.16-1.21]	1.20 [1.17-1.23]	0.97 [0.93-1.01]	1.08 [1.06-1.11]	1.10 [1.08-1.12]	1.11 [1.08-1.13]	1.12 [1.09-1.15]	0.95 [0.92-0.99]	1.07 [1.05-1.09]	26,675	21,323	18,953	6,459	23,816
S01	1.13 [1.11-1.15]	1.18 [1.16-1.21]	1.20 [1.18-1.23]	0.97 [0.94-1.01]	1.08 [1.06-1.11]	1.10 [1.08-1.12]	1.12 [1.09-1.14]	1.14 [1.11-1.17]	0.96 [0.93-1.00]	1.07 [1.05-1.09]	28,522	21,362	18,583	8,473	24,850
S02	1.11 [1.07-1.14]	1.16 [1.12-1.20]	1.21 [1.16-1.27]	1.02 [0.98-1.07]	1.04 [1.00-1.07]	1.08 [1.04-1.11]	1.09 [1.05-1.13]	1.14 [1.09-1.20]	1.01 [0.96-1.06]	1.02 [0.99-1.06]	10,815	7,891	4,370	4,829	8,181
S03	1.08 [0.94-1.25]	1.14 [0.91-1.41]	1.16 [0.86-1.57]	1.04 [0.88-1.23]	1.07 [0.92-1.25]	1.04 [0.90-1.21]	1.05 [0.84-1.31]	1.07 [0.79-1.45]	1.02 [0.85-1.21]	1.06 [0.90-1.24]	483	198	110	336	419
S05	1.14 [1.06-1.21]	1.15 [1.07-1.23]	1.21 [1.11-1.31]	0.95 [0.83-1.09]	1.10 [1.02-1.18]	1.11 [1.03-1.18]	1.08 [1.01-1.16]	1.14 [1.05-1.23]	0.93 [0.81-1.07]	1.09 [1.02-1.17]	2,317	1,933	1,618	523	1,974
S06	1.11 [1.07-1.15]	1.13 [1.08-1.17]	1.14 [1.09-1.19]	1.05 [0.97-1.14]	1.06 [1.01-1.10]	1.09 [1.05-1.13]	1.07 [1.03-1.11]	1.08 [1.03-1.13]	1.04 [0.95-1.13]	1.05 [1.01-1.09]	7,461	6,446	5,546	1,470	6,400
S09	1.16 [1.13-1.19]	1.21 [1.18-1.25]	1.25 [1.21-1.30]	1.05 [1.01-1.10]	1.11 [1.08-1.14]	1.11 [1.08-1.14]	1.11 [1.07-1.15]	1.15 [1.11-1.19]	1.04 [0.99-1.08]	1.08 [1.05-1.12]	15,718	10,281	8,656	6,130	13,300
S10	1.05 [0.94-1.17]	1.13 [0.99-1.29]	1.09 [0.93-1.29]	0.96 [0.81-1.15]	0.97 [0.86-1.10]	1.00 [0.89-1.12]	1.02 [0.89-1.17]	0.97 [0.82-1.15]	0.96 [0.80-1.15]	0.96 [0.85-1.09]	794	563	376	321	682
S11	1.19 [1.03-1.38]	1.19 [1.01-1.40]	1.46 [1.17-1.82]	1.05 [0.82-1.33]	1.16 [0.98-1.36]	1.13 [0.97-1.31]	1.09 [0.92-1.29]	1.34 [1.06-1.68]	1.01 [0.79-1.30]	1.16 [0.98-1.38]	456	361	215	174	377
S12	1.18 [1.09-1.27]	1.18 [1.09-1.27]	1.18 [1.08-1.28]	1.00 [0.77-1.29]	1.15 [1.06-1.25]	1.15 [1.06-1.24]	1.11 [1.02-1.20]	1.10 [1.01-1.20]	1.01 [0.77-1.32]	1.15 [1.06-1.24]	1,813	1,759	1,558	146	1,611
S13	1.20 [1.04-1.38]	1.20 [1.03-1.40]	1.21 [1.00-1.47]	1.01 [0.79-1.30]	1.14 [0.98-1.33]	1.12 [0.97-1.30]	1.09 [0.92-1.27]	1.12 [0.92-1.36]	0.98 [0.76-1.27]	1.10 [0.94-1.29]	493	393	268	156	411
S14	1.07 [0.90-1.27]	1.07 [0.90-1.28]	1.08 [0.89-1.32]		1.01 [0.84-1.22]	1.07 [0.90-1.27]	1.04 [0.87-1.24]	1.06 [0.87-1.30]		1.02 [0.84-1.24]	332	301	238	NA	261
S19	1.24 [1.10-1.41]	1.30 [1.13-1.49]	1.28 [1.08-1.52]	1.10 [0.89-1.36]	1.16 [1.02-1.32]	1.15 [1.02-1.30]	1.14 [0.99-1.32]	1.11 [0.93-1.32]	1.06 [0.86-1.32]	1.08 [0.95-1.24]	660	511	333	222	557
S20	1.18 [1.11-1.25]	1.22 [1.15-1.31]	1.25 [1.17-1.34]	0.90 [0.77-1.05]	1.12 [1.05-1.19]	1.12 [1.05-1.19]	1.11 [1.03-1.18]	1.13 [1.05-1.21]	0.89 [0.76-1.04]	1.08 [1.02-1.16]	2,809	2,536	2,233	409	2,554
S21	1.11 [0.97-1.26]	1.15 [1.00-1.33]	1.45 [1.19-1.77]	0.85 [0.70-1.04]	1.05 [0.91-1.22]	1.06 [0.93-1.21]	1.05 [0.90-1.22]	1.32 [1.08-1.63]	0.83 [0.67-1.02]	1.06 [0.91-1.23]	604	466	266	247	477
S22	1.21 [1.17-1.25]	1.21 [1.17-1.25]	1.24 [1.19-1.28]	1.05 [0.91-1.20]	1.15 [1.11-1.19]	1.15 [1.12-1.19]	1.11 [1.07-1.15]	1.13 [1.09-1.17]	1.05 [0.91-1.21]	1.12 [1.08-1.16]	9,018	8,827	8,049	540	7,993
S27	1.11 [1.05-1.18]	1.10 [1.04-1.18]	1.15 [1.07-1.23]	1.02 [0.89-1.16]	1.04 [0.97-1.11]	1.08 [1.02-1.15]	1.04 [0.98-1.11]	1.08 [1.00-1.16]	1.02 [0.89-1.18]	1.04 [0.97-1.11]	2,716	2,484	1,917	559	2,223
S29	1.38 [1.23-1.55]	1.44 [1.28-1.62]	1.48 [1.30-1.68]		1.32 [1.17-1.48]	1.27 [1.13-1.43]	1.28 [1.13-1.44]	1.30 [1.14-1.48]		1.24 [1.10-1.41]	809	759	661	NA	723
S30	1.18 [1.13-1.23]	1.22 [1.16-1.28]	1.29 [1.22-1.36]	0.97 [0.89-1.05]	1.10 [1.05-1.16]	1.12 [1.07-1.17]	1.10 [1.04-1.15]	1.17 [1.11-1.24]	0.95 [0.87-1.03]	1.07 [1.02-1.12]	5,394	4,265	3,620	1,415	4,816
S31	1.11 [1.03-1.19]	1.13 [1.02-1.25]	1.36 [1.19-1.56]	1.03 [0.93-1.15]	1.05 [0.97-1.15]	1.07 [0.99-1.16]	1.02 [0.93-1.13]	1.21 [1.05-1.39]	1.03 [0.93-1.14]	1.05 [0.96-1.14]	1,721	989	558	960	1,380
S32	1.13 [1.09-1.16]	1.14 [1.10-1.17]	1.16 [1.12-1.20]	0.95 [0.84-1.08]	1.08 [1.04-1.12]	1.09 [1.05-1.13]	1.07 [1.04-1.11]	1.10 [1.06-1.14]	0.95 [0.83-1.08]	1.06 [1.02-1.09]	9,580	9,297	8,663	604	8,496
S33	1.08 [0.81-1.45]	1.00 [0.75-1.35]			1.00 [0.74-1.36]	1.02 [0.76-1.37]	0.92 [0.68-1.25]			0.92 [0.68-1.26]	120	108	NA	NA	103
S35	1.08 [0.82-1.43]	1.17 [0.87-1.56]				1.09 [0.82-1.45]	1.17 [0.87-1.59]				124	113	NA	NA	NA
S36	1.11 [1.01-1.22]	1.12 [1.01-1.24]	1.17 [1.03-1.33]	0.99 [0.85-1.15]	1.05 [0.95-1.16]	1.09 [1.00-1.20]	1.06 [0.96-1.18]	1.13 [0.99-1.28]	1.00 [0.86-1.16]	1.05 [0.95-1.16]	1,192	927	589	453	953
S37	1.00 [0.90-1.12]	1.05 [0.93-1.18]	1.02 [0.89-1.17]	0.99 [0.80-1.22]	0.96 [0.85-1.08]	0.98 [0.87-1.09]	1.01 [0.89-1.14]	0.98 [0.85-1.13]	1.00 [0.80-1.24]	0.95 [0.84-1.08]	813	690	509	219	671
S39	1.17 [1.09-1.25]	1.18 [1.09-1.27]	1.22 [1.12-1.34]	1.18 [1.04-1.33]	1.12 [1.04-1.20]	1.09 [1.02-1.17]	1.06 [0.98-1.15]	1.10 [1.00-1.21]	1.13 [0.99-1.28]	1.07 [0.99-1.15]	2,149	1,654	1,258	681	1,862
S40	1.14 [1.08-1.20]	1.17 [1.10-1.23]	1.21 [1.14-1.28]	0.92 [0.81-1.04]	1.10 [1.04-1.16]	1.09 [1.03-1.15]	1.07 [1.01-1.13]	1.11 [1.04-1.18]	0.90 [0.79-1.02]	1.07 [1.01-1.13]	4,026	3,550	3,241	625	3,692
S41	1.22 [1.13-1.32]	1.26 [1.16-1.38]	1.34 [1.21-1.48]	0.98 [0.85-1.14]	1.14 [1.05-1.24]	1.16 [1.07-1.26]	1.14 [1.04-1.25]	1.21 [1.09-1.34]	0.98 [0.84-1.14]	1.10 [1.01-1.20]	1,733	1,450	1,152	451	1,504
S42	1.11 [1.08-1.14]	1.14 [1.10-1.17]	1.14 [1.10-1.18]	1.05 [1.00-1.10]	1.08 [1.05-1.11]	1.08 [1.06-1.11]	1.08 [1.04-1.11]	1.08 [1.05-1.12]	1.04 [0.99-1.09]	1.07 [1.04-1.10]	14,330	10,210	9,071	4,662	12,069
S43	1.10 [1.02-1.18]	1.11 [1.03-1.19]	1.09 [1.01-1.19]	1.02 [0.82-1.26]	1.06 [0.98-1.14]	1.07 [0.99-1.15]	1.05 [0.98-1.13]	1.05 [0.97-1.14]	1.01 [0.81-1.26]	1.05 [0.97-1.13]	1,936	1,858	1,556	216	1,623
S44	1.24 [0.93-1.66]					1.21 [0.90-1.63]					120	NA	NA	NA	NA
S46	1.10 [0.99-1.23]	1.12 [1.00-1.25]	1.07 [0.95-1.22]		1.06 [0.95-1.20]	1.05 [0.94-1.18]	1.04 [0.92-1.16]	0.97 [0.85-1.10]		1.02 [0.90-1.15]	789	762	618	NA	686
S49	1.20 [1.09-1.31]	1.28 [1.16-1.40]	1.27 [1.14-1.40]	1.17 [0.89-1.53]	1.18 [1.07-1.30]	1.12 [1.02-1.23]	1.15 [1.04-1.27]	1.15 [1.04-1.28]	1.14 [0.86-1.51]	1.14 [1.03-1.26]	1,232	1,141	1,037	143	1,120
S50	1.16 [1.10-1.22]	1.19 [1.13-1.26]	1.23 [1.16-1.30]	0.93 [0.84-1.03]	1.09 [1.03-1.14]	1.11 [1.05-1.16]	1.10 [1.04-1.16]	1.13 [1.07-1.20]	0.90 [0.81-1.00]	1.06 [1.01-1.12]	4,521	3,790	3,397	947	4,090
S51	1.15 [1.11-1.20]	1.21 [1.16-1.26]	1.25 [1.19-1.31]	0.98 [0.90-1.07]	1.13 [1.09-1.18]	1.09 [1.04-1.14]	1.09 [1.04-1.14]	1.13 [1.07-1.19]	0.97 [0.89-1.06]	1.10 [1.05-1.14]	6,586	5,723	4,777	1,332	5,836
S52	1.06 [1.04-1.08]	1.10 [1.07-1.13]	1.11 [1.07-1.14]	1.01 [0.98-1.04]	1.02 [1.00-1.05]	1.04 [1.02-1.06]	1.06 [1.03-1.09]	1.07 [1.03-1.10]	1.00 [0.97-1.03]	1.01 [0.99-1.04]	23,610	12,452	10,625	11,920	18,431
S53	0.98 [0.87-1.11]	1.02 [0.86-1.21]	1.07 [0.87-1.31]	0.96 [0.82-1.13]	1.03 [0.90-1.18]	0.95 [0.84-1.08]	0.95 [0.79-1.13]	0.98 [0.80-1.21]	0.96 [0.81-1.13]	1.01 [0.88-1.16]	648	310	218	376	519
S54	1.13 [0.94-1.35]	1.23 [1.00-1.51]	1.35 [1.00-1.83]	1.00 [0.75-1.33]	1.20 [0.98-1.47]	1.09 [0.90-1.32]	1.09 [0.88-1.36]	1.28 [0.94-1.75]	1.00 [0.75-1.34]	1.15 [0.93-1.43]	290	222	106	126	238
S55	1.34 [1.02-1.75]	1.47 [1.11-1.95]			1.35 [0.99-1.84]	1.33 [1.01-1.76]	1.37 [1.03-1.84]			1.43 [1.04-1.97]	139	121	NA	NA	105
S56	1.00 [0.90-1.11]	1.09 [0.97-1.22]	1.09 [0.91-1.29]	0.97 [0.83-1.14]	0.92 [0.82-1.04]	0.98 [0.88-1.09]	1.04 [0.92-1.16]	1.05 [0.87-1.25]	0.96 [0.82-1.14]	0.93 [0.82-1.05]	883	718	286	395	649
S59	1.24 [1.08-1.43]	1.31 [1.12-1.53]	1.39 [1.18-1.64]	1.02 [0.77-1.35]	1.15 [0.99-1.33]	1.17 [1.01-1.35]	1.19 [1.02-1.40]	1.28 [1.08-1.51]	0.95 [0.71-1.27]	1.11 [0.95-1.29]	547	443	399	126	485
S60	1.14 [1.08-1.20]	1.17 [1.10-1.25]	1.20 [1.12-1.28]	1.00 [0.91-1.11]	1.07 [1.01-1.13]	1.10 [1.04-1.16]	1.09 [1.02-1.15]	1.11 [1.04-1.19]	0.99 [0.89-1.10]	1.05 [0.99-1.11]	3,606	2,885	2,342	993	3,200
S61	1.05 [1.02-1.08]	1.09 [1.06-1.13]	1.14 [1.09-1.18]	0.96 [0.92-1.00]	0.99 [0.97-1.02]	1.02 [0.99-1.04]	1.03 [1.00-1.06]	1.06 [1.02-1.11]	0.96 [0.92-1.00]	0.98 [0.95-1.01]	14,534	10,128	6,327	6,292	11,519
S62	1.05 [1.02-1.08]	1.08 [1.04-1.12]	1.09 [1.04-1.15]	1.01 [0.97-1.06]	0.99 [0.95-1.02]	1.02 [0.98-1.05]	1.02 [0.98-1.06]	1.03 [0.98-1.08]	1.00 [0.95-1.04]	0.98 [0.95-1.02]	10,253	7,126	4,076	4,846	7,794
S63	1.19 [1.11-1.29]	1.20 [1.10-1.31]	1.21 [1.09-1.34]	1.14 [0.99-1.31]	1.13 [1.03-1.23]	1.14 [1.06-1.24]	1.11 [1.02-1.21]	1.14 [1.02-1.27]	1.10 [0.95-1.27]	1.09 [1.00-1.19]	1,608	1,353	847	515	1,263
S64	1.00 [0.93-1.07]	1.01 [0.94-1.09]	1.01 [0.91-1.14]	0.94 [0.84-1.05]	0.90 [0.83-0.98]	0.98 [0.91-1.06]	0.96 [0.89-1.04]	0.98 [0.87-1.10]	0.94 [0.84-1.06]	0.90 [0.83-0.98]	1,854	1,453	665	775	1,331
S65	1.02 [0.89-1.17]	1.03 [0.88-1.19]	0.99 [0.80-1.22]	1.03 [0.83-1.27]	0.96 [0.83-1.13]	1.03 [0.90-1.18]	0.99 [0.85-1.15]	0.96 [0.78-1.19]	1.04 [0.83-1.30]	0.98 [0.84-1.15]	507	397	196	219	379
S66	1.03 [0.98-1.09]	1.04 [0.99-1.10]	1.00 [0.92-1.09]	0.99 [0.91-1.07]	0.95 [0.89-1.00]	1.01 [0.95-1.06]	1.00 [0.94-1.05]	0.97 [0.89-1.05]	0.98 [0.90-1.07]	0.95 [0.89-1.01]	3,464	2,821	1,209	1,443	2,435
S67	0.87 [0.76-1.00]	0.78 [0.63-0.97]		0.92 [0.79-1.09]	0.87 [0.75-1.02]	0.86 [0.75-0.98]	0.79 [0.63-0.98]		0.92 [0.78-1.09]	0.88 [0.75-1.03]	501	180	NA	358	396
S68	0.98 [0.90-1.07]	0.97 [0.86-1.09]	0.97 [0.85-1.12]	0.99 [0.88-1.12]	0.92 [0.83-1.02]	0.98 [0.90-1.08]	0.97 [0.86-1.09]	0.99 [0.86-1.14]	0.98 [0.87-1.11]	0.92 [0.84-1.02]	1,198	650	432	652	899
S69	1.21 [1.11-1.32]	1.26 [1.14-1.39]	1.29 [1.15-1.44]	1.02 [0.88-1.18]	1.17 [1.07-1.28]	1.15 [1.05-1.25]	1.13 [1.02-1.25]	1.15 [1.02-1.28]	1.01 [0.87-1.17]	1.13 [1.03-1.24]	1,477	1,110	848	460	1,287
S70	1.16 [1.10-1.22]	1.16 [1.10-1.23]	1.19 [1.13-1.26]	0.94 [0.82-1.07]	1.08 [1.02-1.14]	1.11 [1.05-1.17]	1.07 [1.01-1.13]	1.11 [1.05-1.18]	0.94 [0.82-1.07]	1.06 [1.00-1.12]	3,968	3,487	3,264	576	3,637
S71	1.07 [0.97-1.18]	1.14 [1.01-1.29]	1.30 [1.09-1.55]	0.99 [0.86-1.13]	1.03 [0.92-1.15]	1.02 [0.92-1.13]	1.02 [0.89-1.16]	1.20 [1.00-1.44]	0.95 [0.82-1.09]	1.01 [0.90-1.13]	1,033	612	320	578	818
S72	1.10 [1.08-1.12]	1.11 [1.09-1.14]	1.12 [1.10-1.14]	0.98 [0.90-1.07]	1.08 [1.06-1.11]	1.09 [1.07-1.11]	1.08 [1.06-1.11]	1.08 [1.06-1.11]	0.97 [0.89-1.06]	1.09 [1.06-1.11]	25,942	24,948	24,537	1,190	22,614
S73	1.09 [0.93-1.28]	1.09 [0.91-1.31]	1.17 [0.96-1.42]		1.05 [0.88-1.24]	1.04 [0.88-1.22]	1.02 [0.85-1.23]	1.07 [0.87-1.31]		1.02 [0.86-1.22]	369	293	255	NA	339
S76	1.14 [1.02-1.27]	1.16 [1.03-1.30]	1.14 [1.01-1.30]	1.09 [0.84-1.40]	1.09 [0.96-1.23]	1.07 [0.96-1.20]	1.08 [0.96-1.22]	1.07 [0.94-1.22]	1.05 [0.80-1.36]	1.05 [0.92-1.18]	811	711	581	167	656
S79	1.25 [1.16-1.34]	1.23 [1.14-1.32]	1.25 [1.15-1.34]	1.17 [0.92-1.49]	1.17 [1.09-1.27]	1.20 [1.11-1.29]	1.14 [1.06-1.23]	1.16 [1.07-1.25]	1.21 [0.94-1.55]	1.15 [1.07-1.24]	2,077	1,935	1,848	174	1,915
S80	1.14 [1.10-1.18]	1.18 [1.13-1.22]	1.19 [1.14-1.24]	0.93 [0.85-1.02]	1.09 [1.04-1.13]	1.08 [1.04-1.12]	1.07 [1.03-1.12]	1.07 [1.03-1.12]	0.91 [0.83-0.99]	1.04 [1.00-1.08]	7,694	6,760	6,187	1,180	6,986
S81	1.20 [1.15-1.26]	1.24 [1.18-1.29]	1.29 [1.23-1.36]	1.06 [0.97-1.17]	1.15 [1.10-1.20]	1.12 [1.07-1.17]	1.11 [1.06-1.16]	1.16 [1.10-1.22]	1.04 [0.95-1.14]	1.08 [1.03-1.13]	6,108	5,213	4,640	1,211	5,362
S82	1.09 [1.06-1.11]	1.10 [1.07-1.13]	1.10 [1.07-1.14]	1.04 [0.99-1.08]	1.04 [1.02-1.07]	1.05 [1.03-1.08]	1.04 [1.01-1.06]	1.04 [1.01-1.07]	1.02 [0.97-1.07]	1.03 [1.00-1.06]	16,239	12,559	9,534	4,983	12,630
S83	1.15 [1.08-1.23]	1.17 [1.08-1.25]	1.18 [1.06-1.30]	1.11 [1.00-1.24]	1.06 [0.98-1.14]	1.08 [1.01-1.16]	1.07 [1.00-1.16]	1.07 [0.97-1.19]	1.07 [0.96-1.19]	1.02 [0.94-1.10]	2,258	1,762	925	922	1,721
S86	1.14 [1.04-1.25]	1.18 [1.07-1.30]	1.20 [1.07-1.34]	1.04 [0.82-1.31]	1.10 [0.99-1.22]	1.08 [0.98-1.19]	1.10 [1.00-1.22]	1.11 [0.98-1.24]	1.01 [0.79-1.28]	1.04 [0.93-1.16]	1,067	972	693	193	806
S89	1.23 [1.14-1.33]	1.24 [1.14-1.34]	1.27 [1.16-1.38]	1.05 [0.87-1.26]	1.12 [1.04-1.22]	1.15 [1.07-1.25]	1.12 [1.03-1.22]	1.15 [1.05-1.26]	1.03 [0.85-1.25]	1.08 [0.99-1.17]	1,684	1,472	1,332	274	1,509
S90	1.11 [1.04-1.20]	1.13 [1.04-1.22]	1.11 [1.02-1.21]	1.02 [0.88-1.17]	1.04 [0.96-1.12]	1.06 [0.98-1.14]	1.02 [0.94-1.10]	1.01 [0.92-1.10]	1.01 [0.88-1.17]	1.01 [0.93-1.09]	1,979	1,574	1,345	496	1,751
S91	1.12 [1.05-1.19]	1.23 [1.13-1.34]	1.30 [1.17-1.43]	0.99 [0.90-1.09]	1.08 [1.01-1.17]	1.07 [1.00-1.15]	1.10 [1.01-1.20]	1.16 [1.05-1.29]	0.99 [0.89-1.09]	1.06 [0.98-1.14]	2,322	1,449	1,066	1,076	1,955
S92	1.13 [1.07-1.19]	1.15 [1.09-1.22]	1.20 [1.12-1.28]	0.99 [0.88-1.10]	1.10 [1.04-1.17]	1.08 [1.02-1.14]	1.05 [0.99-1.12]	1.09 [1.02-1.17]	0.97 [0.86-1.08]	1.08 [1.01-1.14]	3,393	2,905	2,176	808	2,775
S93	1.19 [1.09-1.31]	1.13 [1.02-1.25]	1.21 [1.07-1.36]	1.20 [1.00-1.45]	1.11 [1.00-1.23]	1.12 [1.01-1.23]	1.01 [0.91-1.12]	1.08 [0.96-1.22]	1.16 [0.96-1.41]	1.05 [0.95-1.17]	1,122	945	687	299	937
S96	1.14 [0.95-1.38]	1.11 [0.89-1.38]	1.19 [0.89-1.59]	1.22 [0.90-1.65]	1.16 [0.94-1.43]	1.06 [0.87-1.29]	0.95 [0.75-1.20]	1.00 [0.73-1.36]	1.21 [0.88-1.65]	1.06 [0.85-1.32]	264	185	106	114	218
S99	1.15 [1.03-1.28]	1.19 [1.05-1.34]	1.16 [1.01-1.32]	1.17 [0.94-1.45]	1.08 [0.97-1.21]	1.06 [0.94-1.18]	1.02 [0.90-1.16]	1.02 [0.89-1.17]	1.14 [0.91-1.42]	1.03 [0.92-1.16]	852	701	558	220	754
T00	1.12 [1.04-1.21]	1.15 [1.06-1.26]	1.19 [1.08-1.31]	0.98 [0.84-1.13]	1.08 [0.99-1.17]	1.07 [0.99-1.16]	1.05 [0.96-1.15]	1.09 [0.99-1.20]	0.98 [0.84-1.14]	1.04 [0.96-1.13]	1,813	1,458	1,266	446	1,632
T01	1.12 [1.00-1.25]	1.14 [1.01-1.29]	1.18 [1.02-1.36]	1.07 [0.87-1.30]	1.09 [0.97-1.22]	1.05 [0.94-1.18]	0.99 [0.87-1.12]	1.05 [0.90-1.21]	1.05 [0.86-1.29]	1.05 [0.93-1.18]	889	724	543	271	799
T02	1.17 [1.03-1.33]	1.18 [1.02-1.36]	1.31 [1.12-1.54]	0.97 [0.76-1.24]	1.05 [0.91-1.21]	1.15 [1.01-1.32]	1.13 [0.98-1.31]	1.26 [1.07-1.48]	0.94 [0.73-1.20]	1.05 [0.90-1.21]	593	466	389	164	471
T09	1.22 [1.02-1.47]	1.20 [0.99-1.47]	1.22 [0.97-1.52]		1.11 [0.92-1.35]	1.14 [0.95-1.38]	1.11 [0.90-1.36]	1.10 [0.87-1.38]		1.09 [0.89-1.33]	303	247	203	NA	257
T11	1.19 [1.08-1.31]	1.22 [1.10-1.35]	1.28 [1.14-1.43]	0.95 [0.78-1.16]	1.15 [1.05-1.27]	1.12 [1.01-1.23]	1.09 [0.98-1.21]	1.15 [1.03-1.29]	0.93 [0.76-1.15]	1.11 [1.00-1.23]	1,227	1,043	910	253	1,123
T13	1.17 [1.04-1.31]	1.24 [1.09-1.40]	1.26 [1.10-1.44]	0.83 [0.63-1.10]	1.11 [0.98-1.26]	1.10 [0.97-1.24]	1.12 [0.98-1.27]	1.13 [0.98-1.30]	0.83 [0.62-1.10]	1.06 [0.94-1.21]	755	659	595	121	675
T14	1.16 [1.03-1.29]	1.19 [1.05-1.35]	1.23 [1.07-1.41]	0.89 [0.71-1.13]	1.02 [0.90-1.16]	1.09 [0.97-1.22]	1.07 [0.94-1.21]	1.10 [0.95-1.26]	0.86 [0.68-1.09]	0.98 [0.87-1.12]	756	614	504	169	604
T15	1.05 [0.83-1.32]			1.00 [0.74-1.35]	0.95 [0.74-1.23]	1.03 [0.82-1.30]			0.98 [0.72-1.32]	0.95 [0.74-1.23]	180	NA	NA	106	149
T16	1.05 [0.98-1.12]	1.14 [0.89-1.47]	1.34 [0.98-1.83]	1.05 [0.97-1.12]	1.02 [0.94-1.10]	1.03 [0.96-1.11]	1.01 [0.78-1.31]	1.16 [0.83-1.60]	1.03 [0.96-1.11]	1.00 [0.93-1.08]	2,000	153	102	1,875	1,686
T17	1.14 [1.07-1.20]	1.36 [1.25-1.48]	1.30 [1.19-1.43]	0.99 [0.91-1.07]	1.07 [1.00-1.14]	1.08 [1.02-1.15]	1.22 [1.12-1.33]	1.18 [1.08-1.30]	0.97 [0.89-1.05]	1.03 [0.97-1.10]	2,952	1,523	1,296	1,516	2,532
T18	1.16 [1.09-1.24]	1.31 [1.21-1.42]	1.26 [1.15-1.39]	1.01 [0.91-1.11]	1.07 [0.99-1.14]	1.09 [1.02-1.16]	1.18 [1.08-1.28]	1.13 [1.03-1.25]	0.98 [0.89-1.08]	1.02 [0.95-1.10]	2,326	1,419	1,052	1,062	1,901
T19	1.19 [0.96-1.46]	1.29 [1.01-1.64]			1.17 [0.93-1.47]	1.11 [0.89-1.37]	1.18 [0.92-1.51]			1.15 [0.91-1.45]	221	163	NA	NA	186
T20	0.99 [0.89-1.09]	1.11 [0.96-1.29]	1.28 [1.06-1.54]	0.90 [0.80-1.02]	0.99 [0.88-1.10]	0.98 [0.88-1.08]	1.06 [0.91-1.23]	1.20 [0.99-1.46]	0.90 [0.79-1.02]	0.99 [0.89-1.11]	918	406	267	575	787
T21	1.00 [0.92-1.08]	1.14 [1.00-1.29]	1.20 [1.04-1.39]	0.91 [0.82-1.01]	0.99 [0.91-1.09]	0.99 [0.91-1.08]	1.07 [0.94-1.22]	1.11 [0.96-1.29]	0.91 [0.81-1.01]	1.00 [0.91-1.09]	1,351	614	476	791	1,177
T22	1.01 [0.92-1.11]	1.09 [0.95-1.24]	1.13 [0.96-1.34]	0.94 [0.84-1.06]	0.94 [0.85-1.04]	1.00 [0.91-1.09]	1.01 [0.88-1.16]	1.06 [0.89-1.26]	0.94 [0.84-1.06]	0.95 [0.85-1.05]	1,111	507	334	678	943
T23	1.01 [0.93-1.11]	1.03 [0.90-1.18]	1.09 [0.92-1.31]	0.97 [0.87-1.09]	0.97 [0.88-1.07]	1.00 [0.91-1.09]	0.98 [0.85-1.13]	1.02 [0.85-1.22]	0.95 [0.85-1.07]	0.97 [0.88-1.08]	1,140	479	291	746	952
T24	0.92 [0.83-1.02]	0.98 [0.85-1.12]	0.97 [0.82-1.13]	0.90 [0.78-1.04]	0.89 [0.79-0.99]	0.92 [0.83-1.02]	0.94 [0.82-1.08]	0.93 [0.79-1.10]	0.91 [0.79-1.05]	0.90 [0.80-1.00]	916	513	370	469	763
T25	0.88 [0.77-1.02]	0.98 [0.79-1.21]	0.92 [0.71-1.19]	0.92 [0.77-1.10]	0.89 [0.76-1.03]	0.85 [0.74-0.98]	0.91 [0.73-1.13]	0.81 [0.61-1.06]	0.90 [0.75-1.08]	0.87 [0.75-1.02]	461	205	136	289	396
T29	0.93 [0.75-1.15]	1.26 [0.94-1.68]		0.81 [0.61-1.08]	0.94 [0.75-1.19]	0.90 [0.73-1.12]	1.20 [0.89-1.62]		0.80 [0.60-1.07]	0.94 [0.74-1.19]	202	114	NA	109	175
T31	0.97 [0.92-1.02]	1.05 [0.97-1.15]	1.10 [0.99-1.23]	0.92 [0.86-0.99]	0.94 [0.89-1.00]	0.95 [0.90-1.01]	1.00 [0.91-1.09]	1.03 [0.93-1.16]	0.92 [0.86-0.98]	0.94 [0.89-1.00]	3,030	1,224	799	2,007	2,568
T36	1.26 [1.13-1.41]	1.30 [1.13-1.49]	1.50 [1.22-1.85]	1.18 [1.01-1.38]	1.14 [1.00-1.29]	1.17 [1.04-1.32]	1.09 [0.94-1.25]	1.25 [1.01-1.56]	1.17 [1.00-1.37]	1.11 [0.98-1.26]	783	514	224	430	637
T37	1.30 [1.08-1.56]	1.40 [1.14-1.73]	1.55 [1.14-2.09]	1.14 [0.86-1.50]	1.21 [1.00-1.47]	1.21 [1.00-1.46]	1.20 [0.97-1.50]	1.28 [0.93-1.76]	1.17 [0.88-1.55]	1.20 [0.98-1.46]	306	227	111	135	264
T38	1.26 [1.15-1.37]	1.34 [1.21-1.48]	1.37 [1.21-1.54]	1.05 [0.89-1.22]	1.14 [1.04-1.25]	1.14 [1.04-1.25]	1.09 [0.98-1.21]	1.11 [0.98-1.26]	1.02 [0.87-1.20]	1.09 [0.99-1.20]	1,234	955	666	419	1,125
T39	1.10 [1.07-1.13]	1.13 [1.09-1.16]	1.26 [1.20-1.33]	1.02 [0.98-1.06]	1.02 [0.99-1.05]	1.05 [1.02-1.08]	0.99 [0.95-1.02]	1.08 [1.02-1.14]	1.01 [0.97-1.05]	1.01 [0.98-1.04]	13,727	8,586	3,525	7,704	10,684
T40	1.18 [1.13-1.22]	1.21 [1.16-1.26]	1.32 [1.24-1.40]	0.97 [0.90-1.04]	1.08 [1.04-1.13]	1.08 [1.03-1.12]	1.00 [0.95-1.04]	1.06 [0.99-1.13]	0.98 [0.91-1.05]	1.06 [1.01-1.10]	5,986	5,084	2,615	2,003	5,251
T41	1.18 [0.92-1.50]	1.17 [0.90-1.54]			1.14 [0.87-1.49]	1.06 [0.82-1.37]	1.06 [0.80-1.40]			1.11 [0.84-1.47]	163	126	NA	NA	134
T42	1.17 [1.12-1.23]	1.23 [1.17-1.29]	1.30 [1.21-1.39]	0.96 [0.87-1.05]	1.05 [1.00-1.10]	1.08 [1.02-1.13]	1.00 [0.95-1.06]	1.06 [0.99-1.14]	0.98 [0.88-1.08]	1.03 [0.98-1.08]	4,246	3,759	2,072	1,090	3,661
T43	1.16 [1.12-1.21]	1.22 [1.17-1.27]	1.32 [1.24-1.40]	0.97 [0.91-1.03]	1.06 [1.01-1.10]	1.09 [1.05-1.13]	1.02 [0.97-1.06]	1.08 [1.01-1.15]	0.98 [0.92-1.04]	1.04 [1.00-1.09]	6,905	5,667	2,343	2,734	5,711
T44	1.16 [1.07-1.26]	1.22 [1.11-1.33]	1.23 [1.09-1.40]	1.01 [0.89-1.15]	1.07 [0.98-1.16]	1.10 [1.01-1.19]	1.04 [0.94-1.14]	1.02 [0.90-1.17]	1.00 [0.88-1.15]	1.06 [0.97-1.16]	1,483	1,137	600	578	1,285
T45	1.26 [1.18-1.35]	1.31 [1.21-1.42]	1.35 [1.21-1.51]	1.18 [1.07-1.31]	1.15 [1.07-1.24]	1.15 [1.08-1.24]	1.10 [1.01-1.20]	1.14 [1.02-1.28]	1.14 [1.03-1.27]	1.11 [1.03-1.20]	2,123	1,430	819	1,026	1,822
T46	1.19 [1.10-1.30]	1.23 [1.12-1.36]	1.26 [1.13-1.39]	1.00 [0.83-1.21]	1.12 [1.02-1.23]	1.11 [1.02-1.22]	1.06 [0.96-1.17]	1.06 [0.96-1.18]	0.98 [0.81-1.19]	1.09 [0.99-1.19]	1,326	1,135	968	264	1,210

Supplementary Table 3: Hazard ratios and events from all cohorts

	Hazard ratio (99% confidence interval)														
	crude					adjusted					Events (in exposed)				
Outcome	any age	18+	40+	<18	hosp.	any age	18+	40+	<18	hosp.	any age	18+	40+	<18	hosp.
T47	1.15 [1.03-1.29]	1.26 [1.11-1.43]	1.26 [1.07-1.49]	0.95 [0.79-1.15]	1.08 [0.96-1.22]	1.04 [0.92-1.17]	1.00 [0.88-1.15]	0.99 [0.83-1.18]	0.94 [0.77-1.14]	1.04 [0.92-1.17]	777	579	350	291	698
T48	1.20 [1.04-1.40]	1.42 [1.14-1.77]	1.77 [1.30-2.40]	1.05 [0.87-1.26]	1.11 [0.95-1.30]	1.08 [0.93-1.26]	1.11 [0.87-1.41]	1.44 [1.04-1.99]	1.01 [0.83-1.22]	1.01 [0.85-1.19]	450	198	111	286	381
T49	1.02 [0.87-1.21]	1.30 [0.96-1.77]		0.95 [0.79-1.16]	0.99 [0.82-1.18]	0.98 [0.82-1.16]	1.08 [0.78-1.48]		0.92 [0.76-1.12]	0.97 [0.81-1.16]	333	103	NA	244	288
T50	1.18 [1.09-1.27]	1.23 [1.14-1.34]	1.26 [1.13-1.40]	0.96 [0.85-1.09]	1.05 [0.97-1.14]	1.08 [1.00-1.17]	1.02 [0.94-1.12]	1.03 [0.92-1.15]	0.95 [0.83-1.08]	1.02 [0.94-1.11]	1,779	1,390	840	589	1,496
T51	1.14 [1.10-1.19]	1.16 [1.11-1.21]	1.26 [1.18-1.35]	0.98 [0.91-1.05]	1.06 [1.01-1.11]	1.07 [1.03-1.12]	0.98 [0.94-1.02]	1.03 [0.96-1.11]	1.00 [0.93-1.08]	1.06 [1.01-1.11]	5,485	4,942	2,032	1,804	4,568
T52	0.87 [0.74-1.04]			0.83 [0.68-1.01]	0.87 [0.72-1.05]	0.85 [0.72-1.02]			0.83 [0.68-1.01]	0.86 [0.71-1.04]	292	NA	NA	228	249
T54	0.98 [0.82-1.16]	1.01 [0.79-1.29]		0.86 [0.70-1.07]	0.95 [0.79-1.15]	0.95 [0.80-1.14]	0.94 [0.73-1.20]		0.85 [0.68-1.06]	0.94 [0.78-1.14]	337	161	NA	215	270
T56	1.17 [0.91-1.50]	1.17 [0.90-1.54]	1.18 [0.88-1.59]		1.10 [0.85-1.43]	1.10 [0.85-1.42]	1.05 [0.80-1.39]	1.07 [0.79-1.45]		1.12 [0.86-1.45]	153	131	102	NA	138
T58	1.08 [0.81-1.43]					1.07 [0.80-1.43]					115	NA	NA	NA	NA
T59	1.05 [0.90-1.23]	1.08 [0.91-1.27]	1.13 [0.93-1.37]	1.04 [0.77-1.41]	1.04 [0.89-1.23]	1.02 [0.87-1.19]	0.97 [0.82-1.16]	1.03 [0.84-1.25]	1.05 [0.77-1.43]	1.03 [0.87-1.21]	405	330	256	102	360
T62	1.24 [0.98-1.56]			1.09 [0.82-1.46]	1.21 [0.93-1.56]	1.17 [0.92-1.48]			1.06 [0.79-1.43]	1.15 [0.88-1.50]	179	NA	NA	112	146
T63	1.10 [0.89-1.37]	0.99 [0.77-1.26]	0.95 [0.72-1.25]		1.00 [0.78-1.28]	1.02 [0.82-1.27]	0.88 [0.68-1.13]	0.88 [0.66-1.16]		0.95 [0.74-1.22]	200	146	111	NA	152
T65	1.05 [0.89-1.24]	1.14 [0.88-1.47]	1.29 [0.95-1.75]	0.99 [0.80-1.21]	1.01 [0.85-1.20]	1.00 [0.84-1.18]	0.96 [0.73-1.25]	1.10 [0.80-1.52]	0.97 [0.78-1.19]	0.99 [0.83-1.18]	338	142	103	215	300
T68	1.10 [1.02-1.19]	1.13 [1.05-1.22]	1.11 [1.03-1.20]		1.07 [0.99-1.16]	1.12 [1.04-1.21]	1.12 [1.04-1.21]	1.11 [1.02-1.20]		1.11 [1.03-1.21]	1,908	1,849	1,772	NA	1,768
T71	0.96 [0.76-1.22]				0.88 [0.68-1.14]	0.94 [0.74-1.21]				0.89 [0.68-1.16]	167	NA	NA	NA	140
T74	0.91 [0.76-1.08]	1.17 [0.90-1.52]		0.85 [0.68-1.06]	0.91 [0.75-1.09]	0.86 [0.72-1.03]	0.95 [0.72-1.25]		0.82 [0.66-1.03]	0.89 [0.73-1.07]	311	139	NA	195	276
T75	1.09 [0.89-1.34]	1.24 [0.95-1.63]		1.01 [0.76-1.32]	1.00 [0.80-1.26]	1.02 [0.83-1.26]	1.13 [0.85-1.49]		0.97 [0.73-1.28]	0.96 [0.76-1.22]	226	130	NA	130	173
T78	3.07 [2.96-3.18]	1.99 [1.89-2.09]	1.72 [1.61-1.83]	4.81 [4.57-5.06]	2.90 [2.78-3.01]	2.54 [2.45-2.64]	1.58 [1.49-1.66]	1.45 [1.36-1.55]	3.86 [3.66-4.07]	2.42 [2.32-2.52]	10,510	4,247	2,499	7,022	8,795
T79	1.09 [1.03-1.17]	1.11 [1.04-1.18]	1.12 [1.04-1.21]	1.05 [0.90-1.22]	1.04 [0.98-1.12]	1.07 [1.00-1.14]	1.04 [0.97-1.11]	1.07 [0.99-1.15]	1.03 [0.89-1.21]	1.04 [0.97-1.12]	2,569	2,323	1,932	438	2,157
T80	1.33 [1.23-1.45]	1.39 [1.27-1.53]	1.38 [1.25-1.52]	1.09 [0.90-1.33]	1.26 [1.15-1.38]	1.27 [1.16-1.39]	1.28 [1.17-1.41]	1.26 [1.14-1.40]	1.08 [0.89-1.32]	1.24 [1.13-1.36]	1,377	1,160	991	275	1,230
T81	1.29 [1.27-1.31]	1.30 [1.28-1.32]	1.29 [1.26-1.31]	1.22 [1.17-1.27]	1.20 [1.18-1.22]	1.22 [1.20-1.24]	1.19 [1.17-1.21]	1.18 [1.16-1.20]	1.17 [1.12-1.22]	1.16 [1.14-1.18]	40,521	36,170	29,984	6,499	34,896
T82	1.31 [1.27-1.35]	1.34 [1.30-1.39]	1.34 [1.30-1.39]	1.01 [0.91-1.11]	1.19 [1.16-1.23]	1.25 [1.21-1.29]	1.21 [1.17-1.25]	1.21 [1.17-1.25]	0.99 [0.89-1.09]	1.17 [1.13-1.21]	9,620	8,858	8,174	979	8,863
T83	1.14 [1.10-1.18]	1.15 [1.12-1.20]	1.14 [1.09-1.18]	1.02 [0.87-1.20]	1.08 [1.04-1.12]	1.11 [1.07-1.15]	1.10 [1.06-1.14]	1.08 [1.04-1.12]	0.96 [0.81-1.14]	1.07 [1.03-1.11]	8,504	8,359	7,523	379	7,942
T84	1.31 [1.28-1.35]	1.32 [1.28-1.36]	1.34 [1.30-1.38]	1.14 [1.04-1.25]	1.20 [1.17-1.24]	1.24 [1.20-1.27]	1.21 [1.18-1.25]	1.23 [1.19-1.27]	1.11 [1.01-1.22]	1.17 [1.13-1.20]	12,672	12,081	10,652	1,194	11,593
T85	1.30 [1.26-1.35]	1.32 [1.28-1.37]	1.31 [1.26-1.36]	1.17 [1.08-1.27]	1.19 [1.15-1.23]	1.23 [1.19-1.28]	1.21 [1.17-1.26]	1.20 [1.16-1.25]	1.12 [1.03-1.22]	1.15 [1.11-1.20]	8,581	7,413	6,359	1,493	7,828
T86	1.33 [1.22-1.46]	1.36 [1.24-1.49]	1.38 [1.24-1.53]	1.02 [0.82-1.28]	1.17 [1.07-1.28]	1.29 [1.18-1.41]	1.26 [1.15-1.39]	1.25 [1.12-1.40]	1.01 [0.81-1.27]	1.17 [1.06-1.29]	1,200	1,070	842	200	1,095
T87	1.35 [1.20-1.52]	1.30 [1.16-1.47]	1.28 [1.13-1.45]		1.21 [1.07-1.36]	1.25 [1.11-1.41]	1.14 [1.01-1.29]	1.08 [0.95-1.23]		1.17 [1.04-1.32]	716	681	622	NA	663
T88	1.47 [1.39-1.56]	1.49 [1.40-1.59]	1.46 [1.36-1.56]	1.40 [1.23-1.60]	1.36 [1.28-1.45]	1.33 [1.25-1.41]	1.29 [1.20-1.37]	1.28 [1.19-1.38]	1.29 [1.13-1.48]	1.27 [1.19-1.35]	2,944	2,490	1,972	623	2,573
T90	1.29 [1.21-1.38]	1.31 [1.22-1.41]	1.38 [1.26-1.52]	1.15 [1.02-1.29]	1.16 [1.08-1.25]	1.23 [1.15-1.31]	1.19 [1.11-1.29]	1.22 [1.11-1.35]	1.13 [1.00-1.27]	1.14 [1.06-1.23]	2,230	1,854	1,061	718	1,836
T91	1.30 [1.17-1.44]	1.24 [1.11-1.37]	1.28 [1.13-1.44]	1.07 [0.79-1.43]	1.14 [1.02-1.26]	1.19 [1.07-1.32]	1.08 [0.97-1.21]	1.13 [0.99-1.27]	1.08 [0.79-1.46]	1.08 [0.97-1.20]	924	867	662	113	820
T92	1.17 [1.09-1.26]	1.20 [1.11-1.30]	1.24 [1.12-1.36]	1.07 [0.91-1.26]	1.09 [1.00-1.18]	1.12 [1.04-1.21]	1.11 [1.02-1.21]	1.15 [1.04-1.27]	1.04 [0.88-1.23]	1.07 [0.98-1.16]	1,618	1,399	980	379	1,338
T93	1.22 [1.15-1.31]	1.25 [1.16-1.34]	1.28 [1.18-1.39]	1.10 [0.96-1.26]	1.14 [1.06-1.22]	1.15 [1.07-1.23]	1.14 [1.06-1.22]	1.16 [1.06-1.26]	1.05 [0.91-1.21]	1.09 [1.01-1.18]	2,204	1,911	1,283	541	1,790
T94	1.26 [1.01-1.57]	1.24 [0.98-1.56]	1.11 [0.85-1.44]		1.11 [0.87-1.41]	1.21 [0.96-1.52]	1.12 [0.88-1.42]	0.99 [0.76-1.29]		1.06 [0.83-1.35]	196	181	139	NA	168
T95	0.97 [0.79-1.19]	1.31 [0.99-1.72]		0.80 [0.60-1.07]	1.06 [0.85-1.31]	0.96 [0.78-1.18]	1.26 [0.95-1.68]		0.82 [0.61-1.11]	1.08 [0.86-1.34]	216	120	NA	105	200
T98	1.19 [1.01-1.41]	1.34 [1.13-1.60]	1.35 [1.12-1.63]		1.08 [0.91-1.29]	1.10 [0.92-1.30]	1.21 [1.01-1.44]	1.23 [1.01-1.50]		1.04 [0.87-1.24]	344	320	271	NA	309
Codes for special purposes (U00-U89)															
U07	1.27 [1.23-1.31]	1.25 [1.21-1.29]	1.28 [1.24-1.32]	1.14 [1.02-1.29]	1.21 [1.17-1.25]	1.18 [1.15-1.22]	1.13 [1.09-1.17]	1.15 [1.11-1.19]	1.09 [0.96-1.23]	1.15 [1.12-1.19]	11,865	11,558	10,299	834	11,149
U80	1.49 [1.42-1.56]	1.50 [1.43-1.57]	1.49 [1.41-1.57]	1.41 [1.20-1.65]	1.40 [1.33-1.47]	1.40 [1.33-1.47]	1.36 [1.29-1.43]	1.34 [1.27-1.41]	1.33 [1.12-1.57]	1.35 [1.29-1.42]	4,665	4,351	3,975	426	4,395
U81	1.19 [0.96-1.46]	1.18 [0.95-1.46]	1.11 [0.89-1.39]		1.16 [0.94-1.44]	1.14 [0.92-1.41]	1.08 [0.87-1.34]	1.04 [0.83-1.31]		1.14 [0.92-1.42]	227	221	203	NA	215
U82	1.40 [1.35-1.45]	1.39 [1.34-1.44]	1.38 [1.32-1.43]	1.30 [1.17-1.45]	1.32 [1.27-1.37]	1.32 [1.27-1.37]	1.25 [1.20-1.30]	1.24 [1.19-1.29]	1.26 [1.13-1.41]	1.28 [1.23-1.33]	8,962	8,351	7,484	984	8,556
U83	1.35 [1.29-1.41]	1.34 [1.28-1.40]	1.35 [1.29-1.42]	1.22 [1.06-1.41]	1.29 [1.23-1.35]	1.28 [1.22-1.34]	1.20 [1.14-1.26]	1.21 [1.15-1.28]	1.17 [1.01-1.35]	1.25 [1.20-1.31]	5,253	4,881	4,456	564	5,000
U84	1.40 [1.02-1.93]				1.20 [0.87-1.66]	1.35 [0.97-1.87]				1.19 [0.86-1.66]	107	NA	NA	NA	101
U88	1.29 [1.16-1.42]	1.28 [1.15-1.42]	1.30 [1.17-1.45]		1.16 [1.05-1.29]	1.22 [1.10-1.35]	1.16 [1.04-1.29]	1.19 [1.06-1.33]		1.12 [1.01-1.24]	1,024	969	908	NA	973
U89	1.34 [1.22-1.47]	1.34 [1.21-1.47]	1.35 [1.22-1.49]	1.33 [1.00-1.77]	1.29 [1.17-1.42]	1.26 [1.14-1.38]	1.20 [1.08-1.32]	1.21 [1.09-1.34]	1.28 [0.96-1.72]	1.25 [1.13-1.38]	1,213	1,118	1,031	127	1,151
External causes of morbidity (V00-Y99)															
V01	0.99 [0.76-1.29]				1.02 [0.76-1.36]	0.96 [0.73-1.26]				1.00 [0.74-1.33]	135	NA	NA	NA	112
V02	0.99 [0.74-1.32]					0.99 [0.74-1.33]					110	NA	NA	NA	NA
V03	1.02 [0.96-1.09]	1.10 [1.00-1.20]	1.18 [1.06-1.31]	0.93 [0.85-1.02]	0.98 [0.91-1.06]	1.00 [0.94-1.07]	1.05 [0.96-1.15]	1.14 [1.02-1.26]	0.93 [0.85-1.02]	0.97 [0.90-1.05]	2,231	1,164	862	1,202	1,719
V04	1.32 [1.03-1.70]	1.18 [0.87-1.61]			1.34 [1.00-1.78]	1.27 [0.98-1.64]	1.14 [0.83-1.55]			1.33 [0.99-1.78]	160	104	NA	NA	124
V09	1.21 [1.05-1.38]	1.29 [1.09-1.53]	1.30 [1.08-1.56]	1.12 [0.90-1.39]	1.19 [1.03-1.37]	1.13 [0.98-1.30]	1.13 [0.95-1.35]	1.13 [0.94-1.37]	1.08 [0.87-1.35]	1.13 [0.98-1.31]	559	353	310	223	495
V11	1.00 [0.76-1.31]				0.97 [0.71-1.31]	0.95 [0.71-1.26]				0.96 [0.71-1.32]	128	NA	NA	NA	106
V13	0.91 [0.80-1.03]	0.90 [0.77-1.05]	0.90 [0.74-1.11]	0.94 [0.78-1.13]	0.81 [0.70-0.95]	0.91 [0.80-1.03]	0.90 [0.77-1.06]	0.90 [0.73-1.11]	0.97 [0.80-1.17]	0.82 [0.70-0.96]	573	356	207	285	384
V17	0.98 [0.80-1.19]	0.84 [0.63-1.11]		0.97 [0.75-1.25]	0.87 [0.70-1.09]	0.97 [0.79-1.18]	0.79 [0.59-1.05]		0.96 [0.75-1.24]	0.88 [0.70-1.11]	245	108	NA	157	180
V18	1.02 [0.97-1.07]	1.02 [0.94-1.10]	1.05 [0.95-1.16]	1.03 [0.97-1.09]	0.99 [0.93-1.05]	1.00 [0.95-1.05]	1.00 [0.93-1.09]	1.04 [0.94-1.15]	1.00 [0.94-1.07]	0.98 [0.92-1.04]	3,972	1,477	895	2,787	2,917
V19	1.07 [0.95-1.22]	1.01 [0.84-1.23]	1.10 [0.86-1.41]	1.02 [0.87-1.19]	0.95 [0.82-1.10]	1.05 [0.92-1.19]	0.99 [0.81-1.21]	1.10 [0.85-1.42]	1.01 [0.87-1.19]	0.94 [0.81-1.09]	620	239	143	418	450
V23	0.96 [0.86-1.08]	0.97 [0.86-1.09]	0.95 [0.78-1.16]	0.93 [0.79-1.10]	0.89 [0.77-1.01]	0.96 [0.86-1.08]	0.94 [0.83-1.06]	0.91 [0.74-1.12]	0.95 [0.80-1.13]	0.91 [0.79-1.05]	719	596	211	355	490
V27	0.87 [0.69-1.10]	0.84 [0.65-1.09]		0.91 [0.67-1.23]	0.80 [0.61-1.06]	0.82 [0.64-1.04]	0.78 [0.59-1.03]		0.89 [0.65-1.22]	0.78 [0.59-1.04]	176	125	NA	110	125
V28	1.02 [0.92-1.12]	0.98 [0.88-1.09]	1.03 [0.87-1.23]	1.01 [0.88-1.15]	0.92 [0.82-1.03]	0.99 [0.90-1.09]	0.93 [0.82-1.04]	0.96 [0.81-1.15]	0.98 [0.85-1.12]	0.91 [0.81-1.03]	1,012	699	298	536	715
V29	1.05 [0.87-1.26]	1.06 [0.86-1.32]		0.91 [0.69-1.20]	1.08 [0.87-1.33]	1.01 [0.83-1.22]	1.00 [0.80-1.25]		0.91 [0.69-1.21]	1.06 [0.85-1.32]	264	191	NA	125	198
V43	1.14 [1.06-1.22]	1.14 [1.06-1.23]	1.16 [1.05-1.28]	1.04 [0.91-1.19]	1.07 [0.99-1.16]	1.09 [1.01-1.17]	1.05 [0.98-1.14]	1.06 [0.96-1.18]	1.02 [0.88-1.17]	1.04 [0.95-1.12]	1,868	1,641	960	537	1,439
V44	1.19 [0.95-1.50]	1.10 [0.87-1.41]			1.15 [0.88-1.49]	1.11 [0.87-1.40]	1.04 [0.81-1.33]			1.13 [0.86-1.48]	178	155	NA	NA	133
V47	1.16 [1.04-1.28]	1.24 [1.11-1.38]	1.26 [1.07-1.48]	1.04 [0.88-1.23]	1.18 [1.05-1.33]	1.11 [1.00-1.24]	1.16 [1.04-1.30]	1.15 [0.98-1.36]	1.02 [0.86-1.22]	1.16 [1.02-1.31]	892	791	376	351	679
V48	1.14 [1.03-1.25]	1.15 [1.05-1.28]	1.21 [1.07-1.36]	1.13 [0.94-1.36]	1.08 [0.97-1.20]	1.09 [0.99-1.21]	1.07 [0.97-1.19]	1.12 [0.99-1.26]	1.12 [0.92-1.36]	1.05 [0.94-1.17]	1,068	964	679	277	867
V49	1.11 [0.99-1.23]	1.12 [0.99-1.26]	1.21 [1.03-1.41]	1.06 [0.88-1.28]	1.06 [0.94-1.21]	1.08 [0.96-1.20]	1.03 [0.92-1.17]	1.12 [0.95-1.32]	1.06 [0.87-1.29]	1.06 [0.93-1.20]	797	656	376	267	608
V78	1.14 [0.98-1.34]	1.19 [1.01-1.40]	1.17 [0.99-1.38]		1.16 [0.98-1.37]	1.09 [0.93-1.29]	1.12 [0.95-1.32]	1.10 [0.92-1.30]		1.13 [0.96-1.34]	389	378	357	NA	360
V79	1.08 [0.83-1.42]	1.02 [0.76-1.36]	1.01 [0.74-1.38]		1.07 [0.80-1.44]	1.03 [0.78-1.35]	0.89 [0.66-1.21]	0.93 [0.68-1.28]		1.00 [0.74-1.36]	130	111	101	NA	117
V80	0.95 [0.86-1.04]	0.88 [0.79-0.99]	0.76 [0.64-0.90]	1.03 [0.90-1.17]	0.88 [0.79-0.98]	0.93 [0.85-1.02]	0.87 [0.77-0.98]	0.76 [0.64-0.91]	1.01 [0.88-1.15]	0.87 [0.78-0.98]	1,094	648	288	614	774
V82	1.42 [1.05-1.92]	1.52 [1.12-2.07]	1.37 [0.99-1.90]		1.39 [1.02-1.90]	1.42 [1.05-1.93]	1.47 [1.08-2.00]	1.33 [0.95-1.85]		1.41 [1.02-1.93]	120	114	104	NA	108
V86	1.08 [0.90-1.29]	1.04 [0.80-1.35]		1.14 [0.91-1.42]	1.05 [0.85-1.29]	1.02 [0.85-1.23]	0.96 [0.73-1.26]		1.10 [0.87-1.38]	1.03 [0.84-1.27]	300	128	NA	215	237
V89	1.20 [1.02-1.40]	1.28 [1.08-1.53]	1.39 [1.08-1.79]	0.90 [0.67-1.20]	1.15 [0.96-1.39]	1.12 [0.95-1.32]	1.19 [0.99-1.42]	1.23 [0.94-1.59]	0.85 [0.63-1.15]	1.10 [0.91-1.34]	370	315	145	111	262
W00	1.09 [1.01-1.18]	1.10 [1.02-1.19]	1.08 [0.99-1.18]	1.19 [0.94-1.50]	1.04 [0.96-1.14]	1.07 [0.99-1.16]	1.08 [0.99-1.17]	1.06 [0.97-1.16]	1.19 [0.93-1.51]	1.03 [0.95-1.13]	1,522	1,387	1,193	184	1,177
W01	1.14 [1.12-1.16]	1.16 [1.13-1.18]	1.16 [1.14-1.18]	1.03 [0.99-1.08]	1.10 [1.08-1.12]	1.10 [1.08-1.12]	1.09 [1.07-1.11]	1.10 [1.08-1.12]	1.02 [0.97-1.06]	1.08 [1.06-1.10]	34,003	29,400	27,753	5,207	29,222
W02	1.01 [0.94-1.09]	1.01 [0.89-1.15]	1.02 [0.84-1.24]	1.00 [0.92-1.09]	0.99 [0.91-1.07]	0.99 [0.92-1.07]	0.98 [0.85-1.12]	1.00 [0.82-1.22]	1.00 [0.92-1.08]	0.97 [0.89-1.06]	1,916	513	226	1,534	1,445
W03	1.05 [0.99-1.11]	1.06 [0.96-1.16]	1.03 [0.88-1.21]	1.02 [0.95-1.10]	0.98 [0.91-1.05]	1.01 [0.95-1.08]	0.99 [0.90-1.09]	0.97 [0.82-1.14]	1.00 [0.93-1.07]	0.95 [0.88-1.02]	2,654	1,038	363	1,996	1,897
W04	1.01 [0.87-1.18]			0.97 [0.82-1.15]	1.01 [0.86-1.20]	1.00 [0.86-1.17]			0.97 [0.81-1.15]	1.03 [0.88-1.22]	409	NA	NA	327	358
W05	1.24 [1.10-1.39]	1.26 [1.11-1.42]	1.26 [1.12-1.43]		1.15 [1.02-1.30]	1.19 [1.05-1.34]	1.15 [1.01-1.30]	1.14 [1.01-1.30]		1.14 [1.01-1.29]	722	693	670	NA	685
W06	1.17 [1.12-1.21]	1.19 [1.15-1.24]	1.19 [1.14-1.24]	1.03 [0.94-1.13]	1.12 [1.08-1.16]	1.13 [1.09-1.17]	1.11 [1.07-1.16]	1.11 [1.07-1.16]	1.02 [0.93-1.12]	1.10 [1.06-1.14]	8,242	7,160	7,055	1,123	7,666
W07	1.14 [1.09-1.19]	1.19 [1.13-1.25]	1.21 [1.15-1.28]	0.97 [0.88-1.06]	1.08 [1.03-1.13]	1.11 [1.06-1.16]	1.12 [1.06-1.18]	1.13 [1.07-1.20]	0.96 [0.87-1.06]	1.06 [1.01-1.12]	4,869	3,857	3,763	1,039	4,389
W08	1.09 [1.00-1.17]	1.24 [1.11-1.37]	1.22 [1.10-1.37]	0.89 [0.80-1.00]	1.04 [0.96-1.13]	1.06 [0.98-1.15]	1.14 [1.02-1.27]	1.13 [1.01-1.26]	0.89 [0.80-1.00]	1.03 [0.95-1.12]	1,652	949	893	723	1,500
W09	1.05 [1.01-1.10]	1.23 [0.97-1.57]		1.05 [1.01-1.10]	1.02 [0.97-1.07]	1.04 [0.99-1.08]	1.17 [0.91-1.50]		1.04 [0.99-1.09]	1.01 [0.96-1.06]	5,335	162	NA	5,222	4,350
W10	1.15 [1.12-1.18]	1.19 [1.16-1.23]	1.19 [1.16-1.23]	1.02 [0.95-1.08]	1.11 [1.08-1.14]	1.11 [1.08-1.14]	1.11 [1.08-1.15]	1.11 [1.08-1.15]	1.00 [0.94-1.07]	1.09 [1.06-1.12]	13,793	11,995	10,622	2,267	11,894
W11	1.06 [0.98-1.13]	1.06 [0.99-1.14]	1.02 [0.95-1.10]	1.05 [0.80-1.38]	0.99 [0.91-1.07]	1.02 [0.94-1.09]	1.02 [0.94-1.10]	0.98 [0.90-1.05]	1.06 [0.80-1.40]	0.97 [0.89-1.05]	1,786	1,714	1,537	133	1,366
W12	1.19 [0.90-1.57]	1.15 [0.86-1.54]				1.16 [0.88-1.55]	1.14 [0.85-1.54]				124	105	NA	NA	NA
W13	0.99 [0.92-1.07]	1.05 [0.94-1.17]	1.14 [0.98-1.31]	0.95 [0.87-1.05]	0.97 [0.89-1.06]	0.97 [0.90-1.05]	0.98 [0.88-1.10]	1.07 [0.92-1.24]	0.94 [0.85-1.04]	0.97 [0.89-1.06]	1,646	763	427	1,057	1,283
W14	0.99 [0.87-1.12]			0.95 [0.83-1.08]	1.00 [0.87-1.16]	0.99 [0.87-1.12]			0.94 [0.82-1.08]	1.01 [0.87-1.17]	625	NA	NA	562	482
W17	1.05 [0.99-1.11]	1.16 [1.07-1.25]	1.19 [1.09-1.31]	0.97 [0.91-1.04]	1.00 [0.94-1.06]	1.02 [0.97-1.08]	1.09 [1.00-1.18]	1.12 [1.02-1.23]	0.95 [0.88-1.02]	0.99 [0.93-1.05]	3,262	1,473	1,105	1,964	2,609
W18	1.19 [1.16-1.22]	1.21 [1.18-1.24]	1.22 [1.19-1.25]	1.06 [1.00-1.12]	1.14 [1.11-1.16]	1.15 [1.12-1.17]	1.13 [1.10-1.15]	1.13 [1.11-1.16]	1.05 [0.99-1.11]	1.12 [1.09-1.14]	23,221	20,500	19,473	3,144	20,939
W19	1.17 [1.16-1.19]	1.19 [1.17-1.21]	1.19 [1.17-1.20]	1.04 [1.00-1.09]	1.13 [1.12-1.15]	1.14 [1.12-1.16]	1.12 [1.10-1.14]	1.12 [1.10-1.14]	1.03 [0.98-1.08]	1.12 [1.10-1.14]	51,848	47,686	45,247	5,023	46,001
W20	1.03 [0.95-1.11]	1.08 [0.97-1.19]	1.13 [1.00-1.28]	0.99 [0.88-1.11]	0.99 [0.90-1.08]	0.99 [0.91-1.07]	1.01 [0.91-1.12]	1.04 [0.92-1.19]	0.96 [0.85-1.08]	0.97 [0.89-1.07]	1,439	883	576	699	1,132
W21	1.11 [1.03-1.21]	1.11 [0.97-1.27]	0.94 [0.73-1.22]	1.12 [1.02-1.23]	1.04 [0.95-1.15]	1.08 [0.99-1.18]	1.08 [0.94-1.24]	0.88 [0.68-1.15]	1.10 [1.00-1.21]	1.02 [0.92-1.13]	1,513	494	131	1,206	1,094
W22	1.12 [1.08-1.16]	1.17 [1.12-1.23]	1.20 [1.13-1.27]	1.05 [1.00-1.10]	1.07 [1.03-1.12]	1.08 [1.04-1.12]	1.08 [1.03-1.13]	1.09 [1.03-1.16]	1.03 [0.98-1.09]	1.05 [1.01-1.09]	7,690	4,615	3,190	3,846	6,511
W23	1.02 [0.98-1.07]	1.08 [1.01-1.16]	1.14 [1.04-1.25]	1.00 [0.95-1.05]	0.97 [0.92-1.02]	1.00 [0.96-1.05]	1.03 [0.96-1.11]	1.10 [1.01-1.20]	0.99 [0.94-1.05]	0.96 [0.92-1.01]	4,806	1,867	1,202	3,288	3,875
W25	1.02 [0.96-1.09]	1.08 [1.00-1.17]	1.16 [1.02-1.32]	0.95 [0.87-1.03]	0.98 [0.91-1.05]	1.00 [0.94-1.07]	1.02 [0.94-1.10]	1.06 [0.93-1.22]	0.94 [0.86-1.02]	0.97 [0.90-1.05]	2,334	1,509	529	1,307	1,703
W26	1.06 [0.99-1.13]	1.12 [1.04-1.20]	1.10 [0.98-1.23]	1.04 [0.93-1.15]	0.99 [0.92-1.07]	1.02 [0.95-1.09]	1.03 [0.96-1.12]	1.02 [0.91-1.14]	1.03 [0.93-1.14]	0.98 [0.91-1.06]	2,119	1,614	709	977	1,663
W27	0.99 [0.89-1.10]	1.03 [0.92-1.16]	1.08 [0.93-1.25]	0.89 [0.74-1.06]	0.97 [0.87-1.09]	0.95 [0.86-1.06]	0.98 [0.87-1.10]	1.01 [0.87-1.18]	0.89 [0.74-1.06]	0.96 [0.85-1.08]	881	690	391	300	681
W28	0.85 [0.63-1.13]					0.83 [0.62-1.11]					102	NA	NA	NA	NA
W29	0.99 [0.89-1.09]	0.98 [0.88-1.08]	0.99 [0.87-1.13]	0.98 [0.79-1.21]	0.90 [0.80-1.01]	0.98 [0.88-1.08]	0.95 [0.85-1.05]	0.96 [0.84-1.10]	0.98 [0.79-1.23]	0.91 [0.81-1.02]	893	814	516	215	651
W31	1.02 [0.92-1.13]	1.03 [0.92-1.14]	0.95 [0.82-1.09]	1.17 [0.96-1.42]	0.93 [0.82-1.05]	1.02 [0.92-1.13]	1.02 [0.91-1.14]	0.94 [0.82-1.08]	1.16 [0.95-1.43]	0.92 [0.81-1.04]	840	721	444	262	587
W34	1.06 [0.83-1.35]			1.04 [0.77-1.41]		1.03 [0.80-1.33]			1.04 [0.76-1.43]		153	NA	NA	104	NA
W44	1.10 [1.06-1.15]	1.30 [1.21-1.40]	1.30 [1.19-1.41]	1.02 [0.97-1.08]	1.05 [1.00-1.10]	1.06 [1.02-1.11]	1.18 [1.09-1.27]	1.18 [1.08-1.28]	1.01 [0.96-1.06]	1.02 [0.97-1.06]	5,499	1,986	1,443	3,746	4,587
W45	1.07 [0.99-1.15]	1.13 [1.03-1.25]	1.08 [0.95-1.23]	1.06 [0.96-1.18]	1.01 [0.93-1.10]	1.03 [0.96-1.12]	1.05 [0.95-1.16]	0.99 [0.87-1.13]	1.05 [0.94-1.16]	0.99 [0.90-1.07]	1,720	944	526	961	1,327
W46	1.08 [0.86-1.34]	1.17 [0.93-1.46]	1.32 [0.99-1.75]		0.86 [0.69-1.09]	0.99 [0.78-1.24]	0.95 [0.75-1.21]	1.05 [0.78-1.41]		0.85 [0.67-1.08]	191	179	116	NA	168
W49	1.19 [1.03-1.38]	1.29 [1.08-1.54]	1.32 [1.05-1.66]	1.01 [0.81-1.26]	1.20 [1.02-1.42]	1.18 [1.02-1.37]	1.22 [1.01-1.47]	1.25 [0.99-1.58]	1.01 [0.81-1.26]	1.18 [1.00-1.39]	450	287	180	197	361
W50	1.11 [1.05-1.18]	1.17 [1.07-1.27]	1.23 [1.03-1.45]	1.08 [1.00-1.16]	1.06 [0.99-1.14]	1.08 [1.01-1.15]	1.08 [0.99-1.18]	1.11 [0.93-1.33]	1.06 [0.98-1.14]	1.05 [0.98-1.13]	2,632	1,264	312	1,914	1,958
W51	1.06 [0.99-1.14]	1.11 [1.00-1.24]	1.10 [0.89-1.35]	1.04 [0.96-1.13]	1.05 [0.97-1.13]	1.03 [0.96-1.10]	1.05 [0.94-1.17]	1.06 [0.85-1.31]	1.01 [0.93-1.10]	1.02 [0.94-1.11]	1,993	764	216	1,541	1,526
W54	0.98 [0.92-1.05]	1.04 [0.97-1.13]	1.13 [1.03-1.24]	0.88 [0.80-0.97]	0.93 [0.86-0.99]	0.95 [0.89-1.01]	0.97 [0.90-1.05]	1.04 [0.95-1.15]	0.86 [0.78-0.95]	0.91 [0.85-0.98]	2,316	1,462	1,040	1,062	1,865
W55	0.98 [0.89-1.08]	1.00 [0.90-1.11]	0.97 [0.86-1.09]	0.99 [0.81-1.20]	0.92 [0.83-1.03]	0.92 [0.83-1.02]	0.91 [0.81-1.01]	0.88 [0.78-1.00]	0.98 [0.80-1.20]	0.88 [0.79-0.98]	968	800	601	255	781
W57	1.40 [1.29-1.52]	1.45 [1.32-1.59]	1.42 [1.27-1.58]	1.26 [1.09-1.45]	1.29 [1.19-1.41]	1.31 [1.21-1.43]	1.31 [1.19-1.44]	1.28 [1.14-1.43]	1.24 [1.08-1.44]	1.24 [1.13-1.36]	1,567	1,194	788	533	1,301
W60	1.35 [1.04-1.76]	1.47 [1.10-1.96]	1.46 [1.07-1.99]		1.29 [0.97-1.71]	1.27 [0.97-1.67]	1.37 [1.02-1.84]	1.38 [1.01-1.89]		1.24 [0.93-1.66]	144	121	102	NA	121
W64	1.04 [0.86-1.26]	1.10 [0.89-1.36]	1.13 [0.90-1.43]		1.11 [0.91-1.36]	1.03 [0.85-1.25]	1.02 [0.82-1.26]	1.06 [0.84-1.35]		1.11 [0.91-1.37]	276	220	181	NA	241
W78	1.10 [0.97-1.23]	1.11 [0.98-1.25]	1.10 [0.97-1.25]		1.05 [0.93-1.19]	1.07 [0.95-1.21]	1.06 [0.94-1.20]	1.05 [0.92-1.19]		1.06 [0.94-1.20]	744	724	692	NA	703
W79	1.27 [1.16-1.39]	1.26 [1.14-1.39]	1.19 [1.08-1.32]	1.96 [1.56-2.47]	1.22 [1.11-1.34]	1.21 [1.10-1.32]	1.17 [1.06-1.29]	1.13 [1.02-1.25]	1.68 [1.32-2.14]	1.18 [1.07-1.30]	1,299	1,114	994	227	1,180
W80	1.18 [1.02-1.37]	1.23 [1.05-1.44]	1.27 [1.08-1.50]		1.10 [0.95-1.29]	1.12 [0.96-1.30]	1.12 [0.95-1.31]	1.15 [0.98-1.36]		1.06 [0.91-1.24]	484	437	415	NA	446
W84	1.06 [0.90-1.24]	1.06 [0.91-1.24]	1.08 [0.92-1.27]		1.02 [0.87-1.20]	1.03 [0.88-1.21]	0.99 [0.85-1.17]	1.03 [0.87-1.21]		1.01 [0.86-1.19]	431	418	408	NA	406
X04	1.02 [0.78-1.33]					0.99 [0.75-1.29]					132	NA	NA	NA	NA
X08	1.04 [0.79-1.37]				0.98 [0.74-1.31]	1.04 [0.79-1.37]				0.96 [0.72-1.29]	128	NA	NA	NA	111
X09	1.01 [0.80-1.27]	1.02 [0.78-1.34]	1.11 [0.82-1.51]		0.96 [0.75-1.24]	0.98 [0.78-1.25]	0.98 [0.75-1.29]	1.05 [0.77-1.43]		0.94 [0.73-1.21]	177	131	101	NA	151
X10	1.00 [0.91-1.11]	1.19 [0.99-1.43]	1.19 [0.95-1.50]	0.98 [0.87-1.10]	0.99 [0.89-1.11]	0.99 [0.89-1.10]	1.09 [0.90-1.32]	1.10 [0.88-1.39]	0.98 [0.87-1.10]	0.99 [0.89-1.11]	921	278	193	691	814
X11	0.97 [0.81-1.17]			1.01 [0.81-1.26]	0.95 [0.77-1.17]	0.94 [0.78-1.14]			0.99 [0.79-1.24]	0.93 [0.75-1.15]	273	NA	NA	190	222
X12	0.94 [0.82-1.08]	1.01 [0.82-1.24]	1.09 [0.86-1.40]	0.87 [0.74-1.03]	0.92 [0.80-1.06]	0.92 [0.81-1.06]	0.93 [0.75-1.15]	0.99 [0.77-1.28]	0.87 [0.73-1.03]	0.91 [0.78-1.05]	529	218	162	330	445
X15	1.04 [0.89-1.22]			0.99 [0.83-1.17]	1.00 [0.85-1.17]	1.03 [0.88-1.21]			0.99 [0.83-1.17]	1.00 [0.85-1.18]	394	NA	NA	323	357
X16	0.99 [0.82-1.21]	1.14 [0.89-1.45]	1.04 [0.80-1.34]		0.95 [0.77-1.17]	0.96 [0.79-1.17]	1.05 [0.82-1.35]	0.98 [0.75-1.27]		0.95 [0.77-1.18]	255	173	146	NA	222
X19	1.12 [0.89-1.41]				1.02 [0.80-1.31]	1.07 [0.85-1.36]				0.99 [0.77-1.28]	178	NA	NA	NA	152
X23	1.17 [0.97-1.41]	1.19 [0.97-1.47]	1.13 [0.89-1.42]		1.24 [1.01-1.52]	1.09 [0.90-1.32]	1.11 [0.89-1.37]	1.04 [0.82-1.31]		1.18 [0.96-1.46]	280	217	169	NA	226
X31	1.05 [0.85-1.29]	1.00 [0.81-1.24]	1.00 [0.80-1.23]		0.97 [0.78-1.21]	1.06 [0.86-1.31]	0.99 [0.80-1.23]	0.99 [0.80-1.24]		1.01 [0.80-1.26]	244	233	221	NA	210
X40	1.07 [1.02-1.13]	1.16 [1.08-1.24]	1.26 [1.14-1.39]	1.00 [0.94-1.08]	1.02 [0.96-1.08]	1.03 [0.97-1.09]	1.01 [0.94-1.08]	1.07 [0.96-1.18]	1.00 [0.93-1.08]	1.00 [0.94-1.06]	3,316	1,892	979	1,855	2,782
X41	1.15 [1.08-1.24]	1.26 [1.17-1.37]	1.29 [1.16-1.43]	0.98 [0.88-1.10]	1.06 [0.99-1.14]	1.08 [1.01-1.16]	1.06 [0.97-1.15]	1.08 [0.96-1.20]	0.99 [0.88-1.11]	1.05 [0.97-1.13]	1,950	1,468	844	752	1,690
X42	1.22 [1.13-1.31]	1.28 [1.18-1.38]	1.38 [1.25-1.52]	0.98 [0.85-1.14]	1.13 [1.05-1.22]	1.11 [1.03-1.20]	1.04 [0.96-1.13]	1.11 [1.00-1.23]	0.98 [0.84-1.14]	1.10 [1.02-1.19]	1,798	1,604	1,050	440	1,636
X43	1.10 [0.94-1.29]	1.20 [0.98-1.46]	1.16 [0.93-1.45]	0.95 [0.75-1.22]	1.02 [0.86-1.21]	1.05 [0.90-1.24]	1.08 [0.88-1.32]	1.03 [0.82-1.29]	0.92 [0.71-1.18]	1.02 [0.86-1.20]	395	259	210	155	344
X44	1.17 [1.10-1.24]	1.29 [1.20-1.39]	1.30 [1.19-1.42]	1.03 [0.94-1.12]	1.09 [1.03-1.16]	1.09 [1.03-1.16]	1.10 [1.02-1.19]	1.11 [1.01-1.21]	1.01 [0.92-1.10]	1.06 [1.00-1.13]	2,841	1,751	1,372	1,261	2,516
X45	1.12 [1.00-1.26]	1.16 [1.03-1.31]	1.23 [1.02-1.50]	0.92 [0.76-1.11]	1.01 [0.89-1.14]	1.07 [0.95-1.21]	0.99 [0.87-1.13]	1.00 [0.81-1.22]	0.94 [0.78-1.14]	1.00 [0.88-1.14]	720	595	243	276	588
X46	0.91 [0.73-1.12]			0.83 [0.66-1.04]	0.91 [0.72-1.14]	0.88 [0.71-1.09]			0.82 [0.65-1.04]	0.90 [0.71-1.12]	200	NA	NA	158	174
X47	1.11 [0.92-1.34]	1.10 [0.89-1.37]	1.17 [0.91-1.51]		1.08 [0.88-1.33]	1.08 [0.89-1.31]	1.00 [0.80-1.25]	1.12 [0.86-1.45]		1.06 [0.86-1.31]	265	197	148	NA	228
X49	1.35 [1.24-1.47]	1.38 [1.20-1.58]	1.30 [1.09-1.55]	1.37 [1.23-1.52]	1.31 [1.19-1.44]	1.27 [1.16-1.38]	1.20 [1.04-1.38]	1.17 [0.98-1.40]	1.28 [1.14-1.43]	1.24 [1.12-1.36]	1,326	533	310	892	1,128
X50	1.11 [1.06-1.17]	1.15 [1.09-1.21]	1.15 [1.08-1.24]	1.05 [0.96-1.15]	1.05 [0.99-1.11]	1.05 [1.00-1.10]	1.05 [1.00-1.12]	1.06 [0.99-1.14]	1.01 [0.92-1.11]	1.01 [0.95-1.07]	3,812	3,084	1,998	1,224	3,066
X51	1.19 [0.98-1.44]	1.14 [0.94-1.39]	1.16 [0.93-1.45]		1.06 [0.86-1.32]	1.15 [0.95-1.40]	1.10 [0.90-1.34]	1.10 [0.88-1.38]		1.04 [0.84-1.30]	254	237	186	NA	197
X58	1.60 [1.47-1.75]	1.45 [1.31-1.61]	1.39 [1.23-1.56]	2.06 [1.78-2.38]	1.50 [1.36-1.64]	1.44 [1.32-1.58]	1.28 [1.15-1.43]	1.26 [1.11-1.42]	1.79 [1.54-2.08]	1.38 [1.26-1.52]	1,466	973	729	602	1,302
X59	1.19 [1.16-1.22]	1.24 [1.20-1.27]	1.26 [1.22-1.30]	1.08 [1.03-1.12]	1.14 [1.11-1.17]	1.14 [1.11-1.17]	1.13 [1.10-1.17]	1.16 [1.12-1.20]	1.05 [1.00-1.09]	1.11 [1.08-1.14]	16,982	12,513	10,137	5,445	14,181
X60	1.11 [1.08-1.15]	1.13 [1.09-1.17]	1.29 [1.21-1.36]	1.02 [0.98-1.07]	1.03 [0.99-1.06]	1.06 [1.02-1.09]	0.98 [0.95-1.02]	1.09 [1.03-1.16]	1.01 [0.97-1.06]	1.01 [0.98-1.05]	10,841	7,074	2,711	5,994	8,352
X61	1.16 [1.12-1.21]	1.21 [1.16-1.25]	1.32 [1.25-1.40]	0.97 [0.92-1.04]	1.05 [1.01-1.09]	1.08 [1.04-1.13]	1.00 [0.96-1.05]	1.08 [1.02-1.15]	0.98 [0.92-1.05]	1.04 [1.00-1.08]	7,448	6,319	2,718	2,707	6,147
X62	1.16 [1.10-1.22]	1.19 [1.13-1.25]	1.29 [1.20-1.40]	0.98 [0.90-1.06]	1.07 [1.01-1.12]	1.05 [1.00-1.11]	0.97 [0.92-1.02]	1.02 [0.94-1.11]	0.98 [0.91-1.07]	1.04 [0.98-1.09]	4,194	3,494	1,503	1,589	3,638
X63	1.17 [1.06-1.28]	1.21 [1.09-1.34]	1.26 [1.08-1.48]	1.05 [0.90-1.23]	1.06 [0.95-1.17]	1.10 [1.00-1.21]	1.01 [0.90-1.12]	1.01 [0.85-1.19]	1.06 [0.90-1.24]	1.05 [0.95-1.17]	1,055	846	366	422	911
X64	1.20 [1.14-1.26]	1.23 [1.16-1.30]	1.37 [1.26-1.48]	1.05 [0.98-1.14]	1.09 [1.03-1.15]	1.10 [1.05-1.16]	1.02 [0.96-1.09]	1.10 [1.01-1.20]	1.03 [0.95-1.11]	1.07 [1.01-1.13]	3,886	2,796	1,307	1,811	3,242
X65	1.15 [1.10-1.21]	1.16 [1.11-1.22]	1.28 [1.20-1.37]	0.99 [0.92-1.08]	1.08 [1.03-1.13]	1.08 [1.03-1.13]	0.98 [0.93-1.02]	1.04 [0.97-1.12]	1.02 [0.94-1.11]	1.07 [1.02-1.13]	4,934	4,511	1,899	1,543	4,167
X69	1.07 [0.91-1.26]	0.94 [0.77-1.16]		1.03 [0.83-1.27]	0.95 [0.79-1.13]	1.02 [0.86-1.20]	0.81 [0.65-1.00]		1.01 [0.81-1.26]	0.94 [0.79-1.13]	386	211	NA	227	312
X70	0.97 [0.81-1.17]	0.99 [0.79-1.24]		0.98 [0.75-1.27]	0.88 [0.71-1.08]	0.96 [0.79-1.17]	0.87 [0.69-1.10]		1.04 [0.80-1.37]	0.89 [0.72-1.09]	269	174	NA	145	222
X78	1.10 [1.03-1.17]	1.09 [1.00-1.17]	1.30 [1.14-1.48]	1.03 [0.95-1.11]	1.01 [0.95-1.09]	1.02 [0.96-1.09]	0.90 [0.82-0.97]	1.05 [0.91-1.20]	1.01 [0.93-1.09]	0.99 [0.92-1.06]	2,640	1,451	548	1,650	2,162

Supplementary Table 3: Hazard ratios and events from all cohorts

Outcome	Hazard ratio (99% confidence interval)										Events (in exposed)				
	crude					adjusted									
	any age	18+	40+	<18	hosp.	any age	18+	40+	<18	hosp.	any age	18+	40+	<18	hosp.
X79	1.23 [0.90-1.67]					1.08 [0.78-1.50]					109	NA	NA	NA	NA
X83	1.03 [0.83-1.28]	1.13 [0.88-1.47]		0.87 [0.65-1.17]	0.85 [0.67-1.07]	0.95 [0.76-1.18]	0.97 [0.74-1.27]		0.84 [0.62-1.13]	0.82 [0.65-1.04]	219	138	NA	119	187
X84	1.04 [0.87-1.24]	1.04 [0.84-1.28]		0.95 [0.75-1.22]	0.97 [0.80-1.17]	0.94 [0.78-1.13]	0.81 [0.65-1.02]		0.95 [0.74-1.22]	0.91 [0.75-1.10]	316	206	NA	174	277
X99	1.06 [0.96-1.16]	1.09 [0.98-1.21]	1.17 [0.93-1.47]	1.02 [0.90-1.16]	0.96 [0.85-1.07]	1.04 [0.94-1.14]	1.02 [0.92-1.14]	1.03 [0.81-1.31]	1.02 [0.90-1.17]	0.97 [0.87-1.10]	1,053	826	168	620	720
Y00	1.08 [0.94-1.23]	1.12 [0.97-1.29]	1.25 [0.99-1.57]	0.91 [0.73-1.13]	0.95 [0.81-1.11]	1.03 [0.89-1.18]	0.99 [0.85-1.15]	1.06 [0.83-1.35]	0.89 [0.71-1.11]	0.97 [0.83-1.14]	520	450	167	201	395
Y04	1.07 [1.02-1.12]	1.12 [1.07-1.18]	1.23 [1.12-1.35]	1.00 [0.94-1.07]	0.97 [0.92-1.03]	1.03 [0.98-1.08]	1.02 [0.97-1.08]	1.06 [0.96-1.17]	0.99 [0.93-1.06]	0.97 [0.92-1.03]	4,568	3,655	1,001	2,300	3,215
Y07	0.92 [0.77-1.09]	1.01 [0.78-1.30]		0.92 [0.74-1.15]	0.88 [0.73-1.05]	0.88 [0.74-1.05]	0.82 [0.63-1.08]		0.92 [0.74-1.15]	0.87 [0.72-1.04]	317	141	NA	199	279
Y08	0.92 [0.74-1.15]	1.00 [0.79-1.26]			0.86 [0.67-1.11]	0.90 [0.71-1.12]	0.93 [0.73-1.19]			0.89 [0.68-1.16]	188	165	NA	NA	141
Y09	1.09 [0.96-1.23]	1.13 [1.00-1.29]	1.27 [1.02-1.58]	1.08 [0.89-1.32]	1.02 [0.88-1.17]	1.05 [0.92-1.19]	1.02 [0.89-1.16]	1.09 [0.87-1.37]	1.06 [0.86-1.30]	1.02 [0.88-1.17]	611	542	185	245	464
Y28	1.04 [0.81-1.33]				0.93 [0.71-1.24]	0.96 [0.75-1.24]				0.87 [0.65-1.16]	160	NA	NA	NA	115
Y34	1.12 [0.86-1.44]				1.02 [0.77-1.37]	1.03 [0.79-1.34]				0.99 [0.74-1.34]	146	NA	NA	NA	115
Y40	1.51 [1.44-1.58]	1.52 [1.45-1.60]	1.53 [1.45-1.61]	1.30 [1.15-1.47]	1.40 [1.33-1.47]	1.39 [1.32-1.45]	1.34 [1.27-1.41]	1.36 [1.29-1.43]	1.25 [1.10-1.41]	1.32 [1.26-1.39]	4,982	4,461	3,875	743	4,487
Y41	1.39 [1.26-1.55]	1.45 [1.30-1.62]	1.45 [1.29-1.62]	1.17 [0.85-1.62]	1.29 [1.16-1.44]	1.26 [1.14-1.41]	1.27 [1.14-1.42]	1.26 [1.12-1.43]	1.09 [0.78-1.54]	1.21 [1.08-1.35]	981	919	783	106	888
Y42	1.45 [1.38-1.53]	1.43 [1.36-1.51]	1.40 [1.32-1.48]	1.36 [1.14-1.63]	1.33 [1.26-1.40]	1.29 [1.22-1.36]	1.20 [1.14-1.27]	1.18 [1.11-1.25]	1.30 [1.08-1.57]	1.22 [1.15-1.28]	3,793	3,579	3,180	335	3,504
Y43	1.22 [1.17-1.26]	1.23 [1.18-1.28]	1.21 [1.16-1.26]	1.11 [0.99-1.24]	1.15 [1.11-1.19]	1.18 [1.14-1.23]	1.18 [1.13-1.22]	1.16 [1.11-1.21]	1.08 [0.96-1.22]	1.14 [1.10-1.19]	7,081	6,538	5,898	741	6,207
Y44	1.27 [1.18-1.35]	1.26 [1.18-1.34]	1.24 [1.16-1.33]	1.40 [1.03-1.92]	1.19 [1.11-1.27]	1.20 [1.13-1.29]	1.16 [1.08-1.24]	1.14 [1.06-1.22]	1.38 [1.00-1.90]	1.15 [1.07-1.23]	2,396	2,327	2,205	114	2,249
Y45	1.36 [1.31-1.42]	1.35 [1.30-1.41]	1.34 [1.28-1.40]	1.29 [1.10-1.52]	1.25 [1.19-1.30]	1.26 [1.21-1.32]	1.20 [1.15-1.25]	1.20 [1.14-1.25]	1.22 [1.03-1.45]	1.19 [1.14-1.25]	5,815	5,643	5,011	392	5,308
Y46	1.40 [1.25-1.56]	1.40 [1.25-1.57]	1.35 [1.20-1.52]	1.20 [0.88-1.65]	1.23 [1.11-1.38]	1.28 [1.14-1.43]	1.25 [1.11-1.40]	1.20 [1.07-1.36]	1.16 [0.84-1.60]	1.19 [1.06-1.33]	870	793	722	103	812
Y47	1.27 [1.09-1.49]	1.37 [1.16-1.62]	1.40 [1.17-1.67]		1.17 [0.99-1.37]	1.17 [1.00-1.38]	1.22 [1.03-1.45]	1.26 [1.05-1.51]		1.13 [0.96-1.33]	419	376	331	NA	380
Y48	1.32 [1.14-1.52]	1.32 [1.13-1.54]	1.28 [1.08-1.52]		1.20 [1.04-1.39]	1.20 [1.04-1.39]	1.16 [0.99-1.35]	1.13 [0.94-1.35]		1.14 [0.98-1.32]	499	429	328	NA	443
Y49	1.35 [1.24-1.47]	1.35 [1.24-1.47]	1.35 [1.23-1.48]		1.28 [1.18-1.40]	1.29 [1.18-1.41]	1.23 [1.12-1.34]	1.24 [1.13-1.36]		1.27 [1.16-1.39]	1,408	1,363	1,208	NA	1,292
Y51	1.22 [1.13-1.31]	1.27 [1.18-1.36]	1.24 [1.16-1.34]		1.19 [1.10-1.28]	1.18 [1.10-1.27]	1.18 [1.10-1.27]	1.16 [1.08-1.25]		1.18 [1.10-1.27]	1,998	1,976	1,919	NA	1,853
Y52	1.32 [1.26-1.39]	1.34 [1.28-1.40]	1.30 [1.24-1.36]		1.24 [1.18-1.30]	1.26 [1.20-1.32]	1.21 [1.16-1.27]	1.19 [1.13-1.24]		1.21 [1.15-1.27]	5,017	5,007	4,955	NA	4,688
Y53	1.38 [1.25-1.53]	1.39 [1.25-1.54]	1.37 [1.23-1.53]		1.31 [1.18-1.46]	1.25 [1.13-1.39]	1.19 [1.07-1.32]	1.20 [1.07-1.34]		1.22 [1.10-1.36]	1,024	992	928	NA	967
Y54	1.35 [1.28-1.42]	1.37 [1.30-1.44]	1.36 [1.29-1.43]		1.29 [1.22-1.36]	1.27 [1.21-1.34]	1.23 [1.17-1.30]	1.23 [1.17-1.30]		1.24 [1.17-1.30]	4,092	4,067	4,022	NA	3,764
Y55	1.46 [1.28-1.66]	1.43 [1.25-1.63]	1.55 [1.27-1.90]	1.31 [1.05-1.64]	1.32 [1.15-1.51]	1.28 [1.12-1.46]	1.20 [1.04-1.39]	1.24 [1.00-1.53]	1.22 [0.96-1.55]	1.16 [1.01-1.34]	644	559	245	217	541
Y56	1.80 [1.57-2.06]	1.87 [1.62-2.17]	1.88 [1.60-2.21]		1.65 [1.43-1.90]	1.62 [1.41-1.87]	1.63 [1.40-1.89]	1.61 [1.37-1.91]		1.51 [1.31-1.75]	594	516	423	NA	520
Y57	1.43 [1.34-1.53]	1.38 [1.29-1.47]	1.39 [1.29-1.49]	1.84 [1.44-2.33]	1.33 [1.24-1.42]	1.31 [1.22-1.40]	1.21 [1.13-1.30]	1.22 [1.13-1.31]	1.65 [1.28-2.13]	1.25 [1.17-1.34]	2,451	2,304	2,107	210	2,276
Y59	1.52 [1.28-1.81]	1.55 [1.25-1.92]	1.44 [1.13-1.83]	1.62 [1.23-2.13]	1.41 [1.18-1.69]	1.40 [1.17-1.68]	1.38 [1.11-1.72]	1.29 [1.01-1.65]	1.50 [1.13-1.99]	1.30 [1.08-1.57]	348	225	173	150	320
Y60	1.24 [1.17-1.32]	1.27 [1.20-1.35]	1.25 [1.17-1.33]	1.10 [0.87-1.39]	1.17 [1.10-1.24]	1.18 [1.11-1.25]	1.17 [1.10-1.25]	1.16 [1.09-1.24]	1.07 [0.85-1.36]	1.13 [1.06-1.21]	2,782	2,690	2,394	198	2,495
Y65	1.20 [1.02-1.41]	1.24 [1.05-1.47]	1.21 [1.01-1.45]		1.10 [0.93-1.30]	1.14 [0.97-1.34]	1.13 [0.95-1.35]	1.10 [0.92-1.33]		1.08 [0.91-1.28]	377	338	293	NA	335
Y71	1.59 [1.27-1.99]	1.59 [1.26-2.02]	1.56 [1.22-2.00]		1.38 [1.09-1.74]	1.51 [1.20-1.90]	1.42 [1.11-1.82]	1.43 [1.11-1.84]		1.32 [1.04-1.67]	200	181	168	NA	181
Y73	0.97 [0.75-1.25]	0.94 [0.72-1.23]	0.99 [0.75-1.30]		0.92 [0.70-1.21]	0.93 [0.71-1.21]	0.89 [0.67-1.17]	0.90 [0.68-1.20]		0.89 [0.67-1.18]	143	132	120	NA	126
Y76	1.35 [1.00-1.82]	1.37 [1.01-1.85]				1.32 [0.97-1.79]	1.26 [0.92-1.71]				109	110	NA	NA	NA
Y77	1.01 [0.78-1.30]	0.98 [0.77-1.26]	0.99 [0.78-1.28]		1.05 [0.81-1.37]	0.98 [0.76-1.27]	0.91 [0.70-1.17]	0.95 [0.74-1.22]		1.03 [0.79-1.35]	154	149	147	NA	138
Y79	1.20 [1.02-1.41]	1.24 [1.06-1.46]	1.19 [1.00-1.40]		1.15 [0.97-1.36]	1.15 [0.97-1.35]	1.16 [0.98-1.37]	1.11 [0.94-1.32]		1.10 [0.93-1.31]	383	366	338	NA	338
Y82	1.31 [1.01-1.68]	1.27 [0.97-1.68]	1.31 [0.98-1.76]		1.20 [0.92-1.56]	1.24 [0.96-1.61]	1.16 [0.88-1.54]	1.19 [0.88-1.60]		1.19 [0.91-1.55]	149	129	113	NA	135
Y83	1.28 [1.26-1.30]	1.29 [1.27-1.31]	1.27 [1.25-1.29]	1.22 [1.18-1.27]	1.18 [1.17-1.20]	1.21 [1.19-1.22]	1.17 [1.16-1.19]	1.17 [1.15-1.18]	1.17 [1.13-1.22]	1.15 [1.13-1.16]	53,081	47,693	40,066	7,973	46,779
Y84	1.24 [1.21-1.28]	1.25 [1.22-1.29]	1.24 [1.20-1.28]	1.21 [1.08-1.36]	1.16 [1.13-1.20]	1.19 [1.16-1.23]	1.16 [1.12-1.19]	1.14 [1.11-1.18]	1.16 [1.03-1.31]	1.14 [1.10-1.18]	11,136	10,644	9,831	763	10,301
Y85	1.30 [1.19-1.42]	1.30 [1.19-1.42]	1.38 [1.24-1.54]	1.16 [0.93-1.44]	1.18 [1.07-1.29]	1.23 [1.12-1.34]	1.15 [1.05-1.27]	1.25 [1.12-1.40]	1.16 [0.93-1.46]	1.13 [1.02-1.24]	1,249	1,152	803	217	1,067
Y86	1.16 [1.09-1.24]	1.20 [1.12-1.29]	1.24 [1.14-1.35]	0.99 [0.87-1.13]	1.09 [1.02-1.17]	1.11 [1.03-1.18]	1.10 [1.02-1.18]	1.13 [1.03-1.23]	0.97 [0.85-1.11]	1.06 [0.98-1.14]	2,237	1,891	1,258	595	1,858
Y87	1.23 [1.05-1.44]	1.34 [1.14-1.57]	1.39 [1.07-1.80]	1.05 [0.78-1.40]	1.16 [0.98-1.37]	1.15 [0.97-1.35]	1.18 [0.99-1.40]	1.23 [0.94-1.61]	0.98 [0.72-1.33]	1.14 [0.96-1.36]	387	353	139	115	331
Y88	1.17 [1.00-1.37]	1.18 [1.00-1.38]	1.24 [1.04-1.47]		1.02 [0.87-1.20]	1.07 [0.91-1.26]	1.01 [0.85-1.20]	1.06 [0.88-1.27]		0.97 [0.83-1.14]	397	357	312	NA	373
Y89	1.26 [0.96-1.64]	1.31 [1.00-1.72]			1.06 [0.80-1.40]	1.21 [0.92-1.59]	1.19 [0.90-1.57]			1.06 [0.80-1.42]	142	129	NA	NA	118
Y90	1.13 [0.93-1.39]	1.17 [0.94-1.44]	1.19 [0.91-1.55]		0.98 [0.80-1.21]	1.10 [0.90-1.36]	1.07 [0.86-1.33]	1.05 [0.80-1.38]		0.99 [0.80-1.23]	228	201	127	NA	200
Y91	1.15 [1.00-1.33]	1.17 [1.01-1.35]	1.27 [1.05-1.53]		1.10 [0.94-1.29]	1.12 [0.96-1.29]	1.07 [0.92-1.24]	1.10 [0.91-1.34]		1.11 [0.95-1.31]	460	441	265	NA	374
Y95	1.25 [1.22-1.27]	1.24 [1.21-1.27]	1.24 [1.22-1.27]	1.39 [1.12-1.73]	1.20 [1.17-1.22]	1.19 [1.16-1.21]	1.14 [1.11-1.16]	1.13 [1.11-1.16]	1.43 [1.14-1.79]	1.17 [1.14-1.19]	25,680	25,635	25,150	241	24,404
Factors influencing health status and contact with health services (Z00-Z99)															
Z00	1.19 [1.08-1.30]	1.22 [1.11-1.34]	1.25 [1.11-1.40]	1.18 [0.95-1.46]	1.10 [1.00-1.22]	1.12 [1.02-1.23]	1.13 [1.03-1.25]	1.15 [1.02-1.29]	1.17 [0.94-1.46]	1.07 [0.97-1.19]	1,116	978	701	218	893
Z01	2.34 [2.27-2.42]	1.41 [1.34-1.49]	1.43 [1.34-1.53]	3.12 [3.00-3.25]	2.28 [2.20-2.36]	1.96 [1.90-2.03]	1.22 [1.15-1.29]	1.26 [1.18-1.35]	2.48 [2.38-2.59]	1.90 [1.83-1.98]	11,656	3,191	2,154	8,751	9,914
Z02	1.16 [1.10-1.22]	1.17 [1.11-1.23]	1.16 [1.10-1.24]	1.06 [0.89-1.25]	1.10 [1.04-1.16]	1.11 [1.05-1.17]	1.07 [1.02-1.13]	1.08 [1.01-1.14]	1.04 [0.87-1.24]	1.07 [1.01-1.13]	3,845	3,798	2,860	338	3,365
Z03	1.34 [1.32-1.36]	1.32 [1.30-1.35]	1.33 [1.30-1.36]	1.38 [1.34-1.43]	1.26 [1.23-1.28]	1.25 [1.23-1.27]	1.19 [1.16-1.21]	1.19 [1.17-1.22]	1.30 [1.25-1.34]	1.20 [1.17-1.22]	36,893	29,542	24,467	9,073	32,200
Z04	1.22 [1.17-1.27]	1.27 [1.21-1.33]	1.32 [1.24-1.40]	1.14 [1.07-1.21]	1.12 [1.08-1.17]	1.16 [1.11-1.21]	1.16 [1.10-1.21]	1.22 [1.15-1.29]	1.11 [1.04-1.18]	1.09 [1.04-1.14]	6,480	4,365	2,846	2,567	5,152
Z08	1.08 [1.05-1.12]	1.08 [1.05-1.12]	1.08 [1.05-1.12]	0.98 [0.82-1.18]	1.05 [1.01-1.09]	1.07 [1.03-1.10]	1.05 [1.02-1.09]	1.05 [1.02-1.09]	1.01 [0.84-1.22]	1.05 [1.01-1.09]	8,526	8,285	8,052	288	7,717
Z09	1.28 [1.25-1.31]	1.29 [1.26-1.32]	1.29 [1.26-1.32]	1.18 [1.11-1.26]	1.18 [1.15-1.20]	1.19 [1.17-1.22]	1.16 [1.13-1.19]	1.17 [1.14-1.20]	1.14 [1.06-1.21]	1.12 [1.10-1.15]	19,415	17,514	15,298	2,487	17,468
Z11	1.22 [1.20-1.23]	1.21 [1.20-1.23]	1.23 [1.21-1.25]	1.17 [1.14-1.21]	1.15 [1.13-1.17]	1.15 [1.13-1.16]	1.10 [1.08-1.11]	1.10 [1.09-1.12]	1.14 [1.11-1.17]	1.12 [1.10-1.13]	60,391	53,760	39,449	13,770	55,884
Z12	1.16 [1.13-1.19]	1.16 [1.13-1.19]	1.15 [1.12-1.18]	1.30 [0.98-1.74]	1.10 [1.06-1.13]	1.12 [1.09-1.15]	1.13 [1.09-1.16]	1.11 [1.08-1.15]	1.23 [0.91-1.65]	1.07 [1.04-1.11]	12,500	12,458	11,973	125	10,207
Z13	1.29 [1.27-1.31]	1.26 [1.24-1.28]	1.27 [1.24-1.29]	1.37 [1.32-1.42]	1.19 [1.17-1.21]	1.22 [1.19-1.24]	1.16 [1.14-1.18]	1.15 [1.13-1.18]	1.29 [1.24-1.33]	1.14 [1.12-1.17]	39,843	34,601	24,707	8,731	33,396
Z20	1.25 [1.17-1.34]	1.24 [1.15-1.34]	1.25 [1.15-1.36]	1.22 [1.06-1.42]	1.16 [1.08-1.25]	1.19 [1.10-1.28]	1.13 [1.05-1.23]	1.13 [1.04-1.24]	1.19 [1.03-1.39]	1.12 [1.04-1.21]	2,183	1,817	1,522	495	2,010
Z22	1.40 [1.36-1.43]	1.41 [1.38-1.45]	1.48 [1.43-1.53]	1.13 [1.06-1.20]	1.30 [1.27-1.33]	1.32 [1.29-1.35]	1.29 [1.26-1.33]	1.34 [1.30-1.38]	1.10 [1.03-1.17]	1.26 [1.23-1.29]	19,582	18,903	11,068	2,859	17,798
Z24	1.07 [0.91-1.26]	1.13 [0.95-1.35]		1.02 [0.80-1.31]	1.07 [0.89-1.29]	1.07 [0.90-1.27]	1.12 [0.93-1.33]		0.98 [0.76-1.27]	1.09 [0.90-1.32]	353	301	NA	158	279
Z25	2.82 [2.31-3.44]			3.36 [2.69-4.21]	2.63 [2.15-3.22]	2.27 [1.84-2.80]			2.52 [1.98-3.21]	2.17 [1.75-2.69]	375	NA	NA	324	349
Z27	2.36 [2.17-2.57]	1.06 [0.93-1.20]		3.25 [2.94-3.59]	2.26 [2.06-2.48]	2.10 [1.92-2.29]	1.01 [0.89-1.15]		2.71 [2.44-3.01]	2.04 [1.85-2.25]	1,707	585	NA	1,371	1,355
Z29	1.25 [1.21-1.29]	1.25 [1.21-1.29]	1.29 [1.23-1.34]	1.17 [1.09-1.26]	1.20 [1.16-1.24]	1.19 [1.15-1.23]	1.15 [1.11-1.19]	1.17 [1.12-1.22]	1.14 [1.06-1.23]	1.16 [1.12-1.20]	10,193	9,224	6,022	1,986	9,202
Z30	1.16 [1.13-1.20]	1.17 [1.14-1.21]	1.20 [1.14-1.26]	1.03 [0.94-1.13]	1.06 [1.02-1.09]	1.12 [1.09-1.15]	1.10 [1.06-1.13]	1.11 [1.05-1.17]	1.03 [0.94-1.13]	1.05 [1.01-1.08]	10,748	10,535	3,391	1,118	8,192
Z31	1.02 [0.81-1.28]	1.06 [0.85-1.33]			1.20 [0.91-1.57]	0.97 [0.77-1.23]	0.98 [0.77-1.23]			1.15 [0.86-1.53]	170	170	NA	NA	114
Z32	1.23 [1.10-1.37]	1.23 [1.10-1.37]		1.06 [0.86-1.31]	1.09 [0.97-1.23]	1.17 [1.05-1.31]	1.13 [1.01-1.26]		1.07 [0.86-1.34]	1.07 [0.95-1.21]	827	786	NA	218	661
Z33	1.23 [1.18-1.27]	1.26 [1.22-1.31]	1.19 [1.00-1.42]	1.12 [1.05-1.20]	1.12 [1.08-1.17]	1.14 [1.10-1.19]	1.12 [1.07-1.16]	1.08 [0.90-1.29]	1.10 [1.03-1.18]	1.08 [1.04-1.12]	7,874	7,664	280	2,540	6,613
Z34	1.09 [1.07-1.11]	1.12 [1.09-1.14]	0.97 [0.85-1.09]	0.99 [0.95-1.03]	1.03 [1.00-1.05]	1.06 [1.04-1.09]	1.07 [1.04-1.09]	0.96 [0.84-1.09]	0.99 [0.95-1.03]	1.02 [0.99-1.04]	20,140	19,711	555	6,167	15,116
Z35	1.07 [1.04-1.09]	1.08 [1.05-1.11]	0.96 [0.89-1.03]	0.97 [0.92-1.02]	1.00 [0.98-1.02]	1.04 [1.02-1.07]	1.05 [1.02-1.07]	0.97 [0.90-1.05]	0.96 [0.91-1.01]	1.00 [0.98-1.03]	17,420	17,053	1,680	3,937	15,199
Z36	1.12 [1.07-1.18]	1.12 [1.07-1.18]	1.10 [0.89-1.36]	0.99 [0.87-1.12]	1.05 [0.99-1.11]	1.10 [1.04-1.16]	1.09 [1.03-1.14]	1.13 [0.91-1.42]	0.97 [0.85-1.10]	1.05 [0.98-1.11]	3,620	3,545	185	590	2,480
Z37	1.07 [1.06-1.09]	1.12 [1.10-1.13]	1.06 [0.99-1.13]	0.98 [0.96-1.00]	1.00 [0.98-1.01]	1.06 [1.05-1.08]	1.08 [1.07-1.10]	1.12 [1.04-1.20]	0.98 [0.96-1.00]	1.00 [0.99-1.02]	78,196	76,161	2,190	27,275	41,423
Z38	1.09 [0.88-1.35]	1.18 [0.95-1.47]			1.02 [0.80-1.31]	1.09 [0.88-1.36]	1.18 [0.94-1.48]			1.01 [0.79-1.29]	203	194	NA	NA	148
Z39	1.08 [1.04-1.12]	1.09 [1.05-1.14]	1.03 [0.86-1.24]	1.05 [0.97-1.14]	1.03 [0.98-1.08]	1.07 [1.03-1.12]	1.07 [1.03-1.12]	1.06 [0.88-1.29]	1.06 [0.97-1.15]	1.03 [0.98-1.08]	5,909	5,809	242	1,473	4,324
Z40	1.12 [1.02-1.22]	1.11 [1.00-1.22]	1.11 [1.00-1.24]	1.07 [0.85-1.35]	1.02 [0.93-1.12]	1.10 [1.00-1.20]	1.05 [0.95-1.16]	1.06 [0.96-1.19]	1.05 [0.83-1.33]	1.01 [0.92-1.12]	1,164	999	793	209	1,030
Z41	1.34 [1.24-1.45]	1.24 [1.12-1.37]	1.34 [1.18-1.52]	1.39 [1.24-1.57]	1.22 [1.12-1.33]	1.29 [1.19-1.40]	1.13 [1.02-1.26]	1.23 [1.08-1.40]	1.39 [1.23-1.57]	1.21 [1.11-1.32]	1,558	932	558	744	1,282
Z42	1.18 [1.12-1.25]	1.17 [1.11-1.24]	1.15 [1.09-1.23]	1.15 [0.98-1.36]	1.11 [1.05-1.17]	1.16 [1.09-1.22]	1.10 [1.04-1.17]	1.10 [1.03-1.17]	1.15 [0.97-1.36]	1.11 [1.05-1.18]	3,092	2,864	2,308	375	2,820
Z43	1.15 [1.10-1.20]	1.24 [1.18-1.30]	1.18 [1.12-1.24]	0.93 [0.83-1.04]	1.09 [1.04-1.14]	1.11 [1.06-1.16]	1.17 [1.11-1.23]	1.12 [1.07-1.19]	0.87 [0.77-0.98]	1.08 [1.03-1.13]	4,563	3,992	3,496	722	4,114
Z44	1.32 [0.98-1.79]					1.28 [0.93-1.74]					109	NA	NA	NA	NA
Z45	1.20 [1.16-1.24]	1.23 [1.19-1.27]	1.21 [1.16-1.25]	1.08 [0.99-1.18]	1.10 [1.06-1.13]	1.14 [1.11-1.18]	1.13 [1.10-1.17]	1.11 [1.07-1.15]	1.04 [0.96-1.14]	1.07 [1.04-1.11]	9,851	8,771	7,938	1,342	8,975
Z46	1.14 [1.11-1.17]	1.15 [1.12-1.19]	1.14 [1.10-1.17]	1.05 [0.95-1.17]	1.07 [1.04-1.10]	1.11 [1.08-1.14]	1.10 [1.06-1.13]	1.08 [1.05-1.12]	1.02 [0.92-1.14]	1.06 [1.03-1.09]	11,527	10,919	9,939	913	10,056
Z47	1.12 [1.08-1.15]	1.19 [1.14-1.24]	1.21 [1.15-1.28]	1.03 [0.99-1.08]	1.08 [1.05-1.12]	1.07 [1.04-1.11]	1.10 [1.06-1.15]	1.13 [1.07-1.19]	1.02 [0.97-1.06]	1.06 [1.03-1.10]	9,978	5,300	3,416	5,434	8,236
Z48	1.20 [1.14-1.26]	1.30 [1.22-1.38]	1.22 [1.15-1.31]	1.10 [1.01-1.20]	1.15 [1.08-1.21]	1.15 [1.10-1.22]	1.21 [1.14-1.29]	1.15 [1.08-1.23]	1.06 [0.97-1.16]	1.12 [1.06-1.19]	3,747	2,608	2,063	1,292	3,160
Z49	1.44 [1.34-1.54]	1.46 [1.37-1.57]	1.44 [1.34-1.55]		1.25 [1.17-1.34]	1.40 [1.31-1.50]	1.35 [1.25-1.45]	1.30 [1.21-1.40]		1.26 [1.17-1.36]	2,075	2,028	1,871	NA	1,896
Z50	1.19 [1.17-1.20]	1.19 [1.17-1.20]	1.19 [1.17-1.20]	1.12 [1.06-1.18]	1.14 [1.12-1.15]	1.14 [1.13-1.16]	1.11 [1.09-1.12]	1.11 [1.09-1.12]	1.07 [1.02-1.13]	1.12 [1.10-1.13]	70,554	68,190	64,410	3,939	63,974
Z51	1.21 [1.20-1.23]	1.21 [1.19-1.22]	1.20 [1.19-1.21]	1.22 [1.17-1.27]	1.16 [1.14-1.17]	1.17 [1.16-1.18]	1.13 [1.12-1.14]	1.12 [1.11-1.14]	1.17 [1.12-1.22]	1.13 [1.12-1.15]	83,777	79,689	72,749	5,874	74,534
Z52	1.00 [0.86-1.17]	1.02 [0.87-1.19]	0.97 [0.80-1.18]		0.94 [0.79-1.13]	1.01 [0.86-1.18]	1.03 [0.88-1.21]	0.99 [0.82-1.20]		0.96 [0.80-1.15]	394	353	231	NA	273
Z53	1.31 [1.30-1.33]	1.32 [1.31-1.34]	1.31 [1.29-1.33]	1.25 [1.21-1.28]	1.21 [1.20-1.23]	1.23 [1.22-1.25]	1.20 [1.18-1.21]	1.19 [1.17-1.20]	1.19 [1.16-1.23]	1.17 [1.15-1.18]	78,316	68,368	57,099	13,474	67,570
Z54	1.40 [1.24-1.58]	1.35 [1.20-1.53]	1.40 [1.24-1.59]		1.27 [1.11-1.47]	1.36 [1.20-1.54]	1.29 [1.14-1.46]	1.33 [1.17-1.51]		1.27 [1.10-1.46]	669	661	652	NA	518
Z55	1.33 [1.13-1.56]			1.23 [1.03-1.46]	1.18 [0.99-1.41]	1.25 [1.06-1.48]			1.19 [0.99-1.42]	1.15 [0.96-1.38]	436	NA	NA	379	359
Z56	1.17 [1.06-1.31]	1.16 [1.05-1.29]	1.14 [0.99-1.32]	1.00 [0.78-1.29]	1.07 [0.96-1.20]	1.09 [0.98-1.22]	1.00 [0.89-1.11]	0.99 [0.85-1.14]	0.96 [0.73-1.25]	1.04 [0.92-1.16]	836	828	444	163	713
Z57	1.27 [1.20-1.35]	1.26 [1.18-1.34]	1.27 [1.19-1.35]		1.21 [1.14-1.29]	1.21 [1.14-1.29]	1.16 [1.09-1.24]	1.17 [1.10-1.25]		1.16 [1.09-1.24]	2,575	2,573	2,527	NA	2,317
Z58	1.32 [1.23-1.41]	1.23 [1.13-1.33]	1.25 [1.14-1.37]	1.44 [1.27-1.62]	1.24 [1.15-1.33]	1.22 [1.14-1.31]	1.09 [1.00-1.19]	1.11 [1.01-1.22]	1.36 [1.20-1.54]	1.16 [1.08-1.25]	2,140	1,496	1,248	755	1,967
Z59	1.09 [1.01-1.17]	1.09 [1.01-1.17]	1.11 [1.01-1.21]	1.02 [0.87-1.21]	1.00 [0.93-1.08]	1.05 [0.97-1.13]	0.98 [0.91-1.06]	1.00 [0.91-1.10]	1.08 [0.90-1.28]	1.03 [0.96-1.12]	1,829	1,710	1,134	364	1,639
Z60	1.18 [1.15-1.21]	1.18 [1.16-1.21]	1.18 [1.16-1.21]	0.92 [0.80-1.06]	1.12 [1.09-1.15]	1.14 [1.11-1.16]	1.11 [1.08-1.13]	1.11 [1.08-1.13]	0.92 [0.80-1.06]	1.10 [1.08-1.13]	21,873	21,488	20,779	524	19,012
Z61	1.06 [0.96-1.17]	1.03 [0.89-1.18]	1.31 [1.05-1.64]	0.98 [0.87-1.11]	0.95 [0.86-1.06]	1.00 [0.90-1.11]	0.86 [0.74-0.99]	1.05 [0.83-1.32]	0.97 [0.86-1.10]	0.94 [0.85-1.05]	1,042	473	184	706	885
Z62	1.01 [0.82-1.23]			0.96 [0.77-1.21]	0.87 [0.69-1.08]	0.93 [0.76-1.15]			0.94 [0.75-1.19]	0.84 [0.67-1.06]	238	NA	NA	194	198
Z63	1.12 [1.08-1.16]	1.17 [1.12-1.22]	1.23 [1.17-1.30]	0.94 [0.88-1.00]	1.05 [1.01-1.09]	1.05 [1.01-1.09]	1.02 [0.98-1.07]	1.08 [1.02-1.14]	0.92 [0.86-0.98]	1.03 [0.99-1.07]	7,491	5,594	3,787	2,657	6,615
Z64	0.99 [0.78-1.25]	1.01 [0.79-1.29]			0.99 [0.75-1.30]	0.94 [0.74-1.20]	0.90 [0.69-1.17]			0.98 [0.74-1.30]	168	148	NA	NA	122
Z65	1.09 [0.96-1.24]	1.14 [0.99-1.32]	1.18 [0.95-1.46]	0.96 [0.78-1.17]	1.00 [0.87-1.14]	1.04 [0.91-1.18]	0.99 [0.86-1.15]	1.00 [0.80-1.25]	0.99 [0.80-1.23]	1.01 [0.88-1.16]	604	459	202	247	541
Z71	1.65 [1.59-1.70]	1.22 [1.16-1.27]	1.18 [1.12-1.23]	2.95 [2.79-3.12]	1.57 [1.51-1.62]	1.49 [1.44-1.55]	1.11 [1.06-1.16]	1.09 [1.04-1.15]	2.36 [2.22-2.50]	1.43 [1.38-1.48]	9,814	5,511	4,554	4,667	8,735
Z72	1.20 [1.18-1.22]	1.21 [1.19-1.22]	1.22 [1.20-1.24]	1.02 [0.98-1.06]	1.11 [1.09-1.12]	1.17 [1.15-1.18]	1.09 [1.07-1.11]	1.12 [1.10-1.14]	1.02 [0.98-1.07]	1.11 [1.10-1.13]	47,679	46,403	31,807	5,984	36,499
Z73	1.18 [1.14-1.22]	1.18 [1.14-1.22]	1.19 [1.15-1.22]	1.08 [0.91-1.27]	1.13 [1.09-1.16]	1.13 [1.10-1.17]	1.09 [1.06-1.12]	1.10 [1.07-1.14]	1.05 [0.88-1.24]	1.11 [1.07-1.14]	11,575	11,392	10,814	405	10,721
Z74	1.18 [1.14-1.22]	1.17 [1.13-1.21]	1.17 [1.13-1.21]		1.12 [1.08-1.16]	1.16 [1.12-1.20]	1.12 [1.08-1.16]	1.11 [1.08-1.15]		1.12 [1.08-1.16]	8,723	8,665	8,539	NA	7,908
Z75	1.13 [1.10-1.16]	1.12 [1.09-1.15]	1.12 [1.09-1.15]	1.13 [0.96-1.32]	1.10 [1.07-1.13]	1.11 [1.08-1.14]	1.07 [1.04-1.10]	1.07 [1.05-1.10]	1.11 [0.94-1.31]	1.10 [1.07-1.13]	16,971	16,704	16,369	397	15,746
Z76	1.20 [1.10-1.30]	1.17 [1.07-1.28]	1.43 [1.21-1.69]	1.19 [1.01-1.39]	1.15 [1.04-1.26]	1.18 [1.08-1.28]	1.15 [1.05-1.26]	1.36 [1.15-1.61]	1.13 [0.95-1.33]	1.14 [1.04-1.25]	1,414	1,206	381	403	1,143
Z80	1.17 [1.14-1.20]	1.17 [1.15-1.20]	1.15 [1.12-1.18]	1.22 [1.11-1.34]	1.09 [1.06-1.12]	1.11 [1.09-1.14]	1.09 [1.07-1.12]	1.08 [1.05-1.10]	1.17 [1.06-1.29]	1.06 [1.03-1.08]	18,501	18,055	15,189	1,231	15,525
Z81	1.18 [1.11-1.24]	1.16 [1.08-1.24]	1.33 [1.16-1.51]	1.13 [1.05-1.21]	1.07 [1.01-1.13]	1.11 [1.05-1.17]	1.00 [0.92-1.07]	1.14 [1.00-1.31]	1.09 [1.02-1.18]	1.05 [0.99-1.11]	3,400	1,813	534	2,171	2,979
Z82	1.30 [1.28-1.32]	1.23 [1.21-1.25]	1.23 [1.21-1.26]	1.56 [1.51-1.61]	1.21 [1.19-1.23]	1.23 [1.21-1.24]	1.11 [1.09-1.13]	1.11 [1.09-1.14]	1.50 [1.45-1.54]	1.17 [1.15-1.19]	44,479	35,084	27,088	12,010	38,153
Z83	1.23 [1.21-1.27]	1.22 [1.19-1.26]	1.20 [1.16-1.25]	1.24 [1.19-1.29]	1.17 [1.14-1.20]	1.17 [1.14-1.20]	1.13 [1.10-1.16]	1.11 [1.07-1.15]	1.19 [1.14-1.24]	1.13 [1.10-1.16]	17,209	13,223	6,277	6,352	14,733
Z84	1.76 [1.66-1.87]	1.25 [1.14-1.37]	1.41 [1.22-1.64]	1.96 [1.83-2.10]	1.61 [1.52-1.71]	1.69 [1.59-1.80]	1.18 [1.07-1.30]	1.30 [1.12-1.52]	1.88 [1.75-2.02]	1.57 [1.47-1.67]	3,330	1,131	433	2,435	2,975
Z85	1.17 [1.15-1.18]	1.17 [1.15-1.18]	1.17 [1.15-1.18]	1.05 [0.96-1.16]	1.10 [1.08-1.11]	1.13 [1.12-1.15]	1.10 [1.09-1.12]	1.10 [1.09-1.12]	1.06 [0.96-1.17]	1.09 [1.08-1.10]	66,459	65,798	63,639	1,095	60,350
Z86	1.26 [1.25-1.27]	1.26 [1.26-1.27]	1.28 [1.27-1.29]	1.10 [1.08-1.13]	1.17 [1.16-1.18]	1.19 [1.19-1.20]	1.14 [1.13-1.15]	1.16 [1.15-1.17]	1.09 [1.06-1.11]	1.14 [1.13-1.15]	226,366	219,938	182,267	19,235	197,681
Z87	1.29 [1.28-1.30]	1.30 [1.28-1.31]	1.29 [1.28-1.31]	1.25 [1.23-1.28]	1.19 [1.18-1.20]	1.20 [1.19-1.21]	1.16 [1.14-1.17]	1.16 [1.14-1.17]	1.19 [1.17-1.22]	1.13 [1.12-1.15]	123,301	106,361	82,698	24,819	112,318
Z88	1.47 [1.45-1.48]	1.46 [1.45-1.48]	1.46 [1.44-1.47]	1.46 [1.42-1.49]	1.37 [1.36-1.38]	1.33 [1.31-1.34]	1.27 [1.26-1.29]	1.28 [1.26-1.29]	1.34 [1.30-1.37]	1.27 [1.25-1.28]	131,321	120,607	95,640	19,072	112,610
Z89	1.28 [1.21-1.35]	1.29 [1.22-1.36]	1.29 [1.22-1.36]		1.18 [1.11-1.24]	1.23 [1.16-1.30]	1.17 [1.10-1.24]	1.15 [1.09-1.22]		1.17 [1.11-1.24]	3,283	3,232	3,109	NA	3,063
Z90	1.22 [1.21-1.24]	1.22 [1.21-1.24]	1.21 [1.20-1.23]	1.21 [1.14-1.28]	1.13 [1.12-1.15]	1.15 [1.13-1.16]	1.11 [1.09-1.12]	1.10 [1.09-1.12]	1.16 [1.09-1.23]	1.10 [1.08-1.11]	59,573	58,287	52,051	3,107	55,520
Z91	1.76 [1.73-1.78]	1.54 [1.52-1.56]	1.56 [1.53-1.59]	2.13 [2.08-2.18]	1.61 [1.59-1.64]	1.54 [1.52-1.56]	1.29 [1.27-1.31]	1.33 [1.30-1.36]	1.86 [1.81-1.90]	1.46 [1.44-1.48]	66,982	46,826	30,367	27,043	59,336
Z92	1.24 [1.23-1.25]	1.24 [1.23-1.25]	1.23 [1.22-1.24]	1.19 [1.13-1.25]	1.15 [1.14-1.16]	1.17 [1.16-1.18]	1.13 [1.12-1.14]	1.12 [1.11-1.13]	1.14 [1.08-1.19]	1.12 [1.11-1.13]	142,365	139,836	132,899	4,690	128,638
Z93	1.19 [1.16-1.23]	1.22 [1.19-1.26]	1.19 [1.16-1.22]	0.96 [0.87-1.05]	1.12 [1.09-1.15]	1.15 [1.12-1.18]	1.15 [1.12-1.18]	1.13 [1.09-1.16]	0.90 [0.82-0.99]	1.11 [1.08-1.14]	13,568	12,723	11,716	1,158	12,368
Z94	1.36 [1.28-1.44]	1.42 [1.33-1.50]	1.40 [1.31-1.50]	1.09 [0.94-1.26]	1.21 [1.14-1.28]	1.31 [1.23-1.38]	1.32 [1.24-1.40]	1.30 [1.21-1.39]	1.06 [0.91-1.24]	1.20 [1.13-1.27]	2,996	2,708	2,181	449	2,741
Z95	1.22 [1.21-1.24]	1.23 [1.22-1.25]	1.23 [1.21-1.25]	0.95 [0.86-1.05]	1.12 [1.10-1.14]	1.17 [1.15-1.19]	1.13 [1.11-1.15]	1.12 [1.10-1.14]	0.92 [0.83-1.02]	1.10 [1.08-1.12]	45,729	45,044	44,079	1,021	42,251
Z96	1.22 [1.20-1.23]	1.22 [1.20-1.23]	1.22 [1.20-1.23]	1.12 [1.04-1.20]	1.14 [1.13-1.15]	1.17 [1.15-1.18]	1.14 [1.13-1.15]	1.14 [1.13-1.15]	1.08 [1.01-1.17]	1.11 [1.10-1.13]	88,982	87,678	85,483	2,009	81,648
Z97	1.26 [1.22-1.31]	1.29 [1.24-1.33]	1.26 [1.22-1.31]	1.12 [1.01-1.23]	1.18 [1.14-1.22]	1.18 [1.14-1.23]	1.16 [1.12-1.21]	1.16 [1.12-1.21]	1.07 [0.97-1.18]	1.14 [1.10-1.18]	9,194	8,666	6,867	1,135	8,518
Z98	1.29 [1.26-1.33]	1.32 [1.28-1.36]	1.28 [1.25-1.32]	1.08 [0.98-1.19]	1.19 [1.16-1.23]	1.18 [1.15-1.22]	1.16 [1.12-1.19]	1.14 [1.10-1.17]	1.03 [0.93-1.14]	1.13 [1.10-1.16]	12,376	11,819	10,149	1,038	11,673
Z99	1.39 [1.36-1.43]	1.42 [1.38-1.45]	1.41 [1.38-1.45]	0.94 [0.86-1.04]	1.25 [1.22-1.28]	1.28 [1.25-1.31]	1.23 [1.20-1.27]	1.23 [1.19-1.26]	0.91 [0.82-1.01]	1.19 [1.16-1.22]	16,202	15,470	14,479	1,037	15,347

Supplementary Table 4: Hazard ratios and events from all cohorts (excluding non-consulters)

	Hazard ratio (99% confidence interval)										Events (in exposed)				
	crude					adjusted									
Outcome	any age	18+	40+	<18	hosp.	any age	18+	40+	<18	hosp.	any age	18+	40+	<18	hosp.
Certain infectious and parasitic diseases (A00-B99)															
A02	0.98 [0.77-1.25]	0.95 [0.74-1.22]	1.04 [0.79-1.36]		0.93 [0.72-1.19]	0.92 [0.71-1.18]	0.88 [0.68-1.14]	0.95 [0.72-1.26]		0.88 [0.68-1.14]	192	175	151	NA	169
A04	1.18 [1.13-1.22]	1.17 [1.13-1.21]	1.17 [1.12-1.22]	0.95 [0.75-1.22]	1.15 [1.11-1.20]	1.13 [1.09-1.18]	1.11 [1.07-1.15]	1.10 [1.06-1.15]	0.90 [0.70-1.16]	1.12 [1.08-1.17]	8,234	8,145	7,600	217	7,681
A07	1.06 [0.77-1.46]				1.24 [0.90-1.71]	0.99 [0.72-1.37]				1.22 [0.88-1.68]	119	NA	NA	NA	110
A08	1.18 [1.13-1.23]	1.18 [1.12-1.24]	1.21 [1.15-1.28]	1.16 [1.08-1.24]	1.16 [1.11-1.21]	1.14 [1.09-1.19]	1.09 [1.03-1.15]	1.12 [1.06-1.19]	1.14 [1.07-1.22]	1.13 [1.08-1.18]	6,714	4,327	3,700	2,622	6,347
A09	1.18 [1.16-1.21]	1.19 [1.17-1.21]	1.18 [1.16-1.21]	1.04 [0.98-1.12]	1.15 [1.13-1.17]	1.12 [1.10-1.14]	1.09 [1.07-1.11]	1.09 [1.07-1.11]	1.02 [0.95-1.09]	1.11 [1.08-1.13]	34,612	33,538	28,367	2,970	33,614
A15	1.15 [0.88-1.50]	1.16 [0.89-1.51]	1.23 [0.93-1.64]		1.15 [0.87-1.51]	1.12 [0.85-1.46]	1.14 [0.87-1.49]	1.16 [0.87-1.55]		1.10 [0.83-1.46]	162	160	138	NA	151
A16	1.30 [1.10-1.55]	1.27 [1.07-1.52]	1.32 [1.09-1.60]		1.28 [1.07-1.53]	1.25 [1.05-1.49]	1.21 [1.01-1.45]	1.25 [1.03-1.53]		1.23 [1.02-1.48]	409	395	323	NA	344
A18	1.13 [0.88-1.44]	1.25 [0.97-1.61]	1.24 [0.94-1.64]		1.14 [0.88-1.47]	1.07 [0.83-1.38]	1.21 [0.94-1.57]	1.19 [0.90-1.58]		1.11 [0.85-1.44]	187	187	146	NA	161
A31	1.50 [1.16-1.93]	1.52 [1.16-1.98]	1.48 [1.11-1.96]		1.49 [1.16-1.92]	1.40 [1.07-1.82]	1.33 [1.01-1.76]	1.36 [1.02-1.82]		1.35 [1.04-1.75]	183	171	151	NA	172
A38	1.17 [0.85-1.62]			1.07 [0.77-1.49]	1.20 [0.86-1.66]	1.12 [0.81-1.56]			1.05 [0.75-1.46]	1.17 [0.84-1.63]	130	NA	NA	124	131
A39	0.98 [0.75-1.30]				0.92 [0.69-1.23]	0.95 [0.72-1.26]				0.90 [0.67-1.20]	151	NA	NA	NA	136
A40	1.22 [1.12-1.33]	1.25 [1.15-1.36]	1.27 [1.17-1.39]		1.24 [1.14-1.34]	1.19 [1.09-1.30]	1.20 [1.10-1.31]	1.21 [1.11-1.32]		1.22 [1.12-1.33]	1,706	1,687	1,607	NA	1,673
A41	1.16 [1.14-1.18]	1.17 [1.14-1.19]	1.17 [1.14-1.19]	1.05 [0.91-1.20]	1.15 [1.13-1.17]	1.13 [1.11-1.16]	1.11 [1.09-1.14]	1.11 [1.09-1.13]	1.04 [0.89-1.20]	1.14 [1.12-1.16]	32,658	32,404	31,120	638	31,785
A46	1.82 [1.33-2.49]	1.89 [1.38-2.60]	1.98 [1.42-2.75]		1.81 [1.31-2.49]	1.76 [1.28-2.41]	1.78 [1.29-2.47]	1.90 [1.36-2.65]		1.78 [1.29-2.45]	135	139	124	NA	119
A48	1.14 [0.85-1.52]	0.98 [0.73-1.32]	1.12 [0.83-1.52]		1.27 [0.95-1.69]	1.14 [0.85-1.53]	0.96 [0.71-1.29]	1.09 [0.80-1.48]		1.30 [0.97-1.74]	139	123	123	NA	133
A49	1.23 [1.17-1.29]	1.24 [1.18-1.31]	1.22 [1.16-1.29]	1.00 [0.76-1.31]	1.20 [1.14-1.26]	1.19 [1.13-1.25]	1.16 [1.10-1.23]	1.15 [1.09-1.21]	0.98 [0.75-1.30]	1.17 [1.11-1.23]	4,598	4,477	4,236	177	4,369
A63	1.34 [0.97-1.85]	1.32 [0.96-1.82]				1.17 [0.82-1.65]	1.17 [0.84-1.64]				108	107	NA	NA	NA
A86	1.12 [0.87-1.44]	1.04 [0.81-1.34]	1.17 [0.89-1.53]		1.07 [0.82-1.39]	1.09 [0.85-1.40]	0.99 [0.77-1.28]	1.10 [0.84-1.46]		1.03 [0.79-1.34]	179	170	149	NA	159
A87	1.20 [1.01-1.42]	1.26 [1.07-1.49]	1.50 [1.12-2.00]		1.15 [0.97-1.36]	1.10 [0.93-1.31]	1.15 [0.97-1.37]	1.35 [1.00-1.82]		1.10 [0.93-1.31]	437	431	144	NA	404
B00	1.93 [1.76-2.12]	1.66 [1.49-1.85]	1.52 [1.35-1.72]	3.30 [2.76-3.95]	1.82 [1.65-2.00]	1.82 [1.65-2.00]	1.52 [1.36-1.70]	1.40 [1.24-1.58]	3.18 [2.65-3.83]	1.72 [1.57-1.90]	1,698	1,150	857	651	1,547
B01	1.56 [1.38-1.78]	0.97 [0.73-1.30]		1.70 [1.47-1.96]	1.53 [1.34-1.74]	1.53 [1.34-1.74]	0.91 [0.67-1.23]		1.67 [1.44-1.93]	1.51 [1.31-1.73]	831	136	NA	694	762
B02	1.32 [1.23-1.42]	1.29 [1.20-1.38]	1.28 [1.19-1.37]		1.28 [1.19-1.37]	1.26 [1.18-1.36]	1.21 [1.12-1.30]	1.21 [1.12-1.30]		1.23 [1.15-1.32]	2,484	2,436	2,314	NA	2,389
B07	1.24 [1.10-1.40]	1.24 [1.10-1.40]	1.31 [1.15-1.49]		1.25 [1.11-1.41]	1.19 [1.05-1.34]	1.14 [1.01-1.29]	1.23 [1.08-1.40]		1.20 [1.07-1.36]	859	826	673	NA	784
B08	1.63 [1.33-2.00]			1.59 [1.25-2.01]	1.72 [1.39-2.11]	1.54 [1.25-1.89]			1.51 [1.18-1.92]	1.61 [1.30-2.00]	335	NA	NA	248	316
B09	1.12 [0.80-1.58]					1.11 [0.78-1.56]					103	NA	NA	NA	NA
B16	1.11 [0.88-1.41]	1.13 [0.89-1.45]	1.29 [0.97-1.71]		0.99 [0.77-1.26]	1.10 [0.86-1.40]	1.14 [0.89-1.47]	1.28 [0.96-1.71]		0.99 [0.77-1.27]	197	188	143	NA	170
B17	1.17 [0.96-1.44]	1.28 [1.05-1.57]	1.39 [1.11-1.73]		1.11 [0.91-1.36]	1.13 [0.92-1.39]	1.21 [0.98-1.48]	1.28 [1.02-1.61]		1.08 [0.88-1.33]	286	285	238	NA	265
B18	1.07 [0.97-1.18]	1.09 [0.99-1.21]	1.14 [1.02-1.27]		1.00 [0.90-1.10]	1.06 [0.96-1.18]	1.06 [0.95-1.17]	1.09 [0.98-1.22]		1.02 [0.92-1.13]	1,173	1,167	965	NA	1,052
B25	1.26 [1.01-1.58]	1.18 [0.94-1.47]	1.32 [1.02-1.71]		1.08 [0.86-1.34]	1.24 [0.98-1.55]	1.10 [0.87-1.38]	1.29 [0.99-1.68]		1.04 [0.83-1.31]	240	231	186	NA	219
B27	1.10 [0.92-1.31]	0.98 [0.81-1.18]		1.12 [0.90-1.40]	1.03 [0.84-1.26]	1.03 [0.86-1.25]	0.89 [0.73-1.09]		1.08 [0.85-1.36]	0.96 [0.77-1.19]	422	333	NA	278	302
B34	1.50 [1.46-1.55]	1.19 [1.13-1.25]	1.24 [1.16-1.32]	1.66 [1.60-1.73]	1.46 [1.42-1.51]	1.44 [1.39-1.48]	1.06 [1.01-1.12]	1.12 [1.05-1.19]	1.61 [1.55-1.68]	1.40 [1.36-1.45]	14,598	4,503	2,897	10,637	13,533
B35	1.56 [1.40-1.74]	1.63 [1.46-1.82]	1.62 [1.45-1.81]		1.61 [1.45-1.79]	1.46 [1.31-1.63]	1.49 [1.33-1.67]	1.50 [1.34-1.68]		1.52 [1.37-1.70]	1,152	1,122	1,049	NA	1,121
B36	1.76 [1.48-2.08]	1.74 [1.46-2.06]	1.61 [1.35-1.92]		1.63 [1.38-1.93]	1.64 [1.38-1.96]	1.58 [1.33-1.89]	1.49 [1.24-1.78]		1.58 [1.33-1.87]	473	460	431	NA	455
B37	1.27 [1.23-1.31]	1.27 [1.23-1.30]	1.29 [1.25-1.33]	0.98 [0.85-1.14]	1.22 [1.19-1.26]	1.19 [1.15-1.22]	1.15 [1.11-1.18]	1.17 [1.14-1.21]	0.94 [0.81-1.10]	1.16 [1.12-1.19]	13,890	13,960	12,039	586	13,510
B44	1.55 [1.33-1.79]	1.53 [1.32-1.77]	1.58 [1.36-1.85]		1.46 [1.26-1.69]	1.30 [1.12-1.52]	1.31 [1.12-1.53]	1.30 [1.11-1.53]		1.20 [1.02-1.40]	580	575	543	NA	572
B49	1.41 [1.16-1.73]	1.42 [1.16-1.74]	1.30 [1.06-1.60]		1.36 [1.12-1.66]	1.35 [1.10-1.65]	1.34 [1.09-1.65]	1.23 [0.99-1.52]		1.33 [1.09-1.62]	310	297	275	NA	295
B59	1.39 [1.06-1.81]	1.46 [1.12-1.90]	1.20 [0.91-1.56]		1.26 [0.97-1.64]	1.31 [1.00-1.72]	1.38 [1.05-1.81]	1.15 [0.87-1.51]		1.19 [0.90-1.56]	164	167	153	NA	162
B80	1.24 [0.99-1.57]	1.18 [0.91-1.53]			1.18 [0.94-1.50]	1.22 [0.96-1.55]	1.08 [0.82-1.42]			1.13 [0.89-1.44]	231	170	NA	NA	215
B85	1.20 [0.87-1.66]				1.29 [0.92-1.80]	1.17 [0.84-1.64]				1.29 [0.91-1.82]	114	NA	NA	NA	105
B86	2.66 [2.19-3.23]	2.59 [2.13-3.15]	2.52 [2.07-3.07]		2.42 [2.00-2.94]	2.64 [2.16-3.21]	2.48 [2.03-3.03]	2.41 [1.97-2.95]		2.47 [2.03-3.00]	416	398	384	NA	396
B90	1.56 [1.17-2.08]	1.35 [1.01-1.80]	1.42 [1.05-1.92]		1.36 [1.02-1.81]	1.45 [1.08-1.95]	1.25 [0.93-1.68]	1.33 [0.98-1.81]		1.28 [0.96-1.71]	147	147	135	NA	140
B91	1.21 [0.95-1.55]	1.11 [0.87-1.41]	1.17 [0.91-1.50]		1.07 [0.84-1.38]	1.24 [0.97-1.58]	1.13 [0.88-1.44]	1.17 [0.91-1.51]		1.09 [0.85-1.41]	195	192	192	NA	168
B94	1.17 [0.96-1.44]	1.21 [0.98-1.51]	1.15 [0.92-1.44]		1.12 [0.91-1.37]	1.07 [0.86-1.32]	1.08 [0.87-1.36]	1.08 [0.85-1.36]		1.05 [0.85-1.30]	278	251	224	NA	275
B95	1.37 [1.34-1.41]	1.37 [1.34-1.41]	1.37 [1.33-1.41]	1.33 [1.20-1.48]	1.34 [1.31-1.38]	1.33 [1.30-1.36]	1.29 [1.26-1.33]	1.30 [1.26-1.33]	1.30 [1.17-1.45]	1.32 [1.29-1.35]	19,482	18,791	16,505	1,326	18,546
B96	1.15 [1.13-1.17]	1.15 [1.13-1.18]	1.16 [1.13-1.18]	1.00 [0.90-1.10]	1.14 [1.12-1.16]	1.12 [1.10-1.14]	1.09 [1.07-1.11]	1.09 [1.07-1.11]	0.99 [0.90-1.10]	1.12 [1.10-1.14]	37,843	37,301	34,633	1,355	35,990
B97	1.20 [1.16-1.25]	1.17 [1.12-1.22]	1.17 [1.12-1.22]	1.23 [1.14-1.34]	1.17 [1.13-1.22]	1.13 [1.09-1.17]	1.07 [1.03-1.12]	1.07 [1.02-1.12]	1.19 [1.10-1.29]	1.12 [1.08-1.16]	9,079	7,386	6,260	1,997	8,738
B98	1.10 [1.04-1.16]	1.10 [1.04-1.16]	1.09 [1.03-1.16]		1.06 [1.00-1.12]	1.06 [1.00-1.12]	1.05 [0.99-1.11]	1.04 [0.98-1.11]		1.04 [0.98-1.10]	3,561	3,535	3,250	NA	3,313
B99	1.16 [1.05-1.30]	1.15 [1.03-1.28]	1.11 [0.99-1.24]		1.10 [0.99-1.22]	1.14 [1.02-1.27]	1.09 [0.97-1.21]	1.06 [0.94-1.18]		1.10 [0.98-1.22]	1,050	1,022	972	NA	1,024
Neoplasms (C00-D49)															
C00	1.03 [0.76-1.41]	1.26 [0.91-1.73]	1.07 [0.78-1.47]		1.11 [0.81-1.51]	1.03 [0.75-1.41]	1.26 [0.91-1.73]	1.04 [0.76-1.44]		1.10 [0.81-1.50]	110	111	106	NA	106
C01	1.03 [0.82-1.29]	1.07 [0.85-1.35]	1.04 [0.82-1.31]		1.12 [0.89-1.42]	1.01 [0.80-1.28]	1.07 [0.84-1.35]	1.05 [0.83-1.33]		1.17 [0.93-1.49]	207	202	199	NA	192
C02	1.27 [1.07-1.51]	1.29 [1.08-1.52]	1.33 [1.12-1.58]		1.38 [1.16-1.65]	1.26 [1.06-1.50]	1.23 [1.04-1.46]	1.30 [1.09-1.55]		1.40 [1.17-1.67]	396	397	380	NA	375
C04	1.07 [0.77-1.49]	1.12 [0.81-1.55]				1.07 [0.77-1.50]	1.04 [0.75-1.46]				101	102	NA	NA	NA
C05	1.29 [0.95-1.73]	1.42 [1.05-1.92]	1.46 [1.08-1.97]		1.14 [0.84-1.55]	1.30 [0.96-1.76]	1.38 [1.02-1.87]	1.42 [1.04-1.92]		1.17 [0.86-1.61]	132	134	128	NA	115
C06	1.15 [0.90-1.47]	1.19 [0.93-1.53]	1.16 [0.91-1.50]		1.24 [0.96-1.59]	1.14 [0.89-1.46]	1.15 [0.89-1.48]	1.09 [0.85-1.42]		1.22 [0.94-1.57]	180	178	175	NA	172
C07	0.95 [0.73-1.24]	0.95 [0.72-1.25]	0.93 [0.71-1.22]		0.96 [0.72-1.26]	0.94 [0.71-1.22]	0.92 [0.69-1.21]	0.91 [0.69-1.19]		0.94 [0.71-1.26]	149	150	144	NA	136
C09	1.01 [0.81-1.24]	1.00 [0.81-1.24]	1.05 [0.85-1.30]		0.96 [0.77-1.20]	0.99 [0.79-1.23]	0.97 [0.78-1.20]	1.02 [0.82-1.27]		0.98 [0.78-1.23]	238	234	233	NA	205
C10	1.21 [0.92-1.59]	1.17 [0.89-1.54]	1.17 [0.89-1.54]		1.12 [0.85-1.48]	1.19 [0.90-1.58]	1.12 [0.85-1.48]	1.12 [0.84-1.48]		1.16 [0.87-1.54]	145	143	140	NA	134
C14	1.02 [0.79-1.31]	1.01 [0.78-1.30]	0.99 [0.77-1.28]		1.02 [0.79-1.32]	1.02 [0.79-1.31]	0.98 [0.76-1.27]	0.96 [0.74-1.24]		1.05 [0.81-1.35]	165	165	167	NA	158
C15	1.14 [1.06-1.22]	1.14 [1.06-1.22]	1.13 [1.06-1.21]		1.10 [1.03-1.18]	1.14 [1.06-1.22]	1.11 [1.04-1.19]	1.11 [1.04-1.19]		1.10 [1.03-1.18]	2,425	2,422	2,427	NA	2,178
C16	1.04 [0.96-1.12]	1.00 [0.92-1.08]	1.02 [0.95-1.10]		1.04 [0.96-1.12]	1.03 [0.95-1.12]	0.98 [0.91-1.06]	1.00 [0.93-1.09]		1.03 [0.96-1.12]	1,850	1,863	1,848	NA	1,705
C17	1.07 [0.90-1.26]	1.07 [0.90-1.26]	1.04 [0.88-1.23]		1.07 [0.91-1.27]	1.06 [0.90-1.26]	1.03 [0.87-1.22]	1.03 [0.87-1.21]		1.06 [0.90-1.26]	401	399	398	NA	375
C18	1.00 [0.95-1.04]	1.00 [0.96-1.04]	1.01 [0.97-1.05]		1.00 [0.95-1.04]	0.99 [0.95-1.03]	0.99 [0.95-1.03]	1.00 [0.96-1.04]		0.99 [0.95-1.04]	6,291	6,266	6,194	NA	5,511
C19	0.92 [0.84-1.01]	0.95 [0.86-1.04]	0.96 [0.87-1.06]		0.96 [0.87-1.06]	0.93 [0.84-1.02]	0.96 [0.87-1.05]	0.97 [0.88-1.07]		0.97 [0.88-1.07]	1,210	1,195	1,190	NA	1,073
C20	0.93 [0.87-1.00]	0.96 [0.90-1.02]	0.94 [0.88-1.01]		0.94 [0.88-1.01]	0.94 [0.88-1.01]	0.97 [0.91-1.04]	0.96 [0.89-1.02]		0.96 [0.89-1.03]	2,422	2,423	2,391	NA	2,051
C21	1.03 [0.86-1.23]	1.05 [0.88-1.27]	1.01 [0.84-1.21]		0.96 [0.79-1.15]	1.03 [0.86-1.23]	1.04 [0.86-1.25]	0.97 [0.81-1.17]		0.95 [0.79-1.15]	328	327	319	NA	291
C22	1.22 [1.12-1.33]	1.19 [1.09-1.30]	1.19 [1.09-1.30]		1.14 [1.05-1.25]	1.19 [1.09-1.30]	1.15 [1.06-1.26]	1.16 [1.06-1.26]		1.14 [1.05-1.25]	1,536	1,531	1,519	NA	1,432
C23	1.01 [0.79-1.28]	1.05 [0.83-1.33]	0.98 [0.77-1.24]		1.02 [0.80-1.31]	1.01 [0.80-1.29]	1.04 [0.82-1.32]	0.98 [0.77-1.25]		1.05 [0.82-1.35]	191	188	184	NA	170
C24	1.08 [0.91-1.28]	1.04 [0.87-1.24]	1.02 [0.86-1.21]		1.05 [0.87-1.25]	1.09 [0.91-1.30]	1.03 [0.86-1.22]	0.99 [0.83-1.18]		1.04 [0.87-1.25]	360	360	356	NA	319
C25	1.12 [1.05-1.20]	1.08 [1.01-1.16]	1.10 [1.03-1.18]		1.11 [1.03-1.19]	1.11 [1.04-1.20]	1.06 [0.99-1.14]	1.08 [1.00-1.15]		1.11 [1.03-1.19]	2,317	2,322	2,310	NA	2,117
C26	1.08 [0.93-1.26]	1.06 [0.91-1.23]	1.11 [0.95-1.29]		1.01 [0.87-1.17]	1.07 [0.92-1.25]	1.02 [0.88-1.19]	1.08 [0.93-1.26]		0.99 [0.86-1.15]	538	535	526	NA	516
C32	1.09 [0.93-1.26]	1.08 [0.93-1.26]	1.08 [0.93-1.25]		1.08 [0.93-1.26]	1.09 [0.93-1.27]	1.07 [0.92-1.25]	1.06 [0.91-1.23]		1.10 [0.94-1.29]	468	465	467	NA	432
C34	1.12 [1.09-1.16]	1.11 [1.07-1.14]	1.12 [1.08-1.16]		1.09 [1.06-1.13]	1.12 [1.08-1.15]	1.06 [1.03-1.10]	1.07 [1.04-1.11]		1.10 [1.06-1.14]	9,955	9,936	9,945	NA	9,046
C38	1.03 [0.76-1.40]	0.93 [0.68-1.27]	0.97 [0.72-1.31]		0.95 [0.70-1.30]	1.05 [0.77-1.43]	0.92 [0.67-1.26]	0.96 [0.71-1.30]		0.96 [0.70-1.31]	119	115	113	NA	102
C41	1.27 [1.03-1.57]	1.20 [0.97-1.48]	1.18 [0.95-1.46]		1.38 [1.12-1.71]	1.29 [1.04-1.59]	1.19 [0.96-1.47]	1.17 [0.94-1.45]		1.40 [1.12-1.74]	264	261	241	NA	242
C43	0.92 [0.86-0.99]	0.91 [0.85-0.97]	0.89 [0.83-0.96]		0.95 [0.88-1.02]	0.92 [0.85-0.98]	0.90 [0.84-0.97]	0.89 [0.82-0.95]		0.95 [0.88-1.02]	2,218	2,224	2,043	NA	1,999
C44	1.07 [1.04-1.09]	1.07 [1.04-1.10]	1.07 [1.04-1.10]		1.04 [1.02-1.07]	1.05 [1.02-1.07]	1.04 [1.02-1.07]	1.04 [1.02-1.07]		1.03 [1.00-1.05]	18,993	19,024	18,771	NA	17,078
C45	0.96 [0.83-1.10]	0.97 [0.84-1.11]	1.02 [0.89-1.18]		1.01 [0.87-1.16]	0.96 [0.83-1.11]	0.98 [0.85-1.12]	1.03 [0.89-1.18]		1.02 [0.88-1.18]	535	536	542	NA	484
C48	0.96 [0.80-1.16]	1.02 [0.85-1.23]	1.02 [0.84-1.23]		1.00 [0.83-1.22]	0.95 [0.78-1.15]	1.01 [0.84-1.22]	1.00 [0.82-1.21]		0.99 [0.81-1.21]	298	292	287	NA	258
C49	1.04 [0.90-1.19]	1.08 [0.94-1.24]	1.11 [0.96-1.29]		1.11 [0.96-1.28]	1.02 [0.89-1.18]	1.06 [0.91-1.22]	1.08 [0.93-1.25]		1.09 [0.94-1.26]	555	552	506	NA	512
C50	1.03 [1.00-1.07]	1.02 [0.99-1.06]	1.03 [0.99-1.06]		1.02 [0.99-1.06]	1.03 [1.00-1.07]	1.02 [0.98-1.05]	1.02 [0.99-1.06]		1.03 [1.00-1.07]	10,383	10,406	9,875	NA	8,735
C51	1.12 [0.93-1.35]	1.17 [0.97-1.41]	1.19 [0.98-1.44]		1.21 [0.99-1.47]	1.11 [0.92-1.34]	1.14 [0.95-1.38]	1.17 [0.96-1.41]		1.19 [0.97-1.45]	318	316	307	NA	282
C53	0.99 [0.86-1.14]	1.03 [0.89-1.18]	1.04 [0.88-1.23]		1.01 [0.87-1.17]	1.00 [0.87-1.16]	1.03 [0.89-1.19]	1.03 [0.87-1.23]		1.04 [0.89-1.21]	531	540	365	NA	453
C54	1.05 [0.97-1.13]	1.04 [0.97-1.13]	1.03 [0.95-1.11]		1.05 [0.96-1.14]	1.06 [0.98-1.15]	1.07 [0.99-1.16]	1.05 [0.97-1.14]		1.07 [0.98-1.16]	1,784	1,784	1,764	NA	1,474
C55	1.11 [0.91-1.36]	1.01 [0.82-1.23]	1.03 [0.84-1.27]		0.95 [0.76-1.17]	1.09 [0.89-1.34]	0.99 [0.81-1.22]	1.02 [0.83-1.26]		0.95 [0.76-1.18]	252	251	249	NA	215
C56	1.03 [0.95-1.12]	1.03 [0.95-1.12]	1.02 [0.94-1.11]		1.00 [0.92-1.10]	1.04 [0.95-1.13]	1.03 [0.94-1.12]	1.02 [0.94-1.11]		1.01 [0.92-1.11]	1,503	1,516	1,429	NA	1,231
C57	1.12 [0.89-1.42]	1.23 [0.97-1.56]	1.30 [1.02-1.65]		1.17 [0.91-1.50]	1.16 [0.91-1.47]	1.25 [0.99-1.60]	1.33 [1.04-1.69]		1.18 [0.92-1.52]	192	193	190	NA	170
C60	1.12 [0.84-1.49]	1.21 [0.91-1.62]	1.16 [0.87-1.55]		1.20 [0.90-1.61]	1.14 [0.85-1.52]	1.22 [0.91-1.63]	1.21 [0.90-1.63]		1.27 [0.94-1.71]	136	138	132	NA	128
C61	1.03 [0.99-1.06]	1.01 [0.98-1.04]	1.01 [0.98-1.04]		0.99 [0.96-1.02]	1.01 [0.98-1.05]	1.00 [0.96-1.03]	1.00 [0.96-1.03]		0.99 [0.95-1.02]	10,385	10,370	10,387	NA	9,295
C62	0.95 [0.70-1.28]	1.02 [0.75-1.40]			0.86 [0.62-1.20]	0.95 [0.70-1.30]	1.03 [0.74-1.43]			0.88 [0.63-1.23]	135	131	NA	NA	105
C64	1.04 [0.97-1.11]	1.03 [0.96-1.10]	1.03 [0.96-1.10]		1.03 [0.96-1.11]	1.03 [0.96-1.11]	1.00 [0.93-1.07]	1.00 [0.93-1.07]		1.04 [0.96-1.11]	2,215	2,207	2,184	NA	2,005
C65	1.26 [1.00-1.59]	1.24 [0.99-1.56]	1.11 [0.88-1.39]		1.16 [0.92-1.46]	1.29 [1.02-1.62]	1.24 [0.99-1.55]	1.12 [0.89-1.41]		1.21 [0.96-1.54]	226	226	219	NA	192
C66	1.10 [0.90-1.34]	1.03 [0.85-1.25]	1.00 [0.82-1.21]		1.03 [0.84-1.24]	1.09 [0.89-1.34]	1.01 [0.83-1.23]	0.98 [0.80-1.19]		1.03 [0.85-1.26]	278	279	277	NA	266
C67	1.03 [0.98-1.08]	1.02 [0.98-1.07]	1.02 [0.97-1.07]		1.01 [0.96-1.06]	1.03 [0.98-1.08]	1.01 [0.96-1.06]	1.01 [0.96-1.06]		1.01 [0.96-1.06]	4,864	4,851	4,838	NA	4,344
C69	1.00 [0.78-1.28]	0.91 [0.71-1.17]	1.07 [0.83-1.38]		0.93 [0.72-1.21]	1.00 [0.77-1.28]	0.90 [0.70-1.15]	1.04 [0.80-1.34]		0.94 [0.73-1.23]	172	166	160	NA	156
C71	1.11 [1.00-1.22]	1.10 [0.99-1.22]	1.10 [0.99-1.22]		1.10 [0.99-1.23]	1.11 [1.00-1.23]	1.08 [0.97-1.20]	1.08 [0.97-1.20]		1.10 [0.98-1.23]	1,062	1,018	961	NA	908
C73	0.97 [0.85-1.12]	0.98 [0.85-1.12]	1.04 [0.89-1.21]		0.99 [0.86-1.15]	0.97 [0.84-1.12]	0.96 [0.83-1.10]	1.03 [0.88-1.21]		0.98 [0.84-1.13]	562	577	448	NA	509
C76	1.08 [0.90-1.30]	1.07 [0.89-1.30]	1.06 [0.87-1.28]		1.06 [0.88-1.27]	1.09 [0.91-1.32]	1.09 [0.90-1.32]	1.08 [0.89-1.31]		1.07 [0.89-1.29]	320	308	297	NA	306
C77	1.04 [1.01-1.07]	1.04 [1.00-1.07]	1.05 [1.01-1.08]		1.03 [1.00-1.07]	1.04 [1.01-1.08]	1.03 [1.00-1.06]	1.04 [1.01-1.08]		1.05 [1.01-1.08]	10,224	10,231	9,817	NA	9,166
C78	1.04 [1.02-1.07]	1.03 [1.00-1.06]	1.04 [1.01-1.07]		1.04 [1.01-1.07]	1.05 [1.02-1.08]	1.02 [0.99-1.04]	1.03 [1.00-1.06]		1.06 [1.03-1.09]	15,762	15,708	15,524	NA	14,573
C79	1.05 [1.02-1.08]	1.04 [1.01-1.07]	1.05 [1.02-1.09]		1.04 [1.01-1.07]	1.06 [1.03-1.09]	1.03 [1.00-1.06]	1.04 [1.01-1.07]		1.06 [1.03-1.09]	13,276	13,254	13,086	NA	12,441
C80	1.07 [1.02-1.12]	1.05 [1.00-1.11]	1.07 [1.02-1.12]		1.08 [1.02-1.13]	1.07 [1.01-1.12]	1.03 [0.98-1.08]	1.05 [1.00-1.10]		1.08 [1.03-1.14]	4,746	4,731	4,704	NA	4,291
C81	1.51 [1.30-1.77]	1.46 [1.25-1.71]	1.57 [1.32-1.87]		1.28 [1.09-1.52]	1.47 [1.26-1.72]	1.43 [1.22-1.67]	1.49 [1.25-1.78]		1.26 [1.07-1.50]	537	523	410	NA	420
C82	1.14 [1.00-1.31]	1.11 [0.97-1.28]	1.09 [0.95-1.25]		1.07 [0.93-1.23]	1.11 [0.97-1.28]	1.08 [0.94-1.24]	1.06 [0.92-1.21]		1.04 [0.90-1.20]	573	575	557	NA	505
C83	1.18 [1.09-1.28]	1.20 [1.11-1.30]	1.20 [1.11-1.30]		1.15 [1.06-1.25]	1.16 [1.07-1.25]	1.18 [1.09-1.28]	1.18 [1.09-1.28]		1.13 [1.04-1.23]	1,731	1,726	1,678	NA	1,567
C84	2.29 [1.87-2.81]	2.57 [2.09-3.16]	2.26 [1.83-2.77]		2.13 [1.74-2.61]	2.27 [1.85-2.79]	2.52 [2.05-3.11]	2.22 [1.80-2.73]		2.14 [1.74-2.63]	361	356	337	NA	320
C85	1.24 [1.16-1.33]	1.23 [1.14-1.32]	1.24 [1.16-1.34]		1.19 [1.11-1.28]	1.21 [1.12-1.30]	1.19 [1.11-1.28]	1.20 [1.11-1.29]		1.17 [1.09-1.26]	2,245	2,223	2,151	NA	2,026
C88	1.25 [1.03-1.52]	1.23 [1.01-1.50]	1.26 [1.03-1.54]		1.14 [0.93-1.39]	1.25 [1.02-1.52]	1.22 [1.00-1.48]	1.22 [1.00-1.50]		1.11 [0.91-1.36]	296	297	290	NA	278
C90	1.11 [1.02-1.21]	1.12 [1.03-1.22]	1.10 [1.01-1.20]		1.09 [1.00-1.19]	1.09 [1.00-1.19]	1.11 [1.02-1.21]	1.09 [1.00-1.19]		1.08 [0.98-1.18]	1,521	1,527	1,517	NA	1,390
C91	1.04 [0.95-1.13]	1.06 [0.97-1.16]	1.03 [0.94-1.13]		1.01 [0.93-1.11]	1.02 [0.93-1.12]	1.04 [0.95-1.13]	1.01 [0.92-1.11]		1.00 [0.92-1.10]	1,411	1,356	1,334	NA	1,247
C92	1.23 [1.12-1.36]	1.25 [1.13-1.37]	1.27 [1.15-1.40]		1.22 [1.11-1.35]	1.22 [1.11-1.34]	1.23 [1.11-1.35]	1.25 [1.14-1.39]		1.21 [1.10-1.34]	1,236	1,235	1,191	NA	1,137
C93	1.09 [0.84-1.42]	1.05 [0.81-1.36]	1.06 [0.82-1.37]		1.03 [0.80-1.34]	1.10 [0.85-1.43]	1.02 [0.78-1.32]	1.03 [0.80-1.34]		1.04 [0.80-1.35]	173	171	172	NA	162
C95	1.26 [1.03-1.55]	1.20 [0.97-1.48]	1.22 [0.99-1.52]		1.19 [0.98-1.46]	1.26 [1.02-1.54]	1.15 [0.93-1.42]	1.17 [0.94-1.46]		1.19 [0.97-1.46]	280	273	265	NA	273
C97	1.03 [0.93-1.13]	1.06 [0.96-1.17]	1.02 [0.92-1.12]		1.04 [0.95-1.14]	1.01 [0.92-1.11]	1.03 [0.93-1.13]	0.99 [0.90-1.09]		1.03 [0.94-1.13]	1,250	1,261	1,232	NA	1,249
D00	0.93 [0.70-1.23]	0.97 [0.72-1.29]	0.89 [0.67-1.19]		1.04 [0.78-1.39]	0.91 [0.68-1.22]	0.93 [0.69-1.25]	0.84 [0.63-1.13]		1.03 [0.77-1.37]	125	123	119	NA	125
D01	1.01 [0.76-1.35]	1.08 [0.81-1.45]	1.01 [0.75-1.35]		0.98 [0.74-1.30]	0.98 [0.74-1.31]	1.04 [0.77-1.39]	0.94 [0.69-1.26]		0.94 [0.71-1.25]	132	129	120	NA	127
D02	1.02 [0.77-1.34]	1.09 [0.82-1.45]	1.05 [0.80-1.39]		1.03 [0.78-1.37]	0.98 [0.74-1.30]	1.05 [0.79-1.40]	1.01 [0.76-1.34]		1.02 [0.77-1.36]	144	146	142	NA	131
D03	1.03 [0.93-1.15]	1.04 [0.93-1.15]	1.03 [0.92-1.15]		1.03 [0.92-1.15]	1.02 [0.92-1.14]	1.01 [0.91-1.13]	1.01 [0.90-1.12]		1.01 [0.90-1.13]	927	937	893	NA	854
D04	1.18 [1.10-1.27]	1.19 [1.11-1.28]	1.20 [1.12-1.29]		1.21 [1.13-1.30]	1.14 [1.06-1.22]	1.13 [1.06-1.22]	1.13 [1.06-1.22]		1.17 [1.09-1.25]	2,461	2,451	2,425	NA	2,348
D05	0.99 [0.91-1.07]	0.98 [0.90-1.06]	0.97 [0.89-1.06]		0.94 [0.86-1.03]	0.98 [0.90-1.07]	0.97 [0.89-1.05]	0.96 [0.88-1.05]		0.94 [0.86-1.03]	1,529	1,546	1,469	NA	1,310
D06	1.02 [0.93-1.12]	1.02 [0.93-1.11]	0.98 [0.81-1.18]	0.79 [0.59-1.04]	0.96 [0.87-1.05]	1.01 [0.92-1.11]	0.98 [0.89-1.07]	0.96 [0.79-1.16]	0.77 [0.57-1.05]	0.97 [0.88-1.06]	1,411	1,492	288	154	1,211
D07	1.12 [0.97-1.28]	1.19 [1.04-1.38]	1.14 [0.99-1.32]		1.10 [0.96-1.27]	1.07 [0.93-1.23]	1.13 [0.98-1.31]	1.10 [0.94-1.27]		1.07 [0.93-1.24]	559	559	506	NA	522
D09	1.01 [0.87-1.16]	1.04 [0.90-1.20]	1.02 [0.89-1.18]		0.98 [0.85-1.13]	0.99 [0.86-1.15]	1.01 [0.87-1.17]	1.01 [0.88-1.17]		0.98 [0.85-1.13]	523	534	525	NA	519
D10	1.17 [1.00-1.37]	1.20 [1.03-1.40]	1.26 [1.06-1.50]		1.18 [1.00-1.38]	1.12 [0.96-1.31]	1.09 [0.93-1.28]	1.14 [0.95-1.36]		1.12 [0.95-1.32]	489	489	382	NA	439
D11	1.04 [0.91-1.20]	1.00 [0.87-1.15]	1.03 [0.89-1.19]		1.09 [0.94-1.26]	1.04 [0.90-1.20]	0.97 [0.84-1.12]	0.99 [0.86-1.15]		1.09 [0.94-1.26]	553	554	501	NA	489
D12	1.13 [1.10-1.15]	1.12 [1.10-1.15]	1.13 [1.10-1.16]		1.10 [1.08-1.13]	1.07 [1.05-1.10]	1.06 [1.03-1.09]	1.06 [1.04-1.09]		1.07 [1.04-1.09]	18,892	18,874	18,493	NA	17,428
D13	1.13 [1.03-1.24]	1.14 [1.04-1.25]	1.14 [1.04-1.26]		1.10 [1.00-1.21]	1.06 [0.97-1.17]	1.05 [0.96-1.16]	1.05 [0.96-1.16]		1.06 [0.96-1.16]	1,308	1,300	1,229	NA	1,191
D14	1.21 [1.02-1.45]	1.09 [0.91-1.30]	1.21 [1.00-1.47]		1.17 [0.97-1.40]	1.15 [0.96-1.38]	0.99 [0.82-1.19]	1.15 [0.94-1.40]		1.14 [0.94-1.37]	375	355	303	NA	333
D15	1.05 [0.79-1.40]	1.04 [0.78-1.37]	1.02 [0.77-1.35]		1.01 [0.76-1.35]	1.06 [0.79-1.41]	1.02 [0.77-1.35]	1.01 [0.76-1.35]		1.01 [0.75-1.35]	140	139	125	NA	125
D16	1.12 [0.94-1.32]	1.17 [0.98-1.39]	1.05 [0.86-1.29]		1.01 [0.85-1.20]	1.11 [0.93-1.32]	1.12 [0.93-1.34]	1.01 [0.82-1.24]		0.99 [0.83-1.18]	427	376	263	NA	371
D17	1.07 [1.02-1.12]	1.06 [1.01-1.11]	1.07 [1.02-1.13]	1.01 [0.73-1.38]	1.03 [0.98-1.09]	1.03 [0.98-1.08]	1.01 [0.96-1.06]	1.03 [0.98-1.08]	0.94 [0.68-1.30]	1.00 [0.95-1.05]	4,816	4,804	4,102	139	4,278
D18	1.10 [1.03-1.18]	1.10 [1.02-1.18]	1.15 [1.06-1.24]	1.02 [0.80-1.32]	1.06 [0.99-1.14]	1.05 [0.98-1.13]	1.03 [0.96-1.11]	1.06 [0.98-1.15]	1.02 [0.79-1.32]	1.02 [0.95-1.10]	2,318	2,180	1,893	203	2,182
D21	1.23 [1.07-1.42]	1.24 [1.08-1.43]	1.24 [1.07-1.44]		1.19 [1.03-1.38]	1.20 [1.04-1.38]	1.17 [1.02-1.35]	1.18 [1.02-1.38]		1.14 [0.98-1.32]	599	589	509	NA	527
D22	1.07 [1.02-1.13]	1.08 [1.03-1.14]	1.05 [0.98-1.12]	1.10 [0.95-1.28]	1.05 [0.99-1.10]	1.04 [0.99-1.10]	1.04 [0.99-1.10]	1.01 [0.94-1.08]	1.08 [0.92-1.26]	1.03 [0.97-1.08]	4,336	4,215	2,454	578	3,746
D23	1.12 [1.05-1.18]	1.16 [1.09-1.24]	1.16 [1.09-1.24]	1.05 [0.85-1.29]	1.12 [1.06-1.20]	1.07 [1.01-1.14]	1.11 [1.05-1.19]	1.11 [1.03-1.19]	1.02 [0.82-1.26]	1.09 [1.02-1.16]	3,067	2,866	2,409	291	2,680
D24	1.02 [0.94-1.12]	1.00 [0.92-1.08]	1.04 [0.93-1.16]	1.00 [0.76-1.30]	0.99 [0.91-1.09]	1.00 [0.91-1.09]	0.95 [0.87-1.04]	1.00 [0.90-1.12]	0.98 [0.74-1.31]	0.97 [0.89-1.07]	1,501	1,591	881	175	1,283
D25	1.02 [0.99-1.06]	1.05 [1.01-1.09]	1.04 [1.00-1.08]		1.04 [1.00-1.08]	1.01 [0.97-1.05]	1.04 [1.00-1.08]	1.02 [0.98-1.06]		1.03 [0.99-1.07]	8,252	8,312	6,739	NA	6,943
D26	1.05 [0.86-1.28]	1.12 [0.92-1.36]	0.97 [0.78-1.21]		1.01 [0.82-1.24]	1.03 [0.84-1.26]	1.10 [0.90-1.34]	0.95 [0.75-1.18]		0.98 [0.80-1.21]	285	290	211	NA	245
D27	1.00 [0.93-1.08]	1.01 [0.94-1.09]	1.00 [0.91-1.09]	0.97 [0.70-1.36]	0.99 [0.91-1.07]	0.99 [0.92-1.07]	0.99 [0.91-1.06]	0.97 [0.89-1.06]	0.97 [0.68-1.38]	0.98 [0.90-1.06]	1,914	1,975	1,296	116	1,619
D28	1.20 [0.96-1.50]	1.18 [0.94-1.47]	1.16 [0.89-1.52]		1.24 [0.98-1.56]	1.15 [0.91-1.44]	1.14 [0.91-1.42]	1.16 [0.88-1.53]		1.17 [0.92-1.49]	237	234	154	NA	202
D29	0.97 [0.74-1.28]	1.04 [0.79-1.37]	0.99 [0.74-1.31]		1.04 [0.79-1.36]	0.96 [0.73-1.27]	1.03 [0.78-1.37]	0.98 [0.74-1.30]		1.04 [0.79-1.37]	150	147	140	NA	144
D30	0.99 [0.83-1.19]	0.98 [0.82-1.17]	0.93 [0.77-1.11]		0.96 [0.80-1.15]	0.97 [0.81-1.17]	0.95 [0.79-1.14]	0.91 [0.75-1.09]		0.95 [0.80-1.14]	330	329	306	NA	316
D31	1.13 [0.94-1.35]	1.13 [0.94-1.37]	1.11 [0.91-1.36]		1.16 [0.96-1.40]	1.12 [0.93-1.35]	1.09 [0.90-1.33]	1.08 [0.88-1.33]		1.15 [0.95-1.39]	346	321	288	NA	321

Supplementary Table 4: Hazard ratios and events from all cohorts (excluding non-consulters)

	Hazard ratio (99% confidence interval)														
	crude					adjusted					Events (in exposed)				
Outcome	any age	18+	40+	<18	hosp.	any age	18+	40+	<18	hosp.	any age	18+	40+	<18	hosp.
D32	1.08 [0.98-1.18]	1.04 [0.95-1.14]	1.09 [0.99-1.20]		1.07 [0.97-1.17]	1.05 [0.96-1.15]	1.00 [0.91-1.10]	1.04 [0.95-1.15]		1.06 [0.96-1.16]	1,362	1,355	1,300	NA	1,259
D33	0.95 [0.81-1.12]	1.00 [0.85-1.17]	1.04 [0.89-1.23]		0.96 [0.82-1.13]	0.90 [0.76-1.05]	0.93 [0.79-1.09]	0.98 [0.83-1.16]		0.93 [0.79-1.10]	440	434	397	NA	408
D34	1.02 [0.84-1.24]	0.95 [0.79-1.16]	0.92 [0.75-1.14]		0.98 [0.80-1.19]	0.98 [0.81-1.20]	0.91 [0.75-1.11]	0.89 [0.72-1.11]		0.93 [0.76-1.14]	293	295	232	NA	265
D35	1.05 [0.97-1.13]	1.01 [0.94-1.09]	1.03 [0.95-1.11]		1.00 [0.92-1.08]	1.02 [0.94-1.10]	0.97 [0.90-1.05]	0.99 [0.91-1.07]		0.98 [0.91-1.06]	1,885	1,872	1,698	NA	1,715
D36	1.16 [1.02-1.30]	1.16 [1.02-1.31]	1.20 [1.05-1.38]		1.14 [1.01-1.29]	1.09 [0.97-1.24]	1.07 [0.95-1.22]	1.11 [0.97-1.28]		1.08 [0.95-1.23]	783	764	631	NA	709
D37	1.03 [0.97-1.10]	1.05 [0.98-1.12]	1.04 [0.97-1.11]		1.08 [1.01-1.15]	1.02 [0.95-1.09]	1.03 [0.96-1.10]	1.01 [0.94-1.08]		1.08 [1.00-1.15]	2,430	2,436	2,352	NA	2,221
D38	1.17 [1.02-1.34]	1.11 [0.97-1.27]	1.16 [1.01-1.32]		1.14 [1.00-1.31]	1.16 [1.01-1.33]	1.07 [0.94-1.23]	1.12 [0.97-1.28]		1.15 [1.00-1.32]	603	608	590	NA	549
D39	1.06 [0.87-1.28]	1.02 [0.84-1.24]	0.99 [0.80-1.22]		0.99 [0.81-1.21]	1.06 [0.88-1.29]	1.01 [0.83-1.23]	0.96 [0.78-1.19]		1.00 [0.81-1.22]	291	301	235	NA	253
D41	1.06 [0.98-1.14]	1.04 [0.96-1.13]	1.02 [0.95-1.11]		0.99 [0.92-1.08]	1.05 [0.97-1.14]	1.02 [0.94-1.11]	1.00 [0.92-1.08]		1.00 [0.92-1.08]	1,747	1,762	1,725	NA	1,570
D43	1.09 [0.94-1.26]	1.04 [0.89-1.20]	1.00 [0.86-1.16]		0.98 [0.84-1.14]	1.08 [0.93-1.25]	1.02 [0.88-1.19]	0.97 [0.83-1.13]		0.98 [0.84-1.14]	520	499	455	NA	462
D44	1.13 [0.94-1.34]	1.09 [0.91-1.30]	1.19 [0.99-1.44]		1.08 [0.90-1.29]	1.06 [0.89-1.27]	1.02 [0.85-1.23]	1.13 [0.93-1.37]		1.06 [0.89-1.27]	355	347	313	NA	326
D45	0.96 [0.84-1.10]	0.98 [0.86-1.13]	0.95 [0.83-1.09]		0.97 [0.85-1.12]	0.96 [0.83-1.10]	0.94 [0.82-1.08]	0.91 [0.79-1.05]		0.97 [0.84-1.11]	576	578	549	NA	526
D46	1.22 [1.13-1.33]	1.28 [1.18-1.39]	1.25 [1.15-1.35]		1.21 [1.12-1.32]	1.20 [1.10-1.30]	1.24 [1.14-1.34]	1.21 [1.11-1.31]		1.20 [1.10-1.30]	1,810	1,813	1,802	NA	1,715
D47	1.20 [1.12-1.27]	1.21 [1.14-1.29]	1.21 [1.13-1.29]		1.18 [1.11-1.25]	1.15 [1.08-1.23]	1.14 [1.07-1.22]	1.15 [1.07-1.22]		1.14 [1.07-1.22]	2,913	2,936	2,826	NA	2,857
D48	1.01 [0.91-1.12]	1.03 [0.93-1.14]	1.03 [0.92-1.15]		1.00 [0.90-1.11]	0.98 [0.89-1.09]	1.00 [0.90-1.11]	1.00 [0.89-1.11]		0.98 [0.88-1.09]	1,068	1,062	927	NA	965
C03		1.50 [1.06-2.13]					1.50 [1.06-2.13]				NA	103	NA	NA	NA
Diseases of the blood and blood-forming organs and certain disorders involving the immune mechanism (D50-D89)															
D50	1.22 [1.20-1.24]	1.21 [1.19-1.24]	1.22 [1.20-1.24]	1.15 [1.03-1.29]	1.19 [1.17-1.21]	1.17 [1.15-1.19]	1.13 [1.11-1.16]	1.13 [1.11-1.15]	1.13 [1.01-1.27]	1.15 [1.13-1.17]	39,867	39,616	36,296	1,149	38,121
D51	1.25 [1.18-1.33]	1.22 [1.15-1.29]	1.25 [1.18-1.32]		1.22 [1.16-1.30]	1.19 [1.12-1.26]	1.13 [1.07-1.20]	1.17 [1.10-1.24]		1.18 [1.11-1.25]	3,681	3,708	3,427	NA	3,492
D52	1.20 [1.12-1.29]	1.24 [1.16-1.33]	1.25 [1.16-1.34]		1.23 [1.15-1.31]	1.17 [1.10-1.26]	1.18 [1.10-1.26]	1.19 [1.11-1.27]		1.22 [1.14-1.31]	2,700	2,724	2,620	NA	2,664
D53	1.31 [1.15-1.48]	1.31 [1.16-1.48]	1.41 [1.24-1.61]		1.29 [1.14-1.47]	1.24 [1.09-1.41]	1.22 [1.07-1.38]	1.29 [1.14-1.48]		1.26 [1.11-1.43]	801	801	780	NA	774
D56	1.22 [1.11-1.34]	1.20 [1.09-1.32]	1.32 [1.18-1.49]	1.04 [0.77-1.40]	1.19 [1.08-1.30]	1.21 [1.10-1.33]	1.19 [1.08-1.31]	1.25 [1.11-1.42]	1.03 [0.75-1.41]	1.18 [1.07-1.31]	1,404	1,350	846	151	1,257
D57	1.15 [1.04-1.27]	1.15 [1.03-1.27]	1.06 [0.93-1.22]	1.41 [1.12-1.78]	1.12 [1.01-1.24]	1.15 [1.04-1.27]	1.16 [1.04-1.29]	1.05 [0.92-1.21]	1.50 [1.17-1.92]	1.13 [1.02-1.25]	1,281	1,115	625	278	1,122
D58	1.05 [0.87-1.26]	1.05 [0.86-1.27]	1.09 [0.89-1.35]		1.15 [0.95-1.39]	1.06 [0.87-1.28]	1.03 [0.85-1.26]	1.08 [0.87-1.34]		1.16 [0.96-1.41]	328	307	248	NA	311
D59	1.29 [1.10-1.51]	1.30 [1.10-1.52]	1.29 [1.09-1.52]		1.30 [1.11-1.52]	1.27 [1.09-1.49]	1.29 [1.09-1.52]	1.27 [1.07-1.50]		1.31 [1.11-1.54]	470	455	417	NA	459
D61	1.21 [1.12-1.30]	1.23 [1.14-1.32]	1.21 [1.12-1.30]		1.20 [1.12-1.29]	1.18 [1.10-1.27]	1.18 [1.10-1.27]	1.16 [1.08-1.25]		1.20 [1.11-1.28]	2,343	2,320	2,209	NA	2,302
D62	1.04 [0.93-1.16]	1.04 [0.94-1.16]	1.09 [0.94-1.27]		1.03 [0.92-1.15]	1.06 [0.95-1.18]	1.03 [0.93-1.15]	1.07 [0.92-1.24]		1.04 [0.93-1.17]	977	1,021	518	NA	870
D63	1.19 [1.13-1.26]	1.22 [1.15-1.29]	1.16 [1.10-1.23]		1.13 [1.07-1.20]	1.17 [1.11-1.24]	1.17 [1.10-1.23]	1.11 [1.05-1.17]		1.13 [1.07-1.20]	3,874	3,858	3,727	NA	3,695
D64	1.21 [1.19-1.22]	1.20 [1.19-1.22]	1.21 [1.19-1.23]	1.00 [0.89-1.13]	1.18 [1.16-1.19]	1.17 [1.15-1.19]	1.14 [1.12-1.16]	1.15 [1.13-1.17]	0.99 [0.87-1.11]	1.16 [1.14-1.17]	53,146	53,064	49,352	1,024	50,319
D65	1.21 [0.93-1.57]	1.28 [0.97-1.69]	1.15 [0.86-1.54]		1.36 [1.03-1.78]	1.21 [0.93-1.58]	1.27 [0.96-1.68]	1.13 [0.84-1.52]		1.36 [1.04-1.79]	158	152	136	NA	147
D66	1.04 [0.78-1.39]	1.03 [0.76-1.38]			1.24 [0.92-1.67]	1.01 [0.75-1.36]	0.96 [0.71-1.31]			1.22 [0.90-1.67]	134	125	NA	NA	124
D68	1.21 [1.13-1.29]	1.21 [1.13-1.29]	1.23 [1.14-1.33]	0.92 [0.70-1.20]	1.16 [1.09-1.24]	1.13 [1.06-1.21]	1.11 [1.03-1.19]	1.12 [1.03-1.21]	0.88 [0.66-1.17]	1.12 [1.04-1.19]	2,654	2,617	1,958	179	2,511
D69	1.18 [1.14-1.23]	1.16 [1.11-1.21]	1.20 [1.15-1.26]	0.98 [0.85-1.14]	1.15 [1.10-1.20]	1.16 [1.11-1.20]	1.12 [1.08-1.17]	1.16 [1.11-1.21]	1.00 [0.86-1.16]	1.14 [1.10-1.19]	7,552	7,088	6,388	615	7,171
D70	1.17 [1.12-1.22]	1.16 [1.12-1.22]	1.18 [1.13-1.23]	1.09 [0.89-1.35]	1.14 [1.09-1.19]	1.15 [1.10-1.20]	1.14 [1.09-1.19]	1.16 [1.11-1.21]	1.07 [0.86-1.32]	1.13 [1.09-1.18]	6,386	6,187	5,705	313	5,917
D72	1.28 [1.17-1.39]	1.30 [1.19-1.42]	1.26 [1.15-1.38]	1.53 [1.03-2.27]	1.28 [1.18-1.40]	1.18 [1.08-1.29]	1.18 [1.08-1.30]	1.17 [1.06-1.28]	1.38 [0.91-2.09]	1.21 [1.11-1.32]	1,675	1,643	1,446	101	1,633
D73	1.14 [1.04-1.24]	1.14 [1.04-1.24]	1.13 [1.03-1.23]		1.12 [1.02-1.22]	1.10 [1.01-1.20]	1.08 [0.99-1.18]	1.07 [0.97-1.17]		1.10 [1.01-1.20]	1,545	1,531	1,426	NA	1,495
D75	1.13 [1.04-1.22]	1.12 [1.04-1.21]	1.12 [1.04-1.22]		1.10 [1.02-1.19]	1.10 [1.02-1.19]	1.06 [0.98-1.15]	1.07 [0.99-1.16]		1.09 [1.01-1.18]	1,964	1,954	1,841	NA	1,863
D76	1.26 [0.90-1.77]					1.18 [0.83-1.68]					101	NA	NA	NA	NA
D80	1.27 [1.08-1.49]	1.31 [1.11-1.55]	1.20 [1.01-1.43]		1.29 [1.10-1.51]	1.15 [0.97-1.36]	1.17 [0.98-1.39]	1.04 [0.87-1.25]		1.17 [1.00-1.37]	466	425	374	NA	470
D84	1.36 [1.09-1.70]	1.45 [1.13-1.85]	1.51 [1.16-1.97]		1.46 [1.18-1.82]	1.17 [0.93-1.48]	1.25 [0.96-1.62]	1.21 [0.91-1.62]		1.25 [0.99-1.57]	263	217	176	NA	255
D86	1.35 [1.21-1.50]	1.35 [1.22-1.50]	1.38 [1.24-1.54]		1.31 [1.18-1.45]	1.27 [1.14-1.41]	1.26 [1.13-1.41]	1.28 [1.15-1.43]		1.25 [1.12-1.39]	1,104	1,100	1,006	NA	1,041
D89	1.34 [1.20-1.50]	1.28 [1.14-1.44]	1.32 [1.17-1.48]		1.25 [1.12-1.40]	1.26 [1.12-1.41]	1.21 [1.07-1.35]	1.22 [1.08-1.37]		1.20 [1.07-1.35]	939	919	870	NA	878
Endocrine, nutritional and metabolic diseases (E00-E89)															
E02	1.11 [0.91-1.36]	1.17 [0.96-1.43]	1.10 [0.89-1.35]		1.14 [0.93-1.39]	1.10 [0.90-1.35]	1.14 [0.93-1.41]	1.08 [0.87-1.33]		1.14 [0.93-1.40]	300	306	281	NA	292
E03	1.20 [1.18-1.23]	1.20 [1.18-1.22]	1.20 [1.18-1.22]	0.88 [0.76-1.04]	1.18 [1.16-1.20]	1.17 [1.15-1.19]	1.15 [1.13-1.17]	1.15 [1.13-1.18]	0.91 [0.77-1.07]	1.16 [1.13-1.18]	38,325	38,453	34,872	506	33,143
E04	1.06 [1.00-1.12]	1.04 [0.98-1.10]	1.02 [0.96-1.09]		1.04 [0.98-1.10]	1.02 [0.96-1.08]	0.99 [0.93-1.05]	0.97 [0.91-1.03]		1.02 [0.96-1.08]	3,405	3,385	3,022	NA	3,186
E05	1.15 [1.10-1.20]	1.14 [1.09-1.19]	1.14 [1.08-1.19]	0.97 [0.71-1.32]	1.12 [1.07-1.17]	1.11 [1.06-1.16]	1.08 [1.03-1.13]	1.07 [1.02-1.12]	0.98 [0.71-1.36]	1.09 [1.04-1.14]	6,227	6,290	5,358	137	5,817
E06	1.14 [0.99-1.30]	1.13 [0.99-1.30]	1.16 [0.99-1.35]		1.19 [1.04-1.36]	1.10 [0.96-1.27]	1.07 [0.93-1.23]	1.10 [0.94-1.29]		1.14 [0.99-1.31]	636	642	478	NA	616
E07	1.17 [1.06-1.30]	1.19 [1.07-1.32]	1.15 [1.03-1.29]		1.15 [1.03-1.27]	1.11 [1.00-1.23]	1.11 [1.00-1.23]	1.08 [0.97-1.21]		1.09 [0.98-1.21]	1,083	1,084	963	NA	1,031
E10	1.06 [1.02-1.10]	1.07 [1.03-1.11]	1.10 [1.05-1.14]	0.77 [0.64-0.92]	1.07 [1.02-1.11]	1.01 [0.97-1.05]	0.97 [0.94-1.02]	1.00 [0.95-1.04]	0.77 [0.64-0.92]	1.05 [1.00-1.09]	7,209	6,882	6,311	419	6,308
E11	1.08 [1.06-1.09]	1.08 [1.06-1.09]	1.07 [1.06-1.09]	0.78 [0.60-1.03]	1.09 [1.07-1.10]	1.04 [1.03-1.06]	1.01 [0.99-1.02]	1.00 [0.99-1.02]	0.82 [0.61-1.09]	1.07 [1.05-1.09]	60,214	60,130	58,773	189	49,844
E13	1.27 [1.12-1.44]	1.19 [1.05-1.35]	1.24 [1.09-1.41]		1.23 [1.09-1.39]	1.14 [1.01-1.30]	1.04 [0.92-1.19]	1.08 [0.94-1.23]		1.13 [1.00-1.29]	766	765	691	NA	750
E14	1.17 [1.13-1.22]	1.18 [1.14-1.22]	1.18 [1.14-1.22]		1.16 [1.12-1.21]	1.11 [1.07-1.15]	1.07 [1.03-1.11]	1.06 [1.02-1.10]		1.12 [1.08-1.16]	9,171	9,150	8,778	NA	8,648
E16	1.14 [1.10-1.18]	1.12 [1.08-1.16]	1.12 [1.08-1.16]	0.95 [0.80-1.11]	1.09 [1.05-1.13]	1.09 [1.05-1.13]	1.02 [0.99-1.06]	1.02 [0.99-1.06]	0.96 [0.81-1.13]	1.08 [1.04-1.11]	10,080	9,757	9,237	449	9,884
E20	1.30 [1.06-1.60]	1.27 [1.03-1.56]	1.39 [1.12-1.72]		1.31 [1.07-1.60]	1.18 [0.96-1.47]	1.16 [0.93-1.43]	1.26 [1.01-1.58]		1.23 [1.00-1.51]	286	284	263	NA	286
E21	1.06 [1.00-1.13]	1.05 [0.99-1.12]	1.06 [0.99-1.13]		1.03 [0.97-1.10]	1.03 [0.97-1.10]	1.00 [0.94-1.06]	1.01 [0.94-1.08]		1.01 [0.95-1.08]	2,849	2,860	2,785	NA	2,762
E22	1.38 [1.28-1.49]	1.38 [1.28-1.49]	1.41 [1.30-1.52]		1.38 [1.28-1.49]	1.33 [1.23-1.44]	1.31 [1.22-1.42]	1.34 [1.24-1.45]		1.34 [1.24-1.44]	2,225	2,199	2,061	NA	2,120
E23	1.19 [1.06-1.33]	1.28 [1.13-1.44]	1.32 [1.16-1.50]	1.01 [0.72-1.42]	1.24 [1.11-1.39]	1.12 [1.00-1.26]	1.17 [1.04-1.32]	1.24 [1.09-1.41]	0.96 [0.68-1.36]	1.20 [1.07-1.35]	932	844	714	117	890
E24	1.31 [1.05-1.64]	1.13 [0.91-1.42]	1.38 [1.09-1.75]		1.23 [0.99-1.53]	1.13 [0.90-1.43]	0.90 [0.71-1.14]	1.13 [0.87-1.45]		1.09 [0.87-1.37]	245	236	199	NA	244
E26	1.03 [0.76-1.39]	0.98 [0.72-1.33]	0.99 [0.72-1.36]		1.11 [0.83-1.49]	0.99 [0.73-1.35]	0.93 [0.68-1.27]	0.89 [0.64-1.24]		1.11 [0.82-1.49]	126	121	108	NA	121
E27	1.32 [1.23-1.43]	1.30 [1.20-1.40]	1.29 [1.19-1.39]	1.30 [0.90-1.87]	1.26 [1.17-1.36]	1.22 [1.13-1.32]	1.16 [1.07-1.25]	1.17 [1.07-1.26]	1.18 [0.81-1.72]	1.18 [1.10-1.28]	2,178	2,118	1,877	124	2,151
E28	1.10 [1.05-1.16]	1.13 [1.08-1.19]	1.17 [1.03-1.32]	0.94 [0.83-1.06]	1.11 [1.05-1.17]	1.03 [0.98-1.09]	1.02 [0.97-1.07]	1.01 [0.89-1.15]	0.95 [0.84-1.08]	1.05 [1.00-1.11]	4,613	5,088	734	906	4,090
E29	1.43 [1.17-1.76]	1.37 [1.12-1.68]	1.41 [1.14-1.73]		1.27 [1.04-1.55]	1.25 [1.01-1.55]	1.15 [0.93-1.43]	1.18 [0.95-1.47]		1.17 [0.95-1.44]	303	299	286	NA	294
E30	1.29 [0.96-1.72]			1.11 [0.82-1.49]	1.25 [0.92-1.69]	1.25 [0.92-1.69]			1.07 [0.78-1.46]	1.21 [0.89-1.65]	187	NA	NA	172	172
E34	1.06 [0.90-1.23]	1.13 [0.92-1.38]	1.21 [0.95-1.55]	1.07 [0.86-1.34]	1.12 [0.96-1.32]	0.99 [0.85-1.17]	0.98 [0.79-1.21]	1.14 [0.88-1.46]	1.03 [0.82-1.29]	1.08 [0.92-1.27]	531	279	186	276	486
E43	1.24 [0.90-1.72]	0.97 [0.71-1.34]			1.04 [0.75-1.43]	1.21 [0.86-1.68]	0.92 [0.67-1.27]			1.07 [0.77-1.48]	111	112	NA	NA	112
E46	1.19 [1.08-1.30]	1.13 [1.03-1.24]	1.15 [1.04-1.26]		1.15 [1.05-1.25]	1.17 [1.07-1.29]	1.08 [0.99-1.19]	1.10 [1.01-1.21]		1.15 [1.05-1.26]	1,485	1,468	1,370	NA	1,450
E51	1.42 [1.07-1.88]	1.18 [0.89-1.57]	1.42 [1.07-1.88]		1.34 [1.01-1.78]	1.38 [1.03-1.84]	1.09 [0.82-1.46]	1.30 [0.97-1.74]		1.40 [1.05-1.86]	152	144	152	NA	143
E53	1.19 [1.15-1.23]	1.18 [1.14-1.22]	1.17 [1.13-1.22]	1.11 [0.87-1.42]	1.18 [1.14-1.22]	1.14 [1.10-1.19]	1.10 [1.06-1.14]	1.10 [1.06-1.14]	1.07 [0.82-1.39]	1.15 [1.11-1.19]	9,508	9,630	8,639	233	9,356
E55	1.23 [1.19-1.27]	1.21 [1.18-1.25]	1.22 [1.18-1.26]	1.19 [1.02-1.39]	1.20 [1.16-1.24]	1.16 [1.12-1.20]	1.12 [1.08-1.16]	1.12 [1.08-1.16]	1.13 [0.96-1.33]	1.15 [1.12-1.19]	13,124	12,882	11,410	648	12,776
E56	1.14 [0.83-1.57]	1.10 [0.79-1.53]			1.05 [0.77-1.44]	1.08 [0.78-1.50]	1.01 [0.72-1.42]			1.05 [0.77-1.43]	117	109	NA	NA	112
E58	1.36 [0.97-1.90]				1.45 [1.02-2.05]	1.31 [0.93-1.85]				1.43 [1.01-2.03]	106	NA	NA	NA	102
E61	1.22 [1.12-1.32]	1.19 [1.10-1.29]	1.21 [1.11-1.32]	1.16 [0.86-1.58]	1.18 [1.09-1.27]	1.14 [1.05-1.24]	1.09 [1.00-1.19]	1.10 [1.01-1.21]	1.08 [0.79-1.50]	1.13 [1.04-1.22]	1,962	1,918	1,636	151	1,931
E63	1.35 [0.98-1.87]	1.19 [0.86-1.65]	1.33 [0.94-1.88]		1.35 [0.98-1.86]	1.34 [0.96-1.87]	1.12 [0.80-1.57]	1.26 [0.89-1.80]		1.30 [0.94-1.80]	122	116	109	NA	117
E66	1.18 [1.16-1.20]	1.19 [1.18-1.21]	1.19 [1.17-1.21]	1.05 [0.99-1.11]	1.16 [1.14-1.17]	1.11 [1.09-1.12]	1.08 [1.06-1.10]	1.07 [1.06-1.09]	1.04 [0.97-1.11]	1.10 [1.09-1.12]	61,881	63,421	46,219	3,733	58,549
E72	1.24 [0.90-1.70]	1.31 [0.95-1.82]			1.32 [0.96-1.81]	1.18 [0.86-1.64]	1.21 [0.87-1.70]			1.35 [0.98-1.86]	118	107	NA	NA	115
E73	1.25 [1.08-1.45]	1.21 [1.02-1.43]	1.18 [0.96-1.45]	1.27 [0.98-1.64]	1.18 [1.02-1.37]	1.06 [0.91-1.24]	1.03 [0.86-1.23]	0.97 [0.78-1.21]	1.17 [0.89-1.52]	1.05 [0.90-1.23]	568	410	270	213	519
E74	1.04 [0.82-1.32]	1.05 [0.83-1.34]	1.00 [0.76-1.30]		1.13 [0.89-1.43]	1.00 [0.79-1.28]	0.99 [0.78-1.27]	0.95 [0.72-1.25]		1.08 [0.85-1.37]	202	184	150	NA	196
E78	1.09 [1.07-1.10]	1.08 [1.07-1.10]	1.09 [1.07-1.10]		1.07 [1.06-1.09]	1.06 [1.04-1.07]	1.02 [1.01-1.04]	1.02 [1.01-1.04]		1.06 [1.05-1.07]	77,578	77,625	76,760	NA	69,482
E79	1.16 [0.84-1.61]	1.14 [0.82-1.60]				1.11 [0.79-1.55]	1.07 [0.76-1.51]				107	104	NA	NA	NA
E80	1.08 [0.96-1.21]	1.10 [0.98-1.24]	1.09 [0.95-1.25]		1.01 [0.89-1.14]	1.04 [0.92-1.17]	1.06 [0.94-1.20]	1.06 [0.93-1.22]		0.98 [0.87-1.11]	804	786	608	NA	747
E83	1.12 [1.08-1.15]	1.12 [1.09-1.15]	1.12 [1.09-1.15]	0.94 [0.74-1.20]	1.10 [1.07-1.13]	1.09 [1.05-1.12]	1.06 [1.03-1.09]	1.06 [1.03-1.09]	0.92 [0.72-1.18]	1.09 [1.06-1.12]	13,585	13,541	12,929	228	13,207
E85	1.15 [0.99-1.34]	1.13 [0.97-1.31]	1.10 [0.94-1.28]		1.08 [0.93-1.26]	1.10 [0.95-1.29]	1.06 [0.91-1.24]	1.02 [0.88-1.20]		1.04 [0.89-1.22]	493	482	470	NA	473
E86	1.12 [1.10-1.15]	1.12 [1.10-1.14]	1.12 [1.10-1.14]	1.05 [0.95-1.16]	1.11 [1.09-1.13]	1.11 [1.09-1.13]	1.07 [1.05-1.09]	1.07 [1.05-1.09]	1.03 [0.93-1.14]	1.10 [1.08-1.12]	35,956	35,245	33,527	1,281	34,906
E87	1.20 [1.18-1.22]	1.20 [1.18-1.21]	1.20 [1.18-1.21]	0.98 [0.86-1.11]	1.18 [1.16-1.20]	1.17 [1.15-1.18]	1.13 [1.11-1.15]	1.13 [1.11-1.15]	0.97 [0.84-1.11]	1.16 [1.14-1.18]	58,707	58,443	56,881	764	56,673
E88	1.22 [1.14-1.30]	1.19 [1.11-1.28]	1.23 [1.14-1.32]	1.13 [0.80-1.59]	1.18 [1.10-1.26]	1.18 [1.10-1.27]	1.13 [1.06-1.22]	1.16 [1.08-1.25]	1.04 [0.73-1.49]	1.16 [1.08-1.24]	2,559	2,509	2,353	112	2,518
E89	1.12 [1.06-1.19]	1.12 [1.06-1.19]	1.13 [1.07-1.20]		1.09 [1.03-1.15]	1.08 [1.02-1.14]	1.05 [0.99-1.11]	1.06 [0.99-1.12]		1.05 [0.99-1.12]	3,465	3,453	3,140	NA	3,350
Mental, Behavioral and Neurodevelopmental disorders (F01-F99)															
F00	1.02 [0.98-1.06]	1.02 [0.98-1.06]	1.03 [0.99-1.06]		1.04 [1.00-1.08]	1.03 [0.99-1.07]	1.01 [0.98-1.05]	1.02 [0.98-1.06]		1.04 [1.00-1.08]	8,435	8,435	8,431	NA	8,103
F01	1.04 [1.01-1.08]	1.03 [1.00-1.07]	1.03 [1.00-1.07]		1.04 [1.00-1.07]	1.04 [1.00-1.07]	1.00 [0.97-1.04]	1.00 [0.97-1.04]		1.04 [1.00-1.07]	10,012	10,023	10,004	NA	9,758
F02	0.97 [0.89-1.06]	0.95 [0.87-1.03]	0.95 [0.87-1.03]		0.92 [0.85-1.00]	0.99 [0.91-1.08]	0.95 [0.87-1.03]	0.95 [0.87-1.04]		0.94 [0.87-1.03]	1,536	1,537	1,535	NA	1,471
F03	1.03 [1.01-1.05]	1.02 [1.00-1.04]	1.03 [1.00-1.05]		1.03 [1.00-1.05]	1.03 [1.00-1.05]	1.01 [0.98-1.03]	1.01 [0.99-1.04]		1.03 [1.01-1.06]	23,316	23,290	23,245	NA	22,287
F05	1.11 [1.09-1.14]	1.11 [1.09-1.14]	1.10 [1.08-1.13]		1.11 [1.08-1.13]	1.10 [1.07-1.12]	1.07 [1.04-1.10]	1.06 [1.04-1.09]		1.10 [1.08-1.13]	22,934	22,916	22,832	NA	22,664
F06	1.11 [1.05-1.18]	1.09 [1.03-1.16]	1.11 [1.04-1.18]		1.06 [1.00-1.13]	1.09 [1.03-1.16]	1.05 [0.99-1.12]	1.07 [1.00-1.13]		1.06 [1.00-1.13]	3,455	3,439	3,408	NA	3,371
F07	1.16 [0.91-1.49]	1.24 [0.96-1.59]	1.37 [1.04-1.81]		1.07 [0.83-1.38]	1.07 [0.83-1.39]	1.16 [0.89-1.50]	1.30 [0.98-1.73]		1.05 [0.81-1.36]	190	184	149	NA	173
F10	1.18 [1.14-1.21]	1.17 [1.14-1.21]	1.20 [1.16-1.24]	0.92 [0.80-1.05]	1.16 [1.13-1.20]	1.16 [1.12-1.19]	1.10 [1.06-1.13]	1.13 [1.09-1.17]	0.93 [0.80-1.07]	1.17 [1.14-1.21]	13,285	13,280	11,000	701	11,863
F11	1.19 [1.06-1.34]	1.12 [1.00-1.26]	1.18 [1.02-1.37]		1.13 [1.01-1.27]	1.11 [0.99-1.26]	0.94 [0.83-1.06]	1.02 [0.88-1.19]		1.11 [0.98-1.25]	878	901	518	NA	830
F12	1.09 [0.99-1.19]	1.06 [0.97-1.16]	1.14 [0.99-1.30]	0.93 [0.77-1.13]	0.98 [0.90-1.08]	1.02 [0.92-1.12]	0.92 [0.84-1.01]	1.00 [0.87-1.16]	0.96 [0.78-1.18]	0.98 [0.89-1.08]	1,468	1,526	635	369	1,346
F13	1.13 [0.91-1.42]	1.09 [0.87-1.36]	1.31 [1.01-1.68]		1.02 [0.82-1.28]	1.06 [0.84-1.34]	0.91 [0.72-1.16]	1.10 [0.84-1.44]		0.99 [0.79-1.24]	241	239	187	NA	230
F14	1.07 [0.93-1.23]	1.06 [0.92-1.22]	1.17 [0.93-1.46]	0.82 [0.60-1.14]	0.98 [0.85-1.13]	1.01 [0.87-1.18]	0.91 [0.78-1.05]	1.00 [0.79-1.27]	0.93 [0.66-1.31]	0.99 [0.85-1.14]	621	642	234	127	574
F15	1.07 [0.84-1.37]	0.92 [0.72-1.17]			0.96 [0.75-1.22]	1.01 [0.79-1.30]	0.86 [0.67-1.10]			0.96 [0.75-1.23]	207	195	NA	NA	188
F17	1.08 [1.07-1.10]	1.08 [1.07-1.10]	1.09 [1.08-1.11]	0.92 [0.87-0.96]	1.05 [1.04-1.07]	1.08 [1.06-1.09]	0.99 [0.98-1.00]	1.02 [1.00-1.04]	0.98 [0.93-1.04]	1.08 [1.07-1.10]	63,060	65,557	43,722	5,535	57,665
F19	0.98 [0.86-1.12]	0.98 [0.86-1.11]	1.01 [0.84-1.21]	0.70 [0.49-1.00]	0.93 [0.82-1.05]	0.90 [0.79-1.03]	0.83 [0.72-0.95]	0.86 [0.71-1.05]	0.71 [0.49-1.04]	0.93 [0.82-1.07]	693	713	325	104	679
F20	1.14 [1.07-1.23]	1.15 [1.07-1.24]	1.21 [1.12-1.31]		1.14 [1.06-1.23]	1.16 [1.07-1.24]	1.12 [1.05-1.21]	1.21 [1.12-1.31]		1.18 [1.09-1.27]	2,229	2,264	1,868	NA	1,949
F22	1.27 [1.14-1.40]	1.23 [1.11-1.36]	1.24 [1.11-1.38]		1.20 [1.08-1.33]	1.24 [1.11-1.37]	1.15 [1.04-1.28]	1.16 [1.04-1.30]		1.19 [1.07-1.32]	1,115	1,104	983	NA	1,044
F23	1.17 [1.01-1.36]	1.15 [0.99-1.34]	1.25 [1.04-1.49]		1.10 [0.94-1.29]	1.13 [0.97-1.32]	1.09 [0.94-1.28]	1.21 [1.01-1.45]		1.11 [0.94-1.30]	525	522	360	NA	451
F25	1.24 [1.07-1.45]	1.24 [1.07-1.44]	1.26 [1.06-1.49]		1.10 [0.95-1.27]	1.26 [1.08-1.48]	1.18 [1.01-1.38]	1.25 [1.05-1.48]		1.16 [1.00-1.35]	530	532	384	NA	507
F29	1.27 [1.14-1.40]	1.26 [1.14-1.40]	1.30 [1.15-1.46]		1.12 [1.01-1.24]	1.21 [1.09-1.35]	1.15 [1.03-1.28]	1.21 [1.07-1.36]		1.11 [0.99-1.23]	1,133	1,157	847	NA	1,059
F30	1.41 [1.17-1.72]	1.31 [1.08-1.59]	1.45 [1.18-1.79]		1.34 [1.10-1.63]	1.38 [1.14-1.68]	1.23 [1.01-1.50]	1.39 [1.13-1.72]		1.34 [1.10-1.64]	327	325	271	NA	294
F31	1.16 [1.09-1.24]	1.16 [1.09-1.23]	1.17 [1.09-1.26]	0.87 [0.68-1.12]	1.09 [1.03-1.16]	1.08 [1.01-1.16]	1.00 [0.94-1.07]	1.06 [0.98-1.14]	0.87 [0.67-1.13]	1.07 [1.00-1.14]	2,932	3,013	2,046	201	2,779
F32	1.24 [1.22-1.26]	1.23 [1.22-1.25]	1.24 [1.22-1.26]	0.97 [0.91-1.03]	1.20 [1.18-1.22]	1.14 [1.12-1.16]	1.06 [1.04-1.08]	1.08 [1.06-1.10]	1.00 [0.93-1.06]	1.15 [1.13-1.16]	56,319	57,470	43,384	3,400	53,486
F33	1.15 [1.06-1.25]	1.19 [1.09-1.29]	1.09 [1.00-1.19]		1.12 [1.03-1.22]	1.07 [0.98-1.16]	1.03 [0.95-1.13]	0.96 [0.87-1.05]		1.09 [1.00-1.19]	1,658	1,709	1,335	NA	1,580
F34	1.22 [0.99-1.50]	1.25 [1.01-1.55]	1.31 [1.04-1.67]		1.16 [0.94-1.42]	1.09 [0.87-1.35]	1.06 [0.85-1.32]	1.12 [0.88-1.44]		1.06 [0.86-1.31]	277	282	214	NA	260
F38	1.15 [0.84-1.58]	1.16 [0.86-1.58]			1.17 [0.87-1.58]	1.05 [0.76-1.46]	1.07 [0.78-1.47]			1.14 [0.84-1.55]	123	125	NA	NA	127
F39	1.17 [0.93-1.49]	1.09 [0.87-1.38]	1.27 [0.98-1.64]		1.16 [0.93-1.46]	1.09 [0.86-1.39]	0.97 [0.76-1.23]	1.13 [0.86-1.47]		1.11 [0.88-1.39]	224	221	178	NA	227
F40	1.19 [1.13-1.25]	1.18 [1.12-1.25]	1.22 [1.15-1.30]	0.98 [0.83-1.15]	1.16 [1.10-1.22]	1.11 [1.06-1.17]	1.07 [1.01-1.13]	1.11 [1.05-1.18]	0.96 [0.81-1.13]	1.11 [1.05-1.17]	4,691	4,579	3,298	557	4,352
F41	1.22 [1.20-1.24]	1.21 [1.19-1.23]	1.23 [1.21-1.26]	0.97 [0.91-1.03]	1.18 [1.16-1.20]	1.11 [1.10-1.13]	1.05 [1.03-1.06]	1.08 [1.06-1.10]	0.98 [0.92-1.05]	1.12 [1.10-1.14]	46,858	47,954	34,383	3,904	44,519
F42	1.53 [1.38-1.70]	1.51 [1.36-1.67]	1.68 [1.45-1.94]	0.92 [0.69-1.21]	1.43 [1.29-1.58]	1.36 [1.22-1.52]	1.25 [1.12-1.40]	1.47 [1.26-1.70]	0.83 [0.61-1.11]	1.32 [1.18-1.47]	1,227	1,234	650	177	1,147
F43	1.17 [1.09-1.26]	1.15 [1.08-1.24]	1.19 [1.08-1.30]	0.85 [0.69-1.04]	1.10 [1.02-1.18]	1.04 [0.96-1.12]	0.94 [0.87-1.01]	0.99 [0.90-1.10]	0.87 [0.70-1.09]	1.04 [0.97-1.12]	2,340	2,405	1,384	282	2,271
F44	1.23 [1.07-1.41]	1.23 [1.07-1.42]	1.28 [1.07-1.52]		1.13 [0.98-1.29]	1.10 [0.95-1.27]	1.04 [0.90-1.20]	1.06 [0.89-1.28]		1.05 [0.92-1.21]	632	642	402	NA	627
F45	1.30 [1.15-1.47]	1.28 [1.13-1.44]	1.20 [1.05-1.38]		1.22 [1.08-1.38]	1.16 [1.02-1.32]	1.10 [0.97-1.26]	1.06 [0.92-1.22]		1.12 [0.99-1.27]	808	780	608	NA	756
F48	1.45 [1.14-1.85]	1.26 [0.98-1.60]	1.19 [0.89-1.58]		1.08 [0.85-1.38]	1.32 [1.03-1.69]	1.07 [0.82-1.38]	1.00 [0.74-1.36]		0.98 [0.76-1.27]	215	204	144	NA	188
F50	1.20 [1.07-1.35]	1.19 [1.06-1.34]	1.28 [1.06-1.55]	0.92 [0.74-1.14]	1.06 [0.95-1.20]	1.10 [0.97-1.24]	1.03 [0.92-1.17]	1.14 [0.94-1.39]	0.95 [0.76-1.19]	1.03 [0.91-1.16]	943	912	327	293	834
F51	1.29 [1.03-1.63]	1.36 [1.07-1.74]	1.26 [0.95-1.67]		1.32 [1.05-1.66]	1.18 [0.93-1.50]	1.22 [0.95-1.57]	1.10 [0.82-1.48]		1.25 [0.99-1.58]	240	210	153	NA	237
F52	1.18 [1.00-1.38]	1.14 [0.97-1.34]	1.21 [1.03-1.43]		1.17 [1.00-1.38]	1.11 [0.94-1.31]	1.04 [0.88-1.22]	1.09 [0.92-1.29]		1.13 [0.96-1.33]	445	454	430	NA	435
F53	1.29 [1.10-1.52]	1.27 [1.08-1.49]			1.23 [1.06-1.43]	1.23 [1.04-1.45]	1.12 [0.95-1.32]			1.18 [1.01-1.39]	468	499	NA	NA	480
F60	1.29 [1.19-1.39]	1.23 [1.14-1.33]	1.38 [1.24-1.53]	0.87 [0.72-1.06]	1.16 [1.08-1.25]	1.14 [1.05-1.23]	0.98 [0.90-1.06]	1.16 [1.04-1.30]	0.93 [0.76-1.13]	1.11 [1.02-1.19]	2,140	2,237	1,035	348	2,106
F69	1.21 [0.91-1.60]	1.39 [1.04-1.84]	1.57 [1.12-2.21]		1.24 [0.94-1.62]	1.10 [0.82-1.47]	1.15 [0.85-1.55]	1.32 [0.92-1.89]		1.18 [0.89-1.56]	157	150	104	NA	155
F70	1.59 [1.31-1.94]	1.78 [1.45-2.18]	2.06 [1.63-2.61]		1.55 [1.27-1.89]	1.47 [1.20-1.81]	1.63 [1.32-2.02]	1.83 [1.43-2.33]		1.44 [1.17-1.78]	363	335	246	NA	325
F71	2.01 [1.45-2.77]	2.05 [1.48-2.85]			2.06 [1.48-2.87]	1.90 [1.36-2.64]	2.02 [1.44-2.84]			2.08 [1.47-2.94]	149	140	NA	NA	130
F72	1.78 [1.37-2.29]	1.81 [1.38-2.37]	2.37 [1.71-3.28]		1.46 [1.14-1.88]	1.69 [1.30-2.21]	1.93 [1.45-2.56]	2.24 [1.60-3.14]		1.44 [1.11-1.86]	220	195	143	NA	213
F79	1.55 [1.40-1.71]	1.72 [1.55-1.91]	1.82 [1.62-2.05]	0.86 [0.68-1.08]	1.50 [1.36-1.65]	1.49 [1.34-1.64]	1.66 [1.49-1.85]	1.76 [1.56-1.99]	0.86 [0.68-1.08]	1.44 [1.30-1.60]	1,442	1,274	985	264	1,353
F80	1.19 [1.08-1.33]	1.31 [1.12-1.52]	1.28 [1.10-1.50]	1.11 [0.96-1.28]	1.16 [1.04-1.29]	1.16 [1.04-1.29]	1.24 [1.07-1.45]	1.22 [1.04-1.44]	1.08 [0.93-1.25]	1.15 [1.03-1.28]	1,149	511	488	651	1,097
F81	1.54 [1.45-1.64]	1.66 [1.56-1.78]	1.87 [1.74-2.02]	0.97 [0.85-1.10]	1.45 [1.36-1.55]	1.47 [1.38-1.57]	1.63 [1.53-1.75]	1.82 [1.69-1.97]	0.96 [0.84-1.09]	1.41 [1.32-1.51]	3,760	3,214	2,399	879	3,253
F82	0.96 [0.69-1.33]				1.06 [0.76-1.49]	0.92 [0.66-1.28]				1.01 [0.72-1.44]	117	NA	NA	NA	113
F84	1.36 [1.26-1.48]	1.52 [1.36-1.70]	1.92 [1.62-2.27]	1.14 [1.03-1.26]	1.29 [1.18-1.40]	1.24 [1.13-1.35]	1.37 [1.22-1.54]	1.70 [1.43-2.03]	1.10 [0.99-1.22]	1.21 [1.11-1.32]	2,261	1,118	492	1,500	2,041
F89	0.98 [0.84-1.14]			0.91 [0.77-1.07]	0.93 [0.80-1.08]	0.92 [0.78-1.07]			0.87 [0.74-1.03]	0.88 [0.75-1.03]	545	NA	NA	486	542
F90	1.10 [0.98-1.24]	1.17 [0.99-1.37]	1.58 [1.10-2.26]	0.98 [0.85-1.12]	1.11 [0.98-1.25]	1.04 [0.91-1.18]	1.03 [0.87-1.23]	1.20 [0.81-1.79]	0.98 [0.85-1.14]	1.10 [0.97-1.25]	990	516	103	727	916
F91	1.10 [0.86-1.40]			1.21 [0.87-1.69]	1.14 [0.89-1.46]	1.06 [0.83-1.36]			1.18 [0.84-1.66]	1.14 [0.89-1.46]	219	NA	NA	136	209
F95	1.18 [0.85-1.63]				1.23 [0.88-1.71]	1.04 [0.74-1.46]				1.14 [0.81-1.61]	138	NA	NA	NA	131
F98	1.05 [0.78-1.41]			1.13 [0.78-1.64]	1.03 [0.76-1.40]	0.95 [0.69-1.30]			1.08 [0.73-1.59]	1.00 [0.73-1.37]	151	NA	NA	107	148
F99	1.11 [0.98-1.26]	1.15 [1.02-1.30]	1.17 [1.01-1.36]		1.11 [0.98-1.25]	1.05 [0.92-1.20]	1.01 [0.89-1.16]	1.07 [0.92-1.24]		1.08 [0.95-1.23]	752	766	516	NA	734
Diseases of the nervous system (G00-G99)															
G00	1.06 [0.83-1.35]	1.07 [0.83-1.39]	1.05 [0.79-1.41]		0.99 [0.76-1.28]	1.01 [0.78-1.30]	1.01 [0.77-1.32]	1.00 [0.74-1.34]		0.96 [0.74-1.26]	186	169	125	NA	160
G03	0.99 [0.79-1.24]	1.01 [0.80-1.28]	0.99 [0.76-1.30]		0.91 [0.72-1.14]	0.94 [0.74-1.18]	0.92 [0.72-1.17]	0.91 [0.69-1.21]		0.86 [0.68-1.08]	233	204	145	NA	200

Supplementary Table 4: Hazard ratios and events from all cohorts (excluding non-consulters)

	Hazard ratio (99% confidence interval)														
	crude					adjusted					Events (in exposed)				
Outcome	any age	18+	40+	<18	hosp.	any age	18+	40+	<18	hosp.	any age	18+	40+	<18	hosp.
G04	1.17 [1.02-1.34]	1.16 [1.01-1.34]	1.19 [1.03-1.38]		1.09 [0.95-1.25]	1.15 [1.00-1.32]	1.09 [0.94-1.26]	1.13 [0.97-1.31]		1.07 [0.93-1.23]	628	586	514	NA	583
G05	1.39 [1.07-1.81]	1.48 [1.13-1.94]	1.39 [1.05-1.84]		1.52 [1.16-1.99]	1.37 [1.05-1.78]	1.44 [1.10-1.90]	1.35 [1.02-1.80]		1.45 [1.10-1.91]	177	164	150	NA	162
G06	1.04 [0.84-1.29]	1.04 [0.83-1.29]	1.01 [0.81-1.27]		1.08 [0.87-1.34]	1.00 [0.81-1.25]	1.01 [0.80-1.26]	0.99 [0.79-1.24]		1.08 [0.87-1.34]	243	227	211	NA	235
G08	1.12 [0.83-1.51]	1.35 [1.00-1.82]			1.19 [0.88-1.63]	1.05 [0.77-1.43]	1.31 [0.96-1.78]			1.19 [0.87-1.62]	130	135	NA	NA	115
G09	1.19 [0.91-1.56]	1.18 [0.90-1.56]	1.10 [0.82-1.48]		1.15 [0.89-1.50]	1.15 [0.87-1.52]	1.13 [0.85-1.50]	1.06 [0.78-1.43]		1.10 [0.84-1.43]	160	154	127	NA	163
G11	1.06 [0.86-1.29]	1.16 [0.95-1.43]	1.16 [0.94-1.44]		1.15 [0.94-1.40]	1.04 [0.85-1.28]	1.10 [0.90-1.36]	1.08 [0.87-1.35]		1.15 [0.94-1.40]	287	268	241	NA	278
G12	1.08 [0.95-1.24]	1.12 [0.98-1.28]	1.05 [0.92-1.20]		1.07 [0.93-1.22]	1.06 [0.93-1.21]	1.09 [0.96-1.25]	1.03 [0.90-1.18]		1.06 [0.92-1.21]	627	620	598	NA	554
G20	1.01 [0.97-1.06]	1.00 [0.96-1.04]	1.02 [0.97-1.06]		1.01 [0.96-1.05]	1.02 [0.97-1.06]	0.98 [0.94-1.03]	1.01 [0.96-1.05]		1.01 [0.97-1.06]	5,956	5,962	5,945	NA	5,377
G21	1.26 [1.09-1.44]	1.21 [1.05-1.38]	1.24 [1.08-1.43]		1.19 [1.04-1.37]	1.26 [1.09-1.44]	1.17 [1.02-1.34]	1.22 [1.06-1.40]		1.21 [1.06-1.39]	646	648	634	NA	632
G23	1.08 [0.91-1.30]	1.05 [0.88-1.26]	1.04 [0.87-1.25]		1.03 [0.86-1.23]	1.08 [0.90-1.29]	1.05 [0.88-1.26]	1.02 [0.85-1.23]		1.02 [0.85-1.23]	350	343	337	NA	334
G24	1.12 [0.98-1.28]	1.15 [1.00-1.31]	1.11 [0.96-1.28]		1.11 [0.97-1.26]	1.09 [0.95-1.25]	1.07 [0.93-1.23]	1.06 [0.91-1.22]		1.09 [0.96-1.25]	657	613	538	NA	621
G25	1.17 [1.11-1.24]	1.18 [1.11-1.25]	1.16 [1.10-1.23]		1.15 [1.09-1.21]	1.08 [1.02-1.15]	1.05 [0.99-1.12]	1.05 [0.99-1.12]		1.09 [1.03-1.15]	3,742	3,708	3,508	NA	3,700
G30	1.02 [0.99-1.06]	1.03 [0.99-1.06]	1.03 [1.00-1.07]		1.04 [1.01-1.07]	1.02 [0.99-1.06]	1.01 [0.98-1.05]	1.02 [0.99-1.06]		1.04 [1.00-1.07]	11,206	11,223	11,212	NA	10,627
G31	1.11 [1.06-1.15]	1.11 [1.07-1.15]	1.09 [1.05-1.14]		1.09 [1.05-1.13]	1.09 [1.05-1.13]	1.07 [1.03-1.11]	1.06 [1.02-1.10]		1.09 [1.04-1.13]	8,278	8,257	8,155	NA	7,918
G32	1.27 [0.93-1.74]	1.13 [0.83-1.55]	1.42 [1.03-1.97]		1.27 [0.93-1.74]	1.22 [0.88-1.69]	1.05 [0.77-1.45]	1.33 [0.95-1.85]		1.30 [0.94-1.78]	120	116	116	NA	114
G35	1.13 [1.03-1.23]	1.12 [1.02-1.22]	1.15 [1.04-1.27]		1.10 [1.00-1.22]	1.08 [0.98-1.18]	1.01 [0.92-1.11]	1.05 [0.95-1.17]		1.10 [1.00-1.21]	1,393	1,413	1,119	NA	1,181
G37	1.18 [0.99-1.40]	1.13 [0.94-1.34]	1.30 [1.06-1.61]		1.18 [0.99-1.41]	1.13 [0.95-1.35]	1.01 [0.84-1.22]	1.23 [0.99-1.53]		1.18 [0.98-1.41]	384	380	265	NA	355
G40	1.21 [1.17-1.25]	1.22 [1.18-1.26]	1.24 [1.19-1.28]	1.03 [0.91-1.15]	1.16 [1.12-1.20]	1.14 [1.10-1.18]	1.11 [1.07-1.15]	1.12 [1.08-1.16]	1.04 [0.92-1.17]	1.13 [1.09-1.17]	10,243	9,698	8,141	990	9,088
G41	1.17 [1.03-1.33]	1.25 [1.09-1.44]	1.30 [1.12-1.51]	0.92 [0.67-1.27]	1.10 [0.97-1.25]	1.13 [0.99-1.29]	1.17 [1.01-1.35]	1.22 [1.05-1.42]	0.94 [0.68-1.29]	1.10 [0.97-1.25]	711	610	545	124	713
G43	1.18 [1.14-1.22]	1.17 [1.14-1.22]	1.19 [1.14-1.24]	1.01 [0.90-1.13]	1.13 [1.09-1.17]	1.06 [1.02-1.10]	1.00 [0.96-1.04]	1.02 [0.97-1.07]	1.00 [0.89-1.12]	1.05 [1.01-1.08]	10,276	10,449	6,311	1,111	9,772
G44	1.18 [1.07-1.29]	1.14 [1.04-1.25]	1.25 [1.12-1.39]	0.89 [0.64-1.23]	1.17 [1.07-1.28]	1.08 [0.98-1.19]	1.02 [0.93-1.12]	1.11 [0.99-1.24]	0.90 [0.64-1.26]	1.10 [1.01-1.21]	1,442	1,449	992	124	1,402
G45	1.10 [1.06-1.14]	1.11 [1.06-1.15]	1.09 [1.05-1.14]		1.07 [1.03-1.11]	1.07 [1.03-1.11]	1.06 [1.02-1.10]	1.04 [1.00-1.08]		1.06 [1.02-1.10]	7,704	7,732	7,625	NA	7,223
G46	1.12 [0.96-1.30]	1.14 [0.98-1.33]	1.09 [0.94-1.27]		1.07 [0.93-1.24]	1.09 [0.94-1.27]	1.10 [0.95-1.29]	1.04 [0.89-1.21]		1.06 [0.91-1.23]	518	519	506	NA	511
G47	1.31 [1.27-1.35]	1.34 [1.30-1.39]	1.34 [1.29-1.39]	1.21 [1.12-1.31]	1.29 [1.26-1.33]	1.18 [1.15-1.22]	1.14 [1.11-1.18]	1.15 [1.11-1.19]	1.16 [1.07-1.26]	1.20 [1.16-1.23]	13,363	11,232	10,088	2,412	12,881
G50	1.32 [1.20-1.45]	1.33 [1.21-1.46]	1.30 [1.18-1.43]		1.26 [1.15-1.39]	1.22 [1.11-1.34]	1.18 [1.07-1.30]	1.15 [1.04-1.27]		1.19 [1.08-1.30]	1,397	1,402	1,282	NA	1,315
G51	1.15 [1.08-1.23]	1.18 [1.10-1.27]	1.14 [1.06-1.22]	1.23 [0.90-1.67]	1.13 [1.05-1.21]	1.10 [1.03-1.18]	1.10 [1.03-1.19]	1.07 [0.99-1.15]	1.20 [0.87-1.66]	1.10 [1.02-1.18]	2,495	2,419	2,162	146	2,334
G52	1.11 [0.86-1.41]	1.15 [0.90-1.48]	1.16 [0.89-1.51]		1.17 [0.91-1.49]	1.07 [0.84-1.38]	1.08 [0.84-1.39]	1.08 [0.83-1.42]		1.13 [0.88-1.45]	184	187	167	NA	180
G53	1.25 [1.07-1.47]	1.21 [1.03-1.42]	1.23 [1.04-1.44]		1.21 [1.03-1.42]	1.18 [1.00-1.39]	1.14 [0.96-1.34]	1.17 [0.99-1.38]		1.16 [0.99-1.36]	462	461	440	NA	444
G54	1.24 [1.09-1.40]	1.16 [1.03-1.32]	1.23 [1.08-1.40]		1.18 [1.05-1.33]	1.15 [1.02-1.31]	1.03 [0.91-1.17]	1.10 [0.96-1.25]		1.13 [1.00-1.28]	772	768	660	NA	753
G55	1.16 [1.11-1.20]	1.15 [1.11-1.19]	1.17 [1.13-1.22]	0.86 [0.63-1.18]	1.12 [1.08-1.17]	1.08 [1.04-1.12]	1.04 [1.00-1.08]	1.06 [1.01-1.10]	0.77 [0.55-1.09]	1.07 [1.03-1.11]	8,112	8,175	7,096	123	7,942
G56	1.22 [1.18-1.26]	1.21 [1.18-1.25]	1.20 [1.16-1.23]	1.07 [0.77-1.48]	1.18 [1.14-1.21]	1.14 [1.10-1.17]	1.10 [1.07-1.14]	1.10 [1.06-1.13]	1.02 [0.71-1.46]	1.11 [1.08-1.15]	13,152	13,206	11,717	125	12,002
G57	1.25 [1.15-1.37]	1.26 [1.15-1.37]	1.25 [1.14-1.37]		1.16 [1.06-1.27]	1.18 [1.08-1.29]	1.15 [1.05-1.26]	1.15 [1.04-1.26]		1.11 [1.02-1.22]	1,441	1,459	1,257	NA	1,371
G58	1.23 [1.09-1.39]	1.26 [1.12-1.43]	1.31 [1.15-1.48]		1.19 [1.06-1.34]	1.14 [1.01-1.29]	1.13 [1.00-1.28]	1.17 [1.03-1.34]		1.14 [1.01-1.29]	802	802	700	NA	782
G59	1.24 [1.10-1.40]	1.21 [1.07-1.36]	1.22 [1.08-1.37]		1.17 [1.04-1.31]	1.14 [1.01-1.29]	1.04 [0.92-1.18]	1.05 [0.93-1.19]		1.12 [1.00-1.27]	787	788	762	NA	767
G60	1.21 [1.01-1.44]	1.12 [0.94-1.34]	1.20 [1.00-1.45]		1.18 [0.99-1.41]	1.11 [0.92-1.33]	1.02 [0.85-1.23]	1.10 [0.91-1.33]		1.09 [0.91-1.32]	367	355	325	NA	345
G61	1.22 [1.03-1.44]	1.23 [1.05-1.46]	1.25 [1.05-1.48]		1.13 [0.95-1.35]	1.20 [1.01-1.41]	1.19 [1.01-1.41]	1.19 [1.00-1.42]		1.10 [0.92-1.31]	409	406	372	NA	363
G62	1.19 [1.13-1.24]	1.20 [1.14-1.25]	1.19 [1.14-1.25]		1.14 [1.09-1.19]	1.10 [1.05-1.16]	1.07 [1.02-1.12]	1.06 [1.01-1.11]		1.09 [1.04-1.14]	5,343	5,356	5,129	NA	5,257
G63	1.22 [1.15-1.30]	1.20 [1.13-1.27]	1.23 [1.16-1.31]		1.18 [1.11-1.25]	1.14 [1.07-1.21]	1.03 [0.97-1.10]	1.06 [1.00-1.13]		1.13 [1.06-1.19]	3,330	3,329	3,271	NA	3,363
G70	1.06 [0.90-1.25]	1.07 [0.90-1.26]	1.14 [0.96-1.36]		1.15 [0.97-1.36]	1.03 [0.87-1.21]	1.02 [0.86-1.21]	1.09 [0.91-1.29]		1.11 [0.94-1.32]	424	395	376	NA	382
G71	1.03 [0.84-1.26]	1.15 [0.93-1.43]	1.08 [0.85-1.37]		1.02 [0.83-1.26]	1.01 [0.82-1.24]	1.10 [0.88-1.38]	1.04 [0.81-1.33]		1.02 [0.82-1.26]	275	253	188	NA	252
G72	1.36 [1.18-1.57]	1.33 [1.15-1.53]	1.35 [1.17-1.57]		1.29 [1.12-1.48]	1.29 [1.11-1.49]	1.22 [1.06-1.42]	1.27 [1.09-1.48]		1.25 [1.08-1.44]	594	575	536	NA	566
G80	1.07 [0.94-1.22]	1.30 [1.10-1.52]	1.44 [1.19-1.73]	0.78 [0.63-0.96]	1.02 [0.89-1.18]	1.05 [0.92-1.20]	1.31 [1.11-1.54]	1.46 [1.21-1.77]	0.77 [0.62-0.95]	1.02 [0.88-1.17]	676	458	356	253	591
G81	1.10 [1.06-1.14]	1.11 [1.07-1.15]	1.11 [1.07-1.15]	0.98 [0.72-1.33]	1.08 [1.04-1.12]	1.09 [1.05-1.13]	1.07 [1.03-1.11]	1.06 [1.03-1.10]	0.99 [0.72-1.36]	1.08 [1.04-1.12]	9,002	8,870	8,701	138	8,612
G82	1.16 [1.03-1.31]	1.13 [1.00-1.28]	1.10 [0.97-1.25]		1.11 [0.98-1.25]	1.13 [1.00-1.27]	1.10 [0.97-1.24]	1.06 [0.93-1.20]		1.10 [0.97-1.25]	793	752	683	NA	737
G83	1.09 [0.99-1.20]	1.07 [0.97-1.18]	1.11 [1.00-1.23]		1.05 [0.95-1.15]	1.05 [0.95-1.16]	1.00 [0.91-1.11]	1.05 [0.94-1.16]		1.03 [0.93-1.13]	1,227	1,220	1,083	NA	1,205
G90	1.14 [1.00-1.29]	1.09 [0.96-1.24]	1.11 [0.97-1.28]		1.01 [0.89-1.15]	1.07 [0.94-1.22]	0.99 [0.87-1.12]	1.01 [0.88-1.17]		0.96 [0.84-1.09]	706	711	566	NA	663
G91	1.09 [1.00-1.19]	1.11 [1.02-1.22]	1.10 [1.01-1.21]		1.11 [1.01-1.21]	1.08 [0.99-1.18]	1.09 [0.99-1.19]	1.07 [0.98-1.18]		1.10 [1.00-1.20]	1,510	1,436	1,307	NA	1,376
G93	1.15 [1.10-1.20]	1.15 [1.11-1.20]	1.15 [1.10-1.20]	1.02 [0.88-1.19]	1.12 [1.07-1.17]	1.09 [1.04-1.14]	1.06 [1.01-1.11]	1.06 [1.01-1.12]	0.99 [0.85-1.16]	1.08 [1.03-1.13]	6,539	6,403	5,103	553	6,271
G94	1.09 [0.86-1.38]	1.16 [0.90-1.50]	1.17 [0.91-1.52]		1.06 [0.83-1.36]	1.08 [0.85-1.37]	1.14 [0.88-1.48]	1.17 [0.90-1.52]		1.08 [0.84-1.38]	195	179	166	NA	181
G95	1.09 [1.01-1.19]	1.12 [1.03-1.21]	1.14 [1.05-1.24]		1.07 [0.99-1.16]	1.06 [0.97-1.15]	1.07 [0.98-1.16]	1.08 [1.00-1.18]		1.05 [0.97-1.14]	1,733	1,708	1,606	NA	1,636
G96	1.22 [1.05-1.41]	1.23 [1.06-1.43]	1.31 [1.11-1.53]		1.22 [1.06-1.41]	1.13 [0.97-1.31]	1.11 [0.95-1.29]	1.19 [1.01-1.41]		1.18 [1.02-1.37]	566	548	461	NA	540
G97	1.10 [0.94-1.28]	1.14 [0.97-1.33]	1.21 [0.99-1.48]		1.10 [0.94-1.28]	1.01 [0.86-1.18]	1.02 [0.86-1.20]	1.11 [0.90-1.36]		1.05 [0.90-1.23]	497	481	279	NA	474
G98	1.23 [0.97-1.55]	1.18 [0.93-1.49]	1.28 [1.00-1.65]		1.23 [0.98-1.54]	1.13 [0.89-1.43]	1.03 [0.80-1.31]	1.10 [0.84-1.43]		1.12 [0.89-1.42]	222	216	194	NA	221
G99	1.17 [1.10-1.24]	1.16 [1.09-1.24]	1.15 [1.08-1.22]		1.13 [1.07-1.20]	1.11 [1.04-1.19]	1.07 [1.00-1.14]	1.06 [0.99-1.13]		1.09 [1.03-1.17]	2,947	2,941	2,737	NA	2,861
Diseases of the eye and adnexa (H00-H59)															
H00	1.18 [1.08-1.28]	1.21 [1.11-1.33]	1.18 [1.06-1.31]	1.06 [0.87-1.28]	1.19 [1.09-1.30]	1.14 [1.04-1.24]	1.18 [1.08-1.30]	1.16 [1.04-1.29]	1.05 [0.86-1.28]	1.17 [1.06-1.28]	1,657	1,403	1,018	359	1,410
H01	1.30 [1.21-1.41]	1.29 [1.19-1.40]	1.31 [1.21-1.42]		1.30 [1.20-1.40]	1.24 [1.14-1.34]	1.22 [1.13-1.32]	1.24 [1.14-1.35]		1.24 [1.14-1.34]	1,952	1,889	1,824	NA	1,855
H02	1.21 [1.17-1.25]	1.21 [1.17-1.26]	1.22 [1.17-1.26]	1.14 [0.89-1.47]	1.18 [1.13-1.22]	1.16 [1.12-1.20]	1.16 [1.11-1.20]	1.16 [1.12-1.20]	1.10 [0.85-1.43]	1.14 [1.09-1.18]	9,523	9,381	9,001	209	8,646
H03	1.30 [0.94-1.79]	1.23 [0.88-1.73]			1.29 [0.92-1.80]	1.24 [0.89-1.72]	1.14 [0.81-1.62]			1.19 [0.84-1.69]	118	101	NA	NA	107
H04	1.29 [1.23-1.35]	1.31 [1.25-1.38]	1.31 [1.25-1.38]	1.13 [0.93-1.39]	1.26 [1.20-1.32]	1.21 [1.15-1.27]	1.23 [1.17-1.29]	1.23 [1.16-1.29]	1.09 [0.89-1.33]	1.19 [1.13-1.25]	5,073	4,820	4,603	318	4,659
H05	1.31 [1.18-1.46]	1.32 [1.17-1.50]	1.33 [1.17-1.52]	1.23 [1.02-1.48]	1.31 [1.17-1.46]	1.27 [1.14-1.41]	1.25 [1.10-1.42]	1.25 [1.10-1.43]	1.18 [0.97-1.42]	1.27 [1.14-1.42]	1,164	799	693	403	1,031
H06	1.51 [1.19-1.92]	1.55 [1.23-1.96]	1.42 [1.10-1.83]		1.42 [1.12-1.81]	1.45 [1.14-1.85]	1.41 [1.11-1.79]	1.34 [1.03-1.74]		1.38 [1.08-1.76]	219	229	187	NA	195
H10	1.25 [1.17-1.34]	1.32 [1.22-1.43]	1.23 [1.14-1.34]	1.26 [1.06-1.48]	1.27 [1.18-1.36]	1.19 [1.11-1.28]	1.24 [1.14-1.34]	1.16 [1.07-1.26]	1.15 [0.97-1.36]	1.22 [1.13-1.31]	2,558	2,121	1,957	490	2,465
H11	1.14 [1.06-1.24]	1.15 [1.06-1.24]	1.16 [1.07-1.26]		1.13 [1.05-1.23]	1.11 [1.03-1.21]	1.11 [1.02-1.20]	1.12 [1.03-1.22]		1.11 [1.02-1.20]	1,836	1,797	1,678	NA	1,698
H13	1.38 [1.02-1.85]	1.67 [1.21-2.29]	1.65 [1.18-2.29]		1.31 [0.97-1.77]	1.35 [1.00-1.84]	1.62 [1.17-2.24]	1.56 [1.12-2.19]		1.28 [0.94-1.73]	144	126	113	NA	129
H15	1.44 [1.11-1.87]	1.39 [1.07-1.79]	1.38 [1.05-1.81]		1.36 [1.05-1.77]	1.37 [1.05-1.79]	1.31 [1.01-1.69]	1.29 [0.97-1.70]		1.28 [0.98-1.68]	191	189	163	NA	169
H16	1.43 [1.27-1.60]	1.34 [1.19-1.51]	1.40 [1.23-1.58]		1.41 [1.25-1.59]	1.36 [1.21-1.54]	1.29 [1.14-1.45]	1.34 [1.18-1.52]		1.37 [1.21-1.54]	915	881	800	NA	875
H17	1.26 [1.08-1.46]	1.20 [1.03-1.40]	1.27 [1.08-1.48]		1.27 [1.09-1.48]	1.21 [1.03-1.41]	1.16 [0.99-1.35]	1.22 [1.05-1.43]		1.22 [1.04-1.42]	537	520	495	NA	506
H18	1.17 [1.11-1.25]	1.17 [1.10-1.24]	1.13 [1.07-1.21]	1.76 [1.24-2.50]	1.18 [1.11-1.25]	1.14 [1.07-1.21]	1.13 [1.06-1.20]	1.10 [1.03-1.17]	1.53 [1.04-2.25]	1.14 [1.08-1.22]	3,409	3,382	3,032	140	3,216
H19	1.40 [1.11-1.76]	1.44 [1.15-1.82]	1.49 [1.17-1.90]		1.48 [1.17-1.86]	1.27 [1.00-1.61]	1.33 [1.05-1.68]	1.32 [1.03-1.70]		1.33 [1.05-1.69]	240	239	213	NA	230
H20	1.30 [1.16-1.47]	1.19 [1.06-1.34]	1.19 [1.06-1.35]		1.25 [1.12-1.41]	1.28 [1.14-1.44]	1.14 [1.01-1.29]	1.16 [1.02-1.31]		1.23 [1.10-1.39]	900	855	764	NA	847
H21	1.13 [1.04-1.24]	1.12 [1.03-1.23]	1.08 [0.99-1.18]		1.09 [1.00-1.19]	1.11 [1.01-1.21]	1.09 [1.00-1.19]	1.05 [0.96-1.15]		1.07 [0.98-1.17]	1,471	1,462	1,428	NA	1,401
H25	1.10 [1.08-1.12]	1.10 [1.08-1.12]	1.11 [1.09-1.13]		1.09 [1.07-1.11]	1.07 [1.05-1.09]	1.06 [1.04-1.08]	1.08 [1.06-1.10]		1.06 [1.04-1.08]	35,929	36,002	35,913	NA	32,562
H26	1.12 [1.11-1.14]	1.12 [1.10-1.14]	1.12 [1.10-1.14]	1.67 [1.14-2.46]	1.11 [1.09-1.13]	1.09 [1.08-1.11]	1.08 [1.06-1.09]	1.08 [1.06-1.10]	1.66 [1.11-2.48]	1.08 [1.07-1.10]	52,608	52,595	52,245	113	47,168
H27	1.10 [0.97-1.26]	1.16 [1.02-1.33]	1.11 [0.97-1.27]		1.17 [1.03-1.34]	1.09 [0.95-1.25]	1.13 [0.99-1.29]	1.08 [0.94-1.24]		1.15 [1.01-1.32]	663	652	622	NA	628
H28	1.17 [0.97-1.41]	1.23 [1.03-1.49]	1.17 [0.97-1.41]		1.21 [1.00-1.46]	1.12 [0.93-1.36]	1.15 [0.95-1.39]	1.09 [0.90-1.32]		1.18 [0.97-1.43]	321	323	308	NA	302
H31	1.06 [0.94-1.20]	1.05 [0.93-1.18]	1.02 [0.91-1.16]		1.05 [0.93-1.18]	1.05 [0.94-1.19]	1.03 [0.92-1.17]	1.00 [0.88-1.13]		1.04 [0.93-1.18]	819	817	796	NA	785
H33	0.98 [0.92-1.05]	1.02 [0.96-1.09]	0.99 [0.92-1.06]		1.01 [0.94-1.08]	0.97 [0.91-1.04]	1.01 [0.94-1.08]	0.97 [0.91-1.04]		1.00 [0.93-1.07]	2,569	2,550	2,359	NA	2,221
H34	1.09 [1.01-1.17]	1.09 [1.02-1.18]	1.09 [1.01-1.17]		1.06 [0.98-1.14]	1.07 [1.00-1.16]	1.06 [0.98-1.14]	1.04 [0.97-1.13]		1.05 [0.97-1.13]	2,072	2,081	2,052	NA	1,955
H35	1.10 [1.07-1.13]	1.11 [1.09-1.14]	1.10 [1.07-1.13]		1.09 [1.06-1.11]	1.08 [1.05-1.11]	1.08 [1.05-1.11]	1.07 [1.04-1.10]		1.07 [1.04-1.10]	19,118	19,098	18,853	NA	17,748
H36	1.05 [1.02-1.09]	1.06 [1.02-1.09]	1.06 [1.02-1.09]		1.05 [1.01-1.09]	1.03 [0.99-1.06]	0.98 [0.94-1.01]	0.97 [0.94-1.01]		1.04 [1.00-1.08]	8,639	8,638	8,365	NA	8,222
H40	1.11 [1.08-1.13]	1.11 [1.08-1.14]	1.11 [1.08-1.14]		1.10 [1.07-1.13]	1.08 [1.06-1.11]	1.08 [1.06-1.11]	1.09 [1.06-1.12]		1.08 [1.05-1.11]	17,114	17,086	16,991	NA	15,494
H43	1.06 [1.00-1.12]	1.07 [1.01-1.13]	1.08 [1.02-1.15]		1.04 [0.98-1.10]	1.03 [0.97-1.10]	1.03 [0.97-1.09]	1.04 [0.98-1.11]		1.02 [0.96-1.08]	3,277	3,291	3,156	NA	3,071
H44	1.23 [1.11-1.37]	1.21 [1.09-1.34]	1.20 [1.08-1.34]		1.18 [1.06-1.31]	1.19 [1.07-1.32]	1.16 [1.05-1.29]	1.15 [1.03-1.28]		1.14 [1.02-1.26]	1,097	1,065	1,005	NA	1,020
H46	1.41 [1.16-1.70]	1.40 [1.15-1.70]	1.38 [1.11-1.71]		1.38 [1.14-1.68]	1.37 [1.13-1.67]	1.30 [1.06-1.58]	1.28 [1.02-1.60]		1.34 [1.10-1.64]	348	345	257	NA	323
H47	1.12 [1.01-1.24]	1.15 [1.04-1.27]	1.16 [1.03-1.30]	1.21 [0.93-1.57]	1.10 [0.99-1.21]	1.10 [0.99-1.22]	1.10 [0.99-1.22]	1.14 [1.01-1.28]	1.20 [0.91-1.59]	1.10 [0.99-1.21]	1,212	1,132	842	204	1,123
H49	0.96 [0.85-1.09]	1.01 [0.89-1.15]	1.02 [0.89-1.16]		1.01 [0.89-1.14]	0.94 [0.83-1.06]	0.97 [0.85-1.10]	0.97 [0.85-1.11]		1.00 [0.88-1.13]	744	706	639	NA	681
H50	1.11 [1.03-1.19]	1.22 [1.11-1.35]	1.21 [1.08-1.35]	0.97 [0.86-1.08]	1.08 [1.00-1.17]	1.07 [0.99-1.16]	1.16 [1.05-1.28]	1.15 [1.02-1.28]	0.96 [0.85-1.08]	1.06 [0.98-1.15]	2,170	1,246	914	989	1,877
H51	1.22 [0.90-1.65]	1.14 [0.83-1.56]			1.13 [0.84-1.51]	1.15 [0.84-1.56]	1.06 [0.77-1.47]			1.09 [0.81-1.48]	134	115	NA	NA	131
H52	1.06 [1.01-1.11]	1.06 [1.01-1.11]	1.06 [1.01-1.12]	1.02 [0.80-1.29]	1.05 [1.00-1.10]	1.04 [0.99-1.09]	1.04 [0.99-1.09]	1.04 [0.99-1.09]	0.98 [0.77-1.25]	1.03 [0.98-1.08]	5,215	5,023	4,887	251	4,884
H53	1.14 [1.10-1.18]	1.14 [1.11-1.18]	1.15 [1.11-1.19]	1.02 [0.91-1.16]	1.10 [1.07-1.14]	1.07 [1.04-1.11]	1.05 [1.01-1.08]	1.07 [1.03-1.11]	1.01 [0.89-1.14]	1.06 [1.03-1.10]	10,579	10,452	7,983	962	10,026
H54	1.14 [1.10-1.19]	1.14 [1.09-1.18]	1.14 [1.09-1.18]	0.97 [0.75-1.25]	1.13 [1.09-1.17]	1.12 [1.08-1.16]	1.10 [1.05-1.14]	1.09 [1.05-1.13]	0.93 [0.72-1.21]	1.12 [1.07-1.16]	8,810	8,649	8,343	204	8,476
H55	1.08 [0.95-1.22]	1.11 [0.98-1.27]	1.05 [0.92-1.21]	0.88 [0.62-1.25]	1.09 [0.96-1.23]	1.04 [0.91-1.18]	1.04 [0.91-1.20]	0.98 [0.85-1.14]	0.87 [0.61-1.24]	1.06 [0.93-1.20]	741	667	580	108	716
H57	1.19 [1.10-1.29]	1.17 [1.08-1.28]	1.19 [1.10-1.30]	1.28 [0.96-1.71]	1.13 [1.05-1.23]	1.13 [1.04-1.23]	1.09 [1.00-1.19]	1.12 [1.03-1.23]	1.24 [0.92-1.67]	1.09 [1.00-1.18]	1,849	1,714	1,554	171	1,753
H58	1.05 [0.89-1.25]	1.09 [0.92-1.30]	1.06 [0.89-1.26]		1.08 [0.91-1.28]	1.03 [0.87-1.22]	1.05 [0.88-1.25]	0.99 [0.83-1.18]		1.07 [0.90-1.27]	395	389	374	NA	367
H59	1.07 [0.94-1.23]	1.08 [0.95-1.24]	1.03 [0.90-1.18]		1.10 [0.96-1.26]	1.07 [0.94-1.23]	1.08 [0.94-1.24]	1.02 [0.89-1.17]		1.10 [0.96-1.26]	617	610	597	NA	582
Diseases of the ear and mastoid process (H60-H95)															
H60	1.53 [1.41-1.67]	1.55 [1.42-1.70]	1.69 [1.52-1.87]	1.35 [1.09-1.69]	1.48 [1.36-1.61]	1.44 [1.32-1.57]	1.42 [1.30-1.56]	1.58 [1.42-1.75]	1.30 [1.03-1.64]	1.43 [1.31-1.56]	1,834	1,648	1,264	312	1,706
H61	1.32 [1.23-1.41]	1.30 [1.20-1.40]	1.33 [1.23-1.44]	1.26 [1.10-1.44]	1.30 [1.22-1.39]	1.28 [1.19-1.37]	1.24 [1.14-1.34]	1.26 [1.16-1.37]	1.22 [1.06-1.40]	1.26 [1.18-1.35]	2,833	2,130	1,902	764	2,654
H65	1.11 [1.05-1.17]	1.21 [1.09-1.34]	1.19 [1.06-1.34]	1.06 [1.00-1.13]	1.08 [1.02-1.14]	1.07 [1.01-1.13]	1.12 [1.01-1.25]	1.12 [1.00-1.26]	1.03 [0.97-1.10]	1.05 [0.99-1.11]	4,278	1,007	810	3,320	3,717
H66	1.21 [1.13-1.29]	1.38 [1.26-1.52]	1.38 [1.24-1.54]	1.12 [1.03-1.22]	1.20 [1.13-1.29]	1.16 [1.09-1.24]	1.29 [1.17-1.42]	1.29 [1.15-1.44]	1.10 [1.01-1.20]	1.17 [1.09-1.25]	2,960	1,419	1,051	1,672	2,718
H69	1.34 [1.08-1.66]	1.24 [0.99-1.55]	1.29 [0.99-1.69]		1.21 [0.98-1.49]	1.25 [1.00-1.56]	1.10 [0.87-1.39]	1.18 [0.89-1.55]		1.13 [0.91-1.41]	283	250	158	NA	261
H70	1.15 [0.95-1.40]	1.27 [1.03-1.58]	1.29 [1.02-1.63]		1.09 [0.90-1.32]	1.11 [0.91-1.35]	1.17 [0.94-1.46]	1.22 [0.96-1.55]		1.07 [0.88-1.30]	325	260	221	NA	308
H71	0.97 [0.84-1.11]	1.03 [0.89-1.20]	1.08 [0.90-1.29]	0.95 [0.71-1.29]	1.00 [0.86-1.15]	0.96 [0.83-1.10]	1.00 [0.86-1.16]	1.04 [0.87-1.25]	1.00 [0.73-1.36]	0.99 [0.86-1.15]	590	498	334	151	518
H72	1.16 [1.06-1.28]	1.20 [1.08-1.33]	1.17 [1.03-1.32]	1.16 [0.96-1.40]	1.09 [0.99-1.21]	1.11 [1.01-1.22]	1.13 [1.02-1.26]	1.12 [0.99-1.27]	1.13 [0.93-1.38]	1.05 [0.95-1.16]	1,371	1,072	742	393	1,206
H73	1.12 [0.99-1.28]	1.25 [1.06-1.47]	1.29 [1.06-1.57]	1.10 [0.90-1.34]	1.08 [0.94-1.23]	1.09 [0.95-1.25]	1.20 [1.01-1.42]	1.23 [1.01-1.50]	1.08 [0.88-1.32]	1.05 [0.92-1.21]	737	450	304	363	678
H74	1.10 [0.96-1.25]	1.10 [0.95-1.27]	1.22 [1.04-1.44]	0.95 [0.72-1.27]	1.08 [0.94-1.23]	1.08 [0.95-1.24]	1.05 [0.91-1.22]	1.17 [0.99-1.39]	0.96 [0.72-1.29]	1.06 [0.92-1.21]	705	566	432	177	626
H80	0.98 [0.76-1.26]	0.98 [0.76-1.27]	0.93 [0.70-1.24]		1.09 [0.84-1.41]	0.98 [0.76-1.27]	0.98 [0.75-1.27]	0.91 [0.68-1.23]		1.09 [0.84-1.42]	178	176	129	NA	160
H81	1.17 [1.10-1.23]	1.17 [1.11-1.24]	1.17 [1.11-1.24]		1.12 [1.06-1.18]	1.11 [1.04-1.17]	1.09 [1.03-1.16]	1.10 [1.04-1.16]		1.07 [1.01-1.13]	3,769	3,771	3,621	NA	3,536
H83	1.16 [1.06-1.27]	1.20 [1.09-1.31]	1.16 [1.05-1.27]		1.14 [1.04-1.25]	1.09 [0.99-1.19]	1.12 [1.02-1.23]	1.07 [0.97-1.18]		1.09 [0.99-1.19]	1,414	1,390	1,253	NA	1,326
H90	1.19 [1.12-1.26]	1.23 [1.16-1.31]	1.24 [1.17-1.33]	1.00 [0.86-1.17]	1.16 [1.10-1.23]	1.13 [1.07-1.20]	1.15 [1.08-1.22]	1.17 [1.09-1.25]	0.98 [0.84-1.15]	1.12 [1.05-1.18]	3,866	3,387	3,030	592	3,712
H91	1.16 [1.13-1.19]	1.16 [1.13-1.19]	1.16 [1.13-1.19]	0.99 [0.87-1.13]	1.15 [1.12-1.18]	1.12 [1.10-1.15]	1.11 [1.08-1.14]	1.11 [1.09-1.14]	0.96 [0.84-1.11]	1.12 [1.10-1.15]	22,799	22,237	21,579	739	21,877
H92	1.29 [1.17-1.42]	1.40 [1.26-1.56]	1.37 [1.22-1.55]	1.00 [0.81-1.24]	1.28 [1.16-1.41]	1.21 [1.10-1.34]	1.28 [1.15-1.43]	1.25 [1.11-1.42]	1.00 [0.81-1.25]	1.22 [1.11-1.35]	1,323	1,107	823	306	1,266
H93	1.22 [1.13-1.32]	1.21 [1.12-1.30]	1.22 [1.13-1.33]	1.35 [0.92-1.98]	1.23 [1.14-1.32]	1.16 [1.08-1.26]	1.13 [1.04-1.22]	1.15 [1.06-1.24]	1.41 [0.94-2.10]	1.18 [1.09-1.27]	2,067	2,043	1,819	109	1,971
H95	1.14 [0.84-1.55]	1.23 [0.90-1.70]			1.06 [0.78-1.43]	1.14 [0.84-1.56]	1.19 [0.86-1.65]			1.04 [0.76-1.42]	128	117	NA	NA	125
Diseases of the circulatory system (I00-I99)															
I05	1.19 [1.08-1.31]	1.20 [1.09-1.32]	1.23 [1.12-1.36]		1.17 [1.07-1.29]	1.14 [1.04-1.25]	1.14 [1.04-1.26]	1.17 [1.06-1.29]		1.15 [1.04-1.26]	1,292	1,296	1,262	NA	1,208
I06	1.22 [0.90-1.64]	1.24 [0.93-1.66]	1.36 [1.01-1.82]		1.11 [0.82-1.49]	1.15 [0.85-1.56]	1.17 [0.87-1.58]	1.28 [0.95-1.72]		1.05 [0.78-1.42]	126	127	126	NA	119
I07	1.21 [1.14-1.29]	1.21 [1.14-1.28]	1.21 [1.14-1.28]		1.18 [1.11-1.25]	1.17 [1.10-1.25]	1.14 [1.07-1.21]	1.14 [1.07-1.21]		1.15 [1.08-1.22]	3,304	3,293	3,227	NA	3,229
I08	1.14 [1.11-1.17]	1.14 [1.11-1.17]	1.14 [1.11-1.17]		1.10 [1.07-1.13]	1.11 [1.08-1.14]	1.08 [1.05-1.11]	1.08 [1.05-1.11]		1.08 [1.05-1.11]	16,519	16,490	16,300	NA	16,138
I09	1.21 [0.95-1.54]	1.25 [0.99-1.58]	1.36 [1.07-1.72]		1.26 [0.99-1.60]	1.18 [0.93-1.51]	1.21 [0.95-1.53]	1.30 [1.02-1.66]		1.23 [0.96-1.57]	199	200	197	NA	190
I10	1.08 [1.07-1.09]	1.08 [1.07-1.09]	1.09 [1.08-1.10]	0.92 [0.78-1.09]	1.08 [1.07-1.09]	1.06 [1.05-1.07]	1.02 [1.01-1.03]	1.02 [1.01-1.03]	0.93 [0.78-1.10]	1.07 [1.05-1.08]	157,034	157,068	153,830	506	119,978
I11	1.07 [0.96-1.20]	1.07 [0.96-1.20]	1.09 [0.98-1.22]		1.00 [0.89-1.12]	1.03 [0.92-1.15]	1.00 [0.90-1.12]	1.01 [0.91-1.13]		0.98 [0.88-1.10]	921	928	919	NA	850
I12	1.16 [1.12-1.20]	1.16 [1.12-1.20]	1.17 [1.13-1.21]		1.12 [1.09-1.16]	1.13 [1.10-1.17]	1.09 [1.06-1.13]	1.10 [1.06-1.14]		1.12 [1.08-1.16]	9,880	9,878	9,802	NA	9,300
I13	1.23 [1.06-1.43]	1.19 [1.02-1.38]	1.17 [1.00-1.36]		1.13 [0.98-1.32]	1.17 [1.00-1.37]	1.09 [0.93-1.28]	1.07 [0.92-1.26]		1.10 [0.95-1.28]	518	518	518	NA	518
I15	1.17 [1.03-1.34]	1.19 [1.04-1.36]	1.15 [1.00-1.32]		1.06 [0.93-1.21]	1.14 [1.00-1.31]	1.11 [0.97-1.27]	1.07 [0.93-1.23]		1.06 [0.93-1.21]	663	629	589	NA	635
I20	1.13 [1.11-1.15]	1.12 [1.10-1.15]	1.13 [1.11-1.15]		1.10 [1.08-1.12]	1.08 [1.06-1.10]	1.04 [1.02-1.06]	1.04 [1.03-1.06]		1.06 [1.04-1.08]	35,262	35,320	35,085	NA	31,682
I21	1.10 [1.07-1.12]	1.08 [1.06-1.11]	1.09 [1.07-1.12]		1.08 [1.05-1.11]	1.07 [1.05-1.10]	1.04 [1.01-1.06]	1.04 [1.02-1.07]		1.07 [1.04-1.09]	21,458	21,437	21,330	NA	19,587
I22	1.15 [1.07-1.24]	1.16 [1.09-1.25]	1.19 [1.11-1.27]		1.15 [1.08-1.24]	1.12 [1.04-1.20]	1.09 [1.01-1.17]	1.11 [1.03-1.19]		1.14 [1.06-1.22]	2,257	2,265	2,268	NA	2,176
I23	1.25 [0.92-1.69]	1.22 [0.91-1.65]	1.35 [0.99-1.83]		1.36 [1.00-1.85]	1.23 [0.91-1.67]	1.14 [0.84-1.55]	1.32 [0.96-1.80]		1.33 [0.98-1.82]	123	124	123	NA	115
I24	1.13 [1.09-1.18]	1.13 [1.08-1.18]	1.15 [1.11-1.20]		1.12 [1.08-1.17]	1.09 [1.05-1.14]	1.06 [1.01-1.10]	1.07 [1.02-1.11]		1.10 [1.05-1.14]	6,864	6,833	6,812	NA	6,691
I25	1.10 [1.09-1.12]	1.10 [1.09-1.12]	1.10 [1.09-1.12]		1.08 [1.07-1.10]	1.06 [1.05-1.08]	1.03 [1.02-1.05]	1.03 [1.02-1.05]		1.06 [1.04-1.07]	59,154	59,160	58,817	NA	53,341
I26	1.09 [1.05-1.12]	1.07 [1.04-1.11]	1.07 [1.04-1.11]		1.06 [1.02-1.09]	1.05 [1.02-1.09]	1.02 [0.98-1.05]	1.02 [0.99-1.06]		1.03 [0.99-1.06]	10,547	10,591	10,054	NA	9,785
I27	1.21 [1.16-1.26]	1.20 [1.16-1.25]	1.20 [1.15-1.25]		1.19 [1.14-1.24]	1.15 [1.11-1.20]	1.12 [1.08-1.17]	1.11 [1.07-1.15]		1.15 [1.11-1.20]	7,864	7,862	7,772	NA	7,790
I28	1.07 [0.83-1.39]	1.01 [0.78-1.30]	1.03 [0.78-1.34]		0.98 [0.76-1.25]	1.01 [0.77-1.31]	0.91 [0.70-1.19]	0.93 [0.71-1.23]		0.90 [0.69-1.16]	174	165	158	NA	176
I30	1.05 [0.85-1.29]	1.07 [0.87-1.30]	1.06 [0.85-1.32]		1.09 [0.88-1.34]	1.03 [0.84-1.27]	1.02 [0.83-1.25]	1.02 [0.81-1.28]		1.07 [0.86-1.32]	272	270	218	NA	241
I31	1.15 [1.09-1.21]	1.13 [1.07-1.19]	1.15 [1.09-1.22]	0.90 [0.64-1.27]	1.08 [1.02-1.14]	1.11 [1.05-1.17]	1.07 [1.01-1.13]	1.08 [1.02-1.15]	0.93 [0.65-1.34]	1.05 [0.99-1.11]	4,133	4,095	3,840	104	3,884
I33	1.28 [1.13-1.45]	1.20 [1.06-1.37]	1.20 [1.05-1.36]		1.18 [1.04-1.34]	1.22 [1.08-1.39]	1.14 [1.00-1.30]	1.13 [0.99-1.29]		1.14 [1.00-1.30]	730	727	696	NA	694
I34	1.09 [1.06-1.13]	1.10 [1.07-1.14]	1.10 [1.06-1.14]		1.08 [1.04-1.11]	1.06 [1.02-1.09]	1.05 [1.02-1.09]	1.04 [1.01-1.08]		1.05 [1.02-1.09]	10,125	10,126	9,857	NA	9,734
I35	1.15 [1.12-1.18]	1.14 [1.11-1.17]	1.15 [1.12-1.19]		1.12 [1.09-1.15]	1.11 [1.08-1.14]	1.08 [1.05-1.11]	1.09 [1.06-1.12]		1.09 [1.06-1.12]	15,731	15,697	15,580	NA	14,969
I36	1.14 [1.03-1.26]	1.11 [1.00-1.23]	1.12 [1.01-1.24]		1.10 [0.99-1.21]	1.08 [0.97-1.20]	1.05 [0.95-1.17]	1.06 [0.95-1.18]		1.07 [0.97-1.19]	1,131	1,117	1,089	NA	1,122
I37	1.16 [1.05-1.29]	1.18 [1.07-1.31]	1.19 [1.07-1.32]		1.10 [1.00-1.22]	1.12 [1.00-1.24]	1.13 [1.02-1.26]	1.11 [0.99-1.24]		1.07 [0.97-1.19]	1,164	1,156	1,075	NA	1,184
I38	1.21 [1.11-1.31]	1.21 [1.11-1.32]	1.24 [1.14-1.35]		1.15 [1.06-1.25]	1.16 [1.07-1.26]	1.15 [1.05-1.25]	1.17 [1.07-1.27]		1.11 [1.02-1.21]	1,735	1,703	1,663	NA	1,639
I40	1.19 [0.83-1.69]				1.46 [1.03-2.05]	1.15 [0.81-1.65]				1.39 [0.97-1.99]	105	NA	NA	NA	109
I42	1.11 [1.05-1.18]	1.14 [1.07-1.20]	1.09 [1.03-1.16]		1.11 [1.05-1.18]	1.06 [1.00-1.13]	1.06 [1.00-1.12]	1.02 [0.96-1.08]		1.07 [1.01-1.13]	3,509	3,494	3,301	NA	3,416
I43	0.96 [0.72-1.28]	0.98 [0.73-1.32]	0.91 [0.68-1.22]		0.99 [0.75-1.30]	0.93 [0.69-1.25]	0.92 [0.68-1.25]	0.85 [0.63-1.15]		0.95 [0.72-1.26]	132	131	126	NA	138

Supplementary Table 4: Hazard ratios and events from all cohorts (excluding non-consulters)

	Hazard ratio (99% confidence interval)														
	crude					adjusted					Events (in exposed)				
Outcome	any age	18+	40+	<18	hosp.	any age	18+	40+	<18	hosp.	any age	18+	40+	<18	hosp.
I44	1.12 [1.10-1.15]	1.12 [1.10-1.15]	1.12 [1.10-1.15]		1.11 [1.09-1.14]	1.09 [1.07-1.12]	1.07 [1.05-1.10]	1.07 [1.05-1.10]		1.09 [1.07-1.12]	24,310	24,301	24,127	NA	23,421
I45	1.13 [1.10-1.16]	1.12 [1.09-1.15]	1.13 [1.09-1.16]	0.95 [0.72-1.25]	1.11 [1.08-1.14]	1.09 [1.06-1.12]	1.06 [1.03-1.09]	1.07 [1.03-1.10]	0.92 [0.69-1.24]	1.09 [1.05-1.12]	14,712	14,713	14,244	171	14,182
I46	1.15 [1.11-1.20]	1.16 [1.12-1.21]	1.15 [1.11-1.20]		1.14 [1.10-1.19]	1.13 [1.08-1.17]	1.11 [1.06-1.15]	1.10 [1.05-1.14]		1.13 [1.08-1.18]	7,450	7,442	7,219	NA	7,099
I47	1.13 [1.08-1.17]	1.12 [1.08-1.16]	1.13 [1.08-1.17]	0.89 [0.68-1.17]	1.09 [1.04-1.13]	1.08 [1.03-1.12]	1.05 [1.01-1.09]	1.06 [1.01-1.10]	0.90 [0.68-1.21]	1.05 [1.01-1.09]	7,921	7,881	7,256	176	7,406
I48	1.09 [1.07-1.10]	1.09 [1.07-1.10]	1.09 [1.07-1.10]		1.08 [1.06-1.09]	1.06 [1.05-1.08]	1.05 [1.03-1.06]	1.05 [1.03-1.06]		1.06 [1.05-1.08]	65,261	65,329	64,987	NA	59,478
I49	1.09 [1.05-1.13]	1.09 [1.06-1.13]	1.09 [1.05-1.13]	0.93 [0.71-1.22]	1.06 [1.03-1.10]	1.05 [1.01-1.08]	1.03 [0.99-1.06]	1.03 [0.99-1.07]	0.84 [0.63-1.12]	1.03 [1.00-1.07]	9,678	9,684	9,055	187	9,306
I50	1.17 [1.15-1.19]	1.17 [1.15-1.19]	1.18 [1.16-1.19]		1.16 [1.14-1.18]	1.13 [1.11-1.15]	1.10 [1.08-1.11]	1.10 [1.08-1.12]		1.13 [1.11-1.15]	53,392	53,464	53,159	NA	51,208
I51	1.15 [1.13-1.18]	1.15 [1.13-1.18]	1.15 [1.13-1.17]	0.86 [0.66-1.12]	1.12 [1.10-1.15]	1.11 [1.08-1.13]	1.08 [1.06-1.10]	1.07 [1.05-1.10]	0.85 [0.65-1.12]	1.09 [1.07-1.12]	28,705	28,639	28,163	175	28,071
I60	1.15 [1.05-1.26]	1.16 [1.05-1.27]	1.11 [1.01-1.22]		1.08 [0.99-1.19]	1.14 [1.04-1.26]	1.13 [1.03-1.24]	1.08 [0.98-1.19]		1.09 [0.99-1.20]	1,315	1,323	1,242	NA	1,165
I61	1.15 [1.09-1.22]	1.14 [1.07-1.20]	1.13 [1.07-1.20]		1.13 [1.07-1.19]	1.15 [1.09-1.21]	1.12 [1.06-1.18]	1.10 [1.04-1.17]		1.13 [1.07-1.19]	3,764	3,734	3,682	NA	3,472
I62	1.10 [1.03-1.19]	1.13 [1.05-1.22]	1.11 [1.03-1.20]		1.05 [0.98-1.13]	1.09 [1.02-1.18]	1.11 [1.03-1.19]	1.08 [1.00-1.16]		1.06 [0.98-1.14]	2,240	2,234	2,208	NA	2,095
I63	1.04 [1.02-1.07]	1.04 [1.02-1.07]	1.04 [1.01-1.07]		1.04 [1.01-1.06]	1.04 [1.01-1.06]	1.02 [0.99-1.04]	1.01 [0.99-1.04]		1.04 [1.01-1.07]	17,592	17,525	17,374	NA	16,278
I64	1.09 [1.04-1.14]	1.10 [1.04-1.15]	1.09 [1.04-1.14]		1.10 [1.04-1.16]	1.08 [1.02-1.13]	1.06 [1.01-1.12]	1.05 [1.00-1.10]		1.10 [1.04-1.16]	4,490	4,502	4,471	NA	4,030
I65	1.10 [1.05-1.15]	1.08 [1.03-1.14]	1.10 [1.04-1.15]		1.08 [1.02-1.13]	1.08 [1.02-1.13]	1.03 [0.98-1.08]	1.03 [0.98-1.09]		1.07 [1.02-1.12]	4,569	4,530	4,546	NA	4,288
I66	1.12 [0.90-1.37]	1.06 [0.86-1.31]	1.05 [0.85-1.30]		1.03 [0.84-1.26]	1.09 [0.89-1.35]	1.02 [0.82-1.27]	0.99 [0.79-1.23]		1.01 [0.82-1.24]	262	254	244	NA	258
I67	1.11 [1.09-1.13]	1.11 [1.09-1.14]	1.11 [1.09-1.14]		1.10 [1.08-1.12]	1.09 [1.07-1.11]	1.07 [1.05-1.09]	1.06 [1.04-1.09]		1.09 [1.07-1.11]	29,676	29,648	29,521	NA	28,813
I68	1.17 [0.91-1.52]	1.12 [0.87-1.45]	1.18 [0.92-1.53]		1.14 [0.88-1.48]	1.15 [0.88-1.49]	1.08 [0.83-1.41]	1.11 [0.86-1.45]		1.10 [0.85-1.43]	173	170	170	NA	165
I69	1.10 [1.06-1.14]	1.10 [1.06-1.14]	1.09 [1.05-1.13]		1.04 [1.01-1.08]	1.08 [1.04-1.12]	1.06 [1.02-1.10]	1.04 [1.00-1.08]		1.05 [1.01-1.08]	8,442	8,401	8,317	NA	8,146
I70	1.16 [1.11-1.21]	1.15 [1.10-1.19]	1.17 [1.12-1.21]		1.12 [1.08-1.16]	1.13 [1.09-1.18]	1.08 [1.04-1.12]	1.09 [1.05-1.13]		1.12 [1.07-1.16]	7,561	7,587	7,572	NA	7,291
I71	1.11 [1.07-1.15]	1.10 [1.06-1.14]	1.11 [1.07-1.15]		1.08 [1.04-1.12]	1.10 [1.06-1.15]	1.06 [1.03-1.10]	1.06 [1.03-1.11]		1.08 [1.04-1.12]	8,528	8,510	8,457	NA	8,059
I72	1.05 [0.97-1.13]	1.05 [0.98-1.14]	1.08 [1.00-1.17]		1.06 [0.98-1.15]	1.03 [0.95-1.11]	1.00 [0.93-1.08]	1.02 [0.94-1.10]		1.05 [0.98-1.14]	1,971	1,971	1,912	NA	1,891
I73	1.27 [1.24-1.31]	1.27 [1.24-1.30]	1.26 [1.23-1.30]	1.13 [0.84-1.53]	1.21 [1.18-1.25]	1.22 [1.19-1.25]	1.17 [1.13-1.20]	1.16 [1.13-1.19]	1.09 [0.79-1.50]	1.18 [1.15-1.21]	17,224	17,298	16,664	146	16,427
I74	1.08 [1.03-1.15]	1.07 [1.01-1.13]	1.10 [1.05-1.17]		1.06 [1.00-1.12]	1.06 [1.00-1.12]	1.01 [0.95-1.06]	1.03 [0.98-1.09]		1.05 [1.00-1.11]	3,879	3,877	3,819	NA	3,675
I77	1.20 [1.15-1.26]	1.19 [1.13-1.24]	1.21 [1.16-1.27]		1.16 [1.11-1.21]	1.17 [1.12-1.22]	1.11 [1.06-1.16]	1.13 [1.08-1.18]		1.15 [1.10-1.21]	5,583	5,581	5,454	NA	5,331
I78	1.24 [1.13-1.36]	1.26 [1.15-1.39]	1.21 [1.10-1.34]		1.18 [1.07-1.29]	1.19 [1.09-1.31]	1.19 [1.08-1.30]	1.15 [1.04-1.27]		1.14 [1.04-1.25]	1,335	1,335	1,267	NA	1,285
I79	1.14 [1.00-1.31]	1.15 [1.00-1.31]	1.13 [0.99-1.29]		1.08 [0.94-1.24]	1.09 [0.95-1.25]	1.02 [0.88-1.17]	1.01 [0.87-1.16]		1.05 [0.91-1.20]	613	605	600	NA	586
I80	1.19 [1.15-1.23]	1.19 [1.15-1.23]	1.19 [1.15-1.23]		1.17 [1.13-1.21]	1.15 [1.11-1.19]	1.13 [1.09-1.17]	1.13 [1.09-1.17]		1.13 [1.09-1.17]	9,973	9,998	9,379	NA	9,315
I81	1.07 [0.92-1.23]	1.11 [0.96-1.28]	1.06 [0.92-1.23]		0.99 [0.86-1.15]	1.03 [0.89-1.19]	1.07 [0.92-1.23]	1.01 [0.87-1.18]		1.00 [0.86-1.15]	526	541	510	NA	506
I82	1.12 [1.01-1.23]	1.14 [1.03-1.26]	1.15 [1.03-1.27]		1.07 [0.97-1.18]	1.08 [0.98-1.19]	1.08 [0.98-1.19]	1.07 [0.96-1.19]		1.04 [0.94-1.15]	1,206	1,215	1,069	NA	1,156
I83	1.53 [1.49-1.58]	1.55 [1.51-1.60]	1.57 [1.52-1.62]		1.50 [1.46-1.55]	1.50 [1.45-1.54]	1.50 [1.46-1.55]	1.51 [1.46-1.56]		1.47 [1.42-1.52]	13,662	13,715	12,449	NA	12,335
I84	1.24 [1.21-1.27]	1.24 [1.20-1.27]	1.22 [1.19-1.25]	1.08 [0.86-1.37]	1.20 [1.17-1.23]	1.18 [1.15-1.21]	1.16 [1.13-1.19]	1.15 [1.12-1.18]	1.04 [0.81-1.33]	1.15 [1.12-1.18]	17,611	17,688	15,413	230	15,824
I85	1.34 [1.23-1.45]	1.31 [1.21-1.43]	1.34 [1.23-1.45]		1.32 [1.22-1.43]	1.29 [1.18-1.40]	1.22 [1.12-1.33]	1.23 [1.12-1.34]		1.30 [1.19-1.41]	1,692	1,691	1,617	NA	1,657
I86	1.21 [1.09-1.35]	1.25 [1.12-1.39]	1.27 [1.13-1.43]		1.17 [1.05-1.30]	1.15 [1.03-1.28]	1.17 [1.05-1.31]	1.18 [1.05-1.33]		1.15 [1.03-1.28]	1,037	1,024	862	NA	986
I87	1.50 [1.41-1.59]	1.49 [1.40-1.58]	1.54 [1.44-1.64]		1.46 [1.37-1.55]	1.44 [1.35-1.53]	1.40 [1.31-1.49]	1.45 [1.36-1.55]		1.43 [1.34-1.52]	3,287	3,285	3,104	NA	3,171
I88	1.18 [1.04-1.33]	1.26 [1.05-1.52]	1.38 [1.09-1.74]	1.18 [1.02-1.38]	1.14 [1.01-1.29]	1.16 [1.02-1.31]	1.17 [0.97-1.41]	1.35 [1.06-1.71]	1.17 [1.00-1.36]	1.12 [0.98-1.27]	941	367	222	630	815
I89	1.40 [1.33-1.48]	1.39 [1.31-1.46]	1.41 [1.33-1.49]		1.36 [1.29-1.44]	1.34 [1.26-1.41]	1.29 [1.22-1.36]	1.30 [1.23-1.38]		1.32 [1.25-1.40]	4,178	4,197	4,037	NA	4,095
I95	1.12 [1.10-1.14]	1.12 [1.10-1.14]	1.12 [1.10-1.14]	0.92 [0.76-1.11]	1.10 [1.08-1.12]	1.09 [1.07-1.11]	1.07 [1.05-1.09]	1.06 [1.04-1.08]	0.91 [0.74-1.10]	1.09 [1.07-1.11]	35,050	35,052	33,770	379	34,239
I97	1.22 [1.04-1.43]	1.22 [1.04-1.43]	1.19 [1.01-1.41]		1.15 [0.98-1.35]	1.19 [1.01-1.40]	1.16 [0.99-1.37]	1.15 [0.97-1.36]		1.15 [0.98-1.36]	428	427	410	NA	407
I98	1.30 [1.17-1.44]	1.34 [1.21-1.48]	1.32 [1.19-1.46]		1.27 [1.15-1.41]	1.23 [1.11-1.37]	1.25 [1.12-1.38]	1.21 [1.09-1.34]		1.25 [1.13-1.38]	1,123	1,146	1,088	NA	1,127
I99	1.12 [0.98-1.29]	1.12 [0.98-1.28]	1.16 [1.02-1.33]		1.11 [0.97-1.27]	1.10 [0.96-1.26]	1.06 [0.92-1.21]	1.10 [0.96-1.27]		1.10 [0.96-1.26]	624	624	603	NA	594
Diseases of the respiratory system (J00-J99)															
J00	1.31 [1.19-1.43]	1.24 [1.09-1.40]	1.33 [1.15-1.54]	1.35 [1.18-1.54]	1.29 [1.17-1.42]	1.25 [1.14-1.38]	1.15 [1.01-1.31]	1.25 [1.07-1.45]	1.30 [1.14-1.49]	1.26 [1.15-1.39]	1,449	739	527	764	1,355
J01	1.24 [1.04-1.46]	1.25 [1.05-1.48]	1.17 [0.95-1.44]		1.15 [0.97-1.35]	1.11 [0.93-1.33]	1.11 [0.93-1.33]	1.02 [0.83-1.27]		1.07 [0.90-1.27]	434	413	272	NA	409
J02	1.27 [1.19-1.36]	1.27 [1.19-1.37]	1.31 [1.20-1.43]	1.04 [0.91-1.19]	1.21 [1.14-1.30]	1.20 [1.12-1.28]	1.16 [1.08-1.25]	1.19 [1.08-1.30]	1.01 [0.88-1.16]	1.17 [1.09-1.25]	2,827	2,344	1,499	783	2,640
J03	1.19 [1.14-1.23]	1.18 [1.12-1.24]	1.16 [1.03-1.31]	1.14 [1.09-1.19]	1.14 [1.10-1.19]	1.14 [1.09-1.18]	1.08 [1.03-1.14]	1.02 [0.90-1.16]	1.11 [1.06-1.16]	1.11 [1.06-1.15]	9,686	4,572	755	6,950	8,569
J04	1.20 [1.01-1.42]	1.37 [1.15-1.64]	1.25 [1.02-1.52]		1.29 [1.08-1.53]	1.07 [0.90-1.28]	1.20 [0.99-1.44]	1.07 [0.87-1.32]		1.20 [1.00-1.43]	420	382	293	NA	386
J05	1.00 [0.92-1.09]	0.97 [0.73-1.30]	1.15 [0.84-1.56]	1.01 [0.93-1.11]	1.01 [0.92-1.10]	0.98 [0.90-1.07]	0.91 [0.68-1.22]	1.02 [0.74-1.41]	0.99 [0.90-1.08]	0.99 [0.90-1.08]	1,540	135	118	1,417	1,401
J06	1.28 [1.23-1.33]	1.26 [1.18-1.35]	1.26 [1.17-1.36]	1.28 [1.22-1.34]	1.25 [1.20-1.30]	1.22 [1.17-1.27]	1.12 [1.05-1.20]	1.14 [1.05-1.23]	1.25 [1.19-1.31]	1.20 [1.15-1.25]	7,951	2,718	1,916	5,508	7,331
J10	1.22 [1.15-1.30]	1.25 [1.17-1.34]	1.24 [1.16-1.33]	1.17 [0.96-1.43]	1.22 [1.15-1.30]	1.13 [1.06-1.20]	1.11 [1.04-1.19]	1.11 [1.03-1.19]	1.11 [0.90-1.36]	1.14 [1.07-1.22]	3,180	2,962	2,556	344	3,138
J11	1.17 [1.01-1.35]	1.19 [1.02-1.37]	1.24 [1.05-1.46]		1.15 [1.00-1.33]	1.07 [0.92-1.24]	1.06 [0.91-1.23]	1.11 [0.94-1.32]		1.09 [0.94-1.26]	588	556	437	NA	576
J12	1.16 [1.11-1.22]	1.16 [1.10-1.21]	1.17 [1.12-1.23]	1.06 [0.81-1.37]	1.16 [1.11-1.22]	1.10 [1.04-1.15]	1.07 [1.02-1.13]	1.08 [1.03-1.14]	1.00 [0.76-1.31]	1.12 [1.06-1.17]	5,383	5,253	5,042	188	5,318
J13	1.23 [1.10-1.36]	1.30 [1.17-1.44]	1.26 [1.13-1.40]		1.29 [1.16-1.43]	1.14 [1.03-1.27]	1.18 [1.06-1.31]	1.13 [1.01-1.26]		1.21 [1.09-1.35]	1,106	1,094	1,028	NA	1,034
J14	1.23 [1.05-1.44]	1.30 [1.11-1.53]	1.24 [1.05-1.45]		1.25 [1.07-1.46]	1.10 [0.94-1.30]	1.11 [0.94-1.31]	1.04 [0.88-1.23]		1.12 [0.96-1.31]	482	469	452	NA	477
J15	1.28 [1.19-1.37]	1.30 [1.21-1.40]	1.30 [1.21-1.40]	1.28 [0.91-1.79]	1.30 [1.21-1.39]	1.18 [1.10-1.27]	1.18 [1.10-1.27]	1.16 [1.08-1.25]	1.27 [0.90-1.79]	1.22 [1.14-1.31]	2,530	2,435	2,344	123	2,443
J17	1.34 [1.15-1.56]	1.32 [1.13-1.53]	1.23 [1.05-1.44]		1.27 [1.09-1.48]	1.26 [1.08-1.47]	1.22 [1.04-1.43]	1.13 [0.96-1.33]		1.19 [1.02-1.40]	498	495	469	NA	477
J18	1.18 [1.17-1.20]	1.18 [1.16-1.19]	1.18 [1.16-1.19]	1.20 [1.11-1.30]	1.16 [1.15-1.18]	1.14 [1.12-1.15]	1.11 [1.09-1.12]	1.11 [1.09-1.12]	1.18 [1.09-1.27]	1.13 [1.12-1.15]	72,453	70,790	68,632	2,192	69,584
J20	1.21 [1.08-1.35]	1.21 [1.08-1.36]	1.22 [1.08-1.38]		1.22 [1.09-1.36]	1.11 [0.99-1.25]	1.09 [0.97-1.23]	1.11 [0.98-1.27]		1.13 [1.00-1.27]	926	855	777	NA	894
J21	1.17 [1.01-1.35]	1.28 [1.02-1.59]	1.22 [0.97-1.54]	1.05 [0.87-1.26]	1.13 [0.98-1.31]	1.09 [0.94-1.27]	1.11 [0.88-1.40]	1.09 [0.86-1.39]	0.99 [0.82-1.20]	1.04 [0.89-1.20]	564	240	219	330	523
J22	1.24 [1.22-1.26]	1.23 [1.21-1.25]	1.21 [1.19-1.23]	1.35 [1.28-1.42]	1.20 [1.18-1.22]	1.18 [1.16-1.20]	1.14 [1.12-1.16]	1.13 [1.11-1.15]	1.29 [1.22-1.36]	1.16 [1.14-1.18]	44,787	40,875	37,563	4,816	42,632
J30	1.77 [1.69-1.85]	1.58 [1.51-1.66]	1.50 [1.42-1.58]	2.91 [2.64-3.21]	1.73 [1.66-1.80]	1.45 [1.38-1.51]	1.26 [1.20-1.32]	1.23 [1.15-1.30]	2.45 [2.22-2.72]	1.41 [1.35-1.48]	8,255	6,685	4,111	2,475	7,484
J31	1.50 [1.37-1.63]	1.54 [1.41-1.69]	1.46 [1.31-1.62]	1.72 [1.34-2.20]	1.46 [1.34-1.60]	1.25 [1.14-1.37]	1.28 [1.16-1.41]	1.19 [1.07-1.33]	1.45 [1.12-1.89]	1.23 [1.12-1.35]	1,726	1,577	1,128	280	1,598
J32	1.26 [1.19-1.33]	1.29 [1.22-1.37]	1.27 [1.19-1.36]	1.23 [1.00-1.52]	1.24 [1.17-1.31]	1.10 [1.04-1.17]	1.11 [1.04-1.18]	1.12 [1.05-1.19]	1.15 [0.92-1.43]	1.10 [1.04-1.17]	3,916	3,815	2,877	326	3,615
J33	1.40 [1.30-1.50]	1.37 [1.28-1.47]	1.37 [1.27-1.48]	1.87 [1.28-2.73]	1.33 [1.24-1.43]	1.20 [1.12-1.29]	1.16 [1.08-1.25]	1.18 [1.09-1.28]	1.71 [1.15-2.55]	1.15 [1.07-1.24]	2,619	2,550	2,082	117	2,194
J34	1.32 [1.27-1.38]	1.30 [1.25-1.36]	1.30 [1.23-1.37]	1.37 [1.23-1.52]	1.28 [1.23-1.34]	1.20 [1.15-1.25]	1.15 [1.10-1.21]	1.17 [1.11-1.23]	1.28 [1.15-1.43]	1.17 [1.12-1.22]	7,296	6,696	4,250	1,370	6,322
J35	1.18 [1.13-1.24]	1.19 [1.11-1.28]	1.14 [1.01-1.29]	1.19 [1.13-1.26]	1.17 [1.11-1.22]	1.14 [1.09-1.19]	1.12 [1.04-1.20]	1.05 [0.93-1.19]	1.16 [1.10-1.22]	1.13 [1.08-1.19]	6,855	2,312	720	5,158	5,798
J36	1.05 [0.93-1.17]	1.06 [0.95-1.18]	1.07 [0.88-1.30]	1.10 [0.87-1.39]	1.05 [0.93-1.18]	1.05 [0.93-1.18]	1.06 [0.95-1.19]	1.05 [0.87-1.28]	1.13 [0.88-1.45]	1.05 [0.93-1.19]	939	951	301	244	811
J38	1.20 [1.13-1.28]	1.21 [1.14-1.29]	1.23 [1.15-1.31]	1.13 [0.87-1.47]	1.15 [1.08-1.23]	1.14 [1.07-1.22]	1.09 [1.02-1.17]	1.13 [1.06-1.21]	1.05 [0.80-1.38]	1.10 [1.03-1.17]	3,002	2,897	2,632	187	2,757
J39	1.20 [1.10-1.31]	1.21 [1.11-1.32]	1.19 [1.08-1.31]	1.09 [0.81-1.46]	1.14 [1.05-1.24]	1.12 [1.03-1.23]	1.10 [1.00-1.20]	1.09 [0.99-1.20]	1.03 [0.76-1.39]	1.08 [0.99-1.18]	1,589	1,484	1,308	157	1,519
J40	1.31 [1.21-1.41]	1.30 [1.21-1.40]	1.30 [1.20-1.40]		1.23 [1.14-1.32]	1.19 [1.10-1.28]	1.15 [1.07-1.25]	1.15 [1.06-1.24]		1.12 [1.04-1.21]	2,142	2,125	1,985	NA	1,999
J42	1.37 [1.22-1.54]	1.35 [1.20-1.52]	1.31 [1.16-1.47]		1.31 [1.17-1.47]	1.23 [1.10-1.39]	1.19 [1.06-1.34]	1.13 [1.00-1.28]		1.18 [1.04-1.33]	897	880	853	NA	839
J43	1.27 [1.23-1.31]	1.26 [1.22-1.30]	1.27 [1.23-1.31]		1.24 [1.21-1.28]	1.19 [1.15-1.23]	1.13 [1.10-1.17]	1.15 [1.11-1.18]		1.18 [1.14-1.22]	11,572	11,588	11,449	NA	11,218
J44	1.32 [1.30-1.34]	1.32 [1.30-1.34]	1.31 [1.29-1.34]		1.29 [1.27-1.31]	1.19 [1.17-1.21]	1.14 [1.12-1.16]	1.14 [1.12-1.16]		1.17 [1.15-1.19]	49,128	49,132	48,741	NA	45,504
J45	1.72 [1.69-1.74]	1.65 [1.63-1.68]	1.57 [1.54-1.59]	2.22 [2.14-2.29]	1.65 [1.63-1.67]	1.32 [1.30-1.34]	1.21 [1.20-1.23]	1.20 [1.18-1.22]	1.86 [1.79-1.93]	1.29 [1.27-1.31]	85,315	77,417	53,422	15,741	68,712
J46	2.24 [2.01-2.48]	1.81 [1.61-2.05]	1.67 [1.44-1.94]	3.34 [2.75-4.04]	2.05 [1.84-2.28]	1.74 [1.54-1.95]	1.29 [1.12-1.50]	1.17 [0.98-1.39]	2.85 [2.33-3.48]	1.57 [1.40-1.77]	1,451	952	574	616	1,323
J47	1.34 [1.30-1.39]	1.35 [1.30-1.40]	1.33 [1.29-1.38]		1.32 [1.27-1.37]	1.19 [1.14-1.23]	1.17 [1.13-1.22]	1.17 [1.12-1.21]		1.17 [1.12-1.21]	9,197	9,196	8,977	NA	8,996
J61	1.28 [1.16-1.41]	1.30 [1.18-1.44]	1.29 [1.16-1.42]		1.29 [1.17-1.42]	1.22 [1.10-1.35]	1.23 [1.11-1.36]	1.20 [1.09-1.33]		1.23 [1.12-1.36]	1,223	1,228	1,215	NA	1,212
J67	1.39 [1.16-1.66]	1.45 [1.21-1.73]	1.38 [1.15-1.66]		1.33 [1.11-1.59]	1.27 [1.05-1.53]	1.26 [1.04-1.52]	1.26 [1.04-1.52]		1.22 [1.01-1.46]	375	380	350	NA	351
J69	1.09 [1.06-1.13]	1.10 [1.06-1.14]	1.11 [1.07-1.14]		1.10 [1.06-1.14]	1.09 [1.05-1.13]	1.08 [1.04-1.11]	1.08 [1.04-1.11]		1.11 [1.07-1.15]	10,736	10,688	10,511	NA	10,551
J70	1.40 [1.13-1.74]	1.37 [1.11-1.70]	1.44 [1.15-1.79]		1.29 [1.04-1.60]	1.31 [1.05-1.63]	1.25 [1.00-1.56]	1.33 [1.06-1.67]		1.22 [0.98-1.52]	247	249	243	NA	243
J80	1.27 [1.13-1.43]	1.29 [1.15-1.46]	1.30 [1.15-1.47]		1.26 [1.12-1.42]	1.22 [1.08-1.38]	1.23 [1.09-1.39]	1.21 [1.07-1.38]		1.26 [1.12-1.42]	844	832	779	NA	803
J81	1.20 [1.14-1.27]	1.20 [1.14-1.27]	1.20 [1.14-1.27]		1.15 [1.09-1.21]	1.17 [1.11-1.24]	1.13 [1.07-1.20]	1.14 [1.08-1.21]		1.13 [1.07-1.20]	3,873	3,907	3,819	NA	3,671
J82	1.86 [1.54-2.25]	1.85 [1.53-2.23]	1.68 [1.37-2.05]		1.83 [1.52-2.21]	1.35 [1.09-1.68]	1.35 [1.09-1.68]	1.23 [0.98-1.54]		1.46 [1.19-1.79]	404	418	332	NA	399
J84	1.30 [1.25-1.35]	1.33 [1.28-1.38]	1.31 [1.26-1.36]		1.32 [1.27-1.37]	1.25 [1.20-1.30]	1.25 [1.20-1.30]	1.24 [1.19-1.29]		1.28 [1.23-1.33]	7,855	7,858	7,775	NA	7,625
J85	1.30 [1.08-1.56]	1.33 [1.10-1.61]	1.26 [1.04-1.52]		1.27 [1.06-1.53]	1.21 [1.00-1.46]	1.24 [1.02-1.51]	1.17 [0.96-1.43]		1.23 [1.02-1.48]	344	331	314	NA	323
J86	1.18 [1.07-1.31]	1.22 [1.10-1.36]	1.21 [1.09-1.35]		1.13 [1.02-1.25]	1.16 [1.04-1.28]	1.17 [1.05-1.30]	1.14 [1.02-1.28]		1.12 [1.01-1.24]	1,118	1,066	1,004	NA	1,021
J90	1.13 [1.11-1.15]	1.12 [1.10-1.15]	1.13 [1.11-1.15]	0.99 [0.81-1.20]	1.11 [1.09-1.13]	1.11 [1.08-1.13]	1.08 [1.06-1.10]	1.08 [1.06-1.10]	1.04 [0.84-1.28]	1.10 [1.08-1.12]	34,393	34,273	33,495	323	33,010
J91	1.19 [1.05-1.34]	1.15 [1.02-1.30]	1.17 [1.03-1.32]		1.19 [1.05-1.34]	1.16 [1.03-1.32]	1.09 [0.96-1.23]	1.10 [0.97-1.24]		1.18 [1.05-1.33]	822	822	802	NA	813
J92	1.20 [1.14-1.27]	1.23 [1.16-1.30]	1.22 [1.15-1.28]		1.19 [1.13-1.25]	1.17 [1.11-1.24]	1.18 [1.12-1.25]	1.17 [1.11-1.24]		1.17 [1.10-1.23]	4,095	4,094	4,070	NA	4,001
J93	1.08 [1.01-1.16]	1.07 [1.00-1.14]	1.08 [1.01-1.16]	0.97 [0.69-1.35]	1.06 [0.99-1.14]	1.06 [0.99-1.13]	1.03 [0.96-1.10]	1.04 [0.97-1.12]	1.00 [0.71-1.41]	1.05 [0.98-1.13]	2,609	2,543	2,317	121	2,414
J94	1.18 [1.08-1.30]	1.16 [1.05-1.28]	1.16 [1.05-1.28]		1.10 [1.00-1.21]	1.16 [1.06-1.28]	1.11 [1.00-1.22]	1.10 [1.00-1.21]		1.09 [0.99-1.20]	1,258	1,254	1,219	NA	1,197
J95	1.14 [1.02-1.27]	1.08 [0.97-1.20]	1.12 [1.00-1.25]		1.11 [0.99-1.24]	1.12 [1.00-1.24]	1.01 [0.91-1.13]	1.07 [0.95-1.20]		1.10 [0.99-1.23]	924	894	868	NA	847
J96	1.22 [1.19-1.24]	1.20 [1.18-1.23]	1.20 [1.18-1.23]	1.01 [0.78-1.32]	1.19 [1.17-1.22]	1.15 [1.13-1.18]	1.10 [1.08-1.13]	1.10 [1.08-1.13]	0.95 [0.72-1.26]	1.15 [1.12-1.17]	23,696	23,699	23,196	183	23,167
J98	1.16 [1.14-1.19]	1.15 [1.12-1.18]	1.15 [1.13-1.18]	1.19 [1.03-1.37]	1.14 [1.11-1.16]	1.10 [1.08-1.13]	1.06 [1.04-1.09]	1.07 [1.04-1.10]	1.13 [0.97-1.30]	1.10 [1.07-1.12]	20,600	20,132	19,337	685	20,025
J99	1.44 [1.26-1.64]	1.44 [1.27-1.64]	1.46 [1.27-1.66]		1.39 [1.22-1.57]	1.27 [1.11-1.45]	1.29 [1.13-1.48]	1.32 [1.15-1.51]		1.24 [1.09-1.42]	722	724	667	NA	729
Diseases of the digestive system (K00-K95)															
K00	1.15 [1.04-1.26]	1.13 [1.00-1.28]	1.17 [0.91-1.50]	1.11 [0.99-1.26]	1.06 [0.96-1.18]	1.11 [1.01-1.22]	1.08 [0.94-1.23]	1.09 [0.84-1.41]	1.09 [0.96-1.23]	1.05 [0.95-1.17]	1,578	766	178	1,019	1,265
K01	1.15 [1.10-1.21]	1.15 [1.10-1.21]	1.09 [0.97-1.22]	1.15 [1.05-1.26]	1.09 [1.03-1.15]	1.10 [1.05-1.16]	1.08 [1.03-1.13]	1.05 [0.94-1.18]	1.11 [1.01-1.22]	1.05 [0.99-1.10]	5,622	5,317	854	1,757	4,342
K02	1.11 [1.08-1.14]	1.16 [1.13-1.20]	1.19 [1.14-1.25]	0.95 [0.91-1.00]	1.07 [1.04-1.10]	1.06 [1.03-1.10]	1.07 [1.03-1.11]	1.10 [1.05-1.15]	0.95 [0.90-1.00]	1.04 [1.01-1.08]	14,861	10,641	5,775	5,478	13,078
K03	1.21 [1.01-1.45]	1.38 [1.12-1.70]	1.38 [1.08-1.77]	0.96 [0.72-1.29]	1.15 [0.96-1.38]	1.15 [0.95-1.38]	1.30 [1.05-1.62]	1.26 [0.98-1.63]	0.93 [0.68-1.26]	1.12 [0.93-1.34]	411	287	192	162	376
K04	1.11 [1.05-1.17]	1.13 [1.06-1.19]	1.20 [1.12-1.29]	0.87 [0.76-0.99]	1.08 [1.02-1.14]	1.05 [0.99-1.11]	1.03 [0.97-1.10]	1.10 [1.03-1.19]	0.88 [0.77-1.00]	1.04 [0.99-1.10]	4,067	3,592	2,350	797	3,673
K05	1.20 [1.14-1.26]	1.21 [1.15-1.27]	1.24 [1.14-1.36]	1.14 [1.01-1.29]	1.14 [1.08-1.21]	1.14 [1.08-1.20]	1.11 [1.06-1.17]	1.14 [1.04-1.25]	1.13 [0.99-1.29]	1.11 [1.05-1.17]	4,660	5,027	1,456	913	3,958
K06	1.29 [1.13-1.47]	1.26 [1.10-1.44]	1.31 [1.13-1.52]		1.16 [1.02-1.33]	1.24 [1.08-1.42]	1.21 [1.05-1.39]	1.21 [1.04-1.41]		1.12 [0.97-1.29]	662	644	524	NA	605
K07	1.13 [1.05-1.21]	1.19 [1.10-1.28]	1.17 [1.02-1.34]	1.08 [0.97-1.20]	1.14 [1.06-1.23]	1.07 [1.00-1.15]	1.08 [1.00-1.17]	1.05 [0.92-1.21]	1.05 [0.94-1.18]	1.10 [1.02-1.19]	2,700	2,147	622	1,229	2,266
K08	1.16 [1.11-1.21]	1.19 [1.14-1.24]	1.22 [1.15-1.28]	0.94 [0.83-1.06]	1.13 [1.08-1.18]	1.11 [1.06-1.16]	1.10 [1.05-1.16]	1.12 [1.07-1.19]	0.93 [0.82-1.05]	1.09 [1.04-1.14]	6,461	6,008	4,167	965	5,913
K09	1.20 [1.03-1.41]	1.13 [0.96-1.33]	1.12 [0.93-1.35]		1.18 [0.99-1.39]	1.16 [0.98-1.37]	1.06 [0.90-1.25]	1.10 [0.91-1.33]		1.12 [0.94-1.33]	471	431	314	NA	383
K10	1.17 [1.04-1.32]	1.16 [1.03-1.31]	1.19 [1.04-1.36]		1.13 [1.00-1.28]	1.09 [0.96-1.23]	1.04 [0.92-1.18]	1.09 [0.95-1.25]		1.09 [0.97-1.23]	785	785	651	NA	766
K11	1.29 [1.19-1.40]	1.27 [1.17-1.38]	1.28 [1.17-1.40]	1.02 [0.77-1.35]	1.22 [1.12-1.32]	1.24 [1.14-1.35]	1.20 [1.11-1.31]	1.20 [1.09-1.32]	1.00 [0.74-1.35]	1.17 [1.07-1.27]	1,749	1,665	1,405	162	1,616
K12	1.25 [1.17-1.34]	1.24 [1.16-1.33]	1.28 [1.18-1.38]	1.16 [0.97-1.40]	1.19 [1.11-1.27]	1.20 [1.12-1.29]	1.17 [1.09-1.26]	1.20 [1.11-1.30]	1.19 [0.99-1.44]	1.16 [1.08-1.24]	2,730	2,442	2,066	406	2,549
K13	1.28 [1.21-1.36]	1.27 [1.20-1.35]	1.29 [1.21-1.37]	1.22 [0.99-1.50]	1.26 [1.19-1.34]	1.22 [1.15-1.29]	1.19 [1.12-1.26]	1.20 [1.12-1.28]	1.16 [0.94-1.43]	1.22 [1.15-1.29]	3,653	3,476	2,943	328	3,297
K14	1.30 [1.21-1.40]	1.29 [1.19-1.39]	1.29 [1.19-1.39]	1.23 [0.88-1.71]	1.26 [1.17-1.36]	1.23 [1.13-1.33]	1.17 [1.08-1.27]	1.19 [1.10-1.29]	1.12 [0.79-1.58]	1.20 [1.11-1.30]	2,026	1,946	1,744	126	1,896
K20	1.20 [1.16-1.23]	1.19 [1.16-1.23]	1.18 [1.15-1.22]	1.32 [1.06-1.66]	1.15 [1.12-1.19]	1.12 [1.09-1.16]	1.10 [1.06-1.13]	1.09 [1.06-1.13]	1.20 [0.95-1.52]	1.10 [1.06-1.13]	12,640	12,544	11,699	300	11,728
K21	1.22 [1.20-1.24]	1.22 [1.20-1.24]	1.22 [1.20-1.24]	1.03 [0.94-1.13]	1.18 [1.16-1.20]	1.11 [1.09-1.13]	1.10 [1.08-1.11]	1.10 [1.08-1.12]	0.93 [0.84-1.03]	1.09 [1.08-1.11]	51,515	51,270	46,144	1,507	48,545
K22	1.19 [1.16-1.22]	1.19 [1.16-1.22]	1.18 [1.15-1.21]	1.05 [0.85-1.30]	1.16 [1.13-1.19]	1.14 [1.11-1.16]	1.11 [1.09-1.14]	1.11 [1.08-1.14]	0.96 [0.77-1.21]	1.11 [1.08-1.14]	19,006	18,956	18,032	289	17,638
K23	1.36 [1.09-1.69]	1.29 [1.04-1.61]	1.38 [1.10-1.72]		1.36 [1.10-1.69]	1.26 [1.01-1.57]	1.14 [0.91-1.43]	1.21 [0.96-1.52]		1.24 [1.00-1.54]	263	262	259	NA	254
K25	1.19 [1.15-1.23]	1.19 [1.15-1.23]	1.17 [1.13-1.21]		1.16 [1.12-1.20]	1.14 [1.10-1.18]	1.11 [1.07-1.15]	1.10 [1.06-1.14]		1.13 [1.09-1.17]	9,750	9,770	9,274	NA	9,017
K26	1.11 [1.07-1.16]	1.12 [1.07-1.17]	1.11 [1.07-1.16]		1.11 [1.06-1.16]	1.10 [1.05-1.14]	1.08 [1.03-1.12]	1.08 [1.03-1.12]		1.10 [1.06-1.15]	6,495	6,483	6,249	NA	5,953
K27	1.13 [1.03-1.25]	1.13 [1.03-1.25]	1.14 [1.03-1.26]		1.13 [1.03-1.25]	1.06 [0.96-1.18]	1.03 [0.94-1.14]	1.05 [0.95-1.16]		1.09 [0.99-1.21]	1,180	1,186	1,113	NA	1,141
K28	1.29 [1.00-1.66]	1.34 [1.04-1.73]	1.29 [0.99-1.69]		1.39 [1.08-1.77]	1.17 [0.91-1.52]	1.15 [0.88-1.50]	1.08 [0.81-1.43]		1.29 [1.00-1.66]	190	187	161	NA	198
K29	1.20 [1.18-1.22]	1.20 [1.18-1.22]	1.20 [1.18-1.22]	1.01 [0.92-1.12]	1.16 [1.14-1.18]	1.13 [1.12-1.15]	1.11 [1.09-1.13]	1.11 [1.09-1.13]	0.95 [0.86-1.06]	1.11 [1.09-1.13]	48,814	48,504	44,100	1,375	45,096
K30	1.22 [1.18-1.25]	1.21 [1.18-1.25]	1.21 [1.17-1.25]	1.22 [0.95-1.56]	1.19 [1.15-1.23]	1.13 [1.09-1.17]	1.11 [1.07-1.14]	1.12 [1.08-1.16]	1.13 [0.87-1.47]	1.12 [1.08-1.16]	11,654	11,723	10,147	221	10,613
K31	1.18 [1.15-1.21]	1.18 [1.15-1.21]	1.20 [1.17-1.23]	0.93 [0.70-1.23]	1.15 [1.12-1.18]	1.10 [1.07-1.13]	1.07 [1.05-1.10]	1.09 [1.06-1.12]	0.91 [0.67-1.22]	1.08 [1.05-1.11]	17,145	17,143	16,337	175	16,558
K35	1.03 [0.98-1.09]	1.05 [1.00-1.11]	1.01 [0.94-1.09]	0.95 [0.86-1.05]	1.00 [0.95-1.05]	1.00 [0.95-1.06]	1.01 [0.95-1.06]	0.99 [0.92-1.06]	0.93 [0.83-1.03]	0.99 [0.93-1.04]	4,838	4,229	2,120	1,411	4,024
K36	0.98 [0.73-1.33]	1.08 [0.80-1.47]			1.00 [0.73-1.37]	0.96 [0.70-1.30]	0.98 [0.71-1.35]			0.92 [0.66-1.28]	118	120	NA	NA	109
K37	1.04 [0.93-1.16]	1.06 [0.95-1.19]	1.05 [0.88-1.26]	0.99 [0.80-1.22]	1.01 [0.90-1.13]	0.99 [0.88-1.11]	0.98 [0.87-1.10]	0.99 [0.83-1.20]	0.93 [0.75-1.17]	0.96 [0.85-1.08]	965	888	327	309	848
K38	1.02 [0.90-1.16]	1.03 [0.90-1.16]	1.02 [0.86-1.21]	0.87 [0.66-1.15]	0.98 [0.87-1.12]	0.98 [0.86-1.12]	0.94 [0.82-1.07]	0.97 [0.82-1.15]	0.85 [0.63-1.14]	0.96 [0.84-1.10]	747	715	395	172	675
K40	1.04 [1.01-1.07]	1.05 [1.02-1.08]	1.05 [1.02-1.08]	0.94 [0.82-1.09]	1.04 [1.01-1.07]	1.01 [0.99-1.04]	1.03 [1.00-1.06]	1.04 [1.01-1.07]	0.91 [0.79-1.05]	1.02 [0.99-1.05]	14,245	13,659	13,188	626	12,360
K41	1.12 [1.01-1.23]	1.12 [1.01-1.23]	1.14 [1.03-1.26]		1.08 [0.98-1.20]	1.06 [0.96-1.17]	1.08 [0.97-1.19]	1.09 [0.98-1.21]		1.04 [0.94-1.15]	1,186	1,186	1,106	NA	1,093
K42	1.17 [1.12-1.22]	1.15 [1.11-1.20]	1.17 [1.12-1.22]	1.03 [0.86-1.23]	1.16 [1.11-1.21]	1.08 [1.04-1.13]	1.05 [1.01-1.10]	1.06 [1.01-1.11]	0.98 [0.81-1.18]	1.09 [1.05-1.14]	7,649	7,387	6,396	406	7,200
K43	1.16 [1.11-1.21]	1.17 [1.12-1.22]	1.18 [1.13-1.23]	1.07 [0.82-1.41]	1.12 [1.08-1.17]	1.09 [1.04-1.14]	1.07 [1.03-1.12]	1.09 [1.04-1.14]	1.04 [0.78-1.39]	1.08 [1.04-1.13]	6,341	6,260	5,692	180	6,234
K44	1.19 [1.17-1.21]	1.19 [1.17-1.21]	1.18 [1.16-1.20]	1.07 [0.90-1.26]	1.15 [1.14-1.17]	1.11 [1.10-1.13]	1.10 [1.08-1.12]	1.09 [1.07-1.11]	0.97 [0.81-1.16]	1.09 [1.07-1.11]	48,147	48,297	45,224	491	44,437
K45	1.14 [0.94-1.38]	1.07 [0.88-1.30]	0.99 [0.81-1.21]		1.11 [0.92-1.34]	1.06 [0.87-1.29]	0.97 [0.79-1.18]	0.91 [0.74-1.12]		1.02 [0.84-1.24]	299	297	270	NA	301
K46	1.18 [1.08-1.30]	1.19 [1.08-1.31]	1.20 [1.09-1.32]		1.16 [1.06-1.27]	1.09 [0.99-1.20]	1.07 [0.97-1.17]	1.09 [0.98-1.20]		1.10 [1.01-1.21]	1,370	1,359	1,295	NA	1,374
K50	1.44 [1.35-1.52]	1.43 [1.35-1.52]	1.34 [1.25-1.44]	1.44 [1.18-1.75]	1.40 [1.32-1.49]	1.27 [1.20-1.36]	1.24 [1.16-1.32]	1.16 [1.08-1.25]	1.38 [1.12-1.71]	1.27 [1.20-1.35]	3,492	3,412	2,308	415	3,235
K51	1.35 [1.29-1.42]	1.32 [1.26-1.39]	1.29 [1.22-1.36]	1.41 [1.14-1.75]	1.28 [1.22-1.34]	1.24 [1.17-1.30]	1.17 [1.11-1.24]	1.15 [1.09-1.22]	1.30 [1.03-1.65]	1.20 [1.14-1.26]	4,939	4,961	3,728	332	4,485
K52	1.25 [1.22-1.27]	1.25 [1.22-1.28]	1.24 [1.21-1.27]	1.15 [1.06-1.24]	1.21 [1.18-1.23]	1.18 [1.16-1.21]	1.16 [1.13-1.18]	1.15 [1.13-1.18]	1.13 [1.04-1.23]	1.16 [1.14-1.19]	26,610	25,640	22,004	1,885	24,352
K55	1.16 [1.10-1.22]	1.15 [1.10-1.21]	1.16 [1.10-1.22]		1.10 [1.05-1.16]	1.12 [1.06-1.18]	1.09 [1.03-1.14]	1.09 [1.03-1.15]		1.08 [1.03-1.14]	4,398	4,404	4,282	NA	4,230
K56	1.12 [1.09-1.16]	1.12 [1.08-1.15]	1.11 [1.08-1.15]	1.07 [0.88-1.31]	1.10 [1.06-1.13]	1.08 [1.05-1.11]	1.06 [1.02-1.09]	1.06 [1.02-1.09]	1.05 [0.85-1.28]	1.07 [1.04-1.10]	12,145	12,020	11,246	358	11,630
K57	1.16 [1.14-1.17]	1.15 [1.13-1.16]	1.16 [1.14-1.17]		1.12 [1.10-1.14]	1.10 [1.08-1.12]	1.07 [1.06-1.09]	1.08 [1.07-1.10]		1.08 [1.06-1.09]	54,908	54,918	54,410	NA	51,008
K58	1.33 [1.30-1.37]	1.34 [1.30-1.38]	1.35 [1.31-1.39]	1.03 [0.92-1.15]	1.29 [1.25-1.32]	1.16 [1.13-1.19]	1.10 [1.07-1.13]	1.12 [1.08-1.15]	0.98 [0.87-1.10]	1.15 [1.12-1.18]	16,813	17,367	12,036	1,034	16,145
K59	1.19 [1.17-1.21]	1.19 [1.17-1.21]	1.19 [1.17-1.20]	1.13 [1.06-1.20]	1.16 [1.14-1.18]	1.13 [1.12-1.15]	1.11 [1.10-1.13]	1.11 [1.09-1.13]	1.11 [1.03-1.18]	1.12 [1.10-1.14]	52,797	51,061	46,256	3,194	50,573
K60	1.34 [1.27-1.41]	1.35 [1.28-1.43]	1.37 [1.29-1.47]	1.33 [1.09-1.62]	1.32 [1.25-1.40]	1.26 [1.19-1.33]	1.25 [1.18-1.32]	1.28 [1.19-1.37]	1.33 [1.08-1.63]	1.27 [1.20-1.35]	4,155	4,122	2,784	388	3,780
K61	1.29 [1.20-1.40]	1.30 [1.20-1.40]	1.28 [1.17-1.40]	1.18 [0.92-1.52]	1.24 [1.15-1.34]	1.25 [1.16-1.35]	1.20 [1.11-1.30]	1.19 [1.09-1.31]	1.23 [0.94-1.60]	1.22 [1.13-1.32]	2,219	2,206	1,434	240	1,976
K62	1.20 [1.18-1.23]	1.20 [1.18-1.23]	1.19 [1.16-1.22]	1.23 [1.08-1.41]	1.16 [1.14-1.19]	1.14 [1.12-1.17]	1.13 [1.10-1.15]	1.12 [1.09-1.14]	1.17 [1.02-1.35]	1.12 [1.10-1.15]	26,704	26,653	23,349	792	24,350
K63	1.14 [1.12-1.17]	1.15 [1.13-1.17]	1.14 [1.12-1.17]	1.21 [1.00-1.47]	1.13 [1.11-1.15]	1.09 [1.06-1.11]	1.07 [1.04-1.09]	1.06 [1.04-1.09]	1.12 [0.92-1.37]	1.09 [1.07-1.11]	27,517	27,533	25,686	407	26,081
K64	1.19 [1.16-1.22]	1.18 [1.15-1.21]	1.19 [1.16-1.22]	1.05 [0.89-1.24]	1.16 [1.13-1.19]	1.12 [1.09-1.15]	1.09 [1.06-1.12]	1.10 [1.07-1.13]	1.02 [0.85-1.22]	1.10 [1.08-1.13]	19,851	20,018	17,641	520	19,125
K65	1.14 [1.07-1.21]	1.11 [1.04-1.18]	1.10 [1.03-1.17]		1.10 [1.03-1.17]	1.11 [1.04-1.19]	1.06 [0.99-1.13]	1.06 [0.99-1.13]		1.09 [1.03-1.17]	2,819	2,795	2,538	NA	2,658
K66	1.11 [1.07-1.14]	1.10 [1.06-1.14]	1.10 [1.06-1.14]	1.00 [0.84-1.18]	1.06 [1.03-1.10]	1.05 [1.02-1.09]	1.02 [0.99-1.06]	1.03 [0.99-1.07]	0.97 [0.81-1.16]	1.04 [1.00-1.07]	9,791	9,940	7,610	466	9,381

Supplementary Table 4: Hazard ratios and events from all cohorts (excluding non-consulters)

	Hazard ratio (99% confidence interval)										Events (in exposed)				
	crude					adjusted									
Outcome	any age	18+	40+	<18	hosp.	any age	18+	40+	<18	hosp.	any age	18+	40+	<18	hosp.
K70	1.36 [1.28-1.44]	1.35 [1.27-1.43]	1.35 [1.27-1.43]		1.30 [1.22-1.37]	1.31 [1.24-1.39]	1.25 [1.18-1.32]	1.23 [1.15-1.31]		1.29 [1.22-1.37]	3,519	3,533	3,298	NA	3,270
K71	1.27 [1.00-1.60]	1.29 [1.02-1.62]	1.42 [1.11-1.83]		1.18 [0.94-1.48]	1.24 [0.98-1.57]	1.21 [0.95-1.53]	1.33 [1.03-1.72]		1.16 [0.92-1.47]	219	221	194	NA	217
K72	1.25 [1.16-1.34]	1.27 [1.18-1.36]	1.28 [1.19-1.38]		1.28 [1.19-1.37]	1.20 [1.12-1.29]	1.18 [1.10-1.26]	1.18 [1.10-1.27]		1.26 [1.17-1.35]	2,388	2,401	2,273	NA	2,314
K73	1.36 [1.08-1.69]	1.32 [1.06-1.66]	1.38 [1.09-1.74]		1.43 [1.15-1.79]	1.27 [1.01-1.59]	1.24 [0.99-1.57]	1.26 [0.99-1.61]		1.36 [1.09-1.71]	231	224	211	NA	222
K74	1.30 [1.22-1.37]	1.31 [1.24-1.39]	1.30 [1.23-1.38]		1.29 [1.22-1.36]	1.23 [1.16-1.30]	1.20 [1.14-1.28]	1.18 [1.12-1.26]		1.25 [1.19-1.33]	3,771	3,756	3,630	NA	3,691
K75	1.24 [1.16-1.33]	1.25 [1.17-1.34]	1.25 [1.17-1.35]		1.24 [1.15-1.32]	1.17 [1.09-1.26]	1.15 [1.07-1.23]	1.15 [1.07-1.24]		1.20 [1.12-1.29]	2,409	2,394	2,238	NA	2,346
K76	1.20 [1.17-1.23]	1.19 [1.16-1.23]	1.20 [1.17-1.23]	0.79 [0.64-0.99]	1.17 [1.14-1.20]	1.13 [1.10-1.16]	1.09 [1.06-1.12]	1.09 [1.06-1.12]	0.81 [0.64-1.03]	1.12 [1.09-1.15]	16,807	16,847	15,484	271	16,350
K80	1.09 [1.06-1.11]	1.08 [1.06-1.10]	1.08 [1.05-1.10]	0.87 [0.76-0.99]	1.05 [1.03-1.07]	1.04 [1.02-1.07]	1.02 [1.00-1.04]	1.02 [1.00-1.04]	0.89 [0.77-1.03]	1.02 [1.00-1.04]	28,674	28,917	24,159	673	26,681
K81	1.09 [1.04-1.15]	1.08 [1.03-1.13]	1.08 [1.03-1.13]	0.81 [0.58-1.13]	1.10 [1.05-1.15]	1.05 [1.00-1.10]	1.01 [0.97-1.06]	1.01 [0.96-1.07]	0.79 [0.55-1.12]	1.06 [1.02-1.12]	5,284	5,376	4,543	111	5,055
K82	1.07 [1.02-1.13]	1.07 [1.01-1.13]	1.07 [1.01-1.14]	0.92 [0.66-1.29]	1.07 [1.02-1.13]	1.03 [0.97-1.09]	1.00 [0.95-1.06]	1.01 [0.96-1.08]	0.89 [0.62-1.26]	1.04 [0.99-1.10]	3,865	3,904	3,123	118	3,675
K83	1.08 [1.04-1.13]	1.09 [1.05-1.13]	1.08 [1.04-1.13]	0.77 [0.55-1.08]	1.08 [1.04-1.12]	1.05 [1.01-1.09]	1.04 [1.00-1.08]	1.03 [0.99-1.07]	0.76 [0.54-1.07]	1.06 [1.02-1.10]	7,814	7,851	7,057	108	7,473
K85	1.11 [1.06-1.16]	1.11 [1.06-1.16]	1.10 [1.04-1.16]	0.98 [0.72-1.32]	1.09 [1.04-1.14]	1.07 [1.02-1.12]	1.04 [0.99-1.10]	1.03 [0.98-1.09]	1.09 [0.79-1.50]	1.07 [1.02-1.12]	4,845	4,900	4,188	136	4,546
K86	1.12 [1.06-1.18]	1.14 [1.08-1.20]	1.12 [1.06-1.18]		1.12 [1.06-1.17]	1.07 [1.02-1.13]	1.06 [1.00-1.11]	1.04 [0.99-1.10]		1.09 [1.04-1.15]	4,443	4,453	4,212	NA	4,367
K90	1.53 [1.45-1.61]	1.41 [1.33-1.50]	1.46 [1.37-1.56]	2.03 [1.81-2.28]	1.51 [1.43-1.59]	1.37 [1.30-1.45]	1.24 [1.17-1.32]	1.29 [1.20-1.38]	1.71 [1.51-1.93]	1.37 [1.30-1.45]	4,645	3,769	2,721	1,258	4,293
K91	1.19 [1.13-1.25]	1.17 [1.11-1.24]	1.18 [1.12-1.25]	0.90 [0.67-1.20]	1.14 [1.08-1.20]	1.13 [1.07-1.19]	1.08 [1.03-1.15]	1.11 [1.04-1.17]	0.84 [0.62-1.14]	1.10 [1.04-1.16]	3,911	3,857	3,413	157	3,759
K92	1.18 [1.16-1.20]	1.18 [1.16-1.21]	1.17 [1.15-1.20]	1.02 [0.92-1.13]	1.14 [1.12-1.16]	1.13 [1.11-1.15]	1.11 [1.09-1.13]	1.10 [1.08-1.12]	0.99 [0.89-1.11]	1.11 [1.09-1.13]	34,874	34,921	30,925	1,261	33,035
Diseases of the skin and subcutaneous tissue (L00-L99)															
L01	2.19 [1.83-2.62]	2.14 [1.68-2.73]	2.16 [1.65-2.84]	2.38 [1.84-3.06]	2.10 [1.74-2.52]	2.04 [1.69-2.45]	1.85 [1.43-2.38]	1.96 [1.48-2.59]	2.23 [1.71-2.90]	1.96 [1.62-2.38]	499	251	192	282	456
L02	1.25 [1.20-1.31]	1.27 [1.21-1.32]	1.26 [1.19-1.32]	1.17 [1.03-1.34]	1.20 [1.15-1.25]	1.19 [1.14-1.25]	1.16 [1.11-1.22]	1.16 [1.10-1.22]	1.14 [0.99-1.30]	1.17 [1.12-1.23]	6,363	6,163	4,282	796	5,804
L03	1.50 [1.47-1.54]	1.52 [1.49-1.55]	1.51 [1.48-1.54]	1.30 [1.16-1.45]	1.45 [1.42-1.48]	1.45 [1.42-1.48]	1.42 [1.39-1.45]	1.42 [1.39-1.45]	1.26 [1.13-1.42]	1.41 [1.38-1.44]	32,062	31,439	29,524	1,201	30,055
L04	1.26 [1.06-1.49]	1.23 [0.96-1.57]	1.31 [0.99-1.75]	1.20 [0.95-1.52]	1.19 [0.99-1.42]	1.24 [1.04-1.48]	1.18 [0.92-1.52]	1.30 [0.97-1.73]	1.19 [0.94-1.51]	1.17 [0.98-1.40]	426	199	144	237	383
L05	1.04 [0.94-1.15]	1.06 [0.96-1.17]	1.27 [1.05-1.52]	0.93 [0.77-1.11]	1.05 [0.95-1.18]	1.02 [0.92-1.14]	1.01 [0.91-1.11]	1.18 [0.98-1.43]	0.96 [0.79-1.16]	1.05 [0.94-1.18]	1,261	1,297	349	427	987
L08	1.39 [1.34-1.45]	1.39 [1.33-1.44]	1.41 [1.35-1.46]	1.61 [1.34-1.93]	1.38 [1.33-1.44]	1.35 [1.29-1.40]	1.30 [1.25-1.35]	1.32 [1.26-1.37]	1.63 [1.34-1.97]	1.36 [1.31-1.41]	8,468	8,107	7,598	460	8,123
L10	1.80 [1.29-2.51]	2.10 [1.50-2.93]	2.01 [1.44-2.82]		1.85 [1.33-2.56]	1.68 [1.20-2.35]	1.93 [1.37-2.72]	1.77 [1.24-2.50]		1.72 [1.23-2.40]	127	126	123	NA	121
L12	2.66 [2.36-3.00]	2.66 [2.36-3.00]	2.65 [2.36-2.99]		2.54 [2.25-2.87]	2.58 [2.29-2.91]	2.54 [2.25-2.87]	2.54 [2.25-2.86]		2.47 [2.19-2.80]	1,160	1,159	1,151	NA	1,060
L13	2.61 [1.94-3.51]	3.28 [2.40-4.49]	2.95 [2.14-4.06]		2.40 [1.78-3.24]	2.50 [1.85-3.38]	3.12 [2.27-4.28]	2.79 [2.02-3.87]		2.31 [1.70-3.13]	183	181	163	NA	167
L20	24.14 [20.01-29.12]	29.51 [22.97-37.90]	29.60 [22.34-39.24]	17.64 [13.61-22.86]	24.80 [20.43-30.10]	21.95 [18.16-26.53]	26.47 [20.56-34.06]	27.25 [20.53-36.19]	14.94 [11.46-19.48]	22.36 [18.38-27.20]	2,663	1,677	1,233	1,165	2,423
L21	2.74 [2.28-3.30]	2.57 [2.14-3.10]	2.72 [2.24-3.30]		2.65 [2.20-3.18]	2.62 [2.17-3.17]	2.47 [2.04-2.98]	2.57 [2.11-3.14]		2.57 [2.13-3.10]	516	477	451	NA	485
L22	1.01 [0.81-1.27]	0.91 [0.67-1.24]	0.91 [0.67-1.24]		1.03 [0.82-1.31]	1.01 [0.80-1.27]	0.90 [0.66-1.23]	0.89 [0.65-1.22]		1.03 [0.81-1.31]	225	132	131	NA	217
L23	3.18 [2.64-3.82]	2.92 [2.39-3.56]	3.21 [2.56-4.02]	4.71 [2.94-7.55]	3.08 [2.57-3.71]	2.94 [2.44-3.55]	2.69 [2.20-3.30]	2.96 [2.36-3.73]	3.71 [2.25-6.12]	2.90 [2.41-3.50]	536	434	338	126	494
L24	1.35 [1.25-1.46]	1.36 [1.25-1.47]	1.34 [1.24-1.45]		1.32 [1.22-1.43]	1.32 [1.21-1.43]	1.29 [1.19-1.40]	1.26 [1.16-1.37]		1.32 [1.22-1.43]	2,175	2,163	2,136	NA	2,159
L25	2.52 [2.09-3.03]	2.83 [2.34-3.41]	2.54 [2.07-3.12]		2.37 [1.97-2.85]	2.41 [2.00-2.91]	2.66 [2.20-3.22]	2.42 [1.96-2.97]		2.31 [1.92-2.79]	492	490	394	NA	449
L27	1.66 [1.53-1.80]	1.48 [1.36-1.61]	1.52 [1.39-1.66]	2.66 [2.04-3.46]	1.58 [1.45-1.71]	1.55 [1.43-1.69]	1.38 [1.26-1.50]	1.42 [1.30-1.56]	2.33 [1.76-3.08]	1.51 [1.39-1.64]	1,969	1,766	1,532	265	1,883
L28	3.96 [3.48-4.51]	3.92 [3.44-4.46]	3.66 [3.21-4.19]		3.41 [3.00-3.87]	3.72 [3.26-4.25]	3.64 [3.19-4.15]	3.37 [2.95-3.86]		3.21 [2.82-3.65]	1,192	1,155	1,047	NA	1,081
L29	1.61 [1.51-1.72]	1.56 [1.46-1.66]	1.86 [1.72-2.01]	1.32 [1.07-1.63]	1.54 [1.44-1.64]	1.53 [1.44-1.64]	1.42 [1.33-1.52]	1.71 [1.58-1.86]	1.25 [1.00-1.57]	1.46 [1.36-1.56]	3,273	3,208	2,128	340	3,070
L30	8.55 [8.28-8.83]	8.44 [8.14-8.76]	7.93 [7.62-8.26]	9.02 [8.48-9.59]	8.16 [7.90-8.42]	8.01 [7.76-8.28]	7.81 [7.52-8.10]	7.46 [7.16-7.77]	8.32 [7.81-8.85]	7.65 [7.40-7.90]	36,133	26,652	19,541	11,825	32,561
L40	1.99 [1.91-2.07]	1.97 [1.89-2.05]	1.95 [1.87-2.03]	1.59 [1.28-1.97]	1.95 [1.87-2.03]	1.93 [1.85-2.01]	1.86 [1.78-1.93]	1.85 [1.77-1.93]	1.63 [1.30-2.05]	1.90 [1.83-1.98]	9,255	9,284	7,897	327	8,542
L42	1.44 [1.05-1.98]	1.58 [1.15-2.19]			1.23 [0.88-1.70]	1.39 [1.00-1.93]	1.37 [0.97-1.93]			1.15 [0.83-1.61]	127	123	NA	NA	111
L43	1.98 [1.77-2.22]	1.92 [1.71-2.15]	1.91 [1.70-2.15]		1.82 [1.62-2.05]	1.89 [1.68-2.12]	1.80 [1.61-2.03]	1.82 [1.62-2.05]		1.76 [1.56-1.98]	1,043	1,047	982	NA	932
L50	1.79 [1.64-1.95]	1.61 [1.46-1.78]	1.71 [1.52-1.92]	2.20 [1.89-2.57]	1.77 [1.62-1.93]	1.54 [1.41-1.68]	1.37 [1.23-1.52]	1.44 [1.27-1.63]	1.86 [1.59-2.19]	1.51 [1.38-1.65]	1,944	1,290	933	772	1,816
L51	1.31 [1.08-1.58]	1.77 [1.41-2.21]	1.75 [1.37-2.24]		1.42 [1.17-1.73]	1.23 [1.02-1.50]	1.63 [1.29-2.05]	1.61 [1.26-2.07]		1.34 [1.10-1.63]	344	266	220	NA	328
L52	1.37 [1.03-1.82]	1.24 [0.94-1.65]			1.28 [0.95-1.74]	1.28 [0.96-1.71]	1.18 [0.88-1.57]			1.18 [0.86-1.62]	162	158	NA	NA	145
L53	1.59 [1.49-1.70]	1.60 [1.49-1.71]	1.67 [1.56-1.80]	1.19 [0.92-1.54]	1.54 [1.44-1.64]	1.54 [1.44-1.65]	1.50 [1.40-1.61]	1.58 [1.47-1.70]	1.11 [0.85-1.46]	1.50 [1.40-1.60]	2,947	2,799	2,546	210	2,790
L56	2.54 [1.83-3.51]	2.41 [1.75-3.34]	2.49 [1.74-3.55]		2.27 [1.64-3.15]	2.48 [1.79-3.45]	2.28 [1.64-3.17]	2.38 [1.66-3.43]		2.20 [1.57-3.07]	145	144	127	NA	131
L57	1.24 [1.18-1.29]	1.21 [1.16-1.27]	1.24 [1.19-1.30]		1.21 [1.16-1.26]	1.19 [1.14-1.25]	1.16 [1.11-1.22]	1.19 [1.13-1.24]		1.16 [1.11-1.22]	5,879	5,881	5,843	NA	5,558
L60	1.38 [1.26-1.50]	1.38 [1.26-1.51]	1.41 [1.28-1.57]	1.13 [0.91-1.41]	1.25 [1.15-1.37]	1.28 [1.17-1.40]	1.27 [1.16-1.40]	1.29 [1.16-1.44]	1.07 [0.85-1.35]	1.17 [1.06-1.28]	1,640	1,494	1,130	301	1,459
L63	2.57 [1.86-3.56]	2.26 [1.66-3.08]	2.75 [1.89-4.01]		2.62 [1.90-3.61]	2.07 [1.46-2.93]	1.73 [1.23-2.41]	2.29 [1.54-3.41]		2.15 [1.52-3.02]	166	162	118	NA	155
L65	1.72 [1.44-2.05]	1.77 [1.48-2.12]	1.94 [1.59-2.38]		1.81 [1.51-2.16]	1.55 [1.29-1.86]	1.54 [1.27-1.86]	1.73 [1.41-2.14]		1.60 [1.33-1.93]	449	423	329	NA	418
L68	1.14 [0.82-1.59]	1.18 [0.85-1.65]			1.23 [0.88-1.72]	1.10 [0.78-1.56]	1.03 [0.73-1.46]			1.19 [0.85-1.68]	104	108	NA	NA	105
L70	1.25 [1.03-1.52]	1.18 [0.97-1.42]	1.22 [0.88-1.69]	1.22 [0.85-1.75]	1.18 [0.97-1.44]	1.16 [0.95-1.42]	1.06 [0.87-1.29]	1.06 [0.76-1.49]	1.13 [0.77-1.65]	1.13 [0.92-1.38]	350	348	111	117	324
L71	1.39 [1.21-1.61]	1.46 [1.27-1.69]	1.38 [1.19-1.59]		1.42 [1.23-1.64]	1.31 [1.13-1.52]	1.41 [1.22-1.62]	1.30 [1.12-1.51]		1.35 [1.16-1.56]	613	603	566	NA	581
L72	1.07 [1.03-1.12]	1.07 [1.03-1.12]	1.10 [1.04-1.15]	0.97 [0.80-1.18]	1.05 [1.01-1.10]	1.03 [0.99-1.08]	1.01 [0.97-1.06]	1.04 [0.99-1.09]	0.93 [0.76-1.13]	1.03 [0.98-1.07]	6,167	6,011	4,738	372	5,322
L73	1.39 [1.24-1.55]	1.41 [1.26-1.58]	1.37 [1.19-1.58]	1.08 [0.75-1.56]	1.28 [1.14-1.43]	1.29 [1.15-1.45]	1.26 [1.13-1.42]	1.23 [1.06-1.42]	1.02 [0.70-1.49]	1.23 [1.10-1.38]	1,006	1,026	580	104	928
L80	1.62 [1.31-2.01]	1.54 [1.25-1.91]	1.57 [1.25-1.97]		1.67 [1.35-2.08]	1.47 [1.18-1.84]	1.46 [1.17-1.82]	1.42 [1.12-1.80]		1.61 [1.29-2.01]	291	287	247	NA	279
L81	1.21 [1.09-1.33]	1.18 [1.07-1.31]	1.21 [1.09-1.35]	1.66 [1.13-2.45]	1.22 [1.10-1.35]	1.16 [1.05-1.28]	1.13 [1.02-1.25]	1.16 [1.04-1.30]	1.67 [1.12-2.48]	1.17 [1.06-1.30]	1,189	1,109	972	106	1,113
L82	1.15 [1.09-1.22]	1.19 [1.12-1.25]	1.19 [1.12-1.26]		1.16 [1.10-1.23]	1.10 [1.04-1.17]	1.13 [1.06-1.19]	1.13 [1.07-1.20]		1.11 [1.05-1.18]	3,562	3,569	3,440	NA	3,267
L83	1.66 [1.32-2.09]	1.67 [1.31-2.12]	1.61 [1.25-2.06]		1.60 [1.27-2.01]	1.60 [1.26-2.02]	1.53 [1.20-1.96]	1.52 [1.18-1.95]		1.53 [1.21-1.94]	252	232	210	NA	230
L84	1.43 [1.22-1.69]	1.40 [1.19-1.65]	1.35 [1.13-1.59]		1.30 [1.10-1.53]	1.36 [1.15-1.61]	1.29 [1.09-1.53]	1.24 [1.04-1.47]		1.26 [1.06-1.49]	453	447	411	NA	416
L85	1.51 [1.39-1.65]	1.49 [1.37-1.62]	1.46 [1.34-1.59]		1.52 [1.40-1.65]	1.46 [1.35-1.59]	1.41 [1.30-1.54]	1.39 [1.27-1.51]		1.48 [1.36-1.61]	1,816	1,793	1,721	NA	1,731
L88	1.61 [1.15-2.26]	1.64 [1.17-2.28]			1.28 [0.92-1.77]	1.49 [1.05-2.10]	1.46 [1.03-2.06]			1.19 [0.85-1.66]	108	109	NA	NA	112
L89	1.17 [1.14-1.19]	1.15 [1.13-1.18]	1.16 [1.13-1.18]		1.16 [1.14-1.19]	1.15 [1.12-1.18]	1.11 [1.09-1.14]	1.11 [1.09-1.14]		1.16 [1.14-1.19]	21,530	21,508	21,367	NA	21,128
L90	1.24 [1.17-1.31]	1.27 [1.20-1.34]	1.25 [1.17-1.33]	0.93 [0.71-1.22]	1.22 [1.16-1.30]	1.19 [1.12-1.26]	1.20 [1.13-1.27]	1.18 [1.10-1.26]	0.91 [0.69-1.21]	1.18 [1.12-1.26]	3,508	3,505	2,806	182	3,338
L91	1.29 [1.16-1.43]	1.22 [1.09-1.35]	1.25 [1.10-1.42]	1.46 [1.11-1.93]	1.19 [1.07-1.33]	1.23 [1.10-1.37]	1.16 [1.04-1.29]	1.18 [1.04-1.35]	1.45 [1.09-1.92]	1.16 [1.04-1.29]	1,110	1,020	677	199	974
L92	1.24 [1.10-1.39]	1.21 [1.07-1.36]	1.19 [1.04-1.36]	1.04 [0.76-1.43]	1.15 [1.03-1.29]	1.17 [1.04-1.31]	1.11 [0.99-1.26]	1.11 [0.97-1.27]	1.03 [0.74-1.43]	1.11 [0.99-1.24]	917	850	657	134	857
L93	1.71 [1.47-1.98]	1.65 [1.41-1.92]	1.64 [1.39-1.93]		1.69 [1.46-1.97]	1.58 [1.35-1.84]	1.47 [1.26-1.72]	1.47 [1.24-1.74]		1.57 [1.35-1.83]	565	569	467	NA	542
L94	1.33 [1.10-1.62]	1.32 [1.08-1.62]	1.54 [1.24-1.91]		1.31 [1.08-1.59]	1.27 [1.05-1.55]	1.25 [1.01-1.53]	1.39 [1.11-1.74]		1.28 [1.05-1.55]	317	294	266	NA	309
L95	1.61 [1.36-1.90]	1.78 [1.51-2.11]	1.64 [1.37-1.96]		1.55 [1.31-1.83]	1.50 [1.27-1.78]	1.67 [1.41-1.99]	1.50 [1.25-1.81]		1.45 [1.22-1.72]	472	463	409	NA	439
L97	1.44 [1.41-1.48]	1.42 [1.38-1.46]	1.44 [1.40-1.48]		1.40 [1.37-1.44]	1.41 [1.37-1.45]	1.35 [1.31-1.39]	1.36 [1.32-1.40]		1.39 [1.35-1.43]	17,841	17,848	17,693	NA	17,055
L98	1.31 [1.27-1.35]	1.31 [1.27-1.36]	1.31 [1.27-1.35]	1.27 [1.06-1.52]	1.27 [1.23-1.31]	1.26 [1.22-1.30]	1.25 [1.21-1.29]	1.24 [1.20-1.28]	1.28 [1.06-1.54]	1.23 [1.19-1.27]	11,909	11,662	10,675	437	11,106
Diseases of the musculoskeletal system and connective tissue (M00-M99)															
M00	1.34 [1.22-1.47]	1.31 [1.19-1.43]	1.37 [1.24-1.51]	1.45 [1.00-2.09]	1.30 [1.18-1.42]	1.29 [1.17-1.42]	1.23 [1.12-1.35]	1.29 [1.17-1.42]	1.44 [0.98-2.10]	1.27 [1.16-1.39]	1,458	1,378	1,323	105	1,421
M02	1.16 [0.93-1.45]	1.30 [1.00-1.68]	1.14 [0.86-1.53]		1.15 [0.91-1.46]	1.12 [0.89-1.41]	1.21 [0.93-1.58]	1.10 [0.82-1.48]		1.08 [0.85-1.38]	246	178	133	NA	216
M05	1.21 [1.12-1.32]	1.20 [1.11-1.31]	1.19 [1.10-1.30]		1.16 [1.07-1.26]	1.19 [1.10-1.29]	1.16 [1.07-1.26]	1.15 [1.06-1.25]		1.14 [1.05-1.24]	1,718	1,725	1,632	NA	1,666
M06	1.24 [1.20-1.28]	1.25 [1.21-1.29]	1.24 [1.21-1.28]		1.22 [1.19-1.26]	1.18 [1.14-1.21]	1.16 [1.13-1.20]	1.16 [1.12-1.19]		1.17 [1.13-1.20]	13,260	13,321	12,536	NA	12,362
M07	1.48 [1.35-1.62]	1.45 [1.33-1.58]	1.40 [1.27-1.54]		1.44 [1.32-1.57]	1.40 [1.28-1.53]	1.34 [1.22-1.46]	1.29 [1.17-1.43]		1.37 [1.26-1.50]	1,635	1,643	1,351	NA	1,561
M08	1.10 [0.86-1.40]	1.06 [0.75-1.49]		1.18 [0.85-1.65]	1.08 [0.84-1.39]	1.04 [0.81-1.34]	1.04 [0.73-1.47]		1.17 [0.83-1.65]	1.09 [0.84-1.41]	212	105	NA	130	196
M10	1.20 [1.17-1.24]	1.21 [1.18-1.24]	1.20 [1.17-1.23]		1.19 [1.16-1.22]	1.16 [1.13-1.19]	1.14 [1.11-1.17]	1.12 [1.09-1.16]		1.16 [1.13-1.19]	15,577	15,584	15,495	NA	14,894
M11	1.20 [1.10-1.31]	1.17 [1.08-1.28]	1.18 [1.08-1.29]		1.23 [1.13-1.34]	1.14 [1.05-1.25]	1.10 [1.00-1.20]	1.10 [1.01-1.21]		1.18 [1.09-1.29]	1,717	1,713	1,693	NA	1,692
M12	1.36 [1.12-1.66]	1.27 [1.04-1.54]	1.31 [1.07-1.62]		1.11 [0.92-1.35]	1.25 [1.02-1.53]	1.15 [0.94-1.41]	1.14 [0.92-1.42]		1.09 [0.89-1.32]	307	308	260	NA	291
M13	1.21 [1.19-1.23]	1.20 [1.18-1.22]	1.21 [1.18-1.23]	1.12 [0.85-1.49]	1.19 [1.17-1.21]	1.14 [1.12-1.16]	1.11 [1.09-1.13]	1.11 [1.09-1.13]	1.07 [0.80-1.43]	1.13 [1.11-1.15]	37,875	37,923	37,011	188	36,172
M14	1.30 [1.10-1.52]	1.37 [1.17-1.61]	1.42 [1.20-1.68]		1.21 [1.03-1.41]	1.21 [1.03-1.43]	1.20 [1.01-1.42]	1.23 [1.04-1.47]		1.16 [0.99-1.36]	460	460	444	NA	450
M15	1.25 [1.21-1.29]	1.26 [1.22-1.30]	1.23 [1.20-1.27]		1.20 [1.17-1.24]	1.17 [1.13-1.20]	1.16 [1.12-1.19]	1.13 [1.10-1.17]		1.14 [1.11-1.17]	14,249	14,280	14,162	NA	13,843
M16	1.06 [1.04-1.09]	1.06 [1.03-1.08]	1.06 [1.04-1.09]		1.04 [1.01-1.06]	1.04 [1.02-1.07]	1.03 [1.00-1.05]	1.03 [1.00-1.05]		1.02 [0.99-1.04]	19,401	19,372	19,104	NA	17,634
M17	1.15 [1.13-1.17]	1.15 [1.13-1.17]	1.14 [1.12-1.16]		1.12 [1.10-1.14]	1.10 [1.08-1.12]	1.09 [1.07-1.11]	1.08 [1.06-1.10]		1.08 [1.06-1.10]	33,741	33,770	33,007	NA	30,919
M18	1.29 [1.19-1.39]	1.25 [1.16-1.35]	1.27 [1.18-1.37]		1.20 [1.11-1.29]	1.17 [1.08-1.26]	1.11 [1.02-1.20]	1.14 [1.05-1.23]		1.11 [1.03-1.20]	2,057	2,056	2,034	NA	1,969
M19	1.23 [1.21-1.25]	1.23 [1.21-1.25]	1.22 [1.21-1.24]		1.19 [1.17-1.21]	1.16 [1.14-1.17]	1.13 [1.12-1.15]	1.12 [1.11-1.14]		1.13 [1.12-1.15]	59,981	60,071	59,234	NA	57,853
M20	1.21 [1.16-1.26]	1.19 [1.15-1.24]	1.18 [1.13-1.23]	1.24 [0.98-1.58]	1.15 [1.11-1.20]	1.15 [1.11-1.20]	1.13 [1.08-1.18]	1.12 [1.08-1.17]	1.22 [0.95-1.57]	1.11 [1.06-1.15]	7,141	7,127	6,234	260	6,336
M21	1.12 [1.06-1.18]	1.13 [1.07-1.20]	1.12 [1.06-1.19]	0.97 [0.78-1.20]	1.10 [1.05-1.16]	1.06 [1.01-1.12]	1.06 [1.01-1.13]	1.06 [1.00-1.12]	0.97 [0.77-1.21]	1.07 [1.01-1.13]	4,036	3,820	3,497	304	3,919
M22	1.22 [1.10-1.35]	1.19 [1.07-1.31]	1.15 [1.02-1.31]	1.22 [0.89-1.68]	1.23 [1.11-1.36]	1.13 [1.02-1.25]	1.08 [0.97-1.20]	1.06 [0.93-1.21]	1.15 [0.82-1.61]	1.15 [1.03-1.28]	1,172	1,146	671	151	1,057
M23	1.11 [1.07-1.15]	1.12 [1.08-1.15]	1.11 [1.07-1.15]	1.06 [0.90-1.24]	1.08 [1.04-1.12]	1.05 [1.02-1.09]	1.05 [1.02-1.09]	1.04 [1.00-1.08]	1.02 [0.86-1.21]	1.03 [1.00-1.07]	10,814	10,779	8,874	553	9,402
M24	1.18 [1.12-1.24]	1.17 [1.11-1.23]	1.15 [1.08-1.22]	1.10 [0.94-1.29]	1.14 [1.08-1.19]	1.11 [1.06-1.17]	1.08 [1.03-1.14]	1.07 [1.01-1.14]	1.07 [0.90-1.26]	1.09 [1.04-1.15]	4,616	4,470	3,246	549	4,331
M25	1.19 [1.16-1.21]	1.18 [1.16-1.21]	1.17 [1.15-1.20]	1.10 [1.00-1.20]	1.16 [1.13-1.18]	1.12 [1.10-1.14]	1.10 [1.07-1.12]	1.09 [1.07-1.11]	1.05 [0.96-1.16]	1.11 [1.09-1.13]	34,297	33,572	30,035	1,682	32,837
M30	1.17 [0.92-1.48]	1.30 [0.98-1.72]	1.25 [0.94-1.66]		1.14 [0.90-1.45]	1.08 [0.85-1.38]	1.10 [0.82-1.49]	1.09 [0.81-1.47]		1.02 [0.80-1.31]	222	147	137	NA	200
M31	1.26 [1.17-1.36]	1.25 [1.16-1.35]	1.24 [1.15-1.33]		1.19 [1.11-1.29]	1.20 [1.11-1.29]	1.18 [1.09-1.27]	1.15 [1.07-1.24]		1.14 [1.05-1.23]	2,102	2,113	2,038	NA	1,942
M32	1.52 [1.36-1.69]	1.54 [1.38-1.71]	1.50 [1.33-1.69]		1.48 [1.33-1.64]	1.43 [1.28-1.59]	1.40 [1.25-1.56]	1.38 [1.22-1.56]		1.39 [1.25-1.55]	1,094	1,102	841	NA	1,030
M33	1.93 [1.53-2.43]	2.01 [1.59-2.53]	1.94 [1.52-2.47]		1.74 [1.37-2.22]	1.83 [1.44-2.32]	1.92 [1.51-2.43]	1.89 [1.48-2.42]		1.68 [1.31-2.15]	248	246	217	NA	207
M34	1.45 [1.23-1.72]	1.44 [1.22-1.70]	1.44 [1.21-1.71]		1.43 [1.21-1.69]	1.38 [1.16-1.64]	1.34 [1.13-1.60]	1.33 [1.12-1.60]		1.36 [1.14-1.61]	423	418	389	NA	398
M35	1.28 [1.24-1.33]	1.29 [1.25-1.33]	1.29 [1.24-1.33]	1.19 [0.99-1.43]	1.26 [1.22-1.31]	1.21 [1.17-1.25]	1.20 [1.16-1.24]	1.20 [1.16-1.25]	1.12 [0.92-1.36]	1.20 [1.15-1.24]	10,397	10,287	9,374	430	9,715
M40	1.20 [1.11-1.29]	1.15 [1.07-1.23]	1.18 [1.09-1.27]		1.15 [1.07-1.24]	1.14 [1.06-1.23]	1.08 [1.00-1.16]	1.11 [1.03-1.20]		1.11 [1.03-1.20]	2,299	2,286	2,196	NA	2,274
M41	1.19 [1.14-1.25]	1.20 [1.15-1.26]	1.18 [1.12-1.24]	0.96 [0.80-1.16]	1.13 [1.08-1.18]	1.13 [1.08-1.18]	1.13 [1.08-1.18]	1.10 [1.05-1.16]	0.95 [0.78-1.15]	1.08 [1.03-1.13]	5,972	5,807	5,191	404	5,718
M43	1.21 [1.15-1.27]	1.21 [1.15-1.26]	1.20 [1.14-1.26]	0.95 [0.70-1.29]	1.15 [1.10-1.21]	1.13 [1.08-1.19]	1.10 [1.04-1.15]	1.10 [1.04-1.15]	0.88 [0.64-1.20]	1.09 [1.04-1.14]	5,217	5,181	4,835	145	5,034
M45	1.30 [1.18-1.43]	1.38 [1.25-1.52]	1.32 [1.19-1.46]		1.35 [1.22-1.49]	1.22 [1.10-1.35]	1.26 [1.14-1.40]	1.21 [1.09-1.34]		1.26 [1.14-1.40]	1,245	1,261	1,131	NA	1,197
M46	1.29 [1.23-1.36]	1.29 [1.23-1.36]	1.28 [1.22-1.35]		1.24 [1.18-1.30]	1.19 [1.13-1.25]	1.16 [1.10-1.22]	1.15 [1.09-1.21]		1.17 [1.11-1.23]	4,513	4,531	4,319	NA	4,386
M47	1.22 [1.20-1.25]	1.24 [1.21-1.27]	1.22 [1.19-1.25]		1.19 [1.17-1.22]	1.14 [1.11-1.17]	1.12 [1.10-1.15]	1.11 [1.08-1.13]		1.13 [1.10-1.15]	25,023	25,080	24,283	NA	24,218
M48	1.18 [1.15-1.22]	1.18 [1.15-1.22]	1.19 [1.15-1.22]		1.16 [1.12-1.19]	1.11 [1.08-1.15]	1.09 [1.05-1.12]	1.08 [1.05-1.12]		1.10 [1.07-1.14]	14,272	14,276	14,003	NA	13,875
M49	1.01 [0.86-1.18]	1.03 [0.88-1.22]	1.11 [0.94-1.31]		1.02 [0.87-1.20]	0.99 [0.84-1.16]	1.01 [0.86-1.19]	1.09 [0.92-1.29]		1.03 [0.88-1.22]	420	417	403	NA	397
M50	1.22 [1.15-1.29]	1.19 [1.13-1.26]	1.20 [1.13-1.27]		1.13 [1.07-1.20]	1.15 [1.08-1.21]	1.08 [1.02-1.14]	1.08 [1.02-1.15]		1.08 [1.02-1.14]	3,530	3,530	3,277	NA	3,384
M51	1.19 [1.16-1.22]	1.18 [1.15-1.21]	1.19 [1.16-1.22]	0.98 [0.79-1.22]	1.15 [1.12-1.18]	1.11 [1.08-1.14]	1.06 [1.04-1.09]	1.07 [1.04-1.11]	0.88 [0.70-1.11]	1.09 [1.06-1.12]	16,572	16,674	14,545	277	15,952
M53	1.12 [1.01-1.25]	1.11 [1.00-1.23]	1.11 [0.99-1.25]		1.11 [1.00-1.23]	1.03 [0.93-1.15]	0.98 [0.88-1.10]	0.99 [0.88-1.12]		1.03 [0.92-1.14]	1,024	1,019	808	NA	1,002
M54	1.19 [1.17-1.21]	1.18 [1.16-1.20]	1.19 [1.17-1.21]	1.00 [0.91-1.09]	1.14 [1.12-1.16]	1.11 [1.09-1.13]	1.07 [1.05-1.09]	1.08 [1.06-1.10]	0.98 [0.89-1.07]	1.08 [1.07-1.10]	42,416	42,685	35,152	1,735	40,355
M60	1.33 [1.18-1.51]	1.32 [1.16-1.50]	1.36 [1.18-1.55]		1.31 [1.15-1.48]	1.29 [1.14-1.47]	1.24 [1.09-1.42]	1.29 [1.12-1.48]		1.29 [1.13-1.47]	785	716	637	NA	744
M62	1.18 [1.11-1.25]	1.18 [1.11-1.25]	1.18 [1.11-1.26]	0.88 [0.67-1.15]	1.12 [1.06-1.19]	1.14 [1.07-1.21]	1.11 [1.05-1.18]	1.13 [1.06-1.20]	0.81 [0.61-1.08]	1.11 [1.05-1.18]	3,455	3,385	3,050	174	3,341
M65	1.25 [1.20-1.30]	1.24 [1.19-1.29]	1.23 [1.17-1.28]	1.24 [1.03-1.48]	1.21 [1.17-1.27]	1.16 [1.11-1.21]	1.14 [1.09-1.19]	1.13 [1.08-1.18]	1.18 [0.98-1.42]	1.15 [1.10-1.20]	6,878	6,642	5,811	408	6,406
M66	1.20 [1.05-1.37]	1.19 [1.05-1.36]	1.12 [0.99-1.28]		1.10 [0.97-1.26]	1.14 [1.00-1.30]	1.13 [0.99-1.29]	1.06 [0.92-1.21]		1.06 [0.92-1.21]	692	685	647	NA	620
M67	1.18 [1.12-1.25]	1.19 [1.12-1.25]	1.25 [1.18-1.33]	1.02 [0.87-1.20]	1.14 [1.08-1.20]	1.12 [1.06-1.18]	1.11 [1.05-1.17]	1.17 [1.09-1.24]	0.98 [0.83-1.16]	1.09 [1.03-1.15]	4,100	3,879	2,927	502	3,701
M70	1.28 [1.20-1.36]	1.27 [1.20-1.36]	1.27 [1.19-1.35]		1.22 [1.15-1.30]	1.19 [1.11-1.27]	1.15 [1.08-1.23]	1.15 [1.08-1.23]		1.14 [1.07-1.22]	2,910	2,902	2,712	NA	2,800
M71	1.23 [1.14-1.34]	1.25 [1.15-1.35]	1.22 [1.12-1.32]		1.25 [1.15-1.35]	1.16 [1.07-1.26]	1.16 [1.07-1.26]	1.12 [1.03-1.22]		1.18 [1.09-1.28]	1,909	1,906	1,787	NA	1,819
M72	1.02 [0.96-1.08]	1.05 [0.99-1.11]	1.04 [0.98-1.10]		1.04 [0.99-1.11]	1.00 [0.94-1.06]	1.02 [0.96-1.08]	1.02 [0.96-1.08]		1.03 [0.97-1.09]	3,398	3,408	3,255	NA	3,060
M75	1.18 [1.14-1.21]	1.19 [1.15-1.22]	1.17 [1.13-1.21]		1.13 [1.10-1.17]	1.10 [1.06-1.13]	1.09 [1.05-1.12]	1.07 [1.03-1.10]		1.07 [1.04-1.11]	11,012	11,050	10,280	NA	10,333
M76	1.26 [1.13-1.41]	1.29 [1.15-1.44]	1.32 [1.17-1.49]		1.28 [1.15-1.43]	1.16 [1.04-1.31]	1.16 [1.03-1.30]	1.18 [1.04-1.34]		1.19 [1.06-1.33]	945	938	795	NA	918
M77	1.34 [1.25-1.45]	1.31 [1.22-1.41]	1.30 [1.20-1.40]		1.26 [1.17-1.35]	1.23 [1.14-1.33]	1.17 [1.09-1.27]	1.17 [1.08-1.26]		1.18 [1.09-1.27]	2,210	2,213	1,934	NA	2,089
M79	1.27 [1.25-1.29]	1.27 [1.25-1.30]	1.28 [1.26-1.31]	1.04 [0.95-1.14]	1.23 [1.20-1.25]	1.18 [1.16-1.20]	1.15 [1.13-1.17]	1.16 [1.14-1.19]	1.01 [0.91-1.11]	1.16 [1.14-1.18]	37,345	36,967	31,656	1,573	35,587
M80	1.19 [1.13-1.25]	1.21 [1.14-1.27]	1.20 [1.14-1.27]		1.18 [1.13-1.25]	1.14 [1.08-1.20]	1.13 [1.07-1.19]	1.12 [1.07-1.18]		1.14 [1.08-1.20]	4,601	4,591	4,563	NA	4,381
M81	1.21 [1.18-1.23]	1.20 [1.17-1.22]	1.20 [1.18-1.23]		1.19 [1.16-1.21]	1.15 [1.12-1.17]	1.11 [1.09-1.13]	1.12 [1.09-1.14]		1.13 [1.11-1.15]	28,513	28,498	28,165	NA	27,378
M83	1.30 [1.01-1.69]	1.10 [0.85-1.42]	1.35 [1.03-1.78]		1.24 [0.96-1.60]	1.20 [0.92-1.57]	1.05 [0.81-1.37]	1.25 [0.95-1.66]		1.18 [0.91-1.53]	173	174	162	NA	171
M84	1.15 [1.08-1.22]	1.16 [1.09-1.24]	1.16 [1.09-1.24]	0.88 [0.65-1.20]	1.14 [1.07-1.21]	1.10 [1.03-1.17]	1.08 [1.02-1.15]	1.09 [1.02-1.16]	0.87 [0.63-1.20]	1.12 [1.05-1.19]	3,150	3,100	2,746	144	2,964
M85	1.20 [1.15-1.26]	1.20 [1.14-1.25]	1.17 [1.12-1.23]	1.01 [0.70-1.44]	1.18 [1.13-1.24]	1.12 [1.07-1.17]	1.09 [1.04-1.14]	1.08 [1.03-1.13]	0.94 [0.65-1.37]	1.12 [1.07-1.17]	6,142	6,124	5,801	115	6,060
M86	1.22 [1.14-1.30]	1.22 [1.14-1.30]	1.23 [1.15-1.32]	1.41 [1.02-1.96]	1.22 [1.14-1.30]	1.18 [1.11-1.27]	1.13 [1.06-1.21]	1.15 [1.07-1.24]	1.36 [0.97-1.91]	1.22 [1.14-1.30]	2,723	2,618	2,525	139	2,667
M87	1.14 [1.03-1.27]	1.11 [1.00-1.24]	1.16 [1.04-1.30]		1.11 [1.00-1.23]	1.09 [0.98-1.22]	1.05 [0.94-1.17]	1.10 [0.98-1.23]		1.09 [0.98-1.21]	1,018	1,002	926	NA	984
M88	1.11 [0.97-1.27]	1.05 [0.92-1.20]	1.11 [0.97-1.27]		1.02 [0.89-1.17]	1.10 [0.96-1.25]	1.03 [0.90-1.18]	1.08 [0.94-1.24]		1.00 [0.87-1.15]	667	678	656	NA	598
M89	1.22 [1.14-1.30]	1.19 [1.11-1.27]	1.21 [1.12-1.30]	1.03 [0.77-1.37]	1.14 [1.07-1.22]	1.15 [1.07-1.23]	1.10 [1.02-1.18]	1.11 [1.03-1.20]	1.00 [0.74-1.35]	1.10 [1.03-1.18]	2,554	2,501	2,102	157	2,432
M90	1.06 [0.95-1.18]	1.03 [0.93-1.15]	1.02 [0.91-1.13]		0.97 [0.87-1.08]	1.06 [0.95-1.18]	1.03 [0.92-1.14]	1.00 [0.90-1.12]		0.99 [0.88-1.10]	982	978	944	NA	895
M91	1.17 [0.87-1.58]				1.17 [0.86-1.59]	1.12 [0.83-1.53]				1.12 [0.82-1.53]	137	NA	NA	NA	122
M92	1.38 [1.10-1.72]	1.36 [1.08-1.71]	1.33 [1.00-1.77]		1.27 [1.01-1.59]	1.27 [1.01-1.60]	1.24 [0.98-1.57]	1.20 [0.89-1.62]		1.16 [0.91-1.47]	266	244	142	NA	242
M93	1.31 [1.11-1.55]	1.21 [1.02-1.43]	1.25 [1.02-1.53]		1.13 [0.95-1.33]	1.22 [1.03-1.45]	1.10 [0.92-1.31]	1.16 [0.94-1.43]		1.05 [0.88-1.25]	462	406	276	NA	408
M94	1.25 [1.16-1.35]	1.28 [1.18-1.38]	1.33 [1.21-1.45]	0.99 [0.75-1.32]	1.25 [1.15-1.35]	1.12 [1.03-1.22]	1.12 [1.03-1.22]	1.18 [1.07-1.29]	0.98 [0.73-1.32]	1.17 [1.08-1.27]	1,962	1,982	1,444	170	1,932
M95	1.27 [1.06-1.52]	1.23 [1.02-1.47]	1.29 [1.02-1.63]		1.22 [1.02-1.47]	1.22 [1.01-1.46]	1.14 [0.94-1.37]	1.18 [0.93-1.51]		1.16 [0.96-1.40]	391	374	208	NA	346
M96	1.24 [1.14-1.36]	1.23 [1.12-1.34]	1.25 [1.15-1.37]		1.25 [1.14-1.36]	1.19 [1.08-1.30]	1.15 [1.05-1.26]	1.17 [1.07-1.29]		1.23 [1.12-1.34]	1,544	1,537	1,475	NA	1,548
M99	1.25 [1.06-1.47]	1.18 [1.01-1.40]	1.24 [1.05-1.47]		1.18 [1.00-1.39]	1.13 [0.96-1.34]	1.07 [0.90-1.26]	1.09 [0.92-1.30]		1.10 [0.94-1.30]	438	433	415	NA	420
Diseases of the genitourinary system (N00-N99)															
N02	1.17 [1.05-1.29]	1.19 [1.07-1.32]	1.20 [1.07-1.33]		1.12 [1.01-1.24]	1.14 [1.03-1.27]	1.14 [1.03-1.27]	1.16 [1.04-1.29]		1.11 [1.00-1.23]	1,082	1,092	959	NA	1,042
N03	1.20 [1.14-1.25]	1.21 [1.15-1.27]	1.21 [1.16-1.27]		1.20 [1.15-1.26]	1.15 [1.10-1.21]	1.13 [1.08-1.19]	1.14 [1.08-1.19]		1.17 [1.12-1.23]	5,200	5,207	5,138	NA	5,039
N04	1.28 [1.12-1.46]	1.30 [1.14-1.49]	1.22 [1.06-1.40]		1.14 [1.00-1.30]	1.21 [1.06-1.38]	1.23 [1.07-1.41]	1.14 [0.99-1.32]		1.12 [0.98-1.28]	698	661	586	NA	655
N05	1.37 [1.20-1.56]	1.30 [1.14-1.49]	1.31 [1.14-1.51]		1.26 [1.11-1.44]	1.33 [1.16-1.52]	1.23 [1.07-1.41]	1.24 [1.07-1.43]		1.24 [1.09-1.41]	722	674	591	NA	697
N08	1.23 [1.15-1.32]	1.22 [1.14-1.30]	1.22 [1.14-1.31]		1.16 [1.09-1.24]	1.19 [1.11-1.27]	1.11 [1.03-1.19]	1.10 [1.03-1.19]		1.16 [1.08-1.24]	2,513	2,519	2,385	NA	2,486

Supplementary Table 4: Hazard ratios and events from all cohorts (excluding non-consulters)

	Hazard ratio (99% confidence interval)										Events (in exposed)				
	crude					adjusted									
Outcome	any age	18+	40+	<18	hosp.	any age	18+	40+	<18	hosp.	any age	18+	40+	<18	hosp.
N10	1.10 [0.98-1.25]	1.10 [0.97-1.24]	1.16 [1.00-1.36]	0.92 [0.66-1.30]	1.06 [0.94-1.20]	1.05 [0.92-1.19]	1.01 [0.89-1.15]	1.08 [0.92-1.26]	0.95 [0.66-1.37]	1.03 [0.90-1.17]	776	778	472	111	728
N11	1.18 [1.00-1.40]	1.15 [0.97-1.36]	1.14 [0.95-1.38]		1.05 [0.89-1.25]	1.14 [0.96-1.35]	1.09 [0.92-1.29]	1.07 [0.89-1.30]		1.05 [0.88-1.24]	409	408	319	NA	383
N12	1.11 [1.05-1.17]	1.13 [1.08-1.19]	1.15 [1.08-1.23]	1.01 [0.87-1.16]	1.06 [1.01-1.12]	1.04 [0.99-1.10]	1.03 [0.98-1.09]	1.06 [0.99-1.13]	1.00 [0.86-1.16]	1.04 [0.98-1.10]	4,313	4,485	2,601	647	4,050
N13	1.01 [0.98-1.05]	1.02 [0.98-1.05]	1.03 [0.99-1.06]	0.83 [0.69-1.00]	1.00 [0.97-1.04]	1.00 [0.97-1.04]	1.00 [0.96-1.04]	1.01 [0.98-1.05]	0.81 [0.67-0.97]	1.01 [0.98-1.05]	9,107	9,032	8,165	372	8,586
N14	1.13 [0.87-1.46]	1.16 [0.89-1.51]	1.24 [0.95-1.61]		1.17 [0.91-1.51]	1.12 [0.86-1.45]	1.09 [0.83-1.42]	1.17 [0.90-1.54]		1.14 [0.88-1.48]	178	178	170	NA	177
N15	1.12 [0.91-1.38]	1.11 [0.90-1.36]	1.07 [0.86-1.34]		1.03 [0.84-1.26]	1.08 [0.87-1.34]	1.04 [0.84-1.28]	1.02 [0.81-1.28]		1.01 [0.82-1.24]	263	269	220	NA	264
N16	1.31 [0.98-1.75]	1.28 [0.96-1.71]	1.27 [0.93-1.74]		1.15 [0.87-1.52]	1.29 [0.96-1.74]	1.19 [0.88-1.60]	1.17 [0.85-1.62]		1.18 [0.89-1.56]	147	149	118	NA	149
N17	1.14 [1.13-1.16]	1.14 [1.12-1.15]	1.15 [1.13-1.16]	0.80 [0.66-0.98]	1.13 [1.11-1.14]	1.12 [1.10-1.14]	1.08 [1.07-1.10]	1.09 [1.07-1.10]	0.82 [0.66-1.01]	1.12 [1.10-1.14]	63,036	63,147	61,984	322	61,515
N18	1.15 [1.13-1.16]	1.15 [1.13-1.17]	1.15 [1.13-1.17]	1.00 [0.70-1.42]	1.12 [1.11-1.14]	1.11 [1.10-1.13]	1.08 [1.07-1.10]	1.08 [1.06-1.10]	0.98 [0.68-1.41]	1.11 [1.09-1.12]	55,729	55,773	55,339	109	54,713
N19	1.20 [1.15-1.25]	1.20 [1.16-1.25]	1.19 [1.14-1.23]		1.19 [1.14-1.23]	1.16 [1.12-1.21]	1.14 [1.09-1.18]	1.12 [1.08-1.17]		1.16 [1.12-1.21]	7,585	7,557	7,382	NA	7,161
N20	1.04 [1.00-1.08]	1.04 [1.00-1.08]	1.04 [1.00-1.08]	0.78 [0.61-0.99]	1.04 [1.00-1.08]	1.01 [0.97-1.05]	0.99 [0.95-1.03]	0.99 [0.95-1.03]	0.82 [0.63-1.06]	1.03 [0.98-1.07]	7,234	7,316	6,153	196	6,744
N21	0.94 [0.87-1.02]	0.92 [0.85-0.99]	0.94 [0.87-1.02]		0.91 [0.84-0.99]	0.94 [0.87-1.02]	0.91 [0.84-0.99]	0.93 [0.86-1.01]		0.92 [0.85-1.00]	1,683	1,664	1,588	NA	1,555
N23	1.06 [0.98-1.16]	1.04 [0.95-1.13]	1.01 [0.92-1.12]		0.99 [0.91-1.08]	1.00 [0.91-1.09]	0.96 [0.88-1.05]	0.93 [0.84-1.03]		0.95 [0.87-1.04]	1,495	1,565	1,034	NA	1,354
N25	1.20 [1.02-1.40]	1.24 [1.05-1.45]	1.24 [1.05-1.46]		1.07 [0.91-1.25]	1.17 [1.00-1.37]	1.19 [1.01-1.40]	1.19 [1.01-1.40]		1.07 [0.92-1.26]	467	457	431	NA	439
N26	1.08 [0.96-1.21]	1.08 [0.96-1.21]	1.05 [0.93-1.18]		1.03 [0.92-1.15]	1.04 [0.92-1.17]	1.00 [0.89-1.13]	0.97 [0.86-1.10]		1.02 [0.91-1.15]	857	858	791	NA	858
N27	1.24 [1.06-1.45]	1.22 [1.04-1.44]	1.28 [1.08-1.51]		1.12 [0.95-1.30]	1.23 [1.05-1.44]	1.17 [1.00-1.38]	1.23 [1.03-1.45]		1.10 [0.94-1.29]	487	460	417	NA	459
N28	1.10 [1.06-1.13]	1.09 [1.06-1.13]	1.09 [1.06-1.13]	0.89 [0.70-1.13]	1.08 [1.04-1.11]	1.07 [1.03-1.10]	1.03 [1.00-1.07]	1.04 [1.00-1.07]	0.93 [0.73-1.20]	1.06 [1.03-1.10]	11,855	11,792	11,234	220	11,417
N30	1.16 [1.11-1.21]	1.16 [1.11-1.21]	1.12 [1.06-1.17]	1.16 [0.86-1.57]	1.12 [1.07-1.17]	1.12 [1.07-1.17]	1.10 [1.05-1.15]	1.06 [1.01-1.11]	1.11 [0.82-1.52]	1.10 [1.05-1.15]	5,348	5,414	4,754	155	5,061
N31	1.23 [1.13-1.34]	1.28 [1.18-1.40]	1.26 [1.15-1.38]		1.15 [1.06-1.25]	1.15 [1.05-1.25]	1.16 [1.06-1.27]	1.15 [1.05-1.26]		1.11 [1.02-1.20]	1,598	1,581	1,374	NA	1,545
N32	1.13 [1.10-1.16]	1.12 [1.09-1.16]	1.12 [1.09-1.15]	0.94 [0.76-1.15]	1.10 [1.07-1.13]	1.09 [1.06-1.12]	1.07 [1.04-1.10]	1.07 [1.04-1.10]	0.92 [0.74-1.15]	1.08 [1.05-1.11]	15,374	15,340	14,355	299	14,514
N34	1.23 [0.99-1.54]	1.17 [0.94-1.47]	1.14 [0.89-1.46]		1.11 [0.88-1.40]	1.21 [0.97-1.52]	1.14 [0.91-1.44]	1.11 [0.87-1.43]		1.10 [0.87-1.39]	242	233	190	NA	206
N35	1.20 [1.15-1.26]	1.18 [1.13-1.24]	1.19 [1.14-1.25]	1.14 [0.88-1.48]	1.14 [1.09-1.20]	1.16 [1.11-1.22]	1.12 [1.07-1.18]	1.14 [1.08-1.20]	1.09 [0.83-1.42]	1.12 [1.07-1.18]	5,195	5,138	4,621	212	4,820
N36	1.15 [1.05-1.26]	1.16 [1.06-1.27]	1.13 [1.03-1.24]		1.07 [0.97-1.17]	1.11 [1.02-1.22]	1.12 [1.03-1.23]	1.07 [0.98-1.18]		1.05 [0.95-1.15]	1,420	1,392	1,235	NA	1,284
N39	1.12 [1.11-1.14]	1.12 [1.11-1.14]	1.12 [1.11-1.14]	1.05 [0.97-1.13]	1.10 [1.09-1.12]	1.09 [1.08-1.11]	1.07 [1.06-1.08]	1.07 [1.06-1.09]	1.02 [0.95-1.11]	1.09 [1.07-1.10]	71,936	71,311	66,045	2,343	67,612
N40	1.08 [1.06-1.10]	1.08 [1.05-1.10]	1.07 [1.05-1.10]		1.06 [1.04-1.09]	1.05 [1.02-1.07]	1.04 [1.02-1.07]	1.03 [1.01-1.06]		1.04 [1.01-1.06]	23,087	23,094	23,034	NA	21,241
N41	1.03 [0.94-1.12]	1.01 [0.93-1.11]	1.00 [0.91-1.09]		1.00 [0.92-1.09]	1.00 [0.91-1.09]	0.98 [0.90-1.08]	0.97 [0.89-1.06]		0.98 [0.89-1.07]	1,382	1,385	1,366	NA	1,291
N42	1.04 [0.97-1.12]	1.02 [0.95-1.10]	1.04 [0.97-1.12]		1.00 [0.93-1.08]	1.02 [0.94-1.10]	1.00 [0.93-1.08]	1.01 [0.94-1.09]		0.99 [0.92-1.07]	1,963	1,959	1,954	NA	1,837
N43	1.05 [0.98-1.13]	1.11 [1.02-1.20]	1.08 [1.00-1.18]	1.02 [0.86-1.20]	1.11 [1.02-1.19]	1.04 [0.96-1.12]	1.08 [1.00-1.18]	1.06 [0.97-1.15]	0.98 [0.83-1.17]	1.10 [1.01-1.18]	2,105	1,693	1,601	461	1,909
N44	1.19 [0.96-1.47]			1.12 [0.89-1.40]	1.20 [0.94-1.53]	1.16 [0.93-1.45]			1.11 [0.88-1.40]	1.19 [0.93-1.52]	335	NA	NA	310	269
N45	1.08 [0.99-1.17]	1.12 [1.02-1.23]	1.10 [1.00-1.22]	1.05 [0.82-1.34]	1.06 [0.97-1.15]	1.04 [0.95-1.14]	1.05 [0.96-1.16]	1.04 [0.94-1.15]	1.00 [0.78-1.30]	1.04 [0.95-1.14]	1,561	1,400	1,224	233	1,425
N47	1.26 [1.19-1.32]	1.22 [1.15-1.29]	1.23 [1.16-1.31]	1.34 [1.22-1.46]	1.22 [1.16-1.29]	1.22 [1.16-1.29]	1.17 [1.10-1.24]	1.18 [1.11-1.26]	1.32 [1.20-1.45]	1.21 [1.15-1.28]	5,234	3,611	3,141	1,811	4,483
N48	1.25 [1.18-1.33]	1.29 [1.21-1.38]	1.24 [1.15-1.32]	1.25 [1.09-1.45]	1.22 [1.15-1.30]	1.21 [1.14-1.29]	1.22 [1.14-1.31]	1.17 [1.09-1.26]	1.23 [1.06-1.42]	1.20 [1.13-1.28]	3,468	2,865	2,645	697	3,136
N49	1.15 [0.98-1.35]	1.32 [1.12-1.55]	1.24 [1.05-1.47]		1.15 [0.98-1.35]	1.13 [0.96-1.33]	1.25 [1.06-1.48]	1.18 [0.99-1.40]		1.15 [0.97-1.36]	455	442	414	NA	427
N50	1.11 [1.04-1.18]	1.14 [1.07-1.21]	1.14 [1.07-1.22]	1.00 [0.85-1.17]	1.10 [1.04-1.17]	1.07 [1.00-1.14]	1.09 [1.02-1.16]	1.09 [1.02-1.17]	0.96 [0.81-1.14]	1.08 [1.01-1.15]	3,220	2,832	2,528	536	2,959
N60	1.14 [1.04-1.24]	1.09 [1.00-1.19]	1.11 [1.00-1.22]		1.06 [0.97-1.16]	1.10 [1.00-1.20]	1.04 [0.95-1.13]	1.07 [0.97-1.18]		1.05 [0.95-1.15]	1,436	1,450	1,115	NA	1,265
N61	1.27 [1.15-1.41]	1.29 [1.17-1.43]	1.41 [1.23-1.61]		1.25 [1.12-1.39]	1.23 [1.10-1.37]	1.18 [1.06-1.32]	1.29 [1.12-1.48]		1.22 [1.10-1.36]	1,109	1,127	649	NA	1,004
N62	1.16 [1.03-1.32]	1.20 [1.07-1.36]	1.24 [1.07-1.44]		1.15 [1.01-1.31]	1.10 [0.97-1.26]	1.12 [0.99-1.27]	1.15 [0.99-1.34]		1.12 [0.98-1.27]	754	784	536	NA	661
N63	1.11 [1.02-1.21]	1.10 [1.01-1.19]	1.16 [1.06-1.27]		1.06 [0.98-1.16]	1.07 [0.98-1.16]	1.04 [0.96-1.14]	1.09 [0.99-1.20]		1.05 [0.96-1.14]	1,637	1,646	1,346	NA	1,444
N64	1.19 [1.10-1.30]	1.17 [1.08-1.27]	1.20 [1.09-1.31]		1.19 [1.09-1.29]	1.15 [1.06-1.26]	1.09 [1.01-1.19]	1.13 [1.02-1.24]		1.16 [1.06-1.26]	1,640	1,676	1,263	NA	1,517
N70	1.04 [0.93-1.16]	1.09 [0.98-1.21]	0.97 [0.84-1.12]		1.04 [0.93-1.16]	1.01 [0.90-1.13]	1.03 [0.92-1.15]	0.92 [0.79-1.07]		1.01 [0.90-1.14]	963	997	495	NA	845
N71	1.12 [0.97-1.30]	1.21 [1.05-1.39]	1.25 [1.05-1.49]		1.11 [0.96-1.29]	1.06 [0.91-1.23]	1.14 [0.99-1.32]	1.17 [0.97-1.40]		1.05 [0.90-1.22]	550	577	354	NA	518
N72	1.08 [0.97-1.20]	1.09 [0.98-1.22]	1.10 [0.95-1.27]		1.01 [0.90-1.13]	1.04 [0.93-1.16]	1.06 [0.95-1.19]	1.06 [0.92-1.23]		0.98 [0.88-1.11]	946	995	506	NA	811
N73	1.07 [1.02-1.11]	1.08 [1.03-1.12]	1.07 [1.01-1.14]	1.02 [0.87-1.19]	1.04 [1.00-1.09]	1.02 [0.97-1.06]	1.00 [0.95-1.05]	1.01 [0.95-1.08]	1.04 [0.88-1.22]	1.01 [0.97-1.06]	5,763	6,012	2,721	547	5,310
N75	1.03 [0.92-1.16]	1.03 [0.92-1.15]	1.03 [0.86-1.22]		1.01 [0.90-1.14]	1.01 [0.90-1.13]	0.98 [0.87-1.10]	1.00 [0.84-1.20]		1.01 [0.89-1.14]	858	904	333	NA	743
N76	1.32 [1.21-1.44]	1.32 [1.21-1.44]	1.37 [1.23-1.52]	1.10 [0.85-1.43]	1.33 [1.22-1.45]	1.25 [1.14-1.36]	1.21 [1.10-1.32]	1.28 [1.15-1.43]	1.13 [0.86-1.47]	1.28 [1.18-1.40]	1,675	1,653	1,035	208	1,517
N77	1.18 [1.10-1.28]	1.21 [1.12-1.30]	1.36 [1.21-1.53]	0.90 [0.74-1.10]	1.14 [1.06-1.23]	1.10 [1.01-1.19]	1.07 [1.00-1.16]	1.19 [1.05-1.35]	0.88 [0.71-1.09]	1.08 [1.00-1.17]	2,075	2,268	875	308	2,002
N80	1.07 [1.02-1.12]	1.09 [1.04-1.14]	1.03 [0.97-1.11]	0.96 [0.83-1.11]	1.05 [1.01-1.11]	1.01 [0.96-1.06]	1.01 [0.96-1.05]	0.98 [0.92-1.05]	0.93 [0.80-1.09]	1.01 [0.96-1.06]	5,599	5,944	2,392	578	4,982
N81	1.14 [1.11-1.18]	1.14 [1.10-1.18]	1.13 [1.09-1.17]		1.11 [1.07-1.15]	1.10 [1.06-1.13]	1.09 [1.05-1.12]	1.08 [1.04-1.12]		1.07 [1.03-1.11]	10,329	10,342	9,347	NA	9,208
N82	1.25 [1.08-1.45]	1.20 [1.04-1.39]	1.18 [1.01-1.39]		1.22 [1.06-1.42]	1.19 [1.03-1.39]	1.13 [0.97-1.31]	1.10 [0.93-1.29]		1.19 [1.03-1.38]	528	523	427	NA	510
N83	1.08 [1.04-1.12]	1.08 [1.05-1.12]	1.06 [1.01-1.11]	0.96 [0.86-1.08]	1.05 [1.01-1.09]	1.03 [0.99-1.07]	1.00 [0.97-1.04]	1.01 [0.96-1.06]	0.94 [0.84-1.06]	1.01 [0.98-1.05]	9,134	9,544	4,765	968	8,230
N84	1.09 [1.05-1.13]	1.09 [1.05-1.13]	1.09 [1.05-1.14]		1.06 [1.01-1.10]	1.08 [1.04-1.12]	1.07 [1.03-1.12]	1.08 [1.04-1.13]		1.05 [1.01-1.09]	7,674	7,738	6,706	NA	6,387
N85	1.11 [1.07-1.16]	1.12 [1.07-1.16]	1.10 [1.05-1.15]	1.06 [0.82-1.35]	1.08 [1.04-1.13]	1.08 [1.04-1.13]	1.07 [1.02-1.11]	1.06 [1.01-1.11]	1.00 [0.77-1.31]	1.07 [1.02-1.11]	6,475	6,591	5,058	214	5,716
N86	1.09 [1.01-1.18]	1.10 [1.02-1.19]	1.14 [0.98-1.33]	0.88 [0.70-1.09]	1.06 [0.98-1.16]	1.05 [0.97-1.14]	1.05 [0.97-1.14]	1.11 [0.95-1.30]	0.88 [0.70-1.11]	1.02 [0.94-1.11]	1,855	2,036	466	256	1,621
N87	1.03 [0.96-1.11]	1.06 [0.98-1.14]	1.00 [0.87-1.13]	1.19 [0.94-1.51]	1.04 [0.96-1.13]	1.00 [0.93-1.08]	1.01 [0.93-1.08]	0.96 [0.84-1.10]	1.23 [0.95-1.58]	1.04 [0.96-1.12]	2,147	2,231	608	245	1,742
N88	1.15 [1.06-1.24]	1.14 [1.06-1.23]	1.11 [1.02-1.21]		1.11 [1.02-1.20]	1.12 [1.04-1.21]	1.11 [1.03-1.20]	1.07 [0.98-1.16]		1.09 [1.01-1.18]	1,943	1,984	1,583	NA	1,724
N89	1.19 [1.12-1.26]	1.20 [1.14-1.27]	1.24 [1.13-1.35]	0.92 [0.77-1.09]	1.12 [1.05-1.19]	1.13 [1.07-1.21]	1.11 [1.05-1.18]	1.18 [1.08-1.29]	0.89 [0.74-1.07]	1.08 [1.01-1.15]	3,319	3,497	1,512	389	3,009
N90	1.29 [1.22-1.37]	1.26 [1.19-1.34]	1.31 [1.22-1.40]	1.01 [0.77-1.33]	1.21 [1.14-1.29]	1.23 [1.16-1.31]	1.19 [1.12-1.27]	1.23 [1.15-1.32]	1.04 [0.78-1.38]	1.17 [1.10-1.24]	3,385	3,431	2,367	178	3,050
N91	1.22 [1.01-1.47]	1.23 [1.02-1.48]	1.65 [1.20-2.27]		1.16 [0.95-1.40]	1.16 [0.95-1.41]	1.09 [0.90-1.33]	1.61 [1.16-2.24]		1.09 [0.89-1.32]	343	349	121	NA	305
N92	1.13 [1.09-1.16]	1.14 [1.11-1.18]	1.13 [1.09-1.18]	1.01 [0.88-1.16]	1.10 [1.07-1.14]	1.07 [1.04-1.11]	1.06 [1.02-1.09]	1.07 [1.03-1.11]	1.01 [0.87-1.16]	1.06 [1.03-1.10]	12,290	12,542	7,436	711	10,871
N93	1.11 [1.07-1.16]	1.12 [1.07-1.17]	1.11 [1.04-1.18]	0.99 [0.86-1.14]	1.04 [0.99-1.08]	1.07 [1.02-1.12]	1.04 [0.99-1.08]	1.05 [0.99-1.12]	0.99 [0.86-1.15]	1.01 [0.97-1.06]	5,970	6,258	2,939	622	5,380
N94	1.17 [1.11-1.23]	1.18 [1.12-1.24]	1.17 [1.08-1.26]	1.13 [0.98-1.31]	1.14 [1.09-1.20]	1.09 [1.04-1.15]	1.06 [1.01-1.12]	1.09 [1.00-1.17]	1.10 [0.94-1.28]	1.08 [1.03-1.14]	4,819	5,103	1,880	626	4,356
N95	1.18 [1.14-1.23]	1.15 [1.11-1.20]	1.18 [1.13-1.22]		1.14 [1.10-1.19]	1.16 [1.11-1.20]	1.11 [1.07-1.15]	1.14 [1.09-1.18]		1.12 [1.08-1.17]	7,543	7,524	7,504	NA	6,451
N97	0.89 [0.79-1.00]	0.93 [0.83-1.05]			1.01 [0.89-1.16]	0.88 [0.78-0.99]	0.93 [0.82-1.04]			1.01 [0.88-1.16]	781	805	NA	NA	534
N99	1.13 [1.04-1.22]	1.13 [1.05-1.22]	1.11 [1.02-1.21]		1.11 [1.02-1.20]	1.10 [1.01-1.19]	1.08 [1.00-1.17]	1.07 [0.98-1.16]		1.09 [1.00-1.18]	1,838	1,819	1,505	NA	1,733
Pregnancy, childbirth and the puerperium (O00-O9A)															
O00	0.96 [0.89-1.05]	1.01 [0.93-1.09]		1.02 [0.81-1.28]	0.96 [0.88-1.05]	0.95 [0.87-1.04]	0.97 [0.89-1.06]		1.03 [0.81-1.31]	0.96 [0.88-1.05]	1,619	1,737	NA	247	1,479
O02	1.01 [0.95-1.06]	1.02 [0.97-1.07]	0.91 [0.77-1.07]	0.91 [0.77-1.07]	1.01 [0.95-1.06]	0.99 [0.93-1.04]	1.00 [0.95-1.06]	0.94 [0.80-1.12]	0.93 [0.78-1.11]	1.00 [0.95-1.06]	3,955	4,214	375	441	3,411
O03	1.00 [0.95-1.04]	0.99 [0.95-1.03]	0.93 [0.81-1.06]	0.92 [0.81-1.04]	0.98 [0.93-1.02]	0.99 [0.95-1.04]	0.97 [0.92-1.01]	0.95 [0.82-1.08]	0.93 [0.82-1.06]	0.98 [0.94-1.03]	5,746	6,180	560	794	5,036
O04	0.99 [0.94-1.03]	0.98 [0.94-1.02]	0.90 [0.75-1.07]	0.97 [0.88-1.06]	0.94 [0.90-0.98]	0.98 [0.93-1.03]	0.95 [0.91-0.99]	0.92 [0.76-1.11]	1.02 [0.92-1.12]	0.95 [0.90-0.99]	5,800	6,654	311	1,416	5,051
O08	0.99 [0.86-1.14]	0.98 [0.86-1.12]		0.99 [0.74-1.34]	0.98 [0.86-1.13]	0.96 [0.83-1.11]	0.93 [0.81-1.06]		1.10 [0.79-1.53]	0.99 [0.86-1.14]	578	644	NA	131	554
O10	0.96 [0.85-1.09]	1.00 [0.88-1.12]	1.05 [0.80-1.37]		0.95 [0.84-1.07]	0.94 [0.83-1.07]	0.94 [0.83-1.07]	0.94 [0.71-1.25]		0.93 [0.82-1.06]	744	787	142	NA	692
O11	0.98 [0.72-1.35]	1.04 [0.76-1.41]			1.07 [0.77-1.49]	0.93 [0.67-1.30]	0.94 [0.68-1.30]			1.04 [0.74-1.45]	115	121	NA	NA	108
O12	1.05 [0.96-1.15]	1.07 [0.98-1.17]		0.91 [0.72-1.15]	1.04 [0.94-1.14]	1.01 [0.92-1.12]	1.01 [0.92-1.11]		0.90 [0.70-1.14]	1.00 [0.90-1.10]	1,328	1,473	NA	234	1,175
O13	1.00 [0.94-1.06]	1.02 [0.97-1.08]	0.91 [0.73-1.13]	0.94 [0.80-1.09]	1.02 [0.96-1.08]	0.99 [0.93-1.04]	1.01 [0.95-1.07]	0.92 [0.73-1.15]	0.94 [0.80-1.10]	1.01 [0.95-1.08]	3,626	4,006	201	519	2,911
O14	1.02 [0.95-1.09]	1.01 [0.95-1.08]	0.92 [0.70-1.21]	0.99 [0.83-1.17]	1.00 [0.93-1.08]	1.01 [0.94-1.08]	1.00 [0.93-1.07]	0.92 [0.69-1.22]	1.02 [0.85-1.22]	0.98 [0.91-1.06]	2,545	2,861	136	448	1,996
O16	1.00 [0.94-1.07]	1.00 [0.94-1.06]	0.81 [0.63-1.04]	0.92 [0.77-1.11]	0.99 [0.92-1.06]	1.00 [0.93-1.06]	0.99 [0.93-1.05]	0.82 [0.63-1.06]	0.92 [0.76-1.11]	0.98 [0.91-1.05]	2,949	3,170	152	376	2,303
O20	1.00 [0.95-1.05]	1.03 [0.98-1.08]	0.89 [0.72-1.10]	0.93 [0.83-1.04]	0.98 [0.94-1.03]	0.98 [0.93-1.03]	0.99 [0.94-1.04]	0.96 [0.77-1.20]	0.94 [0.84-1.06]	0.98 [0.93-1.03]	4,983	5,510	220	945	4,460
O21	1.03 [0.97-1.09]	1.06 [1.01-1.12]		0.88 [0.78-0.99]	0.98 [0.93-1.04]	0.98 [0.92-1.04]	0.98 [0.93-1.04]		0.88 [0.77-1.00]	0.96 [0.91-1.02]	3,415	3,979	NA	821	3,147
O22	1.03 [0.90-1.18]	1.00 [0.87-1.13]			0.99 [0.87-1.13]	1.04 [0.91-1.20]	1.01 [0.88-1.15]			1.01 [0.88-1.15]	654	664	NA	NA	603
O23	1.07 [1.02-1.13]	1.10 [1.04-1.16]		0.94 [0.84-1.06]	1.05 [1.00-1.11]	1.04 [0.99-1.10]	1.03 [0.98-1.09]		0.95 [0.84-1.08]	1.04 [0.98-1.10]	4,038	4,631	NA	895	3,752
O24	0.97 [0.93-1.02]	0.99 [0.94-1.03]	0.92 [0.80-1.07]	0.86 [0.75-0.99]	1.00 [0.95-1.04]	0.97 [0.93-1.02]	0.99 [0.94-1.03]	0.93 [0.80-1.08]	0.92 [0.79-1.06]	0.99 [0.94-1.04]	5,280	5,620	470	646	4,797
O26	1.06 [1.03-1.09]	1.08 [1.05-1.11]	0.83 [0.72-0.96]	0.89 [0.83-0.95]	1.03 [1.00-1.06]	1.03 [1.00-1.06]	1.02 [1.00-1.05]	0.83 [0.72-0.97]	0.89 [0.83-0.95]	1.01 [0.98-1.04]	15,473	17,384	467	2,941	13,341
O28	1.00 [0.91-1.09]	0.99 [0.91-1.07]		0.94 [0.78-1.14]	0.96 [0.88-1.05]	0.96 [0.87-1.05]	0.93 [0.85-1.02]		0.94 [0.76-1.15]	0.94 [0.86-1.03]	1,479	1,654	NA	327	1,361
O30	0.93 [0.86-1.01]	0.94 [0.87-1.02]		1.20 [0.95-1.51]	0.95 [0.88-1.03]	0.92 [0.85-1.00]	0.93 [0.86-1.01]		1.23 [0.96-1.57]	0.94 [0.86-1.02]	1,737	1,884	NA	242	1,571
O31	1.01 [0.72-1.40]	1.12 [0.80-1.55]			1.06 [0.77-1.47]	0.97 [0.69-1.38]	1.03 [0.73-1.46]			1.05 [0.75-1.47]	106	111	NA	NA	105
O32	0.94 [0.91-0.98]	0.98 [0.94-1.01]	0.90 [0.77-1.05]	0.86 [0.78-0.96]	0.96 [0.92-1.00]	0.94 [0.90-0.98]	0.98 [0.94-1.02]	0.95 [0.80-1.12]	0.87 [0.78-0.98]	0.96 [0.92-1.00]	7,303	7,977	390	1,013	6,152
O33	0.94 [0.73-1.21]	0.97 [0.76-1.23]			0.91 [0.71-1.18]	0.90 [0.70-1.17]	0.93 [0.72-1.19]			0.90 [0.69-1.17]	173	180	NA	NA	152
O34	1.00 [0.97-1.03]	1.01 [0.98-1.04]	0.90 [0.81-1.01]	0.84 [0.76-0.92]	0.98 [0.95-1.01]	1.01 [0.97-1.04]	1.01 [0.98-1.04]	0.96 [0.86-1.07]	0.86 [0.78-0.95]	0.98 [0.95-1.01]	13,334	13,998	906	1,369	13,034
O35	0.95 [0.87-1.03]	0.97 [0.90-1.05]	0.88 [0.68-1.13]	0.91 [0.73-1.13]	0.97 [0.90-1.06]	0.94 [0.86-1.02]	0.97 [0.89-1.05]	0.93 [0.71-1.22]	0.93 [0.74-1.18]	0.98 [0.90-1.06]	1,742	1,903	149	253	1,577
O36	1.02 [1.00-1.05]	1.04 [1.02-1.07]	0.91 [0.81-1.02]	0.93 [0.88-0.98]	1.02 [0.99-1.04]	1.00 [0.98-1.03]	1.01 [0.99-1.04]	0.93 [0.82-1.05]	0.95 [0.89-1.00]	1.01 [0.98-1.04]	20,280	23,035	702	4,313	17,222
O40	1.04 [0.96-1.13]	1.05 [0.97-1.14]	0.84 [0.62-1.13]	0.86 [0.70-1.06]	1.05 [0.97-1.14]	1.02 [0.94-1.11]	1.02 [0.94-1.11]	0.90 [0.66-1.22]	0.93 [0.74-1.16]	1.04 [0.96-1.13]	1,777	1,934	104	274	1,727
O41	1.04 [0.97-1.12]	1.07 [1.00-1.14]		0.92 [0.77-1.10]	1.03 [0.96-1.12]	1.03 [0.96-1.11]	1.05 [0.98-1.13]		1.00 [0.82-1.21]	1.03 [0.95-1.11]	2,213	2,433	NA	359	1,866
O42	0.96 [0.93-0.99]	0.98 [0.95-1.01]	0.77 [0.65-0.91]	0.92 [0.85-1.00]	0.99 [0.96-1.02]	0.98 [0.94-1.01]	0.99 [0.96-1.02]	0.85 [0.71-1.01]	0.95 [0.87-1.03]	1.00 [0.97-1.04]	11,697	12,869	345	1,995	9,405
O43	0.95 [0.88-1.04]	0.97 [0.90-1.06]		0.89 [0.71-1.11]	0.98 [0.90-1.07]	0.95 [0.87-1.04]	0.98 [0.90-1.06]		0.97 [0.76-1.23]	0.98 [0.90-1.07]	1,629	1,778	NA	250	1,426
O44	0.94 [0.85-1.04]	0.94 [0.85-1.04]		0.80 [0.59-1.10]	0.97 [0.87-1.07]	0.92 [0.83-1.02]	0.91 [0.83-1.01]		0.80 [0.58-1.12]	0.96 [0.86-1.06]	1,135	1,198	NA	115	1,029
O45	0.93 [0.79-1.08]	0.93 [0.81-1.09]			0.98 [0.84-1.15]	0.90 [0.76-1.05]	0.93 [0.79-1.08]			1.00 [0.85-1.17]	468	524	NA	NA	437
O46	1.04 [0.99-1.09]	1.07 [1.02-1.11]	0.97 [0.79-1.20]	0.90 [0.80-1.01]	1.02 [0.97-1.06]	1.02 [0.98-1.07]	1.03 [0.99-1.08]	1.01 [0.81-1.26]	0.91 [0.81-1.03]	1.01 [0.96-1.06]	5,711	6,317	225	943	4,948
O47	1.06 [1.00-1.12]	1.06 [1.01-1.12]	0.94 [0.69-1.28]	0.90 [0.78-1.04]	1.02 [0.97-1.08]	1.04 [0.98-1.10]	1.02 [0.97-1.08]	0.94 [0.68-1.31]	0.88 [0.76-1.03]	1.01 [0.95-1.07]	3,995	4,324	102	564	3,297
O48	0.97 [0.93-1.01]	0.95 [0.92-0.99]	0.75 [0.60-0.95]	0.93 [0.84-1.04]	0.96 [0.92-1.00]	0.99 [0.95-1.03]	0.98 [0.94-1.02]	0.84 [0.66-1.07]	0.95 [0.85-1.07]	0.96 [0.92-1.01]	6,866	7,465	173	1,051	5,232
O60	1.00 [0.95-1.04]	1.00 [0.96-1.04]	0.92 [0.77-1.09]	0.96 [0.87-1.08]	1.00 [0.96-1.05]	0.99 [0.94-1.03]	0.97 [0.93-1.02]	0.93 [0.77-1.11]	1.00 [0.89-1.12]	1.00 [0.95-1.05]	6,252	6,849	313	1,041	5,501
O61	1.04 [0.95-1.15]	1.05 [0.96-1.16]		1.12 [0.86-1.45]	1.05 [0.94-1.17]	1.01 [0.91-1.12]	1.03 [0.93-1.13]		1.08 [0.81-1.43]	1.00 [0.90-1.12]	1,157	1,260	NA	195	898
O62	0.98 [0.93-1.03]	0.98 [0.93-1.02]	0.81 [0.64-1.02]	0.88 [0.78-0.99]	1.00 [0.95-1.05]	0.99 [0.94-1.04]	0.99 [0.94-1.04]	0.86 [0.67-1.10]	0.91 [0.80-1.04]	1.00 [0.95-1.06]	4,615	5,070	175	805	3,840
O63	0.94 [0.91-0.97]	0.95 [0.92-0.98]	0.84 [0.70-1.00]	0.93 [0.85-1.01]	0.98 [0.95-1.02]	0.95 [0.91-0.98]	0.96 [0.93-0.99]	0.93 [0.77-1.12]	0.92 [0.84-1.01]	0.98 [0.94-1.02]	9,989	11,007	303	1,674	6,834
O64	0.95 [0.88-1.02]	0.95 [0.88-1.02]		0.90 [0.71-1.13]	0.97 [0.89-1.06]	0.95 [0.87-1.02]	0.97 [0.90-1.04]		0.84 [0.66-1.08]	0.98 [0.90-1.07]	1,892	2,061	NA	227	1,397
O65	0.78 [0.58-1.04]	0.85 [0.64-1.12]				0.79 [0.59-1.07]	0.89 [0.67-1.18]				123	135	NA	NA	NA
O66	0.93 [0.87-1.01]	0.95 [0.88-1.02]		0.79 [0.64-0.97]	0.95 [0.87-1.03]	0.94 [0.87-1.02]	0.97 [0.89-1.04]		0.80 [0.64-0.99]	0.95 [0.88-1.04]	1,929	2,082	NA	288	1,536
O67	0.97 [0.83-1.12]	0.98 [0.85-1.13]			1.00 [0.86-1.17]	0.98 [0.84-1.14]	1.01 [0.87-1.17]			0.99 [0.84-1.17]	524	546	NA	NA	429
O68	0.96 [0.94-0.98]	0.98 [0.96-1.01]	0.83 [0.74-0.94]	0.87 [0.82-0.92]	0.97 [0.94-0.99]	0.97 [0.95-0.99]	0.99 [0.97-1.02]	0.89 [0.79-1.01]	0.89 [0.84-0.94]	0.97 [0.94-1.00]	21,677	24,275	734	3,819	16,077
O69	0.97 [0.90-1.05]	1.00 [0.93-1.07]		0.88 [0.71-1.08]	0.99 [0.92-1.07]	0.98 [0.90-1.05]	1.01 [0.94-1.09]		0.91 [0.73-1.14]	1.00 [0.92-1.08]	1,991	2,174	NA	277	1,718
O70	0.94 [0.92-0.96]	0.96 [0.94-0.97]	0.83 [0.73-0.94]	0.87 [0.83-0.92]	0.94 [0.92-0.97]	0.96 [0.94-0.98]	0.98 [0.96-1.00]	0.95 [0.83-1.08]	0.91 [0.86-0.96]	0.96 [0.94-0.99]	29,008	32,532	669	5,391	21,905
O71	0.94 [0.87-1.02]	0.97 [0.90-1.04]		0.97 [0.80-1.19]	0.96 [0.88-1.05]	0.95 [0.88-1.03]	0.99 [0.92-1.07]		1.08 [0.87-1.34]	0.98 [0.89-1.07]	1,739	1,921	NA	305	1,447
O72	0.98 [0.95-1.00]	0.99 [0.96-1.02]	0.89 [0.79-1.00]	0.94 [0.87-1.00]	1.00 [0.97-1.03]	0.99 [0.96-1.02]	1.01 [0.98-1.03]	0.97 [0.85-1.10]	0.96 [0.89-1.03]	1.00 [0.97-1.03]	15,399	16,864	701	2,538	12,591
O73	0.99 [0.90-1.10]	0.98 [0.89-1.08]		0.99 [0.74-1.33]	1.02 [0.92-1.14]	1.00 [0.90-1.11]	0.98 [0.88-1.08]		0.94 [0.69-1.29]	1.02 [0.92-1.14]	1,084	1,137	NA	141	919
O74	1.15 [0.94-1.40]	1.06 [0.88-1.28]			1.05 [0.86-1.29]	1.08 [0.88-1.32]	1.01 [0.83-1.23]			1.03 [0.83-1.28]	304	325	NA	NA	257
O75	0.95 [0.91-0.99]	0.96 [0.92-0.99]	0.86 [0.71-1.04]	0.76 [0.68-0.84]	0.98 [0.94-1.02]	0.96 [0.93-1.00]	0.97 [0.94-1.01]	0.94 [0.77-1.14]	0.76 [0.68-0.85]	0.99 [0.95-1.04]	7,301	8,033	277	1,099	6,158
O80	0.98 [0.94-1.02]	0.98 [0.95-1.02]	0.89 [0.71-1.12]	0.81 [0.73-0.90]	0.94 [0.91-0.98]	1.01 [0.97-1.05]	1.01 [0.97-1.05]	0.96 [0.76-1.22]	0.86 [0.77-0.96]	0.99 [0.95-1.03]	7,943	8,617	197	1,153	7,142
O81	1.02 [0.75-1.38]	0.95 [0.70-1.29]				1.01 [0.74-1.39]	0.98 [0.71-1.35]				111	111	NA	NA	NA
O82	1.05 [0.96-1.16]	1.02 [0.93-1.11]		1.25 [0.88-1.76]	0.99 [0.90-1.10]	1.04 [0.95-1.15]	1.01 [0.92-1.11]		1.31 [0.91-1.90]	0.98 [0.89-1.09]	1,228	1,269	NA	106	1,051
O85	0.95 [0.83-1.09]	0.96 [0.84-1.09]		0.73 [0.54-1.00]	1.00 [0.88-1.15]	0.92 [0.80-1.07]	0.94 [0.82-1.08]		0.77 [0.56-1.07]	0.98 [0.85-1.14]	627	700	NA	122	572
O86	1.08 [1.01-1.16]	1.07 [1.00-1.14]	1.16 [0.88-1.51]	0.93 [0.78-1.11]	1.11 [1.03-1.19]	1.06 [0.98-1.14]	1.04 [0.97-1.11]	1.11 [0.84-1.48]	0.95 [0.79-1.15]	1.09 [1.01-1.17]	2,427	2,666	149	411	2,090
O87	1.16 [0.98-1.39]	1.24 [1.04-1.47]			1.17 [0.97-1.40]	1.18 [0.98-1.41]	1.26 [1.05-1.50]			1.17 [0.96-1.41]	377	390	NA	NA	331
O88	1.09 [0.81-1.47]	1.18 [0.89-1.57]			1.11 [0.83-1.48]	1.05 [0.77-1.43]	1.14 [0.85-1.53]			1.06 [0.79-1.42]	137	152	NA	NA	134
O89	1.08 [0.78-1.50]	1.27 [0.93-1.73]			1.13 [0.81-1.56]	1.04 [0.74-1.46]	1.23 [0.89-1.71]			1.10 [0.79-1.54]	115	126	NA	NA	101
O90	1.07 [1.00-1.14]	1.07 [1.00-1.13]	0.96 [0.74-1.24]	0.90 [0.77-1.06]	1.05 [0.99-1.12]	1.05 [0.98-1.12]	1.03 [0.97-1.10]	0.97 [0.74-1.27]	0.88 [0.74-1.05]	1.03 [0.96-1.10]	2,926	3,204	155	466	2,551
O91	0.84 [0.69-1.03]	0.93 [0.77-1.14]			0.89 [0.72-1.11]	0.82 [0.66-1.02]	0.93 [0.76-1.14]			0.86 [0.68-1.07]	264	293	NA	NA	220
O92	1.00 [0.85-1.17]	1.01 [0.87-1.18]			0.98 [0.82-1.16]	0.98 [0.82-1.16]	1.01 [0.86-1.19]			1.01 [0.84-1.21]	413	454	NA	NA	345
O94	1.21 [0.87-1.67]	1.13 [0.83-1.54]			1.18 [0.86-1.61]	1.12 [0.80-1.58]	1.02 [0.74-1.43]			1.14 [0.82-1.58]	117	122	NA	NA	113
O98	1.06 [0.95-1.17]	1.05 [0.95-1.16]		0.82 [0.64-1.06]	1.05 [0.95-1.17]	1.04 [0.93-1.16]	1.03 [0.92-1.14]		0.83 [0.63-1.08]	1.03 [0.93-1.15]	1,067	1,182	NA	191	988
O99	1.08 [1.05-1.10]	1.08 [1.05-1.11]	0.95 [0.85-1.07]	0.93 [0.88-0.99]	1.06 [1.03-1.09]	1.05 [1.02-1.07]	1.03 [1.00-1.05]	0.94 [0.84-1.07]	0.95 [0.89-1.02]	1.04 [1.01-1.07]	17,744	19,624	770	3,254	15,517
O01		0.91 [0.65-1.26]					0.89 [0.63-1.26]				NA	104	NA	NA	NA
O15		1.07 [0.77-1.49]					1.02 [0.72-1.43]				NA	104	NA	NA	NA
Congenital malformations, deformations and chromosomal abnormalities (Q00-Q99)															
Q02	0.97 [0.73-1.28]			0.96 [0.71-1.31]	0.90 [0.67-1.21]	0.93 [0.69-1.24]			0.96 [0.70-1.30]	0.88 [0.65-1.19]	151	NA	NA	135	146
Q04	1.03 [0.80-1.32]	1.13 [0.82-1.55]			1.12 [0.87-1.44]	0.96 [0.74-1.25]	1.02 [0.74-1.43]			1.11 [0.86-1.44]	196	123	NA	NA	190
Q05	1.13 [0.91-1.39]	1.19 [0.97-1.46]	1.08 [0.84-1.37]		1.16 [0.94-1.43]	1.07 [0.87-1.33]	1.11 [0.90-1.37]	0.99 [0.77-1.26]		1.07 [0.86-1.33]	271	275	181	NA	239
Q07	1.04 [0.84-1.30]	1.15 [0.92-1.45]			1.07 [0.87-1.31]	0.97 [0.78-1.21]	0.99 [0.78-1.26]			0.99 [0.80-1.22]	265	232	NA	NA	267
Q10	1.30 [1.02-1.66]			1.25 [0.94-1.67]	1.20 [0.94-1.53]	1.31 [1.02-1.66]			1.25 [0.94-1.68]	1.19 [0.93-1.52]	210	NA	NA	152	199
Q12	1.29 [0.99-1.68]	1.15 [0.84-1.57]			1.21 [0.92-1.61]	1.27 [0.97-1.67]	1.09 [0.79-1.51]			1.18 [0.89-1.58]	180	124	NA	NA	150
Q17	0.97 [0.77-1.22]			0.94 [0.74-1.20]	1.08 [0.83-1.39]	0.95 [0.75-1.19]			0.93 [0.73-1.18]	1.07 [0.82-1.39]	240	NA	NA	222	195
Q18	1.13 [0.93-1.38]	1.18 [0.91-1.52]	1.19 [0.86-1.67]	1.18 [0.86-1.62]	1.10 [0.88-1.37]	1.10 [0.89-1.35]	1.08 [0.83-1.40]	1.18 [0.85-1.66]	1.19 [0.86-1.65]	1.09 [0.87-1.36]	296	188	101	131	244
Q21	1.10 [1.00-1.21]	1.12 [1.01-1.24]	1.16 [1.03-1.30]	0.86 [0.68-1.08]	1.08 [0.98-1.19]	1.05 [0.95-1.16]	1.06 [0.95-1.18]	1.09 [0.96-1.23]	0.85 [0.67-1.07]	1.04 [0.94-1.15]	1,219	1,049	814	223	1,111
Q22	0.99 [0.71-1.38]					1.00 [0.71-1.40]					105	NA	NA	NA	NA
Q23	1.04 [0.89-1.20]	1.04 [0.90-1.22]	0.99 [0.83-1.17]		0.91 [0.79-1.06]	1.01 [0.87-1.18]	1.01 [0.87-1.18]	0.97 [0.82-1.15]		0.89 [0.77-1.04]	523	476	384	NA	462
Q24	1.09 [0.92-1.29]	1.21 [1.01-1.45]	1.16 [0.94-1.43]		1.11 [0.94-1.31]	1.02 [0.85-1.21]	1.11 [0.92-1.33]	1.03 [0.82-1.28]		1.06 [0.89-1.26]	413	376	250	NA	398
Q25	0.96 [0.80-1.16]	1.07 [0.87-1.33]	1.20 [0.93-1.55]	0.79 [0.58-1.06]	0.94 [0.78-1.13]	0.93 [0.77-1.12]	1.01 [0.81-1.25]	1.10 [0.85-1.43]	0.71 [0.51-0.97]	0.91 [0.75-1.10]	345	252	173	125	328
Q27	1.03 [0.85-1.25]	1.09 [0.88-1.34]	1.07 [0.84-1.36]		0.91 [0.75-1.11]	0.99 [0.81-1.20]	1.03 [0.83-1.27]	0.99 [0.77-1.27]		0.90 [0.74-1.09]	314	258	192	NA	303
Q28	1.27 [0.99-1.63]	1.36 [1.06-1.74]	1.33 [1.01-1.75]		1.27 [0.99-1.62]	1.24 [0.96-1.59]	1.29 [1.00-1.67]	1.28 [0.96-1.69]		1.24 [0.96-1.60]	198	197	154	NA	181

Supplementary Table 4: Hazard ratios and events from all cohorts (excluding non-consulters)

	Hazard ratio (99% confidence interval)										Events (in exposed)				
	crude					adjusted									
Outcome	any age	18+	40+	<18	hosp.	any age	18+	40+	<18	hosp.	any age	18+	40+	<18	hosp.
Q31	0.96 [0.71-1.31]				0.84 [0.62-1.14]	0.91 [0.67-1.24]				0.80 [0.59-1.09]	115	NA	NA	NA	116
Q35	1.11 [0.78-1.58]					1.07 [0.74-1.53]					101	NA	NA	NA	NA
Q38	1.12 [0.99-1.26]	1.11 [0.95-1.30]	1.11 [0.95-1.30]	1.13 [0.95-1.35]	1.11 [0.98-1.25]	1.08 [0.96-1.22]	1.05 [0.90-1.23]	1.04 [0.88-1.23]	1.12 [0.93-1.33]	1.07 [0.95-1.22]	889	492	454	402	802
Q39	1.21 [0.98-1.49]	1.23 [1.00-1.51]	1.23 [1.00-1.52]		1.00 [0.81-1.23]	1.14 [0.92-1.41]	1.15 [0.93-1.42]	1.15 [0.93-1.43]		0.92 [0.74-1.14]	269	267	254	NA	246
Q40	1.30 [1.00-1.69]	1.22 [0.94-1.60]	1.13 [0.86-1.49]		1.24 [0.96-1.61]	1.19 [0.90-1.56]	1.07 [0.81-1.41]	1.02 [0.77-1.35]		1.17 [0.90-1.53]	160	160	143	NA	161
Q43	1.19 [1.03-1.38]	1.15 [0.99-1.34]	1.20 [1.02-1.42]		1.28 [1.11-1.49]	1.13 [0.98-1.32]	1.08 [0.92-1.25]	1.13 [0.95-1.34]		1.22 [1.05-1.42]	550	501	390	NA	522
Q44	0.95 [0.74-1.21]	0.95 [0.75-1.21]	1.00 [0.76-1.31]		0.99 [0.78-1.26]	0.92 [0.72-1.18]	0.89 [0.69-1.14]	0.93 [0.71-1.23]		0.96 [0.76-1.23]	184	184	148	NA	186
Q50	1.22 [1.03-1.46]	1.26 [1.07-1.49]	1.03 [0.78-1.36]		1.12 [0.94-1.34]	1.15 [0.96-1.38]	1.16 [0.97-1.38]	0.94 [0.70-1.26]		1.10 [0.92-1.32]	406	425	139	NA	357
Q51	0.98 [0.82-1.17]	0.97 [0.82-1.15]	0.99 [0.77-1.27]		1.00 [0.84-1.20]	0.94 [0.78-1.12]	0.92 [0.77-1.10]	0.90 [0.69-1.17]		0.98 [0.81-1.18]	366	387	169	NA	328
Q52	1.14 [0.86-1.51]	1.12 [0.83-1.53]			1.06 [0.79-1.42]	1.06 [0.79-1.42]	1.11 [0.81-1.53]			1.05 [0.77-1.42]	155	119	NA	NA	129
Q53	0.87 [0.77-0.99]			0.91 [0.79-1.04]	0.85 [0.74-0.98]	0.87 [0.76-0.99]			0.90 [0.79-1.03]	0.84 [0.73-0.97]	711	NA	NA	680	608
Q54	1.11 [0.93-1.32]	1.14 [0.90-1.44]	1.16 [0.91-1.47]	1.10 [0.84-1.44]	0.96 [0.79-1.15]	1.11 [0.93-1.33]	1.10 [0.86-1.39]	1.13 [0.89-1.44]	1.12 [0.86-1.47]	0.97 [0.80-1.17]	363	207	202	168	323
Q55	1.14 [0.97-1.33]			1.16 [0.98-1.37]	1.15 [0.97-1.37]	1.13 [0.96-1.33]			1.15 [0.97-1.36]	1.13 [0.95-1.35]	543	NA	NA	478	453
Q60	1.03 [0.86-1.23]	1.08 [0.91-1.29]	1.02 [0.84-1.24]		1.10 [0.93-1.31]	0.99 [0.83-1.19]	1.01 [0.85-1.22]	0.96 [0.79-1.17]		1.10 [0.92-1.31]	373	372	304	NA	379
Q61	1.00 [0.90-1.11]	1.01 [0.91-1.12]	1.05 [0.94-1.17]		1.01 [0.91-1.12]	0.97 [0.88-1.08]	0.96 [0.86-1.06]	1.01 [0.90-1.13]		1.00 [0.90-1.11]	1,056	1,039	915	NA	987
Q62	0.92 [0.69-1.22]				0.93 [0.70-1.24]	0.91 [0.68-1.22]				0.94 [0.70-1.25]	143	NA	NA	NA	139
Q63	1.00 [0.88-1.14]	1.05 [0.92-1.20]	0.98 [0.83-1.14]	0.93 [0.68-1.27]	0.97 [0.85-1.10]	0.94 [0.82-1.07]	0.98 [0.85-1.12]	0.90 [0.76-1.06]	0.90 [0.65-1.24]	0.92 [0.80-1.05]	677	628	432	132	642
Q64	1.12 [0.81-1.55]				1.07 [0.76-1.49]	1.07 [0.77-1.49]				1.07 [0.76-1.50]	117	NA	NA	NA	105
Q65	1.13 [0.96-1.34]	1.05 [0.87-1.27]	1.17 [0.90-1.52]	1.00 [0.76-1.31]	1.07 [0.91-1.26]	1.07 [0.90-1.26]	0.98 [0.81-1.19]	1.09 [0.83-1.43]	0.96 [0.73-1.28]	1.04 [0.88-1.23]	445	330	163	161	420
Q66	1.25 [1.06-1.48]	1.23 [1.02-1.49]	1.41 [1.14-1.76]	1.06 [0.79-1.44]	1.23 [1.03-1.45]	1.15 [0.97-1.37]	1.13 [0.93-1.38]	1.31 [1.04-1.64]	1.01 [0.75-1.37]	1.16 [0.98-1.38]	464	336	251	153	432
Q67	1.22 [1.00-1.49]	1.14 [0.87-1.49]	1.35 [0.98-1.84]	1.01 [0.76-1.34]	1.13 [0.92-1.39]	1.19 [0.97-1.46]	1.06 [0.81-1.40]	1.30 [0.95-1.79]	0.97 [0.72-1.29]	1.12 [0.91-1.38]	315	169	121	157	293
Q68	1.35 [0.96-1.90]					1.25 [0.88-1.78]					108	NA	NA	NA	NA
Q74	1.13 [0.92-1.39]	1.25 [0.97-1.61]	1.26 [0.90-1.75]	1.13 [0.83-1.54]	1.08 [0.88-1.33]	1.08 [0.88-1.34]	1.15 [0.88-1.50]	1.18 [0.83-1.66]	1.10 [0.80-1.51]	1.06 [0.85-1.31]	295	193	109	138	262
Q75	0.92 [0.69-1.21]			0.82 [0.61-1.11]	0.85 [0.64-1.13]	0.89 [0.67-1.19]			0.81 [0.60-1.09]	0.84 [0.63-1.12]	144	NA	NA	129	138
Q76	1.19 [0.99-1.42]	1.14 [0.94-1.38]	1.12 [0.90-1.40]		1.14 [0.95-1.37]	1.08 [0.90-1.31]	1.00 [0.82-1.22]	0.97 [0.77-1.22]		1.07 [0.89-1.30]	360	315	223	NA	328
Q78	1.16 [0.92-1.47]	1.25 [0.95-1.63]	1.19 [0.89-1.58]		1.18 [0.93-1.50]	1.09 [0.85-1.39]	1.11 [0.84-1.47]	1.06 [0.79-1.42]		1.12 [0.88-1.43]	211	165	137	NA	198
Q79	1.26 [1.07-1.48]	1.23 [1.05-1.46]	1.04 [0.81-1.32]	0.97 [0.70-1.35]	1.14 [0.97-1.34]	1.05 [0.88-1.25]	0.97 [0.81-1.16]	0.83 [0.64-1.08]	0.90 [0.64-1.27]	0.98 [0.83-1.16]	481	470	181	121	453
Q82	1.45 [1.20-1.75]	1.64 [1.30-2.07]	1.65 [1.28-2.13]	1.06 [0.77-1.46]	1.39 [1.14-1.69]	1.36 [1.12-1.65]	1.49 [1.17-1.90]	1.51 [1.16-1.96]	1.03 [0.74-1.43]	1.32 [1.09-1.61]	358	242	196	120	314
Q83	0.96 [0.74-1.25]	0.99 [0.78-1.26]			0.98 [0.74-1.29]	0.93 [0.71-1.21]	0.95 [0.74-1.22]			0.96 [0.72-1.27]	165	190	NA	NA	140
Q85	0.91 [0.73-1.12]	0.99 [0.79-1.25]	1.06 [0.81-1.38]		0.98 [0.80-1.21]	0.88 [0.70-1.09]	0.93 [0.74-1.18]	1.01 [0.77-1.33]		0.95 [0.77-1.18]	248	207	150	NA	243
Q87	1.16 [0.96-1.40]	1.31 [1.05-1.64]	1.36 [1.03-1.80]	1.07 [0.79-1.44]	1.12 [0.93-1.35]	1.07 [0.88-1.30]	1.19 [0.95-1.51]	1.25 [0.93-1.67]	1.05 [0.76-1.43]	1.08 [0.89-1.31]	363	246	152	139	332
Q89	1.06 [0.87-1.29]	1.18 [0.93-1.49]	0.98 [0.73-1.31]	0.92 [0.66-1.27]	1.09 [0.89-1.33]	1.02 [0.83-1.25]	1.08 [0.85-1.38]	0.94 [0.70-1.26]	0.89 [0.64-1.25]	1.06 [0.86-1.31]	312	203	127	124	275
Q90	3.11 [2.52-3.84]	3.08 [2.48-3.82]	3.71 [2.89-4.75]		2.01 [1.54-2.63]	3.04 [2.45-3.77]	3.34 [2.66-4.19]	3.78 [2.93-4.87]		1.93 [1.46-2.55]	412	385	312	NA	190
Q93	1.43 [1.07-1.91]			1.20 [0.85-1.68]	1.44 [1.07-1.93]	1.31 [0.97-1.76]			1.13 [0.80-1.60]	1.40 [1.03-1.89]	161	NA	NA	114	159
Q99	1.02 [0.78-1.32]				1.14 [0.88-1.49]	0.94 [0.72-1.24]				1.10 [0.84-1.45]	173	NA	NA	NA	177
Symptoms, signs and abnormal clinical and laboratory findings, not elsewhere classified (R00-R99)															
R00	1.11 [1.09-1.13]	1.10 [1.08-1.13]	1.11 [1.09-1.14]	0.99 [0.91-1.08]	1.09 [1.07-1.11]	1.06 [1.04-1.08]	1.03 [1.01-1.06]	1.05 [1.02-1.07]	0.98 [0.90-1.06]	1.06 [1.04-1.08]	30,681	30,445	25,366	2,001	29,090
R01	1.14 [1.08-1.20]	1.15 [1.09-1.21]	1.17 [1.10-1.24]	1.24 [1.07-1.44]	1.14 [1.08-1.20]	1.09 [1.04-1.15]	1.09 [1.03-1.15]	1.11 [1.04-1.17]	1.18 [1.02-1.37]	1.11 [1.05-1.17]	4,686	4,248	3,696	640	4,483
R02	1.14 [1.09-1.20]	1.14 [1.09-1.20]	1.16 [1.10-1.21]		1.13 [1.08-1.19]	1.12 [1.07-1.18]	1.08 [1.02-1.13]	1.08 [1.03-1.14]		1.13 [1.08-1.19]	4,934	4,916	4,712	NA	4,767
R03	1.12 [1.08-1.15]	1.10 [1.06-1.14]	1.13 [1.09-1.17]	1.06 [0.91-1.23]	1.08 [1.05-1.12]	1.08 [1.05-1.12]	1.05 [1.02-1.09]	1.08 [1.04-1.12]	1.02 [0.87-1.20]	1.06 [1.03-1.10]	10,725	10,827	8,673	547	10,140
R04	1.23 [1.19-1.27]	1.23 [1.20-1.27]	1.24 [1.20-1.28]	1.13 [0.98-1.30]	1.20 [1.16-1.23]	1.17 [1.13-1.20]	1.15 [1.11-1.19]	1.15 [1.11-1.19]	1.12 [0.97-1.29]	1.15 [1.12-1.19]	12,489	12,004	11,284	703	11,680
R05	1.24 [1.20-1.28]	1.22 [1.18-1.27]	1.24 [1.19-1.28]	1.34 [1.23-1.45]	1.21 [1.17-1.25]	1.16 [1.12-1.19]	1.11 [1.07-1.15]	1.12 [1.08-1.16]	1.28 [1.18-1.40]	1.14 [1.11-1.18]	12,434	10,823	9,545	1,997	11,902
R06	1.32 [1.30-1.34]	1.21 [1.19-1.23]	1.21 [1.19-1.24]	1.77 [1.71-1.84]	1.28 [1.26-1.30]	1.25 [1.23-1.27]	1.10 [1.08-1.13]	1.11 [1.08-1.13]	1.73 [1.66-1.80]	1.22 [1.20-1.24]	40,702	30,863	26,850	10,957	38,707
R07	1.16 [1.15-1.18]	1.17 [1.15-1.19]	1.18 [1.16-1.20]	0.92 [0.85-1.00]	1.13 [1.11-1.15]	1.09 [1.08-1.11]	1.08 [1.06-1.09]	1.08 [1.07-1.10]	0.92 [0.84-1.00]	1.08 [1.06-1.10]	52,023	52,322	44,229	1,976	47,918
R09	1.20 [1.14-1.26]	1.19 [1.13-1.25]	1.19 [1.13-1.26]	1.08 [0.86-1.36]	1.15 [1.09-1.20]	1.13 [1.07-1.19]	1.10 [1.04-1.15]	1.10 [1.04-1.16]	1.09 [0.85-1.38]	1.11 [1.05-1.16]	4,818	4,733	4,154	248	4,634
R10	1.16 [1.15-1.18]	1.16 [1.15-1.18]	1.19 [1.17-1.21]	1.03 [0.99-1.08]	1.13 [1.12-1.15]	1.10 [1.08-1.11]	1.07 [1.05-1.08]	1.10 [1.08-1.12]	1.01 [0.97-1.05]	1.08 [1.07-1.10]	64,379	63,596	39,642	8,806	57,549
R11	1.15 [1.13-1.17]	1.15 [1.13-1.17]	1.15 [1.13-1.18]	1.07 [1.02-1.12]	1.11 [1.09-1.13]	1.09 [1.07-1.11]	1.06 [1.04-1.08]	1.08 [1.05-1.10]	1.05 [1.00-1.10]	1.08 [1.06-1.10]	38,199	35,420	28,135	5,415	35,888
R12	1.21 [1.14-1.29]	1.22 [1.15-1.30]	1.25 [1.16-1.34]	0.96 [0.69-1.35]	1.18 [1.11-1.26]	1.12 [1.04-1.19]	1.10 [1.03-1.18]	1.14 [1.06-1.22]	0.88 [0.61-1.25]	1.11 [1.03-1.18]	2,857	2,875	2,333	116	2,654
R13	1.20 [1.17-1.23]	1.20 [1.18-1.23]	1.20 [1.17-1.23]	1.14 [0.95-1.37]	1.17 [1.15-1.20]	1.13 [1.11-1.16]	1.11 [1.09-1.14]	1.12 [1.09-1.14]	1.05 [0.87-1.28]	1.13 [1.10-1.16]	22,523	22,408	21,197	423	21,803
R14	1.18 [1.13-1.24]	1.18 [1.12-1.24]	1.19 [1.13-1.26]	0.99 [0.80-1.23]	1.17 [1.12-1.23]	1.10 [1.05-1.16]	1.07 [1.01-1.12]	1.09 [1.03-1.15]	0.97 [0.78-1.22]	1.11 [1.06-1.17]	4,885	4,849	3,924	275	4,641
R15	1.11 [1.07-1.15]	1.11 [1.07-1.15]	1.11 [1.07-1.15]	0.99 [0.71-1.36]	1.10 [1.06-1.14]	1.09 [1.05-1.13]	1.07 [1.03-1.11]	1.08 [1.04-1.12]	0.98 [0.70-1.37]	1.10 [1.06-1.14]	9,490	9,401	9,092	131	9,238
R16	1.30 [1.22-1.38]	1.35 [1.27-1.43]	1.32 [1.24-1.41]	0.95 [0.74-1.21]	1.22 [1.15-1.29]	1.23 [1.16-1.31]	1.24 [1.17-1.32]	1.22 [1.15-1.31]	0.91 [0.71-1.16]	1.19 [1.12-1.26]	3,350	3,290	2,897	211	3,195
R17	1.17 [1.09-1.25]	1.19 [1.11-1.27]	1.18 [1.10-1.26]		1.17 [1.09-1.25]	1.15 [1.07-1.23]	1.14 [1.07-1.22]	1.14 [1.06-1.22]		1.17 [1.09-1.25]	2,578	2,562	2,356	NA	2,370
R18	1.16 [1.12-1.21]	1.17 [1.12-1.22]	1.17 [1.13-1.23]	0.85 [0.61-1.19]	1.15 [1.11-1.20]	1.14 [1.10-1.19]	1.13 [1.08-1.18]	1.14 [1.09-1.18]	0.87 [0.62-1.22]	1.16 [1.11-1.21]	6,807	6,790	6,356	121	6,439
R19	1.22 [1.19-1.24]	1.21 [1.19-1.24]	1.20 [1.18-1.23]	1.02 [0.91-1.14]	1.19 [1.16-1.21]	1.14 [1.12-1.17]	1.12 [1.09-1.14]	1.12 [1.09-1.14]	0.99 [0.88-1.11]	1.13 [1.11-1.16]	27,081	27,030	23,324	1,097	25,303
R20	1.16 [1.11-1.21]	1.16 [1.11-1.20]	1.14 [1.09-1.20]	1.03 [0.87-1.23]	1.12 [1.07-1.17]	1.07 [1.03-1.12]	1.04 [1.00-1.09]	1.03 [0.98-1.08]	1.03 [0.86-1.24]	1.06 [1.02-1.11]	6,862	6,943	5,153	450	6,589
R21	1.53 [1.48-1.58]	1.60 [1.54-1.66]	1.70 [1.63-1.77]	1.16 [1.08-1.25]	1.46 [1.41-1.51]	1.47 [1.41-1.52]	1.50 [1.44-1.56]	1.59 [1.53-1.66]	1.14 [1.06-1.23]	1.42 [1.37-1.47]	10,581	8,610	7,270	2,391	9,807
R22	1.29 [1.23-1.35]	1.25 [1.19-1.31]	1.29 [1.23-1.37]	1.43 [1.23-1.67]	1.25 [1.19-1.31]	1.22 [1.16-1.28]	1.15 [1.09-1.21]	1.20 [1.14-1.27]	1.40 [1.20-1.63]	1.19 [1.13-1.25]	5,293	4,889	4,066	625	4,884
R23	1.20 [1.14-1.27]	1.21 [1.14-1.30]	1.23 [1.15-1.31]	1.17 [1.05-1.32]	1.20 [1.14-1.28]	1.16 [1.09-1.23]	1.14 [1.06-1.22]	1.15 [1.07-1.23]	1.13 [1.00-1.27]	1.17 [1.10-1.24]	3,748	2,862	2,563	996	3,607
R25	1.14 [1.08-1.20]	1.16 [1.10-1.22]	1.15 [1.09-1.22]	1.01 [0.83-1.22]	1.12 [1.06-1.18]	1.07 [1.02-1.13]	1.06 [1.00-1.12]	1.06 [1.00-1.12]	0.98 [0.80-1.19]	1.08 [1.02-1.14]	4,131	3,892	3,457	341	4,004
R26	1.14 [1.12-1.16]	1.13 [1.11-1.15]	1.13 [1.11-1.16]	1.01 [0.88-1.16]	1.12 [1.10-1.14]	1.11 [1.09-1.13]	1.08 [1.06-1.10]	1.08 [1.06-1.10]	1.00 [0.86-1.15]	1.11 [1.08-1.13]	36,004	35,529	35,015	644	35,245
R27	1.09 [0.98-1.21]	1.09 [0.98-1.21]	1.10 [0.98-1.23]	1.01 [0.74-1.39]	1.05 [0.94-1.16]	1.05 [0.95-1.17]	1.02 [0.92-1.14]	1.03 [0.92-1.16]	0.99 [0.71-1.37]	1.03 [0.93-1.15]	1,100	985	894	138	1,029
R29	1.12 [1.10-1.14]	1.12 [1.10-1.14]	1.12 [1.11-1.14]	0.86 [0.72-1.03]	1.11 [1.09-1.13]	1.09 [1.07-1.11]	1.06 [1.05-1.08]	1.07 [1.05-1.09]	0.86 [0.72-1.03]	1.10 [1.08-1.11]	47,875	47,735	46,731	428	47,120
R30	1.26 [1.19-1.34]	1.20 [1.13-1.28]	1.23 [1.14-1.31]	1.09 [0.90-1.32]	1.20 [1.13-1.27]	1.18 [1.11-1.25]	1.09 [1.02-1.16]	1.10 [1.02-1.18]	1.08 [0.89-1.32]	1.14 [1.07-1.21]	3,306	3,194	2,419	366	3,154
R31	1.10 [1.07-1.12]	1.10 [1.07-1.12]	1.09 [1.06-1.11]	1.09 [0.93-1.28]	1.06 [1.03-1.08]	1.07 [1.05-1.10]	1.06 [1.03-1.08]	1.05 [1.02-1.07]	1.07 [0.90-1.25]	1.04 [1.02-1.07]	22,024	21,931	20,276	540	19,990
R32	1.10 [1.08-1.13]	1.10 [1.07-1.13]	1.09 [1.06-1.12]	1.10 [0.91-1.33]	1.08 [1.05-1.11]	1.08 [1.05-1.11]	1.05 [1.03-1.08]	1.05 [1.02-1.08]	1.09 [0.89-1.33]	1.07 [1.05-1.10]	17,893	17,683	16,966	401	17,449
R33	1.10 [1.08-1.12]	1.09 [1.07-1.11]	1.10 [1.07-1.12]	1.00 [0.85-1.17]	1.09 [1.07-1.11]	1.07 [1.05-1.10]	1.05 [1.03-1.07]	1.06 [1.04-1.08]	0.97 [0.82-1.15]	1.08 [1.06-1.10]	33,293	33,293	31,650	526	31,859
R34	1.24 [1.11-1.39]	1.23 [1.09-1.38]	1.22 [1.09-1.38]		1.25 [1.11-1.40]	1.19 [1.06-1.33]	1.14 [1.01-1.29]	1.14 [1.01-1.29]		1.23 [1.10-1.38]	932	910	864	NA	886
R35	1.25 [1.20-1.31]	1.22 [1.17-1.28]	1.21 [1.16-1.28]	1.01 [0.81-1.27]	1.18 [1.13-1.24]	1.18 [1.13-1.24]	1.13 [1.08-1.19]	1.13 [1.08-1.19]	0.99 [0.78-1.25]	1.13 [1.08-1.18]	5,488	5,424	4,643	250	5,141
R36	1.30 [0.94-1.80]	1.42 [1.01-1.99]	1.41 [1.00-1.99]		1.34 [0.97-1.85]	1.31 [0.94-1.83]	1.36 [0.97-1.92]	1.26 [0.88-1.80]		1.32 [0.95-1.83]	121	114	109	NA	114
R39	1.20 [1.15-1.26]	1.21 [1.16-1.26]	1.20 [1.15-1.26]	1.02 [0.81-1.27]	1.15 [1.10-1.20]	1.13 [1.08-1.18]	1.12 [1.07-1.17]	1.12 [1.06-1.17]	0.98 [0.78-1.24]	1.10 [1.05-1.15]	6,208	6,194	5,460	255	5,844
R40	1.13 [1.07-1.18]	1.13 [1.08-1.19]	1.14 [1.08-1.20]	1.00 [0.81-1.24]	1.11 [1.06-1.17]	1.09 [1.04-1.14]	1.06 [1.01-1.12]	1.07 [1.02-1.13]	1.00 [0.80-1.25]	1.09 [1.04-1.15]	5,149	4,996	4,648	281	5,070
R41	1.12 [1.10-1.14]	1.12 [1.10-1.15]	1.11 [1.09-1.13]	0.94 [0.78-1.13]	1.11 [1.09-1.13]	1.10 [1.08-1.12]	1.08 [1.06-1.10]	1.07 [1.05-1.09]	0.94 [0.78-1.15]	1.10 [1.08-1.13]	32,986	32,816	32,286	373	31,660
R42	1.14 [1.11-1.17]	1.14 [1.11-1.17]	1.13 [1.10-1.16]	0.95 [0.83-1.09]	1.11 [1.08-1.14]	1.08 [1.06-1.11]	1.06 [1.03-1.08]	1.06 [1.03-1.09]	0.95 [0.82-1.10]	1.07 [1.04-1.10]	17,602	17,551	15,276	708	16,910
R43	1.11 [0.88-1.39]	1.05 [0.83-1.32]	1.19 [0.92-1.54]		1.13 [0.89-1.42]	1.03 [0.81-1.31]	0.96 [0.75-1.22]	1.06 [0.81-1.38]		1.08 [0.85-1.37]	220	215	177	NA	208
R44	1.14 [1.09-1.21]	1.13 [1.08-1.20]	1.15 [1.09-1.22]	0.96 [0.75-1.22]	1.10 [1.05-1.16]	1.11 [1.05-1.17]	1.07 [1.01-1.13]	1.09 [1.03-1.15]	0.97 [0.76-1.25]	1.09 [1.03-1.15]	4,448	4,331	3,971	219	4,241
R45	1.13 [1.09-1.18]	1.12 [1.08-1.16]	1.15 [1.10-1.20]	0.96 [0.86-1.08]	1.10 [1.06-1.15]	1.09 [1.05-1.13]	1.03 [0.99-1.07]	1.07 [1.02-1.11]	1.00 [0.88-1.13]	1.09 [1.05-1.14]	8,788	8,371	6,945	1,012	8,519
R46	1.14 [1.06-1.21]	1.10 [1.03-1.18]	1.09 [1.02-1.17]	1.07 [0.81-1.41]	1.13 [1.05-1.20]	1.13 [1.05-1.20]	1.06 [0.99-1.14]	1.06 [0.98-1.13]	1.03 [0.77-1.37]	1.15 [1.07-1.23]	2,727	2,567	2,411	189	2,636
R47	1.09 [1.05-1.13]	1.09 [1.05-1.13]	1.08 [1.04-1.12]	1.11 [0.85-1.45]	1.07 [1.04-1.11]	1.07 [1.03-1.11]	1.04 [1.00-1.08]	1.03 [0.99-1.07]	1.15 [0.87-1.52]	1.07 [1.03-1.11]	8,917	8,804	8,409	185	8,525
R48	1.28 [1.03-1.59]	1.18 [0.94-1.47]	1.28 [0.95-1.71]		1.29 [1.04-1.60]	1.15 [0.92-1.45]	1.05 [0.83-1.32]	1.17 [0.86-1.58]		1.22 [0.98-1.53]	281	256	138	NA	260
R49	1.19 [1.10-1.30]	1.23 [1.13-1.34]	1.18 [1.08-1.29]		1.19 [1.10-1.30]	1.11 [1.01-1.20]	1.09 [1.00-1.19]	1.05 [0.96-1.15]		1.11 [1.02-1.21]	1,663	1,626	1,483	NA	1,584
R50	1.18 [1.14-1.21]	1.19 [1.15-1.23]	1.19 [1.15-1.23]	1.13 [1.07-1.20]	1.15 [1.12-1.19]	1.13 [1.10-1.16]	1.12 [1.08-1.15]	1.13 [1.09-1.17]	1.11 [1.04-1.18]	1.13 [1.09-1.16]	14,839	11,749	9,530	3,766	13,835
R51	1.17 [1.14-1.20]	1.17 [1.14-1.20]	1.19 [1.16-1.22]	1.06 [0.99-1.13]	1.13 [1.10-1.16]	1.09 [1.06-1.12]	1.05 [1.03-1.08]	1.08 [1.05-1.11]	1.04 [0.97-1.12]	1.08 [1.05-1.10]	22,028	21,466	14,228	2,813	20,771
R52	1.25 [1.19-1.32]	1.24 [1.17-1.30]	1.24 [1.17-1.31]	0.85 [0.64-1.13]	1.21 [1.15-1.27]	1.15 [1.09-1.21]	1.09 [1.03-1.15]	1.11 [1.04-1.17]	0.83 [0.62-1.13]	1.14 [1.08-1.20]	4,190	4,190	3,532	160	4,153
R53	1.13 [1.10-1.16]	1.13 [1.10-1.16]	1.13 [1.10-1.16]	1.08 [0.95-1.21]	1.11 [1.08-1.14]	1.09 [1.06-1.12]	1.06 [1.03-1.09]	1.06 [1.03-1.09]	1.05 [0.93-1.19]	1.09 [1.06-1.12]	16,722	16,192	14,781	916	16,261
R54	1.11 [1.09-1.14]	1.12 [1.09-1.14]	1.11 [1.09-1.14]		1.10 [1.08-1.13]	1.10 [1.07-1.12]	1.08 [1.05-1.10]	1.07 [1.05-1.10]		1.10 [1.07-1.13]	22,174	22,166	22,113	NA	21,393
R55	1.12 [1.10-1.14]	1.12 [1.10-1.14]	1.13 [1.11-1.15]	0.93 [0.84-1.04]	1.11 [1.09-1.13]	1.09 [1.07-1.11]	1.07 [1.05-1.09]	1.08 [1.06-1.10]	0.91 [0.82-1.02]	1.09 [1.07-1.11]	31,010	30,710	28,043	1,171	29,017
R56	1.12 [1.09-1.16]	1.16 [1.12-1.21]	1.17 [1.12-1.21]	1.01 [0.95-1.08]	1.09 [1.05-1.12]	1.10 [1.06-1.13]	1.10 [1.06-1.14]	1.11 [1.06-1.16]	1.00 [0.93-1.07]	1.08 [1.05-1.12]	10,570	8,105	6,985	2,794	9,840
R57	1.10 [1.03-1.17]	1.11 [1.04-1.19]	1.11 [1.04-1.19]		1.12 [1.05-1.20]	1.08 [1.01-1.16]	1.07 [1.00-1.14]	1.06 [1.00-1.14]		1.13 [1.06-1.20]	2,785	2,763	2,680	NA	2,712
R58	1.29 [1.10-1.53]	1.22 [1.03-1.44]	1.22 [1.03-1.45]		1.17 [1.00-1.38]	1.24 [1.04-1.46]	1.14 [0.96-1.35]	1.15 [0.97-1.37]		1.16 [0.98-1.36]	450	442	419	NA	433
R59	1.26 [1.21-1.31]	1.25 [1.21-1.30]	1.26 [1.21-1.31]	1.14 [1.00-1.29]	1.22 [1.18-1.27]	1.24 [1.19-1.28]	1.20 [1.15-1.25]	1.21 [1.16-1.26]	1.13 [0.99-1.28]	1.21 [1.17-1.26]	8,395	7,815	6,886	863	7,786
R60	1.24 [1.21-1.28]	1.24 [1.20-1.27]	1.25 [1.21-1.28]	0.97 [0.71-1.33]	1.23 [1.19-1.26]	1.19 [1.16-1.22]	1.16 [1.13-1.19]	1.17 [1.14-1.20]	0.96 [0.69-1.33]	1.19 [1.16-1.22]	16,685	16,642	16,239	132	16,254
R61	1.25 [1.14-1.36]	1.25 [1.14-1.36]	1.18 [1.07-1.30]	1.16 [0.82-1.64]	1.19 [1.09-1.29]	1.15 [1.05-1.26]	1.14 [1.04-1.25]	1.07 [0.97-1.19]	1.06 [0.74-1.52]	1.13 [1.03-1.24]	1,558	1,567	1,177	116	1,457
R62	1.10 [0.99-1.21]	1.06 [0.86-1.29]	1.18 [0.91-1.52]	1.09 [0.97-1.21]	1.05 [0.95-1.17]	1.06 [0.96-1.18]	1.02 [0.83-1.25]	1.12 [0.86-1.45]	1.04 [0.93-1.17]	1.03 [0.93-1.14]	1,316	298	179	1,034	1,224
R63	1.16 [1.14-1.18]	1.15 [1.13-1.18]	1.16 [1.14-1.19]	1.03 [0.94-1.12]	1.15 [1.13-1.17]	1.12 [1.09-1.14]	1.08 [1.06-1.11]	1.10 [1.07-1.12]	0.99 [0.91-1.09]	1.12 [1.10-1.14]	30,767	29,783	27,511	1,713	29,257
R64	1.14 [1.01-1.29]	1.11 [0.99-1.26]	1.08 [0.96-1.22]		1.06 [0.94-1.20]	1.14 [1.01-1.29]	1.09 [0.96-1.24]	1.08 [0.95-1.22]		1.09 [0.96-1.23]	794	795	786	NA	767
R65	1.13 [1.05-1.23]	1.11 [1.02-1.20]	1.14 [1.05-1.23]		1.14 [1.06-1.23]	1.09 [1.01-1.18]	1.05 [0.97-1.14]	1.06 [0.98-1.15]		1.12 [1.03-1.21]	1,988	1,968	1,911	NA	1,966
R68	1.19 [1.12-1.27]	1.16 [1.09-1.24]	1.17 [1.09-1.25]	1.20 [0.93-1.55]	1.18 [1.11-1.26]	1.18 [1.11-1.26]	1.13 [1.05-1.20]	1.13 [1.05-1.21]	1.14 [0.88-1.49]	1.18 [1.11-1.26]	3,069	2,919	2,766	189	2,985
R69	1.15 [1.13-1.18]	1.14 [1.12-1.17]	1.15 [1.12-1.18]	1.01 [0.91-1.13]	1.13 [1.11-1.16]	1.12 [1.10-1.15]	1.09 [1.06-1.11]	1.10 [1.07-1.13]	1.00 [0.89-1.12]	1.11 [1.09-1.14]	24,225	23,904	20,373	1,101	21,557
R70	1.33 [1.07-1.65]	1.31 [1.04-1.63]	1.29 [1.03-1.61]		1.22 [0.97-1.54]	1.28 [1.03-1.60]	1.22 [0.97-1.54]	1.21 [0.96-1.53]		1.18 [0.93-1.49]	239	236	221	NA	218
R71	1.10 [0.85-1.42]	1.01 [0.78-1.31]	1.13 [0.86-1.49]		0.99 [0.77-1.29]	1.08 [0.83-1.41]	0.98 [0.75-1.28]	1.10 [0.83-1.45]		1.00 [0.77-1.31]	162	162	149	NA	152
R72	1.12 [1.01-1.26]	1.10 [0.99-1.23]	1.10 [0.98-1.24]		1.09 [0.98-1.22]	1.10 [0.98-1.23]	1.04 [0.93-1.17]	1.07 [0.94-1.20]		1.07 [0.96-1.20]	954	949	803	NA	912
R73	1.16 [1.10-1.23]	1.16 [1.10-1.23]	1.17 [1.11-1.24]	0.86 [0.60-1.23]	1.17 [1.10-1.23]	1.10 [1.04-1.16]	1.08 [1.02-1.14]	1.07 [1.01-1.14]	0.84 [0.58-1.22]	1.12 [1.06-1.19]	4,007	3,975	3,673	102	3,905
R74	1.12 [1.02-1.22]	1.08 [0.99-1.18]	1.12 [1.03-1.23]	1.15 [0.81-1.63]	1.08 [0.99-1.18]	1.07 [0.98-1.17]	1.00 [0.92-1.10]	1.05 [0.96-1.15]	1.09 [0.75-1.58]	1.06 [0.98-1.16]	1,650	1,608	1,431	111	1,580
R76	1.28 [1.09-1.51]	1.28 [1.09-1.50]	1.30 [1.08-1.56]		1.27 [1.08-1.50]	1.25 [1.06-1.47]	1.23 [1.04-1.45]	1.25 [1.04-1.50]		1.23 [1.04-1.45]	472	450	366	NA	439
R77	1.22 [1.10-1.36]	1.21 [1.09-1.34]	1.25 [1.12-1.40]		1.21 [1.09-1.34]	1.16 [1.05-1.30]	1.13 [1.01-1.26]	1.18 [1.05-1.32]		1.17 [1.05-1.30]	1,135	1,102	997	NA	1,085
R78	1.29 [0.97-1.71]	1.19 [0.90-1.59]	1.04 [0.78-1.40]		1.05 [0.79-1.40]	1.28 [0.96-1.71]	1.15 [0.86-1.54]	1.00 [0.74-1.35]		1.06 [0.79-1.43]	145	141	128	NA	125
R79	1.13 [1.11-1.16]	1.12 [1.10-1.15]	1.14 [1.11-1.16]	1.07 [0.89-1.28]	1.12 [1.09-1.15]	1.09 [1.07-1.12]	1.06 [1.04-1.09]	1.07 [1.05-1.10]	1.07 [0.89-1.29]	1.09 [1.07-1.12]	21,522	21,380	20,454	415	20,755
R80	1.19 [1.06-1.35]	1.22 [1.08-1.38]	1.24 [1.08-1.42]		1.18 [1.05-1.34]	1.17 [1.03-1.32]	1.17 [1.03-1.32]	1.18 [1.02-1.35]		1.17 [1.03-1.32]	800	758	622	NA	750
R81	1.01 [0.79-1.29]	1.04 [0.81-1.33]			1.18 [0.91-1.54]	0.98 [0.76-1.26]	1.01 [0.79-1.30]			1.19 [0.91-1.56]	182	176	NA	NA	154
R82	1.09 [1.00-1.19]	1.09 [1.00-1.19]	1.06 [0.96-1.16]	1.00 [0.75-1.34]	1.06 [0.97-1.15]	1.05 [0.96-1.15]	1.02 [0.93-1.11]	0.99 [0.89-1.09]	1.01 [0.74-1.38]	1.04 [0.96-1.14]	1,597	1,574	1,223	150	1,580
R84	1.23 [1.06-1.43]	1.22 [1.05-1.43]	1.31 [1.12-1.54]		1.14 [0.99-1.32]	1.13 [0.97-1.32]	1.08 [0.92-1.26]	1.18 [1.00-1.39]		1.05 [0.90-1.22]	544	490	467	NA	530
R85	1.25 [1.02-1.53]	1.25 [1.02-1.54]	1.18 [0.94-1.47]		1.23 [1.00-1.51]	1.17 [0.95-1.44]	1.17 [0.95-1.45]	1.08 [0.86-1.36]		1.15 [0.94-1.42]	290	281	231	NA	276
R87	1.06 [0.96-1.16]	1.08 [0.98-1.18]	0.98 [0.85-1.14]		0.97 [0.88-1.08]	1.02 [0.93-1.13]	1.03 [0.94-1.14]	0.95 [0.81-1.10]		0.97 [0.87-1.08]	1,293	1,332	464	NA	982
R89	1.31 [1.07-1.61]	1.39 [1.13-1.72]	1.41 [1.13-1.75]		1.31 [1.07-1.61]	1.28 [1.04-1.58]	1.31 [1.06-1.63]	1.33 [1.07-1.67]		1.29 [1.05-1.59]	295	276	256	NA	289
R90	1.14 [1.01-1.28]	1.10 [0.98-1.24]	1.15 [1.01-1.30]		1.19 [1.06-1.34]	1.10 [0.98-1.24]	1.05 [0.93-1.18]	1.10 [0.96-1.24]		1.17 [1.04-1.32]	832	809	712	NA	793
R91	1.18 [1.14-1.22]	1.17 [1.14-1.21]	1.19 [1.15-1.23]	1.17 [0.82-1.66]	1.17 [1.13-1.20]	1.14 [1.10-1.18]	1.10 [1.06-1.14]	1.12 [1.08-1.16]	1.19 [0.82-1.72]	1.14 [1.10-1.18]	10,382	10,345	10,063	108	9,895
R93	1.15 [1.11-1.19]	1.13 [1.09-1.16]	1.14 [1.10-1.18]	1.07 [0.85-1.34]	1.11 [1.08-1.15]	1.11 [1.07-1.15]	1.06 [1.03-1.10]	1.08 [1.04-1.12]	1.05 [0.83-1.33]	1.09 [1.06-1.13]	9,957	9,914	9,169	257	9,482
R94	1.18 [1.15-1.21]	1.18 [1.15-1.21]	1.17 [1.14-1.21]	0.98 [0.84-1.14]	1.15 [1.12-1.18]	1.14 [1.12-1.18]	1.11 [1.08-1.14]	1.11 [1.09-1.15]	0.96 [0.82-1.13]	1.13 [1.10-1.16]	18,296	18,265	16,589	564	17,461
Injury, poisoning and certain other consequences of external causes (S00-T88)															
S00	1.12 [1.09-1.15]	1.13 [1.10-1.17]	1.14 [1.11-1.18]	0.95 [0.86-1.04]	1.11 [1.08-1.14]	1.09 [1.06-1.12]	1.09 [1.06-1.12]	1.09 [1.06-1.12]	0.95 [0.86-1.04]	1.09 [1.06-1.12]	17,330	16,214	15,353	1,393	16,648
S01	1.13 [1.10-1.17]	1.13 [1.10-1.17]	1.13 [1.10-1.17]	0.98 [0.90-1.06]	1.12 [1.09-1.15]	1.11 [1.08-1.14]	1.09 [1.06-1.12]	1.09 [1.06-1.12]	0.97 [0.90-1.06]	1.11 [1.08-1.14]	17,034	15,476	14,575	1,875	16,101
S02	1.09 [1.04-1.15]	1.11 [1.05-1.18]	1.16 [1.09-1.24]	0.97 [0.85-1.10]	1.11 [1.05-1.17]	1.07 [1.01-1.13]	1.07 [1.01-1.13]	1.12 [1.05-1.19]	0.97 [0.85-1.11]	1.09 [1.03-1.15]	4,481	4,079	3,073	826	4,016
S03	1.08 [0.83-1.41]	1.16 [0.83-1.62]			1.07 [0.81-1.41]	1.01 [0.77-1.33]	1.08 [0.77-1.52]			1.06 [0.80-1.40]	178	112	NA	NA	156
S05	1.10 [1.01-1.21]	1.11 [1.01-1.22]	1.15 [1.05-1.27]		1.15 [1.05-1.26]	1.08 [0.99-1.19]	1.06 [0.96-1.17]	1.11 [1.00-1.22]		1.15 [1.05-1.26]	1,394	1,339	1,256	NA	1,329
S06	1.06 [1.01-1.11]	1.08 [1.02-1.13]	1.08 [1.02-1.13]	1.06 [0.86-1.32]	1.04 [0.99-1.10]	1.04 [0.99-1.09]	1.04 [0.99-1.10]	1.04 [0.99-1.10]	1.07 [0.86-1.34]	1.04 [0.99-1.09]	4,809	4,639	4,322	291	4,469
S09	1.13 [1.09-1.17]	1.15 [1.10-1.19]	1.18 [1.13-1.23]	0.96 [0.87-1.06]	1.12 [1.08-1.16]	1.09 [1.05-1.13]	1.07 [1.03-1.12]	1.11 [1.06-1.16]	0.94 [0.84-1.04]	1.10 [1.05-1.14]	8,595	7,571	6,909	1,248	8,073
S10	1.06 [0.89-1.25]	1.15 [0.96-1.37]	1.07 [0.88-1.31]		0.92 [0.77-1.09]	1.01 [0.85-1.20]	1.06 [0.89-1.27]	1.00 [0.82-1.23]		0.91 [0.76-1.08]	404	380	299	NA	379
S11	1.17 [0.92-1.49]	1.17 [0.91-1.49]	1.26 [0.93-1.70]		1.16 [0.91-1.48]	1.08 [0.85-1.39]	1.10 [0.85-1.41]	1.20 [0.88-1.63]		1.18 [0.92-1.51]	207	202	147	NA	204
S12	1.15 [1.04-1.26]	1.18 [1.07-1.30]	1.12 [1.01-1.23]		1.15 [1.04-1.26]	1.12 [1.01-1.24]	1.12 [1.02-1.24]	1.06 [0.96-1.18]		1.13 [1.03-1.25]	1,299	1,294	1,234	NA	1,242
S13	1.19 [0.96-1.47]	1.13 [0.91-1.40]	1.08 [0.84-1.39]		1.05 [0.85-1.30]	1.12 [0.90-1.40]	1.05 [0.84-1.30]	1.02 [0.79-1.32]		1.02 [0.82-1.27]	260	258	185	NA	236
S14	0.96 [0.76-1.23]	0.92 [0.72-1.17]	1.02 [0.80-1.31]		0.93 [0.72-1.19]	0.98 [0.76-1.25]	0.91 [0.71-1.16]	1.03 [0.80-1.32]		0.92 [0.71-1.19]	187	185	171	NA	165
S19	1.14 [0.95-1.37]	1.27 [1.05-1.53]	1.18 [0.95-1.46]		1.20 [1.00-1.45]	1.06 [0.88-1.28]	1.13 [0.93-1.38]	1.06 [0.85-1.32]		1.14 [0.94-1.38]	356	335	252	NA	339
S20	1.18 [1.09-1.27]	1.16 [1.07-1.25]	1.14 [1.05-1.24]		1.14 [1.05-1.24]	1.13 [1.04-1.22]	1.07 [0.99-1.16]	1.06 [0.97-1.15]		1.11 [1.02-1.20]	1,930	1,889	1,767	NA	1,858
S21	1.24 [0.99-1.55]	1.19 [0.95-1.49]	1.36 [1.05-1.76]		1.20 [0.95-1.50]	1.22 [0.97-1.54]	1.10 [0.87-1.39]	1.29 [0.99-1.68]		1.21 [0.96-1.53]	261	249	191	NA	236
S22	1.17 [1.12-1.22]	1.15 [1.10-1.20]	1.16 [1.11-1.21]	1.01 [0.72-1.42]	1.13 [1.08-1.18]	1.13 [1.08-1.18]	1.07 [1.03-1.12]	1.08 [1.03-1.13]	1.03 [0.72-1.47]	1.10 [1.05-1.15]	6,461	6,410	6,121	111	6,132
S27	1.11 [1.02-1.22]	1.09 [1.00-1.19]	1.13 [1.03-1.24]		1.05 [0.96-1.15]	1.09 [1.00-1.19]	1.05 [0.96-1.15]	1.09 [0.99-1.19]		1.05 [0.96-1.15]	1,547	1,499	1,347	NA	1,387
S29	1.37 [1.18-1.59]	1.40 [1.21-1.63]	1.38 [1.18-1.62]		1.29 [1.11-1.50]	1.29 [1.10-1.50]	1.29 [1.11-1.51]	1.26 [1.08-1.49]		1.22 [1.05-1.43]	557	551	514	NA	534
S30	1.19 [1.12-1.26]	1.17 [1.10-1.25]	1.22 [1.14-1.30]	0.93 [0.74-1.16]	1.15 [1.08-1.22]	1.14 [1.07-1.21]	1.09 [1.02-1.16]	1.14 [1.07-1.22]	0.94 [0.75-1.17]	1.12 [1.05-1.19]	3,322	3,159	2,858	267	3,236
S31	1.11 [0.97-1.27]	1.15 [0.99-1.33]	1.31 [1.10-1.57]	1.02 [0.78-1.34]	1.16 [1.01-1.33]	1.08 [0.94-1.24]	1.05 [0.90-1.22]	1.20 [1.00-1.44]	1.05 [0.79-1.40]	1.14 [0.99-1.32]	684	565	400	183	619
S32	1.10 [1.06-1.15]	1.10 [1.05-1.14]	1.12 [1.08-1.17]	0.88 [0.64-1.22]	1.08 [1.04-1.13]	1.07 [1.03-1.12]	1.05 [1.01-1.10]	1.08 [1.03-1.13]	0.97 [0.69-1.36]	1.06 [1.02-1.11]	7,154	7,141	6,881	123	6,811
S36	1.09 [0.94-1.26]	1.14 [0.98-1.32]	1.08 [0.92-1.28]		1.04 [0.90-1.21]	1.08 [0.94-1.25]	1.10 [0.95-1.28]	1.05 [0.89-1.25]		1.05 [0.90-1.22]	564	534	406	NA	494
S37	0.94 [0.81-1.10]	1.01 [0.86-1.19]	0.97 [0.81-1.15]		0.95 [0.81-1.12]	0.93 [0.79-1.09]	0.99 [0.84-1.17]	0.94 [0.79-1.13]		0.95 [0.81-1.12]	467	449	383	NA	438
S39	1.08 [0.98-1.20]	1.09 [0.99-1.21]	1.11 [0.99-1.24]	1.09 [0.79-1.50]	1.13 [1.02-1.25]	1.02 [0.92-1.13]	1.01 [0.91-1.12]	1.03 [0.92-1.15]	1.08 [0.77-1.51]	1.08 [0.98-1.20]	1,191	1,128	941	133	1,161
S40	1.11 [1.04-1.19]	1.13 [1.05-1.20]	1.13 [1.05-1.21]	0.84 [0.60-1.16]	1.12 [1.05-1.20]	1.07 [1.00-1.15]	1.06 [0.99-1.13]	1.07 [0.99-1.14]	0.86 [0.61-1.21]	1.09 [1.02-1.16]	2,849	2,771	2,646	114	2,777
S41	1.22 [1.09-1.37]	1.24 [1.10-1.39]	1.23 [1.09-1.39]		1.22 [1.09-1.36]	1.16 [1.04-1.30]	1.15 [1.02-1.29]	1.13 [1.00-1.28]		1.17 [1.05-1.31]	1,080	1,037	930	NA	1,040
S42	1.09 [1.05-1.13]	1.09 [1.05-1.14]	1.10 [1.06-1.15]	1.02 [0.90-1.15]	1.07 [1.03-1.12]	1.07 [1.03-1.11]	1.05 [1.01-1.10]	1.06 [1.02-1.11]	1.02 [0.90-1.16]	1.06 [1.02-1.11]	8,104	7,361	6,993	886	7,380

Supplementary Table 4: Hazard ratios and events from all cohorts (excluding non-consulters)

	Hazard ratio (99% confidence interval)														
	crude					adjusted					Events (in exposed)				
Outcome	any age	18+	40+	<18	hosp.	any age	18+	40+	<18	hosp.	any age	18+	40+	<18	hosp.
S43	1.04 [0.95-1.15]	1.06 [0.96-1.16]	1.04 [0.94-1.15]		1.08 [0.98-1.19]	1.02 [0.93-1.12]	1.03 [0.93-1.13]	1.01 [0.91-1.12]		1.07 [0.97-1.18]	1,282	1,285	1,185	NA	1,199
S46	1.05 [0.89-1.23]	1.05 [0.89-1.23]	1.01 [0.86-1.20]		1.02 [0.87-1.19]	1.01 [0.86-1.19]	1.01 [0.85-1.19]	0.94 [0.79-1.12]		0.98 [0.84-1.15]	483	465	423	NA	463
S49	1.10 [0.98-1.24]	1.19 [1.06-1.34]	1.13 [1.01-1.28]		1.13 [1.01-1.27]	1.04 [0.92-1.17]	1.10 [0.98-1.24]	1.06 [0.94-1.20]		1.10 [0.97-1.23]	891	874	826	NA	858
S50	1.15 [1.08-1.23]	1.12 [1.05-1.20]	1.14 [1.07-1.22]	0.88 [0.67-1.15]	1.11 [1.04-1.18]	1.11 [1.04-1.19]	1.06 [0.99-1.13]	1.08 [1.01-1.16]	0.89 [0.68-1.18]	1.08 [1.01-1.16]	3,024	2,893	2,715	174	2,898
S51	1.15 [1.09-1.22]	1.19 [1.12-1.26]	1.17 [1.11-1.25]	0.93 [0.74-1.17]	1.18 [1.12-1.25]	1.09 [1.03-1.15]	1.10 [1.04-1.17]	1.09 [1.03-1.16]	0.98 [0.77-1.25]	1.13 [1.07-1.20]	4,342	4,218	3,853	250	4,175
S52	1.08 [1.04-1.11]	1.09 [1.05-1.13]	1.09 [1.05-1.13]	0.97 [0.89-1.05]	1.06 [1.02-1.09]	1.05 [1.02-1.09]	1.05 [1.02-1.09]	1.06 [1.02-1.10]	0.95 [0.87-1.04]	1.04 [1.00-1.08]	10,338	8,549	7,791	2,009	8,934
S53	1.04 [0.84-1.29]	1.01 [0.79-1.28]	0.93 [0.71-1.22]		1.16 [0.92-1.46]	0.98 [0.78-1.22]	0.93 [0.73-1.20]	0.87 [0.66-1.15]		1.13 [0.89-1.43]	260	189	148	NA	224
S54	1.10 [0.78-1.53]	1.14 [0.80-1.61]			1.30 [0.92-1.83]	1.06 [0.75-1.51]	1.02 [0.71-1.46]			1.23 [0.86-1.75]	103	101	NA	NA	104
S56	0.97 [0.80-1.18]	0.99 [0.81-1.21]	1.04 [0.81-1.33]		0.94 [0.77-1.16]	0.95 [0.78-1.16]	0.94 [0.77-1.16]	1.00 [0.78-1.30]		0.94 [0.76-1.17]	309	287	172	NA	254
S59	1.26 [1.05-1.52]	1.18 [0.97-1.43]	1.25 [1.03-1.53]		1.19 [0.99-1.44]	1.20 [0.99-1.46]	1.09 [0.89-1.33]	1.15 [0.94-1.42]		1.16 [0.96-1.40]	362	332	314	NA	345
S60	1.15 [1.06-1.23]	1.15 [1.07-1.25]	1.18 [1.09-1.28]	0.88 [0.68-1.14]	1.11 [1.03-1.20]	1.11 [1.03-1.20]	1.10 [1.01-1.18]	1.12 [1.03-1.21]	0.90 [0.69-1.18]	1.08 [1.00-1.17]	2,231	2,105	1,883	195	2,148
S61	1.07 [1.02-1.12]	1.07 [1.02-1.12]	1.09 [1.04-1.15]	0.94 [0.85-1.04]	1.05 [1.01-1.10]	1.03 [0.98-1.07]	1.01 [0.96-1.05]	1.04 [0.99-1.10]	0.94 [0.85-1.05]	1.03 [0.98-1.08]	6,425	5,633	4,340	1,254	5,766
S62	1.05 [1.00-1.11]	1.06 [1.00-1.12]	1.07 [1.00-1.14]	1.01 [0.89-1.15]	1.01 [0.95-1.07]	1.03 [0.97-1.09]	1.01 [0.95-1.07]	1.02 [0.96-1.09]	1.00 [0.87-1.14]	1.00 [0.94-1.06]	4,095	3,643	2,795	831	3,577
S63	1.19 [1.05-1.35]	1.19 [1.05-1.35]	1.20 [1.04-1.38]		1.15 [1.01-1.31]	1.16 [1.02-1.32]	1.14 [1.00-1.30]	1.17 [1.01-1.35]		1.11 [0.98-1.27]	771	750	581	NA	673
S64	0.96 [0.85-1.10]	0.94 [0.83-1.07]	0.95 [0.81-1.12]	0.86 [0.64-1.16]	0.94 [0.82-1.07]	0.95 [0.84-1.09]	0.90 [0.79-1.03]	0.93 [0.78-1.10]	0.89 [0.65-1.21]	0.93 [0.81-1.06]	711	662	390	153	590
S65	0.89 [0.70-1.14]	0.97 [0.76-1.26]	0.90 [0.66-1.23]		0.96 [0.74-1.24]	0.88 [0.69-1.13]	0.94 [0.73-1.22]	0.87 [0.63-1.19]		0.96 [0.74-1.24]	193	171	111	NA	163
S66	1.07 [0.97-1.18]	0.98 [0.89-1.08]	0.95 [0.84-1.08]	1.02 [0.81-1.28]	0.97 [0.88-1.08]	1.05 [0.95-1.16]	0.94 [0.85-1.04]	0.93 [0.82-1.06]	1.03 [0.81-1.31]	0.96 [0.87-1.07]	1,297	1,228	703	260	1,045
S67	0.78 [0.59-1.04]				0.73 [0.53-0.99]	0.77 [0.57-1.03]				0.74 [0.54-1.02]	137	NA	NA	NA	108
S68	0.98 [0.84-1.15]	0.94 [0.78-1.13]	1.02 [0.83-1.25]	0.94 [0.70-1.26]	0.92 [0.77-1.09]	0.97 [0.83-1.14]	0.96 [0.80-1.16]	1.03 [0.84-1.26]	0.96 [0.71-1.29]	0.92 [0.77-1.10]	447	324	264	147	359
S69	1.23 [1.09-1.39]	1.22 [1.07-1.38]	1.20 [1.05-1.37]	1.04 [0.74-1.48]	1.20 [1.07-1.36]	1.17 [1.03-1.32]	1.11 [0.97-1.26]	1.10 [0.96-1.26]	1.16 [0.80-1.67]	1.15 [1.02-1.30]	868	783	665	108	816
S70	1.13 [1.06-1.21]	1.11 [1.04-1.19]	1.13 [1.06-1.21]	1.02 [0.74-1.42]	1.10 [1.03-1.18]	1.09 [1.02-1.16]	1.04 [0.97-1.11]	1.07 [1.00-1.15]	1.05 [0.75-1.48]	1.08 [1.01-1.15]	2,873	2,763	2,668	126	2,791
S71	1.07 [0.89-1.28]	1.15 [0.95-1.40]	1.23 [0.98-1.54]		1.13 [0.94-1.35]	1.01 [0.84-1.21]	1.07 [0.88-1.30]	1.15 [0.91-1.46]		1.08 [0.89-1.30]	391	342	231	NA	352
S72	1.07 [1.05-1.10]	1.08 [1.05-1.11]	1.08 [1.06-1.11]	0.95 [0.76-1.19]	1.09 [1.06-1.11]	1.07 [1.04-1.10]	1.06 [1.04-1.09]	1.06 [1.04-1.09]	0.98 [0.78-1.24]	1.09 [1.06-1.12]	19,778	19,593	19,431	244	18,303
S73	1.07 [0.86-1.34]	1.15 [0.92-1.44]	1.17 [0.92-1.47]		1.12 [0.90-1.40]	1.03 [0.82-1.29]	1.08 [0.86-1.36]	1.10 [0.86-1.39]		1.11 [0.89-1.39]	237	221	209	NA	225
S76	0.93 [0.79-1.10]	0.93 [0.79-1.10]	0.97 [0.82-1.15]		1.06 [0.90-1.24]	0.88 [0.75-1.04]	0.90 [0.76-1.06]	0.92 [0.77-1.10]		1.01 [0.85-1.19]	426	408	371	NA	398
S79	1.16 [1.06-1.27]	1.14 [1.04-1.24]	1.19 [1.08-1.30]		1.16 [1.06-1.26]	1.12 [1.02-1.23]	1.08 [0.98-1.18]	1.13 [1.03-1.24]		1.14 [1.04-1.24]	1,565	1,541	1,495	NA	1,499
S80	1.11 [1.06-1.17]	1.12 [1.07-1.17]	1.11 [1.06-1.17]	0.90 [0.71-1.13]	1.08 [1.03-1.13]	1.06 [1.01-1.11]	1.04 [0.99-1.10]	1.04 [0.98-1.09]	0.89 [0.70-1.13]	1.04 [0.99-1.09]	5,411	5,250	5,013	225	5,227
S81	1.19 [1.13-1.26]	1.19 [1.12-1.26]	1.22 [1.15-1.29]	1.07 [0.84-1.37]	1.17 [1.10-1.24]	1.11 [1.05-1.18]	1.09 [1.03-1.15]	1.12 [1.06-1.19]	1.09 [0.84-1.40]	1.10 [1.04-1.17]	4,074	3,930	3,716	224	3,896
S82	1.07 [1.03-1.11]	1.06 [1.02-1.10]	1.05 [1.01-1.09]	1.09 [0.96-1.24]	1.05 [1.01-1.09]	1.04 [1.00-1.07]	1.01 [0.97-1.05]	1.00 [0.96-1.05]	1.09 [0.96-1.24]	1.03 [0.99-1.07]	8,511	8,003	6,755	899	7,470
S83	1.14 [1.02-1.27]	1.17 [1.05-1.31]	1.12 [0.98-1.29]	1.39 [1.04-1.86]	1.11 [0.99-1.25]	1.08 [0.96-1.21]	1.10 [0.97-1.23]	1.05 [0.91-1.20]	1.35 [0.99-1.84]	1.06 [0.94-1.19]	984	931	636	183	866
S86	1.10 [0.95-1.27]	1.15 [0.99-1.33]	1.15 [0.98-1.35]		1.08 [0.93-1.26]	1.06 [0.91-1.23]	1.09 [0.94-1.27]	1.07 [0.91-1.27]		1.03 [0.88-1.20]	559	549	440	NA	473
S89	1.16 [1.05-1.29]	1.18 [1.06-1.31]	1.19 [1.07-1.32]		1.13 [1.02-1.25]	1.11 [1.00-1.23]	1.10 [0.99-1.22]	1.11 [1.00-1.24]		1.10 [0.99-1.22]	1,187	1,155	1,076	NA	1,133
S90	1.08 [0.98-1.20]	1.08 [0.97-1.19]	1.05 [0.95-1.17]	0.97 [0.68-1.38]	1.05 [0.95-1.15]	1.04 [0.94-1.15]	0.99 [0.89-1.10]	0.98 [0.88-1.09]	1.00 [0.70-1.44]	1.01 [0.92-1.12]	1,242	1,161	1,059	106	1,185
S91	1.13 [1.01-1.25]	1.19 [1.06-1.33]	1.23 [1.09-1.40]	1.03 [0.80-1.33]	1.10 [0.99-1.23]	1.07 [0.96-1.19]	1.08 [0.97-1.22]	1.13 [0.99-1.28]	0.98 [0.76-1.28]	1.07 [0.96-1.19]	1,101	981	818	207	1,021
S92	1.11 [1.03-1.20]	1.11 [1.03-1.20]	1.16 [1.06-1.26]	1.09 [0.81-1.47]	1.12 [1.04-1.22]	1.06 [0.98-1.15]	1.03 [0.95-1.12]	1.07 [0.98-1.16]	1.12 [0.82-1.53]	1.09 [1.00-1.18]	1,920	1,849	1,594	166	1,747
S93	1.10 [0.96-1.26]	1.06 [0.92-1.21]	1.10 [0.95-1.28]		1.06 [0.92-1.21]	1.04 [0.91-1.20]	0.98 [0.85-1.13]	1.03 [0.89-1.20]		1.02 [0.89-1.18]	631	615	515	NA	599
S96	1.11 [0.81-1.53]	1.07 [0.77-1.47]			1.06 [0.77-1.46]	1.02 [0.73-1.41]	0.90 [0.64-1.26]			0.93 [0.66-1.30]	118	112	NA	NA	108
S99	1.08 [0.93-1.26]	1.07 [0.92-1.24]	1.06 [0.90-1.25]		1.03 [0.89-1.20]	0.97 [0.83-1.14]	0.93 [0.79-1.09]	0.95 [0.81-1.13]		0.99 [0.85-1.15]	531	504	448	NA	498
T00	1.10 [0.99-1.22]	1.11 [1.00-1.24]	1.13 [1.01-1.26]		1.12 [1.00-1.24]	1.06 [0.95-1.18]	1.03 [0.92-1.15]	1.06 [0.95-1.19]		1.09 [0.98-1.21]	1,147	1,089	1,021	NA	1,114
T01	1.04 [0.88-1.22]	1.03 [0.88-1.22]	1.09 [0.92-1.30]		1.08 [0.93-1.26]	0.98 [0.83-1.16]	0.93 [0.78-1.10]	0.99 [0.83-1.19]		1.04 [0.89-1.22]	527	490	424	NA	518
T02	1.20 [1.00-1.45]	1.21 [1.00-1.47]	1.26 [1.03-1.55]		1.07 [0.88-1.30]	1.19 [0.98-1.44]	1.17 [0.96-1.42]	1.22 [1.00-1.50]		1.06 [0.87-1.29]	328	303	282	NA	291
T09	1.32 [1.03-1.70]	1.22 [0.95-1.58]	1.33 [1.02-1.75]		1.19 [0.93-1.53]	1.25 [0.96-1.62]	1.16 [0.89-1.50]	1.22 [0.92-1.61]		1.15 [0.89-1.48]	193	176	162	NA	178
T11	1.21 [1.07-1.37]	1.18 [1.04-1.34]	1.21 [1.06-1.37]		1.23 [1.09-1.39]	1.15 [1.01-1.30]	1.08 [0.95-1.23]	1.11 [0.97-1.27]		1.19 [1.05-1.35]	852	822	748	NA	837
T13	1.19 [1.02-1.39]	1.24 [1.06-1.45]	1.20 [1.02-1.41]		1.14 [0.98-1.33]	1.13 [0.97-1.32]	1.16 [0.99-1.36]	1.09 [0.93-1.29]		1.10 [0.94-1.28]	521	503	469	NA	495
T14	1.21 [1.03-1.42]	1.08 [0.92-1.28]	1.17 [0.99-1.39]		1.15 [0.98-1.35]	1.14 [0.97-1.35]	1.01 [0.85-1.19]	1.07 [0.89-1.27]		1.11 [0.94-1.31]	454	428	376	NA	412
T16	0.97 [0.82-1.14]			0.96 [0.80-1.14]	1.04 [0.87-1.24]	0.95 [0.81-1.12]			0.98 [0.81-1.17]	1.03 [0.86-1.23]	472	NA	NA	403	434
T17	1.16 [1.06-1.27]	1.24 [1.11-1.37]	1.18 [1.06-1.32]	1.04 [0.87-1.25]	1.13 [1.03-1.24]	1.10 [1.00-1.21]	1.15 [1.03-1.28]	1.11 [0.99-1.24]	1.03 [0.86-1.24]	1.09 [0.99-1.19]	1,432	1,080	979	381	1,305
T18	1.15 [1.03-1.27]	1.24 [1.11-1.39]	1.18 [1.04-1.34]	0.93 [0.74-1.16]	1.06 [0.95-1.18]	1.09 [0.98-1.21]	1.16 [1.03-1.30]	1.10 [0.97-1.25]	0.88 [0.69-1.11]	1.02 [0.91-1.13]	1,102	907	732	240	961
T19	1.21 [0.88-1.65]	1.38 [1.01-1.90]			1.20 [0.87-1.65]	1.11 [0.80-1.54]	1.33 [0.96-1.84]			1.18 [0.85-1.64]	121	114	NA	NA	110
T20	1.11 [0.94-1.32]	1.18 [0.95-1.47]	1.18 [0.93-1.51]	0.99 [0.76-1.28]	1.06 [0.88-1.26]	1.09 [0.92-1.30]	1.12 [0.90-1.40]	1.14 [0.89-1.46]	0.99 [0.76-1.30]	1.06 [0.89-1.28]	402	245	188	171	346
T21	1.05 [0.92-1.21]	1.18 [0.99-1.39]	1.20 [1.00-1.44]	0.93 [0.74-1.17]	1.08 [0.93-1.24]	1.05 [0.91-1.20]	1.12 [0.95-1.33]	1.14 [0.95-1.37]	0.95 [0.75-1.20]	1.07 [0.93-1.23]	632	437	369	218	577
T22	1.11 [0.95-1.29]	1.06 [0.88-1.28]	1.09 [0.88-1.36]	1.11 [0.87-1.42]	1.06 [0.90-1.23]	1.10 [0.94-1.28]	1.01 [0.84-1.23]	1.07 [0.86-1.33]	1.13 [0.88-1.46]	1.05 [0.90-1.23]	496	316	240	199	462
T23	1.11 [0.95-1.31]	1.03 [0.84-1.26]	1.12 [0.89-1.42]	1.15 [0.91-1.47]	0.98 [0.83-1.16]	1.10 [0.94-1.30]	0.97 [0.79-1.20]	1.07 [0.84-1.36]	1.16 [0.90-1.49]	0.97 [0.81-1.15]	467	283	208	203	394
T24	0.85 [0.72-1.00]	0.91 [0.75-1.10]	0.93 [0.76-1.13]		0.87 [0.73-1.04]	0.85 [0.71-1.01]	0.87 [0.72-1.06]	0.91 [0.74-1.12]		0.88 [0.74-1.05]	400	318	278	NA	357
T25	0.80 [0.63-1.02]	0.88 [0.65-1.18]	0.84 [0.61-1.17]		0.90 [0.70-1.15]	0.77 [0.60-0.99]	0.84 [0.62-1.13]	0.79 [0.56-1.10]		0.88 [0.69-1.14]	198	127	106	NA	177
T31	1.00 [0.90-1.10]	1.00 [0.88-1.13]	1.07 [0.93-1.24]	0.99 [0.85-1.15]	0.99 [0.90-1.10]	0.98 [0.89-1.08]	0.95 [0.84-1.08]	1.03 [0.89-1.19]	1.00 [0.86-1.16]	0.99 [0.89-1.10]	1,231	730	569	544	1,109
T36	1.26 [1.04-1.52]	1.20 [0.99-1.46]	1.37 [1.04-1.81]		1.22 [1.01-1.48]	1.15 [0.94-1.40]	1.05 [0.86-1.29]	1.24 [0.93-1.65]		1.19 [0.98-1.45]	341	316	158	NA	317
T37	1.33 [1.01-1.76]	1.37 [1.02-1.84]			1.30 [0.99-1.71]	1.20 [0.90-1.61]	1.14 [0.84-1.56]			1.25 [0.95-1.66]	162	149	NA	NA	160
T38	1.15 [1.01-1.30]	1.13 [0.99-1.28]	1.14 [0.98-1.32]		1.09 [0.97-1.24]	1.06 [0.93-1.20]	0.96 [0.84-1.10]	0.98 [0.84-1.14]		1.04 [0.92-1.18]	742	709	538	NA	752
T39	1.09 [1.04-1.14]	1.07 [1.02-1.12]	1.20 [1.12-1.28]	0.95 [0.87-1.04]	1.04 [0.99-1.09]	1.04 [0.99-1.09]	0.94 [0.90-0.99]	1.06 [0.99-1.14]	0.96 [0.87-1.05]	1.03 [0.98-1.08]	5,432	5,003	2,411	1,548	4,851
T40	1.17 [1.10-1.25]	1.16 [1.09-1.23]	1.21 [1.12-1.30]	0.98 [0.83-1.16]	1.08 [1.02-1.15]	1.08 [1.01-1.15]	0.98 [0.92-1.05]	1.03 [0.95-1.12]	1.00 [0.84-1.19]	1.05 [0.99-1.12]	3,171	3,202	1,882	465	3,091
T42	1.11 [1.03-1.19]	1.14 [1.07-1.23]	1.18 [1.08-1.29]	0.75 [0.60-0.93]	1.04 [0.97-1.12]	1.03 [0.96-1.11]	0.98 [0.91-1.05]	1.02 [0.93-1.12]	0.80 [0.63-1.00]	1.03 [0.96-1.11]	2,336	2,354	1,432	251	2,213
T43	1.14 [1.07-1.21]	1.14 [1.07-1.20]	1.21 [1.11-1.31]	0.91 [0.80-1.05]	1.06 [1.00-1.13]	1.07 [1.00-1.13]	0.98 [0.92-1.04]	1.05 [0.96-1.15]	0.95 [0.83-1.10]	1.06 [1.00-1.13]	3,376	3,399	1,555	672	3,099
T44	1.09 [0.96-1.23]	1.10 [0.97-1.24]	1.11 [0.95-1.30]	0.92 [0.68-1.24]	1.08 [0.96-1.22]	1.05 [0.92-1.19]	0.98 [0.86-1.11]	0.98 [0.83-1.16]	0.94 [0.69-1.28]	1.08 [0.96-1.23]	772	755	455	136	752
T45	1.21 [1.08-1.35]	1.19 [1.06-1.33]	1.21 [1.06-1.39]	1.14 [0.90-1.45]	1.14 [1.03-1.27]	1.11 [0.99-1.24]	1.06 [0.94-1.19]	1.06 [0.92-1.23]	1.13 [0.88-1.45]	1.10 [0.99-1.23]	1,026	946	621	238	1,002
T46	1.09 [0.98-1.22]	1.10 [0.98-1.23]	1.12 [1.00-1.26]		1.06 [0.95-1.18]	1.04 [0.93-1.16]	0.99 [0.88-1.12]	1.00 [0.89-1.13]		1.03 [0.92-1.16]	920	888	795	NA	900
T47	1.20 [1.01-1.42]	1.21 [1.02-1.44]	1.17 [0.95-1.44]		1.13 [0.96-1.34]	1.08 [0.91-1.29]	1.00 [0.83-1.20]	0.99 [0.79-1.23]		1.09 [0.92-1.29]	421	406	269	NA	416
T48	1.26 [0.96-1.65]	1.42 [1.04-1.96]			1.22 [0.93-1.60]	1.03 [0.77-1.38]	1.07 [0.76-1.52]			1.06 [0.80-1.41]	178	122	NA	NA	165
T49	1.05 [0.78-1.40]				1.05 [0.77-1.42]	0.99 [0.74-1.33]				1.03 [0.76-1.40]	138	NA	NA	NA	120
T50	1.11 [0.99-1.24]	1.11 [0.99-1.25]	1.17 [1.02-1.34]	1.02 [0.75-1.38]	1.10 [0.98-1.23]	1.03 [0.92-1.16]	0.97 [0.86-1.09]	1.03 [0.90-1.19]	1.03 [0.75-1.41]	1.06 [0.95-1.19]	957	881	636	133	880
T51	1.12 [1.05-1.20]	1.07 [1.00-1.14]	1.17 [1.07-1.29]	0.96 [0.81-1.13]	1.04 [0.97-1.11]	1.05 [0.98-1.13]	0.92 [0.86-0.98]	1.00 [0.91-1.10]	1.07 [0.89-1.28]	1.04 [0.97-1.11]	2,733	2,806	1,315	451	2,525
T52	1.02 [0.74-1.40]				1.03 [0.74-1.43]	0.96 [0.69-1.34]				1.02 [0.73-1.42]	111	NA	NA	NA	106
T54	0.90 [0.66-1.24]				0.97 [0.71-1.32]	0.89 [0.64-1.23]				0.98 [0.72-1.35]	122	NA	NA	NA	116
T59	0.94 [0.75-1.18]	0.93 [0.74-1.17]	1.04 [0.82-1.33]		0.85 [0.68-1.07]	0.94 [0.74-1.18]	0.86 [0.68-1.09]	0.99 [0.78-1.27]		0.84 [0.67-1.06]	217	209	186	NA	206
T63	0.95 [0.69-1.33]					0.85 [0.60-1.20]					103	NA	NA	NA	NA
T65	1.01 [0.76-1.34]				0.95 [0.71-1.27]	0.97 [0.73-1.30]				0.93 [0.69-1.25]	142	NA	NA	NA	135
T68	1.07 [0.98-1.18]	1.08 [0.98-1.18]	1.08 [0.99-1.19]		1.06 [0.96-1.16]	1.09 [0.99-1.20]	1.08 [0.98-1.19]	1.09 [0.99-1.20]		1.10 [1.00-1.20]	1,443	1,438	1,411	NA	1,420
T74	1.03 [0.75-1.40]				1.04 [0.77-1.41]	0.96 [0.70-1.32]				1.04 [0.76-1.42]	128	NA	NA	NA	123
T78	2.27 [2.13-2.40]	1.67 [1.55-1.79]	1.55 [1.43-1.68]	5.30 [4.70-5.99]	2.18 [2.05-2.31]	1.96 [1.84-2.09]	1.41 [1.31-1.52]	1.37 [1.26-1.49]	4.23 [3.72-4.80]	1.90 [1.78-2.02]	4,543	2,758	1,866	2,136	4,068
T79	1.08 [0.99-1.17]	1.09 [1.00-1.19]	1.09 [0.99-1.19]		1.04 [0.95-1.14]	1.06 [0.97-1.15]	1.05 [0.96-1.15]	1.05 [0.96-1.15]		1.05 [0.96-1.14]	1,685	1,643	1,504	NA	1,516
T80	1.25 [1.11-1.40]	1.24 [1.10-1.40]	1.30 [1.14-1.47]		1.22 [1.09-1.37]	1.19 [1.06-1.34]	1.17 [1.04-1.32]	1.21 [1.07-1.38]		1.21 [1.07-1.36]	873	832	746	NA	824
T81	1.18 [1.16-1.21]	1.19 [1.16-1.21]	1.18 [1.15-1.20]	1.16 [1.06-1.28]	1.15 [1.13-1.18]	1.13 [1.11-1.16]	1.11 [1.09-1.14]	1.11 [1.08-1.13]	1.15 [1.05-1.27]	1.12 [1.10-1.15]	26,204	25,714	22,140	1,560	24,446
T82	1.21 [1.16-1.26]	1.21 [1.16-1.26]	1.20 [1.15-1.25]	1.03 [0.82-1.30]	1.14 [1.10-1.19]	1.17 [1.12-1.22]	1.13 [1.09-1.18]	1.11 [1.06-1.16]	1.02 [0.81-1.29]	1.13 [1.09-1.18]	7,103	6,922	6,570	258	6,949
T83	1.06 [1.01-1.10]	1.07 [1.03-1.12]	1.06 [1.01-1.11]	0.88 [0.64-1.21]	1.04 [1.00-1.08]	1.04 [0.99-1.08]	1.04 [0.99-1.08]	1.02 [0.98-1.07]	0.91 [0.65-1.27]	1.04 [1.00-1.09]	6,466	6,498	5,893	129	6,378
T84	1.21 [1.17-1.26]	1.22 [1.17-1.26]	1.21 [1.17-1.26]	1.14 [0.90-1.44]	1.17 [1.13-1.21]	1.16 [1.12-1.21]	1.15 [1.11-1.20]	1.15 [1.10-1.19]	1.13 [0.88-1.44]	1.14 [1.10-1.18]	8,565	8,481	7,868	274	8,290
T85	1.16 [1.11-1.22]	1.20 [1.15-1.26]	1.19 [1.14-1.25]	0.88 [0.73-1.07]	1.13 [1.08-1.18]	1.12 [1.07-1.17]	1.13 [1.08-1.19]	1.12 [1.07-1.18]	0.86 [0.71-1.05]	1.11 [1.06-1.16]	5,497	5,315	4,722	348	5,290
T86	1.17 [1.03-1.32]	1.16 [1.02-1.31]	1.17 [1.02-1.34]		1.13 [1.00-1.28]	1.14 [1.00-1.30]	1.12 [0.98-1.27]	1.09 [0.95-1.26]		1.15 [1.01-1.30]	735	709	601	NA	729
T87	1.17 [1.01-1.36]	1.11 [0.96-1.29]	1.15 [0.99-1.34]		1.12 [0.97-1.30]	1.11 [0.95-1.29]	1.03 [0.88-1.20]	1.02 [0.88-1.20]		1.10 [0.94-1.28]	517	510	492	NA	501
T88	1.31 [1.21-1.42]	1.32 [1.21-1.43]	1.27 [1.16-1.39]	1.54 [1.15-2.07]	1.28 [1.18-1.39]	1.20 [1.10-1.30]	1.18 [1.08-1.28]	1.15 [1.05-1.26]	1.49 [1.09-2.02]	1.20 [1.11-1.31]	1,807	1,713	1,432	179	1,718
T90	1.20 [1.08-1.33]	1.15 [1.04-1.29]	1.27 [1.12-1.44]	1.33 [0.99-1.80]	1.11 [0.99-1.23]	1.15 [1.03-1.28]	1.09 [0.97-1.21]	1.18 [1.03-1.34]	1.34 [0.98-1.83]	1.09 [0.98-1.22]	1,087	1,021	722	164	982
T91	1.19 [1.04-1.38]	1.12 [0.97-1.29]	1.12 [0.96-1.31]		1.11 [0.97-1.28]	1.12 [0.97-1.30]	1.03 [0.89-1.19]	1.03 [0.88-1.21]		1.06 [0.92-1.22]	580	563	458	NA	557
T92	1.09 [0.97-1.22]	1.11 [0.98-1.25]	1.14 [1.00-1.30]		1.06 [0.94-1.20]	1.05 [0.94-1.19]	1.04 [0.92-1.18]	1.09 [0.95-1.24]		1.04 [0.92-1.18]	829	805	636	NA	736
T93	1.19 [1.08-1.32]	1.19 [1.08-1.32]	1.18 [1.05-1.32]	1.36 [0.93-1.98]	1.15 [1.04-1.28]	1.13 [1.02-1.26]	1.10 [0.99-1.23]	1.09 [0.96-1.22]	1.34 [0.91-1.97]	1.11 [1.00-1.24]	1,116	1,088	839	104	1,020
T94	1.02 [0.75-1.39]	1.10 [0.81-1.50]	1.06 [0.76-1.48]		1.18 [0.86-1.62]	1.02 [0.74-1.39]	1.00 [0.73-1.38]	0.97 [0.69-1.37]		1.12 [0.81-1.55]	122	119	105	NA	116
T95	1.13 [0.81-1.56]					1.10 [0.78-1.53]					106	NA	NA	NA	NA
T98	1.16 [0.93-1.45]	1.33 [1.06-1.67]	1.29 [1.02-1.63]		1.08 [0.87-1.35]	1.13 [0.89-1.41]	1.27 [1.01-1.60]	1.23 [0.97-1.56]		1.04 [0.83-1.30]	226	232	210	NA	213
T56					1.10 [0.80-1.53]					1.12 [0.81-1.56]	NA	NA	NA	NA	102
Codes for special purposes (U00-U89)															
U07	1.15 [1.11-1.20]	1.13 [1.09-1.18]	1.16 [1.12-1.21]	0.90 [0.71-1.14]	1.15 [1.11-1.20]	1.09 [1.05-1.14]	1.06 [1.02-1.10]	1.08 [1.04-1.12]	0.85 [0.66-1.09]	1.11 [1.07-1.16]	9,064	9,073	8,310	248	8,991
U80	1.38 [1.30-1.47]	1.39 [1.31-1.47]	1.36 [1.28-1.45]	1.47 [1.04-2.10]	1.37 [1.30-1.46]	1.32 [1.25-1.41]	1.29 [1.22-1.37]	1.26 [1.18-1.34]	1.43 [0.99-2.08]	1.33 [1.26-1.41]	3,571	3,519	3,276	126	3,550
U81	1.13 [0.87-1.47]	1.05 [0.81-1.36]	0.99 [0.76-1.29]		1.08 [0.83-1.39]	1.10 [0.84-1.44]	0.99 [0.76-1.29]	0.95 [0.72-1.24]		1.04 [0.80-1.35]	173	177	166	NA	171
U82	1.26 [1.21-1.32]	1.26 [1.20-1.32]	1.24 [1.19-1.30]	0.99 [0.79-1.24]	1.26 [1.20-1.31]	1.21 [1.16-1.26]	1.16 [1.11-1.22]	1.15 [1.10-1.21]	1.02 [0.80-1.29]	1.23 [1.18-1.29]	6,721	6,637	6,117	265	6,688
U83	1.20 [1.13-1.27]	1.22 [1.15-1.29]	1.22 [1.15-1.29]	1.01 [0.75-1.37]	1.23 [1.17-1.30]	1.16 [1.09-1.23]	1.13 [1.06-1.19]	1.13 [1.06-1.20]	1.01 [0.74-1.38]	1.21 [1.15-1.28]	3,966	3,917	3,637	158	3,971
U88	1.20 [1.06-1.35]	1.16 [1.03-1.31]	1.23 [1.09-1.40]		1.12 [0.99-1.26]	1.15 [1.01-1.30]	1.09 [0.96-1.24]	1.16 [1.02-1.32]		1.08 [0.96-1.22]	802	792	762	NA	798
U89	1.24 [1.11-1.39]	1.21 [1.08-1.36]	1.21 [1.08-1.36]		1.24 [1.11-1.39]	1.19 [1.06-1.33]	1.12 [0.99-1.26]	1.12 [0.99-1.26]		1.20 [1.07-1.35]	925	895	846	NA	919
External causes of morbidity (V00-Y99)															
V03	1.16 [1.03-1.30]	1.10 [0.97-1.25]	1.20 [1.05-1.37]	1.07 [0.84-1.38]	1.06 [0.94-1.20]	1.13 [1.01-1.27]	1.06 [0.93-1.20]	1.16 [1.01-1.33]	1.09 [0.84-1.41]	1.05 [0.93-1.19]	913	746	632	206	768
V09	1.19 [0.97-1.46]	1.28 [1.03-1.58]	1.21 [0.98-1.51]		1.18 [0.97-1.45]	1.10 [0.90-1.36]	1.13 [0.91-1.41]	1.10 [0.88-1.38]		1.13 [0.92-1.38]	303	271	254	NA	290
V13	0.82 [0.64-1.06]	0.84 [0.64-1.10]			0.76 [0.58-1.01]	0.82 [0.64-1.06]	0.87 [0.66-1.15]			0.77 [0.58-1.03]	178	154	NA	NA	131
V18	1.02 [0.91-1.14]	1.02 [0.90-1.17]	1.07 [0.93-1.25]	1.03 [0.87-1.24]	1.03 [0.92-1.16]	1.00 [0.89-1.12]	1.02 [0.89-1.17]	1.10 [0.94-1.28]	0.99 [0.83-1.19]	1.02 [0.91-1.15]	1,051	653	498	451	849
V19	1.09 [0.83-1.43]	0.96 [0.68-1.34]			1.06 [0.79-1.42]	1.07 [0.82-1.41]	0.98 [0.69-1.38]			1.05 [0.78-1.42]	174	103	NA	NA	141
V23	0.93 [0.73-1.19]	0.86 [0.67-1.10]	0.80 [0.58-1.11]		0.80 [0.61-1.05]	0.91 [0.71-1.17]	0.81 [0.62-1.05]	0.80 [0.58-1.11]		0.82 [0.62-1.08]	194	168	101	NA	150
V28	0.99 [0.81-1.21]	0.98 [0.79-1.21]	0.95 [0.73-1.23]		0.88 [0.71-1.10]	0.94 [0.76-1.16]	0.94 [0.75-1.18]	0.88 [0.67-1.16]		0.87 [0.69-1.09]	286	253	163	NA	230
V43	1.11 [1.00-1.24]	1.08 [0.97-1.20]	1.12 [0.99-1.27]	0.99 [0.71-1.38]	1.04 [0.93-1.16]	1.05 [0.95-1.18]	1.02 [0.92-1.14]	1.04 [0.92-1.19]	0.99 [0.70-1.40]	1.02 [0.91-1.14]	1,016	1,011	687	116	883
V47	1.14 [0.96-1.35]	1.29 [1.09-1.52]	1.30 [1.05-1.60]		1.28 [1.08-1.53]	1.11 [0.93-1.32]	1.25 [1.05-1.48]	1.20 [0.97-1.49]		1.25 [1.04-1.49]	424	415	282	NA	390
V48	1.07 [0.93-1.22]	1.03 [0.90-1.18]	1.10 [0.95-1.28]		1.03 [0.89-1.18]	1.03 [0.90-1.19]	0.99 [0.86-1.13]	1.05 [0.90-1.22]		1.01 [0.87-1.16]	623	617	513	NA	560
V49	1.06 [0.89-1.25]	1.07 [0.91-1.27]	1.05 [0.86-1.28]		1.11 [0.93-1.32]	1.02 [0.86-1.21]	1.01 [0.85-1.19]	0.99 [0.81-1.22]		1.10 [0.92-1.31]	420	424	280	NA	362
V78	1.11 [0.92-1.35]	1.13 [0.94-1.37]	1.15 [0.95-1.40]		1.14 [0.94-1.38]	1.07 [0.89-1.30]	1.08 [0.89-1.31]	1.09 [0.90-1.33]		1.12 [0.92-1.36]	317	313	300	NA	302
V80	0.93 [0.80-1.10]	0.88 [0.75-1.04]	0.84 [0.66-1.07]	0.90 [0.65-1.24]	0.89 [0.75-1.07]	0.92 [0.78-1.08]	0.87 [0.74-1.03]	0.86 [0.67-1.10]	0.89 [0.63-1.25]	0.89 [0.75-1.07]	428	412	165	123	341
V89	1.24 [0.97-1.59]	1.25 [0.97-1.60]	1.54 [1.11-2.15]		1.07 [0.83-1.39]	1.15 [0.89-1.48]	1.13 [0.87-1.47]	1.37 [0.97-1.94]		1.02 [0.78-1.34]	188	187	107	NA	163
W00	1.05 [0.95-1.17]	1.04 [0.93-1.16]	1.06 [0.95-1.19]		1.07 [0.95-1.20]	1.04 [0.93-1.16]	1.03 [0.92-1.15]	1.05 [0.94-1.18]		1.05 [0.94-1.18]	938	942	832	NA	806
W01	1.11 [1.09-1.14]	1.12 [1.09-1.14]	1.11 [1.09-1.14]	1.02 [0.91-1.13]	1.10 [1.08-1.13]	1.08 [1.05-1.10]	1.07 [1.05-1.10]	1.07 [1.04-1.09]	1.01 [0.91-1.13]	1.09 [1.06-1.11]	23,340	22,519	21,652	1,105	21,582
W02	1.00 [0.85-1.18]	1.09 [0.88-1.35]	1.10 [0.81-1.48]	0.97 [0.76-1.23]	0.94 [0.78-1.13]	0.98 [0.82-1.16]	1.07 [0.86-1.33]	1.07 [0.78-1.47]	0.98 [0.77-1.25]	0.92 [0.76-1.10]	445	250	126	236	354
W03	1.02 [0.89-1.17]	0.99 [0.84-1.16]	1.04 [0.85-1.28]	0.95 [0.78-1.17]	0.95 [0.82-1.11]	1.01 [0.88-1.16]	0.95 [0.80-1.12]	1.00 [0.81-1.23]	0.95 [0.77-1.17]	0.95 [0.81-1.10]	659	429	257	327	523
W04	0.92 [0.69-1.23]				1.07 [0.80-1.44]	0.93 [0.69-1.25]				1.13 [0.84-1.53]	134	NA	NA	NA	133
W05	1.15 [1.00-1.34]	1.18 [1.02-1.37]	1.19 [1.03-1.38]		1.13 [0.98-1.30]	1.12 [0.96-1.30]	1.14 [0.98-1.32]	1.11 [0.96-1.29]		1.12 [0.97-1.29]	551	549	537	NA	550
W06	1.10 [1.06-1.16]	1.11 [1.06-1.16]	1.11 [1.06-1.16]	1.05 [0.85-1.30]	1.11 [1.06-1.16]	1.08 [1.03-1.13]	1.06 [1.01-1.11]	1.06 [1.01-1.11]	1.04 [0.84-1.29]	1.09 [1.05-1.15]	6,095	5,838	5,790	270	5,961
W07	1.13 [1.06-1.20]	1.13 [1.06-1.20]	1.14 [1.07-1.21]	1.01 [0.82-1.25]	1.10 [1.03-1.17]	1.10 [1.04-1.17]	1.08 [1.02-1.16]	1.09 [1.02-1.16]	1.04 [0.84-1.28]	1.09 [1.02-1.16]	3,396	3,132	3,091	278	3,259
W08	1.20 [1.07-1.34]	1.17 [1.02-1.33]	1.15 [1.01-1.31]	0.98 [0.77-1.24]	1.14 [1.02-1.28]	1.16 [1.03-1.30]	1.09 [0.96-1.25]	1.08 [0.94-1.23]	0.97 [0.76-1.23]	1.12 [1.00-1.26]	956	753	726	210	914
W09	0.98 [0.88-1.09]			0.97 [0.86-1.09]	1.00 [0.88-1.13]	0.96 [0.86-1.08]			0.96 [0.85-1.08]	0.99 [0.87-1.12]	1,045	NA	NA	979	903
W10	1.10 [1.07-1.14]	1.11 [1.07-1.15]	1.12 [1.08-1.17]	0.97 [0.84-1.12]	1.09 [1.05-1.13]	1.07 [1.03-1.11]	1.05 [1.02-1.09]	1.07 [1.03-1.11]	0.94 [0.81-1.09]	1.07 [1.03-1.11]	9,070	8,726	8,027	569	8,461
W11	1.02 [0.92-1.12]	1.04 [0.94-1.15]	1.00 [0.90-1.11]		1.03 [0.92-1.14]	0.98 [0.89-1.09]	1.01 [0.92-1.12]	0.96 [0.87-1.07]		1.01 [0.90-1.12]	1,096	1,061	1,007	NA	935
W13	1.10 [0.94-1.28]	1.01 [0.84-1.20]	1.04 [0.85-1.28]	1.03 [0.80-1.33]	1.06 [0.90-1.24]	1.07 [0.91-1.25]	0.96 [0.80-1.15]	1.00 [0.81-1.23]	1.03 [0.79-1.34]	1.06 [0.90-1.25]	514	363	263	193	437
W14	1.13 [0.82-1.57]					1.13 [0.81-1.57]					124	NA	NA	NA	NA
W17	1.08 [0.97-1.19]	1.13 [1.01-1.27]	1.14 [1.01-1.29]	1.00 [0.83-1.20]	1.06 [0.96-1.18]	1.06 [0.96-1.17]	1.09 [0.97-1.22]	1.09 [0.96-1.24]	0.99 [0.82-1.20]	1.04 [0.93-1.15]	1,223	909	770	377	1,085
W18	1.15 [1.12-1.18]	1.16 [1.13-1.19]	1.16 [1.13-1.19]	0.95 [0.82-1.10]	1.13 [1.10-1.16]	1.12 [1.09-1.15]	1.10 [1.07-1.13]	1.10 [1.07-1.13]	0.94 [0.81-1.09]	1.11 [1.08-1.14]	16,837	16,373	15,857	597	16,089
W19	1.13 [1.11-1.15]	1.13 [1.11-1.15]	1.13 [1.11-1.15]	0.97 [0.86-1.08]	1.12 [1.10-1.14]	1.10 [1.08-1.12]	1.09 [1.07-1.11]	1.08 [1.06-1.11]	0.96 [0.85-1.07]	1.11 [1.09-1.13]	38,290	37,557	36,306	1,031	36,033
W20	1.05 [0.91-1.20]	1.02 [0.88-1.19]	1.16 [0.98-1.37]	0.90 [0.66-1.23]	1.04 [0.89-1.20]	1.01 [0.87-1.16]	0.96 [0.82-1.12]	1.09 [0.91-1.29]	0.87 [0.63-1.20]	0.97 [0.84-1.13]	593	490	388	133	512
W21	1.04 [0.84-1.28]	0.94 [0.72-1.23]		0.94 [0.71-1.24]	0.91 [0.73-1.15]	0.97 [0.78-1.21]	0.87 [0.66-1.15]		0.94 [0.71-1.26]	0.87 [0.68-1.10]	301	168	NA	185	242
W22	1.13 [1.06-1.20]	1.12 [1.05-1.19]	1.13 [1.05-1.21]	1.09 [0.96-1.23]	1.10 [1.04-1.17]	1.08 [1.02-1.15]	1.04 [0.98-1.11]	1.05 [0.98-1.13]	1.07 [0.94-1.22]	1.06 [1.00-1.13]	3,559	2,946	2,430	836	3,332
W23	1.08 [0.99-1.17]	1.07 [0.96-1.19]	1.09 [0.97-1.23]	1.00 [0.88-1.15]	1.02 [0.93-1.11]	1.05 [0.97-1.15]	1.03 [0.92-1.14]	1.07 [0.94-1.20]	1.00 [0.87-1.14]	1.00 [0.92-1.10]	1,671	1,042	813	717	1,445
W25	1.06 [0.93-1.20]	1.07 [0.94-1.21]	1.14 [0.95-1.37]	0.98 [0.78-1.24]	1.09 [0.96-1.24]	1.03 [0.90-1.17]	0.99 [0.86-1.14]	1.08 [0.89-1.30]	0.98 [0.77-1.24]	1.07 [0.93-1.22]	784	683	341	251	687
W26	1.00 [0.88-1.13]	0.99 [0.87-1.13]	1.04 [0.88-1.22]	0.87 [0.66-1.14]	0.94 [0.83-1.07]	0.95 [0.84-1.08]	0.93 [0.82-1.06]	0.96 [0.81-1.13]	0.87 [0.65-1.16]	0.92 [0.81-1.05]	808	755	427	169	694
W27	0.98 [0.82-1.18]	1.02 [0.85-1.22]	1.04 [0.84-1.29]		0.98 [0.82-1.18]	0.95 [0.79-1.14]	0.95 [0.78-1.15]	0.95 [0.76-1.19]		0.93 [0.77-1.13]	358	326	230	NA	315
W29	0.92 [0.78-1.10]	0.87 [0.74-1.04]	0.93 [0.77-1.13]		0.88 [0.74-1.05]	0.91 [0.76-1.08]	0.85 [0.72-1.02]	0.92 [0.76-1.11]		0.87 [0.73-1.05]	369	370	297	NA	330
W31	0.90 [0.75-1.08]	0.92 [0.76-1.12]	0.94 [0.76-1.15]		0.94 [0.77-1.15]	0.90 [0.75-1.09]	0.95 [0.78-1.15]	0.93 [0.75-1.14]		0.94 [0.76-1.15]	328	301	249	NA	262
W44	1.11 [1.03-1.20]	1.23 [1.12-1.35]	1.22 [1.10-1.36]	0.97 [0.86-1.09]	1.09 [1.01-1.18]	1.06 [0.98-1.15]	1.15 [1.04-1.27]	1.15 [1.03-1.29]	0.96 [0.85-1.08]	1.05 [0.97-1.14]	2,046	1,286	1,042	853	1,818

Supplementary Table 4: Hazard ratios and events from all cohorts (excluding non-consulters)

	Hazard ratio (99% confidence interval)										Events (in exposed)				
	crude					adjusted									
Outcome	any age	18+	40+	<18	hosp.	any age	18+	40+	<18	hosp.	any age	18+	40+	<18	hosp.
W45	1.03 [0.89-1.18]	1.07 [0.92-1.25]	0.97 [0.81-1.17]	1.28 [0.98-1.66]	1.03 [0.89-1.19]	1.00 [0.86-1.15]	1.02 [0.87-1.19]	0.93 [0.77-1.12]	1.21 [0.92-1.59]	1.00 [0.86-1.16]	632	489	319	218	530
W46	0.97 [0.69-1.36]	1.12 [0.80-1.57]			1.09 [0.78-1.52]	0.93 [0.65-1.31]	1.00 [0.71-1.43]			1.10 [0.77-1.56]	105	105	NA	NA	103
W49	1.36 [1.04-1.77]	1.28 [0.97-1.68]	1.34 [0.98-1.84]		1.35 [1.03-1.76]	1.34 [1.02-1.76]	1.21 [0.91-1.61]	1.29 [0.94-1.77]		1.29 [0.98-1.70]	179	158	121	NA	166
W50	1.11 [0.96-1.28]	1.13 [0.96-1.33]	1.15 [0.90-1.47]	1.15 [0.93-1.42]	1.09 [0.94-1.26]	1.08 [0.94-1.25]	1.03 [0.87-1.22]	1.08 [0.84-1.39]	1.13 [0.90-1.40]	1.08 [0.92-1.25]	685	494	190	326	558
W51	0.97 [0.82-1.14]	0.96 [0.79-1.18]	1.01 [0.76-1.34]	1.07 [0.85-1.35]	1.08 [0.91-1.28]	0.95 [0.80-1.12]	0.90 [0.73-1.11]	0.98 [0.73-1.30]	1.03 [0.81-1.32]	1.05 [0.88-1.26]	476	274	142	259	415
W54	1.03 [0.93-1.15]	1.05 [0.94-1.17]	1.11 [0.98-1.26]	0.95 [0.75-1.20]	1.04 [0.93-1.16]	0.99 [0.89-1.10]	0.98 [0.87-1.10]	1.03 [0.91-1.18]	0.94 [0.73-1.19]	1.00 [0.89-1.12]	1,020	882	683	219	914
W55	0.93 [0.80-1.07]	0.95 [0.82-1.10]	0.99 [0.84-1.16]		0.95 [0.82-1.10]	0.86 [0.74-0.99]	0.87 [0.75-1.01]	0.90 [0.76-1.06]		0.91 [0.78-1.05]	530	526	411	NA	485
W57	1.38 [1.22-1.56]	1.34 [1.18-1.52]	1.33 [1.14-1.54]	1.25 [0.88-1.77]	1.33 [1.17-1.51]	1.31 [1.15-1.49]	1.24 [1.09-1.41]	1.24 [1.06-1.44]	1.22 [0.85-1.75]	1.27 [1.12-1.45]	798	765	525	113	723
W64	0.91 [0.69-1.20]	1.05 [0.80-1.38]	1.01 [0.75-1.36]		1.03 [0.79-1.36]	0.90 [0.68-1.19]	0.99 [0.75-1.31]	0.98 [0.72-1.33]		1.04 [0.79-1.38]	156	156	130	NA	154
W78	1.03 [0.90-1.19]	1.04 [0.90-1.19]	1.03 [0.89-1.19]		1.04 [0.90-1.20]	1.03 [0.89-1.18]	1.02 [0.88-1.18]	1.01 [0.87-1.17]		1.06 [0.92-1.22]	602	592	570	NA	587
W79	1.10 [0.98-1.23]	1.15 [1.02-1.29]	1.09 [0.97-1.23]		1.12 [1.00-1.26]	1.08 [0.96-1.21]	1.11 [0.98-1.25]	1.06 [0.93-1.20]		1.11 [0.99-1.25]	892	847	785	NA	864
W80	1.19 [0.99-1.42]	1.15 [0.95-1.38]	1.21 [1.01-1.46]		1.14 [0.95-1.36]	1.14 [0.95-1.37]	1.09 [0.91-1.32]	1.13 [0.94-1.37]		1.10 [0.92-1.32]	370	364	354	NA	359
W84	1.01 [0.84-1.21]	0.98 [0.82-1.18]	1.00 [0.83-1.20]		0.97 [0.81-1.16]	0.99 [0.82-1.19]	0.93 [0.77-1.13]	0.96 [0.80-1.16]		0.97 [0.81-1.17]	339	335	334	NA	340
X10	1.07 [0.90-1.28]	1.15 [0.90-1.48]	1.26 [0.95-1.68]	1.06 [0.84-1.34]	1.01 [0.84-1.22]	1.06 [0.89-1.27]	1.08 [0.84-1.40]	1.19 [0.89-1.59]	1.06 [0.83-1.34]	0.99 [0.83-1.20]	391	192	144	224	354
X11	0.93 [0.67-1.28]					0.91 [0.65-1.26]					111	NA	NA	NA	NA
X12	0.94 [0.75-1.18]	1.03 [0.78-1.35]	1.07 [0.79-1.46]		0.97 [0.76-1.23]	0.92 [0.73-1.16]	0.97 [0.73-1.29]	1.00 [0.73-1.37]		0.93 [0.73-1.19]	236	157	123	NA	202
X15	1.06 [0.80-1.40]				1.01 [0.76-1.35]	1.04 [0.78-1.38]				1.02 [0.76-1.37]	146	NA	NA	NA	144
X16	0.95 [0.72-1.24]	0.95 [0.69-1.29]	0.96 [0.71-1.31]		0.93 [0.70-1.23]	0.93 [0.71-1.22]	0.90 [0.66-1.24]	0.92 [0.68-1.26]		0.91 [0.68-1.20]	156	127	120	NA	140
X23	1.02 [0.77-1.36]	1.06 [0.79-1.42]	1.01 [0.74-1.39]		1.07 [0.79-1.44]	0.97 [0.72-1.30]	1.00 [0.74-1.34]	0.96 [0.69-1.33]		1.00 [0.74-1.36]	148	136	108	NA	122
X31	1.09 [0.83-1.42]	0.92 [0.71-1.20]	0.98 [0.75-1.27]		0.94 [0.72-1.23]	1.10 [0.84-1.44]	0.91 [0.70-1.19]	0.99 [0.76-1.29]		0.98 [0.75-1.28]	171	170	169	NA	161
X40	1.07 [0.98-1.17]	1.06 [0.96-1.17]	1.18 [1.03-1.33]	1.01 [0.85-1.19]	1.01 [0.92-1.10]	1.02 [0.93-1.12]	0.94 [0.85-1.05]	1.02 [0.90-1.17]	1.02 [0.86-1.22]	0.99 [0.90-1.08]	1,430	1,164	705	453	1,340
X41	1.11 [0.99-1.23]	1.15 [1.03-1.29]	1.14 [1.00-1.30]	0.95 [0.73-1.23]	1.03 [0.92-1.14]	1.04 [0.94-1.17]	1.00 [0.89-1.12]	1.01 [0.88-1.16]	0.97 [0.74-1.26]	1.02 [0.91-1.14]	987	922	613	184	955
X42	1.17 [1.05-1.30]	1.17 [1.05-1.30]	1.23 [1.09-1.39]	0.98 [0.70-1.37]	1.10 [0.99-1.22]	1.07 [0.96-1.19]	0.99 [0.89-1.11]	1.07 [0.94-1.21]	0.99 [0.70-1.40]	1.06 [0.95-1.18]	1,058	1,045	793	116	1,065
X43	1.00 [0.80-1.26]	1.04 [0.81-1.32]	0.99 [0.77-1.29]		0.98 [0.78-1.23]	0.98 [0.77-1.23]	0.97 [0.76-1.25]	0.91 [0.70-1.19]		0.97 [0.77-1.22]	222	194	169	NA	212
X44	1.09 [1.00-1.19]	1.11 [1.01-1.23]	1.14 [1.03-1.27]	0.96 [0.79-1.16]	1.09 [1.00-1.19]	1.03 [0.94-1.13]	0.99 [0.89-1.09]	1.02 [0.91-1.13]	0.97 [0.80-1.18]	1.06 [0.97-1.16]	1,565	1,291	1,118	330	1,524
X45	1.16 [0.96-1.40]	1.03 [0.86-1.25]	1.04 [0.80-1.36]		0.97 [0.80-1.17]	1.08 [0.89-1.32]	0.88 [0.72-1.07]	0.90 [0.68-1.19]		0.96 [0.79-1.16]	335	325	155	NA	306
X47	1.03 [0.77-1.38]	0.99 [0.74-1.34]	1.14 [0.82-1.56]		1.01 [0.75-1.35]	1.03 [0.77-1.38]	0.91 [0.67-1.24]	1.11 [0.80-1.53]		0.98 [0.72-1.33]	136	131	117	NA	128
X49	1.24 [1.06-1.45]	1.20 [0.98-1.47]	1.13 [0.90-1.42]	1.41 [1.11-1.78]	1.28 [1.09-1.49]	1.17 [1.00-1.37]	1.07 [0.87-1.32]	1.06 [0.84-1.34]	1.34 [1.05-1.70]	1.20 [1.02-1.41]	527	304	217	248	496
X50	1.07 [0.99-1.16]	1.11 [1.02-1.20]	1.07 [0.98-1.17]	1.08 [0.84-1.37]	1.05 [0.97-1.14]	1.01 [0.93-1.10]	1.03 [0.95-1.12]	1.00 [0.91-1.10]	1.02 [0.79-1.32]	1.01 [0.93-1.09]	1,878	1,798	1,374	227	1,687
X51	1.02 [0.77-1.33]	1.01 [0.77-1.33]	1.07 [0.79-1.44]		1.04 [0.78-1.38]	1.00 [0.76-1.32]	1.00 [0.76-1.31]	1.01 [0.75-1.37]		1.04 [0.78-1.38]	142	146	118	NA	130
X58	1.36 [1.20-1.55]	1.40 [1.22-1.61]	1.25 [1.07-1.45]	2.16 [1.53-3.05]	1.29 [1.13-1.47]	1.26 [1.10-1.44]	1.29 [1.12-1.49]	1.17 [1.00-1.36]	1.91 [1.32-2.76]	1.23 [1.08-1.41]	773	679	542	152	736
X59	1.18 [1.14-1.22]	1.18 [1.14-1.23]	1.20 [1.15-1.25]	1.06 [0.95-1.18]	1.16 [1.12-1.20]	1.13 [1.09-1.18]	1.11 [1.07-1.15]	1.13 [1.09-1.18]	1.04 [0.93-1.17]	1.13 [1.09-1.18]	9,634	8,773	7,825	1,161	8,935
X60	1.10 [1.05-1.16]	1.07 [1.01-1.13]	1.22 [1.13-1.32]	0.93 [0.84-1.04]	1.05 [0.99-1.11]	1.05 [0.99-1.11]	0.94 [0.89-1.00]	1.08 [1.00-1.17]	0.94 [0.84-1.06]	1.04 [0.98-1.10]	4,252	4,065	1,812	1,141	3,773
X61	1.15 [1.08-1.21]	1.14 [1.08-1.20]	1.23 [1.13-1.33]	0.91 [0.79-1.04]	1.07 [1.01-1.13]	1.07 [1.01-1.14]	0.98 [0.93-1.04]	1.06 [0.98-1.15]	0.95 [0.82-1.11]	1.06 [1.00-1.13]	3,720	3,786	1,811	657	3,405
X62	1.17 [1.08-1.26]	1.14 [1.06-1.23]	1.19 [1.07-1.32]	0.99 [0.82-1.20]	1.07 [1.00-1.16]	1.06 [0.98-1.15]	0.96 [0.89-1.04]	1.00 [0.89-1.12]	1.01 [0.83-1.23]	1.04 [0.97-1.13]	2,099	2,140	1,029	360	2,013
X63	1.12 [0.97-1.31]	1.12 [0.97-1.30]	1.16 [0.94-1.43]		1.07 [0.93-1.24]	1.07 [0.92-1.25]	0.97 [0.84-1.14]	1.01 [0.81-1.25]		1.08 [0.93-1.26]	530	542	265	NA	517
X64	1.18 [1.08-1.28]	1.11 [1.03-1.21]	1.26 [1.13-1.41]	1.05 [0.87-1.26]	1.10 [1.01-1.19]	1.09 [1.00-1.19]	0.96 [0.88-1.04]	1.09 [0.97-1.22]	1.01 [0.83-1.23]	1.08 [1.00-1.18]	1,809	1,700	930	400	1,675
X65	1.12 [1.05-1.21]	1.08 [1.01-1.16]	1.21 [1.10-1.33]	0.95 [0.79-1.13]	1.07 [1.00-1.15]	1.05 [0.98-1.13]	0.93 [0.87-1.00]	1.02 [0.92-1.13]	1.06 [0.88-1.29]	1.07 [0.99-1.15]	2,511	2,604	1,232	382	2,353
X69	0.93 [0.71-1.23]	0.78 [0.58-1.05]			0.92 [0.69-1.22]	0.88 [0.67-1.18]	0.68 [0.50-0.93]			0.94 [0.70-1.25]	155	118	NA	NA	140
X70	1.01 [0.72-1.40]				0.92 [0.67-1.28]	1.01 [0.71-1.42]				0.95 [0.67-1.33]	106	NA	NA	NA	104
X78	1.08 [0.97-1.21]	1.08 [0.96-1.21]	1.18 [0.99-1.41]	0.98 [0.80-1.20]	1.06 [0.95-1.19]	0.97 [0.86-1.10]	0.90 [0.80-1.02]	0.99 [0.83-1.20]	0.99 [0.80-1.23]	1.02 [0.91-1.15]	966	850	367	317	881
X84	1.06 [0.79-1.42]	0.98 [0.73-1.32]			1.10 [0.83-1.47]	0.95 [0.69-1.29]	0.83 [0.60-1.13]			1.03 [0.76-1.40]	147	127	NA	NA	143
X99	1.13 [0.91-1.40]	1.02 [0.81-1.29]			0.97 [0.76-1.22]	1.11 [0.89-1.39]	0.97 [0.76-1.23]			0.97 [0.76-1.24]	281	228	NA	NA	210
Y00	1.00 [0.77-1.30]	0.99 [0.76-1.30]			0.92 [0.70-1.20]	0.92 [0.70-1.21]	0.90 [0.68-1.19]			0.89 [0.67-1.18]	169	159	NA	NA	152
Y04	1.06 [0.96-1.16]	1.05 [0.96-1.16]	1.15 [1.00-1.32]	0.91 [0.75-1.10]	1.02 [0.93-1.13]	1.01 [0.92-1.12]	0.96 [0.86-1.06]	1.04 [0.90-1.20]	0.98 [0.80-1.21]	1.02 [0.92-1.13]	1,401	1,331	584	345	1,207
Y07	0.94 [0.69-1.28]				0.88 [0.66-1.18]	0.88 [0.64-1.20]				0.87 [0.64-1.17]	131	NA	NA	NA	120
Y09	1.09 [0.86-1.38]	1.01 [0.80-1.29]	1.17 [0.84-1.63]		1.09 [0.85-1.39]	1.04 [0.82-1.32]	0.92 [0.72-1.18]	1.05 [0.74-1.47]		1.07 [0.83-1.36]	219	201	109	NA	190
Y40	1.43 [1.35-1.52]	1.41 [1.33-1.50]	1.44 [1.35-1.54]	1.09 [0.84-1.42]	1.36 [1.28-1.44]	1.33 [1.25-1.42]	1.28 [1.21-1.37]	1.32 [1.23-1.41]	1.09 [0.83-1.43]	1.29 [1.22-1.37]	3,502	3,418	3,069	191	3,384
Y41	1.30 [1.14-1.48]	1.27 [1.11-1.45]	1.32 [1.14-1.51]		1.21 [1.06-1.38]	1.20 [1.05-1.37]	1.16 [1.01-1.33]	1.19 [1.03-1.37]		1.13 [0.99-1.30]	700	692	604	NA	662
Y42	1.27 [1.19-1.36]	1.23 [1.15-1.31]	1.24 [1.16-1.33]		1.22 [1.14-1.30]	1.15 [1.07-1.23]	1.08 [1.01-1.15]	1.09 [1.02-1.17]		1.13 [1.06-1.21]	2,822	2,793	2,555	NA	2,773
Y43	1.13 [1.08-1.19]	1.14 [1.08-1.19]	1.14 [1.09-1.20]	1.13 [0.85-1.49]	1.09 [1.04-1.15]	1.11 [1.06-1.17]	1.11 [1.05-1.17]	1.12 [1.06-1.17]	1.14 [0.86-1.53]	1.09 [1.04-1.15]	4,678	4,587	4,253	173	4,330
Y44	1.17 [1.08-1.27]	1.19 [1.10-1.28]	1.16 [1.07-1.26]		1.13 [1.05-1.23]	1.13 [1.05-1.22]	1.12 [1.03-1.21]	1.09 [1.01-1.18]		1.10 [1.02-1.19]	1,923	1,922	1,854	NA	1,879
Y45	1.24 [1.18-1.31]	1.24 [1.18-1.31]	1.24 [1.17-1.31]	1.15 [0.80-1.67]	1.19 [1.13-1.25]	1.18 [1.11-1.24]	1.14 [1.08-1.20]	1.15 [1.08-1.21]	1.08 [0.72-1.61]	1.15 [1.09-1.21]	4,335	4,354	3,954	104	4,160
Y46	1.27 [1.11-1.46]	1.24 [1.07-1.42]	1.27 [1.10-1.46]		1.20 [1.05-1.38]	1.20 [1.04-1.38]	1.14 [0.99-1.32]	1.17 [1.01-1.35]		1.17 [1.02-1.35]	617	589	572	NA	608
Y47	1.16 [0.94-1.41]	1.34 [1.09-1.65]	1.28 [1.03-1.58]		1.18 [0.96-1.44]	1.08 [0.88-1.33]	1.25 [1.01-1.54]	1.21 [0.97-1.50]		1.15 [0.94-1.41]	296	287	256	NA	289
Y48	1.07 [0.88-1.30]	1.15 [0.94-1.41]	1.11 [0.89-1.39]		1.06 [0.87-1.28]	1.00 [0.82-1.22]	1.06 [0.86-1.30]	1.02 [0.82-1.28]		1.03 [0.84-1.25]	294	289	230	NA	287
Y49	1.25 [1.13-1.39]	1.30 [1.18-1.45]	1.25 [1.12-1.39]		1.27 [1.14-1.40]	1.23 [1.10-1.36]	1.23 [1.11-1.37]	1.19 [1.07-1.33]		1.26 [1.13-1.40]	1,081	1,086	990	NA	1,047
Y51	1.11 [1.02-1.21]	1.15 [1.06-1.25]	1.13 [1.04-1.23]		1.11 [1.02-1.21]	1.10 [1.01-1.19]	1.11 [1.02-1.21]	1.09 [1.00-1.19]		1.12 [1.03-1.21]	1,663	1,665	1,630	NA	1,611
Y52	1.20 [1.14-1.27]	1.24 [1.17-1.30]	1.19 [1.13-1.25]		1.18 [1.12-1.25]	1.16 [1.10-1.22]	1.16 [1.10-1.22]	1.12 [1.06-1.18]		1.16 [1.10-1.22]	4,300	4,307	4,266	NA	4,218
Y53	1.32 [1.17-1.49]	1.28 [1.13-1.44]	1.32 [1.16-1.49]		1.20 [1.06-1.36]	1.23 [1.08-1.39]	1.14 [1.01-1.30]	1.20 [1.06-1.37]		1.14 [1.00-1.29]	816	818	782	NA	794
Y54	1.23 [1.16-1.30]	1.26 [1.18-1.33]	1.25 [1.18-1.33]		1.22 [1.15-1.29]	1.18 [1.11-1.25]	1.17 [1.10-1.24]	1.17 [1.10-1.24]		1.18 [1.11-1.25]	3,479	3,486	3,452	NA	3,355
Y55	1.26 [1.05-1.50]	1.20 [1.01-1.44]	1.43 [1.12-1.83]		1.17 [0.98-1.40]	1.12 [0.94-1.35]	1.05 [0.87-1.26]	1.21 [0.93-1.57]		1.03 [0.86-1.25]	396	394	190	NA	356
Y56	1.67 [1.38-2.01]	1.63 [1.35-1.96]	1.70 [1.39-2.09]		1.58 [1.31-1.90]	1.53 [1.26-1.85]	1.47 [1.21-1.78]	1.51 [1.22-1.86]		1.49 [1.23-1.80]	382	372	320	NA	355
Y57	1.24 [1.15-1.35]	1.23 [1.13-1.33]	1.25 [1.15-1.36]		1.23 [1.14-1.34]	1.16 [1.07-1.26]	1.12 [1.03-1.22]	1.14 [1.05-1.24]		1.18 [1.09-1.28]	1,862	1,861	1,749	NA	1,827
Y59	1.28 [0.98-1.66]	1.18 [0.89-1.57]	1.25 [0.92-1.70]		1.37 [1.05-1.79]	1.20 [0.92-1.58]	1.09 [0.81-1.46]	1.14 [0.83-1.57]		1.27 [0.96-1.66]	185	142	121	NA	172
Y60	1.15 [1.06-1.24]	1.16 [1.07-1.25]	1.20 [1.11-1.30]		1.15 [1.07-1.24]	1.11 [1.02-1.19]	1.09 [1.01-1.18]	1.14 [1.05-1.24]		1.12 [1.04-1.21]	2,050	2,048	1,848	NA	1,931
Y65	1.06 [0.86-1.31]	1.08 [0.87-1.34]	1.13 [0.91-1.41]		1.04 [0.84-1.29]	1.02 [0.82-1.26]	1.01 [0.81-1.26]	1.06 [0.85-1.33]		1.03 [0.83-1.28]	256	251	229	NA	246
Y71	1.32 [0.99-1.77]	1.31 [0.98-1.75]	1.37 [1.02-1.85]		1.28 [0.96-1.71]	1.29 [0.96-1.73]	1.23 [0.92-1.65]	1.31 [0.97-1.77]		1.25 [0.93-1.68]	139	140	130	NA	136
Y77	1.00 [0.74-1.36]	0.96 [0.71-1.30]	0.94 [0.70-1.26]		1.07 [0.79-1.44]	0.99 [0.73-1.34]	0.91 [0.67-1.23]	0.91 [0.68-1.23]		1.04 [0.77-1.41]	121	118	118	NA	117
Y79	1.03 [0.84-1.26]	1.07 [0.87-1.31]	1.04 [0.85-1.28]		1.06 [0.86-1.30]	0.99 [0.80-1.21]	1.01 [0.82-1.24]	1.00 [0.81-1.23]		1.01 [0.82-1.25]	260	258	254	NA	254
Y82	1.27 [0.91-1.78]				1.34 [0.97-1.84]	1.23 [0.88-1.72]				1.32 [0.96-1.83]	101	NA	NA	NA	106
Y83	1.17 [1.15-1.19]	1.17 [1.15-1.19]	1.16 [1.13-1.18]	1.12 [1.03-1.22]	1.13 [1.11-1.15]	1.12 [1.10-1.14]	1.10 [1.07-1.12]	1.09 [1.07-1.11]	1.11 [1.02-1.22]	1.10 [1.08-1.12]	34,875	34,170	29,806	1,936	33,031
Y84	1.14 [1.09-1.18]	1.15 [1.11-1.19]	1.13 [1.08-1.17]	0.98 [0.75-1.27]	1.10 [1.06-1.15]	1.10 [1.06-1.15]	1.09 [1.05-1.13]	1.06 [1.02-1.11]	0.95 [0.72-1.25]	1.09 [1.05-1.13]	8,348	8,265	7,747	205	8,111
Y85	1.17 [1.03-1.34]	1.22 [1.07-1.39]	1.26 [1.08-1.46]		1.08 [0.95-1.24]	1.11 [0.97-1.27]	1.11 [0.97-1.27]	1.17 [1.00-1.37]		1.04 [0.91-1.19]	682	677	520	NA	623
Y86	1.12 [1.01-1.24]	1.12 [1.01-1.24]	1.13 [1.01-1.27]	1.20 [0.88-1.65]	1.09 [0.99-1.21]	1.08 [0.98-1.20]	1.04 [0.94-1.16]	1.07 [0.95-1.20]	1.19 [0.85-1.65]	1.06 [0.95-1.17]	1,172	1,110	841	141	1,073
Y87	1.27 [0.97-1.65]	1.16 [0.89-1.52]			1.12 [0.85-1.46]	1.19 [0.90-1.56]	1.01 [0.76-1.34]			1.07 [0.80-1.42]	178	175	NA	NA	162
Y88	1.01 [0.82-1.23]	1.12 [0.91-1.38]	1.09 [0.88-1.35]		0.92 [0.76-1.12]	0.94 [0.76-1.16]	1.01 [0.82-1.25]	0.95 [0.76-1.19]		0.86 [0.70-1.06]	265	265	232	NA	259
Y90	1.25 [0.92-1.70]	1.29 [0.96-1.73]			1.01 [0.76-1.34]	1.28 [0.93-1.74]	1.24 [0.91-1.67]			1.07 [0.80-1.43]	130	137	NA	NA	129
Y91	1.11 [0.88-1.39]	0.97 [0.77-1.21]	1.12 [0.87-1.45]		1.08 [0.86-1.35]	1.06 [0.84-1.34]	0.90 [0.71-1.13]	1.01 [0.77-1.31]		1.08 [0.86-1.36]	219	219	164	NA	204
Y95	1.17 [1.14-1.20]	1.16 [1.13-1.19]	1.17 [1.14-1.20]		1.15 [1.13-1.18]	1.12 [1.10-1.15]	1.09 [1.06-1.12]	1.09 [1.06-1.12]		1.13 [1.10-1.15]	21,092	21,111	20,890	NA	21,025
Factors influencing health status and contact with health services (Z00-Z99)															
Z00	1.12 [0.99-1.28]	1.11 [0.97-1.27]	1.18 [1.01-1.37]		1.06 [0.93-1.22]	1.06 [0.93-1.22]	1.05 [0.91-1.20]	1.11 [0.95-1.30]		1.04 [0.90-1.19]	653	636	488	NA	572
Z01	1.97 [1.86-2.09]	1.23 [1.14-1.33]	1.28 [1.17-1.40]	3.98 [3.62-4.38]	1.92 [1.81-2.04]	1.72 [1.62-1.83]	1.11 [1.02-1.20]	1.18 [1.08-1.30]	3.14 [2.83-3.47]	1.67 [1.57-1.77]	4,661	2,018	1,434	2,782	4,087
Z02	1.09 [1.02-1.17]	1.09 [1.02-1.17]	1.12 [1.04-1.20]		1.08 [1.00-1.15]	1.05 [0.98-1.13]	1.03 [0.96-1.10]	1.06 [0.99-1.15]		1.05 [0.98-1.13]	2,679	2,719	2,137	NA	2,456
Z03	1.23 [1.20-1.25]	1.22 [1.19-1.25]	1.22 [1.19-1.25]	1.35 [1.25-1.45]	1.20 [1.17-1.22]	1.16 [1.13-1.19]	1.13 [1.10-1.15]	1.13 [1.10-1.16]	1.27 [1.17-1.37]	1.15 [1.12-1.17]	22,897	21,255	18,261	2,474	21,583
Z04	1.14 [1.07-1.21]	1.16 [1.09-1.23]	1.21 [1.12-1.30]	1.02 [0.88-1.18]	1.12 [1.05-1.19]	1.10 [1.04-1.17]	1.08 [1.02-1.15]	1.15 [1.07-1.24]	1.02 [0.88-1.19]	1.09 [1.03-1.16]	3,339	3,015	2,076	561	2,990
Z08	1.01 [0.97-1.06]	1.01 [0.96-1.05]	1.01 [0.97-1.05]		1.01 [0.97-1.06]	1.01 [0.96-1.05]	0.99 [0.95-1.03]	0.99 [0.95-1.04]		1.01 [0.97-1.06]	6,074	5,996	5,867	NA	5,865
Z09	1.17 [1.13-1.20]	1.16 [1.13-1.20]	1.17 [1.13-1.21]	1.09 [0.95-1.26]	1.13 [1.10-1.16]	1.11 [1.07-1.14]	1.08 [1.05-1.11]	1.09 [1.05-1.13]	1.05 [0.90-1.22]	1.09 [1.06-1.12]	12,760	12,375	11,061	645	12,274
Z11	1.12 [1.10-1.14]	1.11 [1.09-1.13]	1.13 [1.11-1.16]	1.05 [1.00-1.11]	1.10 [1.08-1.12]	1.07 [1.05-1.09]	1.03 [1.02-1.05]	1.05 [1.03-1.07]	1.05 [0.99-1.11]	1.08 [1.06-1.10]	40,378	39,851	30,858	4,427	39,570
Z12	1.08 [1.04-1.12]	1.07 [1.03-1.12]	1.07 [1.03-1.11]		1.06 [1.02-1.11]	1.06 [1.02-1.10]	1.06 [1.02-1.10]	1.05 [1.01-1.09]		1.04 [1.00-1.09]	7,780	7,718	7,479	NA	6,871
Z13	1.16 [1.14-1.19]	1.16 [1.13-1.18]	1.16 [1.13-1.19]	1.31 [1.21-1.43]	1.13 [1.10-1.15]	1.11 [1.09-1.14]	1.09 [1.06-1.11]	1.09 [1.06-1.11]	1.26 [1.15-1.37]	1.09 [1.07-1.12]	24,529	23,895	18,218	2,161	22,043
Z20	1.15 [1.05-1.26]	1.13 [1.03-1.25]	1.12 [1.01-1.24]	1.20 [0.89-1.63]	1.12 [1.02-1.23]	1.10 [1.00-1.21]	1.05 [0.96-1.16]	1.03 [0.93-1.15]	1.19 [0.87-1.64]	1.08 [0.99-1.19]	1,470	1,397	1,211	144	1,420
Z22	1.27 [1.24-1.31]	1.28 [1.24-1.32]	1.33 [1.29-1.39]	0.93 [0.84-1.04]	1.25 [1.21-1.28]	1.23 [1.19-1.26]	1.21 [1.17-1.24]	1.24 [1.20-1.29]	0.94 [0.83-1.06]	1.22 [1.18-1.25]	14,010	14,384	8,745	1,011	13,522
Z24	0.99 [0.77-1.27]	1.09 [0.87-1.38]			0.95 [0.73-1.23]	1.03 [0.80-1.34]	1.10 [0.87-1.39]			0.95 [0.73-1.25]	183	216	NA	NA	161
Z25	2.61 [1.80-3.78]			3.84 [2.29-6.41]	1.94 [1.36-2.76]	2.29 [1.56-3.37]			2.95 [1.70-5.12]	1.72 [1.19-2.49]	141	NA	NA	103	128
Z27	1.85 [1.61-2.12]	0.99 [0.85-1.17]		3.29 [2.68-4.03]	1.66 [1.44-1.92]	1.73 [1.50-2.00]	0.99 [0.84-1.17]		2.78 [2.24-3.44]	1.56 [1.34-1.81]	725	439	NA	470	626
Z29	1.15 [1.11-1.20]	1.15 [1.11-1.20]	1.18 [1.12-1.24]	1.00 [0.87-1.16]	1.15 [1.10-1.20]	1.11 [1.07-1.16]	1.09 [1.04-1.14]	1.10 [1.05-1.16]	1.02 [0.87-1.18]	1.13 [1.08-1.17]	6,989	6,965	4,751	618	6,741
Z30	1.08 [1.03-1.13]	1.10 [1.05-1.14]	1.13 [1.05-1.22]	0.86 [0.72-1.04]	1.01 [0.97-1.06]	1.05 [1.01-1.10]	1.04 [1.00-1.09]	1.07 [0.99-1.16]	0.89 [0.73-1.08]	1.01 [0.96-1.06]	6,248	6,431	1,870	366	5,440
Z32	1.16 [1.00-1.35]	1.14 [0.99-1.32]			1.06 [0.91-1.23]	1.13 [0.97-1.32]	1.10 [0.95-1.28]			1.06 [0.90-1.23]	516	574	NA	NA	470
Z33	1.15 [1.10-1.21]	1.17 [1.12-1.23]	1.13 [0.88-1.43]	0.96 [0.86-1.08]	1.11 [1.06-1.17]	1.09 [1.04-1.15]	1.07 [1.02-1.13]	1.09 [0.85-1.40]	0.97 [0.86-1.09]	1.08 [1.02-1.13]	4,967	5,590	185	944	4,635
Z34	1.00 [0.97-1.03]	1.03 [1.00-1.06]	0.90 [0.76-1.06]	0.91 [0.84-0.98]	0.99 [0.96-1.03]	0.99 [0.96-1.03]	1.01 [0.98-1.04]	0.94 [0.79-1.12]	0.92 [0.85-0.99]	0.99 [0.96-1.03]	12,482	13,959	362	2,327	10,399
Z35	0.98 [0.95-1.02]	0.99 [0.96-1.03]	0.86 [0.78-0.95]	0.85 [0.78-0.92]	0.98 [0.95-1.01]	0.98 [0.95-1.02]	0.99 [0.96-1.02]	0.91 [0.82-1.00]	0.86 [0.79-0.95]	0.99 [0.95-1.02]	11,651	12,347	1,065	1,587	10,964
Z36	1.03 [0.96-1.11]	1.02 [0.95-1.09]	0.87 [0.64-1.18]	0.82 [0.65-1.04]	1.02 [0.94-1.11]	1.03 [0.96-1.11]	1.02 [0.95-1.09]	0.93 [0.68-1.28]	0.84 [0.66-1.07]	1.02 [0.94-1.11]	2,276	2,369	102	220	1,690
Z37	0.96 [0.94-0.98]	0.99 [0.97-1.00]	0.93 [0.84-1.03]	0.86 [0.83-0.90]	0.95 [0.93-0.98]	0.98 [0.96-1.00]	0.99 [0.97-1.01]	1.04 [0.94-1.16]	0.89 [0.86-0.93]	0.97 [0.95-1.00]	41,764	48,042	1,140	8,899	22,630
Z38	0.91 [0.68-1.22]	1.17 [0.88-1.55]			1.06 [0.78-1.44]	0.91 [0.68-1.24]	1.22 [0.91-1.64]			1.03 [0.75-1.41]	124	140	NA	NA	110
Z39	0.99 [0.93-1.04]	0.99 [0.94-1.04]	1.00 [0.78-1.27]	1.00 [0.86-1.16]	1.00 [0.95-1.07]	1.00 [0.94-1.05]	1.00 [0.94-1.05]	1.06 [0.82-1.37]	1.02 [0.87-1.19]	1.00 [0.94-1.07]	3,837	4,159	173	583	3,048
Z40	1.03 [0.90-1.18]	0.98 [0.86-1.12]	1.05 [0.91-1.22]		0.93 [0.82-1.06]	1.03 [0.90-1.18]	0.94 [0.82-1.08]	1.01 [0.87-1.18]		0.94 [0.82-1.08]	644	633	491	NA	604
Z41	1.16 [1.02-1.32]	1.14 [0.99-1.31]	1.17 [0.98-1.40]	1.23 [0.90-1.67]	1.14 [1.00-1.29]	1.11 [0.97-1.27]	1.07 [0.93-1.23]	1.08 [0.90-1.30]	1.22 [0.89-1.67]	1.14 [0.99-1.30]	691	605	345	150	643
Z42	1.09 [1.01-1.18]	1.11 [1.03-1.20]	1.09 [1.00-1.18]		1.08 [1.00-1.17]	1.08 [0.99-1.17]	1.06 [0.98-1.15]	1.05 [0.96-1.14]		1.09 [1.01-1.18]	1,845	1,833	1,503	NA	1,767
Z43	1.08 [1.02-1.15]	1.14 [1.07-1.22]	1.12 [1.05-1.19]	0.79 [0.61-1.01]	1.08 [1.01-1.14]	1.07 [1.01-1.14]	1.11 [1.04-1.19]	1.09 [1.02-1.16]	0.75 [0.58-0.97]	1.08 [1.02-1.15]	2,884	2,747	2,509	191	2,782
Z45	1.13 [1.09-1.18]	1.14 [1.10-1.19]	1.12 [1.07-1.16]	1.01 [0.82-1.25]	1.08 [1.04-1.12]	1.10 [1.05-1.14]	1.08 [1.04-1.13]	1.05 [1.01-1.10]	1.00 [0.81-1.24]	1.06 [1.02-1.11]	6,871	6,695	6,183	301	6,645
Z46	1.06 [1.02-1.10]	1.06 [1.02-1.10]	1.06 [1.01-1.10]	1.05 [0.83-1.35]	1.03 [0.99-1.07]	1.04 [1.00-1.08]	1.03 [0.99-1.07]	1.03 [0.99-1.07]	1.00 [0.78-1.29]	1.02 [0.98-1.06]	7,883	7,782	7,237	238	7,350
Z47	1.12 [1.06-1.18]	1.15 [1.08-1.22]	1.15 [1.07-1.23]	1.03 [0.91-1.16]	1.12 [1.06-1.19]	1.08 [1.02-1.14]	1.08 [1.01-1.15]	1.10 [1.03-1.18]	1.02 [0.90-1.15]	1.09 [1.03-1.16]	3,808	3,024	2,258	1,008	3,426
Z48	1.13 [1.05-1.22]	1.15 [1.06-1.24]	1.11 [1.02-1.21]	1.11 [0.91-1.36]	1.08 [1.00-1.17]	1.10 [1.02-1.19]	1.11 [1.02-1.20]	1.07 [0.98-1.17]	1.09 [0.89-1.34]	1.07 [0.99-1.15]	1,972	1,715	1,445	322	1,763
Z49	1.27 [1.17-1.38]	1.27 [1.17-1.38]	1.23 [1.13-1.33]		1.17 [1.08-1.27]	1.26 [1.16-1.37]	1.21 [1.12-1.32]	1.16 [1.06-1.26]		1.19 [1.09-1.29]	1,674	1,681	1,583	NA	1,635
Z50	1.11 [1.09-1.13]	1.12 [1.10-1.13]	1.11 [1.10-1.13]	0.98 [0.87-1.10]	1.10 [1.09-1.12]	1.08 [1.07-1.10]	1.07 [1.05-1.08]	1.06 [1.05-1.08]	0.95 [0.84-1.08]	1.09 [1.07-1.10]	53,539	53,191	51,299	958	51,059
Z51	1.14 [1.12-1.15]	1.13 [1.12-1.15]	1.13 [1.11-1.14]	1.14 [1.03-1.26]	1.11 [1.10-1.13]	1.11 [1.09-1.12]	1.08 [1.07-1.10]	1.08 [1.06-1.09]	1.11 [1.01-1.23]	1.09 [1.08-1.11]	61,301	60,872	56,491	1,396	58,085
Z52	0.90 [0.71-1.15]	0.92 [0.72-1.17]	0.89 [0.67-1.18]		0.88 [0.68-1.14]	0.90 [0.70-1.15]	0.92 [0.72-1.18]	0.91 [0.68-1.22]		0.89 [0.68-1.16]	188	186	127	NA	151
Z53	1.21 [1.19-1.23]	1.20 [1.18-1.22]	1.20 [1.18-1.22]	1.19 [1.12-1.27]	1.16 [1.14-1.18]	1.15 [1.13-1.17]	1.12 [1.10-1.13]	1.12 [1.11-1.14]	1.15 [1.08-1.23]	1.12 [1.11-1.14]	50,321	48,556	42,356	3,302	46,665
Z54	1.32 [1.13-1.55]	1.24 [1.06-1.45]	1.24 [1.06-1.45]		1.27 [1.07-1.50]	1.29 [1.10-1.51]	1.20 [1.03-1.41]	1.19 [1.01-1.40]		1.26 [1.06-1.49]	465	467	457	NA	398
Z56	1.14 [0.97-1.33]	1.13 [0.97-1.32]	1.08 [0.89-1.32]		1.06 [0.91-1.25]	1.11 [0.94-1.31]	1.00 [0.85-1.18]	1.01 [0.83-1.24]		1.02 [0.86-1.20]	486	497	291	NA	449
Z57	1.20 [1.12-1.30]	1.21 [1.12-1.31]	1.23 [1.14-1.32]		1.18 [1.10-1.28]	1.16 [1.08-1.25]	1.15 [1.06-1.24]	1.16 [1.07-1.25]		1.14 [1.05-1.23]	1,984	1,989	1,956	NA	1,884
Z58	1.17 [1.06-1.29]	1.12 [1.01-1.24]	1.16 [1.04-1.29]	1.52 [1.17-1.97]	1.16 [1.06-1.28]	1.10 [1.00-1.21]	1.02 [0.92-1.14]	1.05 [0.93-1.17]	1.53 [1.16-2.00]	1.09 [0.99-1.20]	1,301	1,149	987	214	1,258
Z59	1.08 [0.97-1.19]	1.04 [0.94-1.15]	1.06 [0.94-1.19]	1.18 [0.85-1.66]	1.03 [0.93-1.14]	1.05 [0.94-1.16]	0.98 [0.88-1.09]	0.99 [0.88-1.12]	1.27 [0.88-1.82]	1.05 [0.95-1.17]	1,145	1,128	824	118	1,092
Z60	1.12 [1.09-1.15]	1.12 [1.09-1.15]	1.12 [1.09-1.15]	0.62 [0.44-0.86]	1.10 [1.07-1.13]	1.10 [1.07-1.13]	1.07 [1.04-1.10]	1.07 [1.04-1.10]	0.63 [0.44-0.91]	1.08 [1.05-1.11]	16,880	16,832	16,418	103	15,460
Z61	1.11 [0.93-1.33]	1.02 [0.84-1.23]	1.36 [1.02-1.81]	0.86 [0.64-1.16]	0.94 [0.79-1.13]	1.07 [0.89-1.28]	0.88 [0.72-1.08]	1.17 [0.87-1.58]	0.87 [0.64-1.19]	0.95 [0.79-1.14]	385	312	145	146	354
Z63	1.11 [1.05-1.17]	1.09 [1.04-1.16]	1.13 [1.06-1.21]	0.85 [0.73-0.98]	1.07 [1.01-1.12]	1.04 [0.99-1.10]	0.99 [0.93-1.04]	1.03 [0.96-1.10]	0.88 [0.76-1.02]	1.04 [0.99-1.10]	4,178	3,937	2,867	628	3,969
Z65	1.03 [0.85-1.26]	1.12 [0.91-1.37]	1.06 [0.81-1.39]		0.98 [0.81-1.19]	1.01 [0.82-1.24]	0.96 [0.77-1.19]	0.96 [0.72-1.28]		0.99 [0.81-1.21]	303	282	155	NA	296
Z71	1.29 [1.23-1.36]	1.09 [1.03-1.15]	1.06 [1.00-1.12]	3.28 [2.89-3.72]	1.24 [1.18-1.31]	1.22 [1.16-1.29]	1.03 [0.97-1.09]	1.01 [0.95-1.07]	2.65 [2.32-3.03]	1.19 [1.13-1.26]	5,302	3,984	3,453	1,481	4,947
Z72	1.13 [1.11-1.15]	1.13 [1.11-1.15]	1.14 [1.12-1.17]	0.98 [0.89-1.08]	1.09 [1.07-1.12]	1.11 [1.09-1.13]	1.05 [1.03-1.07]	1.08 [1.05-1.10]	1.02 [0.93-1.13]	1.10 [1.08-1.13]	28,055	28,553	21,098	1,459	23,916
Z73	1.11 [1.07-1.16]	1.11 [1.07-1.15]	1.11 [1.07-1.16]		1.08 [1.04-1.12]	1.08 [1.04-1.13]	1.05 [1.01-1.09]	1.06 [1.02-1.10]		1.07 [1.03-1.11]	9,071	9,107	8,747	NA	8,793
Z74	1.13 [1.08-1.17]	1.11 [1.06-1.15]	1.11 [1.07-1.16]		1.10 [1.06-1.15]	1.11 [1.06-1.16]	1.08 [1.03-1.12]	1.08 [1.03-1.12]		1.10 [1.06-1.15]	6,919	6,929	6,848	NA	6,546
Z75	1.09 [1.06-1.12]	1.08 [1.05-1.11]	1.09 [1.05-1.12]		1.08 [1.05-1.11]	1.08 [1.04-1.11]	1.05 [1.02-1.08]	1.05 [1.02-1.09]		1.08 [1.05-1.12]	13,694	13,705	13,520	NA	13,300
Z76	1.16 [1.04-1.30]	1.09 [0.97-1.22]	1.29 [1.04-1.59]	1.04 [0.76-1.43]	1.13 [1.00-1.27]	1.17 [1.04-1.32]	1.09 [0.97-1.23]	1.25 [1.01-1.55]	1.00 [0.71-1.41]	1.13 [1.00-1.28]	897	896	283	127	775
Z80	1.09 [1.06-1.12]	1.09 [1.06-1.13]	1.08 [1.05-1.12]	0.96 [0.79-1.16]	1.05 [1.02-1.09]	1.05 [1.02-1.08]	1.04 [1.01-1.07]	1.03 [1.00-1.07]	0.94 [0.76-1.15]	1.02 [0.99-1.06]	12,119	12,203	10,395	341	10,864
Z81	1.11 [1.01-1.22]	1.06 [0.96-1.16]	1.32 [1.12-1.57]	1.10 [0.95-1.28]	1.04 [0.95-1.14]	1.05 [0.96-1.16]	0.93 [0.84-1.03]	1.19 [1.00-1.42]	1.08 [0.92-1.26]	1.02 [0.93-1.12]	1,492	1,220	391	614	1,416
Z82	1.16 [1.14-1.18]	1.11 [1.09-1.13]	1.13 [1.10-1.15]	1.60 [1.51-1.71]	1.12 [1.10-1.15]	1.11 [1.09-1.14]	1.04 [1.02-1.06]	1.05 [1.03-1.08]	1.57 [1.47-1.68]	1.09 [1.07-1.12]	27,494	25,100	19,859	3,863	25,152
Z83	1.12 [1.08-1.16]	1.09 [1.05-1.13]	1.12 [1.07-1.18]	1.15 [1.06-1.26]	1.09 [1.05-1.13]	1.08 [1.04-1.12]	1.04 [1.00-1.08]	1.05 [1.00-1.11]	1.13 [1.03-1.24]	1.06 [1.02-1.10]	9,665	9,145	4,517	1,816	8,861
Z84	1.53 [1.38-1.69]	1.12 [0.99-1.27]	1.22 [1.00-1.49]	1.86 [1.61-2.14]	1.41 [1.28-1.56]	1.52 [1.37-1.69]	1.09 [0.95-1.24]	1.17 [0.95-1.43]	1.86 [1.60-2.16]	1.40 [1.26-1.55]	1,369	755	300	758	1,296
Z85	1.10 [1.08-1.12]	1.10 [1.08-1.12]	1.11 [1.09-1.12]	0.97 [0.79-1.19]	1.07 [1.05-1.09]	1.08 [1.06-1.09]	1.06 [1.04-1.07]	1.06 [1.05-1.08]	1.00 [0.80-1.23]	1.07 [1.05-1.08]	49,392	49,283	47,881	296	47,097
Z86	1.16 [1.15-1.17]	1.16 [1.15-1.17]	1.17 [1.16-1.18]	0.98 [0.94-1.03]	1.12 [1.11-1.13]	1.12 [1.11-1.13]	1.08 [1.07-1.09]	1.09 [1.08-1.10]	1.02 [0.97-1.07]	1.10 [1.09-1.11]	160,334	161,425	136,870	6,238	149,123
Z87	1.17 [1.16-1.19]	1.17 [1.16-1.19]	1.18 [1.16-1.19]	1.16 [1.11-1.21]	1.13 [1.12-1.15]	1.11 [1.09-1.12]	1.08 [1.07-1.10]	1.09 [1.07-1.11]	1.12 [1.07-1.17]	1.09 [1.08-1.10]	79,896	76,878	61,747	7,339	77,310
Z88	1.33 [1.31-1.34]	1.32 [1.30-1.34]	1.32 [1.31-1.34]	1.30 [1.23-1.37]	1.30 [1.28-1.32]	1.22 [1.21-1.24]	1.19 [1.18-1.21]	1.20 [1.19-1.22]	1.22 [1.15-1.28]	1.21 [1.20-1.23]	89,234	88,383	72,465	5,464	81,876
Z89	1.12 [1.05-1.20]	1.13 [1.06-1.21]	1.14 [1.07-1.22]		1.08 [1.01-1.15]	1.09 [1.01-1.16]	1.06 [0.99-1.13]	1.06 [0.99-1.14]		1.08 [1.01-1.15]	2,548	2,522	2,480	NA	2,463
Z90	1.13 [1.11-1.15]	1.12 [1.11-1.14]	1.12 [1.10-1.14]	1.03 [0.92-1.16]	1.09 [1.07-1.11]	1.08 [1.06-1.09]	1.05 [1.03-1.07]	1.05 [1.04-1.07]	1.02 [0.90-1.15]	1.06 [1.04-1.08]	43,872	43,913	39,685	991	42,789
Z91	1.52 [1.49-1.54]	1.38 [1.35-1.41]	1.38 [1.35-1.41]	2.28 [2.17-2.39]	1.44 [1.42-1.47]	1.36 [1.33-1.39]	1.21 [1.18-1.23]	1.23 [1.20-1.26]	2.01 [1.91-2.12]	1.33 [1.30-1.35]	36,843	32,108	22,361	8,172	34,862
Z92	1.12 [1.11-1.13]	1.12 [1.11-1.13]	1.12 [1.11-1.13]	1.04 [0.94-1.15]	1.08 [1.07-1.10]	1.08 [1.07-1.09]	1.05 [1.04-1.07]	1.05 [1.04-1.06]	1.00 [0.90-1.11]	1.06 [1.05-1.08]	110,285	110,141	105,614	1,366	104,999
Z93	1.11 [1.07-1.15]	1.13 [1.09-1.17]	1.11 [1.07-1.15]	0.82 [0.68-1.00]	1.09 [1.06-1.13]	1.08 [1.05-1.12]	1.09 [1.05-1.13]	1.08 [1.04-1.12]	0.78 [0.64-0.96]	1.09 [1.05-1.13]	9,435	9,293	8,700	319	9,097

Supplementary Table 4: Hazard ratios and events from all cohorts (excluding non-consulters)

	Hazard ratio (99% confidence interval)														
	crude					adjusted					Events (in exposed)				
Outcome	any age	18+	40+	<18	hosp.	any age	18+	40+	<18	hosp.	any age	18+	40+	<18	hosp.
Z94	1.19 [1.10-1.29]	1.19 [1.10-1.29]	1.21 [1.11-1.32]	1.06 [0.73-1.52]	1.07 [0.99-1.15]	1.16 [1.07-1.26]	1.15 [1.06-1.24]	1.17 [1.07-1.27]	1.01 [0.69-1.49]	1.07 [0.99-1.16]	1,846	1,784	1,541	106	1,801
Z95	1.10 [1.08-1.12]	1.11 [1.09-1.13]	1.11 [1.09-1.13]	0.72 [0.58-0.90]	1.06 [1.05-1.08]	1.07 [1.05-1.09]	1.05 [1.03-1.07]	1.04 [1.02-1.06]	0.71 [0.56-0.89]	1.05 [1.04-1.07]	36,396	36,311	35,702	248	35,794
Z96	1.13 [1.11-1.14]	1.13 [1.11-1.14]	1.12 [1.11-1.14]	0.94 [0.80-1.10]	1.10 [1.09-1.11]	1.09 [1.08-1.11]	1.08 [1.06-1.09]	1.08 [1.06-1.09]	0.95 [0.80-1.12]	1.08 [1.07-1.09]	68,729	68,441	67,217	531	66,196
Z97	1.18 [1.13-1.23]	1.20 [1.15-1.25]	1.16 [1.11-1.22]	1.03 [0.85-1.24]	1.16 [1.11-1.21]	1.12 [1.07-1.17]	1.12 [1.07-1.17]	1.09 [1.04-1.15]	1.05 [0.86-1.28]	1.13 [1.08-1.18]	6,590	6,639	5,281	374	6,440
Z98	1.18 [1.13-1.22]	1.19 [1.15-1.24]	1.17 [1.13-1.22]	0.98 [0.79-1.22]	1.14 [1.10-1.18]	1.10 [1.06-1.14]	1.08 [1.04-1.13]	1.07 [1.03-1.12]	0.96 [0.77-1.21]	1.08 [1.04-1.12]	8,583	8,554	7,525	293	8,577
Z99	1.24 [1.20-1.28]	1.23 [1.20-1.27]	1.23 [1.19-1.27]	0.85 [0.68-1.06]	1.20 [1.16-1.23]	1.17 [1.13-1.20]	1.12 [1.08-1.16]	1.12 [1.08-1.15]	0.83 [0.66-1.05]	1.15 [1.11-1.18]	12,321	12,205	11,685	270	12,359

Supplementary Table 5: GBD-mapped results (hazard ratios and events from all cohorts)

	Hazard ratio (99% confidence interval)														
	crude					adjusted					Events (in exposed)				
Outcome	any age	18+	40+	<18	hosp.	any age	18+	40+	<18	hosp.	any age	18+	40+	<18	hosp.
Infectious and parasitic diseases															
20	1.32 [1.31-1.33]	1.29 [1.28-1.31]	1.29 [1.27-1.30]	1.38 [1.37-1.40]	1.25 [1.24-1.26]	1.26 [1.25-1.27]	1.19 [1.18-1.20]	1.18 [1.17-1.19]	1.35 [1.33-1.36]	1.22 [1.21-1.23]	220,610	162,198	132,012	69,009	190,523
30	1.26 [1.15-1.37]	1.32 [1.21-1.45]	1.36 [1.22-1.51]	0.89 [0.70-1.13]	1.28 [1.16-1.41]	1.20 [1.09-1.31]	1.25 [1.13-1.37]	1.24 [1.12-1.38]	0.95 [0.74-1.21]	1.22 [1.10-1.35]	1,239	1,145	900	165	1,020
40	1.13 [1.10-1.17]	1.16 [1.13-1.20]	1.14 [1.09-1.19]	1.09 [1.00-1.17]	1.07 [1.04-1.11]	1.07 [1.04-1.11]	1.07 [1.04-1.10]	1.06 [1.01-1.10]	1.06 [0.98-1.15]	1.04 [1.01-1.07]	11,723	11,485	5,586	1,693	9,824
90	1.13 [1.10-1.17]	1.16 [1.13-1.19]	1.14 [1.09-1.18]	1.09 [1.01-1.18]	1.07 [1.04-1.10]	1.07 [1.04-1.10]	1.07 [1.03-1.10]	1.06 [1.01-1.10]	1.06 [0.98-1.15]	1.04 [1.00-1.07]	11,559	11,337	5,549	1,649	9,710
100	1.84 [1.39-2.43]	1.90 [1.42-2.52]				1.69 [1.26-2.26]	1.70 [1.26-2.29]				125	119	NA	NA	NA
102	1.84 [1.39-2.43]	1.90 [1.42-2.52]				1.69 [1.26-2.26]	1.70 [1.26-2.29]				125	119	NA	NA	NA
110	1.27 [1.25-1.28]	1.31 [1.29-1.33]	1.29 [1.27-1.31]	1.17 [1.14-1.19]	1.20 [1.18-1.21]	1.19 [1.18-1.21]	1.17 [1.15-1.19]	1.16 [1.14-1.17]	1.13 [1.10-1.16]	1.15 [1.14-1.17]	72,317	56,748	46,663	19,725	65,055
120	1.21 [0.96-1.53]			1.10 [0.82-1.46]	1.15 [0.89-1.48]	1.14 [0.90-1.45]			1.05 [0.79-1.41]	1.09 [0.84-1.42]	181	NA	NA	121	150
170	1.13 [1.04-1.23]	1.16 [1.03-1.31]	1.17 [1.02-1.35]	1.10 [0.98-1.23]	1.04 [0.94-1.14]	1.08 [0.99-1.18]	1.07 [0.95-1.21]	1.08 [0.93-1.25]	1.07 [0.95-1.20]	1.01 [0.92-1.11]	1,275	671	457	706	1,047
180	1.20 [1.10-1.30]	1.23 [1.12-1.36]	1.21 [1.09-1.35]	1.01 [0.85-1.20]	1.12 [1.03-1.23]	1.15 [1.05-1.26]	1.13 [1.02-1.25]	1.11 [0.99-1.24]	0.99 [0.83-1.18]	1.10 [1.00-1.20]	1,317	1,037	855	342	1,129
185	1.19 [1.12-1.27]	1.24 [1.16-1.32]	1.29 [1.20-1.39]	1.04 [0.84-1.29]	1.11 [1.04-1.19]	1.17 [1.09-1.24]	1.15 [1.07-1.22]	1.19 [1.10-1.28]	1.12 [0.90-1.41]	1.12 [1.04-1.20]	2,473	2,366	1,765	218	2,050
186	1.10 [0.86-1.39]	1.24 [0.95-1.60]	1.35 [1.00-1.82]		1.06 [0.82-1.39]	1.06 [0.83-1.36]	1.17 [0.89-1.53]	1.26 [0.93-1.72]		1.03 [0.79-1.35]	171	142	108	NA	139
190	1.20 [1.13-1.28]	1.23 [1.16-1.32]	1.29 [1.19-1.38]	1.07 [0.84-1.37]	1.11 [1.04-1.19]	1.17 [1.09-1.25]	1.14 [1.07-1.22]	1.18 [1.09-1.27]	1.18 [0.92-1.52]	1.12 [1.04-1.20]	2,324	2,246	1,675	179	1,931
200	1.17 [1.07-1.29]	1.22 [1.11-1.34]	1.26 [1.14-1.40]		1.06 [0.96-1.17]	1.12 [1.02-1.23]	1.06 [0.96-1.16]	1.12 [1.00-1.25]		1.07 [0.97-1.19]	1,058	1,048	825	NA	888
210	1.00 [0.85-1.17]	1.10 [0.91-1.32]	1.05 [0.84-1.32]	0.86 [0.65-1.15]	0.97 [0.79-1.18]	0.99 [0.84-1.17]	1.14 [0.94-1.38]	1.07 [0.85-1.35]	0.81 [0.60-1.10]	0.98 [0.80-1.19]	368	276	177	115	243
220	0.97 [0.80-1.16]	1.04 [0.84-1.28]	1.03 [0.80-1.33]		0.92 [0.73-1.15]	0.97 [0.80-1.17]	1.09 [0.88-1.36]	1.06 [0.81-1.38]		0.93 [0.74-1.18]	284	209	133	NA	175
330	1.21 [1.06-1.37]	1.24 [1.04-1.49]	1.18 [0.94-1.49]	1.17 [1.00-1.37]	1.17 [1.02-1.33]	1.14 [1.00-1.29]	1.14 [0.94-1.37]	1.09 [0.86-1.39]	1.11 [0.95-1.31]	1.11 [0.97-1.27]	650	291	168	414	562
362	1.20 [1.06-1.37]	1.23 [1.02-1.48]	1.19 [0.94-1.51]	1.18 [1.00-1.38]	1.15 [1.01-1.32]	1.14 [1.00-1.29]	1.12 [0.93-1.36]	1.11 [0.87-1.43]	1.12 [0.95-1.32]	1.10 [0.96-1.27]	626	272	159	406	542
370	1.36 [1.35-1.37]	1.30 [1.29-1.32]	1.29 [1.28-1.31]	1.47 [1.45-1.49]	1.29 [1.28-1.30]	1.30 [1.29-1.31]	1.20 [1.19-1.21]	1.19 [1.18-1.20]	1.43 [1.41-1.45]	1.26 [1.25-1.27]	170,698	122,439	104,438	54,657	149,347
Respiratory infectious															
380	1.28 [1.27-1.29]	1.29 [1.28-1.30]	1.27 [1.26-1.29]	1.25 [1.24-1.27]	1.22 [1.21-1.23]	1.22 [1.21-1.23]	1.18 [1.16-1.19]	1.17 [1.15-1.18]	1.21 [1.20-1.23]	1.18 [1.17-1.19]	207,228	140,428	122,072	73,961	178,108
390	1.30 [1.29-1.31]	1.29 [1.28-1.30]	1.27 [1.26-1.28]	1.39 [1.36-1.42]	1.24 [1.22-1.25]	1.23 [1.22-1.24]	1.17 [1.16-1.19]	1.16 [1.15-1.18]	1.33 [1.30-1.36]	1.19 [1.18-1.20]	147,648	127,381	117,393	23,454	131,283
400	1.25 [1.23-1.27]	1.34 [1.31-1.37]	1.36 [1.31-1.41]	1.22 [1.20-1.24]	1.20 [1.18-1.21]	1.20 [1.18-1.21]	1.19 [1.16-1.22]	1.19 [1.15-1.24]	1.18 [1.16-1.20]	1.16 [1.14-1.17]	60,682	16,586	6,996	48,823	50,826
410	1.22 [1.19-1.24]	1.45 [1.37-1.53]	1.43 [1.35-1.53]	1.19 [1.16-1.22]	1.18 [1.15-1.20]	1.18 [1.15-1.20]	1.31 [1.24-1.38]	1.31 [1.22-1.39]	1.16 [1.13-1.19]	1.15 [1.12-1.17]	22,933	3,432	2,487	19,831	19,093
Maternal conditions															
420	1.07 [1.05-1.08]	1.12 [1.10-1.13]	1.05 [0.99-1.13]	0.99 [0.97-1.01]	0.97 [0.95-0.99]	1.05 [1.04-1.07]	1.08 [1.06-1.09]	1.09 [1.01-1.17]	0.98 [0.97-1.00]	0.97 [0.95-0.99]	81,715	77,703	2,347	31,825	37,492
Neonatal conditions															
490	1.06 [0.91-1.23]			1.02 [0.86-1.20]	0.98 [0.82-1.17]	1.02 [0.87-1.19]			0.99 [0.84-1.17]	0.98 [0.82-1.17]	448	NA	NA	371	336
530	1.01 [0.86-1.18]			1.02 [0.87-1.20]	0.94 [0.79-1.13]	0.99 [0.84-1.16]			1.01 [0.86-1.19]	0.94 [0.79-1.13]	399	NA	NA	379	318
Nutritional deficiencies															
540	1.32 [1.31-1.34]	1.32 [1.31-1.34]	1.32 [1.30-1.33]	1.28 [1.23-1.33]	1.25 [1.24-1.27]	1.25 [1.23-1.26]	1.20 [1.19-1.21]	1.19 [1.18-1.21]	1.22 [1.17-1.28]	1.20 [1.19-1.22]	81,934	77,944	69,277	6,889	74,156
550	1.20 [1.12-1.28]	1.17 [1.09-1.25]	1.16 [1.08-1.24]	1.28 [0.98-1.67]	1.15 [1.07-1.23]	1.18 [1.10-1.26]	1.09 [1.02-1.17]	1.10 [1.02-1.18]	1.24 [0.94-1.64]	1.16 [1.08-1.24]	2,309	2,231	2,032	156	2,146
560	1.16 [0.99-1.36]	1.21 [1.03-1.41]	1.15 [0.98-1.36]		1.12 [0.95-1.31]	1.14 [0.98-1.34]	1.16 [0.99-1.36]	1.11 [0.94-1.32]		1.11 [0.95-1.31]	439	434	386	NA	402
580	1.32 [1.31-1.34]	1.32 [1.30-1.34]	1.32 [1.30-1.34]	1.28 [1.21-1.35]	1.25 [1.23-1.27]	1.25 [1.23-1.27]	1.20 [1.18-1.22]	1.20 [1.18-1.22]	1.24 [1.18-1.31]	1.20 [1.19-1.22]	53,162	50,841	45,714	4,007	47,985
590	1.35 [1.33-1.37]	1.35 [1.33-1.38]	1.33 [1.31-1.36]	1.30 [1.22-1.38]	1.28 [1.25-1.30]	1.26 [1.24-1.28]	1.21 [1.19-1.23]	1.20 [1.18-1.22]	1.22 [1.15-1.30]	1.22 [1.20-1.24]	37,096	35,187	30,940	3,365	34,254
Malignant neoplasms															
610	1.09 [1.08-1.10]	1.09 [1.08-1.10]	1.09 [1.08-1.10]	1.08 [1.00-1.16]	1.06 [1.05-1.07]	1.08 [1.07-1.09]	1.07 [1.06-1.08]	1.06 [1.05-1.07]	1.07 [0.99-1.16]	1.05 [1.04-1.07]	108,133	107,036	103,666	1,800	86,707
620	1.16 [1.09-1.24]	1.18 [1.11-1.26]	1.19 [1.12-1.27]		1.13 [1.06-1.21]	1.16 [1.09-1.23]	1.14 [1.07-1.21]	1.15 [1.07-1.22]		1.15 [1.08-1.24]	2,437	2,425	2,348	NA	2,035
621	1.18 [1.10-1.28]	1.19 [1.11-1.29]	1.19 [1.10-1.28]		1.16 [1.07-1.26]	1.18 [1.09-1.27]	1.15 [1.06-1.24]	1.15 [1.06-1.24]		1.18 [1.08-1.28]	1,693	1,685	1,613	NA	1,427
622	1.19 [0.90-1.58]	1.20 [0.90-1.60]	1.36 [1.01-1.83]			1.19 [0.89-1.58]	1.17 [0.87-1.56]	1.31 [0.97-1.77]			115	111	108	NA	NA
623	1.12 [1.01-1.24]	1.17 [1.05-1.29]	1.17 [1.05-1.29]		1.08 [0.97-1.21]	1.11 [1.00-1.23]	1.12 [1.01-1.24]	1.12 [1.01-1.24]		1.11 [0.99-1.25]	875	875	873	NA	729
630	1.18 [1.12-1.25]	1.15 [1.09-1.22]	1.17 [1.10-1.23]		1.13 [1.07-1.20]	1.17 [1.11-1.24]	1.12 [1.06-1.18]	1.13 [1.07-1.19]		1.14 [1.07-1.21]	3,259	3,259	3,242	NA	2,722
640	1.08 [1.01-1.15]	1.07 [1.00-1.13]	1.09 [1.02-1.16]		1.06 [0.99-1.14]	1.07 [1.01-1.14]	1.04 [0.98-1.11]	1.06 [0.99-1.13]		1.06 [0.99-1.14]	2,508	2,508	2,476	NA	2,102
650	0.99 [0.97-1.02]	0.99 [0.97-1.02]	0.99 [0.97-1.02]		0.98 [0.95-1.01]	0.99 [0.96-1.02]	0.99 [0.96-1.02]	0.98 [0.96-1.01]		0.98 [0.95-1.02]	11,626	11,618	11,444	NA	9,393
660	1.25 [1.16-1.34]	1.25 [1.16-1.34]	1.23 [1.14-1.32]		1.18 [1.09-1.27]	1.21 [1.13-1.31]	1.18 [1.10-1.27]	1.17 [1.08-1.26]		1.17 [1.08-1.26]	1,925	1,919	1,894	NA	1,708
670	1.16 [1.10-1.23]	1.14 [1.08-1.21]	1.15 [1.09-1.22]		1.13 [1.07-1.20]	1.15 [1.09-1.22]	1.10 [1.04-1.17]	1.11 [1.05-1.18]		1.13 [1.07-1.21]	3,016	3,008	2,998	NA	2,573
680	1.15 [1.12-1.18]	1.15 [1.12-1.18]	1.15 [1.12-1.18]		1.11 [1.08-1.14]	1.14 [1.11-1.17]	1.09 [1.06-1.12]	1.09 [1.06-1.12]		1.12 [1.09-1.15]	13,085	13,068	13,034	NA	11,190
690	1.08 [1.06-1.10]	1.09 [1.07-1.11]	1.09 [1.07-1.11]	1.11 [0.86-1.44]	1.04 [1.02-1.07]	1.06 [1.04-1.08]	1.05 [1.03-1.07]	1.05 [1.03-1.07]	1.01 [0.77-1.32]	1.03 [1.00-1.05]	27,302	27,276	26,659	150	23,012
691	0.95 [0.90-1.01]	0.97 [0.92-1.02]	0.96 [0.90-1.01]		0.97 [0.91-1.02]	0.94 [0.89-1.00]	0.95 [0.90-1.01]	0.94 [0.89-0.99]		0.96 [0.90-1.02]	3,170	3,159	2,886	NA	2,682
692	1.10 [1.08-1.12]	1.11 [1.08-1.13]	1.10 [1.08-1.13]		1.06 [1.03-1.08]	1.07 [1.05-1.09]	1.07 [1.04-1.09]	1.06 [1.04-1.08]		1.03 [1.01-1.06]	25,094	25,077	24,709	NA	21,264
700	1.04 [1.02-1.07]	1.04 [1.01-1.06]	1.03 [1.01-1.06]		1.02 [0.99-1.05]	1.04 [1.01-1.07]	1.03 [1.00-1.05]	1.02 [0.99-1.05]		1.03 [1.00-1.06]	14,359	14,360	13,755	NA	11,473
710	0.96 [0.86-1.07]	0.96 [0.86-1.07]	0.96 [0.84-1.09]		1.02 [0.90-1.15]	0.97 [0.87-1.09]	0.97 [0.87-1.08]	0.98 [0.86-1.12]		1.05 [0.93-1.19]	755	752	523	NA	615
720	1.04 [0.98-1.11]	1.04 [0.98-1.11]	1.03 [0.97-1.09]		1.03 [0.96-1.10]	1.06 [0.99-1.12]	1.06 [1.00-1.13]	1.05 [0.98-1.11]		1.04 [0.97-1.12]	2,358	2,355	2,327	NA	1,822
730	1.03 [0.97-1.10]	1.01 [0.95-1.08]	1.02 [0.96-1.09]		1.00 [0.93-1.08]	1.04 [0.97-1.11]	1.02 [0.95-1.09]	1.02 [0.96-1.10]		1.01 [0.94-1.09]	2,094	2,078	1,979	NA	1,630
740	1.06 [1.03-1.09]	1.06 [1.03-1.09]	1.05 [1.03-1.08]		1.01 [0.98-1.04]	1.04 [1.01-1.06]	1.03 [1.00-1.06]	1.02 [1.00-1.05]		1.00 [0.97-1.03]	13,721	13,725	13,723	NA	11,486
742	1.04 [0.89-1.22]	1.02 [0.87-1.20]	0.99 [0.78-1.26]		1.02 [0.84-1.24]	1.02 [0.87-1.20]	0.99 [0.84-1.17]	0.95 [0.74-1.22]		1.03 [0.84-1.25]	362	345	146	NA	242
745	1.11 [1.06-1.17]	1.10 [1.05-1.16]	1.11 [1.06-1.18]		1.05 [0.99-1.11]	1.09 [1.04-1.15]	1.05 [1.00-1.11]	1.06 [1.01-1.12]		1.05 [0.99-1.11]	3,343	3,296	3,251	NA	2,871
750	1.08 [1.04-1.12]	1.09 [1.05-1.13]	1.08 [1.04-1.13]		1.03 [0.99-1.07]	1.07 [1.03-1.12]	1.06 [1.02-1.10]	1.06 [1.02-1.10]		1.03 [0.99-1.08]	6,425	6,415	6,366	NA	5,376
751	1.12 [1.04-1.21]	1.14 [1.06-1.23]	1.13 [1.04-1.22]	1.01 [0.84-1.22]	1.09 [1.01-1.18]	1.10 [1.03-1.19]	1.11 [1.03-1.20]	1.10 [1.02-1.20]	1.02 [0.84-1.24]	1.08 [1.00-1.17]	1,820	1,586	1,437	278	1,454
752	1.08 [0.96-1.21]	1.07 [0.95-1.20]	1.06 [0.94-1.19]		1.03 [0.91-1.17]	1.07 [0.95-1.21]	1.04 [0.92-1.17]	1.03 [0.91-1.16]		1.03 [0.90-1.17]	688	689	682	NA	578
753	1.14 [1.02-1.28]	1.17 [1.04-1.32]	1.16 [1.03-1.30]		1.08 [0.95-1.22]	1.16 [1.03-1.30]	1.14 [1.01-1.28]	1.12 [0.99-1.26]		1.11 [0.98-1.26]	690	689	680	NA	586
754	1.06 [0.96-1.18]	1.06 [0.95-1.18]	1.06 [0.94-1.20]		1.02 [0.91-1.15]	1.04 [0.93-1.16]	1.02 [0.92-1.14]	1.03 [0.92-1.17]		1.01 [0.90-1.14]	819	798	618	NA	675
755	0.98 [0.88-1.10]	1.00 [0.89-1.12]	1.01 [0.90-1.13]		1.02 [0.91-1.16]	0.99 [0.89-1.11]	1.01 [0.90-1.13]	1.02 [0.91-1.14]		1.04 [0.92-1.18]	725	723	725	NA	614
760	1.29 [1.25-1.34]	1.29 [1.25-1.34]	1.26 [1.22-1.31]	1.34 [1.13-1.59]	1.22 [1.17-1.26]	1.26 [1.22-1.31]	1.26 [1.22-1.31]	1.22 [1.18-1.27]	1.31 [1.09-1.56]	1.20 [1.15-1.25]	7,749	7,541	7,011	353	6,306
761	1.71 [1.54-1.90]	1.72 [1.54-1.91]	1.62 [1.42-1.85]	1.41 [1.11-1.79]	1.46 [1.30-1.65]	1.64 [1.48-1.83]	1.65 [1.48-1.85]	1.52 [1.33-1.74]	1.33 [1.04-1.71]	1.43 [1.26-1.61]	969	870	588	185	701
762	1.32 [1.26-1.38]	1.32 [1.26-1.38]	1.29 [1.23-1.35]	1.28 [1.02-1.61]	1.24 [1.18-1.30]	1.28 [1.23-1.34]	1.28 [1.22-1.34]	1.25 [1.19-1.31]	1.28 [1.01-1.62]	1.22 [1.17-1.29]	5,070	4,947	4,641	198	4,166
763	1.16 [1.09-1.24]	1.17 [1.10-1.25]	1.16 [1.09-1.24]		1.11 [1.04-1.19]	1.13 [1.06-1.21]	1.14 [1.07-1.22]	1.13 [1.06-1.21]		1.10 [1.02-1.17]	2,394	2,385	2,354	NA	2,040
770	1.15 [1.09-1.21]	1.18 [1.12-1.25]	1.16 [1.10-1.22]	0.99 [0.85-1.15]	1.11 [1.05-1.17]	1.13 [1.07-1.18]	1.15 [1.09-1.21]	1.12 [1.06-1.19]	0.99 [0.84-1.16]	1.10 [1.05-1.17]	3,956	3,611	3,460	400	3,325
780	1.19 [1.18-1.20]	1.19 [1.18-1.21]	1.19 [1.18-1.20]	1.01 [0.96-1.07]	1.14 [1.13-1.16]	1.15 [1.14-1.16]	1.10 [1.09-1.11]	1.09 [1.08-1.10]	1.00 [0.95-1.06]	1.12 [1.11-1.14]	108,970	106,556	101,983	3,358	85,808
Other neoplasms															
790	1.16 [1.15-1.17]	1.16 [1.15-1.18]	1.16 [1.15-1.17]	1.12 [1.09-1.16]	1.10 [1.09-1.11]	1.11 [1.10-1.12]	1.09 [1.08-1.10]	1.09 [1.07-1.10]	1.09 [1.05-1.13]	1.07 [1.06-1.08]	93,227	87,836	73,590	8,908	75,311
Diabetes mellitus															
800	1.24 [1.23-1.26]	1.25 [1.24-1.27]	1.25 [1.23-1.26]	1.01 [0.95-1.07]	1.19 [1.18-1.21]	1.18 [1.17-1.19]	1.12 [1.11-1.14]	1.11 [1.10-1.13]	0.99 [0.94-1.06]	1.15 [1.14-1.17]	78,753	76,641	72,970	2,904	60,805
Endocrine, blood, immune disorders															
810	1.27 [1.26-1.27]	1.28 [1.27-1.28]	1.27 [1.26-1.28]	1.17 [1.15-1.19]	1.19 [1.18-1.20]	1.20 [1.20-1.21]	1.17 [1.16-1.17]	1.16 [1.15-1.17]	1.13 [1.11-1.16]	1.15 [1.15-1.16]	290,840	273,746	227,123	32,523	238,894
811	1.40 [1.31-1.50]	1.42 [1.32-1.53]	1.54 [1.40-1.70]	1.34 [1.16-1.56]	1.27 [1.18-1.37]	1.37 [1.27-1.47]	1.37 [1.27-1.48]	1.42 [1.28-1.57]	1.31 [1.12-1.53]	1.25 [1.16-1.35]	2,201	1,898	1,101	518	1,811
812	1.31 [1.23-1.40]	1.37 [1.27-1.47]	1.30 [1.17-1.44]	1.36 [1.23-1.51]	1.22 [1.13-1.31]	1.28 [1.20-1.37]	1.32 [1.22-1.43]	1.23 [1.10-1.37]	1.36 [1.22-1.51]	1.21 [1.12-1.30]	2,519	1,759	859	1,054	1,979
813	1.34 [1.23-1.46]	1.37 [1.24-1.51]	1.34 [1.20-1.49]	1.24 [1.04-1.48]	1.28 [1.17-1.41]	1.32 [1.20-1.44]	1.32 [1.20-1.46]	1.28 [1.15-1.43]	1.24 [1.03-1.48]	1.29 [1.17-1.42]	1,318	1,053	844	333	1,142
814	1.27 [1.26-1.27]	1.28 [1.27-1.28]	1.27 [1.26-1.28]	1.16 [1.14-1.19]	1.19 [1.18-1.20]	1.20 [1.20-1.21]	1.17 [1.16-1.17]	1.16 [1.15-1.17]	1.13 [1.11-1.15]	1.15 [1.15-1.16]	288,595	272,399	226,681	31,345	237,480
Mental and substance use disorders															
820	1.22 [1.21-1.23]	1.24 [1.23-1.25]	1.25 [1.24-1.26]	1.08 [1.06-1.09]	1.15 [1.14-1.16]	1.16 [1.16-1.17]	1.10 [1.09-1.11]	1.12 [1.11-1.13]	1.06 [1.04-1.07]	1.13 [1.12-1.14]	240,813	217,026	151,120	52,334	198,517
830	1.37 [1.35-1.38]	1.38 [1.36-1.39]	1.39 [1.37-1.41]	1.14 [1.10-1.17]	1.26 [1.25-1.28]	1.23 [1.22-1.25]	1.13 [1.12-1.14]	1.15 [1.13-1.17]	1.13 [1.09-1.17]	1.20 [1.18-1.21]	83,481	80,770	58,389	11,116	73,730
831	1.37 [1.35-1.38]	1.38 [1.36-1.39]	1.39 [1.37-1.41]	1.14 [1.10-1.17]	1.26 [1.25-1.28]	1.23 [1.22-1.25]	1.13 [1.12-1.14]	1.15 [1.13-1.17]	1.13 [1.09-1.17]	1.20 [1.18-1.21]	83,481	80,770	58,389	11,116	73,730
840	1.30 [1.24-1.36]	1.32 [1.26-1.38]	1.33 [1.26-1.41]	1.08 [0.94-1.23]	1.16 [1.11-1.22]	1.19 [1.13-1.24]	1.09 [1.03-1.14]	1.13 [1.07-1.20]	1.09 [0.95-1.25]	1.12 [1.07-1.18]	4,470	4,378	2,830	601	3,878
850	1.25 [1.20-1.30]	1.24 [1.20-1.29]	1.31 [1.25-1.37]	0.97 [0.87-1.08]	1.14 [1.09-1.19]	1.22 [1.17-1.27]	1.14 [1.10-1.19]	1.23 [1.17-1.29]	0.99 [0.88-1.11]	1.15 [1.10-1.20]	6,436	6,180	4,492	860	5,088
860	1.20 [1.18-1.23]	1.23 [1.20-1.25]	1.26 [1.23-1.29]	0.98 [0.93-1.03]	1.13 [1.11-1.16]	1.17 [1.14-1.19]	1.11 [1.09-1.14]	1.15 [1.12-1.18]	0.97 [0.92-1.02]	1.15 [1.12-1.17]	23,745	22,275	16,448	3,835	18,898
870	1.14 [1.10-1.17]	1.18 [1.14-1.22]	1.31 [1.25-1.38]	0.97 [0.92-1.03]	1.03 [1.00-1.07]	1.06 [1.03-1.10]	1.00 [0.97-1.04]	1.08 [1.03-1.14]	0.98 [0.93-1.03]	1.02 [0.99-1.06]	9,576	8,354	3,688	3,466	7,900
871	1.19 [1.13-1.25]	1.23 [1.16-1.30]	1.34 [1.24-1.44]	0.98 [0.86-1.11]	1.11 [1.04-1.17]	1.09 [1.03-1.16]	1.00 [0.94-1.06]	1.08 [1.00-1.17]	0.99 [0.87-1.13]	1.08 [1.02-1.14]	3,240	3,030	1,739	616	2,801
872	1.12 [1.03-1.21]	1.16 [1.07-1.26]	1.42 [1.22-1.65]	1.02 [0.89-1.16]	0.98 [0.90-1.07]	1.04 [0.95-1.13]	0.97 [0.89-1.05]	1.13 [0.97-1.33]	1.03 [0.89-1.18]	0.99 [0.91-1.08]	1,455	1,412	404	530	1,208
873	1.03 [0.90-1.19]	1.04 [0.90-1.20]	1.16 [0.91-1.48]	0.95 [0.76-1.18]	0.93 [0.80-1.08]	0.97 [0.85-1.12]	0.93 [0.80-1.08]	0.98 [0.76-1.27]	0.92 [0.73-1.16]	0.94 [0.81-1.10]	503	438	148	200	403
874	1.08 [1.03-1.14]	1.12 [1.06-1.18]	1.28 [1.17-1.41]	0.96 [0.89-1.04]	0.95 [0.90-1.01]	1.01 [0.96-1.07]	0.95 [0.90-1.00]	1.06 [0.96-1.17]	0.97 [0.89-1.05]	0.96 [0.91-1.02]	3,794	3,351	1,033	1,749	3,051
875	1.16 [1.09-1.23]	1.23 [1.15-1.32]	1.31 [1.19-1.43]	0.99 [0.90-1.10]	1.06 [0.99-1.13]	1.08 [1.02-1.16]	1.03 [0.96-1.11]	1.08 [0.98-1.19]	1.00 [0.90-1.11]	1.05 [0.98-1.12]	2,491	1,933	1,086	935	2,148
880	1.33 [1.31-1.34]	1.34 [1.33-1.36]	1.35 [1.33-1.37]	1.16 [1.13-1.19]	1.24 [1.22-1.25]	1.20 [1.18-1.21]	1.12 [1.10-1.13]	1.13 [1.12-1.15]	1.13 [1.10-1.16]	1.16 [1.15-1.18]	78,552	73,087	49,431	15,339	69,390
890	1.28 [1.19-1.37]	1.29 [1.18-1.41]	1.42 [1.22-1.64]	1.13 [1.02-1.24]	1.19 [1.10-1.28]	1.17 [1.08-1.26]	1.08 [0.99-1.18]	1.19 [1.02-1.39]	1.09 [0.99-1.20]	1.13 [1.04-1.22]	2,058	1,258	443	1,189	1,711
900	1.32 [1.28-1.38]	1.54 [1.44-1.65]	2.15 [1.90-2.44]	1.22 [1.17-1.27]	1.23 [1.18-1.28]	1.18 [1.14-1.23]	1.34 [1.24-1.44]	1.73 [1.51-1.97]	1.15 [1.11-1.20]	1.14 [1.09-1.19]	7,896	2,095	695	6,653	6,860
910	1.22 [1.16-1.28]	1.22 [1.11-1.33]	1.39 [1.14-1.70]	1.17 [1.11-1.24]	1.14 [1.08-1.20]	1.13 [1.08-1.19]	1.08 [0.99-1.19]	1.14 [0.93-1.40]	1.14 [1.08-1.20]	1.11 [1.05-1.17]	4,568	1,169	251	3,997	3,979
911	1.22 [1.15-1.28]	1.23 [1.12-1.35]	1.75 [1.34-2.29]	1.16 [1.09-1.22]	1.14 [1.08-1.21]	1.12 [1.06-1.19]	1.09 [0.99-1.21]	1.26 [0.94-1.68]	1.12 [1.06-1.19]	1.11 [1.04-1.17]	3,883	1,020	139	3,444	3,393
912	1.24 [1.11-1.39]	1.14 [0.90-1.44]	1.10 [0.82-1.46]	1.27 [1.12-1.43]	1.15 [1.02-1.30]	1.17 [1.05-1.31]	1.04 [0.81-1.32]	1.02 [0.76-1.37]	1.22 [1.07-1.38]	1.11 [0.98-1.25]	867	174	113	730	754
920	1.58 [1.49-1.67]	1.89 [1.77-2.02]	2.15 [1.98-2.33]	1.09 [1.00-1.18]	1.37 [1.29-1.45]	1.45 [1.37-1.53]	1.74 [1.63-1.86]	1.94 [1.78-2.11]	1.05 [0.97-1.15]	1.30 [1.22-1.38]	3,632	2,534	1,643	1,483	3,148
930	1.16 [1.15-1.17]	1.18 [1.17-1.19]	1.18 [1.17-1.20]	1.03 [1.01-1.05]	1.09 [1.08-1.10]	1.13 [1.12-1.14]	1.06 [1.05-1.07]	1.08 [1.07-1.09]	1.03 [1.01-1.05]	1.11 [1.10-1.12]	149,446	139,440	95,176	29,738	126,704
Neurological conditions															
940	1.24 [1.23-1.25]	1.24 [1.23-1.25]	1.24 [1.22-1.25]	1.18 [1.15-1.21]	1.17 [1.16-1.18]	1.17 [1.16-1.18]	1.13 [1.12-1.14]	1.13 [1.12-1.14]	1.14 [1.11-1.16]	1.13 [1.12-1.14]	161,265	145,227	126,193	21,702	138,222
950	1.10 [1.08-1.11]	1.09 [1.08-1.11]	1.09 [1.08-1.11]	1.02 [0.78-1.33]	1.07 [1.05-1.08]	1.08 [1.07-1.10]	1.06 [1.04-1.08]	1.06 [1.04-1.07]	0.99 [0.75-1.30]	1.07 [1.05-1.08]	47,325	47,210	47,009	139	42,954
960	1.07 [1.03-1.11]	1.06 [1.03-1.10]	1.07 [1.04-1.11]		1.04 [1.00-1.08]	1.06 [1.02-1.10]	1.03 [1.00-1.07]	1.04 [1.01-1.08]		1.04 [1.00-1.08]	7,838	7,840	7,793	NA	6,778
970	1.30 [1.27-1.33]	1.35 [1.32-1.39]	1.37 [1.33-1.41]	1.07 [1.02-1.12]	1.18 [1.15-1.21]	1.20 [1.17-1.23]	1.18 [1.15-1.21]	1.19 [1.16-1.23]	1.05 [1.00-1.10]	1.13 [1.10-1.16]	17,589	14,393	11,345	4,197	14,416
980	1.23 [1.15-1.31]	1.22 [1.15-1.30]	1.23 [1.14-1.32]	1.14 [0.85-1.53]	1.14 [1.06-1.22]	1.16 [1.08-1.24]	1.08 [1.01-1.15]	1.09 [1.01-1.18]	1.15 [0.85-1.55]	1.12 [1.04-1.21]	2,212	2,199	1,684	125	1,677
990	1.32 [1.28-1.35]	1.32 [1.29-1.36]	1.33 [1.29-1.38]	1.20 [1.14-1.26]	1.21 [1.18-1.25]	1.16 [1.13-1.19]	1.08 [1.05-1.11]	1.10 [1.06-1.14]	1.13 [1.07-1.20]	1.12 [1.09-1.15]	16,417	14,623	8,600	3,948	14,478
1,000	1.33 [1.24-1.42]	1.31 [1.23-1.40]	1.37 [1.26-1.49]	1.22 [1.05-1.42]	1.23 [1.15-1.32]	1.20 [1.12-1.28]	1.12 [1.04-1.20]	1.15 [1.06-1.26]	1.19 [1.02-1.39]	1.15 [1.07-1.23]	2,317	2,109	1,353	470	2,084
1,010	1.29 [1.28-1.30]	1.30 [1.28-1.31]	1.29 [1.28-1.30]	1.19 [1.16-1.23]	1.20 [1.19-1.22]	1.20 [1.19-1.22]	1.15 [1.14-1.17]	1.15 [1.14-1.16]	1.15 [1.12-1.18]	1.15 [1.14-1.16]	102,753	90,649	79,225	15,059	89,516
Sense organ diseases															
1,020	1.24 [1.24-1.25]	1.26 [1.25-1.27]	1.25 [1.24-1.26]	1.18 [1.16-1.20]	1.18 [1.17-1.19]	1.19 [1.18-1.20]	1.17 [1.16-1.18]	1.17 [1.16-1.18]	1.14 [1.12-1.17]	1.14 [1.13-1.15]	179,196	154,467	141,161	29,279	145,632
1,030	1.18 [1.15-1.20]	1.18 [1.15-1.20]	1.18 [1.15-1.20]	1.29 [1.00-1.67]	1.13 [1.11-1.16]	1.14 [1.12-1.17]	1.13 [1.11-1.16]	1.13 [1.11-1.16]	1.26 [0.97-1.64]	1.11 [1.08-1.14]	21,887	21,771	21,555	166	18,784
1,040	1.21 [1.20-1.22]	1.21 [1.19-1.22]	1.21 [1.20-1.22]	1.59 [1.36-1.86]	1.15 [1.14-1.17]	1.16 [1.15-1.18]	1.14 [1.13-1.15]	1.14 [1.13-1.16]	1.56 [1.33-1.83]	1.12 [1.11-1.14]	93,063	92,792	92,062	471	77,516
1,050	1.10 [1.07-1.13]	1.15 [1.12-1.19]	1.15 [1.11-1.18]	1.02 [0.97-1.06]	1.06 [1.03-1.09]	1.07 [1.04-1.10]	1.10 [1.07-1.14]	1.09 [1.06-1.13]	0.99 [0.95-1.04]	1.04 [1.01-1.07]	14,365	9,472	8,504	5,228	12,293
1,070	1.18 [1.17-1.20]	1.19 [1.17-1.21]	1.18 [1.16-1.20]	1.15 [1.10-1.21]	1.13 [1.11-1.15]	1.14 [1.12-1.15]	1.12 [1.10-1.13]	1.11 [1.09-1.13]	1.11 [1.05-1.16]	1.10 [1.09-1.12]	49,522	46,488	41,633	4,784	43,251
1,080	1.23 [1.21-1.26]	1.25 [1.22-1.27]	1.24 [1.22-1.27]	1.16 [1.11-1.21]	1.18 [1.16-1.20]	1.18 [1.16-1.20]	1.17 [1.14-1.19]	1.17 [1.14-1.19]	1.12 [1.07-1.17]	1.14 [1.12-1.16]	35,538	30,845	29,334	5,219	32,606
1,090	1.29 [1.28-1.31]	1.31 [1.30-1.33]	1.30 [1.28-1.32]	1.24 [1.21-1.27]	1.22 [1.20-1.24]	1.23 [1.22-1.25]	1.22 [1.20-1.23]	1.21 [1.19-1.23]	1.20 [1.17-1.23]	1.18 [1.16-1.19]	73,023	56,768	48,813	19,041	61,598
Cardiovascular diseases															
1,100	1.26 [1.25-1.27]	1.26 [1.25-1.27]	1.26 [1.25-1.27]	1.14 [1.11-1.18]	1.18 [1.18-1.19]	1.20 [1.20-1.21]	1.15 [1.14-1.16]	1.15 [1.14-1.16]	1.11 [1.07-1.14]	1.15 [1.14-1.16]	264,656	257,075	239,279	12,155	181,766
1,110	1.23 [1.20-1.25]	1.22 [1.20-1.25]	1.23 [1.20-1.25]	0.97 [0.81-1.16]	1.16 [1.13-1.18]	1.18 [1.15-1.20]	1.14 [1.11-1.16]	1.14 [1.11-1.16]	0.96 [0.80-1.16]	1.13 [1.11-1.16]	23,896	23,737	23,254	315	22,215
1,120	1.28 [1.24-1.31]	1.28 [1.25-1.32]	1.29 [1.26-1.33]	1.08 [0.87-1.33]	1.17 [1.14-1.21]	1.23 [1.20-1.27]	1.17 [1.14-1.20]	1.17 [1.14-1.21]	1.08 [0.87-1.34]	1.16 [1.12-1.19]	13,243	13,058	12,889	224	11,938
1,130	1.22 [1.21-1.23]	1.22 [1.21-1.24]	1.22 [1.21-1.24]	1.06 [0.87-1.31]	1.15 [1.13-1.16]	1.16 [1.15-1.18]	1.12 [1.11-1.13]	1.11 [1.10-1.13]	1.06 [0.86-1.31]	1.11 [1.10-1.13]	84,291	84,251	83,224	233	68,531
1,140	1.16 [1.14-1.17]	1.16 [1.14-1.17]	1.16 [1.14-1.17]	0.99 [0.86-1.13]	1.12 [1.11-1.14]	1.13 [1.12-1.15]	1.10 [1.08-1.11]	1.10 [1.08-1.11]	0.99 [0.86-1.14]	1.12 [1.10-1.13]	60,756	60,404	59,576	523	53,772
1,150	1.23 [1.20-1.27]	1.24 [1.21-1.28]	1.24 [1.21-1.28]	1.07 [0.97-1.19]	1.16 [1.13-1.19]	1.18 [1.14-1.21]	1.14 [1.11-1.18]	1.14 [1.11-1.18]	1.06 [0.95-1.18]	1.13 [1.09-1.16]	13,408	12,890	11,745	917	11,840
1,160	1.24 [1.23-1.25]	1.24 [1.23-1.25]	1.24 [1.23-1.25]	1.15 [1.11-1.18]	1.18 [1.17-1.19]	1.20 [1.19-1.21]	1.16 [1.15-1.17]	1.16 [1.15-1.16]	1.11 [1.07-1.15]	1.15 [1.14-1.16]	193,587	187,338	175,285	9,860	159,624
Respiratory diseases															
1,170	1.62 [1.61-1.63]	1.55 [1.54-1.56]	1.46 [1.45-1.47]	1.88 [1.85-1.90]	1.49 [1.48-1.50]	1.39 [1.38-1.40]	1.24 [1.23-1.25]	1.22 [1.21-1.23]	1.65 [1.63-1.67]	1.32 [1.31-1.33]	296,188	223,550	171,643	91,824	226,829
1,180	1.44 [1.42-1.46]	1.44 [1.42-1.45]	1.43 [1.42-1.45]	1.47 [1.29-1.69]	1.34 [1.32-1.36]	1.28 [1.27-1.30]	1.20 [1.18-1.22]	1.20 [1.19-1.22]	1.34 [1.16-1.54]	1.22 [1.20-1.24]	68,734	68,403	67,240	607	59,581
1,190	2.12 [2.11-2.14]	1.98 [1.96-1.99]	1.84 [1.82-1.86]	2.50 [2.46-2.53]	1.93 [1.91-1.95]	1.61 [1.60-1.63]	1.34 [1.33-1.36]	1.31 [1.29-1.33]	2.06 [2.03-2.09]	1.51 [1.50-1.53]	165,315	117,360	75,382	63,614	122,153
1,200	1.33 [1.32-1.34]	1.31 [1.30-1.32]	1.29 [1.28-1.30]	1.41 [1.39-1.44]	1.26 [1.25-1.27]	1.25 [1.24-1.26]	1.17 [1.16-1.18]	1.17 [1.16-1.18]	1.33 [1.31-1.35]	1.20 [1.19-1.21]	165,151	130,657	112,436	41,511	141,040
Digestive diseases															

Supplementary Table 5: GBD-mapped results (hazard ratios and events from all cohorts)

	Hazard ratio (99% confidence interval)										Events (in exposed)				
	crude					adjusted									
Outcome	any age	18+	40+	<18	hosp.	any age	18+	40+	<18	hosp.	any age	18+	40+	<18	hosp.
1,210	1.28 [1.27-1.29]	1.29 [1.28-1.30]	1.29 [1.28-1.30]	1.19 [1.17-1.20]	1.19 [1.19-1.20]	1.21 [1.20-1.21]	1.18 [1.17-1.19]	1.18 [1.17-1.19]	1.14 [1.12-1.16]	1.14 [1.14-1.15]	315,904	282,825	227,352	50,932	238,006
1,220	1.26 [1.23-1.29]	1.27 [1.24-1.29]	1.26 [1.23-1.29]	1.26 [1.11-1.44]	1.19 [1.16-1.21]	1.20 [1.17-1.23]	1.16 [1.14-1.19]	1.16 [1.13-1.18]	1.23 [1.08-1.42]	1.16 [1.13-1.18]	20,957	20,678	19,339	624	18,027
1,230	1.43 [1.38-1.48]	1.45 [1.40-1.50]	1.45 [1.40-1.50]	1.09 [0.84-1.42]	1.34 [1.29-1.38]	1.34 [1.30-1.39]	1.28 [1.24-1.33]	1.28 [1.24-1.33]	1.03 [0.79-1.35]	1.31 [1.26-1.36]	8,739	8,692	8,048	147	7,676
1,240	1.09 [1.06-1.11]	1.10 [1.07-1.13]	1.06 [1.02-1.12]	1.05 [1.02-1.09]	1.02 [0.99-1.05]	1.05 [1.02-1.08]	1.03 [1.00-1.07]	1.01 [0.97-1.06]	1.03 [0.99-1.06]	1.00 [0.97-1.03]	16,027	9,242	3,848	9,326	11,863
1,241	1.32 [1.31-1.34]	1.33 [1.31-1.35]	1.33 [1.31-1.35]	1.23 [1.17-1.28]	1.22 [1.21-1.24]	1.22 [1.21-1.24]	1.19 [1.17-1.20]	1.19 [1.17-1.20]	1.16 [1.11-1.22]	1.16 [1.15-1.18]	69,990	66,962	59,204	5,411	60,277
1,242	1.21 [1.18-1.24]	1.21 [1.18-1.24]	1.20 [1.17-1.23]	1.08 [0.99-1.18]	1.14 [1.11-1.17]	1.15 [1.12-1.17]	1.11 [1.09-1.14]	1.11 [1.08-1.14]	1.04 [0.95-1.14]	1.10 [1.07-1.13]	17,069	16,153	14,775	1,354	15,433
1,244	1.38 [1.36-1.40]	1.40 [1.38-1.42]	1.37 [1.35-1.39]	1.28 [1.24-1.32]	1.26 [1.24-1.28]	1.28 [1.26-1.30]	1.25 [1.23-1.27]	1.23 [1.21-1.25]	1.23 [1.19-1.27]	1.21 [1.19-1.22]	49,842	43,247	34,394	9,371	41,117
1,246	1.17 [1.16-1.19]	1.18 [1.16-1.19]	1.17 [1.15-1.18]	1.05 [0.99-1.12]	1.11 [1.09-1.13]	1.11 [1.10-1.13]	1.08 [1.07-1.10]	1.08 [1.06-1.09]	1.02 [0.96-1.09]	1.08 [1.06-1.09]	49,054	48,597	40,227	2,480	42,828
1,248	1.20 [1.16-1.23]	1.22 [1.18-1.25]	1.20 [1.16-1.24]	0.95 [0.84-1.07]	1.13 [1.10-1.17]	1.14 [1.10-1.17]	1.11 [1.08-1.14]	1.09 [1.06-1.13]	0.93 [0.82-1.06]	1.10 [1.07-1.14]	11,248	11,026	9,607	635	9,978
1,250	1.30 [1.29-1.31]	1.31 [1.30-1.31]	1.30 [1.29-1.30]	1.23 [1.21-1.25]	1.21 [1.20-1.22]	1.21 [1.21-1.22]	1.18 [1.17-1.19]	1.18 [1.17-1.19]	1.17 [1.15-1.19]	1.15 [1.15-1.16]	283,770	261,648	216,773	36,299	224,276
Genitourinary diseases															
1,260	1.22 [1.21-1.23]	1.22 [1.21-1.23]	1.22 [1.21-1.23]	1.19 [1.17-1.20]	1.15 [1.14-1.16]	1.17 [1.16-1.18]	1.14 [1.13-1.14]	1.14 [1.13-1.15]	1.15 [1.13-1.17]	1.12 [1.12-1.13]	263,963	236,324	191,230	42,723	211,374
1,270	1.22 [1.21-1.23]	1.22 [1.21-1.23]	1.22 [1.21-1.23]	1.07 [1.02-1.11]	1.16 [1.15-1.17]	1.17 [1.16-1.18]	1.13 [1.12-1.14]	1.13 [1.12-1.14]	1.04 [1.00-1.09]	1.14 [1.13-1.15]	124,174	121,174	114,202	5,781	112,699
1,271	1.35 [1.06-1.73]	1.31 [0.98-1.76]	1.34 [0.98-1.83]		1.20 [0.93-1.54]	1.34 [1.04-1.73]	1.26 [0.93-1.70]	1.24 [0.90-1.71]		1.18 [0.91-1.53]	174	121	101	NA	160
1,272	1.45 [1.37-1.55]	1.45 [1.36-1.54]	1.45 [1.36-1.55]		1.29 [1.21-1.37]	1.35 [1.27-1.44]	1.24 [1.16-1.32]	1.22 [1.14-1.31]		1.27 [1.19-1.35]	2,642	2,638	2,538	NA	2,542
1,273	1.22 [1.21-1.23]	1.22 [1.21-1.23]	1.22 [1.21-1.23]	1.07 [1.02-1.11]	1.16 [1.15-1.17]	1.17 [1.16-1.18]	1.13 [1.12-1.14]	1.13 [1.12-1.14]	1.04 [1.00-1.09]	1.14 [1.13-1.15]	124,119	121,149	114,183	5,750	112,653
1,280	1.16 [1.14-1.19]	1.16 [1.14-1.18]	1.16 [1.14-1.18]		1.10 [1.08-1.12]	1.12 [1.10-1.14]	1.10 [1.08-1.12]	1.10 [1.08-1.12]		1.07 [1.05-1.09]	30,069	30,057	29,975	NA	26,000
1,290	1.14 [1.11-1.17]	1.14 [1.11-1.17]	1.13 [1.10-1.17]	0.97 [0.88-1.07]	1.06 [1.03-1.09]	1.09 [1.06-1.12]	1.06 [1.04-1.09]	1.06 [1.03-1.09]	0.94 [0.85-1.04]	1.04 [1.01-1.07]	14,150	13,909	11,059	1,052	11,806
1,300	1.23 [1.22-1.24]	1.22 [1.21-1.24]	1.22 [1.20-1.23]	1.22 [1.19-1.24]	1.15 [1.14-1.16]	1.18 [1.17-1.19]	1.14 [1.13-1.15]	1.13 [1.12-1.15]	1.18 [1.16-1.21]	1.13 [1.12-1.14]	147,268	124,802	109,348	28,564	124,457
1,310	0.95 [0.87-1.03]	0.97 [0.89-1.05]	1.00 [0.78-1.27]		0.98 [0.89-1.09]	0.94 [0.86-1.02]	0.95 [0.87-1.03]	1.00 [0.78-1.29]		0.97 [0.87-1.07]	1,353	1,353	137	NA	823
1,320	1.19 [1.18-1.21]	1.20 [1.18-1.21]	1.19 [1.18-1.21]	1.15 [1.12-1.19]	1.13 [1.11-1.14]	1.14 [1.13-1.15]	1.11 [1.10-1.13]	1.12 [1.11-1.14]	1.11 [1.08-1.15]	1.10 [1.08-1.11]	80,226	76,332	47,501	12,194	61,923
Skin diseases															
1,330	1.96 [1.94-1.97]	1.74 [1.72-1.75]	1.67 [1.66-1.69]	2.81 [2.77-2.86]	1.83 [1.82-1.85]	1.86 [1.85-1.88]	1.60 [1.59-1.62]	1.55 [1.54-1.57]	2.69 [2.65-2.73]	1.77 [1.75-1.78]	212,545	160,139	127,239	64,263	175,421
Musculoskeletal diseases															
1,340	1.29 [1.28-1.30]	1.29 [1.29-1.30]	1.30 [1.29-1.31]	1.17 [1.15-1.19]	1.20 [1.19-1.21]	1.21 [1.20-1.22]	1.18 [1.17-1.18]	1.18 [1.17-1.19]	1.12 [1.10-1.15]	1.15 [1.14-1.15]	267,151	245,258	205,775	34,599	208,501
1,350	1.41 [1.38-1.44]	1.40 [1.37-1.43]	1.39 [1.36-1.42]	1.27 [1.05-1.54]	1.31 [1.28-1.35]	1.32 [1.28-1.35]	1.27 [1.24-1.30]	1.26 [1.23-1.29]	1.20 [0.98-1.47]	1.24 [1.21-1.28]	17,774	17,725	16,515	291	15,460
1,360	1.28 [1.27-1.29]	1.28 [1.27-1.29]	1.28 [1.27-1.29]	1.18 [1.05-1.32]	1.20 [1.19-1.21]	1.20 [1.19-1.21]	1.17 [1.16-1.18]	1.16 [1.15-1.17]	1.10 [0.98-1.24]	1.14 [1.13-1.15]	117,124	117,031	113,542	802	100,636
1,370	1.36 [1.33-1.39]	1.36 [1.33-1.39]	1.36 [1.33-1.39]		1.27 [1.24-1.30]	1.29 [1.26-1.32]	1.24 [1.21-1.27]	1.23 [1.20-1.26]		1.22 [1.19-1.25]	19,181	19,179	18,861	NA	17,324
1,380	1.30 [1.28-1.31]	1.30 [1.29-1.31]	1.30 [1.29-1.32]	1.15 [1.11-1.20]	1.20 [1.19-1.21]	1.20 [1.19-1.21]	1.15 [1.14-1.16]	1.15 [1.14-1.17]	1.10 [1.05-1.14]	1.14 [1.13-1.15]	96,513	93,934	79,147	6,828	84,881
1,390	1.30 [1.29-1.30]	1.30 [1.29-1.31]	1.31 [1.30-1.32]	1.18 [1.16-1.20]	1.20 [1.19-1.21]	1.21 [1.20-1.22]	1.18 [1.17-1.19]	1.19 [1.18-1.20]	1.13 [1.11-1.15]	1.15 [1.14-1.16]	209,238	189,096	159,953	29,805	171,806
Congenital anomalies															
1,400	1.19 [1.17-1.21]	1.26 [1.23-1.29]	1.29 [1.25-1.33]	1.09 [1.06-1.12]	1.11 [1.09-1.13]	1.13 [1.11-1.15]	1.15 [1.12-1.18]	1.17 [1.14-1.21]	1.06 [1.04-1.09]	1.08 [1.05-1.10]	32,251	17,025	11,591	17,158	25,999
1,410	1.28 [1.11-1.48]	1.32 [1.13-1.54]	1.32 [1.10-1.59]	1.08 [0.81-1.45]	1.17 [1.01-1.37]	1.17 [1.01-1.36]	1.19 [1.01-1.39]	1.16 [0.96-1.41]	1.02 [0.76-1.38]	1.09 [0.93-1.28]	476	404	268	116	395
1,420	1.03 [0.87-1.21]			1.02 [0.85-1.22]	0.98 [0.82-1.18]	0.98 [0.83-1.16]			0.96 [0.80-1.16]	0.96 [0.79-1.15]	348	NA	NA	286	292
1,430	2.77 [2.46-3.12]	3.76 [3.28-4.32]	4.16 [3.54-4.89]	1.03 [0.81-1.31]	2.00 [1.68-2.37]	2.68 [2.37-3.02]	3.86 [3.35-4.45]	4.05 [3.44-4.78]	1.00 [0.79-1.29]	1.90 [1.59-2.27]	855	725	526	161	367
1,440	1.17 [1.12-1.22]	1.22 [1.15-1.28]	1.23 [1.16-1.31]	1.04 [0.96-1.11]	1.10 [1.05-1.16]	1.12 [1.07-1.17]	1.13 [1.07-1.19]	1.14 [1.07-1.21]	1.02 [0.94-1.09]	1.07 [1.02-1.12]	5,055	3,427	2,545	1,913	4,275
1,450	1.29 [1.18-1.41]	1.54 [1.33-1.78]	1.71 [1.40-2.08]	1.14 [1.02-1.27]	1.22 [1.11-1.34]	1.21 [1.11-1.33]	1.38 [1.19-1.61]	1.55 [1.26-1.89]	1.09 [0.97-1.21]	1.16 [1.05-1.28]	1,285	485	262	873	1,126
1,460	1.17 [1.14-1.19]	1.23 [1.20-1.26]	1.26 [1.21-1.30]	1.09 [1.07-1.12]	1.10 [1.08-1.13]	1.11 [1.09-1.13]	1.11 [1.08-1.14]	1.14 [1.10-1.18]	1.06 [1.04-1.09]	1.07 [1.04-1.09]	26,608	12,766	8,409	15,480	21,781
Oral conditions															
1,470	1.18 [1.17-1.19]	1.27 [1.25-1.29]	1.29 [1.26-1.31]	1.08 [1.07-1.10]	1.11 [1.10-1.12]	1.13 [1.12-1.14]	1.15 [1.14-1.17]	1.17 [1.15-1.19]	1.06 [1.04-1.08]	1.08 [1.07-1.09]	91,561	50,669	26,576	49,756	70,760
1,480	1.11 [1.09-1.13]	1.25 [1.22-1.28]	1.28 [1.24-1.32]	1.02 [1.00-1.04]	1.05 [1.03-1.07]	1.07 [1.05-1.09]	1.12 [1.10-1.15]	1.15 [1.11-1.19]	1.01 [0.99-1.03]	1.03 [1.01-1.05]	42,689	17,603	8,465	28,068	34,584
1,490	1.29 [1.25-1.34]	1.33 [1.28-1.38]	1.33 [1.24-1.42]	1.21 [1.14-1.29]	1.19 [1.15-1.24]	1.22 [1.17-1.26]	1.20 [1.15-1.24]	1.21 [1.13-1.29]	1.16 [1.09-1.24]	1.15 [1.10-1.19]	8,466	7,996	2,186	2,842	6,400
1,502	1.22 [1.20-1.24]	1.27 [1.25-1.29]	1.30 [1.27-1.32]	1.13 [1.11-1.15]	1.15 [1.13-1.16]	1.16 [1.15-1.18]	1.16 [1.14-1.18]	1.18 [1.15-1.21]	1.10 [1.08-1.12]	1.11 [1.10-1.13]	61,180	38,767	21,493	29,069	47,317
Unintentional injuries															
1,520	1.16 [1.16-1.17]	1.21 [1.20-1.22]	1.21 [1.20-1.22]	1.06 [1.04-1.07]	1.11 [1.10-1.12]	1.12 [1.11-1.13]	1.13 [1.12-1.14]	1.13 [1.12-1.14]	1.04 [1.02-1.05]	1.09 [1.08-1.10]	246,179	184,392	152,605	74,802	198,640
1,530	1.16 [1.16-1.17]	1.21 [1.20-1.22]	1.21 [1.20-1.22]	1.06 [1.04-1.07]	1.11 [1.10-1.12]	1.12 [1.11-1.13]	1.13 [1.12-1.14]	1.13 [1.12-1.14]	1.04 [1.02-1.05]	1.09 [1.08-1.10]	246,179	184,392	152,605	74,802	198,640
1,540	1.13 [1.08-1.17]	1.19 [1.13-1.27]	1.25 [1.16-1.35]	1.07 [1.02-1.13]	1.07 [1.03-1.12]	1.08 [1.03-1.12]	1.05 [0.99-1.12]	1.09 [1.01-1.18]	1.05 [0.99-1.11]	1.05 [1.00-1.10]	5,432	2,840	1,623	3,151	4,586
1,550	1.14 [1.13-1.15]	1.17 [1.16-1.19]	1.18 [1.17-1.19]	1.02 [1.00-1.04]	1.10 [1.09-1.11]	1.10 [1.09-1.12]	1.11 [1.09-1.12]	1.11 [1.10-1.13]	1.01 [0.99-1.03]	1.08 [1.07-1.10]	123,750	97,427	89,267	29,486	104,225
1,560	1.01 [0.96-1.06]	1.11 [1.02-1.20]	1.12 [1.02-1.23]	0.96 [0.90-1.03]	0.98 [0.92-1.04]	0.99 [0.93-1.04]	1.03 [0.95-1.12]	1.04 [0.94-1.14]	0.95 [0.89-1.02]	0.97 [0.91-1.03]	3,270	1,460	1,057	2,004	2,799
1,570	0.88 [0.67-1.14]				0.83 [0.62-1.11]	0.84 [0.64-1.10]				0.81 [0.60-1.09]	128	NA	NA	NA	104
1,575	1.06 [1.04-1.08]	1.11 [1.08-1.13]	1.12 [1.09-1.16]	1.02 [1.00-1.05]	1.01 [0.99-1.04]	1.03 [1.01-1.05]	1.04 [1.01-1.06]	1.05 [1.02-1.09]	1.01 [0.98-1.04]	1.00 [0.98-1.02]	28,689	16,072	8,886	16,270	22,319
1,580	0.98 [0.72-1.33]					0.92 [0.67-1.27]					105	NA	NA	NA	NA
1,590	1.25 [1.24-1.26]	1.28 [1.27-1.29]	1.27 [1.26-1.28]	1.14 [1.12-1.16]	1.17 [1.16-1.19]	1.19 [1.18-1.20]	1.17 [1.16-1.18]	1.17 [1.15-1.18]	1.10 [1.08-1.13]	1.14 [1.13-1.15]	123,759	105,231	88,344	24,731	106,478
Intentional injuries															
1,600	1.10 [1.08-1.12]	1.14 [1.11-1.16]	1.27 [1.23-1.32]	1.00 [0.98-1.03]	1.01 [0.99-1.04]	1.05 [1.03-1.07]	1.00 [0.98-1.03]	1.09 [1.05-1.13]	0.99 [0.96-1.02]	1.00 [0.98-1.03]	23,875	16,855	6,452	12,388	17,833
1,610	1.11 [1.09-1.14]	1.14 [1.11-1.17]	1.28 [1.23-1.34]	1.00 [0.97-1.04]	1.02 [1.00-1.05]	1.05 [1.03-1.08]	0.99 [0.96-1.02]	1.09 [1.04-1.14]	0.99 [0.96-1.03]	1.01 [0.98-1.04]	17,707	12,089	5,089	9,073	13,698
1,620	1.07 [1.03-1.11]	1.13 [1.08-1.18]	1.24 [1.15-1.34]	1.01 [0.96-1.07]	0.98 [0.94-1.03]	1.03 [0.99-1.07]	1.03 [0.98-1.07]	1.08 [1.00-1.17]	1.00 [0.95-1.05]	0.98 [0.94-1.02]	6,784	5,326	1,554	3,482	4,828

Supplementary Table 6: phecode-mapped results (hazard ratios and events from all cohorts)

	Hazard ratio (99% confidence interval)										Events (in exposed)				
	crude					adjusted									
Outcome	any age	18+	40+	<18	hosp.	any age	18+	40+	<18	hosp.	any age	18+	40+	<18	hosp.
infectious diseases															
8	1.29 [1.27-1.31]	1.32 [1.30-1.34]	1.29 [1.27-1.32]	1.19 [1.15-1.23]	1.21 [1.19-1.23]	1.20 [1.18-1.22]	1.17 [1.15-1.19]	1.15 [1.13-1.17]	1.14 [1.10-1.18]	1.16 [1.14-1.18]	51,853	44,673	36,068	10,855	47,318
8.5	1.25 [1.20-1.31]	1.26 [1.20-1.32]	1.28 [1.21-1.34]	1.11 [0.99-1.25]	1.15 [1.10-1.21]	1.17 [1.11-1.22]	1.14 [1.09-1.20]	1.15 [1.09-1.21]	1.07 [0.95-1.21]	1.09 [1.04-1.15]	4,846	4,328	3,579	751	4,083
8.51	1.20 [0.95-1.50]	1.29 [0.96-1.74]			1.18 [0.92-1.51]	1.13 [0.90-1.43]	1.22 [0.90-1.65]			1.15 [0.90-1.48]	187	110	NA	NA	162
8.52	1.28 [1.23-1.33]	1.28 [1.23-1.33]	1.27 [1.22-1.32]	1.16 [0.94-1.43]	1.21 [1.17-1.26]	1.23 [1.19-1.28]	1.19 [1.15-1.24]	1.18 [1.14-1.23]	1.10 [0.88-1.37]	1.19 [1.14-1.24]	6,908	6,779	6,507	226	6,244
8.6	1.20 [1.17-1.23]	1.31 [1.26-1.37]	1.31 [1.25-1.38]	1.14 [1.10-1.17]	1.15 [1.12-1.18]	1.15 [1.12-1.18]	1.17 [1.12-1.22]	1.17 [1.12-1.23]	1.11 [1.08-1.15]	1.12 [1.09-1.16]	14,673	5,645	4,555	9,469	13,130
8.7	1.19 [0.97-1.47]	1.21 [0.93-1.57]		1.48 [1.07-2.04]	1.14 [0.90-1.43]	1.12 [0.90-1.39]	1.06 [0.81-1.39]		1.40 [1.01-1.95]	1.10 [0.88-1.39]	221	139	NA	101	185
10	1.26 [1.15-1.38]	1.33 [1.21-1.45]	1.36 [1.23-1.51]	0.89 [0.70-1.14]	1.28 [1.16-1.41]	1.20 [1.10-1.32]	1.25 [1.14-1.37]	1.25 [1.12-1.39]	0.95 [0.74-1.22]	1.22 [1.11-1.35]	1,244	1,150	905	165	1,025
31	1.39 [1.04-1.85]				1.28 [0.95-1.72]	1.26 [0.94-1.70]				1.13 [0.83-1.53]	117	NA	NA	NA	110
38	1.24 [1.22-1.26]	1.25 [1.23-1.27]	1.25 [1.22-1.27]	1.07 [1.00-1.16]	1.18 [1.16-1.21]	1.20 [1.18-1.22]	1.17 [1.15-1.19]	1.16 [1.14-1.18]	1.05 [0.97-1.13]	1.17 [1.15-1.19]	35,491	34,297	32,322	1,893	32,907
38.1	1.21 [1.17-1.25]	1.21 [1.17-1.26]	1.23 [1.19-1.28]	1.03 [0.92-1.16]	1.15 [1.12-1.20]	1.17 [1.13-1.21]	1.13 [1.09-1.17]	1.14 [1.10-1.19]	1.02 [0.90-1.15]	1.14 [1.10-1.18]	9,006	8,421	8,036	710	8,335
38.2	1.51 [1.44-1.59]	1.52 [1.44-1.60]	1.52 [1.44-1.60]	1.27 [1.08-1.50]	1.40 [1.33-1.47]	1.45 [1.38-1.53]	1.41 [1.34-1.48]	1.41 [1.33-1.49]	1.23 [1.04-1.45]	1.38 [1.31-1.45]	4,414	4,102	3,811	391	3,973
41	1.29 [1.27-1.32]	1.31 [1.29-1.34]	1.31 [1.29-1.34]	1.13 [1.07-1.19]	1.22 [1.20-1.25]	1.23 [1.21-1.25]	1.20 [1.18-1.23]	1.20 [1.18-1.22]	1.09 [1.04-1.15]	1.19 [1.17-1.21]	34,181	31,214	28,566	3,812	30,330
41.1	1.60 [1.57-1.64]	1.59 [1.56-1.63]	1.57 [1.54-1.61]	1.70 [1.61-1.80]	1.50 [1.46-1.53]	1.53 [1.50-1.57]	1.46 [1.43-1.50]	1.45 [1.41-1.48]	1.65 [1.56-1.75]	1.46 [1.43-1.50]	23,132	20,137	17,620	3,862	20,430
41.2	1.38 [1.34-1.42]	1.40 [1.36-1.44]	1.40 [1.35-1.45]	1.25 [1.18-1.32]	1.29 [1.25-1.33]	1.32 [1.29-1.36]	1.29 [1.26-1.34]	1.29 [1.24-1.33]	1.22 [1.15-1.29]	1.27 [1.23-1.31]	13,748	11,189	8,920	3,252	12,204
41.21	1.06 [0.81-1.39]	1.00 [0.74-1.34]	1.10 [0.82-1.49]		1.03 [0.78-1.37]	1.00 [0.76-1.32]	0.90 [0.67-1.22]	1.03 [0.76-1.40]		1.00 [0.75-1.33]	130	107	105	NA	117
41.4	1.17 [1.15-1.19]	1.18 [1.15-1.20]	1.19 [1.16-1.21]	1.04 [0.98-1.11]	1.11 [1.09-1.14]	1.13 [1.10-1.15]	1.09 [1.07-1.11]	1.10 [1.08-1.13]	1.03 [0.96-1.09]	1.10 [1.07-1.12]	26,594	24,751	22,561	2,705	24,487
53	1.42 [1.34-1.50]	1.41 [1.33-1.49]	1.40 [1.32-1.49]	1.41 [1.20-1.67]	1.32 [1.24-1.40]	1.34 [1.26-1.42]	1.29 [1.21-1.37]	1.29 [1.21-1.37]	1.35 [1.13-1.60]	1.26 [1.19-1.33]	3,475	3,158	2,946	394	3,139
53.1	1.29 [1.13-1.47]	1.32 [1.16-1.51]	1.33 [1.16-1.52]		1.24 [1.08-1.42]	1.22 [1.07-1.40]	1.21 [1.06-1.39]	1.24 [1.08-1.42]		1.19 [1.03-1.36]	621	608	584	NA	571
54	2.46 [2.33-2.60]	2.02 [1.87-2.19]	1.73 [1.57-1.90]	3.00 [2.78-3.24]	2.28 [2.14-2.42]	2.29 [2.16-2.42]	1.77 [1.63-1.92]	1.54 [1.39-1.69]	2.85 [2.63-3.08]	2.15 [2.02-2.29]	3,948	1,866	1,186	2,363	3,327
70	1.20 [1.12-1.28]	1.23 [1.15-1.32]	1.29 [1.19-1.39]	1.06 [0.79-1.41]	1.10 [1.02-1.18]	1.16 [1.08-1.25]	1.14 [1.06-1.22]	1.17 [1.08-1.27]	1.18 [0.87-1.59]	1.11 [1.03-1.19]	2,021	1,972	1,495	123	1,714
70.1	1.10 [0.86-1.39]	1.24 [0.95-1.60]	1.35 [1.00-1.82]		1.06 [0.82-1.39]	1.06 [0.83-1.36]	1.17 [0.89-1.53]	1.26 [0.93-1.72]		1.03 [0.79-1.35]	171	142	108	NA	139
70.2	1.21 [1.10-1.34]	1.24 [1.13-1.37]	1.38 [1.23-1.55]		1.11 [1.00-1.23]	1.21 [1.10-1.35]	1.23 [1.11-1.36]	1.30 [1.16-1.47]		1.12 [1.00-1.25]	973	963	720	NA	804
70.3	1.17 [1.07-1.29]	1.22 [1.11-1.34]	1.26 [1.14-1.40]		1.06 [0.96-1.17]	1.12 [1.02-1.23]	1.06 [0.96-1.16]	1.12 [1.00-1.25]		1.07 [0.97-1.19]	1,058	1,048	825	NA	888
70.4	1.42 [1.27-1.59]	1.40 [1.24-1.56]	1.42 [1.25-1.60]		1.34 [1.19-1.50]	1.33 [1.18-1.49]	1.26 [1.12-1.42]	1.28 [1.13-1.46]		1.27 [1.13-1.44]	799	736	640	NA	703
70.9	1.48 [1.39-1.58]	1.52 [1.42-1.63]	1.52 [1.41-1.63]	1.28 [1.00-1.63]	1.39 [1.30-1.49]	1.36 [1.27-1.45]	1.32 [1.23-1.42]	1.31 [1.22-1.41]	1.22 [0.95-1.57]	1.33 [1.24-1.42]	2,397	2,317	2,020	190	2,198
71.1	1.83 [1.38-2.43]	1.90 [1.42-2.54]				1.65 [1.22-2.22]	1.69 [1.25-2.30]				120	115	NA	NA	NA
78	1.41 [1.31-1.52]	1.40 [1.29-1.52]	1.42 [1.28-1.56]	1.44 [1.22-1.69]	1.33 [1.22-1.45]	1.30 [1.20-1.41]	1.25 [1.15-1.36]	1.29 [1.17-1.43]	1.32 [1.12-1.56]	1.26 [1.15-1.37]	1,723	1,411	1,005	409	1,392
79	1.50 [1.48-1.52]	1.34 [1.30-1.38]	1.33 [1.28-1.39]	1.53 [1.51-1.56]	1.44 [1.42-1.46]	1.43 [1.41-1.45]	1.17 [1.13-1.21]	1.16 [1.11-1.21]	1.49 [1.46-1.51]	1.39 [1.37-1.41]	48,807	10,855	6,395	39,583	43,067
79.1	1.61 [1.52-1.72]	1.39 [1.14-1.68]	1.43 [1.10-1.87]	1.63 [1.53-1.74]	1.53 [1.44-1.64]	1.56 [1.46-1.66]	1.27 [1.04-1.55]	1.29 [0.99-1.70]	1.58 [1.48-1.69]	1.49 [1.40-1.60]	2,710	260	139	2,482	2,395
79.2	1.15 [1.06-1.24]	1.11 [1.00-1.24]		1.16 [1.06-1.26]	1.08 [0.98-1.19]	1.10 [1.01-1.19]	1.02 [0.91-1.14]		1.10 [1.01-1.21]	1.04 [0.94-1.15]	1,551	787	NA	1,319	1,099
80	1.38 [1.35-1.42]	1.39 [1.35-1.43]	1.39 [1.35-1.43]	1.25 [1.17-1.35]	1.27 [1.24-1.31]	1.30 [1.26-1.33]	1.25 [1.22-1.29]	1.26 [1.22-1.30]	1.19 [1.11-1.28]	1.23 [1.19-1.26]	14,249	12,851	10,760	2,107	12,312
81	1.32 [1.27-1.38]	1.36 [1.30-1.42]	1.33 [1.27-1.39]	1.19 [1.07-1.33]	1.20 [1.14-1.25]	1.27 [1.21-1.33]	1.25 [1.20-1.31]	1.22 [1.16-1.29]	1.14 [1.02-1.27]	1.19 [1.13-1.24]	5,621	4,971	4,303	899	5,162
110.1	1.73 [1.61-1.86]	1.78 [1.65-1.91]	1.72 [1.59-1.86]	1.48 [1.22-1.79]	1.64 [1.53-1.77]	1.59 [1.48-1.71]	1.57 [1.45-1.70]	1.53 [1.41-1.66]	1.38 [1.13-1.69]	1.55 [1.44-1.67]	2,264	2,009	1,827	302	2,033
110.11	1.33 [1.12-1.57]	1.45 [1.22-1.72]	1.39 [1.16-1.65]		1.38 [1.16-1.64]	1.18 [0.99-1.40]	1.26 [1.05-1.50]	1.22 [1.02-1.47]		1.25 [1.05-1.50]	371	359	335	NA	340
110.12	1.89 [1.66-2.16]	1.95 [1.70-2.23]	1.87 [1.62-2.16]		1.71 [1.49-1.96]	1.75 [1.53-2.01]	1.73 [1.51-1.99]	1.69 [1.45-1.96]		1.63 [1.41-1.88]	648	617	535	NA	560
110.13	1.75 [1.50-2.04]	1.76 [1.49-2.06]	1.77 [1.50-2.09]		1.72 [1.47-2.01]	1.61 [1.37-1.88]	1.54 [1.30-1.81]	1.54 [1.30-1.83]		1.63 [1.38-1.91]	493	453	431	NA	459
110.2	1.87 [1.63-2.14]	1.86 [1.62-2.14]	1.77 [1.53-2.05]		1.68 [1.46-1.92]	1.72 [1.50-1.97]	1.66 [1.44-1.92]	1.58 [1.36-1.84]		1.60 [1.39-1.84]	651	592	538	NA	596
112	1.36 [1.33-1.39]	1.37 [1.34-1.40]	1.38 [1.34-1.41]	1.11 [1.04-1.20]	1.26 [1.23-1.29]	1.25 [1.22-1.28]	1.20 [1.17-1.23]	1.21 [1.18-1.24]	1.06 [0.99-1.14]	1.19 [1.16-1.22]	18,913	17,961	15,081	1,965	17,283
112.3	1.56 [1.37-1.78]	1.61 [1.41-1.85]	1.61 [1.40-1.86]		1.47 [1.28-1.68]	1.46 [1.28-1.67]	1.43 [1.24-1.65]	1.44 [1.25-1.67]		1.42 [1.24-1.63]	649	594	562	NA	604
117	1.57 [1.40-1.76]	1.52 [1.35-1.71]	1.52 [1.34-1.72]	1.48 [1.10-2.00]	1.45 [1.29-1.62]	1.46 [1.30-1.64]	1.36 [1.20-1.54]	1.36 [1.20-1.55]	1.46 [1.07-1.98]	1.36 [1.21-1.53]	829	722	658	120	761
117.4	1.68 [1.51-1.88]	1.84 [1.64-2.06]	1.89 [1.68-2.13]		1.51 [1.35-1.69]	1.40 [1.24-1.57]	1.46 [1.30-1.65]	1.50 [1.32-1.70]		1.27 [1.13-1.43]	857	811	745	NA	784
130	1.20 [0.97-1.49]	1.18 [0.92-1.50]	1.22 [0.92-1.62]		1.11 [0.88-1.40]	1.13 [0.90-1.41]	1.04 [0.81-1.34]	1.10 [0.82-1.48]		1.05 [0.83-1.33]	202	155	112	NA	175
130.1	1.20 [0.89-1.62]					1.08 [0.79-1.47]					109	NA	NA	NA	NA
132	2.28 [1.99-2.61]	2.33 [2.01-2.70]	2.36 [2.03-2.75]		2.00 [1.73-2.31]	2.23 [1.95-2.56]	2.18 [1.88-2.53]	2.22 [1.90-2.59]		2.00 [1.72-2.31]	669	574	548	NA	572
132.1	1.12 [0.91-1.37]	1.19 [0.89-1.59]		0.95 [0.71-1.26]	1.04 [0.83-1.30]	1.11 [0.90-1.36]	1.09 [0.81-1.47]		0.95 [0.71-1.26]	1.03 [0.82-1.29]	228	117	NA	115	189
133	0.97 [0.81-1.16]	1.03 [0.85-1.27]	1.00 [0.78-1.29]		0.92 [0.74-1.15]	0.97 [0.80-1.16]	1.08 [0.87-1.33]	1.02 [0.79-1.32]		0.93 [0.74-1.17]	302	223	141	NA	189
134	1.33 [1.05-1.68]	1.54 [1.14-2.08]			1.35 [1.06-1.74]	1.29 [1.01-1.65]	1.48 [1.08-2.02]			1.35 [1.05-1.74]	187	114	NA	NA	165
134.1	1.18 [1.06-1.32]	1.20 [1.02-1.41]	1.16 [0.95-1.42]	1.14 [0.98-1.31]	1.13 [1.00-1.27]	1.12 [1.00-1.26]	1.12 [0.95-1.32]	1.08 [0.87-1.33]	1.08 [0.93-1.26]	1.08 [0.96-1.22]	787	358	218	490	683
136	1.28 [1.19-1.37]	1.29 [1.20-1.39]	1.24 [1.15-1.35]	1.23 [1.01-1.48]	1.22 [1.14-1.32]	1.22 [1.13-1.31]	1.18 [1.09-1.27]	1.15 [1.06-1.24]	1.20 [0.99-1.45]	1.20 [1.11-1.29]	2,133	1,902	1,709	291	1,987
neoplasms															
145	1.20 [1.04-1.38]	1.21 [1.05-1.40]	1.20 [1.04-1.38]		1.13 [0.97-1.31]	1.19 [1.03-1.37]	1.15 [1.00-1.33]	1.12 [0.97-1.30]		1.15 [0.98-1.34]	472	475	452	NA	410
145.1	1.04 [0.81-1.35]	1.08 [0.84-1.40]	1.10 [0.85-1.42]		1.04 [0.80-1.36]	1.03 [0.80-1.33]	1.05 [0.81-1.36]	1.05 [0.81-1.37]		1.03 [0.79-1.35]	146	146	144	NA	128
145.2	1.23 [1.10-1.37]	1.24 [1.11-1.39]	1.23 [1.10-1.38]		1.23 [1.09-1.39]	1.22 [1.09-1.36]	1.19 [1.06-1.33]	1.19 [1.06-1.34]		1.26 [1.11-1.42]	754	754	735	NA	644
145.3	1.06 [0.89-1.27]	1.07 [0.89-1.28]	1.04 [0.86-1.26]		1.06 [0.87-1.29]	1.05 [0.88-1.25]	1.01 [0.84-1.22]	0.99 [0.82-1.20]		1.04 [0.86-1.27]	297	288	258	NA	246
145.4	1.47 [1.12-1.92]	1.38 [1.06-1.81]	1.44 [1.09-1.91]		1.32 [0.99-1.76]	1.46 [1.11-1.91]	1.38 [1.05-1.82]	1.42 [1.07-1.88]		1.32 [0.98-1.77]	139	139	132	NA	118
145.5	1.13 [0.88-1.45]	1.15 [0.90-1.48]	1.12 [0.87-1.44]		1.10 [0.84-1.43]	1.14 [0.89-1.47]	1.08 [0.84-1.39]	1.06 [0.82-1.37]		1.13 [0.86-1.48]	151	150	145	NA	128
149	1.10 [0.91-1.34]	1.12 [0.92-1.36]	1.07 [0.87-1.30]		0.95 [0.77-1.18]	1.09 [0.89-1.33]	1.07 [0.87-1.31]	1.00 [0.82-1.23]		0.97 [0.78-1.20]	235	235	234	NA	202
149.1	1.08 [0.95-1.24]	1.12 [0.99-1.28]	1.14 [1.00-1.30]		1.06 [0.92-1.22]	1.07 [0.94-1.22]	1.06 [0.93-1.22]	1.09 [0.95-1.24]		1.08 [0.93-1.25]	531	531	530	NA	435
149.2	1.19 [0.90-1.58]	1.20 [0.90-1.60]	1.36 [1.01-1.83]			1.19 [0.89-1.58]	1.17 [0.87-1.56]	1.31 [0.97-1.77]			115	111	108	NA	NA
149.3	1.21 [0.98-1.48]	1.25 [1.02-1.54]	1.28 [1.04-1.57]		1.22 [0.98-1.52]	1.23 [1.00-1.52]	1.23 [0.99-1.51]	1.26 [1.02-1.55]		1.31 [1.05-1.64]	221	221	221	NA	191
149.4	1.14 [1.02-1.28]	1.17 [1.05-1.31]	1.15 [1.03-1.29]		1.07 [0.95-1.21]	1.16 [1.03-1.30]	1.13 [1.01-1.27]	1.12 [1.00-1.26]		1.11 [0.98-1.26]	737	736	726	NA	624
149.9	1.21 [0.96-1.51]	1.17 [0.94-1.47]	1.28 [1.01-1.61]		1.23 [0.96-1.57]	1.20 [0.95-1.51]	1.13 [0.90-1.42]	1.22 [0.96-1.54]		1.21 [0.94-1.55]	183	180	177	NA	156
150	1.18 [1.11-1.24]	1.15 [1.09-1.22]	1.16 [1.10-1.23]		1.13 [1.06-1.20]	1.17 [1.10-1.23]	1.12 [1.06-1.18]	1.13 [1.07-1.19]		1.13 [1.07-1.20]	3,271	3,271	3,254	NA	2,734
151	1.08 [1.01-1.15]	1.07 [1.00-1.13]	1.09 [1.02-1.16]		1.06 [0.99-1.14]	1.07 [1.01-1.14]	1.04 [0.98-1.11]	1.06 [0.99-1.13]		1.06 [0.99-1.14]	2,515	2,515	2,483	NA	2,109
153.2	1.02 [0.98-1.05]	1.01 [0.98-1.05]	1.01 [0.98-1.05]		1.00 [0.96-1.03]	1.00 [0.97-1.04]	1.00 [0.96-1.03]	1.00 [0.96-1.03]		0.99 [0.96-1.03]	8,495	8,486	8,349	NA	7,040
153.3	0.97 [0.93-1.02]	0.98 [0.94-1.02]	0.98 [0.94-1.02]		0.96 [0.91-1.01]	0.98 [0.94-1.02]	0.98 [0.94-1.03]	0.98 [0.94-1.03]		0.97 [0.92-1.02]	4,787	4,786	4,707	NA	3,800
155	1.24 [1.16-1.33]	1.25 [1.16-1.34]	1.23 [1.14-1.32]		1.18 [1.09-1.27]	1.21 [1.13-1.30]	1.18 [1.10-1.27]	1.16 [1.08-1.25]		1.17 [1.08-1.26]	1,928	1,922	1,897	NA	1,711
155.1	1.31 [1.18-1.45]	1.34 [1.21-1.49]	1.32 [1.19-1.47]		1.26 [1.13-1.40]	1.25 [1.13-1.39]	1.24 [1.11-1.38]	1.22 [1.10-1.36]		1.24 [1.12-1.39]	915	909	900	NA	833
157	1.16 [1.10-1.23]	1.14 [1.08-1.21]	1.15 [1.09-1.22]		1.13 [1.07-1.20]	1.15 [1.09-1.22]	1.10 [1.04-1.17]	1.11 [1.05-1.18]		1.13 [1.07-1.21]	3,016	3,008	2,998	NA	2,573
158	1.10 [1.04-1.16]	1.10 [1.04-1.17]	1.11 [1.05-1.17]		1.09 [1.03-1.16]	1.07 [1.01-1.13]	1.06 [1.00-1.12]	1.05 [1.00-1.12]		1.09 [1.02-1.15]	3,061	3,036	2,947	NA	2,623
159	1.11 [1.08-1.14]	1.11 [1.08-1.14]	1.11 [1.08-1.14]		1.06 [1.03-1.09]	1.09 [1.06-1.12]	1.06 [1.03-1.09]	1.07 [1.04-1.10]		1.05 [1.03-1.09]	13,238	13,203	12,938	NA	12,018
159.2	1.11 [0.97-1.27]	1.15 [1.00-1.31]	1.12 [0.98-1.29]		1.07 [0.93-1.23]	1.09 [0.95-1.25]	1.08 [0.95-1.24]	1.09 [0.95-1.25]		1.06 [0.92-1.22]	530	529	521	NA	469
159.3	1.08 [0.96-1.21]	1.07 [0.95-1.20]	1.06 [0.94-1.19]		1.03 [0.91-1.17]	1.07 [0.95-1.21]	1.04 [0.92-1.17]	1.03 [0.91-1.16]		1.03 [0.90-1.17]	688	689	682	NA	578
159.4	1.04 [0.90-1.20]	1.07 [0.93-1.23]	1.06 [0.92-1.23]		1.04 [0.89-1.21]	1.03 [0.89-1.19]	1.06 [0.92-1.23]	1.05 [0.91-1.21]		1.03 [0.88-1.21]	451	444	439	NA	373
164	1.17 [0.94-1.46]	1.12 [0.89-1.40]	1.11 [0.87-1.40]		1.16 [0.91-1.47]	1.16 [0.92-1.45]	1.09 [0.87-1.38]	1.09 [0.86-1.39]		1.16 [0.91-1.48]	193	179	163	NA	161
165	1.18 [1.12-1.23]	1.18 [1.13-1.24]	1.17 [1.12-1.23]		1.10 [1.05-1.16]	1.13 [1.08-1.19]	1.09 [1.04-1.15]	1.08 [1.03-1.13]		1.08 [1.03-1.14]	4,438	4,415	4,368	NA	4,129
165.1	1.15 [1.12-1.18]	1.15 [1.12-1.18]	1.15 [1.12-1.18]		1.11 [1.08-1.14]	1.14 [1.11-1.17]	1.09 [1.06-1.12]	1.09 [1.06-1.12]		1.12 [1.09-1.15]	13,101	13,084	13,050	NA	11,204
170.1	1.20 [1.04-1.37]	1.17 [1.01-1.36]	1.15 [0.98-1.35]	1.25 [0.92-1.70]	1.15 [0.98-1.33]	1.19 [1.03-1.37]	1.14 [0.98-1.32]	1.12 [0.95-1.32]	1.26 [0.92-1.73]	1.15 [0.99-1.34]	507	424	364	109	416
170.2	1.09 [0.98-1.21]	1.13 [1.01-1.26]	1.13 [1.01-1.27]		1.10 [0.98-1.24]	1.06 [0.96-1.18]	1.10 [0.99-1.23]	1.08 [0.97-1.22]		1.10 [0.98-1.23]	871	796	711	NA	743
172.11	0.99 [0.94-1.04]	1.00 [0.96-1.05]	0.99 [0.95-1.04]		0.98 [0.94-1.04]	0.97 [0.93-1.02]	0.98 [0.94-1.03]	0.97 [0.93-1.02]		0.97 [0.93-1.03]	4,204	4,190	3,863	NA	3,528
172.2	1.10 [1.08-1.12]	1.11 [1.08-1.13]	1.10 [1.08-1.13]		1.06 [1.03-1.08]	1.07 [1.05-1.09]	1.07 [1.04-1.09]	1.06 [1.04-1.08]		1.03 [1.01-1.06]	25,094	25,077	24,709	NA	21,264
172.3	1.25 [1.18-1.33]	1.26 [1.19-1.34]	1.26 [1.19-1.34]		1.20 [1.13-1.28]	1.19 [1.12-1.27]	1.18 [1.11-1.25]	1.17 [1.10-1.24]		1.16 [1.09-1.23]	3,098	3,093	3,069	NA	2,827
173	1.04 [0.88-1.23]	1.06 [0.89-1.26]	1.04 [0.86-1.25]		0.96 [0.79-1.15]	0.98 [0.83-1.17]	1.01 [0.84-1.20]	0.99 [0.82-1.19]		0.92 [0.76-1.12]	331	303	276	NA	259
174	0.96 [0.74-1.25]	0.96 [0.74-1.25]	0.93 [0.72-1.21]		0.92 [0.70-1.22]	0.95 [0.73-1.24]	0.94 [0.72-1.22]	0.93 [0.71-1.21]		0.92 [0.70-1.22]	126	126	123	NA	108
174.1	1.01 [0.95-1.07]	1.01 [0.95-1.07]	1.00 [0.94-1.06]		0.96 [0.90-1.03]	0.99 [0.93-1.06]	1.00 [0.93-1.06]	0.98 [0.92-1.05]		0.96 [0.89-1.03]	2,263	2,265	2,184	NA	1,812
174.11	1.06 [1.04-1.09]	1.06 [1.04-1.08]	1.05 [1.03-1.08]		1.04 [1.01-1.07]	1.06 [1.03-1.08]	1.04 [1.02-1.07]	1.03 [1.01-1.06]		1.05 [1.02-1.07]	18,072	18,066	17,357	NA	14,324
174.3	0.99 [0.74-1.34]	0.96 [0.71-1.29]				0.99 [0.73-1.33]	0.93 [0.69-1.26]				104	104	NA	NA	NA
175	1.22 [1.21-1.24]	1.22 [1.21-1.24]	1.21 [1.20-1.23]	1.21 [1.14-1.28]	1.13 [1.12-1.15]	1.15 [1.13-1.16]	1.11 [1.09-1.12]	1.10 [1.09-1.12]	1.16 [1.09-1.23]	1.10 [1.08-1.11]	59,573	58,287	52,051	3,107	55,520
180.1	1.14 [1.12-1.18]	1.14 [1.11-1.17]	1.14 [1.11-1.17]	1.06 [0.79-1.42]	1.07 [1.04-1.10]	1.11 [1.08-1.14]	1.08 [1.06-1.11]	1.08 [1.05-1.11]	1.09 [0.81-1.48]	1.07 [1.04-1.10]	13,997	13,964	13,435	117	12,876
180.3	1.08 [1.04-1.13]	1.10 [1.06-1.15]	1.06 [0.97-1.14]	1.04 [0.93-1.17]	1.03 [0.98-1.08]	1.05 [1.00-1.10]	1.03 [0.99-1.08]	1.01 [0.93-1.09]	1.05 [0.93-1.19]	1.03 [0.98-1.08]	4,977	4,983	1,335	736	3,779
182	1.04 [0.98-1.11]	1.04 [0.98-1.11]	1.03 [0.97-1.10]		1.03 [0.96-1.10]	1.06 [0.99-1.12]	1.06 [1.00-1.13]	1.05 [0.98-1.11]		1.04 [0.98-1.12]	2,510	2,506	2,472	NA	1,945
184	1.06 [0.89-1.25]	1.06 [0.89-1.25]	1.00 [0.82-1.21]		1.09 [0.91-1.32]	1.05 [0.89-1.25]	1.05 [0.88-1.24]	0.96 [0.79-1.16]		1.11 [0.92-1.35]	330	313	243	NA	266
184.1	1.17 [0.98-1.41]	1.20 [1.00-1.45]	1.20 [1.00-1.45]		1.16 [0.95-1.41]	1.19 [0.99-1.44]	1.22 [1.01-1.47]	1.21 [1.00-1.46]		1.17 [0.95-1.43]	276	276	270	NA	228
184.11	1.14 [1.11-1.17]	1.13 [1.10-1.16]	1.13 [1.10-1.16]	1.00 [0.75-1.33]	1.06 [1.04-1.09]	1.11 [1.08-1.14]	1.08 [1.05-1.11]	1.08 [1.05-1.11]	1.01 [0.75-1.36]	1.06 [1.03-1.09]	14,907	14,868	14,399	123	13,494
184.2	1.14 [1.05-1.24]	1.14 [1.05-1.24]	1.14 [1.04-1.25]		1.15 [1.05-1.26]	1.12 [1.03-1.22]	1.10 [1.01-1.20]	1.09 [0.99-1.20]		1.14 [1.04-1.26]	1,277	1,254	1,078	NA	1,074
185	1.08 [1.06-1.10]	1.08 [1.06-1.10]	1.08 [1.05-1.10]		1.02 [1.00-1.05]	1.06 [1.03-1.08]	1.05 [1.02-1.07]	1.04 [1.02-1.07]		1.02 [0.99-1.04]	20,634	20,611	20,204	NA	17,639
187	1.25 [1.01-1.53]	1.26 [1.03-1.55]	1.18 [0.96-1.46]		1.18 [0.95-1.47]	1.22 [0.99-1.51]	1.20 [0.98-1.49]	1.16 [0.93-1.44]		1.17 [0.94-1.47]	217	216	203	NA	184
187.1	1.15 [1.12-1.19]	1.15 [1.12-1.18]	1.15 [1.12-1.18]	1.10 [0.80-1.51]	1.07 [1.04-1.10]	1.12 [1.09-1.15]	1.09 [1.06-1.12]	1.09 [1.06-1.12]	1.14 [0.82-1.58]	1.07 [1.04-1.10]	13,776	13,744	13,331	101	12,748
187.2	1.15 [1.12-1.18]	1.15 [1.12-1.18]	1.14 [1.11-1.18]	1.07 [0.84-1.38]	1.07 [1.04-1.10]	1.12 [1.09-1.15]	1.09 [1.06-1.12]	1.09 [1.06-1.12]	1.13 [0.87-1.46]	1.07 [1.04-1.10]	13,848	13,807	13,264	160	12,744
187.8	1.12 [0.88-1.43]	1.13 [0.88-1.45]	1.21 [0.92-1.59]		1.13 [0.86-1.47]	1.11 [0.87-1.43]	1.11 [0.86-1.43]	1.17 [0.88-1.54]		1.11 [0.85-1.46]	158	151	122	NA	130
189	1.14 [1.10-1.18]	1.14 [1.10-1.18]	1.14 [1.10-1.18]		1.08 [1.04-1.12]	1.11 [1.07-1.15]	1.09 [1.05-1.13]	1.08 [1.05-1.12]		1.07 [1.03-1.11]	8,077	8,029	7,939	NA	7,248
189.11	1.11 [1.05-1.18]	1.10 [1.04-1.17]	1.11 [1.05-1.18]		1.06 [0.99-1.12]	1.09 [1.03-1.15]	1.05 [0.99-1.11]	1.06 [1.00-1.12]		1.05 [0.99-1.12]	2,914	2,867	2,824	NA	2,501
189.12	1.22 [1.01-1.47]	1.22 [1.01-1.47]	1.22 [1.01-1.48]		1.11 [0.91-1.36]	1.24 [1.02-1.50]	1.18 [0.98-1.43]	1.21 [1.00-1.47]		1.15 [0.94-1.41]	282	280	277	NA	241
189.2	1.09 [1.02-1.16]	1.08 [1.01-1.16]	1.09 [1.02-1.17]		1.01 [0.95-1.09]	1.07 [1.00-1.15]	1.05 [0.98-1.12]	1.07 [1.00-1.14]		1.02 [0.95-1.09]	2,176	2,175	2,143	NA	1,863
189.21	1.08 [1.04-1.12]	1.09 [1.05-1.13]	1.08 [1.04-1.13]		1.03 [0.99-1.07]	1.07 [1.03-1.12]	1.06 [1.02-1.10]	1.06 [1.02-1.10]		1.03 [0.99-1.08]	6,425	6,415	6,366	NA	5,376
189.4	1.08 [0.94-1.25]	1.08 [0.94-1.24]	1.09 [0.95-1.26]		0.98 [0.84-1.13]	1.08 [0.93-1.24]	1.06 [0.92-1.22]	1.06 [0.92-1.22]		0.99 [0.85-1.15]	473	474	471	NA	416
190	1.02 [0.85-1.22]	0.99 [0.82-1.20]	1.08 [0.89-1.31]		1.00 [0.83-1.22]	0.99 [0.82-1.19]	0.97 [0.80-1.18]	1.04 [0.85-1.27]		1.00 [0.82-1.22]	280	257	241	NA	239
191	1.13 [1.01-1.25]	1.10 [0.98-1.23]	1.05 [0.93-1.18]	1.24 [0.96-1.59]	1.08 [0.96-1.20]	1.10 [0.99-1.22]	1.06 [0.94-1.18]	0.99 [0.88-1.12]	1.25 [0.97-1.61]	1.07 [0.95-1.20]	881	747	651	166	748
191.1	1.12 [0.89-1.41]	1.09 [0.84-1.42]	1.12 [0.84-1.49]		1.06 [0.83-1.35]	1.08 [0.86-1.36]	1.05 [0.80-1.37]	1.12 [0.84-1.49]		1.07 [0.84-1.36]	186	135	112	NA	161
191.11	1.12 [1.04-1.21]	1.15 [1.06-1.24]	1.13 [1.04-1.23]	0.99 [0.81-1.21]	1.09 [1.00-1.19]	1.11 [1.03-1.19]	1.12 [1.03-1.21]	1.10 [1.01-1.20]	1.02 [0.83-1.24]	1.09 [1.00-1.18]	1,672	1,469	1,338	243	1,326
193	1.06 [0.96-1.18]	1.06 [0.95-1.18]	1.06 [0.94-1.20]		1.02 [0.91-1.15]	1.04 [0.93-1.16]	1.02 [0.92-1.14]	1.03 [0.92-1.17]		1.01 [0.90-1.14]	819	798	618	NA	675
194	1.15 [0.95-1.38]	1.06 [0.87-1.30]	1.14 [0.93-1.41]		1.05 [0.87-1.27]	1.13 [0.93-1.35]	1.01 [0.82-1.25]	1.08 [0.87-1.34]		1.05 [0.87-1.28]	281	228	209	NA	249
195	1.11 [1.03-1.19]	1.12 [1.04-1.20]	1.12 [1.04-1.20]		1.07 [0.99-1.16]	1.08 [1.00-1.16]	1.07 [0.99-1.15]	1.07 [0.99-1.15]		1.06 [0.98-1.14]	1,820	1,800	1,741	NA	1,597
195.1	1.16 [1.15-1.18]	1.16 [1.15-1.18]	1.16 [1.15-1.18]	1.09 [1.01-1.18]	1.10 [1.08-1.11]	1.13 [1.11-1.14]	1.10 [1.09-1.11]	1.10 [1.09-1.11]	1.09 [1.01-1.18]	1.09 [1.08-1.10]	80,474	79,523	75,993	1,693	72,445
195.3	1.28 [1.01-1.62]	1.28 [1.00-1.64]	1.21 [0.95-1.55]		1.30 [1.00-1.68]	1.29 [1.02-1.64]	1.26 [0.98-1.62]	1.19 [0.92-1.53]		1.34 [1.03-1.73]	172	161	158	NA	150
197	1.27 [1.25-1.29]	1.28 [1.26-1.30]	1.27 [1.24-1.29]	1.16 [1.10-1.22]	1.19 [1.17-1.21]	1.20 [1.18-1.22]	1.17 [1.15-1.19]	1.16 [1.14-1.18]	1.12 [1.06-1.18]	1.15 [1.13-1.17]	40,144	37,469	31,884	4,078	34,553
198	1.07 [1.04-1.09]	1.06 [1.04-1.08]	1.06 [1.04-1.09]	1.01 [0.78-1.30]	1.05 [1.03-1.07]	1.07 [1.05-1.09]	1.04 [1.02-1.06]	1.05 [1.02-1.07]	1.01 [0.78-1.31]	1.07 [1.05-1.09]	22,768	22,653	22,271	164	19,792
198.1	1.06 [1.03-1.09]	1.05 [1.03-1.08]	1.05 [1.03-1.08]	1.14 [0.86-1.51]	1.04 [1.01-1.07]	1.06 [1.04-1.09]	1.04 [1.01-1.07]	1.04 [1.01-1.07]	1.14 [0.86-1.53]	1.05 [1.02-1.08]	14,180	14,120	13,563	140	11,924
198.2	1.06 [1.03-1.09]	1.05 [1.02-1.08]	1.05 [1.02-1.09]	1.03 [0.76-1.40]	1.05 [1.01-1.08]	1.07 [1.04-1.10]	1.03 [1.00-1.07]	1.04 [1.01-1.07]	1.05 [0.77-1.44]	1.07 [1.04-1.11]	10,415	10,342	10,137	109	9,342
198.3	1.05 [1.01-1.10]	1.04 [1.00-1.09]	1.05 [1.01-1.10]		1.04 [1.00-1.09]	1.06 [1.02-1.10]	1.04 [0.99-1.08]	1.04 [1.00-1.09]		1.06 [1.01-1.10]	5,766	5,739	5,623	NA	4,965
198.4	1.05 [1.03-1.08]	1.04 [1.02-1.07]	1.06 [1.03-1.09]		1.05 [1.01-1.08]	1.06 [1.03-1.09]	1.03 [1.00-1.06]	1.04 [1.01-1.07]		1.06 [1.03-1.10]	12,201	12,178	11,983	NA	10,663
198.5	1.12 [1.06-1.18]	1.11 [1.06-1.17]	1.13 [1.08-1.19]		1.10 [1.04-1.16]	1.13 [1.07-1.19]	1.08 [1.03-1.14]	1.11 [1.05-1.17]		1.13 [1.07-1.19]	3,672	3,626	3,549	NA	3,245
198.6	1.07 [1.04-1.10]	1.07 [1.04-1.10]	1.08 [1.05-1.11]	1.06 [0.77-1.45]	1.05 [1.02-1.08]	1.07 [1.04-1.11]	1.05 [1.02-1.08]	1.05 [1.02-1.09]	1.10 [0.79-1.51]	1.08 [1.04-1.11]	11,056	10,974	10,800	107	9,843
198.7	1.01 [0.90-1.13]	1.00 [0.89-1.13]	0.99 [0.88-1.12]		0.96 [0.86-1.09]	1.02 [0.91-1.15]	0.99 [0.88-1.11]	0.98 [0.87-1.11]		0.99 [0.88-1.12]	694	688	665	NA	638
199	1.10 [1.06-1.15]	1.11 [1.06-1.15]	1.12 [1.07-1.16]	0.96 [0.80-1.14]	1.05 [1.00-1.09]	1.08 [1.04-1.13]	1.06 [1.02-1.11]	1.07 [1.03-1.12]	0.94 [0.78-1.13]	1.05 [1.00-1.09]	6,264	6,052	5,681	319	5,352
199.4	0.94 [0.78-1.12]	0.91 [0.72-1.15]	0.89 [0.67-1.19]	0.89 [0.69-1.15]	0.87 [0.72-1.06]	0.90 [0.75-1.08]	0.83 [0.65-1.05]	0.83 [0.62-1.11]	0.89 [0.69-1.16]	0.85 [0.70-1.03]	291	163	106	148	255
200	1.27 [1.21-1.34]	1.29 [1.22-1.35]	1.26 [1.20-1.33]	1.30 [0.99-1.71]	1.20 [1.14-1.27]	1.23 [1.16-1.29]	1.21 [1.15-1.28]	1.20 [1.13-1.26]	1.27 [0.96-1.68]	1.18 [1.12-1.25]	3,886	3,804	3,634	147	3,493
200.1	1.10 [0.99-1.23]	1.13 [1.01-1.25]	1.06 [0.96-1.19]		1.06 [0.95-1.19]	1.09 [0.98-1.21]	1.06 [0.95-1.18]	1.00 [0.89-1.11]		1.04 [0.93-1.17]	839	837	776	NA	704
201	1.71 [1.54-1.90]	1.72 [1.54-1.91]	1.62 [1.42-1.85]	1.41 [1.11-1.79]	1.46 [1.30-1.65]	1.64 [1.48-1.83]	1.65 [1.48-1.85]	1.52 [1.33-1.74]	1.33 [1.04-1.71]	1.43 [1.26-1.61]	969	870	588	185	701
202	1.33 [1.26-1.40]	1.32 [1.26-1.40]	1.31 [1.24-1.38]	1.19 [0.94-1.49]	1.22 [1.16-1.28]	1.26 [1.20-1.33]	1.22 [1.16-1.28]	1.19 [1.12-1.25]	1.19 [0.94-1.51]	1.18 [1.12-1.24]	3,821	3,718	3,407	197	3,596
202.2	1.32 [1.27-1.38]	1.33 [1.27-1.39]	1.30 [1.24-1.36]	1.29 [1.01-1.66]	1.25 [1.19-1.31]	1.29 [1.23-1.35]	1.29 [1.23-1.35]	1.25 [1.19-1.31]	1.30 [1.00-1.67]	1.23 [1.17-1.30]	4,824	4,724	4,442	168	3,988
202.21	1.16 [1.04-1.29]	1.10 [0.99-1.23]	1.10 [0.98-1.23]		1.04 [0.93-1.17]	1.10 [0.99-1.23]	1.04 [0.93-1.16]	1.05 [0.94-1.17]		1.02 [0.90-1.15]	790	782	744	NA	658
202.24	1.31 [1.22-1.42]	1.32 [1.22-1.43]	1.29 [1.19-1.39]		1.24 [1.14-1.35]	1.27 [1.18-1.37]	1.28 [1.19-1.39]	1.24 [1.15-1.34]		1.22 [1.12-1.33]	1,690	1,672	1,575	NA	1,437
204	1.19 [1.10-1.28]	1.30 [1.20-1.41]	1.29 [1.19-1.41]	0.90 [0.74-1.10]	1.11 [1.03-1.20]	1.15 [1.07-1.25]	1.23 [1.13-1.34]	1.20 [1.10-1.31]	0.93 [0.76-1.14]	1.09 [1.01-1.18]	1,708	1,506	1,380	250	1,559
204.1	1.07 [1.00-1.15]	1.12 [1.04-1.21]	1.09 [1.02-1.18]	0.97 [0.81-1.15]	1.04 [0.97-1.12]	1.05 [0.99-1.13]	1.08 [1.01-1.17]	1.05 [0.98-1.13]	0.97 [0.81-1.15]	1.03 [0.95-1.11]	2,123	1,828	1,763	321	1,756
204.11	1.00 [0.87-1.16]	1.23 [0.98-1.54]	1.22 [0.95-1.58]	0.94 [0.79-1.12]	0.97 [0.82-1.13]	0.99 [0.86-1.14]	1.18 [0.94-1.49]	1.15 [0.89-1.50]	0.94 [0.79-1.13]	0.96 [0.82-1.13]	470	184	142	307	369
204.12	1.04 [0.96-1.13]	1.07 [0.99-1.16]	1.04 [0.96-1.12]		1.01 [0.93-1.10]	1.02 [0.94-1.11]	1.03 [0.95-1.12]	1.00 [0.92-1.08]		1.00 [0.91-1.09]	1,512	1,480	1,464	NA	1,269
204.2	1.28 [1.18-1.38]	1.29 [1.20-1.39]	1.28 [1.19-1.39]	1.09 [0.81-1.49]	1.24 [1.15-1.35]	1.25 [1.16-1.35]	1.26 [1.16-1.36]	1.25 [1.15-1.35]	1.10 [0.80-1.51]	1.24 [1.14-1.35]	1,752	1,680	1,574	107	1,488
204.21	1.32 [1.21-1.44]	1.33 [1.22-1.45]	1.33 [1.22-1.46]		1.29 [1.17-1.41]	1.30 [1.19-1.42]	1.30 [1.19-1.42]	1.30 [1.19-1.43]		1.29 [1.17-1.41]	1,349	1,282	1,203	NA	1,151
204.22	1.17 [1.00-1.37]	1.21 [1.03-1.41]	1.16 [0.98-1.36]		1.16 [0.98-1.37]	1.14 [0.97-1.33]	1.16 [0.99-1.36]	1.11 [0.94-1.31]		1.16 [0.98-1.38]	413	406	374	NA	353
204.3	1.17 [0.94-1.46]	1.18 [0.95-1.47]	1.10 [0.88-1.37]		1.11 [0.89-1.40]	1.19 [0.95-1.48]	1.14 [0.91-1.42]	1.07 [0.85-1.34]		1.15 [0.91-1.44]	216	213	211	NA	199
204.4	1.16 [1.09-1.24]	1.17 [1.10-1.25]	1.17 [1.09-1.24]		1.11 [1.04-1.19]	1.13 [1.06-1.21]	1.14 [1.07-1.22]	1.13 [1.06-1.21]		1.09 [1.02-1.17]	2,358	2,351	2,322	NA	2,008
208	1.23 [1.22-1.25]	1.23 [1.22-1.25]	1.22 [1.20-1.24]	1.38 [1.20-1.60]	1.15 [1.13-1.17]	1.16 [1.14-1.17]	1.12 [1.10-1.14]	1.11 [1.09-1.13]	1.30 [1.12-1.51]	1.11 [1.09-1.12]	43,766	43,635	41,594	536	37,979

Supplementary Table 6: phecode-mapped results (hazard ratios and events from all cohorts)

	Hazard ratio (99% confidence interval)														
	crude					adjusted					Events (in exposed)				
Outcome	any age	18+	40+	<18	hosp.	any age	18+	40+	<18	hosp.	any age	18+	40+	<18	hosp.
210	1.15 [1.07-1.24]	1.17 [1.08-1.26]	1.16 [1.07-1.26]	1.09 [0.88-1.35]	1.13 [1.04-1.23]	1.12 [1.04-1.21]	1.08 [1.00-1.17]	1.08 [0.99-1.17]	1.09 [0.88-1.36]	1.11 [1.02-1.21]	1,714	1,585	1,262	221	1,388
211	1.28 [1.24-1.31]	1.28 [1.24-1.31]	1.27 [1.24-1.31]	1.06 [0.81-1.39]	1.18 [1.15-1.21]	1.15 [1.11-1.18]	1.12 [1.09-1.15]	1.11 [1.08-1.14]	1.00 [0.76-1.33]	1.09 [1.06-1.12]	13,365	13,319	12,827	139	12,195
212	1.25 [1.13-1.40]	1.25 [1.12-1.40]	1.26 [1.11-1.42]	1.31 [0.97-1.79]	1.13 [1.00-1.27]	1.20 [1.08-1.34]	1.15 [1.02-1.29]	1.18 [1.04-1.34]	1.25 [0.91-1.72]	1.10 [0.98-1.24]	835	772	632	108	677
213	1.18 [1.07-1.29]	1.27 [1.13-1.43]	1.20 [1.03-1.40]	1.07 [0.94-1.22]	1.08 [0.98-1.21]	1.14 [1.03-1.25]	1.18 [1.04-1.33]	1.09 [0.93-1.28]	1.06 [0.92-1.21]	1.05 [0.94-1.17]	1,123	647	390	604	878
214	1.16 [1.12-1.20]	1.16 [1.12-1.20]	1.13 [1.09-1.18]	1.13 [0.98-1.30]	1.07 [1.03-1.11]	1.10 [1.07-1.14]	1.08 [1.05-1.12]	1.06 [1.02-1.10]	1.07 [0.93-1.23]	1.03 [0.99-1.07]	7,797	7,547	6,147	516	6,248
214.1	1.16 [1.12-1.22]	1.16 [1.11-1.21]	1.13 [1.07-1.18]	1.20 [1.02-1.41]	1.06 [1.01-1.11]	1.11 [1.06-1.16]	1.09 [1.04-1.14]	1.06 [1.01-1.11]	1.13 [0.95-1.33]	1.03 [0.98-1.08]	5,078	4,904	3,769	379	3,941
215	1.23 [1.14-1.32]	1.25 [1.16-1.34]	1.22 [1.13-1.33]	1.07 [0.87-1.32]	1.14 [1.05-1.23]	1.16 [1.07-1.25]	1.15 [1.07-1.24]	1.12 [1.03-1.22]	1.04 [0.84-1.29]	1.09 [1.01-1.18]	1,812	1,661	1,316	235	1,475
216	1.18 [1.15-1.21]	1.20 [1.16-1.23]	1.18 [1.14-1.23]	1.14 [1.09-1.20]	1.10 [1.07-1.13]	1.13 [1.10-1.16]	1.12 [1.09-1.16]	1.11 [1.07-1.15]	1.11 [1.05-1.16]	1.07 [1.03-1.10]	13,503	10,708	6,986	3,861	10,387
217	1.19 [1.00-1.41]			1.16 [0.96-1.40]	1.16 [0.96-1.41]	1.17 [0.98-1.39]			1.15 [0.95-1.39]	1.14 [0.94-1.39]	338	NA	NA	287	271
217.1	1.34 [1.24-1.45]	1.36 [1.25-1.47]	1.33 [1.23-1.45]		1.28 [1.18-1.39]	1.27 [1.17-1.38]	1.24 [1.14-1.35]	1.23 [1.13-1.34]		1.25 [1.15-1.36]	1,597	1,510	1,419	NA	1,467
218.1	1.07 [1.04-1.10]	1.08 [1.05-1.11]	1.09 [1.06-1.12]	1.14 [0.89-1.44]	1.06 [1.03-1.09]	1.04 [1.01-1.07]	1.05 [1.02-1.08]	1.05 [1.02-1.08]	1.12 [0.87-1.44]	1.03 [1.00-1.06]	12,552	12,549	10,372	190	9,894
218.2	1.12 [0.96-1.29]	1.10 [0.95-1.27]	1.08 [0.92-1.27]		1.09 [0.93-1.29]	1.09 [0.94-1.27]	1.08 [0.93-1.25]	1.04 [0.88-1.23]		1.07 [0.90-1.26]	424	420	330	NA	342
220	1.04 [0.98-1.10]	1.06 [1.00-1.12]	1.04 [0.97-1.11]	1.07 [0.90-1.28]	1.03 [0.96-1.09]	1.02 [0.96-1.08]	1.01 [0.96-1.07]	0.99 [0.93-1.07]	1.04 [0.87-1.25]	1.01 [0.95-1.07]	2,900	2,818	1,877	349	2,332
221	1.25 [1.06-1.47]	1.26 [1.06-1.49]	1.14 [0.93-1.40]		1.20 [1.00-1.44]	1.19 [1.00-1.41]	1.19 [1.00-1.41]	1.11 [0.90-1.37]		1.17 [0.97-1.40]	347	332	218	NA	287
222	1.23 [1.02-1.49]	1.21 [0.99-1.49]	1.14 [0.92-1.42]		1.17 [0.95-1.44]	1.19 [0.98-1.45]	1.16 [0.95-1.43]	1.10 [0.89-1.38]		1.14 [0.92-1.40]	263	232	208	NA	225
223	1.11 [0.96-1.28]	1.08 [0.94-1.25]	1.08 [0.94-1.26]		1.03 [0.88-1.20]	1.08 [0.93-1.25]	1.02 [0.88-1.18]	1.03 [0.89-1.20]		1.01 [0.87-1.17]	450	443	422	NA	400
224	1.38 [1.15-1.64]	1.39 [1.12-1.72]	1.34 [1.04-1.72]	1.31 [0.99-1.73]	1.41 [1.16-1.71]	1.34 [1.12-1.61]	1.28 [1.03-1.60]	1.26 [0.98-1.63]	1.27 [0.95-1.70]	1.39 [1.14-1.69]	319	214	154	132	270
224.1	1.17 [0.97-1.43]	1.21 [0.99-1.48]	1.21 [0.99-1.48]		1.16 [0.94-1.43]	1.16 [0.95-1.41]	1.18 [0.96-1.44]	1.15 [0.94-1.42]		1.14 [0.92-1.40]	258	249	244	NA	232
225.1	1.13 [1.06-1.21]	1.09 [1.02-1.17]	1.11 [1.04-1.19]	1.39 [1.02-1.89]	1.08 [1.00-1.15]	1.08 [1.01-1.15]	1.02 [0.95-1.09]	1.04 [0.97-1.12]	1.33 [0.97-1.83]	1.05 [0.98-1.13]	2,253	2,182	2,041	112	2,001
225.2	1.12 [0.85-1.49]	1.13 [0.84-1.50]				1.05 [0.79-1.40]	1.06 [0.78-1.42]				121	112	NA	NA	NA
226	1.07 [0.93-1.24]	1.04 [0.90-1.21]	1.04 [0.89-1.23]		1.06 [0.91-1.24]	1.03 [0.88-1.19]	0.98 [0.84-1.14]	1.00 [0.85-1.19]		1.01 [0.86-1.19]	432	419	333	NA	381
227.1	1.11 [0.99-1.23]	1.11 [1.00-1.23]	1.11 [1.00-1.24]		1.04 [0.93-1.16]	1.05 [0.94-1.17]	0.99 [0.89-1.10]	0.99 [0.88-1.10]		1.02 [0.91-1.14]	848	845	808	NA	769
227.2	1.08 [0.97-1.21]	1.07 [0.96-1.19]	1.04 [0.93-1.16]		1.00 [0.89-1.12]	1.05 [0.94-1.17]	1.02 [0.91-1.14]	1.00 [0.90-1.13]		0.98 [0.87-1.10]	776	774	722	NA	665
227.3	1.21 [1.09-1.35]	1.22 [1.10-1.35]	1.24 [1.10-1.40]		1.05 [0.94-1.18]	1.16 [1.04-1.29]	1.15 [1.04-1.28]	1.17 [1.03-1.32]		1.03 [0.91-1.15]	891	868	670	NA	734
228	1.17 [1.11-1.23]	1.16 [1.10-1.23]	1.19 [1.12-1.26]	1.14 [1.02-1.28]	1.11 [1.05-1.17]	1.11 [1.05-1.17]	1.07 [1.01-1.13]	1.09 [1.02-1.16]	1.13 [1.01-1.27]	1.07 [1.01-1.13]	3,729	3,083	2,567	790	3,237
229	1.21 [1.15-1.27]	1.20 [1.14-1.27]	1.18 [1.11-1.26]	1.11 [0.99-1.24]	1.11 [1.05-1.17]	1.15 [1.09-1.20]	1.12 [1.06-1.19]	1.10 [1.03-1.17]	1.07 [0.95-1.20]	1.07 [1.01-1.14]	3,836	3,264	2,316	811	3,041
229.1	1.66 [1.22-2.26]					1.59 [1.16-2.17]					103	NA	NA	NA	NA
860	1.24 [1.13-1.37]	1.30 [1.17-1.44]	1.27 [1.13-1.42]	1.07 [0.86-1.33]	1.15 [1.04-1.27]	1.21 [1.10-1.34]	1.25 [1.12-1.39]	1.22 [1.09-1.37]	1.08 [0.86-1.35]	1.16 [1.05-1.28]	1,036	882	709	215	945
endocrine/metabolic															
240	1.14 [1.06-1.23]	1.12 [1.03-1.21]	1.07 [0.99-1.16]		1.11 [1.02-1.20]	1.08 [1.00-1.17]	1.02 [0.95-1.11]	0.99 [0.91-1.08]		1.07 [0.99-1.16]	1,654	1,603	1,419	NA	1,455
241.1	1.15 [1.06-1.24]	1.13 [1.05-1.22]	1.14 [1.05-1.24]		1.07 [0.99-1.16]	1.10 [1.02-1.19]	1.06 [0.98-1.15]	1.07 [0.98-1.16]		1.04 [0.96-1.14]	1,583	1,550	1,318	NA	1,388
241.2	1.15 [1.07-1.24]	1.15 [1.07-1.24]	1.15 [1.06-1.24]		1.10 [1.02-1.19]	1.09 [1.01-1.17]	1.07 [1.00-1.16]	1.07 [0.99-1.15]		1.07 [0.99-1.15]	1,836	1,827	1,669	NA	1,617
242	1.29 [1.25-1.34]	1.29 [1.24-1.33]	1.27 [1.22-1.32]	1.28 [1.09-1.50]	1.21 [1.17-1.25]	1.23 [1.18-1.27]	1.18 [1.14-1.22]	1.17 [1.12-1.21]	1.24 [1.05-1.46]	1.16 [1.12-1.21]	8,314	8,168	6,931	419	7,289
242.1	1.37 [1.27-1.47]	1.36 [1.26-1.46]	1.36 [1.25-1.48]	1.36 [1.07-1.74]	1.28 [1.18-1.38]	1.30 [1.21-1.40]	1.24 [1.15-1.34]	1.25 [1.14-1.36]	1.38 [1.07-1.78]	1.23 [1.14-1.33]	1,916	1,856	1,320	182	1,654
242.2	1.11 [0.95-1.29]	1.10 [0.94-1.28]	1.07 [0.92-1.25]		1.02 [0.87-1.19]	1.07 [0.92-1.25]	1.03 [0.88-1.21]	1.00 [0.85-1.18]		0.98 [0.84-1.16]	424	424	389	NA	369
242.3	1.47 [1.22-1.78]	1.42 [1.18-1.72]	1.46 [1.19-1.80]		1.35 [1.11-1.65]	1.43 [1.18-1.73]	1.25 [1.03-1.53]	1.35 [1.09-1.67]		1.32 [1.07-1.62]	277	274	229	NA	237
244.1	1.24 [1.18-1.30]	1.25 [1.19-1.32]	1.24 [1.18-1.31]	1.13 [0.82-1.56]	1.16 [1.10-1.22]	1.16 [1.11-1.22]	1.14 [1.08-1.20]	1.12 [1.06-1.18]	1.10 [0.79-1.55]	1.11 [1.06-1.17]	4,029	3,999	3,602	103	3,734
244.2	1.29 [1.16-1.44]	1.31 [1.17-1.47]	1.27 [1.13-1.43]	1.54 [1.15-2.07]	1.22 [1.09-1.36]	1.23 [1.10-1.37]	1.23 [1.09-1.37]	1.19 [1.05-1.34]	1.44 [1.06-1.95]	1.18 [1.05-1.32]	923	831	723	132	841
244.4	1.34 [1.32-1.36]	1.34 [1.32-1.36]	1.33 [1.31-1.35]	1.20 [1.11-1.31]	1.26 [1.24-1.28]	1.28 [1.26-1.30]	1.25 [1.23-1.26]	1.24 [1.22-1.26]	1.18 [1.08-1.28]	1.22 [1.20-1.24]	50,313	49,752	44,667	1,561	41,359
244.5	1.17 [0.98-1.41]	1.42 [1.14-1.77]	1.38 [1.08-1.75]		1.11 [0.91-1.35]	1.10 [0.91-1.33]	1.28 [1.02-1.60]	1.23 [0.96-1.57]		1.04 [0.85-1.27]	286	221	178	NA	253
245	1.22 [1.10-1.35]	1.22 [1.10-1.36]	1.24 [1.10-1.40]		1.20 [1.07-1.34]	1.17 [1.05-1.30]	1.13 [1.01-1.26]	1.16 [1.02-1.31]		1.14 [1.02-1.28]	883	841	636	NA	787
245.21	1.25 [1.11-1.41]	1.26 [1.11-1.43]	1.27 [1.10-1.46]		1.24 [1.09-1.41]	1.21 [1.07-1.37]	1.17 [1.03-1.33]	1.19 [1.03-1.38]		1.18 [1.04-1.35]	654	621	460	NA	579
246	1.26 [1.16-1.37]	1.30 [1.19-1.41]	1.28 [1.17-1.40]		1.18 [1.08-1.29]	1.17 [1.07-1.27]	1.18 [1.08-1.29]	1.16 [1.06-1.27]		1.11 [1.02-1.22]	1,422	1,394	1,233	NA	1,293
246.7	1.14 [0.96-1.35]	1.16 [0.97-1.40]	1.22 [1.00-1.49]		1.10 [0.92-1.31]	1.07 [0.90-1.28]	1.07 [0.89-1.29]	1.11 [0.91-1.36]		1.06 [0.89-1.27]	360	310	269	NA	329
250	1.36 [1.33-1.40]	1.37 [1.34-1.41]	1.36 [1.33-1.40]	0.90 [0.74-1.11]	1.27 [1.23-1.30]	1.26 [1.23-1.30]	1.18 [1.15-1.22]	1.17 [1.13-1.20]	0.91 [0.74-1.12]	1.22 [1.18-1.25]	14,114	14,014	13,328	236	12,768
250.1	1.23 [1.19-1.26]	1.30 [1.25-1.34]	1.32 [1.27-1.36]	0.97 [0.91-1.04]	1.14 [1.11-1.18]	1.15 [1.12-1.19]	1.12 [1.09-1.16]	1.13 [1.09-1.17]	0.96 [0.90-1.03]	1.11 [1.07-1.15]	10,587	8,646	7,458	2,324	8,645
250.11	1.09 [1.02-1.17]	1.21 [1.11-1.31]	1.26 [1.12-1.41]	0.91 [0.83-1.00]	0.99 [0.92-1.06]	1.05 [0.98-1.12]	1.08 [0.98-1.17]	1.06 [0.95-1.20]	0.94 [0.85-1.03]	0.99 [0.91-1.06]	2,084	1,291	718	1,077	1,809
250.12	1.51 [1.32-1.73]	1.61 [1.40-1.85]	1.71 [1.47-1.98]		1.32 [1.15-1.51]	1.39 [1.21-1.60]	1.34 [1.16-1.54]	1.40 [1.19-1.63]		1.29 [1.12-1.49]	527	525	450	NA	501
250.13	1.30 [1.20-1.40]	1.33 [1.24-1.43]	1.32 [1.22-1.43]		1.17 [1.09-1.27]	1.21 [1.13-1.31]	1.13 [1.05-1.22]	1.11 [1.02-1.21]		1.16 [1.08-1.26]	1,764	1,755	1,438	NA	1,616
250.14	1.52 [1.36-1.71]	1.52 [1.35-1.70]	1.67 [1.47-1.88]		1.30 [1.16-1.45]	1.38 [1.23-1.55]	1.22 [1.08-1.37]	1.30 [1.14-1.48]		1.25 [1.11-1.40]	762	763	667	NA	739
250.2	1.26 [1.24-1.27]	1.26 [1.25-1.28]	1.25 [1.24-1.26]	1.12 [1.00-1.26]	1.20 [1.18-1.22]	1.19 [1.18-1.21]	1.13 [1.11-1.14]	1.11 [1.10-1.13]	1.10 [0.98-1.23]	1.16 [1.15-1.18]	76,947	76,654	73,593	821	60,531
250.21	1.19 [1.09-1.29]	1.18 [1.08-1.28]	1.16 [1.06-1.26]		1.09 [1.00-1.19]	1.12 [1.03-1.22]	1.03 [0.94-1.12]	1.00 [0.91-1.09]		1.08 [0.99-1.18]	1,328	1,311	1,230	NA	1,202
250.22	1.45 [1.36-1.55]	1.43 [1.34-1.53]	1.43 [1.34-1.53]		1.28 [1.20-1.37]	1.35 [1.26-1.44]	1.22 [1.14-1.31]	1.20 [1.12-1.29]		1.26 [1.18-1.35]	2,350	2,348	2,312	NA	2,274
250.23	1.24 [1.20-1.28]	1.25 [1.21-1.28]	1.24 [1.20-1.28]	1.09 [0.79-1.49]	1.16 [1.13-1.20]	1.17 [1.14-1.21]	1.09 [1.06-1.13]	1.08 [1.04-1.11]	1.14 [0.82-1.58]	1.14 [1.11-1.18]	10,348	10,339	9,952	102	9,448
250.24	1.41 [1.34-1.48]	1.41 [1.34-1.48]	1.43 [1.36-1.50]		1.28 [1.22-1.35]	1.27 [1.21-1.34]	1.14 [1.08-1.20]	1.16 [1.10-1.22]		1.21 [1.15-1.27]	4,276	4,270	4,152	NA	4,098
250.3	1.21 [1.03-1.43]	1.30 [1.09-1.56]	1.43 [1.17-1.76]		1.14 [0.96-1.34]	1.10 [0.93-1.30]	1.11 [0.92-1.34]	1.19 [0.96-1.48]		1.11 [0.93-1.31]	380	304	247	NA	363
250.41	1.34 [1.27-1.42]	1.34 [1.27-1.42]	1.34 [1.26-1.43]	1.33 [1.01-1.77]	1.29 [1.21-1.37]	1.24 [1.17-1.32]	1.21 [1.13-1.28]	1.20 [1.12-1.28]	1.29 [0.96-1.72]	1.22 [1.15-1.30]	3,035	2,933	2,683	150	2,778
250.42	1.28 [1.20-1.36]	1.29 [1.20-1.38]	1.28 [1.19-1.37]	1.15 [0.97-1.37]	1.21 [1.14-1.29]	1.18 [1.11-1.26]	1.13 [1.06-1.21]	1.12 [1.04-1.21]	1.08 [0.90-1.29]	1.16 [1.08-1.24]	2,477	2,209	1,978	337	2,265
250.5	1.19 [1.06-1.34]	1.16 [1.02-1.33]	1.10 [0.82-1.47]	1.16 [0.96-1.41]	1.14 [0.99-1.30]	1.16 [1.02-1.31]	1.09 [0.95-1.25]	1.01 [0.75-1.36]	1.16 [0.95-1.42]	1.12 [0.98-1.29]	674	512	106	271	537
250.6	1.46 [1.38-1.54]	1.45 [1.37-1.53]	1.47 [1.39-1.55]		1.33 [1.26-1.40]	1.31 [1.24-1.38]	1.16 [1.10-1.23]	1.17 [1.11-1.24]		1.24 [1.18-1.31]	3,551	3,548	3,456	NA	3,443
250.7	1.25 [1.21-1.29]	1.25 [1.21-1.29]	1.24 [1.20-1.28]	1.07 [0.78-1.46]	1.17 [1.13-1.21]	1.18 [1.14-1.22]	1.09 [1.06-1.13]	1.08 [1.05-1.12]	1.11 [0.81-1.53]	1.15 [1.11-1.19]	9,859	9,848	9,450	104	8,998
251	1.25 [1.22-1.29]	1.27 [1.24-1.31]	1.27 [1.23-1.31]	1.07 [0.99-1.15]	1.16 [1.13-1.20]	1.18 [1.14-1.21]	1.11 [1.08-1.14]	1.10 [1.07-1.14]	1.05 [0.97-1.14]	1.14 [1.10-1.17]	12,636	11,294	10,504	1,633	11,955
251.1	1.25 [1.22-1.29]	1.27 [1.24-1.31]	1.27 [1.23-1.31]	1.06 [0.98-1.15]	1.16 [1.13-1.20]	1.18 [1.14-1.21]	1.11 [1.08-1.14]	1.10 [1.07-1.14]	1.05 [0.97-1.14]	1.14 [1.10-1.17]	12,590	11,257	10,471	1,622	11,910
252.1	1.17 [1.11-1.23]	1.17 [1.11-1.23]	1.16 [1.10-1.22]		1.09 [1.03-1.15]	1.12 [1.06-1.18]	1.08 [1.02-1.14]	1.07 [1.02-1.14]		1.06 [1.00-1.12]	3,538	3,509	3,366	NA	3,236
252.2	1.40 [1.25-1.57]	1.42 [1.27-1.60]	1.42 [1.26-1.60]		1.26 [1.13-1.41]	1.27 [1.13-1.43]	1.26 [1.12-1.41]	1.26 [1.12-1.43]		1.20 [1.07-1.35]	818	784	706	NA	772
253	1.39 [1.20-1.62]	1.39 [1.19-1.64]	1.47 [1.22-1.76]		1.35 [1.15-1.57]	1.29 [1.11-1.50]	1.23 [1.04-1.46]	1.31 [1.09-1.58]		1.30 [1.10-1.52]	460	388	304	NA	418
253.1	1.47 [1.39-1.56]	1.46 [1.38-1.55]	1.50 [1.41-1.60]	1.33 [1.06-1.68]	1.39 [1.30-1.47]	1.40 [1.32-1.49]	1.36 [1.28-1.44]	1.39 [1.31-1.49]	1.27 [1.00-1.61]	1.34 [1.26-1.43]	3,085	2,955	2,737	205	2,799
253.11	1.27 [1.00-1.60]	1.23 [0.93-1.63]			1.01 [0.78-1.30]	1.17 [0.92-1.49]	1.13 [0.84-1.50]			0.98 [0.75-1.27]	181	118	NA	NA	145
253.2	1.44 [1.30-1.58]	1.54 [1.38-1.73]	1.57 [1.38-1.79]	1.31 [1.11-1.54]	1.24 [1.12-1.37]	1.34 [1.22-1.48]	1.36 [1.21-1.53]	1.41 [1.24-1.61]	1.24 [1.05-1.47]	1.19 [1.08-1.32]	1,120	777	605	415	1,010
253.3	1.44 [1.22-1.70]	1.43 [1.19-1.72]	1.61 [1.30-1.99]		1.30 [1.09-1.55]	1.35 [1.14-1.61]	1.29 [1.06-1.57]	1.43 [1.15-1.77]		1.24 [1.03-1.48]	360	282	227	NA	313
253.7	1.52 [1.42-1.63]	1.52 [1.41-1.62]	1.52 [1.42-1.64]		1.44 [1.34-1.55]	1.46 [1.36-1.57]	1.42 [1.33-1.53]	1.43 [1.33-1.54]		1.40 [1.30-1.51]	2,343	2,311	2,288	NA	2,147
255	1.23 [1.13-1.34]	1.22 [1.11-1.34]	1.17 [1.06-1.28]		1.17 [1.06-1.28]	1.18 [1.08-1.29]	1.12 [1.02-1.23]	1.06 [0.96-1.17]		1.15 [1.05-1.26]	1,257	1,192	1,101	NA	1,161
255.11	1.60 [1.35-1.90]	1.46 [1.22-1.74]	1.61 [1.33-1.96]		1.40 [1.17-1.67]	1.32 [1.10-1.58]	1.12 [0.93-1.36]	1.24 [1.01-1.52]		1.21 [1.01-1.45]	357	319	263	NA	327
255.12	1.21 [0.95-1.54]	1.16 [0.91-1.49]	1.14 [0.87-1.50]		1.16 [0.90-1.50]	1.13 [0.88-1.44]	1.04 [0.80-1.34]	0.97 [0.73-1.29]		1.14 [0.88-1.48]	163	150	124	NA	142
255.21	1.72 [1.60-1.85]	1.73 [1.60-1.87]	1.75 [1.61-1.90]	1.45 [1.21-1.74]	1.55 [1.44-1.67]	1.47 [1.36-1.58]	1.43 [1.32-1.55]	1.45 [1.33-1.59]	1.31 [1.08-1.58]	1.38 [1.28-1.49]	2,070	1,833	1,510	338	1,932
256	1.20 [1.16-1.25]	1.24 [1.19-1.28]	1.25 [1.14-1.38]	1.13 [1.06-1.21]	1.17 [1.12-1.22]	1.10 [1.06-1.14]	1.07 [1.03-1.12]	1.04 [0.94-1.15]	1.10 [1.03-1.18]	1.10 [1.06-1.15]	7,246	6,975	990	2,350	5,906
256.4	1.21 [1.16-1.26]	1.24 [1.19-1.29]	1.27 [1.15-1.41]	1.14 [1.06-1.22]	1.17 [1.12-1.22]	1.10 [1.06-1.15]	1.07 [1.03-1.12]	1.05 [0.94-1.16]	1.10 [1.03-1.18]	1.10 [1.06-1.15]	6,957	6,700	902	2,307	5,676
257	1.71 [1.47-1.98]	1.76 [1.51-2.05]	1.76 [1.50-2.08]		1.45 [1.25-1.69]	1.50 [1.28-1.75]	1.42 [1.21-1.67]	1.40 [1.18-1.66]		1.32 [1.13-1.55]	466	445	385	NA	430
257.1	1.71 [1.47-2.00]	1.76 [1.51-2.06]	1.77 [1.50-2.09]		1.47 [1.26-1.71]	1.50 [1.28-1.75]	1.43 [1.21-1.68]	1.40 [1.18-1.67]		1.34 [1.14-1.57]	449	430	373	NA	417
258	1.47 [1.22-1.79]	1.45 [1.19-1.77]	1.45 [1.17-1.79]		1.23 [1.01-1.49]	1.33 [1.09-1.63]	1.24 [1.01-1.53]	1.24 [0.99-1.54]		1.18 [0.97-1.44]	272	253	221	NA	255
259	1.25 [1.15-1.35]	1.35 [1.16-1.57]	1.46 [1.21-1.77]	1.20 [1.09-1.32]	1.17 [1.08-1.28]	1.16 [1.06-1.26]	1.17 [1.00-1.37]	1.28 [1.05-1.56]	1.12 [1.02-1.23]	1.12 [1.03-1.23]	1,544	426	270	1,169	1,378
259.1	1.43 [1.16-1.77]	1.40 [1.11-1.76]	1.27 [0.97-1.67]		1.35 [1.08-1.69]	1.25 [1.00-1.56]	1.21 [0.95-1.54]	1.10 [0.83-1.46]		1.24 [0.98-1.57]	227	188	129	NA	208
259.3	1.18 [0.98-1.43]			1.16 [0.95-1.40]	1.00 [0.81-1.23]	1.16 [0.95-1.41]			1.12 [0.92-1.37]	1.00 [0.81-1.23]	297	NA	NA	295	257
259.4	1.22 [1.06-1.41]			1.22 [1.05-1.40]	1.20 [1.03-1.40]	1.17 [1.01-1.35]			1.16 [1.00-1.34]	1.17 [1.00-1.36]	521	NA	NA	516	449
260	1.21 [1.13-1.30]	1.18 [1.10-1.27]	1.16 [1.08-1.25]	1.31 [0.99-1.74]	1.16 [1.08-1.24]	1.19 [1.11-1.28]	1.10 [1.03-1.19]	1.09 [1.01-1.18]	1.27 [0.95-1.71]	1.16 [1.08-1.25]	2,081	2,012	1,832	139	1,930
260.1	1.16 [1.05-1.28]	1.12 [1.01-1.24]	1.10 [1.00-1.22]		1.09 [0.98-1.21]	1.17 [1.06-1.30]	1.09 [0.99-1.21]	1.08 [0.97-1.20]		1.12 [1.01-1.25]	1,044	1,034	1,014	NA	970
260.22	1.07 [0.80-1.42]	1.05 [0.78-1.41]	1.10 [0.82-1.48]		1.15 [0.86-1.55]	1.10 [0.82-1.47]	1.06 [0.78-1.42]	1.09 [0.81-1.48]		1.20 [0.89-1.61]	123	121	113	NA	118
260.6	1.21 [1.17-1.26]	1.21 [1.16-1.26]	1.23 [1.18-1.28]	1.07 [0.98-1.17]	1.15 [1.11-1.20]	1.16 [1.12-1.21]	1.12 [1.07-1.16]	1.15 [1.10-1.19]	1.03 [0.94-1.13]	1.13 [1.09-1.18]	7,715	6,704	5,932	1,361	6,978
261	1.27 [1.00-1.62]	1.29 [1.00-1.67]	1.21 [0.92-1.60]		1.21 [0.94-1.54]	1.19 [0.93-1.53]	1.14 [0.87-1.48]	1.10 [0.83-1.46]		1.18 [0.92-1.52]	176	155	132	NA	165
261.2	1.31 [1.27-1.35]	1.31 [1.27-1.35]	1.29 [1.25-1.33]	1.26 [1.11-1.44]	1.24 [1.20-1.28]	1.23 [1.20-1.27]	1.18 [1.15-1.22]	1.17 [1.13-1.21]	1.19 [1.03-1.36]	1.20 [1.16-1.23]	12,428	12,315	10,879	652	11,582
261.4	1.37 [1.34-1.40]	1.38 [1.34-1.42]	1.35 [1.32-1.39]	1.34 [1.25-1.45]	1.30 [1.26-1.33]	1.26 [1.23-1.29]	1.22 [1.19-1.25]	1.20 [1.17-1.24]	1.26 [1.17-1.35]	1.23 [1.19-1.26]	18,051	16,575	14,208	2,279	16,868
261.41	1.33 [1.11-1.60]	1.24 [1.01-1.52]	1.29 [1.04-1.60]		1.31 [1.08-1.58]	1.22 [1.02-1.47]	1.13 [0.91-1.39]	1.17 [0.93-1.46]		1.23 [1.02-1.50]	306	242	217	NA	285
262	1.40 [1.32-1.49]	1.40 [1.31-1.49]	1.40 [1.30-1.50]	1.39 [1.20-1.61]	1.29 [1.22-1.38]	1.28 [1.20-1.36]	1.23 [1.15-1.31]	1.23 [1.15-1.32]	1.31 [1.12-1.52]	1.22 [1.14-1.30]	2,987	2,637	2,192	540	2,778
263	1.21 [1.02-1.44]	1.10 [0.92-1.31]	1.28 [1.06-1.55]		1.13 [0.95-1.35]	1.17 [0.98-1.40]	1.01 [0.84-1.21]	1.20 [0.99-1.46]		1.12 [0.94-1.34]	345	308	278	NA	325
264.3	0.98 [0.85-1.14]			1.00 [0.86-1.16]	0.90 [0.77-1.05]	0.93 [0.80-1.08]			0.96 [0.83-1.11]	0.88 [0.75-1.03]	434	NA	NA	435	401
264.9	1.08 [1.03-1.14]	1.10 [0.94-1.28]	1.25 [1.01-1.55]	1.07 [1.01-1.13]	1.01 [0.95-1.07]	1.03 [0.98-1.09]	1.00 [0.86-1.18]	1.17 [0.94-1.46]	1.03 [0.97-1.09]	0.98 [0.93-1.04]	3,528	408	227	3,186	3,157
269	1.33 [1.23-1.45]	1.38 [1.26-1.52]	1.44 [1.29-1.60]	1.17 [0.99-1.39]	1.24 [1.13-1.36]	1.28 [1.18-1.40]	1.26 [1.14-1.39]	1.30 [1.16-1.44]	1.16 [0.98-1.38]	1.21 [1.10-1.32]	1,442	1,128	889	396	1,232
270.1	1.25 [0.99-1.57]	1.33 [0.99-1.80]			1.20 [0.93-1.54]	1.20 [0.95-1.52]	1.22 [0.90-1.67]			1.17 [0.90-1.51]	188	108	NA	NA	158
270.32	1.35 [1.25-1.46]	1.36 [1.26-1.47]	1.36 [1.26-1.47]		1.28 [1.18-1.38]	1.27 [1.17-1.37]	1.25 [1.16-1.35]	1.23 [1.14-1.33]		1.23 [1.14-1.33]	1,905	1,906	1,896	NA	1,811
270.33	1.25 [1.10-1.41]	1.22 [1.07-1.38]	1.18 [1.04-1.35]		1.18 [1.04-1.34]	1.20 [1.06-1.36]	1.14 [1.00-1.29]	1.10 [0.96-1.25]		1.14 [1.00-1.30]	655	623	590	NA	596
270.35	1.35 [1.11-1.64]	1.28 [1.06-1.56]	1.36 [1.12-1.66]		1.24 [1.01-1.52]	1.32 [1.08-1.60]	1.26 [1.03-1.53]	1.32 [1.08-1.62]		1.22 [0.99-1.50]	267	266	265	NA	240
271.3	1.39 [1.27-1.52]	1.37 [1.22-1.54]	1.33 [1.16-1.53]	1.35 [1.19-1.53]	1.31 [1.19-1.44]	1.22 [1.11-1.34]	1.13 [1.00-1.28]	1.11 [0.96-1.29]	1.22 [1.07-1.39]	1.17 [1.06-1.28]	1,313	728	486	679	1,157
271.9	1.36 [1.09-1.69]	1.40 [1.05-1.87]		1.26 [0.94-1.70]	1.31 [1.05-1.63]	1.28 [1.02-1.60]	1.28 [0.95-1.73]		1.23 [0.91-1.68]	1.27 [1.01-1.60]	227	118	NA	123	206
272.1	1.27 [1.23-1.30]	1.26 [1.23-1.30]	1.26 [1.23-1.29]		1.17 [1.14-1.20]	1.19 [1.16-1.22]	1.12 [1.10-1.15]	1.12 [1.09-1.15]		1.13 [1.10-1.16]	16,512	16,499	16,297	NA	14,971
272.11	1.23 [1.22-1.24]	1.23 [1.22-1.24]	1.23 [1.22-1.24]	1.19 [0.96-1.48]	1.15 [1.14-1.17]	1.17 [1.16-1.18]	1.11 [1.10-1.13]	1.11 [1.10-1.12]	1.17 [0.94-1.47]	1.12 [1.11-1.14]	90,425	90,380	89,053	219	77,085
272.12	1.58 [1.34-1.86]	1.69 [1.43-2.00]	1.54 [1.30-1.84]		1.46 [1.23-1.73]	1.41 [1.19-1.68]	1.46 [1.23-1.74]	1.33 [1.11-1.60]		1.40 [1.17-1.66]	388	381	336	NA	352
272.13	1.40 [1.23-1.59]	1.44 [1.26-1.63]	1.42 [1.25-1.61]		1.30 [1.14-1.48]	1.26 [1.11-1.44]	1.16 [1.02-1.33]	1.18 [1.03-1.35]		1.21 [1.06-1.38]	639	636	622	NA	582
272.9	1.26 [1.16-1.36]	1.26 [1.17-1.37]	1.31 [1.21-1.42]		1.17 [1.08-1.28]	1.16 [1.07-1.26]	1.09 [1.01-1.19]	1.13 [1.04-1.23]		1.12 [1.03-1.22]	1,554	1,553	1,527	NA	1,455
274.1	1.36 [1.33-1.39]	1.36 [1.33-1.39]	1.36 [1.33-1.39]		1.27 [1.24-1.30]	1.29 [1.26-1.32]	1.24 [1.21-1.27]	1.23 [1.20-1.26]		1.22 [1.19-1.25]	19,181	19,179	18,861	NA	17,324
274.11	1.31 [1.15-1.48]	1.33 [1.17-1.51]	1.26 [1.11-1.44]		1.21 [1.06-1.38]	1.26 [1.11-1.44]	1.24 [1.09-1.41]	1.16 [1.02-1.33]		1.19 [1.04-1.36]	640	643	625	NA	591
274.2	1.33 [1.17-1.50]	1.31 [1.16-1.48]	1.37 [1.21-1.55]		1.30 [1.15-1.47]	1.25 [1.10-1.41]	1.19 [1.05-1.35]	1.25 [1.11-1.42]		1.25 [1.10-1.42]	781	781	774	NA	744
274.21	1.31 [1.22-1.42]	1.32 [1.23-1.43]	1.29 [1.20-1.39]		1.25 [1.16-1.35]	1.22 [1.13-1.32]	1.20 [1.11-1.29]	1.17 [1.08-1.26]		1.18 [1.10-1.28]	1,911	1,885	1,775	NA	1,754
275	1.21 [1.18-1.24]	1.22 [1.19-1.25]	1.20 [1.17-1.23]	1.08 [0.96-1.20]	1.15 [1.12-1.18]	1.16 [1.13-1.19]	1.12 [1.09-1.15]	1.10 [1.08-1.13]	1.06 [0.95-1.19]	1.13 [1.10-1.16]	17,831	17,352	16,193	875	16,376
275.3	1.25 [1.20-1.30]	1.26 [1.21-1.32]	1.25 [1.20-1.30]	1.02 [0.83-1.26]	1.19 [1.14-1.24]	1.17 [1.13-1.22]	1.12 [1.07-1.17]	1.11 [1.06-1.16]	1.01 [0.82-1.25]	1.16 [1.11-1.21]	6,400	6,267	5,950	249	6,060
275.5	1.18 [1.14-1.22]	1.18 [1.15-1.22]	1.17 [1.13-1.21]	0.96 [0.81-1.15]	1.13 [1.09-1.16]	1.14 [1.10-1.18]	1.10 [1.07-1.14]	1.09 [1.05-1.12]	0.95 [0.79-1.13]	1.12 [1.08-1.16]	10,042	9,830	9,362	334	9,210
275.53	1.25 [1.18-1.32]	1.27 [1.20-1.34]	1.23 [1.16-1.30]	1.14 [0.95-1.36]	1.16 [1.09-1.22]	1.19 [1.12-1.26]	1.16 [1.09-1.22]	1.12 [1.05-1.19]	1.13 [0.94-1.35]	1.13 [1.07-1.20]	3,624	3,453	3,070	337	3,359
276.1	1.20 [1.11-1.30]	1.23 [1.14-1.33]	1.22 [1.13-1.33]	1.18 [0.88-1.57]	1.18 [1.09-1.28]	1.13 [1.04-1.22]	1.11 [1.02-1.20]	1.10 [1.01-1.20]	1.11 [0.82-1.49]	1.16 [1.07-1.26]	1,686	1,622	1,478	125	1,596
276.11	1.15 [1.10-1.20]	1.16 [1.11-1.21]	1.17 [1.12-1.22]	0.87 [0.69-1.09]	1.12 [1.07-1.17]	1.14 [1.09-1.19]	1.11 [1.06-1.16]	1.12 [1.07-1.17]	0.84 [0.67-1.06]	1.13 [1.07-1.18]	5,363	5,226	5,096	190	5,020
276.12	1.38 [1.36-1.41]	1.38 [1.36-1.41]	1.38 [1.36-1.41]	0.99 [0.86-1.14]	1.32 [1.29-1.35]	1.33 [1.30-1.35]	1.28 [1.25-1.30]	1.28 [1.25-1.30]	0.99 [0.86-1.14]	1.29 [1.26-1.31]	29,451	29,107	28,537	500	26,962
276.13	1.29 [1.25-1.33]	1.29 [1.25-1.32]	1.28 [1.25-1.32]	0.97 [0.79-1.20]	1.21 [1.17-1.24]	1.24 [1.21-1.28]	1.18 [1.15-1.22]	1.17 [1.14-1.21]	0.96 [0.78-1.19]	1.20 [1.16-1.23]	13,239	13,106	12,753	237	12,419
276.14	1.22 [1.20-1.25]	1.23 [1.20-1.26]	1.22 [1.19-1.25]	1.12 [1.02-1.24]	1.17 [1.15-1.20]	1.17 [1.14-1.20]	1.13 [1.10-1.16]	1.12 [1.09-1.15]	1.10 [0.99-1.22]	1.15 [1.12-1.18]	21,009	20,373	19,152	1,090	19,482
276.41	1.24 [1.21-1.27]	1.24 [1.21-1.27]	1.24 [1.21-1.28]	1.04 [0.94-1.15]	1.18 [1.15-1.21]	1.18 [1.15-1.21]	1.12 [1.09-1.15]	1.12 [1.09-1.15]	1.02 [0.92-1.13]	1.15 [1.12-1.18]	16,781	16,079	15,262	999	15,629
276.42	1.27 [1.17-1.37]	1.29 [1.19-1.39]	1.27 [1.17-1.37]	0.93 [0.71-1.22]	1.18 [1.10-1.28]	1.18 [1.10-1.28]	1.14 [1.05-1.24]	1.13 [1.04-1.23]	0.86 [0.65-1.14]	1.13 [1.04-1.22]	1,779	1,711	1,536	138	1,654
276.5	1.20 [1.18-1.21]	1.19 [1.17-1.21]	1.19 [1.17-1.21]	1.13 [1.08-1.18]	1.14 [1.12-1.16]	1.16 [1.14-1.18]	1.12 [1.10-1.14]	1.12 [1.10-1.14]	1.10 [1.05-1.15]	1.13 [1.11-1.15]	47,668	43,859	41,005	4,893	43,955
276.6	1.32 [1.28-1.37]	1.33 [1.29-1.37]	1.32 [1.28-1.37]	1.02 [0.80-1.29]	1.26 [1.22-1.30]	1.26 [1.22-1.30]	1.20 [1.17-1.24]	1.19 [1.15-1.23]	1.02 [0.79-1.30]	1.22 [1.19-1.27]	10,985	10,880	10,645	184	10,552
276.8	1.34 [1.20-1.50]	1.53 [1.32-1.79]	1.59 [1.34-1.89]	1.14 [0.97-1.33]	1.24 [1.10-1.39]	1.26 [1.13-1.41]	1.37 [1.17-1.60]	1.38 [1.15-1.65]	1.09 [0.93-1.28]	1.17 [1.04-1.32]	808	442	348	400	694
277	1.22 [1.19-1.26]	1.24 [1.20-1.28]	1.22 [1.18-1.26]	1.16 [1.09-1.24]	1.16 [1.12-1.19]	1.17 [1.14-1.21]	1.15 [1.11-1.19]	1.14 [1.10-1.18]	1.13 [1.06-1.21]	1.14 [1.10-1.17]	12,880	10,907	9,674	2,445	11,980
277.2	1.37 [1.05-1.78]	1.38 [1.06-1.82]	1.44 [1.07-1.93]		1.23 [0.93-1.62]	1.31 [1.00-1.70]	1.24 [0.94-1.64]	1.25 [0.92-1.70]		1.17 [0.88-1.54]	149	137	116	NA	131
277.4	1.16 [1.06-1.27]	1.20 [1.10-1.32]	1.20 [1.07-1.34]	1.11 [0.91-1.36]	1.07 [0.97-1.18]	1.09 [0.99-1.19]	1.11 [1.00-1.22]	1.12 [1.00-1.25]	1.04 [0.84-1.28]	1.02 [0.92-1.12]	1,218	1,090	769	256	1,055
277.5	1.30 [1.18-1.43]	1.37 [1.23-1.51]	1.37 [1.23-1.53]	1.00 [0.78-1.27]	1.22 [1.10-1.34]	1.21 [1.10-1.34]	1.19 [1.07-1.32]	1.19 [1.06-1.33]	0.97 [0.76-1.25]	1.17 [1.06-1.30]	1,100	974	865	169	1,035
277.51	1.57 [1.31-1.88]	1.59 [1.31-1.92]	1.70 [1.39-2.08]		1.39 [1.15-1.68]	1.48 [1.23-1.78]	1.39 [1.14-1.70]	1.50 [1.22-1.85]		1.35 [1.11-1.64]	305	284	257	NA	280
278.1	1.30 [1.29-1.32]	1.32 [1.30-1.33]	1.32 [1.31-1.34]	1.18 [1.14-1.22]	1.22 [1.21-1.24]	1.20 [1.19-1.21]	1.15 [1.14-1.17]	1.15 [1.13-1.16]	1.13 [1.10-1.17]	1.15 [1.14-1.17]	88,230	86,031	60,487	11,250	77,698
278.3	1.45 [1.08-1.95]	1.54 [1.14-2.08]				1.33 [0.98-1.80]	1.39 [1.02-1.90]				113	107	NA	NA	NA
278.4	1.51 [1.30-1.76]	1.51 [1.26-1.81]	1.53 [1.25-1.89]	1.48 [1.16-1.88]	1.39 [1.19-1.62]	1.36 [1.17-1.59]	1.26 [1.04-1.52]	1.28 [1.03-1.59]	1.37 [1.06-1.76]	1.28 [1.09-1.50]	482	318	233	203	448
279	1.66 [1.31-2.09]	1.62 [1.23-2.13]	1.55 [1.13-2.14]		1.50 [1.18-1.90]	1.47 [1.15-1.87]	1.33 [0.99-1.78]	1.30 [0.93-1.82]		1.39 [1.09-1.77]	204	141	105	NA	190
279.1	1.52 [1.38-1.67]	1.57 [1.41-1.76]	1.49 [1.32-1.68]	1.45 [1.22-1.72]	1.44 [1.30-1.59]	1.34 [1.21-1.48]	1.33 [1.18-1.49]	1.23 [1.09-1.40]	1.31 [1.10-1.56]	1.30 [1.18-1.44]	1,161	828	681	384	1,085
279.11	1.51 [1.34-1.70]	1.54 [1.35-1.77]	1.48 [1.28-1.71]	1.46 [1.16-1.82]	1.44 [1.27-1.62]	1.33 [1.18-1.51]	1.31 [1.14-1.51]	1.23 [1.05-1.44]	1.32 [1.05-1.66]	1.30 [1.15-1.47]	744	565	463	216	696
279.7	1.37 [1.21-1.55]	1.32 [1.15-1.52]	1.37 [1.17-1.60]	1.32 [1.02-1.70]	1.28 [1.12-1.47]	1.29 [1.14-1.47]	1.22 [1.06-1.41]	1.26 [1.08-1.48]	1.27 [0.98-1.65]	1.23 [1.07-1.41]	648	521	418	168	549
279.8	1.64 [1.31-2.06]	1.66 [1.28-2.16]	1.49 [1.10-2.02]		1.67 [1.32-2.11]	1.49 [1.18-1.89]	1.48 [1.13-1.95]	1.32 [0.97-1.81]		1.53 [1.20-1.95]	207	151	109	NA	193
hematopoietic															
275.1	1.30 [1.20-1.42]	1.32 [1.21-1.43]	1.29 [1.18-1.42]		1.22 [1.12-1.34]	1.26 [1.16-1.38]	1.25 [1.15-1.37]	1.23 [1.12-1.35]		1.20 [1.10-1.32]	1,332	1,290	1,110	NA	1,125
280.1	1.32 [1.31-1.34]	1.32 [1.30-1.34]	1.32 [1.30-1.34]	1.28 [1.21-1.35]	1.25 [1.23-1.27]	1.25 [1.23-1.27]	1.20 [1.18-1.22]	1.20 [1.18-1.22]	1.24 [1.18-1.31]	1.20 [1.19-1.22]	53,162	50,841	45,714	4,007	47,985
280.2	1.22 [1.15-1.30]	1.21 [1.14-1.28]	1.23 [1.16-1.31]	1.11 [0.90-1.36]	1.15 [1.08-1.22]	1.18 [1.12-1.26]	1.14 [1.08-1.21]	1.16 [1.09-1.24]	1.10 [0.89-1.37]	1.13 [1.06-1.20]	3,120	3,000	2,489	254	2,730
281.1	1.16 [0.87-1.53]	1.18 [0.88-1.57]	1.29 [0.95-1.74]		0.94 [0.68-1.28]	1.14 [0.85-1.52]	1.09 [0.81-1.46]	1.23 [0.90-1.67]		0.93 [0.67-1.28]	125	122	114	NA	104
281.11	1.41 [1.33-1.48]	1.39 [1.32-1.47]	1.40 [1.32-1.48]		1.33 [1.25-1.40]	1.31 [1.23-1.38]	1.25 [1.18-1.32]	1.26 [1.19-1.33]		1.26 [1.18-1.33]	3,472	3,466	3,227	NA	3,117
281.12	1.38 [1.32-1.44]	1.35 [1.29-1.42]	1.36 [1.30-1.43]	1.43 [1.07-1.91]	1.29 [1.23-1.35]	1.29 [1.23-1.35]	1.23 [1.17-1.29]	1.24 [1.18-1.31]	1.33 [0.99-1.80]	1.23 [1.17-1.29]	4,681	4,667	4,277	132	4,210
281.13	1.28 [1.21-1.35]	1.31 [1.24-1.38]	1.30 [1.23-1.38]		1.25 [1.18-1.32]	1.23 [1.17-1.31]	1.22 [1.15-1.29]	1.20 [1.14-1.28]		1.24 [1.17-1.31]	3,437	3,417	3,254	NA	3,181

Supplementary Table 6: phecode-mapped results (hazard ratios and events from all cohorts)

	Hazard ratio (99% confidence interval)										Events (in exposed)				
	crude					adjusted									
Outcome	any age	18+	40+	<18	hosp.	any age	18+	40+	<18	hosp.	any age	18+	40+	<18	hosp.
281.9	1.48 [1.32-1.66]	1.46 [1.30-1.64]	1.56 [1.38-1.76]		1.42 [1.26-1.60]	1.39 [1.23-1.56]	1.32 [1.17-1.49]	1.38 [1.22-1.57]		1.37 [1.22-1.55]	835	811	786	NA	785
282.5	1.31 [1.23-1.40]	1.37 [1.27-1.47]	1.30 [1.17-1.44]	1.36 [1.23-1.51]	1.22 [1.13-1.31]	1.28 [1.20-1.37]	1.32 [1.22-1.43]	1.23 [1.10-1.37]	1.36 [1.22-1.51]	1.21 [1.12-1.30]	2,519	1,759	859	1,054	1,979
282.8	1.38 [1.29-1.48]	1.41 [1.32-1.52]	1.51 [1.38-1.66]	1.31 [1.14-1.51]	1.26 [1.17-1.35]	1.35 [1.26-1.44]	1.36 [1.27-1.47]	1.40 [1.27-1.54]	1.29 [1.12-1.49]	1.25 [1.16-1.34]	2,393	2,059	1,188	572	1,987
282.9	1.27 [1.13-1.43]	1.30 [1.14-1.49]	1.28 [1.09-1.50]	1.22 [0.98-1.52]	1.22 [1.08-1.39]	1.25 [1.11-1.41]	1.24 [1.08-1.43]	1.21 [1.03-1.43]	1.23 [0.98-1.54]	1.22 [1.07-1.39]	710	538	390	216	618
283	1.44 [1.28-1.62]	1.47 [1.30-1.67]	1.43 [1.25-1.64]	1.27 [0.96-1.67]	1.36 [1.20-1.54]	1.42 [1.26-1.59]	1.43 [1.25-1.63]	1.38 [1.20-1.58]	1.25 [0.94-1.67]	1.36 [1.20-1.55]	745	639	566	133	654
283.1	1.57 [1.36-1.81]	1.58 [1.36-1.83]	1.50 [1.29-1.75]		1.48 [1.27-1.72]	1.53 [1.33-1.77]	1.55 [1.33-1.80]	1.44 [1.24-1.69]		1.47 [1.26-1.72]	517	490	444	NA	455
284	1.31 [1.24-1.39]	1.33 [1.25-1.41]	1.30 [1.22-1.38]	1.08 [0.89-1.31]	1.25 [1.18-1.32]	1.27 [1.20-1.35]	1.26 [1.19-1.33]	1.22 [1.15-1.30]	1.05 [0.86-1.28]	1.24 [1.17-1.31]	3,302	3,131	2,874	259	3,032
285	1.30 [1.28-1.31]	1.30 [1.29-1.32]	1.30 [1.29-1.32]	1.17 [1.11-1.23]	1.23 [1.21-1.24]	1.24 [1.23-1.26]	1.21 [1.19-1.22]	1.21 [1.19-1.22]	1.14 [1.08-1.21]	1.20 [1.18-1.21]	69,443	67,794	61,959	3,476	62,148
285.1	1.13 [1.04-1.23]	1.13 [1.04-1.23]	1.18 [1.05-1.33]	0.99 [0.79-1.23]	1.07 [0.98-1.18]	1.12 [1.03-1.22]	1.10 [1.01-1.20]	1.12 [0.99-1.26]	0.98 [0.78-1.23]	1.08 [0.99-1.19]	1,430	1,401	686	211	1,170
285.2	1.43 [1.34-1.52]	1.43 [1.34-1.52]	1.41 [1.32-1.50]		1.30 [1.22-1.38]	1.35 [1.27-1.44]	1.28 [1.20-1.37]	1.25 [1.17-1.34]		1.27 [1.19-1.36]	2,731	2,710	2,583	NA	2,620
285.22	1.14 [1.07-1.22]	1.15 [1.07-1.23]	1.13 [1.06-1.21]		1.10 [1.02-1.18]	1.14 [1.06-1.22]	1.13 [1.06-1.21]	1.11 [1.04-1.19]		1.11 [1.03-1.20]	2,146	2,101	2,044	NA	1,845
286.1	1.85 [1.41-2.43]	1.82 [1.32-2.51]			1.46 [1.09-1.97]	1.65 [1.25-2.19]	1.53 [1.09-2.14]			1.41 [1.04-1.91]	148	104	NA	NA	117
286.11	1.26 [1.06-1.50]	1.38 [1.13-1.69]	1.32 [1.00-1.74]	1.17 [0.91-1.52]	1.18 [0.98-1.43]	1.15 [0.96-1.37]	1.19 [0.96-1.47]	1.14 [0.85-1.52]	1.11 [0.85-1.44]	1.11 [0.91-1.35]	334	231	116	158	269
286.12	1.22 [1.09-1.36]	1.27 [1.13-1.43]	1.35 [1.16-1.57]	1.06 [0.81-1.38]	1.11 [0.99-1.25]	1.11 [0.99-1.25]	1.12 [0.99-1.26]	1.16 [0.99-1.36]	0.98 [0.75-1.28]	1.03 [0.92-1.17]	744	663	399	152	652
286.13	1.15 [0.94-1.40]	1.13 [0.90-1.43]	1.18 [0.90-1.57]		1.20 [0.97-1.50]	1.11 [0.90-1.36]	1.03 [0.81-1.31]	1.09 [0.82-1.45]		1.17 [0.94-1.47]	242	177	121	NA	204
286.3	1.05 [0.94-1.17]	1.04 [0.94-1.16]		0.96 [0.76-1.22]	0.98 [0.88-1.10]	1.03 [0.92-1.15]	1.00 [0.90-1.12]		0.94 [0.74-1.20]	0.98 [0.87-1.10]	820	811	NA	176	673
286.5	1.31 [1.11-1.54]	1.35 [1.15-1.59]	1.34 [1.13-1.58]		1.11 [0.93-1.32]	1.25 [1.06-1.47]	1.24 [1.05-1.46]	1.24 [1.04-1.46]		1.07 [0.90-1.27]	370	371	361	NA	327
286.6	1.28 [1.22-1.35]	1.32 [1.25-1.39]	1.33 [1.25-1.41]	1.09 [0.97-1.24]	1.18 [1.13-1.25]	1.19 [1.13-1.25]	1.18 [1.12-1.24]	1.18 [1.11-1.26]	1.03 [0.91-1.17]	1.13 [1.07-1.19]	4,176	3,783	2,765	675	3,675
286.7	1.29 [1.22-1.35]	1.32 [1.25-1.39]	1.34 [1.26-1.42]	1.09 [0.96-1.23]	1.19 [1.13-1.25]	1.19 [1.13-1.25]	1.18 [1.11-1.24]	1.18 [1.11-1.26]	1.03 [0.90-1.17]	1.13 [1.07-1.19]	3,974	3,599	2,602	650	3,500
287	1.23 [1.20-1.27]	1.27 [1.23-1.31]	1.28 [1.24-1.33]	1.06 [1.00-1.13]	1.18 [1.14-1.21]	1.20 [1.16-1.23]	1.20 [1.16-1.24]	1.20 [1.16-1.25]	1.06 [1.00-1.13]	1.17 [1.13-1.20]	11,780	9,406	8,184	2,747	10,458
287.1	1.18 [1.11-1.24]	1.32 [1.17-1.49]	1.42 [1.24-1.61]	1.14 [1.07-1.21]	1.12 [1.06-1.19]	1.13 [1.07-1.20]	1.20 [1.07-1.36]	1.30 [1.14-1.48]	1.11 [1.04-1.18]	1.09 [1.02-1.16]	3,222	720	613	2,545	2,877
287.2	1.10 [1.01-1.19]	1.32 [1.07-1.63]	1.44 [1.10-1.89]	1.05 [0.97-1.15]	1.11 [1.02-1.21]	1.08 [1.00-1.17]	1.22 [0.98-1.51]	1.29 [0.98-1.70]	1.05 [0.97-1.15]	1.10 [1.01-1.20]	1,587	218	136	1,406	1,341
287.3	1.26 [1.22-1.30]	1.27 [1.22-1.31]	1.29 [1.24-1.34]	1.07 [0.96-1.20]	1.20 [1.15-1.24]	1.22 [1.18-1.27]	1.20 [1.16-1.25]	1.21 [1.16-1.26]	1.06 [0.95-1.19]	1.19 [1.15-1.23]	8,323	7,736	6,795	855	7,552
287.31	1.25 [1.16-1.34]	1.30 [1.20-1.41]	1.31 [1.20-1.43]	1.12 [0.97-1.30]	1.18 [1.09-1.27]	1.21 [1.12-1.30]	1.23 [1.13-1.34]	1.23 [1.12-1.34]	1.10 [0.95-1.28]	1.16 [1.07-1.26]	1,872	1,487	1,252	453	1,617
287.32	1.39 [1.20-1.62]	1.34 [1.15-1.57]	1.34 [1.14-1.58]		1.27 [1.08-1.48]	1.35 [1.16-1.58]	1.25 [1.07-1.47]	1.24 [1.05-1.46]		1.28 [1.09-1.50]	452	420	376	NA	404
288	1.26 [1.20-1.34]	1.26 [1.19-1.34]	1.25 [1.18-1.33]	1.22 [1.06-1.40]	1.20 [1.13-1.27]	1.20 [1.13-1.26]	1.15 [1.08-1.22]	1.15 [1.08-1.23]	1.18 [1.02-1.36]	1.15 [1.08-1.22]	3,404	3,053	2,603	521	3,043
288.11	1.23 [1.19-1.27]	1.24 [1.20-1.28]	1.23 [1.19-1.28]	1.14 [1.04-1.24]	1.18 [1.14-1.22]	1.20 [1.16-1.24]	1.19 [1.15-1.23]	1.19 [1.15-1.23]	1.11 [1.02-1.22]	1.17 [1.13-1.21]	9,777	8,724	7,840	1,334	8,508
288.2	1.43 [1.34-1.53]	1.44 [1.35-1.55]	1.40 [1.30-1.51]	1.41 [1.18-1.68]	1.35 [1.26-1.45]	1.31 [1.22-1.40]	1.27 [1.18-1.37]	1.25 [1.16-1.35]	1.31 [1.10-1.58]	1.26 [1.17-1.35]	2,421	2,176	1,866	358	2,199
288.3	2.09 [1.82-2.40]	2.13 [1.84-2.46]	2.08 [1.77-2.45]	2.02 [1.49-2.76]	1.95 [1.69-2.25]	1.82 [1.58-2.10]	1.75 [1.50-2.05]	1.77 [1.49-2.09]	1.71 [1.23-2.36]	1.72 [1.49-2.00]	636	546	440	138	573
289	1.25 [1.18-1.31]	1.25 [1.18-1.31]	1.25 [1.18-1.32]	1.19 [0.91-1.56]	1.18 [1.12-1.25]	1.19 [1.13-1.26]	1.14 [1.08-1.21]	1.15 [1.09-1.22]	1.13 [0.85-1.51]	1.16 [1.09-1.22]	3,575	3,494	3,277	140	3,174
289.3	1.34 [1.26-1.43]	1.35 [1.26-1.44]	1.37 [1.26-1.48]	1.22 [1.06-1.41]	1.22 [1.14-1.30]	1.25 [1.17-1.33]	1.22 [1.14-1.31]	1.22 [1.13-1.32]	1.19 [1.03-1.38]	1.16 [1.09-1.24]	2,606	2,270	1,648	551	2,423
289.4	1.33 [1.30-1.36]	1.35 [1.32-1.39]	1.33 [1.28-1.37]	1.25 [1.21-1.30]	1.25 [1.22-1.29]	1.29 [1.26-1.32]	1.26 [1.23-1.30]	1.25 [1.21-1.29]	1.22 [1.17-1.26]	1.23 [1.20-1.27]	18,440	11,712	9,577	7,528	15,745
289.5	1.25 [1.17-1.34]	1.24 [1.16-1.32]	1.26 [1.17-1.35]	0.92 [0.72-1.18]	1.16 [1.08-1.24]	1.20 [1.12-1.28]	1.15 [1.07-1.23]	1.16 [1.08-1.25]	0.95 [0.74-1.22]	1.14 [1.07-1.23]	2,222	2,116	1,898	169	2,030
289.8	1.16 [1.06-1.27]	1.13 [1.04-1.23]	1.13 [1.03-1.24]		1.10 [1.00-1.21]	1.12 [1.02-1.22]	1.03 [0.94-1.12]	1.03 [0.94-1.13]		1.09 [1.00-1.20]	1,244	1,232	1,141	NA	1,125
289.9	1.27 [1.07-1.52]	1.17 [0.98-1.41]	1.16 [0.95-1.41]		1.14 [0.94-1.37]	1.21 [1.01-1.45]	1.11 [0.92-1.34]	1.08 [0.88-1.32]		1.12 [0.92-1.35]	313	282	246	NA	273
mental disorders															
290	1.17 [1.14-1.19]	1.17 [1.15-1.19]	1.17 [1.14-1.19]		1.13 [1.10-1.15]	1.14 [1.12-1.17]	1.11 [1.09-1.14]	1.11 [1.08-1.13]		1.12 [1.10-1.14]	26,809	26,795	26,750	NA	24,968
290.1	1.07 [1.05-1.09]	1.06 [1.04-1.08]	1.06 [1.05-1.08]		1.04 [1.02-1.06]	1.06 [1.04-1.08]	1.04 [1.02-1.06]	1.04 [1.02-1.06]		1.04 [1.02-1.06]	34,205	34,185	34,155	NA	31,222
290.11	1.07 [1.04-1.10]	1.07 [1.04-1.10]	1.08 [1.05-1.11]		1.06 [1.03-1.09]	1.06 [1.03-1.09]	1.05 [1.02-1.08]	1.06 [1.03-1.09]		1.06 [1.02-1.09]	13,779	13,770	13,766	NA	12,505
290.12	1.01 [0.91-1.11]	1.01 [0.91-1.11]	1.00 [0.91-1.10]		0.95 [0.86-1.05]	1.03 [0.93-1.13]	1.00 [0.91-1.10]	0.99 [0.90-1.09]		0.98 [0.89-1.08]	1,100	1,101	1,101	NA	1,046
290.16	1.10 [1.07-1.13]	1.10 [1.06-1.13]	1.09 [1.06-1.12]		1.06 [1.03-1.10]	1.08 [1.05-1.12]	1.05 [1.02-1.08]	1.04 [1.00-1.07]		1.06 [1.03-1.09]	11,780	11,779	11,773	NA	11,049
290.2	1.18 [1.15-1.20]	1.18 [1.15-1.20]	1.18 [1.15-1.20]	0.98 [0.76-1.27]	1.14 [1.12-1.17]	1.15 [1.13-1.18]	1.11 [1.09-1.13]	1.11 [1.08-1.13]	0.98 [0.75-1.27]	1.14 [1.11-1.16]	25,766	25,664	25,415	166	24,422
290.3	1.08 [1.00-1.17]	1.09 [1.00-1.18]	1.26 [1.11-1.42]	0.93 [0.81-1.06]	0.97 [0.90-1.06]	1.01 [0.93-1.09]	0.90 [0.82-0.97]	0.99 [0.86-1.13]	0.96 [0.83-1.11]	0.99 [0.91-1.08]	1,605	1,475	563	539	1,395
291.1	1.16 [1.05-1.29]	1.12 [1.00-1.24]	1.28 [1.11-1.47]	0.94 [0.75-1.18]	1.07 [0.95-1.20]	1.13 [1.02-1.26]	1.04 [0.94-1.16]	1.19 [1.04-1.38]	0.93 [0.74-1.18]	1.08 [0.96-1.21]	923	842	497	197	725
291.4	1.17 [1.11-1.23]	1.17 [1.11-1.23]	1.18 [1.12-1.24]	1.03 [0.77-1.38]	1.10 [1.04-1.15]	1.13 [1.08-1.19]	1.10 [1.05-1.16]	1.11 [1.06-1.17]	1.11 [0.82-1.51]	1.09 [1.04-1.15]	4,526	4,455	4,306	120	4,215
291.8	1.25 [1.20-1.31]	1.27 [1.21-1.33]	1.28 [1.22-1.35]	1.08 [0.96-1.21]	1.18 [1.13-1.24]	1.18 [1.12-1.23]	1.13 [1.07-1.19]	1.15 [1.09-1.21]	1.03 [0.92-1.17]	1.14 [1.09-1.20]	5,157	4,582	4,131	734	4,836
292	1.17 [1.16-1.19]	1.18 [1.16-1.20]	1.17 [1.15-1.19]	1.09 [1.01-1.18]	1.14 [1.12-1.16]	1.14 [1.12-1.16]	1.11 [1.09-1.13]	1.11 [1.09-1.13]	1.04 [0.96-1.13]	1.13 [1.11-1.15]	41,977	40,673	39,528	1,764	38,419
292.1	1.17 [1.13-1.20]	1.17 [1.13-1.20]	1.16 [1.13-1.20]	1.09 [0.97-1.22]	1.11 [1.08-1.15]	1.13 [1.09-1.16]	1.08 [1.05-1.12]	1.08 [1.04-1.11]	1.04 [0.92-1.18]	1.10 [1.06-1.13]	11,628	11,115	10,439	719	10,584
292.12	1.29 [1.12-1.48]	1.32 [1.11-1.56]	1.31 [1.03-1.66]	1.16 [0.96-1.40]	1.23 [1.06-1.42]	1.15 [1.00-1.33]	1.10 [0.92-1.32]	1.13 [0.88-1.45]	1.09 [0.90-1.32]	1.16 [1.00-1.35]	570	352	175	309	512
292.2	1.15 [1.09-1.21]	1.14 [1.08-1.21]	1.16 [1.10-1.23]		1.10 [1.04-1.16]	1.12 [1.06-1.18]	1.09 [1.03-1.15]	1.10 [1.04-1.17]		1.09 [1.03-1.15]	3,771	3,754	3,715	NA	3,562
292.3	1.17 [1.12-1.23]	1.18 [1.12-1.24]	1.19 [1.13-1.26]	1.01 [0.88-1.15]	1.12 [1.07-1.18]	1.12 [1.07-1.18]	1.10 [1.04-1.16]	1.11 [1.05-1.17]	0.96 [0.83-1.10]	1.10 [1.04-1.15]	4,779	4,370	3,995	582	4,269
292.4	1.17 [1.15-1.19]	1.17 [1.15-1.19]	1.17 [1.14-1.19]	1.14 [1.01-1.29]	1.14 [1.12-1.16]	1.14 [1.12-1.16]	1.11 [1.09-1.13]	1.10 [1.08-1.12]	1.10 [0.97-1.25]	1.13 [1.11-1.15]	32,842	32,350	31,720	712	30,123
292.6	1.20 [1.14-1.27]	1.22 [1.16-1.29]	1.25 [1.17-1.32]	1.10 [0.99-1.23]	1.14 [1.08-1.21]	1.14 [1.08-1.20]	1.11 [1.05-1.17]	1.14 [1.07-1.21]	1.07 [0.96-1.20]	1.12 [1.06-1.18]	4,107	3,484	2,954	864	3,714
293	1.37 [1.32-1.42]	1.46 [1.37-1.55]	1.46 [1.36-1.57]	1.33 [1.27-1.39]	1.31 [1.25-1.36]	1.28 [1.23-1.33]	1.24 [1.16-1.32]	1.21 [1.12-1.31]	1.26 [1.20-1.32]	1.23 [1.18-1.28]	7,400	2,382	1,762	5,150	6,239
293.1	1.39 [1.33-1.46]	1.33 [1.26-1.41]	1.29 [1.21-1.37]	1.48 [1.37-1.60]	1.32 [1.26-1.39]	1.30 [1.24-1.36]	1.20 [1.13-1.26]	1.18 [1.10-1.25]	1.41 [1.31-1.53]	1.26 [1.20-1.32]	5,012	3,433	2,592	1,903	4,347
295	1.30 [1.02-1.65]	1.37 [1.08-1.75]	1.40 [1.06-1.85]		1.18 [0.92-1.51]	1.18 [0.92-1.51]	1.18 [0.92-1.52]	1.23 [0.93-1.64]		1.12 [0.87-1.44]	180	173	130	NA	166
295.1	1.27 [1.21-1.34]	1.26 [1.20-1.33]	1.31 [1.23-1.39]	1.01 [0.86-1.20]	1.14 [1.07-1.20]	1.25 [1.19-1.32]	1.16 [1.10-1.23]	1.25 [1.17-1.33]	1.05 [0.88-1.25]	1.16 [1.09-1.23]	3,632	3,578	2,596	355	2,819
295.2	1.28 [1.18-1.38]	1.27 [1.17-1.37]	1.31 [1.20-1.43]	0.96 [0.71-1.30]	1.18 [1.09-1.29]	1.22 [1.13-1.33]	1.16 [1.07-1.26]	1.20 [1.10-1.31]	0.95 [0.69-1.29]	1.18 [1.08-1.28]	1,605	1,558	1,321	117	1,411
295.3	1.23 [1.15-1.32]	1.28 [1.19-1.38]	1.40 [1.28-1.53]	0.91 [0.77-1.07]	1.12 [1.04-1.21]	1.17 [1.09-1.26]	1.14 [1.06-1.23]	1.25 [1.14-1.37]	0.92 [0.77-1.09]	1.11 [1.03-1.20]	1,942	1,826	1,197	386	1,665
296	1.36 [1.23-1.50]	1.37 [1.24-1.52]	1.38 [1.22-1.55]	1.18 [0.93-1.48]	1.21 [1.09-1.34]	1.21 [1.09-1.34]	1.13 [1.01-1.26]	1.16 [1.02-1.31]	1.09 [0.85-1.39]	1.13 [1.01-1.25]	1,042	934	691	202	923
296.1	1.30 [1.24-1.36]	1.32 [1.26-1.38]	1.34 [1.26-1.42]	1.07 [0.94-1.22]	1.16 [1.11-1.22]	1.18 [1.13-1.24]	1.08 [1.03-1.14]	1.14 [1.07-1.21]	1.07 [0.94-1.23]	1.12 [1.07-1.18]	4,635	4,532	2,958	616	4,028
296.2	1.37 [1.35-1.38]	1.38 [1.36-1.40]	1.39 [1.37-1.41]	1.14 [1.10-1.17]	1.26 [1.25-1.28]	1.23 [1.22-1.25]	1.13 [1.12-1.14]	1.15 [1.14-1.17]	1.13 [1.09-1.17]	1.20 [1.18-1.21]	83,464	80,758	58,420	11,105	73,790
296.22	1.24 [1.13-1.35]	1.24 [1.13-1.36]	1.22 [1.10-1.35]		1.13 [1.02-1.24]	1.14 [1.03-1.25]	1.04 [0.95-1.15]	1.05 [0.95-1.17]		1.11 [1.01-1.22]	1,145	1,119	882	NA	1,000
297	0.98 [0.83-1.16]	1.08 [0.89-1.31]	1.07 [0.81-1.41]	0.89 [0.69-1.14]	0.91 [0.75-1.09]	0.92 [0.77-1.09]	0.95 [0.77-1.16]	0.96 [0.72-1.27]	0.85 [0.66-1.10]	0.86 [0.71-1.04]	346	239	123	155	264
297.2	1.08 [1.02-1.14]	1.09 [1.02-1.17]	1.27 [1.13-1.41]	1.00 [0.93-1.07]	0.99 [0.93-1.05]	1.01 [0.96-1.07]	0.92 [0.85-0.99]	1.05 [0.94-1.18]	0.98 [0.91-1.06]	0.97 [0.91-1.03]	3,237	1,886	767	1,924	2,653
300	1.27 [1.20-1.34]	1.28 [1.20-1.36]	1.29 [1.20-1.38]	1.20 [1.07-1.35]	1.16 [1.10-1.23]	1.18 [1.11-1.25]	1.11 [1.05-1.19]	1.12 [1.04-1.21]	1.18 [1.05-1.33]	1.13 [1.06-1.20]	3,259	2,730	1,951	846	2,914
300.1	1.34 [1.33-1.36]	1.36 [1.34-1.37]	1.36 [1.34-1.39]	1.16 [1.13-1.20]	1.25 [1.23-1.27]	1.21 [1.19-1.22]	1.12 [1.10-1.13]	1.14 [1.12-1.15]	1.14 [1.10-1.17]	1.17 [1.16-1.19]	66,705	63,188	43,294	11,799	59,643
300.11	1.37 [1.26-1.49]	1.37 [1.26-1.49]	1.32 [1.18-1.47]	1.31 [1.08-1.59]	1.22 [1.12-1.33]	1.22 [1.12-1.34]	1.12 [1.02-1.23]	1.09 [0.97-1.22]	1.32 [1.08-1.62]	1.14 [1.04-1.25]	1,455	1,347	823	315	1,256
300.12	1.29 [1.25-1.34]	1.30 [1.25-1.34]	1.30 [1.24-1.36]	1.13 [1.03-1.23]	1.22 [1.17-1.26]	1.17 [1.13-1.21]	1.08 [1.04-1.12]	1.08 [1.03-1.13]	1.11 [1.01-1.22]	1.14 [1.10-1.19]	7,923	7,372	5,281	1,355	7,048
300.13	1.26 [1.21-1.30]	1.30 [1.25-1.35]	1.30 [1.24-1.36]	1.13 [1.05-1.21]	1.19 [1.14-1.24]	1.16 [1.12-1.21]	1.13 [1.09-1.18]	1.14 [1.08-1.19]	1.08 [1.01-1.16]	1.13 [1.09-1.18]	7,880	6,570	4,367	2,286	6,833
300.3	1.64 [1.52-1.76]	1.67 [1.54-1.80]	1.84 [1.65-2.06]	1.25 [1.09-1.43]	1.47 [1.36-1.58]	1.43 [1.32-1.54]	1.33 [1.22-1.44]	1.53 [1.37-1.72]	1.15 [1.00-1.32]	1.34 [1.24-1.46]	2,087	1,795	888	635	1,802
300.4	1.52 [1.26-1.83]	1.50 [1.25-1.82]	1.56 [1.27-1.91]		1.28 [1.06-1.55]	1.32 [1.09-1.60]	1.21 [0.99-1.48]	1.29 [1.04-1.59]		1.16 [0.95-1.42]	301	290	242	NA	262
300.8	1.22 [1.06-1.40]	1.21 [1.05-1.40]	1.36 [1.13-1.64]	1.11 [0.82-1.50]	1.15 [0.99-1.34]	1.13 [0.98-1.31]	1.05 [0.90-1.22]	1.17 [0.96-1.42]	1.07 [0.78-1.48]	1.11 [0.95-1.30]	495	450	278	118	412
300.9	1.29 [1.21-1.37]	1.31 [1.23-1.40]	1.43 [1.31-1.57]	0.98 [0.87-1.12]	1.18 [1.10-1.26]	1.09 [1.02-1.17]	0.98 [0.92-1.05]	1.08 [0.98-1.19]	0.96 [0.84-1.09]	1.09 [1.02-1.17]	2,542	2,350	1,193	641	2,345
301	1.38 [1.29-1.48]	1.41 [1.32-1.52]	1.57 [1.43-1.72]	1.03 [0.88-1.19]	1.24 [1.15-1.33]	1.19 [1.11-1.28]	1.09 [1.01-1.17]	1.24 [1.12-1.37]	1.03 [0.88-1.20]	1.16 [1.08-1.25]	2,122	2,002	1,095	450	1,961
301.1	1.52 [1.18-1.97]	1.51 [1.16-1.95]			1.38 [1.06-1.80]	1.38 [1.06-1.80]	1.28 [0.97-1.67]			1.32 [1.01-1.74]	157	147	NA	NA	138
301.2	1.30 [1.22-1.39]	1.36 [1.27-1.45]	1.66 [1.48-1.86]	1.00 [0.89-1.13]	1.17 [1.09-1.25]	1.09 [1.01-1.17]	0.98 [0.91-1.06]	1.20 [1.06-1.36]	0.99 [0.87-1.12]	1.08 [1.01-1.16]	2,267	2,190	734	715	2,100
302	1.49 [1.33-1.68]	1.48 [1.32-1.67]	1.48 [1.31-1.68]		1.33 [1.17-1.51]	1.36 [1.21-1.54]	1.26 [1.12-1.43]	1.27 [1.12-1.45]		1.26 [1.11-1.43]	707	698	616	NA	598
303.1	1.40 [1.26-1.55]	1.42 [1.28-1.58]	1.45 [1.26-1.66]	1.26 [1.03-1.54]	1.22 [1.10-1.36]	1.21 [1.08-1.34]	1.11 [0.99-1.24]	1.14 [0.99-1.32]	1.19 [0.96-1.46]	1.11 [1.00-1.24]	1,022	914	529	282	934
303.3	1.41 [1.27-1.58]	1.34 [1.20-1.50]	1.32 [1.17-1.50]	1.16 [0.86-1.56]	1.26 [1.12-1.41]	1.26 [1.12-1.41]	1.13 [1.01-1.27]	1.14 [1.00-1.30]	1.02 [0.75-1.39]	1.15 [1.02-1.29]	848	773	600	119	748
303.4	1.44 [1.34-1.55]	1.41 [1.30-1.53]	1.40 [1.27-1.54]	1.28 [1.08-1.51]	1.28 [1.18-1.38]	1.26 [1.16-1.36]	1.13 [1.04-1.23]	1.15 [1.05-1.28]	1.19 [1.00-1.41]	1.16 [1.07-1.26]	1,793	1,582	1,064	401	1,624
304	1.21 [1.10-1.33]	1.23 [1.11-1.35]	1.26 [1.11-1.42]	0.99 [0.80-1.22]	1.11 [1.01-1.23]	1.12 [1.02-1.24]	1.04 [0.94-1.15]	1.06 [0.93-1.20]	1.04 [0.83-1.29]	1.09 [0.99-1.21]	1,141	1,048	647	224	965
305.2	1.29 [1.20-1.38]	1.29 [1.18-1.41]	1.41 [1.22-1.64]	1.15 [1.04-1.26]	1.20 [1.11-1.29]	1.17 [1.09-1.26]	1.08 [0.98-1.18]	1.19 [1.02-1.39]	1.11 [1.01-1.22]	1.14 [1.05-1.23]	2,118	1,258	443	1,249	1,770
305.21	1.22 [1.09-1.36]	1.25 [1.08-1.45]	1.40 [1.10-1.79]	1.09 [0.95-1.26]	1.13 [1.00-1.27]	1.14 [1.02-1.28]	1.08 [0.93-1.26]	1.21 [0.94-1.56]	1.06 [0.92-1.23]	1.09 [0.96-1.24]	847	460	159	533	682
306	1.26 [1.25-1.28]	1.27 [1.26-1.28]	1.28 [1.27-1.29]	1.04 [1.01-1.07]	1.18 [1.17-1.19]	1.20 [1.19-1.21]	1.14 [1.13-1.15]	1.16 [1.15-1.17]	1.04 [1.01-1.08]	1.16 [1.15-1.17]	150,754	149,919	121,961	10,438	132,415
306.9	1.30 [1.19-1.41]	1.28 [1.17-1.39]	1.40 [1.26-1.57]	1.13 [0.94-1.37]	1.20 [1.10-1.31]	1.19 [1.09-1.29]	1.11 [1.02-1.22]	1.21 [1.08-1.36]	1.10 [0.91-1.34]	1.12 [1.03-1.23]	1,410	1,263	803	305	1,255
312	1.24 [1.11-1.39]	1.14 [0.90-1.44]	1.10 [0.82-1.46]	1.27 [1.12-1.43]	1.15 [1.02-1.30]	1.17 [1.05-1.31]	1.04 [0.81-1.32]	1.02 [0.76-1.37]	1.22 [1.07-1.38]	1.11 [0.98-1.25]	867	174	113	730	754
313	1.25 [1.09-1.44]	1.33 [1.00-1.77]		1.21 [1.05-1.41]	1.13 [0.97-1.32]	1.14 [0.99-1.32]	1.11 [0.82-1.50]		1.16 [1.00-1.35]	1.06 [0.91-1.24]	559	115	NA	511	481
313.1	1.22 [1.15-1.28]	1.23 [1.12-1.35]	1.75 [1.34-2.29]	1.16 [1.09-1.22]	1.14 [1.08-1.21]	1.12 [1.06-1.19]	1.09 [0.99-1.21]	1.26 [0.94-1.68]	1.12 [1.06-1.19]	1.11 [1.04-1.17]	3,883	1,020	139	3,444	3,393
313.2	1.36 [1.19-1.57]	1.40 [1.11-1.76]		1.29 [1.10-1.52]	1.24 [1.07-1.44]	1.23 [1.06-1.42]	1.16 [0.91-1.48]		1.21 [1.03-1.42]	1.17 [1.00-1.36]	581	187	NA	447	513
313.3	1.32 [1.28-1.38]	1.54 [1.44-1.65]	2.15 [1.90-2.44]	1.22 [1.17-1.27]	1.23 [1.18-1.28]	1.18 [1.14-1.23]	1.34 [1.24-1.44]	1.73 [1.51-1.97]	1.15 [1.11-1.20]	1.14 [1.09-1.19]	7,896	2,095	695	6,653	6,860
315	1.14 [1.10-1.19]	1.22 [1.16-1.28]	1.23 [1.17-1.30]	1.01 [0.95-1.08]	1.08 [1.03-1.12]	1.10 [1.06-1.15]	1.12 [1.07-1.18]	1.14 [1.08-1.21]	0.97 [0.91-1.04]	1.06 [1.01-1.10]	6,293	4,238	3,699	2,319	5,799
315.1	1.56 [1.50-1.62]	1.92 [1.83-2.00]	2.15 [2.03-2.27]	1.13 [1.07-1.19]	1.36 [1.31-1.42]	1.44 [1.39-1.50]	1.79 [1.71-1.88]	1.99 [1.88-2.11]	1.09 [1.03-1.15]	1.29 [1.24-1.35]	8,341	5,297	3,441	3,817	6,832
315.2	1.22 [1.15-1.29]	1.38 [1.22-1.56]	1.41 [1.24-1.61]	1.16 [1.09-1.24]	1.14 [1.08-1.22]	1.16 [1.10-1.24]	1.29 [1.13-1.46]	1.31 [1.14-1.49]	1.11 [1.04-1.19]	1.12 [1.05-1.19]	2,868	666	603	2,234	2,631
315.3	1.58 [1.49-1.67]	1.90 [1.78-2.02]	2.15 [1.98-2.33]	1.09 [1.00-1.18]	1.37 [1.29-1.45]	1.45 [1.37-1.53]	1.74 [1.63-1.87]	1.94 [1.78-2.11]	1.05 [0.97-1.15]	1.30 [1.22-1.38]	3,630	2,534	1,641	1,482	3,146
316	1.19 [1.17-1.21]	1.21 [1.19-1.24]	1.27 [1.24-1.29]	0.98 [0.94-1.03]	1.11 [1.09-1.14]	1.15 [1.13-1.17]	1.09 [1.07-1.11]	1.15 [1.12-1.18]	0.98 [0.94-1.02]	1.12 [1.10-1.15]	27,927	26,131	17,706	5,543	22,075
316.1	1.24 [1.05-1.47]	1.22 [1.02-1.45]	1.24 [1.03-1.50]		1.15 [0.96-1.37]	1.21 [1.01-1.44]	1.18 [0.98-1.42]	1.22 [1.01-1.47]		1.15 [0.96-1.37]	330	303	285	NA	304
317	1.20 [1.17-1.22]	1.22 [1.19-1.24]	1.26 [1.23-1.29]	0.99 [0.95-1.04]	1.13 [1.11-1.15]	1.16 [1.14-1.18]	1.10 [1.08-1.12]	1.15 [1.12-1.18]	0.99 [0.94-1.04]	1.14 [1.12-1.17]	25,955	24,196	16,882	4,875	20,728
317.1	1.22 [1.19-1.24]	1.22 [1.20-1.25]	1.23 [1.20-1.26]	1.02 [0.96-1.09]	1.13 [1.11-1.16]	1.17 [1.15-1.19]	1.11 [1.09-1.13]	1.12 [1.10-1.15]	1.01 [0.95-1.08]	1.13 [1.10-1.15]	24,191	23,744	18,465	2,336	20,394
317.11	1.44 [1.38-1.51]	1.46 [1.40-1.52]	1.46 [1.40-1.53]		1.35 [1.29-1.41]	1.37 [1.31-1.43]	1.29 [1.23-1.35]	1.28 [1.23-1.34]		1.34 [1.28-1.41]	5,266	5,262	4,799	NA	4,536
318	1.13 [1.12-1.14]	1.15 [1.14-1.16]	1.15 [1.14-1.16]	0.99 [0.97-1.02]	1.06 [1.05-1.07]	1.12 [1.11-1.13]	1.03 [1.02-1.04]	1.06 [1.04-1.07]	1.00 [0.98-1.02]	1.09 [1.08-1.10]	112,963	109,604	65,828	22,575	86,891
neurological															
320	1.24 [1.15-1.33]	1.28 [1.18-1.39]	1.25 [1.12-1.41]	1.21 [1.08-1.35]	1.11 [1.03-1.20]	1.16 [1.08-1.25]	1.14 [1.05-1.25]	1.13 [1.00-1.27]	1.18 [1.05-1.33]	1.06 [0.98-1.15]	1,921	1,447	691	782	1,575
323	1.23 [1.14-1.33]	1.27 [1.17-1.39]	1.26 [1.15-1.39]	1.02 [0.87-1.20]	1.16 [1.07-1.26]	1.18 [1.09-1.28]	1.17 [1.07-1.29]	1.16 [1.05-1.28]	1.00 [0.85-1.18]	1.13 [1.04-1.23]	1,586	1,255	1,049	404	1,359
323.2	1.16 [0.92-1.46]	1.23 [0.96-1.58]	1.32 [0.99-1.76]		1.13 [0.88-1.46]	1.13 [0.89-1.43]	1.15 [0.89-1.49]	1.25 [0.93-1.67]		1.12 [0.86-1.45]	168	147	109	NA	139
323.8	1.11 [0.85-1.46]				0.94 [0.71-1.26]	1.07 [0.81-1.41]				0.94 [0.70-1.26]	134	NA	NA	NA	106
324	1.26 [1.24-1.28]	1.27 [1.25-1.29]	1.26 [1.24-1.28]	1.18 [1.12-1.25]	1.18 [1.16-1.20]	1.18 [1.16-1.20]	1.14 [1.13-1.16]	1.14 [1.12-1.16]	1.14 [1.07-1.20]	1.13 [1.11-1.15]	44,811	42,291	39,029	3,658	41,695
325	1.16 [0.95-1.42]	1.32 [1.06-1.65]	1.14 [0.86-1.51]		1.29 [1.04-1.60]	1.12 [0.92-1.38]	1.26 [1.00-1.58]	1.06 [0.79-1.42]		1.29 [1.03-1.61]	239	199	116	NA	206
327	1.48 [1.45-1.51]	1.58 [1.54-1.62]	1.58 [1.54-1.62]	1.31 [1.27-1.36]	1.38 [1.35-1.41]	1.32 [1.29-1.35]	1.27 [1.24-1.31]	1.27 [1.24-1.31]	1.25 [1.21-1.30]	1.27 [1.24-1.29]	23,995	15,545	13,302	9,075	21,559
327.3	1.51 [1.48-1.55]	1.63 [1.59-1.68]	1.63 [1.58-1.67]	1.32 [1.27-1.37]	1.40 [1.37-1.44]	1.35 [1.32-1.38]	1.31 [1.27-1.35]	1.30 [1.26-1.34]	1.26 [1.22-1.31]	1.29 [1.26-1.32]	20,679	13,191	11,662	7,845	18,668
327.4	1.25 [0.97-1.61]	1.24 [0.93-1.65]			1.21 [0.93-1.58]	1.12 [0.86-1.45]	1.01 [0.75-1.36]			1.13 [0.86-1.48]	153	123	NA	NA	143
327.41	1.34 [1.23-1.46]	1.32 [1.21-1.44]	1.36 [1.23-1.51]	1.26 [1.03-1.55]	1.24 [1.14-1.36]	1.18 [1.08-1.29]	1.09 [0.99-1.20]	1.14 [1.03-1.27]	1.19 [0.96-1.47]	1.16 [1.05-1.26]	1,414	1,254	999	268	1,302
327.5	1.50 [1.22-1.84]	1.90 [1.45-2.51]		1.25 [0.93-1.66]	1.37 [1.11-1.71]	1.37 [1.11-1.69]	1.61 [1.21-2.15]		1.22 [0.91-1.64]	1.28 [1.02-1.59]	254	144	NA	130	222
327.6	1.33 [1.02-1.72]	1.52 [1.11-2.06]			1.28 [0.98-1.67]	1.12 [0.85-1.47]	1.14 [0.81-1.59]			1.19 [0.90-1.56]	157	106	NA	NA	144
331	1.20 [1.13-1.27]	1.21 [1.14-1.29]	1.22 [1.14-1.30]	1.03 [0.83-1.28]	1.14 [1.07-1.21]	1.16 [1.09-1.23]	1.13 [1.06-1.20]	1.13 [1.06-1.21]	1.03 [0.82-1.28]	1.13 [1.06-1.21]	2,687	2,498	2,370	212	2,445
331.1	1.17 [1.10-1.25]	1.21 [1.14-1.30]	1.18 [1.10-1.26]	0.96 [0.83-1.13]	1.14 [1.07-1.22]	1.15 [1.08-1.23]	1.17 [1.09-1.25]	1.13 [1.05-1.22]	0.97 [0.83-1.13]	1.14 [1.06-1.22]	2,544	2,204	1,937	412	2,170
331.9	1.16 [1.12-1.21]	1.18 [1.14-1.22]	1.16 [1.12-1.20]		1.13 [1.09-1.17]	1.14 [1.10-1.18]	1.12 [1.08-1.16]	1.10 [1.06-1.14]		1.13 [1.09-1.17]	8,113	8,032	7,940	NA	7,506
332	1.06 [1.02-1.09]	1.05 [1.01-1.09]	1.06 [1.02-1.10]		1.03 [0.99-1.07]	1.05 [1.01-1.09]	1.02 [0.99-1.06]	1.03 [0.99-1.07]		1.03 [0.99-1.07]	7,432	7,435	7,409	NA	6,407
333	1.28 [1.22-1.34]	1.30 [1.24-1.36]	1.28 [1.22-1.35]	1.07 [0.91-1.27]	1.20 [1.15-1.26]	1.16 [1.11-1.22]	1.12 [1.07-1.17]	1.11 [1.06-1.17]	1.04 [0.87-1.24]	1.13 [1.07-1.18]	4,924	4,668	4,350	353	4,614
333.1	1.18 [1.09-1.27]	1.17 [1.09-1.26]	1.15 [1.06-1.24]		1.12 [1.04-1.20]	1.08 [1.00-1.16]	1.03 [0.95-1.11]	1.02 [0.94-1.10]		1.05 [0.97-1.14]	1,851	1,814	1,743	NA	1,735
333.2	1.28 [1.12-1.45]	1.32 [1.15-1.52]	1.38 [1.19-1.61]	1.09 [0.82-1.44]	1.21 [1.06-1.38]	1.19 [1.04-1.35]	1.18 [1.02-1.36]	1.23 [1.06-1.44]	1.06 [0.80-1.41]	1.16 [1.02-1.33]	616	517	453	126	571
333.3	1.33 [1.09-1.61]	1.32 [1.08-1.63]	1.25 [1.01-1.55]		1.22 [0.99-1.52]	1.31 [1.08-1.60]	1.31 [1.06-1.61]	1.24 [1.00-1.55]		1.23 [0.99-1.53]	266	239	216	NA	218
333.4	1.12 [1.02-1.23]	1.21 [1.09-1.35]	1.21 [1.08-1.37]	0.81 [0.67-0.97]	1.02 [0.93-1.13]	1.06 [0.96-1.17]	1.10 [0.99-1.23]	1.11 [0.99-1.25]	0.77 [0.64-0.93]	1.00 [0.90-1.10]	1,090	844	708	296	994
333.8	1.17 [1.00-1.36]	1.12 [0.96-1.30]	1.08 [0.93-1.27]		1.02 [0.87-1.19]	1.14 [0.97-1.33]	1.07 [0.92-1.25]	1.03 [0.88-1.21]		1.01 [0.86-1.18]	428	418	405	NA	393
334	1.22 [1.17-1.28]	1.24 [1.19-1.30]	1.23 [1.18-1.29]	1.10 [0.91-1.34]	1.15 [1.10-1.20]	1.16 [1.11-1.21]	1.14 [1.09-1.19]	1.13 [1.08-1.18]	1.06 [0.87-1.30]	1.12 [1.07-1.17]	5,259	5,092	4,666	266	4,683
334.1	1.17 [1.01-1.36]	1.25 [1.07-1.46]	1.20 [1.02-1.43]		1.15 [0.99-1.34]	1.13 [0.97-1.31]	1.16 [0.99-1.36]	1.10 [0.93-1.31]		1.14 [0.97-1.33]	454	380	328	NA	400
334.2	1.08 [0.97-1.20]	1.12 [1.00-1.24]	1.10 [0.99-1.22]		1.06 [0.95-1.19]	1.04 [0.93-1.15]	1.07 [0.96-1.20]	1.06 [0.95-1.18]		1.05 [0.94-1.18]	857	824	796	NA	732
335	1.23 [1.15-1.31]	1.22 [1.15-1.30]	1.23 [1.14-1.32]	1.14 [0.85-1.53]	1.14 [1.06-1.22]	1.16 [1.08-1.24]	1.08 [1.01-1.15]	1.09 [1.01-1.18]	1.15 [0.85-1.55]	1.12 [1.04-1.21]	2,212	2,199	1,684	125	1,677
337	1.26 [1.15-1.40]	1.23 [1.11-1.36]	1.22 [1.08-1.36]	1.01 [0.77-1.33]	1.13 [1.02-1.25]	1.17 [1.05-1.29]	1.07 [0.97-1.19]	1.08 [0.96-1.22]	0.94 [0.71-1.25]	1.06 [0.95-1.18]	1,018	945	747	143	926
337.1	1.36 [1.11-1.67]	1.28 [1.05-1.57]	1.28 [1.04-1.57]		1.26 [1.02-1.56]	1.26 [1.03-1.56]	1.15 [0.93-1.42]	1.13 [0.91-1.40]		1.20 [0.97-1.49]	246	243	237	NA	222
338.1	1.40 [1.30-1.51]	1.46 [1.35-1.58]	1.46 [1.34-1.60]	1.08 [0.89-1.31]	1.28 [1.18-1.38]	1.29 [1.19-1.39]	1.27 [1.17-1.38]	1.29 [1.18-1.42]	1.02 [0.84-1.25]	1.20 [1.11-1.30]	1,800	1,608	1,278	284	1,630
338.2	1.46 [1.37-1.56]	1.48 [1.39-1.58]	1.46 [1.36-1.57]	1.20 [0.97-1.47]	1.32 [1.24-1.41]	1.25 [1.16-1.33]	1.15 [1.08-1.24]	1.15 [1.07-1.25]	1.10 [0.89-1.36]	1.20 [1.12-1.28]	2,496	2,367	1,855	261	2,412
339	1.28 [1.26-1.31]	1.29 [1.27-1.32]	1.32 [1.29-1.35]	1.20 [1.17-1.24]	1.19 [1.17-1.21]	1.18 [1.16-1.20]	1.13 [1.11-1.15]	1.15 [1.13-1.18]	1.15 [1.12-1.19]	1.13 [1.11-1.14]	39,639	32,105	20,115	11,841	34,211
340	1.32 [1.29-1.35]	1.32 [1.29-1.36]	1.33 [1.29-1.38]	1.20 [1.14-1.27]	1.22 [1.19-1.25]	1.16 [1.13-1.19]	1.08 [1.05-1.11]	1.10 [1.06-1.13]	1.14 [1.08-1.20]	1.12 [1.09-1.15]	16,716	14,898	8,783	4,008	14,746
340.1	1.29 [1.18-1.40]	1.31 [1.19-1.43]	1.22 [1.08-1.37]	1.15 [0.98-1.37]	1.19 [1.09-1.30]	1.13 [1.04-1.24]	1.06 [0.96-1.16]	0.98 [0.86-1.11]	1.11 [0.94-1.33]	1.09 [0.99-1.19]	1,393	1,201	647	386	1,256
341	1.27 [1.13-1.43]	1.29 [1.14-1.46]	1.36 [1.17-1.58]	1.00 [0.75-1.34]	1.20 [1.06-1.37]	1.20 [1.07-1.36]	1.17 [1.03-1.32]	1.24 [1.06-1.45]	0.98 [0.73-1.32]	1.17 [1.02-1.33]	691	624	413	120	575
342	1.17 [1.14-1.20]	1.19 [1.15-1.23]	1.17 [1.14-1.21]	0.98 [0.86-1.13]	1.12 [1.08-1.15]	1.14 [1.11-1.18]	1.12 [1.08-1.15]	1.10 [1.07-1.13]	0.98 [0.85-1.12]	1.11 [1.08-1.15]	11,632	11,192	10,789	541	10,542
343	1.01 [0.94-1.09]	1.39 [1.25-1.55]	1.66 [1.46-1.90]	0.79 [0.72-0.87]	0.92 [0.85-1.00]	0.96 [0.89-1.04]	1.33 [1.19-1.49]	1.59 [1.38-1.82]	0.77 [0.70-0.85]	0.90 [0.83-0.97]	1,750	801	552	1,019	1,431
344	1.19 [1.13-1.26]	1.22 [1.15-1.29]	1.22 [1.15-1.30]	0.86 [0.73-1.01]	1.09 [1.02-1.15]	1.13 [1.07-1.20]	1.12 [1.05-1.19]	1.13 [1.06-1.21]	0.84 [0.71-0.99]	1.07 [1.00-1.13]	2,968	2,727	2,329	349	2,621
345	1.31 [1.28-1.34]	1.36 [1.33-1.40]	1.38 [1.34-1.42]	1.08 [1.02-1.13]	1.19 [1.16-1.22]	1.21 [1.18-1.24]	1.19 [1.15-1.22]	1.20 [1.16-1.23]	1.06 [1.00-1.11]	1.14 [1.11-1.17]	17,304	14,194	11,163	4,102	14,182
345.1	1.26 [1.19-1.34]	1.43 [1.33-1.54]	1.39 [1.27-1.51]	0.99 [0.90-1.09]	1.13 [1.06-1.20]	1.17 [1.10-1.24]	1.24 [1.15-1.34]	1.18 [1.09-1.29]	0.98 [0.89-1.08]	1.09 [1.03-1.17]	2,752	1,824	1,416	1,074	2,522
345.11	1.25 [1.19-1.32]	1.32 [1.24-1.41]	1.40 [1.30-1.51]	1.09 [1.00-1.18]	1.14 [1.08-1.21]	1.15 [1.09-1.21]	1.13 [1.06-1.21]	1.18 [1.09-1.28]	1.07 [0.99-1.16]	1.10 [1.04-1.16]	3,745	2,481	1,757	1,554	3,343
345.12	1.46 [1.33-1.59]	1.54 [1.39-1.70]	1.45 [1.30-1.62]	1.28 [1.09-1.50]	1.30 [1.19-1.42]	1.29 [1.18-1.41]	1.25 [1.12-1.38]	1.15 [1.02-1.30]	1.27 [1.08-1.49]	1.20 [1.09-1.31]	1,394	1,060	796	422	1,261
345.3	1.14 [1.12-1.17]	1.26 [1.23-1.30]	1.27 [1.23-1.31]	1.02 [0.99-1.05]	1.08 [1.05-1.10]	1.11 [1.08-1.13]	1.15 [1.12-1.19]	1.16 [1.13-1.20]	1.01 [0.98-1.04]	1.07 [1.04-1.09]	21,453	12,161	9,715	10,228	18,506
346.1	1.10 [1.00-1.22]	1.12 [1.01-1.25]	1.12 [1.00-1.26]	0.84 [0.66-1.07]	1.05 [0.94-1.17]	1.07 [0.96-1.19]	1.05 [0.93-1.17]	1.05 [0.93-1.19]	0.86 [0.67-1.10]	1.04 [0.93-1.16]	930	802	685	168	831
346.2	1.19 [0.93-1.52]				1.14 [0.88-1.47]	1.14 [0.89-1.47]				1.11 [0.86-1.44]	164	NA	NA	NA	151
346.3	1.20 [0.89-1.62]					1.14 [0.84-1.56]					101	NA	NA	NA	NA

Supplementary Table 6: phecode-mapped results (hazard ratios and events from all cohorts)

	Hazard ratio (99% confidence interval)										Events (in exposed)				
	crude					adjusted									
Outcome	any age	18+	40+	<18	hosp.	any age	18+	40+	<18	hosp.	any age	18+	40+	<18	hosp.
347	1.43 [1.14-1.79]	1.45 [1.13-1.86]	1.47 [1.09-1.99]		1.42 [1.12-1.81]	1.27 [1.00-1.61]	1.20 [0.93-1.56]	1.26 [0.92-1.72]		1.31 [1.02-1.67]	209	161	106	NA	184
348	1.21 [1.10-1.32]	1.29 [1.17-1.42]	1.25 [1.13-1.39]	0.94 [0.73-1.21]	1.14 [1.04-1.26]	1.16 [1.05-1.27]	1.18 [1.07-1.30]	1.15 [1.03-1.27]	0.92 [0.71-1.19]	1.14 [1.03-1.26]	1,144	1,046	915	157	1,020
348.2	1.17 [1.09-1.25]	1.16 [1.08-1.24]	1.10 [1.01-1.20]	1.16 [1.02-1.32]	1.10 [1.02-1.18]	1.10 [1.02-1.17]	1.04 [0.97-1.12]	1.02 [0.94-1.12]	1.10 [0.96-1.26]	1.05 [0.98-1.13]	2,306	1,973	1,237	644	2,012
348.4	1.20 [1.08-1.33]	1.20 [1.06-1.36]	1.14 [0.98-1.32]	1.20 [1.01-1.42]	1.15 [1.03-1.28]	1.15 [1.03-1.28]	1.08 [0.95-1.23]	1.03 [0.88-1.20]	1.15 [0.96-1.37]	1.11 [0.99-1.24]	884	617	439	340	787
348.7	1.17 [1.10-1.24]	1.19 [1.11-1.27]	1.18 [1.10-1.27]	1.05 [0.90-1.22]	1.12 [1.05-1.20]	1.13 [1.06-1.20]	1.10 [1.03-1.18]	1.09 [1.01-1.18]	1.02 [0.88-1.20]	1.10 [1.03-1.18]	2,569	2,282	1,947	443	2,292
348.8	1.40 [1.28-1.54]	1.43 [1.29-1.58]	1.45 [1.30-1.61]	0.93 [0.73-1.17]	1.32 [1.20-1.46]	1.30 [1.18-1.43]	1.26 [1.14-1.40]	1.26 [1.13-1.40]	0.92 [0.73-1.17]	1.30 [1.18-1.43]	1,143	988	901	178	1,078
348.9	1.14 [1.08-1.21]	1.16 [1.09-1.23]	1.18 [1.11-1.26]	1.01 [0.87-1.17]	1.07 [1.00-1.13]	1.10 [1.03-1.16]	1.08 [1.01-1.14]	1.09 [1.02-1.16]	0.97 [0.83-1.12]	1.05 [0.98-1.11]	3,027	2,691	2,402	461	2,737
349	1.32 [1.24-1.41]	1.34 [1.25-1.43]	1.40 [1.30-1.50]	1.03 [0.85-1.25]	1.24 [1.16-1.32]	1.25 [1.17-1.33]	1.20 [1.12-1.28]	1.26 [1.17-1.35]	1.00 [0.82-1.22]	1.21 [1.13-1.29]	2,442	2,267	1,993	271	2,248
350.1	1.24 [1.19-1.29]	1.29 [1.23-1.35]	1.29 [1.23-1.35]	1.10 [1.01-1.20]	1.17 [1.12-1.22]	1.15 [1.11-1.20]	1.15 [1.10-1.20]	1.15 [1.10-1.21]	1.06 [0.96-1.16]	1.12 [1.07-1.17]	6,355	5,322	4,542	1,312	5,761
350.2	1.21 [1.19-1.23]	1.20 [1.19-1.22]	1.20 [1.18-1.22]	1.10 [1.03-1.17]	1.15 [1.13-1.17]	1.16 [1.15-1.18]	1.12 [1.10-1.14]	1.12 [1.10-1.14]	1.07 [1.00-1.15]	1.13 [1.11-1.15]	45,075	43,012	42,038	2,374	42,416
350.3	1.18 [1.10-1.27]	1.18 [1.08-1.29]	1.20 [1.09-1.31]	1.17 [1.03-1.33]	1.12 [1.04-1.21]	1.12 [1.04-1.21]	1.07 [0.98-1.16]	1.09 [0.99-1.20]	1.12 [0.98-1.28]	1.09 [1.01-1.18]	1,904	1,344	1,171	629	1,698
350.6	1.35 [1.14-1.61]	1.28 [1.07-1.52]	1.36 [1.11-1.66]		1.24 [1.04-1.49]	1.23 [1.03-1.47]	1.12 [0.93-1.34]	1.14 [0.92-1.41]		1.17 [0.98-1.41]	348	318	243	NA	305
351	1.35 [1.32-1.38]	1.35 [1.32-1.38]	1.34 [1.31-1.37]	1.14 [1.00-1.31]	1.24 [1.21-1.27]	1.24 [1.21-1.27]	1.19 [1.16-1.21]	1.17 [1.15-1.20]	1.08 [0.94-1.24]	1.16 [1.14-1.19]	22,138	21,937	19,241	596	19,088
352	1.30 [1.17-1.45]	1.32 [1.18-1.47]	1.33 [1.19-1.49]		1.26 [1.13-1.41]	1.23 [1.10-1.37]	1.20 [1.07-1.35]	1.22 [1.09-1.37]		1.20 [1.07-1.35]	886	849	795	NA	813
352.1	1.42 [1.32-1.53]	1.43 [1.33-1.55]	1.44 [1.33-1.56]		1.32 [1.22-1.42]	1.29 [1.20-1.39]	1.22 [1.13-1.32]	1.24 [1.15-1.35]		1.22 [1.13-1.32]	1,837	1,821	1,646	NA	1,651
352.2	1.21 [1.15-1.28]	1.24 [1.17-1.31]	1.21 [1.14-1.29]	1.09 [0.95-1.24]	1.15 [1.09-1.21]	1.15 [1.09-1.21]	1.13 [1.06-1.19]	1.10 [1.04-1.17]	1.09 [0.95-1.24]	1.11 [1.05-1.17]	3,683	3,203	2,781	595	3,191
353	1.28 [1.25-1.32]	1.27 [1.24-1.31]	1.29 [1.25-1.32]	1.14 [0.99-1.31]	1.18 [1.15-1.22]	1.18 [1.14-1.21]	1.11 [1.08-1.14]	1.12 [1.08-1.15]	1.07 [0.93-1.24]	1.12 [1.09-1.15]	12,810	12,750	10,597	528	11,564
353.1	1.31 [1.09-1.58]	1.31 [1.09-1.57]	1.32 [1.05-1.66]		1.22 [1.01-1.48]	1.19 [0.98-1.44]	1.15 [0.95-1.39]	1.15 [0.91-1.46]		1.16 [0.95-1.41]	292	278	183	NA	258
353.2	1.27 [1.00-1.61]	1.20 [0.95-1.53]	1.23 [0.96-1.59]		1.18 [0.92-1.51]	1.14 [0.89-1.45]	0.98 [0.77-1.27]	1.03 [0.79-1.35]		1.09 [0.85-1.41]	169	168	144	NA	154
356	1.28 [1.11-1.46]	1.26 [1.10-1.46]	1.26 [1.09-1.47]		1.17 [1.01-1.35]	1.17 [1.02-1.34]	1.12 [0.97-1.30]	1.11 [0.95-1.30]		1.07 [0.93-1.24]	529	483	419	NA	473
357	1.36 [1.32-1.40]	1.36 [1.32-1.40]	1.36 [1.32-1.41]	1.02 [0.84-1.23]	1.24 [1.20-1.28]	1.24 [1.20-1.28]	1.15 [1.11-1.19]	1.15 [1.11-1.19]	0.99 [0.81-1.20]	1.17 [1.14-1.21]	9,917	9,762	9,264	284	9,311
358	1.27 [1.00-1.61]	1.34 [1.02-1.75]	1.21 [0.90-1.62]		1.11 [0.86-1.42]	1.21 [0.95-1.55]	1.25 [0.95-1.65]	1.13 [0.83-1.52]		1.09 [0.84-1.40]	174	136	113	NA	157
358.1	1.09 [0.95-1.26]	1.11 [0.96-1.28]	1.16 [0.99-1.35]		1.13 [0.97-1.32]	1.05 [0.90-1.21]	1.04 [0.90-1.21]	1.07 [0.92-1.25]		1.09 [0.93-1.27]	459	446	408	NA	400
359.1	1.20 [1.04-1.38]	1.22 [1.03-1.45]	1.13 [0.93-1.38]	1.00 [0.79-1.27]	1.04 [0.89-1.21]	1.17 [1.01-1.35]	1.18 [0.99-1.40]	1.08 [0.89-1.32]	1.00 [0.79-1.28]	1.03 [0.88-1.21]	488	341	243	172	400
359.2	1.35 [1.27-1.43]	1.37 [1.28-1.47]	1.35 [1.26-1.46]	1.21 [1.07-1.36]	1.26 [1.18-1.34]	1.29 [1.21-1.37]	1.26 [1.17-1.35]	1.26 [1.17-1.35]	1.18 [1.04-1.33]	1.23 [1.15-1.31]	2,818	2,185	1,838	728	2,470
sense organs															
360	1.30 [1.20-1.41]	1.33 [1.23-1.44]	1.30 [1.19-1.41]	1.29 [0.99-1.67]	1.25 [1.15-1.36]	1.24 [1.15-1.34]	1.26 [1.16-1.37]	1.23 [1.12-1.34]	1.17 [0.89-1.53]	1.20 [1.10-1.31]	1,642	1,518	1,390	159	1,453
360.2	1.17 [0.99-1.38]	1.13 [0.96-1.34]	1.13 [0.96-1.34]		1.09 [0.91-1.30]	1.15 [0.97-1.35]	1.12 [0.94-1.32]	1.11 [0.93-1.32]		1.04 [0.86-1.24]	351	339	325	NA	286
361	1.08 [1.02-1.15]	1.11 [1.04-1.18]	1.09 [1.01-1.16]	1.01 [0.76-1.35]	1.06 [0.99-1.14]	1.05 [0.98-1.12]	1.07 [1.00-1.14]	1.05 [0.98-1.12]	1.01 [0.75-1.34]	1.05 [0.98-1.13]	2,275	2,204	2,000	128	1,834
361.1	1.05 [1.00-1.10]	1.08 [1.02-1.13]	1.04 [0.99-1.10]	1.10 [0.89-1.35]	1.03 [0.98-1.09]	1.03 [0.98-1.08]	1.05 [1.00-1.11]	1.02 [0.97-1.08]	1.08 [0.87-1.34]	1.03 [0.97-1.09]	3,916	3,790	3,415	243	3,116
361.2	1.19 [0.96-1.46]	1.18 [0.96-1.46]	1.16 [0.94-1.44]		1.15 [0.92-1.44]	1.17 [0.95-1.45]	1.14 [0.92-1.42]	1.15 [0.92-1.43]		1.14 [0.91-1.43]	219	216	206	NA	197
362	1.17 [1.14-1.19]	1.17 [1.15-1.20]	1.16 [1.14-1.19]	1.16 [0.96-1.39]	1.12 [1.10-1.15]	1.13 [1.11-1.16]	1.12 [1.10-1.15]	1.11 [1.09-1.13]	1.14 [0.94-1.38]	1.10 [1.08-1.13]	23,935	23,741	23,343	308	21,152
362.2	1.15 [1.13-1.18]	1.16 [1.13-1.19]	1.15 [1.13-1.18]		1.12 [1.09-1.14]	1.12 [1.09-1.15]	1.11 [1.09-1.14]	1.10 [1.08-1.13]		1.09 [1.07-1.12]	19,942	19,927	19,823	NA	17,772
362.29	1.15 [1.13-1.18]	1.16 [1.13-1.19]	1.15 [1.13-1.18]		1.12 [1.09-1.14]	1.12 [1.09-1.15]	1.11 [1.09-1.14]	1.10 [1.08-1.13]		1.09 [1.07-1.12]	19,942	19,927	19,823	NA	17,772
362.3	1.24 [1.01-1.53]	1.30 [1.05-1.60]	1.24 [0.99-1.54]		1.11 [0.90-1.38]	1.19 [0.97-1.47]	1.17 [0.94-1.45]	1.09 [0.87-1.38]		1.12 [0.90-1.39]	233	218	194	NA	207
362.31	1.23 [1.07-1.42]	1.24 [1.08-1.43]	1.20 [1.04-1.38]		1.17 [1.00-1.35]	1.19 [1.04-1.38]	1.18 [1.03-1.36]	1.15 [1.00-1.33]		1.14 [0.98-1.33]	521	516	501	NA	457
362.4	1.19 [1.14-1.26]	1.20 [1.15-1.27]	1.18 [1.12-1.24]		1.13 [1.07-1.19]	1.16 [1.10-1.22]	1.13 [1.08-1.19]	1.10 [1.05-1.16]		1.11 [1.05-1.17]	4,086	4,030	3,898	NA	3,633
362.6	1.09 [0.89-1.33]	1.09 [0.89-1.34]	1.02 [0.82-1.26]		1.02 [0.81-1.27]	1.07 [0.87-1.31]	1.07 [0.87-1.32]	0.98 [0.78-1.22]		1.00 [0.80-1.25]	234	230	199	NA	191
362.7	1.25 [1.22-1.29]	1.26 [1.22-1.30]	1.25 [1.21-1.29]	1.16 [0.90-1.49]	1.18 [1.14-1.22]	1.19 [1.15-1.23]	1.10 [1.07-1.14]	1.09 [1.05-1.13]	1.19 [0.91-1.54]	1.16 [1.12-1.20]	10,332	10,279	9,810	164	9,418
362.8	1.23 [1.07-1.42]	1.20 [1.04-1.38]	1.22 [1.06-1.42]		1.20 [1.04-1.40]	1.19 [1.03-1.37]	1.13 [0.97-1.30]	1.15 [0.99-1.34]		1.18 [1.01-1.38]	513	482	450	NA	439
363	1.12 [1.02-1.23]	1.13 [1.03-1.25]	1.11 [1.00-1.22]		1.05 [0.95-1.17]	1.10 [0.99-1.21]	1.09 [0.99-1.21]	1.06 [0.96-1.17]		1.04 [0.94-1.16]	1,077	1,054	1,007	NA	966
363.3	1.21 [1.05-1.39]	1.20 [1.04-1.38]	1.16 [1.00-1.34]		1.16 [1.00-1.34]	1.17 [1.02-1.36]	1.15 [0.99-1.33]	1.11 [0.95-1.28]		1.15 [0.99-1.34]	510	498	476	NA	477
363.4	1.05 [0.78-1.42]					1.03 [0.76-1.40]					104	NA	NA	NA	NA
364	1.38 [1.32-1.44]	1.36 [1.30-1.43]	1.24 [1.18-1.31]	1.91 [1.67-2.18]	1.29 [1.23-1.36]	1.31 [1.25-1.37]	1.27 [1.22-1.34]	1.19 [1.13-1.25]	1.70 [1.48-1.95]	1.24 [1.18-1.30]	5,208	4,964	3,982	735	4,460
364.1	1.41 [1.26-1.59]	1.40 [1.24-1.57]	1.37 [1.21-1.56]		1.39 [1.23-1.57]	1.34 [1.19-1.51]	1.30 [1.15-1.48]	1.28 [1.12-1.46]		1.33 [1.18-1.50]	774	722	635	NA	694
364.2	1.18 [1.02-1.37]	1.19 [1.02-1.38]	1.24 [1.06-1.44]		1.16 [1.00-1.36]	1.12 [0.96-1.30]	1.11 [0.96-1.29]	1.17 [1.00-1.37]		1.10 [0.94-1.29]	451	447	426	NA	410
364.4	1.21 [1.12-1.30]	1.21 [1.12-1.30]	1.20 [1.11-1.29]		1.18 [1.09-1.27]	1.18 [1.09-1.27]	1.16 [1.08-1.25]	1.15 [1.07-1.24]		1.15 [1.06-1.24]	1,875	1,859	1,825	NA	1,731
364.41	2.36 [2.13-2.62]	2.37 [2.13-2.63]	2.17 [1.74-2.71]	2.00 [1.73-2.32]	2.24 [1.98-2.52]	2.01 [1.80-2.25]	1.98 [1.77-2.21]	1.80 [1.42-2.28]	1.77 [1.51-2.06]	1.94 [1.71-2.21]	1,203	1,051	218	602	862
364.5	1.26 [1.14-1.40]	1.21 [1.09-1.34]	1.20 [1.08-1.33]		1.16 [1.04-1.29]	1.22 [1.10-1.35]	1.15 [1.04-1.27]	1.14 [1.03-1.27]		1.13 [1.02-1.26]	978	969	946	NA	860
364.9	1.73 [1.50-2.00]	1.70 [1.47-1.96]	1.65 [1.39-1.94]		1.66 [1.43-1.93]	1.57 [1.35-1.83]	1.50 [1.29-1.74]	1.48 [1.25-1.75]		1.51 [1.29-1.76]	507	497	381	NA	466
365	1.18 [1.15-1.20]	1.18 [1.15-1.20]	1.18 [1.15-1.20]	1.30 [1.01-1.67]	1.13 [1.11-1.16]	1.14 [1.12-1.17]	1.13 [1.11-1.16]	1.13 [1.11-1.16]	1.26 [0.97-1.64]	1.11 [1.08-1.14]	21,888	21,771	21,556	166	18,785
365.11	1.16 [1.11-1.20]	1.15 [1.10-1.20]	1.16 [1.11-1.20]		1.11 [1.06-1.16]	1.13 [1.09-1.18]	1.12 [1.08-1.17]	1.13 [1.08-1.18]		1.09 [1.04-1.14]	5,891	5,870	5,851	NA	5,142
365.2	1.14 [1.06-1.22]	1.13 [1.06-1.22]	1.15 [1.07-1.24]		1.09 [1.01-1.18]	1.10 [1.03-1.19]	1.10 [1.02-1.18]	1.11 [1.04-1.20]		1.07 [0.99-1.16]	1,881	1,879	1,858	NA	1,581
366	1.21 [1.20-1.22]	1.21 [1.20-1.23]	1.21 [1.20-1.23]	1.49 [1.28-1.73]	1.16 [1.14-1.17]	1.17 [1.15-1.18]	1.14 [1.13-1.16]	1.14 [1.13-1.16]	1.44 [1.23-1.67]	1.13 [1.11-1.14]	77,912	77,601	76,878	503	66,285
366.1	1.47 [1.15-1.86]	1.49 [1.15-1.93]	1.40 [1.05-1.86]		1.32 [1.00-1.73]	1.43 [1.12-1.83]	1.38 [1.05-1.80]	1.26 [0.94-1.70]		1.28 [0.97-1.69]	171	143	113	NA	130
366.2	1.17 [1.15-1.19]	1.17 [1.16-1.19]	1.18 [1.17-1.20]		1.12 [1.11-1.14]	1.13 [1.12-1.15]	1.12 [1.10-1.13]	1.13 [1.11-1.14]		1.10 [1.08-1.12]	46,074	46,065	45,897	NA	39,385
366.3	1.08 [0.85-1.36]	1.12 [0.87-1.45]	1.08 [0.82-1.43]		1.00 [0.76-1.32]	1.07 [0.84-1.37]	1.11 [0.85-1.45]	1.05 [0.78-1.40]		1.00 [0.75-1.32]	164	141	116	NA	122
367	1.12 [1.09-1.16]	1.13 [1.09-1.17]	1.12 [1.08-1.17]	1.12 [1.01-1.23]	1.09 [1.05-1.14]	1.09 [1.05-1.13]	1.09 [1.05-1.13]	1.08 [1.04-1.12]	1.09 [0.99-1.20]	1.07 [1.03-1.11]	8,017	7,057	6,729	1,094	7,038
367.1	1.08 [1.02-1.15]	1.08 [1.02-1.15]	1.08 [1.01-1.15]	1.10 [0.91-1.33]	1.05 [0.99-1.12]	1.06 [1.00-1.12]	1.06 [0.99-1.12]	1.06 [0.99-1.13]	1.07 [0.88-1.30]	1.03 [0.97-1.10]	2,804	2,561	2,398	308	2,335
367.2	1.17 [1.11-1.23]	1.14 [1.08-1.21]	1.13 [1.07-1.20]	1.35 [1.13-1.61]	1.13 [1.06-1.19]	1.13 [1.07-1.20]	1.09 [1.03-1.15]	1.07 [1.01-1.14]	1.31 [1.09-1.57]	1.09 [1.03-1.16]	3,421	3,124	3,017	338	3,137
367.8	1.12 [1.02-1.22]	1.25 [1.13-1.39]	1.20 [1.08-1.34]	0.98 [0.84-1.14]	1.04 [0.94-1.14]	1.08 [0.99-1.18]	1.20 [1.08-1.34]	1.15 [1.03-1.28]	0.95 [0.81-1.11]	1.01 [0.92-1.12]	1,287	887	845	424	1,107
367.9	1.20 [1.16-1.24]	1.22 [1.18-1.25]	1.21 [1.17-1.25]	1.00 [0.89-1.12]	1.14 [1.11-1.18]	1.16 [1.12-1.20]	1.14 [1.11-1.18]	1.14 [1.10-1.17]	0.96 [0.86-1.08]	1.12 [1.09-1.16]	11,421	10,807	10,273	773	10,552
368	1.24 [1.21-1.27]	1.25 [1.22-1.28]	1.25 [1.21-1.29]	1.18 [1.12-1.24]	1.16 [1.13-1.19]	1.16 [1.13-1.18]	1.11 [1.08-1.14]	1.13 [1.09-1.16]	1.13 [1.07-1.19]	1.11 [1.08-1.14]	16,638	14,327	10,510	3,753	14,631
368.1	1.19 [1.12-1.27]	1.23 [1.15-1.31]	1.21 [1.13-1.30]	1.15 [0.99-1.33]	1.17 [1.09-1.25]	1.15 [1.08-1.23]	1.16 [1.08-1.24]	1.16 [1.08-1.24]	1.14 [0.98-1.33]	1.14 [1.07-1.22]	2,540	2,123	2,005	465	2,205
368.2	1.23 [1.16-1.31]	1.18 [1.11-1.26]	1.21 [1.13-1.30]	1.27 [1.11-1.45]	1.15 [1.09-1.23]	1.15 [1.08-1.22]	1.05 [0.99-1.13]	1.08 [1.00-1.16]	1.24 [1.08-1.42]	1.10 [1.04-1.18]	2,891	2,440	2,009	609	2,537
368.3	1.15 [1.00-1.32]	1.14 [0.98-1.32]	1.09 [0.93-1.27]		1.15 [0.99-1.33]	1.12 [0.97-1.29]	1.08 [0.93-1.27]	1.04 [0.89-1.22]		1.11 [0.95-1.29]	499	421	411	NA	441
368.4	1.17 [1.08-1.25]	1.16 [1.08-1.25]	1.13 [1.05-1.22]	1.28 [0.99-1.67]	1.10 [1.02-1.19]	1.13 [1.05-1.22]	1.10 [1.02-1.18]	1.07 [0.99-1.15]	1.30 [0.99-1.70]	1.09 [1.01-1.18]	1,907	1,794	1,661	157	1,685
368.9	1.25 [1.20-1.31]	1.26 [1.21-1.32]	1.24 [1.18-1.31]	1.18 [1.09-1.29]	1.16 [1.11-1.21]	1.16 [1.11-1.21]	1.11 [1.06-1.16]	1.11 [1.05-1.17]	1.13 [1.03-1.23]	1.12 [1.07-1.16]	6,221	5,337	3,782	1,474	5,632
369	1.42 [1.33-1.51]	1.47 [1.33-1.63]	1.45 [1.30-1.63]	1.35 [1.25-1.45]	1.33 [1.25-1.43]	1.36 [1.28-1.45]	1.35 [1.22-1.50]	1.35 [1.20-1.51]	1.30 [1.21-1.41]	1.30 [1.21-1.39]	2,692	1,009	790	1,757	2,239
369.2	1.54 [1.39-1.71]	1.56 [1.39-1.76]	1.42 [1.25-1.60]	1.67 [1.34-2.09]	1.40 [1.25-1.56]	1.44 [1.29-1.60]	1.42 [1.26-1.60]	1.28 [1.13-1.46]	1.55 [1.24-1.95]	1.30 [1.16-1.46]	985	792	694	229	858
369.5	1.43 [1.36-1.50]	1.43 [1.34-1.52]	1.35 [1.26-1.45]	1.42 [1.32-1.54]	1.35 [1.28-1.42]	1.34 [1.27-1.41]	1.29 [1.21-1.38]	1.24 [1.16-1.33]	1.32 [1.22-1.43]	1.28 [1.22-1.35]	4,406	2,770	2,435	1,760	3,999
370	1.72 [1.58-1.87]	1.66 [1.52-1.81]	1.61 [1.46-1.77]	1.90 [1.52-2.38]	1.61 [1.47-1.76]	1.61 [1.48-1.75]	1.53 [1.40-1.67]	1.50 [1.36-1.66]	1.73 [1.37-2.18]	1.54 [1.41-1.69]	1,569	1,397	1,162	243	1,348
370.1	1.69 [1.51-1.90]	1.64 [1.46-1.86]	1.53 [1.34-1.75]	1.99 [1.44-2.75]	1.58 [1.40-1.79]	1.58 [1.41-1.79]	1.52 [1.34-1.73]	1.42 [1.24-1.63]	1.85 [1.32-2.59]	1.52 [1.34-1.73]	802	725	595	111	686
370.3	2.82 [2.23-3.57]	2.47 [1.85-3.28]	2.15 [1.57-2.93]	4.44 [2.90-6.81]	2.39 [1.85-3.07]	2.53 [1.97-3.24]	2.06 [1.53-2.79]	1.98 [1.43-2.72]	3.71 [2.36-5.83]	2.14 [1.65-2.78]	243	153	117	105	206
371	1.44 [1.28-1.63]	1.47 [1.29-1.67]	1.40 [1.22-1.59]		1.37 [1.21-1.56]	1.36 [1.20-1.54]	1.37 [1.20-1.56]	1.31 [1.15-1.50]		1.32 [1.16-1.50]	717	645	599	NA	637
371.1	1.38 [1.28-1.48]	1.32 [1.23-1.43]	1.31 [1.20-1.42]	1.40 [1.14-1.72]	1.32 [1.22-1.42]	1.33 [1.23-1.43]	1.25 [1.15-1.35]	1.24 [1.14-1.35]	1.36 [1.10-1.68]	1.29 [1.19-1.39]	1,890	1,678	1,459	272	1,670
371.2	1.89 [1.38-2.58]					1.58 [1.14-2.19]					117	NA	NA	NA	NA
371.3	1.31 [1.26-1.36]	1.33 [1.27-1.39]	1.33 [1.27-1.40]	1.23 [1.14-1.32]	1.24 [1.18-1.29]	1.25 [1.20-1.30]	1.25 [1.19-1.31]	1.25 [1.19-1.32]	1.20 [1.11-1.29]	1.19 [1.14-1.24]	6,529	4,781	3,800	2,103	5,206
371.33	3.77 [2.55-5.57]					3.42 [2.29-5.11]					103	NA	NA	NA	NA
372	1.24 [1.16-1.31]	1.24 [1.16-1.32]	1.28 [1.19-1.36]	1.11 [0.92-1.33]	1.17 [1.10-1.25]	1.19 [1.12-1.26]	1.18 [1.11-1.26]	1.21 [1.13-1.29]	1.08 [0.89-1.30]	1.15 [1.08-1.23]	2,749	2,548	2,268	293	2,312
374	1.31 [1.27-1.34]	1.31 [1.28-1.35]	1.31 [1.27-1.35]	1.23 [1.11-1.35]	1.22 [1.19-1.26]	1.24 [1.21-1.28]	1.22 [1.19-1.26]	1.22 [1.19-1.26]	1.19 [1.07-1.31]	1.17 [1.14-1.21]	13,550	12,700	11,939	1,055	11,510
374.1	1.36 [1.31-1.42]	1.35 [1.29-1.41]	1.34 [1.28-1.40]	1.55 [1.11-2.16]	1.26 [1.21-1.32]	1.31 [1.26-1.37]	1.27 [1.22-1.33]	1.27 [1.21-1.32]	1.61 [1.14-2.28]	1.23 [1.17-1.28]	5,689	5,602	5,528	105	4,912
374.2	1.24 [0.96-1.61]	1.32 [1.00-1.74]	1.16 [0.87-1.54]		1.18 [0.90-1.54]	1.20 [0.92-1.56]	1.23 [0.92-1.63]	1.11 [0.83-1.49]		1.14 [0.86-1.50]	144	129	117	NA	131
374.3	1.25 [1.19-1.32]	1.27 [1.20-1.33]	1.28 [1.21-1.35]	1.12 [0.95-1.32]	1.17 [1.11-1.23]	1.17 [1.12-1.23]	1.15 [1.09-1.22]	1.16 [1.10-1.23]	1.09 [0.92-1.29]	1.11 [1.05-1.17]	4,020	3,713	3,520	367	3,570
375	1.40 [1.35-1.45]	1.44 [1.38-1.50]	1.44 [1.39-1.51]	1.20 [1.09-1.32]	1.31 [1.26-1.37]	1.30 [1.25-1.35]	1.32 [1.27-1.37]	1.32 [1.26-1.37]	1.15 [1.04-1.26]	1.24 [1.19-1.29]	7,312	6,384	6,029	1,027	6,318
375.2	1.44 [1.35-1.53]	1.54 [1.44-1.65]	1.51 [1.41-1.62]	1.24 [1.07-1.43]	1.36 [1.28-1.46]	1.35 [1.27-1.44]	1.43 [1.33-1.53]	1.39 [1.29-1.49]	1.20 [1.03-1.38]	1.30 [1.22-1.39]	2,649	2,219	2,093	459	2,286
376	1.40 [1.32-1.49]	1.43 [1.30-1.57]	1.47 [1.33-1.64]	1.33 [1.23-1.43]	1.32 [1.23-1.41]	1.35 [1.27-1.44]	1.32 [1.20-1.45]	1.35 [1.21-1.50]	1.29 [1.19-1.39]	1.29 [1.21-1.38]	2,911	1,174	928	1,822	2,424
377	1.12 [1.03-1.22]	1.19 [1.07-1.31]	1.19 [1.05-1.36]	1.09 [0.96-1.25]	1.11 [1.01-1.22]	1.08 [0.99-1.18]	1.09 [0.98-1.20]	1.16 [1.02-1.32]	1.04 [0.91-1.20]	1.07 [0.98-1.18]	1,372	969	557	607	1,184
377.1	1.31 [1.10-1.55]	1.29 [1.07-1.56]	1.36 [1.11-1.66]		1.23 [1.03-1.47]	1.25 [1.05-1.49]	1.20 [0.99-1.46]	1.26 [1.03-1.55]		1.21 [1.01-1.45]	353	289	255	NA	332
377.3	1.31 [1.18-1.45]	1.34 [1.20-1.50]	1.28 [1.14-1.45]	1.28 [1.00-1.64]	1.21 [1.08-1.35]	1.28 [1.15-1.42]	1.24 [1.11-1.39]	1.19 [1.05-1.35]	1.29 [1.00-1.66]	1.19 [1.06-1.33]	927	819	660	176	798
378	1.19 [1.02-1.38]	1.29 [1.06-1.57]	1.21 [0.95-1.52]	0.99 [0.79-1.24]	1.06 [0.90-1.25]	1.11 [0.95-1.30]	1.16 [0.94-1.42]	1.11 [0.87-1.41]	0.94 [0.75-1.19]	1.02 [0.87-1.20]	416	247	179	192	367
378.1	1.07 [1.03-1.11]	1.26 [1.18-1.36]	1.28 [1.17-1.39]	1.00 [0.96-1.05]	1.03 [0.98-1.08]	1.04 [1.00-1.08]	1.18 [1.10-1.28]	1.19 [1.09-1.30]	0.99 [0.94-1.03]	1.01 [0.97-1.06]	6,070	1,823	1,238	4,451	5,057
378.2	1.12 [1.03-1.23]	1.19 [1.07-1.32]	1.15 [1.03-1.29]	0.95 [0.80-1.11]	1.06 [0.97-1.17]	1.07 [0.98-1.17]	1.09 [0.98-1.21]	1.04 [0.93-1.17]	0.92 [0.78-1.09]	1.04 [0.94-1.14]	1,222	903	763	368	1,120
378.5	1.06 [0.97-1.16]	1.10 [1.00-1.21]	1.13 [1.02-1.26]	0.97 [0.80-1.18]	1.02 [0.93-1.13]	1.01 [0.92-1.11]	1.02 [0.92-1.13]	1.05 [0.94-1.17]	0.92 [0.75-1.12]	1.00 [0.91-1.10]	1,185	965	849	258	1,024
379	1.32 [1.21-1.43]	1.34 [1.22-1.46]	1.34 [1.22-1.47]	1.20 [1.00-1.45]	1.26 [1.16-1.37]	1.24 [1.14-1.35]	1.23 [1.12-1.34]	1.24 [1.13-1.36]	1.15 [0.95-1.39]	1.19 [1.09-1.30]	1,573	1,315	1,159	302	1,384
379.1	1.62 [1.29-2.04]	1.65 [1.31-2.07]	1.46 [1.13-1.90]		1.56 [1.24-1.98]	1.50 [1.19-1.90]	1.43 [1.12-1.81]	1.28 [0.98-1.68]		1.42 [1.11-1.81]	207	197	151	NA	182
379.2	1.12 [1.07-1.18]	1.13 [1.07-1.18]	1.14 [1.09-1.20]		1.08 [1.03-1.13]	1.08 [1.03-1.14]	1.06 [1.01-1.12]	1.08 [1.03-1.13]		1.06 [1.01-1.11]	4,314	4,273	4,086	NA	3,817
379.3	1.15 [1.11-1.18]	1.15 [1.11-1.19]	1.16 [1.12-1.20]		1.10 [1.06-1.14]	1.11 [1.07-1.15]	1.09 [1.06-1.13]	1.10 [1.06-1.14]		1.07 [1.03-1.11]	9,161	9,105	9,014	NA	7,958
379.4	1.08 [0.95-1.22]	1.08 [0.94-1.23]	1.05 [0.91-1.21]	1.08 [0.79-1.47]	1.01 [0.88-1.15]	1.03 [0.90-1.17]	0.99 [0.86-1.14]	0.98 [0.84-1.13]	1.00 [0.72-1.38]	0.98 [0.85-1.12]	610	533	469	106	548
379.5	1.19 [1.11-1.28]	1.18 [1.10-1.27]	1.15 [1.07-1.24]		1.15 [1.07-1.24]	1.15 [1.07-1.24]	1.12 [1.04-1.21]	1.09 [1.01-1.17]		1.13 [1.05-1.22]	2,010	1,955	1,871	NA	1,834
379.9	1.41 [1.28-1.56]	1.40 [1.25-1.56]	1.41 [1.25-1.59]	1.45 [1.19-1.78]	1.29 [1.17-1.43]	1.29 [1.16-1.43]	1.22 [1.09-1.37]	1.23 [1.09-1.40]	1.41 [1.15-1.74]	1.22 [1.10-1.35]	1,057	841	684	275	952
380	1.35 [1.29-1.40]	1.45 [1.37-1.54]	1.45 [1.36-1.55]	1.26 [1.19-1.33]	1.27 [1.22-1.33]	1.30 [1.24-1.35]	1.35 [1.27-1.43]	1.34 [1.26-1.43]	1.22 [1.15-1.29]	1.24 [1.19-1.30]	5,997	3,053	2,561	3,129	5,249
380.1	1.60 [1.52-1.70]	1.72 [1.61-1.83]	1.85 [1.71-2.00]	1.33 [1.21-1.46]	1.48 [1.39-1.57]	1.51 [1.42-1.60]	1.54 [1.44-1.65]	1.67 [1.54-1.81]	1.28 [1.16-1.41]	1.43 [1.34-1.52]	3,397	2,476	1,794	1,182	2,917
380.4	1.28 [1.22-1.35]	1.32 [1.21-1.43]	1.35 [1.23-1.48]	1.25 [1.18-1.33]	1.22 [1.16-1.29]	1.24 [1.18-1.30]	1.22 [1.12-1.34]	1.26 [1.14-1.38]	1.21 [1.14-1.29]	1.19 [1.13-1.26]	4,195	1,476	1,265	2,803	3,729
381.1	1.21 [1.18-1.24]	1.36 [1.26-1.47]	1.35 [1.24-1.48]	1.20 [1.17-1.23]	1.17 [1.14-1.20]	1.17 [1.14-1.20]	1.22 [1.13-1.32]	1.24 [1.13-1.35]	1.16 [1.13-1.19]	1.14 [1.11-1.17]	16,747	1,588	1,181	15,302	13,800
381.11	1.26 [1.22-1.31]	1.53 [1.43-1.64]	1.52 [1.40-1.65]	1.21 [1.16-1.25]	1.21 [1.16-1.25]	1.21 [1.17-1.26]	1.39 [1.29-1.49]	1.37 [1.26-1.50]	1.17 [1.12-1.22]	1.18 [1.13-1.22]	8,185	2,130	1,463	6,314	7,188
381.2	1.43 [1.27-1.62]	1.47 [1.26-1.71]	1.46 [1.21-1.76]	1.41 [1.17-1.70]	1.32 [1.15-1.50]	1.33 [1.17-1.51]	1.26 [1.07-1.48]	1.25 [1.03-1.52]	1.41 [1.17-1.71]	1.24 [1.08-1.42]	669	422	266	315	577
381.3	1.15 [1.03-1.29]	1.33 [1.14-1.55]	1.20 [1.01-1.44]	1.05 [0.89-1.22]	1.06 [0.94-1.20]	1.10 [0.98-1.24]	1.19 [1.02-1.40]	1.10 [0.91-1.32]	1.02 [0.87-1.19]	1.04 [0.92-1.18]	766	422	301	386	673
381.9	1.33 [1.21-1.46]	1.55 [1.34-1.78]	1.41 [1.19-1.67]	1.28 [1.13-1.45]	1.23 [1.11-1.36]	1.27 [1.15-1.41]	1.41 [1.22-1.63]	1.28 [1.07-1.52]	1.25 [1.10-1.41]	1.19 [1.07-1.32]	1,112	505	339	676	979
382	1.35 [1.25-1.46]	1.47 [1.34-1.61]	1.44 [1.29-1.61]	1.19 [1.07-1.33]	1.28 [1.18-1.38]	1.25 [1.16-1.35]	1.27 [1.15-1.40]	1.25 [1.11-1.41]	1.15 [1.03-1.29]	1.21 [1.11-1.31]	1,858	1,132	791	841	1,639
383	1.09 [0.91-1.30]	1.12 [0.93-1.34]	1.14 [0.93-1.40]		1.11 [0.92-1.35]	1.07 [0.90-1.29]	1.09 [0.91-1.32]	1.12 [0.90-1.38]		1.09 [0.89-1.33]	291	286	209	NA	238
384	1.22 [1.14-1.30]	1.32 [1.18-1.48]	1.34 [1.16-1.55]	1.19 [1.10-1.28]	1.14 [1.06-1.22]	1.17 [1.10-1.25]	1.23 [1.09-1.38]	1.23 [1.06-1.42]	1.16 [1.07-1.25]	1.12 [1.05-1.20]	2,421	755	449	1,806	2,153
384.4	1.24 [1.18-1.31]	1.25 [1.16-1.35]	1.31 [1.19-1.43]	1.17 [1.09-1.26]	1.13 [1.07-1.20]	1.19 [1.13-1.26]	1.18 [1.09-1.27]	1.20 [1.10-1.32]	1.14 [1.06-1.23]	1.11 [1.05-1.18]	3,386	1,686	1,115	1,886	2,890
385	1.19 [1.10-1.29]	1.24 [1.12-1.37]	1.33 [1.18-1.51]	1.10 [0.99-1.23]	1.12 [1.03-1.21]	1.15 [1.06-1.24]	1.16 [1.04-1.29]	1.24 [1.09-1.41]	1.06 [0.95-1.19]	1.09 [1.00-1.18]	1,687	906	622	880	1,471
385.3	1.16 [1.09-1.25]	1.19 [1.09-1.30]	1.23 [1.10-1.38]	1.11 [1.01-1.23]	1.09 [1.01-1.18]	1.14 [1.06-1.22]	1.14 [1.04-1.25]	1.17 [1.04-1.31]	1.10 [0.99-1.22]	1.10 [1.01-1.18]	2,024	1,168	765	1,002	1,691
385.5	1.20 [1.07-1.33]	1.17 [1.01-1.36]	1.22 [1.01-1.48]	1.20 [1.03-1.39]	1.12 [1.00-1.25]	1.15 [1.02-1.28]	1.09 [0.93-1.27]	1.13 [0.92-1.37]	1.16 [1.00-1.35]	1.08 [0.96-1.22]	838	412	244	488	748
386	1.25 [1.20-1.31]	1.26 [1.20-1.32]	1.25 [1.20-1.31]	1.27 [0.94-1.71]	1.16 [1.11-1.22]	1.17 [1.11-1.22]	1.14 [1.09-1.20]	1.14 [1.09-1.20]	1.20 [0.88-1.64]	1.10 [1.05-1.16]	4,860	4,798	4,556	122	4,363
386.1	1.25 [1.17-1.34]	1.26 [1.17-1.35]	1.25 [1.17-1.35]		1.16 [1.07-1.25]	1.17 [1.09-1.26]	1.15 [1.07-1.24]	1.15 [1.07-1.23]		1.10 [1.02-1.19]	2,001	2,001	1,918	NA	1,736
386.2	1.25 [1.17-1.33]	1.25 [1.18-1.34]	1.26 [1.18-1.34]		1.18 [1.10-1.26]	1.15 [1.08-1.23]	1.13 [1.05-1.20]	1.14 [1.07-1.22]		1.12 [1.04-1.19]	2,583	2,554	2,427	NA	2,389
386.3	1.26 [1.17-1.35]	1.27 [1.18-1.37]	1.24 [1.15-1.34]	1.23 [0.96-1.58]	1.17 [1.09-1.27]	1.16 [1.08-1.25]	1.15 [1.07-1.24]	1.12 [1.03-1.21]	1.15 [0.89-1.49]	1.10 [1.02-1.19]	1,901	1,784	1,557	168	1,633
386.9	1.24 [1.21-1.26]	1.25 [1.22-1.27]	1.24 [1.22-1.27]	1.11 [1.05-1.18]	1.16 [1.13-1.18]	1.15 [1.13-1.18]	1.12 [1.09-1.14]	1.12 [1.09-1.15]	1.07 [1.01-1.14]	1.10 [1.08-1.13]	24,441	22,855	19,322	2,819	22,003
388	1.37 [1.19-1.58]	1.39 [1.16-1.67]	1.43 [1.16-1.77]	1.24 [0.99-1.54]	1.29 [1.11-1.51]	1.30 [1.13-1.51]	1.25 [1.03-1.51]	1.31 [1.05-1.62]	1.19 [0.95-1.49]	1.24 [1.06-1.44]	494	300	217	221	438
389	1.23 [1.21-1.25]	1.24 [1.22-1.26]	1.24 [1.22-1.26]	1.16 [1.11-1.21]	1.17 [1.16-1.19]	1.18 [1.16-1.19]	1.15 [1.13-1.17]	1.15 [1.14-1.17]	1.12 [1.07-1.17]	1.14 [1.12-1.16]	46,430	41,503	39,398	5,674	42,616
389.1	1.33 [1.26-1.40]	1.39 [1.31-1.47]	1.37 [1.29-1.46]	1.03 [0.91-1.18]	1.22 [1.16-1.29]	1.23 [1.17-1.30]	1.24 [1.17-1.32]	1.23 [1.16-1.31]	0.99 [0.87-1.13]	1.16 [1.10-1.23]	3,710	3,237	2,952	573	3,432
389.2	1.23 [1.16-1.31]	1.28 [1.16-1.41]	1.28 [1.14-1.44]	1.21 [1.13-1.30]	1.17 [1.09-1.24]	1.18 [1.11-1.26]	1.19 [1.07-1.31]	1.18 [1.05-1.33]	1.17 [1.09-1.26]	1.14 [1.07-1.21]	2,886	1,019	692	1,992	2,626
389.3	1.07 [0.92-1.23]	1.08 [0.94-1.25]	1.11 [0.96-1.28]		1.08 [0.94-1.25]	1.01 [0.87-1.17]	1.02 [0.88-1.18]	1.03 [0.89-1.19]		1.04 [0.90-1.21]	524	520	519	NA	501
389.4	1.37 [1.29-1.46]	1.38 [1.29-1.47]	1.35 [1.27-1.45]	1.35 [1.04-1.75]	1.30 [1.21-1.38]	1.28 [1.20-1.37]	1.24 [1.16-1.32]	1.22 [1.14-1.31]	1.32 [1.01-1.73]	1.23 [1.15-1.31]	2,572	2,496	2,204	176	2,306
389.5	1.39 [1.06-1.82]				1.27 [0.96-1.68]	1.18 [0.89-1.56]				1.15 [0.86-1.54]	144	NA	NA	NA	125
circulatory system															
394	1.21 [1.17-1.24]	1.22 [1.18-1.25]	1.22 [1.18-1.26]		1.14 [1.11-1.18]	1.16 [1.12-1.20]	1.14 [1.10-1.17]	1.14 [1.10-1.17]		1.12 [1.08-1.15]	12,028	11,979	11,829	NA	11,236
394.2	1.20 [1.17-1.23]	1.20 [1.17-1.23]	1.20 [1.18-1.23]	0.99 [0.83-1.19]	1.14 [1.11-1.17]	1.15 [1.12-1.18]	1.12 [1.09-1.14]	1.11 [1.09-1.14]	0.94 [0.78-1.13]	1.11 [1.09-1.14]	19,411	19,234	18,706	330	17,719
394.3	1.22 [1.18-1.26]	1.22 [1.18-1.27]	1.23 [1.19-1.28]	0.99 [0.79-1.25]	1.15 [1.11-1.19]	1.16 [1.12-1.21]	1.13 [1.09-1.17]	1.13 [1.09-1.17]	0.99 [0.78-1.26]	1.12 [1.08-1.16]	8,616	8,497	8,289	188	7,873
394.4	1.41 [1.16-1.70]	1.37 [1.13-1.66]	1.46 [1.20-1.78]		1.31 [1.08-1.60]	1.34 [1.11-1.63]	1.28 [1.05-1.56]	1.34 [1.10-1.64]		1.27 [1.04-1.55]	271	264	257	NA	250
394.7	1.25 [1.21-1.28]	1.24 [1.20-1.27]	1.24 [1.20-1.27]	1.01 [0.81-1.25]	1.18 [1.15-1.21]	1.20 [1.16-1.23]	1.14 [1.11-1.18]	1.14 [1.11-1.17]	0.99 [0.80-1.24]	1.15 [1.12-1.19]	12,847	12,740	12,413	219	12,071
395.1	1.19 [1.17-1.22]	1.20 [1.17-1.23]	1.20 [1.17-1.23]	1.02 [0.85-1.21]	1.14 [1.11-1.17]	1.15 [1.12-1.17]	1.12 [1.09-1.14]	1.11 [1.09-1.14]	0.96 [0.80-1.16]	1.11 [1.09-1.14]	18,964	18,789	18,269	326	17,378
395.2	1.22 [1.20-1.25]	1.23 [1.20-1.26]	1.23 [1.20-1.26]	0.97 [0.79-1.18]	1.17 [1.14-1.19]	1.17 [1.15-1.20]	1.14 [1.11-1.16]	1.14 [1.11-1.16]	0.97 [0.79-1.19]	1.13 [1.10-1.16]	19,913	19,749	19,498	249	18,136
395.3	1.23 [1.12-1.34]	1.21 [1.11-1.32]	1.24 [1.13-1.35]		1.17 [1.07-1.28]	1.16 [1.06-1.26]	1.12 [1.02-1.22]	1.13 [1.04-1.24]		1.13 [1.04-1.24]	1,417	1,373	1,313	NA	1,334
395.4	1.22 [1.12-1.32]	1.26 [1.15-1.37]	1.29 [1.18-1.41]	0.95 [0.75-1.20]	1.13 [1.04-1.23]	1.15 [1.06-1.25]	1.17 [1.07-1.27]	1.17 [1.07-1.28]	0.94 [0.74-1.20]	1.10 [1.01-1.20]	1,558	1,448	1,318	167	1,463
395.6	1.21 [1.17-1.26]	1.23 [1.19-1.28]	1.22 [1.17-1.27]	0.85 [0.63-1.15]	1.11 [1.06-1.15]	1.17 [1.12-1.21]	1.15 [1.10-1.20]	1.13 [1.08-1.18]	0.85 [0.63-1.16]	1.08 [1.04-1.13]	6,087	6,033	5,856	108	5,680
396	1.21 [1.19-1.23]	1.22 [1.20-1.24]	1.22 [1.20-1.24]	1.16 [1.12-1.20]	1.14 [1.13-1.16]	1.14 [1.13-1.16]	1.11 [1.09-1.13]	1.12 [1.10-1.13]	1.12 [1.08-1.16]	1.10 [1.09-1.12]	51,043	45,453	36,501	9,356	45,151
401.1	1.24 [1.23-1.25]	1.24 [1.23-1.25]	1.24 [1.23-1.25]	1.21 [1.12-1.31]	1.17 [1.16-1.18]	1.19 [1.18-1.20]	1.13 [1.12-1.14]	1.12 [1.11-1.13]	1.17 [1.08-1.27]	1.14 [1.13-1.15]	207,382	206,548	200,764	1,786	153,264
401.2	1.30 [1.13-1.49]	1.29 [1.12-1.48]	1.28 [1.11-1.47]		1.17 [1.02-1.35]	1.22 [1.06-1.40]	1.15 [1.00-1.33]	1.14 [0.99-1.31]		1.14 [0.99-1.31]	579	577	573	NA	561
401.21	1.21 [1.10-1.33]	1.21 [1.10-1.33]	1.22 [1.11-1.35]		1.06 [0.96-1.17]	1.15 [1.04-1.27]	1.08 [0.98-1.19]	1.10 [0.99-1.21]		1.04 [0.94-1.15]	1,103	1,092	1,084	NA	972
401.22	1.29 [1.26-1.33]	1.30 [1.27-1.34]	1.31 [1.27-1.35]		1.19 [1.16-1.23]	1.25 [1.21-1.29]	1.19 [1.15-1.23]	1.19 [1.16-1.23]		1.18 [1.14-1.22]	11,437	11,390	11,255	NA	10,333
401.3	1.26 [1.15-1.39]	1.29 [1.17-1.42]	1.26 [1.14-1.40]	1.03 [0.81-1.32]	1.12 [1.02-1.24]	1.22 [1.11-1.34]	1.17 [1.05-1.30]	1.13 [1.02-1.25]	1.05 [0.81-1.35]	1.12 [1.01-1.23]	1,158	1,009	950	166	1,068
402	1.16 [1.11-1.20]	1.16 [1.12-1.21]	1.20 [1.14-1.25]	1.13 [1.03-1.24]	1.10 [1.06-1.15]	1.11 [1.07-1.16]	1.10 [1.06-1.15]	1.13 [1.08-1.19]	1.11 [1.01-1.22]	1.07 [1.03-1.12]	7,371	6,868	4,612	1,282	6,126
411.1	1.29 [1.26-1.33]	1.29 [1.25-1.33]	1.29 [1.25-1.32]		1.17 [1.13-1.20]	1.21 [1.17-1.24]	1.15 [1.11-1.18]	1.14 [1.10-1.17]		1.12 [1.08-1.15]	12,002	12,009	11,890	NA	10,376

Supplementary Table 6: phecode-mapped results (hazard ratios and events from all cohorts)

	Hazard ratio (99% confidence interval)										Events (in exposed)				
	crude					adjusted									
Outcome	any age	18+	40+	<18	hosp.	any age	18+	40+	<18	hosp.	any age	18+	40+	<18	hosp.
411.2	1.19 [1.17-1.20]	1.18 [1.17-1.20]	1.19 [1.17-1.20]	1.07 [0.79-1.45]	1.12 [1.10-1.14]	1.14 [1.12-1.16]	1.09 [1.07-1.10]	1.09 [1.07-1.10]	1.11 [0.81-1.51]	1.10 [1.08-1.11]	45,699	45,672	45,192	109	39,874
411.3	1.28 [1.26-1.29]	1.27 [1.26-1.29]	1.28 [1.26-1.30]		1.17 [1.15-1.19]	1.20 [1.18-1.21]	1.14 [1.12-1.15]	1.14 [1.12-1.15]		1.12 [1.10-1.14]	44,789	44,808	44,361	NA	37,819
411.4	1.21 [1.19-1.23]	1.21 [1.19-1.23]	1.21 [1.19-1.23]	1.00 [0.74-1.34]	1.14 [1.12-1.16]	1.15 [1.14-1.17]	1.11 [1.09-1.12]	1.11 [1.09-1.13]	0.99 [0.73-1.34]	1.11 [1.09-1.13]	45,156	45,122	44,671	115	37,893
411.41	1.11 [1.03-1.19]	1.10 [1.02-1.18]	1.13 [1.05-1.21]	1.20 [0.88-1.63]	1.06 [0.98-1.14]	1.06 [0.99-1.14]	1.04 [0.97-1.12]	1.07 [0.99-1.15]	1.15 [0.84-1.58]	1.02 [0.95-1.10]	1,925	1,855	1,737	112	1,686
411.8	1.22 [1.21-1.23]	1.22 [1.21-1.24]	1.22 [1.21-1.24]	1.06 [0.81-1.37]	1.14 [1.13-1.16]	1.16 [1.15-1.18]	1.11 [1.10-1.13]	1.11 [1.10-1.12]	1.08 [0.83-1.41]	1.11 [1.10-1.12]	75,749	75,716	75,062	144	64,357
411.9	1.24 [1.20-1.28]	1.25 [1.21-1.29]	1.24 [1.20-1.28]		1.17 [1.13-1.21]	1.18 [1.13-1.22]	1.13 [1.09-1.17]	1.11 [1.07-1.15]		1.14 [1.10-1.18]	8,471	8,469	8,361	NA	7,812
414	1.22 [1.19-1.26]	1.24 [1.20-1.27]	1.23 [1.20-1.27]	1.09 [0.87-1.37]	1.16 [1.13-1.19]	1.16 [1.13-1.19]	1.12 [1.09-1.15]	1.11 [1.08-1.14]	1.06 [0.84-1.34]	1.12 [1.09-1.15]	14,411	14,312	13,974	209	13,480
414.2	1.35 [1.15-1.59]	1.39 [1.18-1.64]	1.36 [1.16-1.60]		1.24 [1.05-1.46]	1.27 [1.08-1.50]	1.26 [1.07-1.49]	1.19 [1.01-1.41]		1.19 [1.01-1.42]	408	407	396	NA	370
415	1.13 [1.10-1.16]	1.12 [1.09-1.15]	1.11 [1.08-1.14]	1.01 [0.84-1.20]	1.07 [1.04-1.10]	1.08 [1.05-1.11]	1.05 [1.02-1.08]	1.04 [1.01-1.07]	0.97 [0.81-1.16]	1.04 [1.01-1.07]	14,034	13,958	13,043	322	12,255
415.11	1.13 [1.10-1.16]	1.12 [1.09-1.15]	1.12 [1.09-1.15]	1.07 [0.88-1.29]	1.07 [1.04-1.10]	1.08 [1.05-1.11]	1.05 [1.02-1.08]	1.04 [1.01-1.07]	1.03 [0.84-1.26]	1.04 [1.01-1.07]	14,114	14,073	13,190	277	12,322
415.2	1.44 [1.34-1.55]	1.44 [1.34-1.55]	1.42 [1.33-1.53]		1.35 [1.26-1.46]	1.30 [1.21-1.40]	1.25 [1.16-1.34]	1.21 [1.12-1.30]		1.24 [1.15-1.34]	2,022	2,013	1,981	NA	1,894
415.21	1.36 [1.28-1.44]	1.33 [1.25-1.41]	1.32 [1.24-1.40]		1.25 [1.18-1.33]	1.28 [1.21-1.36]	1.21 [1.14-1.28]	1.20 [1.13-1.27]		1.20 [1.13-1.28]	2,975	2,949	2,858	NA	2,740
416	1.27 [1.24-1.29]	1.26 [1.24-1.29]	1.26 [1.24-1.29]	1.11 [0.96-1.28]	1.20 [1.17-1.22]	1.20 [1.18-1.23]	1.15 [1.13-1.18]	1.15 [1.13-1.18]	1.07 [0.92-1.25]	1.16 [1.14-1.19]	25,068	24,757	24,144	504	23,402
418	1.30 [1.28-1.32]	1.31 [1.30-1.33]	1.32 [1.30-1.33]	1.13 [1.08-1.17]	1.19 [1.17-1.20]	1.20 [1.18-1.21]	1.16 [1.15-1.18]	1.16 [1.15-1.18]	1.09 [1.04-1.13]	1.13 [1.11-1.14]	68,204	65,789	53,445	6,513	57,864
418.1	1.32 [1.28-1.35]	1.31 [1.28-1.34]	1.32 [1.28-1.36]	1.13 [1.02-1.26]	1.21 [1.18-1.24]	1.21 [1.17-1.24]	1.14 [1.11-1.17]	1.14 [1.11-1.18]	1.12 [1.00-1.24]	1.14 [1.11-1.17]	14,587	14,318	12,440	977	13,330
420.1	1.30 [1.14-1.49]	1.25 [1.09-1.44]	1.41 [1.19-1.67]	1.05 [0.76-1.44]	1.22 [1.05-1.41]	1.23 [1.07-1.42]	1.14 [0.98-1.31]	1.29 [1.09-1.54]	1.08 [0.78-1.50]	1.17 [1.01-1.36]	530	496	351	102	453
420.2	1.22 [1.17-1.28]	1.23 [1.18-1.28]	1.22 [1.17-1.28]	1.02 [0.88-1.18]	1.13 [1.08-1.18]	1.16 [1.12-1.22]	1.13 [1.08-1.18]	1.13 [1.08-1.18]	0.99 [0.85-1.16]	1.10 [1.05-1.15]	5,741	5,523	4,918	442	5,058
420.21	1.24 [1.09-1.43]	1.20 [1.04-1.38]	1.28 [1.09-1.50]		1.18 [1.01-1.37]	1.20 [1.04-1.38]	1.13 [0.98-1.31]	1.20 [1.02-1.41]		1.15 [0.99-1.34]	508	477	360	NA	415
420.22	1.27 [1.07-1.50]	1.32 [1.11-1.57]	1.33 [1.11-1.59]		1.22 [1.02-1.45]	1.22 [1.03-1.45]	1.21 [1.01-1.44]	1.22 [1.01-1.46]		1.17 [0.98-1.39]	341	318	289	NA	314
420.3	1.30 [1.24-1.37]	1.32 [1.25-1.38]	1.32 [1.25-1.39]	1.12 [0.91-1.38]	1.21 [1.15-1.28]	1.25 [1.19-1.31]	1.22 [1.16-1.28]	1.23 [1.16-1.29]	1.10 [0.89-1.36]	1.18 [1.12-1.24]	4,356	4,164	3,977	235	3,937
425.1	1.21 [1.16-1.27]	1.23 [1.18-1.29]	1.22 [1.16-1.28]	1.17 [0.96-1.43]	1.15 [1.10-1.20]	1.15 [1.10-1.20]	1.12 [1.07-1.17]	1.10 [1.05-1.16]	1.18 [0.95-1.45]	1.11 [1.06-1.16]	4,772	4,639	4,297	242	4,273
425.11	1.26 [1.10-1.46]	1.31 [1.14-1.52]	1.27 [1.09-1.48]		1.13 [0.97-1.31]	1.21 [1.05-1.40]	1.20 [1.03-1.39]	1.17 [1.00-1.36]		1.11 [0.95-1.29]	473	459	422	NA	420
425.12	1.16 [1.03-1.32]	1.21 [1.06-1.37]	1.19 [1.04-1.36]		1.08 [0.95-1.23]	1.11 [0.98-1.26]	1.10 [0.97-1.26]	1.09 [0.95-1.25]		1.05 [0.93-1.20]	640	607	554	NA	586
425.2	0.98 [0.78-1.25]	1.07 [0.85-1.36]	0.98 [0.77-1.25]		0.97 [0.76-1.23]	0.94 [0.74-1.20]	0.99 [0.78-1.27]	0.92 [0.72-1.18]		0.93 [0.73-1.18]	178	172	160	NA	167
425.8	1.53 [1.23-1.90]	1.50 [1.21-1.86]	1.45 [1.17-1.81]		1.34 [1.07-1.69]	1.47 [1.18-1.84]	1.34 [1.08-1.68]	1.30 [1.04-1.63]		1.34 [1.06-1.69]	210	210	199	NA	181
426	1.18 [1.07-1.30]	1.20 [1.09-1.33]	1.20 [1.08-1.33]		1.17 [1.06-1.30]	1.12 [1.01-1.24]	1.11 [1.00-1.23]	1.10 [1.00-1.23]		1.15 [1.04-1.27]	1,037	1,020	995	NA	981
426.2	1.15 [1.03-1.29]	1.17 [1.04-1.31]	1.17 [1.05-1.31]		1.12 [1.00-1.26]	1.10 [0.98-1.24]	1.10 [0.98-1.23]	1.09 [0.97-1.22]		1.10 [0.98-1.24]	837	828	811	NA	795
426.21	1.27 [1.23-1.31]	1.25 [1.21-1.30]	1.27 [1.23-1.31]		1.20 [1.17-1.24]	1.22 [1.18-1.26]	1.17 [1.14-1.21]	1.18 [1.14-1.22]		1.18 [1.14-1.22]	10,840	10,780	10,661	NA	10,115
426.23	1.18 [1.11-1.25]	1.20 [1.13-1.28]	1.20 [1.13-1.27]		1.15 [1.08-1.23]	1.13 [1.07-1.20]	1.12 [1.06-1.19]	1.12 [1.06-1.20]		1.13 [1.06-1.20]	2,963	2,939	2,877	NA	2,714
426.24	1.13 [1.08-1.19]	1.15 [1.09-1.20]	1.15 [1.09-1.20]		1.08 [1.03-1.13]	1.10 [1.05-1.16]	1.09 [1.04-1.14]	1.09 [1.04-1.14]		1.07 [1.01-1.12]	4,628	4,597	4,547	NA	4,207
426.25	1.23 [1.08-1.40]	1.24 [1.09-1.41]	1.21 [1.06-1.39]		1.13 [0.99-1.30]	1.19 [1.04-1.36]	1.18 [1.03-1.35]	1.15 [1.01-1.32]		1.10 [0.96-1.27]	606	599	585	NA	546
426.3	1.22 [1.15-1.29]	1.22 [1.15-1.30]	1.21 [1.14-1.29]		1.17 [1.11-1.25]	1.17 [1.10-1.24]	1.14 [1.08-1.21]	1.13 [1.06-1.20]		1.14 [1.07-1.21]	3,251	3,247	3,221	NA	3,027
426.31	1.21 [1.18-1.24]	1.21 [1.18-1.24]	1.20 [1.17-1.24]	1.03 [0.88-1.20]	1.16 [1.13-1.19]	1.15 [1.12-1.18]	1.12 [1.09-1.15]	1.11 [1.08-1.14]	0.99 [0.84-1.17]	1.12 [1.09-1.15]	14,848	14,703	14,078	420	13,663
426.32	1.19 [1.16-1.22]	1.19 [1.16-1.22]	1.19 [1.16-1.22]		1.14 [1.11-1.17]	1.14 [1.11-1.17]	1.10 [1.07-1.13]	1.10 [1.07-1.13]		1.11 [1.08-1.14]	15,083	15,061	14,924	NA	13,937
426.4	1.16 [1.02-1.33]	1.16 [1.01-1.35]	1.21 [1.01-1.46]	1.06 [0.85-1.33]	1.07 [0.92-1.24]	1.11 [0.97-1.27]	1.09 [0.94-1.27]	1.15 [0.95-1.39]	1.01 [0.80-1.27]	1.03 [0.88-1.20]	550	434	257	207	425
426.8	1.11 [0.94-1.32]	1.16 [0.97-1.39]	1.21 [1.00-1.46]		1.02 [0.86-1.22]	1.04 [0.88-1.24]	1.01 [0.84-1.22]	1.08 [0.88-1.31]		0.98 [0.82-1.17]	347	311	282	NA	323
426.9	1.39 [1.24-1.56]	1.45 [1.29-1.62]	1.40 [1.25-1.57]		1.23 [1.10-1.38]	1.28 [1.14-1.43]	1.25 [1.11-1.40]	1.20 [1.06-1.35]		1.18 [1.05-1.32]	779	769	753	NA	751
426.91	1.20 [1.17-1.23]	1.22 [1.19-1.25]	1.21 [1.18-1.24]	1.10 [0.86-1.41]	1.13 [1.10-1.16]	1.15 [1.12-1.18]	1.12 [1.09-1.15]	1.11 [1.08-1.14]	1.08 [0.83-1.39]	1.10 [1.07-1.13]	16,208	16,143	15,877	166	15,540
427	1.18 [1.07-1.30]	1.21 [1.09-1.33]	1.18 [1.07-1.31]		1.11 [1.01-1.23]	1.12 [1.01-1.23]	1.11 [1.00-1.22]	1.09 [0.98-1.21]		1.07 [0.97-1.19]	1,087	1,035	940	NA	986
427.1	1.22 [1.19-1.26]	1.22 [1.18-1.26]	1.22 [1.18-1.26]	1.16 [1.03-1.31]	1.14 [1.10-1.18]	1.15 [1.12-1.19]	1.11 [1.08-1.15]	1.11 [1.07-1.15]	1.11 [0.98-1.25]	1.10 [1.06-1.13]	10,826	10,396	9,373	764	9,433
427.11	1.25 [1.21-1.30]	1.25 [1.20-1.29]	1.25 [1.20-1.30]	1.12 [0.99-1.27]	1.17 [1.13-1.22]	1.17 [1.13-1.22]	1.14 [1.09-1.18]	1.14 [1.10-1.19]	1.08 [0.95-1.23]	1.12 [1.08-1.16]	7,882	7,502	6,648	652	6,834
427.12	1.14 [1.08-1.20]	1.13 [1.07-1.19]	1.13 [1.07-1.20]	1.26 [0.95-1.68]	1.04 [0.98-1.10]	1.08 [1.02-1.14]	1.03 [0.97-1.09]	1.03 [0.97-1.09]	1.17 [0.87-1.58]	1.01 [0.95-1.07]	3,264	3,198	3,007	134	2,923
427.2	1.16 [1.15-1.18]	1.16 [1.15-1.18]	1.16 [1.15-1.18]	1.06 [0.85-1.32]	1.12 [1.11-1.13]	1.13 [1.11-1.14]	1.10 [1.09-1.11]	1.09 [1.08-1.11]	1.01 [0.80-1.27]	1.10 [1.09-1.11]	81,212	81,194	80,479	207	70,255
427.3	1.19 [1.16-1.21]	1.20 [1.17-1.23]	1.20 [1.17-1.23]	1.08 [0.99-1.19]	1.12 [1.09-1.15]	1.14 [1.12-1.17]	1.12 [1.09-1.15]	1.12 [1.09-1.15]	1.04 [0.95-1.15]	1.10 [1.07-1.13]	16,985	16,114	15,079	1,293	15,400
427.4	1.07 [0.98-1.17]	1.07 [0.98-1.18]	1.07 [0.97-1.17]		1.03 [0.94-1.14]	1.04 [0.95-1.15]	1.01 [0.92-1.11]	0.99 [0.90-1.09]		1.03 [0.93-1.13]	1,133	1,113	1,059	NA	1,006
427.41	1.07 [0.98-1.17]	1.07 [0.98-1.18]	1.07 [0.97-1.17]		1.03 [0.94-1.14]	1.04 [0.95-1.15]	1.01 [0.92-1.11]	0.99 [0.90-1.09]		1.03 [0.93-1.13]	1,133	1,113	1,059	NA	1,006
427.42	1.23 [1.19-1.27]	1.24 [1.20-1.28]	1.23 [1.19-1.27]	1.08 [0.89-1.30]	1.18 [1.14-1.22]	1.19 [1.15-1.23]	1.15 [1.11-1.19]	1.14 [1.10-1.18]	1.04 [0.85-1.26]	1.17 [1.13-1.21]	9,596	9,458	9,070	275	8,594
427.5	1.25 [1.19-1.32]	1.26 [1.19-1.32]	1.27 [1.20-1.34]	1.28 [1.03-1.58]	1.19 [1.13-1.25]	1.17 [1.12-1.24]	1.14 [1.09-1.21]	1.16 [1.10-1.23]	1.21 [0.97-1.51]	1.14 [1.08-1.20]	4,023	3,903	3,607	234	3,683
427.6	1.20 [1.14-1.27]	1.20 [1.14-1.27]	1.20 [1.13-1.27]	1.08 [0.83-1.40]	1.13 [1.07-1.20]	1.13 [1.07-1.20]	1.10 [1.04-1.16]	1.09 [1.03-1.16]	1.05 [0.80-1.37]	1.08 [1.02-1.15]	3,507	3,421	3,165	167	3,181
427.61	1.17 [1.03-1.32]	1.12 [0.99-1.28]	1.15 [1.01-1.32]		1.07 [0.94-1.23]	1.07 [0.94-1.22]	1.03 [0.90-1.17]	1.05 [0.91-1.20]		1.02 [0.89-1.17]	662	641	602	NA	581
427.7	1.22 [1.19-1.25]	1.22 [1.20-1.25]	1.22 [1.19-1.26]	1.15 [1.10-1.20]	1.15 [1.13-1.18]	1.14 [1.12-1.17]	1.10 [1.07-1.12]	1.10 [1.07-1.13]	1.11 [1.06-1.17]	1.10 [1.08-1.13]	20,616	18,181	12,895	4,809	18,176
427.8	1.12 [1.04-1.19]	1.13 [1.06-1.21]	1.12 [1.05-1.20]		1.05 [0.98-1.12]	1.07 [1.00-1.14]	1.05 [0.98-1.13]	1.04 [0.97-1.11]		1.02 [0.95-1.09]	2,271	2,274	2,214	NA	2,127
427.9	1.24 [1.20-1.27]	1.25 [1.21-1.28]	1.24 [1.20-1.28]	1.12 [1.04-1.21]	1.16 [1.12-1.19]	1.14 [1.11-1.17]	1.10 [1.07-1.14]	1.09 [1.06-1.13]	1.07 [0.99-1.16]	1.09 [1.05-1.12]	11,971	11,314	8,436	1,752	10,457
428.1	1.29 [1.27-1.32]	1.29 [1.27-1.31]	1.29 [1.27-1.31]		1.23 [1.21-1.25]	1.23 [1.21-1.25]	1.17 [1.15-1.19]	1.17 [1.15-1.19]		1.18 [1.16-1.20]	41,385	41,364	41,173	NA	38,454
428.2	1.26 [1.25-1.28]	1.26 [1.24-1.27]	1.26 [1.25-1.28]	1.09 [0.90-1.33]	1.20 [1.19-1.22]	1.20 [1.19-1.22]	1.15 [1.14-1.17]	1.15 [1.14-1.17]	1.10 [0.90-1.35]	1.17 [1.15-1.19]	64,824	64,744	64,197	270	58,996
429.1	1.17 [0.91-1.50]	1.30 [0.99-1.71]	1.46 [1.09-1.97]		1.00 [0.77-1.30]	1.12 [0.87-1.44]	1.19 [0.89-1.57]	1.30 [0.96-1.77]		0.95 [0.72-1.24]	147	126	108	NA	127
429.2	1.28 [1.21-1.36]	1.28 [1.20-1.36]	1.31 [1.23-1.40]	1.26 [1.02-1.55]	1.23 [1.15-1.31]	1.21 [1.14-1.29]	1.16 [1.09-1.23]	1.18 [1.10-1.26]	1.19 [0.96-1.48]	1.20 [1.12-1.28]	2,811	2,689	2,383	252	2,514
429.3	1.28 [1.17-1.41]	1.33 [1.21-1.47]	1.27 [1.14-1.40]	1.29 [1.06-1.58]	1.22 [1.11-1.33]	1.19 [1.09-1.31]	1.18 [1.07-1.30]	1.14 [1.02-1.27]	1.19 [0.96-1.46]	1.16 [1.06-1.28]	1,266	1,070	925	263	1,157
430	1.17 [1.06-1.29]	1.19 [1.07-1.32]	1.22 [1.09-1.37]	0.94 [0.75-1.18]	1.10 [0.99-1.23]	1.15 [1.04-1.28]	1.15 [1.03-1.28]	1.17 [1.04-1.32]	0.91 [0.72-1.15]	1.11 [0.99-1.24]	1,004	879	764	190	854
430.1	1.16 [1.08-1.24]	1.16 [1.08-1.25]	1.14 [1.06-1.22]		1.10 [1.02-1.18]	1.15 [1.07-1.24]	1.13 [1.05-1.21]	1.11 [1.03-1.19]		1.10 [1.02-1.20]	1,910	1,880	1,738	NA	1,556
430.2	1.17 [1.12-1.23]	1.17 [1.12-1.23]	1.17 [1.12-1.22]	0.88 [0.65-1.19]	1.14 [1.09-1.20]	1.16 [1.11-1.22]	1.14 [1.09-1.20]	1.13 [1.08-1.19]	0.88 [0.64-1.20]	1.14 [1.09-1.20]	4,912	4,837	4,715	104	4,230
430.3	1.14 [1.07-1.22]	1.16 [1.09-1.24]	1.15 [1.08-1.23]		1.08 [1.01-1.16]	1.12 [1.05-1.20]	1.12 [1.04-1.20]	1.10 [1.03-1.18]		1.09 [1.01-1.17]	2,297	2,267	2,229	NA	2,071
433	1.21 [1.18-1.25]	1.20 [1.17-1.24]	1.22 [1.18-1.25]		1.15 [1.12-1.19]	1.17 [1.13-1.20]	1.12 [1.09-1.15]	1.12 [1.09-1.16]		1.13 [1.10-1.17]	12,642	12,611	12,487	NA	11,809
433.1	1.17 [1.13-1.22]	1.17 [1.12-1.22]	1.17 [1.12-1.22]		1.11 [1.06-1.16]	1.14 [1.10-1.19]	1.09 [1.04-1.14]	1.08 [1.04-1.13]		1.10 [1.05-1.15]	5,593	5,582	5,554	NA	4,973
433.11	1.10 [0.98-1.23]	1.11 [0.99-1.24]	1.12 [1.00-1.26]		1.10 [0.97-1.24]	1.10 [0.98-1.23]	1.08 [0.96-1.21]	1.08 [0.96-1.22]		1.11 [0.98-1.25]	775	772	759	NA	681
433.12	1.13 [1.05-1.21]	1.16 [1.08-1.25]	1.14 [1.06-1.23]		1.08 [1.00-1.16]	1.10 [1.03-1.18]	1.11 [1.03-1.19]	1.07 [1.00-1.15]		1.07 [1.00-1.15]	2,085	2,078	2,071	NA	1,934
433.2	1.09 [1.07-1.11]	1.09 [1.07-1.12]	1.09 [1.07-1.11]	1.17 [0.93-1.47]	1.07 [1.04-1.09]	1.08 [1.06-1.10]	1.05 [1.03-1.07]	1.04 [1.02-1.07]	1.22 [0.96-1.55]	1.07 [1.05-1.09]	22,286	22,175	21,851	191	19,622
433.21	1.11 [1.09-1.14]	1.12 [1.09-1.15]	1.11 [1.08-1.14]		1.09 [1.06-1.12]	1.11 [1.08-1.14]	1.07 [1.04-1.10]	1.07 [1.04-1.10]		1.10 [1.07-1.13]	14,370	14,329	14,170	NA	12,566
433.3	1.18 [1.16-1.20]	1.18 [1.16-1.20]	1.18 [1.16-1.20]	1.08 [0.82-1.41]	1.14 [1.12-1.16]	1.15 [1.13-1.17]	1.11 [1.09-1.13]	1.11 [1.09-1.13]	1.08 [0.82-1.43]	1.13 [1.11-1.15]	35,644	35,555	35,303	135	33,238
433.31	1.19 [1.15-1.23]	1.19 [1.15-1.23]	1.19 [1.15-1.23]		1.12 [1.08-1.16]	1.14 [1.10-1.18]	1.11 [1.07-1.15]	1.10 [1.07-1.14]		1.10 [1.06-1.14]	9,101	9,072	8,944	NA	8,049
433.5	1.24 [1.13-1.36]	1.23 [1.12-1.35]	1.24 [1.13-1.37]		1.17 [1.06-1.29]	1.21 [1.10-1.33]	1.14 [1.04-1.25]	1.15 [1.05-1.27]		1.18 [1.07-1.30]	1,115	1,101	1,043	NA	999
433.8	1.18 [1.15-1.22]	1.19 [1.15-1.23]	1.18 [1.15-1.22]	0.94 [0.73-1.21]	1.10 [1.06-1.13]	1.15 [1.12-1.19]	1.11 [1.07-1.14]	1.10 [1.06-1.13]	0.91 [0.70-1.17]	1.09 [1.06-1.13]	10,401	10,290	10,123	153	9,596
440	1.26 [1.22-1.30]	1.26 [1.22-1.30]	1.27 [1.23-1.31]		1.18 [1.14-1.22]	1.22 [1.18-1.26]	1.14 [1.10-1.18]	1.15 [1.11-1.19]		1.17 [1.13-1.21]	9,463	9,462	9,410	NA	8,712
440.1	1.38 [1.23-1.55]	1.39 [1.24-1.56]	1.41 [1.25-1.59]		1.26 [1.12-1.43]	1.35 [1.20-1.52]	1.29 [1.14-1.45]	1.29 [1.14-1.46]		1.26 [1.12-1.43]	741	736	717	NA	670
440.2	1.28 [1.23-1.34]	1.28 [1.22-1.34]	1.29 [1.23-1.35]		1.19 [1.14-1.25]	1.24 [1.19-1.30]	1.16 [1.11-1.21]	1.16 [1.10-1.21]		1.19 [1.14-1.25]	5,105	5,107	5,091	NA	4,665
440.9	1.21 [1.14-1.28]	1.20 [1.13-1.27]	1.23 [1.16-1.30]		1.13 [1.07-1.20]	1.16 [1.10-1.23]	1.10 [1.04-1.16]	1.12 [1.05-1.19]		1.12 [1.05-1.18]	3,247	3,246	3,234	NA	3,045
441	1.25 [1.20-1.30]	1.25 [1.20-1.30]	1.25 [1.20-1.31]		1.15 [1.10-1.21]	1.19 [1.14-1.24]	1.14 [1.10-1.20]	1.14 [1.09-1.19]		1.13 [1.08-1.19]	5,570	5,532	5,358	NA	5,112
441.1	1.23 [1.14-1.33]	1.21 [1.12-1.31]	1.21 [1.12-1.31]		1.13 [1.04-1.22]	1.17 [1.08-1.26]	1.12 [1.03-1.21]	1.11 [1.02-1.20]		1.11 [1.02-1.20]	1,671	1,643	1,568	NA	1,502
441.2	1.24 [1.05-1.45]	1.24 [1.05-1.46]	1.22 [1.03-1.44]		1.11 [0.94-1.31]	1.17 [0.99-1.37]	1.08 [0.91-1.28]	1.09 [0.92-1.29]		1.10 [0.93-1.30]	399	390	379	NA	380
442	1.17 [1.10-1.25]	1.18 [1.11-1.25]	1.18 [1.10-1.25]		1.11 [1.04-1.18]	1.14 [1.07-1.21]	1.09 [1.02-1.16]	1.09 [1.02-1.16]		1.10 [1.03-1.17]	2,555	2,547	2,443	NA	2,312
442.1	1.15 [1.09-1.22]	1.15 [1.09-1.22]	1.17 [1.11-1.23]		1.07 [1.01-1.13]	1.12 [1.06-1.18]	1.09 [1.03-1.15]	1.09 [1.03-1.15]		1.06 [1.00-1.12]	3,563	3,514	3,430	NA	3,251
442.11	1.19 [1.15-1.23]	1.19 [1.15-1.24]	1.20 [1.16-1.24]		1.14 [1.10-1.18]	1.18 [1.14-1.22]	1.13 [1.09-1.17]	1.13 [1.09-1.17]		1.15 [1.11-1.19]	8,355	8,345	8,343	NA	7,485
442.2	1.12 [0.99-1.26]	1.11 [0.98-1.24]	1.13 [1.01-1.28]		1.02 [0.90-1.15]	1.10 [0.97-1.24]	1.04 [0.92-1.17]	1.06 [0.94-1.20]		1.02 [0.90-1.15]	708	708	705	NA	633
442.3	1.22 [1.09-1.36]	1.22 [1.09-1.37]	1.21 [1.08-1.35]		1.14 [1.02-1.29]	1.18 [1.05-1.32]	1.11 [0.99-1.24]	1.09 [0.97-1.23]		1.13 [1.01-1.27]	790	789	770	NA	715
442.8	1.17 [1.06-1.30]	1.18 [1.07-1.30]	1.18 [1.06-1.31]		1.14 [1.02-1.26]	1.14 [1.03-1.27]	1.10 [0.99-1.22]	1.10 [0.99-1.23]		1.13 [1.02-1.25]	976	970	903	NA	896
443	1.40 [1.37-1.43]	1.40 [1.37-1.43]	1.39 [1.36-1.42]	1.44 [1.24-1.68]	1.29 [1.26-1.32]	1.32 [1.29-1.35]	1.24 [1.21-1.27]	1.23 [1.20-1.26]	1.35 [1.15-1.58]	1.25 [1.22-1.28]	21,801	21,629	20,652	481	19,729
443.1	1.59 [1.52-1.66]	1.56 [1.49-1.63]	1.59 [1.51-1.67]	1.51 [1.29-1.78]	1.46 [1.40-1.53]	1.43 [1.37-1.50]	1.35 [1.29-1.42]	1.38 [1.32-1.46]	1.41 [1.19-1.68]	1.33 [1.27-1.40]	5,376	5,213	4,374	438	4,803
443.7	1.39 [1.33-1.47]	1.39 [1.32-1.46]	1.38 [1.31-1.45]		1.30 [1.23-1.37]	1.32 [1.25-1.39]	1.20 [1.14-1.26]	1.18 [1.12-1.24]		1.27 [1.21-1.34]	3,937	3,934	3,870	NA	3,675
443.8	1.40 [1.37-1.43]	1.40 [1.37-1.43]	1.39 [1.36-1.42]	1.44 [1.24-1.68]	1.29 [1.26-1.32]	1.32 [1.29-1.35]	1.24 [1.21-1.27]	1.23 [1.20-1.26]	1.35 [1.15-1.58]	1.25 [1.22-1.28]	21,801	21,629	20,652	481	19,729
443.9	1.35 [1.32-1.39]	1.36 [1.32-1.39]	1.35 [1.32-1.38]		1.25 [1.22-1.28]	1.29 [1.26-1.32]	1.21 [1.18-1.24]	1.20 [1.17-1.23]		1.22 [1.19-1.26]	16,835	16,835	16,695	NA	15,327
444	1.18 [1.13-1.23]	1.17 [1.12-1.22]	1.19 [1.14-1.25]		1.11 [1.06-1.17]	1.14 [1.09-1.19]	1.07 [1.02-1.12]	1.09 [1.04-1.14]		1.11 [1.06-1.17]	4,919	4,896	4,775	NA	4,434
444.1	1.19 [1.12-1.26]	1.18 [1.12-1.25]	1.21 [1.14-1.28]		1.12 [1.05-1.18]	1.15 [1.08-1.21]	1.08 [1.02-1.14]	1.09 [1.03-1.16]		1.12 [1.05-1.18]	3,172	3,161	3,123	NA	2,865
444.2	1.12 [0.92-1.37]	1.10 [0.90-1.34]	1.21 [0.99-1.48]		1.03 [0.83-1.26]	1.12 [0.91-1.37]	1.01 [0.83-1.24]	1.12 [0.91-1.38]		1.06 [0.86-1.31]	249	249	248	NA	228
446	1.39 [1.21-1.59]	1.47 [1.19-1.81]	1.41 [1.12-1.76]	1.28 [1.07-1.53]	1.31 [1.13-1.52]	1.28 [1.12-1.48]	1.23 [0.99-1.53]	1.17 [0.92-1.48]	1.29 [1.07-1.55]	1.23 [1.06-1.43]	525	229	193	309	456
446.2	1.26 [1.05-1.51]			1.24 [1.03-1.49]	1.24 [1.02-1.51]	1.27 [1.05-1.53]			1.26 [1.04-1.52]	1.26 [1.03-1.54]	294	NA	NA	288	261
446.3	1.52 [1.16-1.98]	1.52 [1.15-2.00]	1.48 [1.11-1.97]		1.16 [0.87-1.55]	1.51 [1.16-1.98]	1.47 [1.11-1.94]	1.42 [1.06-1.89]		1.15 [0.86-1.54]	140	134	120	NA	116
446.4	1.53 [1.29-1.80]	1.50 [1.27-1.77]	1.56 [1.31-1.86]		1.44 [1.21-1.72]	1.37 [1.16-1.63]	1.32 [1.11-1.57]	1.36 [1.13-1.64]		1.30 [1.08-1.56]	373	365	318	NA	308
446.5	1.31 [1.22-1.40]	1.31 [1.23-1.41]	1.30 [1.22-1.40]		1.23 [1.15-1.33]	1.22 [1.14-1.31]	1.21 [1.12-1.30]	1.19 [1.11-1.28]		1.16 [1.07-1.25]	2,049	2,051	2,043	NA	1,841
446.6	1.49 [1.14-1.94]	1.47 [1.12-1.93]	1.44 [1.07-1.92]		1.30 [0.97-1.74]	1.40 [1.07-1.83]	1.37 [1.04-1.81]	1.30 [0.97-1.76]		1.23 [0.92-1.66]	141	131	115	NA	118
446.8	1.30 [0.96-1.75]	1.44 [1.05-1.98]				1.24 [0.91-1.68]	1.29 [0.93-1.78]				113	102	NA	NA	NA
446.9	1.59 [1.45-1.74]	1.55 [1.41-1.70]	1.52 [1.38-1.67]		1.42 [1.30-1.56]	1.47 [1.34-1.61]	1.41 [1.28-1.54]	1.37 [1.24-1.51]		1.34 [1.22-1.48]	1,278	1,236	1,120	NA	1,148
447	1.30 [1.26-1.35]	1.32 [1.27-1.37]	1.33 [1.28-1.38]	1.06 [0.87-1.29]	1.21 [1.16-1.26]	1.25 [1.20-1.30]	1.19 [1.15-1.24]	1.20 [1.15-1.25]	1.03 [0.84-1.27]	1.20 [1.15-1.24]	7,470	7,317	7,026	253	6,714
447.1	1.30 [1.24-1.36]	1.30 [1.24-1.36]	1.32 [1.26-1.38]		1.19 [1.14-1.25]	1.25 [1.19-1.31]	1.16 [1.11-1.22]	1.18 [1.13-1.24]		1.20 [1.14-1.26]	4,377	4,368	4,339	NA	3,969
448	1.30 [1.21-1.40]	1.35 [1.25-1.45]	1.31 [1.21-1.42]	1.03 [0.77-1.36]	1.24 [1.15-1.34]	1.24 [1.15-1.34]	1.24 [1.14-1.34]	1.21 [1.12-1.31]	1.01 [0.76-1.35]	1.21 [1.12-1.31]	1,829	1,727	1,615	121	1,655
450	1.52 [1.46-1.59]	1.52 [1.46-1.59]	1.50 [1.44-1.57]	1.15 [0.91-1.47]	1.41 [1.35-1.48]	1.43 [1.37-1.49]	1.37 [1.31-1.44]	1.36 [1.30-1.42]	1.08 [0.84-1.38]	1.37 [1.31-1.43]	5,602	5,490	5,228	177	5,193
451	1.25 [1.22-1.29]	1.24 [1.21-1.28]	1.25 [1.21-1.28]	1.15 [0.98-1.36]	1.19 [1.15-1.22]	1.20 [1.17-1.23]	1.16 [1.12-1.19]	1.16 [1.13-1.20]	1.11 [0.93-1.32]	1.15 [1.12-1.19]	13,733	13,610	12,516	371	11,862
451.2	1.24 [1.21-1.27]	1.23 [1.20-1.26]	1.24 [1.21-1.28]	1.05 [0.87-1.27]	1.18 [1.14-1.21]	1.19 [1.15-1.22]	1.15 [1.12-1.18]	1.16 [1.13-1.20]	1.01 [0.82-1.23]	1.14 [1.11-1.18]	12,447	12,365	11,446	279	10,705
452	1.18 [1.11-1.26]	1.19 [1.12-1.27]	1.17 [1.09-1.25]	0.95 [0.74-1.21]	1.09 [1.02-1.17]	1.13 [1.06-1.20]	1.11 [1.04-1.18]	1.08 [1.01-1.16]	0.94 [0.73-1.21]	1.07 [1.00-1.15]	2,397	2,320	2,043	157	2,130
454	1.31 [1.22-1.41]	1.36 [1.26-1.47]	1.36 [1.24-1.49]	1.03 [0.88-1.21]	1.24 [1.15-1.34]	1.22 [1.14-1.32]	1.22 [1.13-1.32]	1.21 [1.10-1.33]	0.99 [0.84-1.17]	1.21 [1.12-1.31]	1,918	1,691	1,175	412	1,611
454.1	1.63 [1.59-1.67]	1.63 [1.59-1.67]	1.68 [1.64-1.72]	1.05 [0.86-1.27]	1.52 [1.48-1.56]	1.57 [1.54-1.61]	1.55 [1.52-1.59]	1.59 [1.55-1.63]	1.01 [0.82-1.24]	1.48 [1.45-1.52]	19,256	19,220	17,136	254	15,892
454.11	2.21 [2.12-2.30]	2.19 [2.11-2.28]	2.19 [2.10-2.27]		2.05 [1.97-2.14]	2.14 [2.06-2.23]	2.07 [1.99-2.15]	2.05 [1.97-2.14]		2.02 [1.93-2.10]	8,205	8,205	7,948	NA	7,356
456	1.88 [1.76-2.01]	1.85 [1.73-1.98]	1.86 [1.74-1.99]		1.75 [1.63-1.87]	1.78 [1.66-1.90]	1.69 [1.58-1.81]	1.71 [1.59-1.83]		1.69 [1.58-1.81]	2,673	2,670	2,587	NA	2,497
458	1.21 [1.17-1.25]	1.22 [1.17-1.26]	1.21 [1.16-1.26]	1.10 [0.94-1.29]	1.13 [1.09-1.18]	1.17 [1.12-1.21]	1.13 [1.09-1.18]	1.13 [1.08-1.17]	1.08 [0.92-1.27]	1.12 [1.07-1.16]	7,942	7,770	6,977	443	7,319
458.1	1.21 [1.18-1.24]	1.21 [1.18-1.24]	1.21 [1.18-1.24]	1.12 [0.90-1.38]	1.15 [1.12-1.18]	1.16 [1.13-1.19]	1.11 [1.09-1.14]	1.12 [1.09-1.15]	1.04 [0.83-1.30]	1.12 [1.10-1.15]	17,005	16,905	16,578	233	16,016
458.2	1.23 [1.14-1.32]	1.27 [1.18-1.37]	1.25 [1.16-1.34]		1.20 [1.11-1.29]	1.18 [1.10-1.27]	1.17 [1.08-1.26]	1.14 [1.05-1.23]		1.17 [1.09-1.26]	1,976	1,972	1,937	NA	1,851
458.9	1.20 [1.18-1.22]	1.21 [1.19-1.23]	1.20 [1.19-1.22]	1.06 [0.97-1.16]	1.15 [1.13-1.16]	1.16 [1.14-1.17]	1.12 [1.11-1.14]	1.12 [1.10-1.14]	1.04 [0.95-1.14]	1.13 [1.11-1.15]	44,151	43,521	41,490	1,343	41,026
459	1.20 [1.07-1.34]	1.22 [1.09-1.37]	1.25 [1.10-1.41]		1.11 [0.99-1.25]	1.17 [1.04-1.31]	1.14 [1.02-1.29]	1.17 [1.03-1.32]		1.11 [0.98-1.26]	745	707	634	NA	661
459.1	1.35 [1.19-1.53]	1.34 [1.17-1.52]	1.34 [1.17-1.54]		1.30 [1.14-1.48]	1.30 [1.14-1.48]	1.22 [1.07-1.40]	1.22 [1.06-1.41]		1.29 [1.13-1.48]	644	582	533	NA	597
459.9	1.25 [1.23-1.26]	1.25 [1.24-1.26]	1.25 [1.24-1.27]	1.03 [0.96-1.11]	1.15 [1.14-1.17]	1.18 [1.17-1.20]	1.14 [1.13-1.15]	1.14 [1.13-1.15]	0.99 [0.92-1.07]	1.12 [1.11-1.14]	82,816	81,957	77,655	1,925	75,883
respiratory															
464	1.46 [1.31-1.63]	1.38 [1.21-1.56]	1.38 [1.18-1.62]	1.44 [1.19-1.74]	1.28 [1.14-1.44]	1.31 [1.17-1.47]	1.18 [1.03-1.35]	1.16 [0.99-1.37]	1.36 [1.12-1.66]	1.19 [1.05-1.34]	851	617	388	306	714
465	1.30 [1.28-1.33]	1.36 [1.30-1.42]	1.36 [1.29-1.43]	1.29 [1.27-1.32]	1.25 [1.22-1.27]	1.25 [1.22-1.27]	1.18 [1.13-1.24]	1.19 [1.13-1.26]	1.25 [1.22-1.28]	1.20 [1.18-1.23]	25,551	5,142	3,385	21,050	22,206
465.2	1.32 [1.27-1.38]	1.39 [1.31-1.46]	1.44 [1.34-1.54]	1.19 [1.13-1.26]	1.23 [1.18-1.28]	1.24 [1.19-1.29]	1.24 [1.17-1.31]	1.26 [1.18-1.36]	1.15 [1.09-1.22]	1.18 [1.13-1.23]	6,206	3,525	2,082	3,263	5,339
465.4	1.34 [1.19-1.51]	1.47 [1.28-1.68]	1.41 [1.21-1.65]	1.32 [1.07-1.63]	1.33 [1.17-1.50]	1.23 [1.09-1.39]	1.29 [1.12-1.48]	1.19 [1.01-1.40]	1.29 [1.05-1.60]	1.23 [1.08-1.40]	724	538	396	242	633
470	1.55 [1.50-1.59]	1.53 [1.48-1.58]	1.52 [1.45-1.59]	1.56 [1.48-1.64]	1.42 [1.37-1.47]	1.38 [1.33-1.42]	1.29 [1.25-1.34]	1.29 [1.23-1.35]	1.44 [1.36-1.52]	1.28 [1.24-1.33]	11,262	8,889	4,277	4,190	8,388
471	1.56 [1.49-1.64]	1.54 [1.47-1.62]	1.52 [1.44-1.61]	1.41 [1.21-1.64]	1.42 [1.35-1.50]	1.29 [1.23-1.36]	1.25 [1.19-1.32]	1.25 [1.18-1.32]	1.21 [1.03-1.42]	1.20 [1.14-1.27]	4,446	4,224	3,253	471	3,366
472	1.75 [1.66-1.86]	1.70 [1.59-1.81]	1.59 [1.47-1.72]	1.96 [1.78-2.17]	1.62 [1.52-1.72]	1.44 [1.35-1.53]	1.33 [1.24-1.43]	1.24 [1.14-1.34]	1.68 [1.52-1.87]	1.34 [1.26-1.43]	3,383	2,421	1,625	1,259	2,875
473	1.35 [1.28-1.42]	1.37 [1.30-1.45]	1.35 [1.27-1.43]	1.23 [1.06-1.41]	1.24 [1.18-1.31]	1.24 [1.18-1.31]	1.18 [1.12-1.25]	1.18 [1.11-1.25]	1.12 [0.96-1.29]	1.16 [1.10-1.23]	3,700	3,315	2,863	499	3,171
473.1	1.39 [1.07-1.81]	1.40 [1.07-1.84]	1.31 [0.98-1.75]		1.19 [0.90-1.58]	1.24 [0.95-1.63]	1.15 [0.86-1.53]	1.10 [0.81-1.49]		1.09 [0.82-1.45]	143	129	108	NA	118
473.3	1.19 [1.09-1.30]	1.19 [1.08-1.31]	1.22 [1.10-1.35]	1.17 [0.94-1.47]	1.14 [1.03-1.25]	1.13 [1.03-1.24]	1.10 [0.99-1.22]	1.14 [1.03-1.27]	1.10 [0.88-1.39]	1.12 [1.02-1.23]	1,165	1,007	912	198	1,063
473.4	1.35 [1.27-1.44]	1.35 [1.27-1.44]	1.32 [1.24-1.42]	1.25 [1.05-1.48]	1.23 [1.15-1.31]	1.23 [1.16-1.32]	1.17 [1.10-1.26]	1.14 [1.07-1.23]	1.20 [1.01-1.42]	1.15 [1.08-1.23]	2,591	2,269	2,050	364	2,279
474	1.14 [1.07-1.22]	1.16 [1.08-1.24]	1.14 [1.00-1.30]	1.11 [1.01-1.22]	1.07 [1.00-1.15]	1.11 [1.04-1.18]	1.11 [1.03-1.19]	1.09 [0.95-1.25]	1.10 [1.00-1.21]	1.06 [0.99-1.15]	2,461	1,982	525	1,193	1,763
474.1	1.22 [1.20-1.24]	1.31 [1.27-1.36]	1.25 [1.14-1.36]	1.20 [1.17-1.22]	1.17 [1.15-1.19]	1.17 [1.15-1.19]	1.17 [1.13-1.21]	1.08 [0.98-1.19]	1.16 [1.14-1.18]	1.14 [1.11-1.16]	32,053	8,122	1,121	27,861	26,714
474.2	1.28 [1.26-1.31]	1.30 [1.24-1.36]	1.23 [1.13-1.35]	1.28 [1.25-1.30]	1.24 [1.21-1.27]	1.23 [1.21-1.26]	1.17 [1.11-1.23]	1.10 [1.01-1.21]	1.23 [1.20-1.26]	1.20 [1.17-1.23]	25,871	4,000	1,120	23,193	20,766
475	1.42 [1.36-1.47]	1.46 [1.40-1.52]	1.42 [1.35-1.49]	1.31 [1.20-1.44]	1.32 [1.27-1.38]	1.22 [1.18-1.28]	1.20 [1.15-1.26]	1.18 [1.13-1.25]	1.21 [1.10-1.33]	1.17 [1.12-1.22]	6,587	5,843	4,146	1,286	5,596
476	2.14 [2.08-2.20]	1.76 [1.71-1.82]	1.67 [1.60-1.74]	2.83 [2.72-2.95]	2.03 [1.98-2.09]	1.73 [1.68-1.78]	1.32 [1.27-1.36]	1.29 [1.23-1.35]	2.38 [2.28-2.48]	1.66 [1.61-1.71]	17,750	10,536	6,066	9,124	15,193
477	1.29 [1.26-1.33]	1.30 [1.26-1.34]	1.29 [1.25-1.34]	1.26 [1.18-1.34]	1.20 [1.16-1.24]	1.24 [1.20-1.27]	1.21 [1.17-1.25]	1.20 [1.16-1.24]	1.21 [1.14-1.29]	1.17 [1.14-1.21]	11,914	9,611	8,860	2,635	10,369
478	1.30 [1.17-1.44]	1.29 [1.15-1.45]	1.22 [1.06-1.40]	1.29 [1.07-1.56]	1.18 [1.06-1.32]	1.18 [1.06-1.31]	1.12 [1.00-1.27]	1.06 [0.92-1.22]	1.24 [1.02-1.50]	1.11 [0.99-1.24]	939	717	506	306	821
479	1.43 [1.39-1.47]	1.44 [1.40-1.49]	1.39 [1.34-1.45]	1.37 [1.31-1.44]	1.31 [1.27-1.35]	1.29 [1.26-1.33]	1.24 [1.20-1.28]	1.21 [1.17-1.26]	1.28 [1.22-1.34]	1.20 [1.17-1.24]	13,200	9,664	7,027	4,500	11,197
480	1.27 [1.26-1.29]	1.27 [1.26-1.28]	1.26 [1.25-1.28]	1.30 [1.26-1.35]	1.21 [1.20-1.22]	1.21 [1.20-1.22]	1.16 [1.15-1.18]	1.16 [1.14-1.17]	1.25 [1.21-1.30]	1.17 [1.16-1.19]	98,311	90,942	86,748	8,658	89,044
480.1	1.30 [1.25-1.35]	1.32 [1.27-1.38]	1.31 [1.25-1.36]	1.13 [1.00-1.28]	1.24 [1.19-1.29]	1.22 [1.17-1.27]	1.19 [1.14-1.24]	1.17 [1.12-1.22]	1.11 [0.98-1.25]	1.19 [1.14-1.23]	7,315	6,757	6,320	696	6,770
480.11	1.28 [1.26-1.29]	1.28 [1.26-1.29]	1.27 [1.25-1.29]	1.29 [1.24-1.34]	1.21 [1.19-1.23]	1.21 [1.19-1.22]	1.16 [1.15-1.18]	1.15 [1.14-1.17]	1.24 [1.19-1.30]	1.17 [1.15-1.18]	62,603	57,581	54,753	5,925	57,060
480.12	1.43 [1.28-1.60]	1.55 [1.38-1.74]	1.58 [1.40-1.77]		1.35 [1.20-1.51]	1.27 [1.13-1.42]	1.30 [1.15-1.47]	1.30 [1.15-1.47]		1.20 [1.07-1.36]	800	775	743	NA	747
480.2	1.28 [1.23-1.33]	1.29 [1.24-1.34]	1.31 [1.25-1.36]	1.14 [1.00-1.29]	1.23 [1.18-1.28]	1.19 [1.14-1.24]	1.16 [1.11-1.20]	1.16 [1.11-1.21]	1.08 [0.95-1.23]	1.17 [1.12-1.22]	7,159	6,672	6,277	619	6,720
480.3	1.47 [1.33-1.62]	1.49 [1.35-1.65]	1.49 [1.34-1.65]		1.34 [1.21-1.48]	1.36 [1.23-1.50]	1.33 [1.19-1.47]	1.33 [1.19-1.48]		1.26 [1.14-1.40]	1,055	989	895	NA	950
480.5	1.24 [1.19-1.29]	1.26 [1.21-1.31]	1.24 [1.19-1.29]	1.25 [1.06-1.47]	1.18 [1.14-1.23]	1.19 [1.14-1.24]	1.17 [1.12-1.22]	1.16 [1.11-1.21]	1.21 [1.02-1.43]	1.15 [1.10-1.20]	6,951	6,624	6,456	366	6,113
481	1.35 [1.29-1.41]	1.39 [1.32-1.45]	1.36 [1.29-1.43]	1.23 [1.12-1.34]	1.28 [1.22-1.33]	1.22 [1.17-1.28]	1.19 [1.13-1.25]	1.18 [1.11-1.24]	1.15 [1.05-1.25]	1.19 [1.13-1.24]	5,744	4,672	3,759	1,414	5,332
483	1.08 [1.04-1.12]	1.32 [1.21-1.43]	1.31 [1.19-1.43]	1.03 [0.98-1.07]	1.04 [1.00-1.09]	1.04 [1.00-1.08]	1.14 [1.05-1.24]	1.15 [1.04-1.26]	1.00 [0.96-1.04]	1.01 [0.97-1.05]	6,647	1,420	1,248	5,295	5,902

Supplementary Table 6: phecode-mapped results (hazard ratios and events from all cohorts)

	Hazard ratio (99% confidence interval)										Events (in exposed)				
	crude					adjusted									
Outcome	any age	18+	40+	<18	hosp.	any age	18+	40+	<18	hosp.	any age	18+	40+	<18	hosp.
495	2.12 [2.11-2.14]	1.98 [1.96-2.00]	1.84 [1.82-1.87]	2.50 [2.46-2.53]	1.93 [1.91-1.95]	1.61 [1.59-1.62]	1.34 [1.33-1.36]	1.31 [1.29-1.33]	2.06 [2.02-2.09]	1.51 [1.50-1.53]	165,066	117,603	75,502	63,223	122,304
495.2	2.95 [2.79-3.13]	2.46 [2.25-2.68]	2.08 [1.85-2.33]	3.34 [3.10-3.60]	2.59 [2.43-2.76]	2.29 [2.15-2.44]	1.55 [1.40-1.72]	1.35 [1.18-1.54]	2.76 [2.55-2.99]	2.01 [1.88-2.15]	3,968	1,559	809	2,673	3,324
496	1.48 [1.46-1.50]	1.48 [1.46-1.50]	1.48 [1.46-1.50]	1.60 [1.27-2.02]	1.37 [1.35-1.39]	1.30 [1.28-1.31]	1.21 [1.19-1.23]	1.21 [1.20-1.23]	1.39 [1.09-1.76]	1.23 [1.21-1.24]	56,567	56,445	55,924	218	50,540
496.1	1.35 [1.32-1.39]	1.35 [1.32-1.39]	1.36 [1.32-1.39]	1.21 [0.93-1.57]	1.28 [1.25-1.32]	1.25 [1.22-1.29]	1.18 [1.15-1.21]	1.18 [1.15-1.22]	1.17 [0.89-1.53]	1.21 [1.18-1.25]	15,001	14,949	14,688	151	13,692
496.2	1.45 [1.33-1.58]	1.41 [1.29-1.54]	1.41 [1.29-1.55]		1.29 [1.18-1.42]	1.28 [1.17-1.40]	1.18 [1.08-1.30]	1.18 [1.07-1.30]		1.16 [1.05-1.28]	1,286	1,246	1,191	NA	1,145
496.21	1.46 [1.44-1.48]	1.45 [1.44-1.47]	1.45 [1.43-1.47]	1.81 [1.49-2.20]	1.36 [1.34-1.38]	1.29 [1.27-1.31]	1.20 [1.19-1.22]	1.21 [1.19-1.22]	1.54 [1.26-1.89]	1.22 [1.21-1.24]	63,230	63,040	62,368	310	55,206
496.3	1.49 [1.45-1.54]	1.51 [1.47-1.56]	1.50 [1.45-1.55]	1.29 [1.06-1.58]	1.39 [1.35-1.44]	1.29 [1.25-1.33]	1.27 [1.23-1.31]	1.26 [1.22-1.30]	1.15 [0.93-1.42]	1.22 [1.18-1.26]	11,900	11,734	11,381	269	11,013
497	1.44 [1.36-1.53]	1.44 [1.36-1.53]	1.45 [1.36-1.54]	1.32 [0.96-1.82]	1.30 [1.23-1.39]	1.29 [1.21-1.37]	1.23 [1.16-1.31]	1.25 [1.17-1.33]	1.24 [0.89-1.73]	1.19 [1.12-1.27]	2,869	2,824	2,580	108	2,545
499	1.23 [0.96-1.58]				1.14 [0.87-1.50]	1.11 [0.86-1.43]				1.03 [0.78-1.36]	160	NA	NA	NA	134
500	1.37 [1.22-1.53]	1.34 [1.19-1.51]	1.44 [1.28-1.63]		1.22 [1.08-1.37]	1.30 [1.16-1.47]	1.22 [1.08-1.38]	1.32 [1.17-1.50]		1.18 [1.05-1.34]	763	735	709	NA	688
500.1	1.58 [1.37-1.82]	1.57 [1.36-1.81]	1.56 [1.35-1.81]		1.42 [1.22-1.65]	1.41 [1.22-1.63]	1.36 [1.17-1.58]	1.37 [1.18-1.60]		1.28 [1.10-1.49]	511	506	478	NA	453
500.2	1.35 [1.25-1.46]	1.35 [1.24-1.46]	1.35 [1.25-1.46]		1.28 [1.18-1.39]	1.28 [1.18-1.39]	1.24 [1.14-1.34]	1.24 [1.14-1.34]		1.22 [1.12-1.32]	1,684	1,684	1,678	NA	1,572
501	1.17 [1.14-1.20]	1.18 [1.14-1.21]	1.17 [1.14-1.21]	0.94 [0.79-1.12]	1.13 [1.09-1.16]	1.15 [1.11-1.18]	1.12 [1.09-1.16]	1.12 [1.08-1.15]	0.92 [0.77-1.10]	1.13 [1.10-1.17]	13,257	13,076	12,692	313	12,447
502	1.45 [1.40-1.50]	1.47 [1.42-1.52]	1.45 [1.40-1.50]		1.38 [1.33-1.44]	1.37 [1.32-1.42]	1.35 [1.30-1.40]	1.34 [1.29-1.39]		1.33 [1.28-1.38]	8,675	8,663	8,561	NA	7,999
503	1.30 [1.24-1.36]	1.28 [1.23-1.34]	1.31 [1.25-1.37]	0.98 [0.73-1.32]	1.19 [1.13-1.25]	1.25 [1.19-1.31]	1.18 [1.13-1.24]	1.21 [1.15-1.26]	0.97 [0.71-1.31]	1.17 [1.11-1.22]	4,997	4,933	4,786	110	4,488
504	1.54 [1.45-1.64]	1.52 [1.43-1.62]	1.55 [1.45-1.65]		1.45 [1.35-1.54]	1.45 [1.36-1.54]	1.38 [1.29-1.47]	1.40 [1.31-1.50]		1.39 [1.30-1.48]	2,740	2,710	2,635	NA	2,545
505	2.40 [2.08-2.78]	2.31 [2.00-2.68]	2.11 [1.80-2.47]		2.24 [1.93-2.59]	1.72 [1.47-2.02]	1.55 [1.31-1.82]	1.49 [1.25-1.77]		1.69 [1.44-1.98]	600	574	451	NA	570
506	1.16 [1.11-1.21]	1.16 [1.11-1.21]	1.19 [1.13-1.24]	1.06 [0.95-1.18]	1.09 [1.04-1.14]	1.12 [1.08-1.17]	1.09 [1.04-1.14]	1.11 [1.06-1.17]	1.07 [0.96-1.19]	1.08 [1.03-1.13]	5,655	5,112	4,419	862	4,745
507	1.20 [1.19-1.22]	1.20 [1.19-1.22]	1.21 [1.19-1.22]	1.10 [1.02-1.20]	1.15 [1.13-1.17]	1.17 [1.15-1.18]	1.13 [1.11-1.15]	1.13 [1.11-1.15]	1.11 [1.02-1.20]	1.13 [1.12-1.15]	50,126	49,149	47,087	1,605	45,275
508	1.22 [1.19-1.25]	1.21 [1.18-1.24]	1.21 [1.18-1.24]	1.19 [1.09-1.31]	1.16 [1.13-1.19]	1.15 [1.12-1.18]	1.10 [1.07-1.13]	1.10 [1.07-1.12]	1.13 [1.03-1.24]	1.12 [1.09-1.15]	18,974	18,039	17,062	1,293	17,468
509.1	1.32 [1.30-1.34]	1.31 [1.29-1.34]	1.30 [1.28-1.33]	1.21 [1.07-1.36]	1.24 [1.22-1.26]	1.23 [1.21-1.25]	1.17 [1.14-1.19]	1.16 [1.13-1.18]	1.12 [0.99-1.26]	1.18 [1.16-1.21]	30,130	29,782	28,727	709	27,859
509.2	1.31 [1.29-1.34]	1.31 [1.29-1.34]	1.30 [1.28-1.33]	1.17 [1.02-1.33]	1.24 [1.21-1.26]	1.22 [1.20-1.25]	1.16 [1.14-1.19]	1.16 [1.13-1.18]	1.09 [0.95-1.25]	1.18 [1.15-1.20]	25,270	24,998	24,069	587	23,419
509.5	1.28 [1.17-1.40]	1.29 [1.17-1.42]	1.27 [1.15-1.41]	1.16 [0.92-1.48]	1.17 [1.06-1.29]	1.21 [1.10-1.32]	1.16 [1.05-1.28]	1.16 [1.05-1.29]	1.12 [0.88-1.43]	1.13 [1.03-1.25]	1,209	1,081	969	172	1,062
509.8	1.51 [1.45-1.57]	1.53 [1.47-1.59]	1.53 [1.46-1.59]	0.89 [0.74-1.07]	1.37 [1.31-1.42]	1.32 [1.27-1.38]	1.25 [1.20-1.31]	1.24 [1.19-1.30]	0.82 [0.68-0.99]	1.22 [1.17-1.28]	6,613	6,428	6,141	280	6,314
510	1.27 [1.22-1.32]	1.29 [1.24-1.34]	1.30 [1.25-1.35]	0.95 [0.82-1.10]	1.20 [1.16-1.25]	1.18 [1.13-1.22]	1.14 [1.10-1.19]	1.15 [1.11-1.20]	0.87 [0.75-1.01]	1.14 [1.10-1.19]	7,653	7,307	6,975	453	7,123
510.2	1.02 [0.79-1.30]	1.06 [0.82-1.38]	1.08 [0.81-1.43]		0.94 [0.73-1.22]	0.95 [0.74-1.22]	0.96 [0.73-1.25]	0.95 [0.71-1.27]		0.86 [0.66-1.13]	150	138	113	NA	132
512	1.25 [1.23-1.28]	1.24 [1.22-1.26]	1.23 [1.21-1.26]	1.25 [1.17-1.33]	1.18 [1.16-1.21]	1.17 [1.15-1.20]	1.12 [1.09-1.14]	1.11 [1.09-1.13]	1.17 [1.10-1.25]	1.14 [1.11-1.16]	28,140	26,101	24,543	2,583	25,833
512.1	1.96 [1.92-2.00]	1.49 [1.38-1.60]	1.41 [1.30-1.53]	2.00 [1.96-2.04]	1.87 [1.83-1.91]	1.88 [1.84-1.92]	1.24 [1.15-1.34]	1.20 [1.11-1.31]	1.92 [1.88-1.97]	1.80 [1.76-1.84]	29,127	1,915	1,583	27,345	26,362
512.2	1.25 [1.13-1.37]	1.27 [1.15-1.40]	1.26 [1.12-1.42]	1.16 [0.89-1.52]	1.12 [1.01-1.24]	1.13 [1.02-1.25]	1.09 [0.99-1.21]	1.06 [0.94-1.20]	1.15 [0.87-1.52]	1.04 [0.94-1.16]	1,041	993	700	145	921
512.7	1.35 [1.33-1.38]	1.33 [1.31-1.36]	1.33 [1.31-1.36]	1.39 [1.33-1.46]	1.26 [1.24-1.28]	1.25 [1.23-1.27]	1.18 [1.16-1.20]	1.18 [1.16-1.20]	1.33 [1.27-1.40]	1.19 [1.17-1.21]	37,295	34,544	29,430	4,423	33,881
512.8	1.38 [1.35-1.41]	1.35 [1.32-1.39]	1.36 [1.32-1.39]	1.37 [1.32-1.43]	1.29 [1.26-1.32]	1.27 [1.24-1.30]	1.19 [1.15-1.22]	1.19 [1.15-1.22]	1.30 [1.25-1.35]	1.21 [1.18-1.24]	20,701	14,452	12,383	6,900	18,750
512.9	1.35 [1.30-1.41]	1.35 [1.28-1.42]	1.34 [1.27-1.42]	1.33 [1.25-1.42]	1.26 [1.21-1.32]	1.25 [1.20-1.31]	1.17 [1.11-1.24]	1.16 [1.10-1.24]	1.26 [1.18-1.35]	1.19 [1.14-1.25]	6,008	3,624	3,038	2,578	5,501
513	1.52 [1.50-1.54]	1.34 [1.32-1.36]	1.33 [1.31-1.35]	1.76 [1.72-1.79]	1.43 [1.41-1.44]	1.43 [1.41-1.44]	1.18 [1.16-1.20]	1.18 [1.16-1.20]	1.70 [1.67-1.73]	1.36 [1.35-1.38]	74,870	41,003	34,545	35,908	67,136
513.3	1.46 [1.35-1.58]	1.53 [1.40-1.67]	1.58 [1.45-1.73]	1.05 [0.89-1.24]	1.34 [1.24-1.45]	1.33 [1.23-1.44]	1.30 [1.18-1.42]	1.31 [1.19-1.44]	0.99 [0.83-1.17]	1.25 [1.15-1.36]	1,670	1,361	1,267	342	1,554
513.31	1.34 [0.97-1.84]					1.30 [0.94-1.80]					104	NA	NA	NA	NA
513.4	1.31 [1.19-1.45]	1.28 [1.15-1.43]	1.19 [1.04-1.37]	1.34 [1.11-1.63]	1.17 [1.05-1.31]	1.15 [1.04-1.28]	1.05 [0.94-1.19]	0.99 [0.85-1.14]	1.21 [0.99-1.48]	1.07 [0.95-1.19]	972	785	495	292	827
513.8	1.29 [1.15-1.44]	1.34 [1.19-1.51]	1.30 [1.15-1.47]		1.26 [1.11-1.42]	1.22 [1.08-1.37]	1.22 [1.08-1.38]	1.19 [1.05-1.36]		1.22 [1.08-1.37]	734	715	651	NA	679
514	1.26 [1.22-1.29]	1.25 [1.22-1.29]	1.25 [1.22-1.29]	1.29 [1.09-1.53]	1.20 [1.17-1.24]	1.20 [1.17-1.24]	1.15 [1.12-1.18]	1.15 [1.12-1.18]	1.26 [1.06-1.50]	1.17 [1.14-1.21]	13,726	13,469	13,019	377	12,284
514.1	1.41 [1.13-1.76]	1.46 [1.15-1.86]	1.41 [1.09-1.82]		1.25 [0.99-1.58]	1.29 [1.02-1.62]	1.28 [1.00-1.64]	1.19 [0.92-1.56]		1.15 [0.91-1.46]	210	177	157	NA	181
516	1.31 [1.20-1.44]	1.37 [1.24-1.51]	1.45 [1.31-1.61]	1.04 [0.86-1.26]	1.20 [1.09-1.31]	1.17 [1.07-1.29]	1.15 [1.04-1.27]	1.23 [1.10-1.37]	0.97 [0.80-1.19]	1.10 [1.00-1.20]	1,289	1,065	969	267	1,185
516.1	1.33 [1.30-1.36]	1.35 [1.31-1.38]	1.34 [1.31-1.38]	1.24 [1.17-1.31]	1.23 [1.20-1.26]	1.25 [1.22-1.28]	1.22 [1.18-1.25]	1.21 [1.18-1.24]	1.19 [1.12-1.26]	1.19 [1.16-1.22]	18,561	15,989	14,425	3,187	16,162
519	1.61 [1.40-1.85]	1.68 [1.45-1.96]	1.59 [1.36-1.86]		1.50 [1.30-1.73]	1.45 [1.26-1.67]	1.40 [1.20-1.64]	1.38 [1.17-1.63]		1.37 [1.18-1.58]	556	475	423	NA	500
519.2	1.21 [1.10-1.34]	1.21 [1.10-1.34]	1.25 [1.12-1.38]		1.13 [1.01-1.25]	1.17 [1.06-1.29]	1.12 [1.01-1.24]	1.17 [1.05-1.30]		1.11 [1.00-1.24]	999	921	863	NA	843
519.8	1.37 [1.35-1.39]	1.35 [1.34-1.37]	1.32 [1.30-1.34]	1.43 [1.40-1.46]	1.29 [1.27-1.30]	1.27 [1.26-1.29]	1.20 [1.19-1.22]	1.19 [1.17-1.21]	1.35 [1.32-1.38]	1.22 [1.20-1.23]	82,314	62,929	55,037	22,170	74,222
519.9	1.27 [1.16-1.38]	1.23 [1.12-1.36]	1.28 [1.15-1.43]	1.16 [0.95-1.42]	1.16 [1.05-1.27]	1.20 [1.10-1.32]	1.13 [1.03-1.25]	1.19 [1.06-1.33]	1.13 [0.92-1.38]	1.12 [1.01-1.23]	1,228	1,032	850	253	1,056
digestive															
520	1.16 [1.12-1.21]	1.15 [1.06-1.25]	1.22 [1.02-1.47]	1.14 [1.09-1.19]	1.09 [1.05-1.15]	1.12 [1.08-1.17]	1.05 [0.96-1.14]	1.12 [0.93-1.35]	1.11 [1.06-1.16]	1.07 [1.02-1.13]	6,500	1,359	276	5,703	4,997
520.1	1.15 [1.07-1.22]	1.14 [0.95-1.36]		1.13 [1.06-1.21]	1.06 [0.98-1.14]	1.12 [1.05-1.20]	1.10 [0.91-1.32]		1.11 [1.04-1.19]	1.05 [0.97-1.13]	2,644	273	NA	2,518	2,085
520.2	1.21 [1.18-1.24]	1.23 [1.19-1.27]	1.15 [1.07-1.24]	1.18 [1.14-1.22]	1.13 [1.10-1.17]	1.15 [1.13-1.18]	1.13 [1.09-1.16]	1.10 [1.02-1.18]	1.14 [1.10-1.17]	1.10 [1.07-1.13]	17,520	9,951	1,617	10,828	12,168
521	1.21 [1.01-1.46]	1.45 [1.09-1.94]		1.14 [0.91-1.43]	1.07 [0.88-1.31]	1.15 [0.96-1.40]	1.34 [0.99-1.80]		1.11 [0.88-1.40]	1.05 [0.86-1.29]	303	113	NA	208	256
521.1	1.11 [1.09-1.13]	1.25 [1.22-1.28]	1.28 [1.24-1.32]	1.02 [1.00-1.04]	1.05 [1.03-1.07]	1.07 [1.05-1.09]	1.12 [1.10-1.15]	1.15 [1.11-1.19]	1.01 [0.99-1.03]	1.03 [1.01-1.05]	42,689	17,603	8,465	28,068	34,584
521.2	1.11 [0.83-1.48]				1.10 [0.81-1.48]	1.00 [0.74-1.36]				1.01 [0.74-1.38]	121	NA	NA	NA	114
521.4	1.10 [0.91-1.34]	1.19 [0.88-1.60]		1.05 [0.84-1.32]	1.11 [0.90-1.37]	1.08 [0.88-1.32]	1.08 [0.79-1.47]		1.05 [0.83-1.33]	1.10 [0.89-1.37]	285	104	NA	216	240
522	1.14 [1.10-1.18]	1.22 [1.17-1.27]	1.25 [1.19-1.32]	1.00 [0.95-1.05]	1.07 [1.03-1.11]	1.09 [1.05-1.13]	1.10 [1.05-1.15]	1.13 [1.07-1.19]	0.99 [0.94-1.05]	1.04 [1.00-1.08]	8,999	5,838	3,471	3,950	7,404
522.1	1.28 [1.09-1.51]	1.35 [1.11-1.65]	1.32 [1.01-1.73]	1.17 [0.91-1.50]	1.22 [1.02-1.47]	1.16 [0.98-1.38]	1.12 [0.91-1.39]	1.08 [0.81-1.44]	1.13 [0.88-1.46]	1.13 [0.94-1.36]	370	243	126	169	312
522.5	1.12 [1.08-1.16]	1.20 [1.14-1.26]	1.25 [1.17-1.33]	1.00 [0.94-1.05]	1.05 [1.00-1.09]	1.07 [1.03-1.11]	1.08 [1.03-1.14]	1.12 [1.05-1.20]	0.99 [0.94-1.05]	1.02 [0.98-1.07]	6,595	3,939	2,296	3,226	5,462
523	1.33 [1.24-1.42]	1.36 [1.27-1.47]	1.40 [1.29-1.53]	1.02 [0.89-1.16]	1.20 [1.12-1.29]	1.26 [1.18-1.35]	1.25 [1.16-1.35]	1.27 [1.16-1.39]	0.96 [0.84-1.10]	1.16 [1.08-1.24]	2,347	1,883	1,363	637	2,033
523.1	1.33 [1.19-1.50]	1.46 [1.26-1.68]	1.47 [1.23-1.76]	1.21 [1.01-1.43]	1.32 [1.16-1.49]	1.27 [1.12-1.43]	1.32 [1.14-1.53]	1.33 [1.10-1.60]	1.19 [0.99-1.42]	1.27 [1.12-1.44]	783	499	306	361	685
523.31	1.34 [1.19-1.52]	1.38 [1.21-1.57]	1.52 [1.23-1.89]	1.24 [1.00-1.55]	1.21 [1.05-1.39]	1.30 [1.14-1.47]	1.26 [1.10-1.44]	1.37 [1.10-1.72]	1.27 [1.01-1.59]	1.19 [1.04-1.37]	661	587	207	214	508
523.32	1.26 [1.22-1.31]	1.32 [1.28-1.37]	1.28 [1.19-1.37]	1.18 [1.11-1.26]	1.17 [1.12-1.22]	1.19 [1.14-1.23]	1.19 [1.14-1.23]	1.16 [1.08-1.25]	1.12 [1.05-1.20]	1.12 [1.07-1.17]	7,692	7,339	1,866	2,575	5,756
524	1.22 [0.95-1.57]			1.08 [0.80-1.47]	1.09 [0.81-1.45]	1.19 [0.92-1.54]			1.05 [0.77-1.45]	1.03 [0.76-1.39]	159	NA	NA	106	120
524.3	1.16 [1.12-1.21]	1.13 [1.07-1.20]	1.07 [0.92-1.25]	1.14 [1.09-1.19]	1.09 [1.04-1.14]	1.12 [1.08-1.17]	1.06 [1.00-1.13]	1.00 [0.85-1.16]	1.11 [1.07-1.16]	1.07 [1.03-1.12]	7,826	2,563	373	6,447	5,587
525	1.18 [1.15-1.22]	1.25 [1.21-1.29]	1.29 [1.24-1.34]	1.04 [0.99-1.08]	1.12 [1.08-1.15]	1.13 [1.10-1.16]	1.14 [1.10-1.17]	1.15 [1.11-1.20]	1.02 [0.98-1.07]	1.08 [1.05-1.12]	13,088	9,194	5,859	5,019	10,876
526.1	1.21 [1.08-1.36]	1.19 [1.05-1.36]	1.11 [0.95-1.29]	1.16 [0.93-1.44]	1.16 [1.01-1.32]	1.20 [1.06-1.35]	1.13 [0.99-1.29]	1.07 [0.91-1.25]	1.13 [0.90-1.42]	1.14 [0.99-1.30]	721	553	374	224	544
526.3	1.21 [1.06-1.38]	1.31 [1.13-1.52]		1.19 [1.02-1.39]	1.08 [0.93-1.25]	1.16 [1.01-1.32]	1.23 [1.06-1.44]		1.15 [0.98-1.35]	1.05 [0.90-1.22]	601	427	NA	439	449
526.41	1.44 [1.30-1.59]	1.47 [1.33-1.63]	1.51 [1.31-1.73]	1.38 [1.13-1.69]	1.36 [1.22-1.51]	1.27 [1.14-1.41]	1.22 [1.10-1.36]	1.28 [1.11-1.47]	1.24 [1.00-1.54]	1.23 [1.10-1.37]	1,041	967	522	270	907
526.5	1.16 [0.99-1.37]	1.17 [0.99-1.38]	1.12 [0.94-1.35]		1.06 [0.90-1.26]	1.09 [0.92-1.29]	1.03 [0.87-1.23]	1.01 [0.83-1.22]		1.03 [0.87-1.23]	364	344	278	NA	336
526.9	1.29 [1.16-1.43]	1.31 [1.17-1.47]	1.36 [1.20-1.54]	1.31 [1.02-1.68]	1.18 [1.06-1.32]	1.18 [1.05-1.31]	1.14 [1.01-1.28]	1.19 [1.04-1.36]	1.26 [0.98-1.63]	1.12 [1.00-1.26]	863	771	601	176	762
527	1.31 [1.24-1.39]	1.36 [1.28-1.45]	1.36 [1.27-1.46]	1.16 [1.04-1.30]	1.24 [1.16-1.32]	1.24 [1.17-1.32]	1.25 [1.17-1.33]	1.23 [1.15-1.33]	1.12 [1.00-1.25]	1.19 [1.12-1.27]	3,061	2,405	1,914	848	2,583
527.1	1.28 [0.99-1.65]	1.51 [1.15-1.99]			1.25 [0.95-1.66]	1.18 [0.91-1.53]	1.37 [1.03-1.82]			1.19 [0.89-1.58]	146	126	NA	NA	121
527.2	1.37 [1.25-1.50]	1.36 [1.23-1.49]	1.35 [1.21-1.50]	1.31 [1.08-1.59]	1.27 [1.16-1.40]	1.28 [1.17-1.40]	1.22 [1.11-1.35]	1.22 [1.09-1.36]	1.26 [1.03-1.54]	1.21 [1.10-1.34]	1,278	1,081	890	286	1,110
527.7	1.21 [1.05-1.41]	1.29 [1.07-1.56]	1.34 [1.10-1.64]	1.02 [0.81-1.28]	1.08 [0.93-1.26]	1.11 [0.95-1.29]	1.13 [0.93-1.38]	1.19 [0.97-1.47]	0.94 [0.75-1.19]	1.02 [0.87-1.19]	448	267	240	193	421
527.8	1.39 [1.18-1.65]	1.40 [1.18-1.66]	1.37 [1.14-1.65]		1.28 [1.07-1.53]	1.32 [1.11-1.56]	1.26 [1.06-1.51]	1.22 [1.00-1.48]		1.23 [1.02-1.47]	358	327	272	NA	303
528	1.37 [1.31-1.44]	1.38 [1.31-1.46]	1.39 [1.31-1.48]	1.26 [1.13-1.42]	1.29 [1.22-1.36]	1.30 [1.24-1.37]	1.27 [1.20-1.34]	1.27 [1.20-1.35]	1.23 [1.09-1.38]	1.25 [1.18-1.32]	4,011	3,375	2,729	821	3,297
528.11	1.41 [1.33-1.51]	1.45 [1.35-1.57]	1.43 [1.32-1.56]	1.33 [1.18-1.48]	1.32 [1.23-1.41]	1.34 [1.25-1.43]	1.32 [1.22-1.42]	1.30 [1.19-1.42]	1.28 [1.14-1.43]	1.27 [1.18-1.36]	2,454	1,776	1,423	831	2,177
528.12	1.38 [1.19-1.62]	1.42 [1.16-1.73]	1.46 [1.15-1.85]	1.34 [1.07-1.67]	1.29 [1.09-1.53]	1.31 [1.12-1.53]	1.22 [0.99-1.51]	1.25 [0.98-1.61]	1.28 [1.02-1.61]	1.24 [1.05-1.47]	419	246	180	203	362
528.3	1.06 [0.96-1.17]	1.11 [0.99-1.24]	1.16 [1.01-1.35]	0.95 [0.80-1.11]	1.04 [0.93-1.15]	1.04 [0.94-1.15]	1.02 [0.91-1.15]	1.09 [0.94-1.26]	0.97 [0.82-1.15]	1.05 [0.94-1.17]	979	716	438	367	843
528.41	1.17 [0.99-1.39]	1.25 [1.00-1.55]	1.38 [1.04-1.82]	1.10 [0.87-1.39]	1.18 [0.98-1.43]	1.13 [0.95-1.35]	1.20 [0.96-1.50]	1.25 [0.93-1.66]	1.07 [0.84-1.35]	1.14 [0.93-1.38]	356	197	119	193	273
528.5	1.37 [1.28-1.47]	1.40 [1.29-1.52]	1.36 [1.24-1.49]	1.37 [1.22-1.53]	1.34 [1.24-1.44]	1.30 [1.22-1.40]	1.26 [1.16-1.37]	1.23 [1.12-1.36]	1.33 [1.19-1.50]	1.27 [1.18-1.37]	2,191	1,468	1,105	863	1,819
528.6	1.40 [1.23-1.59]	1.36 [1.20-1.55]	1.43 [1.25-1.64]		1.34 [1.16-1.53]	1.33 [1.17-1.52]	1.24 [1.09-1.42]	1.29 [1.12-1.49]		1.30 [1.13-1.49]	584	568	519	NA	499
528.7	1.32 [1.15-1.52]	1.33 [1.15-1.53]	1.28 [1.08-1.52]		1.25 [1.08-1.46]	1.27 [1.10-1.46]	1.25 [1.07-1.45]	1.18 [0.99-1.40]		1.21 [1.04-1.42]	503	448	327	NA	407
529	1.39 [1.31-1.47]	1.40 [1.32-1.49]	1.37 [1.29-1.46]	1.32 [1.14-1.53]	1.29 [1.21-1.37]	1.28 [1.21-1.36]	1.24 [1.17-1.32]	1.23 [1.15-1.31]	1.26 [1.09-1.47]	1.21 [1.14-1.29]	3,114	2,746	2,364	495	2,705
529.1	1.42 [1.28-1.56]	1.49 [1.34-1.65]	1.48 [1.33-1.66]	1.29 [1.02-1.64]	1.34 [1.21-1.49]	1.31 [1.18-1.44]	1.30 [1.16-1.45]	1.28 [1.14-1.44]	1.25 [0.97-1.60]	1.26 [1.14-1.41]	1,040	899	793	182	922
530	1.39 [1.29-1.49]	1.37 [1.27-1.48]	1.35 [1.25-1.46]		1.26 [1.17-1.36]	1.27 [1.18-1.37]	1.21 [1.12-1.31]	1.21 [1.12-1.31]		1.19 [1.10-1.28]	1,815	1,780	1,646	NA	1,634
530.1	1.33 [1.30-1.36]	1.32 [1.29-1.36]	1.30 [1.27-1.34]	1.49 [1.35-1.64]	1.23 [1.20-1.26]	1.22 [1.19-1.25]	1.18 [1.15-1.20]	1.16 [1.13-1.19]	1.34 [1.21-1.49]	1.15 [1.12-1.18]	18,438	17,762	16,047	1,158	15,910
530.11	1.36 [1.34-1.37]	1.36 [1.35-1.38]	1.36 [1.34-1.37]	1.23 [1.18-1.29]	1.25 [1.23-1.26]	1.21 [1.20-1.23]	1.18 [1.17-1.19]	1.17 [1.16-1.19]	1.11 [1.06-1.17]	1.15 [1.13-1.16]	73,030	70,858	62,008	4,889	64,783
530.12	1.30 [1.26-1.34]	1.29 [1.25-1.34]	1.28 [1.24-1.32]	1.30 [1.03-1.64]	1.18 [1.14-1.22]	1.22 [1.18-1.26]	1.18 [1.14-1.22]	1.18 [1.14-1.22]	1.27 [1.00-1.61]	1.13 [1.09-1.17]	9,007	8,920	8,459	197	7,512
530.14	1.32 [1.29-1.35]	1.33 [1.30-1.36]	1.32 [1.29-1.35]	1.20 [1.08-1.33]	1.21 [1.19-1.24]	1.20 [1.18-1.23]	1.18 [1.15-1.20]	1.17 [1.14-1.20]	1.08 [0.97-1.20]	1.14 [1.11-1.16]	21,183	20,879	18,546	980	18,033
530.2	1.36 [1.31-1.41]	1.38 [1.33-1.43]	1.36 [1.31-1.42]	1.13 [0.93-1.37]	1.27 [1.23-1.33]	1.25 [1.20-1.30]	1.22 [1.17-1.27]	1.21 [1.16-1.26]	1.00 [0.82-1.23]	1.20 [1.16-1.25]	7,168	7,034	6,487	274	6,482
530.3	1.30 [1.25-1.36]	1.31 [1.26-1.36]	1.30 [1.24-1.35]	1.29 [0.99-1.67]	1.25 [1.20-1.30]	1.22 [1.18-1.28]	1.21 [1.16-1.26]	1.20 [1.15-1.25]	1.18 [0.90-1.55]	1.20 [1.15-1.25]	6,260	6,186	5,883	150	5,507
530.5	1.38 [1.29-1.47]	1.36 [1.28-1.46]	1.39 [1.30-1.49]	1.22 [0.89-1.68]	1.30 [1.22-1.39]	1.25 [1.17-1.33]	1.20 [1.12-1.28]	1.21 [1.13-1.30]	1.06 [0.76-1.48]	1.20 [1.13-1.29]	2,436	2,396	2,214	104	2,267
530.6	1.22 [1.08-1.38]	1.22 [1.08-1.37]	1.22 [1.08-1.38]		1.14 [1.01-1.29]	1.13 [1.00-1.28]	1.13 [1.00-1.27]	1.12 [0.99-1.27]		1.08 [0.95-1.22]	721	717	710	NA	661
530.7	1.20 [1.10-1.30]	1.25 [1.14-1.37]	1.32 [1.18-1.47]	1.11 [0.94-1.30]	1.12 [1.02-1.22]	1.11 [1.02-1.21]	1.09 [0.99-1.19]	1.18 [1.05-1.32]	1.05 [0.89-1.24]	1.07 [0.98-1.17]	1,404	1,173	826	389	1,217
530.9	1.35 [1.29-1.42]	1.35 [1.28-1.42]	1.34 [1.27-1.42]	1.08 [0.90-1.28]	1.23 [1.17-1.30]	1.22 [1.16-1.28]	1.18 [1.12-1.24]	1.17 [1.11-1.24]	1.00 [0.83-1.20]	1.14 [1.08-1.20]	4,130	4,046	3,156	336	3,611
531	1.21 [1.18-1.25]	1.22 [1.19-1.26]	1.21 [1.18-1.25]	1.26 [1.05-1.51]	1.16 [1.12-1.20]	1.17 [1.13-1.21]	1.14 [1.10-1.17]	1.13 [1.09-1.17]	1.21 [1.01-1.47]	1.15 [1.11-1.19]	10,355	10,202	9,563	325	8,880
531.1	1.21 [1.15-1.28]	1.21 [1.14-1.28]	1.21 [1.14-1.28]		1.16 [1.10-1.24]	1.21 [1.14-1.28]	1.16 [1.10-1.23]	1.16 [1.09-1.23]		1.18 [1.11-1.25]	3,261	3,239	3,113	NA	2,785
531.2	1.29 [1.26-1.33]	1.30 [1.26-1.33]	1.29 [1.25-1.32]	1.30 [1.10-1.54]	1.21 [1.17-1.24]	1.22 [1.18-1.25]	1.17 [1.14-1.20]	1.17 [1.13-1.20]	1.27 [1.07-1.52]	1.16 [1.13-1.20]	13,006	12,851	12,008	368	11,348
531.3	1.19 [1.15-1.23]	1.21 [1.17-1.25]	1.20 [1.16-1.24]	1.21 [0.99-1.48]	1.15 [1.11-1.19]	1.16 [1.12-1.20]	1.14 [1.10-1.18]	1.13 [1.09-1.17]	1.18 [0.95-1.46]	1.14 [1.10-1.18]	8,838	8,712	8,214	259	7,495
531.4	1.29 [1.19-1.40]	1.28 [1.18-1.39]	1.29 [1.18-1.40]		1.18 [1.09-1.28]	1.19 [1.10-1.29]	1.12 [1.03-1.22]	1.14 [1.04-1.24]		1.12 [1.03-1.22]	1,511	1,492	1,381	NA	1,373
531.5	1.53 [1.24-1.89]	1.55 [1.25-1.92]	1.45 [1.15-1.82]		1.48 [1.19-1.83]	1.29 [1.04-1.61]	1.25 [0.99-1.56]	1.09 [0.85-1.40]		1.32 [1.06-1.64]	232	226	183	NA	225
532	1.31 [1.29-1.34]	1.32 [1.29-1.34]	1.31 [1.29-1.34]	1.21 [1.11-1.32]	1.23 [1.21-1.25]	1.22 [1.19-1.24]	1.18 [1.16-1.21]	1.18 [1.16-1.21]	1.12 [1.02-1.23]	1.17 [1.15-1.19]	30,132	29,322	27,273	1,407	27,584
535	1.32 [1.31-1.34]	1.33 [1.31-1.35]	1.33 [1.31-1.35]	1.23 [1.17-1.28]	1.22 [1.21-1.24]	1.22 [1.21-1.24]	1.19 [1.17-1.20]	1.19 [1.17-1.20]	1.16 [1.11-1.22]	1.16 [1.15-1.18]	69,990	66,962	59,204	5,411	60,277
535.1	1.41 [1.34-1.48]	1.40 [1.33-1.47]	1.40 [1.32-1.47]	1.14 [0.93-1.38]	1.27 [1.21-1.34]	1.30 [1.24-1.37]	1.24 [1.18-1.31]	1.24 [1.17-1.31]	1.11 [0.91-1.36]	1.22 [1.15-1.29]	3,754	3,588	3,256	257	3,299
535.2	1.43 [1.31-1.55]	1.39 [1.28-1.51]	1.40 [1.28-1.52]		1.32 [1.21-1.44]	1.34 [1.23-1.45]	1.28 [1.18-1.39]	1.26 [1.16-1.38]		1.27 [1.16-1.38]	1,556	1,553	1,492	NA	1,404
535.6	1.31 [1.28-1.34]	1.31 [1.28-1.34]	1.30 [1.27-1.33]	1.30 [1.14-1.48]	1.20 [1.16-1.23]	1.23 [1.20-1.26]	1.19 [1.16-1.22]	1.18 [1.15-1.22]	1.21 [1.05-1.38]	1.16 [1.13-1.19]	15,226	14,943	13,538	637	12,984
535.8	1.33 [1.31-1.36]	1.34 [1.31-1.37]	1.33 [1.30-1.36]	1.35 [1.25-1.47]	1.24 [1.21-1.27]	1.23 [1.20-1.25]	1.18 [1.16-1.21]	1.18 [1.15-1.20]	1.27 [1.17-1.38]	1.17 [1.15-1.20]	23,457	22,460	20,228	1,673	20,800
537	1.30 [1.27-1.33]	1.31 [1.28-1.33]	1.30 [1.27-1.33]	1.11 [0.97-1.26]	1.21 [1.18-1.23]	1.18 [1.16-1.21]	1.15 [1.12-1.17]	1.14 [1.12-1.17]	1.04 [0.91-1.19]	1.13 [1.10-1.15]	22,728	22,438	21,097	633	20,728
539	1.32 [0.99-1.75]	1.41 [1.05-1.90]			1.20 [0.90-1.59]	1.11 [0.83-1.50]	1.20 [0.88-1.63]			1.11 [0.83-1.48]	119	112	NA	NA	117
540	1.15 [1.07-1.24]	1.16 [1.06-1.27]	1.10 [0.97-1.25]	1.08 [0.97-1.21]	1.06 [0.98-1.15]	1.10 [1.01-1.18]	1.06 [0.97-1.16]	1.05 [0.92-1.19]	1.05 [0.94-1.18]	1.04 [0.95-1.13]	1,735	1,171	581	837	1,394
540.1	1.14 [1.08-1.21]	1.14 [1.06-1.23]	1.14 [1.01-1.29]	1.10 [1.01-1.18]	1.07 [1.00-1.14]	1.08 [1.02-1.15]	1.02 [0.95-1.10]	1.06 [0.94-1.21]	1.06 [0.98-1.15]	1.03 [0.96-1.10]	2,929	1,691	600	1,758	2,275
540.11	1.08 [1.05-1.11]	1.09 [1.05-1.13]	1.05 [1.00-1.11]	1.04 [1.00-1.08]	1.01 [0.98-1.04]	1.04 [1.01-1.07]	1.03 [1.00-1.07]	1.01 [0.95-1.06]	1.02 [0.98-1.06]	0.99 [0.96-1.03]	13,415	7,735	3,340	7,734	9,842
550	1.27 [1.23-1.31]	1.29 [1.25-1.34]	1.30 [1.25-1.34]	1.13 [1.00-1.28]	1.18 [1.14-1.22]	1.17 [1.13-1.21]	1.15 [1.11-1.19]	1.15 [1.11-1.19]	1.06 [0.93-1.21]	1.12 [1.09-1.17]	8,593	8,112	7,263	632	7,848
550.1	1.10 [1.07-1.12]	1.11 [1.09-1.14]	1.11 [1.08-1.13]	1.02 [0.96-1.08]	1.06 [1.04-1.09]	1.06 [1.04-1.08]	1.08 [1.06-1.10]	1.07 [1.05-1.10]	0.98 [0.93-1.04]	1.04 [1.02-1.06]	23,888	21,407	19,508	3,081	18,644
550.2	1.31 [1.29-1.32]	1.32 [1.30-1.33]	1.30 [1.29-1.32]	1.27 [1.17-1.38]	1.22 [1.20-1.23]	1.20 [1.18-1.21]	1.17 [1.16-1.19]	1.16 [1.15-1.18]	1.17 [1.08-1.28]	1.14 [1.12-1.15]	66,300	66,090	60,673	1,682	57,225
550.3	1.19 [1.10-1.28]	1.18 [1.09-1.27]	1.16 [1.08-1.26]		1.12 [1.03-1.21]	1.13 [1.05-1.22]	1.12 [1.04-1.21]	1.10 [1.02-1.19]		1.07 [0.98-1.16]	1,699	1,670	1,555	NA	1,454
550.4	1.29 [1.25-1.32]	1.29 [1.26-1.33]	1.29 [1.25-1.34]	1.19 [1.10-1.28]	1.20 [1.17-1.24]	1.17 [1.14-1.21]	1.14 [1.11-1.18]	1.14 [1.10-1.17]	1.09 [1.00-1.18]	1.13 [1.09-1.16]	11,993	10,583	8,923	1,665	10,301
550.5	1.27 [1.23-1.31]	1.28 [1.24-1.32]	1.29 [1.25-1.34]	1.13 [1.00-1.27]	1.18 [1.14-1.22]	1.17 [1.13-1.20]	1.14 [1.10-1.18]	1.15 [1.11-1.19]	1.05 [0.93-1.19]	1.13 [1.09-1.16]	10,222	9,728	8,749	664	9,462
555.1	1.70 [1.63-1.77]	1.65 [1.58-1.72]	1.56 [1.47-1.64]	1.80 [1.65-1.96]	1.55 [1.48-1.62]	1.47 [1.41-1.54]	1.35 [1.29-1.42]	1.29 [1.22-1.36]	1.67 [1.53-1.82]	1.38 [1.32-1.45]	6,324	5,403	3,344	1,728	5,217
555.2	1.51 [1.46-1.56]	1.51 [1.46-1.57]	1.45 [1.39-1.51]	1.44 [1.31-1.58]	1.36 [1.31-1.42]	1.35 [1.30-1.40]	1.29 [1.24-1.34]	1.24 [1.19-1.30]	1.36 [1.23-1.50]	1.25 [1.20-1.30]	8,057	7,573	5,375	1,254	6,659
555.21	1.62 [1.52-1.73]	1.60 [1.50-1.71]	1.57 [1.45-1.69]	1.51 [1.30-1.77]	1.44 [1.35-1.54]	1.39 [1.30-1.49]	1.30 [1.22-1.39]	1.27 [1.17-1.38]	1.43 [1.21-1.68]	1.29 [1.21-1.38]	2,605	2,415	1,624	490	2,356
556	1.49 [1.37-1.62]	1.50 [1.38-1.64]	1.45 [1.32-1.59]	1.52 [1.16-1.98]	1.36 [1.25-1.48]	1.38 [1.26-1.50]	1.35 [1.23-1.47]	1.31 [1.19-1.45]	1.44 [1.09-1.90]	1.31 [1.20-1.43]	1,455	1,391	1,105	173	1,293
556.1	1.54 [1.45-1.63]	1.53 [1.44-1.63]	1.46 [1.37-1.57]	1.60 [1.36-1.88]	1.40 [1.32-1.49]	1.37 [1.29-1.46]	1.30 [1.22-1.39]	1.28 [1.19-1.37]	1.48 [1.25-1.74]	1.30 [1.22-1.38]	2,973	2,814	2,066	458	2,642
557	1.78 [1.72-1.85]	1.60 [1.53-1.67]	1.61 [1.53-1.70]	2.04 [1.93-2.17]	1.67 [1.61-1.73]	1.56 [1.50-1.62]	1.36 [1.30-1.42]	1.37 [1.30-1.44]	1.76 [1.65-1.87]	1.49 [1.43-1.55]	8,443	5,443	3,833	3,599	7,356
557.1	1.57 [1.50-1.64]	1.59 [1.52-1.67]	1.62 [1.53-1.71]	1.38 [1.26-1.50]	1.45 [1.38-1.52]	1.41 [1.35-1.47]	1.38 [1.31-1.45]	1.40 [1.32-1.48]	1.30 [1.18-1.42]	1.33 [1.27-1.40]	5,403	4,409	3,104	1,479	4,508
558	1.35 [1.33-1.37]	1.39 [1.37-1.41]	1.37 [1.34-1.39]	1.21 [1.16-1.25]	1.25 [1.22-1.27]	1.26 [1.24-1.29]	1.24 [1.22-1.27]	1.23 [1.21-1.25]	1.17 [1.13-1.22]	1.19 [1.17-1.21]	37,902	32,476	26,597	7,035	31,731
559	1.29 [1.23-1.35]	1.29 [1.23-1.36]	1.22 [1.16-1.29]	1.43 [1.17-1.74]	1.21 [1.16-1.28]	1.21 [1.15-1.27]	1.17 [1.11-1.23]	1.13 [1.07-1.19]	1.32 [1.07-1.62]	1.16 [1.11-1.23]	4,140	4,029	3,460	279	3,698
560.1	1.18 [1.11-1.25]	1.20 [1.13-1.27]	1.20 [1.12-1.28]	0.87 [0.70-1.08]	1.14 [1.07-1.21]	1.12 [1.06-1.19]	1.11 [1.04-1.18]	1.12 [1.05-1.19]	0.84 [0.67-1.05]	1.11 [1.04-1.18]	2,870	2,729	2,425	226	2,599
560.2	1.23 [1.16-1.31]	1.26 [1.18-1.35]	1.28 [1.19-1.37]	1.11 [0.96-1.28]	1.16 [1.09-1.23]	1.17 [1.10-1.24]	1.13 [1.06-1.21]	1.16 [1.08-1.24]	1.07 [0.92-1.24]	1.11 [1.04-1.18]	2,820	2,405	2,190	498	2,602
560.3	1.19 [1.12-1.26]	1.19 [1.12-1.27]	1.17 [1.10-1.24]	0.90 [0.69-1.17]	1.09 [1.03-1.16]	1.11 [1.05-1.18]	1.08 [1.02-1.15]	1.07 [1.01-1.14]	0.85 [0.65-1.11]	1.06 [1.00-1.12]	2,843	2,772	2,543	141	2,633
560.4	1.21 [1.18-1.25]	1.21 [1.18-1.25]	1.19 [1.16-1.23]	1.16 [1.03-1.31]	1.14 [1.11-1.17]	1.15 [1.12-1.19]	1.12 [1.09-1.15]	1.10 [1.07-1.14]	1.11 [0.98-1.26]	1.10 [1.07-1.14]	12,494	12,100	11,131	705	11,306
561	1.32 [1.30-1.34]	1.33 [1.31-1.35]	1.32 [1.29-1.34]	1.22 [1.15-1.30]	1.23 [1.21-1.25]	1.23 [1.21-1.25]	1.20 [1.18-1.22]	1.19 [1.17-1.21]	1.16 [1.08-1.23]	1.17 [1.15-1.19]	40,513	39,486	34,220	2,734	35,935
561.1	1.62 [1.51-1.74]	1.58 [1.46-1.70]	1.56 [1.44-1.69]	1.75 [1.42-2.14]	1.51 [1.40-1.62]	1.50 [1.39-1.61]	1.40 [1.29-1.51]	1.40 [1.29-1.52]	1.53 [1.24-1.89]	1.42 [1.31-1.53]	2,038	1,820	1,646	276	1,878
561.2	1.31 [1.27-1.36]	1.32 [1.27-1.37]	1.30 [1.25-1.36]	1.16 [1.04-1.30]	1.22 [1.17-1.26]	1.20 [1.16-1.25]	1.15 [1.11-1.20]	1.15 [1.10-1.20]	1.11 [0.99-1.24]	1.14 [1.10-1.19]	7,064	6,608	5,225	903	6,356
562.1	1.25 [1.24-1.27]	1.25 [1.23-1.26]	1.25 [1.23-1.26]	1.15 [0.93-1.43]	1.17 [1.15-1.18]	1.17 [1.16-1.19]	1.14 [1.12-1.15]	1.14 [1.12-1.15]	1.06 [0.84-1.32]	1.11 [1.10-1.13]	72,988	72,950	71,810	235	63,834
563	1.27 [1.26-1.29]	1.29 [1.27-1.30]	1.27 [1.25-1.29]	1.20 [1.16-1.23]	1.20 [1.18-1.21]	1.20 [1.18-1.21]	1.17 [1.15-1.18]	1.16 [1.14-1.18]	1.15 [1.12-1.19]	1.15 [1.14-1.17]	73,619	64,164	56,852	12,054	66,813
564	1.27 [1.26-1.29]	1.29 [1.27-1.30]	1.27 [1.26-1.29]	1.19 [1.16-1.23]	1.20 [1.18-1.21]	1.20 [1.18-1.21]	1.17 [1.15-1.18]	1.16 [1.14-1.18]	1.15 [1.11-1.18]	1.15 [1.14-1.17]	75,465	65,892	58,307	12,257	68,411
564.1	1.50 [1.47-1.53]	1.52 [1.49-1.55]	1.53 [1.49-1.56]	1.29 [1.21-1.37]	1.39 [1.36-1.42]	1.27 [1.24-1.30]	1.19 [1.17-1.22]	1.20 [1.17-1.23]	1.21 [1.14-1.29]	1.22 [1.19-1.25]	24,237	23,632	16,259	3,011	21,882
564.8	1.28 [1.23-1.32]	1.27 [1.23-1.32]	1.26 [1.21-1.31]	1.32 [1.11-1.58]	1.20 [1.15-1.24]	1.21 [1.16-1.25]	1.17 [1.12-1.21]	1.15 [1.11-1.20]	1.26 [1.05-1.51]	1.16 [1.11-1.20]	7,607	7,483	6,803	353	6,879
564.9	1.31 [1.29-1.32]	1.32 [1.30-1.34]	1.30 [1.29-1.32]	1.17 [1.12-1.23]	1.20 [1.18-1.21]	1.20 [1.18-1.21]	1.16 [1.15-1.18]	1.16 [1.14-1.17]	1.09 [1.03-1.14]	1.14 [1.12-1.15]	57,424	54,908	47,972	4,667	53,093
565	1.40 [1.37-1.43]	1.41 [1.38-1.44]	1.38 [1.35-1.42]	1.30 [1.22-1.38]	1.29 [1.26-1.32]	1.30 [1.28-1.33]	1.27 [1.24-1.30]	1.25 [1.22-1.28]	1.25 [1.18-1.33]	1.23 [1.20-1.26]	21,100	19,711	14,491	2,957	17,782
565.1	1.24 [1.21-1.27]	1.24 [1.21-1.28]	1.23 [1.20-1.26]	1.35 [1.13-1.61]	1.15 [1.12-1.18]	1.17 [1.14-1.20]	1.13 [1.10-1.16]	1.12 [1.09-1.15]	1.29 [1.07-1.55]	1.11 [1.08-1.14]	15,436	15,327	14,143	342	13,376
567	1.19 [1.14-1.25]	1.18 [1.13-1.24]	1.17 [1.11-1.23]	1.04 [0.87-1.25]	1.13 [1.07-1.19]	1.16 [1.10-1.21]	1.10 [1.05-1.16]	1.10 [1.04-1.16]	1.05 [0.87-1.26]	1.12 [1.06-1.18]	4,231	4,044	3,566	335	3,710
568	1.19 [1.16-1.22]	1.20 [1.17-1.23]	1.18 [1.14-1.21]	1.11 [1.02-1.21]	1.11 [1.08-1.14]	1.12 [1.09-1.15]	1.09 [1.06-1.12]	1.07 [1.04-1.11]	1.07 [0.99-1.17]	1.08 [1.05-1.11]	14,367	13,909	10,436	1,499	12,929
568.1	1.20 [1.17-1.23]	1.20 [1.17-1.24]	1.18 [1.15-1.22]	1.12 [1.03-1.22]	1.12 [1.09-1.15]	1.12 [1.09-1.15]	1.09 [1.06-1.12]	1.08 [1.04-1.11]	1.08 [0.99-1.19]	1.08 [1.05-1.11]	13,670	13,248	9,849	1,391	12,346
569	1.28 [1.26-1.30]	1.28 [1.26-1.30]	1.26 [1.24-1.28]	1.38 [1.27-1.50]	1.19 [1.17-1.21]	1.19 [1.17-1.21]	1.15 [1.13-1.17]	1.14 [1.12-1.15]	1.28 [1.17-1.39]	1.14 [1.12-1.16]	40,631	39,999	36,652	1,586	36,108
569.1	1.24 [1.14-1.35]	1.26 [1.15-1.37]	1.24 [1.13-1.35]		1.19 [1.09-1.30]	1.17 [1.07-1.27]	1.14 [1.05-1.25]	1.13 [1.03-1.24]		1.15 [1.06-1.26]	1,433	1,394	1,310	NA	1,340
569.2	1.24 [1.16-1.34]	1.25 [1.16-1.35]	1.22 [1.12-1.32]	1.16 [0.92-1.47]	1.17 [1.08-1.26]	1.16 [1.08-1.25]	1.13 [1.04-1.22]	1.11 [1.02-1.20]	1.08 [0.85-1.38]	1.12 [1.03-1.20]	1,834	1,732	1,472	184	1,640
571	1.48 [1.41-1.55]	1.51 [1.44-1.58]	1.50 [1.43-1.58]		1.39 [1.32-1.46]	1.37 [1.30-1.44]	1.33 [1.26-1.40]	1.31 [1.25-1.38]		1.34 [1.27-1.41]	4,213	4,197	4,021	NA	3,916

Supplementary Table 6: phecode-mapped results (hazard ratios and events from all cohorts)

	Hazard ratio (99% confidence interval)										Events (in exposed)				
	crude					adjusted									
Outcome	any age	18+	40+	<18	hosp.	any age	18+	40+	<18	hosp.	any age	18+	40+	<18	hosp.
571.5	1.45 [1.41-1.49]	1.45 [1.41-1.50]	1.46 [1.42-1.50]	1.19 [1.04-1.36]	1.34 [1.30-1.38]	1.29 [1.25-1.33]	1.21 [1.17-1.25]	1.20 [1.17-1.24]	1.14 [0.99-1.31]	1.23 [1.20-1.27]	12,671	12,461	10,860	655	11,737
571.51	1.47 [1.40-1.54]	1.50 [1.43-1.57]	1.50 [1.43-1.57]		1.38 [1.32-1.45]	1.36 [1.30-1.43]	1.32 [1.26-1.39]	1.31 [1.24-1.37]		1.33 [1.27-1.40]	4,611	4,572	4,331	NA	4,277
571.6	1.44 [1.28-1.62]	1.41 [1.25-1.59]	1.46 [1.29-1.65]		1.34 [1.18-1.51]	1.31 [1.16-1.48]	1.25 [1.10-1.41]	1.28 [1.13-1.45]		1.26 [1.11-1.43]	697	687	654	NA	630
571.8	1.34 [1.28-1.40]	1.35 [1.29-1.41]	1.37 [1.31-1.44]	0.85 [0.66-1.09]	1.29 [1.23-1.35]	1.27 [1.21-1.33]	1.22 [1.17-1.28]	1.24 [1.18-1.30]	0.85 [0.65-1.09]	1.27 [1.21-1.33]	5,162	5,076	4,753	152	4,657
571.81	1.46 [1.38-1.54]	1.47 [1.39-1.55]	1.46 [1.38-1.54]		1.37 [1.29-1.44]	1.36 [1.29-1.44]	1.30 [1.23-1.37]	1.28 [1.21-1.35]		1.33 [1.26-1.41]	3,513	3,470	3,245	NA	3,220
572	1.20 [1.17-1.24]	1.22 [1.18-1.26]	1.22 [1.18-1.26]	0.94 [0.81-1.10]	1.17 [1.13-1.21]	1.17 [1.14-1.21]	1.15 [1.11-1.19]	1.15 [1.11-1.19]	0.93 [0.80-1.09]	1.17 [1.13-1.21]	9,491	9,236	8,539	441	8,376
573	1.33 [1.30-1.36]	1.33 [1.31-1.36]	1.33 [1.30-1.36]	1.11 [1.00-1.23]	1.25 [1.22-1.27]	1.23 [1.20-1.25]	1.17 [1.14-1.19]	1.16 [1.14-1.19]	1.06 [0.95-1.19]	1.18 [1.16-1.21]	22,960	22,590	20,335	975	20,969
573.1	1.28 [1.08-1.52]	1.34 [1.13-1.59]	1.46 [1.22-1.74]		1.29 [1.09-1.54]	1.24 [1.04-1.48]	1.24 [1.04-1.48]	1.35 [1.12-1.62]		1.28 [1.07-1.53]	360	355	347	NA	347
573.2	1.57 [1.31-1.89]	1.68 [1.38-2.04]	1.78 [1.43-2.20]		1.39 [1.16-1.68]	1.43 [1.18-1.72]	1.46 [1.19-1.79]	1.56 [1.25-1.95]		1.32 [1.09-1.61]	299	259	213	NA	275
573.3	1.38 [1.32-1.45]	1.44 [1.38-1.51]	1.41 [1.34-1.49]	1.11 [0.99-1.24]	1.27 [1.21-1.33]	1.30 [1.24-1.36]	1.28 [1.22-1.35]	1.27 [1.20-1.34]	1.08 [0.96-1.21]	1.23 [1.17-1.29]	5,156	4,624	3,839	829	4,592
573.5	1.22 [1.16-1.28]	1.24 [1.18-1.31]	1.23 [1.17-1.30]	1.12 [0.94-1.33]	1.19 [1.12-1.25]	1.19 [1.13-1.25]	1.18 [1.12-1.24]	1.17 [1.11-1.24]	1.13 [0.95-1.34]	1.18 [1.12-1.25]	3,810	3,566	3,200	362	3,246
573.7	1.26 [1.23-1.29]	1.26 [1.23-1.29]	1.26 [1.23-1.29]	1.12 [1.03-1.22]	1.19 [1.16-1.21]	1.21 [1.18-1.23]	1.17 [1.14-1.19]	1.17 [1.14-1.19]	1.09 [1.00-1.19]	1.16 [1.14-1.19]	20,802	20,176	17,720	1,533	18,675
573.9	1.21 [1.14-1.30]	1.17 [1.09-1.26]	1.21 [1.12-1.30]	1.24 [1.05-1.46]	1.15 [1.08-1.23]	1.15 [1.07-1.23]	1.06 [0.98-1.14]	1.10 [1.02-1.19]	1.14 [0.96-1.36]	1.12 [1.04-1.20]	2,353	2,104	1,804	390	2,149
574.1	1.16 [1.14-1.18]	1.17 [1.15-1.19]	1.15 [1.13-1.17]	1.05 [0.97-1.12]	1.10 [1.08-1.12]	1.10 [1.09-1.12]	1.08 [1.06-1.09]	1.07 [1.05-1.08]	1.02 [0.95-1.10]	1.06 [1.04-1.08]	39,352	39,031	32,181	1,957	34,401
574.11	1.14 [1.09-1.19]	1.15 [1.11-1.20]	1.13 [1.08-1.19]	1.11 [0.90-1.38]	1.06 [1.01-1.11]	1.09 [1.04-1.14]	1.07 [1.02-1.11]	1.05 [1.00-1.10]	1.12 [0.89-1.39]	1.03 [0.98-1.08]	5,154	5,128	4,363	220	4,513
574.12	1.15 [1.12-1.18]	1.16 [1.13-1.19]	1.14 [1.10-1.17]	1.07 [0.96-1.19]	1.09 [1.06-1.12]	1.09 [1.06-1.12]	1.07 [1.04-1.10]	1.05 [1.02-1.08]	1.04 [0.93-1.16]	1.05 [1.02-1.08]	13,627	13,549	10,395	881	11,886
574.2	1.12 [1.08-1.16]	1.13 [1.09-1.17]	1.11 [1.07-1.15]	1.00 [0.86-1.17]	1.07 [1.03-1.10]	1.07 [1.03-1.11]	1.04 [1.01-1.08]	1.03 [0.99-1.07]	0.98 [0.84-1.15]	1.04 [1.00-1.08]	8,555	8,498	6,989	445	7,591
574.3	1.19 [1.15-1.23]	1.19 [1.15-1.24]	1.19 [1.14-1.24]	1.06 [0.89-1.26]	1.13 [1.09-1.18]	1.12 [1.08-1.17]	1.09 [1.05-1.13]	1.09 [1.04-1.13]	1.03 [0.86-1.24]	1.10 [1.05-1.14]	7,288	7,250	6,041	326	6,430
575	1.18 [1.13-1.23]	1.19 [1.14-1.24]	1.18 [1.13-1.24]	0.99 [0.84-1.17]	1.11 [1.06-1.16]	1.11 [1.07-1.16]	1.09 [1.04-1.14]	1.08 [1.03-1.14]	0.96 [0.81-1.15]	1.07 [1.02-1.12]	5,528	5,479	4,323	344	4,861
575.1	1.19 [1.13-1.26]	1.21 [1.15-1.28]	1.18 [1.12-1.25]		1.14 [1.08-1.21]	1.15 [1.08-1.21]	1.12 [1.06-1.19]	1.10 [1.04-1.17]		1.12 [1.06-1.18]	3,424	3,395	3,235	NA	3,222
575.2	1.12 [1.06-1.17]	1.12 [1.06-1.17]	1.12 [1.06-1.18]	0.99 [0.78-1.25]	1.09 [1.04-1.15]	1.10 [1.05-1.16]	1.07 [1.02-1.13]	1.07 [1.01-1.13]	0.98 [0.77-1.25]	1.09 [1.03-1.15]	3,876	3,819	3,208	174	3,365
575.6	1.12 [1.02-1.23]	1.12 [1.02-1.23]	1.11 [0.99-1.24]		1.07 [0.97-1.17]	1.04 [0.94-1.14]	1.01 [0.92-1.11]	1.00 [0.89-1.12]		1.00 [0.91-1.11]	1,095	1,093	688	NA	958
575.7	1.19 [1.14-1.25]	1.20 [1.15-1.26]	1.19 [1.14-1.26]	1.04 [0.86-1.27]	1.11 [1.06-1.17]	1.13 [1.08-1.18]	1.11 [1.06-1.16]	1.10 [1.04-1.16]	1.01 [0.82-1.24]	1.08 [1.03-1.14]	4,563	4,517	3,721	260	4,022
575.8	1.16 [1.12-1.19]	1.17 [1.13-1.20]	1.16 [1.12-1.20]	1.03 [0.88-1.21]	1.12 [1.09-1.16]	1.11 [1.08-1.15]	1.08 [1.05-1.12]	1.08 [1.04-1.11]	1.00 [0.85-1.17]	1.10 [1.06-1.14]	10,320	10,204	9,066	394	9,357
575.9	1.14 [1.05-1.23]	1.12 [1.03-1.21]	1.14 [1.05-1.23]		1.09 [1.00-1.18]	1.11 [1.02-1.20]	1.04 [0.96-1.13]	1.07 [0.98-1.16]		1.08 [0.99-1.17]	1,554	1,523	1,424	NA	1,382
577	1.23 [1.16-1.30]	1.25 [1.18-1.32]	1.26 [1.19-1.33]	0.85 [0.67-1.09]	1.16 [1.09-1.22]	1.16 [1.09-1.22]	1.12 [1.05-1.18]	1.13 [1.07-1.20]	0.84 [0.66-1.08]	1.12 [1.06-1.19]	3,389	3,298	3,114	158	3,154
577.1	1.20 [1.16-1.25]	1.21 [1.17-1.26]	1.19 [1.14-1.24]	1.03 [0.89-1.18]	1.13 [1.09-1.18]	1.14 [1.10-1.18]	1.11 [1.07-1.15]	1.08 [1.04-1.13]	1.02 [0.88-1.18]	1.10 [1.06-1.15]	7,005	6,863	5,652	497	6,079
577.2	1.30 [1.22-1.39]	1.35 [1.26-1.44]	1.31 [1.21-1.41]		1.20 [1.12-1.29]	1.22 [1.13-1.30]	1.17 [1.09-1.26]	1.13 [1.05-1.22]		1.18 [1.10-1.26]	2,132	2,104	1,822	NA	1,943
577.3	1.16 [1.07-1.25]	1.16 [1.08-1.26]	1.15 [1.06-1.24]		1.06 [0.98-1.15]	1.10 [1.02-1.19]	1.08 [0.99-1.16]	1.06 [0.97-1.15]		1.04 [0.96-1.13]	1,616	1,602	1,492	NA	1,478
578.1	1.20 [1.17-1.24]	1.23 [1.20-1.27]	1.21 [1.17-1.25]	1.10 [1.01-1.21]	1.12 [1.08-1.15]	1.14 [1.11-1.18]	1.12 [1.09-1.16]	1.11 [1.08-1.15]	1.07 [0.97-1.17]	1.09 [1.06-1.13]	11,643	10,993	9,429	1,234	10,327
578.2	1.25 [1.22-1.29]	1.25 [1.22-1.29]	1.25 [1.22-1.29]	1.15 [1.02-1.29]	1.17 [1.14-1.21]	1.20 [1.16-1.23]	1.15 [1.12-1.19]	1.15 [1.11-1.19]	1.07 [0.95-1.21]	1.15 [1.11-1.18]	11,586	11,080	10,263	766	10,329
578.8	1.39 [1.36-1.43]	1.39 [1.36-1.43]	1.37 [1.34-1.41]	1.34 [1.23-1.47]	1.26 [1.23-1.30]	1.30 [1.26-1.33]	1.25 [1.22-1.29]	1.24 [1.21-1.28]	1.26 [1.15-1.38]	1.20 [1.17-1.24]	16,108	15,501	12,518	1,407	13,033
578.9	1.32 [1.30-1.34]	1.33 [1.31-1.35]	1.30 [1.28-1.33]	1.24 [1.17-1.32]	1.23 [1.21-1.25]	1.23 [1.21-1.25]	1.19 [1.17-1.22]	1.17 [1.15-1.20]	1.17 [1.10-1.25]	1.18 [1.16-1.20]	32,769	31,805	26,516	2,905	29,328
579	1.11 [1.05-1.16]	1.11 [1.06-1.17]	1.09 [1.03-1.15]	1.02 [0.86-1.22]	1.07 [1.01-1.13]	1.09 [1.04-1.15]	1.06 [1.01-1.12]	1.05 [0.99-1.11]	1.02 [0.86-1.23]	1.07 [1.01-1.13]	3,647	3,445	3,048	318	3,157
579.2	1.38 [1.32-1.45]	1.44 [1.38-1.51]	1.41 [1.34-1.49]	1.11 [0.99-1.24]	1.27 [1.21-1.33]	1.30 [1.24-1.36]	1.28 [1.22-1.35]	1.27 [1.20-1.34]	1.08 [0.96-1.21]	1.23 [1.17-1.29]	5,156	4,624	3,839	829	4,592
579.8	1.32 [1.27-1.37]	1.35 [1.31-1.41]	1.32 [1.26-1.37]	1.18 [1.08-1.29]	1.25 [1.20-1.30]	1.22 [1.18-1.27]	1.20 [1.16-1.25]	1.18 [1.13-1.23]	1.13 [1.02-1.24]	1.19 [1.14-1.23]	7,913	7,159	5,915	1,249	7,191
genitourinary															
580	1.34 [1.23-1.47]	1.48 [1.33-1.63]	1.46 [1.30-1.62]	1.01 [0.85-1.21]	1.23 [1.12-1.36]	1.29 [1.17-1.41]	1.35 [1.22-1.50]	1.33 [1.19-1.49]	1.01 [0.84-1.21]	1.20 [1.09-1.33]	1,254	973	825	316	1,065
580.11	1.38 [1.13-1.69]	1.38 [1.10-1.72]	1.39 [1.10-1.76]		1.24 [1.00-1.54]	1.35 [1.10-1.66]	1.29 [1.03-1.62]	1.33 [1.04-1.68]		1.24 [0.99-1.54]	242	206	176	NA	212
580.12	1.54 [1.35-1.75]	1.56 [1.36-1.78]	1.47 [1.27-1.70]		1.36 [1.19-1.56]	1.45 [1.27-1.66]	1.44 [1.25-1.66]	1.34 [1.15-1.56]		1.31 [1.14-1.51]	595	551	470	NA	522
580.14	1.34 [1.28-1.39]	1.34 [1.29-1.40]	1.35 [1.29-1.41]		1.26 [1.21-1.32]	1.27 [1.22-1.32]	1.22 [1.17-1.27]	1.22 [1.17-1.28]		1.22 [1.17-1.28]	6,043	6,015	5,937	NA	5,629
580.2	1.42 [1.35-1.49]	1.47 [1.39-1.55]	1.45 [1.37-1.53]	1.02 [0.86-1.21]	1.28 [1.21-1.35]	1.34 [1.27-1.41]	1.29 [1.22-1.36]	1.26 [1.19-1.33]	1.02 [0.86-1.21]	1.26 [1.19-1.33]	3,904	3,623	3,315	349	3,595
580.31	1.43 [1.35-1.52]	1.45 [1.36-1.53]	1.43 [1.35-1.52]		1.28 [1.21-1.36]	1.35 [1.27-1.43]	1.26 [1.18-1.33]	1.22 [1.15-1.31]		1.26 [1.19-1.34]	2,958	2,908	2,705	NA	2,814
580.32	1.47 [1.36-1.59]	1.48 [1.36-1.62]	1.48 [1.35-1.63]	1.31 [1.10-1.56]	1.34 [1.23-1.46]	1.40 [1.29-1.53]	1.35 [1.23-1.48]	1.34 [1.22-1.48]	1.26 [1.05-1.51]	1.30 [1.20-1.42]	1,569	1,272	1,077	350	1,408
585.1	1.23 [1.21-1.24]	1.22 [1.21-1.23]	1.23 [1.21-1.24]	1.07 [0.98-1.17]	1.17 [1.16-1.19]	1.19 [1.17-1.20]	1.13 [1.12-1.15]	1.14 [1.12-1.15]	1.07 [0.97-1.17]	1.16 [1.15-1.18]	76,985	76,432	74,248	1,369	71,764
585.2	1.30 [1.26-1.34]	1.31 [1.27-1.36]	1.29 [1.25-1.34]	1.08 [0.91-1.29]	1.23 [1.19-1.27]	1.24 [1.20-1.28]	1.21 [1.17-1.25]	1.19 [1.15-1.23]	1.05 [0.87-1.26]	1.20 [1.16-1.24]	9,785	9,554	9,235	316	8,723
585.3	1.26 [1.24-1.28]	1.26 [1.24-1.28]	1.25 [1.24-1.27]	1.14 [0.97-1.34]	1.19 [1.17-1.20]	1.21 [1.19-1.22]	1.15 [1.14-1.17]	1.15 [1.13-1.16]	1.12 [0.95-1.33]	1.16 [1.15-1.18]	65,241	65,046	64,301	393	61,464
585.31	1.42 [1.34-1.50]	1.42 [1.34-1.50]	1.39 [1.31-1.48]	1.11 [0.85-1.45]	1.24 [1.17-1.31]	1.38 [1.30-1.46]	1.30 [1.22-1.38]	1.26 [1.18-1.34]	1.11 [0.84-1.46]	1.24 [1.17-1.32]	3,115	3,038	2,793	143	2,877
586	1.16 [1.11-1.21]	1.18 [1.13-1.23]	1.17 [1.12-1.22]	1.03 [0.91-1.16]	1.08 [1.03-1.12]	1.11 [1.06-1.16]	1.08 [1.04-1.13]	1.07 [1.02-1.12]	1.02 [0.90-1.15]	1.06 [1.01-1.10]	5,806	5,314	4,760	665	5,263
586.11	1.23 [1.14-1.33]	1.23 [1.14-1.33]	1.24 [1.14-1.35]	0.98 [0.78-1.22]	1.11 [1.03-1.20]	1.19 [1.10-1.28]	1.13 [1.04-1.23]	1.13 [1.04-1.24]	0.96 [0.76-1.21]	1.10 [1.02-1.20]	1,692	1,553	1,408	194	1,574
586.12	1.08 [0.93-1.24]	1.17 [0.89-1.52]		1.04 [0.88-1.22]	0.98 [0.85-1.14]	1.04 [0.90-1.20]	1.06 [0.81-1.40]		1.01 [0.85-1.19]	0.97 [0.83-1.12]	472	133	NA	357	438
586.2	1.19 [1.16-1.23]	1.19 [1.15-1.23]	1.20 [1.16-1.24]	0.88 [0.70-1.11]	1.13 [1.09-1.17]	1.14 [1.11-1.18]	1.10 [1.06-1.14]	1.11 [1.07-1.14]	0.86 [0.68-1.10]	1.11 [1.07-1.15]	9,727	9,633	9,308	183	8,957
586.3	1.25 [1.10-1.43]	1.25 [1.09-1.42]	1.21 [1.06-1.39]		1.13 [0.98-1.29]	1.22 [1.07-1.40]	1.14 [0.99-1.31]	1.11 [0.97-1.28]		1.12 [0.97-1.29]	563	554	529	NA	511
586.4	1.07 [1.01-1.13]	1.08 [1.02-1.14]	1.07 [1.01-1.13]	0.99 [0.84-1.16]	1.01 [0.95-1.07]	1.05 [1.00-1.11]	1.04 [0.98-1.10]	1.04 [0.98-1.10]	0.98 [0.83-1.15]	1.02 [0.96-1.08]	3,404	3,160	2,732	390	3,021
587	1.39 [1.26-1.54]	1.45 [1.31-1.61]	1.40 [1.24-1.57]	1.07 [0.80-1.43]	1.15 [1.04-1.27]	1.36 [1.23-1.51]	1.35 [1.22-1.51]	1.26 [1.12-1.42]	1.04 [0.77-1.40]	1.18 [1.07-1.31]	980	905	712	117	909
588	1.36 [1.20-1.53]	1.42 [1.25-1.61]	1.47 [1.29-1.68]		1.19 [1.05-1.35]	1.30 [1.15-1.48]	1.31 [1.15-1.49]	1.34 [1.17-1.54]		1.18 [1.04-1.34]	667	632	584	NA	618
589	1.25 [1.13-1.38]	1.22 [1.10-1.35]	1.20 [1.08-1.34]	1.09 [0.80-1.48]	1.13 [1.02-1.25]	1.20 [1.08-1.33]	1.12 [1.01-1.25]	1.08 [0.97-1.21]	1.12 [0.82-1.54]	1.11 [1.00-1.23]	1,044	956	864	118	953
590	1.17 [1.13-1.20]	1.19 [1.15-1.23]	1.23 [1.18-1.28]	1.05 [0.99-1.12]	1.08 [1.04-1.12]	1.10 [1.07-1.14]	1.07 [1.03-1.11]	1.11 [1.06-1.16]	1.03 [0.96-1.10]	1.05 [1.02-1.09]	9,587	8,432	5,143	2,655	8,346
591	1.19 [1.17-1.20]	1.19 [1.17-1.20]	1.19 [1.17-1.20]	1.11 [1.07-1.15]	1.13 [1.11-1.14]	1.15 [1.13-1.16]	1.11 [1.09-1.12]	1.11 [1.10-1.13]	1.08 [1.04-1.12]	1.11 [1.09-1.12]	90,056	84,342	77,587	8,358	80,678
592.1	1.27 [1.22-1.32]	1.27 [1.22-1.31]	1.23 [1.18-1.28]	1.40 [1.21-1.62]	1.16 [1.12-1.21]	1.20 [1.16-1.25]	1.17 [1.12-1.21]	1.14 [1.09-1.18]	1.33 [1.15-1.55]	1.13 [1.09-1.17]	7,370	7,189	6,222	517	6,559
592.11	1.28 [1.06-1.53]	1.26 [1.05-1.52]	1.26 [1.04-1.52]		1.17 [0.97-1.41]	1.25 [1.04-1.50]	1.17 [0.97-1.42]	1.20 [0.99-1.46]		1.17 [0.96-1.42]	293	284	260	NA	266
592.12	1.31 [1.23-1.40]	1.30 [1.22-1.39]	1.29 [1.20-1.38]		1.19 [1.11-1.28]	1.24 [1.16-1.33]	1.20 [1.12-1.29]	1.19 [1.10-1.28]		1.16 [1.08-1.24]	2,115	2,088	1,855	NA	1,906
592.13	1.45 [1.28-1.64]	1.43 [1.26-1.62]	1.31 [1.12-1.53]		1.30 [1.14-1.48]	1.31 [1.15-1.48]	1.24 [1.09-1.41]	1.15 [0.98-1.35]		1.20 [1.05-1.37]	657	639	393	NA	594
592.2	1.35 [1.16-1.58]	1.34 [1.13-1.59]	1.25 [1.03-1.52]		1.12 [0.94-1.33]	1.30 [1.11-1.53]	1.25 [1.05-1.49]	1.19 [0.97-1.45]		1.09 [0.91-1.29]	404	334	251	NA	327
593	1.20 [1.18-1.22]	1.20 [1.18-1.22]	1.19 [1.17-1.21]	1.12 [1.05-1.20]	1.12 [1.10-1.14]	1.15 [1.13-1.17]	1.12 [1.10-1.14]	1.12 [1.10-1.14]	1.08 [1.01-1.15]	1.09 [1.07-1.12]	32,057	30,628	27,600	2,331	27,062
594	1.15 [1.11-1.18]	1.15 [1.12-1.19]	1.15 [1.11-1.19]	0.94 [0.83-1.05]	1.07 [1.04-1.10]	1.10 [1.07-1.13]	1.07 [1.04-1.10]	1.07 [1.03-1.10]	0.92 [0.81-1.03]	1.05 [1.02-1.08]	10,973	10,813	8,693	738	9,287
594.1	1.16 [1.12-1.20]	1.17 [1.13-1.21]	1.16 [1.12-1.21]	0.97 [0.84-1.11]	1.07 [1.03-1.11]	1.11 [1.07-1.15]	1.07 [1.04-1.11]	1.07 [1.03-1.11]	0.94 [0.81-1.08]	1.05 [1.01-1.08]	8,334	8,207	6,743	513	7,256
594.2	1.02 [0.96-1.08]	1.01 [0.95-1.08]	1.03 [0.96-1.10]		0.95 [0.89-1.02]	1.00 [0.94-1.07]	0.98 [0.92-1.05]	1.00 [0.94-1.07]		0.96 [0.90-1.02]	2,439	2,414	2,229	NA	2,124
594.3	1.11 [1.06-1.16]	1.11 [1.06-1.16]	1.10 [1.05-1.17]	0.84 [0.70-1.02]	1.02 [0.97-1.07]	1.06 [1.01-1.11]	1.03 [0.98-1.09]	1.02 [0.97-1.08]	0.82 [0.67-1.00]	1.00 [0.95-1.06]	3,964	3,923	3,048	261	3,273
594.8	1.17 [1.11-1.25]	1.19 [1.12-1.26]	1.14 [1.05-1.23]	1.03 [0.87-1.22]	1.05 [0.98-1.12]	1.08 [1.02-1.15]	1.06 [1.00-1.13]	1.01 [0.94-1.09]	0.98 [0.83-1.18]	1.01 [0.94-1.08]	2,585	2,517	1,569	334	2,068
595	1.05 [1.02-1.08]	1.06 [1.02-1.09]	1.06 [1.02-1.09]	1.03 [0.92-1.15]	1.00 [0.97-1.04]	1.04 [1.01-1.07]	1.02 [0.99-1.06]	1.03 [1.00-1.07]	1.00 [0.90-1.12]	1.01 [0.98-1.05]	10,444	10,029	8,820	863	9,209
596	1.23 [1.20-1.26]	1.22 [1.20-1.25]	1.22 [1.19-1.25]	1.15 [1.05-1.27]	1.14 [1.12-1.17]	1.17 [1.14-1.19]	1.13 [1.11-1.16]	1.14 [1.11-1.16]	1.10 [0.99-1.21]	1.11 [1.09-1.14]	21,205	20,503	18,963	1,124	18,799
596.1	1.21 [1.13-1.29]	1.20 [1.13-1.28]	1.22 [1.14-1.30]		1.13 [1.06-1.21]	1.15 [1.08-1.23]	1.12 [1.05-1.19]	1.13 [1.06-1.21]		1.10 [1.02-1.17]	2,419	2,403	2,326	NA	2,096
596.5	1.35 [1.26-1.43]	1.39 [1.30-1.48]	1.40 [1.30-1.51]	0.98 [0.82-1.16]	1.19 [1.12-1.27]	1.24 [1.16-1.32]	1.20 [1.12-1.28]	1.22 [1.13-1.31]	0.95 [0.79-1.13]	1.13 [1.06-1.21]	2,392	2,148	1,849	319	2,190
597	1.23 [1.15-1.32]	1.29 [1.20-1.38]	1.23 [1.14-1.32]	1.04 [0.86-1.25]	1.15 [1.07-1.23]	1.18 [1.10-1.26]	1.21 [1.12-1.29]	1.15 [1.07-1.24]	1.00 [0.83-1.22]	1.12 [1.04-1.20]	2,182	1,971	1,700	293	1,908
597.1	1.31 [1.26-1.36]	1.30 [1.26-1.35]	1.30 [1.25-1.35]	1.30 [1.17-1.45]	1.20 [1.15-1.25]	1.24 [1.20-1.29]	1.21 [1.16-1.25]	1.20 [1.16-1.25]	1.26 [1.13-1.41]	1.17 [1.12-1.21]	7,751	7,187	6,246	879	6,658
597.2	0.99 [0.83-1.18]	1.02 [0.86-1.21]	1.02 [0.85-1.22]		0.96 [0.81-1.15]	0.97 [0.81-1.16]	0.97 [0.82-1.16]	0.98 [0.81-1.17]		0.97 [0.81-1.16]	321	310	281	NA	303
598	1.33 [1.28-1.38]	1.35 [1.30-1.41]	1.33 [1.27-1.38]	1.24 [1.09-1.41]	1.21 [1.16-1.26]	1.23 [1.18-1.28]	1.20 [1.15-1.25]	1.18 [1.13-1.23]	1.17 [1.03-1.34]	1.15 [1.10-1.19]	6,555	6,226	5,317	651	5,794
598.9	1.20 [1.13-1.28]	1.21 [1.13-1.30]	1.16 [1.07-1.25]	1.18 [1.03-1.35]	1.12 [1.05-1.20]	1.15 [1.07-1.22]	1.10 [1.03-1.18]	1.06 [0.98-1.15]	1.15 [1.00-1.32]	1.10 [1.02-1.17]	2,432	2,073	1,563	562	2,206
599	1.21 [1.18-1.23]	1.21 [1.19-1.24]	1.19 [1.17-1.22]	1.14 [1.05-1.25]	1.13 [1.10-1.15]	1.16 [1.14-1.19]	1.13 [1.11-1.15]	1.11 [1.09-1.14]	1.10 [1.00-1.21]	1.11 [1.09-1.13]	24,847	24,175	22,258	1,266	22,715
599.1	1.06 [1.03-1.09]	1.07 [1.04-1.10]	1.07 [1.04-1.10]	1.05 [0.97-1.15]	1.01 [0.99-1.04]	1.05 [1.02-1.08]	1.03 [1.01-1.06]	1.04 [1.01-1.07]	1.02 [0.94-1.12]	1.02 [0.99-1.05]	13,110	12,270	10,762	1,400	11,521
599.2	1.17 [1.15-1.19]	1.16 [1.14-1.18]	1.16 [1.14-1.18]	1.16 [1.08-1.25]	1.12 [1.10-1.14]	1.13 [1.11-1.15]	1.10 [1.08-1.11]	1.10 [1.08-1.12]	1.10 [1.02-1.19]	1.10 [1.09-1.12]	43,140	42,266	39,647	1,817	39,109
599.3	1.35 [1.29-1.41]	1.34 [1.28-1.41]	1.36 [1.29-1.44]	1.18 [1.08-1.29]	1.21 [1.16-1.27]	1.24 [1.19-1.30]	1.17 [1.11-1.23]	1.18 [1.11-1.25]	1.14 [1.05-1.25]	1.14 [1.09-1.20]	5,315	4,379	3,136	1,412	4,639
599.4	1.23 [1.21-1.26]	1.23 [1.21-1.26]	1.22 [1.19-1.24]	1.20 [1.12-1.29]	1.16 [1.14-1.18]	1.18 [1.15-1.20]	1.14 [1.11-1.16]	1.13 [1.10-1.15]	1.14 [1.06-1.23]	1.13 [1.11-1.16]	31,011	29,430	27,205	2,064	28,402
599.5	1.35 [1.30-1.40]	1.33 [1.29-1.38]	1.32 [1.27-1.38]	1.22 [1.10-1.35]	1.23 [1.19-1.28]	1.26 [1.21-1.30]	1.20 [1.15-1.25]	1.19 [1.14-1.24]	1.19 [1.07-1.32]	1.17 [1.12-1.21]	7,985	7,287	6,145	1,046	6,959
599.6	1.21 [1.11-1.33]	1.30 [1.18-1.43]	1.26 [1.14-1.39]	0.95 [0.73-1.23]	1.23 [1.11-1.35]	1.15 [1.05-1.27]	1.17 [1.06-1.30]	1.15 [1.03-1.27]	0.92 [0.71-1.21]	1.20 [1.09-1.32]	1,225	1,113	1,027	145	1,131
599.7	1.28 [1.00-1.63]	1.35 [1.04-1.75]	1.39 [1.05-1.85]		1.19 [0.92-1.54]	1.25 [0.97-1.60]	1.23 [0.94-1.61]	1.24 [0.92-1.66]		1.15 [0.88-1.48]	176	156	132	NA	155
599.9	1.39 [1.31-1.47]	1.35 [1.27-1.43]	1.33 [1.25-1.42]	1.47 [1.25-1.74]	1.24 [1.17-1.32]	1.28 [1.21-1.36]	1.20 [1.13-1.28]	1.19 [1.11-1.27]	1.44 [1.21-1.70]	1.18 [1.11-1.26]	2,972	2,692	2,310	412	2,602
600	1.16 [1.14-1.19]	1.16 [1.14-1.18]	1.16 [1.14-1.18]		1.10 [1.08-1.12]	1.12 [1.10-1.14]	1.10 [1.08-1.12]	1.10 [1.08-1.12]		1.07 [1.05-1.09]	30,069	30,057	29,975	NA	26,000
601.1	1.15 [1.08-1.23]	1.14 [1.07-1.22]	1.11 [1.03-1.18]		1.04 [0.97-1.12]	1.10 [1.02-1.17]	1.08 [1.00-1.15]	1.05 [0.98-1.12]		1.01 [0.93-1.08]	2,047	2,045	1,963	NA	1,726
601.11	1.09 [0.93-1.28]	1.10 [0.94-1.29]	1.07 [0.91-1.25]		0.95 [0.80-1.13]	1.05 [0.90-1.24]	1.04 [0.89-1.22]	1.01 [0.86-1.19]		0.92 [0.77-1.10]	362	362	356	NA	300
601.12	1.08 [0.98-1.19]	1.07 [0.97-1.17]	1.03 [0.94-1.14]		0.97 [0.88-1.08]	1.03 [0.94-1.14]	1.02 [0.93-1.13]	1.00 [0.91-1.10]		0.94 [0.85-1.04]	984	985	957	NA	840
601.3	1.21 [1.15-1.28]	1.26 [1.18-1.33]	1.25 [1.16-1.35]	1.12 [1.02-1.23]	1.11 [1.04-1.18]	1.15 [1.09-1.22]	1.15 [1.08-1.22]	1.15 [1.07-1.24]	1.09 [0.99-1.20]	1.09 [1.02-1.15]	3,388	2,598	1,768	1,211	2,758
601.4	1.45 [1.35-1.56]	1.55 [1.37-1.75]	1.54 [1.35-1.76]	1.37 [1.25-1.50]	1.31 [1.21-1.43]	1.40 [1.30-1.51]	1.40 [1.24-1.58]	1.41 [1.23-1.61]	1.33 [1.21-1.46]	1.29 [1.18-1.40]	1,874	694	572	1,233	1,539
601.8	1.36 [1.25-1.49]	1.39 [1.26-1.53]	1.37 [1.23-1.52]	1.28 [1.08-1.52]	1.22 [1.11-1.34]	1.31 [1.20-1.43]	1.28 [1.16-1.41]	1.25 [1.12-1.39]	1.25 [1.05-1.48]	1.21 [1.10-1.33]	1,355	1,073	883	362	1,125
602	1.11 [1.05-1.18]	1.11 [1.05-1.18]	1.13 [1.06-1.20]		1.03 [0.97-1.10]	1.07 [1.00-1.13]	1.06 [1.00-1.13]	1.08 [1.01-1.14]		1.01 [0.95-1.08]	2,640	2,638	2,612	NA	2,322
603	1.20 [1.14-1.25]	1.23 [1.16-1.30]	1.24 [1.15-1.33]	1.12 [1.06-1.19]	1.11 [1.06-1.17]	1.15 [1.10-1.20]	1.12 [1.06-1.19]	1.14 [1.06-1.23]	1.10 [1.03-1.17]	1.10 [1.04-1.15]	5,311	2,922	1,852	2,965	4,266
603.1	1.12 [1.07-1.18]	1.21 [1.14-1.28]	1.19 [1.12-1.27]	1.04 [0.97-1.12]	1.10 [1.04-1.16]	1.10 [1.05-1.15]	1.16 [1.09-1.23]	1.14 [1.07-1.22]	1.02 [0.95-1.10]	1.09 [1.03-1.14]	4,538	2,690	2,253	2,075	3,697
604	1.38 [1.31-1.45]	1.42 [1.34-1.51]	1.40 [1.31-1.50]	1.30 [1.20-1.41]	1.27 [1.20-1.34]	1.33 [1.26-1.40]	1.32 [1.24-1.41]	1.30 [1.21-1.40]	1.27 [1.17-1.39]	1.25 [1.18-1.33]	4,085	2,747	2,122	1,637	3,445
604.1	1.46 [1.42-1.50]	1.43 [1.38-1.48]	1.38 [1.32-1.44]	1.46 [1.41-1.50]	1.37 [1.33-1.41]	1.40 [1.37-1.44]	1.32 [1.28-1.37]	1.28 [1.23-1.34]	1.41 [1.36-1.46]	1.34 [1.30-1.38]	16,264	7,471	4,922	10,132	12,324
605	1.44 [1.30-1.61]	1.44 [1.29-1.60]	1.44 [1.29-1.61]		1.29 [1.16-1.45]	1.31 [1.17-1.46]	1.24 [1.11-1.38]	1.24 [1.10-1.39]		1.22 [1.08-1.37]	853	849	789	NA	760
608	1.22 [1.18-1.27]	1.25 [1.20-1.31]	1.26 [1.20-1.33]	1.12 [1.06-1.19]	1.15 [1.10-1.20]	1.17 [1.12-1.21]	1.16 [1.11-1.21]	1.17 [1.11-1.23]	1.08 [1.02-1.15]	1.12 [1.08-1.17]	7,430	5,100	3,792	2,982	6,096
610.1	1.21 [1.10-1.34]	1.17 [1.06-1.30]	1.15 [1.03-1.29]		1.14 [1.02-1.27]	1.16 [1.05-1.29]	1.13 [1.02-1.25]	1.09 [0.97-1.23]		1.13 [1.02-1.27]	906	891	698	NA	762
610.2	1.06 [0.89-1.27]	1.01 [0.84-1.20]	0.91 [0.74-1.13]		0.99 [0.81-1.21]	1.03 [0.86-1.24]	0.97 [0.81-1.17]	0.88 [0.71-1.09]		0.99 [0.81-1.22]	285	273	193	NA	214
610.3	1.15 [0.96-1.36]	1.10 [0.92-1.30]	1.08 [0.90-1.31]		1.03 [0.85-1.25]	1.12 [0.94-1.34]	1.06 [0.89-1.27]	1.07 [0.88-1.30]		1.04 [0.86-1.27]	304	302	243	NA	241
610.4	1.05 [0.99-1.11]	1.04 [0.98-1.11]	1.02 [0.94-1.11]	1.13 [1.00-1.28]	0.99 [0.93-1.06]	1.01 [0.95-1.07]	0.99 [0.93-1.06]	0.98 [0.90-1.07]	1.12 [0.98-1.27]	0.97 [0.90-1.04]	2,632	2,432	1,299	675	1,975
610.8	1.17 [1.10-1.25]	1.14 [1.07-1.22]	1.11 [1.03-1.19]	1.37 [1.01-1.85]	1.10 [1.02-1.18]	1.13 [1.06-1.21]	1.09 [1.02-1.16]	1.05 [0.97-1.13]	1.34 [0.98-1.84]	1.09 [1.01-1.17]	2,183	2,148	1,645	118	1,789
611.1	1.09 [0.81-1.46]	1.09 [0.81-1.46]	1.07 [0.80-1.44]			1.06 [0.78-1.43]	0.97 [0.72-1.32]	1.01 [0.75-1.37]			109	110	108	NA	NA
611.3	1.16 [1.09-1.23]	1.17 [1.09-1.24]	1.16 [1.08-1.25]	1.19 [0.93-1.53]	1.10 [1.02-1.18]	1.11 [1.04-1.18]	1.09 [1.02-1.16]	1.08 [1.00-1.16]	1.16 [0.90-1.50]	1.08 [1.00-1.16]	2,320	2,264	1,839	163	1,932
612.2	1.28 [1.18-1.39]	1.28 [1.17-1.39]	1.27 [1.13-1.42]	1.30 [1.09-1.55]	1.16 [1.05-1.27]	1.21 [1.11-1.32]	1.15 [1.05-1.26]	1.11 [0.99-1.25]	1.26 [1.05-1.51]	1.12 [1.01-1.23]	1,388	1,242	739	339	1,060
612.3	1.13 [0.96-1.33]	1.15 [0.96-1.38]		0.97 [0.76-1.25]	1.04 [0.86-1.26]	1.06 [0.90-1.26]	1.05 [0.87-1.26]		0.93 [0.73-1.20]	0.99 [0.82-1.21]	352	278	NA	154	250
613	1.25 [1.18-1.34]	1.25 [1.17-1.33]	1.21 [1.12-1.30]	1.37 [1.09-1.71]	1.18 [1.11-1.27]	1.19 [1.12-1.27]	1.15 [1.07-1.23]	1.11 [1.03-1.20]	1.35 [1.07-1.70]	1.15 [1.08-1.24]	2,374	2,310	1,728	217	2,070
613.1	1.31 [1.22-1.40]	1.33 [1.24-1.43]	1.32 [1.20-1.45]	1.25 [1.02-1.52]	1.26 [1.17-1.36]	1.25 [1.16-1.34]	1.21 [1.12-1.30]	1.20 [1.09-1.32]	1.22 [0.99-1.50]	1.24 [1.15-1.34]	1,921	1,833	1,124	267	1,658
613.5	1.47 [1.25-1.73]	1.41 [1.20-1.66]	1.35 [1.10-1.66]		1.32 [1.12-1.55]	1.36 [1.15-1.60]	1.22 [1.03-1.44]	1.19 [0.96-1.47]		1.24 [1.05-1.47]	395	379	231	NA	365
613.7	1.23 [1.10-1.37]	1.22 [1.09-1.37]	1.22 [1.08-1.39]		1.13 [1.00-1.27]	1.16 [1.04-1.31]	1.13 [1.00-1.27]	1.12 [0.99-1.28]		1.09 [0.96-1.23]	738	719	578	NA	617
613.8	1.27 [1.13-1.42]	1.29 [1.15-1.45]	1.26 [1.11-1.44]		1.24 [1.09-1.40]	1.23 [1.09-1.38]	1.23 [1.09-1.38]	1.19 [1.04-1.36]		1.21 [1.07-1.37]	733	715	539	NA	639
613.9	1.23 [1.01-1.49]	1.18 [0.97-1.43]	1.16 [0.93-1.45]		1.17 [0.95-1.43]	1.14 [0.93-1.39]	1.07 [0.88-1.31]	1.08 [0.86-1.35]		1.15 [0.93-1.42]	262	249	198	NA	228
614	1.05 [0.78-1.41]					0.95 [0.70-1.29]					103	NA	NA	NA	NA
614.1	1.14 [1.10-1.18]	1.16 [1.12-1.20]	1.13 [1.07-1.19]	1.12 [1.00-1.26]	1.08 [1.04-1.12]	1.08 [1.04-1.12]	1.07 [1.03-1.11]	1.04 [0.99-1.10]	1.11 [0.98-1.25]	1.05 [1.01-1.09]	7,098	7,047	3,522	775	6,138
614.3	1.07 [0.99-1.15]	1.08 [1.00-1.17]	1.06 [0.95-1.18]	1.08 [0.85-1.37]	1.00 [0.92-1.09]	1.03 [0.95-1.11]	1.01 [0.93-1.09]	1.00 [0.89-1.11]	1.06 [0.83-1.35]	0.98 [0.90-1.07]	1,521	1,491	779	181	1,282
614.31	1.10 [0.86-1.40]	1.18 [0.93-1.51]			1.02 [0.78-1.32]	1.05 [0.82-1.35]	1.08 [0.84-1.40]			1.00 [0.76-1.30]	163	159	NA	NA	136
614.32	1.08 [0.98-1.18]	1.10 [1.00-1.21]	1.10 [0.97-1.25]	1.23 [0.91-1.67]	1.01 [0.91-1.12]	1.04 [0.95-1.15]	1.03 [0.93-1.13]	1.04 [0.91-1.18]	1.22 [0.89-1.67]	0.99 [0.89-1.10]	1,042	1,034	523	112	875
614.33	1.13 [1.05-1.21]	1.16 [1.08-1.25]	1.14 [1.01-1.29]	1.08 [0.95-1.23]	1.03 [0.95-1.11]	1.04 [0.96-1.12]	1.00 [0.92-1.08]	1.05 [0.92-1.19]	1.03 [0.90-1.18]	0.97 [0.90-1.05]	1,898	1,761	568	611	1,609
614.4	1.17 [1.05-1.31]	1.23 [1.10-1.37]	1.25 [1.09-1.42]		1.14 [1.02-1.28]	1.08 [0.97-1.21]	1.12 [1.01-1.26]	1.13 [0.99-1.30]		1.08 [0.96-1.22]	792	791	507	NA	699
614.5	1.15 [1.08-1.22]	1.15 [1.08-1.22]	1.21 [1.11-1.31]	1.00 [0.86-1.16]	1.11 [1.04-1.18]	1.11 [1.04-1.18]	1.07 [1.01-1.14]	1.13 [1.04-1.23]	1.00 [0.85-1.16]	1.08 [1.01-1.15]	2,641	2,545	1,310	441	2,182
614.51	1.09 [1.01-1.18]	1.12 [1.04-1.21]	1.12 [1.01-1.24]	0.95 [0.72-1.26]	1.04 [0.96-1.14]	1.04 [0.96-1.13]	1.06 [0.98-1.15]	1.07 [0.96-1.19]	0.96 [0.72-1.28]	1.02 [0.93-1.12]	1,444	1,441	782	126	1,136
614.52	1.36 [1.25-1.48]	1.44 [1.31-1.59]	1.57 [1.40-1.78]	1.26 [1.09-1.47]	1.32 [1.21-1.45]	1.28 [1.17-1.39]	1.29 [1.17-1.42]	1.42 [1.25-1.61]	1.21 [1.03-1.41]	1.26 [1.15-1.39]	1,382	1,081	680	452	1,188
614.53	1.06 [0.97-1.15]	1.04 [0.96-1.13]	1.06 [0.93-1.20]	0.92 [0.77-1.11]	1.01 [0.92-1.10]	1.04 [0.95-1.12]	0.98 [0.91-1.07]	1.02 [0.89-1.16]	0.92 [0.76-1.11]	1.00 [0.91-1.10]	1,427	1,374	515	297	1,115
614.54	1.40 [1.27-1.54]	1.43 [1.30-1.59]	1.49 [1.30-1.70]	1.12 [0.91-1.37]	1.32 [1.19-1.46]	1.33 [1.20-1.46]	1.28 [1.15-1.42]	1.32 [1.15-1.52]	1.07 [0.87-1.32]	1.28 [1.15-1.42]	1,088	976	536	262	910
615	1.13 [1.09-1.17]	1.15 [1.11-1.18]	1.09 [1.03-1.14]	1.16 [1.06-1.27]	1.08 [1.04-1.12]	1.06 [1.02-1.10]	1.04 [1.00-1.07]	1.00 [0.95-1.05]	1.12 [1.02-1.22]	1.03 [0.99-1.07]	8,651	8,539	3,717	1,438	7,115
617	1.34 [1.21-1.47]	1.35 [1.22-1.48]	1.31 [1.18-1.46]		1.25 [1.13-1.38]	1.26 [1.14-1.39]	1.24 [1.12-1.37]	1.19 [1.07-1.33]		1.21 [1.10-1.34]	1,059	1,046	826	NA	966
618	1.24 [1.10-1.40]	1.24 [1.11-1.40]	1.18 [1.04-1.34]		1.12 [0.99-1.26]	1.16 [1.03-1.31]	1.15 [1.02-1.30]	1.08 [0.95-1.23]		1.07 [0.94-1.21]	678	678	569	NA	603
618.1	1.21 [1.18-1.24]	1.20 [1.17-1.24]	1.20 [1.17-1.23]	1.28 [0.97-1.69]	1.15 [1.12-1.18]	1.15 [1.12-1.18]	1.13 [1.10-1.16]	1.12 [1.09-1.15]	1.28 [0.96-1.69]	1.10 [1.07-1.13]	14,347	14,319	13,052	141	12,136
618.2	1.14 [1.10-1.18]	1.13 [1.09-1.17]	1.13 [1.09-1.17]		1.08 [1.04-1.13]	1.11 [1.06-1.15]	1.09 [1.05-1.13]	1.09 [1.05-1.13]		1.06 [1.02-1.11]	6,596	6,598	6,086	NA	5,466
618.5	1.24 [1.08-1.42]	1.24 [1.08-1.43]	1.23 [1.07-1.42]		1.08 [0.94-1.25]	1.18 [1.03-1.36]	1.16 [1.01-1.34]	1.15 [1.00-1.32]		1.04 [0.90-1.20]	480	479	470	NA	425
618.6	1.20 [1.08-1.33]	1.22 [1.10-1.36]	1.21 [1.08-1.34]		1.14 [1.02-1.27]	1.12 [1.00-1.25]	1.12 [1.00-1.25]	1.09 [0.98-1.22]		1.07 [0.96-1.20]	818	816	777	NA	750
619	1.37 [1.12-1.69]	1.45 [1.17-1.79]	1.52 [1.14-2.04]		1.17 [0.93-1.48]	1.27 [1.02-1.56]	1.28 [1.03-1.60]	1.37 [1.01-1.86]		1.08 [0.85-1.37]	225	209	111	NA	166
619.1	1.13 [1.10-1.16]	1.15 [1.12-1.18]	1.12 [1.08-1.16]	1.11 [1.05-1.17]	1.08 [1.05-1.11]	1.07 [1.04-1.10]	1.04 [1.01-1.07]	1.04 [1.00-1.07]	1.06 [1.00-1.13]	1.04 [1.01-1.07]	14,639	13,619	6,998	3,120	12,225
619.2	1.16 [1.12-1.21]	1.18 [1.14-1.23]	1.19 [1.14-1.25]	1.10 [0.96-1.27]	1.12 [1.08-1.17]	1.11 [1.07-1.15]	1.10 [1.06-1.14]	1.12 [1.07-1.17]	1.08 [0.93-1.25]	1.08 [1.04-1.13]	6,636	6,594	4,757	535	5,644
619.3	1.18 [1.14-1.23]	1.19 [1.14-1.24]	1.19 [1.12-1.26]	1.09 [0.97-1.21]	1.11 [1.07-1.16]	1.13 [1.09-1.18]	1.12 [1.07-1.16]	1.13 [1.06-1.19]	1.05 [0.94-1.18]	1.08 [1.03-1.13]	6,068	6,016	2,852	856	4,976
619.4	1.28 [1.22-1.33]	1.29 [1.23-1.35]	1.30 [1.22-1.39]	1.08 [0.98-1.18]	1.16 [1.11-1.21]	1.20 [1.15-1.26]	1.17 [1.12-1.22]	1.21 [1.13-1.29]	1.04 [0.95-1.15]	1.11 [1.06-1.17]	5,278	4,969	2,047	1,166	4,498
619.5	1.21 [1.16-1.27]	1.20 [1.15-1.26]	1.38 [1.29-1.49]	1.09 [0.97-1.22]	1.14 [1.08-1.20]	1.17 [1.12-1.23]	1.14 [1.09-1.20]	1.27 [1.18-1.37]	1.08 [0.95-1.21]	1.10 [1.05-1.16]	4,461	4,302	1,748	737	3,762
620	1.48 [1.22-1.81]	1.48 [1.21-1.80]	1.44 [1.15-1.80]		1.41 [1.14-1.73]	1.41 [1.15-1.73]	1.33 [1.09-1.63]	1.29 [1.03-1.62]		1.33 [1.07-1.64]	254	255	195	NA	220
621	1.17 [1.09-1.27]	1.17 [1.08-1.26]	1.14 [1.05-1.23]		1.07 [0.98-1.16]	1.15 [1.06-1.24]	1.13 [1.04-1.22]	1.10 [1.02-1.20]		1.06 [0.98-1.16]	1,550	1,551	1,362	NA	1,255
622	1.21 [1.02-1.44]	1.28 [1.07-1.52]	1.22 [1.00-1.50]		1.27 [1.05-1.53]	1.15 [0.96-1.37]	1.20 [1.01-1.44]	1.15 [0.94-1.42]		1.22 [1.01-1.47]	316	304	218	NA	268
622.1	1.12 [1.09-1.16]	1.13 [1.09-1.16]	1.12 [1.09-1.16]	1.17 [0.93-1.48]	1.08 [1.05-1.12]	1.10 [1.07-1.13]	1.09 [1.06-1.12]	1.09 [1.06-1.13]	1.09 [0.86-1.39]	1.07 [1.03-1.10]	11,263	11,250	9,786	199	8,858
622.2	1.10 [1.03-1.16]	1.10 [1.04-1.17]	1.09 [1.02-1.16]		1.06 [0.99-1.13]	1.08 [1.01-1.15]	1.10 [1.03-1.17]	1.08 [1.01-1.15]		1.05 [0.98-1.12]	2,477	2,478	2,127	NA	1,887
623	1.13 [1.06-1.20]	1.14 [1.07-1.21]	1.14 [1.06-1.22]	1.05 [0.83-1.33]	1.09 [1.02-1.17]	1.09 [1.02-1.16]	1.06 [1.00-1.13]	1.07 [0.99-1.15]	1.01 [0.79-1.29]	1.06 [0.99-1.13]	2,484	2,432	1,760	179	2,138

Supplementary Table 6: phecode-mapped results (hazard ratios and events from all cohorts)

Outcome	Hazard ratio (99% confidence interval): crude, any age	crude, 18+	crude, 40+	crude, <18	crude, hosp.	adjusted, any age	adjusted, 18+	adjusted, 40+	adjusted, <18	adjusted, hosp.	Events (in exposed): any age	18+	40+	<18	hosp.
624.1	1.64 [1.48-1.83]	1.63 [1.46-1.81]	1.64 [1.47-1.83]		1.50 [1.34-1.68]	1.53 [1.37-1.70]	1.48 [1.32-1.65]	1.50 [1.34-1.69]		1.43 [1.28-1.60]	927	915	837	NA	823
624.2	1.30 [1.18-1.43]	1.28 [1.16-1.41]	1.28 [1.16-1.41]		1.22 [1.10-1.35]	1.25 [1.13-1.38]	1.22 [1.10-1.34]	1.20 [1.09-1.33]		1.19 [1.07-1.32]	999	996	979	NA	857
624.9	1.38 [1.33-1.43]	1.38 [1.33-1.43]	1.37 [1.32-1.42]	1.48 [1.12-1.94]	1.27 [1.23-1.33]	1.26 [1.21-1.31]	1.22 [1.18-1.27]	1.21 [1.16-1.26]	1.38 [1.04-1.84]	1.20 [1.16-1.25]	6,882	6,834	5,957	148	6,068
625	1.23 [1.18-1.30]	1.27 [1.21-1.34]	1.22 [1.14-1.32]	1.14 [1.02-1.28]	1.13 [1.07-1.19]	1.16 [1.10-1.22]	1.14 [1.08-1.20]	1.11 [1.03-1.20]	1.12 [0.99-1.26]	1.08 [1.03-1.14]	4,109	3,978	1,652	764	3,414
625.1	1.33 [1.25-1.41]	1.36 [1.28-1.44]	1.37 [1.23-1.52]	1.32 [1.16-1.49]	1.24 [1.16-1.32]	1.21 [1.14-1.29]	1.18 [1.11-1.26]	1.22 [1.09-1.36]	1.25 [1.10-1.42]	1.16 [1.09-1.24]	2,946	2,923	828	678	2,459
626	1.21 [1.16-1.26]	1.21 [1.16-1.26]	1.19 [1.12-1.26]	1.18 [1.07-1.29]	1.11 [1.07-1.16]	1.14 [1.10-1.19]	1.09 [1.04-1.14]	1.10 [1.03-1.17]	1.14 [1.04-1.25]	1.09 [1.04-1.14]	5,712	5,363	2,693	1,214	4,937
626.1	1.17 [1.10-1.26]	1.19 [1.11-1.27]	1.14 [1.04-1.25]	1.19 [0.99-1.42]	1.09 [1.02-1.18]	1.11 [1.03-1.19]	1.08 [1.00-1.16]	1.05 [0.96-1.15]	1.19 [0.99-1.43]	1.05 [0.98-1.14]	1,996	1,872	1,047	334	1,662
626.11	1.29 [1.13-1.47]	1.30 [1.13-1.49]	1.52 [1.19-1.93]	1.14 [0.92-1.41]	1.15 [1.00-1.32]	1.22 [1.06-1.39]	1.14 [0.99-1.32]	1.41 [1.10-1.81]	1.11 [0.89-1.38]	1.10 [0.95-1.27]	609	491	160	239	485
626.12	1.20 [1.17-1.23]	1.20 [1.17-1.23]	1.17 [1.14-1.21]	1.23 [1.13-1.35]	1.14 [1.11-1.18]	1.13 [1.10-1.16]	1.10 [1.07-1.13]	1.08 [1.05-1.11]	1.19 [1.09-1.31]	1.10 [1.07-1.13]	14,375	13,834	9,103	1,385	12,061
626.13	1.21 [1.14-1.30]	1.23 [1.15-1.31]	1.23 [1.12-1.34]	1.18 [1.00-1.39]	1.15 [1.07-1.24]	1.12 [1.05-1.20]	1.10 [1.02-1.18]	1.12 [1.03-1.23]	1.13 [0.95-1.34]	1.10 [1.03-1.19]	2,162	2,046	1,125	396	1,793
626.14	1.22 [1.17-1.27]	1.23 [1.18-1.28]	1.19 [1.13-1.25]	1.15 [0.99-1.34]	1.16 [1.11-1.22]	1.14 [1.09-1.19]	1.12 [1.07-1.17]	1.10 [1.05-1.17]	1.08 [0.93-1.27]	1.11 [1.06-1.16]	5,245	5,150	3,165	464	4,438
626.2	1.22 [1.16-1.29]	1.23 [1.17-1.30]	1.18 [1.08-1.28]	1.21 [1.07-1.37]	1.16 [1.09-1.23]	1.12 [1.06-1.19]	1.08 [1.02-1.15]	1.05 [0.96-1.15]	1.13 [1.00-1.28]	1.11 [1.04-1.18]	3,238	2,983	1,225	753	2,703
626.21	1.12 [0.92-1.37]	1.04 [0.82-1.32]		1.12 [0.87-1.44]	1.01 [0.81-1.25]	1.04 [0.85-1.27]	0.94 [0.73-1.20]		1.09 [0.84-1.41]	0.97 [0.77-1.22]	267	164	NA	170	203
626.4	1.18 [0.94-1.48]	1.21 [0.96-1.52]	1.34 [1.00-1.78]		1.11 [0.88-1.40]	1.02 [0.81-1.29]	0.99 [0.78-1.26]	1.16 [0.86-1.57]		1.05 [0.82-1.33]	184	176	107	NA	169
626.8	0.93 [0.86-1.01]	0.95 [0.87-1.03]	0.97 [0.75-1.26]		0.97 [0.88-1.08]	0.92 [0.85-1.00]	0.93 [0.86-1.01]	0.98 [0.76-1.27]		0.96 [0.86-1.07]	1,306	1,306	125	NA	800
627	1.19 [1.02-1.40]	1.22 [1.04-1.43]	1.22 [1.04-1.43]		1.11 [0.93-1.32]	1.12 [0.95-1.32]	1.14 [0.97-1.34]	1.13 [0.96-1.33]		1.05 [0.88-1.26]	360	361	353	NA	283
627.1	1.24 [1.20-1.28]	1.23 [1.19-1.27]	1.23 [1.19-1.27]		1.18 [1.14-1.23]	1.19 [1.15-1.23]	1.17 [1.13-1.21]	1.16 [1.12-1.20]		1.15 [1.11-1.20]	8,737	8,737	8,697	NA	6,993
627.2	1.39 [1.10-1.76]	1.49 [1.17-1.89]	1.49 [1.17-1.90]		1.38 [1.07-1.78]	1.26 [0.99-1.60]	1.30 [1.02-1.66]	1.33 [1.04-1.71]		1.30 [1.01-1.68]	175	174	163	NA	148
627.3	1.30 [1.21-1.39]	1.32 [1.23-1.42]	1.30 [1.22-1.40]		1.22 [1.14-1.32]	1.23 [1.14-1.32]	1.23 [1.15-1.32]	1.22 [1.13-1.30]		1.18 [1.10-1.28]	2,050	2,051	2,045	NA	1,791
627.4	1.46 [1.22-1.74]	1.45 [1.21-1.74]	1.40 [1.16-1.68]		1.49 [1.22-1.81]	1.39 [1.16-1.67]	1.36 [1.13-1.64]	1.31 [1.08-1.58]		1.41 [1.16-1.73]	292	290	270	NA	249
627.5	1.39 [1.09-1.76]	1.61 [1.26-2.06]			1.48 [1.15-1.89]	1.25 [0.98-1.60]	1.33 [1.03-1.72]			1.38 [1.07-1.78]	187	175	NA	NA	163
628	1.14 [1.11-1.17]	1.15 [1.12-1.19]	1.13 [1.08-1.17]	1.10 [1.03-1.16]	1.08 [1.05-1.12]	1.07 [1.04-1.10]	1.04 [1.01-1.07]	1.04 [1.00-1.08]	1.05 [0.98-1.12]	1.04 [1.01-1.07]	13,108	12,192	6,131	2,839	10,981
pregnancy complications															
634	1.07 [1.06-1.08]	1.12 [1.10-1.13]	1.06 [0.99-1.14]	0.98 [0.96-1.00]	0.98 [0.96-1.00]	1.06 [1.05-1.07]	1.08 [1.07-1.09]	1.10 [1.02-1.18]	0.98 [0.96-1.00]	0.98 [0.96-1.00]	80,817	77,228	2,350	30,496	38,506
634.1	1.10 [1.05-1.14]	1.11 [1.07-1.16]	1.03 [0.92-1.15]	1.07 [0.98-1.18]	1.05 [1.00-1.09]	1.07 [1.02-1.11]	1.07 [1.03-1.12]	1.01 [0.89-1.14]	1.07 [0.97-1.17]	1.03 [0.99-1.08]	5,887	5,819	624	1,182	4,714
634.3	1.04 [0.98-1.11]	1.09 [1.03-1.16]	1.02 [0.81-1.29]	1.02 [0.90-1.16]	0.99 [0.92-1.05]	1.02 [0.95-1.08]	1.03 [0.97-1.10]	1.01 [0.79-1.29]	0.99 [0.87-1.13]	0.98 [0.92-1.05]	2,641	2,620	150	632	2,156
635	1.01 [0.76-1.33]	1.08 [0.82-1.43]			0.94 [0.71-1.26]	0.99 [0.75-1.32]	1.09 [0.82-1.45]			0.97 [0.72-1.30]	120	120	NA	NA	106
635.2	1.09 [1.06-1.13]	1.13 [1.09-1.16]	1.02 [0.90-1.17]	1.03 [0.97-1.09]	1.05 [1.01-1.08]	1.07 [1.04-1.10]	1.07 [1.04-1.11]	1.01 [0.88-1.16]	1.01 [0.96-1.08]	1.03 [1.00-1.07]	11,241	11,068	479	3,014	8,809
635.3	1.05 [1.03-1.08]	1.08 [1.06-1.10]	0.97 [0.89-1.06]	0.98 [0.94-1.02]	1.02 [1.00-1.04]	1.05 [1.02-1.07]	1.07 [1.05-1.09]	1.00 [0.92-1.10]	0.97 [0.93-1.02]	1.02 [0.99-1.04]	23,598	23,373	1,079	6,369	17,714
636	1.16 [1.12-1.21]	1.17 [1.13-1.22]	0.95 [0.75-1.20]	1.05 [0.97-1.14]	1.07 [1.02-1.12]	1.12 [1.07-1.16]	1.10 [1.06-1.14]	0.91 [0.71-1.16]	1.04 [0.95-1.13]	1.05 [1.00-1.10]	6,347	6,178	146	1,471	4,703
636.2	1.08 [1.04-1.11]	1.11 [1.07-1.14]	1.06 [0.93-1.21]	1.02 [0.96-1.08]	1.03 [0.99-1.06]	1.05 [1.01-1.08]	1.05 [1.02-1.09]	1.03 [0.90-1.18]	1.02 [0.96-1.09]	1.02 [0.98-1.06]	9,664	9,541	479	2,675	7,666
636.3	1.06 [1.03-1.10]	1.10 [1.06-1.14]	0.97 [0.83-1.13]	1.00 [0.94-1.07]	1.00 [0.96-1.04]	1.03 [0.99-1.07]	1.03 [1.00-1.07]	0.98 [0.84-1.15]	1.00 [0.93-1.06]	0.99 [0.95-1.03]	8,064	7,869	356	2,475	6,494
636.8	1.06 [0.92-1.22]	1.06 [0.92-1.22]			0.99 [0.86-1.15]	1.05 [0.91-1.21]	1.04 [0.90-1.20]			1.00 [0.87-1.17]	483	482	NA	NA	431
638	1.10 [1.04-1.16]	1.14 [1.07-1.20]	0.94 [0.83-1.06]	1.03 [0.90-1.18]	1.03 [0.97-1.09]	1.07 [1.00-1.13]	1.09 [1.03-1.16]	0.96 [0.85-1.09]	0.99 [0.86-1.13]	1.02 [0.96-1.09]	2,961	2,932	563	561	2,662
639	1.05 [0.95-1.16]	1.09 [0.98-1.20]		0.99 [0.83-1.18]	0.97 [0.87-1.09]	1.01 [0.91-1.12]	1.01 [0.91-1.12]		0.99 [0.83-1.18]	0.97 [0.87-1.09]	942	916	NA	336	792
642	1.09 [1.06-1.12]	1.12 [1.08-1.15]	1.05 [0.93-1.19]	1.05 [0.98-1.12]	1.06 [1.02-1.10]	1.06 [1.03-1.10]	1.08 [1.05-1.12]	1.03 [0.91-1.17]	1.03 [0.96-1.10]	1.04 [1.00-1.08]	9,573	9,424	560	2,369	6,712
642.1	1.10 [1.05-1.16]	1.13 [1.07-1.18]	0.98 [0.80-1.20]	1.11 [1.02-1.22]	1.06 [1.00-1.12]	1.07 [1.02-1.13]	1.09 [1.03-1.14]	0.96 [0.78-1.18]	1.11 [1.01-1.22]	1.03 [0.97-1.09]	4,171	4,093	207	1,292	2,917
643	1.11 [1.06-1.15]	1.16 [1.11-1.21]		1.03 [0.97-1.10]	1.02 [0.97-1.06]	1.04 [1.00-1.09]	1.05 [1.00-1.09]		1.01 [0.94-1.08]	0.99 [0.94-1.03]	5,975	5,800	NA	2,422	4,869
643.1	1.08 [1.03-1.14]	1.14 [1.09-1.20]		1.01 [0.94-1.09]	1.01 [0.96-1.07]	1.02 [0.97-1.08]	1.04 [0.98-1.09]		0.99 [0.91-1.07]	0.98 [0.93-1.04]	4,069	3,948	NA	1,725	3,330
644	1.07 [1.03-1.11]	1.10 [1.06-1.13]	1.00 [0.86-1.15]	1.01 [0.95-1.08]	1.00 [0.97-1.04]	1.06 [1.02-1.09]	1.08 [1.04-1.12]	0.98 [0.85-1.14]	1.01 [0.94-1.08]	1.01 [0.97-1.04]	8,673	8,547	393	2,495	6,718
645	1.03 [1.00-1.06]	1.05 [1.02-1.07]	0.87 [0.75-1.01]	0.99 [0.93-1.04]	0.98 [0.95-1.01]	1.02 [1.00-1.05]	1.04 [1.01-1.07]	0.89 [0.77-1.04]	0.98 [0.92-1.03]	0.98 [0.94-1.01]	12,342	12,175	376	3,275	8,546
646	1.18 [1.16-1.20]	1.21 [1.18-1.23]	1.01 [0.93-1.11]	1.07 [1.03-1.10]	1.09 [1.07-1.12]	1.12 [1.10-1.14]	1.10 [1.08-1.13]	0.98 [0.89-1.08]	1.04 [1.01-1.08]	1.06 [1.04-1.08]	31,167	30,354	1,005	9,895	23,736
647	1.11 [1.04-1.18]	1.16 [1.09-1.23]		0.99 [0.88-1.10]	1.07 [1.00-1.14]	1.08 [1.01-1.15]	1.11 [1.04-1.18]		0.97 [0.86-1.08]	1.04 [0.97-1.12]	2,723	2,693	NA	851	2,184
647.1	1.16 [1.12-1.20]	1.19 [1.15-1.24]	1.02 [0.82-1.27]	1.04 [0.97-1.10]	1.08 [1.04-1.13]	1.11 [1.07-1.15]	1.09 [1.05-1.14]	0.97 [0.78-1.21]	1.03 [0.96-1.09]	1.06 [1.02-1.11]	7,294	7,072	177	2,648	5,969
647.3	1.09 [0.99-1.20]	1.10 [1.00-1.21]		0.95 [0.80-1.13]	1.04 [0.94-1.15]	1.05 [0.95-1.15]	1.03 [0.93-1.14]		0.92 [0.77-1.10]	1.01 [0.91-1.12]	1,088	1,078	NA	356	897
649	1.21 [1.17-1.25]	1.26 [1.22-1.30]	1.11 [0.97-1.27]	1.09 [1.02-1.15]	1.13 [1.09-1.17]	1.15 [1.11-1.19]	1.16 [1.12-1.20]	1.06 [0.92-1.21]	1.06 [0.99-1.13]	1.10 [1.06-1.14]	9,473	9,451	495	2,958	8,033
649.1	1.07 [1.03-1.11]	1.09 [1.06-1.13]	1.04 [0.93-1.16]	1.00 [0.92-1.09]	1.02 [0.98-1.06]	1.05 [1.01-1.09]	1.07 [1.03-1.11]	1.03 [0.92-1.16]	1.02 [0.94-1.11]	1.01 [0.97-1.05]	7,581	7,552	683	1,507	6,382
650	1.08 [1.05-1.11]	1.09 [1.06-1.12]	1.05 [0.89-1.24]	0.94 [0.89-1.00]	0.97 [0.94-1.00]	1.09 [1.06-1.12]	1.09 [1.06-1.12]	1.11 [0.94-1.32]	0.96 [0.91-1.02]	1.00 [0.97-1.03]	12,482	12,266	312	2,989	10,079
651	1.07 [1.06-1.09]	1.12 [1.10-1.13]	1.06 [0.99-1.13]	0.98 [0.96-1.00]	1.00 [0.98-1.01]	1.06 [1.05-1.08]	1.08 [1.07-1.10]	1.12 [1.04-1.20]	0.98 [0.96-1.00]	1.00 [0.99-1.02]	78,231	76,189	2,196	27,276	41,408
652	1.03 [1.01-1.06]	1.06 [1.03-1.08]	0.95 [0.86-1.06]	0.98 [0.93-1.03]	1.00 [0.97-1.02]	1.02 [1.00-1.05]	1.04 [1.01-1.07]	0.96 [0.86-1.07]	0.97 [0.92-1.02]	0.99 [0.96-1.02]	16,378	16,241	755	3,926	12,111
653	1.05 [1.03-1.08]	1.08 [1.06-1.10]	0.89 [0.81-0.99]	1.00 [0.96-1.04]	1.02 [1.00-1.05]	1.05 [1.02-1.07]	1.06 [1.03-1.08]	0.94 [0.84-1.04]	1.00 [0.96-1.04]	1.03 [1.00-1.05]	23,548	23,281	790	6,589	17,376
654	1.04 [0.97-1.12]	1.07 [1.00-1.15]		0.97 [0.85-1.11]	1.01 [0.93-1.10]	1.03 [0.95-1.11]	1.06 [0.98-1.14]		0.97 [0.85-1.11]	1.00 [0.92-1.09]	1,807	1,769	NA	573	1,361
654.1	1.10 [1.08-1.13]	1.12 [1.09-1.15]	1.03 [0.94-1.12]	0.98 [0.92-1.04]	1.02 [0.99-1.04]	1.08 [1.06-1.11]	1.10 [1.07-1.13]	1.05 [0.96-1.15]	0.97 [0.91-1.04]	1.02 [0.99-1.04]	15,511	15,518	1,145	2,277	14,592
654.2	1.09 [1.01-1.17]	1.11 [1.02-1.19]		1.09 [0.93-1.27]	1.01 [0.92-1.10]	1.07 [0.99-1.16]	1.08 [1.00-1.17]		1.07 [0.91-1.26]	1.01 [0.93-1.10]	1,621	1,598	NA	416	1,254
655	1.08 [1.06-1.09]	1.11 [1.10-1.13]	0.97 [0.90-1.04]	1.01 [0.98-1.04]	1.02 [1.01-1.04]	1.06 [1.04-1.07]	1.07 [1.06-1.09]	0.98 [0.91-1.05]	1.00 [0.98-1.03]	1.02 [1.00-1.03]	52,442	51,477	1,728	17,094	35,811
656	1.18 [1.14-1.22]	1.22 [1.18-1.25]	1.14 [0.99-1.32]	1.08 [1.02-1.14]	1.09 [1.05-1.13]	1.11 [1.08-1.15]	1.09 [1.05-1.12]	1.06 [0.91-1.23]	1.07 [1.01-1.13]	1.05 [1.01-1.09]	9,997	9,657	395	3,308	8,356
656.2	1.08 [0.96-1.21]	1.11 [0.98-1.25]	1.10 [0.97-1.25]		1.03 [0.92-1.16]	1.04 [0.93-1.17]	1.06 [0.94-1.20]	1.04 [0.92-1.19]		1.04 [0.92-1.17]	782	726	692	NA	740
656.7	1.02 [0.87-1.20]			1.04 [0.89-1.22]	1.00 [0.84-1.20]	1.02 [0.87-1.20]			1.02 [0.87-1.20]	1.01 [0.84-1.21]	366	NA	NA	365	298
661	1.03 [1.01-1.05]	1.05 [1.03-1.07]	0.89 [0.80-0.99]	0.99 [0.96-1.04]	1.02 [0.99-1.04]	1.02 [1.00-1.04]	1.04 [1.02-1.06]	0.93 [0.83-1.05]	0.98 [0.94-1.03]	1.01 [0.98-1.04]	21,601	21,334	668	6,145	14,205
663	1.09 [1.03-1.15]	1.10 [1.04-1.16]	1.18 [0.93-1.49]	0.99 [0.88-1.11]	1.03 [0.97-1.10]	1.08 [1.02-1.14]	1.10 [1.04-1.16]	1.22 [0.95-1.56]	0.99 [0.88-1.12]	1.04 [0.98-1.11]	3,144	3,121	152	714	2,453
665	1.04 [1.02-1.05]	1.06 [1.05-1.08]	0.94 [0.86-1.02]	0.97 [0.95-1.00]	0.98 [0.96-1.00]	1.04 [1.02-1.05]	1.06 [1.04-1.07]	1.01 [0.93-1.11]	0.98 [0.95-1.00]	0.99 [0.97-1.01]	49,808	48,741	1,227	15,768	34,052
668	1.20 [1.06-1.37]	1.21 [1.06-1.37]		0.91 [0.71-1.17]	1.14 [0.99-1.31]	1.16 [1.02-1.32]	1.16 [1.02-1.32]		0.89 [0.69-1.15]	1.12 [0.97-1.28]	626	615	NA	157	500
669	1.05 [1.02-1.07]	1.07 [1.05-1.09]	0.96 [0.87-1.06]	1.01 [0.97-1.05]	1.01 [0.99-1.04]	1.04 [1.02-1.06]	1.05 [1.03-1.08]	1.00 [0.90-1.11]	1.01 [0.97-1.05]	1.01 [0.99-1.04]	24,245	23,891	905	6,935	16,759
671	1.12 [1.05-1.20]	1.15 [1.08-1.23]	1.10 [0.87-1.39]	0.97 [0.83-1.13]	1.04 [0.97-1.12]	1.09 [1.02-1.17]	1.10 [1.03-1.18]	1.05 [0.83-1.34]	0.93 [0.80-1.09]	1.03 [0.96-1.11]	2,196	2,187	153	441	1,872
674	1.19 [1.14-1.25]	1.22 [1.17-1.27]	1.11 [0.94-1.31]	1.07 [0.98-1.17]	1.13 [1.07-1.18]	1.15 [1.10-1.20]	1.15 [1.10-1.21]	1.08 [0.91-1.29]	1.05 [0.96-1.15]	1.09 [1.04-1.15]	5,402	5,356	301	1,445	4,289
676	1.10 [0.97-1.24]	1.11 [0.99-1.26]		0.95 [0.73-1.24]	1.04 [0.90-1.19]	1.06 [0.93-1.21]	1.07 [0.94-1.22]		0.90 [0.69-1.19]	1.02 [0.89-1.18]	632	626	NA	145	487
dermatologic															
681	1.61 [1.58-1.63]	1.65 [1.62-1.67]	1.64 [1.61-1.66]	1.38 [1.32-1.44]	1.49 [1.47-1.52]	1.53 [1.50-1.55]	1.50 [1.48-1.53]	1.49 [1.47-1.52]	1.34 [1.28-1.40]	1.44 [1.42-1.46]	45,803	41,863	37,950	5,186	40,017
681.1	1.31 [1.23-1.39]	1.40 [1.31-1.50]	1.37 [1.27-1.47]	1.17 [1.05-1.30]	1.24 [1.16-1.32]	1.25 [1.18-1.33]	1.27 [1.19-1.37]	1.23 [1.14-1.33]	1.15 [1.03-1.28]	1.20 [1.12-1.28]	2,930	2,174	1,782	913	2,545
681.2	1.36 [1.28-1.44]	1.48 [1.38-1.59]	1.55 [1.43-1.69]	1.16 [1.06-1.28]	1.30 [1.22-1.38]	1.32 [1.24-1.40]	1.37 [1.28-1.48]	1.45 [1.33-1.58]	1.16 [1.05-1.28]	1.29 [1.20-1.37]	2,886	1,939	1,415	1,128	2,437
681.3	1.64 [1.61-1.67]	1.66 [1.63-1.69]	1.66 [1.63-1.69]	1.42 [1.34-1.52]	1.52 [1.49-1.54]	1.56 [1.53-1.58]	1.52 [1.49-1.55]	1.51 [1.48-1.54]	1.38 [1.29-1.47]	1.46 [1.44-1.49]	37,614	35,648	32,976	2,784	33,044
681.5	1.64 [1.61-1.67]	1.66 [1.63-1.69]	1.66 [1.63-1.69]	1.42 [1.34-1.52]	1.52 [1.49-1.54]	1.56 [1.53-1.58]	1.52 [1.49-1.55]	1.51 [1.48-1.54]	1.38 [1.29-1.47]	1.46 [1.44-1.49]	37,614	35,648	32,976	2,784	33,044
681.6	1.64 [1.61-1.67]	1.66 [1.63-1.69]	1.66 [1.63-1.69]	1.42 [1.34-1.52]	1.52 [1.49-1.54]	1.56 [1.53-1.58]	1.52 [1.49-1.55]	1.51 [1.48-1.54]	1.38 [1.29-1.47]	1.46 [1.44-1.49]	37,614	35,648	32,976	2,784	33,044
681.7	1.53 [1.45-1.62]	1.55 [1.47-1.65]	1.54 [1.45-1.65]	1.31 [1.13-1.53]	1.41 [1.33-1.50]	1.43 [1.35-1.51]	1.37 [1.29-1.46]	1.37 [1.28-1.46]	1.25 [1.07-1.46]	1.36 [1.28-1.44]	3,265	2,986	2,431	454	2,937
686	1.55 [1.50-1.60]	1.52 [1.47-1.57]	1.52 [1.47-1.57]	1.71 [1.58-1.84]	1.46 [1.42-1.51]	1.48 [1.44-1.53]	1.39 [1.35-1.44]	1.38 [1.33-1.43]	1.67 [1.54-1.80]	1.43 [1.38-1.48]	12,334	10,798	9,706	1,884	11,127
686.1	1.37 [1.34-1.41]	1.38 [1.34-1.42]	1.37 [1.32-1.43]	1.29 [1.22-1.36]	1.26 [1.22-1.30]	1.30 [1.26-1.34]	1.23 [1.19-1.27]	1.23 [1.18-1.28]	1.26 [1.19-1.33]	1.23 [1.19-1.27]	12,261	9,906	6,135	3,705	9,961
686.2	2.28 [2.08-2.51]	2.65 [2.21-3.17]	2.27 [1.83-2.82]	2.22 [2.00-2.48]	2.16 [1.95-2.39]	2.10 [1.91-2.32]	2.19 [1.81-2.65]	1.96 [1.57-2.46]	2.09 [1.87-2.33]	2.01 [1.81-2.23]	1,345	383	245	1,019	1,124
686.3	1.15 [1.09-1.21]	1.17 [1.11-1.24]	1.30 [1.15-1.48]	1.09 [1.02-1.17]	1.09 [1.02-1.16]	1.11 [1.05-1.17]	1.08 [1.02-1.14]	1.17 [1.03-1.34]	1.06 [0.98-1.14]	1.09 [1.02-1.16]	3,813	3,135	557	2,154	2,607
686.4	1.30 [1.18-1.43]	1.32 [1.18-1.49]	1.29 [1.12-1.48]	1.26 [1.08-1.47]	1.25 [1.13-1.39]	1.24 [1.12-1.36]	1.21 [1.08-1.37]	1.20 [1.04-1.39]	1.22 [1.05-1.42]	1.19 [1.07-1.33]	1,099	710	487	470	912
686.5	1.84 [1.54-2.21]	1.90 [1.56-2.30]	1.85 [1.49-2.30]		1.58 [1.31-1.91]	1.71 [1.42-2.06]	1.66 [1.36-2.03]	1.65 [1.32-2.07]		1.52 [1.26-1.84]	344	293	228	NA	306
687	1.25 [1.20-1.29]	1.33 [1.26-1.40]	1.33 [1.26-1.41]	1.16 [1.10-1.22]	1.18 [1.14-1.23]	1.19 [1.14-1.24]	1.21 [1.15-1.28]	1.21 [1.14-1.28]	1.12 [1.06-1.18]	1.14 [1.10-1.19]	7,127	3,782	3,302	3,510	6,405
687.1	1.47 [1.43-1.50]	1.68 [1.64-1.73]	1.76 [1.70-1.81]	1.18 [1.14-1.22]	1.38 [1.35-1.41]	1.39 [1.36-1.42]	1.53 [1.49-1.58]	1.61 [1.56-1.66]	1.14 [1.10-1.18]	1.33 [1.30-1.36]	21,832	13,595	10,995	9,151	19,112
687.2	1.38 [1.34-1.43]	1.37 [1.31-1.42]	1.36 [1.31-1.42]	1.42 [1.33-1.52]	1.31 [1.27-1.36]	1.29 [1.25-1.34]	1.22 [1.17-1.27]	1.23 [1.18-1.29]	1.36 [1.27-1.45]	1.25 [1.20-1.29]	8,990	6,898	5,433	2,598	7,743
687.3	1.60 [1.38-1.85]	1.59 [1.35-1.87]	1.46 [1.22-1.74]	1.44 [1.04-2.00]	1.48 [1.26-1.73]	1.53 [1.31-1.78]	1.44 [1.21-1.70]	1.31 [1.09-1.58]	1.42 [1.02-1.99]	1.42 [1.21-1.66]	485	395	330	101	424
687.4	1.27 [1.23-1.30]	1.27 [1.23-1.31]	1.27 [1.23-1.32]	1.15 [1.06-1.24]	1.16 [1.13-1.20]	1.16 [1.12-1.20]	1.11 [1.07-1.14]	1.11 [1.07-1.15]	1.09 [1.00-1.18]	1.10 [1.06-1.13]	10,595	9,813	7,028	1,678	9,380
689	1.54 [1.51-1.58]	1.49 [1.46-1.53]	1.47 [1.43-1.51]	1.88 [1.78-2.00]	1.44 [1.40-1.47]	1.46 [1.43-1.49]	1.38 [1.34-1.41]	1.36 [1.32-1.39]	1.79 [1.68-1.90]	1.38 [1.35-1.42]	21,581	18,840	16,230	3,546	18,853
690	27.42 [23.35-32.19]	19.22 [15.25-24.22]	13.15 [10.11-17.10]	32.12 [26.19-39.39]	24.24 [20.43-28.76]	25.91 [22.01-30.51]	16.97 [13.43-21.46]	12.33 [9.45-16.10]	29.95 [24.37-36.80]	22.62 [19.02-26.91]	2,347	781	453	1,711	1,917
690.1	3.29 [2.87-3.77]	3.13 [2.71-3.62]	3.22 [2.77-3.75]	3.22 [2.24-4.64]	2.86 [2.48-3.29]	3.09 [2.69-3.56]	2.85 [2.45-3.30]	2.91 [2.49-3.40]	3.12 [2.12-4.57]	2.72 [2.36-3.15]	776	682	616	114	678
691	1.49 [1.27-1.76]	1.85 [1.48-2.30]	1.99 [1.56-2.54]	1.24 [0.97-1.58]	1.44 [1.20-1.71]	1.39 [1.17-1.64]	1.71 [1.36-2.13]	1.74 [1.36-2.24]	1.18 [0.92-1.51]	1.34 [1.12-1.60]	380	223	183	168	325
691.1	2.79 [1.97-3.95]				2.74 [1.90-3.95]	2.58 [1.81-3.68]				2.50 [1.71-3.64]	119	NA	NA	NA	105
694.1	1.97 [1.69-2.29]	1.85 [1.57-2.18]	1.90 [1.59-2.27]	2.46 [1.72-3.53]	1.83 [1.56-2.15]	1.80 [1.54-2.11]	1.65 [1.40-1.96]	1.67 [1.39-2.01]	2.34 [1.60-3.42]	1.73 [1.46-2.04]	492	410	343	106	427
694.2	1.29 [1.20-1.39]	1.29 [1.19-1.39]	1.27 [1.17-1.38]	1.50 [1.26-1.78]	1.24 [1.15-1.34]	1.23 [1.14-1.32]	1.20 [1.11-1.30]	1.19 [1.10-1.30]	1.45 [1.21-1.72]	1.19 [1.10-1.29]	1,910	1,583	1,347	395	1,633
694.3	1.62 [1.43-1.84]	1.79 [1.56-2.04]	1.70 [1.47-1.96]		1.51 [1.32-1.73]	1.51 [1.32-1.72]	1.64 [1.43-1.88]	1.53 [1.32-1.77]		1.42 [1.24-1.63]	660	603	524	NA	569
695.1	1.32 [1.19-1.47]	1.81 [1.56-2.10]	2.03 [1.71-2.39]	0.99 [0.86-1.15]	1.27 [1.13-1.42]	1.26 [1.13-1.40]	1.62 [1.39-1.89]	1.81 [1.52-2.15]	0.98 [0.84-1.13]	1.21 [1.08-1.36]	896	483	390	442	760
695.21	3.51 [2.89-4.26]	3.96 [3.22-4.86]	3.58 [2.87-4.48]		3.07 [2.49-3.78]	3.29 [2.70-4.02]	3.61 [2.93-4.46]	3.32 [2.64-4.17]		2.92 [2.36-3.61]	375	341	282	NA	311
695.22	2.81 [2.55-3.09]	2.76 [2.50-3.03]	2.76 [2.51-3.04]		2.57 [2.32-2.84]	2.69 [2.44-2.96]	2.59 [2.35-2.85]	2.58 [2.34-2.84]		2.48 [2.24-2.75]	1,545	1,526	1,504	NA	1,375
695.3	1.55 [1.39-1.72]	1.59 [1.43-1.78]	1.52 [1.36-1.70]		1.50 [1.34-1.68]	1.44 [1.29-1.61]	1.49 [1.33-1.66]	1.40 [1.25-1.57]		1.43 [1.27-1.60]	890	873	798	NA	784
695.41	1.86 [1.66-2.10]	1.86 [1.66-2.10]	1.84 [1.62-2.10]		1.68 [1.49-1.90]	1.67 [1.48-1.88]	1.61 [1.42-1.82]	1.58 [1.38-1.81]		1.55 [1.36-1.75]	786	768	620	NA	700
695.42	1.79 [1.65-1.95]	1.75 [1.61-1.90]	1.69 [1.54-1.86]	1.73 [1.30-2.30]	1.62 [1.49-1.76]	1.63 [1.49-1.77]	1.52 [1.39-1.65]	1.48 [1.34-1.63]	1.73 [1.29-2.32]	1.49 [1.36-1.63]	1,578	1,517	1,106	150	1,379
695.7	3.00 [2.81-3.19]	2.88 [2.70-3.07]	2.79 [2.60-2.98]	5.26 [3.96-7.00]	2.65 [2.48-2.84]	2.79 [2.61-2.98]	2.64 [2.47-2.82]	2.55 [2.38-2.73]	4.75 [3.54-6.38]	2.51 [2.34-2.69]	3,243	3,039	2,718	259	2,749
695.8	1.82 [1.66-1.98]	1.88 [1.70-2.08]	1.74 [1.57-1.93]	1.63 [1.36-1.94]	1.67 [1.52-1.83]	1.72 [1.57-1.88]	1.67 [1.51-1.85]	1.56 [1.40-1.74]	1.59 [1.33-1.90]	1.59 [1.45-1.75]	1,463	1,166	1,027	344	1,272
695.81	1.49 [1.25-1.79]	1.50 [1.22-1.84]	1.49 [1.12-1.96]	1.47 [1.11-1.96]	1.36 [1.12-1.66]	1.38 [1.15-1.66]	1.33 [1.07-1.64]	1.28 [0.96-1.71]	1.36 [1.01-1.82]	1.24 [1.01-1.52]	329	233	127	139	264
695.9	1.78 [1.69-1.87]	1.85 [1.75-1.96]	1.88 [1.77-1.99]	1.45 [1.29-1.64]	1.66 [1.57-1.75]	1.68 [1.60-1.78]	1.70 [1.60-1.80]	1.71 [1.61-1.82]	1.37 [1.20-1.55]	1.61 [1.52-1.70]	4,146	3,600	3,135	714	3,688
696.3	1.80 [1.46-2.23]	1.94 [1.54-2.44]	2.09 [1.59-2.74]		1.69 [1.34-2.13]	1.70 [1.37-2.11]	1.74 [1.37-2.21]	1.92 [1.45-2.54]		1.60 [1.27-2.03]	250	211	159	NA	208
696.4	2.23 [2.17-2.30]	2.19 [2.13-2.26]	2.21 [2.14-2.28]	2.04 [1.84-2.26]	2.11 [2.04-2.17]	2.13 [2.07-2.20]	2.02 [1.96-2.08]	2.03 [1.97-2.10]	2.01 [1.80-2.23]	2.04 [1.98-2.11]	14,006	13,506	11,116	1,195	11,883
696.41	2.30 [2.23-2.37]	2.27 [2.20-2.34]	2.28 [2.20-2.36]	2.11 [1.90-2.35]	2.17 [2.10-2.24]	2.20 [2.13-2.27]	2.08 [2.02-2.15]	2.09 [2.02-2.17]	2.08 [1.86-2.33]	2.11 [2.04-2.18]	12,400	11,939	9,842	1,114	10,571
696.42	1.73 [1.62-1.85]	1.69 [1.58-1.81]	1.70 [1.58-1.83]	1.37 [1.00-1.86]	1.59 [1.48-1.71]	1.62 [1.51-1.74]	1.52 [1.42-1.63]	1.53 [1.42-1.65]	1.33 [0.97-1.82]	1.52 [1.41-1.63]	2,322	2,269	1,879	116	1,993
697	1.57 [1.46-1.70]	1.55 [1.43-1.67]	1.51 [1.39-1.64]	1.78 [1.27-2.51]	1.43 [1.31-1.55]	1.44 [1.33-1.56]	1.39 [1.29-1.51]	1.34 [1.23-1.46]	1.67 [1.17-2.37]	1.32 [1.22-1.44]	1,717	1,649	1,425	102	1,485
698	1.72 [1.64-1.80]	1.68 [1.60-1.76]	1.99 [1.87-2.12]	1.46 [1.31-1.62]	1.59 [1.51-1.67]	1.60 [1.52-1.68]	1.50 [1.42-1.58]	1.78 [1.67-1.90]	1.36 [1.21-1.51]	1.50 [1.42-1.58]	4,866	4,324	2,764	1,002	4,307
700	1.49 [1.31-1.69]	1.47 [1.29-1.67]	1.50 [1.31-1.72]		1.32 [1.16-1.51]	1.39 [1.22-1.58]	1.33 [1.16-1.51]	1.35 [1.18-1.55]		1.26 [1.10-1.44]	634	609	550	NA	566
701	1.36 [1.31-1.40]	1.38 [1.34-1.43]	1.38 [1.33-1.43]	1.18 [1.10-1.28]	1.27 [1.22-1.31]	1.28 [1.24-1.33]	1.28 [1.24-1.33]	1.27 [1.23-1.32]	1.17 [1.09-1.27]	1.22 [1.18-1.26]	10,113	8,842	6,975	1,829	8,668
701.1	2.28 [1.79-2.90]	2.04 [1.60-2.62]	2.21 [1.71-2.87]		2.22 [1.73-2.86]	2.14 [1.68-2.74]	1.87 [1.45-2.41]	2.07 [1.58-2.70]		2.13 [1.65-2.75]	204	183	169	NA	184
701.2	1.19 [1.14-1.25]	1.24 [1.18-1.31]	1.20 [1.13-1.27]	1.00 [0.90-1.12]	1.12 [1.07-1.18]	1.13 [1.08-1.18]	1.15 [1.10-1.21]	1.11 [1.05-1.18]	0.99 [0.88-1.11]	1.09 [1.04-1.15]	4,378	3,873	2,861	789	3,863
701.3	1.46 [1.18-1.81]	1.50 [1.19-1.89]	1.69 [1.31-2.16]		1.33 [1.06-1.67]	1.37 [1.10-1.70]	1.38 [1.08-1.75]	1.50 [1.16-1.94]		1.27 [1.00-1.60]	221	182	166	NA	186
701.4	1.29 [1.17-1.42]	1.31 [1.16-1.47]	1.33 [1.11-1.58]	1.27 [1.11-1.46]	1.19 [1.06-1.32]	1.22 [1.10-1.35]	1.22 [1.08-1.38]	1.22 [1.02-1.47]	1.26 [1.10-1.45]	1.14 [1.02-1.27]	1,088	689	306	579	854
701.5	1.29 [1.20-1.39]	1.39 [1.27-1.51]	1.37 [1.24-1.51]	1.10 [0.96-1.27]	1.20 [1.11-1.30]	1.21 [1.12-1.30]	1.25 [1.15-1.36]	1.24 [1.12-1.37]	1.04 [0.90-1.19]	1.15 [1.06-1.25]	1,791	1,376	1,000	545	1,521
701.6	1.67 [1.43-1.96]	1.82 [1.51-2.18]	1.71 [1.41-2.08]	1.58 [1.18-2.12]	1.55 [1.31-1.83]	1.56 [1.33-1.84]	1.60 [1.32-1.93]	1.56 [1.27-1.90]	1.51 [1.11-2.04]	1.46 [1.23-1.74]	456	322	278	150	387
702.1	1.37 [1.32-1.42]	1.35 [1.30-1.40]	1.37 [1.32-1.41]		1.29 [1.24-1.34]	1.30 [1.25-1.34]	1.26 [1.22-1.31]	1.27 [1.22-1.32]		1.23 [1.18-1.28]	8,337	8,281	8,115	NA	7,420
702.2	1.22 [1.17-1.28]	1.25 [1.19-1.31]	1.26 [1.20-1.31]		1.18 [1.13-1.24]	1.16 [1.11-1.21]	1.17 [1.12-1.22]	1.17 [1.12-1.23]		1.13 [1.07-1.18]	4,915	4,889	4,688	NA	4,220
703	1.42 [1.34-1.49]	1.48 [1.39-1.58]	1.51 [1.39-1.63]	1.32 [1.22-1.43]	1.33 [1.25-1.41]	1.30 [1.23-1.38]	1.33 [1.24-1.42]	1.32 [1.22-1.43]	1.24 [1.14-1.34]	1.24 [1.17-1.32]	3,705	2,360	1,554	1,741	2,910
703.1	1.42 [1.34-1.51]	1.52 [1.41-1.64]	1.58 [1.43-1.75]	1.34 [1.24-1.46]	1.36 [1.27-1.46]	1.31 [1.23-1.39]	1.36 [1.25-1.47]	1.37 [1.23-1.52]	1.26 [1.16-1.37]	1.27 [1.19-1.37]	2,927	1,683	991	1,606	2,260
704	1.17 [1.12-1.21]	1.17 [1.13-1.22]	1.16 [1.11-1.21]	1.13 [1.02-1.25]	1.10 [1.05-1.14]	1.12 [1.08-1.17]	1.10 [1.06-1.14]	1.09 [1.04-1.14]	1.08 [0.97-1.20]	1.07 [1.03-1.12]	6,656	6,255	4,344	951	5,126
704.1	1.96 [1.76-2.19]	2.02 [1.79-2.27]	2.00 [1.74-2.30]	1.84 [1.49-2.28]	1.86 [1.66-2.08]	1.75 [1.56-1.95]	1.73 [1.53-1.96]	1.72 [1.49-1.98]	1.72 [1.38-2.15]	1.66 [1.47-1.87]	1,010	811	577	271	853
704.11	2.87 [2.32-3.56]	2.84 [2.24-3.59]	2.62 [1.97-3.48]		2.70 [2.15-3.40]	2.27 [1.80-2.85]	2.08 [1.61-2.68]	2.06 [1.53-2.79]		2.19 [1.71-2.79]	290	234	151	NA	243
704.2	1.25 [1.00-1.56]	1.28 [1.00-1.64]			1.10 [0.86-1.41]	1.19 [0.95-1.49]	1.12 [0.87-1.45]			1.05 [0.82-1.35]	211	157	NA	NA	167
704.8	1.40 [1.10-1.79]	1.35 [1.05-1.74]	1.41 [1.06-1.88]		1.29 [1.00-1.67]	1.31 [1.02-1.68]	1.20 [0.92-1.56]	1.30 [0.97-1.74]		1.22 [0.94-1.59]	169	152	114	NA	148
705.1	5.39 [4.03-7.21]	4.31 [3.17-5.86]	3.59 [2.55-5.05]		5.00 [3.69-6.77]	5.05 [3.73-6.83]	3.75 [2.73-5.14]	3.11 [2.18-4.44]		4.73 [3.45-6.47]	228	171	132	NA	191
705.3	1.56 [1.40-1.72]	1.54 [1.39-1.71]	1.51 [1.28-1.77]	1.32 [1.07-1.63]	1.32 [1.19-1.47]	1.40 [1.26-1.56]	1.28 [1.14-1.42]	1.23 [1.04-1.46]	1.25 [1.01-1.56]	1.26 [1.12-1.40]	991	944	362	240	847
705.8	1.36 [1.27-1.45]	1.38 [1.29-1.48]	1.34 [1.24-1.45]	1.24 [1.07-1.45]	1.23 [1.15-1.32]	1.24 [1.16-1.32]	1.21 [1.13-1.30]	1.17 [1.08-1.27]	1.18 [1.01-1.37]	1.16 [1.09-1.25]	2,502	2,240	1,590	476	2,119
706.1	1.46 [1.31-1.63]	1.43 [1.26-1.62]	1.45 [1.15-1.84]	1.36 [1.17-1.58]	1.30 [1.16-1.47]	1.34 [1.19-1.50]	1.24 [1.09-1.41]	1.26 [0.99-1.62]	1.27 [1.09-1.49]	1.23 [1.09-1.40]	911	642	175	518	738
706.2	1.16 [1.13-1.20]	1.18 [1.14-1.21]	1.17 [1.13-1.22]	1.11 [1.04-1.20]	1.09 [1.06-1.13]	1.12 [1.08-1.15]	1.09 [1.06-1.13]	1.10 [1.06-1.14]	1.08 [1.00-1.17]	1.07 [1.03-1.10]	11,104	10,136	7,216	1,829	8,562
706.8	2.65 [1.99-3.52]	2.55 [1.85-3.52]	2.85 [2.04-3.98]		2.47 [1.86-3.29]	2.49 [1.86-3.34]	2.30 [1.65-3.19]	2.56 [1.81-3.60]		2.42 [1.81-3.24]	171	139	127	NA	165
707	1.54 [1.50-1.57]	1.53 [1.49-1.56]	1.52 [1.49-1.56]	1.28 [0.99-1.64]	1.45 [1.41-1.48]	1.48 [1.45-1.52]	1.42 [1.39-1.45]	1.41 [1.38-1.44]	1.28 [0.99-1.66]	1.42 [1.39-1.46]	22,548	22,452	22,090	165	20,699
707.1	1.22 [1.20-1.25]	1.21 [1.19-1.24]	1.22 [1.19-1.24]	0.99 [0.82-1.20]	1.18 [1.16-1.21]	1.20 [1.17-1.22]	1.15 [1.13-1.18]	1.15 [1.13-1.17]	0.97 [0.80-1.18]	1.18 [1.16-1.21]	26,249	26,064	25,754	284	24,650
709.2	1.66 [1.54-1.80]	1.69 [1.56-1.84]	1.67 [1.54-1.82]		1.51 [1.39-1.64]	1.48 [1.36-1.61]	1.46 [1.34-1.59]	1.43 [1.31-1.56]		1.35 [1.24-1.47]	1,608	1,600	1,483	NA	1,444
709.3	1.73 [1.52-1.98]	1.64 [1.44-1.87]	1.65 [1.44-1.89]		1.54 [1.34-1.77]	1.61 [1.40-1.84]	1.48 [1.30-1.70]	1.49 [1.29-1.71]		1.45 [1.26-1.67]	611	591	535	NA	530
709.4	1.53 [1.19-1.96]	1.52 [1.18-1.94]	1.44 [1.11-1.88]		1.46 [1.11-1.91]	1.47 [1.14-1.90]	1.41 [1.09-1.82]	1.36 [1.04-1.79]		1.39 [1.05-1.83]	164	162	140	NA	135
709.5	2.10 [1.78-2.47]	2.12 [1.78-2.52]	2.04 [1.69-2.45]		1.92 [1.60-2.31]	2.03 [1.71-2.40]	2.00 [1.67-2.39]	1.94 [1.61-2.35]		1.84 [1.52-2.22]	412	363	315	NA	322
709.6	1.79 [1.47-2.18]	1.77 [1.45-2.17]	1.78 [1.42-2.22]		1.51 [1.24-1.85]	1.60 [1.31-1.96]	1.56 [1.26-1.92]	1.58 [1.25-1.98]		1.38 [1.12-1.69]	290	267	211	NA	259
709.7	1.40 [1.34-1.47]	1.39 [1.33-1.46]	1.41 [1.34-1.49]	1.29 [1.14-1.45]	1.29 [1.23-1.35]	1.28 [1.23-1.35]	1.23 [1.17-1.30]	1.24 [1.18-1.31]	1.26 [1.11-1.43]	1.21 [1.15-1.27]	4,677	4,045	3,723	689	4,076
938	1.97 [1.65-2.36]	1.97 [1.63-2.37]	1.97 [1.62-2.41]		1.64 [1.35-1.98]	1.87 [1.55-2.24]	1.84 [1.52-2.22]	1.82 [1.49-2.24]		1.61 [1.32-1.97]	350	320	279	NA	290
938.1	1.48 [1.15-1.90]	1.69 [1.25-2.30]			1.50 [1.15-1.97]	1.43 [1.11-1.84]	1.60 [1.17-2.20]			1.45 [1.10-1.91]	171	111	NA	NA	150
938.2	1.32 [1.27-1.37]	1.31 [1.26-1.35]	1.32 [1.28-1.37]		1.25 [1.20-1.30]	1.25 [1.21-1.30]	1.22 [1.18-1.27]	1.24 [1.19-1.28]		1.20 [1.15-1.24]	7,794	7,772	7,653	NA	6,959
939	7.81 [7.68-7.95]	6.82 [6.67-6.96]	6.01 [5.86-6.15]	9.08 [8.86-9.31]	6.95 [6.83-7.07]	7.23 [7.10-7.35]	6.12 [5.98-6.25]	5.50 [5.36-5.64]	8.34 [8.13-8.56]	6.48 [6.36-6.60]	84,723	46,492	32,628	43,598	71,715
947	1.90 [1.81-1.99]	1.77 [1.66-1.88]	1.81 [1.69-1.95]	2.04 [1.91-2.18]	1.83 [1.74-1.92]	1.64 [1.56-1.72]	1.46 [1.37-1.55]	1.50 [1.39-1.61]	1.77 [1.65-1.89]	1.58 [1.50-1.66]	5,321	2,835	2,084	2,772	4,679

Supplementary Table 6: phecode-mapped results (hazard ratios and events from all cohorts)

	Hazard ratio (99% confidence interval)														
	crude					adjusted					Events (in exposed)				
Outcome	any age	18+	40+	<18	hosp.	any age	18+	40+	<18	hosp.	any age	18+	40+	<18	hosp.
musculoskeletal															
710	1.49 [1.15-1.92]	1.61 [1.24-2.08]	1.48 [1.14-1.93]		1.57 [1.19-2.06]	1.50 [1.16-1.94]	1.56 [1.20-2.03]	1.41 [1.08-1.84]		1.61 [1.22-2.12]	154	151	143	NA	135
710.11	1.35 [1.12-1.62]	1.35 [1.10-1.66]	1.39 [1.12-1.73]		1.34 [1.10-1.62]	1.31 [1.09-1.58]	1.24 [1.00-1.53]	1.27 [1.02-1.58]		1.33 [1.10-1.62]	309	231	214	NA	278
710.12	1.39 [1.25-1.54]	1.43 [1.28-1.59]	1.45 [1.29-1.64]	1.27 [0.98-1.65]	1.26 [1.13-1.40]	1.32 [1.19-1.46]	1.29 [1.15-1.44]	1.32 [1.17-1.49]	1.24 [0.95-1.62]	1.22 [1.10-1.36]	953	825	735	160	855
710.19	1.37 [1.31-1.45]	1.39 [1.32-1.47]	1.38 [1.30-1.46]	1.37 [1.20-1.56]	1.28 [1.22-1.35]	1.32 [1.25-1.39]	1.25 [1.19-1.33]	1.23 [1.16-1.31]	1.34 [1.17-1.54]	1.26 [1.20-1.33]	4,040	3,496	3,260	622	3,674
711	1.36 [1.11-1.66]	1.30 [1.04-1.63]	1.36 [1.07-1.73]		1.29 [1.05-1.59]	1.23 [1.00-1.51]	1.12 [0.88-1.41]	1.16 [0.90-1.49]		1.22 [0.98-1.51]	254	196	169	NA	227
711.1	1.48 [1.39-1.59]	1.50 [1.39-1.61]	1.54 [1.42-1.66]	1.40 [1.20-1.64]	1.35 [1.26-1.45]	1.41 [1.32-1.51]	1.37 [1.27-1.48]	1.41 [1.30-1.52]	1.37 [1.16-1.61]	1.31 [1.22-1.41]	2,261	1,904	1,742	421	1,990
711.2	1.24 [0.92-1.67]					1.14 [0.84-1.54]					111	NA	NA	NA	NA
711.3	1.31 [0.98-1.76]	1.29 [0.96-1.74]				1.22 [0.90-1.65]	1.14 [0.83-1.55]				119	108	NA	NA	NA
712	1.38 [1.21-1.57]	1.36 [1.18-1.56]	1.31 [1.13-1.53]	1.47 [1.08-1.98]	1.29 [1.12-1.48]	1.34 [1.17-1.53]	1.28 [1.10-1.48]	1.25 [1.07-1.46]	1.42 [1.03-1.96]	1.29 [1.12-1.49]	597	491	420	123	525
713	1.25 [1.11-1.40]	1.40 [1.21-1.64]	1.31 [1.10-1.56]	1.13 [0.95-1.34]	1.18 [1.04-1.34]	1.20 [1.07-1.35]	1.29 [1.10-1.51]	1.21 [1.01-1.45]	1.11 [0.94-1.32]	1.15 [1.02-1.31]	735	431	309	347	629
713.5	1.69 [1.46-1.96]	1.70 [1.46-1.97]	1.67 [1.44-1.94]		1.42 [1.22-1.64]	1.52 [1.31-1.77]	1.39 [1.19-1.63]	1.34 [1.14-1.57]		1.34 [1.15-1.55]	468	468	452	NA	443
714	1.46 [1.36-1.56]	1.43 [1.34-1.53]	1.45 [1.35-1.56]		1.32 [1.23-1.42]	1.32 [1.23-1.41]	1.25 [1.16-1.34]	1.27 [1.18-1.36]		1.23 [1.15-1.32]	2,226	2,188	2,056	NA	2,038
714.1	1.41 [1.37-1.44]	1.40 [1.37-1.43]	1.39 [1.36-1.43]	1.28 [1.05-1.57]	1.31 [1.28-1.34]	1.32 [1.28-1.35]	1.27 [1.24-1.30]	1.26 [1.23-1.29]	1.22 [1.00-1.50]	1.24 [1.21-1.27]	17,617	17,574	16,389	279	15,331
714.2	1.33 [1.19-1.48]	1.38 [1.09-1.73]		1.34 [1.19-1.51]	1.25 [1.10-1.41]	1.28 [1.14-1.43]	1.26 [1.00-1.60]		1.30 [1.15-1.47]	1.23 [1.08-1.39]	852	183	NA	723	706
715	1.38 [1.33-1.43]	1.37 [1.32-1.43]	1.38 [1.32-1.44]	1.08 [0.83-1.41]	1.27 [1.22-1.32]	1.25 [1.20-1.31]	1.20 [1.15-1.25]	1.20 [1.15-1.25]	1.09 [0.83-1.42]	1.19 [1.15-1.24]	6,405	6,314	5,957	153	5,911
715.1	1.43 [1.24-1.64]	1.44 [1.25-1.66]	1.43 [1.22-1.67]		1.28 [1.12-1.48]	1.26 [1.09-1.45]	1.21 [1.04-1.40]	1.17 [0.99-1.37]		1.19 [1.03-1.37]	537	510	400	NA	502
715.2	1.50 [1.39-1.62]	1.56 [1.45-1.68]	1.48 [1.37-1.61]		1.42 [1.32-1.54]	1.39 [1.29-1.50]	1.39 [1.28-1.50]	1.31 [1.20-1.42]		1.33 [1.23-1.45]	1,810	1,796	1,543	NA	1,588
716	1.48 [1.31-1.68]	1.49 [1.31-1.70]	1.54 [1.34-1.78]		1.34 [1.18-1.53]	1.33 [1.17-1.51]	1.25 [1.08-1.43]	1.31 [1.13-1.52]		1.26 [1.10-1.43]	648	570	502	NA	583
716.1	1.37 [1.34-1.41]	1.37 [1.34-1.41]	1.36 [1.33-1.40]		1.27 [1.24-1.30]	1.26 [1.23-1.29]	1.22 [1.19-1.25]	1.21 [1.18-1.24]		1.19 [1.16-1.22]	18,108	18,032	17,859	NA	16,872
716.2	1.25 [1.24-1.27]	1.25 [1.24-1.27]	1.25 [1.23-1.27]	1.17 [1.00-1.37]	1.17 [1.15-1.19]	1.18 [1.16-1.20]	1.16 [1.14-1.18]	1.15 [1.14-1.17]	1.12 [0.95-1.33]	1.12 [1.10-1.14]	45,851	45,760	44,156	393	39,506
716.8	1.65 [1.26-2.15]	1.56 [1.19-2.05]	1.48 [1.11-1.97]		1.43 [1.09-1.88]	1.52 [1.16-2.01]	1.37 [1.03-1.81]	1.27 [0.94-1.72]		1.37 [1.04-1.81]	143	140	115	NA	127
716.9	1.32 [1.31-1.33]	1.32 [1.30-1.33]	1.31 [1.30-1.33]	1.24 [1.14-1.35]	1.23 [1.22-1.24]	1.23 [1.21-1.24]	1.19 [1.18-1.20]	1.18 [1.17-1.19]	1.16 [1.07-1.27]	1.16 [1.15-1.18]	121,759	121,279	116,846	1,514	106,193
717	1.38 [1.34-1.43]	1.39 [1.34-1.44]	1.38 [1.34-1.43]		1.32 [1.28-1.37]	1.30 [1.26-1.35]	1.29 [1.25-1.33]	1.28 [1.24-1.32]		1.26 [1.22-1.31]	9,733	9,731	9,658	NA	8,595
720	1.30 [1.27-1.34]	1.30 [1.27-1.34]	1.30 [1.26-1.33]	1.10 [0.82-1.48]	1.20 [1.17-1.24]	1.19 [1.16-1.22]	1.15 [1.11-1.18]	1.13 [1.10-1.16]	1.02 [0.75-1.38]	1.14 [1.11-1.17]	14,146	14,135	13,686	120	13,127
721	1.35 [1.33-1.38]	1.36 [1.34-1.38]	1.35 [1.32-1.37]	1.06 [0.84-1.34]	1.26 [1.23-1.28]	1.23 [1.21-1.26]	1.19 [1.17-1.21]	1.18 [1.16-1.20]	0.99 [0.78-1.27]	1.18 [1.16-1.20]	32,132	32,112	30,898	193	29,839
721.1	1.35 [1.32-1.38]	1.36 [1.33-1.39]	1.36 [1.33-1.39]	0.96 [0.72-1.29]	1.25 [1.23-1.28]	1.23 [1.20-1.26]	1.19 [1.16-1.22]	1.19 [1.16-1.22]	0.93 [0.69-1.27]	1.18 [1.15-1.20]	20,283	20,277	19,516	117	18,927
721.2	1.32 [1.18-1.49]	1.32 [1.17-1.49]	1.26 [1.12-1.42]		1.23 [1.09-1.39]	1.24 [1.10-1.39]	1.19 [1.05-1.34]	1.11 [0.98-1.25]		1.18 [1.04-1.33]	704	702	682	NA	661
721.8	1.27 [1.21-1.35]	1.26 [1.19-1.33]	1.26 [1.19-1.34]	1.12 [0.87-1.45]	1.19 [1.12-1.26]	1.19 [1.13-1.26]	1.14 [1.08-1.21]	1.15 [1.08-1.22]	1.03 [0.79-1.34]	1.13 [1.07-1.20]	3,522	3,420	3,251	165	3,312
722	1.30 [1.27-1.33]	1.29 [1.26-1.32]	1.31 [1.28-1.35]	1.18 [1.05-1.32]	1.19 [1.16-1.22]	1.18 [1.15-1.21]	1.12 [1.09-1.14]	1.13 [1.10-1.16]	1.11 [0.98-1.25]	1.12 [1.09-1.15]	16,846	16,747	13,505	801	15,012
722.1	1.36 [1.23-1.49]	1.33 [1.21-1.47]	1.36 [1.22-1.50]		1.22 [1.10-1.34]	1.23 [1.11-1.36]	1.13 [1.02-1.25]	1.14 [1.02-1.27]		1.13 [1.02-1.25]	1,051	1,049	910	NA	969
722.3	1.26 [0.99-1.59]	1.27 [1.00-1.61]	1.22 [0.93-1.61]		1.17 [0.92-1.49]	1.14 [0.89-1.46]	1.13 [0.88-1.45]	1.08 [0.81-1.43]		1.14 [0.89-1.46]	179	169	127	NA	164
722.6	1.35 [1.31-1.39]	1.34 [1.31-1.39]	1.34 [1.30-1.38]	1.48 [1.17-1.86]	1.24 [1.20-1.28]	1.22 [1.19-1.26]	1.17 [1.13-1.20]	1.16 [1.13-1.20]	1.40 [1.10-1.78]	1.16 [1.13-1.20]	11,334	11,306	10,383	207	10,584
722.7	1.33 [1.21-1.45]	1.35 [1.23-1.47]	1.29 [1.17-1.42]		1.25 [1.14-1.38]	1.24 [1.13-1.36]	1.19 [1.08-1.31]	1.14 [1.04-1.26]		1.21 [1.10-1.33]	1,179	1,175	1,087	NA	1,058
722.8	1.40 [1.09-1.80]	1.46 [1.13-1.88]	1.46 [1.12-1.90]		1.27 [0.99-1.63]	1.21 [0.93-1.58]	1.18 [0.90-1.54]	1.18 [0.89-1.55]		1.18 [0.91-1.52]	155	155	139	NA	150
722.9	1.30 [1.26-1.33]	1.28 [1.25-1.32]	1.30 [1.26-1.34]	1.13 [0.98-1.31]	1.19 [1.16-1.22]	1.19 [1.16-1.23]	1.12 [1.09-1.15]	1.13 [1.10-1.17]	1.07 [0.92-1.23]	1.13 [1.10-1.16]	13,980	13,913	11,719	508	12,608
723	1.29 [1.18-1.41]	1.31 [1.20-1.43]	1.27 [1.16-1.39]		1.21 [1.11-1.33]	1.21 [1.10-1.32]	1.16 [1.06-1.27]	1.12 [1.02-1.23]		1.17 [1.07-1.29]	1,259	1,248	1,137	NA	1,127
723.1	1.09 [0.98-1.21]	1.14 [1.00-1.30]	1.18 [1.01-1.39]	1.05 [0.89-1.23]	1.03 [0.92-1.15]	1.00 [0.90-1.12]	0.99 [0.87-1.14]	1.04 [0.88-1.23]	1.01 [0.86-1.20]	0.97 [0.86-1.09]	862	542	356	391	759
724.8	1.25 [1.04-1.49]	1.22 [1.02-1.47]	1.18 [0.97-1.42]		1.18 [0.98-1.42]	1.15 [0.95-1.39]	1.08 [0.89-1.31]	1.04 [0.86-1.27]		1.14 [0.95-1.38]	300	296	275	NA	282
724.9	1.26 [1.20-1.33]	1.26 [1.20-1.33]	1.26 [1.19-1.32]	1.02 [0.78-1.33]	1.19 [1.13-1.25]	1.18 [1.12-1.25]	1.14 [1.08-1.20]	1.14 [1.08-1.21]	0.94 [0.71-1.23]	1.14 [1.08-1.20]	4,121	4,031	3,820	148	3,835
726	1.33 [1.30-1.36]	1.33 [1.30-1.36]	1.33 [1.30-1.37]	1.17 [1.01-1.36]	1.21 [1.18-1.25]	1.21 [1.18-1.24]	1.18 [1.15-1.21]	1.18 [1.15-1.21]	1.12 [0.96-1.30]	1.14 [1.11-1.17]	17,418	17,298	15,635	462	15,207
726.1	1.29 [1.27-1.32]	1.30 [1.27-1.33]	1.29 [1.26-1.32]	1.14 [0.98-1.31]	1.19 [1.16-1.22]	1.19 [1.16-1.22]	1.17 [1.14-1.19]	1.16 [1.13-1.18]	1.05 [0.90-1.22]	1.12 [1.09-1.15]	19,439	19,293	17,857	489	17,313
726.2	1.42 [1.26-1.60]	1.40 [1.25-1.58]	1.43 [1.26-1.62]		1.26 [1.11-1.42]	1.27 [1.12-1.43]	1.22 [1.07-1.38]	1.22 [1.07-1.39]		1.16 [1.02-1.32]	685	685	612	NA	609
726.3	1.51 [1.41-1.62]	1.52 [1.41-1.63]	1.49 [1.38-1.60]	1.43 [1.09-1.88]	1.36 [1.26-1.46]	1.40 [1.30-1.50]	1.36 [1.26-1.46]	1.32 [1.22-1.43]	1.38 [1.04-1.84]	1.28 [1.19-1.38]	2,124	2,041	1,733	154	1,784
726.4	1.40 [1.14-1.72]	1.32 [1.08-1.62]	1.35 [1.09-1.67]		1.26 [1.02-1.55]	1.30 [1.06-1.60]	1.16 [0.94-1.42]	1.16 [0.93-1.45]		1.19 [0.96-1.48]	242	239	207	NA	220
727	1.32 [1.24-1.40]	1.33 [1.25-1.42]	1.32 [1.23-1.42]	1.19 [1.00-1.43]	1.19 [1.12-1.27]	1.21 [1.13-1.28]	1.19 [1.11-1.27]	1.17 [1.08-1.25]	1.09 [0.91-1.31]	1.12 [1.05-1.20]	2,592	2,437	1,881	326	2,241
727.1	1.35 [1.31-1.38]	1.35 [1.31-1.40]	1.35 [1.30-1.40]	1.24 [1.16-1.32]	1.25 [1.21-1.29]	1.24 [1.21-1.28]	1.21 [1.17-1.25]	1.20 [1.16-1.24]	1.21 [1.13-1.29]	1.18 [1.14-1.22]	11,691	9,598	8,155	2,518	10,204
727.2	1.51 [1.22-1.87]	1.47 [1.19-1.82]	1.46 [1.15-1.85]		1.28 [1.02-1.61]	1.42 [1.14-1.76]	1.31 [1.05-1.64]	1.32 [1.03-1.69]		1.23 [0.98-1.55]	224	216	171	NA	188
727.4	1.30 [1.25-1.36]	1.30 [1.24-1.36]	1.33 [1.26-1.40]	1.15 [1.01-1.30]	1.21 [1.15-1.27]	1.22 [1.16-1.28]	1.20 [1.14-1.26]	1.22 [1.15-1.29]	1.10 [0.97-1.26]	1.15 [1.09-1.21]	4,579	4,263	3,032	650	3,641
727.5	1.36 [1.27-1.46]	1.35 [1.26-1.45]	1.36 [1.26-1.46]		1.30 [1.21-1.40]	1.27 [1.18-1.36]	1.24 [1.15-1.33]	1.23 [1.14-1.32]		1.23 [1.14-1.32]	2,135	2,099	2,008	NA	1,917
727.6	1.23 [1.08-1.39]	1.24 [1.09-1.40]	1.21 [1.06-1.38]		1.10 [0.96-1.25]	1.16 [1.02-1.32]	1.16 [1.02-1.31]	1.11 [0.96-1.27]		1.05 [0.91-1.20]	605	596	521	NA	508
727.7	1.04 [0.93-1.17]	1.27 [1.10-1.48]	1.37 [1.15-1.62]	0.83 [0.71-0.98]	0.94 [0.84-1.06]	0.97 [0.86-1.09]	1.13 [0.97-1.32]	1.19 [1.00-1.42]	0.79 [0.67-0.93]	0.90 [0.80-1.02]	768	436	329	373	684
728	1.28 [1.16-1.41]	1.32 [1.19-1.46]	1.35 [1.22-1.51]		1.17 [1.06-1.30]	1.19 [1.08-1.32]	1.20 [1.08-1.33]	1.23 [1.10-1.38]		1.14 [1.03-1.26]	1,065	1,014	878	NA	978
728.2	1.42 [1.35-1.50]	1.44 [1.35-1.53]	1.43 [1.30-1.58]	1.38 [1.27-1.50]	1.30 [1.23-1.38]	1.26 [1.19-1.34]	1.20 [1.12-1.28]	1.20 [1.09-1.33]	1.28 [1.18-1.40]	1.19 [1.12-1.27]	3,422	2,351	986	1,653	2,994
728.7	1.12 [1.07-1.17]	1.12 [1.07-1.17]	1.12 [1.07-1.17]	1.23 [0.91-1.67]	1.06 [1.01-1.11]	1.08 [1.03-1.13]	1.07 [1.02-1.11]	1.07 [1.02-1.12]	1.18 [0.85-1.62]	1.04 [0.99-1.09]	5,021	4,955	4,699	114	4,137
728.71	1.03 [0.98-1.08]	1.03 [0.98-1.08]	1.04 [0.99-1.09]		0.98 [0.92-1.03]	1.01 [0.96-1.07]	1.01 [0.96-1.07]	1.02 [0.97-1.08]		0.97 [0.92-1.03]	3,475	3,470	3,427	NA	2,770
729	1.40 [1.38-1.43]	1.41 [1.38-1.44]	1.42 [1.39-1.45]	1.22 [1.13-1.32]	1.30 [1.28-1.33]	1.31 [1.28-1.33]	1.26 [1.24-1.29]	1.27 [1.25-1.30]	1.19 [1.10-1.28]	1.23 [1.21-1.26]	26,684	25,680	22,682	1,788	23,758
729.1	1.68 [1.54-1.83]	1.66 [1.53-1.81]	1.66 [1.51-1.82]		1.48 [1.35-1.62]	1.38 [1.26-1.51]	1.29 [1.18-1.42]	1.30 [1.18-1.44]		1.27 [1.16-1.39]	1,338	1,334	1,127	NA	1,213
729.3	1.38 [1.21-1.57]	1.37 [1.20-1.57]	1.42 [1.21-1.67]	1.21 [0.88-1.67]	1.28 [1.11-1.47]	1.26 [1.10-1.44]	1.22 [1.06-1.40]	1.25 [1.05-1.48]	1.11 [0.80-1.55]	1.20 [1.04-1.38]	584	547	360	104	503
731	1.07 [0.98-1.16]	1.04 [0.96-1.13]	1.05 [0.96-1.14]		1.02 [0.94-1.11]	1.06 [0.98-1.16]	1.03 [0.94-1.12]	1.03 [0.94-1.12]		1.04 [0.96-1.14]	1,375	1,340	1,287	NA	1,220
731.1	1.16 [1.04-1.29]	1.14 [1.03-1.28]	1.17 [1.05-1.31]		1.07 [0.95-1.20]	1.13 [1.01-1.26]	1.10 [0.99-1.23]	1.13 [1.01-1.26]		1.04 [0.93-1.18]	890	887	851	NA	738
732	1.27 [1.15-1.40]	1.30 [1.16-1.46]	1.31 [1.13-1.51]	1.17 [1.01-1.36]	1.17 [1.05-1.30]	1.17 [1.06-1.29]	1.13 [1.01-1.28]	1.16 [1.00-1.34]	1.10 [0.94-1.28]	1.10 [0.99-1.23]	1,066	703	437	473	860
732.1	1.31 [1.19-1.43]	1.36 [1.20-1.53]	1.38 [1.17-1.63]	1.22 [1.08-1.38]	1.22 [1.10-1.35]	1.21 [1.10-1.33]	1.20 [1.06-1.36]	1.24 [1.05-1.47]	1.14 [1.00-1.29]	1.13 [1.02-1.25]	1,218	665	355	690	981
732.7	1.37 [1.15-1.64]	1.25 [1.01-1.55]		1.50 [1.16-1.94]	1.20 [0.98-1.47]	1.25 [1.04-1.50]	1.07 [0.86-1.34]		1.39 [1.07-1.81]	1.12 [0.91-1.38]	319	206	NA	172	247
733	1.31 [1.27-1.34]	1.32 [1.28-1.35]	1.32 [1.29-1.36]	1.18 [1.10-1.27]	1.22 [1.19-1.26]	1.20 [1.17-1.23]	1.17 [1.14-1.20]	1.18 [1.15-1.22]	1.13 [1.05-1.22]	1.16 [1.13-1.19]	16,105	14,991	12,630	2,032	14,549
733.2	1.18 [1.05-1.32]	1.20 [1.06-1.36]	1.21 [1.05-1.38]	0.98 [0.76-1.27]	1.09 [0.96-1.24]	1.12 [0.99-1.26]	1.11 [0.98-1.27]	1.11 [0.97-1.28]	0.97 [0.74-1.26]	1.06 [0.93-1.21]	699	580	488	150	588
733.4	1.28 [1.18-1.39]	1.27 [1.17-1.38]	1.28 [1.17-1.39]	1.22 [0.93-1.59]	1.16 [1.07-1.26]	1.21 [1.11-1.31]	1.16 [1.07-1.26]	1.16 [1.06-1.27]	1.16 [0.88-1.53]	1.13 [1.04-1.23]	1,542	1,451	1,285	146	1,373
733.6	1.41 [1.31-1.52]	1.42 [1.32-1.54]	1.50 [1.37-1.64]	1.20 [1.04-1.40]	1.31 [1.22-1.42]	1.26 [1.16-1.36]	1.19 [1.10-1.29]	1.24 [1.12-1.37]	1.17 [1.00-1.37]	1.22 [1.13-1.31]	1,967	1,708	1,170	495	1,812
733.8	1.24 [1.17-1.30]	1.25 [1.19-1.32]	1.28 [1.20-1.37]	1.15 [1.02-1.29]	1.15 [1.08-1.21]	1.16 [1.10-1.22]	1.13 [1.07-1.20]	1.16 [1.08-1.24]	1.11 [0.98-1.25]	1.12 [1.06-1.19]	3,449	3,079	2,264	732	2,846
733.9	1.33 [1.11-1.60]	1.37 [1.14-1.64]	1.44 [1.17-1.77]		1.22 [1.00-1.48]	1.20 [0.99-1.45]	1.20 [0.99-1.45]	1.31 [1.06-1.62]		1.14 [0.94-1.40]	288	278	215	NA	243
735	1.29 [1.13-1.48]	1.42 [1.22-1.66]	1.43 [1.21-1.70]	1.01 [0.78-1.32]	1.17 [1.02-1.35]	1.21 [1.05-1.39]	1.29 [1.10-1.51]	1.31 [1.10-1.56]	0.98 [0.75-1.29]	1.13 [0.98-1.30]	539	419	334	150	485
735.1	1.33 [1.17-1.50]	1.41 [1.22-1.63]	1.39 [1.19-1.63]	1.18 [0.94-1.48]	1.28 [1.12-1.46]	1.20 [1.05-1.36]	1.25 [1.08-1.45]	1.22 [1.03-1.43]	1.09 [0.86-1.38]	1.19 [1.04-1.36]	658	457	379	228	598
735.2	1.42 [1.34-1.50]	1.42 [1.34-1.50]	1.42 [1.33-1.51]	1.21 [1.04-1.40]	1.27 [1.20-1.35]	1.32 [1.24-1.39]	1.29 [1.22-1.37]	1.28 [1.21-1.37]	1.15 [0.99-1.34]	1.20 [1.13-1.28]	3,176	2,826	2,467	467	2,731
735.21	1.37 [1.29-1.46]	1.36 [1.28-1.45]	1.35 [1.26-1.44]	1.34 [1.00-1.79]	1.26 [1.18-1.34]	1.27 [1.19-1.35]	1.23 [1.16-1.31]	1.23 [1.15-1.31]	1.31 [0.97-1.77]	1.17 [1.10-1.26]	2,492	2,411	2,256	127	2,165
735.23	1.22 [1.12-1.32]	1.26 [1.17-1.37]	1.26 [1.16-1.37]		1.17 [1.08-1.28]	1.13 [1.05-1.23]	1.15 [1.06-1.25]	1.16 [1.06-1.26]		1.11 [1.01-1.21]	1,423	1,419	1,339	NA	1,217
735.3	1.28 [1.23-1.33]	1.27 [1.22-1.32]	1.27 [1.22-1.32]	1.20 [1.02-1.40]	1.18 [1.13-1.23]	1.21 [1.16-1.25]	1.18 [1.13-1.23]	1.18 [1.14-1.23]	1.16 [0.99-1.36]	1.13 [1.08-1.18]	6,813	6,626	5,717	433	5,659
736	1.20 [1.15-1.24]	1.24 [1.19-1.30]	1.23 [1.18-1.29]	1.03 [0.95-1.12]	1.12 [1.07-1.16]	1.12 [1.08-1.17]	1.14 [1.09-1.19]	1.13 [1.08-1.19]	0.99 [0.91-1.08]	1.08 [1.03-1.12]	6,788	5,505	4,784	1,559	6,095
736.2	1.17 [1.02-1.34]	1.23 [1.06-1.42]	1.19 [1.00-1.40]	1.05 [0.77-1.43]	1.07 [0.92-1.24]	1.12 [0.97-1.29]	1.17 [1.01-1.35]	1.14 [0.96-1.36]	0.98 [0.72-1.35]	1.03 [0.89-1.20]	510	442	326	107	419
737	1.24 [1.07-1.45]	1.27 [1.08-1.50]	1.36 [1.13-1.63]		1.16 [0.99-1.36]	1.16 [0.99-1.35]	1.14 [0.96-1.35]	1.25 [1.03-1.51]		1.11 [0.95-1.30]	444	370	302	NA	408
737.1	1.24 [1.16-1.32]	1.22 [1.15-1.31]	1.23 [1.15-1.31]	1.10 [0.83-1.45]	1.18 [1.10-1.25]	1.17 [1.10-1.25]	1.13 [1.05-1.20]	1.14 [1.07-1.22]	1.02 [0.77-1.35]	1.13 [1.06-1.21]	2,707	2,616	2,513	136	2,564
737.2	1.41 [1.18-1.69]	1.41 [1.17-1.70]	1.41 [1.15-1.73]		1.23 [1.02-1.47]	1.31 [1.09-1.57]	1.24 [1.02-1.51]	1.27 [1.03-1.57]		1.15 [0.95-1.38]	332	303	256	NA	303
737.3	1.24 [1.20-1.28]	1.27 [1.23-1.32]	1.27 [1.22-1.32]	1.07 [0.99-1.15]	1.14 [1.10-1.18]	1.16 [1.12-1.20]	1.16 [1.12-1.21]	1.16 [1.11-1.21]	1.03 [0.96-1.12]	1.09 [1.05-1.13]	8,868	7,537	6,538	1,747	8,022
738	1.34 [1.22-1.48]	1.32 [1.20-1.46]	1.29 [1.13-1.47]	1.21 [1.00-1.47]	1.18 [1.06-1.31]	1.24 [1.12-1.37]	1.16 [1.04-1.29]	1.09 [0.95-1.25]	1.19 [0.98-1.45]	1.12 [1.01-1.24]	1,092	944	573	293	924
738.4	1.32 [1.26-1.37]	1.31 [1.25-1.37]	1.30 [1.24-1.36]	1.06 [0.79-1.42]	1.20 [1.15-1.25]	1.21 [1.16-1.27]	1.15 [1.10-1.21]	1.14 [1.09-1.19]	0.99 [0.73-1.34]	1.12 [1.07-1.17]	5,500	5,432	5,196	123	5,084
739	1.16 [1.07-1.26]	1.27 [1.16-1.39]	1.29 [1.18-1.43]	0.89 [0.74-1.06]	1.09 [1.00-1.19]	1.10 [1.01-1.20]	1.18 [1.08-1.29]	1.20 [1.09-1.33]	0.88 [0.74-1.06]	1.06 [0.98-1.16]	1,464	1,226	1,038	308	1,323
740.1	1.14 [1.12-1.17]	1.14 [1.12-1.16]	1.13 [1.11-1.16]	1.11 [0.84-1.48]	1.09 [1.07-1.11]	1.10 [1.08-1.12]	1.08 [1.06-1.10]	1.07 [1.05-1.09]	1.07 [0.80-1.43]	1.06 [1.04-1.08]	27,965	27,940	27,430	132	24,100
740.11	1.19 [1.15-1.23]	1.18 [1.15-1.22]	1.18 [1.15-1.22]		1.11 [1.07-1.15]	1.14 [1.10-1.17]	1.12 [1.08-1.15]	1.11 [1.08-1.15]		1.08 [1.04-1.11]	9,407	9,401	9,174	NA	7,603
740.12	1.31 [1.17-1.47]	1.29 [1.15-1.45]	1.33 [1.18-1.51]		1.12 [0.99-1.26]	1.23 [1.09-1.38]	1.20 [1.07-1.35]	1.25 [1.11-1.42]		1.08 [0.96-1.22]	727	717	616	NA	637
740.2	1.34 [1.23-1.46]	1.35 [1.24-1.48]	1.32 [1.21-1.44]		1.22 [1.12-1.34]	1.23 [1.13-1.35]	1.22 [1.11-1.33]	1.19 [1.08-1.30]		1.15 [1.05-1.26]	1,275	1,275	1,262	NA	1,159
740.9	1.34 [1.32-1.36]	1.33 [1.31-1.35]	1.33 [1.31-1.35]	1.30 [1.12-1.51]	1.25 [1.23-1.27]	1.24 [1.22-1.25]	1.19 [1.17-1.21]	1.19 [1.17-1.21]	1.21 [1.04-1.41]	1.18 [1.16-1.20]	48,612	48,412	47,100	490	44,277
741	1.37 [1.30-1.44]	1.39 [1.32-1.47]	1.35 [1.26-1.44]	1.21 [1.06-1.37]	1.25 [1.18-1.32]	1.25 [1.19-1.32]	1.24 [1.17-1.31]	1.18 [1.11-1.27]	1.15 [1.00-1.31]	1.18 [1.11-1.25]	3,589	3,312	2,302	621	3,109
741.1	1.15 [0.95-1.40]	1.20 [0.98-1.46]	1.10 [0.88-1.37]		1.07 [0.88-1.31]	1.06 [0.87-1.30]	1.11 [0.90-1.36]	0.98 [0.78-1.24]		1.02 [0.84-1.25]	260	240	187	NA	237
741.2	1.33 [1.22-1.44]	1.35 [1.23-1.47]	1.35 [1.22-1.48]	1.16 [0.95-1.41]	1.26 [1.15-1.37]	1.24 [1.14-1.35]	1.23 [1.12-1.34]	1.23 [1.12-1.36]	1.11 [0.90-1.36]	1.20 [1.10-1.31]	1,473	1,291	1,032	273	1,313
741.3	1.23 [1.17-1.29]	1.27 [1.20-1.33]	1.25 [1.18-1.31]	0.98 [0.84-1.14]	1.17 [1.12-1.23]	1.17 [1.11-1.22]	1.15 [1.09-1.21]	1.13 [1.08-1.19]	0.95 [0.82-1.12]	1.14 [1.08-1.20]	4,840	4,451	4,353	415	4,521
741.4	1.29 [1.25-1.34]	1.31 [1.26-1.36]	1.30 [1.25-1.35]	1.17 [1.07-1.28]	1.20 [1.16-1.24]	1.21 [1.16-1.25]	1.18 [1.14-1.23]	1.18 [1.13-1.23]	1.12 [1.03-1.23]	1.15 [1.11-1.20]	8,354	7,184	6,528	1,383	7,485
741.5	1.29 [1.14-1.45]	1.37 [1.21-1.55]	1.43 [1.25-1.62]		1.26 [1.11-1.43]	1.22 [1.08-1.38]	1.25 [1.10-1.43]	1.28 [1.12-1.47]		1.21 [1.06-1.37]	712	683	641	NA	659
741.6	1.19 [0.88-1.59]					1.10 [0.81-1.49]					112	NA	NA	NA	NA
742	1.06 [0.82-1.35]	1.08 [0.84-1.40]	1.14 [0.88-1.47]		0.99 [0.76-1.29]	1.03 [0.80-1.33]	1.03 [0.79-1.33]	1.11 [0.85-1.43]		0.98 [0.75-1.27]	150	149	143	NA	135
742.1	1.38 [1.22-1.57]	1.32 [1.16-1.50]	1.40 [1.20-1.64]	1.39 [1.04-1.85]	1.19 [1.04-1.37]	1.31 [1.15-1.49]	1.20 [1.05-1.37]	1.26 [1.08-1.49]	1.39 [1.03-1.87]	1.15 [1.00-1.32]	607	559	382	118	490
742.2	1.39 [1.21-1.58]	1.45 [1.25-1.69]	1.52 [1.13-2.04]	1.28 [1.07-1.53]	1.28 [1.11-1.49]	1.25 [1.09-1.43]	1.26 [1.07-1.47]	1.33 [0.98-1.81]	1.18 [0.98-1.42]	1.19 [1.02-1.38]	599	429	111	333	467
742.8	1.33 [1.25-1.40]	1.30 [1.23-1.38]	1.31 [1.22-1.41]	1.27 [1.11-1.46]	1.19 [1.12-1.26]	1.21 [1.14-1.29]	1.15 [1.08-1.22]	1.15 [1.07-1.24]	1.20 [1.04-1.38]	1.12 [1.06-1.19]	3,081	2,901	1,818	588	2,634
742.9	1.26 [1.22-1.31]	1.29 [1.24-1.33]	1.30 [1.24-1.36]	1.13 [1.05-1.21]	1.15 [1.11-1.20]	1.16 [1.12-1.20]	1.14 [1.10-1.18]	1.14 [1.09-1.20]	1.07 [0.99-1.15]	1.09 [1.05-1.13]	8,330	7,532	4,477	2,078	6,999
743.1	1.28 [1.23-1.34]	1.29 [1.24-1.36]	1.30 [1.24-1.36]		1.22 [1.17-1.28]	1.21 [1.16-1.27]	1.18 [1.13-1.24]	1.19 [1.13-1.25]		1.17 [1.12-1.23]	4,996	4,983	4,950	NA	4,693
743.11	1.30 [1.28-1.33]	1.30 [1.28-1.32]	1.30 [1.28-1.32]	1.38 [1.07-1.78]	1.23 [1.20-1.25]	1.22 [1.20-1.24]	1.17 [1.15-1.19]	1.17 [1.15-1.20]	1.30 [1.00-1.69]	1.16 [1.14-1.18]	35,604	35,547	35,063	173	32,807
743.12	1.33 [1.07-1.66]	1.37 [1.10-1.71]	1.39 [1.11-1.73]		1.38 [1.10-1.73]	1.27 [1.02-1.59]	1.25 [0.99-1.56]	1.27 [1.01-1.60]		1.29 [1.02-1.62]	213	212	212	NA	204
743.13	1.40 [1.27-1.56]	1.42 [1.27-1.57]	1.38 [1.24-1.53]		1.30 [1.17-1.45]	1.28 [1.15-1.43]	1.24 [1.11-1.38]	1.23 [1.10-1.37]		1.20 [1.07-1.34]	934	908	848	NA	812
743.2	1.22 [1.12-1.32]	1.26 [1.15-1.37]	1.24 [1.14-1.35]		1.16 [1.07-1.26]	1.17 [1.08-1.27]	1.16 [1.07-1.27]	1.16 [1.07-1.27]		1.14 [1.05-1.24]	1,509	1,434	1,396	NA	1,370
743.21	1.22 [1.16-1.29]	1.24 [1.17-1.30]	1.24 [1.17-1.31]		1.16 [1.10-1.22]	1.15 [1.09-1.22]	1.13 [1.07-1.19]	1.13 [1.07-1.19]		1.11 [1.05-1.18]	3,940	3,922	3,867	NA	3,706
743.4	1.39 [1.13-1.71]	1.37 [1.11-1.69]	1.34 [1.09-1.65]		1.18 [0.95-1.45]	1.32 [1.07-1.62]	1.25 [1.01-1.55]	1.26 [1.02-1.56]		1.14 [0.92-1.40]	262	252	246	NA	238
743.9	1.27 [1.19-1.36]	1.29 [1.20-1.39]	1.28 [1.18-1.38]	1.14 [0.98-1.32]	1.18 [1.10-1.27]	1.19 [1.11-1.28]	1.16 [1.07-1.25]	1.15 [1.06-1.24]	1.10 [0.94-1.28]	1.13 [1.05-1.22]	2,210	1,849	1,526	459	1,919
745	1.30 [1.28-1.33]	1.31 [1.28-1.33]	1.30 [1.27-1.32]	1.22 [1.17-1.28]	1.21 [1.19-1.23]	1.21 [1.19-1.23]	1.16 [1.14-1.18]	1.15 [1.13-1.18]	1.17 [1.11-1.22]	1.15 [1.13-1.17]	36,720	32,923	28,365	5,197	33,207
congenital anomalies															
747	1.31 [1.12-1.54]	1.46 [1.23-1.74]	1.36 [1.11-1.66]	0.96 [0.70-1.31]	1.17 [0.98-1.39]	1.26 [1.07-1.48]	1.36 [1.13-1.62]	1.25 [1.01-1.54]	0.95 [0.69-1.32]	1.16 [0.97-1.38]	397	328	237	102	319
747.1	1.20 [1.12-1.28]	1.25 [1.16-1.36]	1.24 [1.13-1.37]	1.04 [0.93-1.16]	1.10 [1.03-1.18]	1.14 [1.06-1.22]	1.14 [1.05-1.24]	1.14 [1.04-1.26]	1.00 [0.89-1.12]	1.06 [0.98-1.14]	2,218	1,555	1,027	854	1,912
747.11	1.15 [1.08-1.23]	1.23 [1.14-1.33]	1.27 [1.16-1.39]	1.00 [0.90-1.10]	1.11 [1.04-1.19]	1.10 [1.03-1.17]	1.14 [1.05-1.24]	1.16 [1.05-1.27]	0.97 [0.87-1.07]	1.06 [0.99-1.14]	2,389	1,558	1,130	961	2,019
747.12	1.11 [1.02-1.22]	1.16 [1.04-1.30]	1.14 [1.00-1.29]	1.01 [0.88-1.17]	1.02 [0.93-1.13]	1.09 [1.00-1.20]	1.10 [0.98-1.23]	1.09 [0.96-1.24]	1.00 [0.86-1.15]	1.01 [0.91-1.12]	1,159	764	576	465	981
747.13	1.23 [1.20-1.26]	1.24 [1.20-1.27]	1.24 [1.21-1.28]	0.98 [0.87-1.12]	1.17 [1.14-1.21]	1.18 [1.15-1.21]	1.14 [1.11-1.17]	1.15 [1.12-1.18]	0.95 [0.84-1.08]	1.14 [1.10-1.17]	15,224	14,686	14,457	616	13,912
747.2	1.18 [1.06-1.30]	1.31 [1.15-1.48]	1.28 [1.10-1.48]	1.02 [0.87-1.21]	1.10 [0.99-1.23]	1.13 [1.02-1.26]	1.23 [1.08-1.39]	1.18 [1.01-1.37]	1.01 [0.86-1.19]	1.09 [0.98-1.22]	942	624	441	391	823
748	1.05 [0.92-1.21]	1.30 [1.02-1.64]	1.46 [1.12-1.90]	0.98 [0.83-1.16]	0.98 [0.85-1.13]	0.94 [0.81-1.08]	1.09 [0.85-1.40]	1.27 [0.97-1.67]	0.86 [0.73-1.02]	0.88 [0.76-1.02]	491	168	143	335	454
749	1.13 [1.07-1.19]	1.18 [1.04-1.34]	1.35 [1.13-1.61]	1.09 [1.03-1.16]	1.09 [1.02-1.16]	1.10 [1.04-1.17]	1.08 [0.95-1.23]	1.25 [1.04-1.50]	1.07 [1.01-1.14]	1.08 [1.01-1.15]	3,107	593	284	2,653	2,401
749.1	1.03 [0.87-1.21]			1.02 [0.85-1.22]	0.98 [0.82-1.18]	0.98 [0.83-1.16]			0.96 [0.80-1.16]	0.96 [0.79-1.15]	348	NA	NA	286	292
749.2	1.06 [0.93-1.21]			1.00 [0.87-1.15]	1.00 [0.87-1.14]	1.01 [0.89-1.16]			0.97 [0.84-1.12]	0.98 [0.85-1.12]	560	NA	NA	490	501
750	1.34 [1.02-1.76]	1.36 [1.01-1.83]			1.37 [1.03-1.81]	1.24 [0.93-1.64]	1.13 [0.82-1.54]			1.29 [0.97-1.72]	135	111	NA	NA	126
750.1	1.19 [0.99-1.42]	1.18 [0.96-1.45]	1.22 [0.98-1.51]		1.13 [0.94-1.36]	1.09 [0.90-1.31]	1.02 [0.82-1.26]	1.07 [0.85-1.34]		1.06 [0.88-1.29]	294	224	200	NA	267
750.11	1.42 [1.08-1.87]	1.53 [1.14-2.07]	1.54 [1.13-2.10]		1.45 [1.09-1.94]	1.31 [0.99-1.73]	1.40 [1.03-1.90]	1.39 [1.02-1.91]		1.38 [1.03-1.85]	134	114	108	NA	121
750.13	1.14 [1.07-1.22]	1.15 [1.02-1.31]	1.18 [1.04-1.35]	1.11 [1.03-1.20]	1.10 [1.02-1.18]	1.11 [1.04-1.18]	1.08 [0.95-1.23]	1.09 [0.95-1.25]	1.10 [1.02-1.18]	1.07 [1.00-1.16]	2,319	657	591	1,694	1,931
750.14	1.27 [1.08-1.49]	1.30 [1.09-1.53]	1.29 [1.08-1.53]		1.11 [0.93-1.31]	1.17 [0.99-1.39]	1.16 [0.98-1.38]	1.16 [0.97-1.39]		1.03 [0.87-1.23]	376	352	326	NA	334
750.15	1.26 [1.00-1.59]	1.18 [0.93-1.50]	1.17 [0.91-1.49]		1.22 [0.96-1.55]	1.12 [0.88-1.42]	1.00 [0.78-1.28]	1.01 [0.78-1.30]		1.13 [0.88-1.44]	178	168	152	NA	168
750.21	1.18 [1.07-1.31]	1.22 [1.09-1.37]	1.21 [1.06-1.39]	1.01 [0.85-1.21]	1.14 [1.02-1.26]	1.11 [1.00-1.23]	1.09 [0.97-1.23]	1.10 [0.96-1.26]	0.99 [0.82-1.19]	1.07 [0.96-1.20]	970	742	530	303	832
750.22	1.20 [1.02-1.41]	1.23 [1.03-1.45]	1.14 [0.94-1.38]		1.15 [0.97-1.36]	1.13 [0.96-1.34]	1.09 [0.92-1.30]	1.02 [0.84-1.25]		1.10 [0.93-1.31]	381	328	256	NA	338
751.1	1.13 [1.06-1.21]	1.22 [0.96-1.54]		1.11 [1.03-1.19]	1.13 [1.04-1.21]	1.10 [1.03-1.19]	1.11 [0.87-1.42]		1.08 [1.00-1.16]	1.10 [1.02-1.19]	2,131	164	NA	2,016	1,782
751.11	1.17 [1.08-1.27]	1.20 [1.10-1.30]	1.21 [1.06-1.37]	1.23 [1.07-1.41]	1.13 [1.03-1.23]	1.11 [1.02-1.20]	1.10 [1.01-1.20]	1.10 [0.97-1.26]	1.16 [1.00-1.34]	1.09 [1.00-1.19]	1,604	1,319	564	541	1,301
751.12	1.03 [0.98-1.09]	1.22 [1.06-1.41]	1.17 [0.99-1.39]	1.01 [0.96-1.07]	0.99 [0.93-1.04]	1.02 [0.97-1.07]	1.14 [0.98-1.33]	1.12 [0.94-1.33]	0.99 [0.94-1.05]	0.98 [0.93-1.04]	3,929	455	327	3,525	3,175
751.2	1.12 [0.97-1.28]	1.22 [0.99-1.52]	1.22 [0.94-1.59]	1.03 [0.87-1.23]	1.04 [0.89-1.20]	1.08 [0.93-1.24]	1.16 [0.93-1.44]	1.15 [0.88-1.51]	1.01 [0.84-1.21]	1.02 [0.88-1.19]	485	199	133	316	427
751.21	1.16 [1.07-1.26]	1.14 [1.05-1.24]	1.19 [1.08-1.30]	1.05 [0.86-1.29]	1.07 [0.99-1.17]	1.11 [1.03-1.21]	1.04 [0.95-1.14]	1.10 [1.01-1.21]	1.00 [0.82-1.23]	1.05 [0.96-1.15]	1,502	1,317	1,141	239	1,307
751.22	1.16 [1.08-1.25]	1.18 [1.08-1.28]	1.14 [1.03-1.26]	1.09 [0.96-1.24]	1.06 [0.98-1.15]	1.10 [1.02-1.19]	1.06 [0.97-1.15]	1.03 [0.93-1.14]	1.07 [0.94-1.21]	1.02 [0.94-1.11]	1,806	1,332	944	630	1,582
751.3	1.00 [0.85-1.18]	1.01 [0.77-1.31]		0.99 [0.81-1.21]	0.96 [0.81-1.15]	0.98 [0.83-1.16]	0.98 [0.75-1.29]		0.97 [0.79-1.19]	0.96 [0.80-1.15]	338	130	NA	228	300
752.1	1.29 [1.06-1.56]	1.28 [1.03-1.59]	1.46 [1.11-1.92]		1.17 [0.96-1.43]	1.18 [0.97-1.44]	1.08 [0.86-1.36]	1.18 [0.88-1.57]		1.11 [0.91-1.36]	263	198	127	NA	234
752.11	1.30 [1.17-1.44]	1.35 [1.20-1.52]	1.33 [1.14-1.55]	1.12 [0.95-1.32]	1.19 [1.07-1.33]	1.18 [1.06-1.31]	1.18 [1.04-1.33]	1.15 [0.98-1.35]	1.04 [0.88-1.24]	1.11 [0.99-1.23]	964	722	395	361	829
752.2	1.14 [1.05-1.23]	1.35 [1.20-1.52]	1.38 [1.17-1.63]	1.04 [0.94-1.14]	1.07 [0.99-1.16]	1.06 [0.98-1.15]	1.17 [1.04-1.33]	1.18 [0.99-1.40]	1.00 [0.91-1.10]	1.02 [0.94-1.11]	1,632	710	345	1,073	1,471
753	1.18 [0.96-1.45]	1.28 [0.96-1.70]		1.05 [0.79-1.40]	1.11 [0.89-1.39]	1.11 [0.90-1.37]	1.22 [0.91-1.63]		0.98 [0.73-1.31]	1.08 [0.86-1.35]	223	119	NA	117	193
753.1	1.22 [1.02-1.46]	1.26 [1.00-1.58]	1.23 [0.93-1.62]	1.25 [0.97-1.63]	1.16 [0.95-1.40]	1.17 [0.98-1.41]	1.19 [0.94-1.51]	1.18 [0.88-1.56]	1.21 [0.93-1.57]	1.13 [0.93-1.38]	313	185	123	150	261
753.2	1.11 [0.86-1.44]				1.08 [0.82-1.42]	1.07 [0.82-1.39]				1.08 [0.82-1.43]	142	NA	NA	NA	126
754	1.22 [1.07-1.40]	1.33 [1.13-1.57]	1.41 [1.16-1.72]	1.04 [0.84-1.28]	1.12 [0.97-1.29]	1.14 [0.99-1.31]	1.16 [0.98-1.38]	1.22 [0.99-1.50]	0.97 [0.78-1.21]	1.07 [0.93-1.24]	544	351	240	233	480
755.1	1.26 [1.15-1.37]	1.37 [1.20-1.57]	1.60 [1.36-1.89]	1.12 [1.01-1.25]	1.12 [1.02-1.23]	1.19 [1.09-1.30]	1.25 [1.09-1.44]	1.44 [1.21-1.71]	1.08 [0.97-1.21]	1.09 [0.99-1.20]	1,329	528	363	872	1,112
755.3	1.02 [0.87-1.20]			0.95 [0.80-1.13]	0.94 [0.78-1.12]	0.98 [0.83-1.16]			0.94 [0.78-1.12]	0.94 [0.78-1.12]	366	NA	NA	297	286
755.4	1.22 [1.08-1.38]	1.16 [0.91-1.46]	1.32 [0.98-1.78]	1.21 [1.05-1.40]	1.06 [0.92-1.22]	1.19 [1.05-1.36]	1.08 [0.85-1.38]	1.20 [0.88-1.63]	1.20 [1.04-1.38]	1.05 [0.91-1.21]	638	174	109	495	523

Supplementary Table 6: phecode-mapped results (hazard ratios and events from all cohorts)

	Hazard ratio (99% confidence interval)														
	crude					adjusted					Events (in exposed)				
Outcome	any age	18+	40+	<18	hosp.	any age	18+	40+	<18	hosp.	any age	18+	40+	<18	hosp.
755.6	1.21 [1.05-1.38]	1.26 [1.03-1.54]	1.33 [1.01-1.75]	1.10 [0.93-1.31]	1.08 [0.93-1.25]	1.15 [1.00-1.33]	1.14 [0.93-1.40]	1.18 [0.89-1.58]	1.07 [0.90-1.27]	1.04 [0.89-1.21]	566	241	124	381	476
755.61	1.04 [0.94-1.15]	1.17 [1.02-1.34]	1.18 [0.97-1.43]	0.89 [0.78-1.02]	0.93 [0.84-1.04]	0.99 [0.89-1.09]	1.07 [0.93-1.23]	1.08 [0.88-1.32]	0.86 [0.75-0.99]	0.91 [0.82-1.02]	947	502	238	525	815
756	1.20 [1.05-1.36]	1.20 [0.98-1.47]	1.31 [1.00-1.72]	1.12 [0.96-1.32]	1.03 [0.89-1.19]	1.12 [0.98-1.29]	1.06 [0.86-1.31]	1.16 [0.87-1.54]	1.08 [0.92-1.28]	0.99 [0.85-1.14]	569	228	125	386	476
756.1	1.00 [0.75-1.33]					0.90 [0.67-1.21]					114	NA	NA	NA	NA
756.21	1.26 [1.04-1.54]	1.27 [0.96-1.68]		1.19 [0.94-1.52]	1.24 [1.00-1.54]	1.24 [1.02-1.52]	1.26 [0.94-1.69]		1.15 [0.90-1.46]	1.23 [0.99-1.53]	267	119	NA	183	223
756.3	1.43 [1.28-1.61]	1.39 [1.21-1.59]	1.24 [0.98-1.56]	1.32 [1.12-1.56]	1.25 [1.10-1.41]	1.18 [1.04-1.33]	1.01 [0.87-1.17]	0.89 [0.69-1.14]	1.19 [1.00-1.42]	1.07 [0.94-1.22]	797	540	173	411	697
756.5	1.27 [1.18-1.37]	1.31 [1.21-1.42]	1.32 [1.20-1.44]	1.07 [0.91-1.25]	1.15 [1.06-1.24]	1.19 [1.10-1.28]	1.20 [1.10-1.30]	1.17 [1.07-1.29]	1.03 [0.88-1.21]	1.11 [1.02-1.20]	1,865	1,550	1,251	417	1,651
757	1.36 [1.22-1.51]	1.77 [1.50-2.10]	1.81 [1.49-2.20]	1.20 [1.04-1.38]	1.32 [1.18-1.49]	1.28 [1.15-1.43]	1.59 [1.34-1.89]	1.58 [1.29-1.93]	1.16 [1.00-1.33]	1.27 [1.12-1.43]	857	373	279	519	718
758	1.28 [1.10-1.49]	1.42 [1.16-1.73]	1.50 [1.15-1.96]	1.05 [0.85-1.29]	1.12 [0.95-1.31]	1.21 [1.04-1.42]	1.26 [1.02-1.54]	1.36 [1.03-1.79]	1.04 [0.84-1.28]	1.09 [0.92-1.29]	444	247	138	235	374
758.1	1.71 [1.59-1.84]	2.48 [2.24-2.74]	2.96 [2.61-3.35]	1.13 [1.02-1.25]	1.38 [1.27-1.51]	1.62 [1.50-1.75]	2.39 [2.15-2.65]	2.81 [2.48-3.20]	1.08 [0.97-1.20]	1.31 [1.20-1.43]	2,051	1,166	762	983	1,424
759	1.13 [1.01-1.26]	1.26 [1.08-1.47]	1.36 [1.13-1.64]	1.05 [0.91-1.22]	1.08 [0.96-1.21]	1.06 [0.95-1.19]	1.14 [0.97-1.34]	1.25 [1.03-1.52]	1.00 [0.86-1.17]	1.04 [0.92-1.17]	797	413	265	454	724
759.1	1.06 [0.92-1.22]	1.11 [0.88-1.38]	1.02 [0.77-1.36]	1.04 [0.87-1.24]	1.04 [0.89-1.21]	1.04 [0.90-1.20]	1.03 [0.82-1.29]	0.99 [0.74-1.32]	1.03 [0.86-1.23]	1.03 [0.88-1.21]	481	185	108	325	399
symptoms															
760	1.30 [1.28-1.32]	1.30 [1.28-1.32]	1.30 [1.28-1.32]	1.15 [1.10-1.19]	1.19 [1.17-1.20]	1.19 [1.17-1.21]	1.13 [1.12-1.15]	1.14 [1.12-1.16]	1.09 [1.04-1.14]	1.12 [1.11-1.14]	60,233	57,895	46,321	5,857	53,676
761	1.34 [1.30-1.39]	1.33 [1.28-1.38]	1.36 [1.30-1.41]	1.19 [1.07-1.31]	1.22 [1.17-1.26]	1.21 [1.16-1.25]	1.13 [1.09-1.18]	1.15 [1.11-1.20]	1.12 [1.01-1.25]	1.13 [1.09-1.17]	7,994	7,406	6,088	1,030	7,247
764	1.29 [1.25-1.33]	1.30 [1.26-1.34]	1.29 [1.25-1.33]	1.16 [0.98-1.38]	1.19 [1.16-1.23]	1.17 [1.13-1.21]	1.11 [1.08-1.15]	1.10 [1.06-1.14]	1.12 [0.94-1.34]	1.12 [1.08-1.15]	10,148	10,113	8,600	361	9,225
765	1.32 [1.22-1.44]	1.31 [1.20-1.42]	1.33 [1.22-1.46]		1.21 [1.11-1.31]	1.19 [1.09-1.30]	1.10 [1.00-1.20]	1.11 [1.02-1.22]		1.12 [1.03-1.23]	1,339	1,340	1,189	NA	1,245
766	1.33 [1.27-1.39]	1.33 [1.27-1.39]	1.33 [1.27-1.40]	1.22 [0.94-1.56]	1.21 [1.15-1.27]	1.18 [1.13-1.24]	1.12 [1.07-1.17]	1.11 [1.06-1.17]	1.16 [0.89-1.51]	1.12 [1.07-1.18]	4,757	4,713	4,155	169	4,450
770	1.37 [1.30-1.44]	1.37 [1.30-1.45]	1.42 [1.34-1.51]	1.24 [1.12-1.37]	1.27 [1.21-1.34]	1.25 [1.19-1.32]	1.20 [1.13-1.27]	1.24 [1.17-1.32]	1.17 [1.06-1.30]	1.20 [1.14-1.27]	4,383	3,601	2,770	1,035	3,877
771	1.36 [1.28-1.44]	1.39 [1.30-1.47]	1.40 [1.31-1.50]	1.25 [1.06-1.47]	1.30 [1.22-1.38]	1.26 [1.19-1.34]	1.24 [1.16-1.32]	1.25 [1.17-1.34]	1.20 [1.01-1.43]	1.24 [1.16-1.32]	2,936	2,660	2,254	385	2,520
771.1	1.40 [1.37-1.43]	1.41 [1.38-1.44]	1.42 [1.39-1.45]	1.21 [1.10-1.32]	1.31 [1.28-1.34]	1.31 [1.28-1.34]	1.26 [1.24-1.29]	1.27 [1.24-1.30]	1.17 [1.07-1.28]	1.24 [1.21-1.27]	21,683	20,952	18,627	1,339	19,449
772	1.22 [1.16-1.29]	1.26 [1.19-1.33]	1.24 [1.16-1.31]	1.05 [0.91-1.22]	1.16 [1.09-1.22]	1.17 [1.11-1.24]	1.16 [1.10-1.23]	1.16 [1.09-1.24]	1.00 [0.86-1.16]	1.13 [1.07-1.20]	3,592	3,276	2,820	484	3,259
772.1	1.25 [1.10-1.42]	1.28 [1.12-1.46]	1.36 [1.19-1.56]		1.16 [1.02-1.32]	1.16 [1.02-1.33]	1.18 [1.03-1.35]	1.26 [1.09-1.44]		1.13 [0.99-1.29]	639	603	562	NA	586
772.4	1.07 [0.88-1.29]	1.34 [1.07-1.68]	1.39 [1.08-1.80]		0.93 [0.76-1.13]	0.97 [0.80-1.18]	1.20 [0.95-1.53]	1.24 [0.96-1.62]		0.88 [0.72-1.08]	273	188	151	NA	244
773	1.37 [1.34-1.40]	1.38 [1.35-1.41]	1.39 [1.36-1.42]	1.21 [1.15-1.28]	1.25 [1.23-1.28]	1.26 [1.23-1.29]	1.21 [1.19-1.24]	1.23 [1.20-1.26]	1.17 [1.10-1.24]	1.19 [1.16-1.21]	25,521	23,243	19,458	3,343	22,908
780	1.16 [1.10-1.23]	1.17 [1.11-1.24]	1.16 [1.09-1.22]	1.04 [0.84-1.29]	1.14 [1.08-1.21]	1.18 [1.12-1.25]	1.16 [1.10-1.23]	1.15 [1.08-1.21]	1.04 [0.84-1.30]	1.17 [1.11-1.24]	3,844	3,690	3,544	221	3,561
781	1.18 [1.16-1.19]	1.19 [1.17-1.21]	1.19 [1.17-1.20]	1.06 [1.02-1.10]	1.13 [1.12-1.15]	1.14 [1.12-1.15]	1.11 [1.10-1.13]	1.11 [1.10-1.13]	1.04 [1.00-1.08]	1.11 [1.10-1.13]	61,354	56,317	52,697	6,297	54,633
782.3	1.33 [1.30-1.36]	1.33 [1.30-1.36]	1.34 [1.31-1.37]	1.21 [1.05-1.40]	1.27 [1.24-1.30]	1.26 [1.23-1.29]	1.22 [1.19-1.24]	1.22 [1.19-1.25]	1.16 [1.00-1.35]	1.22 [1.19-1.25]	20,865	20,570	19,881	481	19,325
782.6	1.26 [1.13-1.39]	1.26 [1.12-1.41]	1.15 [1.01-1.31]	1.39 [1.14-1.70]	1.17 [1.05-1.30]	1.17 [1.05-1.30]	1.13 [1.00-1.27]	1.04 [0.91-1.18]	1.30 [1.06-1.60]	1.11 [0.99-1.24]	935	718	596	259	848
783	1.22 [1.20-1.25]	1.27 [1.24-1.30]	1.27 [1.24-1.31]	1.15 [1.12-1.18]	1.16 [1.14-1.18]	1.17 [1.15-1.19]	1.17 [1.14-1.20]	1.17 [1.14-1.21]	1.12 [1.09-1.15]	1.13 [1.11-1.15]	28,770	16,423	12,706	13,760	25,222
785	1.27 [1.26-1.28]	1.28 [1.27-1.30]	1.31 [1.29-1.32]	1.19 [1.17-1.21]	1.18 [1.17-1.19]	1.19 [1.17-1.20]	1.15 [1.13-1.16]	1.17 [1.15-1.18]	1.14 [1.12-1.16]	1.12 [1.11-1.14]	125,464	102,812	62,876	37,604	101,217
788	1.20 [1.18-1.21]	1.20 [1.18-1.22]	1.20 [1.18-1.22]	1.10 [1.05-1.14]	1.14 [1.12-1.16]	1.15 [1.13-1.17]	1.12 [1.10-1.14]	1.12 [1.10-1.14]	1.05 [1.01-1.10]	1.11 [1.09-1.13]	43,614	40,258	35,478	5,290	38,136
789	1.23 [1.22-1.25]	1.25 [1.23-1.27]	1.25 [1.23-1.27]	1.17 [1.14-1.19]	1.15 [1.13-1.17]	1.16 [1.14-1.17]	1.12 [1.10-1.14]	1.13 [1.11-1.15]	1.13 [1.10-1.15]	1.11 [1.09-1.12]	66,001	49,305	37,038	21,941	57,542
790	1.25 [1.15-1.36]	1.27 [1.17-1.39]	1.26 [1.15-1.38]	1.13 [0.88-1.45]	1.22 [1.13-1.33]	1.18 [1.08-1.28]	1.16 [1.06-1.26]	1.16 [1.05-1.27]	1.12 [0.87-1.46]	1.17 [1.08-1.28]	1,549	1,443	1,261	174	1,429
790.1	1.52 [1.28-1.82]	1.49 [1.24-1.79]	1.48 [1.22-1.79]		1.33 [1.10-1.61]	1.44 [1.20-1.72]	1.37 [1.13-1.66]	1.37 [1.12-1.66]		1.25 [1.03-1.52]	331	291	268	NA	274
790.6	1.24 [1.22-1.26]	1.24 [1.21-1.26]	1.24 [1.22-1.27]	1.18 [1.09-1.29]	1.17 [1.15-1.19]	1.18 [1.15-1.20]	1.14 [1.11-1.16]	1.14 [1.11-1.16]	1.15 [1.06-1.25]	1.14 [1.11-1.16]	28,223	27,256	25,657	1,540	25,681
791	1.22 [1.17-1.27]	1.23 [1.18-1.28]	1.22 [1.17-1.28]	1.08 [0.92-1.28]	1.16 [1.11-1.21]	1.19 [1.14-1.24]	1.13 [1.08-1.18]	1.12 [1.07-1.17]	1.07 [0.91-1.27]	1.16 [1.11-1.21]	6,432	6,203	5,777	376	5,857
793	1.18 [1.06-1.32]	1.11 [0.99-1.24]	1.14 [1.01-1.29]	1.06 [0.77-1.44]	1.13 [1.01-1.26]	1.16 [1.04-1.29]	1.04 [0.92-1.17]	1.08 [0.96-1.22]	1.05 [0.76-1.45]	1.14 [1.02-1.28]	837	744	692	108	772
793.2	1.17 [1.09-1.25]	1.17 [1.09-1.25]	1.18 [1.09-1.27]	1.14 [0.90-1.44]	1.10 [1.02-1.18]	1.12 [1.04-1.20]	1.09 [1.01-1.17]	1.10 [1.02-1.18]	1.08 [0.84-1.38]	1.07 [0.99-1.15]	2,110	1,999	1,786	189	1,876
794	1.06 [0.95-1.19]	1.05 [0.94-1.18]	1.16 [0.87-1.54]	1.12 [0.87-1.44]	1.09 [0.96-1.23]	1.03 [0.92-1.16]	1.02 [0.90-1.15]	1.04 [0.77-1.40]	1.13 [0.87-1.46]	1.08 [0.95-1.23]	710	692	111	160	577
797	1.18 [1.02-1.38]	1.31 [1.12-1.54]	1.21 [1.03-1.43]		1.19 [1.02-1.39]	1.16 [0.99-1.35]	1.25 [1.06-1.47]	1.14 [0.96-1.35]		1.20 [1.03-1.41]	439	404	368	NA	401
797.1	1.09 [0.98-1.20]	1.08 [0.98-1.20]	1.06 [0.96-1.17]		1.07 [0.96-1.19]	1.10 [0.99-1.22]	1.05 [0.95-1.17]	1.02 [0.92-1.13]		1.10 [0.98-1.22]	948	939	911	NA	834
798	1.24 [1.21-1.27]	1.23 [1.20-1.26]	1.22 [1.19-1.25]	1.22 [1.16-1.29]	1.17 [1.14-1.19]	1.18 [1.15-1.20]	1.12 [1.10-1.15]	1.12 [1.09-1.14]	1.18 [1.11-1.25]	1.13 [1.10-1.16]	23,449	20,786	18,510	3,424	21,508
798.1	1.49 [1.40-1.60]	1.49 [1.39-1.60]	1.46 [1.34-1.59]	1.31 [1.12-1.54]	1.34 [1.25-1.44]	1.27 [1.18-1.36]	1.17 [1.09-1.26]	1.15 [1.05-1.26]	1.23 [1.04-1.44]	1.16 [1.08-1.25]	2,283	2,076	1,346	448	2,004
injuries & poisonings															
800	1.09 [1.06-1.13]	1.10 [1.06-1.15]	1.12 [1.08-1.17]	1.02 [0.94-1.09]	1.05 [1.01-1.09]	1.06 [1.03-1.10]	1.04 [1.00-1.08]	1.07 [1.02-1.11]	1.00 [0.93-1.08]	1.04 [1.00-1.08]	8,111	6,863	5,613	1,795	6,526
800.1	1.11 [1.08-1.13]	1.11 [1.09-1.14]	1.12 [1.09-1.14]	1.07 [0.85-1.36]	1.09 [1.07-1.12]	1.10 [1.08-1.12]	1.08 [1.06-1.11]	1.08 [1.06-1.11]	1.07 [0.83-1.37]	1.09 [1.07-1.12]	22,544	22,421	22,271	173	19,750
800.2	1.10 [1.08-1.12]	1.11 [1.09-1.14]	1.12 [1.10-1.14]	0.98 [0.90-1.07]	1.08 [1.06-1.11]	1.09 [1.07-1.11]	1.08 [1.06-1.11]	1.08 [1.06-1.11]	0.97 [0.89-1.06]	1.09 [1.06-1.11]	25,942	24,948	24,537	1,190	22,614
800.3	1.08 [1.05-1.12]	1.11 [1.07-1.16]	1.11 [1.06-1.16]	1.04 [0.99-1.10]	1.05 [1.01-1.09]	1.05 [1.02-1.09]	1.04 [1.00-1.09]	1.04 [0.99-1.10]	1.03 [0.97-1.08]	1.03 [0.99-1.08]	8,131	5,262	3,886	3,466	6,389
800.4	1.10 [1.02-1.18]	1.13 [1.05-1.21]	1.10 [1.02-1.20]	0.93 [0.77-1.12]	1.02 [0.94-1.10]	1.06 [0.99-1.14]	1.07 [0.99-1.15]	1.05 [0.96-1.14]	0.90 [0.74-1.09]	1.01 [0.93-1.09]	1,880	1,726	1,395	278	1,491
801	1.10 [1.06-1.15]	1.12 [1.07-1.17]	1.15 [1.09-1.21]	0.96 [0.88-1.05]	1.07 [1.03-1.12]	1.06 [1.01-1.10]	1.04 [0.99-1.08]	1.06 [1.01-1.12]	0.95 [0.87-1.04]	1.05 [1.00-1.10]	5,848	5,124	3,794	1,304	4,681
801.1	1.10 [0.94-1.30]	1.18 [0.99-1.40]	1.30 [1.04-1.63]	0.99 [0.73-1.33]	1.10 [0.92-1.33]	1.06 [0.90-1.25]	1.11 [0.92-1.32]	1.16 [0.92-1.47]	0.99 [0.73-1.35]	1.08 [0.89-1.30]	362	309	186	111	287
802	1.11 [1.07-1.16]	1.12 [1.08-1.17]	1.14 [1.09-1.19]	0.88 [0.74-1.05]	1.07 [1.02-1.11]	1.08 [1.04-1.13]	1.07 [1.03-1.12]	1.09 [1.04-1.13]	0.89 [0.74-1.07]	1.05 [1.01-1.10]	6,509	6,340	6,052	313	5,836
803	1.09 [1.02-1.16]	1.16 [1.06-1.26]	1.19 [1.08-1.31]	1.01 [0.92-1.11]	1.03 [0.96-1.11]	1.06 [0.99-1.13]	1.09 [1.00-1.19]	1.14 [1.03-1.26]	0.99 [0.89-1.09]	1.02 [0.95-1.10]	2,203	1,257	1,027	1,058	1,715
803.1	1.11 [1.08-1.14]	1.14 [1.10-1.18]	1.14 [1.09-1.18]	1.06 [1.01-1.11]	1.08 [1.05-1.12]	1.09 [1.05-1.12]	1.08 [1.04-1.12]	1.08 [1.04-1.12]	1.06 [1.00-1.11]	1.08 [1.04-1.11]	10,942	7,254	6,760	3,901	9,330
803.2	1.06 [1.04-1.08]	1.10 [1.07-1.13]	1.11 [1.07-1.14]	1.01 [0.98-1.04]	1.02 [1.00-1.05]	1.04 [1.02-1.06]	1.06 [1.03-1.09]	1.07 [1.03-1.10]	1.00 [0.97-1.03]	1.01 [0.99-1.04]	23,610	12,452	10,625	11,920	18,431
803.3	1.15 [1.11-1.20]	1.16 [1.12-1.21]	1.19 [1.14-1.24]	1.01 [0.90-1.12]	1.10 [1.05-1.14]	1.11 [1.07-1.15]	1.08 [1.04-1.13]	1.10 [1.06-1.15]	0.99 [0.89-1.11]	1.08 [1.03-1.12]	7,012	6,527	5,667	901	5,997
804	1.05 [1.02-1.08]	1.09 [1.05-1.12]	1.10 [1.06-1.15]	1.01 [0.98-1.05]	1.00 [0.97-1.03]	1.02 [0.99-1.05]	1.03 [0.99-1.06]	1.04 [1.00-1.09]	1.00 [0.96-1.03]	0.99 [0.96-1.02]	15,344	9,028	5,771	8,096	11,805
805	1.18 [1.14-1.23]	1.18 [1.14-1.22]	1.21 [1.17-1.26]	1.01 [0.89-1.15]	1.14 [1.10-1.19]	1.13 [1.09-1.18]	1.10 [1.06-1.14]	1.13 [1.08-1.17]	1.01 [0.89-1.15]	1.12 [1.08-1.16]	8,082	7,844	7,027	627	7,155
807	1.22 [1.17-1.27]	1.21 [1.16-1.26]	1.24 [1.19-1.29]	1.09 [0.91-1.29]	1.14 [1.10-1.19]	1.16 [1.12-1.21]	1.11 [1.07-1.16]	1.13 [1.08-1.18]	1.11 [0.93-1.33]	1.12 [1.07-1.17]	6,514	6,407	5,894	328	5,765
809	1.19 [1.10-1.28]	1.19 [1.10-1.28]	1.24 [1.14-1.35]	1.05 [0.83-1.34]	1.11 [1.03-1.21]	1.14 [1.06-1.24]	1.10 [1.02-1.20]	1.14 [1.05-1.25]	1.03 [0.81-1.33]	1.10 [1.01-1.20]	1,690	1,602	1,443	177	1,430
817	1.10 [1.00-1.21]	1.14 [1.00-1.30]	1.12 [0.94-1.33]	1.08 [0.95-1.24]	1.06 [0.95-1.18]	1.05 [0.95-1.16]	1.02 [0.89-1.17]	1.03 [0.86-1.22]	1.05 [0.92-1.20]	1.03 [0.92-1.15]	1,015	544	319	574	821
818	1.11 [1.06-1.16]	1.13 [1.07-1.18]	1.15 [1.09-1.21]	1.00 [0.87-1.14]	1.07 [1.01-1.12]	1.09 [1.04-1.14]	1.07 [1.02-1.13]	1.09 [1.04-1.15]	0.99 [0.86-1.14]	1.06 [1.01-1.11]	4,885	4,583	4,148	536	4,312
819	1.12 [1.10-1.15]	1.16 [1.13-1.20]	1.19 [1.16-1.23]	1.04 [1.00-1.08]	1.06 [1.03-1.09]	1.09 [1.07-1.12]	1.09 [1.06-1.12]	1.12 [1.08-1.16]	1.02 [0.98-1.06]	1.05 [1.02-1.07]	18,084	14,150	9,555	6,304	14,386
830	1.15 [1.11-1.19]	1.17 [1.13-1.22]	1.13 [1.07-1.19]	1.08 [1.02-1.15]	1.09 [1.05-1.13]	1.09 [1.05-1.13]	1.07 [1.03-1.11]	1.07 [1.01-1.13]	1.06 [0.99-1.13]	1.06 [1.02-1.10]	8,227	6,917	3,358	2,574	6,858
835	1.23 [1.20-1.25]	1.23 [1.21-1.26]	1.24 [1.21-1.27]	1.16 [1.10-1.22]	1.13 [1.10-1.16]	1.15 [1.12-1.17]	1.13 [1.11-1.16]	1.13 [1.10-1.16]	1.11 [1.05-1.17]	1.07 [1.05-1.10]	21,687	20,302	14,578	3,603	16,501
836	1.30 [1.18-1.42]	1.30 [1.18-1.43]	1.34 [1.21-1.48]		1.19 [1.08-1.31]	1.21 [1.10-1.34]	1.17 [1.06-1.29]	1.21 [1.09-1.34]		1.15 [1.04-1.27]	1,071	1,065	908	NA	967
840	1.22 [1.09-1.35]	1.19 [1.06-1.33]	1.16 [0.99-1.35]	1.21 [1.01-1.46]	1.14 [1.02-1.29]	1.15 [1.03-1.28]	1.09 [0.97-1.23]	1.07 [0.92-1.25]	1.16 [0.96-1.40]	1.10 [0.98-1.25]	860	722	399	299	664
840.3	1.03 [0.87-1.23]	1.01 [0.83-1.22]	0.98 [0.77-1.26]		1.02 [0.83-1.25]	1.00 [0.83-1.20]	0.94 [0.77-1.15]	0.95 [0.74-1.22]		1.01 [0.82-1.24]	291	246	145	NA	218
850	1.27 [1.24-1.30]	1.27 [1.24-1.31]	1.26 [1.22-1.30]	1.22 [1.15-1.30]	1.19 [1.16-1.22]	1.20 [1.17-1.23]	1.16 [1.13-1.19]	1.15 [1.12-1.19]	1.17 [1.10-1.25]	1.15 [1.12-1.18]	16,761	14,625	12,023	3,111	14,552
851	1.28 [1.26-1.30]	1.29 [1.27-1.31]	1.27 [1.25-1.29]	1.23 [1.18-1.27]	1.18 [1.17-1.20]	1.21 [1.19-1.22]	1.17 [1.16-1.19]	1.17 [1.15-1.18]	1.17 [1.13-1.22]	1.15 [1.13-1.16]	53,275	47,864	40,200	8,014	46,944
853	1.33 [1.24-1.42]	1.36 [1.26-1.46]	1.30 [1.20-1.40]	1.15 [0.90-1.48]	1.20 [1.11-1.29]	1.22 [1.14-1.32]	1.19 [1.10-1.28]	1.17 [1.08-1.27]	1.09 [0.84-1.40]	1.14 [1.06-1.23]	1,957	1,860	1,631	163	1,807
854	1.31 [1.27-1.35]	1.34 [1.30-1.38]	1.34 [1.29-1.38]	1.01 [0.92-1.12]	1.19 [1.16-1.23]	1.25 [1.21-1.29]	1.21 [1.17-1.25]	1.20 [1.16-1.25]	0.99 [0.90-1.10]	1.17 [1.13-1.21]	9,866	9,093	8,384	996	9,084
857	1.14 [1.10-1.18]	1.16 [1.12-1.20]	1.14 [1.09-1.18]	1.03 [0.87-1.21]	1.08 [1.04-1.12]	1.11 [1.07-1.15]	1.10 [1.06-1.14]	1.08 [1.04-1.12]	0.97 [0.82-1.15]	1.07 [1.03-1.11]	8,538	8,392	7,525	387	7,974
858	1.31 [1.27-1.34]	1.32 [1.28-1.35]	1.34 [1.30-1.38]	1.13 [1.03-1.24]	1.20 [1.17-1.24]	1.24 [1.20-1.27]	1.21 [1.18-1.24]	1.23 [1.20-1.27]	1.10 [1.00-1.21]	1.17 [1.14-1.20]	13,678	13,074	11,614	1,217	12,548
859	1.31 [1.27-1.34]	1.33 [1.29-1.37]	1.33 [1.29-1.38]	1.16 [1.08-1.25]	1.19 [1.16-1.23]	1.23 [1.19-1.27]	1.21 [1.17-1.25]	1.21 [1.17-1.25]	1.11 [1.03-1.20]	1.16 [1.12-1.19]	12,458	11,132	9,626	1,821	11,466
870	1.09 [1.01-1.18]	1.12 [1.02-1.23]	1.40 [1.24-1.58]	0.92 [0.81-1.04]	1.05 [0.96-1.14]	1.05 [0.96-1.14]	1.01 [0.92-1.12]	1.27 [1.11-1.44]	0.91 [0.80-1.03]	1.04 [0.95-1.14]	1,537	1,102	675	668	1,249
870.1	1.13 [1.08-1.19]	1.15 [1.08-1.22]	1.18 [1.10-1.25]	1.02 [0.93-1.12]	1.09 [1.03-1.15]	1.10 [1.05-1.16]	1.08 [1.02-1.15]	1.11 [1.04-1.18]	1.02 [0.93-1.12]	1.08 [1.02-1.14]	4,027	3,061	2,530	1,212	3,469
870.2	1.08 [0.99-1.19]	1.08 [0.96-1.21]	1.17 [1.02-1.34]	1.05 [0.93-1.20]	1.04 [0.94-1.15]	1.07 [0.98-1.17]	1.02 [0.91-1.15]	1.13 [0.98-1.29]	1.03 [0.90-1.18]	1.04 [0.94-1.15]	1,219	764	555	574	1,011
870.3	1.13 [1.11-1.15]	1.18 [1.16-1.21]	1.20 [1.18-1.23]	0.97 [0.94-1.01]	1.08 [1.06-1.11]	1.10 [1.08-1.12]	1.12 [1.09-1.14]	1.14 [1.11-1.17]	0.96 [0.93-1.00]	1.07 [1.05-1.09]	28,524	21,364	18,584	8,473	24,852
870.4	1.12 [1.05-1.20]	1.20 [1.11-1.29]	1.21 [1.11-1.31]	0.89 [0.77-1.03]	1.08 [1.00-1.16]	1.08 [1.01-1.16]	1.12 [1.03-1.21]	1.12 [1.03-1.22]	0.89 [0.77-1.03]	1.05 [0.97-1.14]	2,051	1,705	1,504	441	1,809
870.5	1.02 [0.98-1.07]	1.12 [1.05-1.21]	1.19 [1.09-1.31]	0.95 [0.90-1.00]	0.98 [0.94-1.03]	1.00 [0.96-1.05]	1.08 [1.00-1.16]	1.12 [1.02-1.23]	0.94 [0.89-1.00]	0.98 [0.93-1.03]	4,608	1,848	1,227	3,072	3,837
870.6	1.15 [1.05-1.27]	1.17 [1.05-1.29]	1.24 [1.10-1.40]	1.04 [0.88-1.24]	1.12 [1.02-1.24]	1.11 [1.01-1.22]	1.09 [0.98-1.21]	1.14 [1.00-1.29]	1.03 [0.86-1.23]	1.12 [1.01-1.24]	1,141	951	686	333	981
870.8	1.13 [1.00-1.28]	1.08 [0.89-1.31]	1.32 [1.00-1.74]	1.10 [0.94-1.27]	1.06 [0.92-1.22]	1.10 [0.97-1.24]	1.02 [0.83-1.24]	1.19 [0.90-1.58]	1.09 [0.93-1.27]	1.05 [0.91-1.21]	652	254	132	452	516
871	1.10 [1.08-1.12]	1.15 [1.12-1.18]	1.20 [1.17-1.24]	0.98 [0.95-1.01]	1.05 [1.03-1.08]	1.05 [1.03-1.07]	1.06 [1.04-1.08]	1.11 [1.08-1.14]	0.97 [0.94-1.01]	1.03 [1.01-1.05]	27,381	20,760	15,409	9,328	22,587
871.3	1.11 [1.02-1.21]	1.24 [1.11-1.40]	1.33 [1.15-1.53]	1.01 [0.90-1.14]	1.09 [0.99-1.20]	1.06 [0.97-1.16]	1.10 [0.98-1.24]	1.19 [1.03-1.38]	0.99 [0.88-1.12]	1.05 [0.95-1.16]	1,299	727	477	700	1,081
871.4	1.12 [0.99-1.27]	1.24 [1.06-1.45]	1.33 [1.12-1.58]	0.94 [0.77-1.14]	1.07 [0.93-1.22]	1.09 [0.96-1.24]	1.13 [0.96-1.32]	1.19 [0.99-1.42]	0.95 [0.78-1.17]	1.05 [0.92-1.21]	640	424	351	253	555
872	0.99 [0.91-1.07]	0.98 [0.88-1.09]	1.01 [0.88-1.15]	0.98 [0.87-1.11]	0.94 [0.85-1.03]	0.99 [0.91-1.08]	0.97 [0.87-1.09]	1.01 [0.88-1.16]	0.97 [0.86-1.10]	0.94 [0.85-1.04]	1,306	732	497	685	995
873	1.13 [1.05-1.23]	1.26 [1.14-1.39]	1.36 [1.21-1.53]	1.00 [0.89-1.12]	1.05 [0.97-1.15]	1.11 [1.02-1.20]	1.15 [1.04-1.28]	1.25 [1.10-1.41]	1.00 [0.89-1.13]	1.04 [0.95-1.13]	1,602	1,037	686	720	1,327
874	1.35 [1.20-1.52]	1.30 [1.16-1.47]	1.28 [1.13-1.45]		1.21 [1.07-1.36]	1.25 [1.11-1.41]	1.14 [1.01-1.29]	1.08 [0.95-1.23]		1.17 [1.04-1.32]	716	681	622	NA	663
905	1.28 [1.10-1.49]	1.29 [1.11-1.51]	1.31 [1.08-1.59]		1.14 [0.96-1.34]	1.20 [1.03-1.40]	1.19 [1.02-1.40]	1.17 [0.96-1.44]		1.09 [0.92-1.29]	419	389	250	NA	337
907	1.10 [1.05-1.16]	1.13 [1.07-1.19]	1.16 [1.09-1.24]	0.99 [0.91-1.09]	1.02 [0.97-1.08]	1.07 [1.01-1.12]	1.04 [0.99-1.10]	1.07 [1.01-1.15]	1.00 [0.90-1.10]	1.00 [0.95-1.06]	4,011	3,421	2,275	1,144	3,175
913	1.10 [0.88-1.36]	0.98 [0.77-1.26]	0.95 [0.72-1.25]		1.00 [0.78-1.28]	1.02 [0.81-1.27]	0.88 [0.68-1.13]	0.88 [0.66-1.16]		0.94 [0.73-1.21]	199	145	111	NA	151
915	1.15 [1.13-1.16]	1.19 [1.17-1.21]	1.21 [1.19-1.23]	0.99 [0.96-1.02]	1.09 [1.07-1.11]	1.10 [1.09-1.12]	1.10 [1.08-1.11]	1.12 [1.10-1.14]	0.97 [0.95-1.00]	1.07 [1.05-1.08]	47,549	38,385	33,586	11,393	42,090
916	1.16 [1.13-1.19]	1.18 [1.15-1.22]	1.20 [1.17-1.24]	0.97 [0.91-1.04]	1.10 [1.07-1.13]	1.11 [1.08-1.14]	1.09 [1.06-1.13]	1.12 [1.08-1.15]	0.96 [0.90-1.03]	1.08 [1.05-1.11]	13,528	11,824	10,631	2,224	12,240
930	5.58 [5.28-5.90]	2.80 [2.54-3.08]	2.02 [1.73-2.34]	7.34 [6.88-7.84]	5.48 [5.15-5.82]	4.37 [4.11-4.63]	1.81 [1.62-2.02]	1.57 [1.34-1.85]	5.82 [5.42-6.25]	4.27 [4.01-4.56]	6,437	1,302	460	5,577	5,437
941	1.53 [1.40-1.66]	1.45 [1.33-1.60]	1.40 [1.27-1.54]	1.85 [1.52-2.25]	1.43 [1.31-1.56]	1.39 [1.27-1.51]	1.27 [1.16-1.40]	1.24 [1.12-1.38]	1.71 [1.40-2.10]	1.32 [1.21-1.45]	1,423	1,192	1,037	298	1,306
942	1.70 [1.28-2.25]				1.66 [1.25-2.21]	1.70 [1.28-2.27]				1.70 [1.27-2.29]	137	NA	NA	NA	130
946	1.96 [1.84-2.08]	1.76 [1.64-1.89]	1.66 [1.54-1.80]	2.76 [2.44-3.11]	1.81 [1.69-1.93]	1.68 [1.58-1.79]	1.44 [1.34-1.54]	1.40 [1.29-1.53]	2.33 [2.05-2.64]	1.60 [1.49-1.71]	2,962	2,249	1,619	968	2,562
949	2.22 [2.08-2.37]	1.91 [1.76-2.07]	1.71 [1.55-1.90]	2.71 [2.46-2.98]	2.07 [1.93-2.22]	1.91 [1.79-2.05]	1.58 [1.45-1.73]	1.47 [1.32-1.64]	2.32 [2.10-2.57]	1.81 [1.68-1.95]	2,834	1,569	948	1,480	2,350
949.1	1.05 [0.90-1.22]	1.08 [0.83-1.40]	1.00 [0.77-1.31]	1.01 [0.84-1.21]	1.01 [0.86-1.18]	1.01 [0.87-1.18]	1.02 [0.79-1.33]	0.96 [0.73-1.25]	0.95 [0.78-1.15]	0.99 [0.84-1.16]	424	164	160	264	405
952	1.03 [0.88-1.20]	1.04 [0.89-1.22]	1.10 [0.93-1.32]		1.00 [0.85-1.19]	1.02 [0.88-1.19]	1.00 [0.85-1.17]	1.06 [0.89-1.27]		1.00 [0.84-1.18]	422	382	303	NA	341
957	1.31 [1.19-1.45]	1.35 [1.21-1.49]	1.33 [1.20-1.47]		1.20 [1.08-1.34]	1.22 [1.10-1.36]	1.20 [1.08-1.33]	1.17 [1.05-1.30]		1.16 [1.04-1.29]	939	936	905	NA	872
958	1.09 [1.03-1.17]	1.11 [1.04-1.18]	1.12 [1.04-1.21]	1.05 [0.90-1.22]	1.04 [0.98-1.12]	1.07 [1.00-1.14]	1.04 [0.97-1.11]	1.07 [0.99-1.15]	1.03 [0.89-1.21]	1.04 [0.97-1.12]	2,569	2,323	1,932	438	2,157
958.1	1.18 [0.92-1.50]	1.16 [0.91-1.49]	1.12 [0.87-1.45]		1.17 [0.91-1.51]	1.13 [0.88-1.45]	1.10 [0.85-1.41]	1.07 [0.82-1.39]		1.17 [0.90-1.52]	160	150	139	NA	140
958.2	1.05 [0.91-1.20]	1.09 [0.95-1.26]	1.09 [0.93-1.29]	1.12 [0.85-1.49]	1.04 [0.90-1.21]	1.00 [0.87-1.16]	1.01 [0.87-1.17]	1.02 [0.86-1.22]	1.08 [0.81-1.44]	1.03 [0.89-1.20]	515	463	342	131	430
960	1.62 [1.58-1.65]	1.63 [1.59-1.66]	1.64 [1.60-1.68]	1.42 [1.33-1.51]	1.49 [1.46-1.53]	1.40 [1.37-1.43]	1.36 [1.33-1.39]	1.37 [1.33-1.40]	1.31 [1.23-1.40]	1.33 [1.30-1.36]	24,400	22,914	18,975	2,787	22,398
960.1	1.52 [1.32-1.76]	1.64 [1.40-1.93]	1.76 [1.44-2.14]	1.30 [1.04-1.64]	1.39 [1.19-1.61]	1.38 [1.19-1.59]	1.38 [1.16-1.63]	1.44 [1.17-1.78]	1.24 [0.98-1.57]	1.29 [1.11-1.51]	521	392	267	193	450
960.2	1.44 [1.42-1.46]	1.45 [1.43-1.47]	1.46 [1.44-1.48]	1.37 [1.33-1.41]	1.36 [1.35-1.38]	1.32 [1.31-1.34]	1.29 [1.27-1.30]	1.30 [1.28-1.32]	1.28 [1.24-1.32]	1.27 [1.25-1.29]	75,992	68,701	52,815	12,829	62,561
961	1.52 [1.42-1.62]	1.54 [1.44-1.65]	1.52 [1.41-1.64]	1.27 [1.05-1.53]	1.41 [1.32-1.50]	1.33 [1.24-1.42]	1.28 [1.19-1.37]	1.26 [1.16-1.36]	1.21 [0.99-1.47]	1.28 [1.19-1.37]	2,474	2,315	1,859	312	2,307
961.1	1.59 [1.49-1.69]	1.56 [1.47-1.67]	1.58 [1.47-1.69]		1.46 [1.36-1.56]	1.39 [1.30-1.49]	1.32 [1.23-1.41]	1.33 [1.24-1.43]		1.32 [1.23-1.41]	2,515	2,482	2,241	NA	2,273
962	1.26 [1.16-1.38]	1.34 [1.22-1.48]	1.35 [1.20-1.52]	1.04 [0.88-1.23]	1.15 [1.05-1.26]	1.15 [1.05-1.26]	1.10 [0.99-1.22]	1.10 [0.97-1.25]	1.02 [0.86-1.21]	1.10 [1.00-1.21]	1,223	986	696	370	1,122
962.3	1.60 [1.49-1.71]	1.60 [1.49-1.72]	1.55 [1.43-1.67]	1.37 [1.13-1.67]	1.44 [1.34-1.55]	1.38 [1.28-1.48]	1.30 [1.21-1.40]	1.27 [1.17-1.37]	1.29 [1.05-1.58]	1.28 [1.19-1.37]	2,226	2,047	1,753	290	2,061
963	1.23 [1.19-1.27]	1.24 [1.20-1.28]	1.22 [1.17-1.27]	1.17 [1.08-1.26]	1.15 [1.11-1.19]	1.18 [1.14-1.22]	1.16 [1.12-1.21]	1.16 [1.11-1.20]	1.12 [1.04-1.22]	1.14 [1.10-1.18]	8,518	7,403	6,255	1,601	7,406
963.1	1.33 [1.05-1.70]	1.56 [1.18-2.04]			1.15 [0.89-1.48]	1.28 [1.00-1.64]	1.45 [1.10-1.91]			1.11 [0.86-1.45]	167	132	NA	NA	144
964	1.24 [1.17-1.32]	1.25 [1.18-1.34]	1.25 [1.17-1.34]	1.07 [0.89-1.28]	1.17 [1.10-1.24]	1.18 [1.11-1.26]	1.15 [1.08-1.23]	1.14 [1.07-1.22]	1.07 [0.89-1.29]	1.13 [1.06-1.21]	2,723	2,514	2,302	305	2,523
964.1	1.27 [1.18-1.36]	1.28 [1.19-1.37]	1.27 [1.18-1.37]		1.19 [1.11-1.28]	1.21 [1.13-1.30]	1.17 [1.09-1.26]	1.16 [1.08-1.25]		1.15 [1.07-1.24]	2,103	2,073	2,008	NA	1,996
965	1.31 [1.29-1.33]	1.35 [1.33-1.37]	1.41 [1.38-1.43]	1.08 [1.05-1.12]	1.22 [1.20-1.24]	1.18 [1.16-1.20]	1.14 [1.12-1.16]	1.19 [1.16-1.21]	1.05 [1.02-1.09]	1.13 [1.11-1.15]	39,157	33,202	24,181	9,715	34,065
965.1	1.38 [1.34-1.42]	1.39 [1.35-1.44]	1.37 [1.32-1.42]	1.26 [1.12-1.42]	1.27 [1.23-1.31]	1.22 [1.18-1.26]	1.17 [1.13-1.21]	1.16 [1.12-1.21]	1.18 [1.04-1.34]	1.16 [1.12-1.20]	10,675	10,555	8,596	759	10,260
965.3	1.17 [1.08-1.26]	1.24 [1.14-1.34]	1.25 [1.13-1.38]	0.93 [0.81-1.07]	1.07 [0.99-1.17]	1.12 [1.03-1.21]	1.10 [1.01-1.19]	1.12 [1.01-1.24]	0.93 [0.81-1.08]	1.06 [0.97-1.15]	1,698	1,436	969	467	1,405
966	1.25 [1.18-1.32]	1.29 [1.22-1.36]	1.33 [1.24-1.43]	1.00 [0.89-1.13]	1.11 [1.05-1.17]	1.14 [1.08-1.20]	1.07 [1.00-1.13]	1.10 [1.03-1.19]	1.02 [0.90-1.15]	1.08 [1.02-1.14]	3,375	3,007	1,949	724	3,019
967	1.38 [1.29-1.47]	1.35 [1.26-1.45]	1.34 [1.23-1.46]	1.26 [1.12-1.43]	1.24 [1.16-1.32]	1.23 [1.15-1.31]	1.15 [1.07-1.23]	1.13 [1.03-1.23]	1.19 [1.05-1.35]	1.15 [1.07-1.23]	2,483	1,986	1,289	744	2,243
969	1.16 [1.12-1.19]	1.20 [1.16-1.24]	1.31 [1.25-1.37]	0.97 [0.92-1.01]	1.06 [1.03-1.09]	1.08 [1.05-1.11]	1.01 [0.98-1.04]	1.08 [1.03-1.13]	0.97 [0.92-1.02]	1.04 [1.01-1.08]	11,596	9,490	4,536	4,368	9,695
971	1.19 [1.13-1.26]	1.25 [1.18-1.32]	1.25 [1.17-1.33]	1.01 [0.89-1.15]	1.14 [1.08-1.20]	1.15 [1.08-1.21]	1.13 [1.07-1.20]	1.13 [1.06-1.21]	0.99 [0.87-1.13]	1.13 [1.07-1.20]	3,453	3,086	2,498	615	3,113
972	1.30 [1.24-1.35]	1.32 [1.27-1.38]	1.30 [1.24-1.35]	0.98 [0.81-1.18]	1.22 [1.17-1.27]	1.23 [1.18-1.28]	1.19 [1.14-1.24]	1.17 [1.12-1.22]	0.95 [0.79-1.15]	1.18 [1.13-1.23]	6,146	5,946	5,731	285	5,717
972.2	1.39 [1.12-1.72]	1.34 [1.08-1.66]	1.38 [1.11-1.71]		1.27 [1.01-1.58]	1.34 [1.08-1.66]	1.23 [0.99-1.54]	1.29 [1.04-1.61]		1.26 [1.00-1.58]	216	215	214	NA	198
973	1.27 [1.17-1.37]	1.33 [1.23-1.45]	1.34 [1.22-1.46]	1.02 [0.86-1.22]	1.20 [1.11-1.30]	1.15 [1.06-1.24]	1.11 [1.02-1.21]	1.13 [1.03-1.25]	1.00 [0.83-1.19]	1.13 [1.05-1.23]	1,780	1,555	1,266	347	1,643
974	1.43 [1.32-1.54]	1.44 [1.33-1.55]	1.41 [1.30-1.52]		1.30 [1.20-1.41]	1.33 [1.23-1.44]	1.28 [1.18-1.38]	1.25 [1.15-1.36]		1.25 [1.15-1.35]	1,860	1,795	1,733	NA	1,708
975	1.33 [1.21-1.47]	1.41 [1.25-1.59]	1.56 [1.31-1.86]	1.16 [1.00-1.34]	1.23 [1.11-1.37]	1.19 [1.08-1.32]	1.18 [1.04-1.33]	1.26 [1.05-1.52]	1.10 [0.95-1.28]	1.11 [0.99-1.24]	1,052	729	331	488	889
976	1.25 [1.09-1.43]	1.75 [1.43-2.14]	1.84 [1.44-2.37]	1.01 [0.84-1.21]	1.24 [1.07-1.43]	1.17 [1.02-1.35]	1.48 [1.20-1.82]	1.56 [1.21-2.03]	0.97 [0.81-1.16]	1.19 [1.02-1.38]	527	265	178	290	465
979	1.31 [1.22-1.40]	1.38 [1.28-1.49]	1.46 [1.33-1.61]	1.04 [0.92-1.17]	1.19 [1.10-1.28]	1.19 [1.11-1.28]	1.14 [1.06-1.24]	1.20 [1.08-1.32]	1.01 [0.89-1.15]	1.13 [1.05-1.22]	2,180	1,741	1,134	656	1,859
981	1.13 [1.09-1.18]	1.16 [1.11-1.21]	1.26 [1.18-1.35]	0.96 [0.90-1.03]	1.05 [1.00-1.10]	1.06 [1.02-1.11]	0.98 [0.93-1.02]	1.04 [0.97-1.11]	0.98 [0.91-1.05]	1.05 [1.00-1.09]	5,797	5,029	2,102	2,038	4,832
983	0.98 [0.82-1.16]	1.01 [0.80-1.29]		0.86 [0.69-1.06]	0.95 [0.79-1.15]	0.96 [0.80-1.14]	0.94 [0.73-1.20]		0.85 [0.68-1.06]	0.94 [0.78-1.14]	338	162	NA	215	271
985	1.16 [0.91-1.49]	1.17 [0.89-1.53]	1.17 [0.87-1.58]		1.10 [0.85-1.42]	1.10 [0.85-1.41]	1.05 [0.80-1.39]	1.06 [0.78-1.44]		1.11 [0.86-1.44]	153	131	102	NA	138
986	1.08 [0.81-1.43]					1.07 [0.80-1.43]					115	NA	NA	NA	NA
987	1.05 [0.90-1.23]	1.08 [0.91-1.27]	1.12 [0.93-1.36]	1.04 [0.77-1.41]	1.04 [0.89-1.22]	1.02 [0.87-1.19]	0.97 [0.82-1.16]	1.02 [0.84-1.25]	1.05 [0.77-1.43]	1.03 [0.87-1.21]	405	330	256	102	360
988	1.31 [1.05-1.63]			1.12 [0.84-1.48]	1.22 [0.95-1.56]	1.23 [0.98-1.53]			1.08 [0.81-1.44]	1.15 [0.90-1.48]	204	NA	NA	117	165
989	0.99 [0.87-1.13]	1.11 [0.89-1.38]	1.21 [0.94-1.58]	0.92 [0.78-1.08]	0.92 [0.80-1.06]	0.95 [0.82-1.09]	0.95 [0.76-1.19]	1.05 [0.80-1.38]	0.89 [0.76-1.05]	0.90 [0.78-1.05]	503	192	139	337	438
990	1.17 [1.14-1.20]	1.17 [1.14-1.20]	1.16 [1.13-1.19]	1.09 [0.91-1.30]	1.09 [1.07-1.12]	1.14 [1.11-1.17]	1.10 [1.08-1.13]	1.10 [1.07-1.13]	1.13 [0.94-1.36]	1.10 [1.07-1.13]	15,940	15,749	15,211	329	15,016
994.2	1.24 [1.22-1.26]	1.25 [1.23-1.27]	1.25 [1.22-1.27]	1.09 [1.01-1.17]	1.19 [1.16-1.21]	1.20 [1.18-1.22]	1.16 [1.14-1.19]	1.16 [1.14-1.18]	1.06 [0.98-1.14]	1.17 [1.15-1.19]	34,356	33,202	31,291	1,833	31,894
other															
1000	1.00 [0.95-1.04]	1.11 [1.04-1.19]	1.13 [1.05-1.23]	0.93 [0.87-0.98]	0.96 [0.92-1.01]	0.98 [0.93-1.02]	1.04 [0.97-1.12]	1.05 [0.97-1.14]	0.92 [0.86-0.98]	0.96 [0.91-1.01]	4,449	2,160	1,498	2,585	3,769
1001	1.12 [1.09-1.16]	1.31 [1.25-1.38]	1.30 [1.22-1.37]	1.02 [0.98-1.07]	1.06 [1.02-1.10]	1.07 [1.04-1.11]	1.18 [1.13-1.25]	1.18 [1.11-1.25]	1.00 [0.96-1.05]	1.03 [0.99-1.07]	8,527	3,990	3,011	4,947	7,126
1002	1.27 [1.24-1.29]	1.27 [1.25-1.29]	1.27 [1.24-1.29]	1.15 [1.09-1.22]	1.20 [1.18-1.22]	1.20 [1.17-1.22]	1.16 [1.13-1.18]	1.15 [1.13-1.18]	1.11 [1.05-1.18]	1.16 [1.14-1.18]	32,744	30,266	27,215	3,638	29,116
1004	1.19 [1.15-1.23]	1.18 [1.13-1.22]	1.27 [1.21-1.33]	1.06 [1.01-1.12]	1.11 [1.07-1.15]	1.11 [1.07-1.15]	1.01 [0.97-1.05]	1.09 [1.03-1.14]	1.04 [0.99-1.10]	1.09 [1.05-1.12]	9,786	6,775	4,516	4,166	8,760
1005	1.28 [1.24-1.33]	1.31 [1.26-1.36]	1.31 [1.25-1.36]	1.05 [0.94-1.16]	1.21 [1.17-1.26]	1.22 [1.18-1.27]	1.20 [1.16-1.25]	1.20 [1.15-1.25]	1.02 [0.92-1.14]	1.18 [1.14-1.23]	7,534	6,857	6,110	890	6,933

Supplementary Table 6: phecode-mapped results (hazard ratios and events from all cohorts)

	Hazard ratio (99% confidence interval)										Events (in exposed)				
	crude					adjusted									
Outcome	any age	18+	40+	<18	hosp.	any age	18+	40+	<18	hosp.	any age	18+	40+	<18	hosp.
1006	0.92 [0.82-1.03]	0.89 [0.75-1.05]	0.90 [0.72-1.11]	0.94 [0.81-1.08]	0.91 [0.80-1.03]	0.91 [0.81-1.02]	0.87 [0.74-1.04]	0.88 [0.70-1.09]	0.93 [0.80-1.09]	0.91 [0.80-1.04]	684	304	174	431	542
1007	1.09 [0.99-1.19]	1.12 [1.01-1.24]	1.16 [1.02-1.32]	1.00 [0.85-1.17]	1.02 [0.92-1.13]	1.08 [0.98-1.19]	1.07 [0.97-1.19]	1.11 [0.98-1.27]	1.02 [0.87-1.21]	1.04 [0.93-1.16]	1,122	929	553	400	858
1008	1.11 [1.06-1.16]	1.13 [1.07-1.18]	1.16 [1.10-1.23]	1.01 [0.92-1.10]	1.05 [1.00-1.11]	1.08 [1.03-1.13]	1.06 [1.01-1.11]	1.09 [1.03-1.15]	1.01 [0.93-1.11]	1.05 [1.00-1.10]	5,009	4,318	3,179	1,306	4,128
1009	1.16 [1.15-1.18]	1.20 [1.18-1.22]	1.23 [1.21-1.26]	1.06 [1.03-1.09]	1.10 [1.09-1.12]	1.11 [1.09-1.13]	1.10 [1.08-1.12]	1.13 [1.10-1.15]	1.04 [1.01-1.07]	1.07 [1.06-1.09]	45,692	35,468	26,227	14,115	38,103
1010	1.44 [1.42-1.46]	1.33 [1.31-1.35]	1.33 [1.31-1.35]	1.97 [1.91-2.03]	1.35 [1.34-1.37]	1.35 [1.33-1.37]	1.22 [1.20-1.23]	1.22 [1.20-1.24]	1.74 [1.68-1.79]	1.29 [1.28-1.31]	60,388	50,680	37,629	13,628	53,513
1011	1.29 [1.26-1.33]	1.30 [1.27-1.34]	1.29 [1.25-1.33]	1.20 [1.12-1.28]	1.21 [1.17-1.24]	1.21 [1.18-1.24]	1.17 [1.14-1.21]	1.17 [1.13-1.21]	1.15 [1.07-1.24]	1.16 [1.12-1.19]	13,419	11,896	9,589	2,259	11,695
1012	0.97 [0.79-1.19]	1.32 [1.00-1.73]		0.79 [0.59-1.06]	1.06 [0.86-1.31]	0.96 [0.78-1.18]	1.27 [0.96-1.68]		0.81 [0.60-1.09]	1.08 [0.87-1.34]	222	125	NA	106	204
1013	1.25 [1.17-1.33]	1.25 [1.17-1.33]	1.27 [1.18-1.36]	1.14 [0.95-1.38]	1.18 [1.11-1.26]	1.20 [1.12-1.28]	1.14 [1.07-1.22]	1.16 [1.08-1.25]	1.15 [0.95-1.39]	1.16 [1.08-1.24]	2,516	2,300	2,119	293	2,317
1014	1.00 [0.95-1.06]	1.12 [1.02-1.22]	1.09 [0.99-1.21]	0.96 [0.90-1.04]	0.98 [0.92-1.04]	0.99 [0.93-1.05]	1.05 [0.96-1.15]	1.03 [0.93-1.14]	0.96 [0.89-1.03]	0.97 [0.91-1.04]	2,854	1,270	983	1,724	2,470
1015	1.47 [1.45-1.50]	1.33 [1.31-1.36]	1.35 [1.32-1.38]	1.66 [1.62-1.71]	1.35 [1.33-1.38]	1.34 [1.32-1.37]	1.15 [1.12-1.17]	1.18 [1.15-1.21]	1.54 [1.50-1.59]	1.28 [1.26-1.31]	36,043	24,828	15,848	15,319	30,824
1019	1.24 [1.22-1.25]	1.24 [1.22-1.25]	1.23 [1.21-1.25]	1.18 [1.14-1.23]	1.16 [1.14-1.17]	1.19 [1.17-1.20]	1.14 [1.13-1.16]	1.15 [1.13-1.17]	1.14 [1.10-1.19]	1.13 [1.11-1.15]	45,241	40,851	33,837	6,336	37,400
1100	1.23 [1.21-1.27]	1.22 [1.19-1.26]	1.20 [1.16-1.25]	1.24 [1.19-1.29]	1.17 [1.14-1.20]	1.17 [1.14-1.20]	1.13 [1.10-1.16]	1.11 [1.07-1.15]	1.19 [1.14-1.24]	1.13 [1.10-1.16]	17,209	13,223	6,277	6,352	14,733

Supplementary Table 7: Strongest associations per category from phecode-mapped results

phecode	phenotype	crude HR (99%CI)	events
circulatory system			
454.11	Varicose veins of lower ext...	2.21 [2.12-2.3]	8,205
456	Chronic venous insufficienc...	1.88 [1.76-2.01]	2,673
454.1	Varicose veins of lower ext...	1.63 [1.59-1.67]	19,256
443.1	Raynaud's syndrome	1.59 [1.52-1.66]	5,376
446.9	Arteritis NOS	1.59 [1.45-1.74]	1,278
450	Noninfectious disorders of ...	1.52 [1.46-1.59]	5,602
congenital anomalies			
758.1	Chromosomal anomalies	1.71 [1.59-1.84]	2,051
756.3	Congenital anomalies of mus...	1.43 [1.28-1.61]	797
757	Congenital anomalies of the...	1.36 [1.22-1.51]	857
752.11	Spina bifida	1.3 [1.17-1.44]	964
756.5	Congenital osteodystrophies	1.27 [1.18-1.37]	1,865
755.1	Congenital deformities of feet	1.26 [1.15-1.37]	1,329
dermatologic			
690	Erythematosquamous dermatosis	27.42 [23.35-32.19]	2,347
939	Atopic/contact dermatitis d...	7.81 [7.68-7.95]	84,723
690.1	Seborrheic dermatitis	3.29 [2.87-3.77]	776
695.7	Prurigo and Lichen	3 [2.81-3.19]	3,243
695.22	Pemphigus and pemphigoid	2.81 [2.55-3.09]	1,545
696.41	Psoriasis vulgaris	2.3 [2.23-2.37]	12,400
digestive			
557	Intestinal malabsorption (n...	1.78 [1.72-1.85]	8,443
555.1	Regional enteritis	1.7 [1.63-1.77]	6,324
555.21	Ulcerative colitis (chronic)	1.62 [1.52-1.73]	2,605
561.1	Diarrhea	1.62 [1.51-1.74]	2,038
557.1	Celiac disease	1.57 [1.5-1.64]	5,403
556.1	Ulceration of intestine	1.54 [1.45-1.63]	2,973
endocrine/metabolic			
255.21	Glucocorticoid deficiency	1.72 [1.6-1.85]	2,070
250.14	Type 1 diabetes with neurol...	1.52 [1.36-1.71]	762
279.1	Immunity deficiency	1.52 [1.38-1.67]	1,161
253.7	Other disorders of neurohyp...	1.52 [1.42-1.63]	2,343
279.11	Deficiency of humoral immunity	1.51 [1.34-1.7]	744
250.12	Type 1 diabetes with renal ...	1.51 [1.32-1.73]	527
genitourinary			
624.1	Dystrophy of female genital...	1.64 [1.48-1.83]	927
580.12	Non-proliferative glomerulo...	1.54 [1.35-1.75]	595
580.32	Nephritis and nephropathy w...	1.47 [1.36-1.59]	1,569
604.1	Redundant prepuce and phimo...	1.46 [1.42-1.5]	16,264
601.4	Balanoposthitis	1.45 [1.35-1.56]	1,874
592.13	Chronic interstitial cystitis	1.45 [1.28-1.64]	657
hematopoietic			
288.3	Eosinophilia	2.09 [1.82-2.4]	636
283.1	Autoimmune hemolytic anemias	1.57 [1.36-1.81]	517
281.9	Deficiency anemias	1.48 [1.32-1.66]	835
283	Acquired hemolytic anemias	1.44 [1.28-1.62]	745
288.2	Elevated white blood cell c...	1.43 [1.34-1.53]	2,421
285.2	Anemia of chronic disease	1.43 [1.34-1.52]	2,731
infectious diseases			
54	Herpes simplex	2.46 [2.33-2.6]	3,948
132	Infestation (lice, mites)	2.28 [1.99-2.61]	669
110.12	Althete's foot	1.89 [1.66-2.16]	648
110.2	Dermatomycoses	1.87 [1.63-2.14]	651
110.1	Dermatophytosis	1.73 [1.61-1.86]	2,264
117.4	Aspergillosis	1.68 [1.51-1.88]	857
injuries & poisonings			
930	Allergic reaction to food	5.58 [5.28-5.9]	6,437
949	Allergies, other	2.22 [2.08-2.37]	2,834
946	Anaphylactic shock NOS	1.96 [1.84-2.08]	2,962
960	Poisoning by antibiotics	1.62 [1.58-1.65]	24,400
962.3	Hormones and synthetic subs...	1.6 [1.49-1.71]	2,226
961.1	Poisoning/allergy of sulfon...	1.59 [1.49-1.69]	2,515

phecode	phenotype	crude HR (99%CI)	events
mental disorders			
300.3	Obsessive-compulsive disorders	1.64 [1.52-1.76]	2,087
315.3	Mental retardation	1.58 [1.49-1.67]	3,630
315.1	Learning disorder	1.56 [1.5-1.62]	8,341
302	Sexual and gender identity ...	1.49 [1.33-1.68]	707
317.11	Alcoholic liver damage	1.44 [1.38-1.51]	5,266
303.4	Somatoform disorder	1.44 [1.34-1.55]	1,793
musculoskeletal			
729.1	Rheumatism, unspecified and...	1.68 [1.54-1.83]	1,338
726.3	Bursitis	1.51 [1.41-1.62]	2,124
715.2	Ankylosing spondylitis	1.5 [1.39-1.62]	1,810
711.1	Pyogenic arthritis	1.48 [1.39-1.59]	2,261
716	Other arthropathies	1.48 [1.31-1.68]	648
714	Rheumatoid arthritis and ot...	1.46 [1.36-1.56]	2,226
neoplasms			
201	Hodgkin's disease	1.71 [1.54-1.9]	969
217.1	Nevus, non-neoplastic	1.34 [1.24-1.45]	1,597
202	Cancer of other lymphoid, h...	1.33 [1.26-1.4]	3,821
202.2	Non-Hodgkins lymphoma	1.32 [1.27-1.38]	4,824
204.21	Myeloid leukemia, acute	1.32 [1.21-1.44]	1,349
202.24	Large cell lymphoma	1.31 [1.22-1.42]	1,690
neurological			
327.3	Sleep apnea	1.51 [1.48-1.55]	20,679
327	Sleep disorders	1.48 [1.45-1.51]	23,995
338.2	Chronic pain	1.46 [1.37-1.56]	2,496
345.12	Partial epilepsy	1.46 [1.33-1.59]	1,394
352.1	Trigeminal nerve disorders ...	1.42 [1.32-1.53]	1,837
338.1	Acute pain	1.4 [1.3-1.51]	1,800
other			
1,015	Effects of other external c...	1.47 [1.45-1.5]	36,043
1,010	Other tests	1.44 [1.42-1.46]	60,388
1,011	Complications of surgical a...	1.29 [1.26-1.33]	13,419
1,005	Other symptoms	1.28 [1.24-1.33]	7,534
1,002	Symptoms concerning nutriti...	1.27 [1.24-1.29]	32,744
1,013	Asphyxia and hypoxemia	1.25 [1.17-1.33]	2,516
pregnancy complications			
649	Other conditions or status ...	1.21 [1.17-1.25]	9,473
668	Complications of the admini...	1.2 [1.06-1.37]	626
674	Other complications of the ...	1.19 [1.14-1.25]	5,402
656	Other perinatal conditions ...	1.18 [1.14-1.22]	9,997
646	Other complications of preg...	1.18 [1.16-1.2]	31,167
636	Early or threatened labor; ...	1.16 [1.12-1.21]	6,347
respiratory			
495.2	Asthma with exacerbation	2.95 [2.79-3.13]	3,968
505	Other pulmonary inflamation...	2.4 [2.08-2.78]	600
476	Allergic rhinitis	2.14 [2.08-2.2]	17,750
495	Asthma	2.12 [2.11-2.14]	165,066
512.1	Wheezing	1.96 [1.92-2]	29,127
472	Chronic pharyngitis and nas...	1.75 [1.66-1.86]	3,383
sense organs			
364.41	Keratoconus	2.36 [2.13-2.62]	1,203
364.9	Cornea replaced by transplant	1.73 [1.5-2]	507
370	Keratitis	1.72 [1.58-1.87]	1,569
370.1	Corneal ulcer	1.69 [1.51-1.9]	802
380.1	Otitis externa	1.6 [1.52-1.7]	3,397
369.2	Eye infection, viral	1.54 [1.39-1.71]	985
symptoms			
798.1	Chronic fatigue syndrome	1.49 [1.4-1.6]	2,283
771.1	Swelling of limb	1.4 [1.37-1.43]	21,683
770	Myalgia and myositis unspec...	1.37 [1.3-1.44]	4,383
773	Pain in limb	1.37 [1.34-1.4]	25,521
771	Musculoskeletal symptoms re...	1.36 [1.28-1.44]	2,936
761	Cervicalgia	1.34 [1.3-1.39]	7,994

Supplementary Table 8: Largest differences in crude hazard ratios between any-age and <18 cohorts					
	Hazard ratio (99% confidence interval)		Events (in exposed)		
outcome	any age	<18	any age	<18	change[†]
Q90 Down syndrome	2.77 [2.46-3.12]	1.03 [0.81-1.31]	855	161	−62.70%
F72 Severe mental retardation	1.67 [1.43-1.94]	1.03 [0.82-1.29]	485	197	−38.42%
I83 Varicose veins of lower extremities	1.63 [1.59-1.67]	1.05 [0.86-1.27]	19,256	254	−35.76%
I27 Other pulmonary heart diseases	1.32 [1.28-1.37]	0.85 [0.63-1.15]	9,635	106	−35.58%
M77 Other enthesopathies	1.47 [1.40-1.56]	0.95 [0.73-1.26]	3,405	136	−35.23%
N02 Recurrent and persistent haematuria	1.32 [1.22-1.43]	0.86 [0.66-1.12]	1,604	139	−35.12%
L25 Unspecified contact dermatitis	2.99 [2.63-3.39]	1.95 [1.49-2.56]	847	164	−34.65%
G82 Paraplegia and tetraplegia	1.15 [1.05-1.25]	0.75 [0.61-0.93]	1,261	198	−34.51%
K72 Hepatic failure, NEC	1.36 [1.28-1.43]	0.89 [0.67-1.18]	3,232	125	−34.37%
I87 Other disorders of veins	1.60 [1.52-1.69]	1.06 [0.79-1.42]	4,228	125	−33.92%
E14 Unspecified diabetes mellitus	1.37 [1.33-1.41]	0.92 [0.75-1.14]	11,113	228	−32.67%
F79 Unspecified mental retardation	1.56 [1.47-1.66]	1.06 [0.96-1.16]	2,891	1,151	−32.39%
Z99 Dependence on enabling machines and devices, NEC	1.39 [1.36-1.43]	0.94 [0.86-1.04]	16,202	1,037	−32.01%
I07 Rheumatic tricuspid valve diseases	1.31 [1.24-1.38]	0.89 [0.67-1.18]	4,088	118	−31.99%
F70 Mild mental retardation	1.72 [1.51-1.97]	1.19 [0.94-1.51]	625	183	−30.95%
K86 Other diseases of pancreas	1.21 [1.17-1.27]	0.86 [0.70-1.05]	5,937	244	−29.38%
F53 Mental and behavioural disorders associated with the puerperium, NEC	1.35 [1.19-1.53]	0.96 [0.73-1.26]	619	135	−28.98%
T13 Other injuries of lower limb, level unspecified	1.17 [1.04-1.31]	0.83 [0.63-1.10]	755	121	−28.80%
G24 Dystonia	1.14 [1.03-1.25]	0.82 [0.67-1.00]	1,028	250	−27.96%
G62 Other polyneuropathies	1.35 [1.30-1.40]	0.97 [0.78-1.22]	6,954	198	−27.76%
N31 Neuromuscular dysfunction of bladder, NEC	1.35 [1.26-1.43]	0.98 [0.82-1.16]	2,392	319	−27.36%
F81 Specific developmental disorders of scholastic skills	1.56 [1.50-1.61]	1.13 [1.07-1.19]	8,353	3,825	−27.33%
N04 Nephrotic syndrome	1.34 [1.22-1.48]	0.99 [0.82-1.20]	1,140	273	−26.10%
D73 Diseases of spleen	1.25 [1.17-1.34]	0.92 [0.72-1.18]	2,222	169	−26.10%
N27 Small kidney of unknown cause	1.30 [1.15-1.47]	0.96 [0.73-1.27]	659	129	−26.07%
F29 Unspecified nonorganic psychosis	1.22 [1.13-1.32]	0.91 [0.77-1.07]	1,809	375	−25.65%
Q28 Other congenital malformations of circulatory system	1.32 [1.12-1.54]	0.98 [0.71-1.35]	391	101	−25.59%
J81 Pulmonary oedema	1.30 [1.24-1.36]	0.97 [0.72-1.31]	4,916	107	−25.39%
I61 Intracerebral haemorrhage	1.17 [1.12-1.23]	0.88 [0.65-1.19]	4,912	104	−25.11%
V89 Motor or nonmotor vehicle accident, type of vehicle unspecified	1.20 [1.02-1.40]	0.90 [0.67-1.20]	370	111	−24.90%
I89 Other noninfective disorders of lymphatic vessels and lymph nodes	1.53 [1.46-1.59]	1.16 [0.91-1.47]	5,461	177	−24.13%
O74 Complications of anaesthesia during labour and delivery	1.16 [1.00-1.34]	0.88 [0.66-1.16]	464	122	−24.07%
L51 Erythema multiforme	1.28 [1.14-1.43]	0.97 [0.83-1.13]	767	416	−24.05%
S20 Superficial injury of thorax	1.18 [1.11-1.25]	0.90 [0.77-1.05]	2,809	409	−23.95%
F42 Obsessive compulsive disorder	1.64 [1.52-1.76]	1.25 [1.09-1.43]	2,087	635	−23.80%
L20 Atopic dermatitis	24.72 [22.44-27.23]	18.85 [16.88-21.04]	6,205	3,853	−23.76%
F60 Specific personality disorders	1.34 [1.27-1.42]	1.02 [0.92-1.14]	3,390	915	−23.63%
T82 Complications of cardiac and vascular prosthetic devices, implants and grafts	1.31 [1.27-1.35]	1.01 [0.91-1.11]	9,620	979	−23.30%
T86 Failure and rejection of transplanted organs and tissues	1.33 [1.22-1.46]	1.02 [0.82-1.28]	1,200	200	−23.26%
S21 Open wound of thorax	1.11 [0.97-1.26]	0.85 [0.70-1.04]	604	247	−23.24%
T14 Injury of unspecified body region	1.16 [1.03-1.29]	0.89 [0.71-1.13]	756	169	−22.77%
M46 Other inflammatory spondylopathies	1.42 [1.36-1.48]	1.10 [0.83-1.45]	5,856	135	−22.71%
Z95 Presence of cardiac and vascular implants and grafts	1.22 [1.21-1.24]	0.95 [0.86-1.05]	45,729	1,021	−22.54%
I37 Pulmonary valve disorders	1.22 [1.12-1.32]	0.95 [0.75-1.20]	1,558	167	−22.20%
F22 Persistent delusional disorders	1.29 [1.19-1.40]	1.00 [0.74-1.36]	1,560	113	−22.18%
I35 Nonrheumatic aortic valve disorders	1.23 [1.20-1.26]	0.95 [0.78-1.17]	19,023	238	−22.16%
R34 Anuria and oliguria	1.21 [1.11-1.33]	0.95 [0.73-1.23]	1,225	145	−22.05%
R18 Ascites	1.20 [1.17-1.24]	0.94 [0.81-1.10]	9,491	441	−21.92%
Z60 Problems related to social environment	1.18 [1.15-1.21]	0.92 [0.80-1.06]	21,873	524	−21.81%
G80 Cerebral palsy	1.01 [0.94-1.09]	0.79 [0.72-0.87]	1,750	1,019	−21.48%
I78 Diseases of capillaries	1.30 [1.21-1.40]	1.03 [0.77-1.36]	1,829	121	−21.45%
F20 Schizophrenia	1.28 [1.21-1.34]	1.00 [0.84-1.20]	3,454	313	−21.31%
I86 Varicose veins of other sites	1.31 [1.22-1.41]	1.03 [0.88-1.21]	1,918	412	−21.15%
Q60 Renal agenesis and other reduction defects of kidney	1.17 [1.03-1.33]	0.92 [0.72-1.18]	628	166	−21.15%
M47 Spondylosis	1.35 [1.33-1.38]	1.07 [0.85-1.35]	32,121	193	−21.02%
E10 Type 1 diabetes mellitus	1.23 [1.19-1.26]	0.97 [0.91-1.04]	10,587	2,324	−20.90%
Q76 Congenital malformations of spine and bony thorax	1.15 [1.02-1.31]	0.91 [0.75-1.12]	639	242	−20.85%
G96 Other disorders of central nervous system	1.30 [1.17-1.44]	1.03 [0.79-1.33]	875	145	−20.75%
J69 Pneumonitis due to solids and liquids	1.17 [1.14-1.20]	0.93 [0.78-1.10]	13,535	323	−20.74%
I69 Sequelae of cerebrovascular disease	1.18 [1.15-1.22]	0.94 [0.73-1.21]	10,401	153	−20.43%
F43 Reaction to severe stress, and adjustment disorders	1.26 [1.20-1.33]	1.00 [0.90-1.12]	3,877	901	−20.36%
R12 Heartburn	1.35 [1.29-1.42]	1.08 [0.90-1.28]	4,130	336	−20.33%
M43 Other deforming dorsopathies	1.29 [1.24-1.34]	1.02 [0.90-1.17]	7,046	575	−20.32%
T11 Other injuries of upper limb, level unspecified	1.19 [1.08-1.31]	0.95 [0.78-1.16]	1,227	253	−20.12%
Z94 Transplanted organ and tissue status	1.36 [1.28-1.44]	1.09 [0.94-1.26]	2,996	449	−20.04%
R52 Pain, NEC	1.40 [1.34-1.46]	1.12 [0.98-1.28]	6,030	610	−20.02%
R16 Hepatomegaly and splenomegaly, NEC	1.38 [1.32-1.45]	1.11 [0.99-1.24]	5,156	829	−19.92%
G90 Disorders of autonomic nervous system	1.26 [1.15-1.40]	1.01 [0.77-1.33]	1,018	143	−19.92%
Z93 Artificial opening status	1.19 [1.16-1.23]	0.96 [0.87-1.05]	13,568	1,158	−19.86%
G41 Status epilepticus	1.20 [1.10-1.31]	0.96 [0.84-1.11]	1,276	479	−19.80%

[†] Percent change of crude hazard ratio from any-age cohort to <18 cohort.

Supplementary Table 9: Largest differences in crude hazard ratios between any-age and 40+ cohorts

outcome	Hazard ratio (99% confidence interval) any age	Hazard ratio (99% confidence interval) 40+	Events (in exposed) any age	Events (in exposed) 40+	change[†]
T78 Adverse effects, NEC	3.07 [2.96-3.18]	1.72 [1.61-1.83]	10,510	2,499	−44.05%
Z01 Other special examinations and investigations of persons withou...	2.34 [2.27-2.42]	1.43 [1.34-1.53]	11,656	2,154	−38.88%
B00 Herpesviral [herpes simplex] infections	2.52 [2.38-2.67]	1.73 [1.58-1.91]	3,846	1,174	−31.16%
J46 Status asthmaticus	2.95 [2.79-3.13]	2.08 [1.85-2.33]	3,968	809	−29.63%
Z71 Persons encountering health services for other counselling and ...	1.65 [1.59-1.70]	1.18 [1.12-1.23]	9,814	4,554	−28.54%
J30 Vasomotor and allergic rhinitis	2.14 [2.08-2.20]	1.67 [1.60-1.74]	17,750	6,066	−22.09%
O82 Single delivery by caesarean section	1.10 [1.03-1.19]	0.86 [0.67-1.11]	1,727	120	−21.99%
O23 Infections of genitourinary tract in pregnancy	1.16 [1.12-1.20]	0.93 [0.73-1.19]	6,682	135	−19.91%
Z84 Family history of other conditions	1.76 [1.66-1.87]	1.41 [1.22-1.64]	3,330	433	−19.90%
V80 Animal rider or occupant of animal drawn vehicle injured in tra...	0.95 [0.86-1.04]	0.76 [0.64-0.90]	1,094	288	−19.87%
H03 Disorders of eyelid in diseases CE	1.61 [1.30-1.98]	1.30 [0.96-1.76]	247	107	−19.28%
O47 False labour	1.16 [1.12-1.21]	0.95 [0.75-1.20]	6,347	146	−18.54%
O42 Premature rupture of membranes	1.03 [1.01-1.06]	0.84 [0.75-0.95]	18,440	557	−18.31%
O26 Maternal care for other conditions predominantly related to pre...	1.16 [1.13-1.18]	0.95 [0.85-1.05]	25,422	756	−17.89%
O48 Prolonged pregnancy	1.02 [0.99-1.05]	0.84 [0.71-1.00]	10,930	279	−17.48%
W21 Striking against or struck by sports equipment	1.11 [1.03-1.21]	0.94 [0.73-1.22]	1,513	131	−15.21%
F91 Conduct disorders	1.27 [1.13-1.42]	1.09 [0.81-1.47]	820	106	−14.35%
T63 Toxic effect of contact with venomous animals	1.10 [0.89-1.37]	0.95 [0.72-1.25]	200	111	−13.87%
B34 Viral infection of unspecified site	1.54 [1.52-1.57]	1.33 [1.27-1.40]	40,130	3,879	−13.70%
O30 Multiple gestation	1.00 [0.94-1.06]	0.86 [0.69-1.07]	2,655	170	−13.61%
X58 Exposure to other specified factors	1.60 [1.47-1.75]	1.39 [1.23-1.56]	1,466	729	−13.41%
J45 Asthma	2.12 [2.11-2.14]	1.84 [1.82-1.87]	165,066	75,502	−13.16%
O40 Polyhydramnios	1.09 [1.03-1.16]	0.95 [0.76-1.19]	2,620	158	−12.81%
B90 Sequelae of tuberculosis	1.60 [1.25-2.03]	1.39 [1.08-1.79]	184	167	−12.71%
J82 Pulmonary eosinophilia, NEC	2.40 [2.08-2.78]	2.11 [1.80-2.47]	600	451	−12.34%
R06 Abnormalities of breathing	1.52 [1.50-1.54]	1.33 [1.31-1.35]	74,870	34,545	−12.29%
T94 Sequelae of injuries involving multiple and unspecified body re...	1.26 [1.01-1.57]	1.11 [0.85-1.44]	196	139	−12.26%
O75 Other CO labour and delivery, NEC	1.04 [1.01-1.07]	0.91 [0.79-1.05]	11,471	410	−12.25%
Q79 Congenital malformations of the musculoskeletal system, NEC	1.35 [1.22-1.51]	1.19 [0.98-1.45]	920	242	−11.73%
Z34 Supervision of normal pregnancy	1.09 [1.07-1.11]	0.97 [0.85-1.09]	20,140	555	−11.39%
Z91 PH of risk factors, NEC	1.76 [1.73-1.78]	1.56 [1.53-1.59]	66,982	30,367	−11.21%
O14 Pre eclampsia	1.10 [1.05-1.16]	0.98 [0.80-1.20]	4,133	205	−11.17%
B01 Varicella [chickenpox]	1.61 [1.52-1.72]	1.43 [1.10-1.87]	2,710	139	−11.12%
O13 Gestational [pregnancy induced] hypertension	1.08 [1.04-1.13]	0.96 [0.81-1.14]	5,577	288	−10.98%
O68 Labour and delivery complicated by fetal stress [distress]	1.04 [1.03-1.06]	0.93 [0.85-1.01]	35,814	1,175	−10.95%
Q50 Congenital malformations of ovaries, fallopian tubes and broad ...	1.25 [1.11-1.41]	1.12 [0.91-1.37]	682	206	−10.74%
L28 Lichen simplex chronicus and prurigo	4.46 [4.06-4.91]	3.99 [3.59-4.42]	1,757	1,387	−10.69%
O63 Long labour	1.02 [0.99-1.04]	0.91 [0.80-1.03]	15,999	487	−10.63%
G10 Huntington disease	1.40 [1.07-1.84]	1.26 [0.94-1.67]	135	113	−10.60%
O70 Perineal laceration during delivery	1.03 [1.02-1.05]	0.93 [0.85-1.01]	48,711	1,170	−10.51%
O04 Medical abortion	1.02 [1.00-1.05]	0.92 [0.81-1.04]	11,751	521	−10.44%
K09 Cysts of oral region, NEC	1.26 [1.14-1.40]	1.13 [0.99-1.30]	971	489	−10.38%
Z35 Supervision of high risk pregnancy	1.07 [1.04-1.09]	0.96 [0.89-1.03]	17,420	1,680	−10.33%
O41 Other disorders of amniotic fluid and membranes	1.10 [1.04-1.16]	0.99 [0.77-1.27]	3,493	132	−10.12%
O61 Failed induction of labour	1.08 [1.01-1.17]	0.98 [0.75-1.27]	1,800	120	−10.00%
H18 Other disorders of cornea	1.38 [1.32-1.44]	1.24 [1.18-1.31]	5,208	3,982	−9.68%
K90 Intestinal malabsorption	1.78 [1.72-1.85]	1.61 [1.53-1.70]	8,443	3,833	−9.59%
J31 Chronic rhinitis, nasopharyngitis and pharyngitis	1.75 [1.66-1.86]	1.59 [1.47-1.72]	3,383	1,625	−9.32%
G09 Sequelae of inflammatory diseases of central nervous system	1.36 [1.14-1.63]	1.24 [0.98-1.56]	311	176	−9.15%
O20 Haemorrhage in early pregnancy	1.06 [1.03-1.10]	0.97 [0.83-1.13]	8,064	356	−9.14%
O99 Other maternal diseases classifiable elsewhere but complicating...	1.16 [1.14-1.19]	1.06 [0.97-1.15]	27,987	1,210	−9.12%
O36 Maternal care for other known or suspected fetal problems	1.12 [1.10-1.14]	1.02 [0.93-1.11]	33,543	1,131	−9.10%
L63 Alopecia areata	2.87 [2.32-3.56]	2.62 [1.97-3.48]	290	151	−8.96%
O28 Abnormal findings on antenatal screening of mother	1.06 [0.99-1.13]	0.97 [0.75-1.25]	2,337	122	−8.78%
B59 Pneumocystosis	1.53 [1.24-1.88]	1.39 [1.12-1.73]	236	205	−8.73%
O43 Placental disorders	1.03 [0.97-1.10]	0.94 [0.71-1.25]	2,479	103	−8.65%
O62 Abnormalities of forces of labour	1.05 [1.01-1.09]	0.96 [0.81-1.15]	7,213	271	−8.58%
K50 Crohn disease [regional enteritis]	1.70 [1.63-1.77]	1.56 [1.47-1.64]	6,324	3,344	−8.52%
L27 Dermatitis due to substances taken internally	1.75 [1.65-1.86]	1.60 [1.49-1.72]	3,074	2,008	−8.50%
G03 Meningitis DTOAUC	1.24 [1.07-1.43]	1.13 [0.92-1.40]	464	200	−8.42%
L30 Other dermatitis	10.30 [10.10-10.50]	9.44 [9.16-9.73]	77,273	27,658	−8.36%
D28 Benign neoplasm of OAU female genital organs	1.25 [1.06-1.47]	1.14 [0.93-1.40]	347	218	−8.33%
H15 Disorders of sclera	1.41 [1.17-1.71]	1.30 [1.04-1.62]	277	208	−8.26%
O16 Unspecified maternal hypertension	1.12 [1.07-1.17]	1.03 [0.86-1.24]	4,610	243	−8.06%
O72 Postpartum haemorrhage	1.05 [1.03-1.08]	0.97 [0.89-1.06]	23,598	1,079	−8.06%
D39 Neoplasm of uncertain or unknown behaviour of female genital or...	1.06 [0.92-1.22]	0.97 [0.83-1.14]	459	329	−8.01%
O34 Maternal care for known or suspected abnormality of pelvic organs	1.09 [1.06-1.11]	1.00 [0.93-1.08]	18,898	1,365	−7.96%
R71 Abnormality of red blood cells	1.22 [1.00-1.49]	1.13 [0.91-1.40]	249	200	−7.95%
N34 Urethritis and urethral syndrome	1.35 [1.16-1.58]	1.25 [1.03-1.52]	404	251	−7.56%
D29 Benign neoplasm of male genital organs	1.23 [1.02-1.49]	1.14 [0.92-1.42]	263	208	−7.26%

[†] Percent change of crude hazard ratio from any-age cohort to 40+ cohort.

Supplementary Table 10: Comparison with previous study of primary-care-captured outcomes

Primary care outcomes			ICD-10 outcomes		
rank	outcome	HR	rank	outcome	HR
1	Food allergy	4.03 [3.95-4.11]	1	930 Allergic reaction to food	4.37 [4.11-4.63]
2	Allergic Conjunctivitis	2.01 [1.98-2.04]	2	370.3 Keratoconjunctivitis	2.53 [1.97-3.24]
3	Allergic Rhinitis	1.92 [1.90-1.93]	6	476 Allergic rhinitis	1.73 [1.68-1.78]
4	Asthma	1.87 [1.86-1.89]	9	J45 Asthma	1.61 [1.59-1.62]
5	Hodgkin lymphoma	1.85 [1.66-2.06]	7	C81 Hodgkin lymphoma	1.64 [1.48-1.83]
6	Molluscum contagiosum	1.81 [1.79-1.83]	12	B08 Other viral infections chara...	1.52 [1.37-1.69]
7	Alopecia Areata	1.77 [1.71-1.83]	4	L63 Alopecia areata	2.27 [1.80-2.85]
8	Crohn's disease	1.62 [1.54-1.69]	13	K50 Crohn disease [regional ente...	1.47 [1.41-1.54]
9	Dermatophyte infection	1.60 [1.59-1.61]	10	B35 Dermatophytosis	1.58 [1.45-1.71]
10	Urticaria	1.58 [1.57-1.60]	8	L50 Urticaria	1.64 [1.55-1.73]
11	Herpes simplex	1.51 [1.49-1.53]	3	B00 Herpesviral [herpes simplex]...	2.34 [2.21-2.49]
12	Impetigo	1.49 [1.48-1.51]	5	L01 Impetigo	2.10 [1.91-2.32]
13	Coeliac disease	1.42 [1.37-1.47]	11	K90 Intestinal malabsorption	1.56 [1.50-1.62]
14	Ulcerative colitis	1.40 [1.34-1.46]	16	K51 Ulcerative colitis	1.35 [1.30-1.40]
15	Autoimmune liver disease	1.32 [1.21-1.43]	19	571.5 Other chronic nonalcoholic...	1.29 [1.25-1.33]
16	Irritable bowel syndrome	1.31 [1.29-1.32]	22	K58 Irritable bowel syndrome	1.27 [1.24-1.30]
17	Cutaneous warts	1.30 [1.29-1.31]	18	B07 Viral warts	1.30 [1.20-1.42]
18	Fibrosis/sclerosis/cirrhosis	1.27 [1.22-1.33]	15	K74 Fibrosis and cirrhosis of liver	1.35 [1.29-1.42]
19	Non-hodgkin lymphoma	1.26 [1.21-1.32]	21	C85 Other and unspecified types ...	1.28 [1.21-1.35]
20	Oesophageal varices	1.25 [1.15-1.36]	14	I85 Oesophageal varices	1.37 [1.29-1.46]
21	Thromboembolic diseases	1.25 [1.23-1.27]	34	I80 Phlebitis and thrombophlebitis	1.20 [1.17-1.23]
22	Oesophageal ulcer	1.25 [1.23-1.27]	26	K20 Oesophagitis	1.22 [1.19-1.25]
23	Metabolic syndrome	1.25 [1.10-1.42]	37	E11 Type 2 diabetes mellitus	1.19 [1.18-1.20]
24	Gastro oesophageal reflux	1.25 [1.24-1.26]	28	K21 Gastro oesophageal reflux di...	1.21 [1.20-1.23]
25	Fatty liver	1.24 [1.22-1.26]	24	K76 Other diseases of liver	1.23 [1.20-1.25]
26	COPD	1.22 [1.21-1.24]	20	J44 Other chronic obstructive pu...	1.29 [1.27-1.31]
27	Obesity	1.22 [1.21-1.23]	33	E66 Obesity	1.20 [1.19-1.21]
28	Gastritis and duodenitis	1.21 [1.20-1.23]	25	K29 Gastritis and duodenitis	1.22 [1.21-1.24]
29	Peripheral neuropathies	1.21 [1.20-1.22]	43	356 Hereditary and idiopathic pe...	1.17 [1.02-1.34]
30	Peripheral artery disease	1.19 [1.17-1.22]	17	I73 Other peripheral vascular di...	1.32 [1.29-1.35]
31	Osteoporosis	1.18 [1.17-1.20]	27	M81 Osteoporosis without patholo...	1.22 [1.20-1.24]
32	Migraine	1.18 [1.17-1.19]	44	G43 Migraine	1.16 [1.13-1.19]
33	Spine fracture	1.18 [1.15-1.21]	52	805 Fracture of vertebral column...	1.13 [1.09-1.18]
34	Barett's oesophagus	1.17 [1.13-1.21]	29	K22 Other diseases of oesophagus	1.21 [1.18-1.23]
35	Diverticular disease	1.17 [1.16-1.18]	41	K57 Diverticular disease of inte...	1.17 [1.16-1.19]
36	Depression	1.17 [1.16-1.17]	23	296.2 Depression	1.23 [1.22-1.25]
37	Anxiety	1.17 [1.16-1.17]	31	300.1 Anxiety disorder	1.21 [1.19-1.22]
38	Heart failure	1.16 [1.15-1.18]	32	I50 Heart failure	1.20 [1.19-1.22]
39	Peptic ulcer disease	1.16 [1.13-1.19]	35	K27 Peptic ulcer, site unspecified	1.20 [1.10-1.30]
40	Epilepsy	1.15 [1.12-1.18]	30	G40 Epilepsy	1.21 [1.18-1.24]
41	Autism	1.15 [1.13-1.17]	39	313.3 Autism	1.18 [1.14-1.23]
42	Coronary artery disease	1.15 [1.14-1.16]	47	411.4 Coronary atherosclerosis	1.15 [1.14-1.17]
43	Alcohol abuse	1.14 [1.11-1.16]	42	317.1 Alcoholism	1.17 [1.15-1.19]
44	Abdominal hernia	1.13 [1.12-1.15]	40	K46 Unspecified abdominal hernia	1.18 [1.09-1.28]
45	Dyslipidemia	1.13 [1.12-1.14]	36	272.1 Hyperlipidemia	1.19 [1.16-1.22]
46	Diabetes mellitus	1.13 [1.12-1.14]	49	E10 Type 1 diabetes mellitus	1.15 [1.12-1.19]
47	Nonmelanoma skin cancer	1.13 [1.11-1.14]	62	C44 Other malignant neoplasms of...	1.07 [1.05-1.09]
48	Pelvis fracture	1.11 [1.07-1.16]	59	802 Fracture of pelvis	1.08 [1.04-1.13]
49	Hypertension	1.11 [1.10-1.12]	38	I10 Essential (primary) hyperten...	1.19 [1.18-1.20]

Supplementary Table 10: Comparison with previous study of primary-care-captured outcomes					
Primary care outcomes			ICD-10 outcomes		
rank	outcome	HR	rank	outcome	HR
50	Pancreatitis	1.11 [1.07-1.15]	50	K85 Acute pancreatitis	1.14 [1.10-1.18]
51	Cholecystitis	1.10 [1.07-1.14]	55	K81 Cholecystitis	1.12 [1.08-1.17]
52	Hip fracture	1.10 [1.08-1.13]	58	S72 Fracture of femur	1.09 [1.07-1.11]
53	Myeloma	1.10 [1.03-1.18]	57	C90 Multiple myeloma and maligna...	1.11 [1.03-1.19]
54	Stroke	1.09 [1.08-1.11]	61	I63 Cerebral infarction	1.08 [1.06-1.10]
55	Myocardial infarction	1.09 [1.07-1.11]	53	I21 Acute myocardial infarction	1.13 [1.11-1.15]
56	ADHD	1.08 [1.06-1.11]	54	313.1 Attention deficit hyperact...	1.12 [1.06-1.19]
57	Melanoma	1.08 [1.05-1.11]	69	C43 Malignant melanoma of skin	0.94 [0.89-1.00]
58	Multiple sclerosis	1.08 [1.01-1.16]	45	G35 Multiple sclerosis	1.16 [1.08-1.24]
59	Pancreatic cancer	1.08 [1.02-1.14]	48	C25 Malignant neoplasm of pancreas	1.15 [1.09-1.22]
60	Vascular dementia	1.07 [1.04-1.11]	60	F01 Vascular dementia	1.08 [1.05-1.12]
61	Appendicitis	1.07 [1.05-1.09]	65	K35 Acute appendicitis	1.04 [1.01-1.07]
62	Wrist fracture	1.07 [1.06-1.08]	68	S52 Fracture of forearm	1.04 [1.02-1.06]
63	Peritonitis	1.07 [1.00-1.13]	46	K65 Peritonitis	1.16 [1.10-1.21]
64	CNS cancers	1.05 [1.01-1.10]	56	C72 Malignant neoplasm of spinal...	1.11 [0.82-1.49]
65	Lung cancer	1.05 [1.02-1.08]	51	C34 Malignant neoplasm of bronch...	1.14 [1.11-1.17]
66	Alzheimer's dementia	1.04 [1.01-1.06]	63	G30 Alzheimer disease	1.06 [1.03-1.09]
67	Breast cancer	1.03 [1.01-1.06]	66	C50 Malignant neoplasm of breast	1.04 [1.01-1.07]
68	Parkinson's disease	1.01 [0.98-1.05]	64	332 Parkinson's disease	1.05 [1.01-1.09]
69	Prostate cancer	1.01 [0.99-1.03]	67	C61 Malignant neoplasm of prostate	1.04 [1.01-1.06]

Hazard ratios from "Cohort studies on 71 outcomes among people with atopic eczema in UK primary care data" ranked by main adjusted hazard ratio from the any-age cohort, compared to corresponding outcomes defined either as category-level ICD-10 code or phecode and their ranking by main adjusted hazard ratio from the any-age cohort. No corresponding outcomes were available for eosinophilic oesophagitis and cigarette smoking. Food allergy=930 Allergic reaction to food; Allergic Conjunctivitis=370.3 Keratoconjunctivitis; Allergic Rhinitis=476 Allergic rhinitis; Asthma=J45 Asthma; Hodgkin lymphoma=C81 Hodgkin lymphoma; Molluscum contagiosum=B08 Other viral infections chara...; Alopecia Areata=L63 Alopecia areata; Crohn's disease=K50 Crohn disease [regional ente...; Dermatophyte infection=B35 Dermatophytosis; Urticaria=L50 Urticaria; Herpes simplex=B00 Herpesviral [herpes simplex]...; Impetigo=L01 Impetigo; Coeliac disease=K90 Intestinal malabsorption; Ulcerative colitis=K51 Ulcerative colitis; Autoimmune liver disease=571.5 Other chronic nonalcoholic...; Irritable bowel syndrome=K58 Irritable bowel syndrome; Cutaneous warts=B07 Viral warts; Fibrosis/sclerosis/cirrhosis=K74 Fibrosis and cirrhosis of liver; Non-Hodgkin lymphoma=C85 Other and unspecified types ...; Oesophageal varices=I85 Oesophageal varices; Thromboembolic diseases=I80 Phlebitis and thrombophlebitis; Oesophageal ulcer=K20 Oesophagitis; Metabolic syndrome=E11 Type 2 diabetes mellitus; Gastro oesophageal reflux=K21 Gastro oesophageal reflux di...; Fatty liver=K76 Other diseases of liver; COPD=J44 Other chronic obstructive pu...; Obesity=E66 Obesity; Gastritis and duodenitis=K29 Gastritis and duodenitis; Peripheral neuropathies=356 Hereditary and idiopathic peripheral neuropathy; Peripheral artery disease=I73 Other peripheral vascular di...; Osteoporosis=M81 Osteoporosis without patholo...; Migraine=G43 Migraine; Spine fracture=805 Fracture of vertebral column...; Barett's oesophagus=K22 Other diseases of oesophagus; Diverticular disease=K57 Diverticular disease of inte...; Depression=296.2 Depression; Anxiety=300.1 Anxiety disorder; Heart failure=I50 Heart failure; Peptic ulcer disease=K27 Peptic ulcer, site unspecified; Epilepsy=G40 Epilepsy; Autism=313.3 Autism; Coronary artery disease=411.4 Coronary atherosclerosis; Alcohol abuse=317.1 Alcoholism; Abdominal hernia=K46 Unspecified abdominal hernia; Dyslipidemia=272.1 Hyperlipidemia; Diabetes mellitus=E10 Type 1 diabetes mellitus; Nonmelanoma skin cancer=C44 Other malignant neoplasms of...; Pelvis fracture=802 Fracture of pelvis; Hypertension=I10 Essential (primary) hyperten...; Pancreatitis=K85 Acute pancreatitis; Cholecystitis=K81 Cholecystitis; Hip fracture=S72 Fracture of femur; Myeloma=C90 Multiple myeloma and maligna...; Stroke=I63 Cerebral infarction; Myocardial infarction=I21 Acute myocardial infarction; ADHD=313.1 Attention deficit hyperact...; Melanoma=C43 Malignant melanoma of skin; Multiple sclerosis=G35 Multiple sclerosis; Pancreatic cancer=C25 Malignant neoplasm of pancreas; Vascular dementia=F01 Vascular dementia; Appendicitis=K35 Acute appendicitis; Wrist fracture=S52 Fracture of forearm; Peritonitis=K65 Peritonitis; CNS cancers=C72 Malignant neoplasm of spinal...; Lung cancer=C34 Malignant neoplasm of bronch...; Alzheimer's dementia=G30 Alzheimer disease; Breast cancer=C50 Malignant neoplasm of breast; Parkinson's disease=332 Parkinson's disease; Prostate cancer=C61 Malignant neoplasm of prostate.
